# Comparative analysis of codon usage patterns in chloroplast genomes of six *Euphorbiaceae* species

**DOI:** 10.7717/peerj.8251

**Published:** 2020-01-06

**Authors:** Zhanjun Wang, Beibei Xu, Bao Li, Qingqing Zhou, Guiyi Wang, Xingzhou Jiang, Chenchen Wang, Zhongdong Xu

**Affiliations:** 1 College of Life Sciences, Hefei Normal University, Hefei, Anhui, China; 2 Cyrus Tang Hematology Center, Soochow University, Soochow, Jiangsu, China

**Keywords:** *Euphorbiaceae* plants, Codon usage bias, Chloroplast genome

## Abstract

*Euphorbiaceae* plants are important as suppliers of biodiesel. In the current study, the codon usage patterns and sources of variance in chloroplast genome sequences of six different *Euphorbiaceae* plant species have been systematically analyzed. Our results revealed that the chloroplast genomes of six *Euphorbiaceae* plant species were biased towards A/T bases and A/T-ending codons, followed by detection of 17 identical high-frequency codons including GCT, TGT, GAT, GAA, TTT, GGA, CAT, AAA, TTA, AAT, CCT, CAA, AGA, TCT, ACT, TAT and TAA. It was found that mutation pressure was a minor factor affecting the variation of codon usage, however, natural selection played a significant role. Comparative analysis of codon usage frequencies of six *Euphorbiaceae* plant species with four model organisms reflected that *Arabidopsis thaliana*, *Populus trichocarpa*, and *Saccharomyces cerevisiae* should be considered as suitable exogenous expression receptor systems for chloroplast genes of six *Euphorbiaceae* plant species. Furthermore, it is optimal to choose *Saccharomyces cerevisiae* as the exogenous expression receptor. The outcome of the present study might provide important reference information for further understanding the codon usage patterns of chloroplast genomes in other plant species.

## Introduction

As an important source of biodiesel, vegetable oil has attracted much attention with the depletion of fuel resources and the gradual increase of fuel price (Aranda-Rickert, Morzán & Fracchia, 2011). In general, biodiesel (Mono-alkyl esters) is synthesized by transesterification of vegetable oil with monohydric alcohol (Knothe, 2005). Biodiesel could be utilized worldwide as it is renewable, biodegradable, eco-friendly, and possess similar characteristics as fossil diesel (Mahmudul et al., 2017). *Euphorbiaceae* includes 300 genera and 8,000 species (Mwine & Damme, 2011) that are widely distributed in tropical and temperate regions (Hecker, 1968). *Euphorbiaceae* plants possess extensive medicinal values and are important economic plants rich in rubber, starch, wood (Kaul, 1988). Recently, *Euphorbiaceae* plants have drawn much more attention as a raw material of biodiesel (Han et al., 2017).

Chloroplasts are the main organelles that regulate plant photosynthesis and have the capability of sensing stress signals from the external environment (Lv et al., 2019). Due to the small sizes and large copy numbers of chloroplast genomes (Xu et al., 2011), they gained the attention of scientists. Moreover, in comparison with the nuclear gene transformation, chloroplast transformation has the advantages of high expression efficiency of exogenous genes, fixed-point integration, no position effect phenomenon, stable heredity and no drift with pollen (Kwak et al., 2019; Ruf et al., 2019). With the rapid development of high-throughput sequencing technology, the chloroplast genomes of 2,242 plants have been sequenced (published on NCBI) by April 5th, 2019, including *Euphorbia esula*, *Hevea brasiliensis* (Tangphatsornruang et al., 2011), *Jatropha curcas* (Asif et al., 2010), *Manihot esculenta* (Daniell et al., 2008), *Ricinus communis* (Rivarola et al., 2011) and *Vernicia fordii* (Li et al., 2017). Recently, Xin et al. (2018) reported the evolutionary analysis based on chloroplast genomes from four different families including *Euphorbiaceae*, *Flacourtiaceae*, *Passifloraceae* and *Violaceae*. *Xin*’s evolutionary tree showed that the six plants mentioned above were clustered into a big clade, which reflected the close genetic relationship among them (Xin et al., 2018). Various scientists have reported the functions of a majority of genes in plant chloroplasts (Kurepa, Montagu & Inzé, 1997; Samach et al., 2011). Chang et al. (2017) reported the effect of *PTAC10* on the development of chloroplasts and color of leaves. In addition, *sel1* mutation impacts the development of chloroplasts and causes etiolated plastid development defects (Pyo et al., 2013). Moreover, *RsgA* plays a key role in maintaining the normal morphology of chloroplasts as described by Janowski et al. (2018). Boynton et al. (1988) transferred *chlamydomonas* chloroplast *atpB* gene into *chlamydomonas atpB* mutant using gene gun, which marked the beginning of chloroplast genetic engineering. With the rapid development of chloroplast gene transformation, Kwak et al. (2019) transferred plasmid DNA into the chloroplasts of various plant species, i.e., *Eruca sativa*, *Nasturtium officinale* and *Nicotiana tabacum* utilizing chitosan-complexed single-walled carbon nanotubes. There are numerous studies to report the applicability of chloroplast transgenic technology for few plants (Havaux, Lütz & Grimm, 2003; Khodakovskaya et al., 2006; Schreuder et al., 2001). However, to construct mature and stable chloroplast transgenic systems in more plants, analysis of codon usage patterns for target genes or recipient plants is urgently needed (Scotti et al., 2009).

Codon usage bias refers to the differences in the usage frequency of synonymous codons when coding DNA which may be caused by different factors on genes during the evolutionary process (Ikemura, 1985). It is generally believed that codon usage not only reflects the origin, evolution and mutation mode of species or genes, but also has an important influence on gene function and protein expression (Pop et al., 2014; Quax et al., 2015; Tuller et al., 2010). Previous researches on codon usage bias of the chloroplast genomes can improve the expression efficiency of exogenous genes by selecting appropriate codons for transgenic research (Zhou, Tong & Shi, 2007). At present, many studies validated the applicability of synonymous codon bias for the chloroplast genome level of within-species and between-species in higher plants, such as *Poaceae* (Zhang et al., 2012), *Asteraceae* (Nie et al., 2013), *Cinnamomum camphora* (Chen et al., 2017), *Morus* (Kong & Yang, 2017), *Strawberry* (Cheng et al., 2017) and *Solanum* (Zhang et al., 2018). However, the codon usage bias of chloroplast genomes in six *Euphorbiaceae* plant species has not been reported.

In this study, we systematically analyzed the codon usage patterns and sources of variance in chloroplast genomes of six *Euphorbiaceae* plant species. In addition, comparative analysis of the codon usage frequencies of these six plants with four model organisms including *Arabidopsis thaliana*, *Populus trichocarpa*, *Escherichia coli* and *Saccharomyces cerevisiae* were performed. The results will provide insight into further improving the efficiency of exogenous gene expression in six *Euphorbiaceae* plant species.

## Materials and Methods

### Genomes and coding sequences

The complete chloroplast genomes of *Euphorbia esula*, *Hevea brasiliensis* (Tangphatsornruang et al., 2011), *Jatropha curcas* (Asif et al., 2010), *Manihot esculenta* (Daniell et al., 2008), *Ricinus communis* (Rivarola et al., 2011), and *Vernicia fordii* (Li et al., 2017) with gene annotations were downloaded from the NCBI GenBank database. The number of raw CDSs of six *Euphorbiaceae* species was 85, 84, 84, 83, 86 and 85 respectively (Table 1; Table S1). In order to avoid sampling errors, each CDS in the chloroplast genomes of six *Euphorbiaceae* species should follow certain rules, i.e., the number of bases in each CDS should be the fold of three; the length of sequence encoding gene must be ≥ 300 bp; high-quality sequences with identified bases, i.e., containing only A, T, G and C bases; each CDS contains proper initiation codon (ATG) and termination codons (TAG,TGA and TAA); and sequences without an intermediate stop codon (He et al., 2016; Li et al., 2016; Zhang et al., 2007). We used Perl scripts written by our team to filter the CDSs according to the five rules mentioned above and simplify the names of CDSs replaced with numbers to avoid miscalculation. The GC content of the first, second and third codon positions (GC1, GC2, GC3) and the average GC content of three positions were calculated by Perl script.

### Analysis of Relative synonymous codon usage (RSCU) and Relative synonymous codon usage frequency (RFSC)

RSCU value for a particular codon refers to the ratio of its actual usage frequency to expected frequency when it is used without bias. The RSCU was calculated as Eq. (1):

(1) \begin{eqnarray*}RSCU= \frac{{x}_{ij}}{\sum _{j}^{{n}_{i}}{x}_{ij}} {n}_{i}\end{eqnarray*}

where *x*_*ij*_ represents the frequency of codon *j* encoding for the *i* th amino acid, and *n*_*i*_ represents the number of synonymous codons encoding the *i* th amino acid (Sharp & Li, 1986). If the RSCU value of one codon equals 1 that reflected no codon usage bias and is used equally with other synonymous codons. However, strong positive codon usage bias could be observed for RSCU value >1. In contrast, RSCU value <1 displayed negative codon usage bias that is used less frequently than other codons (Sharp & Li, 1987).

**Table 1:** Genomic features of chloroplast genomes of six *Euphorbiaceae* plant species. L_aa means the total number of amino acids; GC1, GC2 and GC3 indicate the GC content at the first, second and third codon positions.

**Parameters**	***Euphorbia esula***	***Hevea brasiliensis***	***Jatropha curcas***	***Manihot esculenta***	***Ricinus communis***	***Vernicia fordii***
Accession No.	NC_033910.1	NC_015308.1	NC_012224.1	NC_010433.1	NC_016736.1	NC_034803.1
CDSs number (before processing)	85	84	84	83	86	85
CDSs number (after processing)	53	55	58	55	55	57
L_aa	23,157	23,918	24,407	21,902	24,249	24,582
GC1	0.453	0.451	0.454	0.454	0.451	0.454
GC2	0.372	0.372	0.375	0.375	0.374	0.374
GC3	0.287	0.292	0.294	0.286	0.299	0.296
Average GC at three locations	0.371	0.372	0.374	0.372	0.375	0.375

The RFSC value is equal to the ratio of the actual observed number of one codon to the number of all synonymous codons (Sharp & Li, 1986). The RFSC was calculated using Eq. (2):

(2) \begin{eqnarray*}RFSC= \frac{{x}_{ij}}{\sum _{j}^{{n}_{i}}{x}_{ij}} \end{eqnarray*}

where *x*_*ij*_ represents the frequency of codon *j* encoding for the *i* th amino acid. The RFSC value of a codon exceeding 60% or 0.5 times higher than the average frequency of the synonymous codons indicates high-frequency codon (Zhou, Tong & Shi, 2007).

### Comparative analysis of codon usage frequency

In order to deeply analyze the codon usage patterns of six *Euphorbiaceae* plant species, codon usage bias data of *Arabidopsis thaliana* (http://www.kazusa.or.jp/codon/cgi-bin/showcodon.cgi?species=3702), *Populus trichocarpa* (http://www.kazusa.or.jp/codon/cgi-bin/showcodon.cgi?species=3694), *Escherichia coli* (http://www.kazusa.or.jp/codon/cgi-bin/showcodon.cgi?species=199310) and *Saccharomyces cerevisiae* (http://www.kazusa.or.jp/codon/cgi-bin/showcodon.cgi?species=4932) downloaded from Codon Usage Database were compared with the codon usage frequencies of six *Euphorbiaceae* plants. Furthermore, we calculated the ratio of codon usage frequency for six *Euphorbiaceae* plant species to four model organisms. When the ratio is ≥ 2 or ≤ 0.5, it indicates the difference of codon usage bias between two organisms is greater (Pan et al., 2013).

### Analysis of ENc-plot

ENc (effective number of codons) value is used to analyze the degree of deviation of codon usage from the random selection that depicted the degree of imbalanced use of synonymous codons in genes or genomes of the specific species. The range of ENc value is 20-61. The smaller the ENc value, the stronger the codon usage bias and vice versa (Wu et al., 2018). When ENc value is ≤ 35, the codon usage of genes or genomes has very significant bias (Mensah et al., 2019). GC3s value refers to the ratio of G and C content at the third position of one codon to the total number of gene bases excluding Met and Trp. Using GC3s value as abscissa and ENc value as ordinate to make the ENc-plot, the results revealed the influencing factors of codon usage patterns of genes or genomes, and the relationship between gene base composition and codon usage bias (Wright, 1990). The expected values of ENc were calculated according to the Eq. (3):

(3) \begin{eqnarray*}ENc=2+S+ \frac{29}{{S}^{2}+(1-S)^{2}} \end{eqnarray*}

where S denotes GC3s (Wright, 1990; Zhang et al., 2007). When mutation pressure plays an important role in the formation of codon usage patterns, ENc value lies on or around the expected curve. However, when codon usage is affected by natural selection and other factors, ENc value is far lower than the expected curve (Wright, 1990).

### PR2-plot analysis

PR2-plot is used to analyze the composition of four bases at the third position of codon encoding amino acids. It is a graphical analysis based on A3/(A3 + T3) as ordinate and G3/(G3 + C3) as abscissa (Sueoka, 1999). The distribution of points around the center point (A = T, C = G) shows the degree and direction of the base deviation. It was generally believed that the proportion of A/T and C/G is balanced in degenerate codons of genes or genomes upon single mutation pressure (Xiang et al., 2015).

### Analysis of Neutrality plot

Neutrality plot (GC12 vs. GC3) was performed to investigate the extent of influence between mutation pressure and natural selection on the patterns of codon usage (Sueoka, 1988). GC12 represents the average value of GC contents at the first and second positions of codon while GC3 is the GC content at the third position. GC3 was calculated excluding the three termination codons (TAA, TAG and TGA) and the three codons for Ile (ATT, ATC and ATA). Meanwhile, two single codons for Met (ATG) and Trp (TGG) were also excluded in all three patterns (Sueoka, 1988). GC12 and GC3 of chloroplast genomes in six *Euphorbiaceae* species were calculated by Perl script. The slope of the plot regression was zero indicates no effects of directional mutation pressure (complete selective constraints). The slope 1 depicted that the codon usage bias is completely affected by directional mutation pressure representing complete neutrality (Sueoka, 1988; Wen et al., 2017).

### Correlation analysis (COA)

The codon usage variations in chloroplast genomes of six *Euphorbiaceae* plant species were investigated with correspondence analysis based on RSCU using CodonW (Version 1.4.2; Mensah et al., 2019). Correspondence analysis was performed to compare the usage patterns of 59 codons (excluding codons encoding Met, Trp and three termination codons), and the results produce a series of orthogonal axes that can be used to present the codon usage variation in chloroplast genomes of six *Euphorbiaceae* plant species. The distribution of genes can be drawn according to the synonymous codon usage of the genes in a multidimensional space of 59 axes, followed by the maximum fraction of gene variations, thus the main sources of codon usage variation were analyzed (Xiang et al., 2015). Based on the results of codon usage variation, correlation analysis between axis 1 and codon usage indices including codon adaptation index (CAI), the GC content at the third codon position of synonymous codons (GC3s; Zhang et al., 2007) and the total number of amino acids (L_aa; Wright, 1990) were carried out by SPSS (Version 23). The value is negative means a negative correlation. CAI value is widely used to evaluate the gene expression level and ranges from 0 to 1. The larger the CAI value, the stronger the codon usage bias, otherwise, the weaker the codon usage bias (Sharp & Li, 1986).

## Results and Discussion

### Characteristics of codon usage bias

#### Indices of codon usage

The CDSs processed by Perl scripts contained 53, 55, 58, 55, 55 and 57 respectively for six *Euphorbiaceae* species (Table 1; Table S2).The patterns of codon usage are strongly correlated with GC content, so we calculated the GC contents of the first, second, and third sites of codons (Shackelton, Parrish & Holmes, 2006). It was found that the contents of GC1, GC2, GC3 and the average content of GC at three positions were less than 0.500 (Table 1), indicating that the six chloroplast genomes tended to use A/T bases and A/T-ending codons. In addition, the average GC content of three locations in *Ricinus communis* and *Vernicia fordii* is the same (0.375), but the contents of the other four *Euphorbiaceae* plant species are slightly different (0.371–0.374; Table 1). Zhang et al. (2012) revealed the average third base of codons were biased towards A/T in the 23 *Poaceae* chloroplast genome codons as 0.613, which coincides with the findings of Nie et al. (2013) who reported that the average AT content (0.625) of the *Asteraceae* chloroplast genome was significantly higher than the GC content (0.375). Moreover, *Zhang* and colleagues (2018) also described the higher AT content of the whole genome (0.620) for the chloroplast genome codons in different *Solanum* species. In summary, the chloroplast genomes of six *Euphorbiaceae* plant species, *Poaceae* (Zhang et al., 2012), *Asteraceae* (Nie et al., 2013), and *Solanum* (Zhang et al., 2018) were biased towards A/T bases in codon usage.

#### RSCU and RFSC

The chloroplast genomes of six *Euphorbiaceae* plant species possess 30 identical codons (RSCU >  1) with 29 codons ending with A/T that accounted for 96.67% (Table S1). Thus, the codons of six plants (RSCU >  1) tended to end with A/T. In contrast, the codons with negative bias (RSCU <  1) mostly end with G/C. Six plants possess 32 identical codons (RSCU <  1) with 29 codons ending with C/G that accounted for 90.63%. The variation ranges in the RSCU values were similar in the chloroplast genomes of six *Euphorbiaceae* species, i.e., 0.34–2.15, 0.33–1.93, 0.34–1.91, 0.34–2.05, 0.32–1.92 and 0.32–1.90, respectively (Table S1). Meanwhile, the highest and the lowest RSCU values belonged to AGA and CGC that encode Arg and implied the extremely positive bias in AGA and negative bias in CGC. The high-frequency codons of chloroplast genomes of six *Euphorbiaceae* plant species possess high similarity with a total of 17 identical high-frequency codons including GCT, TGT, GAT, GAA, TTT, GGA, CAT, AAA, TTA, AAT, CCT, CAA, AGA, TCT, ACT, TAT and TAA (Table S1). Two species, i.e., *Manihot esculenta* and *Ricinus communis* possess one more high-frequency codon (GTA) than other four *Euphorbiaceae* plant species.

#### Codon usage frequency

In higher plants, chloroplast transformation could be performed for *Nicotiana tabacum* (Kurepa, Montagu & Inzé, 1997). The main obstacle to extend the technology to other species and, most importantly, to major crops is the limitations probably posed by the currently available tissue culture systems and regeneration protocols for transplastomic plants (Ruf et al., 2001). Considering the differences in codon usage bias among the chloroplast genes of six *Euphorbiaceae* plant species and the receptors for the expression efficiency of genes, codon usage frequencies must be analyzed.

In this study, we compared the codon usage frequencies of chloroplast genomes of six *Euphorbiaceae* plant species with *Arabidopsis thaliana*, *Populus trichocarpa*, *Escherichia coli* and *Saccharomyces cerevisiae* (Table S2). Results suggested slight differences in the codon usage frequencies among six *Euphorbiaceae* plant species with *Arabidopsis thaliana*, *Populus trichocarpa* and *Saccharomyces cerevisiae*, have 13–16 (accounting for 20.31%–25.00% of total codons), 11–13 (17.19%–20.31%), 8–9 (12.50%–14.06%) different codons (Table S2). In contrast, the codon usage for six plants with *Escherichia coli* was relatively higher, i.e., 26–28 different codons (40.63%–43.75%) which suggest the exclusion of the *Escherichia coli* as expression receptor while selecting the receptor system for six plants. Meantime, *Arabidopsis thaliana*, *Populus trichocarpa*, and *Saccharomyces cerevisiae* were considered as suitable gene expression receptor systems for six plants. Furthermore, it was optimal to select *Saccharomyces cerevisiae* as the gene expression receptor for six plants because it had a slight difference in codon usage frequency with six plants. Furthermore, the results indicated that TGA is a different termination codon in usage frequency when comparing all six *Euphorbiaceae* plant species with *Arabidopsis thaliana* and *Escherichia coli*. TAA also showed the difference in comparison of all six plants with *Populus trichocarpa* (Table S2).

Nakamura & Sugiura (2007) and Nakamura & Sugiura (2011) observed no correlation with the translation efficiency of single amino acid (Tyrosine) with the codon usage bias in *Nicotiana tabacum* chloroplast transgenic system, indicating the chloroplast genes have a certain particularity in codon usage. These analyses simply focused on few codons, hence, there were certain limitations in their research results (Nakamura & Sugiura, 2007; Nakamura & Sugiura, 2011). Furthermore, codon optimization for exogenous genes based on the sequence information of *psbA* genes from 133 plants significantly improved the expression efficiency of exogenous genes in transgenic systems of plant chloroplast (Kwon et al., 2016). However, the usage of continuously distributed rare codons might lead to low expression levels or premature termination as previously described by Pan et al. (2013). In this study, significant differences in codon usage frequency was observed for two codons (CGA and AGC) in six plants with four model organisms (Table S2). When the chloroplast genomes of six plants is transformed into *Saccharomyces cerevisiae*, the ratio of codon (CGA) usage frequency was more than 4.00 (Table S2). This difference was probably the main factor affecting the low conversion rate of six plants, followed by premature termination of translation. In order to overexpress the target gene and improve the expression efficiency when verifying the functional genes of six *Euphorbiaceae* plant species, codon usage bias analyses are required. With the rapid development and application of the third generation of gene editing technology, i.e., CRISPR/Cas9 (Ma et al., 2016), the expression efficiency of Cas9 gene in these chloroplast genomes will be improved through substituting 17 codons which are used at a relatively low frequency in synonymous codons (Table S1).

### Sources analysis of variation in codon usage

#### ENc-plot

**Figure 1:**
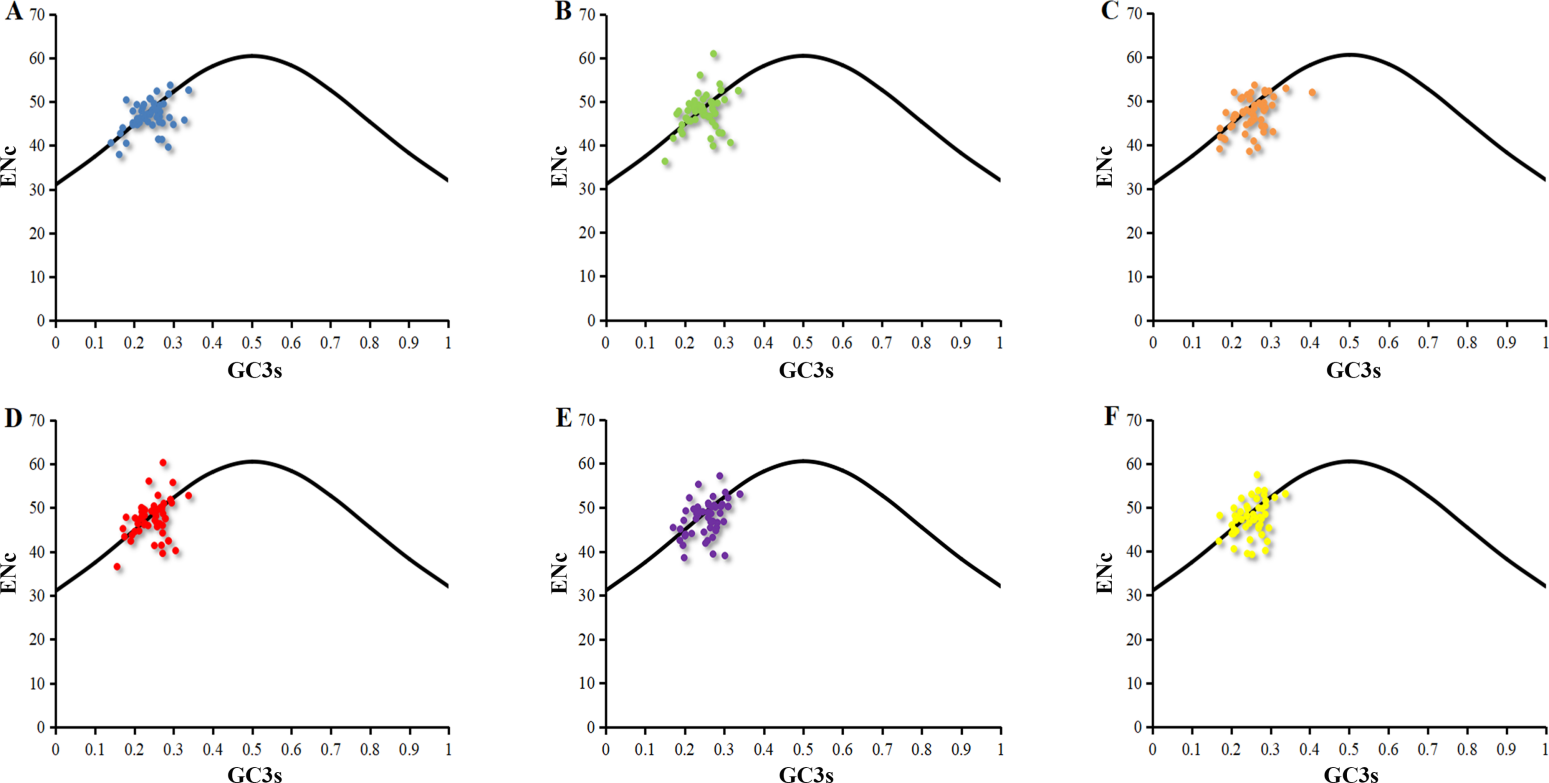
ENc-plot of chloroplast genomes of six *Euphorbiaceae* plant species. (A) *Euphorbia esula*; (B) *Hevea brasiliensis*; (C) *Jatropha curcas*; (D) *Manihot esculenta*; (E) *Ricinus communis*; (F) *Vernicia fordii*.

The distributions of ENc and GC3s of chloroplast genomes of six *Euphorbiaceae* plant species were similar (Fig. 1). Only few points lie in close proximity to the curve, however, a majority of genes with lower ENc values than expected values lay below the curve (Fig. 1). Analysis about points indicated that codon usage bias of chloroplast genomes was affected slightly by the mutation pressure, but natural selection and other factors play the major role (Wright, 1990). Previous researches suggested the codon usage bias of chloroplast genomes of *Populus alba* (Zhou, Long & Li, 2008), *Poaceae* (Zhang et al., 2012), *Asteraceae* (Nie et al., 2013) were influenced by combined the effects of mutation pressure, natural selection and other factors.

#### PR2-plot

**Figure 2:**
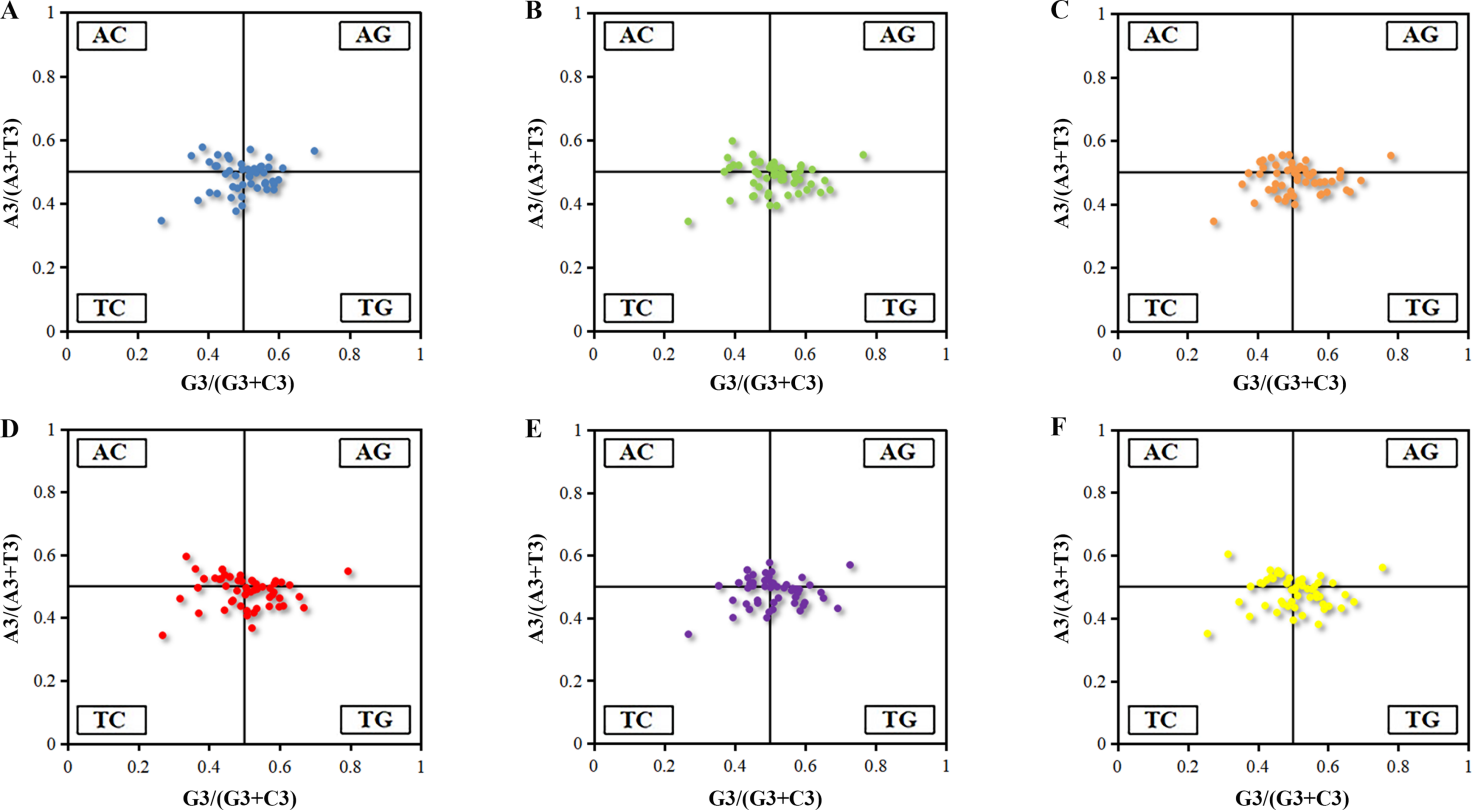
PR2-plot of chloroplast genomes six *Euphorbiaceae* plant species. (A) *Euphorbia esula*; (B) *Hevea brasiliensis*; (C) *Jatropha curcas*; (D) *Manihot esculenta*; (E) *Ricinus communis*; (F) *Vernicia fordii*.

It was an efficient way to reflect the mutation pressure by analyzing the points representing values for G3/(G3 + C3) and A3/(A3 + T3) distributed around the central spot (A = T, C = G). It was revealed that the AT-bias is 0.488, 0.485, 0.482, 0.485, 0.487 and 0.484 for *Euphorbia esula* (Fig. 2A), *Hevea brasiliensis* (Fig. 2B), *Jatropha curcas* (Fig.  2C), *Manihot esculenta* (Fig. 2D), *Ricinus communis* (Fig. 2E) and *Vernicia fordii* (Fig. 2F), while the GC-bias is 0.499, 0.507, 0.509, 0.499, 0.504 and 0.501, respectively. Thus, T/C bias at the third position of codons of chloroplast genes was observed in *Euphorbia esula* and *Manihot esculenta*, however, T/G-bias was observed in other four *Euphorbiaceae* plant species. As a whole, the usage frequency of A/T and G/C in six chloroplast genomes was unbalanced that was not only affected by the mutation pressure, but also with natural selection and other factors. Similar studies have also been reported for the codon usage of chloroplast genomes of *Asteraceae* (Nie et al., 2013) which illustrated that purines were used more frequently than pyrimidine in the chloroplast of *Asteracceae*. The analysis of PR2-plot only reflected the factors that influenced codon usage pattern, hence, further analyses are needed to explore the extent of the influencing factors between mutation pressure and natural selection.

#### Neutrality plot

**Figure 3:**
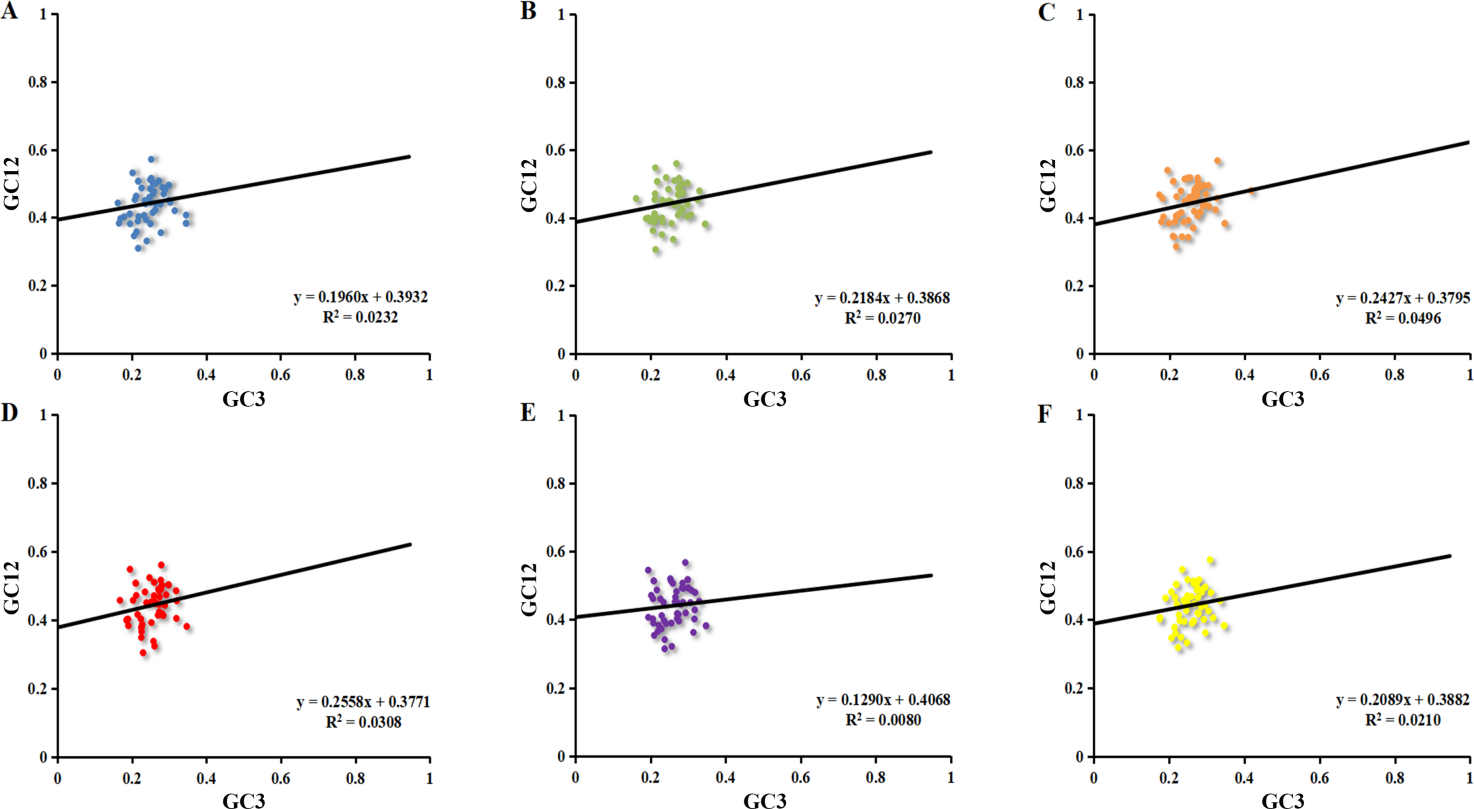
Neutrality plot of chloroplast genomes six *Euphorbiaceae* plant species. (A) *Euphorbia esula*; (B) *Hevea brasiliensis*; (C) *Jatropha curcas*; (D) *Manihot esculenta*; (E) *Ricinus communis*; (F) *Vernicia fordii*.

The neutrality plot reflected the narrow range of GC12 (0.30–0.58) and GC3 (0.16–0.42) value distributions (Fig. 3). The correlation between GC1 and GC2 was very strong (*r*_1_ = 0.530, *r*_2_ = 0.493, *r*_3_ = 0.542, *r*_4_ = 0.511, *r*_5_ = 0.559, *r*_6_ = 0.538, *p* <0.01). However, no significant correlation was found for GC1 with GC3 (*r*_7_ = 0.143, *r*_8_ = 0.092, *r*_9_ = 0.138, *r*_10_ = 0.106, *r*_11_ = 0.030, *r*_12_ = 0.070) or GC2 with GC3 (*r*_13_ = 0.123, *r*_14_ = 0.194, *r*_15_ = 0.257, *r*_16_ = 0.199, *r*_17_ = 0.129, *r*_18_ = 0.184), which indicated mutation pressure had a minor effect on the codon usage bias. Moreover, the slope of neutrality plot revealed that mutation pressure only accounted for 12.90%–25.58% on the codon usage patterns in six chloroplast genomes while natural selection accounted for 74.42%–87.10%. These results demonstrated that natural selection played a significant role in the codon usage patterns.

#### Correspondence analysis (COA)

**Figure 4:**
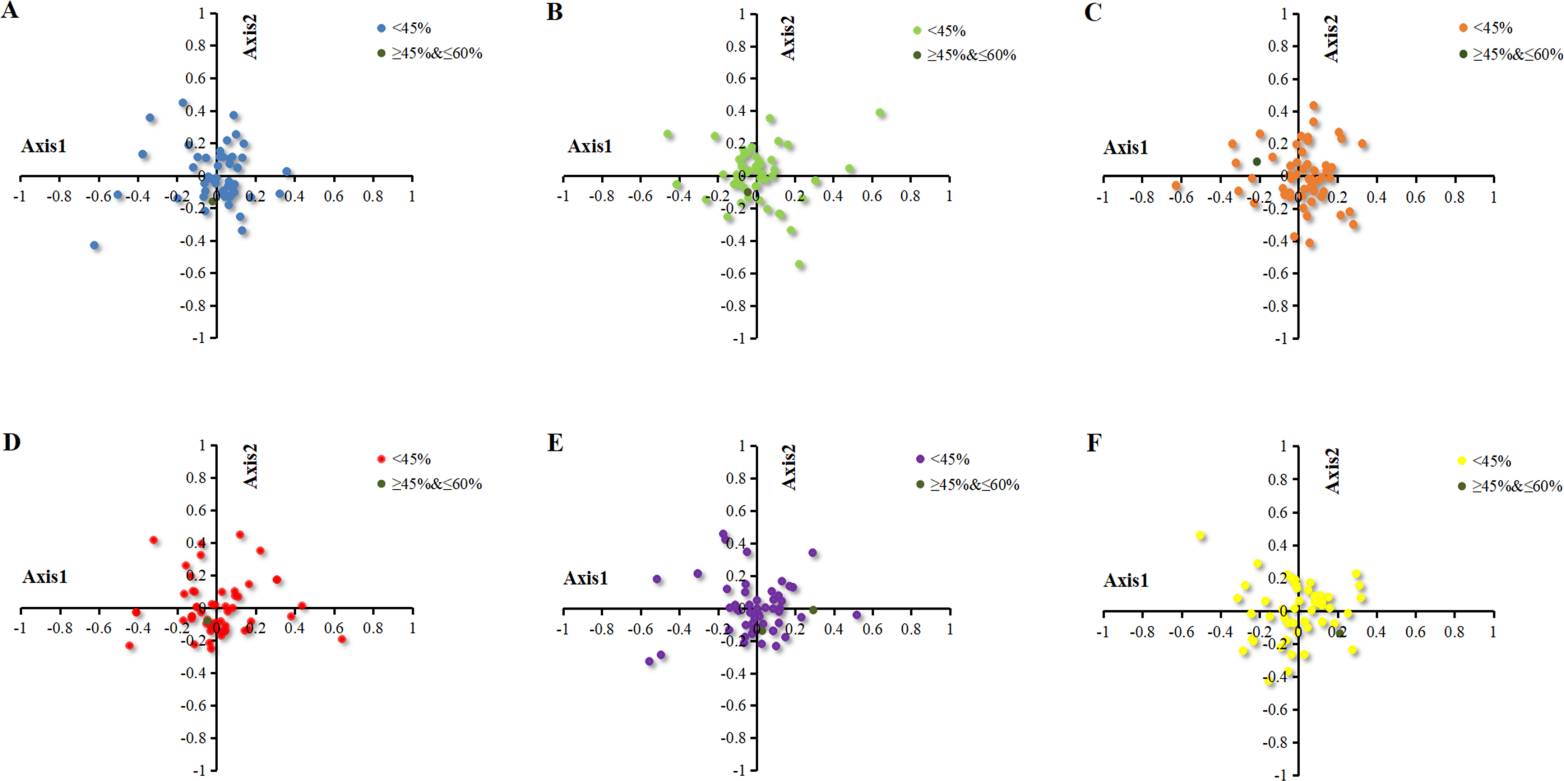
Correspondence analysis of chloroplast genomes of six *Euphorbiaceae* plant species. (A) *Euphorbia esula*; (B) *Hevea brasiliensis*; (C) *Jatropha curcas*; (D) *Manihot esculenta*; (E) *Ricinus communis*; (F) *Vernicia fordii*.

Correspondence analysis is a multivariate statistical method to explore the relationship between the variables in samples (Shields & Sharp, 1987). In the current study, the correspondence analysis based on RSCU was used to reveal the main factors affecting the formation of codon usage patterns in the chloroplast genomes of six *Euphorbiaceae* plant species. The position of the origin represented the average RSCU value for all genes, with respect to axis 1 and axis 2. The first four axes accounted for 36.36%, 35.97%, 33.64%, 35.38%, 36.03% and 34.17% of the overall variation. The first axis accounted for 11.09%, 11.55%, 10.17%, 11.74%, 11.68% and 9.70% of the total variation in six plants respectively. Therefore, axis 1 was the major source of variation, responsible for ∼10% of total variation. This indicated that the codon usage might be not affected by the single factor. To investigate the effects of GC content on CUB, each gene of chloroplast of six *Euphorbiaceae* plant species was distributed on the plane with axis 1 as the abscissa and axis 2 as the ordinate axes with different colors (Fig. 4). There was only a gene with GC content within 45%–60% plotted as bottle green in six plants, while all the other genes with GC content were lower than 45%.

To identify the factors resulting in the dispersion of chloroplast genes along axis 1 and axis 2, the correlation coefficients were calculated on axis 1 with CAI, GC3s and L_aa (Table 2). Based on the correlation analysis of axis 1 and codon usage indices (CAI, GC3s, L_aa), it was found that axis 1 for *Euphorbia esula*, *Jatropha curcas* and *Manihot esculenta* had a significant correlation with GC3s (*p* ≤ 0.01), while *Hevea brasiliensis*, *Ricinus communis* and *Vernicia fordii* had correlation with GC3s (*p* ≤ 0.05), which indicated GC3s is significant for patterns of codon usage (Table 2). *Zhou* and colleagues (2008) reported that the axis 1 for the codon usage bias of chloroplast genome of *Populus alba* significantly correlate with GC3s and gene length, which is in line with the findings of Xu et al. (2011), who reported that axis 1 was significantly correlated with GC3s, gene length and hydrophilicity in the chloroplast genome of the *Oncidium Gower Ramsey*, suggesting the effect of mutation, gene length and expression level.

**Table 2:** Correlation analysis of axis 1 and codon usage index of chloroplast genomes of six *Euphorbiaceae* plant species. CAI means codon adaptation index; GC3s indicates the GC content at the third codon position of synonymous codons; L_aa is defined as total number of amino acids.

	***Euphorbia esula***	***Hevea brasiliensis***	***Jatropha curcas***	***Manihot esculenta***	***Ricinus communis***	***Vernicia fordii***
CAI	−0.023	−0.114	−0.035	−0.185	0.152	−0.307^*^
GC3s	0.421^**^	−0.320^*^	−0.581^**^	−0.352^**^	0.284^*^	0.324^*^
L_aa	0.165	−0.059	−0.020	−0.059	0.068	0.216

**Notes.**

* *P* < 0.05.

** *P* < 0.01.

## Conclusions

The analysis of codon usage bias revealed that codons encoding proteins tended to use A/T in chloroplast genomes of six *Euphorbiaceae* plant species. RSCU analysis showed that the codons with positive bias in the genomes of six *Euphorbiaceae* plant species mostly ending with A/T. In addition, 17 identical high-frequency codons (GCT, TGT, GAT, GAA, TTT, GGA, CAT, AAA, TTA, AAT, CCT, CAA, AGA, TCT, ACT, TAT and TAA) of chloroplast genomes for six *Euphorbiaceae* plant species were sorted out. In the meanwhile, *Manihot esculenta* and *Ricinus communis* possess one more high-frequency codon (GTA) than other four *Euphorbiaceae* plant species. These results assist to optimize and modify codons, followed by further analyzing the relationship between chloroplast gene expression and codon usage bias in six *Euphorbiaceous* plant species. Moreover, natural selection played the dominant role over mutation pressure in the patterns of codon usage. *Arabidopsis thaliana*, *Populus trichocarpa* and *Saccharomyces cerevisiae* were considered as suitable exogenous expression receptor systems for chloroplast genes of six *Euphorbiaceae* plant species. Moreover, *Saccharomyces cerevisiae* is the best choice to be the exogenous expression receptor. The results of this study will increase our understanding of the codon usage patterns of chloroplast genomes in other plant species.

##  Supplemental Information

10.7717/peerj.8251/supp-1 Supplemental Information 1 CDSs (before processing) of six* Euphorbiaceae* plant species

10.7717/peerj.8251/supp-2 Supplemental Information 2 CDSs (after processing) of six* Euphorbiaceae* plant species

10.7717/peerj.8251/supp-3 Table S1 RSCU and RFSC values of the codons in chloroplast genomes of six *Euphorbiaceae* plant species **Notes:**The****bold indicates preference codons (RSCU > 1; (Sharp & Li, 1986)), the shaded portions indicate the lowest RFSC values of synonymous codons for each amino acid (low-frequency codons) and the border indicates high-frequency codons (Zhou, Tong & Shi, 2007).

10.7717/peerj.8251/supp-4 Table S1 Comparison of codon usage frequency between six *Euphorbiaceae* plant species and four organisms ** **Notes:** A: *Arabidopsis thaliana*; E: *Escherichia coli*; Ee: *Euphorbia esula*; Hb: *Hevea brasiliensis*; Jc: *Jatropha curcas*; Me: *Manihot esculenta*; P: *Populus trichocarpa*; Rc: *Ricinus communis*; S: *Saccharomyces cerevisiae*; Vf: *Vernicia fordii*. The shadow indicates ratios ≤ 0.5 or ≥ 2.

## Additional Information and Declarations

### Data Availability

The following information was supplied regarding data availability: The data is available at NCBI: NC_033910.1, NC_015308.1, NC_012224.1, NC_010433.1, NC_016736.1, NC_034803.1
