# Supplementary material for: Comparative analysis of codon usage patterns in chloroplast genomes of six Euphorbiaceae species
Source: PeerJ. 2020 Jan 6;8:e8251. doi: 10.7717/peerj.8251 (PMC6951282; doi:10.7717/peerj.8251)
Supplement: Supplemental Information 1 [file peerj-08-8251-s001.docx]

**1. *Euphorbia esula***

>lcl|NC_033910.1_cds_YP_009348332.1_1 [gene=rps12] [locus_tag=B2L33_pgp041] [db_xref=GeneID:31082722] [protein=ribosomal protein S12] [exception=trans-splicing] [protein_id=YP_009348332.1] [location=complement(join(104232..104257,104794..105025,75634..75747))] [gbkey=CDS]

ATGCCAACTATTAAACAACTTATTAGAAACACAAGACAGCCAATTAGAAATGTTACCAAATCGCCCGCTCTTGGGGGATGTCCTCAACGCCGAGGAACATGTACTAGGGTGTATACTATCACCCCCAAAAAACCAAACTCTGCCTTACGTAAAGTTGCCAGAGTACGATTAACCTCTGGATTTGAAATCACTGCTTATATACCTGGTATTGGCCATAATTTACAAGAACATTCTGTAGTCTTAGTAAGAGGGGGAAGGGTTAAGGATTTACCCGGTGTGAGATATCACATTGTTCGAGGAACCCTAGATGCTGTCGGAGTAAAGGATCGTCAACAAGGGCGTTCTAAATATGGGGTCAAAAAGCCAAAATAA

>lcl|NC_033910.1_cds_YP_009348333.1_2 [gene=rps19] [locus_tag=B2L33_pgp001] [db_xref=GeneID:31082724] [protein=ribosomal protein S19] [protein_id=YP_009348333.1] [location=18..296] [gbkey=CDS]

GTGACACGTTCACTAAAAAAAAATCCTTTTGTAGCAAATCATTTATTAAAAAAAATAAATAAGCTTAACACAAAAGCAGAAAAAGAAATAATAGTAACGTGGTCCCGGGCATCTACCATTATACCTACAATGATCGGCCATACTATTGCTATCCATAATGGAAAGGAGCATTTACCTATTTATATAACAGATCGTATGGTGGGCCATAAATTGGGAGAATTTGCACCTAAAAATTGCAAACAAATTTTTTTTACCGAAAATTTTTATTTACCTATTTGA

>lcl|NC_033910.1_cds_YP_009348334.1_3 [gene=psbA] [locus_tag=B2L33_pgp085] [db_xref=GeneID:31082725] [protein=PsbA] [protein_id=YP_009348334.1] [location=complement(1130..2191)] [gbkey=CDS]

ATGACTGCAATTTTAGAGAGACGCGAAAGCGAAAGCCTATGGGGTCGTTTCTGTAACTGGATAACCAGCACTGAAAACCGTCTTTACATTGGATGGTTTGGTGTTTTGATGATCCCTACTTTATTGACCGCAACTTCTGTATTTATTATCGCTTTCGTTGCTGCCCCTCCGGTAGATATTGATGGTATTCGTGAACCTGTTTCTGGATCTCTACTTTATGGCAACAACATTATTTCTGGTGCCATTATTCCTACTTCTGCGGCTATAGGTTTGCATTTTTACCCAATATGGGAAGCGGCATCTGTTGATGAGTGGTTATACAATGGCGGTCCTTATGAGTTAATTGTCCTACACTTCTTACTTGGTGTAGCTTGTTACATGGGTCGTGAGTGGGAACTTAGTTTCCGTCTAGGTATGCGCCCGTGGATTGCTGTTGCATATTCAGCTCCTGTTGCAGCTGCTACTGCTGTTTTCTTGATCTATCCAATTGGTCAAGGAAGCTTTTCTGATGGTATGCCTCTAGGAATCTCTGGTACTTTCAACTTTATGATTGTATTCCAGGCTGAGCACAACATCCTTATGCACCCATTTCACATGTTAGGCGTAGCTGGTGTATTCGGCGGCTCCCTATTCAGTGCTATGCATGGTTCCTTGGTAACCTCTAGTTTGATCAGGGAAACCACAGAAAATGAATCTGCTAACGAAGGTTACAGATTCGGTCAAGAGGAAGAAACTTATAATATCGTAGCCGCTCATGGTTATTTTGGCCGATTGATCTTCCAATATGCTAGTTTCAACAATTCTCGTTCTTTACATTTCTTCTTAGCTGCTTGGCCTGTAGTAGGCATTTGGTTCACTGCTTTAGGTATTAGCACTATGGCTTTCAACTTAAATGGTTTCAATTTCAACCAATCTGTAGTTGATAGTCAAGGTCGTGTAATTAATACCTGGGCTGATATTATTAACCGTGCTAACCTTGGTATGGAAGTTATGCATGAACGTAATGCTCATAACTTCCCTCTAGACCTAGCTGCTATTGAAGCTCCATCTACAAATGGGTAA

>lcl|NC_033910.1_cds_YP_009348335.1_4 [gene=matK] [locus_tag=B2L33_pgp084] [db_xref=GeneID:31082726] [protein=maturase K] [protein_id=YP_009348335.1] [location=complement(2775..4286)] [gbkey=CDS]

ATGCAAAGATATTTCGACCTAAATAGATCTCGAAAAAGCGATTTCCTATACCCATTTATCTTTCGGGAGTATATTTATACATTTGCTCATGATCATAGTTTAAATAGATCTATTTTGTTGCAAAATGCAAGTTATGACAAAAAGTTGAGTTTATTAATTGTAAAACGTTTAATTACTCGAATCTATGAACAGAATCATTTGATTATTTCTGCTAATGATTACAACCAAAATCTGTTTTTTAGGTATAACAAGAATTTGTACTATCAAATGGTATCAGAGGGCTTCGCAGTTATTGTGGAAATTCCATTTTCCAGACGATTAGTATCTTCTTTAGAAAGGCCAGAGATAGTAAAATCTAATAAATTACGATCAATTCATTCAATATTTCCTTTTTTAGAGGACAAGTTTCCACATTTAAATTATGTGTCAGATGTATTAATACCTTACCCCATCCATCTCGAAAAATTGGTTCAAACCCTTCGTTATTGGGTGAAAGATCCTTCTTCTTTACATTTTTTACGAGTCTTTCTTCATCAGTATTCGAATTTGAGCAGTCTTATTATTCCAAAGAAATCAATTTCTTTTTTTCGAAAAAGTAATCCAAGGTTTTTCTTGTTCCTATATAATTCTCATATAAATGAATATGAATCTATCTTATTTTTTCTCCGTAATCAGTCCTTTCATTTACGATCAACATTTTCTCGAGTTTTTCTTGAACGAATTTTTTTCTATGGAAAAATAGAACATTGTGCAGAAGTTTTTGCTAATGATTTTCAGAACATTCTAGTGTTGTTCAAGGATCCTTTCATGCATTATGTTAGATATCACGGAAAATCTATTCTCGCTTTAAAAGATAAACCCTTTCTGATGAAAAAATGGCAATATTACCTTATCAATTTATGTCAATGTCATTTTTATGTCTGGTTTCACCCAAAAAAGATCTATATAAATTCATTATCAAAAAATTCTCTCAACTTTTTTGGCTATCTTTCAAGTGTACAAAAGAATCCTTTGGTAGTACGGAGTCAAATGCTAGAAAATTCATATCTCATAGATAAAGAGAATACTATGAAGAACCTCGATACAATAATTCCAATTAATCCTTTAATTGTATTATTGTCAAAAATGCAATTTTGTAACGCAGTGGGACATCCTATTAGTAAACCGATTCGGGCTCATTTATCAGATTCTGATATTATCGACCAATTTGCGCGTATATGTCGAAATTTTTCTCATTTTTATAGCGGATCCTCAAAAAAAAAGAGTTTGTATCGTATAAAATATATACTTCGACTTTCGTGTGTTAAAACTTTGGCCCGTAAACACAAAAGTACTGTACGCGCTTTTTTGAAAAGATTAGGTTCGGAATTATTAGAAGAATTTTTTACGGAGGAAGAAGAGATTCTTTCTTTGATCTTCCCAAAAGTTTCTTCTATTTCGCGCAAGTTATATAGAAGACGAATTTGGTATTTGGATATTATTTCTATCAATGATTTGGCACATCATGAATAA

>lcl|NC_033910.1_cds_YP_009348336.1_5 [gene=psbK] [locus_tag=B2L33_pgp083] [db_xref=GeneID:31082727] [protein=PsbK] [protein_id=YP_009348336.1] [location=8569..8754] [gbkey=CDS]

ATGCTTAATAGTTTTAGTTTAATTTGTATCTGTCTTAATTCTGCCCTTTATTCAAGCAATTTTTTCTTCACAAAATTGCCTGAAGCCTATGCTTTTTTGAATCCAATCGTAGATGTTATGCCAGTAATCCCTCTACTCTTTCTTCTATTAGCCTTTGTTTGGCAAGCTGCTGTAAGTTTTCGATGA

>lcl|NC_033910.1_cds_YP_009348337.1_6 [gene=psbI] [locus_tag=B2L33_pgp082] [db_xref=GeneID:31082774] [protein=PsbI] [protein_id=YP_009348337.1] [location=9135..9245] [gbkey=CDS]

ATGCTTACTCTTAAACTCTTTGTTTACACAGTAGTTATATTCTTTGTTTCTCTCTTCATCTTTGGGTTTTTATCTAATGATCCTGGACGTAATCCTGGACGTGAAGAATAG

>lcl|NC_033910.1_cds_YP_009348338.1_7 [gene=ATPA] [locus_tag=B2L33_pgp081] [db_xref=GeneID:31082729] [protein=AtpA] [protein_id=YP_009348338.1] [location=complement(12017..13540)] [gbkey=CDS]

ATGGTAACCATTCGAGCCGACGAGATTAGTAATATTATTCGCGAACGTATTGAGCAATATAATAGGGAAGTAAAGATTGTAAATACTGGTACCGTACTTCAAGTAGGCGACGGCATTGCTCGTATTTATGGCCTTGATGAAGTAATGGCAGGTGAATTAGTAGAATTTGAAGAGGGTACAGTAGGCATTGCTCTGAATTTGGAATCAAATAATGTCGGTGTGGTATTAATGGGTGATGGTTTAATGATACAAGAGGGAAGCTCCGTAAAGGCAACAGGAAGAATTGCTCAGATACCGGTGAGTGAAGCTTATTTGGGTCGTATTGTAAATGCCCTGGCTAAACCTATTGACGGTCGAGGGGAAATTTCCGCTTCTGAATCTCGGTTAATTGAATCTCCCGCTCCAGGTATTATTTCGAGACGTTCCGTATATGAGCCTCTTCAAACAGGACTTATTGCTATTGATTCGATGATCCCTATAGGACGTGGGCAACGAGAATTAATTATTGGGGACAGACAAACCGGTAAAACAGCAGTAGCCACAGATACAATTCTGAATCAACAAGGACAAAATGTAATATGTGTTTATGTAGCTATTGGGCAAAAAGCGTCTTCTGTGGCTCAGGTAGTGACTACATTACAGGAAAGAGGAGCAATGGAGTACACTATTGTGGTAGCCGAAACTGCGGATTCTCCGGCTACATTACAATACCTGGCTCCTTATACAGGAGCAGCTCTGGCTGAATATTTTATGTACCGTGAACGACACACTCTAATCATTTACGATGATCTCTCCAAACAAGCGCAGGCTTATCGCCAAATGTCTCTTCTATTACGAAGACCACCAGGTCGTGAAGCTTATCCAGGAGATGTCTTTTATTTGCATTCACGCCTTTTGGAAAGAGCTGCTAAATCAAGTTCTCGTTTAGGTGAAGGAAGCATGACTGCTTTACCAATAGTCGAGACTCAATCAGGAGACGTTTCAGCTTATATTCCTACTAATGTAATTTCCATTACAGACGGACAAATATTCTTATCCGCCGATCTATTCAATGCTGGAATCCGCCCTGCTATTAATGTGGGGATTTCCGTTTCTAGAGTAGGATCTGCAGCTCAAATTAAAGCTATGAAACAAGTGGCGGGTAAGTTAAAATTGGAATTGGCCCAATTCGCCGAATTAGAGGCCTTTGCACAATTCGCTTCTGATCTAGATAAAGCTACTCAGAATCAATTGGCAAGAGGTCAGCGATTACGTGAGTTGCTCAAACAATCCCAATCTGCGCCTCTCACTGTGGAGGAACAGATAATGACTATTTATACCGGAACAAATGGTTATCTTGATTCATTAGAAATAGGACAAGTAAGGAAATTTCTCGTTGAGTTACGTACCTACTTAAAAACGAATAAACCCCAGTTCGAAGAAATCATATCTTCTACCAAAACATTCACCGAAGAAGCAGAAATCCTTTTGAAAGAAGCTATTCAGGAGCAGAAGGAACGTTTTCTAGTTCAGGAACAAGTATAA

>lcl|NC_033910.1_cds_YP_009348339.1_8 [gene=ATPF] [locus_tag=B2L33_pgp080] [db_xref=GeneID:31082776] [protein=AtpF] [protein_id=YP_009348339.1] [location=complement(join(13587..14056,14723..14867))] [gbkey=CDS]

ATGAAAAATGTAACCGATTCTTTCGTTTTCCTGGGTCACTGGCCATCCGCCGGGAGTTTCGGGTTTAATACCGATATTTTCGCAACAAATCCAATAAATCTAAGCGTAGTCCTTGGTGTATTGACTTTTTTTGGAAAGGGGGTGTTTTGGTTCGGGAAGGGATCATGGAAGTTTTGCAATGAATGGAAAGATAATCTACTTTCATTAAGTGATTTATTAGATAATCGAAAACAACGGATTTTGGATACTATTCGAAATTCAGAAGAACTACGCGAGGGGGCCATTGAACAGCTGGAAAAAGCCCGGGCCCGCTTACGGAAAGTGGAAATAGAAGCGGATCTGTTTCGAACGAATGGATACTCTGAGATAGAACGAGAAAAATTGAATTTGATTAATTCAACTTATAAGACTTTGGAACAATTAGAAAATTACAAAAATGAAACCATTCAGTTTGAACAACAAAGAACGATTAATCAAGTCCGACAACGGGTTTTCCAACAAGCCTTACAAGGAGCTCTAGGAACTCTGAATAGTTGTTTGACCAACGAATTACATTTACGTACCATCAACGCGAATCTTGGCATGTTTGGGGCGATAAAAGAAATAACTGATTAG

>lcl|NC_033910.1_cds_YP_009348340.1_9 [gene=ATPH] [locus_tag=B2L33_pgp079] [db_xref=GeneID:31082777] [protein=AtpH] [protein_id=YP_009348340.1] [location=complement(15283..15528)] [gbkey=CDS]

ATGAATCCATTGATTTCTGCCGCTTCTGTTATTGCTGCTGGGTTGGCTGTTGGGCTTGCTTCTATTGGACCTGGGGTTGGTCAAGGTACTGCCGCGGGGCAAGCTGTAGAAGGTATCGCAAGACAACCCGAGGCAGAGGGAAAAATACGAGGTACTTTATTGCTTAGTCTGGCTTTTATGGAAGCTTTAACAATTTATGGATTAGTTGTAGCCTTAGCACTTTTATTTGCGAATCCTTTTGTTTAA

>lcl|NC_033910.1_cds_YP_009348341.1_10 [gene=ATPI] [locus_tag=B2L33_pgp078] [db_xref=GeneID:31082778] [protein=AtpI] [protein_id=YP_009348341.1] [location=complement(16465..17208)] [gbkey=CDS]

ATGAATGTTCTATCATGTTCCATCAACACCCTAAAAGGGTTATATGATATATCTGGTGTGGAAGTAGGCCAGCATTTCTATTGGAAAATTGGAGGTTTCCAAGTCCACGCCCAAGTACTTATTACTTCTTGGGTTGTAATTGCTATCTTATTAGGTTCAGCCATTGTAGCTGTTCGGAACCCCCAAACCATTCCAACTGGCGGTCAGAATTTCTTCGAATATGTCCTTGAATTCATTCGAGATGTGAGCCAAACTCAGATCGGAGAGGAATACGGCCCATGGGTCCCTTTTATTGGAACTATGTTTCTATTTATTTTTGTTTCTAATTGGGCGGGTGCACTTTTACCTTGGAAGATCATAGAGTTACCTCATGGGGAGTTGGCTGCACCTACGAATGATATAAATACTACCGTTGCTTTAGCTTTACTTACGTCAATAGCCTATTTTTATGCGGGCCTTAGCAAAAAAGGATTAGGTTATTTCAGTAAATACATTCAACCAACTCCAATTCTTTTACCCATTAACATTTTAGAAGATTTCACAAAACCTTTATCACTTAGCTTTCGACTTTTCGGAAATATATTAGCGGATGAATTAGTAGTTGTTGTTCTTGTTTCTTTAGTACCTTCAGTGGTTCCTATACCTGTCATGTTCCTTGGATTATTTACAAGTGGTATTCAAGCTCTTATTTTTGCAACTTTAGCTGCGGCTTATATTGGCGAATCCATGGAGGGGCATCATTAA

>lcl|NC_033910.1_cds_YP_009348342.1_11 [gene=rps2] [locus_tag=B2L33_pgp077] [db_xref=GeneID:31082779] [protein=ribosomal protein S2] [protein_id=YP_009348342.1] [location=complement(17455..18165)] [gbkey=CDS]

ATGAGAAAAAGATATTGGAACATTAATTTGGAAGAGATGATGAAAGCGGGAGTTCATTTTGGTCATGGTACTAGAAAATGGAACCCGAGAATGGCCCCTTATATCTCTGCAAAACGTAAAGGTATTCATATTACAAATCTTACTAGAACTGCTCGTTTTTTATCAGAAGCTTGTGATTTAATTTTCGATGCAGCAAGTAAGAGAAAACAATTCTTAATTGTTGGTACCAAAAATAAAGCAGCGGATTCAGTAGCGCGGGCTGCAATAAGGGCTCGGTGTCATTATGTTAATAAAAAATGGCTCGGCGGTATTTTAACGAATTGGTCCACTACAGAAACTAGACTTCAAAAGTTCAGGGACTTGAGAATAGAACAAAAGACAGGTAGACTCAACCGTCTTCCGAAAGGAGATGGGACTCGATTGAAGAGACAGTTAGCTCACTTACAAACATATCTGGGCGGTATTAAATATATGACAGGGTTACCCGATATTGTAATACTGGTTGATCAGCAAGAAGAATATACGGCTCTTCGGGAATGTATCACTTTGGGAATTCCAACCATTTGTTTAATTGATACAAACTGTGACCCGGATCTCGCAGATATTTCGATTCCAGCGAATGATGACGCTATAGCTTCAATCCGATTAATTCTTAATAAATTAGTATTTGCAATTTGTGAGGGTCGTTCTAGCTATATACGAAATTCCTGA

>lcl|NC_033910.1_cds_YP_009348343.1_12 [gene=rpoC2] [locus_tag=B2L33_pgp076] [db_xref=GeneID:31082780] [protein=RpoC2] [protein_id=YP_009348343.1] [location=complement(18436..22626)] [gbkey=CDS]

ATGGAGGTACTTATGGCCGAGCGGGCCAATCTGGTCTTTCACAATAAAGTGATAGATGGAACTGCCATTAAACGACTTATTAGCAGATTAATAGATCATTTTGGAATGGCATATACATCCCACATCCTAGATCAAGTAAAGACTCTGGGTTTCCAGCAAGCCACTGCTACATCCATTTCATTAGGAATTGATGATCTTTTAACAATACCTTCTAAGGGATGGCTAGTCAAAGATGCTGAACAACAAAGTTTGGTTTTGGAAAAACACTATCATTATGGAAATGTACACGCGGTAGAAAAATTACGCCAATCTATTGAGATATGGTATGCTACAAGTGAATATTTGCGACAAGAAATGAATCTTAATTTTAGGATGACGGAACCCTTTAATCCAGTCCATATAATGTCTTTTTCGGGAGCTAGGGGAAATGCATCTCAAGTACACCAATTAGTTGGTATGAGAGGATTAATGTCGGATCCACAAGGGCAAATGATTGATTTACCCATTCAAAGCAATTTACGCGAAGGACTGTCTTTAACAGAATATATCATTTCGTGTTATGGAGCCCGAAAAGGGGTTGTCGATACTGCTGTACGAACATCAGATGCTGGATATCTTACGCGTAGACTTGTTGAAGTAGTTCAACATATTGTTGTACGTAGAAAAGATTGTGGCACTACCCGAGGGATCCCTGTGAGTCCTCGAAATGGCATGATGTCGGAAAGAGTTTTTATTCAAACATTAATTGGTCGTGTAATAGCAGACAATATATATATGGGTCTACGATGTATTGCCATTCGAAATCAAGATATTGGGATTGGACTTGCGAATCGATTCATAACCTTTCGAACACAAACAATATGTATTCGAACTCCCTTTACTTGTAAGAGTACGTCTTGGATCTGTCGATTATGTTATGGCCGGAGTTCTACTCACGGCGATCTAGTAGAATTGGGAGAAGCCGTAGGTATTATTGCGGGTCAATCCATTGGGGAGCCAGGTACTCAACTAACATTAAGAACGTTTCATACTGGCGGAGTATTCACAGGTGGTACTGCAGAACACGTACGATCCCCGTCTAATGGAAAAATAAAATTTAATGAGGATTTGGTTCATCCCATACGTACACGTCATGGGCATCCTGCGTTTCTATGTTATATAGACTTGTATGTAACTATTGAGAGTCAAGATATTCTACATAATGTGCCTATTCCACACAAAAGTTTCCTTTTAGTTCAAAATGATCAATATGTAGAATCAGAACAAGTAATTGCTGAAATTCGGACGGGCACATACACTTTGAATTTTAAAGAAAAGGTTCGAAAACATATTTATTCCGATTCAGAAGGGGAAATGCACTGGAGTACTCATGTATACCATGCGCCCGAATTTACATACAGTAATGTCCATCTCTTACCAAAAACAAGCCATTTATGGATATTATCAGGATGTTCGTACAGATCCGGTATAGTTCCTTTTTCACTACACAAGGATCAAGACCAAATGAACGTTCATTCTCTTTCTGCCAAAAGAAAAGATAGTTCTAGTCCTTCCCTAAACAATCATCTCCTAAATAATAATCAAGTTAAACACCAATTCTTTAGTTCAGATTTAGTGGGTAAAAAAGAAAGTAGGATTCCTGATTATTCAGTAGTTAATCGAATCATATGTACTGGTCATTGTAATCTCATATATCCTGCTATTCTCTACGAGAATTCTGATTTATTGGCAAAGAGGCGAAGAAATAAATTCATTATTCCATTCCAATCAATTCAAGAACGAGAGAAAGAACAAATGACCTACTACCCTATCTCGATTGAAATACCTAAAAATGGTATTTTCCGTAGAAATAGTGTTTTTTCTTATTTCGACGATCCCCAATACCGAAGAAAGAGTTCAGGAATTACTAAATACGGCGCTATAGGGGTCCATTCAATCGTCAAAAAAGAAGATTTGATTGAGTATCGGGGAGTCAAAGAATTTAAGCCAAAATACCAAATGCAAGTGGATCGCTTTTTTTTCATTCCCGAGGAAGTGTATATTTTACCCGAATCTTCTTCCCTAATGGTACGGAACAATAGTATCGTTGGAGTAGATACACAAATCACTTTAAATACAAGAAGTCGAGGGGGCGGATTGGTCCGAGTGGAGAGAAAAAAAAAAAAAATAGAACTTAAAATCTTTTCTGGAGGTATCCATTTTCCGGGAGAGACAGATAAGATATCCCGACATAGTGGTATCTTGATACCACCAGGAACGGTAAAAACAAATTATAAGGAATCAAAAAAAGTCAAAAATTGGATCTATATTCAACGAATCACACCTACCAAAAAAAAGTATTTTGTTTTGGTTCGACCAGTAATCATATATGAGATAGCGAATGGTATCAATTTAGAAACACTTTTCCCCCAGGATCTATTGCAGGAAAAGGATAATCTGAAACTTCGAGTTGTCAATTATATTCTTTATGGAAATAGTAAACCAATTTGGGGAATTTCTGACACAAGTATTCAATTAGTTCGTACTTGTTTAGTGTTGAATTGGGACCAAGACAAAAAAGGTTCTTCTATCGAGGAGGCCCGCGCTTCTTTTGTTGAAGTAAGCACAAATGGTCTGATTCGTGATTTCCTAAGAATCAATCTATTGAAATCAAAAATTTCATATATCAGTAGTAGAACTACAAAAAGGAATGATCCATCAGGTTCAGGACCGATCTCTAATAATGGATCAGATCGCACCAATATTAATCCATTTTATCCCAGTTATTCCAAGACAAGGATTCAACAATCACTTAAACAAAATCACGGAACTTTTAGTACGTTGTTGAATAGAAATAAGGAATGTCAATCTTTCCTAATTTTGTCATCATCTAATTGTTTTCGAATGGATCCATTCAATGATGTAAAACATCACAATGGAATAAAAGAATCAATTAAAAGAGATCCTATAATTTCAATTAGAAATTCGTTTGGCCCTTTAGGAACAGCCCTTCAAATTGCGAATTTTTTTTTATTAAAACATTTCAATTTAATAACTCATAATCAGATCTCGATAACTAAATATTTGAAACTTGACAATTTAAAACAGACTTTTCAAATAATTAAATATTATTTAATGGATGAAACCGGGCGAATTGTTAATCCTGATCCATGCAGTAAGAGTGTTTTGAATCCATTCACTTTGAATTGGTATTTTCTCCATCATAATTATCATCGTAATTATTGTGAATCTTTCTTCACAATAATTAGACTGGGACAATTTATTTGTGAAAATTTATGTATGGCCAAAAGAGGACCACATCTAAAATCGGGTCAAGTTATAATTGTTCACATTGACTCTGTAGTAATAAGATCGGCTAAGCCTTATTTGGCCACTCCAGGAGCAACTGTTCATGGCCATTATGGAGAAATCCTTTACGAAGGAGATACGTTAGTTACATTTATATATGAAAAATCGAGATCTGGTGATATAACGCAGGGTCTTCCAAAAGTGGAACAAGTGTTAGAAGTGCGTTCAATTGATTCAATATCGATAAACCTAGAAAAGAGAGTTGAGGGTTGGAACGAGTGTATAACAAGACTTCTTGGAATTCCTTGGGGATTCTTGATTGGTGCTGAGTTAACTATAGTGCAAAGTCGTATCTCGTTGGTTAACAAAATCCAAAAAGTTTATCGATCTCAAGGAGTGCAGATACATAATAGGCATATAGAAATTATTGTACGGCAAATAACATCAAAAGTATTGGTTTTAGAAGATGGAATGTCTAATGTTTTTTTACCCGGAGAACTAATTGGATTGTTCCGAGCAGAACGAACGGGACGCGCTTTGGAAGAAGCCATCTGTTACCGACCCATATTATTGGGAATAACGAGAGCATCTCTGAATACTCAAAGTTTCATATCTGAGGCCAGTTTTCAAGAAACTGCTCGCGTTTTAGCAAAAGCTGCTCTCCGCGGTCGTATCGATTGGTTGAAAGGCCTAAAAGAAAACGTTGTTCTAGGCGGTATGATACCCGTTGGTACCGGATTCAAAAGATTCGTGCAAGGCTCAAAGAAACAGAAAAACATGCCTTTGAAAAGCAAAAATAAAAATTTTTTTGAGGAGGAATTTAGAGATAGAGATCTTTTATTCCACCACAGAGAGTTATTTGATTCTTGCATTTCAAGAAATTTCTATGATACATTAGAATAA

>lcl|NC_033910.1_cds_YP_009348344.1_13 [gene=rpoC1] [locus_tag=B2L33_pgp075] [db_xref=GeneID:31082781] [protein=RpoC1] [protein_id=YP_009348344.1] [location=complement(join(22771..24387,25163..25594))] [gbkey=CDS]

ATGATTGATCGGTATAAACATCAACAACTCCGAATTGGCTCAGTTTCGCCGCAACAAATAAGTGCTTGGGCCAATAAAATCCTACCTAACGGAGAGATTGTTGGAGAGGTGACAAAACCCTACACTTTTCATTACAAAACCAATAAACCTGAAAAAGATGGATTATTTTGTGAAAGAATTTTTGGGCCTATAAAAAGTGGAATTTGTGCTTGTGGAAATTATCGAGTAATCAAAAATGAAAAAGAAGACCGAAAATTTTGTGAACAATGCGGGGTCGAATTTGTTGATTCTCGGATACGAAGATATCAAATGGGCTACATCAAACTGGCATGCCCCGTAACTCATGTGTGGTATTTGAAACGTCTTCCTAGTTATATCGCGAATCTTTTAGATAAACCTCTTAAAGAATTAGAAGGCCTAGTATACTGCGATTTTTCTTTTGCTAGGCCCATAGCTAAGAAACCCACTTTTTTACGATTACGAGGTTCATTTCAACATGAAATCCAATCCTGGAAATACAGTATTCCACTTTTTTTTACTACCCAAGGCTTCGATACATTTCGAAATCGAGAAATTTCTACAGGAGCTGGTGCTATCCGAGAACAATTAGCTGATCTGGATTTGCGAATTATTATAGATTGTTCATCGGTAGAATGGAAAGAATTAGGGGAAGAAGGGCCCACAGGGAATGAATGGGAAGATCGAAAGGTTGGAAGAAGAAAAGATTTTTTAGTTAGACGCGTGGAATTAGCTAAGCATTTTATTCGAACAAATATACAACCAGAATGGATGGTTTTATGTCTATTACCTGTTCTTCCTCCCGAGTTGAGACCTATCATTCAGATAGATGGGGGTAAACTGATGAGTTCCGATATTAATGAACTCTATAGAAGAGTTATCTATCGGAACAATACTCTTATTGATCTATTAACAACAAGTAGATCTACGCCAGGGGAATTAGTAATGTGTCAAGAGAAGTTGGTACAAGAAGCCGTGGATACACTTCTTGATAATGGAATCCGCGGACAACCAATGAGGGACGGTCATAATAAAGTTTACAAGTCGTTTTCGGATGTAATTGAAGGAAAAGAAGGAAGATTTCGTGAGACTATGCTTGGCAAACGGGTTGATTATTCGGGTCGTTCTGTCATTGTCGTAGGCCCCTCACTTTCATTACATCGATGTGGATTGCCTCGCGAGATAGCAATAGAGCTTTTCCAGATATTTGTAATTCGTGGTCTAATTAGACAGCATCTTGCTTCGAACATAGGAGTTGCTAAGAGTAAAATTCGGGAAAAAGAACCAATTGTATGGGAAATACTTCAGGAAGTTATGCAGGGGCATCCGGTATTACTGAATAGAGCGCCGACTCTGCATAGATTAGGCATCCAGGCATTCCAACCCATTTTAGTGGAAGGCCGTGCTATTTGTTTACATCCATTAGTTTGTAAGGGATTCAACGCAGACTTTGATGGGGATCAAATGGCTGTTCATGTACCTTTATTGTTGGAAGCTCAAGCGGAGGCTCGTTTACTTATGTTTTCTCATATGAATCTCCTGTCTCCAGCTATTGGAGATCCCATTTCCGTACCAACTCAAGATATACTTATTGGGCTCTATGTATTAACAAGCGGAAATCGTCGAGGTATTTGTGAAAATAGGTATAATCCATTTAATCTCCAAAAGTCTCAAAATCAAAGAATTGCCAACAATAACGAAAAATATACAAAAGAACCCTTTTTTTCTAATTCTTATGGTGCAATTGGTGCTTATCGGCAGAAACGAATCAATTTAGATAGTGCTTTGTGGCTCCGATGGCAACTAGATCAACGCACTATTGCTTCAAGAGAAGCTCCCATCGAAGTTCACTATGAATCTTTGGGTACCTATCATGAGATTTATGAACACTATCTAATAGTAAGAAATATAAAAAAAGAAATTCTTTGTATATACATTCGAACTACTGTTGGTCATATTTCTCTTTATCGCGAAATCGAAGAAGCTATACAAGGGTTTTGCCAAGCCTCCTCAGATGGTATCGGTATCTAA

>lcl|NC_033910.1_cds_YP_009348345.1_14 [gene=rpoB] [locus_tag=B2L33_pgp074] [db_xref=GeneID:31082782] [protein=RpoB] [protein_id=YP_009348345.1] [location=complement(25621..28833)] [gbkey=CDS]

ATGCTCGAGGATGGAAATGATAGAATGTCTACAATACCTGGATTTAATCAGATACAATTTGAAGGATTTTGGAGGTTCATTGATCAGGGCTTAACAGAAGAGTTTTCTAAGTTTCCAAAAATTGAAGATACAGATCAAGAAATTGAATTTCAATTATTTGTGGAAACATATCAATTAGTCGAACCATTGATAAAAGAAAGAGATGCTGTATATGAATCACTTACATATTCTTCTGAATTATATGTATCCGCGGGATTAATTTGGAAAAACAGTAGGGATATGCAAGAACAAACAATTTTTATTGGAAACATTCCTCTAATGAATTCCCTAGGAACTTTTCTAATAAACGGAATATACAGAATTGTAATCAATCAAATATTGCAAAGTCCAGGTATTTTTTACCGCTCCGAGTTGGATCATAACGGAATTTCGGTCTATATCGGGACTATAATATCAGATTGGGGAGGGAGAATAGAATTAGAGATTGATAGAAAAGCAAGGATATGGGCCCGCGTGAGTAGGAAACAGAAAATATCTATTCTAGTTCTATCATCAGCTATGGGTTTGAATCTACGACAAATTTTAGAGAATGTGTGTTACCCTGAGATTTTCTTAGCTTTCCTGAATGATAAAGAAAAAAAAAAAATTGGATCAAAAGAAAATGCCATTTTGGAGTTTTATCAACAATTTACTTGTGTAGGCGGAGATCCAGTATTTTCTGAATCCTTATGTAAGGAATTACAAAAGAAATTCTTTCAACAAAGATGTGAATTAGGAAGGATTGGTCGATTAAATATGAACCGGAGACTTAATCTTGATATACCTCATAACAATACATTTTTGTTACCAAGAGATATATTGGCAGCTGCGGATCGTTTGATTGAAATGAAATTTGGAATGGGTACACTTGACGATATGAATCATTTAAAAAATAAACGTATTCGTTCCGTAGCGAATCTCTTACAAGATCAATTCGGATTAGCCCTGATTCGTTTAGAAAATGTGGTTAGGGGGACTATATGTGGAGCAATTAGGCACAAATTGATACCGACCCCTCAAAATTTGGTAACTTCAACTCCATTAACAACCACTTATGAATCTTTTTTTGGATTACACCCATTATCTCAAGTTTTGGATCGAACTAATCCATTGACACAAATAGTTCATGGGAGAAAATCGAGTTATTTGGGTCCTGGAGGATTAACAGGACGAACTGCTAGTTTTCGAATACGAGATATCTACCCCAGTCACTATGGGCGCATTTGCCCCATTGACACGTCTGAAGGAATCAATGTTGGACTTATTGGATCTTTAGCAATTCATGCCAAGATTGATCATTGGGGGTCTTTAGAAAGCCCATTTTATGAAATTTCTGAGGGATCAAAAAAAGTACGGATGTTTTATTTATCACCAAATAGCGAGGAATACTATATGTTAGCTGCAGGAAATTCCTTGGCGCTGAATCGAGGTGTTCAGGAAGAACAGGTTGCGCCAGCTCGATATCGTCAAGAATTCCTGACTATTGCATGGGAACAGGTCCATCTTCGAAGTATTTTTCCCTTCCAATATTTTTCTATTGGAGCTTCCCTCATTCCTTTTATCGAGCATAATGATGCGAATCGGGCTTTAATGAGTTCGAATATGCAACGTCAAGCAGTTCCACTTTCTCGGTCCGAAAAATGCATTGTTGGAACTGGATTGGAACGCCAAGTGGCTCTAGATTCAGGGGTTCCTGCTATAGCCGAACACGAGGGAAAGATAATTTATACTGATATTGACAAGATCATTTTATCGGGCAATGGCAATACTCTACGCATTCCATTAGTTATGTATGAACGTTCTAACAAAAATACTTGTATGCATCAAAAAACCCAGGTTCAGCAGGGTAAATGTATTAAAAAAGGACAAGTTTTAGCGGATGGTGCCGCTACCGTTGGTGGCGAACTCGCCTTGGGCAAAAACGTATTAGTAGCTTATATGCCATGGGAAGGTTACAATTTTGAAGATGCAGTACTCATTAGCGAACGTCTGGTATATGAAGATATTTATACTTCTTTTCACATACGTAAATATGAAATTCAGACTCATGTGACAAGCCAAGGACCTGAAAGGATCACTAACGAAATCTCGCATCTAGAAGCCCATTTACTCCGAAATTTAGACAAAAATGGAATTGTGATGCTGGGATCTTGGGTAGAGGCAGGCGATATTTTAGTAGGTAAATTAACACCTCAAATAGCGAAAGAATCATCGTATGCTCCAGAAGATAGATTATTAAGAGCCATACTTGGTATTCAAGTATCTACTTCAAAGGAAACTTGTCTAAAATTACCTATAGGTGGTAGGGGTCGAGTTATTGATGTGAGATGGATCCAGAGAAAGGGGGGTTCCTGTTCTAATCCAGAAATGATTCGTGTATATATTTTACAGAAACGTGAAATCAAAGTGGGTGATAAAGTAGCTGGAAGACATGGAAATAAAGGCATCATTTCAAAAATTTTGCCTAGACAAGATATGCCTTATTTGCAAGATGGAAGACCTGTTGATATGGCCTTCAACCCATTAGGAGTACCTTCACGAATGAATGTAGGACAGATATTTGAATGCTCACTCGGGTTAGCGGGAGGTCTGCTAGATAGACATTATCGAATAGCACCCTTTGATGAGAGATATGAACAAGAGGCTTCGAGAAAACTAGTGTTTTCTGAATTATATGAAGCCAGTAAGCAAACAGCAAATCCGTGGGTATTTGAACCCGAGTATCCAGGAAAAAGTAGAATATTTGATGGAAGAACAGGAGATCCTTTTGAGCAGCCTGTTATAATAGGGAAGCCCTATATCTTGAAATTAATTCATCAAGTTGATGATAAAATACATGGCCGTTCCAGTGGACATTATGCACTTGTTACACAACAACCCCTTAGAGGAAGGGCCAAGCAAGGGGGACAACGGGTCGGAGAAATGGAGGTTTGGGCTCTAGAGGGATTTGGTGTTTCTCATATTTTACAAGAAATGCTTACTTATAAATCTGATCATATTAGAGCTCGCCAAGAAGTGCTTGGTACTATGATCATTGGAGGAACAATACCTAAACCTGAAGATGCTCCAGAATCTTTTCGATTGCTCGTTCGAGAACTACGATCTTTGGCTCTAGAATTGAATCATTTCCTTGTATCTGAGAAGAACTTCCAGATTAATAGGAAGGAAGCTTAA

>lcl|NC_033910.1_cds_YP_009348346.1_15 [gene=petN] [locus_tag=B2L33_pgp073] [db_xref=GeneID:31082730] [protein=PetN] [protein_id=YP_009348346.1] [location=30534..30623] [gbkey=CDS]

ATGGATATAGTAAGTCTAGCTTGGGCTGCTTTAATGGTAGTCTTTACATTTTCCCTTTCACTCGTAGTATGGGGGAGAAGTGGACTCTAG

>lcl|NC_033910.1_cds_YP_009348347.1_16 [gene=psbM] [locus_tag=B2L33_pgp072] [db_xref=GeneID:31082784] [protein=PsbM] [protein_id=YP_009348347.1] [location=complement(31757..31861)] [gbkey=CDS]

ATGGAAGTAAATATTCTTGCATTTATTGCTACTGCATTGTTCATTCTAGTTCCTACTGCTTTTTTACTTATAATATACGTAAAAACAGTTAGTCAAGGTGATTAA

>lcl|NC_033910.1_cds_YP_009348348.1_17 [gene=psbD] [locus_tag=B2L33_pgp071] [db_xref=GeneID:31082735] [protein=PsbD] [protein_id=YP_009348348.1] [location=36051..37112] [gbkey=CDS]

ATGACTATAGCCCTTGGTAAATTTACCAAAGACGAAAATGATTTATTTGATATTATGGATGACTGGTTACGGAGGGACCGTTTCGTTTTTGTAGGTTGGTCCGGTCTATTGCTCTTTCCTTGTGCCTATTTCGCCTTAGGGGGTTGGTTCACAGGTACAACCTTTGTAACCTCATGGTATACCCATGGATTGGCCAGTTCTTATTTGGAAGGCTGCAACTTCTTAACCGCCGCAGTTTCTACTCCTGCTAATAGTTTAGCACATTCTTTGTTATTACTATGGGGTCCTGAAGCCCAAGGAGATTTTACTCGTTGGTGTCAATTAGGCGGTTTGTGGACTTTTGTTGCTCTACACGGCGCTTTCGGGCTAATAGGTTTTATGTTACGTCAATTTGAACTTGCTCGATCCGTGCAATTGAGACCTTATAATGCAATCGCATTCTCTGGTCCAATTGCTGTTTTTGTTTCTGTATTCCTGATTTATCCACTAGGGCAGTCTGGTTGGTTTTTTGCACCTAGTTTTGGTGTAGCAGCTATATTTCGATTCATCCTCTTTTTCCAAGGGTTTCATAACTGGACGCTGAACCCATTTCATATGATGGGAGTTGCTGGCGTATTGGGCGCTGCCCTCTTATGCGCTATTCATGGCGCTACTGTGGAAAAGACTTTATTTGAAGATGGTGATGGTGCCAATACATTCTGTGCTTTTAACCCAACTCAAGCTGAAGAAACTTATTCAATGGTCACCGCTAACCGCTTTTGGTCTCAAATCTTTGGGGTTGCTTTTTCCAATAAACGTTGGTTACATTTCTTTATGTTATTTGTACCAGTAACTGGTTTATGGATGAGCGCTCTTGGAGTAGTCGGTCTGGCTCTGAATCTACGTGCCTATGACTTCGTTTCTCAGGAAATCCGTGCAGCGGAAGATCCTGAATTTGAGACTTTCTACACTAAAAATATTCTCTTAAACGAAGGTATTCGTGCTTGGATGGCGGCTCAAGATCAGCCTCATGAAAACCTTATATTCCCTGAGGAGGTTCTACCACGTGGAAACGCTCTTTAA

>lcl|NC_033910.1_cds_YP_009348349.1_18 [gene=psbC] [locus_tag=B2L33_pgp070] [db_xref=GeneID:31082786] [protein=PsbC] [protein_id=YP_009348349.1] [location=37060..38481] [gbkey=CDS]

ATGAAAACCTTATATTCCCTGAGGAGGTTCTACCACGTGGAAACGCTCTTTAATGGAACTTTATCTTTAGCCGGTCGTGACCAAGAAACCACGGGTTTCGCTTGGTGGGCCGGGAATGCTCGACTTATCAATTTATCCGGTAAACTTCTGGGAGCTCATGTAGCTCATGCTGGATTAATCGTATTCTGGGCCGGAGCAATGAACCTATTTGAAGTGGCTCATTTTGTACCGGAGAAACCAATGTATGAACAAGGATTAATTTTACTTCCCCACCTAGCTACTCTAGGTTGGGGGGTAGGTCCTGGTGGGGAAGTTCTAGACACCTTTCCATACTTTGTATCGGGTGTACTTCACTTAATTTCCTCTGCAGTATTGGGCTTTGGCGGCATTTATCATGCACTTCTGGGTCCTGAGACTCTTGAAGAATCTTTTCCTTTTTTTGGTTATGTATGGAAAGATAGAAATAAAATGACAACAATTTTAGGTATTCACTTAATCTTGCTAGGTATAGGTTCTTTTCTTCTAGTATTCAAGGCTCTTTATTTTGGGGGTGTATACGATACCTGGGCTCCGGGGGGTGGGGATGTAAGAAAAATTACCAACTTGACCCTTAGCCCAAGTGTTATTTTTGGTTATTTACTAAAATCCCCCTTTGGCGGAGAAGGATGGATTGTTAGTGTGGACGATTTGGAAGATATAATTGGGGGGCATGTATGGTTAGGTTCTATTTGTATACTTGGTGGAATCTGGCATATCTTAACCAAACCCTTTGCATGGGCTCGCCGTGCACTTGTATGGTCTGGAGAGGCTTACTTATCTTATAGTTTAGCCGCTTTATCCGTTTTTGGTTTCATTGCTTGTTGCTTTGTCTGGTTCAATAATACCGCTTATCCTAGTGAGTTTTACGGGCCTACCGGACCAGAAGCTTCTCAAGCTCAAGCTTTTACTTTTCTAGTTAGAGATCAACGTCTTGGGGCTAATGTGGGATCCGCTCAAGGACCTACTGGGTTAGGTAAATATTTAATGCGTTCTCCTACCGGAGAAGTTATTTTTGGAGGAGAAACTATGCGTTTTTGGGATCTGCGTGCTCCTTGGTTAGAACCTCTAAGAGGTCCGAATGGTTTGGACTTGGGTAGGTTGAAAAAAGACATACAACCTTGGCAAGAACGCCGTTCCGCGGAATATATGACCCACGCGCCTTTAGGTTCATTAAATTCTGTAGGTGGCGTAGCTACCGAGATCAATGCAGTCAATTATGTCTCTCCTCGAAGTTGGTTAGCTACCTCTCATTTTGTTCTAGGTTTCTTCCTATTCGTAGGTCATTTATGGCACGCGGGGAGGGCTCGTGCAGCTGCAGCAGGATTTGAAAAAGGAATTGATCGTGATTTTGAACCCGTTCTCTCCATGACTCCTCTTAATTAA

>lcl|NC_033910.1_cds_YP_009348350.1_19 [gene=psbZ] [locus_tag=B2L33_pgp069] [db_xref=GeneID:31082736] [protein=PsbZ] [protein_id=YP_009348350.1] [location=39153..39341] [gbkey=CDS]

ATGACTATTGCTTTCCAATTGGCTGTTTTTGCATTAATTGCTACTTCATCAATCTTACTGATTAGTGTACCCGTTGTTTTTTCTTCTCCTGATGGGTGGTCGAGTAACAAAAATGTTGTATTTTCCGGTACATCGTTATGGATTGGATTAGTCTTTCTGGTAGGTATCCTTAATTCTCTCATCTCTTGA

>lcl|NC_033910.1_cds_YP_009348351.1_20 [gene=rps14] [locus_tag=B2L33_pgp068] [db_xref=GeneID:31082738] [protein=ribosomal protein S14] [protein_id=YP_009348351.1] [location=complement(40697..40999)] [gbkey=CDS]

ATGGCAAGAAAAAGTTTGATTCAACGGGAGAATAAGAGGCAAAAATTGGAACAAAAATATCATTTGATTCGGCGATCCTCAAAAAAAGAAATAAGCAAAGTTCTGTCCTTGAGTGATAAATGGGAAATTCATGGAAAGTTACAATCCCCACCGCGAAATAGTGCACCGACACGTCTTCATCGACGTTGTTTTTCAACTGGAAGACCGAGAGCTAACTATCGAGACTTTAGGCTATCTGGCCACATACTTCGTGAAATGGTTCATGCATGTTTGTTACCGGGAGCAACAAGATCGAGTTGGTAA

>lcl|NC_033910.1_cds_YP_009348352.1_21 [gene=psaB] [locus_tag=B2L33_pgp067] [db_xref=GeneID:31082789] [protein=PsaB] [protein_id=YP_009348352.1] [location=complement(41118..43322)] [gbkey=CDS]

ATGGCATTAAGATTTCCAAGGTTTAGCCAAGGCTTAGCTCAGGACCCCACTACTCGTCGTATTTGGTTTGGTATTGCTACCGCGCATGACTTTGAAAGTCATGATGATATTACGGAGGAACGTCTTTATCAGAACATTTTTGCTTCTCACTTCGGGCAATTAGCAATAACTTTTTTGTGGACTTCTGGAAATCTCTTTCATGTGGCTTGGCAAGGAAATTTTGAAGCATGGGTACAGGACCCTTTACATGTAAGACCCATTGCTCATGCAATTTGGGATCCTCATTTTGGTCAACCAGCCGTAGAAGCTTTTACTCGAGGGGGTGCCCCTGGACCAGTGAATATCGCTTATTCGGGTGTTTATCAATGGTGGTATACAATCGGTTTACGTACTAATGAAGATCTTTATATTGGAGCTCTTTTTCTATTATTTCTTTCTGCCCTAGCCTTACTCGCGGGTTGGTTACACCTACAACCGAAATGGAAACCGAGCGTTTCATGGTTCAAAAATGCCGAATCTCGTCTCAATCATCATTTGTCAGGGCTATTTGGAGTAAGTTCTTTGGCTTGGACAGGACACTTAGTCCATGTTGCTATTCCTGGCGCCAGGGGGGAATACGTTCGATGGAATAATTTATTAGATGTATTACCACATCCCCAAGGATTAGGCCCACTTTTGACAGGTCAATGGAATCTTTATGCTCAAAATCCCGATTCAGGTAGTCATTTATTTGGTACCTCCCAAGGAGCAGGAACTGCCATTCTAACCCTTCTCGGGGGGTTCCATCCACAAACACAAAGTTTATGGCTGACCGATATTGCACACCATCATTTAGCTATTGCGTTTATTTTTCTCGTTGCCGGTCATATGTATAGAACTAACTTCGGGATTGGGCACAGTATAAAAGATCTTTTAGAAGCACATATTCCTCCGGGGGGGCGATTGGGACGTGGACATAAGGGTCTTTATGACACAATCAACAATTCGCTTCATTTTCAATTAGGTCTTGCTCTAGCGTCTTTAGGGGTTATTACGTCCTTAGTAGCTCAACACATGTACTCATTACCTGCTTATGCGTTCATAGCGCAAGACTTTACTACTCAAGCTGCGTTATATACTCATCACCAATACATCGCAGGATTCATCATGACAGGAGCTTTTGCTCACGGAGCTATATTTTTTATTAGAGATTACAATCCGGAACAGAATGAGAATAATGTATTGGCAAGAATGTTAGACCATAAAGAAGCTATCATATCCCATTTAAGTTGGGCCAGCCTCTTTCTGGGATTCCATACGTTAGGACTTTATGTTCATAATGATGTCATGCTTGCTTTTGGTACTCCGGAGAAACAAATCTTGATCGAACCCATATTTGCCCAATGGATACAATCTGCTCACGGCAAAACTTCATATGGGTTCGATGTACTTTTATCTTCAACGAATAGTCCAGCCTTTAATGCAGGTCGAAGCATATGGTTGCCCGGTTGGTTAAATGCTATTAATGCAAATAGTAATTCATTATTCTTAACAATAGGGCCTGGCGACTTCTTGGTTCATCATGCTATTGCTCTAGGTTTACATACAACCACATTGATACTAGTAAAAGGTGCTTTAGATGCACGTGGTTCGAAGTTAATGCCAGATAAAAAAGATTTTGGTTATAGTTTTCCTTGCGATGGTCCGGGGCGCGGTGGTACTTGTGATATTTCGGCTTGGGACGCATTTTATTTGGCGGTTTTTTGGATGTTAAATACTATTGGCTGGGTTACTTTTTATTGGCATTGGAAGCACATCACATTATGGCAAGGTAATGTTTCACAGTTTAATGAATCTTCCACTTATTTGATGGGATGGTTAAGAGATTATCTATGGTTAAACTCTTCACAACTTATCAATGGATATAACCCTTTTGGTATGAATAGCTTATCGGTCTGGGCATGGATGTTCTTATTTGGACATCTTGTTTGGGCTACTGGATTTATGTTTTTAATTTCTTGGCGTGGATATTGGCAGGAATTAATCGAAACTTTAGCATGGGCTCATGAACGTACACCTTTGGCTAATTTGATTCGATGGAGAGATAAACCAGTAGCTCTTTCCATCGTGCAAGCAAGATTGGTTGGATTAGCCCACTTTTCTGTAGGTTATATCTTCACTTATGCGGCTTTCTTGATTGCCTCTACATCAGGTAAATTTGGTTAA

>lcl|NC_033910.1_cds_YP_009348353.1_22 [gene=psaA] [locus_tag=B2L33_pgp066] [db_xref=GeneID:31082790] [protein=PsaA] [protein_id=YP_009348353.1] [location=complement(43348..45600)] [gbkey=CDS]

ATGATTATTCGTTCGCCGGAACCAGAAGTAAAAATTTTGGTAGATAGGGATCCCATCAAAACTTCTTTTGAGGAATGGGCCAGACCCGGTCATTTCTCAAGAACAATAGCTAAAGGACCTGATACTACCACTTGGATCTGGAACCTACATGCTGATGCTCACGATTTCGATAGCCATACCAATGATTTGGAGGAAATTTCTCGAAAAGTATTTAGTGCTCATTTTGGCCAACTCTCCATCATCTTTCTTTGGCTGAGTGGGATGTATTTCCACGGTGCTCGTTTTTCAAATTATGAAGCATGGCTAAGCGATCCTACTCACATTGGACCTAGTGCCCAAGTGGTTTGGCCAATAGTGGGCCAAGAAATATTGAACGGTGATGTAGGCGGGGGTTTCCGAGGAATACAAATAACCTCTGGTTTTTTTCAGATTTGGAGAGCATCTGGAATAACTAGTGAATTACAACTGTATTGTACCGCAATTGGTGCATTGGTCTTTGCAGCCTTAATGCTTTTTGCTGGTTGGTTCCATTATCACAAAGCTGCTCCAAAATTGGCTTGGTTCCAAGATGTAGAATCCATGTTGAATCACCATTTAGCGGGGCTACTAGGACTTGGGTCTCTTTCTTGGGCGGGACATCAAGTACATGTATCTTTACCAATTAACCAATTTCTAAACGCTGGAGTAGATCCTAAAGAAATCCCACTTCCTCATGAATTTATCTTGAATCGAGATCTTTTAGCTCAACTTTATCCCAGTTTTGCTGAGGGAGCAACTCCTTTTTTCACCTTAAATTGGTCAAAATATTCGGAATTTCTTACTTTTCGTGGAGGATTAGATCCAGTGACTGGAGGTCTATGGCTGACCGATATTGCACACCATCATTTAGCTATTGCAATTCTTTTTCTGATAGCGGGTCACATGTATAGGACTAACTGGGGTATTGGTCATGGTATAAAAGATATTTTAGAGGCTCATAAAGGTCCCTTTACAGGTCAGGGTCATAAAGGCCTATATGAAATCCTAACAACTTCATGGCATGCTCAATTATCTCTTAACCTAGCTATGTTAGGTTCTTTAACCATTGTTGTAGCTCACCATATGTATTCCATGCCCCCTTATCCATACCTAGCTACTGACTATGGTACACAATTGTCATTGTTCACACATCACATGTGGATTGGTGGATTCCTCATAGTTGGTGCTGCTGCGCATGCAGCCATTTTTATGGTAAGAGACTATGATCCAACTACTCGATACAACGATCTATTAGATCGTGTTCTTAGGCATCGTGATGCAATCATATCACATCTCAATTGGGTATGTATATTTTTAGGCTTTCACAGTTTTGGTTTATATATTCATAATGATACCATGAGCGCTTTAGGGCGACCTCAAGATATGTTTTCAGATACTGCTATACAATTACAACCCGTCTTTGCTCAATGGATACAAAACACTCATGCTTTAGCACCTAGTGCAACGGCTCCTGGTGCAACAGCAAGCACCAGTTTAACTTGGGGAGGTGTTGATTTAGTGGCAGTGGGTGGCAAGGTTGCTTTGTTACCGATTCCATTAGGAACCGCGGATTTTTTGGTCCATCACATTCATGCATTTACGATTCATGTGACGGTGTTGATACTTCTGAAAGGAGTTCTATTTGCTCGTAGCTCTCGTTTGATACCGGATAAAGCAAATCTTGGTTTTCGTTTTCCTTGTGATGGACCTGGAAGAGGGGGAACATGTCAAGTATCCGCTTGGGATCACGTCTTTTTAGGGCTATTTTGGATGTACAATTCAATTTCGATAGTCATATTCCATTTCAGTTGGAAAATGCAGTCAGATGTTTGGGGTAGTATAAGTGATCAAGGGGTGGTAACTCATATCACGGGAGGAAACTTCGCACAGAGTTCCATTACTATTAATGGTTGGCTCCGCGATTTCTTATGGGCACAGGCATCTCAGGTAATTCAGTCTTATGGTTCTTCATTATCTGCATATGGACTTTTTTTCCTAGGTGCTCATTTTGTATGGGCTTTTAGTTTAATGTTTCTATTCAGCGGTCGTGGTTATTGGCAAGAACTTATTGAATCAATTGTTTGGGCTCATAATAAATTAAAAGTTGCTCCTGCTACTCAGCCTAGAGCCTTGAGCATTATACAAGGACGTGCTGTAGGAGTAACTCATTACCTTCTGGGTGGAATTGCCACAACATGGGCGTTCTTCTTAGCAAGAATTATTGCAGTAGGATAA

>lcl|NC_033910.1_cds_YP_009348354.1_23 [gene=ycf3] [locus_tag=B2L33_pgp065] [db_xref=GeneID:31082791] [protein=Ycf3] [protein_id=YP_009348354.1] [location=complement(join(46660..46812,47488..47717,48465..48588))] [gbkey=CDS]

ATGCCTAGATCCCGGATAACTGGAAATTTTATTGATAAGACCTTTTCAATTGTAGCCAATATCTTATTACGAATAATTCCGACAACTTCGGGGGAAAAGGAGGCATTTACTTATTACAGAGATGGAATGTCTGCTCAATCCGAAGGAAATTATGCAGAAGCTTTACAGAATTATTATGAAGCTATGCGACTAGAAATTGATCCCTATGATCGAAGTTATATACTATATAATATAGGCCTTATTCACACAAGTAACGGAGAACACACAAAAGCTTTGGAATATTATTTTCGGGCACTAGAACGAAACCCTTTCTTACCACAAGCTTTAAATAATATGGCCGTGATCTGTCATTACCGAGGAGAACAGGCCATTCGGCAGGGAGATTCTGAAATTGCGGAGGCTTGGTTCGACCAAGCCGCGGAGTATTGGAAACAAGCCATAGCACTTACTCCCGGAAATTATATTGAAGCGCAGAATTGGTTGAAGATCACAAGGCGTTTCGAATAA

>lcl|NC_033910.1_cds_YP_009348355.1_24 [gene=rps4] [locus_tag=B2L33_pgp064] [db_xref=GeneID:31082739] [protein=ribosomal protein S4] [protein_id=YP_009348355.1] [location=complement(49402..50007)] [gbkey=CDS]

ATGGCACGTTACCGAGGGCCTCGTTTCAAAAAAATACGCCGTCTGGGGGCTTTACCGGGATTAACTAGTAAAAAGCCTAGAGCCGGGAGCGATCTTAGAAATCAATCACGCTCGGGGAAAAAATCTCAATATCGTATTCGTTTAGAAGAAAAACAAAAATTGCGTTTTCATTACGGTCTTACAGAACGACAATTACTTAAATACGTTCGTATCGCCGCAAAAGCTAAAGGTTCAACAGGTCGGGTTTTACTACAATTACTTGAAATGCGGTTGGATAACATCCTTTTTCGATTGGGTATGGCGTCAACTATTCCTCGAGCCCGCCAATTAGTTAATCATAGACATATTTTAGTTAATGGTCGTATAGTAGATATACCAAGTTATCGCTGCAAACCCCGAGATATTATTACAGCGAGGGATGAACAAAAATCTAGAGCTATGATTCAAAATTATCTCGCTTCATCTCCCCAGGAGGAATTGCCAAAACATTTGACTCTTCACCCATTTCAATATAAAGGATTGGTCAATCAAATAATAGATAGTAAATGGGTCGGCTTGAAAATAAATGAATTGCTAGTAGTAGAATATTATTCTCGTCAGACTTAA

>lcl|NC_033910.1_cds_YP_009348356.1_25 [gene=ndhJ] [locus_tag=B2L33_pgp063] [db_xref=GeneID:31082742] [protein=NdhJ] [protein_id=YP_009348356.1] [location=complement(52400..52876)] [gbkey=CDS]

ATGCAGGGTCCTTTGTCTGCTTGGCTAGTCAAACATGGACTAGTTCATAGATCTTTGGGTTTTGATTACCAAGGAATAGAGACTTTACAAATAAAGCCCGAAGATTGGCATTCCATTGCTGTCATTTTATATGTATATGGTTACAATTATTTGCGTTCACAATGTGCCTATGATGTAGCACCCGGCGGACTTTTAGCTAGCGTATATCATCTTACGAGAATAGAGTATGGTATAGATCAACCAGAAGAAGTATGTATAAAAGTATTTGTCCCAAGAAAGAATCCTAGAATTCCATCTGTTTTCTGGATTTGGAAAAGTGTGGATTTTCAAGAAAGGGAATCCTATGATATGCTGGGAATCGTTTATGAGAATCATCCACGTCTGAAACGTATCTTAATGCCGGAAAGTTGGATAGGGTGGCCCTTACGTAAGGATTATATTGCTCCCAATTTTTATGAAATACAAGATGCTCATTGA

>lcl|NC_033910.1_cds_YP_009348357.1_26 [gene=ndhK] [locus_tag=B2L33_pgp062] [db_xref=GeneID:31082794] [protein=NdhK] [protein_id=YP_009348357.1] [location=complement(52994..53671)] [gbkey=CDS]

ATGAATTCCATTGAGTTTCCCTTACTTGATCGAACAACTCAAATTTCCGTTATTTCAACTACATCAAATGATCTTTCAAATTGGTCAAGACTTTCCAGTTTATGGCCACTTCTCTATGGTACCAGTTGTTGCTTCATTGAATTTGCTTCATTAATAGGATCCCGATTCGACTTTGATCGTTATGGACTAGTACCAAGATCGAGTCCTAGACAAGCGGACTTAATTTTAACAGCCGGCACGGTAACCATGAAAATGGCTCCTTCTTTAGTAAGATTATATGAACAAATGCCTGAACCAAAATATGTTATTGCGATGGGGGCATGTACAATTACAGGAGGAATGTTCAGTACCGATTCTTATAGTACTGTTCGAGGAGTTGATAAGTTAATTCCTGTAGATGTCTATTTGCCAGGCTGTCCACCGAAACCGGAAGCGGTTATAGATGCTATAACAAAACTTCGTAAAAAAATATCTCGAGAAATTTCTGAGGATCGAATTAGATCTCAACCGGGGAATCGATGTTTTACTACCAATCACAAGTTTCATATTGAACGCAATACTCATACTGGAAATTATGATCAAGGATTCCTCTATCAACCGCCGTCTACTTCAAAGATCCCTCCTCAAACATTTTTCAAATATAAAAAGTCAGTATCGTCCATCGAATTAGTAAACTAG

>lcl|NC_033910.1_cds_YP_009348358.1_27 [gene=ndh3] [locus_tag=B2L33_pgp061] [db_xref=GeneID:31082795] [protein=Ndh3] [protein_id=YP_009348358.1] [location=complement(53729..54091)] [gbkey=CDS]

ATGTTTCTGCTTTACGAATATGATATATTCTGGGCGTTTCTAATAATATCAAGTGTTATTCCTATTTTAGCATTTCTAATTTCCGGAGTTTTATCCCCGATTAACACAGGTCCGGAGAAATTTTCTAGTTATGAATCGGGTATCGAACCAATAGGCGATGCTTGGTTACAATTTCGAATCCGTTATTATATGTTTGCTCTAGTTTTTGTTGTTTTTGATGTTGAAACAGTTTTTCTTTATCCATGGGCAATGAGTTTCGATATATTGGGGTTATCCGTATTTATAGAAGCTTTGATTTTCGTGCTTATCTTAATTGTTGGTTCAGTTTATGCATGGAGAAAGGGAGCATTAGAGTGGTCTTAG

>lcl|NC_033910.1_cds_YP_009348359.1_28 [gene=ATPE] [locus_tag=B2L33_pgp060] [db_xref=GeneID:31082745] [protein=AtpE] [protein_id=YP_009348359.1] [location=complement(56483..56884)] [gbkey=CDS]

ATGACCTTAAATCTTTGTGTACTGACCCCAAACCGAATTGTTTGGGATTCAGAAGTGAAAGAAATCATTTTATCTACTAATAGTGGACAAATTGGCGTATTACCAAATCATGCACCAATTGCCACAGCTGTCGATATCGGTATTTTGAGAATACGCCTTAATGACCAATGGTTAACAATGGCTCTGATGGGTGGTTTTGCTCGAATAGGCAATAATGAGATTACTGTTTTAGTAAATGATGCGGAGAAGGGTAGTGACATTGATCCACAAGAAGCTCAGCAAACTCTTGAAATCGCAGAAGCTAACTTGAGGAAAGCGGAAGGAAGGAGACAAATAATTGAGGCAAATCTAGCTCTCAGACGAGCTAGGGCACGAGTAGAGGCTATCAATGCAATTTCGTAA

>lcl|NC_033910.1_cds_YP_009348360.1_29 [gene=ATPB] [locus_tag=B2L33_pgp059] [db_xref=GeneID:31082797] [protein=AtpB] [protein_id=YP_009348360.1] [location=complement(56881..58377)] [gbkey=CDS]

ATGAGAATCAATCCTACTACTTCTGGTCCGGGAGTTTCGACGCTTGAAAAAAAGAACCTGGGGCGTATCGTTCAAATCATTGGGCCAGTACTAGATGTAGCTTTTCCCCCGGGCAAGATGCCTAATATTTACAACGCTTTAGTAGTTAAGGGTCGAGATACTGTCGGTCAAGAAATTAATGTGACTTGTGAAGTACAACAATTATTAGGAAATAATCGAGTTCGGGCTGTAGCTATGAGTGCTACAGATGGTCTAACGAGAGGAATGGAAGTGATTGACACAAAAGCTCCTCTAAGTGTTCCAGTCGGTGGGACGACTCTAGGACGAATTTTCAACGTGCTTGGAGAACCTATTGATGATTTAGGTCCTGTAGATACTCGTGCAACATCCCCTATTCATAGATCTGCACCTGCCTTTATACAGTTAGATACAAAATTATCTATTTTTGAAACAGGAATTAAAGTAGTAGATCTTTTAGCCCCTTATCGCCGTGGAGGAAAAATCGGACTATTCGGCGGGGCTGGAGTGGGTAAAACAGTCCTTATTATGGAATTAATCAACAACATTGCGAAAGCTCATGGGGGTGTATCTGTGTTTGGCGGAGTAGGTGAACGTACTCGTGAGGGAAATGATCTTTACATGGAAATGAAAGAATCTGGAGTAATTAATGAAAAAAATATTGCAGAATCAAAAGTAGCTCTAGTCTATGGTCAGATGAACGAACCGCCGGGAGCTCGTATGAGAGTTGGTTTGACTGCCCTAACTATGGCGGAATATTTCCGAGATGTTAATGAACAAGACGTACTTCTATTTATTGACAATATTTTCCGTTTCGTTCAAGCAGGATCCGAAGTATCGGCCTTATTGGGTAGAATGCCTTCCGCAGTGGGTTATCAACCTACCCTTAGTACCGAAATGGGTTCTTTACAAGAAAGAATAACTTCTACCAAAGAAGGGTCTATAACTTCTATTCAAGCCGTTTATGTACCTGCCGACGATTTGACTGACCCTGCTCCTGCCACCACATTTGCACATTTAGACGCTACTACTGTACTATCAAGAGGATTAGCCGCTAAAGGTATCTATCCAGCAGTAGATCCTTTAGATTCAACGTCAACTATGCTCCAACCTCAGATTGTTGGTGAGGAACATTATGAAACTGCGCAAAGAGTTAAGCAAACTTTACAACGTTACAAAGAACTTCAGGACATTATAGCTATCCTGGGGTTGGACGAATTATCCGAAGAAGATCGCTTAACTGTAGCAAGAGCGCGAAAAATTGAACGTTTCTTATCACAACCCTTTTTCGTAGCAGAAGTATTTACCGGTTCGCCGGGGAAATATGTCGGTCTAGCAGAAACAATTAGAGGGTTTAAATTGATCCTTTCCGGAGAATTAGATAGTCTCCCTGAACAGGCCTTTTATTTGGTAGGTAATATCGATGAAGCTACTGCGAAGGCTACGAACTTAGAAATGGAGAACAATTTGAAGAAATGA

>lcl|NC_033910.1_cds_YP_009348361.1_30 [gene=rbcL] [locus_tag=B2L33_pgp058] [db_xref=GeneID:31082798] [protein=ribulose-1,5-bisphosphate carboxylase/oxygenase large subunit] [protein_id=YP_009348361.1] [location=59162..60589] [gbkey=CDS]

ATGTCACCACAAACAGAGACTAAAGCAAGTGTTGGATTCAAGGCTGGTGTTAAAGATTATAAATTGACTTATTATACTCCTGAATATGAAACCAAAGATACTGATATCTTGGCAGCATTCCGAGTAACTCCTCAACCTGGAGTTCCGCCTGAGGAAGCAGGAGCTGCGGTAGCTGCTGAATCTTCTACTGGTACATGGACAACTGTGTGGACCGATGGGCTTACCAGTCTTGATCGTTATAAAGGACGATGCTACCACATCGAGCCCGTTGCTGGAGAAGAAAATCAATATATTGCTTATGTAGCTTACCCCTTAGACCTTTTTGAAGAAGGTTCTGTTACTAACATGTTTACCTCCATTGTAGGTAATGTATTTGGGTTCAAAGCCCTGCGCGCTCTACGTCTGGAGGATTTGCGAATCCCTCCTGCTTATACTAAAACTTTCCAAGGGCCGCCTCACGGCATCCAAGTTGAGAGAGATAAATTGAACAAGTATGGTCGCCCTCTATTGGGTTGTACTATTAAACCAAAATTGGGGCTATCCGCTAAGAATTACGGTAGAGCGGTTTATGAATGTCTTCGCGGTGGACTTGATTTTACCAAAGACGATGAGAACGTGAACTCTCAACCATTTATGCGTTGGCGAGACCGCTTTTTATTTTGTGCCGAATCCATTTTTAAATCACAGGCTGAAACAGGTGAAATCAAAGGGCATTATTTGAATGCTACTGCGGGTACATGCGAAGAAATGATCAAAAGGGCTGTATTTGCCAGGGAATTAGGAGTTCCTATCGTAATGCATGACTACTTAACAGGGGGATTCACGGCAAATACTAGCTTGGCTCATTATTGCCGAGATAATGGTTTACTTCTTCACATTCACCGCGCAATGCATGCAGTTATTGATAGACAGAAGAATCATGGTATACATTTTCGTGTATTAGCTAAGGCATTACGTATGTCTGGTGGAGATCATATTCACGCTGGTACCGTAGTAGGTAAACTTGAAGGAGAAAGAGAGATCACTTTGGGCTTTGTTGATTTACTGCGTGATGATTTTGTTGAAAAAGATCGAAGCCGCGGTATTTATTTCACTCAAGATTGGGTCTCTTTACCTGGTGTTCTTCCTGTAGCTTCAGGGGGTATTCACGTTTGGCATATGCCTGCTCTGACCGAGATCTTTGGAGATGATTCCGTACTACAATTCGGTGGAGGAACTTTAGGGCACCCTTGGGGAAATGCACCCGGTGCCGTAGCTAATCGAGTAGCTTTAGAAGCATGTGTACAAGCTCGTAATGAGGGACGTGATCTTGCTCGTGAGGGTAATGAAATTATTCGTGAGGCTGCAAAATGGAGTCCTGAACTAGCTGCTGCTTGTGAAGTATGGAAAGAGATTAAATTTGAATTCGAAGCAATGGATACTTTGTAA

>lcl|NC_033910.1_cds_YP_009348362.1_31 [gene=accD] [locus_tag=B2L33_pgp057] [db_xref=GeneID:31082799] [protein=AccD] [protein_id=YP_009348362.1] [location=61309..62808] [gbkey=CDS]

ATGGAAAAATGGTGGTTCAATTCAATCTTATCCAATGTGGAATTAGGATACAGGTGTAGGCTAAGTAAATCAATGGATAGTTTCAGTCCTTTTGAAAATACTAGTATAAGTGAAGACCCAATTCTAAATGATACAGATAAACACACCCATAGTTGGAGTAATAGTGACAACTCTAGTTCCAGTAATGTTGATCATTTAGTCGGTGTCAGGGACATTTGGAATTTCAGCGTTGATGAAACTTTTTTAGTTAAGGATAGTAATAGGGACAGTTATTCCATCTATTTTGATATTGAAAATAAAGTTTTTGAGATTGAGACTGATTATTCTTTTCTGGATGAACTAGAAAGTTCTTTTTATAGTTATTGGAATTCTAGTTATCTGAATAATGGGTCTAGGAGTGGCGACTCCCAATATGATCATTATATGTATGATACTAAATATCGTTGGAATAATTACATCAATAGTTGCATTGACCGTTATCTTCGCTCTCAAATTGGTATTGATAGTTCTATTTTAAGGGGTAGTAACCATTATAGCGAAAGTTATATTTATAGTTACGTTTGTGATGAAAGCGAAAATAGTAGTGAAAACAAGAGTGCCAGTCTAAGAATTAGCACGAATGGTAGTGATTTAACTCTAAGAGAAAGTTCTAATGATCTCGATATAACTCAAAAATACAAACATTTGTGGGTTCAATGCGAAAATTGTTATGGATTAAATTATAAGAAATTTTTTAAGTCAAAAATGAATCTTTGTGAACAATGTGGATATCATTTGAAAATGAGTAGTTCAGATAGAATTGAACTTTTGATTGACCCAGGGACTTGGGATCCTATGGATGAAGACATGGTATCTCTGGATCCCATTGAATTTCATTCAGAAGAGGAACCTTATAAAGATCGTATTGATTCTTATCAAAGAAAGACAGGATTAACCGAGGCTGTTCAAACAGGCACAGGTCAACTAAACGGTATTCCCGTAGCAGTTGGGGTTATGGATTTTCAATTTATGGGGGGTAGTATGGGATCCGTCGTAGGTGAGAAAATTACTCGTTTGATCGAGTATGCTACCAATCAATTTATACCTCTTATTTTAGTGTGTGCTTCCGGAGGAGCACGAATGCAAGAAGGAAGTTTGAGCTTGATGCAAATGGCTAAAATATCTTCTGCTTTATATGATTATCAATCGAATAAAAAGTTATTTTATGTATCAATCCTTACGTCTCCTACAACAGGTGGGGTGACAGCTAGTTTTGGGATGTTGGGAGATATTATTATTGCTGAACCTAACGCCTATATTGCATTTGCGGGTAAAAGAGTAATTGAACAAACATTGAATAAAACAGTACCTGAAGGTTCCCAATCGGCCGAATTTTTATTCCATAAGGGCTTATTCGATTCAATCGTACCACGTAATCTTTTAAAAGGCGTTTTGAATGAGTTACTTCAGCTCCACGATTTCTTTCCTTTGAATCCTAAATCAAGTAGTGCCTTAATTTAA

>lcl|NC_033910.1_cds_YP_009348363.1_32 [gene=psaI] [locus_tag=B2L33_pgp056] [db_xref=GeneID:31082800] [protein=PsaI] [protein_id=YP_009348363.1] [location=64153..64266] [gbkey=CDS]

ATGACAATTCTTAACAACTTACCCTCTATTTTTGTGCCTTTAGTGGGATTAGTATTTCCGGCAATTGCAATGGCTTCTTTATCTCTTCATGTTCAAAAAAACAAGATTTTTTAA

>lcl|NC_033910.1_cds_YP_009348364.1_33 [gene=ycf4] [locus_tag=B2L33_pgp055] [db_xref=GeneID:31082801] [protein=Ycf4] [protein_id=YP_009348364.1] [location=64575..65129] [gbkey=CDS]

ATGAGTTGGCGATCAGAACATATATGGATAGAACTTATAGCGGGGTCTCGAAAAATAAGTAATTTCTGCTGGGCTTTTATCCTCTTTTTAGGTTCATTGGGTTTTTTATTGGTTGGAATTTCCAGTTATCTTGGAAAAAGTTTCATATCTTTATTTCCCTCTCAGCAAATACTTTTTTTTCCACAAGGGATCGTGATGTCTTTCTATGGGATCGCCGGTTTATTTATTAGTTGTTATTTGTGGTGCACAATTTTGTGGAATGTGGGTGGGGGTTATGATCGATTCGATAGAGAAGAAGGAATAGTATGTTTTTTTCGCTGGGGATTTCCTGGAAAAAATCGTCGCATCTTACTCCGATTCCTTATGAAAGATATTCAGTCTATTAGAATAGAAGTTAAAGAGGGTATTTACGCTCGGCGTATCCCTTATATGGAAATCCGAGGCCGAGGGACTGTTCCTTTGACTCGTACTGATGAGAATTTGACTCCACAAGAAATTGAGCAAAAAGTTGCGGAATTGGCCTATTTTTTGCGTGTACCAATTGAAGTATTTTAA

>lcl|NC_033910.1_cds_YP_009348365.1_34 [gene=cemA] [locus_tag=B2L33_pgp054] [db_xref=GeneID:31082802] [protein=CemA] [partial=5'] [protein_id=YP_009348365.1] [location=<65878..66570] [gbkey=CDS]

ACGAAAAATGAAAAAAAAAAATCTATTCCCCTTCTATATCTTACATCTATAGTTTTTTTTCCCTGGTGTATCTCTTTTTTATTTAAAAAAAGTTTTGAATCTTGGGTTATTAATTGGTGTAATACTAGTAAATCCGAAATTTTTTTAAATGATAGCCAAGAAAAAAGCTTTTTAGAAAAATTCATAGAATTAGAGGAGCTCGTTCGCTTGGACGAAATGATAAAAGAATATCCGGAAATACATCTACAAAAGTTTCCTATCGGAATCCAGAAACAAATGATACAATTGATCAAGATACACAATGAGGATTGTATCCATACGATTTTGCACTTCTCGACAAATATAATCTATTTCGTTATTCTAAGTGGTTATTCTATTCTAAGTAATGAAGAACTCTTTTTTCTTAATTCTTGGGTTCAAGAATTCCTATATAACTTAAGTGACACAATAAAAGCTTTTTCCATTCTTTTATTAACCGATTTATGTATAGGATTCCATTCACCCCATGGTTGGGAATTAATGATTGGTTCTGTTTATAAAGATTTTGGATTTGCTCATAACGATCAAATTATATCTGGCCTTGTTTCCACTTTTCCAGTCATTATCGATACAATTTTGAAATATTGGATTTTCCGTTATTTAAATCGTGTATCTCCGTCACTTGTAGTGATTTATCATTCAATGAATGACTGA

>lcl|NC_033910.1_cds_YP_009348366.1_35 [gene=petA] [locus_tag=B2L33_pgp053] [db_xref=GeneID:31082803] [protein=PetA] [protein_id=YP_009348366.1] [location=66927..67889] [gbkey=CDS]

ATGCAAACTCGAAAAACGTTTTCTTGGATAAAGGAAGAGATTACTTATTCCATTTCCATATCACTTATGATATGTATAATAACTTGGGCATCCATTTCAAATGCATATCCCATTTTTGCACAGCAAGGTTATGAAAATCCACGCGAAGCAACTGGCCGTATTGTATGTGCCAATTGTCATTTAGCTAATAAACCGGTAGATATTGAGGTTCCACAAGCGGTACTTCCTGATACTGTATTTGAAGCAGTTGTTCGAATTCCTTATGATATGCAACTGAAACAAGTTCTTGCTAATGGAAAAAAGGGGGCTTTGAATGTAGGAGCTGTTCTTATTTTACCTGAGGGGTTTGAATTAGCCCCTTCCAGTCGTATTTCGCCAGAGATTAAAGAAAAGATAGGAAATCTGTCTTTTCAGAGTTATCGCCCCACTAAAAAAAATATTCTTGTGATAGGTCCTGTTCCTGGTCAGAAATATAGTGAAATTACCTTTCCGATTCTTTCTCCGGACCCCGCCACTAAGAAAGATGTTTACTTTTTAAAATATCCCATATATGTAGGCGGAAACAGAGGAAGGGGTCAGATTTATCCCGACGGGAGCAAGAGTAACAATACGGTTTATAATGCTACAGCCGCAGGGATGATAAGCAAAATAATACGAAAAGAAAAAGGGGGGTACGAAATAACCATAACAGATGCGTCAGAGGGACGTCAAGTGAGTGATATTATACCTCCAGGACCGGAACTTCTTGTTTCAGAAGGCGAATCCATCAAAGTTGATCAACCATTAACAAGTAATCCTAATGTAGGTGGATTTGGTCAGGGGGATGCGGAAATAGTACTTCAGGCCCCATTACGTGTCCAAGGCCTTTTGTTCTTCTTGGCATCCGTTATTTTGGCACAAATCTTTTTGGTTCTTAAAAAGAAACAGTTTGAGAAGGTTCAATTGTCCGAAATGAATTTTTAG

>lcl|NC_033910.1_cds_YP_009348367.1_36 [gene=psbJ] [locus_tag=B2L33_pgp052] [db_xref=GeneID:31082804] [protein=PsbJ] [protein_id=YP_009348367.1] [location=complement(68703..68825)] [gbkey=CDS]

ATGGCCGATACTACTGGAAGAATTCCTCTTTGGATAATAGGTACTGTAACAGGTATTCTTGTGATCGGTTTAATAGGCATTTTCTTTTATGGTTCATATTCCGGGTTGGGTTCATCCCTGTAA

>lcl|NC_033910.1_cds_YP_009348368.1_37 [gene=psbL] [locus_tag=B2L33_pgp051] [db_xref=GeneID:31082805] [protein=PsbL] [protein_id=YP_009348368.1] [location=complement(68977..69093)] [gbkey=CDS]

ATGACACAATCAAACCCGAACGAACAAAATGTTGAATTGAATCGTACCAGTCTCTACTGGGGATTATTACTCATTTTTGTACTTGCTGTTTTATTTTCTAATTATTTCTTCAATTAA

>lcl|NC_033910.1_cds_YP_009348369.1_38 [gene=psbF] [locus_tag=B2L33_pgp050] [db_xref=GeneID:31082806] [protein=PsbF] [protein_id=YP_009348369.1] [location=complement(69120..69239)] [gbkey=CDS]

ATGACGATAGATCGAACCTATCCAATTTTTACAGTACGATGGTTGGCTGTTCACGGACTAGCTGTACCTACCGTTTCTTTTTTGGGGTCAATATCAGCAATGCAGTTCATCCAACGATAA

>lcl|NC_033910.1_cds_YP_009348370.1_39 [gene=psbE] [locus_tag=B2L33_pgp049] [db_xref=GeneID:31082807] [protein=PsbE] [protein_id=YP_009348370.1] [location=complement(69253..69504)] [gbkey=CDS]

ATGTCCGGAAGCACAGGAGAACGTTCTTTTGCTGATATTATCACCAGTATTCGATATTGGGTTATTCATAGCATTACTATACCTTCCCTATTCATTGCAGGTTGGTTATTCGTCAGCACCGGTTTAGCTTACGATGTCTTTGGAAGCCCTCGTCCAAATGAATATTTTACAGAGAGCCGACAGGGAATTCCATTAATAACAGGCCGCTTTGATCCTTTGGAACAACTCGATGAATTTAGTAAATCTTTTTAG

>lcl|NC_033910.1_cds_YP_009348371.1_40 [gene=petL] [locus_tag=B2L33_pgp048] [db_xref=GeneID:31082808] [protein=PetL] [protein_id=YP_009348371.1] [location=71317..71412] [gbkey=CDS]

ATGCCTACTATAACTAGTTTTTTCGGTTTTCTACTAGCGGCTTTAACTATAACCTCAGTTCTATTTATTGGTCTGAGCAAGATAAGACTTATTTGA

>lcl|NC_033910.1_cds_YP_009348372.1_41 [gene=petG] [locus_tag=B2L33_pgp047] [db_xref=GeneID:31082809] [protein=PetG] [protein_id=YP_009348372.1] [location=71599..71712] [gbkey=CDS]

ATGATTGAAGTTTTGCTCTTTGGAATTGTCTTAGGGCTAATTCCTATTACTTTGGCCGGATTATTTGTAACTGCATATTTACAATATAGACGTGGCGATCAGTTAGACCTTTGA

>lcl|NC_033910.1_cds_YP_009348373.1_42 [gene=psaJ] [locus_tag=B2L33_pgp046] [db_xref=GeneID:31082748] [protein=PsaJ] [protein_id=YP_009348373.1] [location=72602..72736] [gbkey=CDS]

ATGCGAGATCTAAAAACATATCTATCCGTGGCACCGGTAATAAGTACTCTATGGTTTGCGTCTTTAGCAGGCCTATTGATAGAGATCAATCGTTTTTTTCCAGATGGATTGACATTGCCTTTTTTTTCATTCTAG

>lcl|NC_033910.1_cds_YP_009348374.1_43 [gene=rpl33] [locus_tag=B2L33_pgp045] [db_xref=GeneID:31082811] [protein=ribosomal protein L33] [protein_id=YP_009348374.1] [location=73210..73410] [gbkey=CDS]

ATGGCCAAGGGGAAAGATGTTCGAGTAAGAATTATTTTAGAATGTACCGGTTGTGTTCGAAAGAGTGTTAATAAGAAATCAACAGGTATTTCGAGATATATTACTCAAAAGAATCGACACAATACGCCTAGTCGATTGGAATTGAGAAAATTCTGTCCCTATTGTTACAAACATAGAATTCACGGGGAGATAAAGAAATAG

>lcl|NC_033910.1_cds_YP_009348375.1_44 [gene=rps18] [locus_tag=B2L33_pgp044] [db_xref=GeneID:31082812] [protein=ribosomal protein S18] [protein_id=YP_009348375.1] [location=73863..74147] [gbkey=CDS]

ATGGATAAATCCAAACGACTTTTCCTTAAGTCCAAGCGATCTTTTCGTAGGCGTTTGCCCCCGATCCAATCGGGGGATCGAATTGATTATAGAAACATGAGTTTAATTAGTCGATTTATTAGTGAACAAGGAAAAATATTATCTAGACGGGTGAATAGATTGAGTTTAAAACAACAACGATTAATTACTATTGCTATAAAACAAGCTCGTATTTTATCTTTGTTACCTTTTCTTAATAATGAAAAACAATTTGAAAAAAAGCGAGTTGGTCACTCTAACTACTGA

>lcl|NC_033910.1_cds_YP_009348376.1_45 [gene=rpl20] [locus_tag=B2L33_pgp043] [db_xref=GeneID:31082813] [protein=ribosomal protein L20] [protein_id=YP_009348376.1] [location=complement(74463..74819)] [gbkey=CDS]

ATGACCAGAATTAGACGAGGATATATAGCTCGGAGACGTAGAACAAAAATTCGTTTATTCGTATCAAGCTTTCGCGGGGCTCATTCAAGACTTACTCGAAGTATTATTCAACAAAAAATAAGAGCTTTGGTTTCGGCCCATCGGGATAGAGATAGACAGAAAAGAAATTTTCGTCGTTTATGGGTCACTCGGATAAATGCAGTAATTCGCGGAAATAGCAGGGTATCAAATAGTTATAGTAATTTAGTAAATAATCTGTACAAGAGACAATTGCTTCTTAATCGTAAAATACTTGCACAAATAGCTATATTAAATAGGAATTGTCTTTATACGATTTCCAATGACATTAGAAAATAA

>lcl|NC_033910.1_cds_YP_009348377.1_46 [gene=rps12] [locus_tag=B2L33_pgp042] [db_xref=GeneID:31082814] [protein=ribosomal protein S12] [exception=trans-splicing] [protein_id=YP_009348377.1] [location=join(complement(75634..75747),146289..146520,147057..147082)] [gbkey=CDS]

ATGCCAACTATTAAACAACTTATTAGAAACACAAGACAGCCAATTAGAAATGTTACCAAATCGCCCGCTCTTGGGGGATGTCCTCAACGCCGAGGAACATGTACTAGGGTGTATACTATCACCCCCAAAAAACCAAACTCTGCCTTACGTAAAGTTGCCAGAGTACGATTAACCTCTGGATTTGAAATCACTGCTTATATACCTGGTATTGGCCATAATTTACAAGAACATTCTGTAGTCTTAGTAAGAGGGGGAAGGGTTAAGGATTTACCCGGTGTGAGATATCACATTGTTCGAGGAACCCTAGATGCTGTCGGAGTAAAGGATCGTCAACAAGGGCGTTCTAAATATGGGGTCAAAAAGCCAAAATAA

>lcl|NC_033910.1_cds_YP_009348378.1_47 [gene=clpP] [locus_tag=B2L33_pgp040] [db_xref=GeneID:31082723] [protein=ClpP] [protein_id=YP_009348378.1] [location=complement(join(75957..76185,76839..77129,77951..78021))] [gbkey=CDS]

ATGCCTATTGGTGTTCCAAAAGTCCCTTTTCGACATCCTGGAGAAGACGATTCACTTTGGATTGACTTATACAACCGACTTTATCGAGAAAGATTACTTTTTTTAGGTCAAGGTATTGATAGCGAGATCTCGAATCAACTTATTGGTCTTATGGTATATCTCAGTATAGAGAGCGAGACCAAAGATTTGTATTTGTTTATAAACTCTCCTGGCGGATGGGTAATACCCGGAGTAGCTATTTATGATACTATGCAATTTGTGCGACCAGATGTACAAACAGTATGCATGGGATTAGCTGCTTCAATGGGATCTTTTATTCTGGTCGGAGGAAAAATCACCAAACGTTTAGCATTCCCTCATGCCAGGGTAATGATTCATCAACCTATTGCTGGTTTTTATGAAGCACAAATAGCAGAATTTGTCCTGGAAGCAGAAGAACTGCTGAAACTGCGTGAAATCCTCACAAGGATTTATGCACAAAGAACGGGAAAACCCTTATGGGTTGTATCTGAAGACATGGAAAGAGATGTTTTTATGTCAGCAACAGAAGCCCAAGCTCACGGAATTGTTGATCTTGTAGCAGTTGCATAA

>lcl|NC_033910.1_cds_YP_009348379.1_48 [gene=psbB] [locus_tag=B2L33_pgp039] [db_xref=GeneID:31082815] [protein=PsbB] [protein_id=YP_009348379.1] [location=78541..80067] [gbkey=CDS]

ATGGGTTTGCCTTGGTATCGTGTTCATACCGTCGTATTGAATGATCCCGGTCGTTTGCTGTCTGTCCATATAATGCATACAGCGTTGGTTGCTGGTTGGGCTGGTTCGATGGCTCTATATGAATTAGCAGTTTTTGATCCCTCTGACCCCGTTCTCGATCCGATGTGGAGACAAGGTATGTTCGTTATACCCTTCATGACTCGTTTAGGAATAACCAATTCGTGGGGTGGTTGGAATATCACAGGAGGAACTATAAGCAATCCGGGTATTTGGAGTTATGAAGGTGTGGCTGGGGCGCATATTGTGTTTTCTGGGTTGTGTTTCTTAGCAGCTATTTGGCATTGGGTGTATTGGGATCTAGAAATCTTTTTTGATGAACGTACCGGAAAACCTTCTTTGGATTTGCCCAAGATCTTTGGAATTCATTTATTTCTCTCAGGGGTAGCTTGCTTTGGGTTTGGCGCTTTTCATGTAACCGGATTGTATGGTCCTGGAATATGGATCTCCGATCCTTATGGATTAACTGGAAAGGTACAACCAGTAAGTCCAGCATGGGGTGTCGAAGGTTTTGATCCCTTTGTTCCGGGAGGAATAGCTTCTCATCATATTGCAGCAGGGACATTGGGCATATTGGCGGGTCTATTTCATCTTAGTGTCCGTCCGCCCCAACGTTTATACAAAGGATTACGTATGGGAAATATTGAAACTGTACTTTCCAGTAGTATCGCTGCTGTCTTTTTTGCAGCTTTTGTTGTTGCTGGAACTATGTGGTATGGTTCAGCAACTACCCCGATTGAATTATTTGGTCCCACTCGTTATCAATGGGATCAAGGATACTTCCAGCAAGAAATATATCGAAGAGTTAGTGCCGGGCTAGCCGAAAATCAAAATTTATCCGAAGCTTGGTCTAAAATTCCCGAAAAATTAACTTTTTATGATTACATTGGCAATAATCCTGCAAAAGGTGGATTGTTCAGAGCAGGTTCCATGGACAACGGGGATGGAATAGCTGTTGGGTGGTTAGGACATCCTATCTTTAGAGATAAAGAAGGGCGTGAACTTTTTGTACGCCGTATGCCTACTTTTTTTGAAACATTTCCAGTTGTTTTGGTAGACGGAGATGGAATTGTTAGAGCCGATGTTCCTTTTCGAAGGGCAGAGTCGAAGTATAGTGTCGAACAAGTAGGTGTAACTGTTGAGTTCTATGGTGGGGAACTAAACGGAGTCAGTTATAGTGATCCTGCTACTGTCAAAAAATATGCTAGACGTGCTCAATTAGGCGAAATTTTTGAATTAGATCGTGCTACTTTGAAATCCGATGGTGTTTTTCGTAGCAGTCCACGGGGTTGGTTTACTTTTGGACATGCTTCGTTCGCTCTGCTCTTTTTCTTCGGACACATTTGGCATGGTGCTCGAACTTTGTTCCGAGATGTTTTTGCTGGGATTGATCCAGATTTAGATGCTCAAGTGGAATTTGGAGCATTCCAAAAACTTGGAGATCCTACTACAAAAAGGCAAGTAGTTTGA

>lcl|NC_033910.1_cds_YP_009348380.1_49 [gene=psbT] [locus_tag=B2L33_pgp038] [db_xref=GeneID:31082721] [protein=PsbT] [protein_id=YP_009348380.1] [location=80253..80360] [gbkey=CDS]

ATGGAAGCATTGGTTTATACATTCCTTTTAGTATCAACTTTAGGAATAATTTTTTTCGCTATCTTTTTTCGAGAACCGCCTAAAGTTCCAACTAAAAAGGTAAAATGA

>lcl|NC_033910.1_cds_YP_009348381.1_50 [gene=psbN] [locus_tag=B2L33_pgp037] [db_xref=GeneID:31082816] [protein=PsbN] [protein_id=YP_009348381.1] [location=complement(80413..80544)] [gbkey=CDS]

ATGGAAACAGCAACCCTAGTCGCCATCTCTATATCTGGTTTACTTGTAAGTTTTACTGGGTATGCCTTATATACTGCTTTTGGGCAACCCTCGCAACAACTAAGAGATCCATTCGAGGAACACGGGGACTAA

>lcl|NC_033910.1_cds_YP_009348382.1_51 [gene=psbH] [locus_tag=B2L33_pgp036] [db_xref=GeneID:31082817] [protein=PsbH] [protein_id=YP_009348382.1] [location=80654..80875] [gbkey=CDS]

ATGGCTACACAAAGCGTGGAGGGTAGTTCTCGATCTGGTCCAAGACGAACTGCTGTAGGGGATTTATTGAAACCATTGAATTCGGAATATGGTAAAGTAGCTCCTGGCTGGGGAACCACTCCTTTAATGGGTATCGCAATGGCTCTATTTGCCATATTCCTATCTATTATTTTGGAGATTTATAATTCTTCTGTTTTACTAGATGGAATTTCAATGAATTAG

>lcl|NC_033910.1_cds_YP_009348383.1_52 [gene=petB] [locus_tag=B2L33_pgp035] [db_xref=GeneID:31082818] [protein=PetB] [protein_id=YP_009348383.1] [location=81726..82412] [gbkey=CDS]

ATACGGTTCTCGGAGGGGGAGTCCTTCTGGTTTACCTATCTCAATAAAGTCTATGATTGGTTCGAAGAACGTCTCGAGATTCAGGCGATTGCAGATGACATAACTAGTAAATATGTTCCTCCTCATGTTAACATATTTTATTGTTTAGGAGGAATTACGCTTACTTGTTTTTTAGTACAAGTAGCTACGGGGTTTGCTATGACTTTTTACTACCGTCCAACCGTTACTGAGGCTTTTTCTTCTGTTCAATACATAATGACTGAAGCTAATTTTGGTTGGTTAATCCGATCAGTTCATCGCTGGTCGGCAAGTATGATGGTTTTAATGATGATCCTGCACGTATTTCGTGTGTATCTCACTGGTGGTTTTAAAAAACCTCGTGAATTGACTTGGGTTACAGGCGTGGTTCTTGCTGTATTGACGGCATCTTTTGGTGTAACTGGTTATTCCTTACCTTGGGACCAAATTGGTTATTGGGCAGTCAAAATTGTAACAGGCGTACCGGAAGCTATTCCTGTAATAGGATCGCCTTTGGTAGAGTTATTACGGGGAAGTGCTAGTGTAGGACAATCCACTTTGACTCGTTTTTATAGTTTACACACTTTTGTATTACCCCTTCTTACTGCTGTATTTATGTTAATGCACTTTCCAATGATACGTAAGCAAGGTATTTCAGGTCCTTTATAG

>lcl|NC_033910.1_cds_YP_009348384.1_53 [gene=petD] [locus_tag=B2L33_pgp034] [db_xref=GeneID:31082819] [protein=PetD] [protein_id=YP_009348384.1] [location=83341..83886] [gbkey=CDS]

ATGTCCGGTTCCTTCGGAGGATGGATCTATAAGAATTCACCTATCCCAATAACAAAAAAACCTGACTTGAATGATCCTGTATTAAGAGCTAAATTGGCTAAGGGAATGGGTCATAATTATTATGGGGAACCCGCATGGCCAAACGATCTTTTATATATTTTTCCAGTAGTAATTTTAGGTACTATTGCATGTAATGTAGGATTAGCGGTTCTAGAACCATCAATGATTGGTGAACCTGCAGATCCATTTGCAACTCCTTTGGAAATATTGCCTGAATGGTATTTCTTTCCTGTATTTCAAATACTCCGTACAGTACCCAATAAGTTATTGGGTGTTCTTTTAATGGTTTCAGTACCTACAGGCTTATTAACAGTACCTTTTTTAGAGAATGTTAATAAATTCCAAAATCCATTTCGTCGTCCAGTTGCGACAACTGTATTTTTGATTGGTACTGTAGTAGCCGTTTGGTTAGGTATTGGAGCAACATTACCTATTGAGAAATCTCTAACTTTAGGTCTTTTTCAAGTTGATTTAATTGAAAAATAA

>lcl|NC_033910.1_cds_YP_009348385.1_54 [gene=rpoA] [locus_tag=B2L33_pgp033] [db_xref=GeneID:31082820] [protein=RpoA] [protein_id=YP_009348385.1] [location=complement(84025..85044)] [gbkey=CDS]

ATGGTTCGAGAGAAAGTAACAATATCTACTCGGACACTGCAGTGGAAATGTGTTGAATCAAGAAAGGACAATAAGCGTCTTTATTACGGACGCTTTATTCTCTCTCCGCTTATGAAAGGCCAATCCGATACGATAGGCATTGCGATTCGAAGAGCTTTGCTTGGAGAAATAGAAGGAACCTGTATCACACGTGCAAAATCTGAAAAAATATCACACGAATTTTCTACTATAACAGGTATTCAAGAATCAATACATGAAATTTTCATGAATTTGAAAGAAATTATATTGAGAAGCAATTTGTATGGAACTTGTGACGCGTCTATTTGTGTCAAGGGTCCTGGATATGTAACTGCTCAAGACATCATTCTACCACCTTTTGTGGAAATCATTGATAATACACAGCATATCGCTAGCCTAACAGAACCAATTGATTTGTGTATTCGATTACAAATCGAGAGGAATCGTGGCTATCGTATAAAACCAACAAAAACCTTGCAAGATGGAAGTTTTCCTCTAGATGCTGTATTCATGCCGGTTCGAAATGCAAATCATAGTGTTCATTCTTATGGAAATGGGAATGAAAAGCAAGAGATACTTTTTCTCGAAATATGGACAAACGGAAGTTTAACTCCTAAAGAAGCACTTCATGAGGCTTCCCGTAATTTGATTGATTTATTTATTCCTTTTCTACATGCAGACGAACAAAACTTACCTTTAGAAAAAAATCAACACAACGTTACTTTACCCCTTTTGACTTTTCATGATAGATTGACTAAATTAAGAAAAAAAAAAAAAGAAATACCATTGAAATACATTTTTATTGACCAATCCGAATTGACTCCTAAGATCTATAATTGCCTCAAAAGGTCTAATATACATACATTATCGGACCTTTTGAATAAGAGTCAAGAAGATCTTATGCAAATTGAAGATTTTCACATAGACGATGTAAAACATATATTGGGTATTTTAGAAATAAAAAAACATTTCGTAATGGATTTACTAAAGAATAAAATCTAA

>lcl|NC_033910.1_cds_YP_009348386.1_55 [gene=rps11] [locus_tag=B2L33_pgp032] [db_xref=GeneID:31082821] [protein=ribosomal protein S11] [protein_id=YP_009348386.1] [location=complement(85111..85527)] [gbkey=CDS]

ATGGCAAAACCTTTACCACGAATTGGTTCACGCAGAACTGGACGCATTGGTTCACGTAAGAATGCACGTAAAATACCAAAAGGAGTTATTCATGTTCAAGCAAGTTTCAACAATACTATTGTGACCGTTACAGATGTACGGGGACGAGTAATTTCGTGGTCCTCGGCTGGAACTTGTGGATTCAAAGGCACAAGAAGAGGAACACCATTTGCTGCTCAAACCACAGCAGGAAATGCTATTCGAACAGTAGTGGATCAAGGCATGCAACGAGCAGAAGTCATGATAAAAGGTCCTGGTCTCGGACGCGATGCGGCATTAAGAGCTATTCGCAGAAGTGGTATACTATTAACTTTTGTCCGGGATGTAACCCCTATGCCACATAATGGCTGCAGACCCCCGAAAAAAAGGCGCGTGTAA

>lcl|NC_033910.1_cds_YP_009348387.1_56 [gene=rpl36] [locus_tag=B2L33_pgp031] [db_xref=GeneID:31082822] [protein=ribosomal protein L36] [protein_id=YP_009348387.1] [location=complement(85643..85756)] [gbkey=CDS]

ATGAAAATAAGAGCTTCTGTTCGTAAAATTTGTGAAAAATGCCGACTGATACGTAGACGGGGACGAATTATAGTAATTTGCTTCAACCCAAGACATAAACAAAGACAAGGATAA

>lcl|NC_033910.1_cds_YP_009348388.1_57 [gene=rps8] [locus_tag=B2L33_pgp030] [db_xref=GeneID:31082823] [protein=ribosomal protein S8] [protein_id=YP_009348388.1] [location=complement(86251..86655)] [gbkey=CDS]

ATGGGTAGGGATCCGATTGCTGACATAATAACCTCTATACGAAATGCTGACATGAATCGAAAAGAAACCGTTCGAATAGCAGCTACTAACATCACCGAAAACATTATAAAATTACTCTTACGAGAGGGTTTTATTGAAAATGTCAGGAAACATCAGGAAGGCAACAAAAAATTTTTGGTTTTAACTTTACGCCATAGAAGGAAAAAAAAAGGACCATATAGAACTAGTCTAAATTTAAAACGGATAAGCCGGCCGGGTCTACGAATCTATTCTAACTATCAAAAAATTCCTAGAATTTTGGGCGGGATGGGCATTGTAATTCTTTCTACTTCTCGGGGTATACTGACAGACCGGGAAGCTCGACTCGAAAGAATCGGCGGAGAAATCTTGTGTTATATATGGTAA

>lcl|NC_033910.1_cds_YP_009348389.1_58 [gene=rpl14] [locus_tag=B2L33_pgp029] [db_xref=GeneID:31082824] [protein=ribosomal protein L14] [protein_id=YP_009348389.1] [location=complement(86923..87291)] [gbkey=CDS]

ATGATCCAATCTCAGACCCATTTGAATGTAGCAGATAACAGTGGAGCCCGAGAATTGATGTGTATTCGAATCATAGGGACTAGTAATCGCCGATATGCTCATATCGGTGACGTTATTGTTGCTGTGATCAAGGAAGCAGCACCCAATTCACCTCTCGAAAGATCAGAAGTAATCAGAGCTGTAATTGTACGTACTTGTAAAGAACTTAAACGTAATAACGGTATAATAATACGATATGATGACAATGCTGCAGTTGTCATTGATCAAGAAGGAAATCCAAAAGGAACACGAATTTTTGGTGCAATTGCCCGGGAATTGAGACAGTTAAATTTTACTAAAATAGTTTCATTAGCACCTGAGGTCTTATAA

>lcl|NC_033910.1_cds_YP_009348390.1_59 [gene=rpl16] [locus_tag=B2L33_pgp028] [db_xref=GeneID:31082825] [protein=ribosomal protein L16] [protein_id=YP_009348390.1] [location=complement(87418..87777)] [gbkey=CDS]

ATGAAAGGAATATCTTTTAGAGGCAATCGTATTTGTTTTGGAAGATATGCTCTTCAAGCACTTGAACCCGCTTGGATTACATCTAGACAAATAGAAGCGGGGCGACGAGCAATGACACGAAATGCACGCCGCGGTGGAAAAATATGGGTACGTATATTTCCCGACAAACCCGTTACTTTAAGACCTACGGAAACACGTATGGGTTCGGGGAAAGGGTCTCCCGAATATTGGGTAGCTGTGGTTAAACCAGGTAGAATACTTTTTGAAATGGGTGGAGTGGCAGAAAATATAGCGAGAAAGTCTATTTCAATAGCAGCATCCAAAATGCCTATACGAACTCAATTCCTTATTTCAAAATAA

>lcl|NC_033910.1_cds_YP_009348391.1_60 [gene=rps3] [locus_tag=B2L33_pgp027] [db_xref=GeneID:31082826] [protein=ribosomal protein S3] [protein_id=YP_009348391.1] [location=complement(89286..89942)] [gbkey=CDS]

ATGGGACAAAAAATAAATCCACTTGGTTTCAGACTTGGTACAACCCAAAGTCATCATTCTCTTTGGTTTGCACAACCAAAAAATTACTCTGAGGGTCTACAAGAAGATCAAAAAATAAGAAACTGTATCAAGAATTTTGTAAAAAAAAATACAAAAATATCTTCTGGCGTTGAGGGAATTGCACGTATAGAGATTCAAAAACGAATCGATGTGATTCAAGTCATAATATATATGGGATTCCCAAAATTATTAATAGAAAATAGACCTAAACGAATCGAAGAATTACAGATACATGTACAAAAAGAACTAAATTGTGTAAACCGAAAACTCAATATTGTTATCACAAGAATTTCAAATCCTTATAGCAACCCTAATATTCTTGCAGAATTTATAGCTGGACAATTAAAAAATCGAGTTTCTTTTCGTAAAGCAATGAAAAAAGCTATTGAATTAACCGAACAGGCGGATACAAAAGGAATTCAAGTACAAATTGCGGGACGCCTTGATGGAAAAGAAATTGCACGAGTCGAATGGATTAGAGAGGGTAGGGTTCCTCTACAAACCATTCGAGCTAAAATTGATTATTGTTCGTATACAGTTAGAACTATTTATGGGGTATTAGGCATAAAAATTTGGACATTTCTAGACAAGAAATAA

>lcl|NC_033910.1_cds_YP_009348392.1_61 [gene=rpl22] [locus_tag=B2L33_pgp026] [db_xref=GeneID:31082827] [protein=ribosomal protein L22] [protein_id=YP_009348392.1] [location=complement(90067..90453)] [gbkey=CDS]

ATGATAAATAAAAAAATAGACCCGTACACAGAAGTATATAGTTTAGGACAACATATACGTATGTCCGCTCACAAAGCACGAAGAATAATCGATCAAATTCGTGGACGTTCTTACGAAGAAACACTTATGATACTAGAACTAATGCCTTATCGAGCATCTTATCCCATTTTAAAATTGATTTCTTCTGCAGCAGCAAATGCTAGTCACAATATGGCTTTCAACGAAACCGATTTAATGATTAGTAAAGCCGAGGTTAACGACGGTACTACTGTGAAAAAATTAAAACCTCAGGCTCGAGGACGGGGTTATCTAATAAAAAAATCAACTTGTCATATAACTATTGTATTAAAAAATATATCCTTAGAAAAAGAATATGAGGATTCTTAA

>lcl|NC_033910.1_cds_YP_009348393.1_62 [gene=rps19] [locus_tag=B2L33_pgp025] [db_xref=GeneID:31082828] [protein=ribosomal protein S19] [protein_id=YP_009348393.1] [location=complement(90506..90784)] [gbkey=CDS]

GTGACACGTTCACTAAAAAAAAATCCTTTTGTAGCAAATCATTTATTAAAAAAAATAAATAAGCTTAACACAAAAGCAGAAAAAGAAATAATAGTAACGTGGTCCCGGGCATCTACCATTATACCTACAATGATCGGCCATACTATTGCTATCCATAATGGAAAGGAGCATTTACCTATTTATATAACAGATCGTATGGTGGGCCATAAATTGGGAGAATTTGCACCTACTTTAAATTTCCGGGGACATGCAAAAAATGATAATAAATCCCGCCGTTAA

>lcl|NC_033910.1_cds_YP_009348394.1_63 [gene=rpl2] [locus_tag=B2L33_pgp024] [db_xref=GeneID:31082829] [protein=ribosomal protein L2] [protein_id=YP_009348394.1] [location=complement(join(90841..91308,91933..92328))] [gbkey=CDS]

ATGGCGATACATTTATACAAAACTTCTACCCCGAGCACACGCAATGGAGCCGTAGACAGTCAAGTGAAATCCAATACACGAAATAATTTGATCTATGGACAGCATCATTGTGGTAAAGGCCGTAATGCCAGAGGAATAATTACCGCAAGGCATAGAGGGGGAGGTCATAAGCGTCTATACCGTAAAATCGATTTTCGACGGAATGAAAAAGACATATATGGTAGAATCGTAACCATAGAATACGACCCTAATCGAAATGCATACATTTGTCTCATACACTATGGGGATGGTGAGAAGAGATATATTTTACATCCCAGAGGGGCTATAATTGGAGATACCATTATTTCTGGTACAGAAGTTCCTATAAAAATGGGAAATGCCCTACCTTTGAGTGCGGTTTTGATTGATCAAAAAGAAGAATCTACTTCAACCGATATGCCCTTAGGCACGGCCATACATAACATAGAAATCACACTTGGAAAGGGTGGACAATTAGCTAGAGCTGCAGGTGCTGTAGCGAAACTGATTGCAAAAGAGGGTAAATCGGCCACATTAAAATTACCTTCTGGGGAGGTTCGTTTAATATCCAAAAACTGCTCAGCAACAGTCGGACAAGTAGGGAATACTGGGGTGAACCAGAAAAGTTTGGGTAGAGCCGGGTCTAAATGTTGGCTAGGTAAGCGTCCTGTAGTAAGAGGAGTGGTTATGAACCCTGTAGACCATCCCCATGGGGGTGGTGAAGGGAGGGCCCCAATTGGTAGAAAAAAACCCGCAACCCCTTGGGGTTATCCTGCACTTGGAAGAAGAAGTAGAAAAAGGAATAAATATAGTGATAATTTGATTCTTCGTCGCCGTAGTAAATAG

>lcl|NC_033910.1_cds_YP_009348395.1_64 [gene=ycf2] [locus_tag=B2L33_pgp023] [db_xref=GeneID:31082749] [protein=Ycf2] [protein_id=YP_009348395.1] [location=92957..99847] [gbkey=CDS]

ATGAAGGGACATCAATTCAAATCCTGGATTTTCGAATTGAGAGAGATATTGAGAGAGATCAAGAATTCTCACTATTTCTTCGATTCATGGACCCAATTCAATTCAGTGGGATCTTTCATTCACATTTTTTTCCATCAAGAACGTTTTATAAAACTCTTGGACTCCCGAATTTGGAGTATCTTACTTTCACGCAATTCACAGGGTTCAACAAGCAATCGATATTTCACGATCAAGGGTGTAGTACTCTTTGTAGTAGTGGTCCTTATATATCGTATTAACAATCGAAAGATGGTCGAAAGAAAAAATCTCTATTTGACAGGGCTTCTTCCTATACCTATGAATTCCATTGGACCCAGAAATGATACATTGGAAGAATTCTTTGGGTCTTACAATATCAATAGGTTGATTGTTTCGCTCCTGTATCTTCCAAAAGGAAAAAAGATCTCTGAGAGCTCTTTCCCGGATCTGAAAGAGAGTACTTGGGTTCTCCCAATAACTAAAAAGTGTATCATGTCTGAATCTAACTGGGGTTCACGGCGGTGGAGGAACTGGATCGGAAAAAAGAGGGATTCTAGTTGTAAGATATCTAATGAAACCGTCGCTGGAATTGAGATCTCATTCAAAGAAAAAGATATCAAATATCTGGAGTTTCTTTTTGTATATTATATGGATGATCCGATCCGCAAGGACCAGGATTGGGATTTGTTTGATCGTCTTTCTCCGAGGAAGGGGCGAAACAGAATCAACTTGAATTCGGGACAGCTATTGGAAATCTTAGTGAAAGACTGGATTTGTTATCTCATGTTTGCTTTTCGTGAAAAAATACCAATTGAAGTGGAGGGTTTCTTCAAACAACAAGGAGCTGGGTCAACTATTCAATCAAATGATATTGAGCATGTTTCCCATCTCTTCTCGAGAAAGAAGTGGGCTATTTCTTTGCAAAATTGTGCTCAATTTCATATGTGGCAATTCCGCCAAGATCTCTTCGTTAGTTGGGGGAATAATCCGCACGAATCGGATTTTTTGAGGAACATATCGAGAGAGAATTGGATTTGGTTAGACAATGTGTGGTTGGTAAACAAGGATCGGTTTTTTAGCAAGGCACGGAATATATCGTCAAATATTCAATATGATTCCACAAGATCTAGTTTCGTTCAAGGAAGGAATTCTAGCCAATTGAAGGGATCTTCTGATCAATCCAGAGATCATTTCGATTCCATTAGTAATGAGGATTCGGAATATCACACATTGATCAATCAAAGAAAGATTCAACAACTAAAAGAAAGATCGATTCTTTGGGATCCTTCCTTTCTTCAAACGGAACGAACAGAGATAGAATCAGACCAATTCCCTAAATGCCTTTCTGGATATTCCTCAATGTCCCGGCTATTCAGGGAAGGTGAGAAGGAGATGAATAATCATCTGCTTCCGGAAGAAATCGAAGAATTTCTTGGGAATCCTACAAGATCCATTCGTTCTTTTTTCTCTGACAGATCGTCAGAACTTCATCTGGGTTCGAATCCTACTGAGAGATCCACTAGAGATCAGAAATTGTTGAAGAAAGAACAAGATGGTTCTTTTGTCCCTTCCAGGCGATCGGAAAATAAAGAAATAGTTAATATATTCAAGATAATCACGTATTTACAAAATACCGTCTCAATTCATCCATCCGATCCGGGATGTGATATGGTTCTGAAGGATGAACTGGATATGGACAGTTCCAATAAGATTTCTTTCTTGAACAAAAATCCATTTTTTGATTTATTTCATCTATTCCATGATCGGAACGGGGGGGGGTACACGTTACACCACGATTTTGAATCAGAAGAGAGATTTCAAGAAATGGCAGATCTATTCACTCTATCAATAACCGAGCCGGATCTGGTGTATCATAAGGGATTTACCCGTTTTATTGATTCCTACGGATTGGATCAAAAACAATTCTTGAATGAGGTATTCAACTCCAGGGATGAATCGAAAAAGAAATCTTTATTGGTTCTACCTCCTATTTTTTATGAAGAGAATGAATCTTTTTATCGAAGGATCAGAAAAAAATGGGTCCGGATCTCCTGCGGGAATGATTTGGAAGATCCAAAACAAAAAACAGTGGTATTTGCTAGCAACAACATAATGGAGGCAGTCAATCAATATGGATTGATCCTAAATCTGATTCAAATCCAATATAGTACCTATGGGTACATAAGAAATGTATTGACTCAATTCTTTTTAATGAATAGATCCGATCGCAACTTCGAATATGGAATTCAAAGGGATCAAATAGGAAATGATACTCTGAATCATAGAACTATAATGAAATATACGATCAACCAACATTTATCGAATTTGAAACAGAGTCAGAAGAAATGGTTCGATCCTCTTATTTTTCGTTCTCGAACCGAGAGATCCATGAATTGGGATCCTAATGCATATAGATACAAATGGTCTAATGAGAGCAAGAATTTCCAGGAACATTTGGAACATTTCATTTCTGAGCAGAAGAGCCGTTTTCTTTTTCAAGTAGTGTTCGATCGATTACGTATTAATCAATATTCGATTGATTGGTCTGAGGTTATCGACAAAAAAGATTTGTCTAAGTCACTTCCTTTCTTTTTGTCCAAGTTACTTCTTTTTTTGTCCAAGTTTCTTCTCTTTTTGTCTAACTCACTTCCTTTTTTCTTTGTGAGTTTCGGGAATATCCCCATTCATAGGTCCGAAATCCATATCTATGAATTGAAAGGTCAGAATGATCAACTCTGCAATCAGCTGTTAGAACCAATAGGTCTTCAAATCGTTCATTTGAAAAAATTGAAACCCTTCTTATTGGATGATCATGATACTTCCCAAAAATCGAAATTTTTAATTAATATTAATGGAGGAACAATATCACCATTTTTGTTCAATAAGATACCAAAGTGGATGATTGACTCATTCCATACTAGAACTAATCGCAGGAAATCTTTTGATAACACGGATTCCTATTTCTCAATCATATCCCACGATCAAGACAATTGGCTGAATCCCGTGAAACCATTTCATAGAAGTTCATTGATATCTTCTTTTTATAAAGCAAATCGACTTCGATTCTTGAATAATCTACATCACTTCTGCTTCTATTGTAACAAAAGATTCCCTTTTTATGTGGAAAAGGCCCGTATCAAGAATTCTGATTTTACGTATGGACAATTCCTCAATATCTTGTTCATTCGCAACAAAATATTTTCTTTGTGCGGCGGTAAAAAAAAACATGCTTTTTTGGAGAGAGATACTATTTCACCAATCGAGTCCCAGGTATCTAACATATTCATACCTAATGATTTTCCACAAAGTGGTAACGAAAGGTATAACTTGTACAAATCTTTCCATTTTCCAATTCGATCCGATCCATTCGTTCGTAGAGCTATTTATTCGATCGCAGACATTTCTGGAACACCTCTAACAGAGGGACAAATAGTCAATTTTGAAAGAACTTATTGTCAACCTCTTTCGGATATGAATCTATCTGATTCAGAAGGGAAGAACTTGCATCAGTATCTCAATTTCAATTCAAACATGGGTTTGATTCACACTCCATGTTCTGAGAAATATTTACCATCCGAAAAGAGGAAAAAACGGAGTCTTTGTCTAAAGAAATGTGTTGAAAAAGGGCAGATGTATAGAACCTTTCAACGAGATAGTGCTTTTTCAACTCTCTCAAAATGGAATCTATTCCAAACATATATGCCATGGTTCCTTACTTCGACAGGGTACAAATATCTAAATTTTCTATTTTTCGATACCTTTTCGGACCTATTACCGATACTAAGTAGCAGTCAAAAATTTGTATCCATTTTTCATGATATTATGCATGGATCAGATATATCATGGCGAATTCTTCAGAAAAAATTGTGTCTTCCACAATGGAATCTGATAAGTGAGATTTCGAGTAAATGTTTACATAATCTTCTTCTGTCCGAAGAAATGATTCATCGAAATAATGAGCCACCATTGATATCGACACATCTGAGATCGCCAAATGTTCGGGAGTTCCTCTATTCAATCCTTTTCCTTCTTCTTGTTGCTGGATATCTCGTTCGTACACATCTTCTTTTTGTTTCCCGAGCCTATAGTGAGTTACAGACAGAGTTCGAAAAGGTCAAATCTTTGATGATTCCATCATACATGATTGAGTTGCGAAAACTTCTGGATAGGTATCCTACATCTGAACTGAATTCTTTCTGGTTAAAGAATCTCTTTCTAGTTGCTCTGGAACAATTAGGAGATTTTCTAGAAGAAATGCGGGGTTCTGCTTCTGGCGGCAACATGCTATGGGGTGGTGGTCCCACTTATGGGGTTAAATCAATCCGTTCTAAGAAGAAATTTTTGAATATCAATCTCATCGATCTCATAAGTATCATACCAAATCCCATCAATCGAATCACTTTTTCGAGAAATACGAGACATCTAAGTCATACAAGTAAAGAGATTTATTCATTGATAAGAAAAATAAAAAACGTGAACGGTGATTGGATTGATGATAAAATAGAATCCTTGGTCGCGAACAGTGATTCGATTGATGATAAAGAAAGAGAATTCTTGGTTCAGTTCTCCACCTTAACGACAGAAAAAAGGATTGATCAAATTCTATTGAGTCTGACTCATAGTGATCATTTATCAAAGAATGACTCTGGTTATCAAATGATTGAAGAGCCGGGAGCAATTTATTTACGATACTTAGTTGACATTCATAAAAAGTATCTAATGAATTATGAGTTCAATACACCCTGTTTAGCAGAAAGACGGATATTCCTTGCTTATTATCAGACAACCGCTTATTCACAAACCTCGTGTGGGGTGAATAGTTTTCATTTCCCATCTCATGGAAAACCCTTTTCGCTCCGCTTAGCCCTATCCCCCTCTAGGGGTATTTTAGTGATAGGTTCTATAGGAACTGGACGATCCTATTTGGTCAAATACCTAGCGACAAACTCCTATCTTCCTTTCATTACAGTATTTCTGAACAAGTTCCTGGATAACAAGCCTAACGGTTTTCTTATTGATGATAGTGACGATATTGATGATAGTGACGATATTGATGATAGTGACGATATTGATGTGAGTGACGATATTGATGTGAGTGACGATATCGACCGTGACTTTGATACGGAGCTGGAGTTTCTAACTAGGATGAATGCGCTAACTATGGATATGATGCCGGAAATAGACCGATTTTATATCACCCTTCAATTCGAATTAGCAAAAGCAATGTCTCCTTGCATAATATGGATTCCAAACATTCATGATCTGGATGTGAATGAGTCGAATTACTTATCCCTCGGTCTATTAGTGAACTATCTCTCCAGGGATTGTGAAAGATGTTCCACTAGAAATATTCTTGTTATTGCTTCGACTCATATTCCCCAAAAAGTGGATCCCGCTCTAATAGCTCCAAATAAATTAAATACATGCATTAAGATACGAAGGCTTCTTATTCCACAACAACGAAAGCACTTTTTTACTCTTTCGTATACTAGGGGATTTCACTTGGAAAATAAAATGTTCCATACTAATGGATTCGGGTCCATAACCATGGGTTCCAATGTACGAGATCTTGTAGCACTTACCAATGAGGCCTTATCGATTAGTATTACACAGAAAAAATCAATTATAGACACTAATATAATTAGATCTGCTCTTCATAGACAAACTTGGGATTTGCGATCCCAGGTAAGATCGGTTCAGGATCATGGGATCCTTTTCTATCAGATAGGAAGGGCTGTTGCACAAAATGTATTTCTAAGTAATTGCCCCATAGATCCTATATCTATCTATATGAAGAAGAAATCATGTAACGAAGGGGATTCTTATTTGTACAAATGGTACTTCGAACTTGGAATGAGCATGAAGAAATTAACGATACTTCTTTATCTTTTGAGTTGTTCTGCCGGATCGGCTGCTCAAGACCTTTGGTCTCTACCCGGACCCGATGAAAAAAATGGGATCACTTATTATGGACTTGTTGAGAATGATTCGGATCTAGTTCATGGTCTATTAGAAGTAGAAGGCGCTCTGGTGGGATCCTCACGTACAGAAAAAGATTGCAGTCAGTTTGATAATGATCGAGTGACATTGCTTCTTCGGCCCGAACCAAGGAGTCCCTTAGATATGATGCAAAATGGATCTTATTCTATCCTTGATCAGAGATTTCTCTATGAAAAATACGAATCGGAGTTTGAAGAAGGAGAAGAAGTCCTCGACCCGCAACAGATAGAGGACGATTTATTCAATCACATAGTTTGGGCTCCTAGAATATGGCGCCCTTGGGGTTTTCTATTTGATTGTATCGAAAGGCCCAATGAATTGGGATTTCCCTATTGGGCCAGGTCATTTCGGGGCAAGCGGATCATTTATGATGAAGAGGATGAGCTTCAAGAGAATGATTCGGGGTTCTTGCAGAGTGGAACCATGCAGTACCAGATACGAGATAGATCTTCCAAAGAACAAGGCTTTTTTCGAATAAGCCAATTCATTTGGGACCCTGCGGATCCACTCTTTTTCCTATTCAAAGATCAGCCCTTTGTCTCTGTGTTTTCACATCGAGAATTCTTTGCAGATGAAGAGATGTCAAAGGGGCTTCTTACTTCCCAAACAGATCCTCCTACATCTATATATAAACGCTGGTTTATCAAGAATACGCAAGAAAAGCACTTCGAATTGTTGATTCATCGCCAGAGATGGCTTAGAACCAATAGTTCATTATCTAATGGATTTTTCCGCTCTAATACTCTATCCGAGAGTTATCAGTATTTATCAAATCTGTTCCTATCTAACGGAACGCTATTGGATCAAATGACAAAGGCATTGTTGAGAAAAAGATGGCTTTTCCCGGATGAAATGAAAATTGGATTCATGTAA

>lcl|NC_033910.1_cds_YP_009348396.1_65 [gene=ycf15] [locus_tag=B2L33_pgp022] [db_xref=GeneID:31082831] [protein=Ycf15] [protein_id=YP_009348396.1] [location=99980..100117] [gbkey=CDS]

ATGTTACTGCTGAAACATGAAAGAATTGAAATCTTAGATCAAAACACTATGTATGGATGGTATGAACTGCCTAAACAAGAATTCTTGAACAGCGAGCAAGCAGAACGATTACTCACTACATCAAAAAATTTCCATTAA

>lcl|NC_033910.1_cds_YP_009348397.1_66 [gene=ndhB] [locus_tag=B2L33_pgp021] [db_xref=GeneID:31082750] [protein=NdhB] [partial=5'] [protein_id=YP_009348397.1] [location=complement(join(101182..101937,102616..103392))] [gbkey=CDS]

ACGATCTGGCATGTACAGAATGAAAACTTCATTCTCGATTCTACGAGAATTTTTATGAAAGCCTTTCATTTGCTTCTCTTCGATGGAAGTTTTATTTTCCCAGAATGTATCCTAATTTTTGGCCTAATTCTTCTTCTGATGATCGATTCAACCTCTGATCAAAAAGATATACCTTGGTTATATTTCATCTCTTCAACAAGTTTAGTAATGAGTATAACGGCCCTATTGTTCCGATGGAGAGAAGAACCTATGATTAGCTTTTCGGGAAATTTCCAAACGAACAATTTCAACGAAATCTTTCAATTTCTTATTTTACTATGTTCAACTCTATGTATTCCTCTATCCGTAGAGTACATTGAATGTACAGAAATGGCTATAACAGAGTTTCTCTTATTCGTATTAACAGCTACTCTAGGAGGAATGTTTTTATGCGGTGCTAACGATTTAATAACTATCTTTGTAGCTCCAGAATGTTTCAGTTTATGCTCCTACCTATTATCTGGATATACCAAGAAAGATGTACGGTCTAATGAGGCTACTATGAAATATTTACTCATGGGTGGGGCAAGCTCTTCTATTCTGGTTCATGCTTTCTCTTGGCTATATGGTTCGTCCGGGGGAGAGATCGAGCTTCAAGAAATAGTGAATGGCCTTATCAATACACAAATGTATAACTCCCCAGGAATTTCAATTGCGCTTATATTCATCACTGTAGGAATTGGGTTCAAGCTTTCCCCAGCCCCTTCTCATCAATGGACTCCTGACGTATACGAAGGATCTCCCACTCCAGTCGTTGCTTTTCTTTCTGTTACTTCGAAAGTAGCTGCTTCAGCTTCAGCCACTCGAATTTTCGATATTCCTTTTTATTTCTCATCAAACGAATGGCATCTTCTTCTGGAAATCCTAGCTATTCTGAGCATGATAGTGGGTAATCTCATTGCTATTACTCAAACAAGCATGAAACGTATGCTTGCATATTCGTCCATAGGTCAAATCGGATATGTAATTATTGGAATAATTGTTGGAGACTCAAATGGTGGATATGCAAGTATGATAACTTATATGTTCTTCTATATCTCCATGAATCTAGGAACTTTTGCTTGTATTGTATTATTTGGTCTACGTACCGGAACTGATAACATTCGAGATTATGCAGGATTATACACGAAAGATCCTTTTTTGGCTCTCTCTTTAGCCCTATGTCTCTTATCCCTAGGAGGTCTTCCTCCACTAGCAGGTTTTTTCGGAAAACTCCATTTATTCTGGTGTGGGTGGCAGGCAGGCCTATATTTCTTGGTTTTAATAGGACTCCTTACGAGCGTTGTTTCTATCTACTATTATCTAAAAATAATCAAGTTATTAATGACTGGACGAAACCAAGAAATAACCCCTCACGTGCGAAATTATAGAAGATCCCCTTTAAGATCAAACAATTCCATCGAATTGAGTATGATTGTATGTGTGATAGCATCTACTATACCAGGAATATCAATGAACCCGATTATTGAAATTGCTCAAGATACCCTTTTTTAG

>lcl|NC_033910.1_cds_YP_009348398.1_67 [gene=rps7] [locus_tag=B2L33_pgp020] [db_xref=GeneID:31082833] [protein=ribosomal protein S7] [protein_id=YP_009348398.1] [location=complement(103706..104173)] [gbkey=CDS]

ATGTCACGTCGAGGTACTGCAGAAGAGAAAACTGCAAAATCCGATCCAATTTATCGTAATCGATTAGTTAACATGTTGGTTAACCGTATTCTGAAACACGGAAAAAAATCATTGGCTTATCAAATTATCTATCGAGCCATGAAAAAGATTCAACAAAAGACAGAAACAAATCCACTATCTGTTTTACGTCAAGCAATACGTGGAGTAACTCCCGATATAGCAGTAAAAGCAAGACGTGTAGGCGGATCGACTCATCAAGTTCCCATTGAAATAGGATCCACACAAGGAAAAGCACTTGCCATTCGTTGGTTATTAGGGGCATCCCGAAAACGTCCGGGTCGAAATATGGCTTTCAAATTAAGTTCCGAATTAGTGGATGCTGCCAAAGGGAGTGGTGATGCCATACGCAAAAAGGAAGAGACTCATAGAATGGCAGAGGCAAATAGAGCTTTTGCACATTTTCGTTAA

>lcl|NC_033910.1_cds_YP_009348399.1_68 [gene=ycf1] [locus_tag=B2L33_pgp019] [db_xref=GeneID:31082759] [protein=Ycf1] [protein_id=YP_009348399.1] [location=115753..117171] [gbkey=CDS]

ATGATTTTTAAATCTTTTATACTAGGTAATCTAGTATCCTTATGCATGAAGATACTCAATTCGGTCGTTGTGGTCGGACTCTATTATGGATTTCTGACCACATTTTCCATGGGGCCCTCTTATCTCTTCCTTCTCCGAGCTCGGGTTATAGAAGAAGGAGAAGAAGGAACTGAGAAGAAGGTATCAGCAACAACAGGTTTTATTACGGGACAGCTCATGATGTTCATATCGATCTATTATGCGCCTCTGCATCTAGCATTGGGTAGACCTCATACAATAACTGTCCTAGCTCTACCCTATCTTTTGTTTCATTTCTTCTGGAATAATCACAAACACTTTTTTGATTATGGATCTACTAACAGAAATTCAATGCGTAATCTTAGCATTCAATTTGTATTCCTGAATAATCTAATTTTTCAATTATTCAACCATTTCATTTTACCAAGTTCAATGTTAGTCAGATTAGTCAACATTTATATGTTTCGATGCAACAACAAGATGTTATTTGTAACAAGTAGTTTTGTTGGTTGGTTAATTGGTCACATTTTATTCATGAAATGGGTTGGATTGATATTAGTCTGGATACAGCAAAATAATTCTATTAGATCTAATGTACTTTTTCGATCTAATAAGTACCTTGTGTCAGAATTGAGAAATTCTATGGCTCGAATCTTTAGTATTCTCTTATTTATTACCTGTGTCTACTCTTTAGGCAGAACACCGTCACCCATTTTTACTAAGAAACTGAAAGAAACCTCAGAAACGGAAGAAAGCGAGGAAGAAACAGATGTAGAAACAACTTCCGAAACGAAGGGGACTAAACAGGAACAAGAGGGATCCACCGAAGAAGATCCTTCTTCTTCCCTTTTTTCGGAAGAAAAGGAGGATCCGGACAAAATCGACGAAACGGAAGAGGTCCAAGTGAATGGAAAGGAAAAAACAAAGGATGAATTCCATTTTCACTTTAAAGAGACATGCTATAAAAATAGACCACTTTATGAAACTTTTTATCTGGATGGGAATCAAGAAAATTCGAAGTTAGAAATATTGATAGAAAAAAAAAATAAAGATCTGTTATGGGTTGAAAAACCTCTTGTAACTATTCTTTTTGATTCTAAACGTTGGAATCGTCCATTTCGATATATAAAAAATCATCAGTTTGAGAATGCTTTAAGAAGAGAAATGGGACAATATTTTTTTTCTACATGTCTAAGTGATGGAAAAGAAAGAATATCTTTTATGTATCCACCCAGTTTGTCAACTTTTTTGGAACTGATACAAAGAAAGATGTCTCTGTTCATAACACACAAATTTTCCTCTGATGAATTGTATAATCATTGGAATTCCAAGAATGAAAAAAAAAAAAAAAATCGAAGTAATGAATTTTTAAATACTTTAAATACTATAAATACTAAATAA

>lcl|NC_033910.1_cds_YP_009348400.1_69 [gene=ndhF] [locus_tag=B2L33_pgp018] [db_xref=GeneID:31082835] [protein=NdhF] [protein_id=YP_009348400.1] [location=complement(117200..119434)] [gbkey=CDS]

ATGGAACATATATATCAATATTCATGGATCATACCTTTCCTTACATTCCCAGTCCCTATGTTAATAGGAGCAGGACTTCTACTTTTTCCGGCGACAACAAAAAAACTTCGTCGTATGTGGGCTTTTCCAAGTGTTTTATTGTTAAGTATAGTTATGGTTTTTGCAATTGATCTGTCTATTCAGCAAATAAATAGCAGTTTTATTTATCAATATATATGGTCATGGACCATCAATAATGATTTTTCTTTAGAGTTCGGACACTTGATTGACCCACTTACTTCTATTTTGTTAATATTAATTACTACAGTTGGAATTATGGTTCTTTTTTATAGTGATAATTATATGTCTCATGATCAAGGTTATTTGAGATTTTTTGCTTATATGAGTTTTTTCAATACTTCAATGTTAGGATTAGTTACTAGTTCGAATTTGATACAAATTTATATTTTTTGGGAATTAGTTGGAATGTGTTCTTATCTTTTAATAGGTTTTTGGTTCACACGTCCTAGTGCATCGAATGCTTGTCAAAAAGCATTTGTAACTAATCGTGTAGGGGATTTTGGTTTATTATTAGGAATTCTTGGTCTGTATTGGATAACGGGCAGCTTCGAATTTCGAGATTTGTTCAAAATATTCAATAACTTGATTTATAATAATCAAGTTAATCTTGTATTCGTTACTTTGTGTACCTTTTTATTATTTTCCGGTGCAATTGCTAAATCGGCACAATTTCCTCTTCATGTATGGTTGCCCGATGCCATGGAGGGGCCTACCCCTATTTCGGCTCTGATACATGCTGCTACTATGGTAGCGGCGGGAATTTTTCTTGTAGCTCGCCTTTTTCCCCTTTTCCTAGTCATACCTTACATAATGAATCTAATAGCTTTAATAGGCATAATAACAGTCTTTTTAGCAGCTACTTTAGCCCTTGCTCAAAAAGATATTAAGAGAAGTTTAGCTTATTCTACAATGTCTCAATTGGGTTATACGATGTTAGCTCTAGGTATGGGGTCTTATCGAGGTGCTTTATTTCATTTGATTACTCATGCCTATTCGAAAGCATTGTTGTTTTTAGGGTCTGGATCTATTATTCATTCAATGGAAACTATTGTTGGTTATTCTCCAGATAAGAGTCAAAATATGGTTTTTATGGGCGGTTTAACAAAACATATTCCAATTACAAAAACTGCTTTTTTATTAGGAACGCTTTCCCTTTGTGGTATTCCACCCTTCGCCTGTTTTTGGTCCAAAGATGAAATTCTTAATGATAGTTGGTTATATTCACCTAGTTTCGCAATAATAGCTTGGTTCACTGCGGGATTAACTGCATTTTATATGTTTCGGATTTATTTACTTATTTTTGAAGGATATTTCAATCTTAATTTTAAAAATTACAGTGGAAAAAAAAACGGTTCATTTTATTCAATATCTTTATGGGGTAAAGAAGGATCAAAAACGTTTAAAAAAAATTTTCGTTTATTACCTTTATTAAAAAAAACGAATAATGACAGGACTTCCTTTTTTCGGAAGAATACATATAAAATTGATGTTAATGTAAGAAATATGAGATGGGCCTTTATTACTGTTAATAATTTTAACACTAAAAGGATTTTTTCCTACCCGCGGGAATCCGACAATACTATGTTATTTCCTATGCTTGTCTTCGTACTATTTTCTGTCTTTATTGGAGCTATAGGAATTCCTTTCAATCAATTCAATCAAGAAGAAATCAAGTTGGATATATTGTCAAAACTACTAACTCCGTCTTTTAAACTTTTGCATGAAAATGAAGAAAATTATGTGGATTGGTATGGATTTGTAATAAATGCAACTTTTTCAGTTAGTATAACTTTTTTCGGAATATTTATCGCGTCCTCCTTCTATCAGCCTGTTTATTTATCTTTACAAAATTTGAATTTCTTTAATTCATCCGCTAAAAAAGGCTTGAAGAAAATTCTTTCGGACAAAATAAAAAATCAGATATATAATTGGTCCTATAATCGAGGTTACATAGATTCCTTTTATGCAATGTCTTTTATTAGGGGTATAAGAAAATTAGCTGAATTAATTTCTTTTTTTGATAAACGAATAATTGATGGAATTATAAATGGAGTCGGTGTTATCAGTTTCTTTGTAGGAGAAAGCATAAAATATGCAGGAAGCGGTCGCATTTCTTCTTATCTTTTATTGTATGTATTTTATGCAGTAATTTTTTTATTAATTTACTATACTTAG

>lcl|NC_033910.1_cds_YP_009348401.1_70 [gene=ccsA] [locus_tag=B2L33_pgp017] [db_xref=GeneID:31082760] [protein=CcsA] [protein_id=YP_009348401.1] [location=120220..121188] [gbkey=CDS]

ATGATCGTTTCGAATTTCGAACATATATTAACTCATATATCTTTTTCAGTCGTGTCAATTGTAATTACAATTCATTTGATAACCTTATTAGTCGATGAATTCGTAGAACTATATGATTCGTCAGAAAAGGGCATGTTAACGACTTTTTTCTGTATAACCGGATTATTAGTTACTCGTTGGTTTTTTGGGGGACATTTACCATTAAGTGATTTATATGAATCATTAATCTTTCTTTCATGGGCATTTTGTGTTATTCATATAATTCCGTATTTTAAAAAATATAAAAATTATTTAAGCGCAATAACCGCGCCAAGTACTTTTTTGACTCAAGGGTTTGCCACTTCGGGTCTTTTAAAAGGCATGCATCAATCCGAAATCTTAGTACCCGCTCTTCAATCCCAGTGGTTAATGATGCACGTAAGTATGATGATTTTTGGCTATGCAGCTCTTTTGTGTGGATCATTATTATCAGTAGCATTTCTAGTAATCAGATTTCAAAAAATTAGAATAATTTTTGATAAAAGCACTAATTTTTTAAATGATTCGTTTTACTTTAATGAGATACAATATATAACGGAAGGAAAGAATGTTTTAAGAAATAGTTCCTTTCTTTCCTCTAGGAATTATTATAGGTTTCAATTAATTCAACAATTAGATGACTGGAGTTATCGGATTATAAGTATAGGTTTTTTTTTTTTAACTATAGGTATTCTTTCGGGAGCAGTCTGGGCTAATGAAGCATGGGGATCATATTGGAATTGGGACCCAAAGGAAACTTGGGCATTTATTACATGGACCATATTCGCGGTTTTTTTTCATACTCGAACAAATAAAAATTTGGAGAGTTTAAATTCGGCAATTATAGCTTCTATCGGTTTTCTTATAATTTGGATATGCTATTTTGGAGTTAATTTATTAGGAATAGGACTACATAGTTATGGTTCATTTACATTACCAATTAACAATTGA

>lcl|NC_033910.1_cds_YP_009348402.1_71 [gene=ndhD] [locus_tag=B2L33_pgp016] [db_xref=GeneID:31082837] [protein=NdhD] [exception=RNA editing] [protein_id=YP_009348402.1] [location=complement(121376..122878)] [gbkey=CDS]

ACGAATTCTTTTCCTTGGTTAACAATATTTGTAGTTTTACCGATAGCCGCGGGTTTATTAATTTTCTTTTTCCCTCATAGAGGAAATAAGCTAATTAGGTGGTATACTTTATATATATGTATAGCGGAGCTCCTTTTAATAACTTATGCATTCTCTTATTATTTCCAATTTGAGGACCCATTAATCCAATTAGCAGAACATTATAAATGGATCCCTTTTTTTGATTTGTACTGGAGATTGGGAATAGATGGTTTTTCTTTAGGACCCATTTTACTGACAGGATTTATCACCACTTTAGCTACTTTAGCGGCTTGGCCGATTACTCGGGATTCCCGATTATTCCATTTTCTGATGTTAGCAATGTATAGCGCTCAAATAGGATTATTTTCATCTCAAGATCTTTTACTTTTTTTTATCATGTGGGAGTTAGAATTAATTCCCGTCTATTTACTTCTATCCATGTGGGGGGGAAAGAAACGTCTGTATTCAGCTACAAAGTTTATTTTGTATACTGCAGGAGGTTCGGTTTTTTTATTACTGGGAGCTTTGGGTCTCGCTTTATATGGTTCCGATGAACCGACATTCAATTTTGAAACATCAGCGAATCAATCATATCCTGTGGCACTAGAAATATTTTTCTATATTGGATTTTTTATTGCTTTTGCTGTCAAATCACCGATTATACCTTTACATACATGGTTACCAGACACTCATGGGGAAGCACATTACAGTACTTGTATGCTTCTAGCTGGCATCCTATTAAAAATGGGGGCGTATGGATTGATTCGAATCAATATGGAATTATTGCCTCATGCTCATTCTATCTTTTCCCCTTGGTTGATAATAATAGGCGTAATGCAAATAATCTATGCAGCTTCAACATCTCCCGGTCAACGAAATTTAAAAAAAAGAATAGCCTATTCTTCTGTATCTCATATGGGTTTCATAATTATAGGAATTTGCTCTATAAGTGAGATGGGACTCAATGGAGCCATTTTACAAATTCTATCACATGGATTTATTGGTGCTGCACTTTTTTTCTTGGCAGGAACTGGTTATGATAGAATACGTCGTCTTTATCTTGACGAAATGGGTGGAATGGCTCCCCTAATGCCAAAACTATTCACGACCTTCAGTATTTTATCACTAGCTTCCCTTGCATTACCGGGCATGAGTGGTTTTTTTGCCGAATTGGTCGTCTTTTTTGGACTAATTAGCGGACAAAAATACCTTTTAACGGCAAAAATCTTCATTACTGTCGTAATGGCAGTTGGAATGATATTAACTCCTATTTATTTATTATCTATGGTACGACAGATGTTCTATGGATACAAATTGTTTAATGCCCCAAACTCTTATTTTTTTGATTCGGGACCTCGGGAATTATTTGTTTCGATCTCTATCCTTCTGCCTGTAATAAGTATTGGTATTTATCCGGATTTCGTTTTCTCATTATCAATTGACAGAGTCGAAGCTATTCTATCTAATTATTTTTATAGATAG

>lcl|NC_033910.1_cds_YP_009348403.1_72 [gene=psaC] [locus_tag=B2L33_pgp015] [db_xref=GeneID:31082838] [protein=PsaC] [protein_id=YP_009348403.1] [location=complement(123018..123263)] [gbkey=CDS]

ATGTCACATTCAGTAAAAATTTATGATACATGTATAGGGTGTACTCAATGTGTCCGAGCCTGCCCCACGGATGTATTAGAAATGATACCTTGGGATGGGTGTAAATCGAAGCAAATTGCTTCTGCTCCAAGAACAGAGGATTGTGTCGGTTGTAAAAGATGTGAATCTGCTTGTCCAACGGATTTCTTGAGTGTTCGAGTTTATTTATGGCATGAAACAACTCGAAGCATGGGTCTAGCTTATTGA

>lcl|NC_033910.1_cds_YP_009348404.1_73 [gene=ndhE] [locus_tag=B2L33_pgp014] [db_xref=GeneID:31082839] [protein=NdhE] [protein_id=YP_009348404.1] [location=complement(123516..123821)] [gbkey=CDS]

ATGATGCTCGAACATGTACTTATTTTGAGTGCTTGTTTATTTTCTATCGGTATCTATGGATTGATCATGAGTCGAAATATGGTTAGAGCCCTTATGTGTCTTGAACTTATACTGAATGCTGTTAATATAAATTTCGTAACATTTTCTGATTTTTTTGATAGTCGCCAACTAAAAGGAAATATTTTTTCAATTTTTGTTATAGCTATCGCAGCCGCTGAAGCAGCTATTGGACTGGCTATTGTTTCGTCTATTTATCGTAACAGAAAATCCACCCGTATCAATCAATCGAATTTATTGAATAAGTAG

>lcl|NC_033910.1_cds_YP_009348405.1_74 [gene=ndhG] [locus_tag=B2L33_pgp013] [db_xref=GeneID:31082840] [protein=NdhG] [protein_id=YP_009348405.1] [location=complement(124044..124574)] [gbkey=CDS]

ATGGATTTACCTGGACTAATACATGATTTTCTTTTAGTCTTTCTGGGGTTAGGTCTTATATTAGGAGGTTTAGGAGTAGTATTATTTACCAACCCCATTTTTTCTGCCTTTTCATTAGGATTCGTTCTTGTTTGTATATCTTTATTTTATATTTTATCAAACTCTCATTTTGTAGCTGCTGCACAGCTCCTTATTTATGTGGGAGCTATAAATGTTTTAATTATATTTGCCGTAATGTTCATGAATGGTTCAGAATATTACAAAGATTTTAATCTTTGGACTGTTGGAAATGGAGTTACTTCCTTAGTTTGTACAAGTATTTTTGTTTCACTAATTACTATTATTCCAGATACGTCATGGTACGGAATTATTTGGACTACAACATCAAATCAGATTATAGAACAAGATTTGATAACTAATGGTCAACAAATTGGAATTCATTTAGCAACAGATTTTTTTCTTCCATTTGAATTCATTTCAATAATTCTTTTAGTTGCTTTGATAGGTGCAATTGCTGTGGCTCGTCAGTAA

>lcl|NC_033910.1_cds_YP_009348406.1_75 [gene=ndhI] [locus_tag=B2L33_pgp012] [db_xref=GeneID:31082841] [protein=NdhI] [protein_id=YP_009348406.1] [location=complement(125036..125533)] [gbkey=CDS]

ATGTTTCCCATGGTAACTGGATTCATGAATTATGGGCAACAAACCATACGAGCTGCAAGGTACATTGGTCAAAGTTTCATGATTACCTTATCCCATGCAAATCGTTTACCTGTAACTATTCAATATCCTTATGAAAAATTAATAACATCGGAGCGTTTCCGTGGTCGAATCCATTTTGAATTTGATAAATGCATTGCTTGTGAAGTATGTGTTCGTGTATGTCCTATAGATCTGCCTGTTGTTGATTGGAAATTGGAAACTGACATTCGAAAGAAACGGTTGCTAAATTACAGTATTGATTTCGGAATCTGTATATTTTGCGGCAACTGCGTTGAGTATTGTCCAACAAATTGTTTATCAATGACAGAAGAATATGAACTTTCTACTTATGATCGTCATGAATTGAATTATAATCAAATTTCTTTGGGTCGTTTACCAATGTCAGTAGTTGAGGATTATACGATTCGAACAATTTTAAATTCAACTAAAATCAACTAA

>lcl|NC_033910.1_cds_YP_009348407.1_76 [gene=ndhA] [locus_tag=B2L33_pgp011] [db_xref=GeneID:31082842] [protein=NdhA] [protein_id=YP_009348407.1] [location=complement(join(125625..126163,127280..127832))] [gbkey=CDS]

ATGATAATTGATACAACAGAAGTACAAGCTATCCATTCTTTTTCTAGGTTAGAATCCTTAAACGATGTCTATGGAATTATATGGGAGTTTATTCCTATTTTGATTCTTGTATTGGGAATCACGATAAGCATACTCGTAATTGTATGGTTAGAAAGAGAAATATCCGCAGGGATACAACAACGTATTGGACCCGAATATGCGGGTCCTTTAGGAGTTCTTCAAGCTCTAGCGGATGGTACAAAACTACTTTTCAAAGAGAATCTTTTTCCATCTAGGGGGGATACTTATTTATTCAGTATTGGACCATCTATAGCAGTCATATCAACTCTATTAAGCTATTCAGTAATTCCTTTTGGCTATCACTTTGGTTTAACTGATCTAAATATTGGTGTTTTTTTATGGATTGCCATTTCAAGTATTGCTCCTATTGGACTTCTTATGTCAGGGTATGGATCAAATAATAAATATTCCTTTTTAGGTGGTCTACGAGCTGCTGCTCAATCGATTAGTTATGAAATACCTTTAACTATTTGTGTGTTATCCATATCTCTACTATCTAATAGTTCAAGTACAGTTGATATAGTTGAAGCCCAATCAAAATCTGGTTTTTGGGGTTGGAATTTATGGCGTCAACCGATAGGATTTTTTATTTTTTTTATTTCTTCTCTAGCAGAATGTGAAAGATTACCTTTTGATTTGCCAGAAGCAGAAGAAGAATTAGTAGCAGGTTATCAAACGGAATATTCGGGTATCAAATTTGGTTTATTTTATATTGCTTCCTATCTAAACTTATTAGTTTCTTCATTATTTGTAACAGTTCTTTACTTGGGCGGTTGGAATATTTCTATTCCGTATATATTCGTTCATGAATTTTTTGAAATAAATAGCATAAGCGGAGTCGTTGGACCAACAATTGGTACCTTTATTACATTAGTTAAAACTTATTTGTTCTTGTTCATTCCTATCACAACAAGATGGACTTTACCGAGACTAAGAATGGACCAACTTCTAAATCTTGGATGGAAATTTCTTTTACCTATCTCTCTCGGTAATCTATTATTAACAACCTCTTTTCAACTCCTTTCACTATAA

>lcl|NC_033910.1_cds_YP_009348408.1_77 [gene=ndhH] [locus_tag=B2L33_pgp010] [db_xref=GeneID:31082843] [protein=NdhH] [protein_id=YP_009348408.1] [location=complement(127834..129015)] [gbkey=CDS]

ATGAATATACCAGCTAAACGAAAAGACCTTATGATAGTCAATATGGGTCCCCAGCACCCATCAATGCACGGTGTTCTTCGACTCATTGTTACTCTAGATGGTGAAGATGTTATTGACTGTGAACCAATATTAGGTTATTTACACAGAGGAATGGAAAAAATTGCGGAAAATCGAACAATTATACAATATTTGCCCTATGTAACACGGTGGGATTATTTAGCTACTATGTTCACAGAAGCAATAACAGTAAATGGTCCCGAATTGTTAGGAAATATTCAAGTGCCCAAAAGAGCTGGCTATATCAGAGTAATTATGTTGGAATTAAGTCGTATAGCTTCTCATTTGTTATGGCTTGGACCTTTTATGGCAGATATTGGTACACAGACGCCTTTCTTCTATATTTTTAGAGAGAGAGAGTTAATATATGATTTATTTGAAGCTGCCACTGGTATGAGAATGATGCATAATTATTTTCGTATCGGGGGGGTAGGGGCTGATCTACCTCATGGTTGGATAGATAAATGTTTAGATTTTTGCGATTATTTTTTAACAGGAGTTGATGAATATCAAAAACTTATTACGCGAAATCCTATTTTTTTAGAACGAGTTGAAGGAGTCGGTATTGTTGGTGCAGAGGAAGCAATAAATTGGGGTTTATCGGGACCAATGCTACGGGCTTCCGGAATACAATGGGATCTTCGTAAAGTTGATCATTATGAGTCTTACGACGAATTTGATTGGGAAGTCCAGTGGCAAAAAGAAGGAGATTCATTAGCTCGTTATTTAGTCCGAATTGGTGAAATGATGGAATCTATAAAAATTATTCAACAGGCTCTTGAAGGAATTCCGGGTGGGCCCTATGAGAATTTAGAAACCCGACGTTTTGATAGAGAAAAGGATCCAGAATGGAACGATTTCGAATATCGATTCATTAGTAAAAAAACTTCACCTACTTTTGAATTACCGAAACAAGAACTTTATGTCAGAGTGGAAGCCCCAAAAGGAGAATTAGGAATTTTTCTGATAGGGGATCAGAGCGGCTTTCCTTGGAGATGGAAAATTCGCCCGCCGGGTTTTATCAATTTGCAAATTCTTCCTGAATTAGTTAAAAGAATGAAATTGGCTGATATTATGACAATACTAGGTAGTATAGATATCATTATGGGAGAAGTTGATCGTTGA

>lcl|NC_033910.1_cds_YP_009348409.1_78 [gene=rps15] [locus_tag=B2L33_pgp009] [db_xref=GeneID:31082844] [protein=ribosomal protein S15] [protein_id=YP_009348409.1] [location=complement(129128..129400)] [gbkey=CDS]

ATGGTAAAAAATGCATTCATTTCAGTTATTTCACAAAAAGAAAAAGACGAAAACAAGGGGTCTGTTGAATTTCAAATAGTAACTTTCACTAATAAGATACGAAGACTTACTTTACATTTGGAATTGCATAGAAAAGACTACTTATCTCAAAGAGGTTTGCGGAAAATTTTAGGAAAACGCCAACGACTGTTGGCTTATTTAGAAAAGAAAAATAGCGTACGTTATAACGAATTAATTAGCCGGTTGGATATTCGGAAATTAAAAACTCGTTAA

>lcl|NC_033910.1_cds_YP_009348410.1_79 [gene=ycf1] [locus_tag=B2L33_pgp008] [db_xref=GeneID:31082845] [protein=Ycf1] [protein_id=YP_009348410.1] [location=complement(129832..135561)] [gbkey=CDS]

ATGATTTTTAAATCTTTTATACTAGGTAATCTAGTATCCTTATGCATGAAGATACTCAATTCGGTCGTTGTGGTCGGACTCTATTATGGATTTCTGACCACATTTTCCATGGGGCCCTCTTATCTCTTCCTTCTCCGAGCTCGGGTTATAGAAGAAGGAGAAGAAGGAACTGAGAAGAAGGTATCAGCAACAACAGGTTTTATTACGGGACAGCTCATGATGTTCATATCGATCTATTATGCGCCTCTGCATCTAGCATTGGGTAGACCTCATACAATAACTGTCCTAGCTCTACCCTATCTTTTGTTTCATTTCTTCTGGAATAATCACAAACACTTTTTTGATTATGGATCTACTAACAGAAATTCAATGCGTAATCTTAGCATTCAATTTGTATTCCTGAATAATCTAATTTTTCAATTATTCAACCATTTCATTTTACCAAGTTCAATGTTAGTCAGATTAGTCAACATTTATATGTTTCGATGCAACAACAAGATGTTATTTGTAACAAGTAGTTTTGTTGGTTGGTTAATTGGTCACATTTTATTCATGAAATGGGTTGGATTGATATTAGTCTGGATACAGCAAAATAATTCTATTAGATCTAATGTACTTTTTCGATCTAATAAGTACCTTGTGTCAGAATTGAGAAATTCTATGGCTCGAATCTTTAGTATTCTCTTATTTATTACCTGTGTCTACTCTTTAGGCAGAACACCGTCACCCATTTTTACTAAGAAACTGAAAGAAACCTCAGAAACGGAAGAAAGCGAGGAAGAAACAGATGTAGAAACAACTTCCGAAACGAAGGGGACTAAACAGGAACAAGAGGGATCCACCGAAGAAGATCCTTCTTCTTCCCTTTTTTCGGAAGAAAAGGAGGATCCGGACAAAATCGACGAAACGGAAGAGGTCCAAGTGAATGGAAAGGAAAAAACAAAGGATGAATTCCATTTTCACTTTAAAGAGACATGCTATAAAAATAGACCACTTTATGAAACTTTTTATCTGGATGGGAATCAAGAAAATTCGAAGTTAGAAATATTGATAGAAAAAAAAAATAAAGATCTGTTATGGGTTGAAAAACCTCTTGTAACTATTCTTTTTGATTCTAAACGTTGGAATCGTCCATTTCGATATATAAAAAATCATCAGTTTGAGAATGCTTTAAGAAGAGAAATGGGACAATATTTTTTTTCTACATGTCTAAGTGATGGAAAAGAAAGAATATCTTTTATGTATCCACCCAGTTTGTCAACTTTTTTGGAACTGATACAAAGAAAGATGTCTCTGTTCATAACACACAAATTTTCCTCTGATGAATTGTATAATCATTGGAATTCCAAGAATGAAAAAAAAAAAAAAAATCGAAGTAATGAATTTTTAAATAGAGTCCAAGCTCTGGATAAAGGCTATATTGCTTCGAAAACATTGGAAAAAAAGACTCGATTGTGTAATGATAAAACTAAAGAACAGTACTTACCTAAAACATATGATCCCTTATTGAATGGATCCTACCGAGGAAAAATACAGTTTTTTTTTTCATCCTCAATCCGAAATAAAACTTTCCGAACAATTTTTAGAGAAAGATTTTGGATAAATAAAATTCATCTTATTCTTCTTATTACTAATTATCAAGAATTTGAAACAAAAAGGAATGTGTTTAATAGTAATCATGAATCGTTTTTAAGAGAAATTGCTTATTTATTAAACTTAATTAATGAATTTGGCGGAAAATCAAGATCAAGTTTCAATTTTAAGGAACTCCCTTTTTTCCCAGATAACACCGAAGAAAAAGTGTATTTAGAAAATCAAATAAAAATTTTACAATTTTTATTTGATACAGTTATAGCGAATCCAAAAAAGAAAACAAGTAAAAAATTTTCTACTGGACTAAAAGAAATAAGTAAACAAGTTCCTCGATGGTCATACAAATTAATTGACGATTTAGAACAACAAGAGGGCAAAGATGATGCAGAAAACCTGGCGGAAGATCATGAAATTCGTTCACGAAAAGCCAAACTTCTAATGATTTTTAGTGATAATATAATTATTTTTAATGATAATCAAAAAAATAGTGATACTTACAATAATAACAGGAATTCGGACCCAATATACATAGACCAAGTTACTTTCATCCGTTATTCCCAACAATCGGACTTTCGGCGAGACATAATAAAAGGATCCATGCGAGCACAAAGACGTAAAATAACTATTTTTGAACTATTTCAAGCAAATGTACATTCTCCTATTTTTTTGGACAGAATAAAAAAATCTTTTTTTTTTTCTTTTGATATTGATATTTATGAACTGATGAAAACATTGTTTATAAATTCTATATCTAAAAACACAGAATTAAAAATTTCGAATTCTACTTATATAGAGAAAAAAAAAAAAAAAAGAAAGAAAAAAGAGGAGGCCAAAAGAAAAGATAACAAAAGAGAGGACAAAGCACGAATAAAAATAGCTGAAACTTGGGATAGGGTTTTTCTTGCTCAAGTACTAAGAGGTTGTGTTTTAATAACCCAATCAATTCTTCGAAAATATATTATATTACCCTCATTAATAATAACTAAGAACATCATTCGTATATTATTTTTTCAAACTCCCGAATGGTCCGACGATTTAAAAGATTGGGGTAGAGAAATGCATGTTAAATGCACCTATAATGGAGTTCAATTATCAGAAAAAGAATTTCCGAAAAATTGGTTAACGGGTGGGATTCAAATAAAGATCCTATTTCCTTTTCGTTTAAAACCTTGGCACAGATCGAAGGTAAAATTCCCTCATAAAAGTAAAAAAAAAAAGAAAATAGAACAAAAGGATTTTTGTTTTTTAACAGTTTGGGGAATGGAAGCGGAACTTCCTTTTGGTTCTCCCCGAAAACGGCTTTCACTTTTTAAACCCATCTTTAAAAAACTTGCAAAAAAAATTATAAAGATAAAAAAAAGTGGTTTTCGAGTTATAAGAATTTTCGAAGAAAGAAGAAAATTATTTCAAAATTTATCAAAAGAAAAAAAACACTCGGTCATCAAAAACATTTTTTTTCGACAAGAAATAATAACGAAACTTTCAAAATCAAAAAGAAATCTAAAATTTTTATCGGAATTTAGAGAAGTAGATGAATTAAATGAAAGTAAAAAAAAAAAAGAGTCGATACTCAATAATAAGAATCGGACGGTTTTGAAATTGTCCACCCCAATTCGACCTATACCTTCTACAAATTATTCACTGATGAAAAAAAAAAAGAAAGATCTTTCTACTAGAAAAAAGAGAATTCTAAATCAAATAGAAAAAATTACAAAAGAAAAGGAAAAAAAAATGCGAACCTCAGAAGTAAATATTAGTCTTAACAAAAAAAAAAAAAGTTCTAATGCTAAAAAAATTAAATCATCAAACAATATTTCACACATATTAAAAAAAAAAAATGTTCGATTAGTGCGTAAATTTTACTTTTTTATAAAATTTTTGATTGAAAATATATACTTAGATATCTTTTTAAGTATCATTAATATTCCAAGGCTCAATGCACAGCTTTTTCTTGAATCAATAAAAAATTTTATTACTAAACACATTTGCAATAATGAATCAAATCACAAAAAAATTGATAAACCAAATCAAAAAAATTTTTACTTTATTTCGATTATAAAAAAGTCAACAGATACAGAGATTGCTGTTATTAATAGGAATTCACAAATTTTTTGTGACATAGCTTTCTTATCACAAGCGTATGTATTTTACAAATTATCACAAACCAAAATTCTTAACTTCTATAAGTTAAGATCGATCTTTCAATACCATAATCTTTTTCTTAAGAACGAAATAAAAGATTATTTTCGAACCCAAGGATTATTTAATTTTGAATTAAAAGAAAACAAAATTCAAAAGTCTTTTTCTTTAATCAATCAATGGAAAAACTGGTTAAGAAGTCATTATCAATATAAATATGATTTATCTCAGCTTAGATGGTCTAGATTAATACCAGAAAAATGTCGAAATAGAATCTATCAACACCATATGGTTGAAAATAAAAAATTAAGCAAATGGGATTTACATGAACAAAACGAATTAATTCATTATGAAAAAAAAAATAATTTTGAGGCAGACGTTTTTGCGAATCAAAAGAATAATTTTAAAAAACACTCTAGATATAGTCTTTTATCCTATAAATCTATTAATTCTGAAAAAAAGAAGGACTTATTTATTTACGAATCACCAACTAATAAAGAAGAGATTCTTTATAATTCTAACACAAATAAACGAAAATTTTTTGACATCTTAGAAGGTATTCCTATCACTAATTATATAGTGGAAGATGATATTATCAATATAGATAAAAACCCACATAGAAAATATTTTGATTGGCGAATTATCAATTTTTGTCTTAGAAACAGGGTCGATATTGAGTCCTGGATCGATACCGGAAGCAAAAAAAAAAAAAAGACTAAGACTACAACTAAAAAATATCAAATAATTGATAAAAGTGATAAGAAAAATATTTCTTTTCTTCCAATTTGCCAAGATCAAGAAATCAATTCATCCAACCAAAAACCTTTTTTTTTTGATTGGATGGGAATGAATGAAGAAATAGAAAATAGTCTTCTTTCGAATTTTGAACTTTGGTTCTTTCGAAAATTTGTAATACTTTACAACACATATAAGAGAAAACCATGGACAATACCCATTCAATTTCTTCTTTTAAATTTTCATAGAACTAAAAATATTAGTAAAAATAAGAAAATCAACGGGAATAAAAAAGGCGACCTTCTTATATCTATACCATCACCATCGAATGAAAAAAAAATTATTGAATTCGAAAATCAAAATCATCAAGAAAACGAATCTGACGACCAAATGGATTTTCAAGCAGTTTTCACAAATGAAGAAAAAGATATTGAAGAAGATTCTATGGGATTAGATATGAAAAACCATAGAAATCAAAATCAAAACAAAAGTCATACGGAAGTAGAGCTTGATTTCTTCCTAAAAGAGTATTTATATTTTCAATTAAGATGGAATGGTTCTTTAAATCAAAAAATAATTGATAATATCAAAACATATTGTTTTCTGCTTAGACTAAGAAATCCACGCGAAATTATTATATCTTCTATTCAAAGGCAAGAAATAAATCTGAATATTCTGATGGTTCAGAAAGATATAACTCTTACAGAATTGATGAAAAAGAGAATATTGATTATCGAACCTGTCCGTCTGTCGGTAAAAACTGATGGACAATTTATTTTATATCAAATGGTAGATATTTTATTAGTTCATAAGAACAAAGAACAAATTAATAAAAAATATAGAAAACAATTCTATGTTGATAAAAATAAAAAGAATTTTACCGAATCTATTGACAGCTATCACAATATAATTGGAAATAGACAAAAAAATGATTATGATTTACTTGTTCCTGAAAATATTTTATCCCCTAAATGTCGTAGAGAATTAAGAATTCGAATTTCTTTCAATTTACAAAATAAAAACGATATTCATATAAATACAGAAATTTGCAATGGGAATAACATAAAAAAAGTCAGTCCCATTTTGGATAAAAGCAAACTTTTTTGGAGAGAAAAAAAGAAACTAATTAAATTGAAATTTTGTCTTTGGCCAAATTTTCGATTAGAGGATTTAGCTTGTATGAATCGCTATTGGTTCGATACTAATAATGGAAGTCGGTTCAGTATGTTAAGGATATATATATATCCGCGCTTGAAATTTTAG

>lcl|NC_033910.1_cds_YP_009348411.1_80 [gene=rps7] [locus_tag=B2L33_pgp007] [db_xref=GeneID:31082769] [protein=ribosomal protein S7] [protein_id=YP_009348411.1] [location=147141..147608] [gbkey=CDS]

ATGTCACGTCGAGGTACTGCAGAAGAGAAAACTGCAAAATCCGATCCAATTTATCGTAATCGATTAGTTAACATGTTGGTTAACCGTATTCTGAAACACGGAAAAAAATCATTGGCTTATCAAATTATCTATCGAGCCATGAAAAAGATTCAACAAAAGACAGAAACAAATCCACTATCTGTTTTACGTCAAGCAATACGTGGAGTAACTCCCGATATAGCAGTAAAAGCAAGACGTGTAGGCGGATCGACTCATCAAGTTCCCATTGAAATAGGATCCACACAAGGAAAAGCACTTGCCATTCGTTGGTTATTAGGGGCATCCCGAAAACGTCCGGGTCGAAATATGGCTTTCAAATTAAGTTCCGAATTAGTGGATGCTGCCAAAGGGAGTGGTGATGCCATACGCAAAAAGGAAGAGACTCATAGAATGGCAGAGGCAAATAGAGCTTTTGCACATTTTCGTTAA

>lcl|NC_033910.1_cds_YP_009348412.1_81 [gene=ndhB] [locus_tag=B2L33_pgp006] [db_xref=GeneID:31082847] [protein=NdhB] [partial=5'] [protein_id=YP_009348412.1] [location=join(<147922..148698,149377..150132)] [gbkey=CDS]

ACGATCTGGCATGTACAGAATGAAAACTTCATTCTCGATTCTACGAGAATTTTTATGAAAGCCTTTCATTTGCTTCTCTTCGATGGAAGTTTTATTTTCCCAGAATGTATCCTAATTTTTGGCCTAATTCTTCTTCTGATGATCGATTCAACCTCTGATCAAAAAGATATACCTTGGTTATATTTCATCTCTTCAACAAGTTTAGTAATGAGTATAACGGCCCTATTGTTCCGATGGAGAGAAGAACCTATGATTAGCTTTTCGGGAAATTTCCAAACGAACAATTTCAACGAAATCTTTCAATTTCTTATTTTACTATGTTCAACTCTATGTATTCCTCTATCCGTAGAGTACATTGAATGTACAGAAATGGCTATAACAGAGTTTCTCTTATTCGTATTAACAGCTACTCTAGGAGGAATGTTTTTATGCGGTGCTAACGATTTAATAACTATCTTTGTAGCTCCAGAATGTTTCAGTTTATGCTCCTACCTATTATCTGGATATACCAAGAAAGATGTACGGTCTAATGAGGCTACTATGAAATATTTACTCATGGGTGGGGCAAGCTCTTCTATTCTGGTTCATGCTTTCTCTTGGCTATATGGTTCGTCCGGGGGAGAGATCGAGCTTCAAGAAATAGTGAATGGCCTTATCAATACACAAATGTATAACTCCCCAGGAATTTCAATTGCGCTTATATTCATCACTGTAGGAATTGGGTTCAAGCTTTCCCCAGCCCCTTCTCATCAATGGACTCCTGACGTATACGAAGGATCTCCCACTCCAGTCGTTGCTTTTCTTTCTGTTACTTCGAAAGTAGCTGCTTCAGCTTCAGCCACTCGAATTTTCGATATTCCTTTTTATTTCTCATCAAACGAATGGCATCTTCTTCTGGAAATCCTAGCTATTCTGAGCATGATAGTGGGTAATCTCATTGCTATTACTCAAACAAGCATGAAACGTATGCTTGCATATTCGTCCATAGGTCAAATCGGATATGTAATTATTGGAATAATTGTTGGAGACTCAAATGGTGGATATGCAAGTATGATAACTTATATGTTCTTCTATATCTCCATGAATCTAGGAACTTTTGCTTGTATTGTATTATTTGGTCTACGTACCGGAACTGATAACATTCGAGATTATGCAGGATTATACACGAAAGATCCTTTTTTGGCTCTCTCTTTAGCCCTATGTCTCTTATCCCTAGGAGGTCTTCCTCCACTAGCAGGTTTTTTCGGAAAACTCCATTTATTCTGGTGTGGGTGGCAGGCAGGCCTATATTTCTTGGTTTTAATAGGACTCCTTACGAGCGTTGTTTCTATCTACTATTATCTAAAAATAATCAAGTTATTAATGACTGGACGAAACCAAGAAATAACCCCTCACGTGCGAAATTATAGAAGATCCCCTTTAAGATCAAACAATTCCATCGAATTGAGTATGATTGTATGTGTGATAGCATCTACTATACCAGGAATATCAATGAACCCGATTATTGAAATTGCTCAAGATACCCTTTTTTAG

>lcl|NC_033910.1_cds_YP_009348413.1_82 [gene=ycf15] [locus_tag=B2L33_pgp005] [db_xref=GeneID:31082770] [protein=Ycf15] [protein_id=YP_009348413.1] [location=complement(151197..151334)] [gbkey=CDS]

ATGTTACTGCTGAAACATGAAAGAATTGAAATCTTAGATCAAAACACTATGTATGGATGGTATGAACTGCCTAAACAAGAATTCTTGAACAGCGAGCAAGCAGAACGATTACTCACTACATCAAAAAATTTCCATTAA

>lcl|NC_033910.1_cds_YP_009348414.1_83 [gene=ycf2] [locus_tag=B2L33_pgp004] [db_xref=GeneID:31082849] [protein=Ycf2] [protein_id=YP_009348414.1] [location=complement(151467..158357)] [gbkey=CDS]

ATGAAGGGACATCAATTCAAATCCTGGATTTTCGAATTGAGAGAGATATTGAGAGAGATCAAGAATTCTCACTATTTCTTCGATTCATGGACCCAATTCAATTCAGTGGGATCTTTCATTCACATTTTTTTCCATCAAGAACGTTTTATAAAACTCTTGGACTCCCGAATTTGGAGTATCTTACTTTCACGCAATTCACAGGGTTCAACAAGCAATCGATATTTCACGATCAAGGGTGTAGTACTCTTTGTAGTAGTGGTCCTTATATATCGTATTAACAATCGAAAGATGGTCGAAAGAAAAAATCTCTATTTGACAGGGCTTCTTCCTATACCTATGAATTCCATTGGACCCAGAAATGATACATTGGAAGAATTCTTTGGGTCTTACAATATCAATAGGTTGATTGTTTCGCTCCTGTATCTTCCAAAAGGAAAAAAGATCTCTGAGAGCTCTTTCCCGGATCTGAAAGAGAGTACTTGGGTTCTCCCAATAACTAAAAAGTGTATCATGTCTGAATCTAACTGGGGTTCACGGCGGTGGAGGAACTGGATCGGAAAAAAGAGGGATTCTAGTTGTAAGATATCTAATGAAACCGTCGCTGGAATTGAGATCTCATTCAAAGAAAAAGATATCAAATATCTGGAGTTTCTTTTTGTATATTATATGGATGATCCGATCCGCAAGGACCAGGATTGGGATTTGTTTGATCGTCTTTCTCCGAGGAAGGGGCGAAACAGAATCAACTTGAATTCGGGACAGCTATTGGAAATCTTAGTGAAAGACTGGATTTGTTATCTCATGTTTGCTTTTCGTGAAAAAATACCAATTGAAGTGGAGGGTTTCTTCAAACAACAAGGAGCTGGGTCAACTATTCAATCAAATGATATTGAGCATGTTTCCCATCTCTTCTCGAGAAAGAAGTGGGCTATTTCTTTGCAAAATTGTGCTCAATTTCATATGTGGCAATTCCGCCAAGATCTCTTCGTTAGTTGGGGGAATAATCCGCACGAATCGGATTTTTTGAGGAACATATCGAGAGAGAATTGGATTTGGTTAGACAATGTGTGGTTGGTAAACAAGGATCGGTTTTTTAGCAAGGCACGGAATATATCGTCAAATATTCAATATGATTCCACAAGATCTAGTTTCGTTCAAGGAAGGAATTCTAGCCAATTGAAGGGATCTTCTGATCAATCCAGAGATCATTTCGATTCCATTAGTAATGAGGATTCGGAATATCACACATTGATCAATCAAAGAAAGATTCAACAACTAAAAGAAAGATCGATTCTTTGGGATCCTTCCTTTCTTCAAACGGAACGAACAGAGATAGAATCAGACCAATTCCCTAAATGCCTTTCTGGATATTCCTCAATGTCCCGGCTATTCAGGGAAGGTGAGAAGGAGATGAATAATCATCTGCTTCCGGAAGAAATCGAAGAATTTCTTGGGAATCCTACAAGATCCATTCGTTCTTTTTTCTCTGACAGATCGTCAGAACTTCATCTGGGTTCGAATCCTACTGAGAGATCCACTAGAGATCAGAAATTGTTGAAGAAAGAACAAGATGGTTCTTTTGTCCCTTCCAGGCGATCGGAAAATAAAGAAATAGTTAATATATTCAAGATAATCACGTATTTACAAAATACCGTCTCAATTCATCCATCCGATCCGGGATGTGATATGGTTCTGAAGGATGAACTGGATATGGACAGTTCCAATAAGATTTCTTTCTTGAACAAAAATCCATTTTTTGATTTATTTCATCTATTCCATGATCGGAACGGGGGGGGGTACACGTTACACCACGATTTTGAATCAGAAGAGAGATTTCAAGAAATGGCAGATCTATTCACTCTATCAATAACCGAGCCGGATCTGGTGTATCATAAGGGATTTACCCGTTTTATTGATTCCTACGGATTGGATCAAAAACAATTCTTGAATGAGGTATTCAACTCCAGGGATGAATCGAAAAAGAAATCTTTATTGGTTCTACCTCCTATTTTTTATGAAGAGAATGAATCTTTTTATCGAAGGATCAGAAAAAAATGGGTCCGGATCTCCTGCGGGAATGATTTGGAAGATCCAAAACAAAAAACAGTGGTATTTGCTAGCAACAACATAATGGAGGCAGTCAATCAATATGGATTGATCCTAAATCTGATTCAAATCCAATATAGTACCTATGGGTACATAAGAAATGTATTGACTCAATTCTTTTTAATGAATAGATCCGATCGCAACTTCGAATATGGAATTCAAAGGGATCAAATAGGAAATGATACTCTGAATCATAGAACTATAATGAAATATACGATCAACCAACATTTATCGAATTTGAAACAGAGTCAGAAGAAATGGTTCGATCCTCTTATTTTTCGTTCTCGAACCGAGAGATCCATGAATTGGGATCCTAATGCATATAGATACAAATGGTCTAATGAGAGCAAGAATTTCCAGGAACATTTGGAACATTTCATTTCTGAGCAGAAGAGCCGTTTTCTTTTTCAAGTAGTGTTCGATCGATTACGTATTAATCAATATTCGATTGATTGGTCTGAGGTTATCGACAAAAAAGATTTGTCTAAGTCACTTCCTTTCTTTTTGTCCAAGTTACTTCTTTTTTTGTCCAAGTTTCTTCTCTTTTTGTCTAACTCACTTCCTTTTTTCTTTGTGAGTTTCGGGAATATCCCCATTCATAGGTCCGAAATCCATATCTATGAATTGAAAGGTCAGAATGATCAACTCTGCAATCAGCTGTTAGAACCAATAGGTCTTCAAATCGTTCATTTGAAAAAATTGAAACCCTTCTTATTGGATGATCATGATACTTCCCAAAAATCGAAATTTTTAATTAATATTAATGGAGGAACAATATCACCATTTTTGTTCAATAAGATACCAAAGTGGATGATTGACTCATTCCATACTAGAACTAATCGCAGGAAATCTTTTGATAACACGGATTCCTATTTCTCAATCATATCCCACGATCAAGACAATTGGCTGAATCCCGTGAAACCATTTCATAGAAGTTCATTGATATCTTCTTTTTATAAAGCAAATCGACTTCGATTCTTGAATAATCTACATCACTTCTGCTTCTATTGTAACAAAAGATTCCCTTTTTATGTGGAAAAGGCCCGTATCAAGAATTCTGATTTTACGTATGGACAATTCCTCAATATCTTGTTCATTCGCAACAAAATATTTTCTTTGTGCGGCGGTAAAAAAAAACATGCTTTTTTGGAGAGAGATACTATTTCACCAATCGAGTCCCAGGTATCTAACATATTCATACCTAATGATTTTCCACAAAGTGGTAACGAAAGGTATAACTTGTACAAATCTTTCCATTTTCCAATTCGATCCGATCCATTCGTTCGTAGAGCTATTTATTCGATCGCAGACATTTCTGGAACACCTCTAACAGAGGGACAAATAGTCAATTTTGAAAGAACTTATTGTCAACCTCTTTCGGATATGAATCTATCTGATTCAGAAGGGAAGAACTTGCATCAGTATCTCAATTTCAATTCAAACATGGGTTTGATTCACACTCCATGTTCTGAGAAATATTTACCATCCGAAAAGAGGAAAAAACGGAGTCTTTGTCTAAAGAAATGTGTTGAAAAAGGGCAGATGTATAGAACCTTTCAACGAGATAGTGCTTTTTCAACTCTCTCAAAATGGAATCTATTCCAAACATATATGCCATGGTTCCTTACTTCGACAGGGTACAAATATCTAAATTTTCTATTTTTCGATACCTTTTCGGACCTATTACCGATACTAAGTAGCAGTCAAAAATTTGTATCCATTTTTCATGATATTATGCATGGATCAGATATATCATGGCGAATTCTTCAGAAAAAATTGTGTCTTCCACAATGGAATCTGATAAGTGAGATTTCGAGTAAATGTTTACATAATCTTCTTCTGTCCGAAGAAATGATTCATCGAAATAATGAGCCACCATTGATATCGACACATCTGAGATCGCCAAATGTTCGGGAGTTCCTCTATTCAATCCTTTTCCTTCTTCTTGTTGCTGGATATCTCGTTCGTACACATCTTCTTTTTGTTTCCCGAGCCTATAGTGAGTTACAGACAGAGTTCGAAAAGGTCAAATCTTTGATGATTCCATCATACATGATTGAGTTGCGAAAACTTCTGGATAGGTATCCTACATCTGAACTGAATTCTTTCTGGTTAAAGAATCTCTTTCTAGTTGCTCTGGAACAATTAGGAGATTTTCTAGAAGAAATGCGGGGTTCTGCTTCTGGCGGCAACATGCTATGGGGTGGTGGTCCCACTTATGGGGTTAAATCAATCCGTTCTAAGAAGAAATTTTTGAATATCAATCTCATCGATCTCATAAGTATCATACCAAATCCCATCAATCGAATCACTTTTTCGAGAAATACGAGACATCTAAGTCATACAAGTAAAGAGATTTATTCATTGATAAGAAAAATAAAAAACGTGAACGGTGATTGGATTGATGATAAAATAGAATCCTTGGTCGCGAACAGTGATTCGATTGATGATAAAGAAAGAGAATTCTTGGTTCAGTTCTCCACCTTAACGACAGAAAAAAGGATTGATCAAATTCTATTGAGTCTGACTCATAGTGATCATTTATCAAAGAATGACTCTGGTTATCAAATGATTGAAGAGCCGGGAGCAATTTATTTACGATACTTAGTTGACATTCATAAAAAGTATCTAATGAATTATGAGTTCAATACACCCTGTTTAGCAGAAAGACGGATATTCCTTGCTTATTATCAGACAACCGCTTATTCACAAACCTCGTGTGGGGTGAATAGTTTTCATTTCCCATCTCATGGAAAACCCTTTTCGCTCCGCTTAGCCCTATCCCCCTCTAGGGGTATTTTAGTGATAGGTTCTATAGGAACTGGACGATCCTATTTGGTCAAATACCTAGCGACAAACTCCTATCTTCCTTTCATTACAGTATTTCTGAACAAGTTCCTGGATAACAAGCCTAACGGTTTTCTTATTGATGATAGTGACGATATTGATGATAGTGACGATATTGATGATAGTGACGATATTGATGTGAGTGACGATATTGATGTGAGTGACGATATCGACCGTGACTTTGATACGGAGCTGGAGTTTCTAACTAGGATGAATGCGCTAACTATGGATATGATGCCGGAAATAGACCGATTTTATATCACCCTTCAATTCGAATTAGCAAAAGCAATGTCTCCTTGCATAATATGGATTCCAAACATTCATGATCTGGATGTGAATGAGTCGAATTACTTATCCCTCGGTCTATTAGTGAACTATCTCTCCAGGGATTGTGAAAGATGTTCCACTAGAAATATTCTTGTTATTGCTTCGACTCATATTCCCCAAAAAGTGGATCCCGCTCTAATAGCTCCAAATAAATTAAATACATGCATTAAGATACGAAGGCTTCTTATTCCACAACAACGAAAGCACTTTTTTACTCTTTCGTATACTAGGGGATTTCACTTGGAAAATAAAATGTTCCATACTAATGGATTCGGGTCCATAACCATGGGTTCCAATGTACGAGATCTTGTAGCACTTACCAATGAGGCCTTATCGATTAGTATTACACAGAAAAAATCAATTATAGACACTAATATAATTAGATCTGCTCTTCATAGACAAACTTGGGATTTGCGATCCCAGGTAAGATCGGTTCAGGATCATGGGATCCTTTTCTATCAGATAGGAAGGGCTGTTGCACAAAATGTATTTCTAAGTAATTGCCCCATAGATCCTATATCTATCTATATGAAGAAGAAATCATGTAACGAAGGGGATTCTTATTTGTACAAATGGTACTTCGAACTTGGAATGAGCATGAAGAAATTAACGATACTTCTTTATCTTTTGAGTTGTTCTGCCGGATCGGCTGCTCAAGACCTTTGGTCTCTACCCGGACCCGATGAAAAAAATGGGATCACTTATTATGGACTTGTTGAGAATGATTCGGATCTAGTTCATGGTCTATTAGAAGTAGAAGGCGCTCTGGTGGGATCCTCACGTACAGAAAAAGATTGCAGTCAGTTTGATAATGATCGAGTGACATTGCTTCTTCGGCCCGAACCAAGGAGTCCCTTAGATATGATGCAAAATGGATCTTATTCTATCCTTGATCAGAGATTTCTCTATGAAAAATACGAATCGGAGTTTGAAGAAGGAGAAGAAGTCCTCGACCCGCAACAGATAGAGGACGATTTATTCAATCACATAGTTTGGGCTCCTAGAATATGGCGCCCTTGGGGTTTTCTATTTGATTGTATCGAAAGGCCCAATGAATTGGGATTTCCCTATTGGGCCAGGTCATTTCGGGGCAAGCGGATCATTTATGATGAAGAGGATGAGCTTCAAGAGAATGATTCGGGGTTCTTGCAGAGTGGAACCATGCAGTACCAGATACGAGATAGATCTTCCAAAGAACAAGGCTTTTTTCGAATAAGCCAATTCATTTGGGACCCTGCGGATCCACTCTTTTTCCTATTCAAAGATCAGCCCTTTGTCTCTGTGTTTTCACATCGAGAATTCTTTGCAGATGAAGAGATGTCAAAGGGGCTTCTTACTTCCCAAACAGATCCTCCTACATCTATATATAAACGCTGGTTTATCAAGAATACGCAAGAAAAGCACTTCGAATTGTTGATTCATCGCCAGAGATGGCTTAGAACCAATAGTTCATTATCTAATGGATTTTTCCGCTCTAATACTCTATCCGAGAGTTATCAGTATTTATCAAATCTGTTCCTATCTAACGGAACGCTATTGGATCAAATGACAAAGGCATTGTTGAGAAAAAGATGGCTTTTCCCGGATGAAATGAAAATTGGATTCATGTAA

>lcl|NC_033910.1_cds_YP_009348415.1_84 [gene=rpl23] [locus_tag=B2L33_pgp003] [db_xref=GeneID:31082771] [protein=ribosomal protein L23] [protein_id=YP_009348415.1] [location=158685..158966] [gbkey=CDS]

ATGGATGGAATCAAATATGCAGTATTTACAGACAAAAGTATTCGGTTATTGGGGAAAAATCAATATACTTTTAATGTCGAATCAGGATCAACTAGGACAGAAATAAAGCATTGGGTCGAACTCTTCTTTGGTGTCAAGGTAATAGCTATGAATAGCCATCGACTCCCGGGAAAGGGTAGAAGAATGAGACCTATTATGGGACATACAATGCATTACAGACGTATGATCATTACGCTTCAACCGGGTTATTCTATTCCACCTCTTAGAAAGAAAAGAACTTAA

>lcl|NC_033910.1_cds_YP_009348416.1_85 [gene=rpl2] [locus_tag=B2L33_pgp002] [db_xref=GeneID:31082851] [protein=ribosomal protein L2] [protein_id=YP_009348416.1] [location=join(158986..159381,160006..160473)] [gbkey=CDS]

ATGGCGATACATTTATACAAAACTTCTACCCCGAGCACACGCAATGGAGCCGTAGACAGTCAAGTGAAATCCAATACACGAAATAATTTGATCTATGGACAGCATCATTGTGGTAAAGGCCGTAATGCCAGAGGAATAATTACCGCAAGGCATAGAGGGGGAGGTCATAAGCGTCTATACCGTAAAATCGATTTTCGACGGAATGAAAAAGACATATATGGTAGAATCGTAACCATAGAATACGACCCTAATCGAAATGCATACATTTGTCTCATACACTATGGGGATGGTGAGAAGAGATATATTTTACATCCCAGAGGGGCTATAATTGGAGATACCATTATTTCTGGTACAGAAGTTCCTATAAAAATGGGAAATGCCCTACCTTTGAGTGCGGTTTTGATTGATCAAAAAGAAGAATCTACTTCAACCGATATGCCCTTAGGCACGGCCATACATAACATAGAAATCACACTTGGAAAGGGTGGACAATTAGCTAGAGCTGCAGGTGCTGTAGCGAAACTGATTGCAAAAGAGGGTAAATCGGCCACATTAAAATTACCTTCTGGGGAGGTTCGTTTAATATCCAAAAACTGCTCAGCAACAGTCGGACAAGTAGGGAATACTGGGGTGAACCAGAAAAGTTTGGGTAGAGCCGGGTCTAAATGTTGGCTAGGTAAGCGTCCTGTAGTAAGAGGAGTGGTTATGAACCCTGTAGACCATCCCCATGGGGGTGGTGAAGGGAGGGCCCCAATTGGTAGAAAAAAACCCGCAACCCCTTGGGGTTATCCTGCACTTGGAAGAAGAAGTAGAAAAAGGAATAAATATAGTGATAATTTGATTCTTCGTCGCCGTAGTAAATAG

**2. *Hevea brasiliensis***

>lcl|NC_015308.1_cds_YP_004327641.1_1 [gene=rps12] [locus_tag=HebrCp092] [db_xref=GeneID:10352012] [protein=ribosomal protein S12] [exception=trans-splicing] [protein_id=YP_004327641.1] [location=complement(join(103189..103214,103751..103982,74171..74284))] [gbkey=CDS]

ATGCCAACTATTAAACAACTTATTAGAAACACAAGACAGCCAATCAGAAATGTCACCAAATCCCCCGCTCTTGGGGGATGTCCTCAGCGCCGAGGAACATGTACTAGGGTGTATACTATCACCCCCAAAAAACCAAACTCTGCCTTACGTAAAGTTGCCAGAGTACGATTAACCTCTGGTTTTGAAATCACTGCTTATATACCTGGTATTGGCCATAATTTACAAGAACATTCTGTAGTCTTAGTAAGAGGGGGAAGGGTTAAGGATTTACCCGGTGTGAGATATCACATTGTTCGAGGAACCCTAGATGCTGTCGGAGTAAAGGATCGTCAACAAGGGCGTTCTAAATATGGGGTCAAAAAGCCAAAATAA

>lcl|NC_015308.1_cds_YP_004327642.1_2 [gene=psbA] [locus_tag=HebrCp002] [db_xref=GeneID:10351963] [protein=photosystem II protein D1] [protein_id=YP_004327642.1] [location=complement(791..1852)] [gbkey=CDS]

ATGACTGCAATTTTAGAGAGACGCGAAAGCGAAAGCCTATGGGGTCGTTTCTGTAACTGGATAACCAGCACTGAAAACCGTCTTTACATTGGATGGTTTGGTGTTTTGATGATCCCTACTTTATTGACCGCAACTTCTGTATTTATTATCGCTTTCATTGCTGCCCCTCCGGTAGATATTGATGGTATTCGTGAACCTGTTTCTGGATCTCTACTTTATGGAAACAATATTATTTCTGGTGCCATTATTCCTACTTCTGCGGCTATAGGTTTGCATTTTTACCCAATATGGGAAGCGGCATCCGTTGATGAATGGTTATACAATGGCGGTCCTTATGAGCTAATTGTTCTACACTTCTTACTTGGTGTAGCTTGTTACATGGGTCGTGAGTGGGAACTTAGTTTCCGTCTGGGTATGCGCCCTTGGATTGCTGTTGCATATTCAGCTCCTGTTGCAGCTGCTACTGCTGTTTTCTTGATTTATCCAATTGGTCAGGGAAGCTTTTCTGATGGTATGCCTCTAGGAATCTCTGGTACTTTCAACTTTATGATTGTATTCCAGGCTGAGCACAACATCCTTATGCACCCATTTCACATGTTAGGCGTAGCTGGTGTATTCGGCGGCTCCCTATTCAGTGCTATGCATGGTTCCTTGGTAACCTCTAGTTTGATCAGGGAAACCACAGAAAATGAATCTGCTAATGAAGGTTACAGATTCGGTCAAGAGGAAGAAACTTATAATATCGTAGCTGCTCATGGTTATTTTGGCCGATTGATCTTCCAATATGCTAGTTTCAACAACTCTCGTTCTTTACATTTCTTCCTAGCTGCTTGGCCTGTAGTAGGTATTTGGTTCACTGCTTTAGGTATTAGCACTATGGCTTTCAACCTAAATGGTTTCAATTTCAACCAATCTGTAGTTGATAGTCAAGGTCGTGTAATTAATACCTGGGCTGATATTATTAACCGTGCTAACCTTGGTATGGAAGTTATGCATGAACGTAATGCTCATAACTTCCCTCTAGACCTAGCTGCTGTCGAAGTTCCATCTACAAATGGATAA

>lcl|NC_015308.1_cds_YP_004327643.1_3 [gene=matK] [locus_tag=HebrCp004] [db_xref=GeneID:10351964] [protein=maturase K] [protein_id=YP_004327643.1] [location=complement(2397..3905)] [gbkey=CDS]

ATGGAGGAAAGATATTTAGAATTAGATAGATCTCGAAAAAACGACTTCCTATACCCATTTATCTTTCGGGAGTATATTTATACATTCGCTCATGATCATAGTTTAAATAGATCTATTTTGTTGGAAAATGTAGGTTATGACAATAAATCTAGTTTTTTAATTGTAAAACGTTTAATTACTCGAATGTATCAACAGAATCATTTGATTATTTCTGCTAATGATTCTAACCAAAATCCATTTTTTAGATACAACAAGAATTTGTATTATCAAATGATATCAGAGGGCTTTGCAGTTATTGTGGAAATTCCATTTTCCCTACGATTAGTATCTTCTTTAGAAAGGTCAGAGATAGTAAAATCTCATAAATTACGATCAATTCATTCAATATTTCCTTTATTAGAGGACAAATTTCCACATTTAAATTATGTGTCAGATATATTAATACCTTACCCCATCCATCTAGAAAAATTGGTTCAAACCCTTCGCTATTGGGTGAAAGATCCCTCTTCTTTGCATTTATTACGACTCTTTCTTCATGAGTATTGGAATTTGAACAGTCTTATTATTCCAAAGAAATCTATTATTATTTTTATAAAAAGGAATCCAAGATTTTTCTTGTTCCTATATAATTCTCATGTATATGAATACGAATCCATCTTCTTTTTTCTCCGTAACCAATCCTTTCATTTACGATCAATATTTTTGCGAGTCCTTCTTGAACGAATTTTTTTCTATGGAAAAATAGAACATTTTGCGGAAGTCTTTGCTAATGATTTTCAGGCCACCCTGTGGTTGTTCAAGGATCCTTTCATGCATTATGTTAGATATCAAGGAAAATCAATTTTGGCTTCAAAAAATAGGCCTTTTCTGATGAAAAAATGGAAATATTACCTTGTCAACTTATGTCAATATCATTTTTATGTCTGGTTTCAACCAGAAAAGATCTATATAAATTCATTATCCAAGCATTCTCTCAACTTTTTGGGCTATCTTTCAAGTGTACAATTAAATCCTTCAGTGGTACGGAGTCAAATGTTAGAATATTCATTTATAATAGATAATACTATAAAGAAACTCGATACAATAGTTCCAATTATTCCTTTAATTGGATCATTGGCAAAAACGAAATTTTGTAACGCAGTAGGACATCCCATTAGTAAACCGATTCGGGCGGATTCGTCGGATTCTGATATTCTCGACCGATTTGTGCGTATATGCAGAAATCTTTCTCATTATTATAGCGGATCCTCAAAAAAAAAGAGTTTGTATCGAATAAAATATATACTTCGACTTTCTTGTGTTAAAACTTTGGCCCGTAAACACAAAAGTACTGTACGCGCTTTTTTGAAAAGATTAGGTTCGGAATTATTAGAAGAGTTTTTTACGGAGGAAGAACAGATTCTTTCTTTGATCTTTCCAAAAGTTTCTTCTAGTTCGCGCAGGTTATATAGAAGACGTGTTTGGTATTTGGATATTATTTCTATCAATGATTTGGCCAATCATGAATAA

>lcl|NC_015308.1_cds_YP_004327644.1_4 [gene=rps16] [locus_tag=HebrCp005] [db_xref=GeneID:10351887] [protein=ribosomal protein S16] [protein_id=YP_004327644.1] [location=complement(join(5391..5617,6525..6564))] [gbkey=CDS]

ATGGTAAAACTTCGTTTGAAACGATGTGGTAGAAAGCAACGAGTCGTTTATCGAATCGTTGCAATTGATGTTCGATCCCGAAGAGAAGGAAGAGATCTTCGGAAAGTGGGGTTTTATGATCCGATAAAAAATCAAACCTATTTAAATGTTCCTGCTATTCTATATTTCCTTGAAAAAGGCGCTCAACCTACAGGAACTGTTCATGATATTTTAAGAAAGGCGGGGGTTTTTACGGAACTTCGCCTTAATCAAACGCAATTTACTTAA

>lcl|NC_015308.1_cds_YP_004327645.1_5 [gene=psbK] [locus_tag=HebrCp007] [db_xref=GeneID:10351965] [protein=photosystem II protein K] [protein_id=YP_004327645.1] [location=8131..8316] [gbkey=CDS]

ATGCTTAATATTTTTAGTTTAATTTTTATCTGTTTTAATTCTGCCATTTTTTCAAGCAATTTTTTCTTTACAAAATTGCCCGAAGCCTACGCCTTTTTGAATCCAATCGTAGATGTTATGCCAGTAATCCCTGTACTCTTTTTTCTATTAGCCTTTGTTTGGCAAGCTGCTGTAAGTTTTCGATGA

>lcl|NC_015308.1_cds_YP_004327646.1_6 [gene=psbI] [locus_tag=HebrCp008] [db_xref=GeneID:10351889] [protein=photosystem II protein I] [protein_id=YP_004327646.1] [location=8742..8852] [gbkey=CDS]

ATGCTTACTCTCAAACTCTTTGTTTACACAGTAGTGATATTCTTTGTTTCTCTCTTCATCTTTGGATTTTTATCTAATGATCCAGGACGTAATCCTGGACGTGAAGAATAA

>lcl|NC_015308.1_cds_YP_004327647.1_7 [gene=psbM] [locus_tag=HebrCp013] [db_xref=GeneID:10351969] [protein=photosystem II protein M] [protein_id=YP_004327647.1] [location=11715..11819] [gbkey=CDS]

ATGGAAGTAAATATTCTTGCATTTATTGCGACTGCACTGTTCATTCTAGTTCCTACTGCTTTTTTACTTATAATATACGTAAAAACAGTTAGTCAAGGTGATTAA

>lcl|NC_015308.1_cds_YP_004327648.1_8 [gene=petN] [locus_tag=HebrCp014] [db_xref=GeneID:10351891] [protein=cytochrome b6/f complex subunit VIII] [protein_id=YP_004327648.1] [location=complement(13181..13270)] [gbkey=CDS]

ATGGATATAGTAAGTCTCGCTTGGGCTGCTTTAATGGTAGTCTTTACATTTTCCCTTTCACTCGTAGTATGGGGAAGAAGTGGACTCTAG

>lcl|NC_015308.1_cds_YP_004327649.1_9 [gene=rpoB] [locus_tag=HebrCp016] [db_xref=GeneID:10351970] [protein=RNA polymerase beta subunit] [protein_id=YP_004327649.1] [location=15119..18331] [gbkey=CDS]

ATGCTCGGGGATGGAAATGGGGGAATGTCTACAATACCTGGATTTAATCAGATACAATTTGAAGGATTTTGTAGGTTCATTGATCAGGGCTTAACAGAAGAACTTTATAAGTTTCCAAAAATTGAAGATACAGATCAAGAAATTGAATTTCAATTATTTGTGGAAACATATCAATTAGTAGAACCCTTGATAAAAGAAAGAGATGCTGTATATGAATCAATTACATATTCTTCTGAATTATATGTATCCGCAGGATTAATTTGGAAAACCAGTAGGGATATGCAAGAACAAACAATTTTTATTGGAAACATTCCTCTAATGAATTCCCTGGGAACTTTTATAATAAATGGAATATACAGAATTGTGATCAATCAAATATTGCAGAGTCCCGGTATCTATTACCGGTCAGAATTGGATCATAATGGAATTTCGGTCTATACTGGCACCATAATATCAGATTGGGGGGGAAGAGTAGAATTAGAGATTGATAGAAAAGCAAGGATATGGGCTCGTGTGAGTAGGAAACAGAAAATATCTATTCTAGTTTTATCATCAGCTATGGGTTTGAATCTAAGAGAAATTTTAGAGAATGTGTGCTACCCTGAAATTTTCTTATCTTTCCTGAATGATAAGGAAAAAAAAAAAATTGGGTCAAAGGAAAATGCCATTTTGGAGTTTTATCAACAATTTACTTGTGTAGGCGGAGATCCAATATTTTCTGAATCCTTATGTAAGGAATTACAAAAGAAATTCTTTCAACAAAGATGTGAATTAGGAAAGATTGGTCGATTAAATATGAACCGGAGACTGAATCTTGATATACCCCATAACAATACATTTTTGTTACCACGAGATATATTGGCAGCTGCAGATCGTTTGATTGAAATGAAATTTGGAATGGGTACACTTGACGATATGAATCATTTAAAAAATAAACGTATTCGTTCTGTAGCGGATCTCTTACAAGATCAATTCGGATTGGCTCTGATTCGTTTAGAAAATGTAGTTAGAGGGACTATATGTGGAGCAATTAGGCATAAATTGATACCGACCCCTCAAAATTTGGTAACTTCAACTCCATTAACAACCACTTATGAATCTTTTTTTGGATTACACCCATTATCTCAAGTTTTGGATCGAACTAATCCATTGACACAAATAGTTCATGGGAGAAAATTGAGTTATTTGGGTCCTGGAGGATTAACAGGACGAACTGCTAGTTTTCGGATACGAGATATCCACCCTAGTCATTATGGGCGCATTTGCCCAATTGACACGTCTGAAGGAATCAATGTTGGACTTATTGGATCTTTAGCAATTCATGCCAAGATTGGTCATTGGGGGTCTTTAGAAAGCCCATTTTATGAAATCTCTGAGGGATCAAAAAAAGTACGGATGTTTTATTTATCGCCAAATAGAGAGGAATACTATATGGTAGCGGCAGGAAATTATTTGGCGCTGAATCGAGGTGTTCAGGAAAAACAGGTTGCTCCGGCTCGATATCGTCAAGAATTCCTGACTATTGCATGGGAACAGGTGCATCTTCGAAGTATTTTTCCCTTCCAATATTTTTCTATTGGAGCTTCCCTCATTCCTTTTATCGAGCATAATGATGCGAATCGGGCTTTAATGAGTTCTAATATGCAACGTCAAGCAGTTCCACTTTCTCGGTCCGAAAAATGCATTGTTGGAACTGGATTGGAACGCCAAGTGGCTCTAGATTCAGGGGTTCCTGCTATAGCCGAACACGAGGGAAAGATAATTTATACTGATATTGACAAGATCATTTTATCGGGCAATGGGGATACTCTACGCATTCCATTAGTTATGTATCAACGTTCCAACAAAAATACTTGTATGCATCAAAAAACCCAGCTTCGGCGGGGTAAATGCATTAAAAAGGGACAAGTTTTAGCAGATGGTGCCGCTACAGTTGGTGGCGAACTTGCCTTGGGCAAAAACGTATTAGTCGCTTATATGCCATGGGAAGGTTACAATTTTGAGGATGCGGTACTCATTAGCGAACGTCTGGTATATGAAGATATTTATACTTCTTTTCACATACGGAAATATGAAATTCAGACTCATGTGACAAGCCAAGGACCTGAAAGGATCACTAACGAAATACCGCATCTAGAGGCCCATTTACTCCGAAATTTAGACAAAAACGGAATTGTGATGCTAGGATCTTGGGTAGAAACGGGCGATATTTTAGTAGGTAAATTAACGCCTCAAATGGCGAAAGAATCATCGTATGCTCCAGAAGATAGATTATTAAGAGCCATACTTGGTATTCAGGTATCTACTTCAAAAGAAACTTGTCTAAAACTACCTATAGGTGGTAGGGGTCGAGTTATTGATGTGAGATGGATTCAGAAAAAAGGGGGTTCCAGTTATAATCCGGAAACGATTCGTGTATATATTTTACAGAAACGTGAAATCAAAGTGGGTGATAAAGTAGCTGGAAGACATGGAAATAAAGGCATCATTTCAAAAATTTTGCCTAGACAAGATATGCCTTATTTGCAAGATGGAAGACCTGTTGATATGGTCTTCAACCCATTAGGAGTACCTTCACGAATGAATGTAGGACAGATATTTGAATGCTCACTCGGGTTAACGGGAGATCTGCTAGATAGACATTATCGAATAGCACCCTTTGATGAGAGATATGAACAAGAGGCTTCGAGAAAACTAGTGTTTTCTGAATTATATGAAGCCAGTAAGCAAACAGCAAATCCGTGGGTATTTGAACCCGAGTATCCGGGAAAAAGTAGAATATTTGATGGAAGAACGGGGGATCCTTTTGAACAGCCTGTTATAATAGGAAAGCCTTATATCTTGAAATTAATTCATCAAGTTGATGATAAAATACATGGACGTTCCAGTGGACATTATGCACTTGTTACACAACAACCCCTTAGAGGAAGGGCCAAGCAAGGTGGACAACGGGTCGGAGAAATGGAGGTTTGGGCTCTAGAGGGATTTGGTGTTTCTCATATTTTACAAGAAATGCTTACTTATAAATCTGATCATATTAGAGCTCGCCAAGAAGTGCTTGGTACTACGATCATTGGAGGAACAATACCTAAACCTGAAGATGCTCCAGAATCTTTTCGATTGCTCGTTCGAGAACTACGATCTTTGGCTCTGGAACTGAATCATTTCCTTGTATCTGAGAAGAACTTCCAGATTAATAGGAAGGAAGCTTAA

>lcl|NC_015308.1_cds_YP_004327650.1_10 [gene=rpoC1] [locus_tag=HebrCp017] [db_xref=GeneID:10352021] [protein=RNA polymerase beta' subunit] [protein_id=YP_004327650.1] [location=join(18358..18789,19575..21185)] [gbkey=CDS]

ATGATTGATCGGTATAAACATCAACAACTCCGAATTGGATCAGTTTCGCCTCAACAAATAAGTGCTTGGGCCAATAAAATCCTACCTAACGGAGAGATTGTTGGAGAGGTGACAAAACCCTATACTTTTCATTACAAAACCAATAAACCTGAAAAAGATGGATTATTTTGTGAAAGAATTTTTGGGCCTATAAAAAGTGGAATTTGTGCTTGTGGAAATTATCGAGTAATCAGGAATGAAAAAGAAGACCAAAAATTTTGTGAACAATGCGGAGTCGAATTTATGGATTCTCGGATACGAAGATATCAAATGGGCTACATCAAACTAGCATGCCCAGTAACTCATGTGTGGTATTTGAAACGTCTTCCTAGTTATATCGCAAATCTTTTAGATAAACCTCTTAAAGAATTAGAAGGCCTAGTATACTGCGATTTTTCTTTTGCTAGGCCCATAGCTAAAAAACCTACTTTTTTACGATTACGAGGTTCATTCGAATATGAAATCCAATCCTGGAAATACAGTATTCCACTTTTTTTTACTACCCAAGGCTTCGATACATTTCGAAATCGAGAAATTTCTACAGGAGCTGGTGCTATCCGAGAACAATTAGCCGATCTGGATTTGCGAATTATTATAGATTATTCATTGGTAGAATGGAAAGAATTAGGGGAAGAAGGGCCTACCGGGAATGAATGGGAAGATCGAAAAGTTGGAAGAAGAAAGGATTTTTTGGTTAGACGTGTGGAATTAGCTAAGCATTTTATTCGAACAAATATCGAACCAGAATGGATGGTTTTATGTCTATTACCAGTTCTTCCTCCCGAGTTGAGACCGATCATTCAGATAGATGGGGGTAAACTAATGAGTTCAGATATTAATGAACTCTATAGAAGAGTTATCTATCGGAACAATACTCTTATTGATCTATTAACAACAAGTAGATCTACCCCAGGGGAATTAGTAATGTGTCAGGAGAAATTGGTACAAGAAGCCGTGGATACACTTCTTGATAATGGAATCCGCGGACAACCAATGAGGGACGGTCATAATAAGGTTTACAAGTCGTTTTCGGATGTAATTGAAGGCAAAGAAGGAAGATTTCGTGAGACTATGCTTGGCAAACGGGTTGATTATTCGGGGCGTTCTGTCATTGTCGTAGGCCCCTCACTTTCATTACATCGATGTGGATTGCCTCGCGAAATAGCAATAGAGCTTTTCCAGATATTTGTAATTCGTGGTCTAATTAGACAACATCTTGCTTCGAACATAGGAGTTGCTAAGAGTAAAATTCGGGAAAAAGAGCCAATTGTATGGGAAATACTTCACGAAGTTATGCAGGGGCATCCAGTATTACTGAATAGAGCGCCGACTCTGCATAGATTAGGCATACAGGCATTCCAACCCATTTTAGTGGAAGGCCGCGCTATTTGTTTACATCCATTAGTTTGTAAGGGATTCAATGCAGACTTTGATGGGGATCAAATGGCTGTTCATGTACCTTTATCGTTGGAGGCTCAAGCAGAGGCTCGTTTACTTATGTTTTCTCATATGAATCTCTTGTCTCCAGCTATTGGGGATCCCATTTCCGTACCAACTCAAGATATGCTTATTGGGCTCTATGTATTAACAAGCGGGAATTGTCGAGGTATTTGTGCAAATAGGTATAATCCATGTAATCGCAGAAATTATCAAAATAAAAGAATTGACGGTAATAACGATAAATATACGAAAGAACCCTTTTTTTCTAATTCCTATGATGCACTTGGCGCTTATCGGCAGAAAAGAATCCATTTAGATAGTCCTTTGTGGCTCCGGTGGCAGCTAGATCAACGCGCTATTACTTCAAGAGAAGCTCCCATCGAAGTTCACTATGAATCTTTGGGTACCTATCATGAGATTTATGAACACTATCTAATAGTAAAAAATATAAAAAAAGAAATTCTTTGTATATACATTCGAACTACTGTTGGTCATATTTCTCTTTATCGAGAAATCGAAGAAGCTATACAAGGGTTTTGCCAAGCCTGCTCAGATAGTATCTAA

>lcl|NC_015308.1_cds_YP_004327651.1_11 [gene=rpoC2] [locus_tag=HebrCp018] [db_xref=GeneID:10351893] [protein=RNA polymerase beta'' subunit] [protein_id=YP_004327651.1] [location=21354..25541] [gbkey=CDS]

ATGGAGGTACTTATGGCCGAACGGGCCAATCTGGTCTTTCACAATAAAGTGATAGATGGAACTGCCATTAAACGACTTATTAGCAGATTAATAGATCATTTTGGAATGGCATATACATCACACATCCTGGATCAAGTAAAGACTCTGGGTTTCCAGCAAGCCACTGCTACATCCATTTCATTAGGAATTGATGATCTTTTAACAATACCTTCTAAGGGATGGCTAGTCCAAGATGCTGAACAACAAAGTTTGATTTTGGAAAAACACTATCATTATGGAAATGTACACGCGATAGAAAAATTACGCCAATCTATTGAGATATGGTATGCTACAAGTGAATATTTGCGACAAGAAATGAACCTGAATTTTAGGATGACAGAACCCTTTAATCCAGTCCATATAATGTCTTTTTCGGGAGCTAGGGGAAATACATCTCAAGTACACCAATTAGTAGGTATGAGAGGATTAATGTCGGATCCACAAGGACAAATGATTGATTTACCCATTCAAAGCAATTTACGCGAGGGACTGTCTTTAACAGAATATATCATTTCTTGCTATGGAGCCCGAAAAGGGGTTGTCGATACTGCTGTACGAACATCAGATGCTGGATATCTTACGCGTAGACTTGTTGAAGTAGTTCAACACATTGTTGTACGTAGAACAGATTGTGGGACCACCCGAGGGATCTCTGTGAGTCCTCGAAATGGGATGATGTCGGAAAGAATTTTGATTCAAACATTAATTGGTCGTGTATTAGCAGACAATATATATATGGGTCTACGATGCATTGCCATTCGAAATCAAGATATTGGGATTGGGCTTGCCAATCGATTCATAACCTTTCGAACACAAACAATATCTATTCGAACTCCCTTTACTTGTAGGAGTACGTCTTGGATCTGTCGATTATGTTATGGTCGGAGTCCTACTCATGGCGATCTAGTAGAATTGGGGGAAGCCGTAGGTATTATTGCGGGTCAATCCATTGGAGAGCCGGGTACTCAACTAACATTAAGAACGTTTCATACCGGCGGAGTATTCACAGGGGGTACTGCAGAACATGTACGAGCCCCCTCTAATGGAAAAATAAAATTTAATGAGGATTTGGTTCATCCCATACGTACACGTCATGGGCATCCTGCTTTTCTATGTTATATAGACTTGTATGTAACTATTGAGAGTCAAGATATTATACATAACGTGACTATTCCACCAAAAAGTTTCCTTTTAGTTCAAAATGATCAATATGTAGAATCAGAACAAGTGATTGCTGAAATTCGGGCGAGAACATACACTTTGAATTTTAAAGAGAAGGTCCGAAAACATATTTATTCCGATTCAGAAGGGGAAATGCACTGGAGTACTGATGTGTACCATGCACCCGAATTTACATATAGTAATGTCCATCTCTTACCAAAAACAAGCCATTTATGGATATTATCAGGAAGTTCGTGCGGATCCAGTATAGTTTCTTTTTCACTACACAAGGATCAAGATCAAATGAATGTTCATTCTCTTTCTGTCAAAAGAAGATATATTTCTAGTCCTTCCGTAAATAATGATCAAGTGAAACACAAATTCTTTAGTTCAGATTTTTCGGGTAAAAAAGAAAGTGAGATTCCTGATTATTCAGAACTTAATCGAATCATATGTACCGGTCATTGTAATCTCATATATCCTACTATTCTCTACGAGAATTCTGATTTATTGGCAAAGAGGCGAAGAAATAAATTCATCATCCCATTCCAATCAATTCAAGAACGAGAGAAAGAACTAATGACCCACTCCGCTATCTCGATTGAAATACCTATAAATGGTATTTTCCGTAGAAATAGTGTTTTTGTTTATTTCGACGATCCCCAATACCGAAGAAAGAGTTCAGGAATTACTAAATATGGGGCTATAGGGGTGCATTCAATCGTCAAAAAAGAAGATTTGATTGAGTATCGGGGAGTCAAAGAATTTAAGCCAAAATACCAAACGAAAGTGGATTGCTTTTTTTTCATTCCCGAGGAAGTGTATATTTTACCCGAATCTTCTTCCCTAATGGTACGGAACAATAGTATCATTGGAGTAGATACACAAATCGCTTTAAATACAAGAAGTCGGGTGGGCGGATTGGTCCGAGTGGAGAGAAAAGAAAAAAAAATGGAACTTAAAATCTTTTCTGGAGATATCCATTTTCCGGGAGAGACAGATAAAATATCCCGACACAGTGGTATCTTAATGCCACCAGGAACGGTAAAAACAAATTCTAAGGAATCAAAAAAAGTGAAAAATTGGATCTATATCCAACGAATCACACCTACCAAGAAAAAGTATTTTGTTTTGGTTCGACCAGTAATCATATATGAGATAGCGAACGGTATAAATTTAGAAACACTTTTCCCCCAGGATCTATTGCAGGAAAAGGATAATCTGAAACTTCGAGTTGTCAATTATATTCTTTATGGGACTGGTAAACCCATTCGGGGAATTTCTGACACAAGTATTCAATTAGTTCGTACTTGTTTAGTGTTGAATTGGGACCAAGACAAAAAAAGTTCTTCTATCGAAGAGGCCCGCGCTTTTTTTGTTGAAATAAGCACAAATGGTCTGATTCGTGATTTCCTAAGAATCAACCTAGTGAAATCCCATATTTCATATATCGGTAGAAAAAGGAATGATCCATCAGGTTCAGAACTGATCTCTAATAATGGGTCAGATCGCACCAATATTAATCCATTTTATCCCATTTATTCCAAGACAAGGATTCAACAATCACTTAAACAAAATCAAGGAACTATTAGTACGTTGTTGAATAGAAATAAGGAATGTAAATCTTTGATAATTTTGTCATCATCTAATTGTTTTCGAATGGATCCATTCAACGATGTAAAACATCACAATGGAATAAAAGAATCAATTAAAAGAGATCCTATAATTCCAATTAGAAATTCGTTGGGCCCTTTAGGAACAGCCCTTCAAATTTCGAATTTTTATTTATTTTACCATTTTAATTTAATATTAATAACTCATAATCAGATCTCGGTAACTAAATATTCGAAACTTGACAATTTAAAACAGACTTTTCAAGTACTTAAATATTATTTAATGGATGAAAACGGGAGAATTGTTAATCCCGATCCATGCAGTAACAGCGTTTTGAATCCATTCAATTTGAATTGGTATTTTCTCCATCATAATTATTGTGAAGAAAGATTCACAATAATTAGCCTGGGACAGTTTATTTGTGAAAATTTATGTATGGCCAAAAAGGGACCACATCTAAAATCGGGTCAAGTTATAATTGTTCACATTGACTCTGTAGTAATAAGATCCGCTAAGCCTTATTTGGCCACTCCAGGAGCAACCGTTCATGGCCATTATGGAGAAATCCTTTACGAAGGAAATACATTAGTTACATTTATATATGAAAAATCGAGATCTGGTGATATAACGCAGGGTCTTCCAAAAGTGGAACAAGTGTTAGAAGTGCGTTCAATTGATTCAATATCAATAAACCTAGAAAAGAGAGTGGAGGGTTGGAACGAGTGTATAACAAGAATTCTGGGAATTCCTTGGGGATTCTTGATTGGTACTGAGCTAACTATAGTGCAAAGTCGTATCTCTTTAGTTAATAAGATCAAAAAGGTTTATCGATCCCAAGGGGTGCAGATCCATAATAGGCATATAGAAATTATTGTACGTCAAATAACATCAAAAGTATTGGTTTCAGAAGACGGAATGTCTAATGTTTTTTCACCCGGAGAACTAATTGGATTGTTGCGAGCGGAACGAACGGGACGCGCTTTGGAAGAAGCCATCTGTTACGGGGCCATATTATTGGGAATAACGCGAGCATCTCTGAATACTCAAAGTTTCATATCCGAGGCTAGTTTTCAAGAAACTACTCGCGTTTTAGCAAAAGCTGCTCTCCGCGGTCGTATCGATTGGTTGAAAGGCCTGAAAGAAAACGTTGTTCTAGGCGGTATGATACCCGTTGGTACCGGATTCAAAGGATTAGTGCAAGGCTCAAGGCAACATAAGAACATTCCTTTGAAAACCAAAAAGAAGCATTTATTCGAGGGGGAATTTAGAGATAGAGATATTTTATTCCACTACAGAGAGTTATTTGATTCTTGCATTTCAAAAAATTTCTATGATACATCAGAACAACCATTTATAGGATTTAATGATTCCTAA

>lcl|NC_015308.1_cds_YP_004327652.1_12 [gene=rps2] [locus_tag=HebrCp019] [db_xref=GeneID:10351894] [protein=ribosomal protein S2] [protein_id=YP_004327652.1] [location=25813..26523] [gbkey=CDS]

ATGATAAGAAGATATTGGAACATTAATTTGGAAGAGATGATGAAAGCAGGAGTTCATTTTGGTCATGGTACTAGAAAATGGAATCCGAGAATGGCACCTTATATCTCTGCAAAGCGTAAAGGTATTCATATTACAAATCTTACTAGAACTGCTCGTTTTTTATCAGAAGCTTGTGATTTAGTTTTTGATGCAGCAAGTAGGAGAAAACAATTCTTAATTGTTGGTACCAAAAATAAAGCAGCGGATTCAGTAGCGCGGGCTGCAATAAGGGCTCGGTGTCATTATGTTAATAAAAAATGGCTCGGCGGTATTTTAACGAATTGGTCCACTACAGAAACTAGACTTCAAAAGTTCAGGGACTTGAGAATGGAACAAAAGACAGGTAGACTCAACCGTCTTCCGAAAGGAGATGCGGCTCGATTGAAGAGACAGTTAACTCACTTGCAAACATATCTGGGCGGGATTAAATATATGACGGGGTTACCCGATATTGTAATAATCGTTGATCAGCAAGAAGAATATACGGCTCTTCGGGAATGTATGACTTTGGGAATTCCAACAATTTGTTTAATTGATACAAACTGTGACCCGGATCTCGCAGATATTTCGATTCCAACGAATGATGACGCTATAGCTTCAATCCGATTAATTCTTAATAAATTAGTATTTGCAATTTGTGAGGGTCATTCTAGCTATATACGAAATCCCTGA

>lcl|NC_015308.1_cds_YP_004327653.1_13 [gene=atpI] [locus_tag=HebrCp020] [db_xref=GeneID:10351895] [protein=ATP synthase CF0 A subunit] [protein_id=YP_004327653.1] [location=26753..27502] [gbkey=CDS]

ATGAATGTTCTATCATGTTCCATCAACACACTAACACTAAAGGGGTTATATGATATATCCGGTGTGGAAGTAGGCCAGCATTTCTATTGGAAAATAGGAGGTTTCCAAGTCCATGCCCAAGTGCTTATTACTTCTTGGGTTGTAATTGCTATCTTATTAGGTTCAGCCATTGTAGCTGTTCGGAATCCACAAACCATTCCAACAGGAGGTCAGAATTTCTTCGAATACGTCCTTGAATTCATTCGAGATGTGAGCAAAACTCAGATTGGAGAGGAATATGGCCCGTGGGTCCCCTTTATTGGAACTATGTTTCTATTTATTTTTGTTTCTAATTGGGCGGGGGCGCTTTTACCTTGGAAGATCATACAGTTACCTCACGGGGAGTTAGCCGCACCGACGAATGATATAAATACTACCGTTGCTTTAGCTTTACTTACGTCAATAGCCTATTTTTATGCGGGCCTTAGCAAAAAAGGATTAGGTTATTTCAGTAAATACATTCAACCAACTCCAATCCTTTTACCCATTAACATTTTAGAAGATTTCACAAAACCTTTATCACTTAGCTTTCGACTTTTCGGCAATATATTAGCGGATGAATTAGTAGTTGTTGTTCTTGTTTCTTTAGTACCTTCAGTGGTTCCTATACCTGTCATGTTCCTTGGATTATTTACAAGTGGTATTCAAGCTCTTATTTTTGCAACTTTAGCTGCGGCTTATATAGGCGAATCCATGGAGGGGCATCATTGA

>lcl|NC_015308.1_cds_YP_004327654.1_14 [gene=atpH] [locus_tag=HebrCp021] [db_xref=GeneID:10351896] [protein=ATP synthase CF0 C subunit] [protein_id=YP_004327654.1] [location=28704..28949] [gbkey=CDS]

ATGAATCCATTGATTTCTGCCGCTTCCGTTATTGCTGCTGGGTTGGCCGTTGGGCTTGCTTCTATTGGACCTGGGGTTGGTCAAGGTACTGCCGCGGGCCAAGCTGTAGAAGGTATCGCAAGACAACCCGAGGCGGAGGGAAAAATACGAGGTACTTTATTGCTTAGTCTGGCTTTTATGGAAGCTTTAACGATTTATGGACTAGTTGTAGCATTAGCACTGTTATTTGCGAATCCTTTTGTTTAA

>lcl|NC_015308.1_cds_YP_004327655.1_15 [gene=atpF] [locus_tag=HebrCp022] [db_xref=GeneID:10351897] [protein=ATP synthase CF0 B subunit] [protein_id=YP_004327655.1] [location=29548..30102] [gbkey=CDS]

ATGAAAAATGTAACCGATTCTTTCGTTTCCTTGGGTCACTGGCCATCCGCCGGGAGTTTCGGGTTTAATACCGATATTTTAGCAACAAATCTAATAAATCTAAGTGTAGTCCTTGGTGTATTGATTTTTTTTGGAAAGGGAGTGTTAAGTGATTTATTAGATAATCGAAAACAAAGGATTTTGGATACTATTCGAAATTCAGAAAAACTACGCGAGGGGGCTATTGAACAGCTGGAAAAAGCCCGGGCCCGCTTACGGAAAGTGGAAATAGAAGCAGATCAGTTTCGAACGAATGGATATTCTGAGATAGAACGAGAAAAATGGAATTTGATTAATTCAACTTATAAGACTTTGGAACAATTAGAAAATTACAAAAATGAAACCATTCATTTTGAACAACAACGAACGATTAATCAAGTCCGACAACGGGTTTTCCAACAAGCCTTACAAGGAGCTCTAGGAACTCTGAATAGTTGTTTGACCAACGAGTTGCATTTACGTACCATCAATGCTAATCTTGGCATGTTTGGGGCGATAAAAGAAATAACTGATTAG

>lcl|NC_015308.1_cds_YP_004327656.1_16 [gene=atpA] [locus_tag=HebrCp023] [db_xref=GeneID:10351898] [protein=ATP synthase CF1 alpha subunit] [protein_id=YP_004327656.1] [location=30166..31689] [gbkey=CDS]

ATGGTAACCATTCGAGCCGACGAGATTAGTAATATTATCCGCGAACGTATTGAGCAATATAATAGGGAAGTAAAGATTGTAAATACCGGTACCGTACTTCAAGTAGGCGACGGCATTGCTCGTATTTATGGTCTTGATGAAGTAATGGCAGGCGAATTAGTAGAATTTGAAGAGGGTACAATAGGCATTGCTCTGAATTTGGAATCAAATAATGTCGGTGTTGTATTAATGGGTGACGGTTTAATGATACAAGAGGGAAGCTCCGTAAAAGCAACAGGAAGAATTGCTCAGATACCTGTGAGTGAGGCTTATTTGGGTCGTGTTATAAATGCCCTAGCTAAACCTATTGACGGTCGAGGTGAAATTTCAGCTTCTGAATCTCGGCTAATTGAATCCCCCGCTCCAGGTATTATTTCGAGACGTTCTGTATATGAGCCTCTTCAAACAGGACTTATTGCTATTGATTCGATGATCCCTATAGGACGTGGTCAACGAGAATTAATTATTGGGGATAGACAGACCGGTAAAACAGCAGTAGCCACAGATACAATTCTCAATCAACAAGGACAAAATGTAATATGTGTTTATGTAGCTATTGGGCAAAAAGCGTCTTCTGTGGCTCAGGTAGTGACTACTTTACAGGAAAGAGGGGCAATGGAGTACACTATTGTGGTAGCCGAAACGGCGGATTCTCCGGCTACATTACAATACCTCGCCCCTTATACAGGAGCGGCTCTGGCTGAATATTTTATGTACCGTGAACGACACACTTTAATCATTTATGATGATCTCTCCAAACAAGCGCAGGCTTATCGCCAAATGTCTCTTCTATTACGAAGACCACCTGGTCGTGAAGCTTATCCAGGAGATGTCTTTTATTTGCATTCACGCCTTTTGGAAAGAGCTGCTAAATTAAGTTCTCGTTTAGGTGAAGGAAGTATGACTGCTTTACCAATAGTCGAGACCCAATCAGGAGACGTTTCGGCTTATATTCCTACTAATGTAATTTCCATTACAGATGGACAAATATTCTTATCCGCCGATCTATTCAATGCTGGAATCAGGCCTGCTATTAATGTGGGTATTTCTGTTTCCAGAGTAGGATCCGCAGCTCAAATAAAAGCTATGAAACAGGTAGCTGGTAAGTTAAAATTGGAATTGGCGCAATTCGCAGAATTAGAAGCCTTTGCGCAATTCGCTTCTGATCTCGATAAAGCTACTCAGAATCAATTGGCAAGAGGTCAACGACTACGCGAGTTGCTCAAACAATCCCAATCAGCTCCTCTCACGGTAGAGGAACAAATAATGACTATTTATACCGGAACGAATGGTTATCTTGATTCATTAGAAATCGGACAAGTAAGGAAATTTCTCGTTGAGTTACGTACCTACTTAAAAACGAATAAACCTCAGTTCCAAGAAATCATATCTTCTACCAAAACATTCACCGAAGAAGCAGAAACCCTTTTGAAAGAAGCTATTCAGGAACAGAAGGAACGTTTTGTAATTCAGGAACAAGTATAA

>lcl|NC_015308.1_cds_YP_004327657.1_17 [gene=psbD] [locus_tag=HebrCp027] [db_xref=GeneID:10351973] [protein=photosystem II protein D2] [protein_id=YP_004327657.1] [location=36144..37205] [gbkey=CDS]

ATGACTATAGCCCTTGGTAAATTTACCAAAGACGAAAATGATTTATTTGATATTATGGATGACTGGTTACGGAGGGACCGTTTCGTTTTTGTAGGTTGGTCCGGTCTATTGCTCTTTCCTTGTGCCTATTTCGCCGTAGGGGGTTGGTTCACAGGTACAACCTTTGTAACCTCATGGTATACCCATGGATTGGCGAGTTCCTATTTGGAAGGCTGCAACTTCTTAACCGCCGCAGTTTCTACTCCTGCTAATAGTTTAGCACATTCTTTGTTATTATTATGGGGTCCTGAAGCACAAGGAGATTTTACTCGTTGGTGTCAATTAGGTGGTTTGTGGACTTTTGTTGCTCTCCACGGTGCTTTTGGACTAATAGGTTTTATGTTACGTCAATTTGAACTTGCTCGATCTGTGCAATTGCGACCTTATAATGCAATCGCATTCTCTGCTCCAATTGCTGTTTTTGTTTCTGTATTCCTGATTTATCCATTAGGCCAGTCTGGTTGGTTTTTTGCGCCTAGTTTTGGTGTAGCAGCTATATTTCGATTCATCCTCTTTTTCCAAGGGTTTCATAACTGGACGCTGAACCCATTTCATATGATGGGAGTTGCCGGCGTATTGGGCGCTGCTCTGCTATGCGCTATTCATGGTGCTACTGTAGAAAATACTTTATTTGAAGATGGTGATGGTGCAAATACATTCCGTGCCTTTAACCCAACTCAAGCTGAAGAAACTTATTCAATGGTCACCGCTAACCGCTTTTGGTCTCAAATCTTTGGGGTTGCTTTTTCCAATAAACGTTGGTTACATTTCTTTATGTTATTTGTACCAGTAACCGGTTTATGGATGAGCGCTCTTGGAGTGGTCGGTCTGGCTCTGAATCTACGTGCCTATGACTTCGTTTCTCAGGAAATCCGTGCAGCGGAAGATCCTGAATTTGAGACTTTCTACACTAAAAATATTCTCTTAAACGAAGGTATTCGTGCTTGGATGGCGGCTCAAGATCAGCCTCATGAAAACCTTATATTCCCTGAGGAGGTTCTACCACGTGGAAACGCTCTTTAA

>lcl|NC_015308.1_cds_YP_004327658.1_18 [gene=psbC] [locus_tag=HebrCp028] [db_xref=GeneID:10351900] [protein=photosystem II 44 kDa protein] [protein_id=YP_004327658.1] [location=37189..38574] [gbkey=CDS]

GTGGAAACGCTCTTTAATGGAACTTTATCTTTAGCCGGTCGTGACCAAGAAACCACCGGTTTCGCTTGGTGGGCCGGGAATGCCCGACTTATCAATTTATCCGGTAAACTACTGGGAGCTCATGTAGCTCATGCTGGATTAATCGTATTCTGGGCTGGAGCAATGAACCTATTTGAAGTGGCTCATTTCGTACCGGAGAAGCCAATGTACGAACAAGGATTAATTTTACTTCCCCACCTAGCTACTCTAGGTTGGGGGGTAGGTCCTGGTGGGGAAGTTATAGACACCTTTCCATACTTTGTATCCGGGGTACTTCACTTAATTTCCTCTGCAGTATTGGGCTTTGGCGGTATTTATCATGCACTTCTGGGTCCTGAGATTCTTGAAGAATCTTTTCCATTTTTTGGTTATGTATGGAAAGATAGAAATAAAATGACAACAATTTTAGGTATTCACTTAATCTTGCTAGGTATAGGTGCTTTTCTTCTAGTATTCAAGGCTCTTTATTTTGGGGGCGTATATGATACCTGGGCTCCGGGGGGGGGAGATGTAAGAAAAATTACCAACTTGACCCTTAGCCCAAGTGTTATTTTTGGTTATTTACTAAAATCTCCCTTTGGAGGAGAAGGATGGATTGTTAGTGTGGACGATTTGGAAGATATAATTGGAGGACATGTATGGTTAGGTTCCATTTGTATACTTGGTGGAATCTGGCATATCTTAACCAAACCTTTTGCATGGGCTCGCCGTGCACTTGTGTGGTCTGGAGAGGCTTACTTGTCTTATAGTTTAGGTGCTTTATCCGTTTTTGGTTTCATTGCTTGTTGCTTTGTCTGGTTCAATAATACCGCGTATCCTAGTGAGTTTTACGGGCCTACTGGACCAGAAGCTTCTCAAGCTCAAGCTTTTACTTTTCTAGTGAGAGATCAACGTCTTGGGGCTAACGTGGGATCCGCTCAAGGACCTACCGGGTTAGGTAAATATTTAATGCGTTCGCCTACCGGAGAAGTTATTTTTGGAGGAGAAACTATGCGTTTTTGGGATTTGCGTGCTCCTTGGTTAGAACCTCTAAGAGGTCCAAATGGTTTGGACTTGAGTAGGTTGAAAAAAGACATACAACCTTGGCAAGAACGCCGTTCCGCGGAATATATGACCCATGCGCCTTTAGGTTCGTTAAATTCTGTAGGTGGCGTAGCTACCGAGATCAATGCAGTCAATTATGTCTCTCCTAGAAGTTGGTTAGCTACCTCTCATTTTGTTCTAGGGTTCTTCCTATTCGTAGGTCATTTATGGCATGCGGGAAGGGCTCGTGCAGCTGCAGCAGGATTTGAAAAAGGAATTGATCGTGATTTTGAACCTGTTCTTTCCATGACTCCTCTTAACTAA

>lcl|NC_015308.1_cds_YP_004327659.1_19 [gene=psbZ] [locus_tag=HebrCp030] [db_xref=GeneID:10351974] [protein=photosystem II protein Z] [protein_id=YP_004327659.1] [location=39252..39440] [gbkey=CDS]

ATGACTATTGCTTTCCAATTGGCTGTTTTTGCATTAATTGCTACTTCATCAATCTTACTGATTAGTGTACCTGTTGTTTTTTCTTCTCCTGATGGTTGGTCGAGTAACAAAAATGTTGTATTTTCCGGTACATCATTATGGATTGGATTAGTCTTTCTGGTAGGTATCCTTAATTCTCTCATCTCTTGA

>lcl|NC_015308.1_cds_YP_004327660.1_20 [gene=rps14] [locus_tag=HebrCp033] [db_xref=GeneID:10351976] [protein=ribosomal protein S14] [protein_id=YP_004327660.1] [location=complement(40421..40723)] [gbkey=CDS]

ATGGCAAGGAAAAGTTTGATTCAGCGGGAGAAGAAGAGGCAAAAATTGGAACAAAAATATCATTTGATGCGTCGATCCTCAAAAAAAGAAATAAGCAAAGTTCCGTCGTTAAGTGATAAATGGGAAATTCATGGAAAGTTACAATCCCCACCGCGGAATAGTGCACCGACACGTCTTCATCGACGTTGTTTTTCGACTGGAAAACCGAGAGCTAACTATCGAGACTTTGGGCTATCCGGACACATACTTCGTGAAATGGTTCATGCATGTTTGTTGCCGGGGGCAACAAGATCAAGTTGGTAA

>lcl|NC_015308.1_cds_YP_004327661.1_21 [gene=psaB] [locus_tag=HebrCp034] [db_xref=GeneID:10351903] [protein=photosystem I P700 chlorophyll a apoprotein A2] [protein_id=YP_004327661.1] [location=complement(40842..43046)] [gbkey=CDS]

ATGGCATTAAGATTTCCAAGGTTTAGCCAAGGCTTAGCTCAGGACCCCACTACTCGTCGTATTTGGTTTGGTATTGCTACCGCGCATGACTTCGAGAGTCATGATGATATTACGGAGGAACGTCTTTATCAGAATATTTTTGCTTCTCACTTCGGGCAATTAGCAATAATTTTTCTGTGGACTTCCGGAAATCTCTTTCATGTAGCTTGGCAAGGAAATTTTGAAGCATGGGTACAGGACCCTTTACATGTAAGACCTATTGCTCATGCAATTTGGGATCCTCATTTTGGTCAACCGGCCGTGGAAGCTTTTACTCGAGGGGGTGCTCCTGGCCCAGTGAATATCGCTTATTCTGGTGTTTATCAATGGTGGTATACAATCGGTTTACGTACTAATGAAGATCTTTATATTGGAGCTCTTTTTCTATTATTTCTTTCTGCCCTAGCCTTACTAGGGGGTTGGTTACACCTACAACCAAAATGGAAACCGAGCGTTTCGTGGTTCAAAAATGCCGAATCTCGTCTCAATCATCATTTGTCAGGACTATTCGGAGTAAGCTCTTTGGCTTGGACAGGACATTTAGTCCATGTCGCTATTCCCGGCTCCCGGGGGGAATACGTTCGATGGAATAATTTCTTAGATGTATTACCACATCCCCAAGGGTTAGGCCCGCTTTTTACAGGTCAGTGGAATCTTTATGCTCAAAATCCCGATTCAGGTAGTCATTTATTTGGTACCTCCCAAGGAGCGGGAACTGCCATTCTAACCCTTCTCGGGGGGTTCCATCCACAAACACAAAGTTTATGGCTGACCGATATTGCACACCATCATTTAGCTATTGCAGTTATTTTTCTCGTTGCCGGTCATATGTATAGAACTAACTTCGGGATTGGGCACAGTATAAAAGATCTTTTAGAAGCACATATTCCTCCGGGGGGGCGATTGGGGCGTGGACATAAGGGTCTTTATGACACAATCAACAATTCGCTTCATTTTCAATTAGGCCTTGCTCTAGCTTCTTTAGGGGTTATTACTTCCTTAGTGGCTCAACACATGTACTCATTACCTGCTTATGCGTTCATAGCGCAAGACTTTACTACTCAAGCTGCGTTATATACTCATCACCAATACATCGCAGGATTCATCATGACAGGAGCTTTTGCTCATGGAGCTATATTTTTTATTAGAGATTACAATCCGGAACAGAATGAGAATAATGTATTGGCAAGAATGTTAGACCATAAAGAAGCTATCATATCCCATTTAAGTTGGGCCAGTCTCTTTCTGGGATTCCATACTTTGGGACTTTATGTTCATAATGATGTCATGCTTGCTTTTGGTACTCCGGAGAAACAAATCTTAATCGAACCCATATTTGCCCAATGGATACAATCTGCTCACGGTAAAACTTCATATGGGTTCGATGTACTTTTATCTTCAACGAATAGTCCGGCCTTCAATGCGGGTCGAAGCATATGGTTGCCCGGCTGGTTAAATGCTATTAATGAAAATAGTAATTCATTATTCTTAACAATAGGGCCTGGAGACTTCTTGGTTCATCATGCTATTGCTCTAGGTTTACATACAACCACATTGATCTTAGTAAAAGGTGCTTTAGATGCACGCGGTTCGAAGTTAATGCCAGATAAAAAGGATTTTGGTTATAGTTTTCCTTGTGATGGTCCGGGACGCGGCGGTACTTGTGATATTTCGGCTTGGGACGCATTTTATTTGGCGGTTTTCTGGATGTTAAATACCATTGGATGGGTTACTTTTTATTGGCATTGGAAGCACATCACATTATGGCAGGGTAATGTTTCACAGTTTAATGAATCTTCCACTTATTTGATGGGATGGTTAAGAGATTATCTATGGTTAAACTCTTCACAACTTATCAATGGATATAACCCTTTTGGTATGAATAGCTTATCGGTCTGGGCGTGGATGTTCTTATTTGGACATCTTGTTTGGGCTACTGGATTTATGTTTTTAATTTCTTGGCGTGGATATTGGCAAGAATTGATTGAAACTTTAGCATGGGCTCATGAGCGTACACCTTTGGCTAATTTGATTCGATGGAGAGATAAACCAGTAGCTCTTTCCATTGTGCAAGCAAGATTGGTTGGATTAGCTCACTTTTCCGTAGGTTATATCTTCACTTATGCGGCTTTCTTGATTGCCTCTACATCAGGTAAATTTGGTTAA

>lcl|NC_015308.1_cds_YP_004327662.1_22 [gene=psaA] [locus_tag=HebrCp035] [db_xref=GeneID:10351904] [protein=photosystem I P700 chlorophyll a apoprotein A1] [protein_id=YP_004327662.1] [location=complement(43072..45324)] [gbkey=CDS]

ATGATTATTCGTTCGCCGGAACCAGAAGTAAAAATTTTGGTAGATAGGGATCCCATCAAAACTTCTTTCGAGGAATGGGCCAGACCCGGTCATTTCTCAAGAACAATAGCTAAAGGACCTGATACTACCACTTGGATCTGGAACCTACATGCTGATGCTCACGATTTCGATAGCCATACCAGTGATTTGGAGGAGATTTCTCGGAAAGTATTTAGTGCTCATTTCGGCCAACTCTCCATCATCTTTCTTTGGCTGAGTGGCATGTATTTCCACGGTGCTCGTTTTTCCAATTATGAAGCATGGCTAAGCGATCCTACTCACATTGGACCTAGCGCTCAAGTGGTTTGGCCAATAGTGGGCCAAGAAATATTGAATGGCGATGTGGGCGGGGGTTTCCGAGGAATACAAATAACTTCCGGTTTTTTTCAGATTTGGAGAGCATCTGGAATAACTAGTGAATTACAACTGTATTGTACCGCAATTGGTGCATTGGTCTTTGCAGCCTTAATGCTTTTTGCTGGTTGGTTCCATTATCACAAAGCTGCTCCAAAATTGGCTTGGTTCCAAGATGTAGAATCTATGTTGAATCACCATTTAGCGGGGCTACTAGGACTTGGGTCTCTTTCTTGGGCGGGACATCAAGTACATGTATCTTTACCAATTAACCAATTTCTAAACGCTGGAGTGGATCCTAAAGAAATCCCACTTCCTCATGAATTTATCTTGAATCGGGATCTTTTGGCTCAACTTTATCCCAGTTTTGCTGAGGGAGCAACCCCATTTTTTACCTTGAATTGGTCAAAATATTCGGACTTTCTTACTTTTCGTGGAGGATTAGATCCAGTGACTGGGGGTCTATGGCTGACCGATACTGCACACCATCATTTAGCTATTGCAATTCTTTTCCTGATAGCGGGTCACATGTATAGGACTAACTGGGCCATTGGTCATGGTATAAAAGAGATTTTAGAGGCTCATAAAGGTCCATTTACAGGTCAGGGCCATAAAGGCCTATATGAGATCCTAACAACGTCATGGCATGCTCAATTATCTCTTAACCTAGCTATGTTAGGTTCTTTAACCATTGTTGTAGCTCACCATATGTATTCCATGCCCCCTTATCCATATCTAGCTACTGATTATGGTACACAACTGTCATTGTTCACACATCACATGTGGATTGGTGGATTTCTCATAGTTGGTGCTGCTGCGCATGCAGCCATTTTTATGGTAAGAGACTATGATCCAACTACTCGATACAACGATCTATTAGATCGTGTCCTTAGGCATCGTGATGCAATCATATCACATCTCAACTGGGTATGTATATTTTTAGGCTTTCACAGTTTTGGTTTATATATTCATAATGATACCATGAGCGCTTTAGGGCGCCCTCAAGATATGTTTTCAGATATTGCTATACAATTACAACCTGTCTTTGCTCAATGGATACAAAACACCCATGCTTTAGCACCTGGTGCAACGGCTCCTGGTGCAACAGCAAGCACCAGTTTAACTTGGGGGGGTGGTGATTTAGTGGCAGTTGGTGGCAAGGTTGCTTTATTACCGATTCCATTAGGAACCGCGGATTTTTTGGTACATCACATTCATGCATTCACGATTCATGTGACGGTATTGATACTTCTGAAAGGAGTTCTATTTGCCCGCAGCTCTCGTTTGATACCGGATAAAGCAAATCTTGGTTTTCGTTTCCCTTGTGATGGACCTGGAAGAGGGGGAACATGTCAAGTATCCGCTTGGGATCATGTCTTCTTAGGGCTATTTTGGATGTACAATTCCATTTCGGTAGTAATATTCCATTTCAGTTGGAAAATGCAGTCAGATGTTTGGGGTAGTATAAGTGATCAAGGGGTGGTAACTCATATCACGGGAGGAAACTTTGCACAGAGTTCCATTACTATTAATGGATGGCTCCGCGATTTCTTATGGGCACAGGCATCCCAGGTAATTCAGTCTTATGGTTCTTCATTATCTGCATATGGCCTTTTTTTCCTAGGTGCTCATTTTGTATGGGCTTTTAGTTTAATGTTTCTATTCAGCGGTCGTGGTTATTGGCAAGAACTTATTGAATCAATCGTTTGGGCTCATAATAAATTAAAAGTTGCTCCTGCTACTCAGCCTAGAGCCTTGAGCATTATACAAGGACGTGCTGTAGGAGTAACCCATTACCTTCTGGGTGGAATTGCCACAACATGGGCGTTCTTCTTAGCAAGAATTATTGCAGTAGGATAA

>lcl|NC_015308.1_cds_YP_004327663.1_23 [gene=ycf3] [locus_tag=HebrCp036] [db_xref=GeneID:10351905] [protein=photosystem I assembly protein ycf3] [protein_id=YP_004327663.1] [location=complement(join(46079..46231,46900..47127,47854..47979))] [gbkey=CDS]

ATGCCTAGATCTCGGATAACTGGAAATTTTATTGATAAGACTTTTTCAGTTGTAGCCAATATCTTATTACGAATAATTCCGACAACTTCGGGAGAAAAAGAGGCATTTACTTATTACAGAGATGGTATGTCTGCTCAATCCGAAGGAAATTATGCAGAAGCTTTACAGAATTATTATGAAGCTATGCGGCTAGAAATTGATCCCTATGATCGAAGTTATATACTCTATAATATAGGCCTTATTCACACAAGTAATGGAGAACACACAAAAGCTTTGGAATATTATTTTCGGTCACTAGAACGAAACCCCTTCTTACCACAAGCTTTAAATAATATGGCCGTGATCTGTCATTACCGAGGAGAACAGGCCATTCGGCAGGGAGATTCTGAAATTGCGGAGGCTTGGTTCGATCAAGCCGCGGAGTATTGGAAACAAGCTATAGCGCTTACTCCCGGAAATTATATTGAAGCGCAGAATTGGTTGAAAATCACAAGGCGTTTCGAATAA

>lcl|NC_015308.1_cds_YP_004327664.1_24 [gene=rps4] [locus_tag=HebrCp038] [db_xref=GeneID:10351977] [protein=ribosomal protein S4] [protein_id=YP_004327664.1] [location=complement(48698..49303)] [gbkey=CDS]

ATGTCACGTTACCGAGGGCCTCGTTTCAAAAAAATACGTCGTCTGGGGGCTTTGCCGGGACTAACTAGTAAAAGGCCTAAAGTCGGGAGCGATCTTAGAAATCAATCACGCTCCGGTAAAAAATCTCAATATCGTATTCGTTTAGAAGAAAAACAAAAATTGCGTTTTCATTATGGTCTTACAGAACGACAATTACTTAAATATGTTCGTATCGCCGCAAAAGCCAAAGGGTCAACAGGTCAGGTTTTACTACAATTACTTGAAATGCGTTTGGATAACATCCTTTTTCGATTGGGTATGGCGTCAACTATTCCTCGAGCCCGCCAATTAGTTAATCATAGACATATTTTAGTTAATGGTCGTATAGTAGATATACCAAGTTATCGCTGCAAACCCCGAGATATTATTACAGCGAGAGCTGAACAAAAATCTAGAGCTATGATTAAAAATTATCTTGATTCATCCCCCCAGGAGGAATTGCCAAAACATTTGACTCTTCACCCATTCCAATATAAAGGATTGGTCAATCAAATAATAGATAGTAAATGGGTTGGCTTGAAAATAAATGAATTACTAGTGGTAGAATATTATTCTCGTCAGACTTAA

>lcl|NC_015308.1_cds_YP_004327665.1_25 [gene=ndhJ] [locus_tag=HebrCp042] [db_xref=GeneID:10351980] [protein=NADH dehydrogenase subunit J] [protein_id=YP_004327665.1] [location=complement(52069..52545)] [gbkey=CDS]

ATGCAGGGTCGTTTGTCTGCTTGGCTAGTCAAACATGGGCTAGTTCATAGATTTTTGGGTTTTGATTACCAAGGAATAGAGACTTTACAAATAAAGCCCGAAGATTGGCATTCCATTGCTGTCATTTTATATGTATATGGTTACAATTATCTGCGTTCGCAATGTGCCTATGATGTAGCACCGGGCGGGCTGTTAGCTAGTGTATATCATCTTACGAGAATAGAGTATGATATAGATCAACCAGAAGAAGTATGTATAAAAGTATTTGCCCCAAGGAAGAATCCTAGAATTCCGTCTGTTTTCTGGGTTTGGAAAAGTGCGGATTTTCAAGAAAGGGAATCTTATGATATGCTGGGAATCTTTTATGATAATCATCCACGCTTGAAACGTATCTTAATGCCGGAAAGTTGGATAGGGTGGCCCTTACGTAAGGATTATATTGTTCCCAATTTTTATGAAATACAAGATGCTCATTGA

>lcl|NC_015308.1_cds_YP_004327666.1_26 [gene=ndhK] [locus_tag=HebrCp043] [db_xref=GeneID:10351908] [protein=NADH dehydrogenase subunit K] [protein_id=YP_004327666.1] [location=complement(52664..53341)] [gbkey=CDS]

ATGAATTCCATTGAGTTTCCTTTACTTGATCGAACAACTCAAATTTCAGTTATTTCAACTACATCAAATGATCTTTCAAATTGGTCAAGACTCTCCAGTTTATGGCCACTTCTCTATGGTACCAGTTGTTGCTTCATTGAATTTGCTTCATTAATAGGCTCACGATTCGACTTTGATCGTTATGGGCTAGTACCAAGATCTAGTCCTAGACAAGCGGACCTGATTTTAACAGCCGGCACCGTAACCATGAAAATGGCTCCTTCTTTAGTGAGATTATATGAACAAATGCCTGAACCAAAATATGTTATTGCTATGGGGGCATGTACAATTACAGGGGGGATGTTCAGTACCGATTCTTATAGTACTGTTCGGGGAGTTGATAAGCTAATTCCTGTAGATGTCTATTTGCCAGGCTGTCCACCTAAACCGGAGGCGGTTATAGATGCTATAACAAAACTTCGTAAAAAAATATCTCGAGAAATTTATGAAGATCGAATTAGGTCTCAACCGGGGAAACGGTGTTTTACTACTAATCACAAGTTTAATATTGAACGCACTACTCATACTGGAAATTATGATCAAGAATTACTCTATCAATCGCCGTCTACTTCAAAGATCCCTCCGGAAACATTTTTCAAATATAAAAGGTCAGTATCGTCCAACGAATTAGTAAATTAG

>lcl|NC_015308.1_cds_YP_004327667.1_27 [gene=ndhC] [locus_tag=HebrCp044] [db_xref=GeneID:10351909] [protein=NADH dehydrogenase subunit 3] [protein_id=YP_004327667.1] [location=complement(53393..53755)] [gbkey=CDS]

ATGTTTCTGATTTACGAATATGATATATTCTGGGCATTTCTAATAATATCAAGTGTTATTCCTATTTTAGCATTTCTAATTTCCGGAGTTTTATCCCCGATTAGCAAAGGGCCGGAGAAACTTTCTAGTTATGAATCGGGTATAGAACCAATGGGTGATGCTTGGTTACAATTTCGAATCCGTTATTATATGTTTGCTCTAGTTTTTGTTGTTTTTGATGTTGAAACAGTTTTTCTTTATCCATGGGCAATGAGTTTCGATATATTGGGGTTATCCGTATTTATAGAAGCTTTCATTTTCGTGCTTATCCTAATTGTTGGTTCAGTTTATGCATGGAGAAAAGGAGCATTAGAATGGTCTTAG

>lcl|NC_015308.1_cds_YP_004327668.1_28 [gene=atpE] [locus_tag=HebrCp047] [db_xref=GeneID:10351982] [protein=ATP synthase CF1 epsilon subunit] [protein_id=YP_004327668.1] [location=complement(55642..56043)] [gbkey=CDS]

ATGACCTTAAATCTTTGTGTACTGACCCCGAATCGAATTGTTTGGGATTCAGAAGTGAAAGAAATCATTTTATCTACTAATAGTGGACAAATTGGCGTATTACCAAACCATGCACCTATTGCCACAGCTGTCGATATCGGTATTTTGAGAATACGCCTTAATGACCAATGGTTAACGATGGCTCTGATGGGTGGTTTTGCTAGAATAGGCAATAATGAGATTACTGTTTTAGTAAATGATGCAGAGAAGGGTAGTGACATTGATCCACAAGAAGCTCAGCAAACTCTTGAAATAGCAGAAGCTAACTTGAGGAAAGCGGAAGGCAGGAGACAAATAATTGAGGCAAATCTAGCTCTCAGACGAGCTAGGGCACGAGTAGAGGCTATCAATGGGATTTCGTAA

>lcl|NC_015308.1_cds_YP_004327669.1_29 [gene=atpB] [locus_tag=HebrCp048] [db_xref=GeneID:10351911] [protein=ATP synthase CF1 beta subunit] [protein_id=YP_004327669.1] [location=complement(56040..57536)] [gbkey=CDS]

ATGAGAATAAATCCTACTACTTCTGGTCCGGGAGTTTCCGCGCTTGAAAAAAAGAACCTGGGGCGTATCGCTCAAATTATTGGGCCAGTGCTAGATGTAGCTTTTCCCCCGGGCAAGATGCCTAATATTTACAACGCTCTGGTAGTTAAGGGTCGAGATACTGCCGGTCAAGAAATTAATGTGACTTGTGAAGTACAACAATTATTAGGAAATAATCGAGTTCGCGCTGTAGCTATGAGTGCTACAGATGGTCTAACGAGAGGAATGGAAGTGATTGACACAGGAGCCCCTCTAAGTGTTCCAGTCGGTGGGGCAACTCTAGGACGAATTTTCAACGTGCTTGGAGAACCTGTTGACGATTTAGGTCCTGTAGATACTCGCGCAACATCCCCTATTCATAGATCTGCGCCTGCCTTTATACAGTTAGATACAAAATTATCTATTTTTGAAACAGGAATAAAAGTAGTAGATCTTTTAGCCCCTTATCGCCGTGGAGGAAAAATCGGACTATTCGGGGGAGCTGGAGTGGGTAAAACAGTACTTATTATGGAATTAATCAATAACATTGCGAAAGCTCATGGGGGTGTATCCGTATTTGGCGGAGTAGGCGAACGTACTCGTGAAGGAAATGATCTTTACATGGAAATGAAAGAATCTGGAGTAATTAATGAAGAAAATATTGCAGAATCAAAAGTGGCTCTAGTCTATGGTCAGATGAACGAACCGCCGGGAGCTCGTATGAGAGTTGGTTTGACTGCCCTAACTATGGCGGAATATTTTCGAGATGTTAATGAACAAGACGTACTTCTATTTATCGACAATATCTTCCGTTTCGTCCAAGCCGGATCCGAAGTATCCGCCTTATTGGGTAGAATGCCTTCCGCTGTGGGTTATCAACCTACCCTTAGTACCGAAATGGGCTCTTTACAAGAAAGAATTACTTCTACCAAAGAAGGGTCCATAACTTCTATTCAAGCAGTTTATGTACCTGCGGACGATTTGACTGACCCTGCTCCTGCCACGACATTTGCACATTTAGATGCTACTACTGTACTATCAAGAGGATTAGCTGCTAAAGGTATCTATCCAGCAGTAGATCCTTTAGATTCAACGTCAACTATGCTCCAACCTCAGATCGTTGGTGAGGAACATTATGAAACTGCGCAAAGAGTTAAGCAAACTTTACAACGTTACAAAGAACTTCAGGACATTATAGCTATCCTTGGGTTGGACGAATTATCCGAAGAGGATCGCTTAACTGTAGCAAGAGCACGAAAAATTGAACGTTTCTTATCACAACCCTTTTTCGTAGCAGAAGTATTTACCGGTTCTCGGGGGAAATATGTCGGTCTAGCAGAAACAATTAGAGGGTTTAAATTGATCCTTTCGGGAGAATTAGATAGTCTCCCTGAGCAGGCCTTTTATTTGGTAGGTAATATTGATGAAGCTACTGCGAAGGCTACGAACTTAGAAATGGAGAACAACTTGAAGAAATGA

>lcl|NC_015308.1_cds_YP_004327670.1_30 [gene=rbcL] [locus_tag=HebrCp049] [db_xref=GeneID:10351912] [protein=ribulose-1,5-bisphosphate carboxylase/oxygenase large subunit] [protein_id=YP_004327670.1] [location=58292..59719] [gbkey=CDS]

ATGTCACCACAAACAGAGACTAAAGCAAGTGTTGGATTCAAGGCTGGTGTTAAAGATTATAAATTGACTTATTATACTCCTGAGTATCAAACCAAAGATACTGATATCTTGGCAGCATTCCGAGTAACTCCTCAACCTGGAGTTCCGCCTGAGGAAGCAGGAGCTGCGGTAGCTGCTGAATCTTCTACTGGTACATGGACAACTGTGTGGACCGATGGACTTACCAGTCTTGATCGTTATAAAGGACGATGCTACGGCATCGAGCCTGTTCCTGGGGAAGAAAATCAATATATTGCTTATGTAGCTTACCCATTAGACCTTTTTGAAGAAGGTTCTGTTACTAACATGTTTACTTCCATTGTGGGTAATGTATTTGGGTTCAAAGCCCTACGCGCCCTACGTCTGGAGGATTTGCGAATCCCTCCTGCTTATTCTAAAACTTTCCAAGGGCCGCCTCATGGCATCCAAGTTGAGAGAGATAAATTGAACAAGTATGGTCGCCCCCTATTGGGTTGTACTATTAAACCAAAATTGGGTCTATCCGCTAAGAATTACGGTAGAGCAGTTTATGAATGTCTTCGCGGTGGACTTGATTTTACCAAAGACGATGAGAATGTGAACTCCCAACCATTTATGCGTTGGAGAGACCGTTTCTTATTTTGTGCCGAAGCAATTTATAAAGCACAGGCTGAAACAGGTGAAATCAAAGGACATTATTTGAATGCTACTGCAGGTACATGCGAAGAAATGATCAAAAGGGCTGTATTTGCCAGAGAATTAGGAGTTCCTATCGTAATGCATGACTACTTAACAGGGGGATTCACTGCAAATACTAGCTTGGCTCATTATTGCCGAGATAATGGTTTACTTCTTCACATTCACCGCGCAATGCATGCAGTTATTGATAGACAGAAGAATCATGGTATGCATTTTCGTGTACTAGCTAAGGCCTTACGTCTATCTGGTGGAGATCATATTCACGCCGGTACCGTAGTAGGTAAACTTGAAGGGGAAAGAGACATTACTTTGGGCTTTGTTGATTTACTGCGTGATGATTTTATTGAAAAAGATCGAAGCCGTGGTATTTATTTCACTCAAGATTGGGTCTCTCTACCAGGTGTTATACCTGTAGCTTCAGGGGGTATTCACGTTTGGCATATGCCTGCTCTGACCGAGATCTTTGGAGATGATTCCGTACTACAATTCGGTGGAGGAACTTTAGGGCACCCTTGGGGAAATGCACCCGGTGCCGTAGCTAATCGAGTAGCTCTAGAAGCATGTGTACAAGCTCGTAATGAGGGACGTGATCTTGCTCGTGAGGGTAATGATATTATCCGTGAGGCTAGCAAATGGAGTCCTGAACTAGCTGCTGCTTGTGAAGTATGGAAGGAAATTAAATTTGAATTTGAAGCAGTGGATACTTTGTAA

>lcl|NC_015308.1_cds_YP_004327671.1_31 [gene=accD] [locus_tag=HebrCp050] [db_xref=GeneID:10351913] [protein=acetyl-CoA carboxylase beta subunit] [protein_id=YP_004327671.1] [location=60462..61970] [gbkey=CDS]

ATGGAAAAATGGCGGTTCAATTCGATCTTATCCAATGTGGAATTAGGATACAGGTGTAGGCTAAGTAAATCAATGGATAGTTTCAGTTCTCTTGAAAATACCAGTATAAGTGAAGACCCAATTCTAAATGATACAGATAAAAACACCTATAGTTGGAGTAATAGTGACAGCTCTAGTTACAGTAATGTTGATCATTTAGTCGGTGTCAGGGACATTCAGAATTTCAGCGCCGATGAAACTTTTTTAGTTAGGGATAGTAATAGGGACAGTTATTCCATATATTTTGATATTGAAAATAAAGTTTTTGAGATTGACAATGATCATTCTTTTCTGAGTGAACTAAAAAGTTCTTTTTATAGTTATTGGAATTCTAGTTATCTGAATAATGGGTTTAGGAGTGGCGACTCCCACTATGATCATTATATGTATGATACTAAATATAGTTGGAATAATTACATCAATAGTTGCATTGACAGTTATCTTCGCTCTCAAATCTGTATTGATAGTTATATTTTAAGTGGTAGTAACAATTACAGCGAAAGTTACATTTATAGTTACATTTGTGGTGAAAGTGGAAATAGTAGTGAAAGCGATAGTTCCAGTCTAAGAACTAGCACGAGTGGTAGCGATTTAATTATAAGAGAAAATTCTAATGATCTCGATATAACTCAAAAATACAAGCATTTGTGGGTTCAATGCGAAAATTGTTATGGATTAAATTATAAGAAATTTTTTAAGTCAAGAATGAATATTTGTGAACAATGTGGATATCATTTGAAAATGAGTAGTTCAGATAGAATTGAACTTTTGATTGACCTAGGCACTTGGGATCCTATGGATGAAGACATGGTATCTCTGGATCCCATTGAATTTCATTCAGAAGAGGAACCTTATAAAGATCGTATTGATTCTTATCAAAGAAAGACAGGATTAACTGAGGCTGTTCAAACAGGCACAGGTCAACTAAACGGCATTCCCGTAGCAATTGGGGTTATGGATTTTCAGTTTATGGGGGGTAGTATGGGATCCGTAGTAGGTGAGAAAATCACTCGTTTGATCGAGTATGCTACCAATAAATTTTTACCTCTTATTTTAGTGTGTGCTTCCGGAGGAGCACGCATGCAAGAAGGAAGTTTGAGCTTGATGCAAATGGCTAAAATATCTTCTGCATTATATGATTATCAATCAAATAAAAAGTTATTTTATGTATCAATCCTTACATCTCCTACGACTGGTGGGGTGACAGCTAGTTTTGGTATGTTGGGGGATATCATTATTGCTGAACCTAATGCCTATATTGCGTTTGCAGGTAAAAGAGTAATTGAACAAACATTGAATAAGACAGTACCTGAAGGTTCGCAATCGGCCGAATTTTTATTCCATAAGGGCTTATTTGATCTAATCGTACCGCGTAATCTTTTAAAAGGCGTTCTGAATGAGTTACTTCAGCTCCACGATTTCTTTCCTTTGAATCATAAATCAAGTAGAAACCTTAAGTTAAAAAGTTAA

>lcl|NC_015308.1_cds_YP_004327672.1_32 [gene=psaI] [locus_tag=HebrCp051] [db_xref=GeneID:10351914] [protein=photosystem I subunit VIII] [protein_id=YP_004327672.1] [location=62719..62832] [gbkey=CDS]

ATGACAATTCTCAACAACTCCCCCTCCATTCTTGTGCCTTTAGTGGGCTTAGTATTTCCGGCAATTGCAATGGCTTCTTTATTTCTTTATATTCAAAAAAACAAGATTTTTTAG

>lcl|NC_015308.1_cds_YP_004327673.1_33 [gene=ycf4] [locus_tag=HebrCp052] [db_xref=GeneID:10351915] [protein=photosystem I assembly protein Ycf4] [protein_id=YP_004327673.1] [location=63287..63841] [gbkey=CDS]

ATGAGTTGGCGATCAGAACGTATATGGATAGAACTTATAGCGGGGTCTCGAAAAACAAGTAATTTCTGCTGGGCCTTTATACTTTTTTTAGGTTCATTGGGATTTTTATTGGTTGGAATTTCCAGCTATCTTGGCAGAAATTTGATATCTTTATTTCCGTCTCAGCAAATAATTTTTTTCCCACAAGGGATCGTGATGTCTTTCTATGGGATCGCCGGTCTATTTATTAGTTCTTATTTGTGGTGCACAATTTTGTGGAATGTAGGTAGTGGTTATGATCGATTCGATAGAAAAGAAGGAATAGTGTGTATTTTTCGCTGGGGATTTCCGGGAAAAAATCGTCGCATCTTACTACGATTCCTTATGAAAGATATTCAGTCTATTAGAATAGAAGTTAAAGAGGGTATTTATGCTCGGCGTGTCCTTTATATGGAAATCAGAGGCCGGGGGGCTATTCCTTTGACTCGTACTGATGAGAATTTGACTCCACGAGAAATTGAGCAAAAAGTAGCGGAATTGGCCTATTTTTTGCGTGTACCAATTGAAGTATTTTGA

>lcl|NC_015308.1_cds_YP_004327674.1_34 [gene=cemA] [locus_tag=HebrCp053] [db_xref=GeneID:10351916] [protein=envelope membrane protein] [exception=RNA editing] [protein_id=YP_004327674.1] [location=64757..65452] [gbkey=CDS]

ACGAAAAAATGGAAAAAAAAAACATTTATTCCCCTTCTATATCTTACATCTATAGTTTTTTTGCCCTGGTGGATCTCTTTTTTATTTAATAAAAGTTTTGAATCTTGGGTTATTAATTGGTGTAATATTAGTAAATCCGAAACTTTTTTAAATGATATCCAAGAAAAAAGTATTCTAGAAAAATTCATAGAATTAGAGGAACTCGTTCGCTTAGACGAAATGATAAAGGAATACCCGGAAACACATCTACAAAAGTTTCGTATCGGAATCCACAAAGAAACGATCCAATTGATCAAGATGCACAATGAGGATCGTATCCATACGATTTTTCACTTCTCGACAAATATAATCTCTTTCGTTATTCTAAGGGGTTATTCTATTCTAAGTAATGAAAAACTTATTATTCTTAATTCTTGGGTTCAAGAATTCCTATATAATTTAAGCGACACAATAAAAGCTTTTTCCATTCTTTTATTAACCGATTTATGTATAGGATTCCATTCACCTCACGGTTGGGAACTAATGATCGGCTCCGTCTACAAAGATTTTGGATTTGCTCATAACGATCAAATTATATCTGGCCTTGTTTCCACTTTTCCAGTCATTCTCGATACCATTTTTAAATATTGGATTTTCCGTTATTTAAATCGTGTATCTCCGTCACTTGTAGTGATTTATCATTCAATGAATGACTGA

>lcl|NC_015308.1_cds_YP_004327675.1_35 [gene=petA] [locus_tag=HebrCp054] [db_xref=GeneID:10351917] [protein=cytochrome f] [protein_id=YP_004327675.1] [location=65684..66646] [gbkey=CDS]

ATGCAAACCAGAAAGACCTTTTCTTGGATAAAGGAAGAGATTACTCGTTCCATTTCCGTATCGCTCATGATATATATAATAACTTGGGCATCCATTTCAAATGCATATCCCATTTTTGCACAGCAGGGTTATGAAAATCCACGGGAAGCAACTGGTCGTATTGTATGTGCCAATTGTCATTTAGCTAATAAACCCGTGGATATTGAGGTTCCACAAGCGGTACTTCCAGATACTGTATTTGAAGCAGTTGTTCGAATTCCTTATAATATGCAACTGAAACAAGTTCTTGCTAATGGTAAAAAGGGGGCTTTGAATGTGGGGGCTGTTCTTATTTTACCTGAGGGGTTTGAATTAGCCCCTCCCGATCGTATTTCGCCAGAGATGAAAGAAAAGATGGGAAATCTGTCTTTTCAGAGTTATCGCCCCACTAAAAAAAATATTCTTGTGATAGGTCCTGTTCCTGGTCAGAAATATAGTGAAATTACCTTTCCTATTCTTTCTCCGGACCCCGCCGCTAAGAAAGATGTTCACTTTTTAAAATATCCCATATATGTAGGCGGAAACAGGGGAAGGGGTCAGATTTATCCCGACGGGAGCAAGAGCAACAATACGGTTTATAATGCTATAGCAGCAGGTATAGTAAGCAAAATCATACGAAAAGAAAAAGGGGGGTACGAAATAACCATAACGAATGCGTCAGAGGGACGTCAAGTGATTGATATTATACCTCCAGGACCAGAACTTCTTGTTTCAGAAGGCGAATCCATCAAACTTGATCAACCATTAACGAGTAATCCTAATGTGGGTGGATTTGGTCAGGGGGATGCAGAAATAGTACTTCAAGACCCATTACGTGTCCAAGGCCTTTTGTTCTTCTTGGCATCCGTTATTTTGGCACAAATCTTTTTGGTTCTTAAAAAGAAACAGTTTGAGAAGGTTCAATTGTCCGAAATGAATTTCTAG

>lcl|NC_015308.1_cds_YP_004327676.1_36 [gene=psbJ] [locus_tag=HebrCp055] [db_xref=GeneID:10351918] [protein=photosystem II protein J] [protein_id=YP_004327676.1] [location=complement(67767..67889)] [gbkey=CDS]

ATGGCCGATACTACTGGAAGAATTCCTCTTTGGATAGTAGGTACTGTAACTGGTATTCTTGTGATCGGTTTAATAGGCATTTTCTTTTATGGTTCATATTCCGGATTGGGTTCATCCCTGTAA

>lcl|NC_015308.1_cds_YP_004327677.1_37 [gene=psbL] [locus_tag=HebrCp056] [db_xref=GeneID:10351919] [protein=photosystem II protein L] [protein_id=YP_004327677.1] [location=complement(68036..68152)] [gbkey=CDS]

ATGACACAATCAAATCCGAACGAACAAAATGTTGAATTGAATCGTACCAGTCTCTACTGGGGGTTATTACTCATTTTTGTACTTGCTGTTTTATTTTCTAATTATTTCTTCAATTAA

>lcl|NC_015308.1_cds_YP_004327678.1_38 [gene=psbF] [locus_tag=HebrCp057] [db_xref=GeneID:10351920] [protein=photosystem II protein VI] [protein_id=YP_004327678.1] [location=complement(68179..68298)] [gbkey=CDS]

ATGACCATAGATCGAACCTATCCAATTTTTACAGTACGATGGTTGGCTGTTCACGGACTAGCTGTACCTACCGTTTCTTTTTTGGGGTCAATATCAGCAATGCAGTTCATCCAACGATAA

>lcl|NC_015308.1_cds_YP_004327679.1_39 [gene=psbE] [locus_tag=HebrCp058] [db_xref=GeneID:10351921] [protein=photosystem II protein V] [protein_id=YP_004327679.1] [location=complement(68307..68558)] [gbkey=CDS]

ATGTCTGGAAGCACAGGAGAACGTTCTTTTGCTGATATTATTACCAGTATTCGATATTGGGTCATTCATAGCATTACTATACCTTCCCTATTCATTGCAGGTTGGTTATTCGTCAGCACGGGTTTAGCTTACGATGTATTTGGAAGCCCTCGTCCAAATGAATATTTTACCGAAAGCCGACAAGGAATTCCATTAATAACTGGCCGTTTTGATCCTTTGGAACAACTCGATGAATTTAGTAAATCTTTTTAG

>lcl|NC_015308.1_cds_YP_004327680.1_40 [gene=petL] [locus_tag=HebrCp059] [db_xref=GeneID:10351922] [protein=cytochrome b6/f complex subunit VI] [protein_id=YP_004327680.1] [location=69922..70017] [gbkey=CDS]

ATGCCTACTATAACTAGTTATTTCGGTTTTCTACTAGCGGCTTTAACTATAACCTCAGTTCTATTTATTGGTCTGAGCAAGATACGACTTATTTGA

>lcl|NC_015308.1_cds_YP_004327681.1_41 [gene=petG] [locus_tag=HebrCp060] [db_xref=GeneID:10351923] [protein=cytochrome b6/f complex subunit V] [protein_id=YP_004327681.1] [location=70197..70310] [gbkey=CDS]

ATGATTGAAGTTTTTCTATTTGGAATCGTCTTAGGTCTAATTCCTATTACTTTGGCCGGATTATTTGTAACTGCATATTTACAATACAGACGTGGTGATCAGTTGGACCTTTGA

>lcl|NC_015308.1_cds_YP_004327682.1_42 [gene=psaJ] [locus_tag=HebrCp063] [db_xref=GeneID:10351984] [protein=photosystem I subunit IX] [protein_id=YP_004327682.1] [location=71203..71337] [gbkey=CDS]

ATGCGAGATCTAAAAACATATTTATCTGTAGCACCAGTAATAAGTACTCTATGGTTTGGGTCTTTAGCAGGTCTATTGATAGAGATCAATCGTTTTTTCCCAGATGCGTTGACATTCCCTTTTTTTTCATTCTAG

>lcl|NC_015308.1_cds_YP_004327683.1_43 [gene=rpl33] [locus_tag=HebrCp064] [db_xref=GeneID:10351925] [protein=ribosomal protein L33] [protein_id=YP_004327683.1] [location=71802..72002] [gbkey=CDS]

ATGGCCAAGGGTAAGGATATCCGAATAAGAGTTATTTTAGAATGTACCACTTGTGCTCGAAACAGTGTTAATAAGAAATCAACAGGTATTTCCAGATATATTACTCAAAAGAATCGACACAATACGCCTAGTCGATTGGAATTGAGAAAATTCTGTCGCTATTGTTACAAACATACAATTCACGGGGAGATAAAGAAATAG

>lcl|NC_015308.1_cds_YP_004327684.1_44 [gene=rps18] [locus_tag=HebrCp065] [db_xref=GeneID:10351926] [protein=ribosomal protein S18] [protein_id=YP_004327684.1] [location=72384..72668] [gbkey=CDS]

ATGGATAAATCCAAACGACTTTTTCTTAAGTCCAAGCGATCTTTTCGTAGGCGTTTGCCCCCGATCCAATCGGGGGATCAAATTGATTATAGAAATATGAGTTTAATTAGTCGATTTATTAGTGAACAAGGAAAAATATTATCTAGACGGGTGAATAGATTGAGTTTAAAACAACAACGATTAATTACTATTGCTATAAAACAAGCTCGTATTTTATCTTCGTTACCTTTTCTTAATAATGAAAAACAATTTGAAAAAAAGCGAGTTGGTCACTATAACTATTGA

>lcl|NC_015308.1_cds_YP_004327685.1_45 [gene=rpl20] [locus_tag=HebrCp066] [db_xref=GeneID:10351927] [protein=ribosomal protein L20] [protein_id=YP_004327685.1] [location=complement(73009..73362)] [gbkey=CDS]

ATGACCAGAATTAGACGAGGATATATAGCTCGGAGGCGTAGAACAAAAATTCGTTTATTCGCATCAAGCTTTCGCGGGGCCCATTCAAGACTTACTCGAACTATTATTCAACAAAAAATAAGAGCTTTGGTTTCGGCCCATCGGGATAGAGATAGGCAAAAAAGAAATTTTCGTCGTTTGTGGGTCACTCGGATAAATGCAGTAATTCGCGAGAGTATGGTATCCTATAGTTATAGTAGATTAATAAACAATCTGTACAAGAGACAGTTACTTCTTAATCGTAAAATACTTGCACAAATAGCTATATTAAATAGGAATTGTCTTTATATGATTTCCAATGACATTCTAAAATAA

>lcl|NC_015308.1_cds_YP_004327686.1_46 [gene=rps12] [locus_tag=HebrCp127] [db_xref=GeneID:10351928] [protein=ribosomal protein S12] [exception=trans-splicing] [protein_id=YP_004327686.1] [location=join(complement(74171..74284),146419..146650,147187..147212)] [gbkey=CDS]

ATGCCAACTATTAAACAACTTATTAGAAACACAAGACAGCCAATCAGAAATGTCACCAAATCCCCCGCTCTTGGGGGATGTCCTCAGCGCCGAGGAACATGTACTAGGGTGTATACTATCACCCCCAAAAAACCAAACTCTGCCTTACGTAAAGTTGCCAGAGTACGATTAACCTCTGGTTTTGAAATCACTGCTTATATACCTGGTATTGGCCATAATTTACAAGAACATTCTGTAGTCTTAGTAAGAGGGGGAAGGGTTAAGGATTTACCCGGTGTGAGATATCACATTGTTCGAGGAACCCTAGATGCTGTCGGAGTAAAGGATCGTCAACAAGGGCGTTCTAAATATGGGGTCAAAAAGCCAAAATAA

>lcl|NC_015308.1_cds_YP_004327687.1_47 [gene=clpP] [locus_tag=HebrCp067] [db_xref=GeneID:10351949] [protein=ATP-dependent Clp protease proteolytic subunit] [protein_id=YP_004327687.1] [location=complement(join(74495..74722,75364..75654,76490..76558))] [gbkey=CDS]

ATGCCTATTGGTGTTCCAAAAGTCCCTTTTCGAAATCCTGGGGAAGACGATTCAATTTGGATTGACGTAAACCGACTTTATCGAGAAAGATTACTTTTTTTAGGTCAAGATGTTGATAGCGAGATCTCGAATCAACTTATTGGTCTTATGGTATATCTCAGTATAGAAAGCGAGACAAAAGATTTGTATTTGTTTATAAACTCTCCCGGCGGATGGGTAATACCCGGAATAGCTATTTATGATACTATGCAATTTGTGCGACCCGATGTACAAACAGTATGCATGGGATTAGCTGCTTCAATGGGATCTTTTATTCTGGTCGGAGGAAAAATTACCAAACGTTTAGCATTCCCTCATGCTAGGGTAATGATTCATCAACCTATTGCTGGTTTTTATGAGGCACAAATAGGAGAATTTGTCCTGGAAGCGGAAGAACTACTGAAACTGCGCGAAATCCTCACAAGGATTTATGCACAAAGAACGGGCAAACCCTTATGGGTTGTATCCGAAGACATGGAAAGAGATGTTTTTATGTCAGCAACCGAAGCCCAAGCTCATGGAATTGTTGATCTTGTAGCAGTTGCATAA

>lcl|NC_015308.1_cds_YP_004327688.1_48 [gene=psbB] [locus_tag=HebrCp068] [db_xref=GeneID:10351929] [protein=photosystem II 47 kDa protein] [protein_id=YP_004327688.1] [location=77025..78551] [gbkey=CDS]

ATGGGTTTGCCTTGGTATCGTGTTCATACCGTCGTATTGAATGATCCCGGTCGTTTGCTGTCTGTCCATATAATGCATACAGCTTTGGTTGCTGGTTGGGCCGGTTCGATGGCTCTATATGAATTAGCAGTTTTTGATCCCTCTGACCCCGTTCTCGATCCAATGTGGAGACAAGGTATGTTCGTTATACCCTTTATGACTCGTTTAGGAATAACCAATTCATGGGGTGGTTGGAGTATCACAGGAGGAACTATAACGAATCCGGGTATTTGGAGTTATGAAGGCGTGGCTGGGGCGCATATTGTGTTTTCTGGCTTGTGCTTCTTGGCAGCTATTTGGCATTGGGTGTATTGGGATCTCGAAATATTTTGCGATGAACGTACAGGAAAACCTTCTTTGGATTTGCCCAAGATCTTTGGAATTCATTTATTTCTCTCCGGGGTGGCTTGCTTTGGGTTTGGCGCTTTTCATGTAACCGGATTGTATGGTCCTGGAATATGGGTGTCCGACCCTTATGGACTAACTGGAAAGGTACAACCTGTAAGTCCAGCATGGGGTGTGGAAGGTTTTGATCCTTTTGTTCCGGGAGGAATAGCCTCTCATCATATTGCAGCGGGGACATTGGGCATATTAGCGGGCCTATTCCATCTTAGTGTCCGTCCGCCCCAACGTTTATACAAAGGATTACGTATGGGAAATATTGAAACTGTCCTTTCCAGTAGTATCGCGGCTGTCTTTTTTGCAGCTTTTGTTGTTGCTGGAACTATGTGGTATGGTTCAGCAACTACCCCGATTGAATTATTTGGTCCCACTCGTTATCAATGGGATCAAGGATACTTCCAGCAAGAAATATATCGAAGAGTTAGTGCTGGGCTAGCCGAAAATCAAAGTTTATCCGAAGCTTGGTCTAAAATTCCCGAAAAATTAGCTTTTTATGATTACATCGGCAATAATCCGGCAAAGGGTGGGTTGTTCAGAGCAGGCTCAATGGACAACGGGGATGGAATAGCTGTTGGGTGGTTAGGACATCCTATCTTTAGAGATAAAGAAGGGCGTGAACTTTTTGTACGTCGTATGCCTACTTTTTTTGAAACATTTCCGGTTGTTTTGGTAGACGGAGATGGAATTGTTAGAGCCGATGTTCCTTTTCGAAGGGCAGAGTCGAAGTATAGTGTCGAACAAGTAGGTGTAACTGTTGAGTTCTATGGTGGCGAACTAAACGGAGTCAGTTATAGTGATCCTGCTACTGTGAAAAAATATGCTAGACGCGCTCAATTGGGTGAAATTTTTGAATTAGATCGTGCTACTTTGAAATCCGATGGTGTTTTTCGTAGCAGCCCAAGGGGTTGGTTTACTTTTGGACATGCTTCGTTCGCTCTGCTCTTTTTCTTCGGACACATTTGGCATGGTGCTCGAACTTTGTTCAGAGATGTTTTTGCTGGTATTGATCCAGATTTAGATGCTCAAGTGGAATTTGGAGCATTCCAAAAACTTGGAGATCCAACTACAAGAAGACAAGTAGTCTGA

>lcl|NC_015308.1_cds_YP_004327689.1_49 [gene=psbT] [locus_tag=HebrCp069] [db_xref=GeneID:10351930] [protein=photosystem II protein T] [protein_id=YP_004327689.1] [location=78711..78818] [gbkey=CDS]

ATGGAAGCATTGGTTTATACATTCCTTTTAGTCTCAACTTTAGGAATAATTTTTTTCGCTATCTTTTTTCGAGAACCGCCTAAAGTTCCAACTAAAAAGGTAAAATGA

>lcl|NC_015308.1_cds_YP_004327690.1_50 [gene=psbN] [locus_tag=HebrCp070] [db_xref=GeneID:10351931] [protein=photosystem II protein N] [protein_id=YP_004327690.1] [location=complement(78879..79010)] [gbkey=CDS]

ATGGAAACAGCAACCCTAGTCGCCATCTCTATATCTGGTTTACTTGTAAGTTTTACTGGGTATGCCTTATATACTGCTTTTGGGCAACCCTCTCAACAACTAAGAGATCCATTCGAGGAACACGGGGACTAG

>lcl|NC_015308.1_cds_YP_004327691.1_51 [gene=psbH] [locus_tag=HebrCp071] [db_xref=GeneID:10351932] [protein=photosystem II phosphoprotein] [protein_id=YP_004327691.1] [location=79121..79342] [gbkey=CDS]

ATGGCTACACAAAGCGTGGAGGGTAGTTCTAGATCTGGTCCAAGACGAACTATTGTAGGGGATTTATTGAAACCATTGAATTCGGAATATGGTAAAGTAGCTCCTGGATGGGGAACTACTCCTTTGATGGGTGTCGCAATGGCTCTATTTGCGATATTCCTATCTATTATTTTGGAGATTTATAATTCTTCCGTTTTACTAGATGGAATTTCAATGAACTAG

>lcl|NC_015308.1_cds_YP_004327692.1_52 [gene=petB] [locus_tag=HebrCp072] [db_xref=GeneID:10351933] [protein=cytochrome b6] [protein_id=YP_004327692.1] [location=join(79467..79472,80252..80893)] [gbkey=CDS]

ATGAGTAAAGTCTATGATTGGTTCGAAGAACGTCTCGAGATTCAGGCAATTGCAGATGATATAACTAGTAAATATGTTCCTCCCCATGTCAACATATTTTATTGTTTAGGAGGAATTACGCTTACTTGTTTTTTAGTACAAGTAGCTACGGGGTTTGCTATGACTTTTTACTACCGTCCGACCGTTACTGAGGCTTTTGCTTCTGTTCAATACATAATGACTGAGGCTAACTTTGGTTGGTTAATCCGCTCAGTGCATCGATGGTCGGCAAGTATGATGGTTTTAATGATGATCCTGCACGTATTTCGTGTATATCTCACTGGTGGCTTTAAAAAACCTCGTGAATTGACTTGGGTTACGGGCGTGGTTCTTGCTGTATTGACCGCATCTTTTGGTGTAACTGGTTATTCCTTACCTTGGGACCAAATTGGTTATTGGGCGGTCAAAATTGTAACAGGCGTGCCGGAAGCTATTCCTGTAATAGGATCGCCTTTGGTGGAGTTATTACGCGGAAGTGCTAGTGTAGGACAATCCACTTTGACTCGTTTTTATAGTTTACACACTTTTGTATTACCTCTTCTTACTGCCGTATTTATGTTAATGCACTTTTCAATGATACGTAAGCAGGGTATTTCAGGTCCTTTATAG

>lcl|NC_015308.1_cds_YP_004327693.1_53 [gene=petD] [locus_tag=HebrCp073] [db_xref=GeneID:10351934] [protein=cytochrome b6/f complex subunit IV] [protein_id=YP_004327693.1] [location=join(81106..81113,81964..82459)] [gbkey=CDS]

ATGGGAGTAACAAAAAAACCTGACTTGAATGATCCTGTATTAAGAGCTAAATTGGCTAAGGGAATGGGTCATAATTATTACGGAGAACCTGCATGGCCCAATGATCTTTTATATATTTTTCCAGTAGTAATTCTAGGTACTATTGCATGTAATGTAGGATTAGCAGTTCTAGAACCATCAATGATTGGTGAACCTGCGGATCCATTTGCAACGCCTTTGGAAATATTGCCTGAATGGTATTTCTTTCCCGTATTTCAAATACTTCGTACAGTACCCAATAAGTTATTAGGTGTTCTTTTAATGGTTTCAGTACCTGCAGGATTATTAACAGTACCTTTTTTGGAGAATGTTAATAAATTCCAAAATCCATTTCGTCGTCCAGTTGCGACAACCGTCTTTTTGATTGGTACTGCGGTAGCCCTTTGGTTAGGTATTGGAGCAACATTACCTATTGATAAATCCCTAACTTTAGGTCTTTTTCAAATTGATTCAATTGTAAAATAA

>lcl|NC_015308.1_cds_YP_004327694.1_54 [gene=rpoA] [locus_tag=HebrCp074] [db_xref=GeneID:10351935] [protein=RNA polymerase alpha subunit] [protein_id=YP_004327694.1] [location=complement(82641..83666)] [gbkey=CDS]

ATGGTTCGAGAGAAAGTAACAATATCCACTCGGACACTGCAGTGGAAATGTGTTGAATCAAGAACCGATAATAAACGTCTTTATTATGGACGCTTTATTCTGTCTCCTCTTATGAAAGGCCAAGCCGACACAATAGGCATTGCGATGCGAAGAGCTTTGCTTGGAGAAATAGAAGGAACATGTATCACACGTGCAAAATCTGAGAAAATACCACACGAATTTTCTACTATAGCAGGTATTCAAGAATCAATACATGAAATTTTAATGAATTTGAAAGAAATTGTATTAAGAAGCAATTTGTATGGAACTTGTAACGCATCTATTTGTGTCGAGGGTCCTGGATATGTAACTGCTCAAGACATCATCTTACCGCCTTTTGTGGAAATCATTGATAATACACAGCATATCGCTAGCCTAACGGAAGCAATTGATTTGTGTATTGGATTACAAATCGAGAGGAATCGCGGCTATCGTATAAACCCAACAAATAACTTTCAAGTTCAAGACGGAAGTTATTCTATAGATGCTGTATTCATGCCTGTTCGAAATGCGAATCATAGTGTTCATTCTTATGGAAATGGGAATGAAAAGCAAGAGATACTTTTTCTCGAAATATGGACAAATGGAAGTTTAACTCCTAAAGAAGCACTTCACGAAGCCTCCCGGAATTTGATTGATTTTTTTATTCCTTTTCTACATGCAGAAGAAGAAAACTTACATTTAGAAAAAAATCAACACAAAGTTACTTTACCCCTTTTTACTTTTCATGATAGATTGACTAAATTAAGAAAAAATAAAAAAGAAATAGCATTGAAATACATTTTTATTGACCAATCAGAATTGACTCCTAAGATCTATAATTGCCTCAAAAGGTCCAATATACATACATTATCGGATCTTTTGAAAAAGAGTCAAGAAGATCTTATGAAAATTGAACATTTTCGCATAGACGATGTAAAACATATATTGGGTATTCTAGAAATAGAAAAACATTTCGCAATTGATTTACCAAAGAATAAAATATAA

>lcl|NC_015308.1_cds_YP_004327695.1_55 [gene=rps11] [locus_tag=HebrCp075] [db_xref=GeneID:10351936] [protein=ribosomal protein S11] [protein_id=YP_004327695.1] [location=complement(83725..84141)] [gbkey=CDS]

ATGGCAAAACCTTTACCAAAAATTGGTTCACGAAGAAACGGACGTATTGGTTCGCGTAAGAATTCACGTAAAATACCAAAAGGAGTTATTCATGTTCAAGCAAGTTTTAACAATACTATTGTGACCGTTACAGATGTACGGGGTCGAGTGATTTCTTGGTCCTCCGCTGGCACTTGTGGATTCAGGGGCACAAGAAGAGGAACGCCATTTGCTGCTCAAACCGCAGCAGGAAATGCTATTCGGACAGTAGTGGATCAAGGTATGCAACGAGCAGAAGTCATGATAAAGGGTCCTGGTCTCGGACGAGATGCGGCATTAAGAGCTATTCGCAGAAGTAGTATACTATTAAGTTTCGTCCGGGATGTAACCCCTATGCCACATAATGGCTGCAGACCCCCTAAAAAAAGGCGCGTGTAA

>lcl|NC_015308.1_cds_YP_004327696.1_56 [gene=rpl36] [locus_tag=HebrCp076] [db_xref=GeneID:10351937] [protein=ribosomal protein L36] [protein_id=YP_004327696.1] [location=complement(84258..84371)] [gbkey=CDS]

ATGAAAATAAGAGCTTCTGTTCGTAAAATTTGTGAAAAATGTCGACTGATACGTAGACGAGGGCGAATTATAGTAATTTGCTTAAACCCGAGACATAAACAAAGACAAGGATAA

>lcl|NC_015308.1_cds_YP_004327697.1_57 [gene=rps8] [locus_tag=HebrCp078] [db_xref=GeneID:10351985] [protein=ribosomal protein S8] [protein_id=YP_004327697.1] [location=complement(84888..85292)] [gbkey=CDS]

ATGGGTAGGGATCCTATTGCTGACATAATAACCTCTATAAGAAATGCTGACATGAATAGAAAAGGAATCGTTCGAATAGCATCTACTAACATCACCGAAAACATTATTAAAATACTTTTAAGAGAAGGTTTTATTGAAAATGTCAGGAAACATAAGGAGGGCAACAAAAAATTTTTGGTTTTAACCCTACGACACAGAAGGAAGAGGAAAGAACCCTATAGAACTAGTCTAAATTTAAAACGGATCAGCCGACCTGGTCTACGAATCTATTCTAACTATCAAAAAATTCCTAGAATTTTGGGCGGGATGGGCATTGTAATTCTTTCTACTTCTCGGGGTATAATGACAGACCGAGAAGCTCGACTCGAAAGAATCGGCGGAGAAATCTTGTGTTATATATGGTAA

>lcl|NC_015308.1_cds_YP_004327698.1_58 [gene=rpl14] [locus_tag=HebrCp079] [db_xref=GeneID:10351939] [protein=ribosomal protein L14] [protein_id=YP_004327698.1] [location=complement(85530..85898)] [gbkey=CDS]

ATGATCCAATCTCAGACCCATTTGAATGTAGCGGATAACAGCGGAGCTCGAGAATTGATGTGTATTCGAATCATAGGGACTAGTAATCGCCGATATGCTCATATTGGTGACGTTATTGTTGCTGTGATCAAGGAAGCAGCACCAAATTCACCTCTAGAAAGATCAGAAGTAATCAGAGCTGTAATTGTACGTACTTGTAAAGAACTCAAACGTGATAATGGTATGATAATACGATATGATGACAACGCTGCAGTTGTCATTGATCAAGAAGGAAATCCAAAGGGAACTCGAATTTTTGGTGCAATCGCCCGGGAATTGAGACAGTTAAATTTTACTAAAATAGTTTCATTAGCACCTGAAGTGTTATAA

>lcl|NC_015308.1_cds_YP_004327699.1_59 [gene=rpl16] [locus_tag=HebrCp080] [db_xref=GeneID:10351940] [protein=ribosomal protein L16] [protein_id=YP_004327699.1] [location=complement(join(86026..86424,87609..87617))] [gbkey=CDS]

ATGCTTAGTCCCAAAAGAACCCGATTCCGTAAACAACATAGAGGAAGAATGAAAGGAATAGCTTTTCGAGGTAATCGTATTTGTTTCGGCAGATATGCTCTTCAGGCACTTGAACCCGCTTGGATTACATCTAGACAAATAGAAGCGGGGCGACGAGCAATGACACGAAATGCACGCCGCGGTGGAAAAATATGGGTACGCATATTTCCCGACAAACCGGTTACTTTAAGACCTACGGAAACACGTATGGGTTCGGGGAAAGGATCTCCCGAATATTGGGTAGCTGTCGTTAAACCAGGTAGAATACTTTATGAAATGGGCGGAGTAGCAGAAAATATAGCGAGAAAAGCTATTTCAATAGCAGCATCAAAAATGCCTATACGAACTCAATTCATTATTTCGGGATAG

>lcl|NC_015308.1_cds_YP_004327700.1_60 [gene=rps3] [locus_tag=HebrCp081] [db_xref=GeneID:10351941] [protein=ribosomal protein S3] [protein_id=YP_004327700.1] [location=complement(87811..88467)] [gbkey=CDS]

ATGGGACAAAAAATAAATCCACTTGGTTTCAGACTTGGTACAACCCAAAGTCATCATTCTCTTTGGTTTGCACAACCAAAAAATTACTCTGAGGGTCTACAAGAAGATCAAAAAATAAGAAACTGTATCAAGAATTATGTAAAAAAAAATGCGAAAATATCTTCTGGTATTGAAGGAATTGCACGTATAGAGATTCAAAAACGAATTGATGTGATTCAGGTCATAATATATATGGGATTCCAAAAATTATTAATAGAAAGTAGACCTAAACGAATCGAAGAATTGCAGATGAATGTACAAAAAGAACTTAATTGTGTGAATCGAAAACTCAATATTACTATTACAAGAATTACAAACCCCTATGGGCACCCTACTATTCTTGCAGAATTTATAGCCGGACAATTAAAGAATAGAGTTTCATTTCGCAAAGCAATGAAAAAAGCTATTGAATTAACTGAACAGACAGATACAAAAGGAATTCAAGTGCAAATTGCGGGACGTCTTGACGGAAAAGAAATTGCACGCGTCGAATGGATTAGAGAGGGTAGAGTTCCTCTACAAACCATTGGAGCTAAAATTGATTATTGTTCGTATACAGTTAGAACTATTTATGGGGTATTGGGCATAAAAATTTGGATATTTCCAGACAAGAAATAA

>lcl|NC_015308.1_cds_YP_004327701.1_61 [gene=rpl22] [locus_tag=HebrCp082] [db_xref=GeneID:10351942] [protein=ribosomal protein L22] [protein_id=YP_004327701.1] [location=complement(88549..88950)] [gbkey=CDS]

ATGATAAATAAAAGAAAGAGAAAGAGAGACCCATATACAGAAGTATATGCTTTAGGCCAACATATATGTATGTCCCCTCACAAAGCACGAAGAATAATTGATCAGATTCGTGGACGTTCTTACGAAGAAACACTTATGATACTCGAGCTCATGCCTTATCGAGCATGTTATCCCCTTTTTAAATTGATTTATTCTGCAGCAGCAAATGCTAGTCACAATATGGGTTTCAACGAAGTCAATTTAATCATTAGTAAAGCCGAAGTCAATGAAGGCACTACTGTGAAAAAATTAAAACCTCAGGCTCGAGGACGGGGTTATCTGATAAAAAGATCAACTTGTCATATAACTATTATATTAAAAAATATATCCTTATATGAAGAATATGAAGAATATAACATATGA

>lcl|NC_015308.1_cds_YP_004327702.1_62 [gene=rps19] [locus_tag=HebrCp083] [db_xref=GeneID:10351943] [protein=ribosomal protein S19] [protein_id=YP_004327702.1] [location=complement(89028..89306)] [gbkey=CDS]

GTGACACGTTCACTAAAAAAAAATCCTTTTGTAGCAAATCATTTATTAAAAAAAATAAATAAGCTTAACAATAAAGCAGAAAAACAAATAATAAAAACGTGGTCCCGGACATCTACCATTATACCCACAATGATTGGCCATACTATTGCTATCCATAATGGAAAGGAACATTTACCTATTTATATAACAGACCGTATGGTGGGTCATAAATTGGGAGAATTTTCACCTACTCTAAATTTCCAGGGACATGCAAAAAATGATAATAAATCTCATCGTTAA

>lcl|NC_015308.1_cds_YP_004327703.1_63 [gene=rpl2] [locus_tag=HebrCp084] [db_xref=GeneID:10351944] [protein=ribosomal protein L2] [protein_id=YP_004327703.1] [location=complement(join(89373..89807,90481..90879))] [gbkey=CDS]

ATGGCGATACATTTATACAAAACTTCTACCCCGAGCACACGCAATGGAGCCGTAGACAGTCAAGCGAAATCCAATACACGAAATACACGAAAGAATTTGATCTATGGACAGCATCGTTGTGGTAAAGGCCGTAATGCCAGAGGAATCATTACCGCAAGACATAGAGGGGGAGGTCATAAGCGTCTATACCGTAAAATCGATTTTCGACGGAATGAAAAAGACATATATGGTAGAATCGTAACCATAGAATACGACCCTAATCGAAATGCATACATTTGTCTCATACACTATGGGGATGGTGAGAAGAGATATATTTTACATCCCAGAGGGGCTATAATTGGAGATACCGTTATTTCTGGTACAGAAGTTCCTATAAAAATGGGAAATGCCCTACCTTTGACCGATATGCCCTTAGGCACGGCCATACATAACATAGAAATCACACTTGGAAAGGGTGGACAATTAGCTAGAGCTGCAGGTGCTGTAGCGAAACTGATTGCAAAAGAGGGGAAATCAGCCACATTAAAATTACCTTCGGGGGAGGTTCGTTTAATATCCAAAAACTGCTCAGCAACAGTCGGACAAGTAGGGAATACTGGGGTGAACCAGAAAAATTTGGGTAGAGCCGGATCTAAATGTTGGCTAGGTAAGCGTCCTGTAGTAAGAGGAGTAGTTATGAACCCTGTAGACCATCCCCATGGGGGTGGTGAAGGGAGGGCCCCAATTGGTAGAAAAAAACCCGCAACCCCTTGGGGTTATCCTGCACTTGGAAGAAGAAGTAGAAAAAGGAATAAATATAGTGATAATTTGATTCTTCGTCGCCGTAGTAAATAG

>lcl|NC_015308.1_cds_YP_004327704.1_64 [gene=rpl23] [locus_tag=HebrCp085] [db_xref=GeneID:10351988] [protein=ribosomal protein L23] [protein_id=YP_004327704.1] [location=complement(90898..91179)] [gbkey=CDS]

ATGGATGGAATCAAATATGCAGTATTTACAGACAAAAGTATTCGGTTATTGGTGAAAAATCAATATACTTTTAATGTCGAATCAGGATCAACTAGGACAGAAATAAAGCATTGGGTCGAACTCTTCTTTGGTGTCAAGGTAATAGCTATGAATAGCCATCGACTCCCGGGAAAGGGTAGAAGAATGAGACCTATTATGGGACATACAATGCATTACAGACGTATGATCATTACGCTTCAACCGGGTTATTCTATTCCACCTCTTAGAAAGAAAAGAACTTAA

>lcl|NC_015308.1_cds_YP_004327705.1_65 [gene=ycf2] [locus_tag=HebrCp087] [db_xref=GeneID:10351986] [protein=hypothetical chloroplast RF2] [protein_id=YP_004327705.1] [location=91507..98418] [gbkey=CDS]

ATGAAAGGACATCAATTCAAATCCTGGATTTTCGAATTGAGAGAGATATTGAGAGAGATCAAGAATTCTCACTATTTCTTAGATTCATGGACCCAATTCAATTCAGTGGGATCTTTCATTCACATTTTTTTCCATCAAGAACGTTTTATAAAACTCTTGGACTCCCGAATTTGGAGTATCTTACTTTCACGCAATTCACAGGGTTCAACAAGCAATCGATATTTCACGATCAAGGGTGTAGTACTATTTGTAGTAGTGGTCCTTATATATCGTATTAACAATCGAAAGATGGTCGAAAGAAAAAATCTCTATTTGACAGGGCTTCTTCCTATACCTATGAATTCCATTGGACCCAGAAATGATACATTGGAAGAATCCTTTGGGTCTTCCAATATCAATAGGTTGATTGTTTCGCTCCTGTATCTTCCAAAAGGAAAAAAGATCTCTGAGAGCTCTTTCCTGGATCCGAAAGAGAGTACTTGGGTTCTCCCAATAACTAAAAAGTGTATCATGTCTGAATCTAACTGGGGTTCGCGGTGGTGGAGGAACTGGATCGGAAAAAAGAGGGATTCTAGTTGTAAGATATCTAATGAAACCGTTGCTGGAATTGAGATCTCATTCAAAGAAAAAGATATCAAATATCTGGAGTTTCTTTTTGTATATTATATGGATGATCCGATCCGCAAGGACCATGATTGGGAATTGTTTGATCGTCTTTCTCCGAGGAAGGGGCGAAACATAATCAACTTGAATTCGGGACAGCTATTCGAAATCTTAGTGAAAGACTGGATTTGTTATCTCATGTTTGCTTTTCGTGAAAAAATACCAATTGAAGTGGAGGGTTTCTTCAAACAACAAGGAGCTGGGTCAACTATTCAATCAAATGATATTGAGCATGTTTCCCATCTCTTCTCGAGAAAGAAGTGGGCTATTTCTTTGCAAAATTGTGCTCAATTTCATATGTGGCAATTCCGCCAAGATCTCTTCGTTAGTTGGGGGAATAATCCGCACGAATCGGATTTTTTGAGTAACATATCGAGGATTTGGTTAGACAATGTGTGGTTGGTAAACAAGGATCGGTTTTTTAGCAAGGCACGGAATATATCGTCAAATATTCAATATGATTCCACAAGATCTAGTTTCGTTCAAGGAAGGAATTCTAGCCAATTGAAGGGATCTTCTGATCAATCCAGAGATCATTTCGATTCCATTAGTAATGAGGATTCGGAATATCACACATTGATCAATCAAAGAAAGATTCAACAACTAAAAGAAAGATCGATTCTTTGGGATCCTTCCTTTCTTCAAACGGAACGAACAGAGATAGAATCAGACCGATTCCCTAAATGCCTTTCTGGATATTCCTCAATGTCCCGGCTATTCACGGAAGGTGAGAAGGAGATGAATAATCATCTGCTTCCGGAAGAAATCGAAGAATTTCTTGGGAATCCTACAAGATCCATTCGTTCTTTTTTCTCTGACAGATCGTCAGAACTTTATCTGGGTTCGAATCCTACTGAGAGGTCCACTAGAGATCAGAAATTGTTGAAGAAAGAACAAGATGTTTCTTTTGTCCCTTCCAGGCGATCGGAAAATAAAGAAATAGTTAATATATTCAAGATAATCACGTATTTACAAAATACCGTCTCAATTCATCCTATTTCATCAGATCCGGGATGTGATATGGTTCTGAAGGATGAACTGGATATGGACAGTTCCAATAAGATTTCTTTCTTGAACAAAAATCCATTTTTTGATTTATTTCATCTATTCCATGATCGGAACGGGGGGGGATACACGTTACACCACGATTTTGAATCAGAAGAGAGATTTCAAGAAATGGCAGATCTATTCACTCTATCAATAACCGAGCCGGATCTGGTGTATCATAAGGGATTTACCTTTTTTATTGATTCCTACGGATTGGATCAAAAACAATTCTTGAATGAGGTATTCAACTCCAGGGATGAATCGAAAAAGAAATCTTTATTGGTTCTACCTCCTATTTTTTATGAAGAGAATGAATCTTTTTATCGAAGGATCAGAAAAAAATGGGTCCGGATCTCCTGCGGGAATGATTTGGAAGATCCAAAACAAAAAATAGTGGTATTTGCTAGCAACAACATAATGGAGGCAGTCAATCAATATGGATTGATCCTAAATCTGATTCAAATCCAATATAGTACCTATGGGTACATAAGAAATGTATTGACTCAATTCTTTTTAATGAATAGATCCGATCGCAACTTCGAATATGGAATTCAAAGGGATCAAATAGGAAATGATACTCTGAATCATAGAACTATAATGAAATATACGATCAACCAACATTTATCGAATTTGAAACAGAGTCAGAAGAAATGGTTCGATCCTCTTATTTTTCTTTCTCGAACCGAGAGATCCATGAATTGGGATCCTAATGCATATAGATACAAATGGTCTAATGGGAGCAAGAATTTCCAGGAACATTTGGAACATTTCATTTCTGAGCAGAAGAGCCGTTTTCTTTTTCAAGTAGTGTTCGATCGATTACGTATTAATCAATATTCGATTGATTGGTCTGAGGTTATCGACAAAAAAGATTTGTCTAAGTCACTTCGTTTCTTTTTGTCCAAGTTACTTCTTTTTTTGTCCAAGTTTCTTCTCTTTTTGTCTAACTCACTTCCTTTTTTCTTTGTGAGTTTCGGGAATATCCCCATTCATAGGTCCGAAATCCATATCTATGAATTGAAAGGTCCGAATGATCAACTCTGCAATCAGCTGGTAGAACCAATAGGTCTTCAAATCGTTCATTTGAAAAAATTGAAACCCTTCTTATTGTTATTGGATGATCATGATACTTCCCAAAAATCTAAATTTTTGATTAATGGAGGAACAATATCACCATTTTTGTTCAATAAGATAACAAAGTGGATGATTGACTCATTCCATACTAGAAATAATCGCAGGAAATCTTTTGATAACACGGATTCCTATTTCTCAATGATATCCCACGATCAAGACAATTGGCTGAATCCCGTGAAACCATTTCATAGAAGTTCATTGATATCTTCTTTTTATAAAGCAAATCGACTTCGATTCTTGAATAATCTACATCACTTCTGCTTCTATTGTAACAAAAGATTCCCTTTTTATGTGGAAAAGGCCCGTATCAAGAATTATGATTTTACGTATGGACAATTCCTCAATATCTTGTTCATTCGCAACAAAATATTTTCTTTGTGCGGCGGTAAAAAAAAACATGCTTTTTTGGAGAGAGATACTATTTCACCAATCGAGTCACAGGTATCTAACATATTCATACCTAATGATTTTCCACAAAGTGGTAACGAAAGGTATAACTTGTACAAATCTTTCCATTTTCCAATTCGATCCGATCCATTCGTTCGTAGAGCTATTTATTCGATCGCAGACATTTCTGGAACACCTCTAACAGAGGGACAAATAGTCAATTTTGAAAGAACTTATTGTCAACCTCTTTCGGATATGAATCTATCTGATTCAGAAGGGAAGAACTTGCATCAGTATCTCAATTTCAATTCAAACATGGGTTTGATTCACACTCCATGTTCTGAGAAATATTTACCATCCGAAAAGAGGAAAAAACGGAGTCTTTGTCTAAAGAAATGTGTTGAAAAAGGGCAGATGTATAGAACCTTTCAACGAGATAATGCTTTTTCAACTCTCTCAAAATGGAATCTATTCCAAACATATATGCCATGGTTCCTTACTTCGACGGGGTACAAATATCTAAATTTGATATTTTTAGATACCTTTTCGGACCTATTACCGATACTAAGTAGCAGTCAAAAATTTGTATCCATTTTTCATGATATTATGCATGGATCAGATATATCATGGCGAATTCTTCAGAAAAAATTGTGTCTTCCACAATGGAATCTGATAAGTGAGATTTCGAGTAAGTGTTTACATAATCTTCTTCTGTCCGAAGAAATGATTCATCGAAATAATGAGCCACCATTGATATCGACACATCTGAGATCGCCAAATGTTCGGGAGTTCCTCTATTCAATCCTTTTCCTTCTTCTTGTTGCTGGATATCTCGTTCGTACACATCTTTTCTTTGTTTCCCGAGCCTATAGTGAGTTACAGACAGAGTTCGAAAAGGTCAAATCTTTGATGATTCCATCATACATGATTGAGTTGCGAAAACTTCTGGATAGGTATCCTACATCTGAACTGAATTCTTTCTGGTTAAAGAATCTCTTTCTAGTTGCTCTGGAACAATTAGGAGATTTTCTAGAAGAAATGCGGGGTTCTGCTTCTGGCGGCAACATGCTATGGGGTGGTGGTCCCGCTTATGGGGTTAAATCAATACGTTCTAAGAAGAAATTTTTTAATATCAATCTCATCGATCTCATAAGTATCATACCAAATCCCATCAATCGAATCACTTTTTCGAGAAATACGAGACATCTAAGTCATACAAGTAAAGAGATTTATTCATTGATAAGAAAAAGAAAAAACGTGAACGGTGATTGGATTGATGATAAAATAGAATCCTTGGTCGCGAACAGTGATTCGATTGATGATAAAGAAAGAGAATTCTTGGTTCAGTTCTCCACCTTAACGACAGAAAAAAGGATTGATCAAATTCTATTGAGTCTGACTCATAGTGATCATTTATCAAAGAATGACTCTGGTTATCAAATGATTGAAGAGCCGGGAGCAATTTATTTACGATACTTAGTTGACATTCATAAAAAGTATCTAATGAATTATGAGTTCAACACACCCTGTTTAGTAGAAAGACGGATATTCCTTGCTTATTATCAGACAACCACTTATTCACAAACCTCGTGTGGGGTGAATAGTTTTCATTTCCCATCTCATGGAAAACCCTTTTCGCTCCGCTTAGCCCTATCCCCCTCTAGGGGTATTTTAGTGATAGGTTCTATAGGAACTGGACGATCCTATTTGGTCAAATACCTAGCGACAAACTCCTATCTTCCTTTCATTACAGTATTTCTGAACAAGTTCCTGGATAACAAGCCTAAGGGTTTTCTTATTGATGATAGTGACGATATTGATGATAGTGACGATATTGATGATAGTGACGATATTGATGATAGTGACGATATTGATGTGAGTGACGATATTGATGTGAGTGACGATATTGACCGTGACTTTGATACGGAGCTGGAGTTTCTAACTAGGATGAATGTGCTAACTATGGATATGATGCCGGAAATAGACCGATTTTATATCACCCTTCAATTCGAATTAGCAAAAGCAATGTCTCCTTGCATAATATGGATTCCAAACATTCATGATCTGGATGTGAATGAGTCGAATTACTTATCCCTCGGTCTATTAGTGAACTATCTCTCCAGGGATTGTGAAAGATGTTCCACTAGAAATATTCTTGTTATTGCTTCGACTCATATTCCCCAAAAAGTGGATCCCGCTCTAATAGCTCCGAATAAATTAAATACATGCATTAAGATACGAAGGCTTCTTATTCCACAACAACGAAAGCACTTTTTTACTCTTTCATATACTAGGGGATTTCACTTGGAAAATAAAATGTTCCATACTAATGGATTCGGGTCCATAACCATGGGTTCCAATGTACGAGATCTTGTAGCACTTACCAATGAGGCCCTATCGATTAGTATTACACAGAAGAAATCAATTATAGACACTAATATAATTAGATCTGCTCTTCATAGACAAACTTGGGATTTGCGATCCCAGGTAAGATCGGTTCAGGATCATGGGATCCTTTTCTATCAGATAGGAAGGGCTGTTGCACAAAATGTATTTCTAAGTAATTGCCCCATAGATCCTATATCTATCTATATGAAGAAGAAATCATGTAACGAAGGGGATTCTTATTTGTACAAATGGTACTTCGAACTTGGAACGAGCATGAAGAAATTAACGATACTTCTTTATCTTTTGAGTTGTTCTGCCGGATCGGTTGCTCAAGACCTTTGGTCTCTACCCGGACCCGATGAAAAAAATGGGATCACTTATTATGGACTTGTTGAGAATGATTCTGATCTAGTTCATGGCCTATTAGAAGTAGAAGGCGCTCTGGTGGGATCCTCACGGACAGAAAAAGATTGCAGTCAGTTTGATAATGATCGAGTGACATTGCTTCTTCGGCCCGAACCAAGGAGTCCCTTAGATATGATGCAAAATGGATCTTGTTCTATCCTTGATCAGAGATTTCTCTATGAAAAATACGAATCGGAGTTTGAAGAAGGGGAAGGAGAAGAAGTCCTCGACCCGCAACAGATAGAGGAGGATTTATTCAATCACATAGTTTGGGCTCCTAGAATATGGCGCCCTTGGGGTTTTCTATTTGATTGTATCGAAAGGCCCAATGAATTGGGATTTCCCTATTGGGCCAGGTCATTTCGGGGCAAGCGGATCATTTATGATGAAGAGGATGAGCTTCAAGAGAATGATTCGGAGTTCTTGCAGAGTGGAACCATGCAGTACCAGATACGAGATAGATCTTCCAAAGAACAAGGCTTTTTTCGAATAAGCCAATTCATTTGGGACCCTGCGGATCCACTCTTTTTCCTATTCAAAGATCAGCCCTTTGTCTCTGTGTTTTCACATCGAGAATTCTTTGCAGATGAAGAGATGTCAAAGGGGCTTCTTACTTCCCAAACAGATCCTCCTACATCTATATATAAACGCTGGTTTATCAAGAATATGCAAGAAAAGCACTTCGAATTGTTGATTCATCGCCAGAGATGGCTTAGAACCAATAGTTCATTATCTAATGGATTTTTCCGTTCTAATACTCTATCCGAGAGTTATCAGTATTTATCAAATCTGTTCCTATCTAACGGAACGCTATTGGATCAAATGACAAAGGCATTGTTGAGAAAAAGATGGCTTTTCCCGGATGAAATGAAAATTGGATTCATGTAA

>lcl|NC_015308.1_cds_YP_004327706.1_66 [gene=ndhB] [locus_tag=HebrCp090] [db_xref=GeneID:10351990] [protein=NADH dehydrogenase subunit 2] [protein_id=YP_004327706.1] [location=complement(join(100137..100892,101575..102351))] [gbkey=CDS]

ATGATCTGGCATGTACAGAATGAAAACTTCATTCTCGATTCTACGAGAATTTTTATGAAAGCCTTTCATTTGCTTCTCTTCGATGGAAGTTTTATTTTCCCAGAATGTATCCTAATTTTTGGCCTAATTCTTCTTCTGATGATCGATTCAACCTCTGATCAAAAAGATATACCTTGGTTATATTTCATCTCTTCAACAAGTTTAGTAATGAGTATAACGGCCCTATTGTTCCGATGGAGAGAAGAACCTATGATTAGCTTTTCGGGAAATTTCCAAACGAACAATTTCAACGAAATCTTTCAATTTCTTATTTTACTATGTTCAACTCTATGTATTCCTCTATCCGTAGAGTACATTGAATGTACAGAAATGGCTATAACAGAGTTTCTCTTATTCGTATTAACAGCTACTCTAGGAGGAATGTTTTTATGCGGTGCTAACGATTTAATAACTATCTTTGTAGCTCCAGAATGTTTCAGTTTATGCTCCTACCTATTATCTGGATATACCAAGAAAGATGTACGGTCTAATGAGGCTACTACGAAATATTTACTCATGGGTGGGGCAAGCTCTTCTATTCTGGTTCATGCTTTCTCTTGGCTATATGGTTCGTCCGGGGGAGAGATCGAGCTTCAAGAAATAGTGAATGGCCTTATCAATACACAAATGTATAACTCCCCAGGAATTTCAATTGCGCTTATATTCATCACTGTAGGAATTGGGTTCAAGCTTTCCCTAGCCCCTTCTCATCAATGGACTCCTGACGTATACGAAGGATCTCCCACTCCAGTCGTTGCTTTTCTTTCTGTTACTTCGAAAGTAGCTGCTTCAGCTTCAGCCACTCGAATTTTCGATATTCCTTTTTATTTCTCATCAAACGAATGGCATCTTCTTCTGGAAATCCTAGCTATTCTGAGCATGATAGTGGGGAATCTCATTGCTATTACTCAAACAAGCATGAAACGTATGCTTGCATATTCGTCCATAGGTCAAATCGGATATGTAATTATTGGAATAATTGTTGGAGACTCAAATGGTGGATATGCAAGCATGATAACTTATATGCTCTTCTATATCTCCATGAATCTAGGAACTTTTGCTTGTATTGTATTATTTGGTCTACGTACCGGAACTGATAACATTCGAGATTATGCAGGATTATACACGAAAGATCCTTTTTTGGCTCTCTCTTTAGCCCTATGTCTCTTATCCCTAGGAGGTCTTCCTCCACTAGCAGGTTTTTTCGGAAAACTCCATTTATTCTGGTGTGGATGGCAGGCAGGCCTATATTTCTTGGTTTTAATAGGACTCCTTACGAGCGTTGTTTCTATCTACTATTATCTAAAAATAATCAAGTTATTAATGACTGGACGAAACCAAGAAATAACCCCTCACGTGCGAAATTATAGAAGATCCCCTTTAAGATCAAACAATTCCATCGAATTGAGTATGATTGTATGTGTGATAGCATCTACTATACCAGGAATATCAATGAACCCGATTGTTGAAATTGCTCAAGATACCCTTTTTTAG

>lcl|NC_015308.1_cds_YP_004327707.1_67 [gene=rps7] [locus_tag=HebrCp091] [db_xref=GeneID:10351991] [protein=ribosomal protein S7] [protein_id=YP_004327707.1] [location=complement(102663..103130)] [gbkey=CDS]

ATGTCACGTCGAGGTACTGCAGAAGAAAAAACTGCAAAATCCGATCCAATTTATCGTAATCGATTAGTTAACATGTTGGTTAACCGTATTCTGAAACACGGAAAAAAATCATTGGCTTATCAAATTATCTATCGAGCCATGAAAAAGATTCAACAAAAGACAGAAACAAATCCACTATCTGTTTTACGTCAAGCAATACGTGGAGTAACTCCCGATATAGCAGTAAAAGCAAGACGTGTAGGCGGATCGACTCAGCAAGTTCCCATTGAAATAGGATCCACACAAGGAAAAGCACTTGCCATTCGTTGGTTATTAGGGGCATCCCGAAAACGTCCGGGTCGAAATATGGCTTTCAAATTAAGTTCCGAATTAGTGGATGCTGCCAAAGGGAGTGGTGATGCCATACGCAAAAAGGAAGAGACTCATAGAATGGCAGAGGCAAATAGAGCTTTTGCACATTTTCGTTAA

>lcl|NC_015308.1_cds_YP_004327708.1_68 [gene=ndhF] [locus_tag=HebrCp104] [db_xref=GeneID:10352004] [protein=NADH dehydrogenase subunit 5] [protein_id=YP_004327708.1] [location=complement(116048..118279)] [gbkey=CDS]

ATGGAACATATATATCAATATTCATGGATCATATCTTTCGTTACATTGCCAGTCCCTATGTTAATAGGAGTGGGACTCCTGCTTTTTCCGGCAGCAACAAAAAAACTGCGTCGTATGTGGGCTTTTCCAAGCGTTTTCTTGTTAAGTATAGTCATGATTTTTTCAATCGATTTGTCTATTCAGCAAATAAATAGTAGTTTTATTTATCAATATATATGGTCGTGGACTATTAATAATGATTTTTCTTTAGAATTCGGACACTTGATTGACCCACTTACTTCTATTTTGTCAGTATTAATTACTACAGTTGGTATTTTGGTTCTTTTTTATAGTGACAATTATATGTCTCATGATCAAGGTTATTTGAGATTTTTTGCTTATATGAGTTTTTTCACTACTTCAATGTTGGGATTAGTTACTAGTTCTAATTTGATACAAATTTATATTTTTTGGGAATTGGTTGGAGTGTGTTCTTATCTATTAATAGGTTTTTGGTTCACACGACCTATTGCATCGAATGCTTGTCAAAAAGCGTTTGTAACTAATCGTGTAGGGGATTTTGGTTTATTATTAGGGATTTTAGGTCTTTATTGGATAACGGGCAGTTTCGAATTTCGGGATTTGTTCAAAATCTTCAATAACTTGATTTATAATAATCAAGTTAATTTTTTATTTGTTACTTTGTGTGCATTTCTATTATTTTCTGGCGCAATTGCTAAATCGGCGCAATTTCCTCTTCATGTATGGTTACCAGATGCCATGGAAGGGCCTACTCCTATTTCAGCTCTGATACATGCTGCTACTATGGTAGCGGCGGGAATTTTTCTTGTAGCTCGCCTTTTTCCTCTTTTCGTAATTATACCTTTCATAATGAATCTAATAGCTTTGATAGGTATAATAACAGTATTTTTAGGAGTTACTTTAGCTCTTGCTCAAAAAGATATTAAGAGAAGTTTAGCCTATTCTACAATGTCTCAATTGGGTTATACGATGTTAGCTCTAGGTATGGGGTCTTATCGGGCTGCTTTATTTCATTTGATTACTCATGCCTATTCGAAAGCATTGTTGTTTTTAGGATCTGGATCCATTATTCATTCAATGGAAGCTATTCTTGGTTATTCTCCAGATAAGAGTCAAAATATGGTTCTTATGGGTGGTTTAACAAAACATATTCCAATTACAAAAACGGCTTTTTTATTAGGAACACTTTCTCTTTGTGGTATTCCACCCTTCGCCTGTTTTTGGTCCAAAGATGAAATTCTTAATGATAGTTGGTTGTATTCACCTATTTTCGCAATAATAGCCTGTTTCACAGCAGGATTAACTGCATTTTATATGTTTCGGGTTTATTTACTTACTTTTGACGGACATTTTAATGCTCATTTTAAAAATTACAGTGGTAAAAAAAACAGTTCATTTTATTCAATCTCTTTATGGGGTAAAGAAGGATCAAAAATGCTTAACAAAAATTTTCGTTTATTAGCTTTATTAACAATGAATAATAAGGAAAGGGACTCTTTTTTTGGTAAGAACACATATCAAATTGATGGTAATGTAAGAAATATGACGTGGCCCTTTATTACTGTTAAAAATTTTAACACTAAAAGGATTTTTTCCTATCCCCATGAATCGGATAATACTATGTTATTTCCTATGCTTATCTTAGTACTATTTACTTTGTTTATTGGAGCCATAGGAATTCCTTTCAATCAATTCAATCAAGAAGGAATACAGTTGGATATAGATATATTGTCAAAACTTTTAACCCCGTCTTTAAACCTTTTGCATCAAAATCCAGAAAATTCTGTGGATTGGTATGAATTTGTAACAAATGCAATTTTTTCAGTCAGTATAGCTTTTTTCGGAATATTTATAGCGTCCTTTTTATATAAGCCTGTTTATTCATCGTTACTAAATTTTAATTTCTTTAATTCGTTTGCTAAAAAAGGCCCTAAGAGAATTTTTTGGGACAAAATAATAAATGTGATATATAATTGGTCCTCTAATCGAGGTTATATAGATGCTTTTTATGCAATATCTTTTATTGGGGGTATAAGAAAATTGGTTGAATTAATTCATTTTTTTGATAAACGAATAATTGATGGAATCACCAATGGGGTCGGTGTTACCAGTTTCTTTGTAGGAGAGGGTATAAAATATGTAGGAAGTGGTCGCATCTCTTCTTATCTCTTATTTTATTTATTTTATGCATTAATTTTTTTATTAATTTGA

>lcl|NC_015308.1_cds_YP_004327709.1_69 [gene=rpl32] [locus_tag=HebrCp105] [db_xref=GeneID:10351950] [protein=ribosomal protein L32] [protein_id=YP_004327709.1] [location=119274..119435] [gbkey=CDS]

ATGGCAGTTCCAAAAAAGCGCACTTCTATATCAAAAAAGCTTATTCGAAAAAATATTTGGAAAAGAAAGGGATATTGGACAGTATTGAAAGCTTTTTCGTTAGCGAAATCTCTTTCTACAGGTAATTCAAAAAGTTTTTTTGTGCAACAAATAAAAGATTAA

>lcl|NC_015308.1_cds_YP_004327710.1_70 [gene=ccsA] [locus_tag=HebrCp107] [db_xref=GeneID:10352005] [protein=cytochrome c biogenesis protein] [protein_id=YP_004327710.1] [location=120443..121411] [gbkey=CDS]

ATGATATTCTCGACTTTAGAACATATATTAACGCATATATCTTTTTCAGTCGTGTCAATTGTAATTACAATTCATTTGATAACCTTATTAGTCGATGAATTCGTAGAACTATATGATTCGTCAGAAAAGGGCATGATAACTACTTTTTTCTGTATAACAGGATTATTAGTTATTCGTTGGATTTTTTTGGGACATTTACCATTAAGTGATTTATATGAATCATTAATCTTTCTTTCATGGGCTTTTTCCATTATTCATATGGTTCCGTATTTTAAAAAACATAAAAATTTTTTAAGCGCAATAACCGCGCCAAGTACTTTTTTTACCCAAGGGTTTGCTACTTCGGGTCTTTTAACTGACATGCATCAATCCGAAATCTTAGTGCCCGCTCTCCAATCTCAGTGGTTAATGATGCACGTAAGTATGATGATATTGGGCTATGCAGCTCTTTTGTGTGGATCATTATTTTCAGTAGCATTTCTAGTAATCACATTTCGAAAAATCATAAGAATTGTTGATAAAAGCAATAATTTATTAAACGATTCGTTTTTCTTTAGTGAGATACAATATATGGCGGAAAGAAAGAATGTTTTAAGAAATATTTCTTTTCTTTCTTCTAGGAATTATTACAGGTTTCAATTGATTCAACAATTAGATGACTGGGGTTGTCGTATTATAAGTATAGGGTTTATCTTTTTAACCATAGGTATTCTTTCGGGAGCAGTCTGGGCTAATGAAGCATGGGGATCATATTGGAATTGGGACCCAAAAGAAACTTGGGCATTTATTACGTGGACCATATTCGCGATTTTTTTTCATATTCGAACAAATAAAAAATTGGAGGGTTTCAATTCCGCAATTGTCGCTTCTATCGGTTTTCTTCTAATTTGGATATGCTATTTTGGAGTTAATTTATTAGGAATAGGACTACATAGTTATGGTTCATTTACATTAACAATTAGCATCTAA

>lcl|NC_015308.1_cds_YP_004327711.1_71 [gene=ndhD] [locus_tag=HebrCp108] [db_xref=GeneID:10351952] [protein=NADH dehydrogenase subunit 4] [protein_id=YP_004327711.1] [location=complement(121688..123190)] [gbkey=CDS]

ATGAATTCTTTTCCTTGGTTAACAATATTTGTAGTTTTACCGATATCCGCGGGTTCCTTAATTTTCCTTTTCCCTCATAGAGGAAATAAAGTAATTAAGTGGTATACTATATTTATATGTGCCTTTGAACTCCTTTTAATGAATTATGTGTTCTCTTATTATTTCCAATTGGACGATCCATTAATCCAATTAACAGAAGATTATAAATGGATCCAATTTTTTGATTTTTACTGGAGATTGGGAATCGATGGATTTTCTTTAGGACCTATTTTACTGACAGGATTTATCACTACTTTAGCTACTTTAGCGGCTCGGCCAATTACTCGGGATTCTCGATTATTCCATTTTCTGATGTTAGCAATGTATAGTGGTCAAATAGGATTATTTTCTTCTCAAGATCTTTTACTTTTTTTTATCATGTGGGAGTTAGAATTAATTCCCGTTTATCTACTTCTATCCATGTGGGGGGGAAAGAAACGTCTGTATTCAGCTACAAAGTTTATTTTGTATACTGCGGGAGGTTCCGTTTTTTTATTAATGGGAGCTTTGGGTATCGCTTTATATGGTTCTAATGAACCGAGATTCCATTTTGAAACATCAGCTAATCAATCATATCCTGTGGCGCTAGAAATATTTTTCTATATTGGATTTCTTATTGCTTTTGCTGTCAAATCACCGATTATACCCTTACATACATGGTTACCAGACACCCATGGGGAAGCACATTATAGTACTTGTATGCTTCTAGCCGGAATCTTATTAAAAATGGGGGCATATGGATTGGTTCGAATCAATATGGAATTATTATCTCACGCTCATTCTATTTTTTCTCCCTGGTTGATAATAGTAGGCGTAATGCAAATAATCTATGCAGCTTCAACATCTCCTGGTCAACGAAATTTAAAAAAAAGAATAGCCTATTCTTCTGTATCTCATATGGGTTTCATAATTATAGGAATTTGCTCTATAAGTGATATGGGACTCAATGGAGCCATTTTACAAATAATATCGCATGGATTTATTGGTGCCGCACTTTTTTTCTTGGCAGGAACGGGTTATGATAGAATACGTCGTGTTTATCTTGATGAAATGGGTGGAATGGCTACCTCAATGCCAAAAATATTCACGACATTCAGTATCTTATCACTAGCTTCCCTTGCATTACCAGGCATGAGCGGTTTTTTTGCGGAATTGATAGTATTTTTTGGAATAATTACCGGCCAAAAATATCTTTTAATGTCAAAAATATTAATTACTTTTGTAATGGCAGTTGGAATGATATTAACTCCTATTTATTTATTATCTATGTTACGCCAGATGTTCTATGGATATAAGCTGTTTAATGCCCCAAACTCTTCTTTTTTTGATTCTGGACCGCGGGAGTTATTTGTTTCGATCTCTATCCTTCTGCCTGTAATAGGTATTGGTATTTATCCGGATTTCGTTTTCTCATTATCAGTTGACAGGGTTGAAGCTATTCTATCTAATTATTTTTATAGATAG

>lcl|NC_015308.1_cds_YP_004327712.1_72 [gene=psaC] [locus_tag=HebrCp109] [db_xref=GeneID:10351953] [protein=photosystem I subunit VII] [protein_id=YP_004327712.1] [location=complement(123333..123578)] [gbkey=CDS]

ATGTCACATTCAGTAAAGATTTATGATACATGTATAGGGTGTACTCAATGTGTCCGAGCCTGCCCCACAGATGTATTAGAAATGATACCTTGGGATGGATGTAAATCCAAGCAAATTGCTTCTGCTCCAAGAACAGAGGACTGTGTCGGTTGTAAGAGATGTGAATCCGCCTGTCCAACGGATTTCTTGAGTGTTCGAGTTTATTTATGGCATGAAACAACTCGAAGCATGGGTCTAGCTTATTGA

>lcl|NC_015308.1_cds_YP_004327713.1_73 [gene=ndhE] [locus_tag=HebrCp110] [db_xref=GeneID:10351954] [protein=NADH dehydrogenase subunit 4L] [protein_id=YP_004327713.1] [location=complement(123825..124130)] [gbkey=CDS]

ATGATGCTCGAACATGTACTTGTTTTGAGTGCCTATTTATTTTCTATTGGTATCTATGGATTGATCACGAGTCGAAATATGGTTAGAGCCCTTATGTGCCTTGAACTTATACTGAATGCAGTTAATCTAAATTTCGTAACATTTTCTGATTTTTTTGATAGCCGCCAATTAAAAGGAAATATTTTTTTCCATTTTGTTATAGCTATCGCAGCCGCTGAAGCAGCTATTGGACCAGCTATTGTTTCATCAATTTATCGTAATAGAAAATCAACCCGTATCAATCAATCGAATTTGTTGAATAAGTAG

>lcl|NC_015308.1_cds_YP_004327714.1_74 [gene=ndhG] [locus_tag=HebrCp111] [db_xref=GeneID:10351955] [protein=NADH dehydrogenase subunit 6] [protein_id=YP_004327714.1] [location=complement(124365..124895)] [gbkey=CDS]

ATGGATTTGCCTGGACTAATTCATGATTTTCTTTTAGTCTTTCTAGGATTAGGTCTTATATTAGGAGGTCTAGGAGTGGTATTACTTACCAACCCAATTTATTCTGCCTTTTCGTTGGGATTGGTTCTTGTTTGTATATCTTTATTCTATATTTTATCAAACTCTCATTTTGTAGCTGCCGCACAGCTCCTTATTTATGTGGGAGCTATAAATGTTTTAATTATATTTGCCGTGATGTTCATGAATGGTTCAGAATATTACAAAGATTTTAATCTTTGGACTGTTGGAAGCGGGGTTACTTCTTTAGTTTGTACAAGTATTTTTGTTTCACTAATTACTATTCTTCCAGATACGTCATGGTATGGAATTATTTGGACTACAAGAACAAATCAGATTATAGAACAAGATTTGATAAGTAATGGTCAACAAATTGGAATTCATTTATCAACAGATTTTTTTCTTCCATTTGAATTCATTTCAATAATTCTTTTAGTTGCTTTGATAGGTGCGATTGCTGTGGCTCGTCAGTAA

>lcl|NC_015308.1_cds_YP_004327715.1_75 [gene=ndhI] [locus_tag=HebrCp112] [db_xref=GeneID:10351956] [protein=NADH dehydrogenase subunit I] [protein_id=YP_004327715.1] [location=complement(125309..125806)] [gbkey=CDS]

ATGTTTCCCATGGTAACTGGGTTCATGAATTATGGGCAACAAACCATACGAGCTGCAAGGTACATTGGTCAAGGTTTTATGATTACCTTATCTCATGCAAATCGTTTACCTGTAACTATTCAATATCCTTATGAAAAATTAATCACATCGGAGCGTTTCCGCGGTCGAATTCATTTTGAATTTGATAAATGCATTGCTTGTGAAGTATGTGTTCGTGTATGTCCTATAGATCTACCTGTTGTTGATTGGAAATTGGAAACTGACATTCGAAAGAAACGGTTGCTTAATTACAGTATTGATTTCGGAATCTGTATATTTTGTGGCAACTGTGTTGAGTATTGTCCGACAAATTGTTTATCGATGACTGAAGAATATGAGCTTTCTACTTATGATCGTCACGAATTGAATTATAATCAAATTGCTTTAGGTCGTTTACCAATGTCAGTAGTTGACGATTATACAATTCGAACAATTTTGAATTCAACTCAAAAAAAATAG

>lcl|NC_015308.1_cds_YP_004327716.1_76 [gene=ndhA] [locus_tag=HebrCp113] [db_xref=GeneID:10351957] [protein=NADH dehydrogenase subunit 1] [protein_id=YP_004327716.1] [location=complement(join(125888..126427,127535..128086))] [gbkey=CDS]

ATGATAATTGATACAACAGAAATACAAGCTATCAATTCTTTTTCTAGATTAGAATCCTTAAACGAGGTCTATGGAATTATATGGGTGTTTGTCCCGATTTTTATTCTTGTATTGGGAATCACGATAGGCATACTAGTAATTGTATGGTTAGAAAGAGAAATATCTGCAGGGATACAACAACGTATTGGACCTGAATATGCCGGTCCTTTAGGAGTTCTTCAAGCTCTAGCGGATGGGACAAAACTACTTTTCAAAGAGAATCTTTTTCCATCTAGGGGGGATATTCGTTTATTCAGTATCGGACCATCCATAGCAGTCATATCAACTCTATTAAGCTATTCGGTAATTCCTTTTGGCTATCACCTTGTTTTAACTGATCTAAATATTGGTGTTTTTTTATGGATTGCCATTTCAAGTATTGCTCCCATCGGACTTCTTATGTCAGGATATGGATCAAATAATAAATATTCCTTTTTGGGTGGTTTACGAGCTGCTGCTCAATCGATTAGTTATGAAATACCATTAACTCTTTGTGTGTTATCCATATCTCTATTATCTAATAGTTCAAGTACAGTTGATATAGTTGAGGCACAATCAAAATCTGGTTTTTGGGGGTGGAATTTGTGGCGTCAACCTATAGGATTTATCATTTTTTTTATTTCTTCTCTAGCAGAATGTGAGAGATTGCCTTTTGATTTACCAGAAGCAGAAGAAGAATTAGTAGCAGGTTATCAAACCGAATATTCGGGCATCAAATTTGGTTTATTTTATATTGCTTCCTATCTAAACTTATTAGTTTCTTCATTATTTGTAACAGTTCTTTACTTGGGCGGTTGGAATATCTCTATTCCGTATATATTCGTTCCTGAGCTTTTTGAAATAAAAAAAATAGGCGGAGTCTTTGGAACAACAATTGGTATCTTTATTACATTGGTTAAAACTTATTTGTTCTTGTTCATTCCTATCACAACAAGATGGACTTTACCTAGACTAAGAATGGACCAACTTTTAAATCTTGGATGGAAATTTCTTTTACCTATTTCTCTCGGTAATCTATTATTAACAACCTCTTTCCAACTTCTTTCACTATAA

>lcl|NC_015308.1_cds_YP_004327717.1_77 [gene=ndhH] [locus_tag=HebrCp114] [db_xref=GeneID:10351958] [protein=NADH dehydrogenase subunit 7] [protein_id=YP_004327717.1] [location=complement(128088..129269)] [gbkey=CDS]

ATGAATGTACCAGCTACACGAAAAGACCTTATGATAGTTAATATGGGCCCCCACCACCCATCAATGCATGGTGTTCTTCGCCTCATCGTTACTCTAGACGGGGAAGATGTTATTGACTGTGAACCAATATTAGGTTATTTACACAGAGGAATGGAAAAAATTGCGGAAAATCGAACAATTATACAATATTTGCCCTATGTAACACGTTGGGATTATTTGGCTACTATGTTCACAGAAGCAATAACAGTAAATGGTCCAGAACTGTTAGGAAATATTCAAGTGCCTAAAAGAGCTGGCTATATCAGAATAATTATGTTGGAATTGAGTCGTATAGCTTCTCATTTGTTATGGCTTGGTCCCTTTATGGCAGATATTGGTGCACAGACTCCTTTCTTCTATATTTTTAGAGAAAGAGAGTTAGTATATGATTTATTCGAAGCTGCCACTGGTATGAGAATGATGCATAATTTTTTTCGTATCGGGGGAGTAGCGTCTGATCTACCTCATGGTTGGATAGATAAATGTTTGGATTTTTGCGATTATTTTTTAACAGGAGTTACTGAATATCAAAAACTTATTACGCGAAATCCTATTTTTTTAGAACGAGTTGAGGGAGTAGGTATTGTTGGTACAGAGGAAGCAAAAAATTGGGGTTTATCGGGACCAATGCTACGAGCTTCCGGAGTACAATGGGATCTTCGTAAAGTTGATCATTATGAGTGTTACGACGAATTTGATTGGGAAATCCAGTGGCAAAAAGAAGGAGATTCCTTAGCTCGTTATTTAGTCCGAATTGGTGAAATGATGGAATCTATAAAAATTATTCAACAGGCTCTGGAAGGAATTCCGGGGGGGCCCTATGAGAATTTAGAAATCCGACGTTTTGATAGAGAAAGGGATTCGGAATGGAACGATTTCGAATATCGATTCATTAGTAAAAAAACTTCTCCTACTTTTGAATTACCGAAACAAGAACTTTATGTGAGAGTCGAAGCCCCAAAAGGAGAATTGGGAATTTTTCTGATAGGAGATCAGAGCGGTTTTCCTTGGAGATGGAAAATTCGTCCGCCGGGTTTTATCAATTTGCAAATTCTTCCTGAATTAGTTAAAAGAATGAAATTGGCCGATATTATGACAATACTAGGTAGTATAGATATCATTATGGGAGAAGTTGATCGTTGA

>lcl|NC_015308.1_cds_YP_004327718.1_78 [gene=rps15] [locus_tag=HebrCp115] [db_xref=GeneID:10351959] [protein=ribosomal protein S15] [protein_id=YP_004327718.1] [location=complement(129367..129639)] [gbkey=CDS]

ATGGTAAAAAATTCATTCATTTCAGTTATTTCACAAGAAGAAAAAGACGAAAACAAGGGATCTGTTGAATTTCAAATAGTAAGTTTCACTAATAAGATACGAAGACTTACTTCACATTTGGAATTGCATAGAAAAGACTATTTATCTCAGAGAGGTTTGCGAAAAATTCTAGGAAAACGACAACGACTCCTGTCTTATTTAGCAAAGAAAAATAGAGTACGTTATAAAGAATTAATTATCCTGTTGGATATTCGGGAATCAAAAACTCGTTAA

>lcl|NC_015308.1_cds_YP_004327719.1_79 [gene=ycf1] [locus_tag=HebrCp116] [db_xref=GeneID:10351960] [protein=hypothetical chloroplast RF1] [protein_id=YP_004327719.1] [location=complement(130080..135779)] [gbkey=CDS]

ATGATTTTTCAATCTTTTATACTAGGTAATCTAGTATCCTTATGCATGAAGATAATCAATTCGGTCGTTGTGGTCGGACTCTATTATGGATTTCTGACCACATTCTCCATGGGACCCTCTTATCTCTTCCTTCTCCGAGCTCGGGTTATAGAAGAAGGAGAAGAAGGAACTGAGAAGAAGGTATCAGCAACAACAGGTTTTATTACGGGACAGCTCATGATGTTCATATCGATCTATTATGCGCCTCTGCATCTAGCATTGGGTAGACCTCATACAATAACTGTCCTAGCTCTACCCTATCTTTTGTTTCATTTCTTCTGGAACAATCACAAACACTTTTTTGATTATGGATCTACTACCAGAAATTCAATGCGTAATCTTAGCATTCAATTTGTATTCCTGAATAATCTCATTTTTCAATTATTCAACCATTTCATTTTACCAAGTTCAATGTTAGTCAGATTAGTCAACATTTATATGTTTCGATGCAACAACAAGATGTTATTTGTAACAAGTAGTTTTGTTGGTTGGTTAATTGGTCACATTTTATTCATGAAATGGGTTGGATTGATATTAGTCTGGATACAGCAAAATAATTCTATTAGATCTAATGTACTTTTTCGATCTAATAAGTACCTTGTGTCAGAATTGAGAAATTCTATGGCTCGAATCTTTAGTATTCTCTTATTTATTACCTGTGTCTACTCTTTAGGCAGAATACCGTCACCCATTTTTACTAAGAAACTGAAAGAAACCTCAGAAACGGAAGAAAGGGAGGAAGAAACAGATGTAGAAATAGAAAAAACTTCCGAAACGAAGGGGACTAAACAGGAACAAGAGGGATCCACCGAAGAAGATCCTTCTTCTTCCCTTTTTTCGGAAGAAAAGGAGGATCCGGACAAAATCGACGAAACGGAAGAGATCCAAGTGAATGGAAAGGAAAAAACAAAGGATGAATTCCATTTTCACTTTAAAGAGACATGCTATAAAAATAGACCACTTTATGAAACTTTTTATCTGGATGGGAATCAAGAAAATTCGAAGTTAGAAATATTGATAGATAAAAAAAATAAAGATCTTTTCTGGTTTGAAAAACCTCTTGTAACTATTCTTTTTGACTCTAAACGTTGGAATCGTCCATTTCGATATATAAAAAATGATCAGTTTGAGAATGCTGTAAGAAAAGAAATGTCACAATATTTTTTTTATACATGTCGGAGTGATGGAAAAGAAAGAATATCTTTTACGTATCCACCCAGTTTGTCAACTTTTTTGGAAATGATACAAAGAAAGATATCTCTGTTTACAACAGAAAAACTCTCCTCTGATGAATTGTATAATCGTTGGAATTATAAGAATGAACAAAAAAAGAAAATCCTAAATAATGAATTTATAAATAGAGTCCAGGCTCTAGATAAGGAATATCTTACTCTGAATACACTCGAAAAAAGGACTAGACTATGTAATGATAAAACTAAAAAAAAGTACTTACCTAAAATTTATGATCCCTTTTTGAGTGGGTCCTGCCGCGGGAAAATCCAATTTTTTTTTTCACCCTCACTCCTAAATAAAACTTCCATAAAAAATTCAATAGAGATGCTTTGGATAAATAAAATTCATCTTATTCTTCTTATTACTAATTATCAAGAATTTGAATCAAAAACAGATGGATTTAATAGAAAAGCATTTTCAATAGAAAATGCTTATTTCTTAAACTTAATTAATGAATTTGTTGGAAAATCAAGATCAAGTTTAAATTTTAAGGAACTTCCCTTATTTCCAGATCACAAAGAAGAAAAAATGTATTTAGAAAATCGAATAAAAATTTTAAAATTTTTATTTCATACAGTTATAGCGAATCCAAAAAATAAAACAATTATAAAAAATTCTACTGGAATAAAAGAAATAAGTAAACAAGTTCCTCGATGGTCATACAAATTAATTGACGATTTGGAACAACAAGAGGGAGAAAATGAAGAAAACATGTCGGAAGATTATGAAATTCGTTCACGAAAAGCCAAACGGGTAGTGATTTTTACTGATAATAAAAAAAATACAGATACTTATAATAATACCAAAGATACAACGAATTCTGATCAAATAGAAGAAGTGACTTTGATACATTATTCACAACAATCGGATTTTCGCCGAGACATAATAAAAGGATCCGTGCGCGCACAAAGACGCAAAATAGCTATTTGGGAACTGTTTCAAGCAAATGTGCATTCTCCTCTTTTTTTGGACAGAATAGACAAATCTCTTTTTTTTTCTTTTGATATTTCCGAACTGATAAAAACAATGTTTATAAATTGGATGTATAAAAACGCAGAATTCACAATTTCAAATACAAAGAAAAAAACAAAAGAAAGTAAGAAAAAAGAAGAGGACAAAAGAGAAGACAACAAAAGAGAGGAAAAAGCTCGGATAGAAATAGCCGAAGCCTGGGATAGCATTCTTTTTGCTCAAATAATAAGAAGTTGTGTTTTAGTAACCCAATCAATTCTTAGAAAATATATTATATTACCTTCATTAATAATAACTAAAAATATCATTCGTATACTTTTTTTTCAAACTCCCGAATGGTCCGAGGATTTAAAAGATTGGAGTAGAGAAATGCATGTTAAATGCACCTATAATGGAGTTCAATTATCAGAAAAAGAATTTCCGAAAAACTGGTTAATAGACGGGATTCAAATAAAGATCCTATTTCCTTTTCGTCTAAAACCTTGGTACAAATCTAAGTTAAAATTCTCTCATAAAGATCAAACGAAAATGAAAAAAAAAGTACAAAAAAATGATTTTTTTTTTTTAACAGTTTTGGGAATGGAAGCTGAACTTCCTTTTGGTTCTCCCCGAAAACGGCTTTCACTTTTTGAACCCATCTTTAAAAAACTCGAAAAAAAAATTAGAAAAATGCAAAAAAATGGTTTTCGAGTTATAACAATTTTAGAAGAAAGAAGAAAATTTTTTCTAAATTTCTTAAAAGAAAAAAAAAACTGGATCATCAAAAACATTTTTTTTCGAAAAGAAATAATAAACAACCTTTCAAAATCAAAAAGAAATCCAATTCTATTATCTGGATTTAGAGAAGTATATGAATTGAATGAACCTAAAAAAGAAAAAGATTCGATAATCAATAACAATAATGAGACGATTAAAAAATTATCCATCCCAATTCGATCTATGACTTGGACAAATTATTCACTGACAGAAAAAAAAATGAAAGATCTTTCTGCTAGAAGAAAGATAATCATAAATCAAATAGAAAAAATTACAAAAGAAAAGGAAAAAAAAATGAGAACCCCGGAAATAAATATTAGTCCTAACAAAAACAAAATAAGTTATAATGCTAAAAAATTAAAATCATCAAAAAATATTTCGCAGATAGTAAAAAGAAGAAATGTTCGATTAGCGCGTAAATTCCATTTTTTTATAAAAATTTTGATTGAAAAGATATACATAGATATCTTTTTAGGTATCATTAATATTCCAAGGATCAATGCACAGCTTTTTCTTAAATCAACAAAAAAAATTATTACTAAATACATTTACAATAATGAAGAAAATCAGAAAAAAATTGATAAAACAAATCAAAATACAATTCACTTTATTTCGATTATAAAAAAGTCACGTAATAGTAATAATAGTAATGTTGTTGTTATTAATAAAAATTCAAAGATTTTTTGTGACATATCCTCCTTGTCACAAGCTTATGTATTTTACAAATTATCACAAATCAAAATTATTAACTTATATAAGTTAAAATCTATCTTTGAATATCATAGCCTTTTTCTGAAGAATGAAATAAAGGATTTTTTTATAGCCCAAGGGCTATTTAATTCCGAATTAAAAAATAAAAATTTTAGAAATTCTGTAATGAATCAATGGAAAAATTGGTTAAGGAGTCATTATCAATATAAATACGATCTATCTCAGATTAGATGGTCTAGATTAACACCACAAAAATGGCGAAATATAATCAATCAACATCAACACCATATGGTTCAAAATAAAAAATTAAACAAATGGAATTTATATGAAAAAGACCGATTAATTCATTACAAAAAAAAAAATGATTTTGAGACAGATTCATTACCAAATCAAAAAGATAATTTTAAAAAACACTATAAATATAATCTTTTATCATATAAATCTATTAATTATGAAAATAAGAAGGACTCATATATTTATGGATCACCATTACAAGTAAATAATAAACAAGAGATTTCTTATAATTACAACACAAAAAAAAGCAAATTATTTGACATGTTGGAAGATATTTCTATCAATAATTATCTAGCGGAAGATGATATTATTGATATGGAGAAAAGCGCGGATAGAAAATATTTTGATTGGAGAATTCTCAATTTTTGTCTTAGAAAGAAGGTTGATATTGAGTCCTGGATCGATACCGGAAGCAAAGATAAAAAAAATACTAAGACTAGGACTAATAAGTATCAAATAATTGATAAAATTGATAAGAAAAATCTTTTTTTTCTTACAATTCACCAAGATCAAGAAGTTAATTCATCCAATCAAAAAGGTTTTTTTTTTGATTGGATGGGAATGAATGAAGAAATAAAAAATTGTCTTATATCCAATTTTGAACTTTGGTTCTTTCGAAAATTTGTGATACTTTACAACACATATAAGATAAAACCATGGGCGATACCCATCCAATTTCTTCTTTTCAATTTTCATAGAAATGAAAATGTTAGTAAAAATAATAAAATTAACCGGAAGAAAAATAGCGATCTTTTTATATCTATATCATCGAATGAAAAAAAAATTATTGAATTAGAGAATCAAAATCACGAAGAAAAAGAATCCGAAGACCAAGGGGACTTTGGGTCAGTTTTCGCAAATCAAGAAAAAGATATTGAAGAAGATTATATAGAATTAGATATGAAAAAACATAGAAATAAAAAGCAAAACAAAAGTCATATGGAAGTAGAACTTGATTTCTTCCTAAAACGGTATTTATGTTTTCAATTAAGATGGAATGGTTCTTTAAATCACAAAATAATCAATAATATCAAAGTATATTGTCTCCTGCTTAGACTGACAAATCCACGAGAAATTATTATATCTTCTATTCAAAGGCAAGAAATAAGTTTGAATATTCTGATGGTTCAGAAGGATTTAACTCTTACGGAATTAATGAAAAAGGGAATATTGATTATCGAACCTGTTCGTCTGTCGGTAAAAAATGATGGACAATTTATTTTGTATCAAATGGTAGGTATCCTATTAGTTCATAAGAACAAACAACAAATTAATCAAAAATACAGAGAAAATTTTTATGTTGATAAAAAGAATTTTACCGAATCTATTGAAAGACATCAAAGTATAATTGGAAATAGAAACAAAAATGATTATGATTTACTTGTTCCTGAAAATATTTTATCCCCTAAACGTCGTAGAGAATTAAGAATTCTTTTCAATTTAAAAAATCAAAATGATATTCATATAAATACAGAAATTTTCAATGGTAATAACATAAAAAATTGTAGTCCCATTTTAGATAAAAGCAAACATTTTGATAGAGATAAAAAGAAACTAATTAAATTACAATTTTTTCTTTGGCCCAATTTTCGATTAGAAGATTTAGCTTGTATGAATCGTTATTGGTTCGATACTAATAATGCTAGTCGGTTCAGTATGGTAAGAATATATATATATCCGCGGTTGAAATTTTGA

>lcl|NC_015308.1_cds_YP_004327720.1_80 [gene=rps7] [locus_tag=HebrCp128] [db_xref=GeneID:10352016] [protein=ribosomal protein S7] [protein_id=YP_004327720.1] [location=147271..147738] [gbkey=CDS]

ATGTCACGTCGAGGTACTGCAGAAGAAAAAACTGCAAAATCCGATCCAATTTATCGTAATCGATTAGTTAACATGTTGGTTAACCGTATTCTGAAACACGGAAAAAAATCATTGGCTTATCAAATTATCTATCGAGCCATGAAAAAGATTCAACAAAAGACAGAAACAAATCCACTATCTGTTTTACGTCAAGCAATACGTGGAGTAACTCCCGATATAGCAGTAAAAGCAAGACGTGTAGGCGGATCGACTCAGCAAGTTCCCATTGAAATAGGATCCACACAAGGAAAAGCACTTGCCATTCGTTGGTTATTAGGGGCATCCCGAAAACGTCCGGGTCGAAATATGGCTTTCAAATTAAGTTCCGAATTAGTGGATGCTGCCAAAGGGAGTGGTGATGCCATACGCAAAAAGGAAGAGACTCATAGAATGGCAGAGGCAAATAGAGCTTTTGCACATTTTCGTTAA

>lcl|NC_015308.1_cds_YP_004327721.1_81 [gene=ndhB] [locus_tag=HebrCp129] [db_xref=GeneID:10351948] [protein=NADH dehydrogenase subunit 2] [protein_id=YP_004327721.1] [location=join(148050..148826,149509..150264)] [gbkey=CDS]

ATGATCTGGCATGTACAGAATGAAAACTTCATTCTCGATTCTACGAGAATTTTTATGAAAGCCTTTCATTTGCTTCTCTTCGATGGAAGTTTTATTTTCCCAGAATGTATCCTAATTTTTGGCCTAATTCTTCTTCTGATGATCGATTCAACCTCTGATCAAAAAGATATACCTTGGTTATATTTCATCTCTTCAACAAGTTTAGTAATGAGTATAACGGCCCTATTGTTCCGATGGAGAGAAGAACCTATGATTAGCTTTTCGGGAAATTTCCAAACGAACAATTTCAACGAAATCTTTCAATTTCTTATTTTACTATGTTCAACTCTATGTATTCCTCTATCCGTAGAGTACATTGAATGTACAGAAATGGCTATAACAGAGTTTCTCTTATTCGTATTAACAGCTACTCTAGGAGGAATGTTTTTATGCGGTGCTAACGATTTAATAACTATCTTTGTAGCTCCAGAATGTTTCAGTTTATGCTCCTACCTATTATCTGGATATACCAAGAAAGATGTACGGTCTAATGAGGCTACTACGAAATATTTACTCATGGGTGGGGCAAGCTCTTCTATTCTGGTTCATGCTTTCTCTTGGCTATATGGTTCGTCCGGGGGAGAGATCGAGCTTCAAGAAATAGTGAATGGCCTTATCAATACACAAATGTATAACTCCCCAGGAATTTCAATTGCGCTTATATTCATCACTGTAGGAATTGGGTTCAAGCTTTCCCTAGCCCCTTCTCATCAATGGACTCCTGACGTATACGAAGGATCTCCCACTCCAGTCGTTGCTTTTCTTTCTGTTACTTCGAAAGTAGCTGCTTCAGCTTCAGCCACTCGAATTTTCGATATTCCTTTTTATTTCTCATCAAACGAATGGCATCTTCTTCTGGAAATCCTAGCTATTCTGAGCATGATAGTGGGGAATCTCATTGCTATTACTCAAACAAGCATGAAACGTATGCTTGCATATTCGTCCATAGGTCAAATCGGATATGTAATTATTGGAATAATTGTTGGAGACTCAAATGGTGGATATGCAAGCATGATAACTTATATGCTCTTCTATATCTCCATGAATCTAGGAACTTTTGCTTGTATTGTATTATTTGGTCTACGTACCGGAACTGATAACATTCGAGATTATGCAGGATTATACACGAAAGATCCTTTTTTGGCTCTCTCTTTAGCCCTATGTCTCTTATCCCTAGGAGGTCTTCCTCCACTAGCAGGTTTTTTCGGAAAACTCCATTTATTCTGGTGTGGATGGCAGGCAGGCCTATATTTCTTGGTTTTAATAGGACTCCTTACGAGCGTTGTTTCTATCTACTATTATCTAAAAATAATCAAGTTATTAATGACTGGACGAAACCAAGAAATAACCCCTCACGTGCGAAATTATAGAAGATCCCCTTTAAGATCAAACAATTCCATCGAATTGAGTATGATTGTATGTGTGATAGCATCTACTATACCAGGAATATCAATGAACCCGATTGTTGAAATTGCTCAAGATACCCTTTTTTAG

>lcl|NC_015308.1_cds_YP_004327722.1_82 [gene=ycf2] [locus_tag=HebrCp132] [db_xref=GeneID:10352018] [protein=hypothetical chloroplast RF2] [protein_id=YP_004327722.1] [location=complement(151983..158894)] [gbkey=CDS]

ATGAAAGGACATCAATTCAAATCCTGGATTTTCGAATTGAGAGAGATATTGAGAGAGATCAAGAATTCTCACTATTTCTTAGATTCATGGACCCAATTCAATTCAGTGGGATCTTTCATTCACATTTTTTTCCATCAAGAACGTTTTATAAAACTCTTGGACTCCCGAATTTGGAGTATCTTACTTTCACGCAATTCACAGGGTTCAACAAGCAATCGATATTTCACGATCAAGGGTGTAGTACTATTTGTAGTAGTGGTCCTTATATATCGTATTAACAATCGAAAGATGGTCGAAAGAAAAAATCTCTATTTGACAGGGCTTCTTCCTATACCTATGAATTCCATTGGACCCAGAAATGATACATTGGAAGAATCCTTTGGGTCTTCCAATATCAATAGGTTGATTGTTTCGCTCCTGTATCTTCCAAAAGGAAAAAAGATCTCTGAGAGCTCTTTCCTGGATCCGAAAGAGAGTACTTGGGTTCTCCCAATAACTAAAAAGTGTATCATGTCTGAATCTAACTGGGGTTCGCGGTGGTGGAGGAACTGGATCGGAAAAAAGAGGGATTCTAGTTGTAAGATATCTAATGAAACCGTTGCTGGAATTGAGATCTCATTCAAAGAAAAAGATATCAAATATCTGGAGTTTCTTTTTGTATATTATATGGATGATCCGATCCGCAAGGACCATGATTGGGAATTGTTTGATCGTCTTTCTCCGAGGAAGGGGCGAAACATAATCAACTTGAATTCGGGACAGCTATTCGAAATCTTAGTGAAAGACTGGATTTGTTATCTCATGTTTGCTTTTCGTGAAAAAATACCAATTGAAGTGGAGGGTTTCTTCAAACAACAAGGAGCTGGGTCAACTATTCAATCAAATGATATTGAGCATGTTTCCCATCTCTTCTCGAGAAAGAAGTGGGCTATTTCTTTGCAAAATTGTGCTCAATTTCATATGTGGCAATTCCGCCAAGATCTCTTCGTTAGTTGGGGGAATAATCCGCACGAATCGGATTTTTTGAGTAACATATCGAGGATTTGGTTAGACAATGTGTGGTTGGTAAACAAGGATCGGTTTTTTAGCAAGGCACGGAATATATCGTCAAATATTCAATATGATTCCACAAGATCTAGTTTCGTTCAAGGAAGGAATTCTAGCCAATTGAAGGGATCTTCTGATCAATCCAGAGATCATTTCGATTCCATTAGTAATGAGGATTCGGAATATCACACATTGATCAATCAAAGAAAGATTCAACAACTAAAAGAAAGATCGATTCTTTGGGATCCTTCCTTTCTTCAAACGGAACGAACAGAGATAGAATCAGACCGATTCCCTAAATGCCTTTCTGGATATTCCTCAATGTCCCGGCTATTCACGGAAGGTGAGAAGGAGATGAATAATCATCTGCTTCCGGAAGAAATCGAAGAATTTCTTGGGAATCCTACAAGATCCATTCGTTCTTTTTTCTCTGACAGATCGTCAGAACTTTATCTGGGTTCGAATCCTACTGAGAGGTCCACTAGAGATCAGAAATTGTTGAAGAAAGAACAAGATGTTTCTTTTGTCCCTTCCAGGCGATCGGAAAATAAAGAAATAGTTAATATATTCAAGATAATCACGTATTTACAAAATACCGTCTCAATTCATCCTATTTCATCAGATCCGGGATGTGATATGGTTCTGAAGGATGAACTGGATATGGACAGTTCCAATAAGATTTCTTTCTTGAACAAAAATCCATTTTTTGATTTATTTCATCTATTCCATGATCGGAACGGGGGGGGATACACGTTACACCACGATTTTGAATCAGAAGAGAGATTTCAAGAAATGGCAGATCTATTCACTCTATCAATAACCGAGCCGGATCTGGTGTATCATAAGGGATTTACCTTTTTTATTGATTCCTACGGATTGGATCAAAAACAATTCTTGAATGAGGTATTCAACTCCAGGGATGAATCGAAAAAGAAATCTTTATTGGTTCTACCTCCTATTTTTTATGAAGAGAATGAATCTTTTTATCGAAGGATCAGAAAAAAATGGGTCCGGATCTCCTGCGGGAATGATTTGGAAGATCCAAAACAAAAAATAGTGGTATTTGCTAGCAACAACATAATGGAGGCAGTCAATCAATATGGATTGATCCTAAATCTGATTCAAATCCAATATAGTACCTATGGGTACATAAGAAATGTATTGACTCAATTCTTTTTAATGAATAGATCCGATCGCAACTTCGAATATGGAATTCAAAGGGATCAAATAGGAAATGATACTCTGAATCATAGAACTATAATGAAATATACGATCAACCAACATTTATCGAATTTGAAACAGAGTCAGAAGAAATGGTTCGATCCTCTTATTTTTCTTTCTCGAACCGAGAGATCCATGAATTGGGATCCTAATGCATATAGATACAAATGGTCTAATGGGAGCAAGAATTTCCAGGAACATTTGGAACATTTCATTTCTGAGCAGAAGAGCCGTTTTCTTTTTCAAGTAGTGTTCGATCGATTACGTATTAATCAATATTCGATTGATTGGTCTGAGGTTATCGACAAAAAAGATTTGTCTAAGTCACTTCGTTTCTTTTTGTCCAAGTTACTTCTTTTTTTGTCCAAGTTTCTTCTCTTTTTGTCTAACTCACTTCCTTTTTTCTTTGTGAGTTTCGGGAATATCCCCATTCATAGGTCCGAAATCCATATCTATGAATTGAAAGGTCCGAATGATCAACTCTGCAATCAGCTGGTAGAACCAATAGGTCTTCAAATCGTTCATTTGAAAAAATTGAAACCCTTCTTATTGTTATTGGATGATCATGATACTTCCCAAAAATCTAAATTTTTGATTAATGGAGGAACAATATCACCATTTTTGTTCAATAAGATAACAAAGTGGATGATTGACTCATTCCATACTAGAAATAATCGCAGGAAATCTTTTGATAACACGGATTCCTATTTCTCAATGATATCCCACGATCAAGACAATTGGCTGAATCCCGTGAAACCATTTCATAGAAGTTCATTGATATCTTCTTTTTATAAAGCAAATCGACTTCGATTCTTGAATAATCTACATCACTTCTGCTTCTATTGTAACAAAAGATTCCCTTTTTATGTGGAAAAGGCCCGTATCAAGAATTATGATTTTACGTATGGACAATTCCTCAATATCTTGTTCATTCGCAACAAAATATTTTCTTTGTGCGGCGGTAAAAAAAAACATGCTTTTTTGGAGAGAGATACTATTTCACCAATCGAGTCACAGGTATCTAACATATTCATACCTAATGATTTTCCACAAAGTGGTAACGAAAGGTATAACTTGTACAAATCTTTCCATTTTCCAATTCGATCCGATCCATTCGTTCGTAGAGCTATTTATTCGATCGCAGACATTTCTGGAACACCTCTAACAGAGGGACAAATAGTCAATTTTGAAAGAACTTATTGTCAACCTCTTTCGGATATGAATCTATCTGATTCAGAAGGGAAGAACTTGCATCAGTATCTCAATTTCAATTCAAACATGGGTTTGATTCACACTCCATGTTCTGAGAAATATTTACCATCCGAAAAGAGGAAAAAACGGAGTCTTTGTCTAAAGAAATGTGTTGAAAAAGGGCAGATGTATAGAACCTTTCAACGAGATAATGCTTTTTCAACTCTCTCAAAATGGAATCTATTCCAAACATATATGCCATGGTTCCTTACTTCGACGGGGTACAAATATCTAAATTTGATATTTTTAGATACCTTTTCGGACCTATTACCGATACTAAGTAGCAGTCAAAAATTTGTATCCATTTTTCATGATATTATGCATGGATCAGATATATCATGGCGAATTCTTCAGAAAAAATTGTGTCTTCCACAATGGAATCTGATAAGTGAGATTTCGAGTAAGTGTTTACATAATCTTCTTCTGTCCGAAGAAATGATTCATCGAAATAATGAGCCACCATTGATATCGACACATCTGAGATCGCCAAATGTTCGGGAGTTCCTCTATTCAATCCTTTTCCTTCTTCTTGTTGCTGGATATCTCGTTCGTACACATCTTTTCTTTGTTTCCCGAGCCTATAGTGAGTTACAGACAGAGTTCGAAAAGGTCAAATCTTTGATGATTCCATCATACATGATTGAGTTGCGAAAACTTCTGGATAGGTATCCTACATCTGAACTGAATTCTTTCTGGTTAAAGAATCTCTTTCTAGTTGCTCTGGAACAATTAGGAGATTTTCTAGAAGAAATGCGGGGTTCTGCTTCTGGCGGCAACATGCTATGGGGTGGTGGTCCCGCTTATGGGGTTAAATCAATACGTTCTAAGAAGAAATTTTTTAATATCAATCTCATCGATCTCATAAGTATCATACCAAATCCCATCAATCGAATCACTTTTTCGAGAAATACGAGACATCTAAGTCATACAAGTAAAGAGATTTATTCATTGATAAGAAAAAGAAAAAACGTGAACGGTGATTGGATTGATGATAAAATAGAATCCTTGGTCGCGAACAGTGATTCGATTGATGATAAAGAAAGAGAATTCTTGGTTCAGTTCTCCACCTTAACGACAGAAAAAAGGATTGATCAAATTCTATTGAGTCTGACTCATAGTGATCATTTATCAAAGAATGACTCTGGTTATCAAATGATTGAAGAGCCGGGAGCAATTTATTTACGATACTTAGTTGACATTCATAAAAAGTATCTAATGAATTATGAGTTCAACACACCCTGTTTAGTAGAAAGACGGATATTCCTTGCTTATTATCAGACAACCACTTATTCACAAACCTCGTGTGGGGTGAATAGTTTTCATTTCCCATCTCATGGAAAACCCTTTTCGCTCCGCTTAGCCCTATCCCCCTCTAGGGGTATTTTAGTGATAGGTTCTATAGGAACTGGACGATCCTATTTGGTCAAATACCTAGCGACAAACTCCTATCTTCCTTTCATTACAGTATTTCTGAACAAGTTCCTGGATAACAAGCCTAAGGGTTTTCTTATTGATGATAGTGACGATATTGATGATAGTGACGATATTGATGATAGTGACGATATTGATGATAGTGACGATATTGATGTGAGTGACGATATTGATGTGAGTGACGATATTGACCGTGACTTTGATACGGAGCTGGAGTTTCTAACTAGGATGAATGTGCTAACTATGGATATGATGCCGGAAATAGACCGATTTTATATCACCCTTCAATTCGAATTAGCAAAAGCAATGTCTCCTTGCATAATATGGATTCCAAACATTCATGATCTGGATGTGAATGAGTCGAATTACTTATCCCTCGGTCTATTAGTGAACTATCTCTCCAGGGATTGTGAAAGATGTTCCACTAGAAATATTCTTGTTATTGCTTCGACTCATATTCCCCAAAAAGTGGATCCCGCTCTAATAGCTCCGAATAAATTAAATACATGCATTAAGATACGAAGGCTTCTTATTCCACAACAACGAAAGCACTTTTTTACTCTTTCATATACTAGGGGATTTCACTTGGAAAATAAAATGTTCCATACTAATGGATTCGGGTCCATAACCATGGGTTCCAATGTACGAGATCTTGTAGCACTTACCAATGAGGCCCTATCGATTAGTATTACACAGAAGAAATCAATTATAGACACTAATATAATTAGATCTGCTCTTCATAGACAAACTTGGGATTTGCGATCCCAGGTAAGATCGGTTCAGGATCATGGGATCCTTTTCTATCAGATAGGAAGGGCTGTTGCACAAAATGTATTTCTAAGTAATTGCCCCATAGATCCTATATCTATCTATATGAAGAAGAAATCATGTAACGAAGGGGATTCTTATTTGTACAAATGGTACTTCGAACTTGGAACGAGCATGAAGAAATTAACGATACTTCTTTATCTTTTGAGTTGTTCTGCCGGATCGGTTGCTCAAGACCTTTGGTCTCTACCCGGACCCGATGAAAAAAATGGGATCACTTATTATGGACTTGTTGAGAATGATTCTGATCTAGTTCATGGCCTATTAGAAGTAGAAGGCGCTCTGGTGGGATCCTCACGGACAGAAAAAGATTGCAGTCAGTTTGATAATGATCGAGTGACATTGCTTCTTCGGCCCGAACCAAGGAGTCCCTTAGATATGATGCAAAATGGATCTTGTTCTATCCTTGATCAGAGATTTCTCTATGAAAAATACGAATCGGAGTTTGAAGAAGGGGAAGGAGAAGAAGTCCTCGACCCGCAACAGATAGAGGAGGATTTATTCAATCACATAGTTTGGGCTCCTAGAATATGGCGCCCTTGGGGTTTTCTATTTGATTGTATCGAAAGGCCCAATGAATTGGGATTTCCCTATTGGGCCAGGTCATTTCGGGGCAAGCGGATCATTTATGATGAAGAGGATGAGCTTCAAGAGAATGATTCGGAGTTCTTGCAGAGTGGAACCATGCAGTACCAGATACGAGATAGATCTTCCAAAGAACAAGGCTTTTTTCGAATAAGCCAATTCATTTGGGACCCTGCGGATCCACTCTTTTTCCTATTCAAAGATCAGCCCTTTGTCTCTGTGTTTTCACATCGAGAATTCTTTGCAGATGAAGAGATGTCAAAGGGGCTTCTTACTTCCCAAACAGATCCTCCTACATCTATATATAAACGCTGGTTTATCAAGAATATGCAAGAAAAGCACTTCGAATTGTTGATTCATCGCCAGAGATGGCTTAGAACCAATAGTTCATTATCTAATGGATTTTTCCGTTCTAATACTCTATCCGAGAGTTATCAGTATTTATCAAATCTGTTCCTATCTAACGGAACGCTATTGGATCAAATGACAAAGGCATTGTTGAGAAAAAGATGGCTTTTCCCGGATGAAATGAAAATTGGATTCATGTAA

>lcl|NC_015308.1_cds_YP_004327723.1_83 [gene=rpl23] [locus_tag=HebrCp134] [db_xref=GeneID:10352020] [protein=ribosomal protein L23] [protein_id=YP_004327723.1] [location=159222..159503] [gbkey=CDS]

ATGGATGGAATCAAATATGCAGTATTTACAGACAAAAGTATTCGGTTATTGGTGAAAAATCAATATACTTTTAATGTCGAATCAGGATCAACTAGGACAGAAATAAAGCATTGGGTCGAACTCTTCTTTGGTGTCAAGGTAATAGCTATGAATAGCCATCGACTCCCGGGAAAGGGTAGAAGAATGAGACCTATTATGGGACATACAATGCATTACAGACGTATGATCATTACGCTTCAACCGGGTTATTCTATTCCACCTCTTAGAAAGAAAAGAACTTAA

>lcl|NC_015308.1_cds_YP_004327724.1_84 [gene=rpl2] [locus_tag=HebrCp135] [db_xref=GeneID:10351946] [protein=ribosomal protein L2] [protein_id=YP_004327724.1] [location=join(159522..159920,160594..161028)] [gbkey=CDS]

ATGGCGATACATTTATACAAAACTTCTACCCCGAGCACACGCAATGGAGCCGTAGACAGTCAAGCGAAATCCAATACACGAAATACACGAAAGAATTTGATCTATGGACAGCATCGTTGTGGTAAAGGCCGTAATGCCAGAGGAATCATTACCGCAAGACATAGAGGGGGAGGTCATAAGCGTCTATACCGTAAAATCGATTTTCGACGGAATGAAAAAGACATATATGGTAGAATCGTAACCATAGAATACGACCCTAATCGAAATGCATACATTTGTCTCATACACTATGGGGATGGTGAGAAGAGATATATTTTACATCCCAGAGGGGCTATAATTGGAGATACCGTTATTTCTGGTACAGAAGTTCCTATAAAAATGGGAAATGCCCTACCTTTGACCGATATGCCCTTAGGCACGGCCATACATAACATAGAAATCACACTTGGAAAGGGTGGACAATTAGCTAGAGCTGCAGGTGCTGTAGCGAAACTGATTGCAAAAGAGGGGAAATCAGCCACATTAAAATTACCTTCGGGGGAGGTTCGTTTAATATCCAAAAACTGCTCAGCAACAGTCGGACAAGTAGGGAATACTGGGGTGAACCAGAAAAATTTGGGTAGAGCCGGATCTAAATGTTGGCTAGGTAAGCGTCCTGTAGTAAGAGGAGTAGTTATGAACCCTGTAGACCATCCCCATGGGGGTGGTGAAGGGAGGGCCCCAATTGGTAGAAAAAAACCCGCAACCCCTTGGGGTTATCCTGCACTTGGAAGAAGAAGTAGAAAAAGGAATAAATATAGTGATAATTTGATTCTTCGTCGCCGTAGTAAATAG

**3. *Jatropha curcas***

>lcl|NC_012224.1_cds_YP_002720093.1_1 [gene=psbA] [locus_tag=JacuC_p001] [db_xref=GeneID:7564824] [protein=photosystem II protein D1] [protein_id=YP_002720093.1] [location=complement(499..1560)] [gbkey=CDS]

ATGACTGCAATTTTAGAGAGACGCGAAAGCGAAAGCCTATGGGGTCGTTTCTGTAACTGGATAACCAGCACTGAAAACCGTCTTTACATTGGATGGTTTGGTGTTTTGATGATCCCTACTTTATTGACCGCAACTTCTGTATTTATTATCGCTTTCATTGCTGCACCTCCGGTAGATATTGATGGTATTCGTGAACCTGTTTCTGGGTCTCTACTTTATGGAAACAATATTATTTCTGGTGCCATTATTCCTACTTCTGCGGCTATAGGTTTGCATTTTTACCCAATATGGGAAGCGGCATCCGTTGATGAATGGTTATACAATGGCGGTCCTTATGAGCTAATTGTTCTACATTTCTTACTTGGTGTAGCTTGTTACATGGGTCGTGAGTGGGAACTTAGTTTCCGTCTGGGTATGCGCCCTTGGATTGCTGTTGCATATTCAGCTCCTGTTGCAGCTGCTACTGCTGTTTTCTTGATCTATCCAATTGGTCAAGGAAGCTTTTCTGATGGTATGCCTCTAGGAATCTCTGGTACTTTCAACTTTATGATTGTATTCCAGGCTGAGCACAACATCCTTATGCACCCATTTCACATGTTAGGCGTAGCTGGTGTATTCGGCGGTTCCCTATTCAGTGCTATGCATGGTTCCTTGGTAACCTCTAGTTTGATCAGGGAAACCACAGAAAATGAATCTGCTAATGAAGGTTACAGATTCGGTCAAGAGGAAGAAACTTATAATATCGTAGCCGCTCATGGTTATTTTGGCCGATTGATCTTCCAATATGCTAGTTTCAACAATTCTCGTTCTTTACATTTCTTCTTAGCTGCTTGGCCTGTAGTAGGTATTTGGTTCACTGCTTTAGGTATTAGCACTATGGCTTTCAACCTAAATGGTTTCAATTTCAACCAATCCGTAGTTGATAGTCAAGGTCGTGTAATTAATACCTGGGCTGATATTATTAACCGTGCTAACCTTGGTATGGAAGTTATGCATGAACGTAATGCTCATAACTTCCCTCTAGACCTAGCTGCTATTGAAGCTCCATCTACAAATGGATAA

>lcl|NC_012224.1_cds_YP_002720094.1_2 [gene=matK] [locus_tag=JacuC_p002] [db_xref=GeneID:7564826] [protein=maturase K] [protein_id=YP_002720094.1] [location=complement(2287..3816)] [gbkey=CDS]

ATGTGGAAATATCAAAGATATTTAGAACTAGATGGATCTCGAAAAAATGACCTCCTATACCCATTTATCTTTCGGGAGTATATTTATATATTTGCTCATGATCATAGTTTAAATAGATCGATTTTGTTGGAAAATGTAGGTTATGACAATAAATCTAGTTTCTTAATTGTAAAACGTTTAATTACTCGAATGTATCAACAGAATCGTTTGGTTTTTTTTTCTTTTTCTGCTAATGATTCTAACCAAAATTCATTTTTTAAGTACAACAAGAATTTGTATTATCAAATGATATCAGAGAGCTTTGCAGTTATTGTGGAAATTCCATTTTCTCTACGGTTAGTATCTTCTTTAGAAAGGTCGGAGATAGTTAAATCTCATAAATTACGATCAATTCATTCAATATTTCCTTTTTTAGAGGACAAATTTCCACATTTAATTTATGTGTCAGATGTATTAATACCTTACCCCATCCATATAGAAAAATTAGTCCAAACCCTTCGCTATTGGATGAAAGATCCCTCTTCTTTGCATTTATTACGACTCTTTCTTCATGAATATTGGAATAGGAACAGTCTTATTATTCAAAAGGGATCTATTTCTATTTTTACAAAAAGTAATCCAAGATTTTTCTTGTTCCTATATAATTCTCATGTATATGAATACGAATCAATCCTCTTTTTTCTTCGTAACCAATCCTTTCATTTACGATCAACATTTTCTCGAGTCCTTCTTGAACGAATTTATTTCTATGGAAAAATAGAACATTTTGCAGAAGTCTTTGCTAATGATTTTCAGACTATCCTATGGTTGGTCAAGTATCCTTTCATGCATTATGTTAGATATCAAGGAAAATCCATTCTGGCTTCAAAAGATGGGCCTCTTCTGATGAAAAAATGGAAATATTACCTTGTCAATTTATGTCAATGTCATTTTTATGTGTGGTTTCAACCAGAAAAGATCTATATAAATTCATTATCCAAGCATTCTCTCAACCTTTTGGGCTATCTTTCAAATGTAAAATTAAATCCTTCGGTCGTACGAAGTCAAATGCTAGAAAATTCATTTCTAATAGATAAAGATAATACTATGAAGAAACTCGATACAATAGTTCCAATTATTCCTTTGATTGGATCATTGTCAAAAACGAAATTTTGTAAGGCAGTAGGACATCCCATTAGTAAACCGGTCCGGACTGATTCATCGGATTCTGATATTATCGACCGATTTGTGTGTATATGCAGAAATCTTTCTCATTATTATAGTGGATCTTCAATAAAAAAGAGTTTGTATCGAGTAAAATATATACTTCGACTTTCTTGTGTTAAAACTTTGATTCGTAAACACAAAAGTACTGTACGCGCTTTTTTGAAAAGATTGGGTTCGGAATTATTAGAAGAATTTTTTACAGAAGAAGAACAAACTCTTTCTTTGATCTTCCCGAGAGTTTCCTCTATTTCGCGCAGGTTATATAGGGGGCGGGTTTGGTATTTGGATATTATTTCTATCAATGATTTGGCCAATCATGAATAA

>lcl|NC_012224.1_cds_YP_002720095.1_3 [gene=psbK] [locus_tag=JacuC_p003] [db_xref=GeneID:7564828] [protein=photosystem II protein K] [protein_id=YP_002720095.1] [location=6684..6878] [gbkey=CDS]

ATGTTTGTTATGCTTAATATTTTTAGTTTAATTTGCATCTGTCTTAATTCTGCCCTTTATTCAAGCAATTTTTTCTTCACAAAATTGCCCGAGGCCTATGCCTTTTTGAATCCAATCGTAGATTTTATGCCAGTAATCCCTGTACTCTTTTTTCTATTAGCCTTTGTTTGGCAAGCTGCTGTAAGTTTTCGATGA

>lcl|NC_012224.1_cds_YP_002720096.1_4 [gene=psbI] [locus_tag=JacuC_p004] [db_xref=GeneID:7564829] [protein=photosystem II protein I] [protein_id=YP_002720096.1] [location=7299..7409] [gbkey=CDS]

ATGCTTACTCTCAAACTCTTTGTTTACACAGTAGTGATATTCTTTGTTTCTCTCTTCATCTTTGGATTTTTATCTAATGATCCAGGACGAAATCCTGGACGTGAAGAATAA

>lcl|NC_012224.1_cds_YP_002720097.1_5 [gene=atpA] [locus_tag=JacuC_p005] [db_xref=GeneID:7564833] [protein=ATP synthase CF1 alpha subunit] [protein_id=YP_002720097.1] [location=complement(11147..12670)] [gbkey=CDS]

ATGGTAACTATTCGAGCCGACGAGATTAGTAATATTATCCGCGAACGTATTGAACAATATAATAGGGAAGTAAAGATTGTAAATACCGGTACTGTACTTCAAGTAGGCGACGGTATTGCTCGTATTCATGGTCTTGATGAAGTAATGGCAGGGGAATTAGTAGAGTTTGAAGAGGGTACAATAGGCATTGCTCTGAATTTGGAATCAAATAATGTCGGTGTTGTATTAATGGGTGACGGTTTAATGATACAAGAGGGAAGCTCCGTAAAAGCAACAGGAAGAATTGCTCAGATACCGGTGAGTGAGGCTTATTTGGGTCGTGTTATAAATGCTTTGGCTAAACCTATTGACGGTCGAGGTGAAATTTCCGCTTCTGAATCTCGGTTAATTGAATCTCCTGCTCCAGGGATTATTTCTAGACGTTCCGTATATGAGCCTCTTCAAACAGGACTTATTGCTATTGATTCGATGATTCCTATAGGACGCGGTCAACGAGAATTAATTATTGGGGATAGACAGACCGGTAAAACAGCAGTAGCCACAGATACAATTCTCAATCAACAAGGACAAAATGTAATATGTGTTTATGTAGCTATTGGGCAAAAAGCGTCTTCTGTGGCTCAGGTAGTGACTACTTTACAGGAAAGAGGAGCAATGGAGTACACTATTGTGGTAGCCGAAACAGCGGATTCCCCGGCTACATTACAATATCTCGCTCCTTATACAGGAGCAGCTCTGGCTGAATATTTTATGTACCGGGAACGACACACTTTAATCATTTATGATGATCTCTCCAAACAAGCGCAGGCTTATCGCCAAATGTCTCTTCTATTACGAAGACCACCGGGTCGTGAAGCTTATCCAGGAGATGTCTTTTATTTGCATTCACGCCTTTTGGAAAGAGCCGCTAAATCAAGTTCTCGTTTAGGTGAAGGAAGTATGACTGCTTTACCAATAGTTGAGACCCAATCAGGAGACGTTTCGGCTTATATTCCTACTAATGTAATTTCCATTACAGATGGACAAATATTCTTATCCGCCGATCTATTCAATGCTGGAATCAGACCCGCTATTAATGTGGGTATTTCCGTTTCCAGAGTAGGATCTGCGGCTCAAATTAAAGCTATGAAACAGGTAGCTGGTAAGTTAAAATTGGAATTGGCACAATTCGCAGAATTAGAAGCCTTTGCGCAATTCGCTTCTGATCTCGATAAAGCTACTCAGAATCAATTGGCAAGAGGTCAACGATTACGCGAGTTGCTCAAACAATCCCAATCCGCTCCTCTCACAGTGGAGGAACAGATAATGACTATTTATGCCGGAACGACGGGTTATCTTGATTCATTAGAAGTTGGACAAATAAAGAAATTTCTCGTTGAGTTACGTACTTACTTAAAAACGAATAAACCTGAGTTCCGAGAAATAATATCTTCTACTAAAACATTCACCGAAGAAGCAGAAAGCCTTTTGAAAGAAGCTATTCAGGAACTGAAGGAGCGTTTTCTTCTTCAGGAACAAGTATAA

>lcl|NC_012224.1_cds_YP_002720098.1_6 [gene=atpF] [locus_tag=JacuC_p006] [db_xref=GeneID:7564834] [protein=ATP synthase CF0 B subunit] [protein_id=YP_002720098.1] [location=complement(join(12729..13082,13808..13966))] [gbkey=CDS]

ATGAAAAACGTAACCGATTCTTTCGTTTCCTTGGGTCACTGGCCATCCGCCGGGAGTTTCGGGTTTAATACCGATATTTTAGCAACAAATCCAATAAATCTAAGTGTAGTCCTTGGTGTATTAATTTTTTTTGGAAAGGGGGTGTGTGCGAGTTGTTTAGATAATCGAAAACAAAGGATTTTGGATACTATTCGAAATTCAGAAGAACTACGTGAGGGGGCCATTGAACGGCTGGAAAAAGCCCGGGCCCGCTTACGGAAAGTGGAAATAGAAGCAGATCAGTTTCGAATGAATGGATACTCTGAGATAGAACGAGAAAAATCGAATTTGATTAATTCAACTTCTAAGACTTTAGAACAATTAGAAAATTACAAAAACGAAACCATTCATTTTGAACAACAAAGAACGATTAATCAAGTCCGACAACGGGTTTTCCAACAAGCCTTACAAGGAGCTCTAGGAACTCTGAATAGTTCTTTGACCAACGAGTTACATTTACGTACCATCAATTAG

>lcl|NC_012224.1_cds_YP_002720099.1_7 [gene=atpH] [locus_tag=JacuC_p007] [db_xref=GeneID:7564835] [protein=ATP synthase CF0 C subunit] [protein_id=YP_002720099.1] [location=complement(14657..14902)] [gbkey=CDS]

ATGAATCCATTGATTTCTGCCGCTTCCGTTATTGCTGCTGGGTTGGCTGTTGGGCTTGCTTCTATTGGACCTGGGGTTGGTCAAGGTACTGCCGCGGGCCAAGCTGTAGAAGGTATCGCAAGACAACCCGAGGCGGAGGGAAAAATACGAGGTACTTTATTGCTTAGTCTGGCTTTTATGGAAGCTTTAACAATTTATGGACTGGTTGTAGCATTAGCACTTTTATTTGCGAATCCTTTTGTTTAA

>lcl|NC_012224.1_cds_YP_002720100.1_8 [gene=atpI] [locus_tag=JacuC_p008] [db_xref=GeneID:7564836] [protein=ATP synthase CF0 A subunit] [protein_id=YP_002720100.1] [location=complement(16131..16874)] [gbkey=CDS]

ATGAATGTTCTATCATGTTCCATAAACACACTAAAAGGGTTATATGATATATCCGGTGTGGAAGTAGGCCAGCATTTCTATTGGAAAATAGGAGGTTTCCAAGTCCATGCCCAAGTACTTATTACTTCTTGGGTTGTAATTACTATCTTATTAGGTTCAGCCATTGTAGCTGTTCGGAATCCACAAACCATTCCGACTGGCGGGCAGAATTTCTTCGAATATGTCCTTGAATTCATTCGGGATGTGAGCAAAACTCAGATTGGAGAGGAATATGGCTCATGGGTCCCCTTTATTGGAACTATGTTTTTATTTATTTTTGTTTCTAATTGGGCTGGGGCGCTTTTGCCTTGGAAGATCATAGAGTTACCTCATGGGGAGTTAGCCGCACCTACGAATGATATAAATACTACCGTTGCTTTAGCTTTACTTACGTCAATAGCATATTTTTATGCGGGCCTTAGGAAAAAAGGATTAGGTTATTTCAGCAAATACATTCAGCCAACTCCAATCCTTTTACCCATTAACATTTTAGAAGATTTCACAAAACCTTTATCACTTAGCTTTCGACTTTTCGGAAATATATTAGCGGACGAATTAGTAGTTGTTGTTCTTGTTTCTTTAGTACCTTCAGTGGTTCCTATACCTGTCATGTTCCTTGGATTATTTACAAGCGGTATTCAAGCTCTTATTTTTGCAACTTTAGCCGCGGCTTATATAGGCGAATCCATGGAGGGGCATCATTGA

>lcl|NC_012224.1_cds_YP_002720101.1_9 [gene=rps2] [locus_tag=JacuC_p009] [db_xref=GeneID:7564837] [protein=ribosomal protein S2] [protein_id=YP_002720101.1] [location=complement(17086..17796)] [gbkey=CDS]

ATGATAAGAAGATATTGGAACATTAATTTGGAAGAGATGATAAAAGCAGGAGTTCATTTTGGTCATGGTACTAGAAAATGGAATCCGAGAATGGCACCTTATATCTCTGCAAAGCGTAAAGGTATTCATATTACAAATCTTACTAGAACTGCTCGTTTTTTATCAGAAGCTTGTGATTTAGTTTTTGATGCAGCAAGCAGGAGAAAACAATTCTTAATTGTTGGTACAAAAAATAAAGCAGCGGATTCAGTAGCGCGGGCTGCAATAAGGGCTCGGTGTCATTATGTTAATAAAAAATGGCTTGGCGGTATTTTAACGAATTGGTCCACTACAGAAACTAGACTTCAAAAGTTCAGGGACTTGAGAATGGAACAAAAGGCAGGTAGACTCAACCGTCTTCCGAAAGGAGATGCGGCTCGATTGAAGAGACAGTTAGCTCACTTGCAAACATATCTGGGCGGGATTAAATATATGACGGGGTTACCGGATGTTGTAATAATCGTTGATCAGCAAGAGGAACATACGGCTCTTCGGGAATGTATCACTTTGGGAATTCCAACGATTTGTTTAATTGATACAAACTGTGATCCGGATCTCGCAGATATTTCGATTCCAGCGAATGATGACGCTATAGCTTCAATCCGATTAATTCTTAATAAATTAGTATTTGCAATTTGTGAAGGGCGTTCTAGCTATATACGAAATCCCTGA

>lcl|NC_012224.1_cds_YP_002720102.1_10 [gene=rpoC2] [locus_tag=JacuC_p010] [db_xref=GeneID:7564838] [protein=RNA polymerase beta'' subunit] [protein_id=YP_002720102.1] [location=complement(18069..22253)] [gbkey=CDS]

ATGGAGGTGCTTATGGTCGAACGGGCCAGTCTGGTCTTTCACAATAAAGTGATAGATGGAACTGCCATTAAACGACTTATTAGCAGATTAATAGATCATTTTGGAATGGCATATACATCACACATCCTGGATCAAGTAAAGACTCTGGGTTTCCAGCAAGCCACTGCTACATCCATTTCATTAGGAATTGATGATCTTTTAACAATACCTTCTAAGGGATGGCTAGTCCAAGATGCTGAACAACAAAGTTTGATTTTGGAAAAACATTATCATTATGGAAATGTACACGTGGTAGAAAAATTACGCCAATCTATTGAGGTATGGTATGCTACAAGTGAATATTTGCGACAAGAAATGAATCTTAATTTTAGGATGACGGAACCCTTTAATCCAGTTCATATAATGTCTTTTTCGGGAGCTAGGGGAAATGCATCTCAAGTACACCAATTAGTAGGTATGAGAGGATTAATGTCGGATCCACAAGGACAAATGATTGATTTACCCATTCAAAGCAATTTACGTGAAGGACTGTCTTTAACAGAATATATCATTTCTTGCTATGGAGCCCGAAAAGGGGTTGTCGATACTGCTGTACGAACATCAGATGCTGGATATCTTACACGTAGACTTGTTGAAGTAGTTCAACATATTGTTGTGCGTAGAACAGATTGTGGCACCACCCGAGGGATCTCCGTGAGTCCTCGAAATGGGACGATGTCGGAAAGGATTTTTATTCAAACATTAATTGGTCGTGTATTAGCAGACAATATATATATGGGTTTACGATGCATTGCCGTTCAAAATCAAGATATTGGGATTGGACTTGCCAATCGATTCATAACCTTTCGAACACAAACAATATCTATTCGAACTCCCTTTACTTGTAGGAGTATGTCTTGGATCTGTCGATTATGTTATGGTCGGAGTCCTACTCATGGCGATCTAGTGGAATTGGGAGAAGCCGTAGGTATTATTGCAGGTCAATCCATTGGAGAGCCGGGTACTCAACTAACATTAAGAACGTTTCATACCGGCGGAGTATTCACAGGGGGTACTGCAGAACATGTGCGAGCCCCCTCTAATGGAAAAATCAAATTTAATGAGGATTTTGTTCATCCCATACGTACACGTCATGGGCATCCTGCTTTTCTATGTTATATAGACTTGTATGTAACTATTGAGAGTCAAGATATTATACATAACGTGACTATTCCACCAAAAAGTTTCCTTTTAGTTCAAAATGATCAATATGTAGAATCAGAACAAGTGATTGCTGAAATTCGGGCGGGAACATACACTTTGAATTTTAAAGAGAAGGTTCGAAAACATATTTATTCCGATTCAGAAGGGGAAATGCACTGGAGTTCTGATGTATACCACGCACCTGAATTTACATATAGTAATGTCCATCTCTTACCAAAAACAAGCCATTTATGGATATTATCGGGAAGTTCGTGCAGATCCAGTATAGTTCCTTTTTCACTACACAAGGATCAAGATCAAATGAACGTTCATTCTATTTCTGTCAAAAGAAGATATATTTCTAGTCTTTCTGTAAATAATGATCAAGTTAAACACAAATTCATTAGTTCAGATTTTTCGGGTAAAAAAGAAAGTAAGATTCCTGATTATTCAGAACTTAATCGAATCATATGTACTGGTCATTGTAATCTCATATATTCTGCTATTCTCTACGAGAATTCTGATTTATTGGCAAAGAGGCGAAGAAATAAATTCATCATCCCATTCCAATCAATTCAAGAACGAGAGAAAGAACTAATGACCCACTCCGCTATCTCGATTGAAATACCTAGAAATGGTATTTTCCGTAGAAATAGTGTTTTTGCTTATTTCGACGATCCCCAATACCGAAGAAAGAGTTCAGGAATTACTAAATCAGGAATTACTAAATATGGGACTATAGGAGTGCATTCAATCGTCAAAAAAGAGGATTTGATTGAGTATCGGGGAGTCAAAGAATTTAAGCCAAAATACCAAATGAAAGTGGATCGCTTTTTTTTCATTCCCGAGGAAGTGTATATTTTCCCCGAATCTTCTTCCCTAATGGTACGGAACAATAGTATCATTGGAGTAGATACACAAATTGCTTTAAATACAAGAAGTCGAGTGGGCGGGTTGGTCCGAGTGGAGAGAAAAAAAAAAAAAATGGAACTTAAAATCTTTTCTGGAGATATCCATTTTCCGGGAGAGACAGATAAGATATCCCGACACAGCGGTATCTTGATACCACCAGGAACGGTAAAAACAAATTCTAAGGAATCAAAAAAAGTGAAAAATTGGATCTATATCCAACGAATCACATCCGCCAAGAAAAAGTATTTTGTTTTGGTTCGACCAGTAATCATATATGAGATAGCGGACGGTATAAATTTAGAAACACTTTTCCCCCAGGATCTATTGCAGGAAAAAGAGAATCTGAAACTTCGAGTTGTCAATTATATTCTTTATGGAAATGGTAAACTAATTCGGGGAATTTCTGACACAAGTATTCAATTAGTTCGTACTTGTTTAGTGTTGAATTGGGACCAAGACAAAAAAAGTTCTTCTATCGAAGAGGCTCGCGCTTCTTTTGTTGAAGTAAGCACAAACGGTCTAATTTGTGATTTCCTAAGAATCAACCTAGCGAAATCCCATATTTCATATATCAGTAGAAAAAGGAATGATCCATCAGGTTCAGGACCGATCTCTAATAATGAGTCAGATCGAACCAATATTAATCCATTTTATCCCATTTATTCCAAGACAAGGATTCAACAATCACTTAAACAAAATCAAGGAACTATTAGTACGTTGTTGAATAGAAATAAGGAATGTCAATCTTTGATAATTTTGTCATCATCTAATTGTTTTCGAATGGATCCATTCAGCGATGCAAAACATCACAATGTAATAAAAGAATCAATTAAAAGAGATCCTATACCTATAATTCCAATTAGAAATTCATTGGGCCCTGTAGGAACAGCCCTTCCAATTGCGAATTTTTATTTATTTTACCTAATATTAATAACTCATAATCAGGTCTCGTTAACTAAATATTGGAAACTTGACAATTTAAAACAGACTTTTCAAGTACTTAAATATTATTTAATGGACGAAAACGGGAGAATTGTTAATCCTGATTCATGCAGTAACAGCGTTTTGAATCCATTCAATTTGAATTGGTATTTTCTCCATCATAATTATTATCATAATTTTTGTGAAGAAAGATTTACAACAATTAGCCTGGGACAGTTTATTTGTGAAAATGTATGTATGGCCAAAAACGGACCCCATCTAAAATCGGGTCAAGTTATAATTGTTCGCATGGACTCGGTAGTAATACGATCAGCAAAGCCCTATTTGGCCGCTCCAGGAGCAACCGTTCATGGCCATTATGGAGAAATCTTTTACGAAGGAGATACATTAGTTACATTTTTATATGAAAAATCGAGATCTGGTGATATAACGCAGGGTCTTCCAAAAGTGGAACAAGTGTTAGAAGTGCGCTCAATTGATTCAATATCGATAAGCCTAGAAAAGAGAGTTGAGGGTTGGAACGAGTGTATAACAAGAATTCTTGGAATTCCTTGGGGATTCTTGATTGGTGCTGAACTAACTATAGTGCAAAGTCGTATCTCTTTGGTTAATAAGATTCAAAAGGTTTATCGATCCCAAGGAGTGCAGATACATAATAGGCATATAGAAATTATTGTACGTCAAATAACATCCAAAGTTTTGGTTTCAGAAGACGGAATGTCAAATGTTTTTTCACCCGGAGAACTAATTGGATTGTTGCGAGCGGAACGAACGGGACGCGCTTTAGAAGAAGCCATCTGTTATCGAGCCATATTATTAGGAATAACGAGAGCATCTCTGAATACTCAAAGTTTCATATCCGAGGCCAGTTTTCAAGAAACTGCTCGCGTTTTAGCAAAAGCCGCTCTCCGCGGTCGTATCGATTGGTTGAAAGGCCTGAAAGAAAACGTTGTTCTAGGTGGTATGATACCCGGTGGTACCGGATTCAAAGGATTAGTGCAAGGCTCAAGGCAACATAATAACATTCCTTTGAAAACCAAAAAGAAGAATTTATTCGAGGGGGAATTTAGAGATAGAGATATTTTATTCCACCACAGAGAGTTATTTGATTCTTGCATTTCAAAAAATTTCTATGATACAGCAAAATAA

>lcl|NC_012224.1_cds_YP_002720103.1_11 [gene=rpoC1] [locus_tag=JacuC_p011] [db_xref=GeneID:7564839] [protein=RNA polymerase beta' subunit] [protein_id=YP_002720103.1] [location=complement(join(22413..24023,24804..25235))] [gbkey=CDS]

ATGATTGATCGGTATAAACATCAACAACTCCGAATTGGATCAGTTTCGCCTCAACAAATAAGTGCTTGGGCCAATAAAATCCTACCTAACGGAGAGATTGTTGGAGAGGTGACAAAACCCTATACTTTTCATTACAAAACCAATAAACCTGAAAAAGATGGATTATTTTGTGAAAGAATTTTTGGGCCTATAAAAAGTGGAATTTGTGCTTGTGGAAATTATCGAGTAATCAGAAATGAAAAAGAAGACCCAAAATTTTGTGAACAATGCGGAGTCGAGTTTGTTGATTCTCGGATACGAAGATATCAAATGGGCTACATCAAACTGGCATGCCCAGTAACTCATGTGTGGTATTTGAAACGTCTTCCTAGTTATATCGCAAATCTTTTAGATAAACCTCTTAAAGAATTAGAGGGCCTAGTATACTGCGATTTTTCTTTTGCTAGGCCCATAGCTAAAAAACCCACTTTTTTACGATTACGAGGTTCATTCGAATATGAAATCCAATCTTGGAAATACAGTATTCCACTTTTTTTTACTACCCAAGGCTTCGATACATTTCGAAATCGAGAAATTTCTACAGGAGCTGGTGCTATCCGAGAACAATTAGCCGATCTGGATTTGCGAATTATTATAGATTATTCATCGGTAGAATGGAAAGAATTAGGGGAAGAAGGGCCTACAGGGAATGAATGGGAAGATCGAAAAGTTGGAAGAAGAAAGGATTTTTTGGTTAGACGCGTGGAATTAGCTAAGCATTTTATTCGAACAAATATAGAACCAGAATGGATGGTTTTATGTCTATTACCGGTTCTTCCTCCCGAGTTGAGACCAATCATTCAGATAGATGGGGGTAAACTAATGAGTTCAGATATTAATGAACTCTATAGAAGAGTTATCTATCGGAACAATACTCTTATTGATCTATTAACAACAAGTAGATCTACGCCAGGGGAATTAGTAATGTGTCAGGAGAAATTGGTACAAGAAGCCGTGGATACACTTCTTGATAATGGAATCCGCGGACAACCTATGAGGGACGGTCATAATAAGGTTTACAAGTCGTTTTCGGATGTAATTGAAGGCAAAGAAGGAAGATTTCGTGAGACTATGCTTGGCAAACGGGTTGATTATTCGGGGCGTTCTGTCATTGTCGTAGGCCCCTCACTTTCATTACATCGATGTGGATTGCCTCGCGAAATAGCAATAGAGCTTTTCCAGATATTTGTAATTCGTGGTCTAATTAGGCAACATCTTGCTTCGAACATAGGAGTTGCTAAGAGTAAAATTCGGGAAAAAGAACCAATTGTATGGGAAATACTTCAAGAAGTTATGCAGGGACATCCGGTATTACTGAATAGAGCGCCGACTCTGCATAGATTAGGCATACAGGCGTTCCAACCCATTTTAGTGGAAGGCCGTGCTATTTGTTTACATCCATTGGTTTGTAAGGGATTCAATGCAGACTTTGATGGGGATCAAATGGCTGTTCATGTACCTTTATCGTTGGAGGCTCAAACAGAGGCTCGTTTACTTATGTTTTCTCATATGAATCTCTTGTCTCCAGCTATTGGAGATCCCATCTCCGTACCAACTCAAGATATGCTTATTGGGCTCTATGTATTAACAAGCGGGAATCGTCGAGGTATTTGTGCAAATAGGTATAATCCATGTAATCGCAGAAATTCTCAAAATGAAAGAATTGACGATAATAACAATAAATATACGAAAGAACCTTTTTTTTCTAATTCTTATGATGCAATTGGCGCTTTTCGGCGGAAAAGAATCAATTTAGATAGCCCTTTGTGGCTCCGGTGGCAACTAGATCAACGCGCTATTGTTTCAAGAGAAGCTCCCCTCGAAGTTCACTATGAATCTTTAGGTACCTATCATGAGATTTATGAACACTATCTAATAGTAAGAAATATAAAAAAAGAAATTATTTGTATATACATTCGAACTACTGTTGGTCATATTTCTTTTTATCGAGAAATCGAAGAAGCTATACAAGGGTTTTGTCAAGCCTGCTCAGATGGTATCTAA

>lcl|NC_012224.1_cds_YP_002720104.1_12 [gene=rpoB] [locus_tag=JacuC_p012] [db_xref=GeneID:7564840] [protein=RNA polymerase beta subunit] [protein_id=YP_002720104.1] [location=complement(25275..28493)] [gbkey=CDS]

ATGCTCGGGGATGGAAATGAGGGACTGTCTACAATACCTGGATTAAATCAGATACAATTTGAAGGATTTTGCGGGTTCATTGATCAGGGCTTAACAGAAGAACTTTATAAGTTTCCAAAAATGGAAGATACAGATCAAGAAATTGAATTTCAATTATTTGTGGAAACATATCAATTAGTAGAGCCATTGATAAAAGAAAGAGATGCTGTATATGAATCACTTACATATTCTTCTGAATTATATGTATCTGCGGGATTAATTTGGAAAACCAGTAGGGATATGCAAGAACAAACAATTTTTATTGGAAACATTCCTCTAATGAATTCCCTGGGAACTTTTATAATAAATGGAATATACAGAATTGTGATCAATCAAATATTGCAAAGTCCCGGTATCTATTACCGGTCAGAATTGGATCATAACGGAATCCCGGTCTATACCGGCACCATAATATCAGATTGGGGGGGAAGAGTAGAATTAGAGATTGATAGAAAAGCAAGGATATGGGCTCGTGTGAGTAGGAAACAGAAGATATCTATTCTAGTTTTATCATCAGCTATGGGTTTGAATCTAAGAGAAATTTTAGAGAATGTGTGCTGCCCTGAAATTTTCTTATCTTTTCTGAATCTGAATGATAAGGGAAAAAAAAAAATGGGGTCAAAGGAAAATGCCATTTTGGAGTTTTATCAACAATTTACTTGTGTAGGCGGAGATCCAGTATTTTCTGAATCCTTATGTAAGGAATTACAAAAGAAATTCTTTCAACAAAGATGTGAATTAGGAAGGATTGGTCGATTAAATATGAACCGGAGACTGAATCTTGATATACCTCATAACAATACATTTTTGTTACCACGAGATATATTGGCAGCTGCGGATCATTTGATTGGAATGAAATTTGGAATGGGTACACTTGACGATATGAATCATTTAAAAAATAAACGTATTCGTTCTGTAGCGGATCTCTTACAAGATCAATTCGGATTGGCTCTGATTCGTTTAGAAAACGTGGTTAGAGGGACTATATGGGGAGCAATTAGGCATAAATTGATACCGACCCCTCAAAATTTGGTAACTTCAACTCCATTAACAACTACTTATGAATCTTTTTTCGGATTACACCCATTATCTCAAGTTTTGGATCGAACTAATCCATTGACACAAATAGTTCATGGGAGAAAATCGAGTTATTTGGGTCCTGGAGGATTAACGGGACGAACTGCTAGTTTTCGAATACGAGATATCCACCCTAGTCACTATGGGCGCATTTGCCCAATTGACACGTCTGAAGGAATCAATGTTGGACTTATTGGGTCTTTAGCAATTCATGCCAAGATTGGTTATTGGGGGTCTTTAGAAAGCCCATTTTATGGAATCTCTGAGGGATCAAAAAAAGCACGGATGCTTTATTTATCACCAAATAGAGAGGAATACTATAGGGTAGCGGCAGGAAATTCTTTGGCGCTGAATCGAGGTGTTCAGGAAGAACAGGTTGCTCCAGCTCGATATCGTCAAGAATTCCTGACTATTGCATGGGAACAGGTGCATCTTCGAAGTATTTTTCCCTTCCAATATTTTTCTATTGGAGCTTCCCTCATTCCTTTTATCGAGCATAATGATGCGAATCGGGCTTTAATGAGTTCTAATATGCAACGTCAGGCAGTTCCACTTTCTCGATCCGAAAAATGCATTGTTGGAACTGGATTGGAACGCCAAGTTTCTCTAGATTCAGGGGTTCCTGCTATAGCCGAACACGAGGGAAAGATAATTTATACTGATATTGACAAAATCATTTTATCGGGTAATGGAGATACTCTACGCATTCCATTAGTTATGTATCAACGTTCCAACAAAAATACTTGTATGCATCAAAAAACCCAGGTTCAGCGGGGTAAATGCATTAAAAAGGGACAAGTTTTAGCGGATGGTGCCGCTACAGTTGGTGGCGAACTCGCCTTGGGCAAAAACGTATTAGTAGCTTATATGCCATGGGAGGGTTACAATTTTGAGGATGCGGTGCTCATTAGCGAACGTCTGGTATATGAAGATATTTATACTTCTTTTCACATACGGAAATATGAAATTCAGACTCATGTGACAAGCCAAGGACCTGAAAAGATCACTAATGAAATACCGCATCTAGAAGCCCATTTACTCCGAAATTTAGACAAAAATGGAATTGTGATCCTAGGATCTTGGGTGGAGACGGGCGATATTTTAGTAGGTAAATTAAGGCCTCAAATGGCGAAAGAATCATCGTATGCTCCAGAAGATAGATTATTAAGAGCTATACTTGGTATTCAGGTATCGACTTCAAAGGAAACTTGTCTAAAACTACCTATAGGTGGTAGGGGTCGAGTTATTGATGTGAGATGGATCCAGAAAAAGGGGGGTTCCAGTTATAATCCGGAAACGATTCGTGTCTATATTTTACAGAAACGTGAAATCAAAGTGGGTGATAAAGTAGCTGGAAGACATGGAAATAAAGGCATCATTTCCAAAATTTTGCCTAGACAGGATATGCCTTATTTGCAAGATGGAAGACCTGTTGATATGGTCTTCAACCCATTAGGAGTACCTTCACGAATGAATGTAGGACAGATATTTGAATGCTCACTCGGGTTAGCGGGGGGTCTGCTAGATAGACATTATCGAATAGCACCCTTTGATGAGAGATATGAACAAGAGGCTTCGAGAAAACTAGTGTTTTCGGAATTATATGAAGCCAGTAAGCAAACAGCAAATCCGTGGGTATTTGAACCCGAGTATCCGGGAAAAAGTAGAATATTTGATGGAAGAACGGGGGATCCTTTTGAACAGCCTGTTATAATAGGAAAGCCTTATATCTTGAAATTAATTCATCAAGTTGATGATAAAATACATGGACGTTCCAGTGGACATTATGCACTTGTTACACAACAACCCCTTAGAGGAAGGGCCAAGCAAGGGGGACAACGGGTCGGAGAAATGGAGGTTTGGGCTCTAGAGGGATTTGGTGTTTCTCATATTTTACAAGAGATGCTTACTTATAAATCTGATCATATTAGAGCTCGCCAAGAAGTACTTGGTACTACGATCATTGGGGGAACAATACCTAAACCTGAAGATGCTCCAGAATCTTTTCGATTGCTCGTTCGAGAACTACGATCTTTGGCTCTGGAACTGGATCATTTCCTTGTATCTGAGAAGAACTTCCAGATTAATAGGAAGGAAGTTTAA

>lcl|NC_012224.1_cds_YP_002720105.1_13 [gene=petN] [locus_tag=JacuC_p013] [db_xref=GeneID:7564842] [protein=cytochrome b6/f complex subunit VIII] [protein_id=YP_002720105.1] [location=30761..30850] [gbkey=CDS]

ATGGATATAGTAAGTCTCGCTTGGGCTGCTTTAATGGTAGTCTTTACATTTTCCCTTTCACTCGTAGTATGGGGAAGAAGTGGACTCTAG

>lcl|NC_012224.1_cds_YP_002720106.1_14 [gene=psbM] [locus_tag=JacuC_p014] [db_xref=GeneID:7564843] [protein=photosystem II protein M] [protein_id=YP_002720106.1] [location=complement(32146..32250)] [gbkey=CDS]

ATGGAAGTAAATATTCTTGCATTTATTGCTACTGCACTGTTCATTCTAGTTCCTACCGCTTTTTTACTTATAATATACGTAAAAACAGTTAGTCAAGGCGATTAA

>lcl|NC_012224.1_cds_YP_002720107.1_15 [gene=psbD] [locus_tag=JacuC_p015] [db_xref=GeneID:7564848] [protein=photosystem II protein D2] [protein_id=YP_002720107.1] [location=37203..38264] [gbkey=CDS]

ATGACTATAGCCCTTGGTAAATTTACCAAAGACGAAAATGATTTATTTGATATTATGGATGACTGGTTACGGAGGGACCGTTTCGTTTTTGTAGGTTGGTCCGGTCTATTGCTCTTTCCTTGCGCCTATTTCGCCTTAGGGGGTTGGTTCACAGGTACAACCTTTGTAACCTCATGGTATACCCATGGATTGGCTAGTTCCTATTTGGAAGGCTGCAACTTCTTAACCGCTGCAGTTTCTACTCCTGCTAATAGTTTAGCGCATTCTTTGTTATTACTATGGGGTCCCGAAGCACAAGGGGATTTTACTCGTTGGTGTCAATTAGGCGGTTTGTGGACTTTTGTTGCTCTCCACGGTGCTTTCGGACTAATAGGTTTTATGTTACGTCAATTTGAACTTGCTCGATCCGTGCAATTGCGACCTTATAATGCAATCGCATTCTCTGGTCCAATTGCTGTTTTTGTTTCTGTATTCCTGATTTATCCACTAGGTCAGTCTGGTTGGTTTTTTGCGCCTAGTTTTGGTGTAGCAGCTATATTTCGATTCATCCTCTTTTTCCAAGGGTTTCATAACTGGACGCTGAACCCATTTCATATGATGGGGGTTGCCGGCGTATTAGGTGCTGCTCTGCTATGCGCTATTCATGGTGCTACTGTAGAAAATACTTTATTTGAAGATGGTGATGGTGCAAATACATTCCGTGCTTTTAACCCAACTCAAGCCGAAGAAACTTATTCAATGGTTACCGCTAACCGCTTTTGGTCCCAAATCTTTGGGGTTGCTTTTTCCAATAAACGTTGGTTACATTTCTTTATGTTATTTGTACCAGTAACCGGTTTATGGATGAGTGCTCTTGGAGTAGTCGGTCTGGCTCTGAATCTACGTGCCTATGACTTCGTTTCTCAGGAGATCCGTGCAGCGGAAGATCCTGAATTTGAGACTTTCTACACTAAAAATATTCTCTTAAACGAAGGTATTCGTGCTTGGATGGCGGCTCAAGATCAGCCTCATGAAAACCTTATATTCCCTGAGGAGGTTCTACCACGTGGAAACGCTCTTTAA

>lcl|NC_012224.1_cds_YP_002720108.1_16 [gene=psbC] [locus_tag=JacuC_p016] [db_xref=GeneID:7564849] [protein=photosystem II 44 kDa protein] [protein_id=YP_002720108.1] [location=38212..39633] [gbkey=CDS]

ATGAAAACCTTATATTCCCTGAGGAGGTTCTACCACGTGGAAACGCTCTTTAATGGAACTTTATCTTTAGCCGGTCGTGACCAAGAAACTACCGGTTTCGCTTGGTGGGCCGGGAATGCCCGACTTATCAATTTATCCGGTAAACTACTGGGAGCTCATGTAGCTCATGCTGGATTAATCGTATTCTGGGCCGGAGCAATGAACCTATTTGAAGTGGCTCATTTCGTACCGGAGAAACCAATGTATGAACAAGGATTAATTTTACTTCCCCACCTAGCTACTCTAGGTTGGGGGGTAGGTCCTGGTGGGGAAGTTATAGACACCTTTCCATACTTTGTATCTGGCGTACTTCACTTAATTTCCTCTGCAGTATTGGGCTTTGGCGGTATTTATCATGCACTTCTGGGTCCTGAGACTCTTGAAGAATCTTTTCCATTTTTTGGTTATGTATGGAAAGATAGAAATAAAATGACAACAATTTTAGGTATTCACTTAATCTTGCTAGGTATAGGTGCTTTTCTTCTAGTATTCAAGGCTCTTTATTTTGGGGGCGTATATGATACCTGGGCTCCGGGGGGGGGAGATGTAAGAAAAATTACCAACTTGACCCTTAGCCCAAGTGTTATTTTTGGTTATTTACTAAAATCCCCCTTTGGAGGAGAAGGATGGATTGTTAGTGTGGACGATTTGGAAGATATAATTGGAGGGCATGTATGGTTAGGTTCCATTTGTATACTTGGGGGAATCTGGCATATCTTAACCAAACCCTTTGCATGGGCTCGCCGTGCACTTGTATGGTCCGGAGAGGCTTACTTGTCTTATAGTTTAGGTGCTTTATCCGTTTTTGGTTTCATTGCTTGTTGCTTTGTCTGGTTCAATAATACCGCTTATCCTAGTGAGTTTTACGGGCCTACTGGACCGGAAGCTTCTCAAGCTCAAGCTTTTACTTTTCTAGTTAGAGATCAACGTCTTGGGGCTAACGTGGGATCCGCTCAAGGACCTACCGGGTTAGGTAAATATTTAATGCGTTCGCCTACTGGAGAAGTTATTTTTGGAGGAGAAACTATGCGTTTTTGGGATCTACGTGCTCCTTGGTTAGAACCTCTAAGAGGTCCAAATGGTTTGGACTTGAGTAGGTTGAAAAAAGACATACAACCTTGGCAAGAACGCCGTTCCGCGGAATATATGACCCATGCGCCTTTAGGTTCGTTGAATTCTGTAGGTGGCGTAGCTACCGAGATCAATGCAGTCAATTATGTCTCTCCTAGAAGTTGGTTAGCTACCTCTCATTTTGTTCTAGGGTTCTTCCTATTCGTAGGTCATTTATGGCACGCGGGAAGGGCTCGTGCAGCTGCAGCAGGATTTGAAAAAGGAATTGATCGTGATTTTGAACCTGTTCTTTCCATGACTCCTCTTAACTAA

>lcl|NC_012224.1_cds_YP_002720109.1_17 [gene=psbZ] [locus_tag=JacuC_p017] [db_xref=GeneID:7564851] [protein=psbZ] [protein_id=YP_002720109.1] [location=40333..40521] [gbkey=CDS]

ATGATTATTGCTTTCCAATTGGCTGTTTTTGCATTAATTGCTACTTCATCAATCTTACTGATTAGTGTACCCGTTGTTTTTTCTTCTCCTGATGGTTGGTCAAGTAACAAAAATGTTGTATTTTCTGGTACATCATTATGGATTGGATTAGTTTTTCTGGTAGGTATCCTTAATTCTCTCATCTCTTGA

>lcl|NC_012224.1_cds_YP_002720110.1_18 [gene=rps14] [locus_tag=JacuC_p018] [db_xref=GeneID:7564854] [protein=ribosomal protein S14] [protein_id=YP_002720110.1] [location=complement(41894..42196)] [gbkey=CDS]

ATGGCAAGGAAAAGTTTGATTCAGCGGGAGAAGAAGAGGCAAAAATTGGAACAAAAATATCATTTGATTCGTCGATCCTCAAAAAAAGAAATAAGCGAAGTTCTATCGTTGAGTGATAAATGGGAAATTCATGGAAAGTTACAATCCCTACCGCGAAATAGTGCACCGACACGTCTTCATCGACGTTGTTTTTCGACTGGAAGACCGAGAGCTAACTATCGAGACTTTGGGCTATCTGGACACATACTTCGTGAAATGGTTCATGCATGTTTGTTACCGGGGGTAACAAGATCAAGTTGGTAA

>lcl|NC_012224.1_cds_YP_002720111.1_19 [gene=psaB] [locus_tag=JacuC_p019] [db_xref=GeneID:7564855] [protein=photosystem I P700 chlorophyll a apoprotein A2] [protein_id=YP_002720111.1] [location=complement(42314..44518)] [gbkey=CDS]

ATGGCATTAAGATTTCCAAGGTTTAGCCAAGGCTTAGCTCAGGACCCCACTACTCGTCGTATTTGGTTTGGTATTGCTACCGCACATGACTTCGAGAGTCATGATGATATTACGGAGGAACGGCTTTATCAGAATATTTTTGCTTCCCACTTCGGACAATTAGCAATAATTTTTCTGTGGACTTCCGGAAATCTCTTTCATGTAGCTTGGCAAGGAAATTTTGAAGCATGGGTACAGGACCCTTTACACGTAAGACCTATTGCTCATGCAATTTGGGATCCTCATTTTGGCCAACCGGCCGTGGAAGCTTTTACTCGAGGGGGCGCCCCTGGCCCAGTGAATATCGCTTATTCTGGTGTTTATCAATGGTGGTATACAATCGGTTTACGTACTAACGAAGATCTTTATATTGGAGCTCTTTTTCTATTATTTCTTTCTGCCCTAGCCTTATTAGGGGGTTGGTTACACCTACAACCGAAATGGAAACCGAGCGTTTCGTGGTTCAAAAACGCCGAATCTCGTCTCAATCATCATTTGTCGGGACTATTCGGAGTAAGCTCTTTAGCTTGGACAGGACATTTAGTCCATGTCGCTATTCCTGGTTCCAGGGGCGAATACGTTCGATGGAATAATTTCTTAGATGTATTACCCCATCCCCAAGGATTAGGCCCCCTTTTTACAGGTCAGTGGAATCTTTATGCTCAAAATCCCGATTCAGGTAGTCATTTATTTGGTACCTCCCAAGGATCAGGAACTGCCATTCTAACCCTTCTCGGGGGGTTCCATCCACAAACACAAAGTTTATGGCTGACCGATATTGCACACCATCATTTAGCTATTGCATTTCTTTTTCTCGTTGCCGGTCATATGTATAGAACTAACTTCGGGATTGGGCACAGTATAAAAGATCTTTTAGAAGCACATATTCCTCCCGGGGGGCGATTGGGGAGTGGACATAAGGGTCTTTATGACACAATCAACAATTCGCTTCATTTTCAATTAGGCCTTGCTCTAGCTTCTTTAGGGGTTATTACTTCCTTAGTAGCTCAACACATGTACTCATTACCTGCTTATGCATTCATAGCGCAAGACTTTACTACTCAAGCCGCGTTATATACTCATCACCAATACATCGCAGGATTCATCATGACAGGAGCTTTTGCTCATGGAGCTATATTTTTTATTAGAGATTACAATCCGGAACAGAACGAGAATAATGTATTGGCAAGAATGTTAGACCATAAAGAAGCTATCATATCCCATTTAAGTTGGGCTAGCCTCTTTCTGGGATTCCATACTTTGGGACTTTATGTTCATAATGATGTTATGCTTGCTTTTGGTACTCCGGAGAAACAAATCTTGATCGAACCCATATTTGCCCAATGGATACAATCTGCTCACGGTAAAACTTCATATGGATTCGATGTACTTTTATCTTCAACGAATAGTGCAGCCTTCAATGCAGGTCGAAGCATATGGTTGCCCGGCTGGTTAAATGCTGTTAATGAAAATAGTAATTCATTATTCTTAACAATAGGGCCTGGAGACTTCTTGGTTCATCATGCTATTGCTCTAGGTTTACATACAACCACATTAATCTTAGTAAAAGGTGCTTTAGATGCACGTGGTTCGAAGTTAATGCCAGATAAAAAGGATTTTGGTTATAGTTTTCCTTGCGATGGTCCGGGACGCGGTGGTACTTGTGATATTTCGGCTTGGGACGCATTTTATTTGGCGGTTTTCTGGATGTTAAATACCATTGGATGGGTTACTTTTTATTGGCATTGGAAGCACATCACATTATGGCAGGGTAATGTTTCACAGTTTAATGAATCTTCCACTTATTTGATGGGATGGTTAAGAGATTATCTATGGTTAAACTCTTCACAACTTATCAATGGATATAACCCTTTCGGTATGAATAGCTTATCGGTCTGGGCGTGGATGTTCTTATTTGGACATCTTGTTTGGGCTACTGGATTTATGTTTTTAATTTCTTGGCGTGGATATTGGCAGGAATTGATTGAAACTTTAGCGTGGGCTCATGAACGTACGCCTTTGGCTAATTTGATTCGATGGAGAGATAAACCAGTAGCTCTTTCCATTGTGCAAGCAAGATTGGTTGGATTAGCCCACTTTTCTGTAGGTTATATCTTCACTTATGCGGCTTTCTTGATTGCCTCTACATCAGGTAAATTTGGTTAA

>lcl|NC_012224.1_cds_YP_002720112.1_20 [gene=psaA] [locus_tag=JacuC_p020] [db_xref=GeneID:7564856] [protein=photosystem I P700 chlorophyll a apoprotein A1] [protein_id=YP_002720112.1] [location=complement(44544..46796)] [gbkey=CDS]

ATGATTATTCGTTCGCCGGAACCAGAAGTCAAAATTTTGGTAGATAGGGATCCCATCAAAACTTCTTTCGAGGAATGGGCCAGACCCGGTCATTTCTCAAGAACAATAGCTAAGGGACCTGATACTACCACTTGGATCTGGAACCTACATGCTGATGCTCACGATTTCGATAGCCATACCAGTGATTTGGAGGAGATTTCTCGAAAAGTATTTAGTGCTCATTTCGGCCAACTCTCCATCATCTTTCTTTGGCTGAGTGGCATGTATTTCCACGGTGCTCGTTTTTCCAATTATGAAGCATGGCTAAGTGATCCTACTCACATTGGACCTAGCGCCCAAGTGGTTTGGCCGATAGTGGGTCAAGAAATATTGAATGGTGATGTGGGGGGGGGGTTCCGAGGAATACAAATAACCTCCGGTTTTTTTCAGCTTTGGAGAGCATCTGGAATAACTAGTGAATTACAACTGTATTGTACTGCAATTGGTGCATTGGTCTTTGCAACTTTAATGCTTTTTGCTGGTTGGTTCCATTATCACAAAGCTGCTCCAAAATTGGCTTGGTTCCAAGATGTAGAATCTATGTTGAATCACCATTTAGCGGGACTACTAGGACTTGGGTCTCTTTCTTGGGCGGGACATCAAGTACATGTATCTTTACCAATTAACCAATTTCTAAACGCTGGGGTGGATCCTAAGGAAATCCCACTTCCTCATGAATTTATCTTGAATCGGGATCTTTTGGCTCAACTTTATCCCAGTTTTGCTGAGGGAGCAACCCCATTTTTCACCTTGAATTGGTCAAAATATTCGGAATTTCTTACTTTTCGTGGAGGATTAGATCCAGTGACTGGGGGTCTATGGCTGACCGATATTGCACACCATCATTTAGCTATTGCAATTCTTTTCCTGATAGCGGGTCACATGTATAAGACTAACTGGGGCATTGGTCATGGTATAAAAGATATTTTAGAGGCTCATAAAGGTCCATTTACAGGTCAGGGCCATAAGGGCCTATATGAGATCCTAACAACGTCATGGCATGCTCAATTATCTCTTAACCTAGCTATGTTAGGTTCTTTAACCATTGTTGTAGCTCACCATATGTATTCCATGCCCCCTTATCCATATTTAGCTACTGACTATGGTACACAACTGTCATTGTTCACACATCACATGTGGATTGGTGGATTTCTCATAGTTGGTGCTGCTGCACATGCAGCCATTTTTATGGTAAGAGACTATGATCCAACTACTCGATACAACGATCTATTAGATCGTGTTCTTAGGCATCGCGATGCAATCATATCACATCTCAACTGGGTATGTATTTTTTTAGGTTTTCATAGTTTTGGTTTATATATTCATAATGATACCATGAGCGCTTTAGGGCGCCCTCAAGATATGTTTTCCGATACTGCTATACAATTACAACCCGTCTTTGCTCAATGGATACAAAACACCCATGCTTTAGCACCTGGTGCAACGGCTCCTGGTGCAACAGCAAGCACCAGTTTGACTTGGGGGGGTGGTGATTTAGTGGCAGTGGGTGGCAAGGTTGCTTTGTTACCAATTCCATTAGGAACCGCAGATTTTTTGGTACATCACATTCATGCATTTACGATTCATGTGACGGTATTGATACTTCTGAAAGGAGTTCTATTTGCCCGTAGCTCTCGTTTGATACCGGATAAAGCAAATCTTGGTTTTCGTTTCCCTTGTGATGGGCCTGGAAGAGGGGGAACATGTCAAGTATCCGCTTGGGATCATGTCTTCTTAGGGTTATTTTGGATGTACAATTCCATTTCGGTTGTAATATTCCATTTCAGTTGGAAAATGCAGTCAGATGTTTGGGGTAGTATAAGTGATCAAGGGGTGGTAACTCATATCACGGGAGGAAACTTTGCACAGAGTTCCATTACTATTAATGGATGGCTCCGCGATTTCTTATGGGCACAGGCATCCCAGGTAATTCAGTCTTATGGTTCTTCATTATCTGCATATGGCCTTTTTTTCCTAGGTGCTCATTTTGTATGGGCTTTTAGTTTAATGTTTCTATTCAGTGGTCGTGGTTATTGGCAAGAACTTATTGAATCAATCGTTTGGGCTCATAATAAATTAAAAGTTGCTCCTGCTACTCAGCCTAGAGCCTTGAGCATTATACAAGGACGTGCTGTAGGAGTAACCCATTACCTTCTGGGTGGAATTGCCACAACATGGGCGTTCTTCTTAGCAAGAATTATTGCAGTAGGATAA

>lcl|NC_012224.1_cds_YP_002720113.1_21 [gene=ycf3] [locus_tag=JacuC_p021] [db_xref=GeneID:7564857] [protein=photosystem I assembly protein Ycf3] [protein_id=YP_002720113.1] [location=complement(join(47666..47815,48502..48729,49421..49549))] [gbkey=CDS]

ATGCCTAGATCCCGGACAACTGGAAATTTTATTGATAAGACCTTTTCAATTGTAGCCAATATCTTATTACGAATAATTCCAACAACTTCGGGAGAAAAAGAGGCATTTACTTATTACAGAGATGGTGTGATGTCTGCTCAATCCGAAGGAAATTATGCAGAAGCTTTACAGAATTATTATGAAGCTTTGCGGCTAGAAATTGATCCCTATGATCGAAGTTATATACTCTATAATATAGGCCTTATTCACACAAGTAATGGAGAACACACAAAAGCTTTGGAATATTATTTTCGGGCACTAGAACGAAACCCCTTCTTACCACAAGCTTTAAATAATATGGCCGTGATCTGTCATTACGGAGAACAGGCCATTCGGCAGGGAGATTCTGAAATTGCGGAAGCTTGGTTCGATCAAGCCGCGGAGTATTGGAAACAAGCTATAGCGCTTACTCCCGGAAATTATATTGAAGCGCAGAATTGGTTGAAGATCACAAGGCGTTTCGAATAA

>lcl|NC_012224.1_cds_YP_002720114.1_22 [gene=rps4] [locus_tag=JacuC_p022] [db_xref=GeneID:7564859] [protein=ribosomal protein S4] [protein_id=YP_002720114.1] [location=complement(50193..50798)] [gbkey=CDS]

ATGTCACGTTACCGAGGGCCTCGTTTCAAAAAAATACGCCGTCTCGGGGCTTTACCGGGACTAACTAGTAAAAGGCCTAGAGCCGGGAGCGATCTTAGAAATCAATCACGCTCCGGTAAAAAATCTCAATATCGTATTCGTTTAGAAGAAAAGCAAAAATTACGTTTTCATTATGGTCTTACAGAACGACAATTGCTTAAATACGTTCGTATCGCCGCAAAAGCCAAAGGGTCAACGGGTCAGGTTTTACTACAATTACTTGAAATGCGGTTGGATAACATCCTTTTTCGATTGGGTATAGCGTCAACTATTCCTCGAGCCCGCCAATTAGTTAATCATAGACATATTTTAGTTAATGGCCGTATAGTAGATATACCAAGTTATCGCTGCAAACCCCGAGATGTTATTACAGTGAGGGATGAACAAAAATCTAGAGCTATGATTCCAAATTATCTTGATTCATCCCCCCAAGAGGAATTGCCAAAACATTTGACTCTTCACCCAATCCAATATAAAGGATTGGTCAATCAAATAATAGATAGTAAATGGGGTGGCTTAAAAATAAATGAATTGCTAGTGGTAGAATATTATTCTCGTCAGACTTAA

>lcl|NC_012224.1_cds_YP_002720115.1_23 [gene=ndhJ] [locus_tag=JacuC_p023] [db_xref=GeneID:7564863] [protein=NADH dehydrogenase subunit J] [protein_id=YP_002720115.1] [location=complement(53707..54183)] [gbkey=CDS]

ATGCAGGGTCGTTTGTCTGCTTGGCTAGTCAAACATGGGCTAGTTCATAGATCTTTGGGTTTTGATTACCAAGGAATAGAGACTTTACAAATAAAGCCCGAAGATTGGCATTCCATTGCTGTCATTTTATATGTATATGGTTACAATTATCTGCGTTCGCAATGTGCCTATGATGTAGCACCGGGCGGGCTGTTAGCTAGTGTATATCATCTTACGAGAATAGAGTATGGTATAGATCAACCAGAAGAAGTATGTATAAAAGTATTTGCCCCAAGGAAGAATCCTAGAATTCCGTCTGTTTTCTGGGTTTGGAAAAGTGCGGATTTTCAAGAAAGGGAATCTTATGATATGCTGGGAATCTTTTATGATAATCATCCGCGTCTGAAACGTATCTTAATGCCGGAAAGTTGGGTAGGGTGGCCCTTACGTAAAGATTATATTGCTCCCAATTTTTATGAAATACAAGACGCTCATTGA

>lcl|NC_012224.1_cds_YP_002720116.1_24 [gene=ndhK] [locus_tag=JacuC_p024] [db_xref=GeneID:7564864] [protein=NADH dehydrogenase subunit K] [protein_id=YP_002720116.1] [location=complement(54312..54989)] [gbkey=CDS]

ATGAATTCCATTGAGTTTCCTTTACTTGATCAAACAACTAAAATTTCAGTTATTTCAACTACATCAAATGATCTTTCAAATTGGTCAAGACTCTCCAGTTTATGGCCACTTCTCTATGGTACCAGTTGTTGCTTCATTGAATTTGCTTCGTTAATAGGCTCACGATTCGACTTTGATCGTTATGGACTAGTACCAAGATCTAGTCCTAGACAAGCGGACCTGATTTTAACAGCCGGCACAGTAACCATGAAAATGGCTCCCTCTTTAGTGAGATTATATGAACAAATGCCAGAACCAAAATATGTTATTGCTATGGGGGCATGTACAATTACAGGGGGGATGTTCAGTACCGATTCTTATAGTACTGTTCGGGGAGTCGATAAGCTAATTCCTGTAGATGTCTATTTGCCAGGCTGTCCACCTAAACCGGAGGCGGTTATAGATGCTATAACAAAACTTCGTAAAAAAATATCTCGAGAAATTTATGAAGATCGAATTAGGTCTCAACCGGGGAATCGGTGTTTTACTACCAATCACAAGTTTAATATTGAACGCACTACTCATACTGGAAATTATGATCGAGGATTACTCTATCAACCGCCGTCTACTTCAAAGATCCCTCCTGAAACATTTTTCAAATATAAAAGGTCAGTATCGTCCCACGAATTAGTAAATTAG

>lcl|NC_012224.1_cds_YP_002720117.1_25 [gene=ndhC] [locus_tag=JacuC_p025] [db_xref=GeneID:7564865] [protein=NADH dehydrogenase subunit 3] [protein_id=YP_002720117.1] [location=complement(55043..55405)] [gbkey=CDS]

ATGTTTCTGATTTACGAATATGATATATTCTGGGCATTTCTAATAATATCAAGTGCTATTCCTATTTTAGCATTTCTAATTTCCGGAGTTTTATCCCCGATTAGCAAAGGGCCGGAGAAACTTTCTAGTTATGAATCGGGTATAGAACCAATGGGCGATGCTTGGTTACAATTTCGAATCCGTTACTATATGTTTGCTCTAGTTTTTGTTGTTTTTGATGTTGAAACCGTTTTTCTTTATCCATGGGCAATGAGTTTCGATATATTGGGGTTATCCGCATTTATAGAAGCTTTAATTTTCGTACTTATCCTAATTGTTGGTTTAGTTTATGCGTGGAGAAAAGGAGCGTTAGAATGGTCTTAG

>lcl|NC_012224.1_cds_YP_002720118.1_26 [gene=atpE] [locus_tag=JacuC_p026] [db_xref=GeneID:7564868] [protein=atpE] [protein_id=YP_002720118.1] [location=complement(57561..57962)] [gbkey=CDS]

ATGACCTTAAATCTTTGTGTACTGACCCCAAATCGAATTGTTTGGGATTCAGAAGTGAAAGAAATCATTTTATCTACTAATAGTGGGCAAATTGGCGTATTACCAAATCATGCGCCTATTGCCACAGCTGTCGATATCGGTATTTTGAGAATACGCCTTAATGCCAAATGGTTAACGATGGCTCTGATGGGTGGTTTTGCTAGAATAGGCAATAATGAGATTACTGTTTTAGTAAATGATGCAGAGAAGGGTAGTGACATTGATCCACAAGAAGCTCAGCAAACTCTTGAAATAGCAGAAGCTAACTTGAGAAAAGCGGAAGGAAAGAGACAAATAATTGAGGCAAATCTAGCTCTCAGACGAGCTAGGGCACGAGTAGAGGCTATCAATGTGATTTCGTAA

>lcl|NC_012224.1_cds_YP_002720119.1_27 [gene=atpB] [locus_tag=JacuC_p027] [db_xref=GeneID:7564869] [protein=ATP synthase CF1 beta subunit] [protein_id=YP_002720119.1] [location=complement(57959..59446)] [gbkey=CDS]

ATGAGAATCAATCCTACTACTTCTGGTCCGGGAGTTCCCGCGCTTGAAAAAAAGAACCTGGGGCGTATCGCTCAAATCATTGGGCCAGTACTAGATGTAGCTTTTTCCCCGGGCAAGATGCCTAATATTTACAACGCTCTGGTAGTTAAGGGTCGAGATACTGTCGGGCAAGAAATTAATGTGACTTGTGAAGTACAACAATTATTAGGAAATAATCGAGTTCGGGCTGTAGCTATGAGTGCTACAGATGGTCTAACGCGAGGAATGGAAGTGATTGACACAGGAGCTCCTCTAAGTGTTCCAGTTGGTGGGGCGACTCTAGGACGAATTTTCAACGTGCTTGGAGAACCTGTTGATGATTTAGGTCCTGTAGATACTCGCGCAACATCACCTATTCATAGATCTGCACCTGCTTTTATACAATTAGATACAAAATTATCTATTTTTGAAACAGGAATTAAAGTAGTAGATCTTTTAGCCCCTTATCGCCGTGGAGGAAAAATCGGACTATTCGGGGGAGCTGGAGTGGGTAAAACAGTACTTATTATGGAATTAATCAACAACATCGCGAAAGCTCATGGAGGCGTATCCGTATTTGGCGGAGTAGGCGAACGAACTCGTGAAGGAAATGATCTTTACATGGAAATGAAAGAATCTGGAGTAATTAATCAAGAAAATATTGCAGAATCAAAAGTGGCTCTAGTCTATGGTCAGATGAACGAACCGCCGGGAGCTCGTATGAGAGTTGGTTTGACTGCCCTAACTATGGCGGAATATTTCCGAGATGTTAATGAACAAGACGTACTTCTATTTATCGACAATATCTTCCGTTTCGTCCAAGCAGGATCCGAAGTATCCGCCTTATTGGGTAGAATGCCTTCTGCTGTGGGTTATCAACCTACCCTTAGTACCGAAATGGGTTCTTTACAAGAAAGAATTACTTCCACCAAAGAGGGGTCCATAACTTCTATTCAAGCAGTTTATGTACCTGCGGACGATTTGACTGACCCTGCTCCTGCCACGACATTTGCACATTTAGATGCGACTACTGTACTATCAAGAGGATTAGCTGCTAAAGGTATCTATCCAGCAGTAGATCCTTTAGATTCAACATCAACTATGCTCCAACCTCAGATCGTTGGTGAGGAACATTATGAAACTGCGCAAAGAGTTAAGCAAACTTTACAACGTTACAAAGAACTTCAGGACATTATAGCTATCCTTGGGCTGGACGAATTATCCGAAGAGGATCGTTTAACTGTAGCAAGAGCACGAAAAATTGAGCGTTTCTTATCACAACCCTTTTTCGTAGCAGAAGTATTTACCGGTTCTCCGGGTAAATATGTCGGTCTAGCAGAAACTATTAGAGGGTTTAAATTGATCCTTTCCGGAGAATTAGATAGTCTCCCTGAACAGGCCTTTTATTTGGTAGGTAATATTGATGAAGCTACTGCGAAGGCTACAAACTTAGAAATGGAGAACAAATGA

>lcl|NC_012224.1_cds_YP_002720120.1_28 [gene=rbcL] [locus_tag=JacuC_p028] [db_xref=GeneID:7564870] [protein=ribulose-1,5-bisphosphate carboxylase/oxygenase large subunit] [protein_id=YP_002720120.1] [location=60251..61678] [gbkey=CDS]

ATGTCACCACAAACAGAGACTAAAGCAAGTGTTGGATTCAAGGCTGGTGTTAAAGATTATAAATTGACTTATTATACTCCTGAGTATCAAACCAAAGATACTGATATCTTGGCAGCATTCCGAGTAACTCCTCAACCTGGAGTTCCGCCTGAGGAAGCAGGAGCTGCGGTAGCTGCTGAATCTTCTACTGGTACATGGACAACTGTGTGGACCGATGGGCTTACCAGTCTTGATCGTTATAAAGGACGATGCTACGACATCGAGCCCGTTGCTGGAGAAGAAAATCAATATATTGCTTATGTAGCTTACCCCTTAGACCTTTTTGAAGAAGGTTCTGTTACTAACATGTTTACTTCCATTGTGGGTAATGTATTTGGGTTCAAAGCCCTACGCGCCCTACGTCTGGAGGATTTGCGAATCCCTACTGCTTATACTAAAACTTTCCAAGGGCCGCCTCATGGTATCCAAGTTGAGAGAGATAAATTGAACAAGTATGGTCGCCCCCTATTGGGTTGTACTATTAAACCTAAATTGGGGCTATCCGCTAAGAATTATGGTAGAGCGGTTTATGAATGTCTTCGCGGTGGACTTGATTTTACCAAAGATGATGAGAACGTGAATTCCCAACCATTTATGCGTTGGAGAGACCGTTTCTTATTTTGTGCCGAAGCAATTTATAAAGCACAGGCTGAAACAGGTGAAATCAAAGGACATTATTTGAATGCTACTGCAGGTACATGTGAAGAAATGATCAAAAGGGCTGTATTTGCCAGAGAATTAGGAGTTCCTATCGTAATGCATGACTACCTAACAGGGGGATTCACCGCAAATACTTCCTTGGCTCATTATTGCCGTGATAATGGTTTACTTCTTCACATTCACCGCGCAATGCATGCAGTTATTGATAGACAGAAGAATCATGGTATGCATTTTCGTGTACTAGCTAAGGCGTTACGTTTGTCTGGTGGAGATCATATTCACGCTGGTACCGTAGTAGGTAAACTTGAAGGGGAAAGAGACATCACTTTGGGCTTTGTTGATTTACTGCGTGATGATTTTATTGAAAAAGATCGAAGCCGCGGTATTTATTTCACTCAAGATTGGGTCTCTCTACCGGGTGTTTTGCCTGTAGCTTCAGGAGGTATTCACGTTTGGCATATGCCTGCTCTGACCGAGATCTTTGGAGATGATTCCGTACTACAATTCGGTGGAGGAACTTTAGGGCACCCTTGGGGAAATGCACCTGGTGCCGTAGCTAATCGAGTAGCTCTAGAAGCATGTGTACAAGCTCGTAATGAAGGACGTGATCTTGCTCGTGAGGGTAATGAAATTATCCGTGAGGCTAGCAAATGGAGTCCTGAACTAGCTGCTGCTTGTGAAGTATGGAAGGAGATTAAATTTGAATTCCAAGCGATGGATACTTTGTAA

>lcl|NC_012224.1_cds_YP_002720121.1_29 [gene=accD] [locus_tag=JacuC_p029] [db_xref=GeneID:7564871] [protein=acetyl-CoA carboxylase beta subunit] [protein_id=YP_002720121.1] [location=62443..63924] [gbkey=CDS]

ATGGAAAAACGGTGGTTCAATTCGATCTTATCCAATGTAGAATTAGGATACAGGTGTAGGTTAAGTAAATCAATGGATAGTTTTAGTCCTCTTGAAAATACCAGTATAAGCGAAGACCCAATTCTAAACGATACAGATAAAAACACCCATTGTTGGAGTAATAGTGACAGCTCTAGTTACAGTAATGTTGATCATTTAGTTGGCATTCGGAATTTCAGCGTTGATGAAACTTTTTTAGTTAGGGATAGTAATAGGGACAGTTATTCCATATATTTTGATATTGAAAATCAAGTTTTTGAGATTGAAACTGATCATTCTTTTCTGAATGAACTAGAAAGTTCTTTTTATAGTTATTGGAATTCGAGTTATCTGAATAATGGGTCTAGCAGTGGTGACTTCCACTATGATCATTATATGTCTGATACTAAATATAGTTGGAATAATTACATCAATAGTTGTATTGACAATTATCTTCGCTCTCAAATCTGTATTGATTTAAGTGGTAGTAACAATTACAGTGAGAGTTACATTTATAGTTACATTTGTGGTGAAAGTGGAAATAGTAGTGAAAGTGAGAGTTCCAGTCTAAGAACTAGCACGAATGGTAGCGATTTAACTATAAGAGAAAGTTCTAATGATCTCGATATAACTCAAAAATACAAGCATTTGTGGGTTCAATGCGAAAATTGTTATGGATTAAACTATAAGAAATTTTTGAAGTCAAGAATGAATATTTGTGAACAATGTGGATATCATTTGAAAATGAGTAGTTCAGATAGAATTGAACTTTCGATTGACCTAGGCACTTGGAATCCTATGGATGAAGACATGGTATCTCTGGATCCCATTGAATTTCATTCAGAAGAGGAACCTTATAAAGATCGTATTGATTCTTATCAAAAAAAGACAGGATTAACCGAGGCTGTTCAAACAGGCACAGGTCAACTAAACGGTATTCCCGTAGCAATTGGGGTTATGGATTTTCAGTTTATGGGGGGTAGTATGGGATCCGTAGTAGGGGAAAAAATCACCCGTTTGATTGAGTATGCTACCAATAAATTTTTACCTCTTATTTTAGTGTGTGCTTCCGGAGGAGCACGCATGCAAGAAGGAAGTTTGAGCTTGATGCAAATGGCTAAAATATCTTCTGCTTTATATGATTATCAATCGAATAAAAAGTTATTTTATGTATCAATCCTTACATCTCCTACGACCGGCGGGGTGACAGCTAGTTTTGGTATGTTGGGGGATATCATTATTGCTGAACCTAACGCCTATATTGCATTTGCGGGTAAAAGAGTAATTGAACAAACATTGAATAAGACAGTACCTGAAGGTTCACAATCGGCTGAATTTTTATTCCATAAGGGTTTATTCGATCCAATCGTACCACGTAATCCTTTAAAAGGCGTTTTGAATGAGTTACTTCAGCTTCACGATTTCTTTCCTTTGAATCATAAATCAAGTAGAGCCTTAAGTTAA

>lcl|NC_012224.1_cds_YP_002720122.1_30 [gene=psaI] [locus_tag=JacuC_p030] [db_xref=GeneID:7564872] [protein=psaI] [protein_id=YP_002720122.1] [location=64938..65060] [gbkey=CDS]

ATGACAATTCTTAACAACCTACCCTCCATTTTTGTGCCTTTAGTGGGCTTAGTATTTCCGGCAATTGCAATGGCTTCTTTATCTCTTCATGTTCAAAAAACAAGATTGTTTAGATCTGATTAG

>lcl|NC_012224.1_cds_YP_002720123.1_31 [gene=ycf4] [locus_tag=JacuC_p031] [db_xref=GeneID:7564873] [protein=photosystem I assembly protein Ycf4] [protein_id=YP_002720123.1] [location=65493..66047] [gbkey=CDS]

ATGAGTTGGCGATCAGAACGTATATGGATAGAACTTATAGCGGGGTCTCGAAAAACAAGTAATTTCTGCTGGGCCCTTATACTTTTTTTAGGTTCATTGGGTTTTTTTTTGGTTGGAATTTCCAGTTATCTTGGCAAAAATTTGATATCTTTATTTCCATCTCAGCAAATAATTTTTTTTCCACAAGGGATCGTGATGTCTTTCTATGGGATCGCCGGTCTATTTATTAGTTCTTATTTGTGGTGCACAATTTTGTGGAATGTAGGTAGTGGTTATGATCGATTCGATAGAAAAGAAGGAATAGTGTGTATTTTTCGCTGGGGATTTCCTGGAAAAAATCGTCGCATCTTACTCCGATTCCTTATGAAAGATATTCAGTCTATTAGGCTAGAAGTTAAAGAGGGTATTTACGCTCGGCGTGCCCTTTATATGGAAATCCGAGGCCGGGGGGCCATTCCTTTGACTCGTACTGATGAGAATTTGACTCCGCGAGAAATTGAGCAAAAAGTAGCCGAATTGGCCTATTTTTTGCGTGTACCAATTGAAGTATTTTGA

>lcl|NC_012224.1_cds_YP_002720124.1_32 [gene=cemA] [locus_tag=JacuC_p032] [db_xref=GeneID:7564874] [protein=envelope membrane protein] [protein_id=YP_002720124.1] [location=66653..67342] [gbkey=CDS]

ATGAAAAGAAAAGCATTTATTCCCCTTCTATATCTTACATCTATAGTTTTTTTGCCCTGGTGGATCTCTTTTTTTTCTTTTAATAAAAGTTTTGAATCTTGGGTTATTAATTGGTGTAATACTAGTAAATCCGAAACTTTTTTAAATGATATCCAAGAAAAAAGTATTCTAGAAAAATTCATAGAATTAGAGGAACTCGTTCGCTTGGACGAAATGATAAAGGAATACCCGGAAACACATCTACAAGGGTTTCGTACCGGAATCCACAAAGAAACGATCCAATTGATCAAGATGCACAATGAAGATCGTATCCATACGATTTTACACTTCTCGACAAATATAATCTGTTTCGTTATTCTAAGTGGGTATTCTATTCTAAGTAATGAAGAACTTATTATTCTTAATTCGTGGGTTCAAGAATTCCTATATAACTTAAGCGACACAATAAAAGCTTTTTCAATTCTTTTATTAACCGATTTATGTATAGGATTCCACTCACCCCACGGTTGGGAACTAATGATTGGCTCTGTCTACAAAGATTTTGGATTTGCTCATAATGATCAAATTATATCTGGCCTTGTTTCCACTTTTCCAGTCATTCTCGATACAATTTTTAAATATTGGATTTTCCGTTATTTAAATCGTGTATCTCCGTCACTTGTAGTGATTTATCATTCAATGAATGACTGA

>lcl|NC_012224.1_cds_YP_002720125.1_33 [gene=petA] [locus_tag=JacuC_p033] [db_xref=GeneID:7564875] [protein=cytochrome f] [protein_id=YP_002720125.1] [location=67604..68566] [gbkey=CDS]

ATGCAAACTAGAAAGACCTTCTCTTGGATAAAGGAAGAGATTACTCGTTCCATTTCTGTATCGCTCATGGTATATATAATAACTTGGGCATCCATTTCAAACGCATATCCCATTTTTGCACAGCAGGGTTATGAAAATCCACGCGAAGCAACTGGCCGTATTGTATGTGCCAATTGTCATTTAGCTAATAAGCCCGTGGATATTGAGGTTCCACAGGCGGTACTTCCTGATACTGTATTTGAAGCAGTTGTTCGAATCCCTTATGATATGCAACTGAAACAAGTTCTTGCTAATGGTAAAAAAGGAGCTTTGAACGTGGGGGCTGTTCTTATTTTACCTGAGGGGTTTGAATTAGCCCCTCCCGATCGTATTTCGCCAGAGATTAAAGAAAAGATGGGAAATCTGTCTTTTCAGAGTTATCGCCCCACTAAAAAAAATATTCTTGTAATAGGCCCTGTTCCTGGTCAGAAATATAGTGAAATTACCTTTCCTATTCTGTCTCCGGACCCCGCCACTAAGAAAGATGTTCGTTTTTTAAAATATCCCATATATGTAGGCGGAAACAGGGGAAGGGGTCAGATTTATCCCGACGGGAGCAAGAGTAACAATACGGTTTATAATGCTACAGCAGCAGGTATAGTAAGCAAAATCATACGAAAAGAAAAAGGGGGGTACGAAATAACCATAACGGATGCGTCAGAGGGACGTCAAGTGATTGATATTATACCTCCAGGGCCAGAACTTCTTGTTTCAGAAGGCGAATCCATCAAACTGGATCAACCATTAACGAGTAATCCTAATGTGGGTGGATTTGGTCAGGGAGATGCAGAAATAGTGCTTCAAGACCCATTACGTGTCCAAGGGCTTTTTTTCTTCTTGGCATCCGTTATTTTGGCACAAATCTTTTTGGTTCTTAAAAAGAAACAGTTTGAGAAGGTTCAATTGTCCGAAATGAATTTTTAG

>lcl|NC_012224.1_cds_YP_002720126.1_34 [gene=psbJ] [locus_tag=JacuC_p034] [db_xref=GeneID:7564876] [protein=psbJ] [protein_id=YP_002720126.1] [location=complement(69656..69778)] [gbkey=CDS]

ATGGCCGATACTACTGGAAGAATTCCTCTTTGGATAATAGGTACTGTAACCGGTATTCTTGTGATCGGTTTAATAGGCATTTTCTTTTATGGTTCATATTCCGGATTGGGTTCATCCCTGTAA

>lcl|NC_012224.1_cds_YP_002720127.1_35 [gene=psbL] [locus_tag=JacuC_p035] [db_xref=GeneID:7564877] [protein=photosystem II protein L] [protein_id=YP_002720127.1] [location=complement(69928..70044)] [gbkey=CDS]

ATGACACAATCAAACCCGAACGAACAAAATGTTGAATTGAATCGTACCAGTCTCTACTGGGGGTTATTGCTCATTTTTGTACTTGCTGTTTTATTTTCTAATTATTTCTTCAATTAA

>lcl|NC_012224.1_cds_YP_002720128.1_36 [gene=psbF] [locus_tag=JacuC_p036] [db_xref=GeneID:7564878] [protein=photosystem II protein VI] [protein_id=YP_002720128.1] [location=complement(70071..70190)] [gbkey=CDS]

ATGACCATAGATCGAACCTATCCAATTTTTACAGTACGATGGTTGGCTGTTCACGGATTAGCTGTACCTACCGTTTCTTTTTTGGGGTCAATATCAGCAATGCAGTTCATCCAACGATAA

>lcl|NC_012224.1_cds_YP_002720129.1_37 [gene=psbE] [locus_tag=JacuC_p037] [db_xref=GeneID:7564879] [protein=photosystem II protein V] [protein_id=YP_002720129.1] [location=complement(70199..70450)] [gbkey=CDS]

ATGTCTGGAAGCACAGGAGAACGTTCTTTTGCTGATATTATTACCAGTATTCGATATTGGGTCATTCATAGCATTACTATACCTTCCCTATTCATTGCAGGTTGGTTATTCGTCAGCACGGGTTTAGCTTACGATGTATTTGGAAGCCCTCGTCCAAATGAATATTTTACAGAGAGCCGACAAGGAATTCCATTAATAACTGGCCGTTTTGATCCTTTGGAACAACTCGATGAATTTAGTAAATCTTTTTAG

>lcl|NC_012224.1_cds_YP_002720130.1_38 [gene=petL] [locus_tag=JacuC_p038] [db_xref=GeneID:7564880] [protein=cytochrome b6/f complex subunit VI] [protein_id=YP_002720130.1] [location=71834..71929] [gbkey=CDS]

ATGCCTACTATAACTAGTTATTTCGGTTTTCTACTAGCGGCTTTAACTATAACCTCAGTTCTATTTATTGGTCTGAGCAAGATACGGCTTATTTGA

>lcl|NC_012224.1_cds_YP_002720131.1_39 [gene=petG] [locus_tag=JacuC_p039] [db_xref=GeneID:7564881] [protein=cytochrome b6/f complex subunit V] [protein_id=YP_002720131.1] [location=72140..72253] [gbkey=CDS]

ATGATTGAAGTTCTTCTATTTGGAATCGTCTTAGGTCTAATTCCTATTACTTTGGCCGGATTATTCGTAACTGCATATTTACAATACAGACGTGGCGATCAATTGGACCTTTGA

>lcl|NC_012224.1_cds_YP_002720132.1_40 [gene=psaJ] [locus_tag=JacuC_p040] [db_xref=GeneID:7564754] [protein=photosystem I subunit IX] [protein_id=YP_002720132.1] [location=73101..73235] [gbkey=CDS]

ATGCGAGATCTAAAAACATATCTATCGGTGGCACCGGTAATAAGTACTCTATGGTTTGGGTCTTTAGCAGGTCTATTGATAGAGATCAATCGTTTTTTCCCAGATGCGTTGACATTCCCCTTTTTTTCATTCTAG

>lcl|NC_012224.1_cds_YP_002720133.1_41 [gene=rpl33] [locus_tag=JacuC_p041] [db_xref=GeneID:7564755] [protein=rpl33] [protein_id=YP_002720133.1] [location=73694..73894] [gbkey=CDS]

ATGGCCAAGGGTAAAGATGTCCGGGTAAGAGTTATTTTAGAATGTACCGGTTGTGTTCGAAACAGTGTTAATAAGAAATCAACAGGCATTTCAAAATATATTACTCAAAAGAATCGACACAATACGCCTAGTCGATTGGAATTGAGAAAATTCTGTCCTTATTGTTACAAACATACAATTCACGGGGAGATAAAGAAATAG

>lcl|NC_012224.1_cds_YP_002720134.1_42 [gene=rps18] [locus_tag=JacuC_p042] [db_xref=GeneID:7564756] [protein=ribosomal protein S18] [protein_id=YP_002720134.1] [location=74204..74530] [gbkey=CDS]

ATGGATAAATCCAAACGACTTTTTCTTAAGTCCAAGCGATCTTTTCGTAGGCGTTTGCCCCCGATCCAATCGGGGGATCGAATTGATTATAGAAACATGAGTTTAATTAGTCGATTTATTAGTGAACAAGGAAAAATATTATCTAGACGGGTGAATAGATTGAGTTTAAAACAACAACGATTAATTACTATTGCTATAAAGCAAGCTCGTATTTTATCTTCGTTACCTTTTCTTAATAATGAAAAACAGTTTGAAAAAAGCGAGTTGGTCACTATAACTACTGATCTTAGAACCAGAAAAAAAAAAATAGACTTACTCCTCAATTGA

>lcl|NC_012224.1_cds_YP_002720135.1_43 [gene=rpl20] [locus_tag=JacuC_p043] [db_xref=GeneID:7564757] [protein=ribosomal protein L20] [protein_id=YP_002720135.1] [location=complement(74821..75174)] [gbkey=CDS]

ATGACCAGAATTAGACGAGGATATATAGCTCGGAGGCGTAGAACAAAAATTCGTTTATTTGCATCAAGCTTTCGCGGGGCTCATTCAAGACTTACTCGAACTATTATTCAACAAAAAATAAGAGCTTTGATTTCGGCCCATCGGGATAGAGATAGGCAAAAAGGAAATTTTCGTCGTTTGTGGGTCACTCGGATAAATGCAGCAATTCGCGAGAATAAGGTATCCAAAAGTTATAGTAGATTAATAAACAATCTGTACAAGAGACAGTTGCTTCTTAATCGTAAAATACTTGCACAAATCGCTATAGTAAATAAGAATTGTCTTTATATGATTGCCAATGACATTATAAAATAA

>lcl|NC_012224.1_cds_YP_002720136.1_44 [gene=rps12] [locus_tag=JacuC_p044] [db_xref=GeneID:7564758] [protein=ribosomal protein S12] [exception=trans-splicing] [protein_id=YP_002720136.1] [location=join(complement(75975..76088),149328..149570)] [gbkey=CDS]

ATGCCAACAATTAAACAACTTATTAGAAACACAAGACAGCCAATCAGAAATGTCACTAAATCCCCCGCTCTTGGGGGATGTCCTCAGCGCCGAGGAACATGTACTAGGGTGTATACTATCACCCCCAAAAAACCAAACTCTGCCTTACGTAAAGTTGCCAGAGTACGATTAACCTCTGGATTTGAAATCACTGCTTATATACCTGGTATTGGCCATAATTCACAAGAACATTCTGTAGTCTTAGTAAGAGGGGGAAGGGTTAAGGATTTACCCGGTGTGAGATATCACATTGTTCGAGGAACCCTAGATGCTGTCGGAGTAAAGGATCGTCAACAAGGGCGTTCTAGTGCGTTGTAG

>lcl|NC_012224.1_cds_YP_002720137.1_45 [gene=clpP] [locus_tag=JacuC_p045] [db_xref=GeneID:7564759] [protein=ATP-dependent Clp protease proteolytic subunit] [protein_id=YP_002720137.1] [location=complement(join(76291..76518,77172..77462,78329..78397))] [gbkey=CDS]

ATGCCTATTGGTGTTCCAAAAGTCCCTTTTCGAAATCCTGGGGAAGACGATTCAATTTGGATTGACGTAAACCGACTTTATCGAGAAAGATTACTTTTTTTAGGTCAAGATGTTGATAGCGAAATCTCGAATCAACTTATTGGTCTTATGGTATATCTCAGTATAGAGAGCGAGACAAAAGATTTGTATTTGTTTATAAACTCTCCTGGCGGATGGGTAATACCCGGAATAGCTATTTATGATACTATGCAATTTGTGCGACCAGATGTACAAACAGTATGCATGGGATTAGCTGCTTCAATGGGATCTTTTATCCTGGTCGGAGGAAAAATTACCAAACGTTTAGCATTCCCTCATGCTAGGGTAATGATTCATCAACCTATTGCTGGTTTTTATGAGGCACAAATAGTAGAATTTGTCCTGGAAGCAGAAGAACTACTGAAACTGCGCGAAATCCTCACAAGGATTTATGCACAAAGAACGGGAAAACCCTTATGGATTGTATCCGAAGACATGGAAAGAGATGTTTTTATGTCAGCAACAGAAGCCCAAGCTCATGGAATTGTTGACCTTGTAGCAGTTGCCTAA

>lcl|NC_012224.1_cds_YP_002720138.1_46 [gene=psbB] [locus_tag=JacuC_p046] [db_xref=GeneID:7564760] [protein=photosystem II 47 kDa protein] [protein_id=YP_002720138.1] [location=78922..80448] [gbkey=CDS]

ATGGGTTTGCCTTGGTATCGTGTTCATACCGTCGTATTGAATGATCCCGGTCGTTTGCTGTCTGTCCATATAATGCATACAGCCTTGGTTGCTGGTTGGGCCGGTTCGATGGCTCTATATGAATTAGCAGTTTTTGATCCCTCTGACCCCGTTCTCGATCCAATGTGGAGACAGGGTATGTTCGTTATACCCTTCATGACTCGTTTAGGAATAACCAATTCGTGGGGTGGTTGGAGTATCACAGGAGGAACTATAACGAATCCGGGTATTTGGAGTTATGAAGGTGTGGCTGGGGCGCATATTGTGTTTTCTGGCTTGTGCTTTTTGGCAGCTATTTGGCATTGGGTGTATTGGGATCTAGAGGTATTTTGCGATGAACGTACAGGAAAACCTTCTTTAGATTTGCCCAAGATCTTTGGAATTCATTTATTTCTCTCAGGAGTGGCTTGCTTTGGGTTTGGTGCTTTTCATGTAACCGGATTGTATGGTCCTGGAATATGGGTGTCCGATCCTTATGGACTAACAGGAAAGGTACAACCTGTAAGTCCAGCATGGGGTGTGGAAGGTTTTGATCCTTTTGTTCCAGGAGGAATAGCTTCTCATCATATTGCAGCGGGGACATTGGGCATATTAGCGGGCCTCTTCCATCTTAGTGTCCGTCCGCCCCAACGTTTATACAAAGGATTGCGTATGGGAAATATTGAAACTGTCCTTTCCAGTAGTATCGCTGCTGTCTTTTTTGCAGCTTTTGTTGTTGCTGGAACTATGTGGTATGGTTCAGCAACTACCCCGATTGAATTATTTGGTCCTACTCGTTATCAATGGGATCAAGGATACTTCCAGCAAGAAATATATCGAAGAGTTAGTGCTGGGCTAGCCGAAAATCAAAGTTTATCCGAAGCTTGGTCTAAAATTCCCGAAAAATTAGCCTTTTATGATTACATCGGCAATAATCCGGCAAAAGGTGGATTGTTCAGAGCAGGCTCAATGGACAACGGGGATGGAATAGCTGTTGGGTGGTTAGGACATCCTATCTTTAGAGATAAAGAAGGGCGTGAACTTTTTGTACGGCGTATGCCTACTTTTTTTGAAACATTTCCTGTTGTTTTGGTAGATGGAGATGGAATTGTTAGAGCCGATGTTCCTTTTCGAAGGGCAGAGTCAAAGTATAGTGTCGAACAAGTAGGTGTAACTGTTGAGTTCTATGGTGGCGAACTAAACGGAGTCAGTTATAGTGATCCTGCTACTGTGAAAAAATATGCTAGACGCGCTCAATTGGGTGAAATTTTTGAATTAGATCGTGCTACTTTGAAATCTGATGGTGTTTTTCGTAGCAGTCCAAGGGGTTGGTTTACTTTTGGACATGCTTCGTTTGCTCTGCTCTTTTTCTTCGGACACATTTGGCACGGTGCTCGAACTTTGTTCAGAGATGTTTTTGCTGGTATTGATCCAGATTTAGACGCTCAAGTGGAATTTGGAGCATTCCAAAAACTTGGAGATCCAACTACAAGAAGACAAGTAGTCTGA

>lcl|NC_012224.1_cds_YP_002720139.1_47 [gene=psbT] [locus_tag=JacuC_p047] [db_xref=GeneID:7564761] [protein=photosystem II protein T] [protein_id=YP_002720139.1] [location=80633..80740] [gbkey=CDS]

ATGGAAGCATTGGTTTATACATTCCTTTTAGTCTCAACTTTAGGAATCATTTTTTTCGCTATCTTTTTTCGAGAACCGCCTAAAGTTCCAACTAAAAAGGTAAAATGA

>lcl|NC_012224.1_cds_YP_002720140.1_48 [gene=psbN] [locus_tag=JacuC_p048] [db_xref=GeneID:7564762] [protein=photosystem II protein N] [protein_id=YP_002720140.1] [location=complement(80802..80933)] [gbkey=CDS]

ATGGAAACAGCAACTCTAGTCGCCATCTCTATATCTGGTTTACTTGTAAGTTTTACTGGGTATGCCTTATATACTGCTTTTGGGCAACCCTCTCAACAACTAAGAGATCCATTCGAGGAACACGGGGACTAG

>lcl|NC_012224.1_cds_YP_002720141.1_49 [gene=psbH] [locus_tag=JacuC_p049] [db_xref=GeneID:7564763] [protein=photosystem II protein H] [protein_id=YP_002720141.1] [location=81037..81258] [gbkey=CDS]

ATGGCTACACAAAGTGTGGAGGGTAGTTCTAGATCTGGTCCAAGACGAACTGTTGTAGGGGATTTATTGAAACCATTGAATTCGGAATATGGTAAAGTAGCTCCTGGATGGGGAACCACTCCCTTGATGGGTGTTGCAATGGCTCTATTTGCGATATTCCTATCTATTATTTTAGAAATTTATAATTCTTCCGTTTTACTAGATGGAATTTCAATGAATTAG

>lcl|NC_012224.1_cds_YP_002720142.1_50 [gene=petB] [locus_tag=JacuC_p050] [db_xref=GeneID:7564764] [protein=cytochrome b6] [protein_id=YP_002720142.1] [location=join(81321..81326,82105..82746)] [gbkey=CDS]

ATGAGTAAAGTTTATGATTGGTTCGAAGAACGTCTCGAGATTCAGGCGATTGCAGATGATATAACTAGTAAATATGTTCCTCCCCATGTCAACATATTTTATTGTTTAGGAGGAATTACGCTTACTTGTTTTTTAGTACAAGTAGCTACGGGATTTGCTATGACTTTTTACTACCGTCCGACCGTTACTGAGGCTTTTGCTTCTGTTCAATACATAATGACTGAAGCTAACTTTGGTTGGTTAATCCGATCAGTTCATCGATGGTCGGCAAGTATGATGGTTTTAATGATGATCCTGCACGTATTTCGTGTGTATCTCACTGGTGGCTTTAAAAAACCTCGTGAATTGACTTGGGTTACGGGCGTGGTTCTTGCTGTATTGACCGCATCTTTTGGTGTAACCGGTTATTCCTTACCTTGGGACCAAATTGGTTATTGGGCAGTCAAAATTGTAACAGGCGTGCCGGAAGCTATTCCTGTAATAGGATCGCCTTTGGTAGAGTTATTACGTGGAAGCGCTAGTGTAGGACAATCCACTTTGACTCGTTTTTATAGTTTACACACTTTTGTATTACCCCTCCTTACTGCCGTATTTATGTTAATGCACTTTTCAATGATACGTAAGCAAGGTATTTCAGGCCCTTTATAG

>lcl|NC_012224.1_cds_YP_002720143.1_51 [gene=petD] [locus_tag=JacuC_p051] [db_xref=GeneID:7564765] [protein=cytochrome b6/f complex subunit IV] [protein_id=YP_002720143.1] [location=join(82940..82947,83809..84304)] [gbkey=CDS]

ATGGGAGTAACAAAAAAACCTGACTTGAATGATCCTGTATTAAGAGCTAAATTGGCTAAGGGAATGGGTCATAATTATTACGGAGAACCTGCATGGCCCAATGATCTTTTATATATTTTTCCAGTAGTAATTCTAGGTACTATTGCGTGTAATGTAGGATTAGCGGTTCTAGAGCCGTCAATGATTGGTGAACCTGCGGATCCATTTGCAACTCCTTTAGAGATATTGCCTGAATGGTATTTTTTTCCTGTATTTCAAATACTTCGTACAGTACCCAATAAGTTATTGGGTGTTCTTTTAATGGTTTCAGTACCTGCAGGATTATTAACAGTACCCTTTTTGGAGAATGTTAATAAATTCCAAAATCCATTTCGTCGCCCAGTTGCGACAACCGTCTTTTTGATTGGTACTGCAGTAGCCCTTTGGTTAGGTATTGGAGCAACATTACCTATTGATAAATCCCTAACTTTAGGTCTTTTTCAAATTGATTCAATTGTAAAATAA

>lcl|NC_012224.1_cds_YP_002720144.1_52 [gene=rpoA] [locus_tag=JacuC_p052] [db_xref=GeneID:7564766] [protein=RNA polymerase alpha subunit] [protein_id=YP_002720144.1] [location=complement(84516..85535)] [gbkey=CDS]

ATGGTTCGAGAGAAAGTAACAATATCTACTCGGACACTGCAGTGGAAATGTGTTGAATCAAGAACGGACAATAAACGTCTTTATTATGGACGCTTTATTCTATCTCCACTTATGAAAGGCCAAGCTGACACAATAGGCATTGCGATGCGAAGAGCTTTGCTTGGAGAAATAGAAGGAACATGTATCACACGTGCAAAATCTGAGAAAATACCACACGAATTTTCTACTATAGCAGGTATTCAAGAATCAATACATGAAATTTTAATGAATTTGAAAAATGTTGTATTGAAAAGCAATTTGTATGGAACTTGTGACGCGTCTATTTATGTCAAAGGTCCTGGATATGTAACTGCCCAAGACATCATCTTACCGCCTTTTGTGGAAATTATTGATAATACACAGCATATCGCTAGCCTAACGGAATCAATTGATTTGTGTATTGGATTACAAATCGAGAGGAATCGCGGCTATCGTATAAAACCGACAAATAACTTTCAGGACGGAAGTTATCCTATAGATGCTGTATTCATGCCTGTTCGAAATGCGAATCATAGTGTTCATTCTTATGGAAATGGAAATGAAAAGCAAGAGATACTTTTTCTCGAAATATGGACAAACGGAAGTTTAACTCCTAAAGAAGCACTTCATGAAGCCTCCCGAAATTTGATTGATTTATTTATTCCTTTTCTACATGCAGAAGAAGAAAACTTACATTTAGAAATAAATCAACACAAGGTTACTTTACCCCTTTTTACTTTTCATGGTAGATTGACTAAATTAAGAAAAAATCAAAAAGAAATAGCATTGAAATACATTTTTATTGACCAATCAGAATTGACCCCTAAGACCTATAATTGCCTCAAAAGGTCCAATATACATACATTATCGGACCTTTTAAATAAGAGTCAAGAAGATCTTATGAAAATTGAAAATTTTCGCATAGACGATGTAAAACATATATTGGGTATTCTAGAAATAGAAAAACATTTCGCAATTGATTTACCAAAGAATAAAATATAA

>lcl|NC_012224.1_cds_YP_002720145.1_53 [gene=rps11] [locus_tag=JacuC_p053] [db_xref=GeneID:7564767] [protein=ribosomal protein S11] [protein_id=YP_002720145.1] [location=complement(85598..86014)] [gbkey=CDS]

ATGGCAAAACCTTTACCAAGAATTGGTTCACGCAGAAATGGGCGTGTTGGCTCACGTAAGAATGCGCGTAAAATACCAAAAGGGGTTATTCATGTTCAAGCAAGTTTCAACAACACTATTGTGACCGTTACAGACGTACGGGGTCGAGTAATTTCTTGGTCCTCCGCTGGCACTTGCGGATTCAGGGGCACAAGAAGAGGCACGCCATTTGCTGCTCAAACCGCAGCAGGCAATGCTATTCGGACAGTAATGGATCAAGGTATGCAACAAGCAGAAGTGATGATAAAAGGCCCTGGTCTCGGACGAGATGCGGCATTAAGAGCTATTCGCAGAAGTGGTATACTATTAAGTTTCGTCCGGGATATAACCCCTATGCCACATAATGGCTGCAGGCCCCCTAAAAAAAGGCGTGTGTAA

>lcl|NC_012224.1_cds_YP_002720146.1_54 [gene=rpl36] [locus_tag=JacuC_p054] [db_xref=GeneID:7564768] [protein=ribosomal protein L36] [protein_id=YP_002720146.1] [location=complement(86130..86243)] [gbkey=CDS]

ATGAAAATAAGAGCTTCTGTTCGTAAAATTTGTGAAAAATGTCGACTGATACGTAGACGGGGACGAATTATAGTAATTTGCTTCAACCCAAGACATAAACAAAGACAAGGATAA

>lcl|NC_012224.1_cds_YP_002720147.1_55 [gene=rps8] [locus_tag=JacuC_p055] [db_xref=GeneID:7564769] [protein=ribosomal protein S8] [protein_id=YP_002720147.1] [location=complement(86689..87093)] [gbkey=CDS]

ATGGGTAGGGATTCTATTGCTGAAATAATAACCTCTATACGAAATACTGACATGAATAGAAAAGGAACTGTTCGAATAGCATCTACTAATATCACCGAAAACATTATTAAAATACTTTTACGAGAAGGTTTTATTGAAAATGTCAGGAAACATCAGGAAGGCAACAAAAAATTTTTGGTCTTAACCCTACGACATAGAAGGAAGATGAAAGGGCCATATAGAACTAGTCTAAATTTAAAACGAATCAGCCGACCTAGTCTACGAATCTATCCTAACTATCAAAAAATTCCTAGAATTTTGGGCGGGATGGGGATTGTAATTCTTTCTACTTCTCGGGGTATAATGACAGACCGAGAAGCTCGACTAGAAAGAATCGGTGGAGAAATCTTGTGTTATATATGGTAA

>lcl|NC_012224.1_cds_YP_002720148.1_56 [gene=rpl14] [locus_tag=JacuC_p056] [db_xref=GeneID:7564770] [protein=ribosomal protein L14] [protein_id=YP_002720148.1] [location=complement(87606..87974)] [gbkey=CDS]

ATGATCCAATCTCAAACCCATTTGAATGTAGCAGATAACAGTGGAGCCCGAGAATTGATGTGTATTCGAATCATGGGGACTAGTAATCGACGATATGCTCATATTGGTGACGTTATTGTTGCTGTGATCAAGGAAGCAACACCAAATTCACCTCTAGAAAGATCCGAAGTAATAAGAGCTGTAATTGTACGTACTTGTAAAGAACTCAAACGCGATAACGGTATAATAATACGATATGATGATAATGCTGCAGTTGTTATTGATCAAGAAGGAAATCCAAAAGGAACTCGAATTTTTGGTGCAATCGCCCGGGAATTGAGACAGTTAAATTTTACTAAAATAGTTTCATTAGCACCCGAAGTATTATAA

>lcl|NC_012224.1_cds_YP_002720149.1_57 [gene=rpl16] [locus_tag=JacuC_p057] [db_xref=GeneID:7564771] [protein=ribosomal protein L16] [protein_id=YP_002720149.1] [location=complement(join(88089..88487,89883..89891))] [gbkey=CDS]

ATGCTTAGTCCCCAAAGGCCCCGATTCCGCAAACAGCATAGAGGACGAATGAAAGGAAAAGCTTTTCGAGCTAATCGTATTTCTTTCGGTAGATATGCTCTTCAGGCACTTGAACCTTCTTGGATTACATCTAGGCAAATAGAAGCGGGGCGACGAACAATGACACGAAATGCACGTCGCGGTGGAAAAATATGGATACGTATATTTCCCGACAAACCAATTACTTTAAGACCTACGGAAACACGTATGGGTTCGGGGAAAGGATCTCCCGAATATTGGGTAGCTGTCGTTAAACCAGGTAGAATCCTTTATGAAATGGGTGGAGTAGCAGAAAATATAGCGAGAAAATCTATTTCAATAACAGCATCAAAAATGCCTGTACGAACTCAACTCATTATTTCGGTATAG

>lcl|NC_012224.1_cds_YP_002720150.1_58 [gene=rps3] [locus_tag=JacuC_p058] [db_xref=GeneID:7564772] [protein=rps3] [protein_id=YP_002720150.1] [location=complement(90100..90765)] [gbkey=CDS]

ATGGGACAAAAAATAAATCCACTTGGTTTCAGACTTGGTACAACTCAAAGTCATCATTCTCTTTGGTTTGCACAACCAAAAAATTATTCTGAGGATCTACAAGAAGATCAAAAAATAAGAAACTGTATCAAAAATTATGTAAAACAAAATACGAAAATATCTTCTGGTGTTGAGGGAATTGCACGTATAGAGATTCAAAAACGAATCGATGTGATTCAGGTCATGATATATATGGGATTCCAAAAATTATTAATGGAAGGTAGACCTAAACAAATCGAAGAATTACAGATGAATGTACAAAAAGAACTTAATTATGTGAACCGAAAACTCAATATTGCTATTAAAATTAAAAGAATTTCAAATCCTTATGGGCAACCTAATATTCTTGCAGAATTTATAGCCGGGCAGTTAAAGAATAGAGTTTCATTTCGCAAAGCAATGAAAAAAGCTATTGAATTAACTGAACAAACGGATACAAAAGGAATTCAAGTCCAAATTGCGGGGCGTCTTGACGGAAAAGAAATTGCACGCGTCGAATGGATTAGAGAGGGTAGAGTTCCTTTACAAACCATTCAAGCTAAAATTGAGTATTGTTCGTATACAGTTAAAACTATTTATGGGGTATTAGGCATAAAAATTTGGACATTTCTAGATAAAAAAGAATAA

>lcl|NC_012224.1_cds_YP_002720151.1_59 [gene=rpl22] [locus_tag=JacuC_p059] [db_xref=GeneID:7564773] [protein=ribosomal protein L22] [protein_id=YP_002720151.1] [location=complement(90862..91287)] [gbkey=CDS]

ATGATAAATAAAAGAAAGCGAAAGAGAGACCCATATACGGAAGTATATGCTTTAGGCCAACATATATGTATGTCCGCTCACAAAGCACGAAGAATAATTGATCAGATTCGTGGACGTTCTTATGAAGAAACACTTATGATACTTGAACTCATGCCCTATCGAGCATGTTATCCCATTTTTAAATTGATTTATTCTGCAGGAGCAAATGCTAGTCACAATATGGGTTTCAACGAAGCCAATTTAATCATTAGTAAAGCTGAAGTCAACGAAAGTGCTACTGTGAAAAAATTAAAACCTCAGGCTCGAGGGCGGGGTTATCTGATAAAAAGATCGACTTGTCATATAACTATTGTATTAAAAGATATATCCTTCTATGAAGAATATGAAGAATATGACAAATATCTAGGAAGAGTATTAAAAATATGA

>lcl|NC_012224.1_cds_YP_002720152.1_60 [gene=rps19] [locus_tag=JacuC_p060] [db_xref=GeneID:7564774] [protein=rps19] [protein_id=YP_002720152.1] [location=complement(91383..91661)] [gbkey=CDS]

GTGACACGTTCACTAAAAAAAAACCCTTTTGTAGCAAATCATTTATTAAAAAAAATAAATAAGCTTAACACAAAAGCAGAAAAAGAAATAATAGCAACGTGGTCCCGGGCATCTACCATTATACCCACAATGATCGGCCATACTATTGCTATCCATAATGGAAAGGGGCATTTACCTATTTATATAACGGATCGCATGGTGGGCCATAAATTGGGAGAATTTTCACCTACTTTAAATTTCCGGGAACATGCAAAAAATGATAATAAATCTCGTCGTTAA

>lcl|NC_012224.1_cds_YP_002720153.1_61 [gene=rpl2] [locus_tag=JacuC_p061] [db_xref=GeneID:7564775] [protein=ribosomal protein L2] [protein_id=YP_002720153.1] [location=complement(join(91732..92202,92832..93224))] [gbkey=CDS]

ATGGCGATACATTTATACAAAACTTCTACCCCGAGCACACGCAATGGAGCCGTAGACAGTCAAGTGAAATCCAATACACGAAATAATTTGATCTATGGACAGCATCGTTGTGGTAAAGGACGTAATGCCAGAGGAATCATTACCGCAAGGCATAGAGGGGGGGGTCATAAGCGTCTATACCGTAAAATCGATTTTCGACGGAATGAAAAAGACATATATGGTAGAATCGTAACCATAGAATACGACCCTAATCGAAATGCATACATTTGTCTCATACACTATGGGGATGGTGAGAAGAGATATATTTTACATCCCAGAGGGGCTATAATTGGAGATACCATTATTTCTGGTACAGAAGTTCCTATAAAAATGGGAAATGCCCTACCTTTGAGTGAGGTTTTGATTGATCAAAAAGAAGAATCTACTTCAACCGATATGCCCTTAGGCACGGCCATACATAACATAGAAATCACACTTGGAAAGGGTGGACAATTAGCTAGAGCTGCAGGTGCTGTAGCGAAACTGATTGCAAAAGAGGGGAAATCGGCCACATTAAAATTACCTTCTGGGGAGGTTCGTTTAATATCCAAAAACTGCTCAGCAACAGTCGGACAAGTAGGCAATACTGGGGTGAACCAGAAAAGTTTGGGTAGAGCCGGATCTAAATGTTGGCTAGGTAAGCGTCCTGTAGTAAGAGGAGTAGTTATGAACCCTGTAGACCATCCCCATGGGGGTGGTGAAGGGAGGGCCCCAATTGGTAGAAAAAAACCCGCAACCCCTTGGGGGTATCCTGCACTTGGAAGAAGAAGTAGAAAAAGGAATAAATATAGTGATAATTTGATTCTTCGTCGCCGTACTAAGTAG

>lcl|NC_012224.1_cds_YP_002720154.1_62 [gene=rpl23] [locus_tag=JacuC_p062] [db_xref=GeneID:7564776] [protein=ribosomal protein L23] [protein_id=YP_002720154.1] [location=complement(93243..93524)] [gbkey=CDS]

ATGGATGGAATCAAATATGCAGTATTTACAGACAAAAGTATTCGGTTATTGGGGAAAAATCAATATACTTTTAATGTCGAATCAGGATCAACTAGGACAGAAATAAAGCATTGGGTCGAGCTCTTCTTTGGTGTCAAGGTAATAGCTATGAATAGCCATCGACTCCCGGGAAAGGGTAGAAGAATGAGACCTATTATGGGACATACAATGCATTACAGACGTATGATCATTACGCTTCAACCGGGTTATTCTATTCCACCTCTTAGAAAGAAAAGAACTTAA

>lcl|NC_012224.1_cds_YP_002720155.1_63 [gene=ycf2] [locus_tag=JacuC_p063] [db_xref=GeneID:7564778] [protein=Ycf2] [protein_id=YP_002720155.1] [location=93852..100748] [gbkey=CDS]

ATGAAAGGACATCAATTCAAATCCTGGATTTTCGAATTGAGAGAGATATTGAGAGAGATCAAGAATTCTCACTATTTCTTAGATTCATGGACCCAATTCAATTCAGTGGGATCTTTCATTCACATTTTTTTCCATCAAGAACGTTTTATAAAACTCTTGGACTCCCGAATTTGGAGTATCTTACTTTCACGCAATTCACAGGGTTCAACAAGCAATCGATATTTCACGATCAAGGGTGTAGTACTATTTGTAGTAGTGGTTCTTATATATCGTATTAACAATCGAAAGATGGTCGAAAGAAAAAATCTCTATTTGACAGGGCTTCTTCCTATACCTATGAATTCCATTGGACCCAGAAATGATACATTGGAAGAATCCTTTGGGTCTTCCAATATCAATAGGTTGATTGTTTCGCTCCTGTATCTTCCAAAAGGAAAAAAGATCTCTGAGAGCTCTTTCCTGGATCCGAAAGAGAGTACTTGGGTTCTCCCAATAACTAAAAAGTCTGAATCTAACTGGGGTTCGCGGTGGTGGAGGAACTGGATCGGAAAAAAGAGGGATTCTAGTTGTAAGATATCTAATGAAACCGTCGCTGGAATTGAGATCTCATTCAAAGAAAAAGATATCAAATATCTGGAGTTTCTTTTTGTATATTATATGGATGATCCGATCCGCAAGGACCATGATTGGGAATTGTTTGATCGTCTTTCTCCGAGGAAGGGGCGAAACATAATCAACTTGAATTCGGGACAGCTATTCGAAATCTTAGTGAAAGACTGGATTTGTTATCTCATGTTTGCTTTTCGCGAAAAAATACCAATTGAAGTGGAGGGTTTCTTCAAACAACAAGGAGCTGGGTCAACTATTCAATCAAATGATATTGAGCATGTTTCCCATCTCTTCTCGAGAAAGAAGTGGGCTATTTCTTTGCAAAATTGTGCTCAATTTCATATGTGGCAATTCCGCCAAGATCTCTTCGTTAGTTGGGGGAATAATCCGCACGAATCGGATTTTTTGAGGAACATATCGAGAGAGGATTGGATTTGGTTAGACAATGTGTGGTTGGTAAACAAGGATCGGTTTTTTAGCAAGGCACGGAATATATCGTCAAATATTCAATATGATTCCACAAGATCTAGTTTCGTTCAAGGAAGGAATTCTAGCCAATTGAAGGGATCTTCTGATCAATCCAGAGATCATTTCGATTCCATTAGTAATGAGGATTCGGAATATCACACATTGATCAATCAAAGAAAGATTCAACAACTAAAAGAAAGATCGATTCTTTGGGATCCTTCCTTTCTTCAAACGGAACGAACAGAGATAGAATCAGACCGATTCCCTAAATGCCTTTCTGGATATTCCTCAATGTCCCGACTATTCACGGAAGGTGAGAAGGAGATGAATAATCATCTGCTTCCGGAAGAAATCGAAGAATTTCTTGGGAATCCTACAAGATCCATTCGTTCTTTTTTCTCTGACAGATCGTCAGAACTTCATCTGGGTTCGAATCCTACTGAGAGGTCCACTAAATTGTTGAAGAAAGAACAAGATGTTTCTTTTGTCCCTTCCAGGCGATCGGAAAATAAAGAAATAGTTAATATATTCAAGATAATCACGTATTTACAAAATACCGTCTCAATTCATCCTATTTCATCAGATCCGGGATGTGATATGGTTCTGAAGGATGAACTGGATATGGACAGTTCCAATAAGATTTCTTTCTTGAACAAAAATCCATTTTTTGATTTATTTCATCTATTCCATGATCGGAACGGGGGGGGATACACGTTACACCACGATTTTGAATCAGAAGAGAGATTTCAAGAAATGGCAGATCTATTCACTCTATCAATAACCGAGCCGGATCTGGTGTATCATAAGGGATTTACCTTTTTTATTGATTCTTACGGATTGGATCAAAAACAATTCTTGAATGAGGTATTCAACTCCAGGGATGAATCGAAAAAGAAATCTTTATTGGTTCTACCTCCTATTTTTTATGAAGAGAATGAATCTTTTTATCGAAGGATCAGAAAAAAATGGGTCCGGATCTCCTGCGGGAATGATTTGGAAGATCCAAAACAAAAAATAGTGGTATTTGCTAGCAACAACATAATGGAGGCAGTCAATCAATATGGATTGATCCTAAATCTGATTCAAATCCAATATAGTACCTATGGGTACATAAGAAATGTATTGACTCAATTCTTTTTAATGAATAGATCCGATCGCAACTTCGAATATGGAATTCAAAGGGATCAAATAGGAAATGATACTCTGAATCATAGAACTATAATGAAATATACGATCAACCAACATTTATCGAATTTGAAACAGAGTCAGAAGAACTGGTTCGATCCTCTTATTTTTCTTTCTCGAACCGAGAGATCCATGAATTGGGATCCTAATGCATATAGATACAAATGGTCTAATGGGAGCAAGAATTTCCAGGAATATTTGGAACATTTCATTTCTGAGCAGAAGAGCCGTTTTCTTTTTCAAGTAGTGTTCGATCGATTACGTATTAATCAATATTCGATTGATTGGTCTGAGGTTATCGACAAAAAAGATTTGTCTAAGTCACTTCGTTTCTTTTTGTCCAAGTTACTTGTTTTTTTGTCCAAGTTTCTTCTCTTTTTGTCTAACTCACTTCCTTTTTTCTTTGTGAGTTTCGGGAATATCCCCATTCATAGGTCCGAAATCCATATCTATGAATTGAAAGGTCCGAATGATCCACTCTGCAATCAGCTGTTAGAACCAATAGGTCTTCAAATCGTTCATTTGAAAAAATGGAAACCCTTCTTATTGGATGATCATGATACTTCCCAAAAATCGAAATTTTTGATTAATGGAGGAACAATATCACCATTTTTGTTCAATAAGATACCAAAGTGGATGATTGACTCATTCCATACTAGAAATAATCGCAGGAAATCTTTTGATAACACGGATTCCTATTTCTCAATGATATCCCACGATCAAGACAATTGGCTGAATCCCGTGAAACCATTTCATAGAAGTTCATTGATATCTTCTTTTTATAAAACAAATCGACTTCGATTCTTGAATAATCTACATCACTTCTGCTTCTATTGTAACAAAAACAAAAGATTCCCTTTTTATGTGGAAAAGGCCCGTATCAAGAATTATGATTTTACGTATGGACAATTCCTCAATATCTTGTTCATTCGCAACAAAATATTTTCTTTGTGCGGCGGTAAAAAAAAACATGCTTTTGGGGAGAGAGATACTATTTCACCAATCGAGTCACAGGTATCTAACATATTCATACCTAATGATTTTCCACAAAGTGGTAACGAAAGGTATAACTTGTACAAATCTTTCCATTTTCCAATTCGATCCGATCCATTCGTTCGTAGAGCTATTTATTTGATCGCAGACATTTCGGGAACACCTCTAACAGAGGGACAAATAGTCAATTTTGAAAGAACTTATTGTCAACCTCTTTCGGATATGAATCTATCTGATTCAGAAGGGAAGAACTTGCATCAGTATCTCAATTTCAATTCAAACATGGGTTTGATTCACACTCCATGTTCTGAGAAATATTTACCATCCGAAAAGAGGAAAAAACGGAGTCTTTGTCTAAAGAAATGTGTTGAAAAAGGGCAGATGTATAGAACCTTTCAACGAGATAGTGCTTTTTCAACTCTCTCAAAATGGAATCTATTCCAAACATATATGCCATGGTTCCTTACTTCGACAGGGTACAAATATCTAAATTTGATATTTTTAGATACCTTTTCGGACCTATTACCGATACTAAGTAGCAGTCAAAAATTTGTATCCATTTTTCATGATATTATGCACGGATCAGATATATCATGGCGAATTCTTCAGAAAAAATGGTGTCTTCCACAATGGAATCTGATAAGTGAGATTTCGAGTAAGTGTTTACATAATCTTCTTCTGTCCGAAGAAATGATTCATCGAAATAATGAGCCACCATTGATATCGACACATCTGAGATCGCCAAATGTTCGGGAGTTCCTCTATTCAATCCTTTTCCTTCTTCTTGTTGCTGGATATCTCGTTCGTACACATCTTCTCTTTGTTTCCCGAGCCTATAGTGAGTTACAGACAGAGTTCGAAAAGGTCAAATCTTTGATGATTCCATCATACATGATTGAGTTGCGAAAACTTCTGGATAGGTATCCTACATCTGAACTGAATTCTTTCTGGTTAAAGAATCTCTTTCTAGTTGCTCTGGAACAATTAGGAGATTTTCTAGAAGAAATGCGGGGTTTTGCTTCTGGCGGCAACATGCTATGGGGTGGTGGTCCCGCTTATGGGGTTAAATCAATACGTTCTAAGAATAAATTTTTGAATATCAATCTCATCGATCTCATAAGTATCATACCAAATCCCATCAATCGAATCACTTTTTCGAGAAATACGAGACATCTAAGTCATACAAGTAAAGAGATTTATTCATTGATAAGAAAAAGAAAAAACGTGAATGGTGATTGGATTGATGATAAAATAGAATCCTTGGTCGCGAACAGTGATTCGATTGATGATAAAGAAAGAGAATTCTTGGTTCAGTTCTCCACCTTAACGACAGAAAACAGGATTGATCAAATTCTATTGAGTCTGACGCATAGTGATCATTTATCAAAGAATGACTCTGGTTATCAAATGATTGAAGAGCCGGGAGCAATTTATTTACGATACTTAGTTGACATTCATAAAAAGTATCTAATGAATTATGAGTTCAATACACCCTGTTTAGCAGAAAGACGGATATTCCTTGCTTATTATCAGACAACCACTTATTCACAAACCTCGTGTGGGGTGAATAGTTTTCATTTCCCATCTCATGGAAAACCCTTTTCGCTCCGCTTAGCCCTATCCCCCTCTAGGGGTATTTTAGTGATAGGTTCTATAGGAACTGGACGATCCTATTTGGTCAAATACCTAGCGACAAACTCCTATCTTCCTTTCATTACAGTATTTCTGAACAAGTTCCTGGATAACAAGCCTAAGGGCTTTCTTATTGATGATAGTGACGATATTGATGATAGTGACGATATTGATGATAGTGACGATATTGATGATAGTGACGATATTGATGTGAGTGACGATATTGATGATAGTGACGATATCGACCGTGACTTTGATACGGAGCTGGAGTTTCTAACTAGGATGAATGCGCTAACTATGGGTATGATGCCGGAAATAGACCGATTTTATATCACCCTTCAATTCGAATTAGCAAAAGCAATGTCTCCTTGCATAATATGGATTCCAAACATTCATGATTTGGATGTGAATGAGTCGAATTACTTATCCCTCGGTCTATTAGTGAACTATCTCTCCAGGGATTGTGAAAGATGTTCCACTAGAAATATTCTTGTTATTGCTTCGACTCATATTCCCCAAAAAGTGGATCCCGCTCTAATAGCTCCGAATAAATTAAATACATGCATTAAGATACGAAGGCTTCTTATTCCACAACAACGAAAGCACTTTTTTACTCTTTCATATACTAGGGGATTTCACTTGGAAAAGAAAATGCTCCATACTAATGGATTCGGGTCCATAACCATGGGTTCCAATGTACGAGATCTTGTAGCACTTACCAATGAGGCCCTATCGATTAGTATTACACAGAAGAAATCAATTCTAGACACTAATATAATTAGATCCGCTCTTCATAGACAAACTTGGGATTTGCGATCCCAGGTAAGATCGGTTCAGGATCATGGGATCCTTTTCTATCAGATAGGAAGGGCTGTTGCACAAAATGTATTTCTAAGTAATTGCCCCATAGATCCTATATCTATCTATATGAAGAAGAAATCATGTAACGAAGGGGATTCTTATTTGTACAAATGGTACTTCGAACTTGGAATGAGCATGAAGAAATTAACGATACTTCTTTATCTTTTGAGTTGTTCCGCCGGATCGGTTGCTCAAGACCTTTGGTCTCTACCCGGACCCGATGAAAAAAATGGGATCACTTATTATGGACTTGTTGAGAATGATTCTGATCTAGTTCATGGCCTATTAGAAGTCGAAGGCGCTCTGGTGGGATCCTCACGTACAGAAAAAGATTGCAGTCAGTTTGATAATGATCGAGTGACATTGCTTCTTCGGCCCGAACCAAAGAGTCCCTTAGATATGATGCAAAATGGATCTTGTTCTATCCTTGATCAGAGATTTCTCTATGAAAAATACGAATCGGAGTTTGAAGAAGGGGAAGGAGTCCTCGACCCGCAACAGATAGAGGAGGATTTATTCAATCACATAGTTTGGGCTCCTAGAATATGGCGCCCTTGGGGTTTTCTATTTGATTGTATCGAAAGGCCCAATGAATTGGGATTTCCCTATTGGGCCAGGTCATTTCGGGGCAAGCGGATCATTTATGATGAAGAGGATGAGCTTCAAGAGAATGATTCGGAGTTCTTGCAGAGTGGAACCATGCAGTACCAGATACGAGATAGATCTTCCAAAGAACAAGGCTTTTTTCGAATAAGCCAATTCATTTGGGACCCTGCGGATCCACTCTTTTTCCTATTCAAAGATCAGCCCTTTGTCTCTGTGTTTTCACATCGAGAATTCTTTGCAGATGAAGAGATGTCAAAGGGGCTTCTTACTTCCCAAACAGATCCTCCTACATCTATATATAAACGCTGGTTTATCAAGAATACGCAAGAAAAGCACTTCGAATTGTTGATTCATCGCCAGAGATGGCTTAGAACCAATAGTTCATTATCTAATGGATTTTTCCGTTCTAATACTCTATCCGAGAGTTATCAGTATTTATCAAATCTGTTCCTATCTAACGGAACGCTATTGGATCAAATGACAAAGGCATTGTTGAGAAAAAGATGGCTTTTCCCGGATGAAATGAAAATTGGATTCATGTAA

>lcl|NC_012224.1_cds_YP_002720156.1_64 [gene=ORF126] [locus_tag=JacuC_p064] [db_xref=GeneID:7564779] [protein=ORF126] [protein_id=YP_002720156.1] [location=complement(101219..101599)] [gbkey=CDS]

ATGAATGGGGAGTCCGCTTTGAAAGCGTCCGCCCTGCAACCACCCCCGAGTATATGCTTCAACAGGAATTACACAAGGGTAGTTGATACAATAGAAACCTCTGGTAAAATGCCCGCCCGTAACCCAACAGATAAAGTACATTACATAGTCCGTTTTAGGGATTGGCGACTTACCCATTCAGTGACTTTGGCACTGGATGTTCCAAAAAGAAAATGGGTACTCTCGGGTCGGGTGAATTCAATAATAGACGTCTGTTGGCATTCCAGCCTTCCTTCTCCTTTCAGGGCCTATCCGAAAGAGAATCCAGTACTTCTTGGTCGTGAATATCTGAATAGGACAAACCGCCCCGTGGATATCTTTGCTTCGGAACAAAACAATTAG

>lcl|NC_012224.1_cds_YP_002720157.1_65 [gene=ndhB] [locus_tag=JacuC_p065] [db_xref=GeneID:7564781] [protein=NADH dehydrogenase subunit 2] [protein_id=YP_002720157.1] [location=complement(join(102419..103168,103851..104627))] [gbkey=CDS]

ATGATCTGGCATGTACAGAATGAAAACTTCATTCTCGATTCTACGAGAATTTTTATGAAAGCCTTTCATTTGCTTCTCTTCGATGGAAGTTTTATTTTCCCAGAATGTATCCTAATTTTTGGCCTAATTCTTCTTCTGATGATCGATTCAACCTCTGATCAAAAAGATATACCTTGGTTATATTTCATCTCTTCAACAAGTTTAGTAATGAGTATAACGGCCCTATTGTTCCGATGGAGAGAAGAACCTATGATTAGCTTTTCGGGAAATTTCCAAACGAACAATTTCAACGAAATCTTTCAATTTCTTATTTTACTATGTTCCACTCTATGTATTCCTCTATCCGTAGAGTACATTGAATGTACAGAAATGGCTATAACAGAGTTTCTCTTATTCCTATTAACAGCTACTCTAGGAGGAATGTTTTTATGCGGTGCTAACGATTTAATAACTATCTTTGTAGCTCCAGAATGTTTCAGTTTATGCTCCTACCTATTATCTGGATATACCAAGAAAGATGTACGGTCTAATGAGGCTACTACGAAATATTTACTCATGGGTGGGGCAAGCTCTTCTATTCTGGTTCATGCTTTCTCTTGGCTATATGGTTCGTCCGGGGGAGAGATCGAGCTTCAAGAAATAGTGAATGGCCTTATCAATACACAAATGTATAACTCCCCAGGAATTTCAATTGCGCTTATATTCATCACTGTAGGAATCGGGTTCAAGCTTTCCCCAGCCCCTTCTCATCAATGGACTCCTGACGTATACGAAGGATCTCCCACTCCAGTCGTTGCTTTTCTTTCTGTTACTTCGAAAGTAGCTGCTTCAGCTTCAGCCACTCGAATTTTCGATATTCCTTTTTATTTCTCATCAAACGAATGGCATCTTCTTCTGGAAATCCTAGCTTTTCTGAGCATGATAGTGGGGAATCTCATTGCTATTACTCAAACAAGCATGAAACGTATGCTTGCATATTCGTCCATAGGTCAAATCGGATATGTAATTATTGGAATAATTGTTGGAGACTCAAATGGTGGATATGCAAGCATGATAACTTATATGCTCTTCTATATCTCCATGAATCTAGGAACTTTTGCTTGTATTGTATTATTTGGTCTACGTACCGGAACTGATAACATTCGAGATTATGCAGGATTATACACGAAAGATCCTTTTTTGGCTCTCTCTTTAGCCCTATGTCTCTTATCCCTAGGAGGTCTTCCTCCACTAGCAGGTTTTTTCGGAAAACTCCATTTATTCTGGTGTGGATGGCAGGCAGGCCTATATTTCTTGGTTTTAATAGGACTCCTTACGAGCGTTGTTTCTATCTACTATTATCTAAAAATAATCAAGTTATTAATGACTGGACGAAACCAAGAAAGAACCTCTCACGTGCGAAATTATAGAACTTTAAGATCAAACAATTCCATCGAATTGAGTATGATTGTATGTGTGATAGCATCTACTATACCGGGAATATCAATGAACCCGATTATTGAAATTGCTCAAGATACCCTTTTTTAG

>lcl|NC_012224.1_cds_YP_002720158.1_66 [gene=rps7] [locus_tag=JacuC_p066] [db_xref=GeneID:7564782] [protein=ribosomal protein S7] [protein_id=YP_002720158.1] [location=complement(104932..105399)] [gbkey=CDS]

ATGTCACGTCGAGGTACTGCAGAAGAAAAAACTGCAAAATCCGATCCAATTTATCGTAATCGATTAGTTAACATGTTGGTTAACCGTATTCTGAAACACGGAAAAAAATCATTGGCTTATCAAATTATCTATCGAGCCATGAAAAAGATTCAACAAAAGACAGAAACAAATCCACTATCTGTTTTACGTCAAGCAATACGTGGAGTAACTCCCGATATAGCAGTAAAAGCAAGGCGTGTAGGCGGATCGACTCATCAAGTTCCCATTGAAATAGGATCCACACAAGGAAAAGCACTTGCCATTCGTTGGTTATTAGGGGCATCCCGAAAACGTCCGGGTCGAAATATGGCTTTCAAATTAAGTTCCGAATTAGTGGATGCTGCAAAAGGGAGTGGTGATGCCATACGCAAAAAGGAAGAGACTCATAGAATGGCAGAGGCAAATAGAGCTTTTGCACATTTTCGTTAA

>lcl|NC_012224.1_cds_YP_002720159.1_67 [gene=ndhF] [locus_tag=JacuC_p068] [db_xref=GeneID:7564793] [protein=NADH dehydrogenase subunit 5] [protein_id=YP_002720159.1] [location=complement(118957..121323)] [gbkey=CDS]

ATGGAACATATATATCAATATTCATGGATCATACCTTTCGTTACGTTCACCGTGCCTATATTAATAGGAGCGGGACTCCTACTTTTTCCGGCAGCAACAAAAAAACTTCGTCGTATATGGGTTTTTCCAAGCGTTTTATTGTTAAATATAGTCATGATTTTTTCAATCGATCTGTCTATTCAGCAAATAAATAGCAGTTTTTTATATCAATATATATGGTCGTGGACTATCAATAATGATTTTTCTTTAGAGTTCGGATACTTGATTGACCCACTTACTTCTATTTTGTCAGTATTAATTACTACAGTTGGAATTTTGGTTCTTTTTTATAGTGATAATTATATGTCTCATGATCAAGGCTATTTGAGATTTTTTGCTTATATGAGTTTTTTCAATACTTCAATGTTGGGATTAGTTACTAGTTCTAATTTGATACAAATTTATATTTTTTGGGAATTGGTTGGAATGTGTTCTTATCTATTAATAGGTTTTTGGTTCACACGACCTATTGCATCAAACGCTTGTCAAAAAGCGTTTGTAACTAATCGTGTAGGGGATTTTGGTTTATTATTAGGAATTTTAGGTCTTTATTGGATAACAGGCAGTTTCGAATTTCGGGATTTGTTCAAAATATTCAATAACTTGATTTCTAATAATCAGGTTAATCTTTTATTTGTTACTTTGTGTGCCTTTCTATTATTTTCCGGTGCAATTGCGAAATCGGCGCAATTTCCTCTTCATGTATGGTTACCGGATGCCATGGAAGGGCCTACTCCTATTTCGGCTCTGATACATGCTGCTACTATGGTAGCGGCGGGGATTTTTCTTATAGCTCGACTTTTTCCTCTTTTTGTAGTCACACCTTACATAATGAATCTAATCGCTTTGATAGGTATAATAACAGTATTTTTAGGAGCTACTTTAGCTCTTGCTCAAAAAGATATTAAGAGAAGTTTAGCCTATTCTACAATGTCTCAATTGGGTTATATGATGTTAGCTCTAGGTATGGGGTCTTATCGAGTCGCTTTATTTCATTTGATTACTCATGCCTATTCGAAGGCATTGTTGTTTTTAGGATCTGGATCGATTATTCATTCAATGGAAGCTATTGTTGGTTATTCTCCAGATAAGAGTCAAAATATGGTTCTGATGGGTGGTTTAACAAAACATATTCCAATTACAAAAACTGCTTTTTTATTAGGAACACTTTCCCTTTGTGGTATTCCACCTTTCGCCTGTTTTTGGTCCAAAGATGAGATTCTTAATGATAGTTGGTTGTATTCACCTATTTTTGCAATAATAGCTTGTTTCACAGCAGGATTAACTGCATTTTATATGTTTCGGGTTTATTTACTTACTTTTGAAGGACATTTAAATGTTCATTTTCAAAATTACAAAAACAGTTCATTTTATTCAATCTCTTTATGGGGTAAAGAAGAATCTAAAATGCTTAACAACAATTTTCGTTTATTAGCTTTATCAGCAATGAATAATAATGAAAGGACTTCTTTTTTTTGGAGGAACACATATCAAATTGGCGGTAATTTAAGAAATATGACATGGCCTTTTATTACTATTAAAAATTTTAATACTAAAAGGGTTTTTTCCTATCCCCATGAATCGGATAATACTATGTTACTTCCTATGCTTGTTTTGGTACTATTTACTTTATTTGTTGGAGCCATAGGAATTCCCTTCAATCAATTCAATGAAGAAGGAATGCAGTTGGATATTTTGTCAAAACTTTTAACTCCGTCTTTAAACCTTTTGTACCAAAACCAAAGTGAGTCTGTGGATTGGTATGAATTTGTAACAAATGCCATTTTTTCGGTCAGTATAGCTTTTTTCGGAATATTTATAGCGTCCTTTTTATATAAGCCCGTTTATTCATCGTTACAAAATTGGAATTTCTTTAATTTGTTCACTAAGTTGACTAAAAAGGGTCCTAATAGAATTCTTTGGGACAAAATACTAAATGTGATATATAATTGGTCCTATAATCGAGGTTACATAGATGCTTTTTATGCAATATCTTTTATTGGGGGTATAAGAAAATTGGCCGAATTAATTCATTTTTTTGATAAAAGAATAATTGATGGAATTATCAATGGAGTCGGTCTTACCAGTTTCTTTGTAGGAGAGGGTATAAAATATGTAGGAAGTGGTCGCATCTCTTCTTATCTCTTATTGTATTTATTTTGTGCATTAATCTTTTTAGTAATTTACTACTTTTTTTCAATTTGTAAAATTGAAAAAAAATTTCAATTATTTATTATATATTCTTTTATTTATTTTTTTTATTTTACTTTATTTATATTTCTTTTTTTTTTTATTTTCTATTTTTTTTTATTTTCTATTTATATATAA

>lcl|NC_012224.1_cds_YP_002720160.1_68 [gene=rpl32] [locus_tag=JacuC_p069] [db_xref=GeneID:7564794] [protein=rpl32] [protein_id=YP_002720160.1] [location=121972..122124] [gbkey=CDS]

ATGGCAGTTCCAAAAAAGCGTATTTCTATATCAAAAAAGCGTATTCGAAAAAATATTTGGAAAAGGAAGGGATATTGGGTAGTATTGAAAGCTTTTTCATTAGCGAAATCCCTTTCTACAGGTAATTCAAAAAGCTTTTTGTGCGACAAATAA

>lcl|NC_012224.1_cds_YP_002720161.1_69 [gene=ccsA] [locus_tag=JacuC_p070] [db_xref=GeneID:7564796] [protein=cytochrome c biogenesis protein] [protein_id=YP_002720161.1] [location=123330..124304] [gbkey=CDS]

ATGATATTTTCGACTTTAGAACATATATTAACTCATATATCTTTTTCAGTCGTGTCAATTGTAATTACAATTCATTTGATAACCTTATTAGTCGATGAATTCGTAGAATTATATGATTCGTCAGAAAAGGGCATGATAATTACTTTTTTCTGTATAACAGGATTATTAGTTACTCGTTGGATTTTTGGGGGACATTTACCATTAAGTGATTTATATGAATCATTAATCTTTCTTTCCTGGGTTTTTTCCATTATTCATATGGTTCCGTATTTTAAAAAACACAAAAATTTTCTAAGCGCAATAACCGCGCCAAGTACTTTTTTTACCCAAGGATTTACTACTTCGGGTCTTTTAACTGACATGCATCACTCCGAAATCTTAGTGCCCGCTCTCCAATCCCATTGGTTAATGATGCACGTAAGTATGATGATATTGGGCTATGCAGCTCTTTTGTGTGGATCATTATTATCAGTAGCATTTCTAGTAATCACATTTCGAAAAATTATAAGAATTGTTGATAAAAACAATAATTTATTAAATGATTCATTTTCTTTTAGTGAGATACAATATATGACGGAAAGAAAGAATGTTTTAAGAAATATTTCTTTTCTTTCTTCTACTAATAGGAATTATTACAGGTTTCAATTGATTCAACAATTAGATGACTGGGGTTATCGTATTATAAGTATAGGGTTTATCTTTTTAACCATAGGTATTCTTTCGGGAGCAGTCTGGGCTAATGAAGCGTGGGGATCATATTGGAGTTGGGACCCAAAGGAAACTTGGGCATTTATTACGTGGACCATATTTGCGATTTATTTTCATACTCGAACAAATAAAAATTTTGAGGATTTAAATTCGGCAATTGTCGCTTTTATCGGTTTTCTTCTAATTTGGATATGTTATTTTGGAGTTAATTTATTAGGAATAGGTTTGCATAGTTATGGTTCATTTACATTAACAATTAACATCTAA

>lcl|NC_012224.1_cds_YP_002720162.1_70 [gene=ndhD] [locus_tag=JacuC_p071] [db_xref=GeneID:7564797] [protein=NADH dehydrogenase subunit 4] [exception=RNA editing] [protein_id=YP_002720162.1] [location=complement(124604..126106)] [gbkey=CDS]

ACGAATTCTTTTCCTTGGTTAACAATACTTGTAGTTTTACCGATATCCGCGGGTTCCTTAATTTTCCTTTTCCCTCATAGAGGAAATAAGGTAATTAGGTGGTATACTTTATGTATATGTGTTTTAGAGCTTCTTTTAATGACTTATGTGTTCTCTTATTATTTCCAATTGGACGATCCATTAATTCAATTAACAGAAGATTATAAATGGATCAAGTTTTTTGATTTTTACTGGAGATTGGGAATAGATGGATTTTCTTTAGGACCTATTTTATTGACAGGATTTATCACTACTTTAGCTACTTTAGCGGCTCGGCCAATTAATCGGGATTCCCGATTATTCCATTTTCTGATGTTAGCAATGTATAGTGGTCAAATAGGATTATTTTCTTCTCAAGATCTTTTACTTTTTTTTATCATGTGGGAGTTAGAATTAATTCCCATTTATCTACTTCTATCAATGTGGGGGGGAAAGAAACGTCTGTATTCAGCTACAAAGTTTATTTTGTATACTGCAGGGGGTTCTGTTTTTTTATTAATGGGAGCTTTGGGTATCGCTTTATATGGTTCCAATGAACCAAGATTCCATTTTGAAATATCAGCCAATCAATCATATCCTGCGGCGCTAGAAATATTTTTCTATATTGGATTTCTTATTGCTTTTGCTGTCAAATCACCGATTATACCCTTACATACATGGTTACCAGACACGCATGGGGAAGCACATTACAGTACTTGTATGCTTCTAGCCGGAATCTTATTAAAAATGGGGGCATATGGATTGATTCGAATCAATATGGGATTCTTACCTCATGCTCATTCTATCTTTTCTCCCTGGTTGATAATAGTAGGCGTAATGCAAATAATCTATGCAGCTTCAACATCTCCTGGTCAACGAAATTTAAAAAAAAGAATAGCATATTCTTCTGTATCTCATATGGGTTTCATAATTATAGGAATTTGCTCTATAAGTGATATGGGGCTCAACGGAGCCATTTTACAAATAATATCACATGGATTTATTGGTGCTGCACTTTTTTTCTTGGCAGGAACGGGTTATGATAGAATACGTCGTGTTTATCTTGACGAAATGGGTGGAATGGCTCCCCCAATGCCAAAAATATTTACGACATTCAGTATCTTATCACTAGCTTCTCTTGCATTACCGGGCATGAGTGGTTTTTTTTCGGAATTGATAGTCTTTTTTGGAATAATTACCGGTCAAAAATATCTTTTAATGTCAAAAATATTAATTACTTTTGTAATGGCAGTTGGAATGATATTAACTCCTATTTATTTATTATCTATGTTACGCCAGATATTCTATGGGTACAGGCTATTTAATGTCCAAAACTCTTATTTTTTTGATTCTGGACCGCGAGAGTTATTTGTTTCGATCTCTATCCTTCTGCCTGTAATAGGCATTGGTATTTATCCGGATTTCGTTTTCTCATTATCAATTGACAAGGTCGAAGCTATTCTATCTAATTATTTTTATAGATAG

>lcl|NC_012224.1_cds_YP_002720163.1_71 [gene=psaC] [locus_tag=JacuC_p072] [db_xref=GeneID:7564798] [protein=photosystem I subunit VII] [protein_id=YP_002720163.1] [location=complement(126256..126501)] [gbkey=CDS]

ATGTCACATTCAGTAAAGATTTATGATACATGTATAGGGTGTACTCAATGTGTCCGAGCCTGTCCCACCGATGTATTAGAAATGATACCTTGGGATGGATGTAAATCTAAGCAAATTGCTTCTGCTCCAAGAACAGAGGACTGCGTTGGTTGTAAGAGATGCGAATCCGCCTGTCCAACAGATTTCTTGAGTGTTCGAGTTTATTTATGGCATGAAACAACTCGAAGCATGGGTCTAGCTTATTGA

>lcl|NC_012224.1_cds_YP_002720164.1_72 [gene=ndhE] [locus_tag=JacuC_p073] [db_xref=GeneID:7564799] [protein=NADH dehydrogenase subunit 4L] [protein_id=YP_002720164.1] [location=complement(126758..127063)] [gbkey=CDS]

ATGATGCTCGAACATGTACTTGTTTTGAGTGCCTATTTATTTTCTATCGGTATCTATGGATTGATCACGAGTCGAAATATGGTTAGAGCCCTTATGTGCCTTGAACTTATACTGAATGCAGTTAATATAAATTTCGTAACATTTTCTGATTTTTTTGATAGTCGCCAACTAAAGGGAAATATTTTTTCAATTTTTGTTATAGCTATCGCAGCCGCTGAAGCAGCTATTGGACCAGCTATTGTTTCATCAATTTATCGTAACAGAAAATCAACCCGTATCAATCAATCGAATTTGTTGAATAAGTAG

>lcl|NC_012224.1_cds_YP_002720165.1_73 [gene=ndhG] [locus_tag=JacuC_p074] [db_xref=GeneID:7564800] [protein=NADH dehydrogenase subunit 6] [protein_id=YP_002720165.1] [location=complement(127302..127832)] [gbkey=CDS]

ATGGATTTGCCTGGACTAATACATGATTTTCTTTTAGTCTTTCTGGGATTAGGTCTTATATTAGGAGGTCTAGGAGTGGTATTATTTACCAATCCAATTTATTCTGCTTTTTCGTTGGGATTTGTTCTTGTTTGTATATCTTTATTCTATATTTTATCAAACTCTCATTTTGTAGCTGCTGCACAGCTCCTTATTTATGTGGGAGCTATAAATGTTTTAATTCTATTTGCCGTGATGTTCATGAATGGTTCAGAATATTACAAAGATTTTAATCTTTGGACTGTTGGAAACGGGGTTACTTCCTTAGTTTGTACAAGCATTTTTATTTCACTAATTACTATTATTTCAGATACATCATGGTACGGGATTATTTGGACTACAAGAACAAATCAGATTATAGAACAAGATTTAATAAGTAATGGGCAACAAATTGGAATTCATTTATCAACAGATTTTTTTCTTCCATTTGACTTTATTTCAGTAATTCTTTTAGTTGCTTTGATAGGTGCGATTGCTGTGGCTCGTCAGTAA

>lcl|NC_012224.1_cds_YP_002720166.1_74 [gene=ndhI] [locus_tag=JacuC_p075] [db_xref=GeneID:7564801] [protein=NADH dehydrogenase subunit I] [protein_id=YP_002720166.1] [location=complement(128386..128898)] [gbkey=CDS]

ATGTTTCCTATGGTAACTGGGTTCATGAATTATGGGCAACAAACCATGCAAGCTGCAAGGTACATTGGTCAAGGTTTCATGATTACCTTATCCCATGCAAACCGTTTACCTGTAACGATTCAATATCCTTATGAAAAATTAATCACATCGGAGCGTTTCCGCGGTCGAATCCATTTTGAATTTGATAAATGCATTGCTTGTGAAGTATGTGTTCGGGTATGCCCTATAGATCTACCTGTTGTTGATTGGCAATTGGAAACTGACATTCGAAAGAAACGGTTGCTTAATTACAGTATCGATTTTGGAATCTGTATATTTTGTGGCAACTGTGTTGAGTATTGTCCAACAAATTGTTTATCAATGACTGAAGAATATGAACTTTCTACTTATGATCGTCACGAATTGAATTATAATCAAATTGCTTTAGGTCGTTTACCAATGTCAGTAGTTGACGATTATACAATTCGAACAATTTTGAATTCAACTCAACAAAAAATCAACAAAAAAAAATAG

>lcl|NC_012224.1_cds_YP_002720167.1_75 [gene=ndhA] [locus_tag=JacuC_p076] [db_xref=GeneID:7564802] [protein=NADH dehydrogenase subunit 1] [protein_id=YP_002720167.1] [location=complement(join(128980..129519,130655..131206))] [gbkey=CDS]

ATGATAATTGATACAACAGAAGTACAAGCTATCAATTCTTTTTCTAGATTAGAATCCTTAAACGAGGTCTATGGACTTCTATGGGCCTTTGTCCCTATTTTGATTCTTGTATTTGGAATCACGATAGGCATACTAGTAATTGTATGGTTAGAAAGAGAAATATCTGCAGGGATACAACAACGTATTGGACCTGAATATGCCGGTCCTTTAGGGGTTCTTCAAGCTCTAGCGGATGGGACAAAACTTCTTTTCAAAGAGAATCTTTTTCCATCTAGGGGAGATACTCGTTTATTCAGTATCGGACCATCCATAGCAGTCATATCAACTTTATTAAGCTATTCAGTAATTCCTTTTGGCTATCACTTTGTTTTAACTGATCTAAATATTGGTGTTTTTTTATGGATTGCCATTTCAAGTATTGCTCCCATTGGACTTCTTATGTCAGGATATGGATCCAATAATAAATATTCTTTTTTAGGTGGTCTACGAGCTGCTGCTCAATCGATTAGTTATGAAATACCATTAAGTCTTTGTGTGTTATCCATATCTCTATTATCTAACAGTTCAAGTACCGTTGATATAGTTGAGGCACAATCAAAATACGGTTTTTGGGGGTGGAATTTGTGGCGTCAACCTATAGGATTTATCATTTTTTTTATTTCTTCTCTAGCAGAGTGTGAAAGATTGCCTTTTGATTTACCAGAAGCAGAAGAAGAATTAGTAGCAGGCTATCAAACCGAATATTCTGGTATAAAATTTGGTTTATTTTATATTGCTTCCTATCTAAACTTATTAGTTTCTTCATTATTTGTAACAGTTCTTTACTTGGGTGGTTGGAATATCCCTATTTCGTATCTATTCGTTCCTGAGCTTTTTCAAATAAATAAAATAGGTGGAGTCTTTGAAACAACAATAGGTATCTTTATTACATTGGTTAAAACTTATTTGTTCTTGTTCATTCCTATTACAACAAGATGGACTTTACCTAGACTAAGAATGGACCAACTTTTAAATCTTGGATGGAAATTTCTTTTACCTATTTCTCTCGGTAATTTATTATTAACAACCTCTTTCCAACTCCTTTCACTATAA

>lcl|NC_012224.1_cds_YP_002720168.1_76 [gene=ndhH] [locus_tag=JacuC_p077] [db_xref=GeneID:7564803] [protein=NADH dehydrogenase subunit 7] [protein_id=YP_002720168.1] [location=complement(131208..132389)] [gbkey=CDS]

ATGAATGTACCAGCTATGCGAAAAGACCTTATGATAGTCAATATGGGTCCCCACCACCCATCAATGCATGGTGTTCTTCGACTCATCGTTACTCTAGATGGCGAAGATGTTATTGACTGTGAACCAATATTAGGTTATTTACACAGAGGCATGGAAAAAATTGCGGAAAATCGAACAATTATACAATATTTGCCCTATGTAACACGTTGGGATTATTTGGCTACTATGTTCACAGAAGCAATAACAGTAAATGGCCCAGAACTGTTAGGAAATATTCAAGTGCCTAAAAGAGCTAGCTATATCAGGGTAATTATGTTGGAATTGAGTCGTATAGCTTCTCATTTATTATGGCTTGGCCCTTTTATGGCGGATATTGGGGCACAGACTCCTTTCTTCTATATTTTTAGAGAGAGAGAGTTAGTATATGATTTATTCGAAGCTGCCACTGGTATGAGAATGATGCATAATTATTTTCGTATTGGGGGAGTAGCGGCTGATCTACCTCATGGCTGGATAGATAAATGTTTGGATTTTTGTGATTATTTTTTAACAAGAGTTGCTGAATATCAAAAACTTATTACGCGAAATCCTATTTTTTTAGAACGAGTTGAAGGAGTTGGTATTGTTGGTACAGAGGAAGCAATAAATTGGGGGTTATCAGGACCAATGCTACGAGCTTCCGGAGTACAATGGGATCTTCGTAAAATGGATCGTTATGAGTGTTACGACGAATTTGATTGGGAAGTCCAGTGGCAAAAAGAAGGGGATTCATTAGCTCGTTATTTAGTCCGAATTGGTGAAACGATGGAATCTATAAAAATTATTCAACAGGCTCTGGAAGGAATTCCGGGGGGGCCCTATGAAAATTTAGAAACCCGACGTTTTGATAAAGAAGGGGATCCAGAATGGAACGATTTCGAATATCGATTCATTAGTAAAAGAACTTCTCCTACTTTTGAATTACCGAAACAAGAACTTTATGTGAGAGTCGAAGCTCCAAAAGGAGAATTGGGAATTTTTCTGATAGGGGATCAGAGCGGTTTTCCTTGGAGATGGAAAATTCGCCCCCCGGGTTTTATCAATTTGCAAATTCTTCCTCAATTAGTTAAAAGAATGAAATTGGCTGATATTATGACAATACTAGGTAGTATAGATATCATTATGGGAGAAGTTGATCGTTGA

>lcl|NC_012224.1_cds_YP_002720169.1_77 [gene=rps15] [locus_tag=JacuC_p078] [db_xref=GeneID:7564804] [protein=rps15] [protein_id=YP_002720169.1] [location=complement(132496..132768)] [gbkey=CDS]

ATGGTAAAAAATTCATTCATTTCGGTTATTTCACAAGAAGAAAAAAACGAAAACAAAGAATCCGTTGAATTTCAAATAGTAAGTTTCACTAATAAGATACGAAGACTTACTTCACATTTGGAATTGCATAGAAAAGACTATTCATCTCAGAGGGGTTTGCGGAAAATTCTAGGAAAACGTCAACGATCGCTGTCTTATTTAGCAAAGAAAAAGAGAGTACGTTATAAAGAATTAATTAGCCGGTTGGATATTCGGGAGTCAAAAATTCGTTAA

>lcl|NC_012224.1_cds_YP_002720170.1_78 [gene=ycf1] [locus_tag=JacuC_p079] [db_xref=GeneID:7564805] [protein=Ycf1] [protein_id=YP_002720170.1] [location=complement(133196..138919)] [gbkey=CDS]

ATGATTTTGAAATCTTTTATACTAGGTAATCTAGTATCCTTATGCATGAAGATAATCAATTCGGTCGTTGTGGTCGGACTCTATTATGGATTTCTGACCACATTCTCCACGGGGCCCTCTTATCTCTTCCTTCTTCGAGCTCGGGTTATAGAAGAAGGAGAAGAAGGAACTGAGAAGAAGGTATCAGCAACAACAGGTTTTATTACGGGACAGCTCATGATGTTCATATCGATCTATTATGCGCCTCTGCATCTAGCATTGGGTAGACCTCATACAATAACTGTCCTAGCTCTACCCTATCTTTTGTTTCATTTCTTCTGGAACAATCACAAACACTTTTTTGATTATGGATCTACTACCAGAAATTCAATGCGTAATCTTAGCATTCAATTTGTATTCCTGAATAATCTCATTTTTCAATTATTCAACCATTTCATTTTACCAAGTTCAATGTTAGTCAGATTAGTCAACATTTATATGTTTCGATGCAACAACAAGATGTTATTTGTAACAAGTAGTTTTGTTGGTTGGTTAATTGGTCACATTTTATTCATGAAATGGGTTGGATTGATATTAGTCTGGATACAGCAAAATAATTCTATTAGATCGAATGTACTTTTTCGATCTAATAAGTACCTTGTGTCAGAATTGAGAAATTCTATGGCTCGAATCTTTAGTATTCTCTTATTTATTACCTGTGTCTACTCTTTAGGCAGAATACCGTCACCCATTTTTACTAAGAAACTGAAAGAAACCTCAGAAGCGGAAGAAAGGGAGGAAGAAACAGATGTAGAAATAGAAACAACTTCCGAAACGAAGGGGACTAAACAGGAACAAGAGGGATCCACCGAAGAAGATCCTTCTTCTTCCCTTTTTTCGGAAGAAAAGGAGGATCCGGACAAAATCGACGAAACGGAAGAGATCCAAGTGAATGGAAAGGAAAAAACAAAGGATGAATTCCATTTTCACTTTAAAGAGACATGCTATAAAAATAGACCACTTTATGAAACTTTTTATCTGGATGGGAATCAAGAAAATTCGAAGTTAGAAATATTGATAGATAAAAAAAATAAAGATCTTTTCTGGTTTGAAAAACCTCTTGTAACTATTCTTTTTGACTCTAAACGTTGGAATCGTCCATTTCGATATATAAAAAATGATCAGTTTGAGAATGCTGTAAGAAAAGAAATGTCACAATATTTTTTTTATACATGTCGAAGTGATGGAAAAGAAAGAATATCTTTTACGTATCCACCCAGTTTGTCAACTTTTTTGGAAATGATACAAAGAAGGATGTCCCTGTTTACAACAGAAAAACTCTCCTCCGATGAATTGTATAATCGTTGGAATTATAAGAATGAACAAAAAAAGAAAAATCTAAGTAATGAATTTATAAAAAGAGTCCAGGCTTTAGATAAGGGATATCTTGCTCTGAATACACTCCAAAAAAGGACTAGATTGTGTAATGATAAAACTAAAAAAGAGTACTTACCTAAAATATATGATCCCTTATTGAGTGGGTCATACCGCGGAAAAATCCAATTTTTTTTTTCACCCTCAATTTTAAATAAAACTTCCATAAAAAATTCTATAGAGATGGTTTGGATAAATAAAATTCATCTTTTTCTTCTTATTACTAATTATCAAAAATTCGAACCAAAAACAGATGTAAAATCATTTTCAACAGAAATTGCTTATTTCTTAAACTTAATTAATGAATTTGCCGGAAAATCAAGATCGAGTTTAAATTTTAAGGAACTCTCTTTATTTCCAGATCACAAAGAAGAAAAAATGGATTTAGAAAATCGAATAAAAATTTTCAAATTTTTATTTGATACAGTTATCGCGAATTCAAAAAATAAAACAATTAGAAATAATTCTATTGGAATAAAAGAAATAAGTAAACAAGTTCCTCGATGGTCATATAAATTAATTGACGATTTGGAACAACAAGAGGGAGAAAATGAAGAAAATGAAGAAAACATGGCGGAAGATCATGAAATTCGTTCACGAAAAGCCAAACGTGTAGTGATTTTTACTGATAATCAACAAAATACTGAGACTTATAATAATACCAAAGATACAACGAATTCTGATCAAATAGACAAAGTGACTTTGATACGTTATTCACAACAATCGGACTTTCGTCGAGACATAATAAAAGGATCTATGCGAGCACAAAGACGCAAAATACCTATATTGGAACTGTTTCAAGCAAATGTGCATTCCCCCCTTTTTTTGGACAGAATAGACAAATCCCTTGTTTTTTCTTTTGATATTTCCGAACTAATGAAAACAATGTTTATAAATTGGACGTGTAAAAACGCAGAATTCACAATTTCGGATTCTACTTATACAGAGAAAAAACAAAAAGAAAGTAAAAAAAAAGAAGACGACAAAAGAGAGGAAAAAGCTCGGATAGAAATAGCCGAAGCCTGGGATAGCATTCTTTTTGCTCAAGCAATAAGAGGGTGTGTTTTAGTAACCCAGTCGATTCTTAGAAAATATATTATATTAACTTTTTTAATAATAACTAAAAATATCATTCGTATACTACTTTTTCAAATTCCCGAATGGTCTGAGGATTTTAAAGATTGGAGTAGAGAAATGCATGTTAAATGCACTTATAATGGAGTTCAATTATCCGAAAAAGAATTTCCAAAAAACTGGTTAACAGACGGGATTCAAATAAAGATCCTATTTCCTTTTCGTCTAAAACCTTGGCACAGATCTAAGTTAAAACTCCCTCCCAAAGATCCAATGAAAAAGAAAACACAAAAACGGATTTTTGTTTTTTTAACAGTTTGGGGAATGGAAGTTGAATTGCCTTTTGGTTCTCCCCGAAAACGGCTTTCCCTCTTTGAACCCATCTTTAAAAAACTCGAAAAAAAAATTCGAAAAATAAAAAAAAAAGGTTTTCGAGTTCTAACAATTTTAGAAGAAAGAAGAAAATTTTTTCTAAAATTATCAAAAGAAAAAAAAATTGGGTTATCAAAAACATTTTTTTTCGAAAAGAAAAAAGAAATAATAAACAAATTTTCAAAATCAAAAAGAAATGCAAATTTATTATCTGGATTTATAAAAGTATATGAATTGAATGAAACTAAAAAAGAAAAAAATTCAATAATCGATAACAATAATGGAACGATTCAAAAATTGTCTACTCCAACTCGATCTATGGCTTGGACAAATTATTCACTAACAGAAAAAAAAATGAAAGATCTTTCTTCTAGAAGAAAGATAATCATAAATCAAATAGAAAAAATGAAAAAAGAAAAGGAAACAAAAATTATAACCTCAAAAATACATATTAATCCTAACAAAATAAGTTATAATGTTAAAAAATTAAAATCATCAAAAAATATTTCACAGATACAGATATTAAAAAGGGGAAATGCTCGATTAGTGCGTAAATTCCATTTTTTTATAAAAATTTTGATTCAAAGGATATACATAGATATCTTTTTAAGTATCATTAATATTCCGAGGATCAATGCACAGCTTTTTCTTGAATCAACAAAAAAATTATTACTAAATATGTTTGCAATAATGAAAAAAAAATCACAAAAAATTGATAAAACAAAGCAAAATACAATTCACTTTCTTTCGATTATGATTATAAAAAAGTTACTTAATAATAGTAATATTGCTGTTATTAATAACAATTCACAGATTTTTTGTGATATATCCTCCTTGTCACAAACATATGTATTTTACAAATTATCACAAATCCAAATTATTAATTTATATAAGTTACGATCTATCCTTCAATATCATGGCCTTTTTCTTAAGAATGAGATAAAGGATTATTTTAGAGCCCAAGGGATATTTAATTCCAAATTAAAAGATAAAAATTTTCAAAATTTCGTAATAAATCAATGGAAAAACTGGTTAAGGGGTCATTATCAATATAAATATGATTTAACTCAGATTAGATGGTCTAGATTAATATCACAAAAATGGCGAAATAGAATCAAGCAACACCATATGGTTCAAAATAAAAAATTTAATAAATTCAATTTATATGAAAAAGACCAATTAAATGAAAAAAGACCAATTAATTCATTACGAAAAAAAAATGATAATGATTTTGCGGCAGATTCATTACCGAATCAAAAAAAGAATTTTCAAAAACACTGTAGATATAATCTTTTATCATATAAATCCATTAATTATGAAAATAAGAAGAACTCATATATTTACGGATTCCCATTACAAGTAAATAAACAAGAGATTTCTTATAATTACAACACAAATAAAAGCAAATTATTTGACATGTTGGAAAGTATTCCTATCAATAATTATCTAGGGGAAGATGATATTAGCGATATGGAGAAAAGCCCGACTAGAAAATATTTGGATTGGAGAATTCTCGATTTTTGTCTTAGAAAAAAGGTCGATATTGAGTCCTGGATTGATATCGGAAGCAAAGAAAAAAAAAATACTAAGACTAGGACTAATAAGTATCAAATAATTGATAAAATCGATAAGAAAAATCTTCTTTTTCTTACAATTCACCAAGATCAAGAAGTCAATTCATCCAATCAAAAAAAAAACCTTTTTGATTGGCTGGGAATGAATGAAGAAATACAAAATCGTCTCATATCCAATTTTGAACTTTGGTTCTTTCGAAAATTTGTGATACTTTATAACACATATAAGATAAAACCATGGGCAATACCCATCCAATTTCTTCTTTTCAATTTTCATAGAAATGAAAATGTTAGTAAAAATACTAAAATCAACGAGAAGAAAAACGGCGATCTTTTTATATCATCGAATGAAAAAAAATTCATTGAATTAGAGAATCGAAATCACGAAGAAAAAGAATCCAAAGACAAAATGGACTTTGGATCAGTTTTCGCAAATCAAGAAAAAAATATTGAAGAAGATTATATGGGATTAGATATGAAAAAACATAGAAATAAAAAACAAAACAAAAGTCATACGGAAGTAGAGCTTGATTTCTTCCTAAAACGGTATTTATGTTTTCAATTAAGATGGAATGATTCTTTAAATGAAAAAATAATCAATAATATCAAAGTATATTGTCTCCTGCTTAGACTGACAAATCCACGAGAAATTCTTATATCTTCTATTCAAAGACAAGAAATAAGTCTGAATATTCTGATGGTTCAGAAGGATTTAACTCTTACTGAATTGATGAAAAAGGGAATATTTATTATCGAACCTGTTCGTTTGTCAGTAAAAAATAATGGACAATTTCTTTTGTATCAAACGGTAGGTATCTTATTAGTTCATAAGAACAAACAACAAATTAATCAAAAATACAGAGAAAAAATCTATATTGATAAAAAGAATTTTACCGAACCTATTGAAAGACATCAAAGTCTAATTGGCAATAGAGACAAAAATGATTATGATTTACTTGTTCCTGAAAATATTTTATTCCCTAAACGTCGTAGAGAATTAAGAATTCTAACTTCTTTCAATTTTAAAAACCAAAATGATATTCATAAAAATGATATTCATATAAATACAGAAATTTTCAACGTTAATAAAATAAAAAACTGTGGTCCTATTTTGGATAAAAGTAAACATTTTGATAGAGATAAAAATAAACTAATTAAATTCAAATTTTTTCTTTGGCCCCATTTTCGATTAGAAGATTTAGCTTGTATGAATCGCTATTGGTTTGATACTAATAATGCCAGTTGCTTCAGTATGGTAAGAATATATATATATCCACGGGCGCAATTTTAG

>lcl|NC_012224.1_cds_YP_002720171.1_79 [gene=rps7] [locus_tag=JacuC_p080] [db_xref=GeneID:7564815] [protein=ribosomal protein S7] [protein_id=YP_002720171.1] [location=150189..150656] [gbkey=CDS]

ATGTCACGTCGAGGTACTGCAGAAGAAAAAACTGCAAAATCCGATCCAATTTATCGTAATCGATTAGTTAACATGTTGGTTAACCGTATTCTGAAACACGGAAAAAAATCATTGGCTTATCAAATTATCTATCGAGCCATGAAAAAGATTCAACAAAAGACAGAAACAAATCCACTATCTGTTTTACGTCAAGCAATACGTGGAGTAACTCCCGATATAGCAGTAAAAGCAAGGCGTGTAGGCGGATCGACTCATCAAGTTCCCATTGAAATAGGATCCACACAAGGAAAAGCACTTGCCATTCGTTGGTTATTAGGGGCATCCCGAAAACGTCCGGGTCGAAATATGGCTTTCAAATTAAGTTCCGAATTAGTGGATGCTGCAAAAGGGAGTGGTGATGCCATACGCAAAAAGGAAGAGACTCATAGAATGGCAGAGGCAAATAGAGCTTTTGCACATTTTCGTTAA

>lcl|NC_012224.1_cds_YP_002720172.1_80 [gene=ndhB] [locus_tag=JacuC_p081] [db_xref=GeneID:7564816] [protein=NADH dehydrogenase subunit 2] [protein_id=YP_002720172.1] [location=join(150961..151737,152420..153169)] [gbkey=CDS]

ATGATCTGGCATGTACAGAATGAAAACTTCATTCTCGATTCTACGAGAATTTTTATGAAAGCCTTTCATTTGCTTCTCTTCGATGGAAGTTTTATTTTCCCAGAATGTATCCTAATTTTTGGCCTAATTCTTCTTCTGATGATCGATTCAACCTCTGATCAAAAAGATATACCTTGGTTATATTTCATCTCTTCAACAAGTTTAGTAATGAGTATAACGGCCCTATTGTTCCGATGGAGAGAAGAACCTATGATTAGCTTTTCGGGAAATTTCCAAACGAACAATTTCAACGAAATCTTTCAATTTCTTATTTTACTATGTTCCACTCTATGTATTCCTCTATCCGTAGAGTACATTGAATGTACAGAAATGGCTATAACAGAGTTTCTCTTATTCCTATTAACAGCTACTCTAGGAGGAATGTTTTTATGCGGTGCTAACGATTTAATAACTATCTTTGTAGCTCCAGAATGTTTCAGTTTATGCTCCTACCTATTATCTGGATATACCAAGAAAGATGTACGGTCTAATGAGGCTACTACGAAATATTTACTCATGGGTGGGGCAAGCTCTTCTATTCTGGTTCATGCTTTCTCTTGGCTATATGGTTCGTCCGGGGGAGAGATCGAGCTTCAAGAAATAGTGAATGGCCTTATCAATACACAAATGTATAACTCCCCAGGAATTTCAATTGCGCTTATATTCATCACTGTAGGAATCGGGTTCAAGCTTTCCCCAGCCCCTTCTCATCAATGGACTCCTGACGTATACGAAGGATCTCCCACTCCAGTCGTTGCTTTTCTTTCTGTTACTTCGAAAGTAGCTGCTTCAGCTTCAGCCACTCGAATTTTCGATATTCCTTTTTATTTCTCATCAAACGAATGGCATCTTCTTCTGGAAATCCTAGCTTTTCTGAGCATGATAGTGGGGAATCTCATTGCTATTACTCAAACAAGCATGAAACGTATGCTTGCATATTCGTCCATAGGTCAAATCGGATATGTAATTATTGGAATAATTGTTGGAGACTCAAATGGTGGATATGCAAGCATGATAACTTATATGCTCTTCTATATCTCCATGAATCTAGGAACTTTTGCTTGTATTGTATTATTTGGTCTACGTACCGGAACTGATAACATTCGAGATTATGCAGGATTATACACGAAAGATCCTTTTTTGGCTCTCTCTTTAGCCCTATGTCTCTTATCCCTAGGAGGTCTTCCTCCACTAGCAGGTTTTTTCGGAAAACTCCATTTATTCTGGTGTGGATGGCAGGCAGGCCTATATTTCTTGGTTTTAATAGGACTCCTTACGAGCGTTGTTTCTATCTACTATTATCTAAAAATAATCAAGTTATTAATGACTGGACGAAACCAAGAAAGAACCTCTCACGTGCGAAATTATAGAACTTTAAGATCAAACAATTCCATCGAATTGAGTATGATTGTATGTGTGATAGCATCTACTATACCGGGAATATCAATGAACCCGATTATTGAAATTGCTCAAGATACCCTTTTTTAG

>lcl|NC_012224.1_cds_YP_002720173.1_81 [gene=ORF126] [locus_tag=JacuC_p082] [db_xref=GeneID:7564818] [protein=ORF126] [protein_id=YP_002720173.1] [location=153989..154369] [gbkey=CDS]

ATGAATGGGGAGTCCGCTTTGAAAGCGTCCGCCCTGCAACCACCCCCGAGTATATGCTTCAACAGGAATTACACAAGGGTAGTTGATACAATAGAAACCTCTGGTAAAATGCCCGCCCGTAACCCAACAGATAAAGTACATTACATAGTCCGTTTTAGGGATTGGCGACTTACCCATTCAGTGACTTTGGCACTGGATGTTCCAAAAAGAAAATGGGTACTCTCGGGTCGGGTGAATTCAATAATAGACGTCTGTTGGCATTCCAGCCTTCCTTCTCCTTTCAGGGCCTATCCGAAAGAGAATCCAGTACTTCTTGGTCGTGAATATCTGAATAGGACAAACCGCCCCGTGGATATCTTTGCTTCGGAACAAAACAATTAG

>lcl|NC_012224.1_cds_YP_002720174.1_82 [gene=ycf2] [locus_tag=JacuC_p083] [db_xref=GeneID:7564819] [protein=Ycf2] [protein_id=YP_002720174.1] [location=complement(154840..161736)] [gbkey=CDS]

ATGAAAGGACATCAATTCAAATCCTGGATTTTCGAATTGAGAGAGATATTGAGAGAGATCAAGAATTCTCACTATTTCTTAGATTCATGGACCCAATTCAATTCAGTGGGATCTTTCATTCACATTTTTTTCCATCAAGAACGTTTTATAAAACTCTTGGACTCCCGAATTTGGAGTATCTTACTTTCACGCAATTCACAGGGTTCAACAAGCAATCGATATTTCACGATCAAGGGTGTAGTACTATTTGTAGTAGTGGTTCTTATATATCGTATTAACAATCGAAAGATGGTCGAAAGAAAAAATCTCTATTTGACAGGGCTTCTTCCTATACCTATGAATTCCATTGGACCCAGAAATGATACATTGGAAGAATCCTTTGGGTCTTCCAATATCAATAGGTTGATTGTTTCGCTCCTGTATCTTCCAAAAGGAAAAAAGATCTCTGAGAGCTCTTTCCTGGATCCGAAAGAGAGTACTTGGGTTCTCCCAATAACTAAAAAGTCTGAATCTAACTGGGGTTCGCGGTGGTGGAGGAACTGGATCGGAAAAAAGAGGGATTCTAGTTGTAAGATATCTAATGAAACCGTCGCTGGAATTGAGATCTCATTCAAAGAAAAAGATATCAAATATCTGGAGTTTCTTTTTGTATATTATATGGATGATCCGATCCGCAAGGACCATGATTGGGAATTGTTTGATCGTCTTTCTCCGAGGAAGGGGCGAAACATAATCAACTTGAATTCGGGACAGCTATTCGAAATCTTAGTGAAAGACTGGATTTGTTATCTCATGTTTGCTTTTCGCGAAAAAATACCAATTGAAGTGGAGGGTTTCTTCAAACAACAAGGAGCTGGGTCAACTATTCAATCAAATGATATTGAGCATGTTTCCCATCTCTTCTCGAGAAAGAAGTGGGCTATTTCTTTGCAAAATTGTGCTCAATTTCATATGTGGCAATTCCGCCAAGATCTCTTCGTTAGTTGGGGGAATAATCCGCACGAATCGGATTTTTTGAGGAACATATCGAGAGAGGATTGGATTTGGTTAGACAATGTGTGGTTGGTAAACAAGGATCGGTTTTTTAGCAAGGCACGGAATATATCGTCAAATATTCAATATGATTCCACAAGATCTAGTTTCGTTCAAGGAAGGAATTCTAGCCAATTGAAGGGATCTTCTGATCAATCCAGAGATCATTTCGATTCCATTAGTAATGAGGATTCGGAATATCACACATTGATCAATCAAAGAAAGATTCAACAACTAAAAGAAAGATCGATTCTTTGGGATCCTTCCTTTCTTCAAACGGAACGAACAGAGATAGAATCAGACCGATTCCCTAAATGCCTTTCTGGATATTCCTCAATGTCCCGACTATTCACGGAAGGTGAGAAGGAGATGAATAATCATCTGCTTCCGGAAGAAATCGAAGAATTTCTTGGGAATCCTACAAGATCCATTCGTTCTTTTTTCTCTGACAGATCGTCAGAACTTCATCTGGGTTCGAATCCTACTGAGAGGTCCACTAAATTGTTGAAGAAAGAACAAGATGTTTCTTTTGTCCCTTCCAGGCGATCGGAAAATAAAGAAATAGTTAATATATTCAAGATAATCACGTATTTACAAAATACCGTCTCAATTCATCCTATTTCATCAGATCCGGGATGTGATATGGTTCTGAAGGATGAACTGGATATGGACAGTTCCAATAAGATTTCTTTCTTGAACAAAAATCCATTTTTTGATTTATTTCATCTATTCCATGATCGGAACGGGGGGGGATACACGTTACACCACGATTTTGAATCAGAAGAGAGATTTCAAGAAATGGCAGATCTATTCACTCTATCAATAACCGAGCCGGATCTGGTGTATCATAAGGGATTTACCTTTTTTATTGATTCTTACGGATTGGATCAAAAACAATTCTTGAATGAGGTATTCAACTCCAGGGATGAATCGAAAAAGAAATCTTTATTGGTTCTACCTCCTATTTTTTATGAAGAGAATGAATCTTTTTATCGAAGGATCAGAAAAAAATGGGTCCGGATCTCCTGCGGGAATGATTTGGAAGATCCAAAACAAAAAATAGTGGTATTTGCTAGCAACAACATAATGGAGGCAGTCAATCAATATGGATTGATCCTAAATCTGATTCAAATCCAATATAGTACCTATGGGTACATAAGAAATGTATTGACTCAATTCTTTTTAATGAATAGATCCGATCGCAACTTCGAATATGGAATTCAAAGGGATCAAATAGGAAATGATACTCTGAATCATAGAACTATAATGAAATATACGATCAACCAACATTTATCGAATTTGAAACAGAGTCAGAAGAACTGGTTCGATCCTCTTATTTTTCTTTCTCGAACCGAGAGATCCATGAATTGGGATCCTAATGCATATAGATACAAATGGTCTAATGGGAGCAAGAATTTCCAGGAATATTTGGAACATTTCATTTCTGAGCAGAAGAGCCGTTTTCTTTTTCAAGTAGTGTTCGATCGATTACGTATTAATCAATATTCGATTGATTGGTCTGAGGTTATCGACAAAAAAGATTTGTCTAAGTCACTTCGTTTCTTTTTGTCCAAGTTACTTGTTTTTTTGTCCAAGTTTCTTCTCTTTTTGTCTAACTCACTTCCTTTTTTCTTTGTGAGTTTCGGGAATATCCCCATTCATAGGTCCGAAATCCATATCTATGAATTGAAAGGTCCGAATGATCCACTCTGCAATCAGCTGTTAGAACCAATAGGTCTTCAAATCGTTCATTTGAAAAAATGGAAACCCTTCTTATTGGATGATCATGATACTTCCCAAAAATCGAAATTTTTGATTAATGGAGGAACAATATCACCATTTTTGTTCAATAAGATACCAAAGTGGATGATTGACTCATTCCATACTAGAAATAATCGCAGGAAATCTTTTGATAACACGGATTCCTATTTCTCAATGATATCCCACGATCAAGACAATTGGCTGAATCCCGTGAAACCATTTCATAGAAGTTCATTGATATCTTCTTTTTATAAAACAAATCGACTTCGATTCTTGAATAATCTACATCACTTCTGCTTCTATTGTAACAAAAACAAAAGATTCCCTTTTTATGTGGAAAAGGCCCGTATCAAGAATTATGATTTTACGTATGGACAATTCCTCAATATCTTGTTCATTCGCAACAAAATATTTTCTTTGTGCGGCGGTAAAAAAAAACATGCTTTTGGGGAGAGAGATACTATTTCACCAATCGAGTCACAGGTATCTAACATATTCATACCTAATGATTTTCCACAAAGTGGTAACGAAAGGTATAACTTGTACAAATCTTTCCATTTTCCAATTCGATCCGATCCATTCGTTCGTAGAGCTATTTATTTGATCGCAGACATTTCGGGAACACCTCTAACAGAGGGACAAATAGTCAATTTTGAAAGAACTTATTGTCAACCTCTTTCGGATATGAATCTATCTGATTCAGAAGGGAAGAACTTGCATCAGTATCTCAATTTCAATTCAAACATGGGTTTGATTCACACTCCATGTTCTGAGAAATATTTACCATCCGAAAAGAGGAAAAAACGGAGTCTTTGTCTAAAGAAATGTGTTGAAAAAGGGCAGATGTATAGAACCTTTCAACGAGATAGTGCTTTTTCAACTCTCTCAAAATGGAATCTATTCCAAACATATATGCCATGGTTCCTTACTTCGACAGGGTACAAATATCTAAATTTGATATTTTTAGATACCTTTTCGGACCTATTACCGATACTAAGTAGCAGTCAAAAATTTGTATCCATTTTTCATGATATTATGCACGGATCAGATATATCATGGCGAATTCTTCAGAAAAAATGGTGTCTTCCACAATGGAATCTGATAAGTGAGATTTCGAGTAAGTGTTTACATAATCTTCTTCTGTCCGAAGAAATGATTCATCGAAATAATGAGCCACCATTGATATCGACACATCTGAGATCGCCAAATGTTCGGGAGTTCCTCTATTCAATCCTTTTCCTTCTTCTTGTTGCTGGATATCTCGTTCGTACACATCTTCTCTTTGTTTCCCGAGCCTATAGTGAGTTACAGACAGAGTTCGAAAAGGTCAAATCTTTGATGATTCCATCATACATGATTGAGTTGCGAAAACTTCTGGATAGGTATCCTACATCTGAACTGAATTCTTTCTGGTTAAAGAATCTCTTTCTAGTTGCTCTGGAACAATTAGGAGATTTTCTAGAAGAAATGCGGGGTTTTGCTTCTGGCGGCAACATGCTATGGGGTGGTGGTCCCGCTTATGGGGTTAAATCAATACGTTCTAAGAATAAATTTTTGAATATCAATCTCATCGATCTCATAAGTATCATACCAAATCCCATCAATCGAATCACTTTTTCGAGAAATACGAGACATCTAAGTCATACAAGTAAAGAGATTTATTCATTGATAAGAAAAAGAAAAAACGTGAATGGTGATTGGATTGATGATAAAATAGAATCCTTGGTCGCGAACAGTGATTCGATTGATGATAAAGAAAGAGAATTCTTGGTTCAGTTCTCCACCTTAACGACAGAAAACAGGATTGATCAAATTCTATTGAGTCTGACGCATAGTGATCATTTATCAAAGAATGACTCTGGTTATCAAATGATTGAAGAGCCGGGAGCAATTTATTTACGATACTTAGTTGACATTCATAAAAAGTATCTAATGAATTATGAGTTCAATACACCCTGTTTAGCAGAAAGACGGATATTCCTTGCTTATTATCAGACAACCACTTATTCACAAACCTCGTGTGGGGTGAATAGTTTTCATTTCCCATCTCATGGAAAACCCTTTTCGCTCCGCTTAGCCCTATCCCCCTCTAGGGGTATTTTAGTGATAGGTTCTATAGGAACTGGACGATCCTATTTGGTCAAATACCTAGCGACAAACTCCTATCTTCCTTTCATTACAGTATTTCTGAACAAGTTCCTGGATAACAAGCCTAAGGGCTTTCTTATTGATGATAGTGACGATATTGATGATAGTGACGATATTGATGATAGTGACGATATTGATGATAGTGACGATATTGATGTGAGTGACGATATTGATGATAGTGACGATATCGACCGTGACTTTGATACGGAGCTGGAGTTTCTAACTAGGATGAATGCGCTAACTATGGGTATGATGCCGGAAATAGACCGATTTTATATCACCCTTCAATTCGAATTAGCAAAAGCAATGTCTCCTTGCATAATATGGATTCCAAACATTCATGATTTGGATGTGAATGAGTCGAATTACTTATCCCTCGGTCTATTAGTGAACTATCTCTCCAGGGATTGTGAAAGATGTTCCACTAGAAATATTCTTGTTATTGCTTCGACTCATATTCCCCAAAAAGTGGATCCCGCTCTAATAGCTCCGAATAAATTAAATACATGCATTAAGATACGAAGGCTTCTTATTCCACAACAACGAAAGCACTTTTTTACTCTTTCATATACTAGGGGATTTCACTTGGAAAAGAAAATGCTCCATACTAATGGATTCGGGTCCATAACCATGGGTTCCAATGTACGAGATCTTGTAGCACTTACCAATGAGGCCCTATCGATTAGTATTACACAGAAGAAATCAATTCTAGACACTAATATAATTAGATCCGCTCTTCATAGACAAACTTGGGATTTGCGATCCCAGGTAAGATCGGTTCAGGATCATGGGATCCTTTTCTATCAGATAGGAAGGGCTGTTGCACAAAATGTATTTCTAAGTAATTGCCCCATAGATCCTATATCTATCTATATGAAGAAGAAATCATGTAACGAAGGGGATTCTTATTTGTACAAATGGTACTTCGAACTTGGAATGAGCATGAAGAAATTAACGATACTTCTTTATCTTTTGAGTTGTTCCGCCGGATCGGTTGCTCAAGACCTTTGGTCTCTACCCGGACCCGATGAAAAAAATGGGATCACTTATTATGGACTTGTTGAGAATGATTCTGATCTAGTTCATGGCCTATTAGAAGTCGAAGGCGCTCTGGTGGGATCCTCACGTACAGAAAAAGATTGCAGTCAGTTTGATAATGATCGAGTGACATTGCTTCTTCGGCCCGAACCAAAGAGTCCCTTAGATATGATGCAAAATGGATCTTGTTCTATCCTTGATCAGAGATTTCTCTATGAAAAATACGAATCGGAGTTTGAAGAAGGGGAAGGAGTCCTCGACCCGCAACAGATAGAGGAGGATTTATTCAATCACATAGTTTGGGCTCCTAGAATATGGCGCCCTTGGGGTTTTCTATTTGATTGTATCGAAAGGCCCAATGAATTGGGATTTCCCTATTGGGCCAGGTCATTTCGGGGCAAGCGGATCATTTATGATGAAGAGGATGAGCTTCAAGAGAATGATTCGGAGTTCTTGCAGAGTGGAACCATGCAGTACCAGATACGAGATAGATCTTCCAAAGAACAAGGCTTTTTTCGAATAAGCCAATTCATTTGGGACCCTGCGGATCCACTCTTTTTCCTATTCAAAGATCAGCCCTTTGTCTCTGTGTTTTCACATCGAGAATTCTTTGCAGATGAAGAGATGTCAAAGGGGCTTCTTACTTCCCAAACAGATCCTCCTACATCTATATATAAACGCTGGTTTATCAAGAATACGCAAGAAAAGCACTTCGAATTGTTGATTCATCGCCAGAGATGGCTTAGAACCAATAGTTCATTATCTAATGGATTTTTCCGTTCTAATACTCTATCCGAGAGTTATCAGTATTTATCAAATCTGTTCCTATCTAACGGAACGCTATTGGATCAAATGACAAAGGCATTGTTGAGAAAAAGATGGCTTTTCCCGGATGAAATGAAAATTGGATTCATGTAA

>lcl|NC_012224.1_cds_YP_002720175.1_83 [gene=rpl23] [locus_tag=JacuC_p084] [db_xref=GeneID:7564821] [protein=ribosomal protein L23] [protein_id=YP_002720175.1] [location=162064..162345] [gbkey=CDS]

ATGGATGGAATCAAATATGCAGTATTTACAGACAAAAGTATTCGGTTATTGGGGAAAAATCAATATACTTTTAATGTCGAATCAGGATCAACTAGGACAGAAATAAAGCATTGGGTCGAGCTCTTCTTTGGTGTCAAGGTAATAGCTATGAATAGCCATCGACTCCCGGGAAAGGGTAGAAGAATGAGACCTATTATGGGACATACAATGCATTACAGACGTATGATCATTACGCTTCAACCGGGTTATTCTATTCCACCTCTTAGAAAGAAAAGAACTTAA

>lcl|NC_012224.1_cds_YP_002720176.1_84 [gene=rpl2] [locus_tag=JacuC_p085] [db_xref=GeneID:7564822] [protein=ribosomal protein L2] [protein_id=YP_002720176.1] [location=join(162364..162756,163386..163856)] [gbkey=CDS]

ATGGCGATACATTTATACAAAACTTCTACCCCGAGCACACGCAATGGAGCCGTAGACAGTCAAGTGAAATCCAATACACGAAATAATTTGATCTATGGACAGCATCGTTGTGGTAAAGGACGTAATGCCAGAGGAATCATTACCGCAAGGCATAGAGGGGGGGGTCATAAGCGTCTATACCGTAAAATCGATTTTCGACGGAATGAAAAAGACATATATGGTAGAATCGTAACCATAGAATACGACCCTAATCGAAATGCATACATTTGTCTCATACACTATGGGGATGGTGAGAAGAGATATATTTTACATCCCAGAGGGGCTATAATTGGAGATACCATTATTTCTGGTACAGAAGTTCCTATAAAAATGGGAAATGCCCTACCTTTGAGTGAGGTTTTGATTGATCAAAAAGAAGAATCTACTTCAACCGATATGCCCTTAGGCACGGCCATACATAACATAGAAATCACACTTGGAAAGGGTGGACAATTAGCTAGAGCTGCAGGTGCTGTAGCGAAACTGATTGCAAAAGAGGGGAAATCGGCCACATTAAAATTACCTTCTGGGGAGGTTCGTTTAATATCCAAAAACTGCTCAGCAACAGTCGGACAAGTAGGCAATACTGGGGTGAACCAGAAAAGTTTGGGTAGAGCCGGATCTAAATGTTGGCTAGGTAAGCGTCCTGTAGTAAGAGGAGTAGTTATGAACCCTGTAGACCATCCCCATGGGGGTGGTGAAGGGAGGGCTCCAATTGGTAGAAAAAAACCCGCAACCCCTTGGGGTTATCCTGCACTTGGAAGAAGAAGTAGAAAAAGGAATAAATATAGTGATAATTTGATTCTTCGTCGACGGAGTAAATAG

**4. *Manihot esculenta***

>lcl|NC_010433.1_cds_YP_001718416.1_1 [gene=rps12] [locus_tag=MaesCp046] [db_xref=GeneID:5999972] [protein=ribosomal protein S12] [exception=trans-splicing] [protein_id=YP_001718416.1] [location=complement(join(103390..103415,103952..104183,74362..74475))] [gbkey=CDS]

ATGCCAACTATTAAACAACTTATTAGAAACACAAGACAGCCAATCAGAAATGTCACCAAATCCCCCGCTCTTGGGGGATGTCCTCAGCGCCGAGGAACATGTACTAGGGTGTATACTATCACCCCCAAAAAACCAAACTCTGCCTTACGTAAAGTTGCCAGAGTACGATTAACCTCTGGATTTGAAATCACTGCTTATATACCTGGTATTGGCCATAATTTACAAGAACATTCTGTAGTCTTAGTAAGAGGGGGAAGGGTTAAGGATTTACCCGGTGTGAGATATCACATTGTTCGAGGAACCCTAGATGCTGTCGGAGTAAAGGATCGTCAACAAGGGCGTTCTAAATATGGGGTCAAAAAGCCAAAATAA

>lcl|NC_010433.1_cds_YP_001718417.1_2 [gene=psbA] [locus_tag=MaesCp001] [db_xref=GeneID:5999949] [protein=photosystem II protein D1] [protein_id=YP_001718417.1] [location=complement(462..1523)] [gbkey=CDS]

ATGACTGCAATTTTAGAGAGACGCGAAAGCGAAAGCCTATGGGGTCGTTTCTGTAACTGGATAACCAGCACTGAAAACCGTCTTTACATTGGATGGTTTGGTGTTTTGATGATCCCTACTTTATTGACCGCAACTTCTGTATTTATTATCGCTTTCATTGCTGCCCCTCCGGTAGATATTGATGGTATTCGTGAACCTGTTTCTGGATCTCTACTTTATGGAAACAATATTATTTCTGGTGCCATTATTCCTACTTCTGCGGCTATAGGTTTGCATTTTTACCCAATATGGGAAGCGGCATCTGTTGATGAATGGTTATACAATGGCGGTCCTTATGAGCTAATTGTTCTACACTTCTTACTTGGTGTAGCTTGTTACATGGGTCGTGAGTGGGAACTTAGTTTCCGTCTGGGTATGCGCCCTTGGATTGCTGTTGCATATTCAGCTCCTGTTGCAGCTGCTACTGCTGTTTTCTTGATCTATCCAATTGGTCAGGGAAGCTTTTCTGATGGTATGCCTCTAGGAATCTCTGGTACTTTCAACTTTATGATTGTATTCCAGGCTGAGCACAACATCCTTATGCACCCATTTCACATGTTAGGCGTAGCTGGTGTATTCGGCGGCTCCCTATTCAGTGCTATGCATGGTTCCTTGGTAACCTCTAGTTTGATCAGGGAAACCACAGAAAATGAATCTGCTAATGAAGGTTACAGATTCGGTCAAGAGGAAGAAACTTATAATATCGTAGCTGCTCATGGTTATTTTGGCCGATTGATCTTCCAATATGCTAGTTTCAACAACTCTCGTTCTTTACATTTCTTCCTAGCTGCTTGGCCTGTAGTAGGTATTTGGTTCACTGCTTTAGGTATTAGCACTATGGCTTTCAACCTAAATGGTTTCAATTTCAACCAATCTGTAGTTGATAGTCAAGGTCGTGTAATTAATACCTGGGCTGATATTATTAACCGTGCTAACCTTGGTATGGAAGTTATGCATGAACGTAATGCTCATAACTTCCCTCTAGACCTAGCTGCTGTCGAAGCTCCATCTACAAATGGATAA

>lcl|NC_010433.1_cds_YP_001718418.1_3 [gene=matK] [locus_tag=MaesCp002] [db_xref=GeneID:5999999] [protein=maturase K] [protein_id=YP_001718418.1] [location=complement(2063..3583)] [gbkey=CDS]

ATGGAGGAAAGATATTTAGAATTAGATAGATCTCGAAAAAACGACTTCCTATACCCATTTATCTTTCGGGAGTATATTTATACATTCGCTCATGATCATAGTTTAAATAGATCTATTTTGTTGGAAAATGTAGGTTATGACAATAAATCTAGTTTATTAATTGTAAAACGTTTAATTACTCGAATGTATCAACAGAATCATTTGATTATTTCTGCTAATGATTCTAACCAAAATCTATTTTTTAGGTACAACAAGAATTTGTATTATCAAATGATATCAGAGGGCTTTGCAGTTATTGTGGAAATTCCATTTTCCCTACGATTAGTATCTTCTTTAGATTTAGAAAGGTCAGAGATAGTAAAATCTCATAAATTACGATCAATTCATTCAATATTTCCTTTTTTAGAGGACAAATTTCCACATTTAAATTATGTGTCAGATATATTAATACCTTACCCCATCCATCTAGAAAAATTGGTTCAAACCCTTCGCTATTGGGTGAAAGATCCCTCTTCTTTGCATTTATTACGACTCTTTCTTCATGAGTATTGGAATTTGAACAGTCTTATTATTCCAAAGAAATTTATTACTATTTTTATAAAAAGGAATCCAAGATTTTTCTTGTTCCTATATAATTCTCATGTATATGAATACGAATCCATCTTCTTTTTTCTCCGTAACCAATCCTTTCATTTACGATCAATATTTTTGCGAGTCCTTCTTGAACGAATTTTTTTCTATGGAAAAATAGAACATTTTGCGGAAGTCTTTGCTAATGATTTTCAGGCCATCTTGTGGTTGTTCAAGGATCCTTTCATGCATTATGTTAGATATCAAGGAAAATCAATTCTGGCTTCAAAAGATCGGCCTTTTCTGATGAAAAAATGGAAATATTACCTTGTCAACTTATGTCAATGTCATTTTTATGTCTGGTTTCAACCAGAAAAGATCTATATAAATTCATTATCCAAGCATTCTCTCAACTTTTTGGGCTATCTTTCAAATGTACAATTAAATCCTTTGGTGGTACGGAGTCAAATGTTAGAAAATTCATTTATAATAGATAAAGATAGTACTATGAAGAAACTCGATACAATAGTTCCAATTATTCCTTTAATTGGATCATTGGCAAAAACGAAATTTTGTAACGCAGTAGGACATCCCATTAGTAAACCGATTCGGGCGGATTCGGCGGATTCTGATATTATCGACCGATTTGTGCGTATATGCAGAAATCTTTCTCATTATTATAGCGGATCCTCAAAAAAAAAGAGTTTGTATCGAATAAAATATATACTTCGACTTTCTTGTGTTAAAACTTTGGCCCGTAAACACAAAAGTACTGTACGCGCTTTTTTGAAAAGATTAGGTTCGGAATTATTAGAAGAGTTTTTTACGGAGGAAGAACAGATTCTTTCTTTGATCTTTCCAAAAGTTTCTTCTAGTTCGCGCAGGTTATATAGAGGACGTGTTTGGTATTTGGATATTATTTCTATCAATGATTTGGCCAATCATGAATAA

>lcl|NC_010433.1_cds_YP_001718419.1_4 [gene=rps16] [locus_tag=MaesCp003] [db_xref=GeneID:6000059] [protein=ribosomal protein S16] [protein_id=YP_001718419.1] [location=complement(join(5187..5413,6316..6355))] [gbkey=CDS]

ATGGTAAAACTTCGTTTGAAACGATGTGGTAGAAAGCAACGAACCGTTTATCGAATCGTTGCAATTGATGTTCGATCCCGAAGAGAAGGAAGAGATCTTCGGAAAGTGGGTTTTTATGATCCGATAAAAAATCAAACCTATTTAAACGTTCCTGCTATTCTATATTTCCTTGAAAAAGGCGCTCAACCTACAGGAACTGTTCGTGATATTTTAAGGAAGGCGGGGGTTTTTACGGAACTTCGCCTTAATCAAACGCAATTTACTTAA

>lcl|NC_010433.1_cds_YP_001718420.1_5 [gene=psbK] [locus_tag=MaesCp004] [db_xref=GeneID:6000052] [protein=photosystem II protein K] [protein_id=YP_001718420.1] [location=7958..8143] [gbkey=CDS]

ATGTTTAATTTTTTTAGTTTAATTTGTATCTGTTTTAATTCTGCCATTTATTCAAGCAATTTTTTCTTTACAAAATTGCCCGAAGCCTACGCCTTTTTGAATCCAATCGTAGATGTTATGCCAGTAATCCCTGTACTCTTTTTTCTATTAGCCTTTGTTTGGCAAGCTGCTGTAAGTTTTCGATGA

>lcl|NC_010433.1_cds_YP_001718421.1_6 [gene=psbI] [locus_tag=MaesCp005] [db_xref=GeneID:5999990] [protein=photosystem II protein I] [protein_id=YP_001718421.1] [location=8557..8667] [gbkey=CDS]

ATGCTTACTCTCAAACTCTTTGTTTACACAGTAGTGATATTCTTTGTTTCTCTCTTCATCTTTGGATTTTTATCTAATGATCCAGGACGTAATCCTGGACGTGAAGAATAA

>lcl|NC_010433.1_cds_YP_001718422.1_7 [gene=atpA] [locus_tag=MaesCp006] [db_xref=GeneID:6000076] [protein=ATP synthase CF1 alpha subunit] [protein_id=YP_001718422.1] [location=complement(11244..12767)] [gbkey=CDS]

ATGGTAACCATTCGAGCCGACGAGATTAGTAATATTATCCGCGAACGTATTGAGCAATATAATAGGGAAGTAAAGATTGTAAATACTGGTACCGTACTTCAAGTAGGCGACGGCATTGCTCGTATTTATGGTCTTGATGAAGTAATGGCAGGCGAATTAGTAGAATTTGAAGAGGGTACAATAGGCATTGCTCTGAATTTGGAATCAAATAATGTCGGTGTTGTATTAATGGGTGACGGTTTAATGATACAAGAGGGAAGCTCCGTAAAAGCAACAGGAAGAATTGCTCAGATACCTGTGAGTGAGGCTTATTTGGGTCGTGTTATAAATGCCCTAGCTAAACCTATTGACGGTCGAGGTGAAATTTCAGCTTCTGAATCTCGGTTAATTGAATCTCCTGCTCCAGGTATTATTTCGAGACGTTCTGTATATGAGCCTCTTCAAACAGGACTTATTGCTATTGATTCGATGATCCCTATAGGACGTGGTCAACGAGAATTAATTATTGGGGACAGACAGACTGGTAAAACAGCAGTAGCCACAGATACAATTCTCAATCAACAAGGACAAAATGTAATATGTGTTTATGTAGCTATTGGGCAAAAAGCGTCTTCTGTGGCTCAAGTAGTGACTACTTTACAGGAAAGAGGGGCAATGGAGTACACTATTGTGGTAGCCGAAACGGCGGATTCTCCGGCTACATTACAATACCTCGCCCCTTATACGGGAGCGGCTCTGGCTGAATATTTTATGTACCGTGAACGACACACTTTAATCATTTATGATGATCTCTCCAAACAAGCGCAGGCTTATCGCCAAATGTCTCTTCTATTACGAAGACCACCTGGTCGTGAAGCTTATCCAGGAGATGTCTTTTATTTGCATTCACGCCTTTTGGAAAGAGCCGCTAAATTAAGTTCTCGTTTAGGTGAAGGAAGTATGACTGCTTTACCAATAGTTGAGACCCAATCAGGAGACGTTTCGGCTTATATTCCTACTAATGTAATTTCCATTACAGATGGACAAATATTCTTATCCGCCGATCTATTCAATGCTGGAATCAGGCCTGCTATTAATGTGGGTATTTCTGTTTCCAGAGTAGGATCCGCAGCTCAAATAAAAGCTATGAAACAAGTAGCTGGTAAGTTAAAATTGGAATTGGCGCAATTCGTAGAATTAGAAGCCTTTGCGCAATTCGCTTCTGATCTCGATAAAGCTACTCAGAATCAATTGGCAAGAGGTCAACGACTACGCGAGTTGCTCAAACAATCCCAATCCGCCCCTCTCACGGTGGAGGAACAGATAATGACTATTTATACCGGAACGAATGGTTATCTTGATTCATTAGAAATTGGACAAGTAAGGAAATTTCTCGTTGAGTTACGTACCTACTTAAAAACGAATAAACCTCAGTTCCAAGAAATCATATCTTCTACCAAAACATTCACCGAAGAAGCAGAAACCCTTTTGAAAGAAGCTATTCAGGAACAGAAGGAACGTTTTGTAATTCAGGAACAAGTATAA

>lcl|NC_010433.1_cds_YP_001718423.1_8 [gene=atpF] [locus_tag=MaesCp007] [db_xref=GeneID:6000008] [protein=ATP synthase CF0 B subunit] [protein_id=YP_001718423.1] [location=complement(12838..13392)] [gbkey=CDS]

ATGAAAAATATAACCGATTCTTTCGTTTCCTTGGGTCACTGGCCATCCGCCGGGAGTTTCGGGTTTAATACCGATATTTTAGCAACAAATCTAATAAATCTAAGTGTAGTCCTTGGTGTATTGATTTTTTTTGGAAAGGGGGTGTTAAGTGATTTATTAGATAATCGAAAACAAAGGATTTTGGATACTATTCGAAATTCAGAAAAACTACGCGAGGGGGCTATTGAACAGCTGGAAAAAGCCCGGGCCCGCTTACGGAAAGTGGAAATAGAAGCAGATCAGTTTCGAACGAATGGATATTCTGAGATAGAACGAGAAAAATTGAATTTGATTAATTCAACTTATAAGACTTTGGAACAATTAGAAAATTACAAAAATGAAACCATTCATTTTGAACAACAACGAACGATTAATCAAGTCCGACAACGGGTTTTCCAACAAGCCTTACAAGGAGCTCTAGGAACTCTGAATAGTTGTTTGACCAACGAGTTGCATTTACGTACCATCAATGCTAATCTTGGCATGTTTGGCGCGATAAAAGAAATAACTGATTAG

>lcl|NC_010433.1_cds_YP_001718424.1_9 [gene=atpH] [locus_tag=MaesCp008] [db_xref=GeneID:5999963] [protein=ATP synthase CF0 C subunit] [protein_id=YP_001718424.1] [location=complement(13966..14211)] [gbkey=CDS]

ATGAATCCATTGATTTCTGCCGCTTCCGTTATTGCTGCTGGGTTGGCCGTTGGGCTTGCTTCTATTGGACCTGGGGTTGGTCAAGGTACTGCCGCGGGCCAAGCTGTAGAAGGTATTGCAAGACAACCCGAGGCGGAGGGAAAAATACGAGGTACTTTATTGCTTAGTCTGGCTTTTATGGAAGCTTTAACGATTTATGGACTGGTTGTAGCATTAGCACTGTTATTTGCGAATCCTTTTGTTTAA

>lcl|NC_010433.1_cds_YP_001718425.1_10 [gene=atpI] [locus_tag=MaesCp009] [db_xref=GeneID:5999986] [protein=ATP synthase CF0 A subunit] [protein_id=YP_001718425.1] [location=complement(15427..16176)] [gbkey=CDS]

ATGAATGTTCTATCATGTTCCATCAACACACTAACACTAAGGGGGTTATATGATATATCCGGTGTGGAAGTAGGCCAGCATTTCTATTGGAAAATAGGAGGTTTCCAAGTCCATGCCCAAGTGCTTATTACTTCTTGGGTTGTAATTGCTATCTTATTAGGTTCGGCCATTGTAGCTGTTCGGAATCCACAAACCATTCCAACTGGAGGTCAGAATTTCTTCGAATACGTCCTTGAATTCATTCGAGATGTGAGCAAAACTCAGATTGGAGAGGAATATGGCCCGTGGGTCCCCTTTATTGGAACTATGTTTCTATTTATTTTTGTTTCTAATTGGGCGGGGGCGCTTTTACCTTGGAAGATCATACAGTTACCTCACGGGGAGTTAGCCGCACCTACGAATGATATAAATACTACCGTTGCTTTAGCTTTACTTACGTCAATAGCATATTTTTATGCGGGTCTTAGCAAAAAAGGATTAGGTTATTTCAGTAAATACATTCAACCAACTCCAATCCTTTTACCCATTAACATTTTAGAAGATTTCACAAAACCTTTATCACTTAGCTTTCGACTTTTCGGCAATATATTAGCGGATGAATTAGTAGTTGTTGTTCTTGTTTCTTTAGTACCTTCAGTGGTTCCTATACCTGTCATGTTCCTTGGATTATTTACAAGTGGTATTCAAGCTCTTATTTTTGCAACTTTAGCTGCGGCTTATATAGGCGAATCCATGGAGGGGCATCATTGA

>lcl|NC_010433.1_cds_YP_001718426.1_11 [gene=rps2] [locus_tag=MaesCp010] [db_xref=GeneID:6000041] [protein=ribosomal protein S2] [protein_id=YP_001718426.1] [location=complement(16437..17147)] [gbkey=CDS]

ATGATAAGAAGATATTGGAACATTAATTTTGAAGAGATGATGAAAGCAGGAGTTCATTTTGGTCATGGTACTAGAAAATGGAATCCGAGAATGGCACCTTATATCTCTGCAAAGCGTAAAGGTATTCATATTACAAATCTTACTAGAACTGCTCGTTTTTTATCAGAAGCTTGTGATTTAGTTTTTGATGCAGCAAGTAGGAGAAAGCAATTCTTAATTGTTGGTACCAAAAATAAAGCAGCGGATTCAGTAGCGCGGGCTGCAATAAGGGCTCGGTGTCATTATGTTAATAAAAAATGGCTCGGCGGTATTTTAACGAATTGGTCCACTACAGAAACTAGACTTCAAAAGTTCAGGGACTTGAGAATGGAACAAAAGGCAGGTAGACTCAACCGTCTTCCGAAAGGAGATGCGGCTCGATTGAAGAGACAGTTAGCTCACTTGCAAACATATCTGGGCGGGATTAAATATATGACGGGGTTACCCGATATTGTAATAATCGTTGATCAGCAAGAAGAATATACGGCTCTTCGGGAATGTATGACTTTGGGAATTCCAACAATTTGTTTAATTGATACAAACTGTGACCCGGATCTCGCAGATATTTCGATTCCAACGAATGATGACGCTATAGCTTCAATCCGATTAATTCTTAATAAATTAGTATTTGCAATTTGTGAGGGTCGTTCTAGCTATATACGAAATCCCTGA

>lcl|NC_010433.1_cds_YP_001718427.1_12 [gene=rpoC2] [locus_tag=MaesCp011] [db_xref=GeneID:5999961] [protein=RNA polymerase beta'' subunit] [protein_id=YP_001718427.1] [location=complement(17421..21602)] [gbkey=CDS]

ATGGAGGTACTTATGGCCGAACGGGCCAATCTGGTCTTTCACAATAAAGCGATAGATGGAACTGCCATTAAACGACTTATTAGCAGATTAATAGATCATTTTGGAATGGCATATACATCACACATCCTGGATCAAGTAAAGACTCTGGGTTTCCAGCAAGCCACCGCTACATCCATTTCATTAGGAATTGATGATCTTTTAACAATACCTTCTAAGGGATGGCTAGTCCAAGATGCTGAACAACAAAGTTTGATTTTGGAAAAACACTATCATTATGGAAATGTACACGCGGTAGAAAAATTACGCCAATCTATTGAGATATGGTATGCTACAAGTGAATATTTGCGACAAGAAATGAACCTGAATTTTAGGATGACGGAACCCTTTAATCCAGTCCATATAATGTCTTTTTCGGGAGCTAGGGGAAATACATCTCAAGTACACCAATTAGTAGGTATGAGAGGATTAATGTCGGATCCACAAGGACAAATGATTGATTTACCCATTCAAAGCAATTTACGCGAGGGACTGTCTTTAACAGAATATATCATTTCTTGCTATGGAGCCCGAAAAGGGGTTGTCGATACTGCTGTACGAACATCAGATGCTGGATATCTTACGCGTAGACTTGTTGAAGTAGTTCAACACATTGTTGTACGTAGAACAGATTGTGGGACCGCCCGAGGGATCTCTGTGAGTCCTCGAAATGGGATGATGCCGGAAAGAATTTTTATTCAAACATTCATTGGTCGTGTATTAGCAGACAATATATATATGGGTCTACGATGCATTGCCATTCGAAATCAAGATATTGGGATTGGGCTTGCCAATCGATTCATAACCTTTCGAACACAAACAATATCTATTCGAACTCCCTTTACTTGTAGGAGTACGTCTTGGATCTGTCGATTATGTTATGGTCGGAGTCCTACTCATGGCGATCTAGTAGAATTGGGGGAAGCCGTAGGTATTATTGCGGGTCAATCCATTGGAGAGCCGGGTACTCAACTAACATTAAGAACGTTTCATACCGGCGGAGTATTCACAGGGGGTACTGCAGAACATGTACGAGCCCCCTCTAATGGAAAAATCAAATTTAATGAGGATTTGGTTCATCCCATACGTACACGTCATGGGCATCCTGCTTTTCTATGTTATATAGACTTGTATGTAACTATTAAGAGTCAAGATATTATACATAACGTGACTATTCCACCAAAAAGTTTCCTTTTAGTTCAAAATGATCAATATGTAGAATCAGAACAAGTGATTGCTGAAATTCGGGCGGGAGCATACACTTTGAATTTTAAAGAGAAGGTCCGAAAACATATTTATTCCGATTCAGAAGGGGAAATGCACTGGAGTACTGATGTGTACCATGCACCCGAATTTACATATAGTAATGTCCATCTCTTACCAAAAACAAGTCATTTATGGATATTATCAGGAAGTTCGTGCAGATCCAGTATAGTTCCTTTTTCACTACACAAGGATCAAGATCAAATGAATGTTCATTCTCTTTCTGTCAAAAGAAGATATATTTCTAGTCCTTCCGTAAATAATGATCAAGTGAAACACAAATTCTTTAGTTCAGATTTTTCGGGTAAAAAAGAAAGTGGGATTCCTGATTATTCAGAACTTAATCGAAGCATATGTACTGGTCATTGTAATCTCATATATTCTACTATTCTCTACAAGAATTCTGATTTATTGGCAAAGAGGCGAAGAAATAAATTCATCATTCCATTCCAATCAATTCAAGAACGAGAGAAAGAACTAATGACCCAATCCGCTATCTCGATTGAGATACCTATAAATGGTATTTTCCGTAGAAATAGTGTTTTTGCTTATTTCGACGATCCCCAATACCGAAAAAAGAGTTCAGGAATTACTAAATATGGGGCTATAGGGGTGCATTCAATCGTCAAAAAAGAGGATTTGATTGAGTATCGGGGAGTCAAAGAATTTAAGCCAAAATACCAAACGAAAGTGGATCGCTTTTTTTTCATTCCCGAGGAAGTGTATATTTTACCCGAATCTTCTTCCCTAATGGTACGGAACAATAGTATTATTGGAGTAGATACACAAATCGCTTTAAATACAAGAAGTCGGGTGGGCGGATTGGTCCGAGTGGAGAGAAAAAAAAAAAAAATGGAACTTAAAATCTTTTCTGGAGATATCCATTTTCCGGGAGAGACAGATAAAATATCCCGACACAGTGATATCTTAATACCACCAGGAACGGTAAAAACAAATTCTAAGGAATCAAAAAAAGTGAAAAATTGGATCTATATCCAACGAATCACACCTACCAAGAAAAAGTATTTTGTTTTGGTTCGACCAGTAATCATATATGAGATAGCGAACGGTATAAATTTAGAAACACTTTTCCCCCAGGATCTATTGCAGGAAAAGGATAATCTGAAACTTCGAGTTGTCAATTATATTCTTTATGGGACTGGTAAACCCATTCGGGGAATTTCTGACACAAGTATTCAATTAGTTCGTACTTGTTTAGTGTTGAATTGGGACCAAGACAAAAAAAGTTCTTCTATCGAAGAGGCCCGCGCTGCTTTTGTTGAAATAAGCACAAATGGTCTGATTCGTGATTTCCTAAGAATCAACCTAGTGAAATTCCATATTTCATATATCGGTAGAAAAAGGAATGATCCATCAGGTTCAGAACCGATCTCTAATAATGGGTCAGATCGTACCAATATTAATCCATTTTATCCCATTTATTCCAAGACAAGGGTTCAACAATCACTTAAACAAAATCAAGGAACTATTAGTACGTTGTTGAATATAAATAAGGAATGTCAATCTTTGATAATTTTGTCATCATCTAATTGTTTTCAAATGGATCCATTCAACGATGTAAAACATCACAATGTAATAAAAGAATCAATTAAAAGAGATCCTATAATTCCAATTAGAAATTCGTTGGGCCCTTTAGGAACAGCCCTTCAAATTGCGAATTTTTATTTATTTTACCATTTAAATTTAATAACTCATAATCAGATCTCGGTAACTAAATATTCGAAACTTTACAATTTAAAACAGACTTTTCAAGTACTTAAATATTATTTAATGGATGAAAACGGGAGAATTGTTAATCCCGATCCATGCAGTAACAGCGTTTTGAATCCATTCAATTTGAATTGGTATTTTCTCCATCATAATTATTGTGAATCTTTCTTCACAATAATTAGCCTGGGACAGTTTATTTGTGAAAATTTATGTATGGCCAAAAACGGACCACATCTAAAATCGGGTCAAGTTATAATTGTTCACATTGACTCTGTAGTAATAAGATCCGCTAAGCCTTATTTGGCCACTCCAGGAGCAACCGTTCATGGCCATTATGGAGAAATCCTTTACGAAGGAAATACATTAGTTACATTTATATATGAAAAATCGAGATCTGGCGATATAACGCAGGGTCTTCCAAAAGTGGAACAAGTGTTAGAAGTGCGTTCAATTGATTCAATATCAATAAACCTAGAAAAGAGAGTGGAGGGTTGGAACGAGTGTATAACAAGAATTCTGGGAATTCCTTGGGGATTCTTGATTGGTACTGAGCTAACTATAGTGCAAAGTCGTATCTCTTTAGTTAATAAGATCCAAAAGGTTTATCGATCCCAAGGGGTGCAGATCCATAATAGGCATATAGAAATTATTGTACGTCAAATAACATCAAAAGTATTGGTTTCAGAAGACGGAATGTCTAATGTTTTTTCACCCGGAGAACTAATTGGATTGTTGCGAGCGGAACGAACGGGACGCGCTTTGGAAGAAGCCATCTGTTACGGGGCCATATTATTGGGAATAACGCGAGCATCTCTGAATACTCAAAGTTTCATATCCGAGGCTAGTTTTCAAGAAACTACTCGCGTTTTAGCAAAAGCTGCTCTCCGCGGTCGTATCGATTGGTTGAAAGGCCTGAAAGAAAACGTTGTTCTAGGCGGTATGATACCCGTTGGTACCGGATTCAAAGGATTAGTGCAAGGCTCAAGGCAACATAAGAACATTCCTTTGAAAACCAAAAAGAAGAATTTATTCGAGGGGGAATTTAGAGATAGAGATATCTTATTCCACCACAGAGAGTTATTTGATTCTTGCATTTCCAAAAATTTATATGATACATCAGAACAATCATTTATAGGATTTAATGATTCCTAA

>lcl|NC_010433.1_cds_YP_001718428.1_13 [gene=rpoC1] [locus_tag=MaesCp012] [db_xref=GeneID:6000060] [protein=RNA polymerase beta' subunit] [protein_id=YP_001718428.1] [location=complement(join(21771..23381,24158..24589))] [gbkey=CDS]

ATGATTGATCGGTATAAACATCAACAACTCCGAATTGGATCAGTTTCGCCTCAACAAATAAGTGCTTGGGCCAATAAAATCCTACCTAACGGAGAGATTGTTGGAGAGGTGACAAAACCCTATACTTTTCATTACAAAACCAATAAACCTGAAAAGGATGGATTATTTTGTGAAAGAATTTTTGGGCCAATAAAAAGTGGAATTTGTGCGTGTGGAAATTATCGAGTAATCAGGAATGAAAAAGAAGACCAAAAATTTTGTGAACAATGCGGAGTCGAATTTGTTGATTCTCGGATACGAAGATATCAAATGGGCTACATCAAACTGGCATGCCCAGTAACTCATGTGTGGTATTTGAAACGTCTTCCTAGTTATATCGCAAATCTTTTAGATAAACCTCTTAAAGAATTAGAAGGCCTAGTATACTGCGATTTTTCTTTTGCTAGGCCCATAGCTAAAAAACCTACTTTTTTACGATTACGAGGTTCATTCGAATATGAAATCCAATCCTGGAAATACAGTATTCCGCTTTTTTTTACTACCCAATGCTTCGATACATTTCGAAATCGAGAAATTTCTACAGGAGCTGGTGCTATCCGAGAACAATTAGCCGATCTGGATTTGCGAATTATTATAGATTATTCATCGGTAGAATGGAAAGAATTAGGGGAAGAAGGGCCTACCGGGAATGAATGGGAAGATCGAAAAGTTGGAAGAAGAAAGGATTTTTTGGTTAGACGTGTGGAATTAGCTAAGCATTTTATTCGAACAAATATCGAACCAGAATGGATGGTTTTATGTCTATTACCAGTTCTTCCTCCCGAGTTGAGACCGATCATTCAGATAGATGGGGGTAAACTAATGAGTTCAGATATTAATGAACTCTATAGAAGAGTTATCTATCGGAACAATACTCTTATTGATCTATTAACAACAAGTAGATCTACCCCAGGGGAATTAGTAATGTGTCAGGAGAAATTGGTACAAGAAGCCGTGGATACACTTCTTGATAATGGAATCCGCGGACAACCAATGAGGGACGGTCATAATAAGGTTTACAAGTCGTTTTCGGATGTAATTGAAGGCAAAGAAGGAAGATTTCGTGAGACTATGCTTGGCAAACGGGTTGATTATTCGGGGCGTTCTGTCATTGTCGTAGGCCCCTCACTTTCATTACATCGATGTGGATTGCCTCGCGAAATAGCAATAGAGCTTTTCCAGATATTTGTAATTCGTGGTCTAATTAGACAACATCTTGCTTCGAACATAGGAGTTGCTAAGAGTAAAATTCGGGAAAAAGAGCCAATTGTATGGGAAATACTTCACGAAGTTATGCAGGGGCATCCAGTATTACTGAATAGAGCGCCGACTCTGCATAGATTAGGCATACAGGCATTCCAACCCATTTTAGTGGAAGGCCGCGCTATTTGTTTACATCCATTAGTTTGTAAGGGATTCAATGCAGACTTTGATGGGGATCAAATGGCTGTTCATGTACCCTTATCGTTGGAGGCTCAAGCGGAGGCTCGTTTACTTATGTTTTCTCATATGAATCTTCTGTCTCCAGCTATTGGAGATCCCATTTCCGTACCAACTCAAGATATGCTTATTGGGCTCTATGTATTAACAAGCGGGAATCGTCGAGGTATTTGTGCAAATAGGTATAATCCATGTAATCGCAGAAATTATCAAAATAAAAGAATTGACGGTAATAACGATAAATATACGAAAGAACCCCTTTTTTCTAATTCCTATGATGCACTTGGCGCTTATCGGCAGAAAAGAATCCATTTAGATAGTCCTTTGTGGCTCCGGTGGCAGCTAGATCAACGCGCTATTACTTCAAGAGAAGCTCCCATCGAAGTTCACTATGAATCTTTGGGTACCTATCATGAGATTTATGAACACTATCTAATAGTAAGAAATATAAAAAAAGAAATTCTTTGTATATACATTCGAACTACTGTTGGTCATATTTCTCTTTATCGAGAAATCGAAGAAGCTATACAAGGGTTTTGCCAAGCCTGCTCAGATGGTATCTAA

>lcl|NC_010433.1_cds_YP_001718429.1_14 [gene=rpoB] [locus_tag=MaesCp013] [db_xref=GeneID:6000006] [protein=RNA polymerase beta subunit] [protein_id=YP_001718429.1] [location=complement(24616..27828)] [gbkey=CDS]

ATGCTCGGGGATGGAAATGAGGGAATGTCTACAATACCTGGATTTAATCAGATACAATTTGAAGGATTTTGTAGATTCATTGATCAGGGCTTAACAGAAGAACTTTATAAGTTTCCAAAAATTGAAGATACAGATCAAGAAATTGAATTTCAATTATTTGTGGAAACATATCAATTAGTAGAACCCTTGATAAAAGAAAAAGATGCTGTATATGAATCACTTACATATTCTTCTGAATTATATGTATCCGCAGGATTAATTTGGAAAACCAGTAGGGATATGCAAGAACAAACAATTTTTATTGGAAACATTCCTCTAATGAATTCCCTGGGAACTTTTATAATAAATGGAATATACAGAATTGTGATCAATCAAATATTGCAGAGTCCGGGTATCTATTACCGGTCAGAGTTGGAACATAATGGAATTTCGGTCTATACCGGCACCATAATATCAGATTGGGGGGGGAGAGTAGAATTAGAGATTGATAGAAAAGCAAGGATATGGGCTCGTGTGAGTAGGAAACAGAAAATATCTATTCTAGTTCTATCATCAGCTATGGGTTTGAATCTAAAAGAAATTTTAGAGAATGTGTGCTACCCTGAAATTTTCTTATCTTTCCTGAATGATAAGGAAAAAAAAAAAATTGGGTCAAAGGAAAATGCCATTTTGGAGTTTTATCAACAATTTACTTGTGTAGGCGGAGATCCAATATTTTCTGAATCCTTATGTAAGGAATTACAAAAGAAATTCTTTCAACAAAGATGTGAATTAGGAAAGATTGGTCGATTAAATATGAACCGGAAACTGAATCTTGATATACCCCATAACAATACATTTTTGTTACCACGAGATATATTGGCAGCTGCGGATCGTTTGATTGGAATGAAATTTGGAATGGGTACACTTGACGATATGAATCATTTAAAAAATAAACGTATTCGTTCTGTAGCGGATCTCTTACAAGATCAATTCGGATTGGCTCTGATTCGTTTAGAAAATGTAGTTCGAGGGACTATATGTGGAGCAATTAGGCATAAATTGATACCGACCCCTCAAAATTTGGTAACTTCAACTCCATTAACAACCACTTATGAATCTTTTTTCGGATTACACCCATTATCTCAAGTTTTGGATCGAACTAATCCATTGACACAAATAGTTCATGGGAGAAAATTGAGTTATTTGGGTCCTGGAGGATTAACAGGACGAACTGCTAGTTTTCGGATACGAGATATCCACCCTAGTCATTATGGGCGCATTTGCCCAATTGACACGTCTGAAGGAATCAATGTTGGACTTATTGGATCTTTAGCAATTCATGCCAAGATTGGTTATTGGGGGTCTTTAGAAAGCCCATTTTATGAAATCTCTGAGGGATCAAAAAAAGTACGGATGTTTTATTTATCGCCAAATAGAGAGGAATACTATATGGTAGCGGCAGGAAATTCTTTGGCGCTGAATCGAGGTGTTCAGGAAGAACAGGTTGCTCCGGCTCGATATCGTCAAGAATTCCTGACTATTGCATGGGAACGGGTGCATCTTCGAAGTATTTTTCCCTTCCAATATTTTTCTATTGGAGCTTCCCTCATTCCTTTTATCGAGCATAATGATGCGAATCGGGCTTTAATGAGTTCTAATATGCAACGTCAAGCAGTTCCACTTTCTCGGTCCGAAAAATGCATTGTTGGAACTGGATTGGAACGCCAAGTGGCTCTAGATTCAGGGGTTCCTGCTATAGCCGAACACGAGGGAAAGATAATTTATACTGATATTGACAAGATCATTTTATCAGGTAATGGGGATACTCTACGCATTCCATTAGTTAGGTATCAACGTTCCAACAAAAATACTTGTATGCATCAAAAAACCCAGCTTCGGCGGGGTAAATGCATTAAAAAGGGACAAGTTTTAGCGGATGGTGCCGCTACAGTTGGTGGCGAACTTGCCTTGGGCAAAAACGTATTAGTCGCTTATATGCCATGGGAAGGTTACAATTTTGAGGATGCGGTACTCATTAGCGAGCGTCTGGTATATGAAGATATTTATACTTCTTTTCACATACGGAAATATGAAATTCAGACTCATGTGACAAGCCAAGGACCTGAAAGGATCACTAACGAAATACCGCATCTAGAGGCCCATTTACTCCGAAATTTAGACAAAAACGGAATTGTGATGCTAGGATCTTGGGTAGAGACGGGCGATATTTTAGTAGGTAAATTAACGCCTCAAATGGCGAAAGAATCATCGTATGCTCCGGAAGATAGATTATTAAGAGCCGTACTTGGTATTCAGGTATCTACTTCAAAAGAAACTTGTCTAAAACTACCTATAGGTGGTAGGGGTCGAGTTATTGATGTGAGATGGATCCAGAAAAAGGGGGGTTCCTGTTATAATCCGGAAAGGATTTGTGTATATATTTTACAGAAACGTGAAATCAAAGTGGGTGATAAAGTAGCTGGAAGACATGGAAATAAAGGCATCATTTCCAAAATTTTGCCTAGACAAGATATGCCTTATTTGCAAGATGGAAGACCTGTTGATATGGTCTTCAACCCATTAGGAGTACCTTCACGAATGAATGTAGGACAGATATTTGAATGCTCACTCGGGTTAGCGGGAGGTCTGCTAGATAGACATTATCGAATAGCACCCTTTGATGAGAGATATGAACAAGAGGCTTCGAGAAAACTAGTGTTTTCTGAATTATATGAAGCCAGTAAGCAAACAGCAAATCCGTGGGTATTTGAACCCGAGTATCCGGGAAAAAGTAGAATATTTGATGGAAGAACGGGGGATCCTTTTGAACAGCCTGTTATAATAGGAAAGCCTTATATCTTGAAATTAATTCATCAAGTTGATGATAAAATACATGGACGTTCCAGTGGACATTATGCACTTGTTACACAACAACCCCTTAGAGGAAGGGCCAAGCAAGGGGGACAACGGGTCGGAGAAATGGAGGTTTGGGCTCTAGAGGGGTTTGGTGTTTCTCATATTTTACAAGAAATGCTGACTTATAAATCTGATCATATTAGAGCTCGCCAAGAAGTGCTTGGTACTACGATCATTGGAGGAACAATACCTAAACCTGAAGATGCTCCCGAATCTTTTCGATTGCTCGTTCGAGAACTACGATCTTTGGCTCTGGAACTGAATCATTTCCTTGTATCTGAGAAGAACTTCCAGATTACTAGGAAGGAAGCTTAA

>lcl|NC_010433.1_cds_YP_001718430.1_15 [gene=petN] [locus_tag=MaesCp014] [db_xref=GeneID:5999991] [protein=cytochrome b6/f complex subunit VIII] [protein_id=YP_001718430.1] [location=29373..29462] [gbkey=CDS]

ATGGATATAGTAAGTCTCGCTTGGGCTGCTTTAATGGTAGTCTTTACATTTTCCCTTTCACTCGTAGTATGGGGAAGAAGTGGACTCTAG

>lcl|NC_010433.1_cds_YP_001718431.1_16 [gene=psbM] [locus_tag=MaesCp015] [db_xref=GeneID:5999981] [protein=photosystem II protein M] [protein_id=YP_001718431.1] [location=complement(30812..30916)] [gbkey=CDS]

ATGGAAGTAAATATTCTTGCATTTATTGCTACTGCACTGTTCATTCTAGTTCCTACTGCTTTTTTACTTATAATATACGTAAAAACAGTTAGTCAAGGTGATTAA

>lcl|NC_010433.1_cds_YP_001718432.1_17 [gene=psbD] [locus_tag=MaesCp016] [db_xref=GeneID:6000044] [protein=photosystem II protein D2] [protein_id=YP_001718432.1] [location=35317..36378] [gbkey=CDS]

ATGACTATAGCCCTTGGTAAATTTACCAAAGACGAAAATGATTTATTTGATATTATGGATGACTGGTTACGGAGGGACCGTTTCGTTTTTGTAGGTTGGTCCGGTCTATTGCTCTTTCCTTGTGCCTATTTCGCCGTAGGGGGTTGGTTCACAGGTACAACCTTTGTAACGTCATGGTATACCCATGGATTGGCCAGTTCCTATTTGGAAGGCTGCAACTTCTTAACCGCCGCAGTTTCTACTCCTGCTAATAGTTTAGCACATTCTTTGTTATTATTATGGGGTCCTGAAGCACAAGGAGATTTTACTCGTTGGTGTCAATTAGGTGGTTTGTGGACTTTTGTTGCTCTCCACGGTGCTTTTGGACTAATAGGTTTTATGTTACGTCAATTTGAACTTGCTCGATCTGTGCAATTGCGACCTTATAATGCAATCGCATTCTCTGGTCCAATTGCTGTTTTTGTTTCTGTATTCCTGATTTATCCATTAGGCCAGTCTGGTTGGTTTTTTGCGCCTAGTTTTGGTGTAGCAGCTATATTTCGATTCATCCTCTTTTTCCAAGGGTTTCATAACTGGACGCTGAACCCATTTCATATGATGGGAGTTGCCGGCGTATTGGGCGCTGCTCTGCTATGCGCTATTCATGGTGCTACTGTAGAAAATACTTTATTTGAAGATGGTGATGGTGCAAATACATTCCGTGCCTTTAACCCAACTCAAGCTGAAGAAACTTATTCAATGGTCACCGCTAACCGCTTTTGGTCTCAAATCTTTGGGGTTGCTTTTTCCAATAAACGTTGGTTACATTTCTTTATGTTATTTGTACCAGTAACCGGTTTATGGATGAGCGCTCTTGGAGTAGTCGGTCTGGCTCTGAATCTACGTGCCTATGACTTCGTTTCTCAGGAAATCCGTGCAGCAGAAGATCCTGAATTTGAGACTTTCTACACTAAAAATATTCTCTTAAACGAAGGTATTCGTGCTTGGATGGCGGCTCAAGATCAGCCTCATGAAAACCTTATATTCCCTGAGGAGGTTCTACCACGTGGAAACGCTCTTTAA

>lcl|NC_010433.1_cds_YP_001718433.2_18 [gene=psbC] [locus_tag=MaesCp017] [db_xref=GeneID:6000023] [protein=photosystem II 44 kDa protein] [protein_id=YP_001718433.2] [location=36362..37747] [gbkey=CDS]

GTGGAAACGCTCTTTAATGGAACTTTATCTTTAGCCGGTCGTGACCAAGAAACCACTGGTTTCGCTTGGTGGGCCGGGAATGCCCGACTTATCAATTTATCCGGTAAACTATTGGGAGCTCATGTAGCTCATGCTGGATTAATCGTATTCTGGGCCGGAGCAATGAACCTATTTGAAGTGGCTCATTTTGTACCGGAGAAGCCAATGTACGAACAAGGATTAATTTTACTTCCCCACCTAGCTACTCTAGGTTGGGGGGTAGGTCCTGGTGGGGAAGTTATAGATACCTTTCCATACTTTGTATCCGGTGTACTTCACTTAATTTCCTCTGCAGTATTGGGCTTTGGCGGTATTTATCATGCACTTCTGGGTCCTGAGACTCTTGAAGAATCTTTTCCATTTTTTGGTTATGTATGGAAAGATCGAAATAAAATGACAACAATTTTAGGTATTCACTTAATCTTGCTAGGTATAGGTGCTTTTCTTCTAGTATTCAAGGCTCTTTATTTTGGGGGCGTATATGATACCTGGGCTCCGGGGGGGGGAGATGTAAGAAAAATTACCAACTTGACCCTTAGCCCAAGTGTTATTTTTGGTTATTTACTAAAATCCCCCTTTGGAGGAGAAGGATGGATTGTTAGTGTGGACGATTTGGAAGATATAATTGGAGGGCATGTATGGTTAGGTTCCATTTGTATACTTGGTGGAATCTGGCATATCTTAACCAAACCCTTTGCATGGGCTCGCCGTGCACTTGTGTGGTCTGGAGAGGCTTACTTGTCTTATAGTTTAGGTGCTTTATCCGTTTTTGGTTTCATTGCTTGTTGCTTTGTCTGGTTCAATAATACCGCGTATCCTAGTGAGTTTTACGGACCTACTGGACCAGAAGCTTCTCAAGCTCAAGCTTTTACTTTTCTAGTTAGAGATCAACGTCTTGGGGCTAACGTGGGATCCGCTCAAGGACCTACCGGGTTAGGTAAATATTTAATGCGTTCGCCTACCGGAGAAGTTATTTTTGGAGGCGAAACTATGCGTTTTTGGGATCTGCGTGCTCCTTGGTTAGAACCTCTAAGAGGTCCAAATGGTTTGGACTTGAGTAGGTTGAAAAAAGACATTCAACCTTGGCAAGAACGCCGTTCCGCGGAATATATGACCCATGCGCCTTTAGGTTCGTTAAATTCTGTAGGTGGCGTAGCTACCGAGATCAATGCAGTCAATTATGTCTCTCCTAGAAGTTGGTTAGCTACCTCTCATTTTGTTCTAGGATTCTTCCTATTCGTAGGTCATTTATGGCATGCGGGAAGGGCTCGTGCAGCTGCAGCAGGATTTGAAAAAGGAATTGATCGTGATTTTGAACCTGTTCTTTCCATGACTCCTCTTAACTAA

>lcl|NC_010433.1_cds_YP_001718434.1_19 [gene=psbZ] [locus_tag=MaesCp018] [db_xref=GeneID:6000002] [protein=photosystem II protein Z] [protein_id=YP_001718434.1] [location=38368..38556] [gbkey=CDS]

ATGACTATTGCTTTCCAATTGGCTGTTTTTGCATTAATTGCTACTTCATCAATCTTACTGATTAGTGTACCTGTTGTTTTTTCTTCTCCTGATGGTTGGTCGAGTAACAAAAATGTTGTATTTTCCGGTACATCATTATGGATTGGATTAGTCTTTCTGGTAGGTATCCTTAATTCTCTCATCTCTTGA

>lcl|NC_010433.1_cds_YP_001718435.1_20 [gene=rps14] [locus_tag=MaesCp019] [db_xref=GeneID:6000050] [protein=ribosomal protein S14] [protein_id=YP_001718435.1] [location=complement(39647..39949)] [gbkey=CDS]

ATGGCAAGGAAAAGTTTGATTCAGCGGGAGAAGAAGAGGCAAAAATTGGAACAAAAATATCATTTGATGCGTCGATCCTCAAAAAAAGAAATAAGCAAAGTTCCGTCGTTAAGTGATAAATGGGAAATTCATGGAAAGTTACAATCCCCACCGCGGAATAGTGCACCGACACGTCTTCATCGACGTTGTTTTTCGACTGGAAGACCGAGAGCTAATTATCGAGACTTTGGGCTATCCGGACACATACTTCGTGAAATGGTTCATGCATGTTTGTTGCCGGGGGCAACAAGATCAAGTTGGTAA

>lcl|NC_010433.1_cds_YP_001718436.1_21 [gene=psaB] [locus_tag=MaesCp020] [db_xref=GeneID:6000030] [protein=photosystem I P700 chlorophyll a apoprotein A2] [protein_id=YP_001718436.1] [location=complement(40072..42276)] [gbkey=CDS]

ATGGCATTAAGATTTCCAAGGTTTAGCCAAGGCTTAGCTCAGGACCCCACTACTCGTCGTATTTGGTTTGGTATTGCTACCGCGCATGACTTCGAGAGTCATGATGATATTACGGAGGAACGTCTTTATCAGAATATTTTTGCTTCTCACTTCGGGCAATTAGCAATAATTTTTCTGTGGACTTCCGGAAATCTCTTTCATGTAGCTTGGCAAGGAAATTTTGAAGCATGGGTACAGGACCCTTTACATGTAAGACCTATTGCTCATGCAATTTGGGATCCTCATTTTGGTCAACCGGCCGTGGAAGCTTTTACTCGAGGGGGTGCTCCTGGCCCAGTGAATATCGCTTATTCTGGTGTTTATCAATGGTGGTATACAATCGGTTTACGTACTAATGAAGATCTTTATATTGGAGCTCTTTTTCTATTATTTCTTTCTGCCCTAGCCTTACTAGGGGGTTGGTTACACCTACAACCAAAATGGAAACCGAGCGTTTCGTGGTTCAAAAATGCCGAATCTCGTCTCAATCATCATTTGTCAGGACTATTCGGAGTAAGCTCTTTGGCTTGGACAGGACATTTAGTCCATGTCGCTATTCCCGGCTCCCGGGGGGAATACGTTCGATGGAATAATTTCTTAGATGTATTACCACATCCCCAAGGGTTAGGCCCGTTTTTTACAGGTCAGTGGAATCTTTATGCTCAAAATCCCGATTCAGGTAGTCATTTATTTGGTACCTCCCAAGGAGCGGGAACTGCCATTCTAACCCTTCTCGGGGGGTTCCATCCACAAACACAAAGTTTATGGCTGACCGATATTGCACACCATCATTTAGCTATTGCATTTATTTTTCTCGTTGCCGGTCATATGTATAGAACTAACTTCGGGATTGGGCATAGTATAAAAGATCTTTTAGAAGCGCATATTCCTCCGGGGGGGCGATTGGGGCGTGGACATAAGGGTCTTTATGACACAATCAACAATTCGCTTCATTTTCAATTAGGCCTTGCTCTAGCTTCTTTAGGGGTTATTACTTCCTTAGTGGCTCAACACATGTACTCATTACCTGCTTATGCGTTCATAGCGCAAGACTTTACTACTCAAGCTGCGTTATATACTCATCACCAATACATCGCAGGATTCATCATGACAGGAGCTTTTGCTCATGGAGCTATATTTTTTATTAGAGATTACAATCCGGAACAGAATGAGAATAATGTATTGGCAAGAATGTTAGACCATAAAGAAGCTATCATATCCCATTTAAGTTGGGCCAGTCTCTTTCTTGGATTCCATACTTTGGGACTTTATGTTCATAATGATGTCATGCTTGCTTTTGGTACTCCGGAGAAACAAATCTTAATCGAACCCATATTCGCCCAATGGATACAATCTGCTCACGGTAAAACTTCATATGGGTTCGATGTACTTTTATCTTCAACGAATAGTCCAGCCTTCAATGCAGGTCGAAGCATATGGTTGCCCGGCTGGTTAAATGCTATTAATGAAAATAGTAATTCATTATTCTTAACAATAGGGCCTGGAGACTTCTTGGTTCATCATGCTATTGCTCTAGGGTTACATACAACCACATTGATCTTAGTAAAAGGTGCTTTAGATGCACGCGGTTCGAAGTTAATGCCAGATAAAAAGGATTTTGGTTATAGTTTTCCTTGTGATGGTCCGGGACGCGGCGGTACTTGTGATATTTCGGCTTGGGACGCATTTTATTTGGCGGTTTTCTGGATGTTAAATACCATTGGATGGGTTACTTTTTATTGGCATTGGAAGCACATCACATTATGGCAGGGTAATGTTTCACAGTTTAATGAATCTTCCACTTATTTGATGGGATGGTTAAGAGATTATCTATGGTTAAACTCTTCACAACTTATCAATGGATATAACCCTTTTGGTATGAATAGCTTATCGGTCTGGGCGTGGATGTTCTTATTTGGACATCTTGTTTGGGCTACTGGATTTATGTTTTTAATTTCTTGGCGCGGATATTGGCAAGAATTGATTGAAACTTTAGCATGGGCTCATGAGCGTACACCTTTGGCTAATTTGATTCGATGGAGAGATAAACCAGTAGCTCTTTCCATTGTGCAAGCAAGATTGGTTGGATTAGCCCACTTTTCCGTAGGTTATATCTTCACTTATGCGGCTTTCTTGATTGCCTCTACATCAGGTAAATTTGGTTAA

>lcl|NC_010433.1_cds_YP_001718437.1_22 [gene=psaA] [locus_tag=MaesCp021] [db_xref=GeneID:5999964] [protein=photosystem I P700 chlorophyll a apoprotein A1] [protein_id=YP_001718437.1] [location=complement(42302..44554)] [gbkey=CDS]

ATGATTATTCGTTCGCCGGAACCAGAAGTCAAAATTTTGGTAGATAGGGATCCCATCAAAACTTCTTTCGAGGAATGGGCCAGACCCGGTCATTTCTCAAGAACAATAGCTAAAGGACCTGATACTACTACTTGGATCTGGAACCTACATGCTGATGCTCACGATTTCGATAGCCATACCAGTGATTTGGAGGAGATTTCTCGGAAAGTATTTAGTGCTCATTTCGGCCAACTCTCCATCATCTTTCTTTGGCTGAGTGGCATGTATTTCCACGGTGCTCGTTTTTCCAACTATGAAGCATGGCTAAGCGATCCTACTCACATTGGACCTAGCGCCCAAGTGGTTTGGCCAATAGTGGGTCAAGAAATATTGAATGGCGATGTGGGCGGGGGTTTCCGAGGAATACAAATAACCTCCGGTTTTTTTCAGATTTGGAGAGCATCTGGAATAACTAGTGAATTACAACTGTATTGTACCGCAATTGGTGCATTGGTCTTTGCAGCCTTAATGCTTTTTGCTGGTTGGTTCCATTATCACAAAGCTGCTCCAAAATTGGCTTGGTTCCAAGATGTAGAATCTATGTTGAATCACCATTTAGCGGGGCTACTAGGACTTGGGTCTCTTTCTTGGGCGGGACATCAAGTACATGTATCTTTACCAATTAACCAATTTCTAAACGCTGGAGTGGATCCTAAAGAAATCCCACTTCCTCATGAATTTATCTTGAATCGGGATCTTTTGGCTCAACTTTATCCCAGTTTTGCTGAGGGAGCAACCCCATTTTTCACCTTGAATTGGTCAAAATATTCGGACTTTCTTACTTTTCGTGGAGGATTAGATCCAGTGACTGGGGGTCTATGGCTGACCGATACTGCACACCATCATTTAGCTATTGCAATTCTTTTCCTGATAGCGGGTCACATGTATAGGACTAACTGGGGCATTGGTCATGGTATAAAAGATATTTTAGAGGCTCATAAAGGTCCATTTACAGGTCAGGGTCATAAAGGCCTATATGAGATCCTAACAACATCATGGCATGCTCAATTATCTCTTAACCTAGCTATGTTAGGTTCTTTAACCATTGTTGTAGCTCACCATATGTATTCCATGCCCCCTTATCCATATCTAGCTACTGATTATGGTACACAACTGTCATTGTTCACACATCACATGTGGATTGGTGGATTTCTCATAGTTGGTGCTGCTGCGCATGCAGCCATTTTTATGGTAAGAGACTATGATCCAACTACTCGATACAACGATCTATTAGATCGTGTCCTTAGGCATCGCGATGCAATCATATCACATCTCAACTGGGTATGTATATTTTTAGGCTTTCACAGTTTTGGTTTATATATTCATAATGATACCATGAGCGCTTTAGGGCGCCCTCAAGATATGTTTTCAGATACTGCTATACAATTACAACCTGTCTTTGCTCAATGGATACAAAACACCCATGCTTTAGCACCTGGTGCAACGGCTCCTGGTGCAACAGCAAGCACCAGTTTAACTTGGGGGGGTGGTGATTTAGTGGCAGTCGGCGGCAAGGTTGCTTTGTTACCGATTCCATTAGGAACCGCGGATTTTTTGGTACATCACATTCATGCATTTACGATTCATGTGACGGTATTGATACTTCTGAAAGGAGTTCTATTTGCCCGCAGTTCTCGTTTGATACCGGATAAAGCAAATCTTGGTTTTCGTTTTCCTTGTGATGGACCTGGAAGAGGGGGAACATGTCAAGTATCCGCTTGGGATCATGTCTTCTTAGGACTATTTTGGATGTACAATTCCATTTCGGTAGTAATATTCCATTTCAGTTGGAAAATGCAGTCAGATGTTTGGGGTAGTATAAGTGATCAAGGGGTGGTAACTCATATCACGGGAGGAAACTTTGCACAGAGTTCCATTACTATTAATGGATGGCTCCGTGATTTCTTATGGGCACAGGCGTCCCAGGTAATTCAGTCTTATGGTTCTTCATTATCTGCATATGGCCTTTTTTTCCTAGGTGCTCATTTTGTATGGGCTTTTAGTTTAATGTTTCTATTCAGCGGTCGTGGTTATTGGCAAGAACTTATTGAATCAATCGTTTGGGCTCATAATAAATTAAAAGTTGCTCCTGCTACTCAGCCTAGAGCCTTGAGCATTATACAAGGACGTGCTGTAGGAGTAACCCATTACCTTCTGGGTGGAATTGCCACAACATGGGCGTTCTTCTTAGCAAGAATTATTGCAGTAGGATAA

>lcl|NC_010433.1_cds_YP_001718438.1_23 [gene=ycf3] [locus_tag=MaesCp022] [db_xref=GeneID:5999976] [protein=photosystem I assembly protein Ycf3] [protein_id=YP_001718438.1] [location=complement(join(45304..45456,46120..46347,47072..47197))] [gbkey=CDS]

ATGCCTAGATCTCGGATAAATGGAAATTTTATTGATAAGACTTTTTCAGTTGTAGCCAATATCTTATTACGAATAATTCCGACAACTTCGGGAGAAAAAGAGGCATTTACTTATTACAGAGATGGTATGTCTGCTCAATCCGAAGGAAATTATGCAGAAGCTTTACAGAATTATTATGAAGCTATGCGGCTAGAAATTGATCCCTATGATCGAAGTTATATACTCTATAATATAGGCCTTATTCACACAAGTAATGGAGAACACACAAAAGCTTTGGAATATTATTTTCGGGCACTAGAACGAAACCCCTTCTTACCACAAGCTTTAAATAATATGGCCGTGATCTGTCATTACCGAGGAGAACAGGCCATTCGGCAGGGAGATTCTGAAATTGCGGAGGCTTGGTTCGATCAAGCCGCGGAGTATTGGAAACAAGCTATAGCGCTTACTCCCGGAAATTATATTGAAGCGCAGAATTGGTTGAAAATCACAAGGCGTTTCGAATAA

>lcl|NC_010433.1_cds_YP_001718439.1_24 [gene=rps4] [locus_tag=MaesCp023] [db_xref=GeneID:6000010] [protein=ribosomal protein S4] [protein_id=YP_001718439.1] [location=complement(48002..48607)] [gbkey=CDS]

ATGTCACGTTACCGAGGGCCTCGTTTCAAAAAAATACGCCGTCTGGGGGCTTTGCCGGGACTAACTAGTAAAAGGCCTAGAGTCGGGAGCGATCTTAGAAATCAATCACGCTCCGGTAAAAAATCTCAATATCGTATTCGTTTAGAAGAAAAACAAAAATTGCGTTTTCATTATGGTGTTACAGAACGACAATTACTTAAATACGTTCGTATCGCCGCAAAAGCCAAAGGGTCAACAGGTCAGGTTTTACTACAATTACTTGAAATGCGCTTGGATAACATCCTTTTTCGATTGGGTATGGCGTCAACTATTCCTCGAGCCCGCCAATTAGTTAATCATAGACATATTTTAGTTAATGGTCGTATAGTAGATATACCAAGTTATCGCTGCAAACCCCGAGATATTATTACAGCGAGGGATGAACAAAAATCTAGAGCTATGATTCAAAATGATCTTGATTCATCCCCCCAGGAGGAATTGCCAAAACATTTGACTCTTCACCCATTCCAATATAAAGGATTGGTCAATCAAATAATAGATAGTAAATGGGTTGGCTTGAAAATAAATGAATTGCTAGTGGTAGAATATTATTCTCGTCAGACTTAA

>lcl|NC_010433.1_cds_YP_001718440.1_25 [gene=ndhJ] [locus_tag=MaesCp024] [db_xref=GeneID:5999997] [protein=NADH dehydrogenase subunit J] [protein_id=YP_001718440.1] [location=complement(51488..51964)] [gbkey=CDS]

ATGCAGGGTCGTTTGTCTGCTTGGCTAGTCAAACATGGGCTAGTTCATAGATTTTTGGGTTTTGATTACCAAGGAATAGAGACTTTACAAATAAAGCCCGAAGATTGGCATTCCATTGCTGTCATTTTATATGTATATGGTTACAATTATCTGCGTTCGCAATGTGCCTATGATGTAGCACCGGGCGGGCTGTTAGCTAGTGTATATCATCTTACGAGAATAGAGTATGGTATAGATCAACCAGAAGAAGTATGTATAAAAGTCTTTGCCCCAAGGAAAAATCCTAGAATTCCGTCTGTTTTCTGGGTTTGGAAAAGTGCGGATTTTCAAGAAAGGGAATCTTATGATATGCTGGGAATCTTTTATGATAATCATCCGCGTCTGAAACGTATCTTAATGCCGGAAAGTTGGATAGGGTGGCCCTTACGTAAGGATTATATTGCTCCCAATTTTTATGAAATACAAGATGCTCATTGA

>lcl|NC_010433.1_cds_YP_001718441.1_26 [gene=ndhK] [locus_tag=MaesCp025] [db_xref=GeneID:6000072] [protein=NADH dehydrogenase subunit K] [protein_id=YP_001718441.1] [location=complement(52077..52754)] [gbkey=CDS]

ATGAATTCCATTGAGTTTCCTTTACTTGATCGAACAACTCAAATTTCAGTTATTTCAACTACATCAAATGATCTTTCAAATTGGTCAAGACTCTCCAGTTTATGGCCACTTCTCTATGGTACCAGTTGTTGCTTCATTGAATTTGCTTCATTAATAGGCTCACGATTCGACTTTGATCGTTATGGGCTAGTACCAAGATCTAGTCCTAGACAAGCGGACCTGATTTTAACAGCCGGCACAGTAACCATGAAAATGGCTCCTTCTTTAGTAAGATTATATGAACAAATGCCTGAACCAAAATATGTTATTGCTATGGGGGCATGTACAATTACAGGGGGAATGTTCAGTACCGATTCTTATAGTACTGTTCGGGGGGTCGATAAGCTAATTCCTGTAGATGTCTATTTGCCAGGCTGTCCACCTAAACCGGAGGCGGTTATAGATGCTATAACAAAACTTCGTAAAAAAATATCTCGAGAAATTTATGAAGATCGAATTAGGTCTCAACCGGGGAAACGGTGTTTTACTACTAATCACAAGTTTAATATTGAACGCACTACTCATACCGGAAATTATGATCAAGAATTACTCTATCAATCGCCGTCTACTTCAAAGATCCCTCCTGAAACATTTTTCAAATATAAAAGGTCAGTATCGTCTAACGAATTAGTAAATTAG

>lcl|NC_010433.1_cds_YP_001718442.1_27 [gene=ndhC] [locus_tag=MaesCp026] [db_xref=GeneID:6000062] [protein=NADH dehydrogenase subunit 3] [protein_id=YP_001718442.1] [location=complement(52815..53177)] [gbkey=CDS]

ATGTTTCTGATTTACGAATATGATATATTCTGGGCATTTCTAATAATATCAAGTGTTATTCCTATTTTAGCATTTCTAATTTCCGGAGTTTTATCCCCGATTAGCAAAGGGCCGGAGAAACTTTCTAGTTATGAATCGGGTATAGAACCAATAGGCGATGCTTGGTTACAATTTCGAATCCGTTATTATATGTTTGCTCTAGTTTTTGTTGTTTTTGATGTTGAAACAGTTTTTCTTTATCCATGGGCAATGAGTTTCGATGTATTGGGGTTATCCGTATTTATAGAAGCTTTAATTTTCGTGCTTATCCTAATTGTTGGTTCAGTTTATGCATGGAGAAAAGGAGCATTAGAATGGTCTTAG

>lcl|NC_010433.1_cds_YP_001718443.1_28 [gene=atpE] [locus_tag=MaesCp027] [db_xref=GeneID:5999950] [protein=ATP synthase CF1 epsilon subunit] [protein_id=YP_001718443.1] [location=complement(55411..55812)] [gbkey=CDS]

ATGACCTTAAATCTTTGTGTACTGACCCCGAATCGAATTGTTTGGGATTCAGAAGTGAAAGAAATCATTTTATCTACTAATAGTGGACAAATTGGCGTATTACCAAACCATGCACCTATTGCCACAGCTGTCGATATCGGTATTTTGAGAATACGCCTTAATGACCAATGGTTAACGATGGCTCTGATGGGTGGTTTTGCTAGAATAGGCAATAATGAGATTACTGTTTTAGTAAATGATGCAGAGAAGGGTAGTGACATTGATCCACAAGAAGCTCAGCAAACTCTTGAAATAGCAGAAGCTAACTTGAGGAAAGCGGAAGGCAGGAGACAAATAATTGAGGCAAATCTAGCTCTCAGACGAGCTAGGGCACGAGTAGAGGCTCTCAATGAGATTTCGTAA

>lcl|NC_010433.1_cds_YP_001718444.1_29 [gene=atpB] [locus_tag=MaesCp028] [db_xref=GeneID:5999989] [protein=ATP synthase CF1 beta subunit] [protein_id=YP_001718444.1] [location=complement(55809..57311)] [gbkey=CDS]

ATGAGAATCAATCCTACTACTTCTACTTCTGGTCCGGGAGTTTCCGCGCTTGAAAAAAAGAACCTGGGGCGTATCGCTCAAATCATCGGGCCAGTGCTAGATGTAGCTTTTCCCCCGGGCAAGATGCCTAATATTTACAACGCTCTGGTAGTTAAGGGTCGAGATACTGCCGGTCAAGAAATTAATGTGACTTGTGAAGTACAACAATTATTAGGAAATAATCGAGTTCGGGCTGTAGCTATGAGTGCTACAGATGGTCTAACGAGAGGAATGGAAGTGATTGACACAGGAGCCCCTCTAAGTGTTCCAGTCGGTGGGGCAACTCTAGGACGAATTTTCAACGTGCTTGGAGAACCTGTTGACGATTTAGGTCCTGTAGATACTCGCGCAACATCCCCTATTCATAGATCTGCACCTGCCTTTATACAGTTAGATACAAAATTATCTATTTTTGAAACAGGAATTAAAGTAGTAGATCTTTTAGCCCCTTATCGCCGTGGAGGAAAAATCGGGCTATTCGGGGGAGCTGGAGTGGGTAAAACAGTACTTATTATGGAATTAATCAATAACATTGCGAAAGCTCATGGGGGTGTATCCGTATTTGGCGGAGTAGGCGAACGTACTCGTGAAGGAAATGATCTTTACATGGAAATGAAAGAATCTGGAGTAATTAATGAAGAAAATATTGCAGAATCAAAAGTGGCTCTAGTCTATGGTCAGATGAACGAACCGCCGGGAGCTCGTATGAGAGTTGGTTTGACTGCCCTAACTATGGCGGAATATTTTCGAGATGTTAATGAACAAGACGTACTTCTATTTATCGACAATATCTTCCGTTTCGTCCAAGCAGGATCCGAAGTATCCGCCTTATTGGGTAGAATGCCTTCCGCTGTGGGTTATCAACCTACCCTTAGTACCGAAATGGGCTCTTTACAAGAAAGAATTACTTCTACCAAAGAAGGGTCCATAACTTCTATTCAAGCAGTTTATGTACCTGCGGACGATTTGACTGATCCTGCTCCTGCCACGACATTTGCACATTTAGATGCTACTACTGTACTATCAAGAGGATTAGCTGCTAAAGGTATCTATCCAGCAGTAGATCCTTTAGATTCAACGTCAACTATGCTCCAACCTCAGATTGTTGGTGAGGAACATTATGAAACTGCGCAAAGAGTTAAGCAAACTTTACAACGTTACAAAGAACTTCAGGACATTATAGCTATCCTTGGGTTGGACGAATTATCCGAAGAGGATCGCTTAACTGTAGCAAGAGCACGAAAAATTGAGCGTTTCTTATCACAACCCTTTTTCGTAGCAGAAGTATTTACCGGTTCTCCGGGGAAATATGTCGGTCTAGCAGAAACAATTAGAGGGTTTAAATTGATCCTTTCGGGAGAATTAGATAGTCTCCCTGAGCAGGCCTTTTATTTGGTAGGTAATATTGATGAAGCTACTGCGAAGGCTACGAACTTAGAAATGGAGAACAACTTGAAGAAATGA

>lcl|NC_010433.1_cds_YP_001718445.1_30 [gene=rbcL] [locus_tag=MaesCp029] [db_xref=GeneID:5999955] [protein=ribulose-1,5-bisphosphate carboxylase/oxygenase large subunit] [protein_id=YP_001718445.1] [location=58063..59496] [gbkey=CDS]

ATGTCACCACAAACAGAGACTAAAGCAAGTGTTGGATTCAAGGCTGGTGTTAAAGATTATAAATTGACTTATTATACTCCTGACTATCAAACCAAAGATACTGATATCTTGGCAGCATTCCGAGTAACTCCTCAACCTGGAGTTCCGCCTGAGGAAGCAGGAGCTGCGGTAGCTGCTGAATCTTCTACTGGTACATGGACAACTGTGTGGACCGATGGACTTACCAGTCTTGATCGTTATAAAGGACGATGCTACGGCCTCGAGCCCGTTCCTGGAGAAGAAAATCAATATATTGCTTATGTAGCTTACCCATTAGACCTTTTTGAAGAAGGTTCTGTTACTAACATGTTTACTTCTATTGTGGGTAATGTATTTGGGTTCAAAGCCCTACGCGCCCTACGTCTGGAGGATTTGCGAGTCCCTCCTGCTTATTCTAAAACTTTCCAAGGGCCTCCTCATGGCATCCAAGTTGAGAGAGATAAATTGAACAAGTATGGTCGCCCCCTATTAGGTTGTACTATTAAACCAAAATTGGGGCTATCCGCTAAGAATTACGGTAGAGCAGTTTATGAATGTCTTCGCGGTGGACTTGATTTTACCAAAGACGATGAGAATGTGAACTCCCAACCATTTATGCGTTGGAGAGACCGTTTCTTATTTTGTGCCGAAGCAATTTATAAAGCACAGGCTGAAACAGGTGAAATCAAAGGGCATTATTTGAATGCTACTGCAGGTACATGCGAAGAAATGATCAAAAGGGCTGTATGTGCCAGAGAATTAGGAGTTCCTATCGTAATGCATGACTACTTAACAGGGGGATTCACTGCAAATACTAGCTTGGCTCACTATTGCCGAGATAATGGTTTACTTCTTCACATTCACCGCGCAATGCATGCAGTTATTGATAGACAGAAGAATCATGGTATGCATTTTCGTGTACTAGCTAAGGCTTTACGTCTGTCTGGTGGAGATCATATTCACGCTGGTACCGTAGTAGGTAAACTTGAAGGGGAAAGAGACATTACTTTGGGCTTTGTTGATTTACTGCGTGATGATTTTATTGAAAAAGATCGAAGCCGCGGTATTTATTTCACTCAAGATTGGGTCTCTCTACCAGGTGTTCTGCCTGTAGCTTCAGGGGGTATTCACGTTTGGCATATGCCTGCTCTGACCGAGATCTTTGGAGATGATTCCGTACTACAATTCGGTGGAGGAACTTTAGGGCACCCTTGGGGAAATGCACCCGGTGCCGTAGCTAATCGAGTAGCTCTAGAAGCATGTGTACAAGCTCGTAATGAGGGACGTGATCTTGCTCGTGAGGGTAATGATATTATCCGTGAGGCTAGCAAATGGAGTCCTGAACTAGCTGCTGCTTGTGAAGTATGGAAGGAAATTAAATTTGAATTCGCAGCAGTGGATACTTTGGATAAATAA

>lcl|NC_010433.1_cds_YP_001718446.1_31 [gene=accD] [locus_tag=MaesCp030] [db_xref=GeneID:6000068] [protein=acetyl-CoA carboxylase beta subunit] [protein_id=YP_001718446.1] [location=60304..62031] [gbkey=CDS]

ATGGAAAAACGGTGGTTCAATTCGATCTTATCCAATGTGGAATTAGGATACAGGTGTAGGCTAAGTAAATCAATGGATAGTTTCAGTTCTCTTGAAAATACCAGTATAAGCGAAGACCCAATTCTAAATGATACAGATAAAAACACCCATAGTTGGAGTAATAGTAATAGTAATAGTGACAGCTCTAGTTATAGTAATGTTGATCATTTAGTCGGCGTCAGGGACATTCAGAATTTCGGCGCCGATGAAACTTATTTAGTTAGGGATAGTAATAAGGACAGTTATTCCATATATTTTGATATTGAAAATAAAGTTTTTGAGATTGACAATGATCATTCTTTTCTGAGTGAACTAAAAAGTTCTTTTTATAGTTATTGGAATTCTAGTTATCTGAATAATGGGTCTAGGAGTGGCGACTCCCACTCTGATCATTATATGTATGATACTAAATATAGTTGGAATAATTACATCAATAGTTGCATTGACAGTTATCTTCGCTCTCAAATCTGTATTGATAGTTATATTTTAAGTGGTAGTAACAATTACAGTGAAAGTTACATTTATAGTTACTACATTTATAGTTACTTTTGTGGTGAAAGTGGAAATAGTAGTGAAAGCGAGAGTTCCAGTCTAAGAACTAGCACGAGTGGTAGCGATTTAATTATAAGAGAAAGTTCTAATGATGAAAGTTCTAATGATAATGATGAAAGTTATAATGATAATGATGAAAGTTATAATGATAATGATGAAAGTTATAATGATGATGATGAAAGTTATAATGATGAAAGTTCTAATGATAATGATGAAAGTTATAATGATAATGATGAAAGTTATAATGATGATGATGAAAGTTATAATGATGAAAGTTCTAATGATAATGATGAAAGTTCTAATGATCTCGATATAACTCAAAAATACAAGCATTTGTGGGTTCAATGCGAAAATTGTTATGGATTAAATTATAAGAAATTTTTTAAGTCAAGAATGAATATTTGTGAACAATGTGGATATCATTTGAAAATGAGTAGTTCAGATAGAATTGAACTTTTGATTGACCTAGACACTTGGGATCCTATGGATGAAGACATGGTATCTCTGGATCCCATTGAATTTCATTCAGAAGAGGAACCTTATAAAGATCGTATTGATTCTTATCAAAGAAAGACAGGATTAAGTGAGGCTGTTCAAACAGGCACAGGTCAACTAAACGGCATTCCCGTAGCAATTGGGGTTATGGATTTTCGGTTTATGGGGGGTAGTATGGGATCCGTAGTAGGTGAGAAAATCACTCGTTTGATCGAGTATGCTACCAATAAATTTTTACCTCTTATTTTAGTGTGTGCTTCCGGAGGAGCACGCATGCAAGAAGGAAGTTTGAGCTTGATGCAAATGGCTAAAATATCTTCTGCATTATATGATTATCAATCGAATAAAAAGTTATTTTATGTATCAATCCTTACATCTCCTACGACTGGTGGGGTGACAGCTAGTTTTGGTATGTTGGGGGATATCATTATTGCTGAACCTAATGCCTATATTGCGTTTGCAGGTAAAAGAGTAATTGAACAAACATTGAATAAGACAGTACCTGAAGGTTCGCAATCGGCCGAATTTTTATTCCATAAAGGCTTATTTGATCTAATCGTACCGCGTAATCTTTTAAAGGGCGTTCTGAATGAGTTACTTCAGTTCCACGATTTCTTTCCTTTGAATCATAAATCAAAGTAG

>lcl|NC_010433.1_cds_YP_001718447.1_32 [gene=psaI] [locus_tag=MaesCp031] [db_xref=GeneID:5999994] [protein=photosystem I subunit VIII] [protein_id=YP_001718447.1] [location=62810..62923] [gbkey=CDS]

ATGACAATTCTCAACAACTTACCCTCCATTTTTGTGCCTTTAGTGGGCTTAGTATTTCCGGCAATTGCAATGGCTTCTTTATTTCTTTATGTTCAAAAAAACAAGATTTTTTAG

>lcl|NC_010433.1_cds_YP_001718448.1_33 [gene=ycf4] [locus_tag=MaesCp032] [db_xref=GeneID:6000057] [protein=photosystem I assembly protein Ycf4] [protein_id=YP_001718448.1] [location=63384..63938] [gbkey=CDS]

ATGAGTTGGCGATCAGAACGTATATGGATAGAACTTATAGCGGGGTCTCGAAAAACAAGTAATTTCTGCTGGGCCTTTATACTTTTTTTAGGTTCATTGGGATTTTTATTGGTTGGAATTTCCAGCTATCTTGGCAGAAATTTGATATCTTTATTTCCGTCTCAGCAAATAATTTTTTTCCCACAAGGGATCGTGATGTCTTTCTATGGGATCGCCGGTCTATTTATTAGTTCTTATTTGTGGTGCACAATTTTATGGAATATAGGTAGTGGTTATGATCGATTCGATAGAAAAGAAGGAATAGTGTGTATTTTTCGCTGGGGATTTCCTGGAAAAAATCGTCGCATCTTACTACGATTCCTTATGAAAGATATTCAGTCTATTAGAATAGAAGTTAAAGAGCGTATTTATGCTCGGCGTGTCCTTTATATGGAAATCAGAGGCCGGGGGGCTATTCCTTTGACTGGTACTGATGAGAATTTGACTCCACGAGAAATTGAGCAAAAAGTAGCGGAATTGGCCTATTTTTTGCGTGTACCAATTGAAGTATTTTGA

>lcl|NC_010433.1_cds_YP_001718449.1_34 [gene=cemA] [locus_tag=MaesCp033] [db_xref=GeneID:6000061] [protein=envelope membrane protein] [protein_id=YP_001718449.1] [location=64854..65549] [gbkey=CDS]

ATGAAAAAATGGAAAAAAAAAACATTTATTCCCCTTCTATATCTTACATCTATAGTTTTTTTGCCCTGGTGGGTCTCTTTTTTATTTAATAAAAGTTTGGAATCTTGGATTATTAATTGTTGTAATACTAGTAAATCCGAAACTTTTTTAAATGATATCCAAGAAAAAAGTATTCTAGAAAAATTCATGGAATTAGAAGACCTCGTTCGCTTAAACGAAATAATAAAGGAATACCCGGAAACACATCTACAAAAGTTTCGTATCGGAATCCACAAAGAAACGATCCAATTGATCAAGATGCACAATGAGGATCGTATCCATACGATTTTGCACTTCTCGACAAATATAATCTGTTTCATTATTCTAAGTGGTTATTCTATTCTAAGTAATGAAGAACTTATTATTCTTAATTCTTGGGTTCAAGAATTCCTATATAACTTAAGCGACACAATAAAAGCTTTTTCCATTCTTTTATTAACCGATTTATGTATAGGATTCCATTCACCTCACGGTTGGGAACTAATGATCGGCTCTGTCTACAAAGATTTTGGATTTGCCCATAACGATCAAATTATATCTGGCCTTGTTTCTACTTTTCCAGTCATTCTCGATACCATTTTTAAATATTGGATTTTCCGTTATTTAAATCGCGTATCTCCGTCACTTGTAGTGATTTATCATTCAATGAATGACTGA

>lcl|NC_010433.1_cds_YP_001718450.1_35 [gene=petA] [locus_tag=MaesCp034] [db_xref=GeneID:5999988] [protein=cytochrome f] [protein_id=YP_001718450.1] [location=65788..66750] [gbkey=CDS]

ATGCAAACTAGAAAGACCTTTTCTTGGATAAAAGAAGAGATTACTCGTTCAATTTCCGTATTGCTCATGATATATATAATAACTTGGGCATCCATTTCAAATGCATATCCCATTTTTGCACAGCAGGGTTATGAAAATCCACGCGAAGCAACTGGTCGTATTGTATGTGCCAATTGTCATTTAGCTAATAAACCCGTGGATATTGAGGTTCCACAAGCGGTACTTCCAGATACTGTATTTGAAGCAGTTGTTCGAATTCCTTATGATATGCAACTGAAACAAGTTCTTGCTAATGGTAAAAAGGGGGCTTTGAACGTGGGGGCTGTTCTTATTTTACCTGAGGGGTTTGAATTAGCCCCTCCCGATCGTATTTCGCCAGAGATGAAAGAAAAGATGGGAAATCTGTCTTTTCAGAGTTATCGCCCCACTAAAAAAAATATTCTTGTGATAGGTCCTGTTCCTGGTCAGAAATATAGTGAAATTACCTTTCCTATTCTTTCTCCGGACCCCGCCGCTAAGAAAGATGTTCACTTTTTAAAATATCCCATATATGTAGGCGGAAACAGGGGAAGGGGTCAGATTTATCCCGACGGGAGCAAGAGCAACAATACGGTTTATAATGCTACAGCAGCAGGTATAGTAAGCAAAATCATACGAAAAGAAAAAGGGGGGTACGAAATAACCATAACGGATGCGTCAGAGGGACGTCAAGTGATTGATATTATACCTCCAGGACCAGAACTTCTTGTTTCAGAAGGCGAATCCATTAAACTTGATCAACCATTAACGAGTAATCCTAATGTGGGTGGATTTGGTCAGGGGGATGCAGAAATAGTACTTCAAGACCCATTACGCGTCCAAGGCCTTTTGTTCTTCTTGGCATCCGTTATTTTGGCACAAATCTTTTTGGTTCTTAAAAAGAAACAGTTTGAGAAGGTTCAATTGTCCGAAATGAATTTCTAG

>lcl|NC_010433.1_cds_YP_001718451.1_36 [gene=psbJ] [locus_tag=MaesCp035] [db_xref=GeneID:5999978] [protein=photosystem II protein J] [protein_id=YP_001718451.1] [location=complement(67882..68004)] [gbkey=CDS]

ATGGCCGATACTACTGGAAGAATTCCTCTTTGGATAGTAGGTACTGTAACTGGTATTCTTGTGATCGGTTTAATAGGCATTTTCTTTTATGGTTCATATTCCGGATTGGGTTCATCCCTGTAA

>lcl|NC_010433.1_cds_YP_001718452.1_37 [gene=psbL] [locus_tag=MaesCp036] [db_xref=GeneID:6000067] [protein=photosystem II protein L] [protein_id=YP_001718452.1] [location=complement(68156..68272)] [gbkey=CDS]

ATGACACAATCAAATCCGAACGAACAAAATGTTGAATTGAATCGTACCAGTCTCTACTGGGGGTTATTACTCATTTTTGTACTTGCTGTTTTATTTTCTAATTATTTCTTCAATTGA

>lcl|NC_010433.1_cds_YP_001718453.1_38 [gene=psbF] [locus_tag=MaesCp037] [db_xref=GeneID:5999975] [protein=photosystem II protein VI] [protein_id=YP_001718453.1] [location=complement(68299..68418)] [gbkey=CDS]

ATGACCATAGATCGAACCTATCCAATTTTTACAGTACGATGGTTGGCTGTTCACGGACTAGCTGTACCTACCGTTTCTTTTTTGGGGTCAATATCAGCAATGCAGTTCATCCAACGATAA

>lcl|NC_010433.1_cds_YP_001718454.1_39 [gene=psbE] [locus_tag=MaesCp038] [db_xref=GeneID:5999958] [protein=photosystem II protein V] [protein_id=YP_001718454.1] [location=complement(68427..68678)] [gbkey=CDS]

ATGTCTGGAAGCACAGGAGAACGTTCTTTTGCTGATATTATTACCAGTATTCGATATTGGGTCATTCATAGCATTACTATACCTTCCCTATTCATTGCAGGTTGGTTATTCGTCAGTACGGGTTTAGCTTACGATGTATTTGGAAGTCCTCGTCCAAATGAATATTTTACCGAAAGCCGACAAGGAATTCCATTAATAACTGGCCGTTTTGATCCTTTGGAACAACTCGATGAATTTAGTAAATCTTTTTAG

>lcl|NC_010433.1_cds_YP_001718455.1_40 [gene=petL] [locus_tag=MaesCp039] [db_xref=GeneID:6000071] [protein=cytochrome b6/f complex subunit VI] [protein_id=YP_001718455.1] [location=70200..70295] [gbkey=CDS]

ATGCCTACTATAACTAGTTATTTCGGTTTTCTACTAGCGGCTTTAACTATAACCTCAGTTCTATTTATTGGTCTGAGCAAGATACGACTTATTTGA

>lcl|NC_010433.1_cds_YP_001718456.1_41 [gene=petG] [locus_tag=MaesCp040] [db_xref=GeneID:6000019] [protein=cytochrome b6/f complex subunit V] [protein_id=YP_001718456.1] [location=70487..70600] [gbkey=CDS]

ATGATTGAAGTTTTTCTATTTGGAATCGTCTTAGGTCTAATTCCTATTACTTTGGCCGGATTATTCGTAACTGCATATTTACAATACAGACGTGGTGATCAGTTGGACCTTTGA

>lcl|NC_010433.1_cds_YP_001718457.1_42 [gene=psaJ] [locus_tag=MaesCp041] [db_xref=GeneID:6000014] [protein=photosystem I subunit IX] [protein_id=YP_001718457.1] [location=71329..71463] [gbkey=CDS]

ATGCGAGATCTAAAAACATATTTATCTGTAGCACCGGTAATAAGTACTCTATGGTTTGGGTCTTTAGCAGGTCTATTGATAGAGATCAATCGTTTTTTCCCAGATGCGTTGACATTCCCTTTTTTTTCATTCTAG

>lcl|NC_010433.1_cds_YP_001718458.1_43 [gene=rpl33] [locus_tag=MaesCp042] [db_xref=GeneID:6000070] [protein=ribosomal protein L33] [protein_id=YP_001718458.1] [location=71958..72158] [gbkey=CDS]

ATGGCCAAGGGTAAGGATATCCGAATAAGGGTTATTTTAGAATGTACCACTTGTACTCGAAACAGTGTTAATAAGAAATCAACAGGCATTTCTAGATATATTACTCAAAAGAATCGACACAATACGCCTAGTCGATTGGAATTGAGAAAATTCTGTCGCTATTGTTACAAACATACAATTCACGGGGAGATAAAGAAATAG

>lcl|NC_010433.1_cds_YP_001718459.1_44 [gene=rps18] [locus_tag=MaesCp043] [db_xref=GeneID:6000065] [protein=ribosomal protein S18] [protein_id=YP_001718459.1] [location=72579..72863] [gbkey=CDS]

ATGGATAAATCCAAACGACTTTTTCTTAAGTCCAAGCGATCTTTTCGTAGGCGTTTGCCCCCGATCCAATCGGGGGATCGAATTGATTATAGAAACATGAGTTTAATTAGTCGATTTATTAGTGAACAAGGAAAAATATTATCTAGACGGGTGAATAGATTGAGTTTAAAACAACAACGATTAATTACTATTGCTATAAAACAAGCTCGTATTTTATCTTCGTTACCTTTTCTTAATAATGAAAAACAATTTGAAAAAAAGCGAGTTGGTCGCTATAACTACTGA

>lcl|NC_010433.1_cds_YP_001718460.1_45 [gene=rpl20] [locus_tag=MaesCp044] [db_xref=GeneID:6000024] [protein=ribosomal protein L20] [protein_id=YP_001718460.1] [location=complement(73205..73558)] [gbkey=CDS]

ATGACCAGAATTAGACGAGGATATATAGCTCGAAGGCGTAGAACAAAAATTCGTCTATTCGCATCAAGCTTTCGCGGGGCCCATTCAAGACTTACTCGAACTATTATTCAACAAAAAATAAGAGCTTTGGTTTCGGCCCATCGGGATAGAGATAGGCAAAAAAGAAATTTTCGTCGTTTGTGGGTTACTCGGATAAATGCAGTAATTCGCGAGAGCACGGTATCCTATAGTTATAGTAGATTAATAAACAATCTGTACAAGAGACAGTTACTTCTTAATCGTAAAATACTTGCACAAATAGCTATATTAAATAGGAATTGTCTTTATATGATTTCCAATGACATTCTAAAATAA

>lcl|NC_010433.1_cds_YP_001718461.1_46 [gene=rps12] [locus_tag=MaesCp045] [db_xref=GeneID:5999968] [protein=ribosomal protein S12] [exception=trans-splicing] [protein_id=YP_001718461.1] [location=join(complement(74362..74475),146526..146757,147294..147319)] [gbkey=CDS]

ATGCCAACTATTAAACAACTTATTAGAAACACAAGACAGCCAATCAGAAATGTCACCAAATCCCCCGCTCTTGGGGGATGTCCTCAGCGCCGAGGAACATGTACTAGGGTGTATACTATCACCCCCAAAAAACCAAACTCTGCCTTACGTAAAGTTGCCAGAGTACGATTAACCTCTGGATTTGAAATCACTGCTTATATACCTGGTATTGGCCATAATTTACAAGAACATTCTGTAGTCTTAGTAAGAGGGGGAAGGGTTAAGGATTTACCCGGTGTGAGATATCACATTGTTCGAGGAACCCTAGATGCTGTCGGAGTAAAGGATCGTCAACAAGGGCGTTCTAAATATGGGGTCAAAAAGCCAAAATAA

>lcl|NC_010433.1_cds_YP_001718462.1_47 [gene=clpP] [locus_tag=MaesCp047] [db_xref=GeneID:6000066] [protein=ATP-dependent Clp protease proteolytic subunit] [protein_id=YP_001718462.1] [location=complement(join(74681..74908,75603..75893,76735..76803))] [gbkey=CDS]

ATGCCTATTGGTGTTCCAAAAGTCCCTTTTCGAAATCCTGGGGAAGACGATTCAACTTGGATTGACATAAACCGACTTTATCGAGAAAGATTACTTTTTTTAGGTCAAGATGTTGATAGCGAGATCGCGAATCAACTTATTGGTCTTATGGTATATCTCAGTATAGAAAACGCGACAAAAGATTTGTATTTGTTTATAAACTCTCCCGGCGGATGGGTAATACCCGGAATAGCTATTTATGATACTATGCAATTTGTGCGACCCGATGTACAAACAGTATGCATGGGATTAGCTGCTTCAATGGGATCTTTTATTCTGGTCGGAGGAAAAATTACCAAACGTTTAGCATTCCCTCATGCTAGGGTAATGATTCATCAACCTATTTCTGGTTATTATGAGGCACAAATAGTAGAATTTGTCCTGGAAGCGGAAGAACTACTGAAACTGCGCGAAATCCTCACAAGGATTTATGCACAAAGAACGGGAAAACCTTTATGGGTTGTATCCGAAGACATGGAAAGAGATGTTTTTATGTCAGCAACCGAAGCCCAAGCTCATGGAATTGTTGATCTTGTAGCAGTTGCATAA

>lcl|NC_010433.1_cds_YP_001718463.1_48 [gene=psbB] [locus_tag=MaesCp048] [db_xref=GeneID:5999952] [protein=photosystem II 47 kDa protein] [protein_id=YP_001718463.1] [location=77269..78795] [gbkey=CDS]

ATGGGTTTGCCTTGGTATCGTGTTCATACCGTCGTATTGAATGATCCCGGTCGTTTGCTGTCTGTCCATATAATGCATACAGCTTTGGTTGCTGGTTGGGCCGGTTCGATGGCTCTATATGAATTAGCAGTTTTTGATCCCTCTGACCCCGTTCTCGATCCAATGTGGAGACAGGGTATGTTCGTTATACCTTTTATGACTCGTTTAGGAATAACCAATTCATGGGGTGGTTGGAGTATCACAGGAGGAACTATAACGAATCCGGGTATTTGGAGTTATGAAGGCGTGGCTGGGGCGCATATTGTGTTTTCTGGCTTGTGCTTCTTGGCAGCTATTTGGCATTGGGTGTATTGGGATCTAGAAATATTTTGCGATGAACGTACAGGAAAACCTTCTTTGGATTTGCCCAAGATCTTTGGAATTCATTTATTTCTCTCCGGGGTGGCTTGCTTTGGGTTTGGCGCTTTTCATGTAACCGGATTGTATGGTCCTGGAATATGGGTGTCCGACCCTTATGGACTAACCGGAAAGGTACAACCTGTAAGTCCAGCATGGGGTGTGGAAGGTTTTGATCCTTTTGTTCCGGGAGGAATAGCTTCTCATCATATTGCAGCGGGGACATTGGGCATATTAGCGGGCCTATTCCATCTTAGTGTCCGCCCGCCCCAACGTTTATACAAAGGATTACGTATGGGAAATATTGAAACTGTCCTTTCCAGTAGTATCGCGGCTGTCTTTTTTGCAGCTTTTGTTGTTGCTGGAACTATGTGGTATGGTTCAGCAACTACCCCGATTGAATTATTTGGTCCCACTCGTTATCAATGGGATCAAGGATACTTCCAGCAAGAAATATATCGAAGAGTTAGTGCTGGGCTAGCCGAAAATCAAAGTTTATCCGAAGCTTGGTCTAAAATTCCCGAAAAATTAGCTTTTTATGATTACATCGGCAATAATCCGGCAAAAGGTGGGTTGTTCAGAGCAGGCTCAATGGACAACGGGGATGGAATAGCTGTTGGGTGGTTAGGACATCCTATCTTTAGAGATAAAGAAGGGCGTGAACTTTTTGTACGTCGTATGCCTACTTTTTTTGAAACATTTCCGGTTGTTTTGGTAGACGGAGATGGAATTGTTAGAGCCGATGTTCCTTTTCGAAGGGCGGAGTCGAAGTATAGTGTCGAACAAGTAGGTGTAACTGTTGAGTTCTATGGTGGCGAACTAAACGGAGTCAGTTATAGTGATCCTGTTACTGTGAAAAAATATGCTAGACGCGCTCAATTGGGTGAAATTTTTGAATTAGATCGTGCTACTTTGAAATCCGATGGTGTTTTTCGTAGCAGCCCAAGGGGTTGGTTTACTTTTGGACATGCTTCGTTCGCTCTGCTCTTTTTCTTCGGACACATTTGGCATGGTGCTCGAACTTTGTTCAGAGATGTTTTTGCTGGTATTGATCCAGATTTAGATGCTCAAGTGGAATTTGGAGCATTCCAAAAACTTGGAGATCCGACTACAAGAAGACAAGTAGTCTGA

>lcl|NC_010433.1_cds_YP_001718464.1_49 [gene=psbT] [locus_tag=MaesCp049] [db_xref=GeneID:6000020] [protein=photosystem II protein T] [protein_id=YP_001718464.1] [location=78967..79074] [gbkey=CDS]

ATGGAAGCATTGGTTTATACATTCCTTTTAGTATCAACTTTAGGAATCATTTTTTTCGCTATCTTTTTTCGAGAACCGCCTAAAGTTCCAACTAAAAAGGTAAAATGA

>lcl|NC_010433.1_cds_YP_001718465.1_50 [gene=psbN] [locus_tag=MaesCp050] [db_xref=GeneID:6000069] [protein=photosystem II protein N] [protein_id=YP_001718465.1] [location=complement(79134..79265)] [gbkey=CDS]

ATGGAAACAGCAACCCTAGTCGCCATCTCTATATCTGGTTTACTTGTAAGTTTTACTGGGTATGCCTTATATACTGCTTTTGGGCAACCCTCTCAACAACTAAGAGATCCATTCGAGGAACACGGGGACTAG

>lcl|NC_010433.1_cds_YP_001718466.1_51 [gene=psbH] [locus_tag=MaesCp051] [db_xref=GeneID:5999987] [protein=photosystem II protein H] [protein_id=YP_001718466.1] [location=79369..79590] [gbkey=CDS]

ATGGCTACACAAAGCGTGGAGGGTAGTTCTAGATCTGGTCCAAGACGAACTATTGTAGGGGATTTATTGAAACCGTTGAATTCGGAATACGGTAAAGTAGCTCCCGGATGGGGAACTACTCCTTTGATGGGTGTCGCAATGGCCCTATTTGCGATATTCCTATCTATTATTTTGGAGATTTATAATTCTTCCGTTTTACTAGATGGAATTTCAATGAATTAG

>lcl|NC_010433.1_cds_YP_001718467.1_52 [gene=petB] [locus_tag=MaesCp052] [db_xref=GeneID:5999969] [protein=cytochrome b6] [protein_id=YP_001718467.1] [location=join(79716..79721,80491..81132)] [gbkey=CDS]

ATGAGTAAAGTCTATGATTGGTTCGAAGAACGTCTCGAGATTCAGGCAATTGCAGATGATATAACTAGTAAATATGTTCCTCCCCATGTCAACATATTTTATTGTTTAGGGGGAATTACGCTTACTTGTTTTTTAGTACAAGTAGCTACGGGGTTTGCTATGACTTTTTACTACCGTCCGACGGTTACTGAGGCTTTTGCTTCTGTTCAATACATAATGACTGAAGCTAACTTTGGTTGGTTAATCCGCTCAGTTCATCGATGGTCGGCAAGTATGATGGTTTTAATGATGATCCTGCACGTATTTCGTGTGTATCTCACTGGTGGCTTTAAAAAACCTCGTGAATTGACTTGGGTTACGGGCGTGGTTCTTGCTGTATTGACCGCATCTTTTGGTGTAACTGGTTATTCCTTACCTTGGGACCAAATTGGTTATTGGGCGGTAAAAATTGTAACAGGCGTGCCGGAAGCTATTCCTGTAATAGGATCACCTTTGGTAGAGTTATTACGCGGAAGTGCTAGTGTAGGACAATCCACTTTGACTCGTTTTTATAGTTTACACACTTTTGTATTACCTCTTCTTACTGCCGTATTTATGTTAATGCACTTTCCAATGATACGTAAGCAAGGTATTTCAGGCCCTTTATAG

>lcl|NC_010433.1_cds_YP_001718468.1_53 [gene=petD] [locus_tag=MaesCp053] [db_xref=GeneID:5999970] [protein=cytochrome b6/f complex subunit IV] [protein_id=YP_001718468.1] [location=join(81340..81347,82177..82672)] [gbkey=CDS]

ATGGGAGTAACAAAAAAACCTGACTTGAATGATCCTGTATTAAGAGCTAAATTGGCTAAGGGAATGGGTCATAATTATTACGGAGAACCTGCATGGCCCAATGATCTTTTATATATTTTTCCAGTAGTAATTCTAGGTACTATTGCATGTAATGTAGGATTAGCGGTTCTAGAACCATCAATGATTGGTGAACCTGCGGATCCATTTGCAACGCCTTTGGAAATATTGCCTGAATGGTATTTCTTTCCCGTATTTCAAATACTTCGTACAGTACCCAATAAGTTATTAGGTGTTCTTTTAATGGTTTCAGTACCTGCAGGATTATTAACAGTACCTTTTTTGGAGAATGTTAATAAATTCCAAAATCCATTTCGTCGTCCAGTTGCGACAACCGTCTTTTTGATTGGTACTGCGGTAGCCCTTTGGTTAGGTATTGGAGCAACATTACCTATTGATAAATCCCTAACTTTAGGTCTTTTTCAAATTGATTCAATTGTAAAATAA

>lcl|NC_010433.1_cds_YP_001718469.1_54 [gene=rpoA] [locus_tag=MaesCp054] [db_xref=GeneID:6000056] [protein=RNA polymerase alpha subunit] [protein_id=YP_001718469.1] [location=complement(82862..83893)] [gbkey=CDS]

ATGGTTCGAGAGAAAATAACAATATCCACTCGGACACTGCAGTGGAAATGTATTGAATCAAGAACCGATAATAAACGTCTTTATTATGGACGCTTTATTCTGTCTCCACTTATGAAAGGACAAGCCGACACAATAGGCATTGCGATGCGAAGAGCTTTGCTTGGAGAAATAGAAGGAACATGTATCACACGTGCAAAATCTGAGAAAATACCACACGAATTTTCTACTATAGCAGGTATTCAAGAATCAATACATGAAATTTTAATGAATTTGAAAGAAATTGTATTAAGAAGCAATTTGTATGGAACTTGTGACGCATCCATTTGTGTCAAGGGTCCTGGATATGTAACTGCTCAAGACATCATCTTACCGCCTTTTGTGGAAATCATTGATAATACACAGCATATCGCTAGCCTAACGAAAGCAATTGATTTGTGTATTGGATTACAAATCGAGAGGAATCGTGGCTATCGTATAAAACCAACAAATAACTTTCAAGTTCAAGACGGGAGTTATTCTATAGATGCTGTATTCATGCCTGTTCGAAATGCGAATCATAGTGTTCATTCTTATGGAAATGGGAATGAAAAGCAAGAGATACTTTTTCTCGAAATATGGACAAATGGAAGTTTAACTCCTAAAGAAGCACTTCACGAAGCCTCCCGTAATTTGATTGATTTTTTTATTCCTTTTCTACATGCAGAAGAAGAAAACTTACATTTAGAAATAGAAAAAAATCAACACAAGGTTACTTTACCCCTTTTTTGTTTTCATGATAAATTGACTAAATTAAGAAAAAATAAAAAAGAAATAGTATTGAAATACATTTTTATTGACCAATCAGAATTGACTCCTAAGATCTATAATTGCCTCAAAAGATCCAATATACATACATTATCGGATCTTTTGAATAAGAGTCAAGAAGATCTTATGAAAATTGAACATTTTCGCATAGACGATGTAAAACATATATTGGGTATTCTAGAAATAGAAAAACATTTCACAATTGATTTACCAAAGAATAAAATATAA

>lcl|NC_010433.1_cds_YP_001718470.1_55 [gene=rps11] [locus_tag=MaesCp055] [db_xref=GeneID:6000046] [protein=ribosomal protein S11] [protein_id=YP_001718470.1] [location=complement(83952..84368)] [gbkey=CDS]

ATGGCAAAACCTTTACCAAAAATTAGTTCACGCAGAAATGGACGTATTGGTTCACGTAAGAATTCACGTAAAATACCAAAAGGCGTTATTCATGTTCAAGCAAGTTTCAACAATACTATTGTGACCATTACAGATGTACGGGGTCGAGTGATTTCTTGGTCCTCCGCTGGCACTTGTGGATTCAGGGGCACAAGAAGAGGAACGCCATTTGCTGCTCAAACCGCAGCAGGAAATGCTATTCGGACAGTAGTGGATCAAGGTATGCAACGAGCAGAAGTCATGATAAAGGGTCCTGGTCTCGGACGAGATGCGGCATTAAGAGCTATTCGCAGAAGTGGTATACTATTAAGTTTCGTCCGGGATGTAACCCCTATGCCACATAATGGCTGCCGACCCCCTAAAAAAAGGCGCGTGTAA

>lcl|NC_010433.1_cds_YP_001718471.1_56 [gene=rpl36] [locus_tag=MaesCp056] [db_xref=GeneID:6000043] [protein=ribosomal protein L36] [protein_id=YP_001718471.1] [location=complement(84490..84603)] [gbkey=CDS]

ATGAAAATAAGAGCTTCTGTTCGTAAAATTTGTGAAAAATGTCGACTGATACGTAGACGGGGGCGAATTATAGTAATTTGCTTCAACCCGAGACATAAACAAAGACAAGGATAA

>lcl|NC_010433.1_cds_YP_001718472.1_57 [gene=rps8] [locus_tag=MaesCp057] [db_xref=GeneID:5999992] [protein=ribosomal protein S8] [protein_id=YP_001718472.1] [location=complement(85138..85542)] [gbkey=CDS]

ATGGGTAGGGATCCTATTGCTGACATAATAACCTCTATAAGAAATGCTGACATAAATAGAAAAGGAACCGTTCGAATAGCATCTACTAACATCACTGAAAACATTATTAAAATACTTTTAAGAGAAGGTTTTATTGAAAATGTCAGGAAACATCAGGAGGGCAACAAAAAATTTTTGGTTTTAACCCTACGACATAGAAGGAAGAGGAAAGGACCCTATAGAACTAGTCTAAATTTAAAACGGATCAGCCGACCTGGTCTACGAATCTATTCTAACTATCAAAAAATTCCTAGAATTTTGGGCGGGATGGGCATTGTAATTCTTTCTACTTCTCGGGGTATAATGACAGACCGAGAAGCTCGACTCGAAAGAATAGGCGGAGAAATCTTGTGTTATATATGGTAA

>lcl|NC_010433.1_cds_YP_001718473.1_58 [gene=rpl14] [locus_tag=MaesCp058] [db_xref=GeneID:5999954] [protein=ribosomal protein L14] [protein_id=YP_001718473.1] [location=complement(85787..86155)] [gbkey=CDS]

ATGATCCAATCTCAGACCCATTTGAATGTAGCGGATAACAGCGGAGCTCGAGAATTGATGTGTATTCGAATCATAGGGACTAGTAATCGCCGATATGCTCATATCGGTGACGTTATTGTTGCTGTGATCAAGGAAGCAGCACCAAATTCACCTCTAGAAAGATCAGAAGTAATCAGAGCTGTAATTGTACGTACTTGTAAAGAACTCAAACGTGATAATGGTATGATAATACGATATGATGACAACGCTGCAGTTGTCATTGATCAAGAAGGAAATCCAAAGGGAACTCGAATTTTTGGTGCAATCGCCCGGGAATTGAGACAGTTAAATTTTACTAAAATAGTTTCATTAGCACCTGAAGTGTTATAA

>lcl|NC_010433.1_cds_YP_001718474.1_59 [gene=rpl16] [locus_tag=MaesCp059] [db_xref=GeneID:5999985] [protein=ribosomal protein L16] [protein_id=YP_001718474.1] [location=complement(join(86282..86680,87740..87748))] [gbkey=CDS]

ATGCTTAGTCCCAAAAGAACCCGATTCCGTAAACAACATAGAGGAAGAATGAAAGGAATAGCTTTTCGAGGTAATCGTATTTGTTTCGGCAGATATGCTCTTCAGGCACTTGAACCCGCTTGGATTACATCTAGACAAATAGAAGCGGGGCGACGAGCAATGACACGAAATGCACGCCGCGGTGGAAAAATATGGGTACGTATATTTCCCGACAAACCAGTTACTTTAAGACCTACGGAAACACGTATGGGTTCGGGGAAAGGATCTCCCGAATATTGGGTAGCTGTCGTTAAACCAGGTAGAATACTTTATGAAATGGGCGGAGTAGCCGAAAATATAGCGAGAAAAGCTATTTCAATAGCAGCATCAAAAATGCCTATACGAACTCAATTCATTATTTCAGGATAG

>lcl|NC_010433.1_cds_YP_001718475.1_60 [gene=rps3] [locus_tag=MaesCp060] [db_xref=GeneID:5999953] [protein=ribosomal protein S3] [protein_id=YP_001718475.1] [location=complement(87947..88603)] [gbkey=CDS]

ATGGGACAAAAAATAAATCCACTTGGTTTCAGACTTGGTACAACCCAAAGTCATCATTCTCTTTGGTTTGCACAACCAAAAAATTACTCTGAGGGTCTACAAGAAGATCAAAAAATAAGAAACTGTATCAAGAATTATGTAAAAAAAAATGCGAAAATATCTTCTGGTATTGAAGGAATTGTACGTATAGAGATTCAAAAACGAATTGATGTGATTCAGGTCATAATATATATGGGATTCCCAAAATTATTAATAGAAAGTAGACCTAAACGAATCGAAGAATTACAGATGAATGTACAAAAAGAACTTAATTGTGTGAATCGAAAATTCAATATTGCTATTACAAGAATTCCAAACCCTTACGGGCACCCTAATATTCTTGGAGAATTTATAGCCGGACAATTAAAAAATAGAGTTTCATTTCGCAAAGCAATGAAAAAAGCTATTGAATTAACTGAACAGGCCAATACAAAAGGAATTCAAGTGCAAATTGCTGGGCGTCTTGACGGAAAAGAAATTGCACGCGTCGAATGGATTAGAGAAGGTAGAGTTCCTCTACAAACCATTGGAGCTAAAATTGATTATTGTTCGTATACAGTTAGAACTATTTATGGGGTATTAGGCATAAAAATTTGGACATTTCCAGACAAGAAATAA

>lcl|NC_010433.1_cds_YP_001718476.1_61 [gene=rpl22] [locus_tag=MaesCp061] [db_xref=GeneID:6000051] [protein=ribosomal protein L22] [protein_id=YP_001718476.1] [location=complement(88701..89102)] [gbkey=CDS]

ATGATAAAGATAAAAAAAAGAAAGAGAAACACATATGAAGTATATGCTTTAGGCCAACATATATGTATGTCCCCTCACAAAGCACGAAGAATAATTGATCAGATTCGTGGACGTTCTTACGAAGAAACACTTATGATACTCGAGCTCATGCCTTATCGAGCATGTTATCCCATTTTTAAATTGATTTATTCCGCAGCAGCAAATGCTAGTCACAATATGGGTTTCAACGAAGCCAATTTAATCATTAGTAAAGCCGAAGTCAACGAAGGCACTACTGTGAAAAAATTAAAACCTCAGGCTCGAGGACGGGGTTATCCGATAAAAAGATCAACTTGTCATATATCTATTGTATTAAAAAATATATCCTTATATGAAGAATATGACGAATATATATATATATGA

>lcl|NC_010433.1_cds_YP_001718477.1_62 [gene=rps19] [locus_tag=MaesCp062] [db_xref=GeneID:6000005] [protein=ribosomal protein S19] [protein_id=YP_001718477.1] [location=complement(89184..89462)] [gbkey=CDS]

GTGACACGTTCACTAAAAAAAAATCCTTTTGTAGCAAATCATTTATTAAAAAAAATAAATAAGCTTAATAATAAAGCAGAAAAAGAAATAATAGTAACGTGGTCCCGGGCATCTACCATTATACCCACAATGATCGGCCATACTATCGCTATCCATAATGGAAAGGAACATTTACCTATTTATATAACAGATCGTATGGTGGGTCATAAATTGGGAGAATTTGCACCTACTCTAAATTTTCGAGGACATGCAAAAAATGATAATAAATCTCGTCGTTAA

>lcl|NC_010433.1_cds_YP_001718478.1_63 [gene=rpl2] [locus_tag=MaesCp063] [db_xref=GeneID:6000022] [protein=ribosomal protein L2] [protein_id=YP_001718478.1] [location=complement(join(89524..89958,90632..91030))] [gbkey=CDS]

ATGGCGATACATTTATACAAAACTTCTACCCCGAGCACACGCAATGGAGCCGTAGACAGTCAAGCGAAATCCAATACACGAAATACACGAAAGAATTTGATCTATGGACAGCATCGTTGTGGTAAAGGCCGTAATGCCAGAGGAATCATTACCGCAAGACATAGAGGGGGAGGTCATAAGCGTCTATACCGTAAAATCGATTTTCGACGGAATGAAAAAGACATATATGGTAGAATCGTAACCATAGAATACGACCCTAATCGAAATGCATACATTTGTCTCATACACTATGGGGATGGTGAGAAGAGATATATTTTACATCCCAGAGGGGCTATAATTGGAGATACCATTATTTCTGGTACAGAAGTTCCTATAAAAATGGGAAATGCCCTACCTTTGACCGATATGCCCTTAGGCACGGCCATACATAACATAGAAATCACACTTGGAAAGGGTGGACAATTAGCTAGAGCTGCAGGTGCTGTAGCGAAACTGATTGCAAAAGAGGGGAAATCAGCCACATTAAAATTACCTTCTGGGGAGGTTCGTTTAATATCCAAAAACTGCTCAGCAACAGTCGGACAAGTAGGGAATACTGGGGTGAACCAGAAAAGTTTGGGTAGAGCCGGATCTAAATGTTGGCTAGGTAAGCGTCCTGTAGTAAGAGGAGTAGTTATGAACCCTGTAGACCACCCCCATGGGGGTGGTGAAGGGAGGGCCCCAATTGGTAGAAAAAAACCCGCAACCCCTTGGGGTTATCCTGCACTTGGAAGAAGAAGTAGAAAAAGGAATAAATATAGTGATAATTTGATTCTTCGTCGCCGTAGTAAATAG

>lcl|NC_010433.1_cds_YP_001718479.1_64 [gene=rpl23] [locus_tag=MaesCp064] [db_xref=GeneID:5999982] [protein=ribosomal protein L23] [protein_id=YP_001718479.1] [location=complement(91049..91330)] [gbkey=CDS]

ATGGATGGAATCAAATATGCAGTATTTACAGACAAAAGTATTCGGTTATTGGGGAAAAATCAATATACTTTTAATGTCGAATCAGGATCAACTAGGACAGAAATAAAGCATTGGGTCGAACTCTTCTTTGGTGTCAAGGTAATAGCTATGAATAGCCATCGACTCCCGGGAAAGGGTAGAAGAATGAGACCTATTATGGGACATACAATGCATTACAGACGTATGATCATTACGCTTCAACCGGGTTATTCTATTCCACCTCTTAGAAAGAAAAGAACTTAA

>lcl|NC_010433.1_cds_YP_001718480.1_65 [gene=ycf2] [locus_tag=MaesCp065] [db_xref=GeneID:6000000] [protein=Ycf2] [protein_id=YP_001718480.1] [location=91678..98616] [gbkey=CDS]

ATGAAAGGACATCAATTAAAATCCTGGATTTTCGAATTGAGAGAGATATTGAGAGAGATCAAGTCAGTGGGATCTTTCATTCACATTTTTTTCCATCAAGAACGTTTTATAAAACTCTTGGACTCCCGAATTTGGAGTATCTTACTTTCACGCAATTCACAGGGTTCAACAAGCAATCGATATTTCACGATCAAGGGTGTAGTACTATTTGTAGTAGTGGTCCTTATATATCGTATTAACAATCGAAAGATGGTCGAAAGAAAAAATCTCTATTTGACAGGGCTTCTTCCTATACCTATGAATTCCATTGGACCCAGAAATGATACATTGGAAGAATCCTTTTGGTCTTCCAATATCAATAGGTTGATTGTTTCGCTCCTGTATCTTCCAAAAGGAAAAAAGATCTCTGAGAGCTCTTTCCTGGATCCGAAAGAGAGTACTTGGGTTCTCCCAATAACTAAAAAGTGTATCATGTCTGAATCTAACTGGGGCTCGCGGTGGTGGAGGAACTGGATCGGAAAAAAGAGGGATTCTAGTTGTAAGATATCTAATGAAACCGTCGCTGGAATTGAGATCTCATTCAAAGAAAAAGATATCAAATATCTGGAGTTTCTTTTTGTATATTATATGGATGATCCGATCCGCAAGGACCATGATTGGGAATTGTTTGATCGTCTTTCTCCGAGGAAGGGGCGAAACATAATCAACTTGAATTCGGGACAACTATTCGAAATCTTAGTGAAAGACTGGATTTGTTATCTCATGTTTGCTTTTCGTGAAAAAATACCAATTGAAGTGGAGGGTTTCTTCAAACAACAAGGAGCTGGGTCAACTATTCAATCAAATGATATTGAGCATGTTTCCCATCTCTTCTCGAGAAAGAAGTGGGCTATTTCTTTGCAAAATTGTGCTCAATTTCATATGTGGCAATTCCGCCAAGATCTCTTCGTTAGTTGGGGGAATAATCCGCACGAATCGGATTTTTTGAGGAACATATCGAGAGAGAATTGGATTTGGTTAGACAATGTGTGGTTGGTAAACAAGGATCGGTTTTTTAGCAAGGCACGGAATATATCGTCAAATATTCAATATGATTCCACAAGATCTAGTTTCGTTCAAGGAAGGAATTCTAGCCAATTGAAGGGATCTTCTGATCAATCCAGAGATCATTTCGATTCCATTAGTAATGAGGATTCGGAATATCACACATTGATCAATCAAAGAAAGATTCAACAACTAAAAGAAAGATCGATTCTTTGGGATCCTTCCTTTCTTCAAACGGAACGAACAGAGATAGAATCAGACCGATTCCCTAAATGCCTTTCTGGATATTCCTCAATGTCCCGGCTATTCACGGAAGGTGAGAAGGAGATGAATAATCATCTGCTTCCGGAAGAAATCGAAGAATTTCTTGGGAATCCTACAAGATCCATTCGTTCTTTTTTCTCTGACAGATCGTCAGAACTTCATCTGGGTTCGAATCCTACTGAGAGGTCCACTAGAAATCAGAAATTGTTGAAGAAAGAACAAGATGTTTCTTTTGTCCCTTCCAGGCGATCGGAAAATAAAGAAATAGTTAATATATTCAAGATAATCACGTATTTACAAAATACCGTCTCAATTCATCCTATTTCATCAGATCCGGGATGTGATATGGTTCTGAAGGATGAACTGGATATGGACAGTTCCAATAAGATTTCTTTCTTGAACAAAAATCCATTTTTTGATTTATTTCATCTATTCCATGATCGGAACGGGGGGGGATACACGTTACACCACGATTTTGAATCAGAAGAGAGATTTCAAGAAATGGCAGATCTATTCACTCTATCAATAACCGAGCCGGATCTGGTGTATCATAAGGGATTTACCTTTTTTATTGATTCCTACGGATTGGATCAAAAACAATTCTTGAATGAGGTATTCAACTCCAGGGATGAATCGAAAAAGAAATCTTTATTGGTTCTACCTCCTATTTTTTATGAAGAGAATGAATCTTTTTATCGAAGGATCAGAAAAAAATGGGTCCGGATCTCCTGCGGGAATGATTTGGAAGATCCAAAACAAAAAATAGTGGTATTTGCTAGCAACAACATAATGGAGGCAGTCAATCAATATGGATTGATCCTAAATCTGATTCAAATCCAATATAGTACCTATGGGTACATAAGAAATGTATTGACTCAATTCTTTTTAATGAATAGATCCGATCGCAACTTCGAATATGGAATTCAAAGGGATCAAATAGGAAATGATACTCTGAATCATAGAACTATAATGAAATATACGATCAACCAACATTTATCGAATTTGAAACAGAGTCAGAAGAAATGGTTCGATCCTCTTATTTTTATTTTTCTTTCTCGAACCGAGAGATCCATGAATTGGGATCCTAATGCATATAGATACAAATGGTCTAATGGGAGCAAGAATTTCCAGGAACATTTGGAACATTTCATTTCTGAGCAGAAGAGCCGTTTTCTTTTTCAAGTAGTGTTCGATCGATTACGTATTAATCAATATTCGATTGATTGGTCTGAGGTTATCGACAAAAAAGATTTGTCTAAGTCACTTCGTTTCTTTTTGTCCAAGTTACTTCTTTTTTTGTCCAAGTTTCTTCTCTTTTTGTCTAACTCACTTCCTTTTTTCTTTGTGAGTTTCGGGAATATCCCCATTCATAGGTCCGAAATCCATATCTATGAATTGAAAGGTCCGAATGATCAACTCTGCAATCAGCTGGTAGAACCAATAGGTCTTCAAATCGTTCATTTGAAAAAATTGAAACCCTTCTTATTGTTATTGGATGATCATGATACTTCCCAAAAATCGAAATTTTTGATTAATGGAGGAACAATATCACCATTTTTGTTCAATAAGATACCAAAGTGGATGATTGACTCATTCCATACTAGAAATAATCGCAGGAAATCTTTTGATAACACGGATTCCTATTTCTCAATGATATCCCACGATCAAGACAATTGGCTGAATCCCGTGAAACCATTTCATAGAAGTTCATTGATATCTTCTTTTTATAAAGCAAATCGACTTCGATTCTTGAATAATCTACATCACTTCTGCTTCTATTGTAACAAAAGATTCCCTTTTTATGTGGAAAAGGCCCGTATCAAGAATTATGATTTTACGTATGGACAATTCCTCAATATCTTGTTCATTCGCAACAAGATATTTTCTTTGTGCGGCGGTAAAAAAAAACATGCTTTTTTGGAGAGAGATACTATTTCACCAATCGAGTCACAGGTATCTAACATATTTATACCTAATGATTTTCCACAAAGTGGTAACGAAAGGTATAACTTGTACAAATCTTTCCATTTTCCAATTCGATCCGATCCATTCGTTCGTAGAGCTATTTATTCGATCGCAGACATTTCTGGAACACCTCTAACAGAGGGACAAATAGTCAATTTTGAAAGAACTTATTGTCAACCTCTTTCGGATATGAATCTATCTGATTCAGAAGGGAAGAACTTGCATCAGTATCTCAATTTCAATTCAAACATGGGTTTGATTCACACTCCATGTTCTGAGAAATATTTACCATCCGAAAAGAGGAAAAAACGGAGTCTTTGTCTAAAGAAATGTGTTGAAAAAGGGCAGATGTATAGAACCTTTCAACGAGATAATGCTTTTTCAACTCTCTCAAAATGGAATCTATTCCAAACATATATGCCATGGTTCCTTACTTCGACGGGGTACAAATATCTAAATTTGATATTTTTAGATACCTTTTCGGACCTATTACCGATACTAAGTAGCAGTCAAAAATTTTTATCCATTTTTCATGATATTATGCATGGATCAGATATATCATGGCTAATTTTTCAGAAAAGATTGTGGAAGATATGCCGGAATCTGATAAGTGAGATTTCGAGTAAGTGTTTACATAATCTTCTTCTGTCCGAAGAAATGATTCATCGAAATAATGAGCCACCATTGATATCGACACATCTGAGATCGCCAAATGTTCGGGAGTTCCTCTATTCAATCCTTTTCCTTCTTCTTGTTGCTGGATATCTCGTTTGTACACATCTTCTCTTTGTTTCCCACGCCTATAGTGAGTTACAGACAGAGTTCGAAAAGGTCAAATCTTTGATGATTCCATCATACATGATTGAGTTGCGAAAACTTCTGGATAGGTATCCTACATCTGAACTGAATTCTTTCTGGTTAAAGAATCTCTTTCTAGTTGCTCTGGAACAATTAGGAGATTTTCTAGAAGAAATGCGGGGTTCTGCTTCTGGCGGCAACATGCTATGGGGTGGTGGTCCCGCTTATGGGGTTAAATCAATACGTTCTAAGAAGAAATTTTGGAATATCAATCTCATCGATCTCATAAGTATCATACCAAATCCCATCAATCGAATCACTTTTTCGAGAAATACGAGACATCTAAGTCATACAAGTAAAGAGATTTATTCATTGATAAGAAAAAGAAAAAACGTGAACGGTGATTGGATTGATGATAAAATAGAATCCTTGGTCGCGAACAGTGATTGGATTGATGATAAAGAAAGAGAATTCTTGGTTCAGTTCTCCACCTTAACGACAGAAAAAAGGATTGATCAAATTCTATTGAGTCTGACTCATAGTGATCATTTATCAAAGAATGACTCTGGTTATCAAATGATTGAAGAGCCGGGAGCAATTTATTTACGATACTTAGTTGACATTCATAAAAAGTATCTAATGAATTATGAGTTCAACACACCCTGTTTAGCAGAAAGACGGATATTCCTTGCTTATTATCAGACAACCACTTATTCACAAACCTCGTGTGGGGTGAATAGTTTTCATTTCCCATCTCATGGAAAACCCTTTTCGCTCCGCTTAGCCCTATCCCCCTCTAGGGGTATTTTAGTGATAGGTTCTATAGGAACTGGACGATCCTATTTGGTCAAATACCTAGCGACAAACTCCTATCTTCCTTTCGTTACAGTATTTCTGAACAAGTTCCTGAATAACAAGCCTAAGGGTTTTCTTATTGATGATAGTGACGATATTGATGATAGTGACGATATTGATGATAGTGACGATATTGATGCTAGTGACGATATTGATGTGAGTGACGATATTGATGTGAGTGACGACGATATCGACCGTGACTTTGACTTTGATACGGAGCTGGAGTTTCTAACTACGATGGATGCGCTAACTATTGATATGATGCCGGAAATAGAAATAAACCGATTTTATATCACCCTTCAATTCGAATTAGCAAAAGCAATGTCTCCTTGCATAATATGGATTCCAAACATTCATGATCTGGATGTGAATGAGTCGAATTACTTATCCCTCGGTCTATTAGTGAACTATCTCTCCAGGGATTGTGAAAGATGTTCCACTAGAAATATTCTTGTTATTGCTTCGACTCATATTCCCCAAAAAGTGGATCCCGCTCTAATAGCTCCGAATAAATTAAATACATGCATTAAGATACGAAGGTTTCTTATTCCACAACAACGAAAGCACTTTTTTACTCTTTCATATACTAGGGGATTTCACTTGGAAAATAAAATGTTCCATACTAATGGATTCGGGTCCATAACCATGGGTTCCAATGTACGAGATCTTGTAGCACTTACCAATGAGGCCCTATCGATTAGTATTACACAGAAGAAATCAATTATAGACACTAATATAATTAGATCTGCTCTTCATAGACAAACTTGGGATTTGCGATCCCGGGTAAGATCGGTTCAGGATCATGGGATCTTTTTCTATCAGATAGGAAGGGCTGTTGCACAAAATGTATTTCTAAGTAATTGCCCCATAGATCCTATATCTATCTATATGAAGAAGAAATCATGTAACGAAGGGGATTCTTATTTGTACAAATGGTACTTCGAACTTGGAACGAGCATGAAGAAATTAACGATACTTCTTTATCTTTTGAGTTGTTCTGCCGGATCGGTTGCTCAAGACCTTTGGTCTCTACCCGGACCCGATGAAAAAAATGGGATCACTTATTATGGACTTGTTGAGAATGATTCTGATCTAGTTCATGGCCTATTAGAAGTAGAAGGCGCTCTGGTGGGATCCTCACGGACAGAAAAAGATTGCAGTCAGTTTGATAATGATCGAGTGACATTGCTTCTTCGGCCCGAACCAAGGAGTCCCTTAGATATGATGCAAAATGGATCTTGTTCTATCCTTGATCAGAGATTTCTCTATGAAAAATACGAATCGGAGTTTGAAGAAGGGGAAGGAGAAGAAGTCCTCGACCCGCAACAGATAGAGGAGGATTTATTCACTCACATAGTTTGGGCTCCTAGAATATGGCGCCCTTGGGGTTTTCTATTTGATTGTATTGAAAGGCCCAATGAATTGGGATTTCCCTATTGGGCCAGGTCATTTCGGGGCAAGCGGATCATTTATGATGAAGAGATCATTTATGATGAAGAGATCATTTATGATGAAGAGGATGAGCTTCAAGAGAATGATTCGGAGTTCTTGCAGAGTGGAACCATGCAGTACCAGATACGAGATAGATCTTCCAAAGAACAAGGCTTTTTTCGAATAAGCCAATTCATTTGGGACCCTGCGGATCCACTCTTTTTCCTATTCAAAGATCAGCCCTTTGTCTCTGTGTTTTCACATCGAGAATTCTTTGCAGATGAAGAGATGTCAAAGGGGCTTCTTACTTCCCAAACAGATCCTCCTACATCTATATATAAACGCTGGTTTATCAAGAATACGCAAGAAAAGCGCTTCGAATTGTTGATTCATCGCCAGAGATGGCTTAGAACCAATAGTTCATTATCTAATGGATTTTTCCGTTCTAATACTCTATCCGAGAGTTATCAGTATTTATCAAATCTGTTCCTATCTAACGGAACGCTATTGGATCAAATGACAAAGGCATTGTTGAGAAAAAGATGGCTTTTCCCGGATGAAATGAAAATTGGATTCATGTAA

>lcl|NC_010433.1_cds_YP_001718481.1_66 [gene=ndhB] [locus_tag=MaesCp066] [db_xref=GeneID:6000013] [protein=NADH dehydrogenase subunit 2] [protein_id=YP_001718481.1] [location=complement(join(100339..101094,101777..102553))] [gbkey=CDS]

ATGATCTGGCATGTACAGAATGAAAACTTCATTCTCGATTCTACGAGAATTTTTATGAAAGCCTTTCATTTGCTTCTCTTCGATGGAAGTTTTATTTTCCCAGAATGTATCCTAATTTTTGGCCTAATTCTTCTTCTGATGATCGATTCAACCTCTGATCAAAAAGATATACCTTGGTTATATTTCATCTCTTCAACAAGTTTAGTAATGAGTATAACGGCCCTATTGTTCCGATGGAGAGAAGAACCTATGATTAGCTTTTCGGGAAATTTCCAAACGAACAATTTCAACGAAATCTTTCAATTTCTTATTTTACTATGTTCAACTCTATGTATTCCTCTATCCGTAGAGTACATTGAATGTACAGAAATGGCTATAACAGAGTTTCTCTTATTCGTATTAACAGCTACTCTAGGAGGAATGTTTTTATGCGGTGCTAACGATTTAATAACTATCTTTGTCGCTCCAGAATGTTTCAGTTTATGCTCCTACCTATTATCTGGATATACCAAGAAAGATGTACGGTCTAATGAGGCTACTACGAAATATTTACTCATGGGTGGGGCAAGCTCTTCTATTCTGGTTCATGCTTTCTCTTGGCTATATGGTTCGTCCGGGGGAGAGATCGAGCTTCAAGAAATAGTGAATGGCCTTATCAATACACAAATGTATAACTCCCCAGGAATTTCAATTGCGCTTATATTCATCACTGTAGGAATTGGGTTCAAGCTTTCCCTAGCCCCTTCTCATCAATGGACTCCTGACGTATACGAAGGATCTCCCACTCCAGTCGTTGCTTTTCTTTCTGTTACTTCGAAAGTAGCTGCTTCAGCTTCAGCCACTCGAATTTTCGATATTCCTTTTTATTTCTCATCAAACGAATGGCATCTTCTTCTGGAAATCCTAGCTATTCTGAGCATGATAGTGGGGAATCTCATTGCTATTACTCAAACAAGCATGAAACGTATGCTTGCATATTCGTCCATAGGTCAAATCGGATATGTAATTATTGGAATAATTGTTGGAGACTCTAATGGTGGATATGCAAGCATGATAACTTATATGCTCTTCTATATCTCCATGAATCTAGGAACTTTTGCTTGTATTGTATTATTTGGTCTACGTACCGGAACTGATAACATTCGAGATTATGCAGGATTATACACGAAAGATCCTTTTTTGGCTCTCTCTTTAGCCCTATGTCTCTTATCCCTAGGAGGTCTTCCTCCACTAGCAGGTTTTTTCGGAAAACTCCATTTATTCTGGTGTGGATGGCAGGCAGGCCTATATTTCTTGGTTTTAATAGGACTCCTTACGAGCGTTGTTTCTATCTACTATTATCTAAAAATAATCAAGTTATTAATGACTGGACGAAACCAAGAAATAACCCCTCACGTGCGAAATTATAGAAGATCCCCTTTAAGATCAAACAATTCCATCGAATTGAGTATGATTGTATGTGTGATAGCATCTACTATACCAGGAATATCAATGAACCCGATTGTTGAAATTGCTCAAGATACCCTTTTTTAG

>lcl|NC_010433.1_cds_YP_001718482.1_67 [gene=rps7] [locus_tag=MaesCp067] [db_xref=GeneID:5999962] [protein=ribosomal protein S7] [protein_id=YP_001718482.1] [location=complement(102864..103331)] [gbkey=CDS]

ATGTCACGTCGAGGTACTGCAGAAGAAAAAACTGCAAAATCCGATCCAATTTATCGTAATCGATTAGTTAACATGTTGGTTAACCGTATTCTGAAACACGGAAAAAAATCATTGGCTTATCAAATTATCTATCGAGCCATGAAAAAGATTCAACAAAAGACAGAAACAAATCCACTATCTGTTTTACGTCAAGCAATACGTGGAGTAACTCCCGATATAGCAGTAAAAGCAAGACGTGTAGGCGGATCGACTCATCAAGTTCCCATTGAAATAGGATCCACACAAGGAAAAGCACTTGCCATTCGTTGGTTATTAGGGGCATCCCGAAAACGTCCGGGTCGAAATATGGCTTTCAAATTAAGTTCCGAATTAGTGGATGCTGCCAAAGGGAGTGGTGATGCCATACGCAAAAAGGAAGAGACTCATAGAATGGCAGAGGCAAATAGAGCTTTTGCACATTTTCGTTAA

>lcl|NC_010433.1_cds_YP_001718483.1_68 [gene=ndhF] [locus_tag=MaesCp069] [db_xref=GeneID:6000055] [protein=NADH dehydrogenase subunit 5] [protein_id=YP_001718483.1] [location=complement(116204..118462)] [gbkey=CDS]

ATGGAACATATATATCAATATTCATGGATCATACCTTTCGTTACATTGCCAGTACCTATGTTAATCGGAGCGGGACTCCTGCTTTTTCCGGCAGCAACAAAAAAACTGCGGCGTATGTGGGCTTTTCCAAGCGTTTTCTTGTTAAGTATAGTCATGATTTTTTCAATCGATTTGTCTATTCAGCAAATAAATAGTAGTTTTATTTATCAATATATATGGTCGTGGACTATCAATAATGATTTTTCTTTAGAGTTCGGACACTTGATTGACCCACTTACTTCTATTTTGTCAGTATTAATTACTACAGTTGGCATTTTGGTTCTTTTTTATAGTGACAATTATATGTCTCATGATCAAGGCTATTTGAGATTTTTTGCTTATATGAGTTTTTTCACTACTTCAATGTTGGGATTAGTTACTAGTTCTAATTTGATACAAATTTATATTTTTTGGGAATTGGTTGGAGTGTGTTCTTATCTATTAATAGGTTTTTGGTTCACACGACCTATTGCATCGAATGCTTGTCAAAAAGCGTTTGTAACTAATCGCGTAGGGGATTTTGGTTTATTATTAGGTATTTTAGGTCTTTATTGGATAACGGGCAGTTTCGAATTTCGGGATTTGTTCAAAATCTTCAATAACTTGATTTATAATAATCAGGTTAATTTTTTATTTGTTACTTTGTGTGCCGTTCTATTATTTTCTGGCGCAATTGCTAAATCGGCGCAATTTCCTCTTCATGTATGGTTACCGGATGCCATGGAAGGGCCTACTCCTATTTCGGCTCTGATACATGCTGCTACTATGGTAGCGGCGGGAATTTTTCTTGTAGCTCGCCTTTTTCCTCTTTTCGTAATTATACCTTTCATAATGAATCTAATAGCTTTGATAGGTATAATAACAGTATTTTTAGGAGCTACTTTAGCTCTTGCTCAAAAAGATATTAAGAGAAGTTTAGCCTATTCTACAATGTCTCAATTGGGTTATATGATGTTAGCTCTAGGTATGGGGTCTTATCGGGCCGCTTTATTTCATTTGATTACTCATGCCTATTCGAAAGCATTGTTGTTTTTAGGATCTGGATCCATTATTCATTCAATGGAAGCTATTCTTGGTTATTCTCCAGATAAGAGTCAAAATATGGTTCTTATGGGTGGTTTAACAAAACATATTCCAATTACCAAAACGGCTTTTTTATTAGGAACACTTTCTCTTTGTGGTATTCCCCCCTTCGCCTGTTTTTGGTCCAAAGATGAAATTCTTAATGATAGTTGGTTGTATTCACCTATTTTCGCAATAATAGCCTGTTTCACAGCGGGATTAACCGCATTTTATATGTTTCGGGTTTATTTACTTACTTTTGACGGACATTTTAATGCTCATTTTCAAAGTTACAGTGGTAAAAAAAACAGTTCATTTTATTCAATATCTTTATGGGGTAAAGAAGGATCAAAAATGCTTAACAAAAATTTGCGTTTATTAGCTTTATTAACAATGAATAATAAGGAAAGGGCTTCTTTTTTTTGGAAGAACACATATCAAATTGATGGTAATGTAAGAAATATGACGTGGCCTTTTATTACTATTCAAAATTTTAACACTAAAAGGATTTTTTCCTATCCCCATGAATCGGATAATACTATGTTATTTCCTATGCTTATCTTAGTACTATTTACTTTGTTTATTGGAGCCATAGGAATTCCTTTCAATCAATTCAATCAAGAAGGAATGCTGTTGGATATAGATATATTGTCAAAACTTTTAACTCCGTCTTTAAACCTTTTGCATCAAAATCCAGAAAATTCGGTGGATTGGTATGAATTTGTAACAAATGCAACTTTTTCAGCCAGTATAGCTTTTTTTGGAATATTTATAGCGTCCTTTTTATATAAGCCTGTTTATTCATCGTTACAAAATTTGAATTTCTTTAATTCGTTCGCTAAAAAAGGTCCTAAGAGAATTCTTTGGGACAAAATAATAAATGTGATATATAATTGGTCCTCTAATCGAGGTTATATAGATGCTTTTTATGCAATATCTTTTATTGGAGGTATAAGAAAATTGGCTGAATTAATTCATTTTTTTGATAAACAAATAATTGATGGAACCCCCAATGGGGTCGGTGTTACTAGTTTCTTTGTAGGAGAGGGTATAAAAAATGTAGGAAGTGGTCGCATCTCTTTTTATCTCTTATTTTATTTATTTTATGCGTTAATCTTCTTATTAATTTACTACTCTGTTTATAAATTCATTATTTAG

>lcl|NC_010433.1_cds_YP_001718484.1_69 [gene=rpl32] [locus_tag=MaesCp070] [db_xref=GeneID:5999996] [protein=ribosomal protein L32] [protein_id=YP_001718484.1] [location=119437..119598] [gbkey=CDS]

ATGGCAGTTCCAAAAAAGCGCACTTCTATATCAAAAAAGCGTATTCGAAAAAATCTTTGGAAAAGAAAGGGATATTGGACAGTATTGAAAGCTTTTTCATTAGCGAAATCTCTTTCTACAGGTAATTCAAAAAGTTTTTTTGTGCGACAAATAAAAGATTAA

>lcl|NC_010433.1_cds_YP_001718485.1_70 [gene=ccsA] [locus_tag=MaesCp071] [db_xref=GeneID:6000045] [protein=cytochrome c biogenesis protein] [protein_id=YP_001718485.1] [location=120604..121572] [gbkey=CDS]

ATGATATTCTCGACTTTAGAACATATATTAACACATATATCTTTTTCAGTCGTGTCAATTGTAATTACAATTCATTTGATAACCTTATTAGCCGATGAATTCGTAGAACTATATGATTCGTCAGAAAAGGGCATGATAACTACTTTTTTTTGTATAACAGGATTATTAGTTACTCGTTGGATTTTTTTGGGACATTTACCATTAAGTGATTTATATGAATCATTAATCTTTCTTTCATGGTCTTTTTCCATTATTCATATGGTTCCGTATTTTAAAAAACATAAAAATTTTTTAAGCGCAATAACCGCGCCAAGTACTTTTTTTACCCAAGGGTTTGCTACTTCGGGTCTTTTAACTGACATGCATCAATCCGAAATCTTAGTGCCCGCTCTCCAATCCCAGTGGTTAATGATGCACGTAAGTATGATGATATTGGGCTATGCAGCTCTTTTGTGTGGATCATTATTTTCAGTAGCATTTCTAGTAATCACATTTCGAAAAATCATAAGAATTTTTGATAAAAGCAATAATTTATTAAACAATTCGTTTTTCTTTAGTGAGATACAATATATGGCGGAAAGAAAGAATGTTTTAAGAAATATTTCTTTTCTTTCTTCTAGGAATTATTACAGGTTTCAATTGATTCAACAATTAGATGACTGGGGTTATCGTATTATAAGTATAGGGTTTATCTTTTTAACAATAGGTATTCTTTCGGGAGCAGTCTGGGCTAATGAAGCATGGGGATCGTATTGGAATTGGGACCCAAAAGAAACTTGGGCATTTATTACGTGGACCATATTCGCGATTTATTTCCATATTCGAACAAATAAAAAATTGGAGGGTTTCCATTCCGCAATTGTCGCTTCTATCGGTTTTCTTCTAATTTGGATATGCTATTTTGGAGTTAATTTATTAGGAATAGGACTACATAGTTATGGTTCATTTACATTAACAATTAGTATCTAA

>lcl|NC_010433.1_cds_YP_001718486.1_71 [gene=ndhD] [locus_tag=MaesCp072] [db_xref=GeneID:6000015] [protein=NADH dehydrogenase subunit 4] [protein_id=YP_001718486.1] [location=complement(121844..123346)] [gbkey=CDS]

ATGAATTCTTTTCCTTGGTTAACAATATTTGTAGTTTTACCGATATCCGGGGGTTCCTTAATTTTCCTTTTCCCTCATAGAGGAAATAAAGTAATAAAATGGTATACTATATTTATATGCATCTTTGAGCTACTTTTAATGACTTATGCGTTCTCTTATTATTTCCAATTGGACGATCCATTAATCCAATTAACAGAAGATTATAAATGGATCCAATTTTTTGATTTTTACTGGAGATTGGGAATCGATGGATTTTCTTTAGGACCTATTTTACTGACAGGATTTATCACCACTTTAGCTACTTTAGCGGCTCGGCCAATTACTCGGGATTCCCGATTATTTCATTTTCTGATGTTAGCAATGTATAGTGGTCAAATAGGATTATTTTCTTCTCAAGATCTTTTACTTTTTTTTATCATGTGGGAGTTAGAATTAATTCCCGTTTATCTACTTCTATCCATGTGGGGGGGAAAGAAACGTCTGTATTCAGCTACAAAGTTTATTTTGTATACTGCGGGAGGTTCCGTTTTTTTATTAATGGGAGCTTTGGGTATCGCTTTATATGGTTCTAATGAACCAAGATTCCATTTTGAAACATCAGCTAATCAATCATATCCTGTGGCGCTAGAAATATTTTTCTATATTGGATTTCTTATTGCTTTTGCTGTCAAATCACCGATTATACCCTTACATACATGGTTACCAGACACCCACGGGGAAGCACATTACAGTACTTGTATGCTTCTAGCCGGAATCTTATTAAAAATGGGGGCGTACGGATTGGTTCGAATCAATATGGAATTATTACCTCACGCTCATTCTATTTTTTCTCCCTGGTTGATAATAGTAGGCGTAATGCAAATAATCTATGCAGCTTCAACATCTCCTGGTCAACGAAATTTAAAAAAAAGAATAGCCTATTCTTCTGTATCTCATATGGGTTTCATAATTATAGGAATTTGCTCTATAAGTGATATGGGACTCAATGGAGCCATTTTACAAATAATATCACATGGATTTATTGGTGCCGCACTTTTTTTCTTGGCAGGAACGGGTTATGATAGAATACGTCGTGTTTATCTTGATGAAATGGGCGGAATGGCTACCTCAATGCCAAAAATATTCACGACATTCAGTATCTTATCACTAGCTTCCCTTGCATTACCAGGCATGAGCGGTTTTTTTGCGGAATTGATAGTATTTTTTGGAATAATTACCGGCCAAAAATATCTTTTAATGTCAAAAATATTAATTACTTTTGTAATGGCAGTTGGAATGATATTAACTCCTATTTATTTATTATCTATGTTACGCCAGATGTTTTATGGATACAAGCTGTTTAATGCCCCAAACTCTTATTTTTTTGATTCTGGACCGCGGGAGCTATTTGTTTCGATCTCTATCCTTCTGCCTGTAATAGGGATTGGTTTTTATCCGGATTTCGTTTTCTCATTATCAGTTGACCGGGTTGAAGCTATTCTATCTAATTATTTTTATAGATAG

>lcl|NC_010433.1_cds_YP_001718487.1_72 [gene=psaC] [locus_tag=MaesCp073] [db_xref=GeneID:6000033] [protein=photosystem I subunit VII] [protein_id=YP_001718487.1] [location=complement(123487..123732)] [gbkey=CDS]

ATGTCACATTCAGTAAAGATTTATGATACATGTATAGGGTGTACTCAATGTGTCCGAGCCTGCCCCACAGATGTATTAGAAATGATACCTTGGGATGGATGTAAATCTAAGCAAATTGCTTCTGCTCCAAGAACAGAGGACTGTGTCGGTTGTAAGAGATGTGAATCCGCCTGTCCAACAGATTTCTTGAGTGTTCGAGTTTATTTATGGCATGAAACAACTCGAAGCATGGGTCTAGCTTATTGA

>lcl|NC_010433.1_cds_YP_001718488.1_73 [gene=ndhE] [locus_tag=MaesCp074] [db_xref=GeneID:6000075] [protein=NADH dehydrogenase subunit 4L] [protein_id=YP_001718488.1] [location=complement(123984..124289)] [gbkey=CDS]

ATGATGCTCGAACATGTACTTGTTTTGAGTGCCTATTTATTTTCTATTGGTATCTATGGATTGATCACGAGTCGAAATATGGTTAGAGCCCTTATGTGCCTTGAACTTATACTGAATGCAGTTAATCTAAATTTCGTAACATTTTCTGATTTTTTTGATAGTCGCCAATTAAAAGGAAATATTTTTTCAATTTTTGTTATAGCTATCGCAGCCGCTGAAGCAGCTATTGGACCAGCTATTGTTTCGGCAATTTATCGTAATAGAAAATCAATCCATATCAATCAATCGAATTTGTTGAATAAGTAG

>lcl|NC_010433.1_cds_YP_001718489.1_74 [gene=ndhG] [locus_tag=MaesCp075] [db_xref=GeneID:6000007] [protein=NADH dehydrogenase subunit 6] [protein_id=YP_001718489.1] [location=complement(124548..125078)] [gbkey=CDS]

ATGGATTTGCCTGGACTAATTCATGATTTTCTTTTAGTCTTTCTGGGGTTAGGTCTTATATTAGGAGGTCTAGGAGTAGTATTACTTACCAACCCAATTTATTCTGCCTTTTCGTTGGGATTGGTTCTTGTTTGTATATCTTTATTCTATATTTTATCAAACTCTCATTTTGTAGCTGCCGCACAGCTCCTTATTTATGTGGGAGCTATAAATGTTTTAATTATATTTGCCGTGATGTTCATGAATGGTTCAGAATATTACAAAGATTTTAATCTTTGGACTGTTGGAAGCGGGGTTACTTCCTTAGTTTGTACAAGTATTTTTGTTTCACTAATTACTATTATTCCAGATACGTCATGGTACGGAATTATTTGGACTACAAAAACAAATCAGATTATAGAACAAGATTTGATAAGTAATGGCCAACAAATTGGAATTCATTTATCAACAGATTTTTTTCTTCCATTTGAATTCATTTCAATAATTCTTTTAGTTGCTTTGATAGGTGCGATTGCTGTGGCTCGTCAGTAA

>lcl|NC_010433.1_cds_YP_001718490.1_75 [gene=ndhI] [locus_tag=MaesCp076] [db_xref=GeneID:6000073] [protein=NADH dehydrogenase subunit I] [protein_id=YP_001718490.1] [location=complement(125518..126015)] [gbkey=CDS]

ATGTTTCCCATGGTAACTGGGTTCATGAATTATGGGCAACAAACCATACGAGCTGCAAGGTACATTGGTCAAGGTTTCATGATTACCTTATCTCATGCAAATCGTTTACCTGTAACTGTTCAATATCCTTATGAAAAATTAATCACATCGGAGCGTTTCCGCGGTCGAATTCATTTTGAATTTGATAAATGCATTGCTTGTGAAGTATGTGTTCGTGTATGTCCTATAGATCTACCTGTTGTTGATTGGAAATTGGAAACTGACATTCGAAAGAAACGGTTGCTTAATTACAGTATTGATTTCGGAATCTGTATATTTTGTGGCAACTGTGTTGAGTATTGTCCGACAAATTGTTTATCGATGACTGAAGAATATGAGCTTTCTACTTATGATCGTCACGAATTGAATTATAATCAAATTGCTTTAGGTCGTTTACCAATGTCAGTAGTTGACGATTATACAATTCGAACAATTTTGAATTCAACTCAAAAAAAATAG

>lcl|NC_010433.1_cds_YP_001718491.1_76 [gene=ndhA] [locus_tag=MaesCp077] [db_xref=GeneID:5999995] [protein=NADH dehydrogenase subunit 1] [protein_id=YP_001718491.1] [location=complement(join(126097..126636,127760..128311))] [gbkey=CDS]

ATGATAATTGATACAACAGAAGTACAAGCTATCAATTCTTTTTCTAGATTAGAATCCTTAAACGAGGTCTATGGAATTATATGGGTGTTTGCCCCGATTTTTATTCTTGTATTGGGAATCACGACAGGCATACTAGTAATTGTATGGTTAGAAAGAGAAATATCTGCAGGGATACAACAACGTATTGGACCTGAATATGCCGGTCCTTTAGGAGTTCTTCAAGCTCTAGCGGATGGGACAAAACTACTTTTCAAAGAGAATCTTTTTCCATCTAGGGGGGATATTCGTTTATTCAGTATCGGACCATCCATAGCAGTCATATCAACTATATTAAGCTATTCGGTAATTCCTTTTGGCTATCACCTTGTTTTAACTGATCTAAATATTGGTGTTTTTTTATGGATTGCCATTTCAAGTATTGCTCCCATCGGACTTCTTATGTCAGGATATGGATCAAATAATAAATATTCCTTTTTGGGTGGTTTACGAGCTGCTGCTCAATCGATTAGTTATGAAATACCATTAACTCTTTGTGTGTTATCCATATCTCTATTATCTAATAGTTCAAGTACAGTTGATATAGTTGAGGCACAATCAAAATCTGGTTTTTGGGGGTGGAATTTGTGGCGTCAACCTATAGGATTTATCATTTTTTTTATTTCTTCTCTAGCAGAATGTGAGAGATTACCTTTTGATTTACCAGAAGCAGAAGAAGAATTAGTAGCAGGTTATCAAACCGAATATTCGGGCATCAAATTTGGTTTATTTTATATTGCTTCCTATCTAAACTTATTAGTTTCTTCATTATTTGTAACAGTTCTTTACTTGGGCGGTTGGAATATCTCTATTCCGTATATATTCGTTCCTGAGCTTTTTGAAATAAAAAAAATGAGCGGAGTCTTTGGAACAACAATTGGTATCTTTATTACATTGGTTAAAACTTATTTGTTCTTGTTCATTCCTATCACAACAAGATGGACTTTACCTAGACTAAGAATGGACCAACTTTTAAATCTTGGATGGAAATTTCTTTTACCTATTTCTCTCGGTAATCTATTATTAACAACCTCTTTCGAACTTCTTTCACTATAA

>lcl|NC_010433.1_cds_YP_001718492.1_77 [gene=ndhH] [locus_tag=MaesCp078] [db_xref=GeneID:5999966] [protein=NADH dehydrogenase subunit 7] [protein_id=YP_001718492.1] [location=complement(128313..129494)] [gbkey=CDS]

ATGAATGTACCAGCTACACGAAAAGACCTTATGATAGTTAATATGGGTCCCCACCACCCATCAATGCATGGTGTTCTTCGACTCATCGTTACTCTAGACGGGGAAGATGTTATTGACTGCGAACCAATATTAGGTTATTTACACAGAGGAATGGAAAAAATTGCGGAAAATCGAACAATTATACAATATTTGCCCTATGTAACACGTTGGGATTATTTGGCTACTATGTTCACAGAAGCAATAACAGTAAATGGTCCAGAACTGTTAGGAAATATTCAAGTGCCTAAAAGAGCTGGCTATATCAGAGTAATTATGTTGGAATTGAGTCGTATAGCTTCTCATTTGTTATGGCTTGGCCCTTTTATGGCAGATATTGGTGCACAGACTCCTTTCTTCTATATCTTTAGAGAAAGAGAGTTAGTATATGATTTATTCGAAGCTGCCACTGGTATGAGAATGATGCATAATTTTTTTCGTATCGGAGGAGTAGCGTCTGATCTACCTCATGGTTGGATAGATAAATGTTTGGATTTTTGCGATTATTTTTTAACAGGAGTTACTGAATATCAAAAACTTATTACGCGAAATCCTATTTTTTTAGAACGAGTTGAGGGGGTAGGTATTGTTGGTACAGAAGAAGCAATAAATTGGGGTTTATCAGGACCAATGCTACGAGCTTCTGGAGTACAATGGGATCTTCGTAAAGTTGATCATTATGAGTGTTACGACGAATTTGATTGGGAAATCCAGTGGCAAAAAGAAGGAGATTCCTTAGCTCGTTATTTAGTCCGGATTGGTGAAATGCTGGAATCTATAAAAATTATTCAACAGGCTCTGGAAGGAATTCCGGGGGGGCCCTATGAGAATTTAGAAACCCGACGCTTTGATAGAGAAAGGGATTCGGAATGGAACGATTTCGAATATCGATTCATTAGTAAAAAAACTTCTCCTACTTTTGAATTACCGAAACAAGAACTTTATGTGAGAGTCGAAGCACCAAAAGGAGAATTGGGAATTTTTCTGATAGGGGATCAGAGCGGTTTTCCTTGGAGATGGAAAATTCGTCCGCCAGGTTTTATCAATTTGCAAATTCTTCCTGAATTAGTTAAAAGAATGAAATTGGCTGATATTATGACAATACTAGGTAGTATAGATATCATTATGGGAGAAGTTGATCGTTGA

>lcl|NC_010433.1_cds_YP_001718493.1_78 [gene=rps15] [locus_tag=MaesCp079] [db_xref=GeneID:6000001] [protein=ribosomal protein S15] [protein_id=YP_001718493.1] [location=complement(129592..129864)] [gbkey=CDS]

ATGGTAAAAAATTCATTCATTTCAGTTATTTCACAAGAAGAAAAAGACGAAAACAAGGGATCTGTTGAATTTCAAATAGTAAGTTTCACTAATAAGATACGAAGACTTACTTCACATTTTGAATTGCATAGAAAAGACTATTTATCTCAGAGAGGTTTGCGAAAAATTCTAGGAAAACGACAACGACTGCTGTCTTATTTAGCAAAGAAAAATAGAGTACGTTATAAAGAATTAATTAGCCGGTTGGATATTCGGGAATCAAAAACTCGTTAA

>lcl|NC_010433.1_cds_YP_001718494.1_79 [gene=ycf1] [locus_tag=MaesCp080] [db_xref=GeneID:5999984] [protein=hypothetical chloroplast RF1] [protein_id=YP_001718494.1] [location=complement(130312..135882)] [gbkey=CDS]

ATGATTTTTCAATCTTTTATACCAGGTAATCTAGTATCCTTATGCATGAAGATAATCAATTCGGTCGTTGTGGTCGGACTCTATTATGGATTTCTGACCACATTCTCCATGGGGCCTTCTTATCTCTTCCTTCTCCGAGCTCGGGTTATAGAAGAAGGAGAAGAAGGAACTGAGAAGAAGGTATCAGCAACAACAGGTTTTATTACGGGACAGCTCATGATGTTCATATCGATCTATTATGCGCCTCTGCATCTAGCATTGGGTAGACCTCATACAATAACTGTCCTAGCTCTACCCTATCTTTTGTTTCATTTCTTCTGGAACAATCACAAACACTTTTTTGATTATGGATCTACTACCAGAAATTCAATGCGTAATCTTAGCATTCAATTTGTATTCCTGAATAATCTCATTTTTCAATTATTCAACCATTTCATTTTACCAAGTTCAATGTTAGTCAGATTAGTCAACATTTATATGTTTCGATGCAACAACAAGATGTTATTTGTAACAAGTAGTTTTGTTGGTTGGTTAATTGGTCACATTTTATTCATGAAATGGGTTGGATTGATATTAGTCTGGATACAGCAAAATAATTCTATTAGATCTAATGTACTTTTTCGATCTAATAAGTACCTTGTGTCAGAATTGAGAAATTCTAGGGCTCGAATCTTTAGTATTCTCTTATTTATTACCTGTGTCTACTCTTTAGGCAGAATACCGTCACCCATTTTTACTAAGAAACTGAAAGAAACCTCAGAAACGGAAGAAAGGGAGGAAGAAACAGATGTAGAAATAGAAAAAACTTCCGAAACGAAGGGGACTAAACAGGAACAAGAGGGATCCACCGAAGAAGATCCTTCTTCTTCCCTTTTTTCGGAAGAAAAGGAGGATCCGGACAAAATCGACGAAACGGAAGAGATCCAAGTGAATGGAAAGGAAAAAACAAAGGATGAATTCCATTTTCACTTTAAAGAGACATGCTATAAAAATAGACCACTTTATGAAACTTTTTATCTGGATGGGAATCAAGAAAATTCGAAGTTAGAAATATTGATAGATAAAAAAAAGAAAGATCTTTTCTGGTTTGAAAAACCTCTTGTAACTATTCTTTTTGACTCTAAACGTTGGAATCGTCCATTTCGATATATAAAAAATGATCAGTTTGAGAATGCTGTAAGAAAAGAAATGTCACAATATTTTTTTTATACATGTCGGAGTGATGGAAAAGAAAGAATATCTTTTACGTATCCACCCAGTTTGTCAACTTTTTTGGAAATGATACAAAGAAAGATATCTCCGTTTACAACAGAAAAACTCTCCTCTGATGAATTGTATAATCGTTGGAATTATAAGAATGAACAAAAAAAGAAAACCCTAAATAATGAATTTATAAACAGAGTCCAGGCTCTAGATAAGGGATATCTTACTCTGAATACACTCGAAAAAAGGGCTGGACTATGTAATGATAAAACTAAAAAAGAGTACTTACCTAAAATTTATGATCCTTTTTTGAGTGGGTCCTGCCGCGGGAAAATCCAATTTTTTTCACCCTCATTCCTAAATATCCTAAATAAAACTTCCATAAAAAATTCCATAGAGATGCTTTGGATAAATAAAATTCATCTTATTCTTCTTATTACTAATTATCAAGAATTTGAATCAAAAACAGATATAGCGAATCCAAAAAATAAAACAATTAGAAAAAATTCTACTGGAATAAAAGAAATAAGTAAACAAGTTCCTCGATGGTCATACAAATTAATTAACGATTTGGAACAACAAGAAAAAAACATACCGCAAGATTCTCAAATGCGTTCACGAAAAACCAAACGATTAGAGATTTTTAATAATAAAAATAAAAAAAAAATATATAAATATATGGATACTTATAATAATACCAAAGATACAAAGAATTCTGATAAAATAAAAATAGAAGATAGAATAAAAATAGAAAAAGCGTCTTTGCTATATTATTCACACCAACCGGACTTTCGCCGATGCATAATAAAAGGATCTGTGCGAGCACAAAGACGCAAAATAACTATTTTTGAACTGTTTCGAGCAAATGTGCATTCTCCCCTTTTTTTGGACAGAATAAACAAATATCTTTTTTTTTTTTTTGATATTGTCGAACTGATAAAAAAAATGTTAAAAACAATGTTTATAAATTGGATGTATAAAAACGCAGAATTCATACTTTCGAATATAAAGAAAAAAACAAAAGAAAGTAAGAAAAAAAAAGAGGACAAAAGAGAAGACAACAAAATAGAAGAAGAAGAAGAAGACAACAAAATAGAAGAAGAAGAAGAAGACAACAAAATAGAAGACAACAAAATAGAAGACAACAAAATAGAAGAACCCACAACAGAGACAATCGATCTGGTAGAAATAGCCGAAGCCTGGGATGATATTCTTTTTGCTCAAATAACAAGAGGTTTTATTTTAGTAACCCAATCCATTTTTAGAAAATATATTCTATTACCTTCATTAATAATAATTAAAAATATCATTCGTATACTTTTTTTAAAAACTCCTGAATGGTCCGAGGATTTCAAAGATTGGAGTAGAGAAATACATGTTAAATGCACCTATAATGGAGTTCAATTATCAGAAAAAGAATTTCCGAAAAACTGGTTAATAGAGGGGATTCAAATAAAGATCCTATTTCCTTTTCGTTTAAAACCTTCGCACAAATCTAAGTTAAAATTCTCTCATAAAGATCAAACGAAAATGAAAAAAAAAGCACAAAAAAATGATTATTTTTTTTTAACAGTTTGGGGAACGGAAGCTGACCTGCCTTTTTCGGCGAGTAGAAAAGAACTTTCACTTTTTAAACCCATCTTTAAAAAACTCAAAAAAAAATTTAGAAAAATGCAAAAAAATGGTTTTCGAGTTATAACAATTTTAGAAGAAAGAAAAAAAATTTTTCTAAATTTCTCAAAAGAAAAAAAAAACTGGATCATACAAAACATTTTTTTTCGAAAAGAAATAATAAACAACCTTTCAAAATCAAAAAGAAATCCAATTCTATTATCTGGATTTAAAGAAGTATATGAATTGAATGAACCTAAAAAAGAAAAAGATTCGATAATCAATAACAATAATGGGACGATTCAAAAATTATCCACCCCAATTCGATCTATGGCTTGGACAAATTATTCACTGACAGAAAAAAAAATGAAAGATCTTTCTGCTAGAAGAAAGATAATCATAAATCAAATAGAAAAAATTAAAAAAGAAAAGGAAAAAAAAATTAGAACCTCGGAAATAAATATAAATATTAGTCCTAACAAAATAAGTTATAATGCTAAAAAATTAAAATCATCAAAAAAGATTTCGCAGATAGTAAAAAAAAGAAATGCTCGATTGGCGCATAAATTCCATTTTTTTATAAAAATTTTGATTGAAAGGATATACATAGATATCTTTTTAGGTATCATTAATATTCCTTTAGGTATCATTAATATTCCAAGGATCAATGCACAACTTTTTCTTGAATCAACAAAAAAAATTATTACTAAATACATTTACAATAATGAAGAAAATCACAAAAAAATTGATAAAACAAATCAAACTACAATTCACTTTATTTCGATTATAAAAAAGTCATGTAATAGTAATGTTGTTGTTATTAATAACAACAACAATTCACAGATTTTTTGTGACATATCCTCCTTGTCACAAGCTTATGTATTTTACAAATTATCACAAATCAAAATTATTAACTTATATAAGTTAAGATCTATCTTTGAATATCATAGCCTTTTTCTGAAGAACGAAATAAAGGATTTTTTTATAGCCCAAGGGCTATTTAATTCCGAATTAAAAGATAAAAATTTTCGAAATTCTGTAATGAATCAATGGAAAAATTGGTTAAGGAGTCATTATCAATATAAATACCATTTATCTCAGATTAGATGGTCTAGATTAACACCACAAAAATGGCGAAATAGAATCAATCAACACCATATGCATATGGTTCAAAATAAAAAATTAAACAAATGGAATTTATATGAAAATGAAAAAGACAGATTAATTCATTACAAAAAAAAAAATGATTTTGAGACAGATTTATTACCGAATCAAAAAGATAATTTTAAAAAACACTATAGATATAATCTTTTAGCATATAAATCTATTAATTATGAAAATAAGAAGGACTTAAATTATCTAGCGGAAGATGATATTATCGATATGGAAAAAAGCGCGGATAGAAAATATTTTGATTGGAGCATTCTCAGTTTTTGTCTTAGAAAGAAGGTTGATATTGAGTCCTGGATCGATACCGGAAGAAAAAATAAAAAAAATACTAAGACTAGGACTAATAAGTATAAAATAATTGATAAAATTGATAAGAAAAAAAAGATTTTTCTTACAATTCACCAAGATCAAGAAGTCAATTCATCCAATCAAAAAAAAAAACCTTTTGACTGGATGGGAATGAATGAAGAAATAAAAAATCGTCTTATATCCGATTTTGAAGTTTGGTTCTTTCAAAAATTTTTGATACTTTACAACACATACAAGATAAAACCATGGGCAATACCCATCAAATTTCTTCTTTTCAATTTTCATTTCTATGAAAATGTTAGTAAAAATAAGAAAATTAACGGGAAAAAAAATAAAAATAGCGATCTTTTTATATCTATATCATCGAATGAAAAAAAAATTATTGAATTAGAGCACGAAGAAAAAGAATACGAAGACCCAGGGGGGGGCTTTGGGTCAGTTTTCCAAAATCAAGAAAAAGATATTGAAGAAGATTATATAGGATTAGATATGAAAAAACATAGAAATGAAAAGCAAAACAAAAGTCATATGGAAGTAGAACTTGATTTCTTCCTAAAACGGTATTTATGTTTTCAATTAGAATGGAATAGTTCTTTAAATAACCAACTAATCGAGAATATCAAATTCTATGGTTTCCTGCTTAAAGTGACAAATCCACGAAAAATTATTATATCTTCTATTCAAAGGCAAGAAATAAATTTGAATATTATGATGGTTCGGAACAGTTTTACTCGTACAGAATTAGTGAAAAGGGGAATATTGATTATCGAACCTGTTCGTCTGTCAGTAAAAAATGATGGACAATTTATTTTGTATCAAATGATAGGTATCTTATTAGTTCATAAAAACAAACAACAAATTAATAAAAAATACAGATATTATGTTGATAAAAAGAATTTTACCGAATTTATTGAAAGACATCAAAGTATAATTGGAAATAGAAAGAAAAATGATTATGATTTACTTGTTCCCGAAAATATTTTATCCCCTAAACGTCGGAGAGAATTAAGAATTCTTTTCAATTTAAAAAATAAAAATGATATTCATATAAATACAGAAATTTTCAACGGCAATAACATAAAAAATTGTAGTCCCATTTTAGGTAAAAGCAAACATTTTGATAGAGATAAAAAGAAACTAATTAAATTACAATTTTTTCTTTGGCCAAATTTTCGATTAGAAGATTTAGCTTGTATGAATCGTTATTGGTTCGATACTAATAATGCCAGTCGGTTCAGTATGGTAAGAATATATATATATCCGCGGTTGAAATTTTGA

>lcl|NC_010433.1_cds_YP_001718495.1_80 [gene=rps7] [locus_tag=MaesCp081] [db_xref=GeneID:6000063] [protein=ribosomal protein S7] [protein_id=YP_001718495.1] [location=147378..147845] [gbkey=CDS]

ATGTCACGTCGAGGTACTGCAGAAGAAAAAACTGCAAAATCCGATCCAATTTATCGTAATCGATTAGTTAACATGTTGGTTAACCGTATTCTGAAACACGGAAAAAAATCATTGGCTTATCAAATTATCTATCGAGCCATGAAAAAGATTCAACAAAAGACAGAAACAAATCCACTATCTGTTTTACGTCAAGCAATACGTGGAGTAACTCCCGATATAGCAGTAAAAGCAAGACGTGTAGGCGGATCGACTCATCAAGTTCCCATTGAAATAGGATCCACACAAGGAAAAGCACTTGCCATTCGTTGGTTATTAGGGGCATCCCGAAAACGTCCGGGTCGAAATATGGCTTTCAAATTAAGTTCCGAATTAGTGGATGCTGCCAAAGGGAGTGGTGATGCCATACGCAAAAAGGAAGAGACTCATAGAATGGCAGAGGCAAATAGAGCTTTTGCACATTTTCGTTAA

>lcl|NC_010433.1_cds_YP_001718496.1_81 [gene=ndhB] [locus_tag=MaesCp082] [db_xref=GeneID:6000058] [protein=NADH dehydrogenase subunit 2] [protein_id=YP_001718496.1] [location=join(148156..148932,149615..150370)] [gbkey=CDS]

ATGATCTGGCATGTACAGAATGAAAACTTCATTCTCGATTCTACGAGAATTTTTATGAAAGCCTTTCATTTGCTTCTCTTCGATGGAAGTTTTATTTTCCCAGAATGTATCCTAATTTTTGGCCTAATTCTTCTTCTGATGATCGATTCAACCTCTGATCAAAAAGATATACCTTGGTTATATTTCATCTCTTCAACAAGTTTAGTAATGAGTATAACGGCCCTATTGTTCCGATGGAGAGAAGAACCTATGATTAGCTTTTCGGGAAATTTCCAAACGAACAATTTCAACGAAATCTTTCAATTTCTTATTTTACTATGTTCAACTCTATGTATTCCTCTATCCGTAGAGTACATTGAATGTACAGAAATGGCTATAACAGAGTTTCTCTTATTCGTATTAACAGCTACTCTAGGAGGAATGTTTTTATGCGGTGCTAACGATTTAATAACTATCTTTGTCGCTCCAGAATGTTTCAGTTTATGCTCCTACCTATTATCTGGATATACCAAGAAAGATGTACGGTCTAATGAGGCTACTACGAAATATTTACTCATGGGTGGGGCAAGCTCTTCTATTCTGGTTCATGCTTTCTCTTGGCTATATGGTTCGTCCGGGGGAGAGATCGAGCTTCAAGAAATAGTGAATGGCCTTATCAATACACAAATGTATAACTCCCCAGGAATTTCAATTGCGCTTATATTCATCACTGTAGGAATTGGGTTCAAGCTTTCCCTAGCCCCTTCTCATCAATGGACTCCTGACGTATACGAAGGATCTCCCACTCCAGTCGTTGCTTTTCTTTCTGTTACTTCGAAAGTAGCTGCTTCAGCTTCAGCCACTCGAATTTTCGATATTCCTTTTTATTTCTCATCAAACGAATGGCATCTTCTTCTGGAAATCCTAGCTATTCTGAGCATGATAGTGGGGAATCTCATTGCTATTACTCAAACAAGCATGAAACGTATGCTTGCATATTCGTCCATAGGTCAAATCGGATATGTAATTATTGGAATAATTGTTGGAGACTCTAATGGTGGATATGCAAGCATGATAACTTATATGCTCTTCTATATCTCCATGAATCTAGGAACTTTTGCTTGTATTGTATTATTTGGTCTACGTACCGGAACTGATAACATTCGAGATTATGCAGGATTATACACGAAAGATCCTTTTTTGGCTCTCTCTTTAGCCCTATGTCTCTTATCCCTAGGAGGTCTTCCTCCACTAGCAGGTTTTTTCGGAAAACTCCATTTATTCTGGTGTGGATGGCAGGCAGGCCTATATTTCTTGGTTTTAATAGGACTCCTTACGAGCGTTGTTTCTATCTACTATTATCTAAAAATAATCAAGTTATTAATGACTGGACGAAACCAAGAAATAACCCCTCACGTGCGAAATTATAGAAGATCCCCTTTAAGATCAAACAATTCCATCGAATTGAGTATGATTGTATGTGTGATAGCATCTACTATACCAGGAATATCAATGAACCCGATTGTTGAAATTGCTCAAGATACCCTTTTTTAG

>lcl|NC_010433.1_cds_YP_001718497.1_82 [gene=rpl23] [locus_tag=MaesCp084] [db_xref=GeneID:5999960] [protein=ribosomal protein L23] [protein_id=YP_001718497.1] [location=159379..159660] [gbkey=CDS]

ATGGATGGAATCAAATATGCAGTATTTACAGACAAAAGTATTCGGTTATTGGGGAAAAATCAATATACTTTTAATGTCGAATCAGGATCAACTAGGACAGAAATAAAGCATTGGGTCGAACTCTTCTTTGGTGTCAAGGTAATAGCTATGAATAGCCATCGACTCCCGGGAAAGGGTAGAAGAATGAGACCTATTATGGGACATACAATGCATTACAGACGTATGATCATTACGCTTCAACCGGGTTATTCTATTCCACCTCTTAGAAAGAAAAGAACTTAA

>lcl|NC_010433.1_cds_YP_001718498.1_83 [gene=rpl2] [locus_tag=MaesCp085] [db_xref=GeneID:6000012] [protein=ribosomal protein L2] [protein_id=YP_001718498.1] [location=join(159679..160077,160751..161185)] [gbkey=CDS]

ATGGCGATACATTTATACAAAACTTCTACCCCGAGCACACGCAATGGAGCCGTAGACAGTCAAGCGAAATCCAATACACGAAATACACGAAAGAATTTGATCTATGGACAGCATCGTTGTGGTAAAGGCCGTAATGCCAGAGGAATCATTACCGCAAGACATAGAGGGGGAGGTCATAAGCGTCTATACCGTAAAATCGATTTTCGACGGAATGAAAAAGACATATATGGTAGAATCGTAACCATAGAATACGACCCTAATCGAAATGCATACATTTGTCTCATACACTATGGGGATGGTGAGAAGAGATATATTTTACATCCCAGAGGGGCTATAATTGGAGATACCATTATTTCTGGTACAGAAGTTCCTATAAAAATGGGAAATGCCCTACCTTTGACCGATATGCCCTTAGGCACGGCCATACATAACATAGAAATCACACTTGGAAAGGGTGGACAATTAGCTAGAGCTGCAGGTGCTGTAGCGAAACTGATTGCAAAAGAGGGGAAATCAGCCACATTAAAATTACCTTCTGGGGAGGTTCGTTTAATATCCAAAAACTGCTCAGCAACAGTCGGACAAGTAGGGAATACTGGGGTGAACCAGAAAAGTTTGGGTAGAGCCGGATCTAAATGTTGGCTAGGTAAGCGTCCTGTAGTAAGAGGAGTAGTTATGAACCCTGTAGACCACCCCCATGGGGGTGGTGAAGGGAGGGCCCCAATTGGTAGAAAAAAACCCGCAACCCCTTGGGGTTATCCTGCACTTGGAAGAAGAAGTAGAAAAAGGAATAAATATAGTGATAATTTGATTCTTCGTCGCCGTAGTAAATAG

**5. *Ricinus communis***

>lcl|NC_016736.1_cds_YP_005090157.1_1 [gene=rps12] [locus_tag=RCOM_ORF00001a] [db_xref=GeneID:11542404] [protein=ribosomal protein S12] [exception=trans-splicing] [protein_id=YP_005090157.1] [location=complement(join(104781..105023,74989..75099))] [gbkey=CDS]

ATGCCAACTATTAAACAACTTATTAGAAACACAAGACAGCCAATCAGAAATATCACCAAATCCCCCGCTCTCGGGGGATGTCCTCAGCGCCGAGGAACATGTACTAGGGTGACTATCACCCCCAAAAAACCAAACTCTGCCTTACGTAAAGTTGCCAGAGTACGATTAACCTCTGGATTTGAAATCACTGCTTATATACCTGGTATTGGCCATAATTCACAAGAACATTCTGTAGTCTTAGTAAGAGGGGGAAGGGTTAAGGATTTACCCGGTGTGAGATATCACATTGTTCGAGGGACCCTAGATGCTGTCGGAGTAAAGGATCGTCAACAAGGGCGTTCTAGTGCGTTGTAG

>lcl|NC_016736.1_cds_YP_005090158.1_2 [gene=psbA] [locus_tag=RCOM_ORF00003] [db_xref=GeneID:11542408] [protein=photosystem II protein D1] [protein_id=YP_005090158.1] [location=complement(641..1702)] [gbkey=CDS]

ATGACTGCAATTTTAGAGAGACGCGAAAGCGAAAGCTTATGGGGTCGTTTCTGTAACTGGATAACCAGCACTGAAAACCGTCTTTACATTGGATGGTTTGGTGTTTTGATGATCCCAACTTTATTGACCGCAACTTCTGTATTTATTATCGCTTTCATTGCTGCCCCTCCGGTAGATATTGATGGTATTCGTGAACCTGTTTCTGGATCTTTACTTTATGGAAACAATATTATTTCTGGTGCCATTATTCCTACTTCTGCAGCTATAGGTTTGCATTTTTACCCAATATGGGAAGCGGCGTCTGTTGATGAATGGTTATACAATGGCGGTCCTTATGAGCTAATTGTTCTACACTTCTTACTTGGTGTAGCTTGTTACATGGGCCGTGAGTGGGAGCTTAGTTTCCGTCTGGGTATGCGCCCTTGGATTGCTGTTGCATATTCAGCTCCTGTTGCAGCTGCTACTGCTGTTTTCTTGATCTATCCAATCGGTCAAGGGAGTTTTTCTGATGGTATGCCTCTAGGAATTTCTGGTACTTTCAACTTTATGATTGTATTCCAGGCTGAACACAACATCCTTATGCACCCATTTCATATGTTAGGCGTAGCTGGTGTATTCGGCGGCTCCCTATTCAGTGCTATGCATGGTTCCTTGGTAACCTCTAGTTTGATCAGGGAAACCACAGAAAATGAATCTGCTAATGAAGGTTATAGATTCGGTCAAGAGGAAGAAACTTATAATATCGTAGCTGCTCATGGTTATTTTGGCCGATTGATCTTCCAATATGCTAGTTTCAACAACTCTCGTTCTTTACACTTCTTCCTAGCTGCTTGGCCTGTAGTAGGTATTTGGTTCACTGCTTTAGGTATTAGCACTATGGCTTTCAACCTAAATGGTTTCAATTTCAACCAATCTGTAGTTGATAGTCAAGGTCGTGTAATTAATACCTGGGCTGATATTATCAACCGTGCTAACCTTGGTATGGAAGTTATGCATGAACGTAATGCTCATAACTTCCCTCTAGACCTAGCTGCTGTCGAAGCTCCATCTACAAATGGATAA

>lcl|NC_016736.1_cds_YP_005090159.1_3 [gene=matK] [locus_tag=RCOM_ORF00005] [db_xref=GeneID:11542412] [protein=maturase K] [protein_id=YP_005090159.1] [location=complement(2387..3907)] [gbkey=CDS]

ATGGAGGAATATCAAAGATATTTAGAACTAGATAGATCTCGAAAAAATAACTTCCTATACCCATTTATCTTTCGGGAGTATATTTATACATTTGCTCATGATCACAGTTTAAATAGATCTACTTTGTTGGAAAATTTAGGTTATGACAATAAATCTAGTCTATTAATTGTAAAACGTTTAATTACTCGAATGTATCAACAGAACCATTTGATTATTTCTGCTAATGATTCTAATCAAAATCCATTTTTTAAGTACAACAAGAATTTATATTATCAAATGATATCAGAGGGCTTTGCAGTTATTGTGGAAATTCCATTTTCCCTACGATTAGTATCTTCTTTAGAAAGGTCAGAGATAGTAAAATCTCATAAATTACGATCAATTCATTCAATATTTCCTTTTTTAGAGGACAAATTTCCACATTTAAATTATGTGTCAGATGTATTAATACCTTACCCCATCCATCTAGAAAAATTGGTTCAAATCCTTCGCTATTGGGTGAAAGATCCCTCTTCTTTGCATTTATTACGACTCTTTCTTCATGAGTATTGGAATTGGAACAGTTTTATTATTCCAAAGAAATCAATTTCTATTTTTACAAAAAGTAATCCAAGATTTTTCGTGTTCCTATATAATTCTCATGTATATGAATATGAATCCCTCTTCTTTTTTCTCCGTAACCAATCCTTTCATTTACGATCAACATTTTCTCGAGTACTTCTTGAACGAATTTTTTTCTATGGAAAAATAGAACATTTTGCGGAAGTCTTTGCTAATGATTTTCAGGCCATCCTATGGTTGTTCAAGGACCCTTTCATGCATTATGTTAGATATCAAGGAAAATCTGTTTTGGCTTCAAAAGATGGGCCTCTTCTGATGAAAAAATGGAAATATTACCTTGTCCATTTATGTCAATGTCATTTTTATGTGTGGTTTCAACCGGAAAAGATCTATATAAATTCATTATCTAAGCATTCTCTCAACTTTTTGGGCTATCTTTCAAATGTACAATTTAATCCTTCGTTGGTACGGAGTCAAATGATAGAAAATTCATTTATAATAGATAAAGATAATACTATGAAGAAACTCGATACAATAGTTCCAATTATTCCTTTAATTAGATCATTGGCAAAAATGAAATTTTGTAACGCAGCAGGACATCCCATTAGTAAACCGACCTGGGCGGATTCGGCAGATTCTGAGATTATCGACCGATTTGTGCGTATATACAGAAATCTTTCTCATTATTATAGCGGATCCTCAAAAAAAACGAATTTGTATCGAATAAAATATATACTTCGACTTTCTTGTGTTAAAACTTTGGCTCGTAAACACAAAAGTAGTGTACGCGCTTTTTTGAAAAGATTAGGTTCGGAATTTTTAGAAGAATTTTTTACGGAGGAAGAACAGATTCTTTTTTTGATCTTCCCAAGAGTTTCTTCTATTTCGCGCAGGTTATATAGAGAACGGATTTGGTATTTGGATATTATTTCTATCAATGATTTGGCCAATCATGAATAG

>lcl|NC_016736.1_cds_YP_005090160.1_4 [gene=rps16] [locus_tag=RCOM_ORF00007] [db_xref=GeneID:11542414] [protein=ribosomal protein S16] [protein_id=YP_005090160.1] [location=complement(5267..5419)] [gbkey=CDS]

GTGGGTTTTTATGATCCTTTAAAAAATCAAACCTATTTAAATGTTCCTACTATTCTATATTTCCTTGAAAAAGGCGCTCAACCTACGGGAACTGTTCATGATATTTTAAGCAAGGCGGGGGTTTTTACGGAACTTCGTCTTAATCAAACGTAA

>lcl|NC_016736.1_cds_YP_005090161.1_5 [gene=psbK] [locus_tag=RCOM_ORF00009] [db_xref=GeneID:11542416] [protein=photosystem II protein K] [protein_id=YP_005090161.1] [location=8059..8244] [gbkey=CDS]

ATGCTTAATATTTTTAGTTTAATTTGTATCTGTCTTAATTCTGCCCTTTATTCAAGCAATTTTTTCTTCACAAAATTGCCCGAAGCCTACGCCTTTTTGAATCCAATCGTAGATGTTATGCCAGTAATCCCTGTACTCTTTTTTCTATTAGCCTTTGTTTGGCAGGCTGCTGTAAGTTTTCGATGA

>lcl|NC_016736.1_cds_YP_005090162.1_6 [gene=psbI] [locus_tag=RCOM_ORF00010] [db_xref=GeneID:11542402] [protein=photosystem II protein I] [protein_id=YP_005090162.1] [location=8679..8789] [gbkey=CDS]

ATGCTTACTCTCAAACTCTTTGTTTACACAGTAGTGATATTCTTTGTTTCTCTATTCATCTTTGGATTTTTATCTAATGATCCAGGACGTAATCCCGGACGTGAAGAATAA

>lcl|NC_016736.1_cds_YP_005090163.1_7 [gene=atpA] [locus_tag=RCOM_ORF00014] [db_xref=GeneID:11542288] [protein=ATP synthase CF1 alpha subunit] [protein_id=YP_005090163.1] [location=complement(11342..12865)] [gbkey=CDS]

ATGGTAACCATTCGAGCCGACGAGATTAGTAATATTATCCGCGAACGTATTGAGCAATATAATAGGGAAGTAAAGATTGTAAATACGGGTACCGTACTTCAAGTAGGCGACGGCATTGCTCGTATTTATGGTCTTGATGAAGTAATGGCAGGTGAATTAGTAGAATTTGAAGAGGGTACAATAGGCATTGCTCTGAATTTGGAATCAAATAATGTCGGTGTCGTTTTAATGGGTGACGGTTTAATGATACAAGAGGGAAGTTCCGTAAAAGCAACAGGAAGGATTGCTCAGATACCGGTGAGTGAGGCTTATTTGGGTCGTGTTATAAATGCCCTGGCTAAACCTATTGACGGTCGAGGTGAAATTTCAGCTTCTGAATCGCGGTTAATTGAATCTCCCGCTCCTGGTATTATTTCGAGACGTTCCGTATACGAGCCTCTTCAAACAGGACTTATTGCTATTGATTCGATGATCCCCATAGGACGCGGTCAGCGGGAATTGATTATTGGGGACAGACAGACCGGTAAAACAGCAGTAGCCACAGATACAATTCTCAATCAACAAGGACAAAATGTAATATGTGTTTATGTAGCTATTGGGCAAAAAGCGTCTTCTGTGGCTCAGGTAGTGACTACTTTACAGGAAAGAGGGGCAATGGAGTACACTATTGTGGTAGCCGAAACGGCGGATTCTCCGGCTACATTACAATACCTCGCTCCTTATACAGGAGCAGCTCTGGCTGAATATTTTATGTACCGTGAACGACACACCTTAATCATTTATGATGATCTCTCCAAACAAGCGCAGGCTTATCGCCAAATGTCGCTTCTATTACGAAGACCACCTGGTCGTGAAGCTTATCCCGGAGATGTCTTTTATTTGCATTCACGCCTTTTGGAAAGAGCTGCTAAATCAAGTTCCCGTTTAGGTGAAGGAAGTATGACTGCTTTACCAATAGTCGAGACCCAATCAGGAGACGTTTCGGCTTATATTCCTACTAATGTAATTTCCATTACAGATGGACAAATATTCTTATCCGCCGATTTATTCAATGCTGGAATCAGGCCTGCTATTAATGTGGGTATTTCCGTTTCCAGAGTAGGATCCGCTGCTCAAATTAAAGCTATGAAACAAGTAGCTGGTAAGTTAAAATTGGAATTGGCGCAATTCGCAGAATTAGAAGCCTTTGCGCAATTCGCTTCGGATCTTGATAAAGCTACTCAGAATCAATTGGCAAGAGGTCAACGATTACGCGAGTTGCTCAAACAATCCCAATCAGCTCCTCTCACGGTGGAGGAACAGATAATGACTATTTATACCGGAACGAATGGTTATCTTGATTCATTAGAAATTGGACAAGTAAGGAAATTTCTCGTTGAGTTACGTACCTACTTAAAAACGAATAAACCTCAGTTCCAAGAAATCATATCTTCTACCAAAACATTCACTGAAGAGGCGGAAACCCTTTTGAAAGAAGCTATTCAGGAACAGAAGGAACGTTTTCTACTTCAGGAACAAGTCTAA

>lcl|NC_016736.1_cds_YP_005090164.1_8 [gene=atpF] [locus_tag=RCOM_ORF00015] [db_xref=GeneID:11542289] [protein=ATP synthase CF0 B subunit] [protein_id=YP_005090164.1] [location=complement(join(12926..13396,14113..14256))] [gbkey=CDS]

ATGAAAAATGTAACCGATTCTTTCGTTTCCTTGGGTCACTGGCCATCCGCCGGGAGTTTCGGATTTAATACCGATATTTTAGCAACAAATCCAATAAATCTAAGTGTAGTCCTTGGTGTATTGATTTTTTTTGGAAAGGGGGTGGTTTGGTTCGGGAAGGGATCATGGAAGTTTTGCAATGAATGGAAAGATAATCTACTTTCATTAACGGATTTATTAGATAATCGAAAACAAAGGATTTTGGATACTATTCGAAATTCAGAAGAACTACGTGAGGGGGCCATTGAACAGCTGGAAAAAGCCCGGGCCCGCTTACGGAAAGTGGAAATAGAAGCAGATCAGTTTCGAATGAATGGATACTCTGAGATAGAACGAGAAAAGTTGAATTTGATTAATTCAACTTATAAAACTTTGAAACAATTAGAAAATTACAAAAATGAAACCATTCATTTTGAACAACAAAGAACGATTAATCAAGTCCGACAACGGGTTTTCCAACAAGCCTTACAGGGAGCTCTAGGAACTCTGAATAGTTGTTTGACCAACGAGTTACATTTACGTACCATCAATGCTAATCTTGGCATGTTTGGGGCGATAAAAGAAATAACTGATTAG

>lcl|NC_016736.1_cds_YP_005090165.1_9 [gene=atpH] [locus_tag=RCOM_ORF00016] [db_xref=GeneID:11542290] [protein=ATP synthase CF0 C subunit] [protein_id=YP_005090165.1] [location=complement(14784..15029)] [gbkey=CDS]

ATGAATCCATTGATTTCTGCCGCTTCCGTTATTGCTGCGGGTTTGGCCGTTGGGCTTGCTTCTATTGGACCTGGGGTTGGTCAAGGTACTGCCGCGGGCCAAGCTGTAGAGGGTATCGCAAGACAACCTGAGGCGGAGGGAAAAATACGAGGTACTTTATTGCTTAGTCTGGCTTTTATGGAAGCTTTAACAATTTATGGACTAGTTGTAGCATTAGCACTTTTATTTGCGAATCCTTTTGTTTAA

>lcl|NC_016736.1_cds_YP_005090166.1_10 [gene=atpI] [locus_tag=RCOM_ORF00017] [db_xref=GeneID:11542291] [protein=ATP synthase CF0 A subunit] [protein_id=YP_005090166.1] [location=complement(16029..16715)] [gbkey=CDS]

GTGGAAGTAGGCCAGCATTTCTATTGGAAAATAGGAGGTTTCCAAGTCCATGCCCAAGTACTTATTACTTCTTGGGTTGTAATTGCTATCTTATTAGGTTCCGCCATTGTAGCTGTTCGGAACCCACAAACCATTCCGACTGGCGGTCAGAATTTCTTCGAATATGTTCTTGAATTCATTCGAGATGTGAGCAAAACTCAGATTGGAGAGGAATACGGTCCATGGGTCCCCTTTATTGGAACTATGTTTCTATTTATTTTTGTTTCTAATTGGGCGGGGGCGCTTTTACCTTGGAAGATCATAGAATTACCTCATGGGGAGTTAGCCGCACCTACGAATGATATAAATACTACCGTTGCTTTAGCTTTACTTACGTCAATAGCATATTTTTATGCGGGCCTTAGCAAAAAAGGATTAGGTTATTTCGGTAAATACATTCAACCAACTCCAATCCTTTTACCCATTAACATTTTAGAAGATTTCACAAAACCTTTATCGCTTAGCTTTCGACTTTTCGGAAATATATTAGCGGATGAATTAGTAGTTGTTGTTCTTGTTTCTTTAGTACCTTTAGTGGTTCCTATACCTGTCATGTTCCTTGGATTATTTACAAGTGGTATTCAAGCTCTTATTTTTGCAACTTTAGCTGCGGCTTATATAGGCGAATCCATGGAAGGGCATCATTGA

>lcl|NC_016736.1_cds_YP_005090167.1_11 [gene=rps2] [locus_tag=RCOM_ORF00018] [db_xref=GeneID:11542292] [protein=ribosomal protein S2] [protein_id=YP_005090167.1] [location=complement(17032..17742)] [gbkey=CDS]

ATGATAAGAAGATATTGGAACATTAATTTGGAAGAGATGATGAAAGCAGGAGTTCATTTTGGTCATGGTACTAGAAAATGGAATCCGAGAATGGCACCTTATATCTCTGCAAAGCGTAAGGGTATTCATATTACAAATCTTACTAGAACTGCTCGTTTTTTATCAGAAGCTTGTGATTTAGTTTTTGATGCAGCAAGTAGGAGAAAACAATTCTTAATTGTTGGTACCAAAAATAAAGCGGCGGATTCAGTAGCACGAGCTGCAATAAGGGCTCGGTGTCATTATGTTAATAAAAAATGGCTCGGCGGTATTTTAACGAATTGGTCCACTACAGAAACTAGACTTCAAAAGTTCAGGGACTTGAGAATGGAACAAAAGACAGGTAGACTCAACCGTCTTCCGAAAGGAGATGCGGCTCGGTTGAAGAGACAGTTAGCTCACTTGCAAACATATCTGGGTGGGATTAAATATATGACGGGGTTACCCGATATTGTAATAATCGTTGATCAGCAAGAAGACTATACGGCTCTTCGGGAATGTATCACTTTGGGAATCCCAACGATTTGTTTAATTGATACAAACTGTGACCCGGATCTCGCAGATATTTCGATTCCAGCGAATGATGACGCTATAGCTTCAATCCGACTAATTCTTAATAAATTAGTATTTGCAATTTGTGAGGGTCGTTCTAGCTATATACGAAATCCCTGA

>lcl|NC_016736.1_cds_YP_005090168.1_12 [gene=rpoC2] [locus_tag=RCOM_ORF00019] [db_xref=GeneID:11542293] [protein=RNA polymerase beta subunit] [protein_id=YP_005090168.1] [location=complement(17988..22205)] [gbkey=CDS]

ATGGAGGTACTTATGGCCAAACGGGCCAATCTGGTCTTTCACAATAAAGTGATAGATGGAACTGCCATTAAACGACTTATTAGCAGATTAATAGATCATTTTGGAATGGCATATACATCACACATCCTGGATCAAGTAAAGACTCTGGGTTTCCAGCAAGCCACTGCTACATCCATTTCATTAGGAATTGATGATCTTTTAACAATACCTTCTAAGGGATGGCTAGTTCAAGATGCTGAACAACAAAGTTTGATTTTGGAAAAACACTATCATTATGGAAATGTACACGCGGTAGAAAAATTACGCCAATCTATTGAGATATGGTATGCTACAAGTGAATATTTGCGACAAGAAATGAATCTTAATTTTAGGATGACGGAACCCTTTAATCCAGTCCATATAATGTCTTTTTCGGGAGCTAGGGGAAATGTATCTCAAGTACACCAATTAGTAGGTATGAGAGGATTAATGTCGGATCCACAAGGACAAATGATCGATTTACCTATTCAAAGCAATTTACGCGAAGGACTATCTTTAACAGAATATATCATTTCTTGCTACGGAGCCCGCAAAGGGGTTGTCGATACCGCTGTACGAACATCAGATGCTGGATATCTTACGCGTAGACTTGTTGAAGTAGTTCAACACATTGTTGTACGTAGAACAGATTGTGGCACCACCCGAGGGATTTCTGTGAGTCCTCAAAATGGGATGATGTCGGAAAGAATTTTTATTCAAACATTAATTGGTCGTGTATTAGCAGACAATATATATATGGGTCTACGATGCATTGCCATTCGAAATCAAGATATTGGGATTAGACTTGCCAATCGATTCATAACCTTTCGAACACAAACAATATCTATTCGAACTCCCTTTACTTGTAGGAGTACGTCTTGGATCTGTCGATTATGTTATGGCCGGAGTCCTACTCATGGCGATCTAGTGGAATTGGGGGAAGCCGTAGGTATTATTGCAGGTCAATCCATTGGAGAGCCGGGTACTCAACTAACATTAAGAACATTTCATACCGGCGGAGTATTCACAGGGGGTACTGCAGAACATGTACGAGCCCCCTCTAATGGAAAAATAAAATTTAACGAGGATTTGGTTCATCCCATACGTACACGTCATGGGCATCCTGCTTTTCTATGTTATATAGACTTGTATGTAACTATTGAAAGTCACGATATTATACATAACGCTACTATTCCACCAAAGAGTTTCCTTTTAGTTCAAAACAATCAATATGTAGAATCAGAACAAGTGATTGCTGAAATTCGGGCGGGAACATACACTTTGAATTTTAAAGAGAAGGTTCGAAAACATATTTATTCCGATTCAGAAGGGGAAATGCACTGGAGTACTGATGTATACCATGCACCTGAATTTACATATAGTAATGTCCATCTCTTACCAAAAACAAGTCATTTATGGATATTATCAGGAAATTCGTGCAGATCCAGTATAGTTCCTTTTTCGCTCCATAAGGATCAAGATCAAATGAACGTTCATTCTCTTTCTATCAAAAGAAGATATATTTCTAGTCCTTCCGTAAATTCCGTAAATAATGATCAAGTTAAACCCAAATTCTTTAGTTCAGATTTTTCGGGTAAAAAACCAAGTAGGATTCCTTATTATTCAGAACTTAATCGAATCGTATGTACTGGTCATTGTAATCTCATATATCCCGCGATTCTCTACGAGAATTCTGATTTATTGGCAAAGAGGCGAAGAAATAAATTCATCATCCCATTCCAATCAATTCAAGAACAAGAAAAAAAACTAATGACCCGCTCCTCGGCTATCTCGATTGAAATACCGCTAAATGGTATTTTCCGTAGAAATAGTGTTTTTGCTTATTTTGACGATCCCCAATACCGAAGAAAGAGTTCGGGAATTACTAAATATGGGGCTATAGGGGTGCATTCAATCGTCAAAAAAGAAGATTTGATTGAGTATCGGGGAGTCAAAGAATTTAAGCCAAAATACCAAATGAAGGTAGATCGCTTTTTTTTCATTCCCGAAGAAGTGTATATTTTACCCGAATCTTCTTCCCTAATGGTACGGAACAATAGTATCATTGGAGTAGATACACCAATCACTTTAAATACAAGAAGTCGGGTGGGCGGATTGGTCCGAGTGGAGAGAAAAAAAAAAAAAATTGAGCTTAAAATCTTTTCTGGAGATATCCATTTTCCGGGAGAGACAGATAAAATATCCCGACACAGTGGTATCTTGATACCACCAGGAATGGTAAAAACAAATTCTAAGGAATCAAAAAAACAGAAAAACTGGATCTATATCCAACGAATCGCACCTACCAGGAAAAAGTATTTTGTTTTGGTTCGACTAGTAATCATATATGAGATAGCGAACGGTATAAATTTAGAAACACTTTTCCCCCGGGATTTATTGCAGGAAAAGGATAATCTGAAACTTCGAGTTGTCAATTATATTCTTTCTGGAAATGGTAAACCGATTCGAGGAATTTCTGACACAAGTATTCAATTAGTTCGTACTTGTTTAGTGTTGAATTGGGACCAAGAAAAAAAAAGTTCTTCTATCGAAGAGGCCCGCGCTTCCTTTGTTGAAGTAAACACAAATGGTCTGATTCGTGATTTCTTAAGAATCAACCTAGTGAAATCCCATATTTCATATATCAGTAGAAAAAGGAATGATCCATCGGGTTCAGGGCCGATCTCTAATAATGGGGCAAATCACACCAATATTAATCCATTTTATCCCATCTATTTCAAGACAAGGATTCAACAATCACTTAAACAAAATCAAGGAACTATTAGTACGTTGTTGAATAGAAATAAGGAATGTCAATCTTTGATAATTTTGTCATCATCTAATTGTTTTCGAATGGATCCCTTCAACGATGTAAAACATCACAATGTAATAAAAGAATCAATTAAAAGAGATCCTATAATTCCAATTAGAAATTCGTTGGGCCCTTTAGGAACAGCCCTTCAAATTGCGAATTTGTATTTATTTTACCATTTAAATTTAATAACTCATAATCGGATCTCAGTAACTAAATATTTGAAACTTGACAATTTAAAACAGACTTTTCGAGTACTTAAATATTATTTAATGGACGAAAACGGGAGAGTTGTTAATCCCGATCCATGCAGTAACAGTGTTTTGAATCCATTCAATTTGAATTGGTATTTTCTCCATCATAATTATCATCATAATTATTGTCATAATTATTGTGAAGAAAGCTTCACAATAATTAGCCTGGGACAGTTTATTTGCGAAAATGTCTGTATGGCCAAAAACGGACCACATCTAAAATCGGGTCAAGTTATAATTATTCACATTGGCTCTGTAGTAATAAGATCCGCTAAGCCTTATTTGGCCACTCCGGGAGCAACCGTTCATGGCCATTATGGAGAAATCCTTTACGAAGGAGATACATTAGTTACATTTATATATGAAAAGTCGAGATCTGGTGATATAACGCAGGGTCTTCCAAAAGTAGAACAAGTGTTAGAAGTGCGTTCAATTGATTCAATATCGATAAACCTAGAAAAGAGAGTTGGGGGTTGGAACGAATGTATACCAAGAATTCTTGGAATTCCTTGGGGATTCTTGATTGGTACTGAGCTAACTATAGTGCAAAGTCGCATCTCTTTGGTTAATAAGATCCAAAGGGTTTATCGATCCCAAGGGGTGCAGATCCATAATAGGCATATAGAAATTATTGTACGTCAAATAACATCAAAAGTATTGGTTTCAGAAGACGGAATGTCTAATGTTTTTTCACCCGGAGAACTAATTGGATTGTTGCGAGCGGAACGAACGGGACGTGCTTTGGAAGAAGCCATCTGTTACCGAGCCATATTATTGGGAATAACGAGAGCATCCCTGAATACTCAAAGTTTCATATCCGAGGCCAGTTTTCAAGAAACTGCTCGTGTTTTAGCAAAAGCCGCTCTCCGCGGTCGTATCGATTGGTTGAAAGGCCTGAAAGAAAACGTTGTTCTAGGCGGTATGATACCCGTTGGTACCGGATTCAAAGGATTAGTACAAGGTTCAAGGCAATATAAGAACATTCCTTTGAAAACCAAAAAGAATAATTTATTCGGGGGGGAATTTAGAGATAGAGATATTTTATTCCACCACAGAGAGTTATTTTATTCTTGCATTTCAAAAAATTTCTACGATACATCAGAACAATCATTTATAGGATTTAATGATTCCTAA

>lcl|NC_016736.1_cds_YP_005090169.1_13 [gene=rpoC1] [locus_tag=RCOM_ORF00020] [db_xref=GeneID:11542294] [protein=RNA polymerase beta subunit] [protein_id=YP_005090169.1] [location=complement(join(22374..23903,24750..25184))] [gbkey=CDS]

ATGATTGATCGGTATAAACATCAACAACTCCGAATTGGATCAGTTTCGCCTCAACAAATAAGTGCTTGGGCCAAAAAAATCCTACCTAATGGGGAGATTGTTGGAGAAGTGACAAAACCCTATACTTTTCATTACAAAACCAATAAACCTGAAAAAGGTGGATTGTTTTGTGAAAGAATTTTTGGGCCTATAAAAAGTGGAATTTGTGCTTGTGGAAATTATCGAGTAATCAGAAATGAAAAAGAAGACCAAAAATTTTGTGAACAATGCGGAGTCGAATTTGTTGATTCTCGGATACGAAGATATCAAATGGGCTACATCAAACTGGCATGCCCAGTAACTCATGTGTGGTATTTGAAACGTCTTCCTAGTTATATCGCAAATCTTTTAGATAAACCTCTTAAAGAATTAGAAGGCCTAGTATATTGCGATGTGAAATACAGTATTCCACTTTTTTTTACTGCCCAAGGCTTCGATACATTTCGAAATCGAGAAATTTCTACAGGAGCTGGTGCTATCCGAGAACAATTAGCCGATCTAGATTTGCGAATTATTATAGATTATTCATCGGTAGAATGGAAAGAATTAGGGGAAGAAGGGCCTACGGGGAATGAATGGGAAGATCGAAAAGTTGGAAGAAGAAAGGATTTTTTGGTTCGACGCGTGGAATTAGCTAAGCATTTTATTCGAACAAATATAGAACCAGAATGGATGGTTTTATGTCTATTACCAGTTCTTCCTCCCGAGTTGAGACCGATCATTCAGATAGATGGGGGTAAACTAATGAGTTCAGATATTAATGAACTCTATAGAAGAGTTATCTATCGGAACAATACTCTTATTGATCTATTAACAACAAGTAGATCTACGCCAGGAGAATTAGTAATGTGTCAGGAAAAATTAGTACAAGAAGCCGTGGATACACTTCTTGATAATGGAATCCGCGGACAACCAATGAGAGACGGTCATAATAAGGTTTACAAGTCGTTTTCGGATGTAATTGAAGGCAAAGAAGGAAGATTTCGTGAGACTATGCTTGGAAAACGGGTTGATTATTCGGGGCGTTCTGTCATTGTCGTAGGCCCCTCACTTTCATTACATCGATGTGGATTGCCTCGCGAAATAGCAATAGAACTTTTCCAGATATTTGTAATTCGTGGTCTAATTAGACAACATCTTGCTTCGAACATAGGAGTTGCTAAGAGTAAAATTCGGGAAAAAGAGCCAATTGTATGGGAAATACTTCAGGAAGTTATGCAGGGGCATCCGGTATTACTGAATAGAGCGCCGACTTTGCATAGATTAGGCATACAGGCATTCCAACCCATTTTAGTGGAAGGCCGCGCTATTTGTTTACATCCATTAGTTTGTAAGGGATTCAATGCAGACTTTGATGGGGATCAAATGGCTGTTCATGTACCTTTATCGTTGGAGGCTCAAGCGGAGGCTCGTTTACTTATGTTTTCTCATATGAATCTCTTATCTCCAGCTATTGGAGATCCCATTTCCGTACCAACTCAAGATATGCTTATTGGGCTCTATGTATTAACAAGCAGGAATCGCCGAGGTATTTGTGCAAATAGGTATAATCCATGTAATCACAGAAATTATCAAAATGAAAGAATTTACGATAATAACAATCAATATACGAAAGAATCCTTTTTTTCTAATTCCTATGATGCAATTGGTGCTTATCGGCAGAAAAGAATCAATTTAGATAGTCCTTTGTGGCTCCGTTGGCAACTAGATCAACGCGCTATTGCTTCAAGAGAAGCTCCCGTCGAAGTTCACTATGAATCTTTGGGTACCTATCATGAGATTTATGAACACTATCTAATAGTAAGAAATATAAAAAAAGAAATTCTTTGTATATACATTCGAACTACTGTTGGTCATATTTCTCTTTATCGAGAAATCGAAGAAGCTATACAAGGGTTTTGCCAAGCCGGCTCAGATGGTATCTAA

>lcl|NC_016736.1_cds_YP_005090170.1_14 [gene=rpoB] [locus_tag=RCOM_ORF00021] [db_xref=GeneID:11542295] [protein=RNA polymerase beta subunit] [protein_id=YP_005090170.1] [location=complement(25211..28423)] [gbkey=CDS]
[truncated: 179,014 more chars]
