# Supplementary material for: Comparative analysis of codon usage patterns in chloroplast genomes of six Euphorbiaceae species
Source: PeerJ. 2020 Jan 6;8:e8251. doi: 10.7717/peerj.8251 (PMC6951282; doi:10.7717/peerj.8251)
Supplement: Supplemental Information 2 [file peerj-08-8251-s002.docx]

1. *Euphorbia esula*

>lcl|NC_033910.1_cds_YP_009348416.1_85 [gene=rpl2] [locus_tag=B2L33_pgp002] [db_xref=GeneID:31082851] [protein=ribosomal protein L2] [protein_id=YP_009348416.1] [location=join(158986..159381,160006..160473)] [gbkey=CDS]
ATGGCGATACATTTATACAAAACTTCTACCCCGAGCACACGCAATGGAGCCGTAGACAGTCAAGTGAAATCCAATACACGAAATAATTTGATCTATGGACAGCATCATTGTGGTAAAGGCCGTAATGCCAGAGGAATAATTACCGCAAGGCATAGAGGGGGAGGTCATAAGCGTCTATACCGTAAAATCGATTTTCGACGGAATGAAAAAGACATATATGGTAGAATCGTAACCATAGAATACGACCCTAATCGAAATGCATACATTTGTCTCATACACTATGGGGATGGTGAGAAGAGATATATTTTACATCCCAGAGGGGCTATAATTGGAGATACCATTATTTCTGGTACAGAAGTTCCTATAAAAATGGGAAATGCCCTACCTTTGAGTGCGGTTTTGATTGATCAAAAAGAAGAATCTACTTCAACCGATATGCCCTTAGGCACGGCCATACATAACATAGAAATCACACTTGGAAAGGGTGGACAATTAGCTAGAGCTGCAGGTGCTGTAGCGAAACTGATTGCAAAAGAGGGTAAATCGGCCACATTAAAATTACCTTCTGGGGAGGTTCGTTTAATATCCAAAAACTGCTCAGCAACAGTCGGACAAGTAGGGAATACTGGGGTGAACCAGAAAAGTTTGGGTAGAGCCGGGTCTAAATGTTGGCTAGGTAAGCGTCCTGTAGTAAGAGGAGTGGTTATGAACCCTGTAGACCATCCCCATGGGGGTGGTGAAGGGAGGGCCCCAATTGGTAGAAAAAAACCCGCAACCCCTTGGGGTTATCCTGCACTTGGAAGAAGAAGTAGAAAAAGGAATAAATATAGTGATAATTTGATTCTTCGTCGCCGTAGTAAATAG

>lcl|NC_033910.1_cds_YP_009348335.1_4 [gene=matK] [locus_tag=B2L33_pgp084] [db_xref=GeneID:31082726] [protein=maturase K] [protein_id=YP_009348335.1] [location=complement(2775..4286)] [gbkey=CDS]
ATGCAAAGATATTTCGACCTAAATAGATCTCGAAAAAGCGATTTCCTATACCCATTTATCTTTCGGGAGTATATTTATACATTTGCTCATGATCATAGTTTAAATAGATCTATTTTGTTGCAAAATGCAAGTTATGACAAAAAGTTGAGTTTATTAATTGTAAAACGTTTAATTACTCGAATCTATGAACAGAATCATTTGATTATTTCTGCTAATGATTACAACCAAAATCTGTTTTTTAGGTATAACAAGAATTTGTACTATCAAATGGTATCAGAGGGCTTCGCAGTTATTGTGGAAATTCCATTTTCCAGACGATTAGTATCTTCTTTAGAAAGGCCAGAGATAGTAAAATCTAATAAATTACGATCAATTCATTCAATATTTCCTTTTTTAGAGGACAAGTTTCCACATTTAAATTATGTGTCAGATGTATTAATACCTTACCCCATCCATCTCGAAAAATTGGTTCAAACCCTTCGTTATTGGGTGAAAGATCCTTCTTCTTTACATTTTTTACGAGTCTTTCTTCATCAGTATTCGAATTTGAGCAGTCTTATTATTCCAAAGAAATCAATTTCTTTTTTTCGAAAAAGTAATCCAAGGTTTTTCTTGTTCCTATATAATTCTCATATAAATGAATATGAATCTATCTTATTTTTTCTCCGTAATCAGTCCTTTCATTTACGATCAACATTTTCTCGAGTTTTTCTTGAACGAATTTTTTTCTATGGAAAAATAGAACATTGTGCAGAAGTTTTTGCTAATGATTTTCAGAACATTCTAGTGTTGTTCAAGGATCCTTTCATGCATTATGTTAGATATCACGGAAAATCTATTCTCGCTTTAAAAGATAAACCCTTTCTGATGAAAAAATGGCAATATTACCTTATCAATTTATGTCAATGTCATTTTTATGTCTGGTTTCACCCAAAAAAGATCTATATAAATTCATTATCAAAAAATTCTCTCAACTTTTTTGGCTATCTTTCAAGTGTACAAAAGAATCCTTTGGTAGTACGGAGTCAAATGCTAGAAAATTCATATCTCATAGATAAAGAGAATACTATGAAGAACCTCGATACAATAATTCCAATTAATCCTTTAATTGTATTATTGTCAAAAATGCAATTTTGTAACGCAGTGGGACATCCTATTAGTAAACCGATTCGGGCTCATTTATCAGATTCTGATATTATCGACCAATTTGCGCGTATATGTCGAAATTTTTCTCATTTTTATAGCGGATCCTCAAAAAAAAAGAGTTTGTATCGTATAAAATATATACTTCGACTTTCGTGTGTTAAAACTTTGGCCCGTAAACACAAAAGTACTGTACGCGCTTTTTTGAAAAGATTAGGTTCGGAATTATTAGAAGAATTTTTTACGGAGGAAGAAGAGATTCTTTCTTTGATCTTCCCAAAAGTTTCTTCTATTTCGCGCAAGTTATATAGAAGACGAATTTGGTATTTGGATATTATTTCTATCAATGATTTGGCACATCATGAATAA

>lcl|NC_033910.1_cds_YP_009348384.1_53 [gene=petD] [locus_tag=B2L33_pgp034] [db_xref=GeneID:31082819] [protein=PetD] [protein_id=YP_009348384.1] [location=83341..83886] [gbkey=CDS]
ATGTCCGGTTCCTTCGGAGGATGGATCTATAAGAATTCACCTATCCCAATAACAAAAAAACCTGACTTGAATGATCCTGTATTAAGAGCTAAATTGGCTAAGGGAATGGGTCATAATTATTATGGGGAACCCGCATGGCCAAACGATCTTTTATATATTTTTCCAGTAGTAATTTTAGGTACTATTGCATGTAATGTAGGATTAGCGGTTCTAGAACCATCAATGATTGGTGAACCTGCAGATCCATTTGCAACTCCTTTGGAAATATTGCCTGAATGGTATTTCTTTCCTGTATTTCAAATACTCCGTACAGTACCCAATAAGTTATTGGGTGTTCTTTTAATGGTTTCAGTACCTACAGGCTTATTAACAGTACCTTTTTTAGAGAATGTTAATAAATTCCAAAATCCATTTCGTCGTCCAGTTGCGACAACTGTATTTTTGATTGGTACTGTAGTAGCCGTTTGGTTAGGTATTGGAGCAACATTACCTATTGAGAAATCTCTAACTTTAGGTCTTTTTCAAGTTGATTTAATTGAAAAATAA

>lcl|NC_033910.1_cds_YP_009348378.1_47 [gene=clpP] [locus_tag=B2L33_pgp040] [db_xref=GeneID:31082723] [protein=ClpP] [protein_id=YP_009348378.1] [location=complement(join(75957..76185,76839..77129,77951..78021))] [gbkey=CDS]
ATGCCTATTGGTGTTCCAAAAGTCCCTTTTCGACATCCTGGAGAAGACGATTCACTTTGGATTGACTTATACAACCGACTTTATCGAGAAAGATTACTTTTTTTAGGTCAAGGTATTGATAGCGAGATCTCGAATCAACTTATTGGTCTTATGGTATATCTCAGTATAGAGAGCGAGACCAAAGATTTGTATTTGTTTATAAACTCTCCTGGCGGATGGGTAATACCCGGAGTAGCTATTTATGATACTATGCAATTTGTGCGACCAGATGTACAAACAGTATGCATGGGATTAGCTGCTTCAATGGGATCTTTTATTCTGGTCGGAGGAAAAATCACCAAACGTTTAGCATTCCCTCATGCCAGGGTAATGATTCATCAACCTATTGCTGGTTTTTATGAAGCACAAATAGCAGAATTTGTCCTGGAAGCAGAAGAACTGCTGAAACTGCGTGAAATCCTCACAAGGATTTATGCACAAAGAACGGGAAAACCCTTATGGGTTGTATCTGAAGACATGGAAAGAGATGTTTTTATGTCAGCAACAGAAGCCCAAGCTCACGGAATTGTTGATCTTGTAGCAGTTGCATAA

>lcl|NC_033910.1_cds_YP_009348395.1_64 [gene=ycf2] [locus_tag=B2L33_pgp023] [db_xref=GeneID:31082749] [protein=Ycf2] [protein_id=YP_009348395.1] [location=92957..99847] [gbkey=CDS]
ATGAAGGGACATCAATTCAAATCCTGGATTTTCGAATTGAGAGAGATATTGAGAGAGATCAAGAATTCTCACTATTTCTTCGATTCATGGACCCAATTCAATTCAGTGGGATCTTTCATTCACATTTTTTTCCATCAAGAACGTTTTATAAAACTCTTGGACTCCCGAATTTGGAGTATCTTACTTTCACGCAATTCACAGGGTTCAACAAGCAATCGATATTTCACGATCAAGGGTGTAGTACTCTTTGTAGTAGTGGTCCTTATATATCGTATTAACAATCGAAAGATGGTCGAAAGAAAAAATCTCTATTTGACAGGGCTTCTTCCTATACCTATGAATTCCATTGGACCCAGAAATGATACATTGGAAGAATTCTTTGGGTCTTACAATATCAATAGGTTGATTGTTTCGCTCCTGTATCTTCCAAAAGGAAAAAAGATCTCTGAGAGCTCTTTCCCGGATCTGAAAGAGAGTACTTGGGTTCTCCCAATAACTAAAAAGTGTATCATGTCTGAATCTAACTGGGGTTCACGGCGGTGGAGGAACTGGATCGGAAAAAAGAGGGATTCTAGTTGTAAGATATCTAATGAAACCGTCGCTGGAATTGAGATCTCATTCAAAGAAAAAGATATCAAATATCTGGAGTTTCTTTTTGTATATTATATGGATGATCCGATCCGCAAGGACCAGGATTGGGATTTGTTTGATCGTCTTTCTCCGAGGAAGGGGCGAAACAGAATCAACTTGAATTCGGGACAGCTATTGGAAATCTTAGTGAAAGACTGGATTTGTTATCTCATGTTTGCTTTTCGTGAAAAAATACCAATTGAAGTGGAGGGTTTCTTCAAACAACAAGGAGCTGGGTCAACTATTCAATCAAATGATATTGAGCATGTTTCCCATCTCTTCTCGAGAAAGAAGTGGGCTATTTCTTTGCAAAATTGTGCTCAATTTCATATGTGGCAATTCCGCCAAGATCTCTTCGTTAGTTGGGGGAATAATCCGCACGAATCGGATTTTTTGAGGAACATATCGAGAGAGAATTGGATTTGGTTAGACAATGTGTGGTTGGTAAACAAGGATCGGTTTTTTAGCAAGGCACGGAATATATCGTCAAATATTCAATATGATTCCACAAGATCTAGTTTCGTTCAAGGAAGGAATTCTAGCCAATTGAAGGGATCTTCTGATCAATCCAGAGATCATTTCGATTCCATTAGTAATGAGGATTCGGAATATCACACATTGATCAATCAAAGAAAGATTCAACAACTAAAAGAAAGATCGATTCTTTGGGATCCTTCCTTTCTTCAAACGGAACGAACAGAGATAGAATCAGACCAATTCCCTAAATGCCTTTCTGGATATTCCTCAATGTCCCGGCTATTCAGGGAAGGTGAGAAGGAGATGAATAATCATCTGCTTCCGGAAGAAATCGAAGAATTTCTTGGGAATCCTACAAGATCCATTCGTTCTTTTTTCTCTGACAGATCGTCAGAACTTCATCTGGGTTCGAATCCTACTGAGAGATCCACTAGAGATCAGAAATTGTTGAAGAAAGAACAAGATGGTTCTTTTGTCCCTTCCAGGCGATCGGAAAATAAAGAAATAGTTAATATATTCAAGATAATCACGTATTTACAAAATACCGTCTCAATTCATCCATCCGATCCGGGATGTGATATGGTTCTGAAGGATGAACTGGATATGGACAGTTCCAATAAGATTTCTTTCTTGAACAAAAATCCATTTTTTGATTTATTTCATCTATTCCATGATCGGAACGGGGGGGGGTACACGTTACACCACGATTTTGAATCAGAAGAGAGATTTCAAGAAATGGCAGATCTATTCACTCTATCAATAACCGAGCCGGATCTGGTGTATCATAAGGGATTTACCCGTTTTATTGATTCCTACGGATTGGATCAAAAACAATTCTTGAATGAGGTATTCAACTCCAGGGATGAATCGAAAAAGAAATCTTTATTGGTTCTACCTCCTATTTTTTATGAAGAGAATGAATCTTTTTATCGAAGGATCAGAAAAAAATGGGTCCGGATCTCCTGCGGGAATGATTTGGAAGATCCAAAACAAAAAACAGTGGTATTTGCTAGCAACAACATAATGGAGGCAGTCAATCAATATGGATTGATCCTAAATCTGATTCAAATCCAATATAGTACCTATGGGTACATAAGAAATGTATTGACTCAATTCTTTTTAATGAATAGATCCGATCGCAACTTCGAATATGGAATTCAAAGGGATCAAATAGGAAATGATACTCTGAATCATAGAACTATAATGAAATATACGATCAACCAACATTTATCGAATTTGAAACAGAGTCAGAAGAAATGGTTCGATCCTCTTATTTTTCGTTCTCGAACCGAGAGATCCATGAATTGGGATCCTAATGCATATAGATACAAATGGTCTAATGAGAGCAAGAATTTCCAGGAACATTTGGAACATTTCATTTCTGAGCAGAAGAGCCGTTTTCTTTTTCAAGTAGTGTTCGATCGATTACGTATTAATCAATATTCGATTGATTGGTCTGAGGTTATCGACAAAAAAGATTTGTCTAAGTCACTTCCTTTCTTTTTGTCCAAGTTACTTCTTTTTTTGTCCAAGTTTCTTCTCTTTTTGTCTAACTCACTTCCTTTTTTCTTTGTGAGTTTCGGGAATATCCCCATTCATAGGTCCGAAATCCATATCTATGAATTGAAAGGTCAGAATGATCAACTCTGCAATCAGCTGTTAGAACCAATAGGTCTTCAAATCGTTCATTTGAAAAAATTGAAACCCTTCTTATTGGATGATCATGATACTTCCCAAAAATCGAAATTTTTAATTAATATTAATGGAGGAACAATATCACCATTTTTGTTCAATAAGATACCAAAGTGGATGATTGACTCATTCCATACTAGAACTAATCGCAGGAAATCTTTTGATAACACGGATTCCTATTTCTCAATCATATCCCACGATCAAGACAATTGGCTGAATCCCGTGAAACCATTTCATAGAAGTTCATTGATATCTTCTTTTTATAAAGCAAATCGACTTCGATTCTTGAATAATCTACATCACTTCTGCTTCTATTGTAACAAAAGATTCCCTTTTTATGTGGAAAAGGCCCGTATCAAGAATTCTGATTTTACGTATGGACAATTCCTCAATATCTTGTTCATTCGCAACAAAATATTTTCTTTGTGCGGCGGTAAAAAAAAACATGCTTTTTTGGAGAGAGATACTATTTCACCAATCGAGTCCCAGGTATCTAACATATTCATACCTAATGATTTTCCACAAAGTGGTAACGAAAGGTATAACTTGTACAAATCTTTCCATTTTCCAATTCGATCCGATCCATTCGTTCGTAGAGCTATTTATTCGATCGCAGACATTTCTGGAACACCTCTAACAGAGGGACAAATAGTCAATTTTGAAAGAACTTATTGTCAACCTCTTTCGGATATGAATCTATCTGATTCAGAAGGGAAGAACTTGCATCAGTATCTCAATTTCAATTCAAACATGGGTTTGATTCACACTCCATGTTCTGAGAAATATTTACCATCCGAAAAGAGGAAAAAACGGAGTCTTTGTCTAAAGAAATGTGTTGAAAAAGGGCAGATGTATAGAACCTTTCAACGAGATAGTGCTTTTTCAACTCTCTCAAAATGGAATCTATTCCAAACATATATGCCATGGTTCCTTACTTCGACAGGGTACAAATATCTAAATTTTCTATTTTTCGATACCTTTTCGGACCTATTACCGATACTAAGTAGCAGTCAAAAATTTGTATCCATTTTTCATGATATTATGCATGGATCAGATATATCATGGCGAATTCTTCAGAAAAAATTGTGTCTTCCACAATGGAATCTGATAAGTGAGATTTCGAGTAAATGTTTACATAATCTTCTTCTGTCCGAAGAAATGATTCATCGAAATAATGAGCCACCATTGATATCGACACATCTGAGATCGCCAAATGTTCGGGAGTTCCTCTATTCAATCCTTTTCCTTCTTCTTGTTGCTGGATATCTCGTTCGTACACATCTTCTTTTTGTTTCCCGAGCCTATAGTGAGTTACAGACAGAGTTCGAAAAGGTCAAATCTTTGATGATTCCATCATACATGATTGAGTTGCGAAAACTTCTGGATAGGTATCCTACATCTGAACTGAATTCTTTCTGGTTAAAGAATCTCTTTCTAGTTGCTCTGGAACAATTAGGAGATTTTCTAGAAGAAATGCGGGGTTCTGCTTCTGGCGGCAACATGCTATGGGGTGGTGGTCCCACTTATGGGGTTAAATCAATCCGTTCTAAGAAGAAATTTTTGAATATCAATCTCATCGATCTCATAAGTATCATACCAAATCCCATCAATCGAATCACTTTTTCGAGAAATACGAGACATCTAAGTCATACAAGTAAAGAGATTTATTCATTGATAAGAAAAATAAAAAACGTGAACGGTGATTGGATTGATGATAAAATAGAATCCTTGGTCGCGAACAGTGATTCGATTGATGATAAAGAAAGAGAATTCTTGGTTCAGTTCTCCACCTTAACGACAGAAAAAAGGATTGATCAAATTCTATTGAGTCTGACTCATAGTGATCATTTATCAAAGAATGACTCTGGTTATCAAATGATTGAAGAGCCGGGAGCAATTTATTTACGATACTTAGTTGACATTCATAAAAAGTATCTAATGAATTATGAGTTCAATACACCCTGTTTAGCAGAAAGACGGATATTCCTTGCTTATTATCAGACAACCGCTTATTCACAAACCTCGTGTGGGGTGAATAGTTTTCATTTCCCATCTCATGGAAAACCCTTTTCGCTCCGCTTAGCCCTATCCCCCTCTAGGGGTATTTTAGTGATAGGTTCTATAGGAACTGGACGATCCTATTTGGTCAAATACCTAGCGACAAACTCCTATCTTCCTTTCATTACAGTATTTCTGAACAAGTTCCTGGATAACAAGCCTAACGGTTTTCTTATTGATGATAGTGACGATATTGATGATAGTGACGATATTGATGATAGTGACGATATTGATGTGAGTGACGATATTGATGTGAGTGACGATATCGACCGTGACTTTGATACGGAGCTGGAGTTTCTAACTAGGATGAATGCGCTAACTATGGATATGATGCCGGAAATAGACCGATTTTATATCACCCTTCAATTCGAATTAGCAAAAGCAATGTCTCCTTGCATAATATGGATTCCAAACATTCATGATCTGGATGTGAATGAGTCGAATTACTTATCCCTCGGTCTATTAGTGAACTATCTCTCCAGGGATTGTGAAAGATGTTCCACTAGAAATATTCTTGTTATTGCTTCGACTCATATTCCCCAAAAAGTGGATCCCGCTCTAATAGCTCCAAATAAATTAAATACATGCATTAAGATACGAAGGCTTCTTATTCCACAACAACGAAAGCACTTTTTTACTCTTTCGTATACTAGGGGATTTCACTTGGAAAATAAAATGTTCCATACTAATGGATTCGGGTCCATAACCATGGGTTCCAATGTACGAGATCTTGTAGCACTTACCAATGAGGCCTTATCGATTAGTATTACACAGAAAAAATCAATTATAGACACTAATATAATTAGATCTGCTCTTCATAGACAAACTTGGGATTTGCGATCCCAGGTAAGATCGGTTCAGGATCATGGGATCCTTTTCTATCAGATAGGAAGGGCTGTTGCACAAAATGTATTTCTAAGTAATTGCCCCATAGATCCTATATCTATCTATATGAAGAAGAAATCATGTAACGAAGGGGATTCTTATTTGTACAAATGGTACTTCGAACTTGGAATGAGCATGAAGAAATTAACGATACTTCTTTATCTTTTGAGTTGTTCTGCCGGATCGGCTGCTCAAGACCTTTGGTCTCTACCCGGACCCGATGAAAAAAATGGGATCACTTATTATGGACTTGTTGAGAATGATTCGGATCTAGTTCATGGTCTATTAGAAGTAGAAGGCGCTCTGGTGGGATCCTCACGTACAGAAAAAGATTGCAGTCAGTTTGATAATGATCGAGTGACATTGCTTCTTCGGCCCGAACCAAGGAGTCCCTTAGATATGATGCAAAATGGATCTTATTCTATCCTTGATCAGAGATTTCTCTATGAAAAATACGAATCGGAGTTTGAAGAAGGAGAAGAAGTCCTCGACCCGCAACAGATAGAGGACGATTTATTCAATCACATAGTTTGGGCTCCTAGAATATGGCGCCCTTGGGGTTTTCTATTTGATTGTATCGAAAGGCCCAATGAATTGGGATTTCCCTATTGGGCCAGGTCATTTCGGGGCAAGCGGATCATTTATGATGAAGAGGATGAGCTTCAAGAGAATGATTCGGGGTTCTTGCAGAGTGGAACCATGCAGTACCAGATACGAGATAGATCTTCCAAAGAACAAGGCTTTTTTCGAATAAGCCAATTCATTTGGGACCCTGCGGATCCACTCTTTTTCCTATTCAAAGATCAGCCCTTTGTCTCTGTGTTTTCACATCGAGAATTCTTTGCAGATGAAGAGATGTCAAAGGGGCTTCTTACTTCCCAAACAGATCCTCCTACATCTATATATAAACGCTGGTTTATCAAGAATACGCAAGAAAAGCACTTCGAATTGTTGATTCATCGCCAGAGATGGCTTAGAACCAATAGTTCATTATCTAATGGATTTTTCCGCTCTAATACTCTATCCGAGAGTTATCAGTATTTATCAAATCTGTTCCTATCTAACGGAACGCTATTGGATCAAATGACAAAGGCATTGTTGAGAAAAAGATGGCTTTTCCCGGATGAAATGAAAATTGGATTCATGTAA

>lcl|NC_033910.1_cds_YP_009348332.1_1 [gene=rps12] [locus_tag=B2L33_pgp041] [db_xref=GeneID:31082722] [protein=ribosomal protein S12] [exception=trans-splicing] [protein_id=YP_009348332.1] [location=complement(join(104232..104257,104794..105025,75634..75747))] [gbkey=CDS]
ATGCCAACTATTAAACAACTTATTAGAAACACAAGACAGCCAATTAGAAATGTTACCAAATCGCCCGCTCTTGGGGGATGTCCTCAACGCCGAGGAACATGTACTAGGGTGTATACTATCACCCCCAAAAAACCAAACTCTGCCTTACGTAAAGTTGCCAGAGTACGATTAACCTCTGGATTTGAAATCACTGCTTATATACCTGGTATTGGCCATAATTTACAAGAACATTCTGTAGTCTTAGTAAGAGGGGGAAGGGTTAAGGATTTACCCGGTGTGAGATATCACATTGTTCGAGGAACCCTAGATGCTGTCGGAGTAAAGGATCGTCAACAAGGGCGTTCTAAATATGGGGTCAAAAAGCCAAAATAA

>lcl|NC_033910.1_cds_YP_009348353.1_22 [gene=psaA] [locus_tag=B2L33_pgp066] [db_xref=GeneID:31082790] [protein=PsaA] [protein_id=YP_009348353.1] [location=complement(43348..45600)] [gbkey=CDS]
ATGATTATTCGTTCGCCGGAACCAGAAGTAAAAATTTTGGTAGATAGGGATCCCATCAAAACTTCTTTTGAGGAATGGGCCAGACCCGGTCATTTCTCAAGAACAATAGCTAAAGGACCTGATACTACCACTTGGATCTGGAACCTACATGCTGATGCTCACGATTTCGATAGCCATACCAATGATTTGGAGGAAATTTCTCGAAAAGTATTTAGTGCTCATTTTGGCCAACTCTCCATCATCTTTCTTTGGCTGAGTGGGATGTATTTCCACGGTGCTCGTTTTTCAAATTATGAAGCATGGCTAAGCGATCCTACTCACATTGGACCTAGTGCCCAAGTGGTTTGGCCAATAGTGGGCCAAGAAATATTGAACGGTGATGTAGGCGGGGGTTTCCGAGGAATACAAATAACCTCTGGTTTTTTTCAGATTTGGAGAGCATCTGGAATAACTAGTGAATTACAACTGTATTGTACCGCAATTGGTGCATTGGTCTTTGCAGCCTTAATGCTTTTTGCTGGTTGGTTCCATTATCACAAAGCTGCTCCAAAATTGGCTTGGTTCCAAGATGTAGAATCCATGTTGAATCACCATTTAGCGGGGCTACTAGGACTTGGGTCTCTTTCTTGGGCGGGACATCAAGTACATGTATCTTTACCAATTAACCAATTTCTAAACGCTGGAGTAGATCCTAAAGAAATCCCACTTCCTCATGAATTTATCTTGAATCGAGATCTTTTAGCTCAACTTTATCCCAGTTTTGCTGAGGGAGCAACTCCTTTTTTCACCTTAAATTGGTCAAAATATTCGGAATTTCTTACTTTTCGTGGAGGATTAGATCCAGTGACTGGAGGTCTATGGCTGACCGATATTGCACACCATCATTTAGCTATTGCAATTCTTTTTCTGATAGCGGGTCACATGTATAGGACTAACTGGGGTATTGGTCATGGTATAAAAGATATTTTAGAGGCTCATAAAGGTCCCTTTACAGGTCAGGGTCATAAAGGCCTATATGAAATCCTAACAACTTCATGGCATGCTCAATTATCTCTTAACCTAGCTATGTTAGGTTCTTTAACCATTGTTGTAGCTCACCATATGTATTCCATGCCCCCTTATCCATACCTAGCTACTGACTATGGTACACAATTGTCATTGTTCACACATCACATGTGGATTGGTGGATTCCTCATAGTTGGTGCTGCTGCGCATGCAGCCATTTTTATGGTAAGAGACTATGATCCAACTACTCGATACAACGATCTATTAGATCGTGTTCTTAGGCATCGTGATGCAATCATATCACATCTCAATTGGGTATGTATATTTTTAGGCTTTCACAGTTTTGGTTTATATATTCATAATGATACCATGAGCGCTTTAGGGCGACCTCAAGATATGTTTTCAGATACTGCTATACAATTACAACCCGTCTTTGCTCAATGGATACAAAACACTCATGCTTTAGCACCTAGTGCAACGGCTCCTGGTGCAACAGCAAGCACCAGTTTAACTTGGGGAGGTGTTGATTTAGTGGCAGTGGGTGGCAAGGTTGCTTTGTTACCGATTCCATTAGGAACCGCGGATTTTTTGGTCCATCACATTCATGCATTTACGATTCATGTGACGGTGTTGATACTTCTGAAAGGAGTTCTATTTGCTCGTAGCTCTCGTTTGATACCGGATAAAGCAAATCTTGGTTTTCGTTTTCCTTGTGATGGACCTGGAAGAGGGGGAACATGTCAAGTATCCGCTTGGGATCACGTCTTTTTAGGGCTATTTTGGATGTACAATTCAATTTCGATAGTCATATTCCATTTCAGTTGGAAAATGCAGTCAGATGTTTGGGGTAGTATAAGTGATCAAGGGGTGGTAACTCATATCACGGGAGGAAACTTCGCACAGAGTTCCATTACTATTAATGGTTGGCTCCGCGATTTCTTATGGGCACAGGCATCTCAGGTAATTCAGTCTTATGGTTCTTCATTATCTGCATATGGACTTTTTTTCCTAGGTGCTCATTTTGTATGGGCTTTTAGTTTAATGTTTCTATTCAGCGGTCGTGGTTATTGGCAAGAACTTATTGAATCAATTGTTTGGGCTCATAATAAATTAAAAGTTGCTCCTGCTACTCAGCCTAGAGCCTTGAGCATTATACAAGGACGTGCTGTAGGAGTAACTCATTACCTTCTGGGTGGAATTGCCACAACATGGGCGTTCTTCTTAGCAAGAATTATTGCAGTAGGATAA

>lcl|NC_033910.1_cds_YP_009348351.1_20 [gene=rps14] [locus_tag=B2L33_pgp068] [db_xref=GeneID:31082738] [protein=ribosomal protein S14] [protein_id=YP_009348351.1] [location=complement(40697..40999)] [gbkey=CDS]
ATGGCAAGAAAAAGTTTGATTCAACGGGAGAATAAGAGGCAAAAATTGGAACAAAAATATCATTTGATTCGGCGATCCTCAAAAAAAGAAATAAGCAAAGTTCTGTCCTTGAGTGATAAATGGGAAATTCATGGAAAGTTACAATCCCCACCGCGAAATAGTGCACCGACACGTCTTCATCGACGTTGTTTTTCAACTGGAAGACCGAGAGCTAACTATCGAGACTTTAGGCTATCTGGCCACATACTTCGTGAAATGGTTCATGCATGTTTGTTACCGGGAGCAACAAGATCGAGTTGGTAA

>lcl|NC_033910.1_cds_YP_009348377.1_46 [gene=rps12] [locus_tag=B2L33_pgp042] [db_xref=GeneID:31082814] [protein=ribosomal protein S12] [exception=trans-splicing] [protein_id=YP_009348377.1] [location=join(complement(75634..75747),146289..146520,147057..147082)] [gbkey=CDS]
ATGCCAACTATTAAACAACTTATTAGAAACACAAGACAGCCAATTAGAAATGTTACCAAATCGCCCGCTCTTGGGGGATGTCCTCAACGCCGAGGAACATGTACTAGGGTGTATACTATCACCCCCAAAAAACCAAACTCTGCCTTACGTAAAGTTGCCAGAGTACGATTAACCTCTGGATTTGAAATCACTGCTTATATACCTGGTATTGGCCATAATTTACAAGAACATTCTGTAGTCTTAGTAAGAGGGGGAAGGGTTAAGGATTTACCCGGTGTGAGATATCACATTGTTCGAGGAACCCTAGATGCTGTCGGAGTAAAGGATCGTCAACAAGGGCGTTCTAAATATGGGGTCAAAAAGCCAAAATAA

>lcl|NC_033910.1_cds_YP_009348414.1_83 [gene=ycf2] [locus_tag=B2L33_pgp004] [db_xref=GeneID:31082849] [protein=Ycf2] [protein_id=YP_009348414.1] [location=complement(151467..158357)] [gbkey=CDS]
ATGAAGGGACATCAATTCAAATCCTGGATTTTCGAATTGAGAGAGATATTGAGAGAGATCAAGAATTCTCACTATTTCTTCGATTCATGGACCCAATTCAATTCAGTGGGATCTTTCATTCACATTTTTTTCCATCAAGAACGTTTTATAAAACTCTTGGACTCCCGAATTTGGAGTATCTTACTTTCACGCAATTCACAGGGTTCAACAAGCAATCGATATTTCACGATCAAGGGTGTAGTACTCTTTGTAGTAGTGGTCCTTATATATCGTATTAACAATCGAAAGATGGTCGAAAGAAAAAATCTCTATTTGACAGGGCTTCTTCCTATACCTATGAATTCCATTGGACCCAGAAATGATACATTGGAAGAATTCTTTGGGTCTTACAATATCAATAGGTTGATTGTTTCGCTCCTGTATCTTCCAAAAGGAAAAAAGATCTCTGAGAGCTCTTTCCCGGATCTGAAAGAGAGTACTTGGGTTCTCCCAATAACTAAAAAGTGTATCATGTCTGAATCTAACTGGGGTTCACGGCGGTGGAGGAACTGGATCGGAAAAAAGAGGGATTCTAGTTGTAAGATATCTAATGAAACCGTCGCTGGAATTGAGATCTCATTCAAAGAAAAAGATATCAAATATCTGGAGTTTCTTTTTGTATATTATATGGATGATCCGATCCGCAAGGACCAGGATTGGGATTTGTTTGATCGTCTTTCTCCGAGGAAGGGGCGAAACAGAATCAACTTGAATTCGGGACAGCTATTGGAAATCTTAGTGAAAGACTGGATTTGTTATCTCATGTTTGCTTTTCGTGAAAAAATACCAATTGAAGTGGAGGGTTTCTTCAAACAACAAGGAGCTGGGTCAACTATTCAATCAAATGATATTGAGCATGTTTCCCATCTCTTCTCGAGAAAGAAGTGGGCTATTTCTTTGCAAAATTGTGCTCAATTTCATATGTGGCAATTCCGCCAAGATCTCTTCGTTAGTTGGGGGAATAATCCGCACGAATCGGATTTTTTGAGGAACATATCGAGAGAGAATTGGATTTGGTTAGACAATGTGTGGTTGGTAAACAAGGATCGGTTTTTTAGCAAGGCACGGAATATATCGTCAAATATTCAATATGATTCCACAAGATCTAGTTTCGTTCAAGGAAGGAATTCTAGCCAATTGAAGGGATCTTCTGATCAATCCAGAGATCATTTCGATTCCATTAGTAATGAGGATTCGGAATATCACACATTGATCAATCAAAGAAAGATTCAACAACTAAAAGAAAGATCGATTCTTTGGGATCCTTCCTTTCTTCAAACGGAACGAACAGAGATAGAATCAGACCAATTCCCTAAATGCCTTTCTGGATATTCCTCAATGTCCCGGCTATTCAGGGAAGGTGAGAAGGAGATGAATAATCATCTGCTTCCGGAAGAAATCGAAGAATTTCTTGGGAATCCTACAAGATCCATTCGTTCTTTTTTCTCTGACAGATCGTCAGAACTTCATCTGGGTTCGAATCCTACTGAGAGATCCACTAGAGATCAGAAATTGTTGAAGAAAGAACAAGATGGTTCTTTTGTCCCTTCCAGGCGATCGGAAAATAAAGAAATAGTTAATATATTCAAGATAATCACGTATTTACAAAATACCGTCTCAATTCATCCATCCGATCCGGGATGTGATATGGTTCTGAAGGATGAACTGGATATGGACAGTTCCAATAAGATTTCTTTCTTGAACAAAAATCCATTTTTTGATTTATTTCATCTATTCCATGATCGGAACGGGGGGGGGTACACGTTACACCACGATTTTGAATCAGAAGAGAGATTTCAAGAAATGGCAGATCTATTCACTCTATCAATAACCGAGCCGGATCTGGTGTATCATAAGGGATTTACCCGTTTTATTGATTCCTACGGATTGGATCAAAAACAATTCTTGAATGAGGTATTCAACTCCAGGGATGAATCGAAAAAGAAATCTTTATTGGTTCTACCTCCTATTTTTTATGAAGAGAATGAATCTTTTTATCGAAGGATCAGAAAAAAATGGGTCCGGATCTCCTGCGGGAATGATTTGGAAGATCCAAAACAAAAAACAGTGGTATTTGCTAGCAACAACATAATGGAGGCAGTCAATCAATATGGATTGATCCTAAATCTGATTCAAATCCAATATAGTACCTATGGGTACATAAGAAATGTATTGACTCAATTCTTTTTAATGAATAGATCCGATCGCAACTTCGAATATGGAATTCAAAGGGATCAAATAGGAAATGATACTCTGAATCATAGAACTATAATGAAATATACGATCAACCAACATTTATCGAATTTGAAACAGAGTCAGAAGAAATGGTTCGATCCTCTTATTTTTCGTTCTCGAACCGAGAGATCCATGAATTGGGATCCTAATGCATATAGATACAAATGGTCTAATGAGAGCAAGAATTTCCAGGAACATTTGGAACATTTCATTTCTGAGCAGAAGAGCCGTTTTCTTTTTCAAGTAGTGTTCGATCGATTACGTATTAATCAATATTCGATTGATTGGTCTGAGGTTATCGACAAAAAAGATTTGTCTAAGTCACTTCCTTTCTTTTTGTCCAAGTTACTTCTTTTTTTGTCCAAGTTTCTTCTCTTTTTGTCTAACTCACTTCCTTTTTTCTTTGTGAGTTTCGGGAATATCCCCATTCATAGGTCCGAAATCCATATCTATGAATTGAAAGGTCAGAATGATCAACTCTGCAATCAGCTGTTAGAACCAATAGGTCTTCAAATCGTTCATTTGAAAAAATTGAAACCCTTCTTATTGGATGATCATGATACTTCCCAAAAATCGAAATTTTTAATTAATATTAATGGAGGAACAATATCACCATTTTTGTTCAATAAGATACCAAAGTGGATGATTGACTCATTCCATACTAGAACTAATCGCAGGAAATCTTTTGATAACACGGATTCCTATTTCTCAATCATATCCCACGATCAAGACAATTGGCTGAATCCCGTGAAACCATTTCATAGAAGTTCATTGATATCTTCTTTTTATAAAGCAAATCGACTTCGATTCTTGAATAATCTACATCACTTCTGCTTCTATTGTAACAAAAGATTCCCTTTTTATGTGGAAAAGGCCCGTATCAAGAATTCTGATTTTACGTATGGACAATTCCTCAATATCTTGTTCATTCGCAACAAAATATTTTCTTTGTGCGGCGGTAAAAAAAAACATGCTTTTTTGGAGAGAGATACTATTTCACCAATCGAGTCCCAGGTATCTAACATATTCATACCTAATGATTTTCCACAAAGTGGTAACGAAAGGTATAACTTGTACAAATCTTTCCATTTTCCAATTCGATCCGATCCATTCGTTCGTAGAGCTATTTATTCGATCGCAGACATTTCTGGAACACCTCTAACAGAGGGACAAATAGTCAATTTTGAAAGAACTTATTGTCAACCTCTTTCGGATATGAATCTATCTGATTCAGAAGGGAAGAACTTGCATCAGTATCTCAATTTCAATTCAAACATGGGTTTGATTCACACTCCATGTTCTGAGAAATATTTACCATCCGAAAAGAGGAAAAAACGGAGTCTTTGTCTAAAGAAATGTGTTGAAAAAGGGCAGATGTATAGAACCTTTCAACGAGATAGTGCTTTTTCAACTCTCTCAAAATGGAATCTATTCCAAACATATATGCCATGGTTCCTTACTTCGACAGGGTACAAATATCTAAATTTTCTATTTTTCGATACCTTTTCGGACCTATTACCGATACTAAGTAGCAGTCAAAAATTTGTATCCATTTTTCATGATATTATGCATGGATCAGATATATCATGGCGAATTCTTCAGAAAAAATTGTGTCTTCCACAATGGAATCTGATAAGTGAGATTTCGAGTAAATGTTTACATAATCTTCTTCTGTCCGAAGAAATGATTCATCGAAATAATGAGCCACCATTGATATCGACACATCTGAGATCGCCAAATGTTCGGGAGTTCCTCTATTCAATCCTTTTCCTTCTTCTTGTTGCTGGATATCTCGTTCGTACACATCTTCTTTTTGTTTCCCGAGCCTATAGTGAGTTACAGACAGAGTTCGAAAAGGTCAAATCTTTGATGATTCCATCATACATGATTGAGTTGCGAAAACTTCTGGATAGGTATCCTACATCTGAACTGAATTCTTTCTGGTTAAAGAATCTCTTTCTAGTTGCTCTGGAACAATTAGGAGATTTTCTAGAAGAAATGCGGGGTTCTGCTTCTGGCGGCAACATGCTATGGGGTGGTGGTCCCACTTATGGGGTTAAATCAATCCGTTCTAAGAAGAAATTTTTGAATATCAATCTCATCGATCTCATAAGTATCATACCAAATCCCATCAATCGAATCACTTTTTCGAGAAATACGAGACATCTAAGTCATACAAGTAAAGAGATTTATTCATTGATAAGAAAAATAAAAAACGTGAACGGTGATTGGATTGATGATAAAATAGAATCCTTGGTCGCGAACAGTGATTCGATTGATGATAAAGAAAGAGAATTCTTGGTTCAGTTCTCCACCTTAACGACAGAAAAAAGGATTGATCAAATTCTATTGAGTCTGACTCATAGTGATCATTTATCAAAGAATGACTCTGGTTATCAAATGATTGAAGAGCCGGGAGCAATTTATTTACGATACTTAGTTGACATTCATAAAAAGTATCTAATGAATTATGAGTTCAATACACCCTGTTTAGCAGAAAGACGGATATTCCTTGCTTATTATCAGACAACCGCTTATTCACAAACCTCGTGTGGGGTGAATAGTTTTCATTTCCCATCTCATGGAAAACCCTTTTCGCTCCGCTTAGCCCTATCCCCCTCTAGGGGTATTTTAGTGATAGGTTCTATAGGAACTGGACGATCCTATTTGGTCAAATACCTAGCGACAAACTCCTATCTTCCTTTCATTACAGTATTTCTGAACAAGTTCCTGGATAACAAGCCTAACGGTTTTCTTATTGATGATAGTGACGATATTGATGATAGTGACGATATTGATGATAGTGACGATATTGATGTGAGTGACGATATTGATGTGAGTGACGATATCGACCGTGACTTTGATACGGAGCTGGAGTTTCTAACTAGGATGAATGCGCTAACTATGGATATGATGCCGGAAATAGACCGATTTTATATCACCCTTCAATTCGAATTAGCAAAAGCAATGTCTCCTTGCATAATATGGATTCCAAACATTCATGATCTGGATGTGAATGAGTCGAATTACTTATCCCTCGGTCTATTAGTGAACTATCTCTCCAGGGATTGTGAAAGATGTTCCACTAGAAATATTCTTGTTATTGCTTCGACTCATATTCCCCAAAAAGTGGATCCCGCTCTAATAGCTCCAAATAAATTAAATACATGCATTAAGATACGAAGGCTTCTTATTCCACAACAACGAAAGCACTTTTTTACTCTTTCGTATACTAGGGGATTTCACTTGGAAAATAAAATGTTCCATACTAATGGATTCGGGTCCATAACCATGGGTTCCAATGTACGAGATCTTGTAGCACTTACCAATGAGGCCTTATCGATTAGTATTACACAGAAAAAATCAATTATAGACACTAATATAATTAGATCTGCTCTTCATAGACAAACTTGGGATTTGCGATCCCAGGTAAGATCGGTTCAGGATCATGGGATCCTTTTCTATCAGATAGGAAGGGCTGTTGCACAAAATGTATTTCTAAGTAATTGCCCCATAGATCCTATATCTATCTATATGAAGAAGAAATCATGTAACGAAGGGGATTCTTATTTGTACAAATGGTACTTCGAACTTGGAATGAGCATGAAGAAATTAACGATACTTCTTTATCTTTTGAGTTGTTCTGCCGGATCGGCTGCTCAAGACCTTTGGTCTCTACCCGGACCCGATGAAAAAAATGGGATCACTTATTATGGACTTGTTGAGAATGATTCGGATCTAGTTCATGGTCTATTAGAAGTAGAAGGCGCTCTGGTGGGATCCTCACGTACAGAAAAAGATTGCAGTCAGTTTGATAATGATCGAGTGACATTGCTTCTTCGGCCCGAACCAAGGAGTCCCTTAGATATGATGCAAAATGGATCTTATTCTATCCTTGATCAGAGATTTCTCTATGAAAAATACGAATCGGAGTTTGAAGAAGGAGAAGAAGTCCTCGACCCGCAACAGATAGAGGACGATTTATTCAATCACATAGTTTGGGCTCCTAGAATATGGCGCCCTTGGGGTTTTCTATTTGATTGTATCGAAAGGCCCAATGAATTGGGATTTCCCTATTGGGCCAGGTCATTTCGGGGCAAGCGGATCATTTATGATGAAGAGGATGAGCTTCAAGAGAATGATTCGGGGTTCTTGCAGAGTGGAACCATGCAGTACCAGATACGAGATAGATCTTCCAAAGAACAAGGCTTTTTTCGAATAAGCCAATTCATTTGGGACCCTGCGGATCCACTCTTTTTCCTATTCAAAGATCAGCCCTTTGTCTCTGTGTTTTCACATCGAGAATTCTTTGCAGATGAAGAGATGTCAAAGGGGCTTCTTACTTCCCAAACAGATCCTCCTACATCTATATATAAACGCTGGTTTATCAAGAATACGCAAGAAAAGCACTTCGAATTGTTGATTCATCGCCAGAGATGGCTTAGAACCAATAGTTCATTATCTAATGGATTTTTCCGCTCTAATACTCTATCCGAGAGTTATCAGTATTTATCAAATCTGTTCCTATCTAACGGAACGCTATTGGATCAAATGACAAAGGCATTGTTGAGAAAAAGATGGCTTTTCCCGGATGAAATGAAAATTGGATTCATGTAA

>lcl|NC_033910.1_cds_YP_009348376.1_45 [gene=rpl20] [locus_tag=B2L33_pgp043] [db_xref=GeneID:31082813] [protein=ribosomal protein L20] [protein_id=YP_009348376.1] [location=complement(74463..74819)] [gbkey=CDS]
ATGACCAGAATTAGACGAGGATATATAGCTCGGAGACGTAGAACAAAAATTCGTTTATTCGTATCAAGCTTTCGCGGGGCTCATTCAAGACTTACTCGAAGTATTATTCAACAAAAAATAAGAGCTTTGGTTTCGGCCCATCGGGATAGAGATAGACAGAAAAGAAATTTTCGTCGTTTATGGGTCACTCGGATAAATGCAGTAATTCGCGGAAATAGCAGGGTATCAAATAGTTATAGTAATTTAGTAAATAATCTGTACAAGAGACAATTGCTTCTTAATCGTAAAATACTTGCACAAATAGCTATATTAAATAGGAATTGTCTTTATACGATTTCCAATGACATTAGAAAATAA

>lcl|NC_033910.1_cds_YP_009348401.1_70 [gene=ccsA] [locus_tag=B2L33_pgp017] [db_xref=GeneID:31082760] [protein=CcsA] [protein_id=YP_009348401.1] [location=120220..121188] [gbkey=CDS]
ATGATCGTTTCGAATTTCGAACATATATTAACTCATATATCTTTTTCAGTCGTGTCAATTGTAATTACAATTCATTTGATAACCTTATTAGTCGATGAATTCGTAGAACTATATGATTCGTCAGAAAAGGGCATGTTAACGACTTTTTTCTGTATAACCGGATTATTAGTTACTCGTTGGTTTTTTGGGGGACATTTACCATTAAGTGATTTATATGAATCATTAATCTTTCTTTCATGGGCATTTTGTGTTATTCATATAATTCCGTATTTTAAAAAATATAAAAATTATTTAAGCGCAATAACCGCGCCAAGTACTTTTTTGACTCAAGGGTTTGCCACTTCGGGTCTTTTAAAAGGCATGCATCAATCCGAAATCTTAGTACCCGCTCTTCAATCCCAGTGGTTAATGATGCACGTAAGTATGATGATTTTTGGCTATGCAGCTCTTTTGTGTGGATCATTATTATCAGTAGCATTTCTAGTAATCAGATTTCAAAAAATTAGAATAATTTTTGATAAAAGCACTAATTTTTTAAATGATTCGTTTTACTTTAATGAGATACAATATATAACGGAAGGAAAGAATGTTTTAAGAAATAGTTCCTTTCTTTCCTCTAGGAATTATTATAGGTTTCAATTAATTCAACAATTAGATGACTGGAGTTATCGGATTATAAGTATAGGTTTTTTTTTTTTAACTATAGGTATTCTTTCGGGAGCAGTCTGGGCTAATGAAGCATGGGGATCATATTGGAATTGGGACCCAAAGGAAACTTGGGCATTTATTACATGGACCATATTCGCGGTTTTTTTTCATACTCGAACAAATAAAAATTTGGAGAGTTTAAATTCGGCAATTATAGCTTCTATCGGTTTTCTTATAATTTGGATATGCTATTTTGGAGTTAATTTATTAGGAATAGGACTACATAGTTATGGTTCATTTACATTACCAATTAACAATTGA

>lcl|NC_033910.1_cds_YP_009348358.1_27 [gene=ndh3] [locus_tag=B2L33_pgp061] [db_xref=GeneID:31082795] [protein=Ndh3] [protein_id=YP_009348358.1] [location=complement(53729..54091)] [gbkey=CDS]
ATGTTTCTGCTTTACGAATATGATATATTCTGGGCGTTTCTAATAATATCAAGTGTTATTCCTATTTTAGCATTTCTAATTTCCGGAGTTTTATCCCCGATTAACACAGGTCCGGAGAAATTTTCTAGTTATGAATCGGGTATCGAACCAATAGGCGATGCTTGGTTACAATTTCGAATCCGTTATTATATGTTTGCTCTAGTTTTTGTTGTTTTTGATGTTGAAACAGTTTTTCTTTATCCATGGGCAATGAGTTTCGATATATTGGGGTTATCCGTATTTATAGAAGCTTTGATTTTCGTGCTTATCTTAATTGTTGGTTCAGTTTATGCATGGAGAAAGGGAGCATTAGAGTGGTCTTAG

>lcl|NC_033910.1_cds_YP_009348342.1_11 [gene=rps2] [locus_tag=B2L33_pgp077] [db_xref=GeneID:31082779] [protein=ribosomal protein S2] [protein_id=YP_009348342.1] [location=complement(17455..18165)] [gbkey=CDS]
ATGAGAAAAAGATATTGGAACATTAATTTGGAAGAGATGATGAAAGCGGGAGTTCATTTTGGTCATGGTACTAGAAAATGGAACCCGAGAATGGCCCCTTATATCTCTGCAAAACGTAAAGGTATTCATATTACAAATCTTACTAGAACTGCTCGTTTTTTATCAGAAGCTTGTGATTTAATTTTCGATGCAGCAAGTAAGAGAAAACAATTCTTAATTGTTGGTACCAAAAATAAAGCAGCGGATTCAGTAGCGCGGGCTGCAATAAGGGCTCGGTGTCATTATGTTAATAAAAAATGGCTCGGCGGTATTTTAACGAATTGGTCCACTACAGAAACTAGACTTCAAAAGTTCAGGGACTTGAGAATAGAACAAAAGACAGGTAGACTCAACCGTCTTCCGAAAGGAGATGGGACTCGATTGAAGAGACAGTTAGCTCACTTACAAACATATCTGGGCGGTATTAAATATATGACAGGGTTACCCGATATTGTAATACTGGTTGATCAGCAAGAAGAATATACGGCTCTTCGGGAATGTATCACTTTGGGAATTCCAACCATTTGTTTAATTGATACAAACTGTGACCCGGATCTCGCAGATATTTCGATTCCAGCGAATGATGACGCTATAGCTTCAATCCGATTAATTCTTAATAAATTAGTATTTGCAATTTGTGAGGGTCGTTCTAGCTATATACGAAATTCCTGA

>lcl|NC_033910.1_cds_YP_009348348.1_17 [gene=psbD] [locus_tag=B2L33_pgp071] [db_xref=GeneID:31082735] [protein=PsbD] [protein_id=YP_009348348.1] [location=36051..37112] [gbkey=CDS]
ATGACTATAGCCCTTGGTAAATTTACCAAAGACGAAAATGATTTATTTGATATTATGGATGACTGGTTACGGAGGGACCGTTTCGTTTTTGTAGGTTGGTCCGGTCTATTGCTCTTTCCTTGTGCCTATTTCGCCTTAGGGGGTTGGTTCACAGGTACAACCTTTGTAACCTCATGGTATACCCATGGATTGGCCAGTTCTTATTTGGAAGGCTGCAACTTCTTAACCGCCGCAGTTTCTACTCCTGCTAATAGTTTAGCACATTCTTTGTTATTACTATGGGGTCCTGAAGCCCAAGGAGATTTTACTCGTTGGTGTCAATTAGGCGGTTTGTGGACTTTTGTTGCTCTACACGGCGCTTTCGGGCTAATAGGTTTTATGTTACGTCAATTTGAACTTGCTCGATCCGTGCAATTGAGACCTTATAATGCAATCGCATTCTCTGGTCCAATTGCTGTTTTTGTTTCTGTATTCCTGATTTATCCACTAGGGCAGTCTGGTTGGTTTTTTGCACCTAGTTTTGGTGTAGCAGCTATATTTCGATTCATCCTCTTTTTCCAAGGGTTTCATAACTGGACGCTGAACCCATTTCATATGATGGGAGTTGCTGGCGTATTGGGCGCTGCCCTCTTATGCGCTATTCATGGCGCTACTGTGGAAAAGACTTTATTTGAAGATGGTGATGGTGCCAATACATTCTGTGCTTTTAACCCAACTCAAGCTGAAGAAACTTATTCAATGGTCACCGCTAACCGCTTTTGGTCTCAAATCTTTGGGGTTGCTTTTTCCAATAAACGTTGGTTACATTTCTTTATGTTATTTGTACCAGTAACTGGTTTATGGATGAGCGCTCTTGGAGTAGTCGGTCTGGCTCTGAATCTACGTGCCTATGACTTCGTTTCTCAGGAAATCCGTGCAGCGGAAGATCCTGAATTTGAGACTTTCTACACTAAAAATATTCTCTTAAACGAAGGTATTCGTGCTTGGATGGCGGCTCAAGATCAGCCTCATGAAAACCTTATATTCCCTGAGGAGGTTCTACCACGTGGAAACGCTCTTTAA

>lcl|NC_033910.1_cds_YP_009348404.1_73 [gene=ndhE] [locus_tag=B2L33_pgp014] [db_xref=GeneID:31082839] [protein=NdhE] [protein_id=YP_009348404.1] [location=complement(123516..123821)] [gbkey=CDS]
ATGATGCTCGAACATGTACTTATTTTGAGTGCTTGTTTATTTTCTATCGGTATCTATGGATTGATCATGAGTCGAAATATGGTTAGAGCCCTTATGTGTCTTGAACTTATACTGAATGCTGTTAATATAAATTTCGTAACATTTTCTGATTTTTTTGATAGTCGCCAACTAAAAGGAAATATTTTTTCAATTTTTGTTATAGCTATCGCAGCCGCTGAAGCAGCTATTGGACTGGCTATTGTTTCGTCTATTTATCGTAACAGAAAATCCACCCGTATCAATCAATCGAATTTATTGAATAAGTAG

>lcl|NC_033910.1_cds_YP_009348385.1_54 [gene=rpoA] [locus_tag=B2L33_pgp033] [db_xref=GeneID:31082820] [protein=RpoA] [protein_id=YP_009348385.1] [location=complement(84025..85044)] [gbkey=CDS]
ATGGTTCGAGAGAAAGTAACAATATCTACTCGGACACTGCAGTGGAAATGTGTTGAATCAAGAAAGGACAATAAGCGTCTTTATTACGGACGCTTTATTCTCTCTCCGCTTATGAAAGGCCAATCCGATACGATAGGCATTGCGATTCGAAGAGCTTTGCTTGGAGAAATAGAAGGAACCTGTATCACACGTGCAAAATCTGAAAAAATATCACACGAATTTTCTACTATAACAGGTATTCAAGAATCAATACATGAAATTTTCATGAATTTGAAAGAAATTATATTGAGAAGCAATTTGTATGGAACTTGTGACGCGTCTATTTGTGTCAAGGGTCCTGGATATGTAACTGCTCAAGACATCATTCTACCACCTTTTGTGGAAATCATTGATAATACACAGCATATCGCTAGCCTAACAGAACCAATTGATTTGTGTATTCGATTACAAATCGAGAGGAATCGTGGCTATCGTATAAAACCAACAAAAACCTTGCAAGATGGAAGTTTTCCTCTAGATGCTGTATTCATGCCGGTTCGAAATGCAAATCATAGTGTTCATTCTTATGGAAATGGGAATGAAAAGCAAGAGATACTTTTTCTCGAAATATGGACAAACGGAAGTTTAACTCCTAAAGAAGCACTTCATGAGGCTTCCCGTAATTTGATTGATTTATTTATTCCTTTTCTACATGCAGACGAACAAAACTTACCTTTAGAAAAAAATCAACACAACGTTACTTTACCCCTTTTGACTTTTCATGATAGATTGACTAAATTAAGAAAAAAAAAAAAAGAAATACCATTGAAATACATTTTTATTGACCAATCCGAATTGACTCCTAAGATCTATAATTGCCTCAAAAGGTCTAATATACATACATTATCGGACCTTTTGAATAAGAGTCAAGAAGATCTTATGCAAATTGAAGATTTTCACATAGACGATGTAAAACATATATTGGGTATTTTAGAAATAAAAAAACATTTCGTAATGGATTTACTAAAGAATAAAATCTAA

>lcl|NC_033910.1_cds_YP_009348398.1_67 [gene=rps7] [locus_tag=B2L33_pgp020] [db_xref=GeneID:31082833] [protein=ribosomal protein S7] [protein_id=YP_009348398.1] [location=complement(103706..104173)] [gbkey=CDS]
ATGTCACGTCGAGGTACTGCAGAAGAGAAAACTGCAAAATCCGATCCAATTTATCGTAATCGATTAGTTAACATGTTGGTTAACCGTATTCTGAAACACGGAAAAAAATCATTGGCTTATCAAATTATCTATCGAGCCATGAAAAAGATTCAACAAAAGACAGAAACAAATCCACTATCTGTTTTACGTCAAGCAATACGTGGAGTAACTCCCGATATAGCAGTAAAAGCAAGACGTGTAGGCGGATCGACTCATCAAGTTCCCATTGAAATAGGATCCACACAAGGAAAAGCACTTGCCATTCGTTGGTTATTAGGGGCATCCCGAAAACGTCCGGGTCGAAATATGGCTTTCAAATTAAGTTCCGAATTAGTGGATGCTGCCAAAGGGAGTGGTGATGCCATACGCAAAAAGGAAGAGACTCATAGAATGGCAGAGGCAAATAGAGCTTTTGCACATTTTCGTTAA

>lcl|NC_033910.1_cds_YP_009348406.1_75 [gene=ndhI] [locus_tag=B2L33_pgp012] [db_xref=GeneID:31082841] [protein=NdhI] [protein_id=YP_009348406.1] [location=complement(125036..125533)] [gbkey=CDS]
ATGTTTCCCATGGTAACTGGATTCATGAATTATGGGCAACAAACCATACGAGCTGCAAGGTACATTGGTCAAAGTTTCATGATTACCTTATCCCATGCAAATCGTTTACCTGTAACTATTCAATATCCTTATGAAAAATTAATAACATCGGAGCGTTTCCGTGGTCGAATCCATTTTGAATTTGATAAATGCATTGCTTGTGAAGTATGTGTTCGTGTATGTCCTATAGATCTGCCTGTTGTTGATTGGAAATTGGAAACTGACATTCGAAAGAAACGGTTGCTAAATTACAGTATTGATTTCGGAATCTGTATATTTTGCGGCAACTGCGTTGAGTATTGTCCAACAAATTGTTTATCAATGACAGAAGAATATGAACTTTCTACTTATGATCGTCATGAATTGAATTATAATCAAATTTCTTTGGGTCGTTTACCAATGTCAGTAGTTGAGGATTATACGATTCGAACAATTTTAAATTCAACTAAAATCAACTAA

>lcl|NC_033910.1_cds_YP_009348349.1_18 [gene=psbC] [locus_tag=B2L33_pgp070] [db_xref=GeneID:31082786] [protein=PsbC] [protein_id=YP_009348349.1] [location=37060..38481] [gbkey=CDS]
ATGAAAACCTTATATTCCCTGAGGAGGTTCTACCACGTGGAAACGCTCTTTAATGGAACTTTATCTTTAGCCGGTCGTGACCAAGAAACCACGGGTTTCGCTTGGTGGGCCGGGAATGCTCGACTTATCAATTTATCCGGTAAACTTCTGGGAGCTCATGTAGCTCATGCTGGATTAATCGTATTCTGGGCCGGAGCAATGAACCTATTTGAAGTGGCTCATTTTGTACCGGAGAAACCAATGTATGAACAAGGATTAATTTTACTTCCCCACCTAGCTACTCTAGGTTGGGGGGTAGGTCCTGGTGGGGAAGTTCTAGACACCTTTCCATACTTTGTATCGGGTGTACTTCACTTAATTTCCTCTGCAGTATTGGGCTTTGGCGGCATTTATCATGCACTTCTGGGTCCTGAGACTCTTGAAGAATCTTTTCCTTTTTTTGGTTATGTATGGAAAGATAGAAATAAAATGACAACAATTTTAGGTATTCACTTAATCTTGCTAGGTATAGGTTCTTTTCTTCTAGTATTCAAGGCTCTTTATTTTGGGGGTGTATACGATACCTGGGCTCCGGGGGGTGGGGATGTAAGAAAAATTACCAACTTGACCCTTAGCCCAAGTGTTATTTTTGGTTATTTACTAAAATCCCCCTTTGGCGGAGAAGGATGGATTGTTAGTGTGGACGATTTGGAAGATATAATTGGGGGGCATGTATGGTTAGGTTCTATTTGTATACTTGGTGGAATCTGGCATATCTTAACCAAACCCTTTGCATGGGCTCGCCGTGCACTTGTATGGTCTGGAGAGGCTTACTTATCTTATAGTTTAGCCGCTTTATCCGTTTTTGGTTTCATTGCTTGTTGCTTTGTCTGGTTCAATAATACCGCTTATCCTAGTGAGTTTTACGGGCCTACCGGACCAGAAGCTTCTCAAGCTCAAGCTTTTACTTTTCTAGTTAGAGATCAACGTCTTGGGGCTAATGTGGGATCCGCTCAAGGACCTACTGGGTTAGGTAAATATTTAATGCGTTCTCCTACCGGAGAAGTTATTTTTGGAGGAGAAACTATGCGTTTTTGGGATCTGCGTGCTCCTTGGTTAGAACCTCTAAGAGGTCCGAATGGTTTGGACTTGGGTAGGTTGAAAAAAGACATACAACCTTGGCAAGAACGCCGTTCCGCGGAATATATGACCCACGCGCCTTTAGGTTCATTAAATTCTGTAGGTGGCGTAGCTACCGAGATCAATGCAGTCAATTATGTCTCTCCTCGAAGTTGGTTAGCTACCTCTCATTTTGTTCTAGGTTTCTTCCTATTCGTAGGTCATTTATGGCACGCGGGGAGGGCTCGTGCAGCTGCAGCAGGATTTGAAAAAGGAATTGATCGTGATTTTGAACCCGTTCTCTCCATGACTCCTCTTAATTAA

>lcl|NC_033910.1_cds_YP_009348410.1_79 [gene=ycf1] [locus_tag=B2L33_pgp008] [db_xref=GeneID:31082845] [protein=Ycf1] [protein_id=YP_009348410.1] [location=complement(129832..135561)] [gbkey=CDS]
ATGATTTTTAAATCTTTTATACTAGGTAATCTAGTATCCTTATGCATGAAGATACTCAATTCGGTCGTTGTGGTCGGACTCTATTATGGATTTCTGACCACATTTTCCATGGGGCCCTCTTATCTCTTCCTTCTCCGAGCTCGGGTTATAGAAGAAGGAGAAGAAGGAACTGAGAAGAAGGTATCAGCAACAACAGGTTTTATTACGGGACAGCTCATGATGTTCATATCGATCTATTATGCGCCTCTGCATCTAGCATTGGGTAGACCTCATACAATAACTGTCCTAGCTCTACCCTATCTTTTGTTTCATTTCTTCTGGAATAATCACAAACACTTTTTTGATTATGGATCTACTAACAGAAATTCAATGCGTAATCTTAGCATTCAATTTGTATTCCTGAATAATCTAATTTTTCAATTATTCAACCATTTCATTTTACCAAGTTCAATGTTAGTCAGATTAGTCAACATTTATATGTTTCGATGCAACAACAAGATGTTATTTGTAACAAGTAGTTTTGTTGGTTGGTTAATTGGTCACATTTTATTCATGAAATGGGTTGGATTGATATTAGTCTGGATACAGCAAAATAATTCTATTAGATCTAATGTACTTTTTCGATCTAATAAGTACCTTGTGTCAGAATTGAGAAATTCTATGGCTCGAATCTTTAGTATTCTCTTATTTATTACCTGTGTCTACTCTTTAGGCAGAACACCGTCACCCATTTTTACTAAGAAACTGAAAGAAACCTCAGAAACGGAAGAAAGCGAGGAAGAAACAGATGTAGAAACAACTTCCGAAACGAAGGGGACTAAACAGGAACAAGAGGGATCCACCGAAGAAGATCCTTCTTCTTCCCTTTTTTCGGAAGAAAAGGAGGATCCGGACAAAATCGACGAAACGGAAGAGGTCCAAGTGAATGGAAAGGAAAAAACAAAGGATGAATTCCATTTTCACTTTAAAGAGACATGCTATAAAAATAGACCACTTTATGAAACTTTTTATCTGGATGGGAATCAAGAAAATTCGAAGTTAGAAATATTGATAGAAAAAAAAAATAAAGATCTGTTATGGGTTGAAAAACCTCTTGTAACTATTCTTTTTGATTCTAAACGTTGGAATCGTCCATTTCGATATATAAAAAATCATCAGTTTGAGAATGCTTTAAGAAGAGAAATGGGACAATATTTTTTTTCTACATGTCTAAGTGATGGAAAAGAAAGAATATCTTTTATGTATCCACCCAGTTTGTCAACTTTTTTGGAACTGATACAAAGAAAGATGTCTCTGTTCATAACACACAAATTTTCCTCTGATGAATTGTATAATCATTGGAATTCCAAGAATGAAAAAAAAAAAAAAAATCGAAGTAATGAATTTTTAAATAGAGTCCAAGCTCTGGATAAAGGCTATATTGCTTCGAAAACATTGGAAAAAAAGACTCGATTGTGTAATGATAAAACTAAAGAACAGTACTTACCTAAAACATATGATCCCTTATTGAATGGATCCTACCGAGGAAAAATACAGTTTTTTTTTTCATCCTCAATCCGAAATAAAACTTTCCGAACAATTTTTAGAGAAAGATTTTGGATAAATAAAATTCATCTTATTCTTCTTATTACTAATTATCAAGAATTTGAAACAAAAAGGAATGTGTTTAATAGTAATCATGAATCGTTTTTAAGAGAAATTGCTTATTTATTAAACTTAATTAATGAATTTGGCGGAAAATCAAGATCAAGTTTCAATTTTAAGGAACTCCCTTTTTTCCCAGATAACACCGAAGAAAAAGTGTATTTAGAAAATCAAATAAAAATTTTACAATTTTTATTTGATACAGTTATAGCGAATCCAAAAAAGAAAACAAGTAAAAAATTTTCTACTGGACTAAAAGAAATAAGTAAACAAGTTCCTCGATGGTCATACAAATTAATTGACGATTTAGAACAACAAGAGGGCAAAGATGATGCAGAAAACCTGGCGGAAGATCATGAAATTCGTTCACGAAAAGCCAAACTTCTAATGATTTTTAGTGATAATATAATTATTTTTAATGATAATCAAAAAAATAGTGATACTTACAATAATAACAGGAATTCGGACCCAATATACATAGACCAAGTTACTTTCATCCGTTATTCCCAACAATCGGACTTTCGGCGAGACATAATAAAAGGATCCATGCGAGCACAAAGACGTAAAATAACTATTTTTGAACTATTTCAAGCAAATGTACATTCTCCTATTTTTTTGGACAGAATAAAAAAATCTTTTTTTTTTTCTTTTGATATTGATATTTATGAACTGATGAAAACATTGTTTATAAATTCTATATCTAAAAACACAGAATTAAAAATTTCGAATTCTACTTATATAGAGAAAAAAAAAAAAAAAAGAAAGAAAAAAGAGGAGGCCAAAAGAAAAGATAACAAAAGAGAGGACAAAGCACGAATAAAAATAGCTGAAACTTGGGATAGGGTTTTTCTTGCTCAAGTACTAAGAGGTTGTGTTTTAATAACCCAATCAATTCTTCGAAAATATATTATATTACCCTCATTAATAATAACTAAGAACATCATTCGTATATTATTTTTTCAAACTCCCGAATGGTCCGACGATTTAAAAGATTGGGGTAGAGAAATGCATGTTAAATGCACCTATAATGGAGTTCAATTATCAGAAAAAGAATTTCCGAAAAATTGGTTAACGGGTGGGATTCAAATAAAGATCCTATTTCCTTTTCGTTTAAAACCTTGGCACAGATCGAAGGTAAAATTCCCTCATAAAAGTAAAAAAAAAAAGAAAATAGAACAAAAGGATTTTTGTTTTTTAACAGTTTGGGGAATGGAAGCGGAACTTCCTTTTGGTTCTCCCCGAAAACGGCTTTCACTTTTTAAACCCATCTTTAAAAAACTTGCAAAAAAAATTATAAAGATAAAAAAAAGTGGTTTTCGAGTTATAAGAATTTTCGAAGAAAGAAGAAAATTATTTCAAAATTTATCAAAAGAAAAAAAACACTCGGTCATCAAAAACATTTTTTTTCGACAAGAAATAATAACGAAACTTTCAAAATCAAAAAGAAATCTAAAATTTTTATCGGAATTTAGAGAAGTAGATGAATTAAATGAAAGTAAAAAAAAAAAAGAGTCGATACTCAATAATAAGAATCGGACGGTTTTGAAATTGTCCACCCCAATTCGACCTATACCTTCTACAAATTATTCACTGATGAAAAAAAAAAAGAAAGATCTTTCTACTAGAAAAAAGAGAATTCTAAATCAAATAGAAAAAATTACAAAAGAAAAGGAAAAAAAAATGCGAACCTCAGAAGTAAATATTAGTCTTAACAAAAAAAAAAAAAGTTCTAATGCTAAAAAAATTAAATCATCAAACAATATTTCACACATATTAAAAAAAAAAAATGTTCGATTAGTGCGTAAATTTTACTTTTTTATAAAATTTTTGATTGAAAATATATACTTAGATATCTTTTTAAGTATCATTAATATTCCAAGGCTCAATGCACAGCTTTTTCTTGAATCAATAAAAAATTTTATTACTAAACACATTTGCAATAATGAATCAAATCACAAAAAAATTGATAAACCAAATCAAAAAAATTTTTACTTTATTTCGATTATAAAAAAGTCAACAGATACAGAGATTGCTGTTATTAATAGGAATTCACAAATTTTTTGTGACATAGCTTTCTTATCACAAGCGTATGTATTTTACAAATTATCACAAACCAAAATTCTTAACTTCTATAAGTTAAGATCGATCTTTCAATACCATAATCTTTTTCTTAAGAACGAAATAAAAGATTATTTTCGAACCCAAGGATTATTTAATTTTGAATTAAAAGAAAACAAAATTCAAAAGTCTTTTTCTTTAATCAATCAATGGAAAAACTGGTTAAGAAGTCATTATCAATATAAATATGATTTATCTCAGCTTAGATGGTCTAGATTAATACCAGAAAAATGTCGAAATAGAATCTATCAACACCATATGGTTGAAAATAAAAAATTAAGCAAATGGGATTTACATGAACAAAACGAATTAATTCATTATGAAAAAAAAAATAATTTTGAGGCAGACGTTTTTGCGAATCAAAAGAATAATTTTAAAAAACACTCTAGATATAGTCTTTTATCCTATAAATCTATTAATTCTGAAAAAAAGAAGGACTTATTTATTTACGAATCACCAACTAATAAAGAAGAGATTCTTTATAATTCTAACACAAATAAACGAAAATTTTTTGACATCTTAGAAGGTATTCCTATCACTAATTATATAGTGGAAGATGATATTATCAATATAGATAAAAACCCACATAGAAAATATTTTGATTGGCGAATTATCAATTTTTGTCTTAGAAACAGGGTCGATATTGAGTCCTGGATCGATACCGGAAGCAAAAAAAAAAAAAAGACTAAGACTACAACTAAAAAATATCAAATAATTGATAAAAGTGATAAGAAAAATATTTCTTTTCTTCCAATTTGCCAAGATCAAGAAATCAATTCATCCAACCAAAAACCTTTTTTTTTTGATTGGATGGGAATGAATGAAGAAATAGAAAATAGTCTTCTTTCGAATTTTGAACTTTGGTTCTTTCGAAAATTTGTAATACTTTACAACACATATAAGAGAAAACCATGGACAATACCCATTCAATTTCTTCTTTTAAATTTTCATAGAACTAAAAATATTAGTAAAAATAAGAAAATCAACGGGAATAAAAAAGGCGACCTTCTTATATCTATACCATCACCATCGAATGAAAAAAAAATTATTGAATTCGAAAATCAAAATCATCAAGAAAACGAATCTGACGACCAAATGGATTTTCAAGCAGTTTTCACAAATGAAGAAAAAGATATTGAAGAAGATTCTATGGGATTAGATATGAAAAACCATAGAAATCAAAATCAAAACAAAAGTCATACGGAAGTAGAGCTTGATTTCTTCCTAAAAGAGTATTTATATTTTCAATTAAGATGGAATGGTTCTTTAAATCAAAAAATAATTGATAATATCAAAACATATTGTTTTCTGCTTAGACTAAGAAATCCACGCGAAATTATTATATCTTCTATTCAAAGGCAAGAAATAAATCTGAATATTCTGATGGTTCAGAAAGATATAACTCTTACAGAATTGATGAAAAAGAGAATATTGATTATCGAACCTGTCCGTCTGTCGGTAAAAACTGATGGACAATTTATTTTATATCAAATGGTAGATATTTTATTAGTTCATAAGAACAAAGAACAAATTAATAAAAAATATAGAAAACAATTCTATGTTGATAAAAATAAAAAGAATTTTACCGAATCTATTGACAGCTATCACAATATAATTGGAAATAGACAAAAAAATGATTATGATTTACTTGTTCCTGAAAATATTTTATCCCCTAAATGTCGTAGAGAATTAAGAATTCGAATTTCTTTCAATTTACAAAATAAAAACGATATTCATATAAATACAGAAATTTGCAATGGGAATAACATAAAAAAAGTCAGTCCCATTTTGGATAAAAGCAAACTTTTTTGGAGAGAAAAAAAGAAACTAATTAAATTGAAATTTTGTCTTTGGCCAAATTTTCGATTAGAGGATTTAGCTTGTATGAATCGCTATTGGTTCGATACTAATAATGGAAGTCGGTTCAGTATGTTAAGGATATATATATATCCGCGCTTGAAATTTTAG

>lcl|NC_033910.1_cds_YP_009348411.1_80 [gene=rps7] [locus_tag=B2L33_pgp007] [db_xref=GeneID:31082769] [protein=ribosomal protein S7] [protein_id=YP_009348411.1] [location=147141..147608] [gbkey=CDS]
ATGTCACGTCGAGGTACTGCAGAAGAGAAAACTGCAAAATCCGATCCAATTTATCGTAATCGATTAGTTAACATGTTGGTTAACCGTATTCTGAAACACGGAAAAAAATCATTGGCTTATCAAATTATCTATCGAGCCATGAAAAAGATTCAACAAAAGACAGAAACAAATCCACTATCTGTTTTACGTCAAGCAATACGTGGAGTAACTCCCGATATAGCAGTAAAAGCAAGACGTGTAGGCGGATCGACTCATCAAGTTCCCATTGAAATAGGATCCACACAAGGAAAAGCACTTGCCATTCGTTGGTTATTAGGGGCATCCCGAAAACGTCCGGGTCGAAATATGGCTTTCAAATTAAGTTCCGAATTAGTGGATGCTGCCAAAGGGAGTGGTGATGCCATACGCAAAAAGGAAGAGACTCATAGAATGGCAGAGGCAAATAGAGCTTTTGCACATTTTCGTTAA

>lcl|NC_033910.1_cds_YP_009348392.1_61 [gene=rpl22] [locus_tag=B2L33_pgp026] [db_xref=GeneID:31082827] [protein=ribosomal protein L22] [protein_id=YP_009348392.1] [location=complement(90067..90453)] [gbkey=CDS]
ATGATAAATAAAAAAATAGACCCGTACACAGAAGTATATAGTTTAGGACAACATATACGTATGTCCGCTCACAAAGCACGAAGAATAATCGATCAAATTCGTGGACGTTCTTACGAAGAAACACTTATGATACTAGAACTAATGCCTTATCGAGCATCTTATCCCATTTTAAAATTGATTTCTTCTGCAGCAGCAAATGCTAGTCACAATATGGCTTTCAACGAAACCGATTTAATGATTAGTAAAGCCGAGGTTAACGACGGTACTACTGTGAAAAAATTAAAACCTCAGGCTCGAGGACGGGGTTATCTAATAAAAAAATCAACTTGTCATATAACTATTGTATTAAAAAATATATCCTTAGAAAAAGAATATGAGGATTCTTAA

>lcl|NC_033910.1_cds_YP_009348356.1_25 [gene=ndhJ] [locus_tag=B2L33_pgp063] [db_xref=GeneID:31082742] [protein=NdhJ] [protein_id=YP_009348356.1] [location=complement(52400..52876)] [gbkey=CDS]
ATGCAGGGTCCTTTGTCTGCTTGGCTAGTCAAACATGGACTAGTTCATAGATCTTTGGGTTTTGATTACCAAGGAATAGAGACTTTACAAATAAAGCCCGAAGATTGGCATTCCATTGCTGTCATTTTATATGTATATGGTTACAATTATTTGCGTTCACAATGTGCCTATGATGTAGCACCCGGCGGACTTTTAGCTAGCGTATATCATCTTACGAGAATAGAGTATGGTATAGATCAACCAGAAGAAGTATGTATAAAAGTATTTGTCCCAAGAAAGAATCCTAGAATTCCATCTGTTTTCTGGATTTGGAAAAGTGTGGATTTTCAAGAAAGGGAATCCTATGATATGCTGGGAATCGTTTATGAGAATCATCCACGTCTGAAACGTATCTTAATGCCGGAAAGTTGGATAGGGTGGCCCTTACGTAAGGATTATATTGCTCCCAATTTTTATGAAATACAAGATGCTCATTGA

>lcl|NC_033910.1_cds_YP_009348366.1_35 [gene=petA] [locus_tag=B2L33_pgp053] [db_xref=GeneID:31082803] [protein=PetA] [protein_id=YP_009348366.1] [location=66927..67889] [gbkey=CDS]
ATGCAAACTCGAAAAACGTTTTCTTGGATAAAGGAAGAGATTACTTATTCCATTTCCATATCACTTATGATATGTATAATAACTTGGGCATCCATTTCAAATGCATATCCCATTTTTGCACAGCAAGGTTATGAAAATCCACGCGAAGCAACTGGCCGTATTGTATGTGCCAATTGTCATTTAGCTAATAAACCGGTAGATATTGAGGTTCCACAAGCGGTACTTCCTGATACTGTATTTGAAGCAGTTGTTCGAATTCCTTATGATATGCAACTGAAACAAGTTCTTGCTAATGGAAAAAAGGGGGCTTTGAATGTAGGAGCTGTTCTTATTTTACCTGAGGGGTTTGAATTAGCCCCTTCCAGTCGTATTTCGCCAGAGATTAAAGAAAAGATAGGAAATCTGTCTTTTCAGAGTTATCGCCCCACTAAAAAAAATATTCTTGTGATAGGTCCTGTTCCTGGTCAGAAATATAGTGAAATTACCTTTCCGATTCTTTCTCCGGACCCCGCCACTAAGAAAGATGTTTACTTTTTAAAATATCCCATATATGTAGGCGGAAACAGAGGAAGGGGTCAGATTTATCCCGACGGGAGCAAGAGTAACAATACGGTTTATAATGCTACAGCCGCAGGGATGATAAGCAAAATAATACGAAAAGAAAAAGGGGGGTACGAAATAACCATAACAGATGCGTCAGAGGGACGTCAAGTGAGTGATATTATACCTCCAGGACCGGAACTTCTTGTTTCAGAAGGCGAATCCATCAAAGTTGATCAACCATTAACAAGTAATCCTAATGTAGGTGGATTTGGTCAGGGGGATGCGGAAATAGTACTTCAGGCCCCATTACGTGTCCAAGGCCTTTTGTTCTTCTTGGCATCCGTTATTTTGGCACAAATCTTTTTGGTTCTTAAAAAGAAACAGTTTGAGAAGGTTCAATTGTCCGAAATGAATTTTTAG

>lcl|NC_033910.1_cds_YP_009348360.1_29 [gene=ATPB] [locus_tag=B2L33_pgp059] [db_xref=GeneID:31082797] [protein=AtpB] [protein_id=YP_009348360.1] [location=complement(56881..58377)] [gbkey=CDS]
ATGAGAATCAATCCTACTACTTCTGGTCCGGGAGTTTCGACGCTTGAAAAAAAGAACCTGGGGCGTATCGTTCAAATCATTGGGCCAGTACTAGATGTAGCTTTTCCCCCGGGCAAGATGCCTAATATTTACAACGCTTTAGTAGTTAAGGGTCGAGATACTGTCGGTCAAGAAATTAATGTGACTTGTGAAGTACAACAATTATTAGGAAATAATCGAGTTCGGGCTGTAGCTATGAGTGCTACAGATGGTCTAACGAGAGGAATGGAAGTGATTGACACAAAAGCTCCTCTAAGTGTTCCAGTCGGTGGGACGACTCTAGGACGAATTTTCAACGTGCTTGGAGAACCTATTGATGATTTAGGTCCTGTAGATACTCGTGCAACATCCCCTATTCATAGATCTGCACCTGCCTTTATACAGTTAGATACAAAATTATCTATTTTTGAAACAGGAATTAAAGTAGTAGATCTTTTAGCCCCTTATCGCCGTGGAGGAAAAATCGGACTATTCGGCGGGGCTGGAGTGGGTAAAACAGTCCTTATTATGGAATTAATCAACAACATTGCGAAAGCTCATGGGGGTGTATCTGTGTTTGGCGGAGTAGGTGAACGTACTCGTGAGGGAAATGATCTTTACATGGAAATGAAAGAATCTGGAGTAATTAATGAAAAAAATATTGCAGAATCAAAAGTAGCTCTAGTCTATGGTCAGATGAACGAACCGCCGGGAGCTCGTATGAGAGTTGGTTTGACTGCCCTAACTATGGCGGAATATTTCCGAGATGTTAATGAACAAGACGTACTTCTATTTATTGACAATATTTTCCGTTTCGTTCAAGCAGGATCCGAAGTATCGGCCTTATTGGGTAGAATGCCTTCCGCAGTGGGTTATCAACCTACCCTTAGTACCGAAATGGGTTCTTTACAAGAAAGAATAACTTCTACCAAAGAAGGGTCTATAACTTCTATTCAAGCCGTTTATGTACCTGCCGACGATTTGACTGACCCTGCTCCTGCCACCACATTTGCACATTTAGACGCTACTACTGTACTATCAAGAGGATTAGCCGCTAAAGGTATCTATCCAGCAGTAGATCCTTTAGATTCAACGTCAACTATGCTCCAACCTCAGATTGTTGGTGAGGAACATTATGAAACTGCGCAAAGAGTTAAGCAAACTTTACAACGTTACAAAGAACTTCAGGACATTATAGCTATCCTGGGGTTGGACGAATTATCCGAAGAAGATCGCTTAACTGTAGCAAGAGCGCGAAAAATTGAACGTTTCTTATCACAACCCTTTTTCGTAGCAGAAGTATTTACCGGTTCGCCGGGGAAATATGTCGGTCTAGCAGAAACAATTAGAGGGTTTAAATTGATCCTTTCCGGAGAATTAGATAGTCTCCCTGAACAGGCCTTTTATTTGGTAGGTAATATCGATGAAGCTACTGCGAAGGCTACGAACTTAGAAATGGAGAACAATTTGAAGAAATGA

>lcl|NC_033910.1_cds_YP_009348408.1_77 [gene=ndhH] [locus_tag=B2L33_pgp010] [db_xref=GeneID:31082843] [protein=NdhH] [protein_id=YP_009348408.1] [location=complement(127834..129015)] [gbkey=CDS]
ATGAATATACCAGCTAAACGAAAAGACCTTATGATAGTCAATATGGGTCCCCAGCACCCATCAATGCACGGTGTTCTTCGACTCATTGTTACTCTAGATGGTGAAGATGTTATTGACTGTGAACCAATATTAGGTTATTTACACAGAGGAATGGAAAAAATTGCGGAAAATCGAACAATTATACAATATTTGCCCTATGTAACACGGTGGGATTATTTAGCTACTATGTTCACAGAAGCAATAACAGTAAATGGTCCCGAATTGTTAGGAAATATTCAAGTGCCCAAAAGAGCTGGCTATATCAGAGTAATTATGTTGGAATTAAGTCGTATAGCTTCTCATTTGTTATGGCTTGGACCTTTTATGGCAGATATTGGTACACAGACGCCTTTCTTCTATATTTTTAGAGAGAGAGAGTTAATATATGATTTATTTGAAGCTGCCACTGGTATGAGAATGATGCATAATTATTTTCGTATCGGGGGGGTAGGGGCTGATCTACCTCATGGTTGGATAGATAAATGTTTAGATTTTTGCGATTATTTTTTAACAGGAGTTGATGAATATCAAAAACTTATTACGCGAAATCCTATTTTTTTAGAACGAGTTGAAGGAGTCGGTATTGTTGGTGCAGAGGAAGCAATAAATTGGGGTTTATCGGGACCAATGCTACGGGCTTCCGGAATACAATGGGATCTTCGTAAAGTTGATCATTATGAGTCTTACGACGAATTTGATTGGGAAGTCCAGTGGCAAAAAGAAGGAGATTCATTAGCTCGTTATTTAGTCCGAATTGGTGAAATGATGGAATCTATAAAAATTATTCAACAGGCTCTTGAAGGAATTCCGGGTGGGCCCTATGAGAATTTAGAAACCCGACGTTTTGATAGAGAAAAGGATCCAGAATGGAACGATTTCGAATATCGATTCATTAGTAAAAAAACTTCACCTACTTTTGAATTACCGAAACAAGAACTTTATGTCAGAGTGGAAGCCCCAAAAGGAGAATTAGGAATTTTTCTGATAGGGGATCAGAGCGGCTTTCCTTGGAGATGGAAAATTCGCCCGCCGGGTTTTATCAATTTGCAAATTCTTCCTGAATTAGTTAAAAGAATGAAATTGGCTGATATTATGACAATACTAGGTAGTATAGATATCATTATGGGAGAAGTTGATCGTTGA

>lcl|NC_033910.1_cds_YP_009348343.1_12 [gene=rpoC2] [locus_tag=B2L33_pgp076] [db_xref=GeneID:31082780] [protein=RpoC2] [protein_id=YP_009348343.1] [location=complement(18436..22626)] [gbkey=CDS]
ATGGAGGTACTTATGGCCGAGCGGGCCAATCTGGTCTTTCACAATAAAGTGATAGATGGAACTGCCATTAAACGACTTATTAGCAGATTAATAGATCATTTTGGAATGGCATATACATCCCACATCCTAGATCAAGTAAAGACTCTGGGTTTCCAGCAAGCCACTGCTACATCCATTTCATTAGGAATTGATGATCTTTTAACAATACCTTCTAAGGGATGGCTAGTCAAAGATGCTGAACAACAAAGTTTGGTTTTGGAAAAACACTATCATTATGGAAATGTACACGCGGTAGAAAAATTACGCCAATCTATTGAGATATGGTATGCTACAAGTGAATATTTGCGACAAGAAATGAATCTTAATTTTAGGATGACGGAACCCTTTAATCCAGTCCATATAATGTCTTTTTCGGGAGCTAGGGGAAATGCATCTCAAGTACACCAATTAGTTGGTATGAGAGGATTAATGTCGGATCCACAAGGGCAAATGATTGATTTACCCATTCAAAGCAATTTACGCGAAGGACTGTCTTTAACAGAATATATCATTTCGTGTTATGGAGCCCGAAAAGGGGTTGTCGATACTGCTGTACGAACATCAGATGCTGGATATCTTACGCGTAGACTTGTTGAAGTAGTTCAACATATTGTTGTACGTAGAAAAGATTGTGGCACTACCCGAGGGATCCCTGTGAGTCCTCGAAATGGCATGATGTCGGAAAGAGTTTTTATTCAAACATTAATTGGTCGTGTAATAGCAGACAATATATATATGGGTCTACGATGTATTGCCATTCGAAATCAAGATATTGGGATTGGACTTGCGAATCGATTCATAACCTTTCGAACACAAACAATATGTATTCGAACTCCCTTTACTTGTAAGAGTACGTCTTGGATCTGTCGATTATGTTATGGCCGGAGTTCTACTCACGGCGATCTAGTAGAATTGGGAGAAGCCGTAGGTATTATTGCGGGTCAATCCATTGGGGAGCCAGGTACTCAACTAACATTAAGAACGTTTCATACTGGCGGAGTATTCACAGGTGGTACTGCAGAACACGTACGATCCCCGTCTAATGGAAAAATAAAATTTAATGAGGATTTGGTTCATCCCATACGTACACGTCATGGGCATCCTGCGTTTCTATGTTATATAGACTTGTATGTAACTATTGAGAGTCAAGATATTCTACATAATGTGCCTATTCCACACAAAAGTTTCCTTTTAGTTCAAAATGATCAATATGTAGAATCAGAACAAGTAATTGCTGAAATTCGGACGGGCACATACACTTTGAATTTTAAAGAAAAGGTTCGAAAACATATTTATTCCGATTCAGAAGGGGAAATGCACTGGAGTACTCATGTATACCATGCGCCCGAATTTACATACAGTAATGTCCATCTCTTACCAAAAACAAGCCATTTATGGATATTATCAGGATGTTCGTACAGATCCGGTATAGTTCCTTTTTCACTACACAAGGATCAAGACCAAATGAACGTTCATTCTCTTTCTGCCAAAAGAAAAGATAGTTCTAGTCCTTCCCTAAACAATCATCTCCTAAATAATAATCAAGTTAAACACCAATTCTTTAGTTCAGATTTAGTGGGTAAAAAAGAAAGTAGGATTCCTGATTATTCAGTAGTTAATCGAATCATATGTACTGGTCATTGTAATCTCATATATCCTGCTATTCTCTACGAGAATTCTGATTTATTGGCAAAGAGGCGAAGAAATAAATTCATTATTCCATTCCAATCAATTCAAGAACGAGAGAAAGAACAAATGACCTACTACCCTATCTCGATTGAAATACCTAAAAATGGTATTTTCCGTAGAAATAGTGTTTTTTCTTATTTCGACGATCCCCAATACCGAAGAAAGAGTTCAGGAATTACTAAATACGGCGCTATAGGGGTCCATTCAATCGTCAAAAAAGAAGATTTGATTGAGTATCGGGGAGTCAAAGAATTTAAGCCAAAATACCAAATGCAAGTGGATCGCTTTTTTTTCATTCCCGAGGAAGTGTATATTTTACCCGAATCTTCTTCCCTAATGGTACGGAACAATAGTATCGTTGGAGTAGATACACAAATCACTTTAAATACAAGAAGTCGAGGGGGCGGATTGGTCCGAGTGGAGAGAAAAAAAAAAAAAATAGAACTTAAAATCTTTTCTGGAGGTATCCATTTTCCGGGAGAGACAGATAAGATATCCCGACATAGTGGTATCTTGATACCACCAGGAACGGTAAAAACAAATTATAAGGAATCAAAAAAAGTCAAAAATTGGATCTATATTCAACGAATCACACCTACCAAAAAAAAGTATTTTGTTTTGGTTCGACCAGTAATCATATATGAGATAGCGAATGGTATCAATTTAGAAACACTTTTCCCCCAGGATCTATTGCAGGAAAAGGATAATCTGAAACTTCGAGTTGTCAATTATATTCTTTATGGAAATAGTAAACCAATTTGGGGAATTTCTGACACAAGTATTCAATTAGTTCGTACTTGTTTAGTGTTGAATTGGGACCAAGACAAAAAAGGTTCTTCTATCGAGGAGGCCCGCGCTTCTTTTGTTGAAGTAAGCACAAATGGTCTGATTCGTGATTTCCTAAGAATCAATCTATTGAAATCAAAAATTTCATATATCAGTAGTAGAACTACAAAAAGGAATGATCCATCAGGTTCAGGACCGATCTCTAATAATGGATCAGATCGCACCAATATTAATCCATTTTATCCCAGTTATTCCAAGACAAGGATTCAACAATCACTTAAACAAAATCACGGAACTTTTAGTACGTTGTTGAATAGAAATAAGGAATGTCAATCTTTCCTAATTTTGTCATCATCTAATTGTTTTCGAATGGATCCATTCAATGATGTAAAACATCACAATGGAATAAAAGAATCAATTAAAAGAGATCCTATAATTTCAATTAGAAATTCGTTTGGCCCTTTAGGAACAGCCCTTCAAATTGCGAATTTTTTTTTATTAAAACATTTCAATTTAATAACTCATAATCAGATCTCGATAACTAAATATTTGAAACTTGACAATTTAAAACAGACTTTTCAAATAATTAAATATTATTTAATGGATGAAACCGGGCGAATTGTTAATCCTGATCCATGCAGTAAGAGTGTTTTGAATCCATTCACTTTGAATTGGTATTTTCTCCATCATAATTATCATCGTAATTATTGTGAATCTTTCTTCACAATAATTAGACTGGGACAATTTATTTGTGAAAATTTATGTATGGCCAAAAGAGGACCACATCTAAAATCGGGTCAAGTTATAATTGTTCACATTGACTCTGTAGTAATAAGATCGGCTAAGCCTTATTTGGCCACTCCAGGAGCAACTGTTCATGGCCATTATGGAGAAATCCTTTACGAAGGAGATACGTTAGTTACATTTATATATGAAAAATCGAGATCTGGTGATATAACGCAGGGTCTTCCAAAAGTGGAACAAGTGTTAGAAGTGCGTTCAATTGATTCAATATCGATAAACCTAGAAAAGAGAGTTGAGGGTTGGAACGAGTGTATAACAAGACTTCTTGGAATTCCTTGGGGATTCTTGATTGGTGCTGAGTTAACTATAGTGCAAAGTCGTATCTCGTTGGTTAACAAAATCCAAAAAGTTTATCGATCTCAAGGAGTGCAGATACATAATAGGCATATAGAAATTATTGTACGGCAAATAACATCAAAAGTATTGGTTTTAGAAGATGGAATGTCTAATGTTTTTTTACCCGGAGAACTAATTGGATTGTTCCGAGCAGAACGAACGGGACGCGCTTTGGAAGAAGCCATCTGTTACCGACCCATATTATTGGGAATAACGAGAGCATCTCTGAATACTCAAAGTTTCATATCTGAGGCCAGTTTTCAAGAAACTGCTCGCGTTTTAGCAAAAGCTGCTCTCCGCGGTCGTATCGATTGGTTGAAAGGCCTAAAAGAAAACGTTGTTCTAGGCGGTATGATACCCGTTGGTACCGGATTCAAAAGATTCGTGCAAGGCTCAAAGAAACAGAAAAACATGCCTTTGAAAAGCAAAAATAAAAATTTTTTTGAGGAGGAATTTAGAGATAGAGATCTTTTATTCCACCACAGAGAGTTATTTGATTCTTGCATTTCAAGAAATTTCTATGATACATTAGAATAA

>lcl|NC_033910.1_cds_YP_009348407.1_76 [gene=ndhA] [locus_tag=B2L33_pgp011] [db_xref=GeneID:31082842] [protein=NdhA] [protein_id=YP_009348407.1] [location=complement(join(125625..126163,127280..127832))] [gbkey=CDS]
ATGATAATTGATACAACAGAAGTACAAGCTATCCATTCTTTTTCTAGGTTAGAATCCTTAAACGATGTCTATGGAATTATATGGGAGTTTATTCCTATTTTGATTCTTGTATTGGGAATCACGATAAGCATACTCGTAATTGTATGGTTAGAAAGAGAAATATCCGCAGGGATACAACAACGTATTGGACCCGAATATGCGGGTCCTTTAGGAGTTCTTCAAGCTCTAGCGGATGGTACAAAACTACTTTTCAAAGAGAATCTTTTTCCATCTAGGGGGGATACTTATTTATTCAGTATTGGACCATCTATAGCAGTCATATCAACTCTATTAAGCTATTCAGTAATTCCTTTTGGCTATCACTTTGGTTTAACTGATCTAAATATTGGTGTTTTTTTATGGATTGCCATTTCAAGTATTGCTCCTATTGGACTTCTTATGTCAGGGTATGGATCAAATAATAAATATTCCTTTTTAGGTGGTCTACGAGCTGCTGCTCAATCGATTAGTTATGAAATACCTTTAACTATTTGTGTGTTATCCATATCTCTACTATCTAATAGTTCAAGTACAGTTGATATAGTTGAAGCCCAATCAAAATCTGGTTTTTGGGGTTGGAATTTATGGCGTCAACCGATAGGATTTTTTATTTTTTTTATTTCTTCTCTAGCAGAATGTGAAAGATTACCTTTTGATTTGCCAGAAGCAGAAGAAGAATTAGTAGCAGGTTATCAAACGGAATATTCGGGTATCAAATTTGGTTTATTTTATATTGCTTCCTATCTAAACTTATTAGTTTCTTCATTATTTGTAACAGTTCTTTACTTGGGCGGTTGGAATATTTCTATTCCGTATATATTCGTTCATGAATTTTTTGAAATAAATAGCATAAGCGGAGTCGTTGGACCAACAATTGGTACCTTTATTACATTAGTTAAAACTTATTTGTTCTTGTTCATTCCTATCACAACAAGATGGACTTTACCGAGACTAAGAATGGACCAACTTCTAAATCTTGGATGGAAATTTCTTTTACCTATCTCTCTCGGTAATCTATTATTAACAACCTCTTTTCAACTCCTTTCACTATAA

>lcl|NC_033910.1_cds_YP_009348359.1_28 [gene=ATPE] [locus_tag=B2L33_pgp060] [db_xref=GeneID:31082745] [protein=AtpE] [protein_id=YP_009348359.1] [location=complement(56483..56884)] [gbkey=CDS]
ATGACCTTAAATCTTTGTGTACTGACCCCAAACCGAATTGTTTGGGATTCAGAAGTGAAAGAAATCATTTTATCTACTAATAGTGGACAAATTGGCGTATTACCAAATCATGCACCAATTGCCACAGCTGTCGATATCGGTATTTTGAGAATACGCCTTAATGACCAATGGTTAACAATGGCTCTGATGGGTGGTTTTGCTCGAATAGGCAATAATGAGATTACTGTTTTAGTAAATGATGCGGAGAAGGGTAGTGACATTGATCCACAAGAAGCTCAGCAAACTCTTGAAATCGCAGAAGCTAACTTGAGGAAAGCGGAAGGAAGGAGACAAATAATTGAGGCAAATCTAGCTCTCAGACGAGCTAGGGCACGAGTAGAGGCTATCAATGCAATTTCGTAA

>lcl|NC_033910.1_cds_YP_009348338.1_7 [gene=ATPA] [locus_tag=B2L33_pgp081] [db_xref=GeneID:31082729] [protein=AtpA] [protein_id=YP_009348338.1] [location=complement(12017..13540)] [gbkey=CDS]
ATGGTAACCATTCGAGCCGACGAGATTAGTAATATTATTCGCGAACGTATTGAGCAATATAATAGGGAAGTAAAGATTGTAAATACTGGTACCGTACTTCAAGTAGGCGACGGCATTGCTCGTATTTATGGCCTTGATGAAGTAATGGCAGGTGAATTAGTAGAATTTGAAGAGGGTACAGTAGGCATTGCTCTGAATTTGGAATCAAATAATGTCGGTGTGGTATTAATGGGTGATGGTTTAATGATACAAGAGGGAAGCTCCGTAAAGGCAACAGGAAGAATTGCTCAGATACCGGTGAGTGAAGCTTATTTGGGTCGTATTGTAAATGCCCTGGCTAAACCTATTGACGGTCGAGGGGAAATTTCCGCTTCTGAATCTCGGTTAATTGAATCTCCCGCTCCAGGTATTATTTCGAGACGTTCCGTATATGAGCCTCTTCAAACAGGACTTATTGCTATTGATTCGATGATCCCTATAGGACGTGGGCAACGAGAATTAATTATTGGGGACAGACAAACCGGTAAAACAGCAGTAGCCACAGATACAATTCTGAATCAACAAGGACAAAATGTAATATGTGTTTATGTAGCTATTGGGCAAAAAGCGTCTTCTGTGGCTCAGGTAGTGACTACATTACAGGAAAGAGGAGCAATGGAGTACACTATTGTGGTAGCCGAAACTGCGGATTCTCCGGCTACATTACAATACCTGGCTCCTTATACAGGAGCAGCTCTGGCTGAATATTTTATGTACCGTGAACGACACACTCTAATCATTTACGATGATCTCTCCAAACAAGCGCAGGCTTATCGCCAAATGTCTCTTCTATTACGAAGACCACCAGGTCGTGAAGCTTATCCAGGAGATGTCTTTTATTTGCATTCACGCCTTTTGGAAAGAGCTGCTAAATCAAGTTCTCGTTTAGGTGAAGGAAGCATGACTGCTTTACCAATAGTCGAGACTCAATCAGGAGACGTTTCAGCTTATATTCCTACTAATGTAATTTCCATTACAGACGGACAAATATTCTTATCCGCCGATCTATTCAATGCTGGAATCCGCCCTGCTATTAATGTGGGGATTTCCGTTTCTAGAGTAGGATCTGCAGCTCAAATTAAAGCTATGAAACAAGTGGCGGGTAAGTTAAAATTGGAATTGGCCCAATTCGCCGAATTAGAGGCCTTTGCACAATTCGCTTCTGATCTAGATAAAGCTACTCAGAATCAATTGGCAAGAGGTCAGCGATTACGTGAGTTGCTCAAACAATCCCAATCTGCGCCTCTCACTGTGGAGGAACAGATAATGACTATTTATACCGGAACAAATGGTTATCTTGATTCATTAGAAATAGGACAAGTAAGGAAATTTCTCGTTGAGTTACGTACCTACTTAAAAACGAATAAACCCCAGTTCGAAGAAATCATATCTTCTACCAAAACATTCACCGAAGAAGCAGAAATCCTTTTGAAAGAAGCTATTCAGGAGCAGAAGGAACGTTTTCTAGTTCAGGAACAAGTATAA

>lcl|NC_033910.1_cds_YP_009348394.1_63 [gene=rpl2] [locus_tag=B2L33_pgp024] [db_xref=GeneID:31082829] [protein=ribosomal protein L2] [protein_id=YP_009348394.1] [location=complement(join(90841..91308,91933..92328))] [gbkey=CDS]
ATGGCGATACATTTATACAAAACTTCTACCCCGAGCACACGCAATGGAGCCGTAGACAGTCAAGTGAAATCCAATACACGAAATAATTTGATCTATGGACAGCATCATTGTGGTAAAGGCCGTAATGCCAGAGGAATAATTACCGCAAGGCATAGAGGGGGAGGTCATAAGCGTCTATACCGTAAAATCGATTTTCGACGGAATGAAAAAGACATATATGGTAGAATCGTAACCATAGAATACGACCCTAATCGAAATGCATACATTTGTCTCATACACTATGGGGATGGTGAGAAGAGATATATTTTACATCCCAGAGGGGCTATAATTGGAGATACCATTATTTCTGGTACAGAAGTTCCTATAAAAATGGGAAATGCCCTACCTTTGAGTGCGGTTTTGATTGATCAAAAAGAAGAATCTACTTCAACCGATATGCCCTTAGGCACGGCCATACATAACATAGAAATCACACTTGGAAAGGGTGGACAATTAGCTAGAGCTGCAGGTGCTGTAGCGAAACTGATTGCAAAAGAGGGTAAATCGGCCACATTAAAATTACCTTCTGGGGAGGTTCGTTTAATATCCAAAAACTGCTCAGCAACAGTCGGACAAGTAGGGAATACTGGGGTGAACCAGAAAAGTTTGGGTAGAGCCGGGTCTAAATGTTGGCTAGGTAAGCGTCCTGTAGTAAGAGGAGTGGTTATGAACCCTGTAGACCATCCCCATGGGGGTGGTGAAGGGAGGGCCCCAATTGGTAGAAAAAAACCCGCAACCCCTTGGGGTTATCCTGCACTTGGAAGAAGAAGTAGAAAAAGGAATAAATATAGTGATAATTTGATTCTTCGTCGCCGTAGTAAATAG

>lcl|NC_033910.1_cds_YP_009348389.1_58 [gene=rpl14] [locus_tag=B2L33_pgp029] [db_xref=GeneID:31082824] [protein=ribosomal protein L14] [protein_id=YP_009348389.1] [location=complement(86923..87291)] [gbkey=CDS]
ATGATCCAATCTCAGACCCATTTGAATGTAGCAGATAACAGTGGAGCCCGAGAATTGATGTGTATTCGAATCATAGGGACTAGTAATCGCCGATATGCTCATATCGGTGACGTTATTGTTGCTGTGATCAAGGAAGCAGCACCCAATTCACCTCTCGAAAGATCAGAAGTAATCAGAGCTGTAATTGTACGTACTTGTAAAGAACTTAAACGTAATAACGGTATAATAATACGATATGATGACAATGCTGCAGTTGTCATTGATCAAGAAGGAAATCCAAAAGGAACACGAATTTTTGGTGCAATTGCCCGGGAATTGAGACAGTTAAATTTTACTAAAATAGTTTCATTAGCACCTGAGGTCTTATAA

>lcl|NC_033910.1_cds_YP_009348354.1_23 [gene=ycf3] [locus_tag=B2L33_pgp065] [db_xref=GeneID:31082791] [protein=Ycf3] [protein_id=YP_009348354.1] [location=complement(join(46660..46812,47488..47717,48465..48588))] [gbkey=CDS]
ATGCCTAGATCCCGGATAACTGGAAATTTTATTGATAAGACCTTTTCAATTGTAGCCAATATCTTATTACGAATAATTCCGACAACTTCGGGGGAAAAGGAGGCATTTACTTATTACAGAGATGGAATGTCTGCTCAATCCGAAGGAAATTATGCAGAAGCTTTACAGAATTATTATGAAGCTATGCGACTAGAAATTGATCCCTATGATCGAAGTTATATACTATATAATATAGGCCTTATTCACACAAGTAACGGAGAACACACAAAAGCTTTGGAATATTATTTTCGGGCACTAGAACGAAACCCTTTCTTACCACAAGCTTTAAATAATATGGCCGTGATCTGTCATTACCGAGGAGAACAGGCCATTCGGCAGGGAGATTCTGAAATTGCGGAGGCTTGGTTCGACCAAGCCGCGGAGTATTGGAAACAAGCCATAGCACTTACTCCCGGAAATTATATTGAAGCGCAGAATTGGTTGAAGATCACAAGGCGTTTCGAATAA

>lcl|NC_033910.1_cds_YP_009348362.1_31 [gene=accD] [locus_tag=B2L33_pgp057] [db_xref=GeneID:31082799] [protein=AccD] [protein_id=YP_009348362.1] [location=61309..62808] [gbkey=CDS]
ATGGAAAAATGGTGGTTCAATTCAATCTTATCCAATGTGGAATTAGGATACAGGTGTAGGCTAAGTAAATCAATGGATAGTTTCAGTCCTTTTGAAAATACTAGTATAAGTGAAGACCCAATTCTAAATGATACAGATAAACACACCCATAGTTGGAGTAATAGTGACAACTCTAGTTCCAGTAATGTTGATCATTTAGTCGGTGTCAGGGACATTTGGAATTTCAGCGTTGATGAAACTTTTTTAGTTAAGGATAGTAATAGGGACAGTTATTCCATCTATTTTGATATTGAAAATAAAGTTTTTGAGATTGAGACTGATTATTCTTTTCTGGATGAACTAGAAAGTTCTTTTTATAGTTATTGGAATTCTAGTTATCTGAATAATGGGTCTAGGAGTGGCGACTCCCAATATGATCATTATATGTATGATACTAAATATCGTTGGAATAATTACATCAATAGTTGCATTGACCGTTATCTTCGCTCTCAAATTGGTATTGATAGTTCTATTTTAAGGGGTAGTAACCATTATAGCGAAAGTTATATTTATAGTTACGTTTGTGATGAAAGCGAAAATAGTAGTGAAAACAAGAGTGCCAGTCTAAGAATTAGCACGAATGGTAGTGATTTAACTCTAAGAGAAAGTTCTAATGATCTCGATATAACTCAAAAATACAAACATTTGTGGGTTCAATGCGAAAATTGTTATGGATTAAATTATAAGAAATTTTTTAAGTCAAAAATGAATCTTTGTGAACAATGTGGATATCATTTGAAAATGAGTAGTTCAGATAGAATTGAACTTTTGATTGACCCAGGGACTTGGGATCCTATGGATGAAGACATGGTATCTCTGGATCCCATTGAATTTCATTCAGAAGAGGAACCTTATAAAGATCGTATTGATTCTTATCAAAGAAAGACAGGATTAACCGAGGCTGTTCAAACAGGCACAGGTCAACTAAACGGTATTCCCGTAGCAGTTGGGGTTATGGATTTTCAATTTATGGGGGGTAGTATGGGATCCGTCGTAGGTGAGAAAATTACTCGTTTGATCGAGTATGCTACCAATCAATTTATACCTCTTATTTTAGTGTGTGCTTCCGGAGGAGCACGAATGCAAGAAGGAAGTTTGAGCTTGATGCAAATGGCTAAAATATCTTCTGCTTTATATGATTATCAATCGAATAAAAAGTTATTTTATGTATCAATCCTTACGTCTCCTACAACAGGTGGGGTGACAGCTAGTTTTGGGATGTTGGGAGATATTATTATTGCTGAACCTAACGCCTATATTGCATTTGCGGGTAAAAGAGTAATTGAACAAACATTGAATAAAACAGTACCTGAAGGTTCCCAATCGGCCGAATTTTTATTCCATAAGGGCTTATTCGATTCAATCGTACCACGTAATCTTTTAAAAGGCGTTTTGAATGAGTTACTTCAGCTCCACGATTTCTTTCCTTTGAATCCTAAATCAAGTAGTGCCTTAATTTAA

>lcl|NC_033910.1_cds_YP_009348341.1_10 [gene=ATPI] [locus_tag=B2L33_pgp078] [db_xref=GeneID:31082778] [protein=AtpI] [protein_id=YP_009348341.1] [location=complement(16465..17208)] [gbkey=CDS]
ATGAATGTTCTATCATGTTCCATCAACACCCTAAAAGGGTTATATGATATATCTGGTGTGGAAGTAGGCCAGCATTTCTATTGGAAAATTGGAGGTTTCCAAGTCCACGCCCAAGTACTTATTACTTCTTGGGTTGTAATTGCTATCTTATTAGGTTCAGCCATTGTAGCTGTTCGGAACCCCCAAACCATTCCAACTGGCGGTCAGAATTTCTTCGAATATGTCCTTGAATTCATTCGAGATGTGAGCCAAACTCAGATCGGAGAGGAATACGGCCCATGGGTCCCTTTTATTGGAACTATGTTTCTATTTATTTTTGTTTCTAATTGGGCGGGTGCACTTTTACCTTGGAAGATCATAGAGTTACCTCATGGGGAGTTGGCTGCACCTACGAATGATATAAATACTACCGTTGCTTTAGCTTTACTTACGTCAATAGCCTATTTTTATGCGGGCCTTAGCAAAAAAGGATTAGGTTATTTCAGTAAATACATTCAACCAACTCCAATTCTTTTACCCATTAACATTTTAGAAGATTTCACAAAACCTTTATCACTTAGCTTTCGACTTTTCGGAAATATATTAGCGGATGAATTAGTAGTTGTTGTTCTTGTTTCTTTAGTACCTTCAGTGGTTCCTATACCTGTCATGTTCCTTGGATTATTTACAAGTGGTATTCAAGCTCTTATTTTTGCAACTTTAGCTGCGGCTTATATTGGCGAATCCATGGAGGGGCATCATTAA

>lcl|NC_033910.1_cds_YP_009348388.1_57 [gene=rps8] [locus_tag=B2L33_pgp030] [db_xref=GeneID:31082823] [protein=ribosomal protein S8] [protein_id=YP_009348388.1] [location=complement(86251..86655)] [gbkey=CDS]
ATGGGTAGGGATCCGATTGCTGACATAATAACCTCTATACGAAATGCTGACATGAATCGAAAAGAAACCGTTCGAATAGCAGCTACTAACATCACCGAAAACATTATAAAATTACTCTTACGAGAGGGTTTTATTGAAAATGTCAGGAAACATCAGGAAGGCAACAAAAAATTTTTGGTTTTAACTTTACGCCATAGAAGGAAAAAAAAAGGACCATATAGAACTAGTCTAAATTTAAAACGGATAAGCCGGCCGGGTCTACGAATCTATTCTAACTATCAAAAAATTCCTAGAATTTTGGGCGGGATGGGCATTGTAATTCTTTCTACTTCTCGGGGTATACTGACAGACCGGGAAGCTCGACTCGAAAGAATCGGCGGAGAAATCTTGTGTTATATATGGTAA

>lcl|NC_033910.1_cds_YP_009348339.1_8 [gene=ATPF] [locus_tag=B2L33_pgp080] [db_xref=GeneID:31082776] [protein=AtpF] [protein_id=YP_009348339.1] [location=complement(join(13587..14056,14723..14867))] [gbkey=CDS]
ATGAAAAATGTAACCGATTCTTTCGTTTTCCTGGGTCACTGGCCATCCGCCGGGAGTTTCGGGTTTAATACCGATATTTTCGCAACAAATCCAATAAATCTAAGCGTAGTCCTTGGTGTATTGACTTTTTTTGGAAAGGGGGTGTTTTGGTTCGGGAAGGGATCATGGAAGTTTTGCAATGAATGGAAAGATAATCTACTTTCATTAAGTGATTTATTAGATAATCGAAAACAACGGATTTTGGATACTATTCGAAATTCAGAAGAACTACGCGAGGGGGCCATTGAACAGCTGGAAAAAGCCCGGGCCCGCTTACGGAAAGTGGAAATAGAAGCGGATCTGTTTCGAACGAATGGATACTCTGAGATAGAACGAGAAAAATTGAATTTGATTAATTCAACTTATAAGACTTTGGAACAATTAGAAAATTACAAAAATGAAACCATTCAGTTTGAACAACAAAGAACGATTAATCAAGTCCGACAACGGGTTTTCCAACAAGCCTTACAAGGAGCTCTAGGAACTCTGAATAGTTGTTTGACCAACGAATTACATTTACGTACCATCAACGCGAATCTTGGCATGTTTGGGGCGATAAAAGAAATAACTGATTAG

>lcl|NC_033910.1_cds_YP_009348390.1_59 [gene=rpl16] [locus_tag=B2L33_pgp028] [db_xref=GeneID:31082825] [protein=ribosomal protein L16] [protein_id=YP_009348390.1] [location=complement(87418..87777)] [gbkey=CDS]
ATGAAAGGAATATCTTTTAGAGGCAATCGTATTTGTTTTGGAAGATATGCTCTTCAAGCACTTGAACCCGCTTGGATTACATCTAGACAAATAGAAGCGGGGCGACGAGCAATGACACGAAATGCACGCCGCGGTGGAAAAATATGGGTACGTATATTTCCCGACAAACCCGTTACTTTAAGACCTACGGAAACACGTATGGGTTCGGGGAAAGGGTCTCCCGAATATTGGGTAGCTGTGGTTAAACCAGGTAGAATACTTTTTGAAATGGGTGGAGTGGCAGAAAATATAGCGAGAAAGTCTATTTCAATAGCAGCATCCAAAATGCCTATACGAACTCAATTCCTTATTTCAAAATAA

>lcl|NC_033910.1_cds_YP_009348379.1_48 [gene=psbB] [locus_tag=B2L33_pgp039] [db_xref=GeneID:31082815] [protein=PsbB] [protein_id=YP_009348379.1] [location=78541..80067] [gbkey=CDS]
ATGGGTTTGCCTTGGTATCGTGTTCATACCGTCGTATTGAATGATCCCGGTCGTTTGCTGTCTGTCCATATAATGCATACAGCGTTGGTTGCTGGTTGGGCTGGTTCGATGGCTCTATATGAATTAGCAGTTTTTGATCCCTCTGACCCCGTTCTCGATCCGATGTGGAGACAAGGTATGTTCGTTATACCCTTCATGACTCGTTTAGGAATAACCAATTCGTGGGGTGGTTGGAATATCACAGGAGGAACTATAAGCAATCCGGGTATTTGGAGTTATGAAGGTGTGGCTGGGGCGCATATTGTGTTTTCTGGGTTGTGTTTCTTAGCAGCTATTTGGCATTGGGTGTATTGGGATCTAGAAATCTTTTTTGATGAACGTACCGGAAAACCTTCTTTGGATTTGCCCAAGATCTTTGGAATTCATTTATTTCTCTCAGGGGTAGCTTGCTTTGGGTTTGGCGCTTTTCATGTAACCGGATTGTATGGTCCTGGAATATGGATCTCCGATCCTTATGGATTAACTGGAAAGGTACAACCAGTAAGTCCAGCATGGGGTGTCGAAGGTTTTGATCCCTTTGTTCCGGGAGGAATAGCTTCTCATCATATTGCAGCAGGGACATTGGGCATATTGGCGGGTCTATTTCATCTTAGTGTCCGTCCGCCCCAACGTTTATACAAAGGATTACGTATGGGAAATATTGAAACTGTACTTTCCAGTAGTATCGCTGCTGTCTTTTTTGCAGCTTTTGTTGTTGCTGGAACTATGTGGTATGGTTCAGCAACTACCCCGATTGAATTATTTGGTCCCACTCGTTATCAATGGGATCAAGGATACTTCCAGCAAGAAATATATCGAAGAGTTAGTGCCGGGCTAGCCGAAAATCAAAATTTATCCGAAGCTTGGTCTAAAATTCCCGAAAAATTAACTTTTTATGATTACATTGGCAATAATCCTGCAAAAGGTGGATTGTTCAGAGCAGGTTCCATGGACAACGGGGATGGAATAGCTGTTGGGTGGTTAGGACATCCTATCTTTAGAGATAAAGAAGGGCGTGAACTTTTTGTACGCCGTATGCCTACTTTTTTTGAAACATTTCCAGTTGTTTTGGTAGACGGAGATGGAATTGTTAGAGCCGATGTTCCTTTTCGAAGGGCAGAGTCGAAGTATAGTGTCGAACAAGTAGGTGTAACTGTTGAGTTCTATGGTGGGGAACTAAACGGAGTCAGTTATAGTGATCCTGCTACTGTCAAAAAATATGCTAGACGTGCTCAATTAGGCGAAATTTTTGAATTAGATCGTGCTACTTTGAAATCCGATGGTGTTTTTCGTAGCAGTCCACGGGGTTGGTTTACTTTTGGACATGCTTCGTTCGCTCTGCTCTTTTTCTTCGGACACATTTGGCATGGTGCTCGAACTTTGTTCCGAGATGTTTTTGCTGGGATTGATCCAGATTTAGATGCTCAAGTGGAATTTGGAGCATTCCAAAAACTTGGAGATCCTACTACAAAAAGGCAAGTAGTTTGA

>lcl|NC_033910.1_cds_YP_009348400.1_69 [gene=ndhF] [locus_tag=B2L33_pgp018] [db_xref=GeneID:31082835] [protein=NdhF] [protein_id=YP_009348400.1] [location=complement(117200..119434)] [gbkey=CDS]
ATGGAACATATATATCAATATTCATGGATCATACCTTTCCTTACATTCCCAGTCCCTATGTTAATAGGAGCAGGACTTCTACTTTTTCCGGCGACAACAAAAAAACTTCGTCGTATGTGGGCTTTTCCAAGTGTTTTATTGTTAAGTATAGTTATGGTTTTTGCAATTGATCTGTCTATTCAGCAAATAAATAGCAGTTTTATTTATCAATATATATGGTCATGGACCATCAATAATGATTTTTCTTTAGAGTTCGGACACTTGATTGACCCACTTACTTCTATTTTGTTAATATTAATTACTACAGTTGGAATTATGGTTCTTTTTTATAGTGATAATTATATGTCTCATGATCAAGGTTATTTGAGATTTTTTGCTTATATGAGTTTTTTCAATACTTCAATGTTAGGATTAGTTACTAGTTCGAATTTGATACAAATTTATATTTTTTGGGAATTAGTTGGAATGTGTTCTTATCTTTTAATAGGTTTTTGGTTCACACGTCCTAGTGCATCGAATGCTTGTCAAAAAGCATTTGTAACTAATCGTGTAGGGGATTTTGGTTTATTATTAGGAATTCTTGGTCTGTATTGGATAACGGGCAGCTTCGAATTTCGAGATTTGTTCAAAATATTCAATAACTTGATTTATAATAATCAAGTTAATCTTGTATTCGTTACTTTGTGTACCTTTTTATTATTTTCCGGTGCAATTGCTAAATCGGCACAATTTCCTCTTCATGTATGGTTGCCCGATGCCATGGAGGGGCCTACCCCTATTTCGGCTCTGATACATGCTGCTACTATGGTAGCGGCGGGAATTTTTCTTGTAGCTCGCCTTTTTCCCCTTTTCCTAGTCATACCTTACATAATGAATCTAATAGCTTTAATAGGCATAATAACAGTCTTTTTAGCAGCTACTTTAGCCCTTGCTCAAAAAGATATTAAGAGAAGTTTAGCTTATTCTACAATGTCTCAATTGGGTTATACGATGTTAGCTCTAGGTATGGGGTCTTATCGAGGTGCTTTATTTCATTTGATTACTCATGCCTATTCGAAAGCATTGTTGTTTTTAGGGTCTGGATCTATTATTCATTCAATGGAAACTATTGTTGGTTATTCTCCAGATAAGAGTCAAAATATGGTTTTTATGGGCGGTTTAACAAAACATATTCCAATTACAAAAACTGCTTTTTTATTAGGAACGCTTTCCCTTTGTGGTATTCCACCCTTCGCCTGTTTTTGGTCCAAAGATGAAATTCTTAATGATAGTTGGTTATATTCACCTAGTTTCGCAATAATAGCTTGGTTCACTGCGGGATTAACTGCATTTTATATGTTTCGGATTTATTTACTTATTTTTGAAGGATATTTCAATCTTAATTTTAAAAATTACAGTGGAAAAAAAAACGGTTCATTTTATTCAATATCTTTATGGGGTAAAGAAGGATCAAAAACGTTTAAAAAAAATTTTCGTTTATTACCTTTATTAAAAAAAACGAATAATGACAGGACTTCCTTTTTTCGGAAGAATACATATAAAATTGATGTTAATGTAAGAAATATGAGATGGGCCTTTATTACTGTTAATAATTTTAACACTAAAAGGATTTTTTCCTACCCGCGGGAATCCGACAATACTATGTTATTTCCTATGCTTGTCTTCGTACTATTTTCTGTCTTTATTGGAGCTATAGGAATTCCTTTCAATCAATTCAATCAAGAAGAAATCAAGTTGGATATATTGTCAAAACTACTAACTCCGTCTTTTAAACTTTTGCATGAAAATGAAGAAAATTATGTGGATTGGTATGGATTTGTAATAAATGCAACTTTTTCAGTTAGTATAACTTTTTTCGGAATATTTATCGCGTCCTCCTTCTATCAGCCTGTTTATTTATCTTTACAAAATTTGAATTTCTTTAATTCATCCGCTAAAAAAGGCTTGAAGAAAATTCTTTCGGACAAAATAAAAAATCAGATATATAATTGGTCCTATAATCGAGGTTACATAGATTCCTTTTATGCAATGTCTTTTATTAGGGGTATAAGAAAATTAGCTGAATTAATTTCTTTTTTTGATAAACGAATAATTGATGGAATTATAAATGGAGTCGGTGTTATCAGTTTCTTTGTAGGAGAAAGCATAAAATATGCAGGAAGCGGTCGCATTTCTTCTTATCTTTTATTGTATGTATTTTATGCAGTAATTTTTTTATTAATTTACTATACTTAG

>lcl|NC_033910.1_cds_YP_009348344.1_13 [gene=rpoC1] [locus_tag=B2L33_pgp075] [db_xref=GeneID:31082781] [protein=RpoC1] [protein_id=YP_009348344.1] [location=complement(join(22771..24387,25163..25594))] [gbkey=CDS]
ATGATTGATCGGTATAAACATCAACAACTCCGAATTGGCTCAGTTTCGCCGCAACAAATAAGTGCTTGGGCCAATAAAATCCTACCTAACGGAGAGATTGTTGGAGAGGTGACAAAACCCTACACTTTTCATTACAAAACCAATAAACCTGAAAAAGATGGATTATTTTGTGAAAGAATTTTTGGGCCTATAAAAAGTGGAATTTGTGCTTGTGGAAATTATCGAGTAATCAAAAATGAAAAAGAAGACCGAAAATTTTGTGAACAATGCGGGGTCGAATTTGTTGATTCTCGGATACGAAGATATCAAATGGGCTACATCAAACTGGCATGCCCCGTAACTCATGTGTGGTATTTGAAACGTCTTCCTAGTTATATCGCGAATCTTTTAGATAAACCTCTTAAAGAATTAGAAGGCCTAGTATACTGCGATTTTTCTTTTGCTAGGCCCATAGCTAAGAAACCCACTTTTTTACGATTACGAGGTTCATTTCAACATGAAATCCAATCCTGGAAATACAGTATTCCACTTTTTTTTACTACCCAAGGCTTCGATACATTTCGAAATCGAGAAATTTCTACAGGAGCTGGTGCTATCCGAGAACAATTAGCTGATCTGGATTTGCGAATTATTATAGATTGTTCATCGGTAGAATGGAAAGAATTAGGGGAAGAAGGGCCCACAGGGAATGAATGGGAAGATCGAAAGGTTGGAAGAAGAAAAGATTTTTTAGTTAGACGCGTGGAATTAGCTAAGCATTTTATTCGAACAAATATACAACCAGAATGGATGGTTTTATGTCTATTACCTGTTCTTCCTCCCGAGTTGAGACCTATCATTCAGATAGATGGGGGTAAACTGATGAGTTCCGATATTAATGAACTCTATAGAAGAGTTATCTATCGGAACAATACTCTTATTGATCTATTAACAACAAGTAGATCTACGCCAGGGGAATTAGTAATGTGTCAAGAGAAGTTGGTACAAGAAGCCGTGGATACACTTCTTGATAATGGAATCCGCGGACAACCAATGAGGGACGGTCATAATAAAGTTTACAAGTCGTTTTCGGATGTAATTGAAGGAAAAGAAGGAAGATTTCGTGAGACTATGCTTGGCAAACGGGTTGATTATTCGGGTCGTTCTGTCATTGTCGTAGGCCCCTCACTTTCATTACATCGATGTGGATTGCCTCGCGAGATAGCAATAGAGCTTTTCCAGATATTTGTAATTCGTGGTCTAATTAGACAGCATCTTGCTTCGAACATAGGAGTTGCTAAGAGTAAAATTCGGGAAAAAGAACCAATTGTATGGGAAATACTTCAGGAAGTTATGCAGGGGCATCCGGTATTACTGAATAGAGCGCCGACTCTGCATAGATTAGGCATCCAGGCATTCCAACCCATTTTAGTGGAAGGCCGTGCTATTTGTTTACATCCATTAGTTTGTAAGGGATTCAACGCAGACTTTGATGGGGATCAAATGGCTGTTCATGTACCTTTATTGTTGGAAGCTCAAGCGGAGGCTCGTTTACTTATGTTTTCTCATATGAATCTCCTGTCTCCAGCTATTGGAGATCCCATTTCCGTACCAACTCAAGATATACTTATTGGGCTCTATGTATTAACAAGCGGAAATCGTCGAGGTATTTGTGAAAATAGGTATAATCCATTTAATCTCCAAAAGTCTCAAAATCAAAGAATTGCCAACAATAACGAAAAATATACAAAAGAACCCTTTTTTTCTAATTCTTATGGTGCAATTGGTGCTTATCGGCAGAAACGAATCAATTTAGATAGTGCTTTGTGGCTCCGATGGCAACTAGATCAACGCACTATTGCTTCAAGAGAAGCTCCCATCGAAGTTCACTATGAATCTTTGGGTACCTATCATGAGATTTATGAACACTATCTAATAGTAAGAAATATAAAAAAAGAAATTCTTTGTATATACATTCGAACTACTGTTGGTCATATTTCTCTTTATCGCGAAATCGAAGAAGCTATACAAGGGTTTTGCCAAGCCTCCTCAGATGGTATCGGTATCTAA

>lcl|NC_033910.1_cds_YP_009348405.1_74 [gene=ndhG] [locus_tag=B2L33_pgp013] [db_xref=GeneID:31082840] [protein=NdhG] [protein_id=YP_009348405.1] [location=complement(124044..124574)] [gbkey=CDS]
ATGGATTTACCTGGACTAATACATGATTTTCTTTTAGTCTTTCTGGGGTTAGGTCTTATATTAGGAGGTTTAGGAGTAGTATTATTTACCAACCCCATTTTTTCTGCCTTTTCATTAGGATTCGTTCTTGTTTGTATATCTTTATTTTATATTTTATCAAACTCTCATTTTGTAGCTGCTGCACAGCTCCTTATTTATGTGGGAGCTATAAATGTTTTAATTATATTTGCCGTAATGTTCATGAATGGTTCAGAATATTACAAAGATTTTAATCTTTGGACTGTTGGAAATGGAGTTACTTCCTTAGTTTGTACAAGTATTTTTGTTTCACTAATTACTATTATTCCAGATACGTCATGGTACGGAATTATTTGGACTACAACATCAAATCAGATTATAGAACAAGATTTGATAACTAATGGTCAACAAATTGGAATTCATTTAGCAACAGATTTTTTTCTTCCATTTGAATTCATTTCAATAATTCTTTTAGTTGCTTTGATAGGTGCAATTGCTGTGGCTCGTCAGTAA

>lcl|NC_033910.1_cds_YP_009348334.1_3 [gene=psbA] [locus_tag=B2L33_pgp085] [db_xref=GeneID:31082725] [protein=PsbA] [protein_id=YP_009348334.1] [location=complement(1130..2191)] [gbkey=CDS]
ATGACTGCAATTTTAGAGAGACGCGAAAGCGAAAGCCTATGGGGTCGTTTCTGTAACTGGATAACCAGCACTGAAAACCGTCTTTACATTGGATGGTTTGGTGTTTTGATGATCCCTACTTTATTGACCGCAACTTCTGTATTTATTATCGCTTTCGTTGCTGCCCCTCCGGTAGATATTGATGGTATTCGTGAACCTGTTTCTGGATCTCTACTTTATGGCAACAACATTATTTCTGGTGCCATTATTCCTACTTCTGCGGCTATAGGTTTGCATTTTTACCCAATATGGGAAGCGGCATCTGTTGATGAGTGGTTATACAATGGCGGTCCTTATGAGTTAATTGTCCTACACTTCTTACTTGGTGTAGCTTGTTACATGGGTCGTGAGTGGGAACTTAGTTTCCGTCTAGGTATGCGCCCGTGGATTGCTGTTGCATATTCAGCTCCTGTTGCAGCTGCTACTGCTGTTTTCTTGATCTATCCAATTGGTCAAGGAAGCTTTTCTGATGGTATGCCTCTAGGAATCTCTGGTACTTTCAACTTTATGATTGTATTCCAGGCTGAGCACAACATCCTTATGCACCCATTTCACATGTTAGGCGTAGCTGGTGTATTCGGCGGCTCCCTATTCAGTGCTATGCATGGTTCCTTGGTAACCTCTAGTTTGATCAGGGAAACCACAGAAAATGAATCTGCTAACGAAGGTTACAGATTCGGTCAAGAGGAAGAAACTTATAATATCGTAGCCGCTCATGGTTATTTTGGCCGATTGATCTTCCAATATGCTAGTTTCAACAATTCTCGTTCTTTACATTTCTTCTTAGCTGCTTGGCCTGTAGTAGGCATTTGGTTCACTGCTTTAGGTATTAGCACTATGGCTTTCAACTTAAATGGTTTCAATTTCAACCAATCTGTAGTTGATAGTCAAGGTCGTGTAATTAATACCTGGGCTGATATTATTAACCGTGCTAACCTTGGTATGGAAGTTATGCATGAACGTAATGCTCATAACTTCCCTCTAGACCTAGCTGCTATTGAAGCTCCATCTACAAATGGGTAA

>lcl|NC_033910.1_cds_YP_009348364.1_33 [gene=ycf4] [locus_tag=B2L33_pgp055] [db_xref=GeneID:31082801] [protein=Ycf4] [protein_id=YP_009348364.1] [location=64575..65129] [gbkey=CDS]
ATGAGTTGGCGATCAGAACATATATGGATAGAACTTATAGCGGGGTCTCGAAAAATAAGTAATTTCTGCTGGGCTTTTATCCTCTTTTTAGGTTCATTGGGTTTTTTATTGGTTGGAATTTCCAGTTATCTTGGAAAAAGTTTCATATCTTTATTTCCCTCTCAGCAAATACTTTTTTTTCCACAAGGGATCGTGATGTCTTTCTATGGGATCGCCGGTTTATTTATTAGTTGTTATTTGTGGTGCACAATTTTGTGGAATGTGGGTGGGGGTTATGATCGATTCGATAGAGAAGAAGGAATAGTATGTTTTTTTCGCTGGGGATTTCCTGGAAAAAATCGTCGCATCTTACTCCGATTCCTTATGAAAGATATTCAGTCTATTAGAATAGAAGTTAAAGAGGGTATTTACGCTCGGCGTATCCCTTATATGGAAATCCGAGGCCGAGGGACTGTTCCTTTGACTCGTACTGATGAGAATTTGACTCCACAAGAAATTGAGCAAAAAGTTGCGGAATTGGCCTATTTTTTGCGTGTACCAATTGAAGTATTTTAA

>lcl|NC_033910.1_cds_YP_009348357.1_26 [gene=ndhK] [locus_tag=B2L33_pgp062] [db_xref=GeneID:31082794] [protein=NdhK] [protein_id=YP_009348357.1] [location=complement(52994..53671)] [gbkey=CDS]
ATGAATTCCATTGAGTTTCCCTTACTTGATCGAACAACTCAAATTTCCGTTATTTCAACTACATCAAATGATCTTTCAAATTGGTCAAGACTTTCCAGTTTATGGCCACTTCTCTATGGTACCAGTTGTTGCTTCATTGAATTTGCTTCATTAATAGGATCCCGATTCGACTTTGATCGTTATGGACTAGTACCAAGATCGAGTCCTAGACAAGCGGACTTAATTTTAACAGCCGGCACGGTAACCATGAAAATGGCTCCTTCTTTAGTAAGATTATATGAACAAATGCCTGAACCAAAATATGTTATTGCGATGGGGGCATGTACAATTACAGGAGGAATGTTCAGTACCGATTCTTATAGTACTGTTCGAGGAGTTGATAAGTTAATTCCTGTAGATGTCTATTTGCCAGGCTGTCCACCGAAACCGGAAGCGGTTATAGATGCTATAACAAAACTTCGTAAAAAAATATCTCGAGAAATTTCTGAGGATCGAATTAGATCTCAACCGGGGAATCGATGTTTTACTACCAATCACAAGTTTCATATTGAACGCAATACTCATACTGGAAATTATGATCAAGGATTCCTCTATCAACCGCCGTCTACTTCAAAGATCCCTCCTCAAACATTTTTCAAATATAAAAAGTCAGTATCGTCCATCGAATTAGTAAACTAG

>lcl|NC_033910.1_cds_YP_009348399.1_68 [gene=ycf1] [locus_tag=B2L33_pgp019] [db_xref=GeneID:31082759] [protein=Ycf1] [protein_id=YP_009348399.1] [location=115753..117171] [gbkey=CDS]
ATGATTTTTAAATCTTTTATACTAGGTAATCTAGTATCCTTATGCATGAAGATACTCAATTCGGTCGTTGTGGTCGGACTCTATTATGGATTTCTGACCACATTTTCCATGGGGCCCTCTTATCTCTTCCTTCTCCGAGCTCGGGTTATAGAAGAAGGAGAAGAAGGAACTGAGAAGAAGGTATCAGCAACAACAGGTTTTATTACGGGACAGCTCATGATGTTCATATCGATCTATTATGCGCCTCTGCATCTAGCATTGGGTAGACCTCATACAATAACTGTCCTAGCTCTACCCTATCTTTTGTTTCATTTCTTCTGGAATAATCACAAACACTTTTTTGATTATGGATCTACTAACAGAAATTCAATGCGTAATCTTAGCATTCAATTTGTATTCCTGAATAATCTAATTTTTCAATTATTCAACCATTTCATTTTACCAAGTTCAATGTTAGTCAGATTAGTCAACATTTATATGTTTCGATGCAACAACAAGATGTTATTTGTAACAAGTAGTTTTGTTGGTTGGTTAATTGGTCACATTTTATTCATGAAATGGGTTGGATTGATATTAGTCTGGATACAGCAAAATAATTCTATTAGATCTAATGTACTTTTTCGATCTAATAAGTACCTTGTGTCAGAATTGAGAAATTCTATGGCTCGAATCTTTAGTATTCTCTTATTTATTACCTGTGTCTACTCTTTAGGCAGAACACCGTCACCCATTTTTACTAAGAAACTGAAAGAAACCTCAGAAACGGAAGAAAGCGAGGAAGAAACAGATGTAGAAACAACTTCCGAAACGAAGGGGACTAAACAGGAACAAGAGGGATCCACCGAAGAAGATCCTTCTTCTTCCCTTTTTTCGGAAGAAAAGGAGGATCCGGACAAAATCGACGAAACGGAAGAGGTCCAAGTGAATGGAAAGGAAAAAACAAAGGATGAATTCCATTTTCACTTTAAAGAGACATGCTATAAAAATAGACCACTTTATGAAACTTTTTATCTGGATGGGAATCAAGAAAATTCGAAGTTAGAAATATTGATAGAAAAAAAAAATAAAGATCTGTTATGGGTTGAAAAACCTCTTGTAACTATTCTTTTTGATTCTAAACGTTGGAATCGTCCATTTCGATATATAAAAAATCATCAGTTTGAGAATGCTTTAAGAAGAGAAATGGGACAATATTTTTTTTCTACATGTCTAAGTGATGGAAAAGAAAGAATATCTTTTATGTATCCACCCAGTTTGTCAACTTTTTTGGAACTGATACAAAGAAAGATGTCTCTGTTCATAACACACAAATTTTCCTCTGATGAATTGTATAATCATTGGAATTCCAAGAATGAAAAAAAAAAAAAAAATCGAAGTAATGAATTTTTAAATACTTTAAATACTATAAATACTAAATAA

>lcl|NC_033910.1_cds_YP_009348355.1_24 [gene=rps4] [locus_tag=B2L33_pgp064] [db_xref=GeneID:31082739] [protein=ribosomal protein S4] [protein_id=YP_009348355.1] [location=complement(49402..50007)] [gbkey=CDS]
ATGGCACGTTACCGAGGGCCTCGTTTCAAAAAAATACGCCGTCTGGGGGCTTTACCGGGATTAACTAGTAAAAAGCCTAGAGCCGGGAGCGATCTTAGAAATCAATCACGCTCGGGGAAAAAATCTCAATATCGTATTCGTTTAGAAGAAAAACAAAAATTGCGTTTTCATTACGGTCTTACAGAACGACAATTACTTAAATACGTTCGTATCGCCGCAAAAGCTAAAGGTTCAACAGGTCGGGTTTTACTACAATTACTTGAAATGCGGTTGGATAACATCCTTTTTCGATTGGGTATGGCGTCAACTATTCCTCGAGCCCGCCAATTAGTTAATCATAGACATATTTTAGTTAATGGTCGTATAGTAGATATACCAAGTTATCGCTGCAAACCCCGAGATATTATTACAGCGAGGGATGAACAAAAATCTAGAGCTATGATTCAAAATTATCTCGCTTCATCTCCCCAGGAGGAATTGCCAAAACATTTGACTCTTCACCCATTTCAATATAAAGGATTGGTCAATCAAATAATAGATAGTAAATGGGTCGGCTTGAAAATAAATGAATTGCTAGTAGTAGAATATTATTCTCGTCAGACTTAA

>lcl|NC_033910.1_cds_YP_009348391.1_60 [gene=rps3] [locus_tag=B2L33_pgp027] [db_xref=GeneID:31082826] [protein=ribosomal protein S3] [protein_id=YP_009348391.1] [location=complement(89286..89942)] [gbkey=CDS]
ATGGGACAAAAAATAAATCCACTTGGTTTCAGACTTGGTACAACCCAAAGTCATCATTCTCTTTGGTTTGCACAACCAAAAAATTACTCTGAGGGTCTACAAGAAGATCAAAAAATAAGAAACTGTATCAAGAATTTTGTAAAAAAAAATACAAAAATATCTTCTGGCGTTGAGGGAATTGCACGTATAGAGATTCAAAAACGAATCGATGTGATTCAAGTCATAATATATATGGGATTCCCAAAATTATTAATAGAAAATAGACCTAAACGAATCGAAGAATTACAGATACATGTACAAAAAGAACTAAATTGTGTAAACCGAAAACTCAATATTGTTATCACAAGAATTTCAAATCCTTATAGCAACCCTAATATTCTTGCAGAATTTATAGCTGGACAATTAAAAAATCGAGTTTCTTTTCGTAAAGCAATGAAAAAAGCTATTGAATTAACCGAACAGGCGGATACAAAAGGAATTCAAGTACAAATTGCGGGACGCCTTGATGGAAAAGAAATTGCACGAGTCGAATGGATTAGAGAGGGTAGGGTTCCTCTACAAACCATTCGAGCTAAAATTGATTATTGTTCGTATACAGTTAGAACTATTTATGGGGTATTAGGCATAAAAATTTGGACATTTCTAGACAAGAAATAA

>lcl|NC_033910.1_cds_YP_009348386.1_55 [gene=rps11] [locus_tag=B2L33_pgp032] [db_xref=GeneID:31082821] [protein=ribosomal protein S11] [protein_id=YP_009348386.1] [location=complement(85111..85527)] [gbkey=CDS]
ATGGCAAAACCTTTACCACGAATTGGTTCACGCAGAACTGGACGCATTGGTTCACGTAAGAATGCACGTAAAATACCAAAAGGAGTTATTCATGTTCAAGCAAGTTTCAACAATACTATTGTGACCGTTACAGATGTACGGGGACGAGTAATTTCGTGGTCCTCGGCTGGAACTTGTGGATTCAAAGGCACAAGAAGAGGAACACCATTTGCTGCTCAAACCACAGCAGGAAATGCTATTCGAACAGTAGTGGATCAAGGCATGCAACGAGCAGAAGTCATGATAAAAGGTCCTGGTCTCGGACGCGATGCGGCATTAAGAGCTATTCGCAGAAGTGGTATACTATTAACTTTTGTCCGGGATGTAACCCCTATGCCACATAATGGCTGCAGACCCCCGAAAAAAAGGCGCGTGTAA

>lcl|NC_033910.1_cds_YP_009348352.1_21 [gene=psaB] [locus_tag=B2L33_pgp067] [db_xref=GeneID:31082789] [protein=PsaB] [protein_id=YP_009348352.1] [location=complement(41118..43322)] [gbkey=CDS]
ATGGCATTAAGATTTCCAAGGTTTAGCCAAGGCTTAGCTCAGGACCCCACTACTCGTCGTATTTGGTTTGGTATTGCTACCGCGCATGACTTTGAAAGTCATGATGATATTACGGAGGAACGTCTTTATCAGAACATTTTTGCTTCTCACTTCGGGCAATTAGCAATAACTTTTTTGTGGACTTCTGGAAATCTCTTTCATGTGGCTTGGCAAGGAAATTTTGAAGCATGGGTACAGGACCCTTTACATGTAAGACCCATTGCTCATGCAATTTGGGATCCTCATTTTGGTCAACCAGCCGTAGAAGCTTTTACTCGAGGGGGTGCCCCTGGACCAGTGAATATCGCTTATTCGGGTGTTTATCAATGGTGGTATACAATCGGTTTACGTACTAATGAAGATCTTTATATTGGAGCTCTTTTTCTATTATTTCTTTCTGCCCTAGCCTTACTCGCGGGTTGGTTACACCTACAACCGAAATGGAAACCGAGCGTTTCATGGTTCAAAAATGCCGAATCTCGTCTCAATCATCATTTGTCAGGGCTATTTGGAGTAAGTTCTTTGGCTTGGACAGGACACTTAGTCCATGTTGCTATTCCTGGCGCCAGGGGGGAATACGTTCGATGGAATAATTTATTAGATGTATTACCACATCCCCAAGGATTAGGCCCACTTTTGACAGGTCAATGGAATCTTTATGCTCAAAATCCCGATTCAGGTAGTCATTTATTTGGTACCTCCCAAGGAGCAGGAACTGCCATTCTAACCCTTCTCGGGGGGTTCCATCCACAAACACAAAGTTTATGGCTGACCGATATTGCACACCATCATTTAGCTATTGCGTTTATTTTTCTCGTTGCCGGTCATATGTATAGAACTAACTTCGGGATTGGGCACAGTATAAAAGATCTTTTAGAAGCACATATTCCTCCGGGGGGGCGATTGGGACGTGGACATAAGGGTCTTTATGACACAATCAACAATTCGCTTCATTTTCAATTAGGTCTTGCTCTAGCGTCTTTAGGGGTTATTACGTCCTTAGTAGCTCAACACATGTACTCATTACCTGCTTATGCGTTCATAGCGCAAGACTTTACTACTCAAGCTGCGTTATATACTCATCACCAATACATCGCAGGATTCATCATGACAGGAGCTTTTGCTCACGGAGCTATATTTTTTATTAGAGATTACAATCCGGAACAGAATGAGAATAATGTATTGGCAAGAATGTTAGACCATAAAGAAGCTATCATATCCCATTTAAGTTGGGCCAGCCTCTTTCTGGGATTCCATACGTTAGGACTTTATGTTCATAATGATGTCATGCTTGCTTTTGGTACTCCGGAGAAACAAATCTTGATCGAACCCATATTTGCCCAATGGATACAATCTGCTCACGGCAAAACTTCATATGGGTTCGATGTACTTTTATCTTCAACGAATAGTCCAGCCTTTAATGCAGGTCGAAGCATATGGTTGCCCGGTTGGTTAAATGCTATTAATGCAAATAGTAATTCATTATTCTTAACAATAGGGCCTGGCGACTTCTTGGTTCATCATGCTATTGCTCTAGGTTTACATACAACCACATTGATACTAGTAAAAGGTGCTTTAGATGCACGTGGTTCGAAGTTAATGCCAGATAAAAAAGATTTTGGTTATAGTTTTCCTTGCGATGGTCCGGGGCGCGGTGGTACTTGTGATATTTCGGCTTGGGACGCATTTTATTTGGCGGTTTTTTGGATGTTAAATACTATTGGCTGGGTTACTTTTTATTGGCATTGGAAGCACATCACATTATGGCAAGGTAATGTTTCACAGTTTAATGAATCTTCCACTTATTTGATGGGATGGTTAAGAGATTATCTATGGTTAAACTCTTCACAACTTATCAATGGATATAACCCTTTTGGTATGAATAGCTTATCGGTCTGGGCATGGATGTTCTTATTTGGACATCTTGTTTGGGCTACTGGATTTATGTTTTTAATTTCTTGGCGTGGATATTGGCAGGAATTAATCGAAACTTTAGCATGGGCTCATGAACGTACACCTTTGGCTAATTTGATTCGATGGAGAGATAAACCAGTAGCTCTTTCCATCGTGCAAGCAAGATTGGTTGGATTAGCCCACTTTTCTGTAGGTTATATCTTCACTTATGCGGCTTTCTTGATTGCCTCTACATCAGGTAAATTTGGTTAA

>lcl|NC_033910.1_cds_YP_009348345.1_14 [gene=rpoB] [locus_tag=B2L33_pgp074] [db_xref=GeneID:31082782] [protein=RpoB] [protein_id=YP_009348345.1] [location=complement(25621..28833)] [gbkey=CDS]
ATGCTCGAGGATGGAAATGATAGAATGTCTACAATACCTGGATTTAATCAGATACAATTTGAAGGATTTTGGAGGTTCATTGATCAGGGCTTAACAGAAGAGTTTTCTAAGTTTCCAAAAATTGAAGATACAGATCAAGAAATTGAATTTCAATTATTTGTGGAAACATATCAATTAGTCGAACCATTGATAAAAGAAAGAGATGCTGTATATGAATCACTTACATATTCTTCTGAATTATATGTATCCGCGGGATTAATTTGGAAAAACAGTAGGGATATGCAAGAACAAACAATTTTTATTGGAAACATTCCTCTAATGAATTCCCTAGGAACTTTTCTAATAAACGGAATATACAGAATTGTAATCAATCAAATATTGCAAAGTCCAGGTATTTTTTACCGCTCCGAGTTGGATCATAACGGAATTTCGGTCTATATCGGGACTATAATATCAGATTGGGGAGGGAGAATAGAATTAGAGATTGATAGAAAAGCAAGGATATGGGCCCGCGTGAGTAGGAAACAGAAAATATCTATTCTAGTTCTATCATCAGCTATGGGTTTGAATCTACGACAAATTTTAGAGAATGTGTGTTACCCTGAGATTTTCTTAGCTTTCCTGAATGATAAAGAAAAAAAAAAAATTGGATCAAAAGAAAATGCCATTTTGGAGTTTTATCAACAATTTACTTGTGTAGGCGGAGATCCAGTATTTTCTGAATCCTTATGTAAGGAATTACAAAAGAAATTCTTTCAACAAAGATGTGAATTAGGAAGGATTGGTCGATTAAATATGAACCGGAGACTTAATCTTGATATACCTCATAACAATACATTTTTGTTACCAAGAGATATATTGGCAGCTGCGGATCGTTTGATTGAAATGAAATTTGGAATGGGTACACTTGACGATATGAATCATTTAAAAAATAAACGTATTCGTTCCGTAGCGAATCTCTTACAAGATCAATTCGGATTAGCCCTGATTCGTTTAGAAAATGTGGTTAGGGGGACTATATGTGGAGCAATTAGGCACAAATTGATACCGACCCCTCAAAATTTGGTAACTTCAACTCCATTAACAACCACTTATGAATCTTTTTTTGGATTACACCCATTATCTCAAGTTTTGGATCGAACTAATCCATTGACACAAATAGTTCATGGGAGAAAATCGAGTTATTTGGGTCCTGGAGGATTAACAGGACGAACTGCTAGTTTTCGAATACGAGATATCTACCCCAGTCACTATGGGCGCATTTGCCCCATTGACACGTCTGAAGGAATCAATGTTGGACTTATTGGATCTTTAGCAATTCATGCCAAGATTGATCATTGGGGGTCTTTAGAAAGCCCATTTTATGAAATTTCTGAGGGATCAAAAAAAGTACGGATGTTTTATTTATCACCAAATAGCGAGGAATACTATATGTTAGCTGCAGGAAATTCCTTGGCGCTGAATCGAGGTGTTCAGGAAGAACAGGTTGCGCCAGCTCGATATCGTCAAGAATTCCTGACTATTGCATGGGAACAGGTCCATCTTCGAAGTATTTTTCCCTTCCAATATTTTTCTATTGGAGCTTCCCTCATTCCTTTTATCGAGCATAATGATGCGAATCGGGCTTTAATGAGTTCGAATATGCAACGTCAAGCAGTTCCACTTTCTCGGTCCGAAAAATGCATTGTTGGAACTGGATTGGAACGCCAAGTGGCTCTAGATTCAGGGGTTCCTGCTATAGCCGAACACGAGGGAAAGATAATTTATACTGATATTGACAAGATCATTTTATCGGGCAATGGCAATACTCTACGCATTCCATTAGTTATGTATGAACGTTCTAACAAAAATACTTGTATGCATCAAAAAACCCAGGTTCAGCAGGGTAAATGTATTAAAAAAGGACAAGTTTTAGCGGATGGTGCCGCTACCGTTGGTGGCGAACTCGCCTTGGGCAAAAACGTATTAGTAGCTTATATGCCATGGGAAGGTTACAATTTTGAAGATGCAGTACTCATTAGCGAACGTCTGGTATATGAAGATATTTATACTTCTTTTCACATACGTAAATATGAAATTCAGACTCATGTGACAAGCCAAGGACCTGAAAGGATCACTAACGAAATCTCGCATCTAGAAGCCCATTTACTCCGAAATTTAGACAAAAATGGAATTGTGATGCTGGGATCTTGGGTAGAGGCAGGCGATATTTTAGTAGGTAAATTAACACCTCAAATAGCGAAAGAATCATCGTATGCTCCAGAAGATAGATTATTAAGAGCCATACTTGGTATTCAAGTATCTACTTCAAAGGAAACTTGTCTAAAATTACCTATAGGTGGTAGGGGTCGAGTTATTGATGTGAGATGGATCCAGAGAAAGGGGGGTTCCTGTTCTAATCCAGAAATGATTCGTGTATATATTTTACAGAAACGTGAAATCAAAGTGGGTGATAAAGTAGCTGGAAGACATGGAAATAAAGGCATCATTTCAAAAATTTTGCCTAGACAAGATATGCCTTATTTGCAAGATGGAAGACCTGTTGATATGGCCTTCAACCCATTAGGAGTACCTTCACGAATGAATGTAGGACAGATATTTGAATGCTCACTCGGGTTAGCGGGAGGTCTGCTAGATAGACATTATCGAATAGCACCCTTTGATGAGAGATATGAACAAGAGGCTTCGAGAAAACTAGTGTTTTCTGAATTATATGAAGCCAGTAAGCAAACAGCAAATCCGTGGGTATTTGAACCCGAGTATCCAGGAAAAAGTAGAATATTTGATGGAAGAACAGGAGATCCTTTTGAGCAGCCTGTTATAATAGGGAAGCCCTATATCTTGAAATTAATTCATCAAGTTGATGATAAAATACATGGCCGTTCCAGTGGACATTATGCACTTGTTACACAACAACCCCTTAGAGGAAGGGCCAAGCAAGGGGGACAACGGGTCGGAGAAATGGAGGTTTGGGCTCTAGAGGGATTTGGTGTTTCTCATATTTTACAAGAAATGCTTACTTATAAATCTGATCATATTAGAGCTCGCCAAGAAGTGCTTGGTACTATGATCATTGGAGGAACAATACCTAAACCTGAAGATGCTCCAGAATCTTTTCGATTGCTCGTTCGAGAACTACGATCTTTGGCTCTAGAATTGAATCATTTCCTTGTATCTGAGAAGAACTTCCAGATTAATAGGAAGGAAGCTTAA

>lcl|NC_033910.1_cds_YP_009348361.1_30 [gene=rbcL] [locus_tag=B2L33_pgp058] [db_xref=GeneID:31082798] [protein=ribulose-1,5-bisphosphate carboxylase/oxygenase large subunit] [protein_id=YP_009348361.1] [location=59162..60589] [gbkey=CDS]
ATGTCACCACAAACAGAGACTAAAGCAAGTGTTGGATTCAAGGCTGGTGTTAAAGATTATAAATTGACTTATTATACTCCTGAATATGAAACCAAAGATACTGATATCTTGGCAGCATTCCGAGTAACTCCTCAACCTGGAGTTCCGCCTGAGGAAGCAGGAGCTGCGGTAGCTGCTGAATCTTCTACTGGTACATGGACAACTGTGTGGACCGATGGGCTTACCAGTCTTGATCGTTATAAAGGACGATGCTACCACATCGAGCCCGTTGCTGGAGAAGAAAATCAATATATTGCTTATGTAGCTTACCCCTTAGACCTTTTTGAAGAAGGTTCTGTTACTAACATGTTTACCTCCATTGTAGGTAATGTATTTGGGTTCAAAGCCCTGCGCGCTCTACGTCTGGAGGATTTGCGAATCCCTCCTGCTTATACTAAAACTTTCCAAGGGCCGCCTCACGGCATCCAAGTTGAGAGAGATAAATTGAACAAGTATGGTCGCCCTCTATTGGGTTGTACTATTAAACCAAAATTGGGGCTATCCGCTAAGAATTACGGTAGAGCGGTTTATGAATGTCTTCGCGGTGGACTTGATTTTACCAAAGACGATGAGAACGTGAACTCTCAACCATTTATGCGTTGGCGAGACCGCTTTTTATTTTGTGCCGAATCCATTTTTAAATCACAGGCTGAAACAGGTGAAATCAAAGGGCATTATTTGAATGCTACTGCGGGTACATGCGAAGAAATGATCAAAAGGGCTGTATTTGCCAGGGAATTAGGAGTTCCTATCGTAATGCATGACTACTTAACAGGGGGATTCACGGCAAATACTAGCTTGGCTCATTATTGCCGAGATAATGGTTTACTTCTTCACATTCACCGCGCAATGCATGCAGTTATTGATAGACAGAAGAATCATGGTATACATTTTCGTGTATTAGCTAAGGCATTACGTATGTCTGGTGGAGATCATATTCACGCTGGTACCGTAGTAGGTAAACTTGAAGGAGAAAGAGAGATCACTTTGGGCTTTGTTGATTTACTGCGTGATGATTTTGTTGAAAAAGATCGAAGCCGCGGTATTTATTTCACTCAAGATTGGGTCTCTTTACCTGGTGTTCTTCCTGTAGCTTCAGGGGGTATTCACGTTTGGCATATGCCTGCTCTGACCGAGATCTTTGGAGATGATTCCGTACTACAATTCGGTGGAGGAACTTTAGGGCACCCTTGGGGAAATGCACCCGGTGCCGTAGCTAATCGAGTAGCTTTAGAAGCATGTGTACAAGCTCGTAATGAGGGACGTGATCTTGCTCGTGAGGGTAATGAAATTATTCGTGAGGCTGCAAAATGGAGTCCTGAACTAGCTGCTGCTTGTGAAGTATGGAAAGAGATTAAATTTGAATTCGAAGCAATGGATACTTTGTAA

2. *Hevea brasiliensis*

>lcl|NC_015308.1_cds_YP_004327713.1_73 [gene=ndhE] [locus_tag=HebrCp110] [db_xref=GeneID:10351954] [protein=NADH dehydrogenase subunit 4L] [protein_id=YP_004327713.1] [location=complement(123825..124130)] [gbkey=CDS]
ATGATGCTCGAACATGTACTTGTTTTGAGTGCCTATTTATTTTCTATTGGTATCTATGGATTGATCACGAGTCGAAATATGGTTAGAGCCCTTATGTGCCTTGAACTTATACTGAATGCAGTTAATCTAAATTTCGTAACATTTTCTGATTTTTTTGATAGCCGCCAATTAAAAGGAAATATTTTTTTCCATTTTGTTATAGCTATCGCAGCCGCTGAAGCAGCTATTGGACCAGCTATTGTTTCATCAATTTATCGTAATAGAAAATCAACCCGTATCAATCAATCGAATTTGTTGAATAAGTAG

>lcl|NC_015308.1_cds_YP_004327668.1_28 [gene=atpE] [locus_tag=HebrCp047] [db_xref=GeneID:10351982] [protein=ATP synthase CF1 epsilon subunit] [protein_id=YP_004327668.1] [location=complement(55642..56043)] [gbkey=CDS]
ATGACCTTAAATCTTTGTGTACTGACCCCGAATCGAATTGTTTGGGATTCAGAAGTGAAAGAAATCATTTTATCTACTAATAGTGGACAAATTGGCGTATTACCAAACCATGCACCTATTGCCACAGCTGTCGATATCGGTATTTTGAGAATACGCCTTAATGACCAATGGTTAACGATGGCTCTGATGGGTGGTTTTGCTAGAATAGGCAATAATGAGATTACTGTTTTAGTAAATGATGCAGAGAAGGGTAGTGACATTGATCCACAAGAAGCTCAGCAAACTCTTGAAATAGCAGAAGCTAACTTGAGGAAAGCGGAAGGCAGGAGACAAATAATTGAGGCAAATCTAGCTCTCAGACGAGCTAGGGCACGAGTAGAGGCTATCAATGGGATTTCGTAA

>lcl|NC_015308.1_cds_YP_004327722.1_82 [gene=ycf2] [locus_tag=HebrCp132] [db_xref=GeneID:10352018] [protein=hypothetical chloroplast RF2] [protein_id=YP_004327722.1] [location=complement(151983..158894)] [gbkey=CDS]
ATGAAAGGACATCAATTCAAATCCTGGATTTTCGAATTGAGAGAGATATTGAGAGAGATCAAGAATTCTCACTATTTCTTAGATTCATGGACCCAATTCAATTCAGTGGGATCTTTCATTCACATTTTTTTCCATCAAGAACGTTTTATAAAACTCTTGGACTCCCGAATTTGGAGTATCTTACTTTCACGCAATTCACAGGGTTCAACAAGCAATCGATATTTCACGATCAAGGGTGTAGTACTATTTGTAGTAGTGGTCCTTATATATCGTATTAACAATCGAAAGATGGTCGAAAGAAAAAATCTCTATTTGACAGGGCTTCTTCCTATACCTATGAATTCCATTGGACCCAGAAATGATACATTGGAAGAATCCTTTGGGTCTTCCAATATCAATAGGTTGATTGTTTCGCTCCTGTATCTTCCAAAAGGAAAAAAGATCTCTGAGAGCTCTTTCCTGGATCCGAAAGAGAGTACTTGGGTTCTCCCAATAACTAAAAAGTGTATCATGTCTGAATCTAACTGGGGTTCGCGGTGGTGGAGGAACTGGATCGGAAAAAAGAGGGATTCTAGTTGTAAGATATCTAATGAAACCGTTGCTGGAATTGAGATCTCATTCAAAGAAAAAGATATCAAATATCTGGAGTTTCTTTTTGTATATTATATGGATGATCCGATCCGCAAGGACCATGATTGGGAATTGTTTGATCGTCTTTCTCCGAGGAAGGGGCGAAACATAATCAACTTGAATTCGGGACAGCTATTCGAAATCTTAGTGAAAGACTGGATTTGTTATCTCATGTTTGCTTTTCGTGAAAAAATACCAATTGAAGTGGAGGGTTTCTTCAAACAACAAGGAGCTGGGTCAACTATTCAATCAAATGATATTGAGCATGTTTCCCATCTCTTCTCGAGAAAGAAGTGGGCTATTTCTTTGCAAAATTGTGCTCAATTTCATATGTGGCAATTCCGCCAAGATCTCTTCGTTAGTTGGGGGAATAATCCGCACGAATCGGATTTTTTGAGTAACATATCGAGGATTTGGTTAGACAATGTGTGGTTGGTAAACAAGGATCGGTTTTTTAGCAAGGCACGGAATATATCGTCAAATATTCAATATGATTCCACAAGATCTAGTTTCGTTCAAGGAAGGAATTCTAGCCAATTGAAGGGATCTTCTGATCAATCCAGAGATCATTTCGATTCCATTAGTAATGAGGATTCGGAATATCACACATTGATCAATCAAAGAAAGATTCAACAACTAAAAGAAAGATCGATTCTTTGGGATCCTTCCTTTCTTCAAACGGAACGAACAGAGATAGAATCAGACCGATTCCCTAAATGCCTTTCTGGATATTCCTCAATGTCCCGGCTATTCACGGAAGGTGAGAAGGAGATGAATAATCATCTGCTTCCGGAAGAAATCGAAGAATTTCTTGGGAATCCTACAAGATCCATTCGTTCTTTTTTCTCTGACAGATCGTCAGAACTTTATCTGGGTTCGAATCCTACTGAGAGGTCCACTAGAGATCAGAAATTGTTGAAGAAAGAACAAGATGTTTCTTTTGTCCCTTCCAGGCGATCGGAAAATAAAGAAATAGTTAATATATTCAAGATAATCACGTATTTACAAAATACCGTCTCAATTCATCCTATTTCATCAGATCCGGGATGTGATATGGTTCTGAAGGATGAACTGGATATGGACAGTTCCAATAAGATTTCTTTCTTGAACAAAAATCCATTTTTTGATTTATTTCATCTATTCCATGATCGGAACGGGGGGGGATACACGTTACACCACGATTTTGAATCAGAAGAGAGATTTCAAGAAATGGCAGATCTATTCACTCTATCAATAACCGAGCCGGATCTGGTGTATCATAAGGGATTTACCTTTTTTATTGATTCCTACGGATTGGATCAAAAACAATTCTTGAATGAGGTATTCAACTCCAGGGATGAATCGAAAAAGAAATCTTTATTGGTTCTACCTCCTATTTTTTATGAAGAGAATGAATCTTTTTATCGAAGGATCAGAAAAAAATGGGTCCGGATCTCCTGCGGGAATGATTTGGAAGATCCAAAACAAAAAATAGTGGTATTTGCTAGCAACAACATAATGGAGGCAGTCAATCAATATGGATTGATCCTAAATCTGATTCAAATCCAATATAGTACCTATGGGTACATAAGAAATGTATTGACTCAATTCTTTTTAATGAATAGATCCGATCGCAACTTCGAATATGGAATTCAAAGGGATCAAATAGGAAATGATACTCTGAATCATAGAACTATAATGAAATATACGATCAACCAACATTTATCGAATTTGAAACAGAGTCAGAAGAAATGGTTCGATCCTCTTATTTTTCTTTCTCGAACCGAGAGATCCATGAATTGGGATCCTAATGCATATAGATACAAATGGTCTAATGGGAGCAAGAATTTCCAGGAACATTTGGAACATTTCATTTCTGAGCAGAAGAGCCGTTTTCTTTTTCAAGTAGTGTTCGATCGATTACGTATTAATCAATATTCGATTGATTGGTCTGAGGTTATCGACAAAAAAGATTTGTCTAAGTCACTTCGTTTCTTTTTGTCCAAGTTACTTCTTTTTTTGTCCAAGTTTCTTCTCTTTTTGTCTAACTCACTTCCTTTTTTCTTTGTGAGTTTCGGGAATATCCCCATTCATAGGTCCGAAATCCATATCTATGAATTGAAAGGTCCGAATGATCAACTCTGCAATCAGCTGGTAGAACCAATAGGTCTTCAAATCGTTCATTTGAAAAAATTGAAACCCTTCTTATTGTTATTGGATGATCATGATACTTCCCAAAAATCTAAATTTTTGATTAATGGAGGAACAATATCACCATTTTTGTTCAATAAGATAACAAAGTGGATGATTGACTCATTCCATACTAGAAATAATCGCAGGAAATCTTTTGATAACACGGATTCCTATTTCTCAATGATATCCCACGATCAAGACAATTGGCTGAATCCCGTGAAACCATTTCATAGAAGTTCATTGATATCTTCTTTTTATAAAGCAAATCGACTTCGATTCTTGAATAATCTACATCACTTCTGCTTCTATTGTAACAAAAGATTCCCTTTTTATGTGGAAAAGGCCCGTATCAAGAATTATGATTTTACGTATGGACAATTCCTCAATATCTTGTTCATTCGCAACAAAATATTTTCTTTGTGCGGCGGTAAAAAAAAACATGCTTTTTTGGAGAGAGATACTATTTCACCAATCGAGTCACAGGTATCTAACATATTCATACCTAATGATTTTCCACAAAGTGGTAACGAAAGGTATAACTTGTACAAATCTTTCCATTTTCCAATTCGATCCGATCCATTCGTTCGTAGAGCTATTTATTCGATCGCAGACATTTCTGGAACACCTCTAACAGAGGGACAAATAGTCAATTTTGAAAGAACTTATTGTCAACCTCTTTCGGATATGAATCTATCTGATTCAGAAGGGAAGAACTTGCATCAGTATCTCAATTTCAATTCAAACATGGGTTTGATTCACACTCCATGTTCTGAGAAATATTTACCATCCGAAAAGAGGAAAAAACGGAGTCTTTGTCTAAAGAAATGTGTTGAAAAAGGGCAGATGTATAGAACCTTTCAACGAGATAATGCTTTTTCAACTCTCTCAAAATGGAATCTATTCCAAACATATATGCCATGGTTCCTTACTTCGACGGGGTACAAATATCTAAATTTGATATTTTTAGATACCTTTTCGGACCTATTACCGATACTAAGTAGCAGTCAAAAATTTGTATCCATTTTTCATGATATTATGCATGGATCAGATATATCATGGCGAATTCTTCAGAAAAAATTGTGTCTTCCACAATGGAATCTGATAAGTGAGATTTCGAGTAAGTGTTTACATAATCTTCTTCTGTCCGAAGAAATGATTCATCGAAATAATGAGCCACCATTGATATCGACACATCTGAGATCGCCAAATGTTCGGGAGTTCCTCTATTCAATCCTTTTCCTTCTTCTTGTTGCTGGATATCTCGTTCGTACACATCTTTTCTTTGTTTCCCGAGCCTATAGTGAGTTACAGACAGAGTTCGAAAAGGTCAAATCTTTGATGATTCCATCATACATGATTGAGTTGCGAAAACTTCTGGATAGGTATCCTACATCTGAACTGAATTCTTTCTGGTTAAAGAATCTCTTTCTAGTTGCTCTGGAACAATTAGGAGATTTTCTAGAAGAAATGCGGGGTTCTGCTTCTGGCGGCAACATGCTATGGGGTGGTGGTCCCGCTTATGGGGTTAAATCAATACGTTCTAAGAAGAAATTTTTTAATATCAATCTCATCGATCTCATAAGTATCATACCAAATCCCATCAATCGAATCACTTTTTCGAGAAATACGAGACATCTAAGTCATACAAGTAAAGAGATTTATTCATTGATAAGAAAAAGAAAAAACGTGAACGGTGATTGGATTGATGATAAAATAGAATCCTTGGTCGCGAACAGTGATTCGATTGATGATAAAGAAAGAGAATTCTTGGTTCAGTTCTCCACCTTAACGACAGAAAAAAGGATTGATCAAATTCTATTGAGTCTGACTCATAGTGATCATTTATCAAAGAATGACTCTGGTTATCAAATGATTGAAGAGCCGGGAGCAATTTATTTACGATACTTAGTTGACATTCATAAAAAGTATCTAATGAATTATGAGTTCAACACACCCTGTTTAGTAGAAAGACGGATATTCCTTGCTTATTATCAGACAACCACTTATTCACAAACCTCGTGTGGGGTGAATAGTTTTCATTTCCCATCTCATGGAAAACCCTTTTCGCTCCGCTTAGCCCTATCCCCCTCTAGGGGTATTTTAGTGATAGGTTCTATAGGAACTGGACGATCCTATTTGGTCAAATACCTAGCGACAAACTCCTATCTTCCTTTCATTACAGTATTTCTGAACAAGTTCCTGGATAACAAGCCTAAGGGTTTTCTTATTGATGATAGTGACGATATTGATGATAGTGACGATATTGATGATAGTGACGATATTGATGATAGTGACGATATTGATGTGAGTGACGATATTGATGTGAGTGACGATATTGACCGTGACTTTGATACGGAGCTGGAGTTTCTAACTAGGATGAATGTGCTAACTATGGATATGATGCCGGAAATAGACCGATTTTATATCACCCTTCAATTCGAATTAGCAAAAGCAATGTCTCCTTGCATAATATGGATTCCAAACATTCATGATCTGGATGTGAATGAGTCGAATTACTTATCCCTCGGTCTATTAGTGAACTATCTCTCCAGGGATTGTGAAAGATGTTCCACTAGAAATATTCTTGTTATTGCTTCGACTCATATTCCCCAAAAAGTGGATCCCGCTCTAATAGCTCCGAATAAATTAAATACATGCATTAAGATACGAAGGCTTCTTATTCCACAACAACGAAAGCACTTTTTTACTCTTTCATATACTAGGGGATTTCACTTGGAAAATAAAATGTTCCATACTAATGGATTCGGGTCCATAACCATGGGTTCCAATGTACGAGATCTTGTAGCACTTACCAATGAGGCCCTATCGATTAGTATTACACAGAAGAAATCAATTATAGACACTAATATAATTAGATCTGCTCTTCATAGACAAACTTGGGATTTGCGATCCCAGGTAAGATCGGTTCAGGATCATGGGATCCTTTTCTATCAGATAGGAAGGGCTGTTGCACAAAATGTATTTCTAAGTAATTGCCCCATAGATCCTATATCTATCTATATGAAGAAGAAATCATGTAACGAAGGGGATTCTTATTTGTACAAATGGTACTTCGAACTTGGAACGAGCATGAAGAAATTAACGATACTTCTTTATCTTTTGAGTTGTTCTGCCGGATCGGTTGCTCAAGACCTTTGGTCTCTACCCGGACCCGATGAAAAAAATGGGATCACTTATTATGGACTTGTTGAGAATGATTCTGATCTAGTTCATGGCCTATTAGAAGTAGAAGGCGCTCTGGTGGGATCCTCACGGACAGAAAAAGATTGCAGTCAGTTTGATAATGATCGAGTGACATTGCTTCTTCGGCCCGAACCAAGGAGTCCCTTAGATATGATGCAAAATGGATCTTGTTCTATCCTTGATCAGAGATTTCTCTATGAAAAATACGAATCGGAGTTTGAAGAAGGGGAAGGAGAAGAAGTCCTCGACCCGCAACAGATAGAGGAGGATTTATTCAATCACATAGTTTGGGCTCCTAGAATATGGCGCCCTTGGGGTTTTCTATTTGATTGTATCGAAAGGCCCAATGAATTGGGATTTCCCTATTGGGCCAGGTCATTTCGGGGCAAGCGGATCATTTATGATGAAGAGGATGAGCTTCAAGAGAATGATTCGGAGTTCTTGCAGAGTGGAACCATGCAGTACCAGATACGAGATAGATCTTCCAAAGAACAAGGCTTTTTTCGAATAAGCCAATTCATTTGGGACCCTGCGGATCCACTCTTTTTCCTATTCAAAGATCAGCCCTTTGTCTCTGTGTTTTCACATCGAGAATTCTTTGCAGATGAAGAGATGTCAAAGGGGCTTCTTACTTCCCAAACAGATCCTCCTACATCTATATATAAACGCTGGTTTATCAAGAATATGCAAGAAAAGCACTTCGAATTGTTGATTCATCGCCAGAGATGGCTTAGAACCAATAGTTCATTATCTAATGGATTTTTCCGTTCTAATACTCTATCCGAGAGTTATCAGTATTTATCAAATCTGTTCCTATCTAACGGAACGCTATTGGATCAAATGACAAAGGCATTGTTGAGAAAAAGATGGCTTTTCCCGGATGAAATGAAAATTGGATTCATGTAA

>lcl|NC_015308.1_cds_YP_004327653.1_13 [gene=atpI] [locus_tag=HebrCp020] [db_xref=GeneID:10351895] [protein=ATP synthase CF0 A subunit] [protein_id=YP_004327653.1] [location=26753..27502] [gbkey=CDS]
ATGAATGTTCTATCATGTTCCATCAACACACTAACACTAAAGGGGTTATATGATATATCCGGTGTGGAAGTAGGCCAGCATTTCTATTGGAAAATAGGAGGTTTCCAAGTCCATGCCCAAGTGCTTATTACTTCTTGGGTTGTAATTGCTATCTTATTAGGTTCAGCCATTGTAGCTGTTCGGAATCCACAAACCATTCCAACAGGAGGTCAGAATTTCTTCGAATACGTCCTTGAATTCATTCGAGATGTGAGCAAAACTCAGATTGGAGAGGAATATGGCCCGTGGGTCCCCTTTATTGGAACTATGTTTCTATTTATTTTTGTTTCTAATTGGGCGGGGGCGCTTTTACCTTGGAAGATCATACAGTTACCTCACGGGGAGTTAGCCGCACCGACGAATGATATAAATACTACCGTTGCTTTAGCTTTACTTACGTCAATAGCCTATTTTTATGCGGGCCTTAGCAAAAAAGGATTAGGTTATTTCAGTAAATACATTCAACCAACTCCAATCCTTTTACCCATTAACATTTTAGAAGATTTCACAAAACCTTTATCACTTAGCTTTCGACTTTTCGGCAATATATTAGCGGATGAATTAGTAGTTGTTGTTCTTGTTTCTTTAGTACCTTCAGTGGTTCCTATACCTGTCATGTTCCTTGGATTATTTACAAGTGGTATTCAAGCTCTTATTTTTGCAACTTTAGCTGCGGCTTATATAGGCGAATCCATGGAGGGGCATCATTGA

>lcl|NC_015308.1_cds_YP_004327719.1_79 [gene=ycf1] [locus_tag=HebrCp116] [db_xref=GeneID:10351960] [protein=hypothetical chloroplast RF1] [protein_id=YP_004327719.1] [location=complement(130080..135779)] [gbkey=CDS]
ATGATTTTTCAATCTTTTATACTAGGTAATCTAGTATCCTTATGCATGAAGATAATCAATTCGGTCGTTGTGGTCGGACTCTATTATGGATTTCTGACCACATTCTCCATGGGACCCTCTTATCTCTTCCTTCTCCGAGCTCGGGTTATAGAAGAAGGAGAAGAAGGAACTGAGAAGAAGGTATCAGCAACAACAGGTTTTATTACGGGACAGCTCATGATGTTCATATCGATCTATTATGCGCCTCTGCATCTAGCATTGGGTAGACCTCATACAATAACTGTCCTAGCTCTACCCTATCTTTTGTTTCATTTCTTCTGGAACAATCACAAACACTTTTTTGATTATGGATCTACTACCAGAAATTCAATGCGTAATCTTAGCATTCAATTTGTATTCCTGAATAATCTCATTTTTCAATTATTCAACCATTTCATTTTACCAAGTTCAATGTTAGTCAGATTAGTCAACATTTATATGTTTCGATGCAACAACAAGATGTTATTTGTAACAAGTAGTTTTGTTGGTTGGTTAATTGGTCACATTTTATTCATGAAATGGGTTGGATTGATATTAGTCTGGATACAGCAAAATAATTCTATTAGATCTAATGTACTTTTTCGATCTAATAAGTACCTTGTGTCAGAATTGAGAAATTCTATGGCTCGAATCTTTAGTATTCTCTTATTTATTACCTGTGTCTACTCTTTAGGCAGAATACCGTCACCCATTTTTACTAAGAAACTGAAAGAAACCTCAGAAACGGAAGAAAGGGAGGAAGAAACAGATGTAGAAATAGAAAAAACTTCCGAAACGAAGGGGACTAAACAGGAACAAGAGGGATCCACCGAAGAAGATCCTTCTTCTTCCCTTTTTTCGGAAGAAAAGGAGGATCCGGACAAAATCGACGAAACGGAAGAGATCCAAGTGAATGGAAAGGAAAAAACAAAGGATGAATTCCATTTTCACTTTAAAGAGACATGCTATAAAAATAGACCACTTTATGAAACTTTTTATCTGGATGGGAATCAAGAAAATTCGAAGTTAGAAATATTGATAGATAAAAAAAATAAAGATCTTTTCTGGTTTGAAAAACCTCTTGTAACTATTCTTTTTGACTCTAAACGTTGGAATCGTCCATTTCGATATATAAAAAATGATCAGTTTGAGAATGCTGTAAGAAAAGAAATGTCACAATATTTTTTTTATACATGTCGGAGTGATGGAAAAGAAAGAATATCTTTTACGTATCCACCCAGTTTGTCAACTTTTTTGGAAATGATACAAAGAAAGATATCTCTGTTTACAACAGAAAAACTCTCCTCTGATGAATTGTATAATCGTTGGAATTATAAGAATGAACAAAAAAAGAAAATCCTAAATAATGAATTTATAAATAGAGTCCAGGCTCTAGATAAGGAATATCTTACTCTGAATACACTCGAAAAAAGGACTAGACTATGTAATGATAAAACTAAAAAAAAGTACTTACCTAAAATTTATGATCCCTTTTTGAGTGGGTCCTGCCGCGGGAAAATCCAATTTTTTTTTTCACCCTCACTCCTAAATAAAACTTCCATAAAAAATTCAATAGAGATGCTTTGGATAAATAAAATTCATCTTATTCTTCTTATTACTAATTATCAAGAATTTGAATCAAAAACAGATGGATTTAATAGAAAAGCATTTTCAATAGAAAATGCTTATTTCTTAAACTTAATTAATGAATTTGTTGGAAAATCAAGATCAAGTTTAAATTTTAAGGAACTTCCCTTATTTCCAGATCACAAAGAAGAAAAAATGTATTTAGAAAATCGAATAAAAATTTTAAAATTTTTATTTCATACAGTTATAGCGAATCCAAAAAATAAAACAATTATAAAAAATTCTACTGGAATAAAAGAAATAAGTAAACAAGTTCCTCGATGGTCATACAAATTAATTGACGATTTGGAACAACAAGAGGGAGAAAATGAAGAAAACATGTCGGAAGATTATGAAATTCGTTCACGAAAAGCCAAACGGGTAGTGATTTTTACTGATAATAAAAAAAATACAGATACTTATAATAATACCAAAGATACAACGAATTCTGATCAAATAGAAGAAGTGACTTTGATACATTATTCACAACAATCGGATTTTCGCCGAGACATAATAAAAGGATCCGTGCGCGCACAAAGACGCAAAATAGCTATTTGGGAACTGTTTCAAGCAAATGTGCATTCTCCTCTTTTTTTGGACAGAATAGACAAATCTCTTTTTTTTTCTTTTGATATTTCCGAACTGATAAAAACAATGTTTATAAATTGGATGTATAAAAACGCAGAATTCACAATTTCAAATACAAAGAAAAAAACAAAAGAAAGTAAGAAAAAAGAAGAGGACAAAAGAGAAGACAACAAAAGAGAGGAAAAAGCTCGGATAGAAATAGCCGAAGCCTGGGATAGCATTCTTTTTGCTCAAATAATAAGAAGTTGTGTTTTAGTAACCCAATCAATTCTTAGAAAATATATTATATTACCTTCATTAATAATAACTAAAAATATCATTCGTATACTTTTTTTTCAAACTCCCGAATGGTCCGAGGATTTAAAAGATTGGAGTAGAGAAATGCATGTTAAATGCACCTATAATGGAGTTCAATTATCAGAAAAAGAATTTCCGAAAAACTGGTTAATAGACGGGATTCAAATAAAGATCCTATTTCCTTTTCGTCTAAAACCTTGGTACAAATCTAAGTTAAAATTCTCTCATAAAGATCAAACGAAAATGAAAAAAAAAGTACAAAAAAATGATTTTTTTTTTTTAACAGTTTTGGGAATGGAAGCTGAACTTCCTTTTGGTTCTCCCCGAAAACGGCTTTCACTTTTTGAACCCATCTTTAAAAAACTCGAAAAAAAAATTAGAAAAATGCAAAAAAATGGTTTTCGAGTTATAACAATTTTAGAAGAAAGAAGAAAATTTTTTCTAAATTTCTTAAAAGAAAAAAAAAACTGGATCATCAAAAACATTTTTTTTCGAAAAGAAATAATAAACAACCTTTCAAAATCAAAAAGAAATCCAATTCTATTATCTGGATTTAGAGAAGTATATGAATTGAATGAACCTAAAAAAGAAAAAGATTCGATAATCAATAACAATAATGAGACGATTAAAAAATTATCCATCCCAATTCGATCTATGACTTGGACAAATTATTCACTGACAGAAAAAAAAATGAAAGATCTTTCTGCTAGAAGAAAGATAATCATAAATCAAATAGAAAAAATTACAAAAGAAAAGGAAAAAAAAATGAGAACCCCGGAAATAAATATTAGTCCTAACAAAAACAAAATAAGTTATAATGCTAAAAAATTAAAATCATCAAAAAATATTTCGCAGATAGTAAAAAGAAGAAATGTTCGATTAGCGCGTAAATTCCATTTTTTTATAAAAATTTTGATTGAAAAGATATACATAGATATCTTTTTAGGTATCATTAATATTCCAAGGATCAATGCACAGCTTTTTCTTAAATCAACAAAAAAAATTATTACTAAATACATTTACAATAATGAAGAAAATCAGAAAAAAATTGATAAAACAAATCAAAATACAATTCACTTTATTTCGATTATAAAAAAGTCACGTAATAGTAATAATAGTAATGTTGTTGTTATTAATAAAAATTCAAAGATTTTTTGTGACATATCCTCCTTGTCACAAGCTTATGTATTTTACAAATTATCACAAATCAAAATTATTAACTTATATAAGTTAAAATCTATCTTTGAATATCATAGCCTTTTTCTGAAGAATGAAATAAAGGATTTTTTTATAGCCCAAGGGCTATTTAATTCCGAATTAAAAAATAAAAATTTTAGAAATTCTGTAATGAATCAATGGAAAAATTGGTTAAGGAGTCATTATCAATATAAATACGATCTATCTCAGATTAGATGGTCTAGATTAACACCACAAAAATGGCGAAATATAATCAATCAACATCAACACCATATGGTTCAAAATAAAAAATTAAACAAATGGAATTTATATGAAAAAGACCGATTAATTCATTACAAAAAAAAAAATGATTTTGAGACAGATTCATTACCAAATCAAAAAGATAATTTTAAAAAACACTATAAATATAATCTTTTATCATATAAATCTATTAATTATGAAAATAAGAAGGACTCATATATTTATGGATCACCATTACAAGTAAATAATAAACAAGAGATTTCTTATAATTACAACACAAAAAAAAGCAAATTATTTGACATGTTGGAAGATATTTCTATCAATAATTATCTAGCGGAAGATGATATTATTGATATGGAGAAAAGCGCGGATAGAAAATATTTTGATTGGAGAATTCTCAATTTTTGTCTTAGAAAGAAGGTTGATATTGAGTCCTGGATCGATACCGGAAGCAAAGATAAAAAAAATACTAAGACTAGGACTAATAAGTATCAAATAATTGATAAAATTGATAAGAAAAATCTTTTTTTTCTTACAATTCACCAAGATCAAGAAGTTAATTCATCCAATCAAAAAGGTTTTTTTTTTGATTGGATGGGAATGAATGAAGAAATAAAAAATTGTCTTATATCCAATTTTGAACTTTGGTTCTTTCGAAAATTTGTGATACTTTACAACACATATAAGATAAAACCATGGGCGATACCCATCCAATTTCTTCTTTTCAATTTTCATAGAAATGAAAATGTTAGTAAAAATAATAAAATTAACCGGAAGAAAAATAGCGATCTTTTTATATCTATATCATCGAATGAAAAAAAAATTATTGAATTAGAGAATCAAAATCACGAAGAAAAAGAATCCGAAGACCAAGGGGACTTTGGGTCAGTTTTCGCAAATCAAGAAAAAGATATTGAAGAAGATTATATAGAATTAGATATGAAAAAACATAGAAATAAAAAGCAAAACAAAAGTCATATGGAAGTAGAACTTGATTTCTTCCTAAAACGGTATTTATGTTTTCAATTAAGATGGAATGGTTCTTTAAATCACAAAATAATCAATAATATCAAAGTATATTGTCTCCTGCTTAGACTGACAAATCCACGAGAAATTATTATATCTTCTATTCAAAGGCAAGAAATAAGTTTGAATATTCTGATGGTTCAGAAGGATTTAACTCTTACGGAATTAATGAAAAAGGGAATATTGATTATCGAACCTGTTCGTCTGTCGGTAAAAAATGATGGACAATTTATTTTGTATCAAATGGTAGGTATCCTATTAGTTCATAAGAACAAACAACAAATTAATCAAAAATACAGAGAAAATTTTTATGTTGATAAAAAGAATTTTACCGAATCTATTGAAAGACATCAAAGTATAATTGGAAATAGAAACAAAAATGATTATGATTTACTTGTTCCTGAAAATATTTTATCCCCTAAACGTCGTAGAGAATTAAGAATTCTTTTCAATTTAAAAAATCAAAATGATATTCATATAAATACAGAAATTTTCAATGGTAATAACATAAAAAATTGTAGTCCCATTTTAGATAAAAGCAAACATTTTGATAGAGATAAAAAGAAACTAATTAAATTACAATTTTTTCTTTGGCCCAATTTTCGATTAGAAGATTTAGCTTGTATGAATCGTTATTGGTTCGATACTAATAATGCTAGTCGGTTCAGTATGGTAAGAATATATATATATCCGCGGTTGAAATTTTGA

>lcl|NC_015308.1_cds_YP_004327710.1_70 [gene=ccsA] [locus_tag=HebrCp107] [db_xref=GeneID:10352005] [protein=cytochrome c biogenesis protein] [protein_id=YP_004327710.1] [location=120443..121411] [gbkey=CDS]
ATGATATTCTCGACTTTAGAACATATATTAACGCATATATCTTTTTCAGTCGTGTCAATTGTAATTACAATTCATTTGATAACCTTATTAGTCGATGAATTCGTAGAACTATATGATTCGTCAGAAAAGGGCATGATAACTACTTTTTTCTGTATAACAGGATTATTAGTTATTCGTTGGATTTTTTTGGGACATTTACCATTAAGTGATTTATATGAATCATTAATCTTTCTTTCATGGGCTTTTTCCATTATTCATATGGTTCCGTATTTTAAAAAACATAAAAATTTTTTAAGCGCAATAACCGCGCCAAGTACTTTTTTTACCCAAGGGTTTGCTACTTCGGGTCTTTTAACTGACATGCATCAATCCGAAATCTTAGTGCCCGCTCTCCAATCTCAGTGGTTAATGATGCACGTAAGTATGATGATATTGGGCTATGCAGCTCTTTTGTGTGGATCATTATTTTCAGTAGCATTTCTAGTAATCACATTTCGAAAAATCATAAGAATTGTTGATAAAAGCAATAATTTATTAAACGATTCGTTTTTCTTTAGTGAGATACAATATATGGCGGAAAGAAAGAATGTTTTAAGAAATATTTCTTTTCTTTCTTCTAGGAATTATTACAGGTTTCAATTGATTCAACAATTAGATGACTGGGGTTGTCGTATTATAAGTATAGGGTTTATCTTTTTAACCATAGGTATTCTTTCGGGAGCAGTCTGGGCTAATGAAGCATGGGGATCATATTGGAATTGGGACCCAAAAGAAACTTGGGCATTTATTACGTGGACCATATTCGCGATTTTTTTTCATATTCGAACAAATAAAAAATTGGAGGGTTTCAATTCCGCAATTGTCGCTTCTATCGGTTTTCTTCTAATTTGGATATGCTATTTTGGAGTTAATTTATTAGGAATAGGACTACATAGTTATGGTTCATTTACATTAACAATTAGCATCTAA

>lcl|NC_015308.1_cds_YP_004327706.1_66 [gene=ndhB] [locus_tag=HebrCp090] [db_xref=GeneID:10351990] [protein=NADH dehydrogenase subunit 2] [protein_id=YP_004327706.1] [location=complement(join(100137..100892,101575..102351))] [gbkey=CDS]
ATGATCTGGCATGTACAGAATGAAAACTTCATTCTCGATTCTACGAGAATTTTTATGAAAGCCTTTCATTTGCTTCTCTTCGATGGAAGTTTTATTTTCCCAGAATGTATCCTAATTTTTGGCCTAATTCTTCTTCTGATGATCGATTCAACCTCTGATCAAAAAGATATACCTTGGTTATATTTCATCTCTTCAACAAGTTTAGTAATGAGTATAACGGCCCTATTGTTCCGATGGAGAGAAGAACCTATGATTAGCTTTTCGGGAAATTTCCAAACGAACAATTTCAACGAAATCTTTCAATTTCTTATTTTACTATGTTCAACTCTATGTATTCCTCTATCCGTAGAGTACATTGAATGTACAGAAATGGCTATAACAGAGTTTCTCTTATTCGTATTAACAGCTACTCTAGGAGGAATGTTTTTATGCGGTGCTAACGATTTAATAACTATCTTTGTAGCTCCAGAATGTTTCAGTTTATGCTCCTACCTATTATCTGGATATACCAAGAAAGATGTACGGTCTAATGAGGCTACTACGAAATATTTACTCATGGGTGGGGCAAGCTCTTCTATTCTGGTTCATGCTTTCTCTTGGCTATATGGTTCGTCCGGGGGAGAGATCGAGCTTCAAGAAATAGTGAATGGCCTTATCAATACACAAATGTATAACTCCCCAGGAATTTCAATTGCGCTTATATTCATCACTGTAGGAATTGGGTTCAAGCTTTCCCTAGCCCCTTCTCATCAATGGACTCCTGACGTATACGAAGGATCTCCCACTCCAGTCGTTGCTTTTCTTTCTGTTACTTCGAAAGTAGCTGCTTCAGCTTCAGCCACTCGAATTTTCGATATTCCTTTTTATTTCTCATCAAACGAATGGCATCTTCTTCTGGAAATCCTAGCTATTCTGAGCATGATAGTGGGGAATCTCATTGCTATTACTCAAACAAGCATGAAACGTATGCTTGCATATTCGTCCATAGGTCAAATCGGATATGTAATTATTGGAATAATTGTTGGAGACTCAAATGGTGGATATGCAAGCATGATAACTTATATGCTCTTCTATATCTCCATGAATCTAGGAACTTTTGCTTGTATTGTATTATTTGGTCTACGTACCGGAACTGATAACATTCGAGATTATGCAGGATTATACACGAAAGATCCTTTTTTGGCTCTCTCTTTAGCCCTATGTCTCTTATCCCTAGGAGGTCTTCCTCCACTAGCAGGTTTTTTCGGAAAACTCCATTTATTCTGGTGTGGATGGCAGGCAGGCCTATATTTCTTGGTTTTAATAGGACTCCTTACGAGCGTTGTTTCTATCTACTATTATCTAAAAATAATCAAGTTATTAATGACTGGACGAAACCAAGAAATAACCCCTCACGTGCGAAATTATAGAAGATCCCCTTTAAGATCAAACAATTCCATCGAATTGAGTATGATTGTATGTGTGATAGCATCTACTATACCAGGAATATCAATGAACCCGATTGTTGAAATTGCTCAAGATACCCTTTTTTAG

>lcl|NC_015308.1_cds_YP_004327692.1_52 [gene=petB] [locus_tag=HebrCp072] [db_xref=GeneID:10351933] [protein=cytochrome b6] [protein_id=YP_004327692.1] [location=join(79467..79472,80252..80893)] [gbkey=CDS]
ATGAGTAAAGTCTATGATTGGTTCGAAGAACGTCTCGAGATTCAGGCAATTGCAGATGATATAACTAGTAAATATGTTCCTCCCCATGTCAACATATTTTATTGTTTAGGAGGAATTACGCTTACTTGTTTTTTAGTACAAGTAGCTACGGGGTTTGCTATGACTTTTTACTACCGTCCGACCGTTACTGAGGCTTTTGCTTCTGTTCAATACATAATGACTGAGGCTAACTTTGGTTGGTTAATCCGCTCAGTGCATCGATGGTCGGCAAGTATGATGGTTTTAATGATGATCCTGCACGTATTTCGTGTATATCTCACTGGTGGCTTTAAAAAACCTCGTGAATTGACTTGGGTTACGGGCGTGGTTCTTGCTGTATTGACCGCATCTTTTGGTGTAACTGGTTATTCCTTACCTTGGGACCAAATTGGTTATTGGGCGGTCAAAATTGTAACAGGCGTGCCGGAAGCTATTCCTGTAATAGGATCGCCTTTGGTGGAGTTATTACGCGGAAGTGCTAGTGTAGGACAATCCACTTTGACTCGTTTTTATAGTTTACACACTTTTGTATTACCTCTTCTTACTGCCGTATTTATGTTAATGCACTTTTCAATGATACGTAAGCAGGGTATTTCAGGTCCTTTATAG

>lcl|NC_015308.1_cds_YP_004327670.1_30 [gene=rbcL] [locus_tag=HebrCp049] [db_xref=GeneID:10351912] [protein=ribulose-1,5-bisphosphate carboxylase/oxygenase large subunit] [protein_id=YP_004327670.1] [location=58292..59719] [gbkey=CDS]
ATGTCACCACAAACAGAGACTAAAGCAAGTGTTGGATTCAAGGCTGGTGTTAAAGATTATAAATTGACTTATTATACTCCTGAGTATCAAACCAAAGATACTGATATCTTGGCAGCATTCCGAGTAACTCCTCAACCTGGAGTTCCGCCTGAGGAAGCAGGAGCTGCGGTAGCTGCTGAATCTTCTACTGGTACATGGACAACTGTGTGGACCGATGGACTTACCAGTCTTGATCGTTATAAAGGACGATGCTACGGCATCGAGCCTGTTCCTGGGGAAGAAAATCAATATATTGCTTATGTAGCTTACCCATTAGACCTTTTTGAAGAAGGTTCTGTTACTAACATGTTTACTTCCATTGTGGGTAATGTATTTGGGTTCAAAGCCCTACGCGCCCTACGTCTGGAGGATTTGCGAATCCCTCCTGCTTATTCTAAAACTTTCCAAGGGCCGCCTCATGGCATCCAAGTTGAGAGAGATAAATTGAACAAGTATGGTCGCCCCCTATTGGGTTGTACTATTAAACCAAAATTGGGTCTATCCGCTAAGAATTACGGTAGAGCAGTTTATGAATGTCTTCGCGGTGGACTTGATTTTACCAAAGACGATGAGAATGTGAACTCCCAACCATTTATGCGTTGGAGAGACCGTTTCTTATTTTGTGCCGAAGCAATTTATAAAGCACAGGCTGAAACAGGTGAAATCAAAGGACATTATTTGAATGCTACTGCAGGTACATGCGAAGAAATGATCAAAAGGGCTGTATTTGCCAGAGAATTAGGAGTTCCTATCGTAATGCATGACTACTTAACAGGGGGATTCACTGCAAATACTAGCTTGGCTCATTATTGCCGAGATAATGGTTTACTTCTTCACATTCACCGCGCAATGCATGCAGTTATTGATAGACAGAAGAATCATGGTATGCATTTTCGTGTACTAGCTAAGGCCTTACGTCTATCTGGTGGAGATCATATTCACGCCGGTACCGTAGTAGGTAAACTTGAAGGGGAAAGAGACATTACTTTGGGCTTTGTTGATTTACTGCGTGATGATTTTATTGAAAAAGATCGAAGCCGTGGTATTTATTTCACTCAAGATTGGGTCTCTCTACCAGGTGTTATACCTGTAGCTTCAGGGGGTATTCACGTTTGGCATATGCCTGCTCTGACCGAGATCTTTGGAGATGATTCCGTACTACAATTCGGTGGAGGAACTTTAGGGCACCCTTGGGGAAATGCACCCGGTGCCGTAGCTAATCGAGTAGCTCTAGAAGCATGTGTACAAGCTCGTAATGAGGGACGTGATCTTGCTCGTGAGGGTAATGATATTATCCGTGAGGCTAGCAAATGGAGTCCTGAACTAGCTGCTGCTTGTGAAGTATGGAAGGAAATTAAATTTGAATTTGAAGCAGTGGATACTTTGTAA

>lcl|NC_015308.1_cds_YP_004327649.1_9 [gene=rpoB] [locus_tag=HebrCp016] [db_xref=GeneID:10351970] [protein=RNA polymerase beta subunit] [protein_id=YP_004327649.1] [location=15119..18331] [gbkey=CDS]
ATGCTCGGGGATGGAAATGGGGGAATGTCTACAATACCTGGATTTAATCAGATACAATTTGAAGGATTTTGTAGGTTCATTGATCAGGGCTTAACAGAAGAACTTTATAAGTTTCCAAAAATTGAAGATACAGATCAAGAAATTGAATTTCAATTATTTGTGGAAACATATCAATTAGTAGAACCCTTGATAAAAGAAAGAGATGCTGTATATGAATCAATTACATATTCTTCTGAATTATATGTATCCGCAGGATTAATTTGGAAAACCAGTAGGGATATGCAAGAACAAACAATTTTTATTGGAAACATTCCTCTAATGAATTCCCTGGGAACTTTTATAATAAATGGAATATACAGAATTGTGATCAATCAAATATTGCAGAGTCCCGGTATCTATTACCGGTCAGAATTGGATCATAATGGAATTTCGGTCTATACTGGCACCATAATATCAGATTGGGGGGGAAGAGTAGAATTAGAGATTGATAGAAAAGCAAGGATATGGGCTCGTGTGAGTAGGAAACAGAAAATATCTATTCTAGTTTTATCATCAGCTATGGGTTTGAATCTAAGAGAAATTTTAGAGAATGTGTGCTACCCTGAAATTTTCTTATCTTTCCTGAATGATAAGGAAAAAAAAAAAATTGGGTCAAAGGAAAATGCCATTTTGGAGTTTTATCAACAATTTACTTGTGTAGGCGGAGATCCAATATTTTCTGAATCCTTATGTAAGGAATTACAAAAGAAATTCTTTCAACAAAGATGTGAATTAGGAAAGATTGGTCGATTAAATATGAACCGGAGACTGAATCTTGATATACCCCATAACAATACATTTTTGTTACCACGAGATATATTGGCAGCTGCAGATCGTTTGATTGAAATGAAATTTGGAATGGGTACACTTGACGATATGAATCATTTAAAAAATAAACGTATTCGTTCTGTAGCGGATCTCTTACAAGATCAATTCGGATTGGCTCTGATTCGTTTAGAAAATGTAGTTAGAGGGACTATATGTGGAGCAATTAGGCATAAATTGATACCGACCCCTCAAAATTTGGTAACTTCAACTCCATTAACAACCACTTATGAATCTTTTTTTGGATTACACCCATTATCTCAAGTTTTGGATCGAACTAATCCATTGACACAAATAGTTCATGGGAGAAAATTGAGTTATTTGGGTCCTGGAGGATTAACAGGACGAACTGCTAGTTTTCGGATACGAGATATCCACCCTAGTCATTATGGGCGCATTTGCCCAATTGACACGTCTGAAGGAATCAATGTTGGACTTATTGGATCTTTAGCAATTCATGCCAAGATTGGTCATTGGGGGTCTTTAGAAAGCCCATTTTATGAAATCTCTGAGGGATCAAAAAAAGTACGGATGTTTTATTTATCGCCAAATAGAGAGGAATACTATATGGTAGCGGCAGGAAATTATTTGGCGCTGAATCGAGGTGTTCAGGAAAAACAGGTTGCTCCGGCTCGATATCGTCAAGAATTCCTGACTATTGCATGGGAACAGGTGCATCTTCGAAGTATTTTTCCCTTCCAATATTTTTCTATTGGAGCTTCCCTCATTCCTTTTATCGAGCATAATGATGCGAATCGGGCTTTAATGAGTTCTAATATGCAACGTCAAGCAGTTCCACTTTCTCGGTCCGAAAAATGCATTGTTGGAACTGGATTGGAACGCCAAGTGGCTCTAGATTCAGGGGTTCCTGCTATAGCCGAACACGAGGGAAAGATAATTTATACTGATATTGACAAGATCATTTTATCGGGCAATGGGGATACTCTACGCATTCCATTAGTTATGTATCAACGTTCCAACAAAAATACTTGTATGCATCAAAAAACCCAGCTTCGGCGGGGTAAATGCATTAAAAAGGGACAAGTTTTAGCAGATGGTGCCGCTACAGTTGGTGGCGAACTTGCCTTGGGCAAAAACGTATTAGTCGCTTATATGCCATGGGAAGGTTACAATTTTGAGGATGCGGTACTCATTAGCGAACGTCTGGTATATGAAGATATTTATACTTCTTTTCACATACGGAAATATGAAATTCAGACTCATGTGACAAGCCAAGGACCTGAAAGGATCACTAACGAAATACCGCATCTAGAGGCCCATTTACTCCGAAATTTAGACAAAAACGGAATTGTGATGCTAGGATCTTGGGTAGAAACGGGCGATATTTTAGTAGGTAAATTAACGCCTCAAATGGCGAAAGAATCATCGTATGCTCCAGAAGATAGATTATTAAGAGCCATACTTGGTATTCAGGTATCTACTTCAAAAGAAACTTGTCTAAAACTACCTATAGGTGGTAGGGGTCGAGTTATTGATGTGAGATGGATTCAGAAAAAAGGGGGTTCCAGTTATAATCCGGAAACGATTCGTGTATATATTTTACAGAAACGTGAAATCAAAGTGGGTGATAAAGTAGCTGGAAGACATGGAAATAAAGGCATCATTTCAAAAATTTTGCCTAGACAAGATATGCCTTATTTGCAAGATGGAAGACCTGTTGATATGGTCTTCAACCCATTAGGAGTACCTTCACGAATGAATGTAGGACAGATATTTGAATGCTCACTCGGGTTAACGGGAGATCTGCTAGATAGACATTATCGAATAGCACCCTTTGATGAGAGATATGAACAAGAGGCTTCGAGAAAACTAGTGTTTTCTGAATTATATGAAGCCAGTAAGCAAACAGCAAATCCGTGGGTATTTGAACCCGAGTATCCGGGAAAAAGTAGAATATTTGATGGAAGAACGGGGGATCCTTTTGAACAGCCTGTTATAATAGGAAAGCCTTATATCTTGAAATTAATTCATCAAGTTGATGATAAAATACATGGACGTTCCAGTGGACATTATGCACTTGTTACACAACAACCCCTTAGAGGAAGGGCCAAGCAAGGTGGACAACGGGTCGGAGAAATGGAGGTTTGGGCTCTAGAGGGATTTGGTGTTTCTCATATTTTACAAGAAATGCTTACTTATAAATCTGATCATATTAGAGCTCGCCAAGAAGTGCTTGGTACTACGATCATTGGAGGAACAATACCTAAACCTGAAGATGCTCCAGAATCTTTTCGATTGCTCGTTCGAGAACTACGATCTTTGGCTCTGGAACTGAATCATTTCCTTGTATCTGAGAAGAACTTCCAGATTAATAGGAAGGAAGCTTAA

>lcl|NC_015308.1_cds_YP_004327657.1_17 [gene=psbD] [locus_tag=HebrCp027] [db_xref=GeneID:10351973] [protein=photosystem II protein D2] [protein_id=YP_004327657.1] [location=36144..37205] [gbkey=CDS]
ATGACTATAGCCCTTGGTAAATTTACCAAAGACGAAAATGATTTATTTGATATTATGGATGACTGGTTACGGAGGGACCGTTTCGTTTTTGTAGGTTGGTCCGGTCTATTGCTCTTTCCTTGTGCCTATTTCGCCGTAGGGGGTTGGTTCACAGGTACAACCTTTGTAACCTCATGGTATACCCATGGATTGGCGAGTTCCTATTTGGAAGGCTGCAACTTCTTAACCGCCGCAGTTTCTACTCCTGCTAATAGTTTAGCACATTCTTTGTTATTATTATGGGGTCCTGAAGCACAAGGAGATTTTACTCGTTGGTGTCAATTAGGTGGTTTGTGGACTTTTGTTGCTCTCCACGGTGCTTTTGGACTAATAGGTTTTATGTTACGTCAATTTGAACTTGCTCGATCTGTGCAATTGCGACCTTATAATGCAATCGCATTCTCTGCTCCAATTGCTGTTTTTGTTTCTGTATTCCTGATTTATCCATTAGGCCAGTCTGGTTGGTTTTTTGCGCCTAGTTTTGGTGTAGCAGCTATATTTCGATTCATCCTCTTTTTCCAAGGGTTTCATAACTGGACGCTGAACCCATTTCATATGATGGGAGTTGCCGGCGTATTGGGCGCTGCTCTGCTATGCGCTATTCATGGTGCTACTGTAGAAAATACTTTATTTGAAGATGGTGATGGTGCAAATACATTCCGTGCCTTTAACCCAACTCAAGCTGAAGAAACTTATTCAATGGTCACCGCTAACCGCTTTTGGTCTCAAATCTTTGGGGTTGCTTTTTCCAATAAACGTTGGTTACATTTCTTTATGTTATTTGTACCAGTAACCGGTTTATGGATGAGCGCTCTTGGAGTGGTCGGTCTGGCTCTGAATCTACGTGCCTATGACTTCGTTTCTCAGGAAATCCGTGCAGCGGAAGATCCTGAATTTGAGACTTTCTACACTAAAAATATTCTCTTAAACGAAGGTATTCGTGCTTGGATGGCGGCTCAAGATCAGCCTCATGAAAACCTTATATTCCCTGAGGAGGTTCTACCACGTGGAAACGCTCTTTAA

>lcl|NC_015308.1_cds_YP_004327651.1_11 [gene=rpoC2] [locus_tag=HebrCp018] [db_xref=GeneID:10351893] [protein=RNA polymerase beta'' subunit] [protein_id=YP_004327651.1] [location=21354..25541] [gbkey=CDS]
ATGGAGGTACTTATGGCCGAACGGGCCAATCTGGTCTTTCACAATAAAGTGATAGATGGAACTGCCATTAAACGACTTATTAGCAGATTAATAGATCATTTTGGAATGGCATATACATCACACATCCTGGATCAAGTAAAGACTCTGGGTTTCCAGCAAGCCACTGCTACATCCATTTCATTAGGAATTGATGATCTTTTAACAATACCTTCTAAGGGATGGCTAGTCCAAGATGCTGAACAACAAAGTTTGATTTTGGAAAAACACTATCATTATGGAAATGTACACGCGATAGAAAAATTACGCCAATCTATTGAGATATGGTATGCTACAAGTGAATATTTGCGACAAGAAATGAACCTGAATTTTAGGATGACAGAACCCTTTAATCCAGTCCATATAATGTCTTTTTCGGGAGCTAGGGGAAATACATCTCAAGTACACCAATTAGTAGGTATGAGAGGATTAATGTCGGATCCACAAGGACAAATGATTGATTTACCCATTCAAAGCAATTTACGCGAGGGACTGTCTTTAACAGAATATATCATTTCTTGCTATGGAGCCCGAAAAGGGGTTGTCGATACTGCTGTACGAACATCAGATGCTGGATATCTTACGCGTAGACTTGTTGAAGTAGTTCAACACATTGTTGTACGTAGAACAGATTGTGGGACCACCCGAGGGATCTCTGTGAGTCCTCGAAATGGGATGATGTCGGAAAGAATTTTGATTCAAACATTAATTGGTCGTGTATTAGCAGACAATATATATATGGGTCTACGATGCATTGCCATTCGAAATCAAGATATTGGGATTGGGCTTGCCAATCGATTCATAACCTTTCGAACACAAACAATATCTATTCGAACTCCCTTTACTTGTAGGAGTACGTCTTGGATCTGTCGATTATGTTATGGTCGGAGTCCTACTCATGGCGATCTAGTAGAATTGGGGGAAGCCGTAGGTATTATTGCGGGTCAATCCATTGGAGAGCCGGGTACTCAACTAACATTAAGAACGTTTCATACCGGCGGAGTATTCACAGGGGGTACTGCAGAACATGTACGAGCCCCCTCTAATGGAAAAATAAAATTTAATGAGGATTTGGTTCATCCCATACGTACACGTCATGGGCATCCTGCTTTTCTATGTTATATAGACTTGTATGTAACTATTGAGAGTCAAGATATTATACATAACGTGACTATTCCACCAAAAAGTTTCCTTTTAGTTCAAAATGATCAATATGTAGAATCAGAACAAGTGATTGCTGAAATTCGGGCGAGAACATACACTTTGAATTTTAAAGAGAAGGTCCGAAAACATATTTATTCCGATTCAGAAGGGGAAATGCACTGGAGTACTGATGTGTACCATGCACCCGAATTTACATATAGTAATGTCCATCTCTTACCAAAAACAAGCCATTTATGGATATTATCAGGAAGTTCGTGCGGATCCAGTATAGTTTCTTTTTCACTACACAAGGATCAAGATCAAATGAATGTTCATTCTCTTTCTGTCAAAAGAAGATATATTTCTAGTCCTTCCGTAAATAATGATCAAGTGAAACACAAATTCTTTAGTTCAGATTTTTCGGGTAAAAAAGAAAGTGAGATTCCTGATTATTCAGAACTTAATCGAATCATATGTACCGGTCATTGTAATCTCATATATCCTACTATTCTCTACGAGAATTCTGATTTATTGGCAAAGAGGCGAAGAAATAAATTCATCATCCCATTCCAATCAATTCAAGAACGAGAGAAAGAACTAATGACCCACTCCGCTATCTCGATTGAAATACCTATAAATGGTATTTTCCGTAGAAATAGTGTTTTTGTTTATTTCGACGATCCCCAATACCGAAGAAAGAGTTCAGGAATTACTAAATATGGGGCTATAGGGGTGCATTCAATCGTCAAAAAAGAAGATTTGATTGAGTATCGGGGAGTCAAAGAATTTAAGCCAAAATACCAAACGAAAGTGGATTGCTTTTTTTTCATTCCCGAGGAAGTGTATATTTTACCCGAATCTTCTTCCCTAATGGTACGGAACAATAGTATCATTGGAGTAGATACACAAATCGCTTTAAATACAAGAAGTCGGGTGGGCGGATTGGTCCGAGTGGAGAGAAAAGAAAAAAAAATGGAACTTAAAATCTTTTCTGGAGATATCCATTTTCCGGGAGAGACAGATAAAATATCCCGACACAGTGGTATCTTAATGCCACCAGGAACGGTAAAAACAAATTCTAAGGAATCAAAAAAAGTGAAAAATTGGATCTATATCCAACGAATCACACCTACCAAGAAAAAGTATTTTGTTTTGGTTCGACCAGTAATCATATATGAGATAGCGAACGGTATAAATTTAGAAACACTTTTCCCCCAGGATCTATTGCAGGAAAAGGATAATCTGAAACTTCGAGTTGTCAATTATATTCTTTATGGGACTGGTAAACCCATTCGGGGAATTTCTGACACAAGTATTCAATTAGTTCGTACTTGTTTAGTGTTGAATTGGGACCAAGACAAAAAAAGTTCTTCTATCGAAGAGGCCCGCGCTTTTTTTGTTGAAATAAGCACAAATGGTCTGATTCGTGATTTCCTAAGAATCAACCTAGTGAAATCCCATATTTCATATATCGGTAGAAAAAGGAATGATCCATCAGGTTCAGAACTGATCTCTAATAATGGGTCAGATCGCACCAATATTAATCCATTTTATCCCATTTATTCCAAGACAAGGATTCAACAATCACTTAAACAAAATCAAGGAACTATTAGTACGTTGTTGAATAGAAATAAGGAATGTAAATCTTTGATAATTTTGTCATCATCTAATTGTTTTCGAATGGATCCATTCAACGATGTAAAACATCACAATGGAATAAAAGAATCAATTAAAAGAGATCCTATAATTCCAATTAGAAATTCGTTGGGCCCTTTAGGAACAGCCCTTCAAATTTCGAATTTTTATTTATTTTACCATTTTAATTTAATATTAATAACTCATAATCAGATCTCGGTAACTAAATATTCGAAACTTGACAATTTAAAACAGACTTTTCAAGTACTTAAATATTATTTAATGGATGAAAACGGGAGAATTGTTAATCCCGATCCATGCAGTAACAGCGTTTTGAATCCATTCAATTTGAATTGGTATTTTCTCCATCATAATTATTGTGAAGAAAGATTCACAATAATTAGCCTGGGACAGTTTATTTGTGAAAATTTATGTATGGCCAAAAAGGGACCACATCTAAAATCGGGTCAAGTTATAATTGTTCACATTGACTCTGTAGTAATAAGATCCGCTAAGCCTTATTTGGCCACTCCAGGAGCAACCGTTCATGGCCATTATGGAGAAATCCTTTACGAAGGAAATACATTAGTTACATTTATATATGAAAAATCGAGATCTGGTGATATAACGCAGGGTCTTCCAAAAGTGGAACAAGTGTTAGAAGTGCGTTCAATTGATTCAATATCAATAAACCTAGAAAAGAGAGTGGAGGGTTGGAACGAGTGTATAACAAGAATTCTGGGAATTCCTTGGGGATTCTTGATTGGTACTGAGCTAACTATAGTGCAAAGTCGTATCTCTTTAGTTAATAAGATCAAAAAGGTTTATCGATCCCAAGGGGTGCAGATCCATAATAGGCATATAGAAATTATTGTACGTCAAATAACATCAAAAGTATTGGTTTCAGAAGACGGAATGTCTAATGTTTTTTCACCCGGAGAACTAATTGGATTGTTGCGAGCGGAACGAACGGGACGCGCTTTGGAAGAAGCCATCTGTTACGGGGCCATATTATTGGGAATAACGCGAGCATCTCTGAATACTCAAAGTTTCATATCCGAGGCTAGTTTTCAAGAAACTACTCGCGTTTTAGCAAAAGCTGCTCTCCGCGGTCGTATCGATTGGTTGAAAGGCCTGAAAGAAAACGTTGTTCTAGGCGGTATGATACCCGTTGGTACCGGATTCAAAGGATTAGTGCAAGGCTCAAGGCAACATAAGAACATTCCTTTGAAAACCAAAAAGAAGCATTTATTCGAGGGGGAATTTAGAGATAGAGATATTTTATTCCACTACAGAGAGTTATTTGATTCTTGCATTTCAAAAAATTTCTATGATACATCAGAACAACCATTTATAGGATTTAATGATTCCTAA

>lcl|NC_015308.1_cds_YP_004327652.1_12 [gene=rps2] [locus_tag=HebrCp019] [db_xref=GeneID:10351894] [protein=ribosomal protein S2] [protein_id=YP_004327652.1] [location=25813..26523] [gbkey=CDS]
ATGATAAGAAGATATTGGAACATTAATTTGGAAGAGATGATGAAAGCAGGAGTTCATTTTGGTCATGGTACTAGAAAATGGAATCCGAGAATGGCACCTTATATCTCTGCAAAGCGTAAAGGTATTCATATTACAAATCTTACTAGAACTGCTCGTTTTTTATCAGAAGCTTGTGATTTAGTTTTTGATGCAGCAAGTAGGAGAAAACAATTCTTAATTGTTGGTACCAAAAATAAAGCAGCGGATTCAGTAGCGCGGGCTGCAATAAGGGCTCGGTGTCATTATGTTAATAAAAAATGGCTCGGCGGTATTTTAACGAATTGGTCCACTACAGAAACTAGACTTCAAAAGTTCAGGGACTTGAGAATGGAACAAAAGACAGGTAGACTCAACCGTCTTCCGAAAGGAGATGCGGCTCGATTGAAGAGACAGTTAACTCACTTGCAAACATATCTGGGCGGGATTAAATATATGACGGGGTTACCCGATATTGTAATAATCGTTGATCAGCAAGAAGAATATACGGCTCTTCGGGAATGTATGACTTTGGGAATTCCAACAATTTGTTTAATTGATACAAACTGTGACCCGGATCTCGCAGATATTTCGATTCCAACGAATGATGACGCTATAGCTTCAATCCGATTAATTCTTAATAAATTAGTATTTGCAATTTGTGAGGGTCATTCTAGCTATATACGAAATCCCTGA

>lcl|NC_015308.1_cds_YP_004327642.1_2 [gene=psbA] [locus_tag=HebrCp002] [db_xref=GeneID:10351963] [protein=photosystem II protein D1] [protein_id=YP_004327642.1] [location=complement(791..1852)] [gbkey=CDS]
ATGACTGCAATTTTAGAGAGACGCGAAAGCGAAAGCCTATGGGGTCGTTTCTGTAACTGGATAACCAGCACTGAAAACCGTCTTTACATTGGATGGTTTGGTGTTTTGATGATCCCTACTTTATTGACCGCAACTTCTGTATTTATTATCGCTTTCATTGCTGCCCCTCCGGTAGATATTGATGGTATTCGTGAACCTGTTTCTGGATCTCTACTTTATGGAAACAATATTATTTCTGGTGCCATTATTCCTACTTCTGCGGCTATAGGTTTGCATTTTTACCCAATATGGGAAGCGGCATCCGTTGATGAATGGTTATACAATGGCGGTCCTTATGAGCTAATTGTTCTACACTTCTTACTTGGTGTAGCTTGTTACATGGGTCGTGAGTGGGAACTTAGTTTCCGTCTGGGTATGCGCCCTTGGATTGCTGTTGCATATTCAGCTCCTGTTGCAGCTGCTACTGCTGTTTTCTTGATTTATCCAATTGGTCAGGGAAGCTTTTCTGATGGTATGCCTCTAGGAATCTCTGGTACTTTCAACTTTATGATTGTATTCCAGGCTGAGCACAACATCCTTATGCACCCATTTCACATGTTAGGCGTAGCTGGTGTATTCGGCGGCTCCCTATTCAGTGCTATGCATGGTTCCTTGGTAACCTCTAGTTTGATCAGGGAAACCACAGAAAATGAATCTGCTAATGAAGGTTACAGATTCGGTCAAGAGGAAGAAACTTATAATATCGTAGCTGCTCATGGTTATTTTGGCCGATTGATCTTCCAATATGCTAGTTTCAACAACTCTCGTTCTTTACATTTCTTCCTAGCTGCTTGGCCTGTAGTAGGTATTTGGTTCACTGCTTTAGGTATTAGCACTATGGCTTTCAACCTAAATGGTTTCAATTTCAACCAATCTGTAGTTGATAGTCAAGGTCGTGTAATTAATACCTGGGCTGATATTATTAACCGTGCTAACCTTGGTATGGAAGTTATGCATGAACGTAATGCTCATAACTTCCCTCTAGACCTAGCTGCTGTCGAAGTTCCATCTACAAATGGATAA

>lcl|NC_015308.1_cds_YP_004327666.1_26 [gene=ndhK] [locus_tag=HebrCp043] [db_xref=GeneID:10351908] [protein=NADH dehydrogenase subunit K] [protein_id=YP_004327666.1] [location=complement(52664..53341)] [gbkey=CDS]
ATGAATTCCATTGAGTTTCCTTTACTTGATCGAACAACTCAAATTTCAGTTATTTCAACTACATCAAATGATCTTTCAAATTGGTCAAGACTCTCCAGTTTATGGCCACTTCTCTATGGTACCAGTTGTTGCTTCATTGAATTTGCTTCATTAATAGGCTCACGATTCGACTTTGATCGTTATGGGCTAGTACCAAGATCTAGTCCTAGACAAGCGGACCTGATTTTAACAGCCGGCACCGTAACCATGAAAATGGCTCCTTCTTTAGTGAGATTATATGAACAAATGCCTGAACCAAAATATGTTATTGCTATGGGGGCATGTACAATTACAGGGGGGATGTTCAGTACCGATTCTTATAGTACTGTTCGGGGAGTTGATAAGCTAATTCCTGTAGATGTCTATTTGCCAGGCTGTCCACCTAAACCGGAGGCGGTTATAGATGCTATAACAAAACTTCGTAAAAAAATATCTCGAGAAATTTATGAAGATCGAATTAGGTCTCAACCGGGGAAACGGTGTTTTACTACTAATCACAAGTTTAATATTGAACGCACTACTCATACTGGAAATTATGATCAAGAATTACTCTATCAATCGCCGTCTACTTCAAAGATCCCTCCGGAAACATTTTTCAAATATAAAAGGTCAGTATCGTCCAACGAATTAGTAAATTAG

>lcl|NC_015308.1_cds_YP_004327717.1_77 [gene=ndhH] [locus_tag=HebrCp114] [db_xref=GeneID:10351958] [protein=NADH dehydrogenase subunit 7] [protein_id=YP_004327717.1] [location=complement(128088..129269)] [gbkey=CDS]
ATGAATGTACCAGCTACACGAAAAGACCTTATGATAGTTAATATGGGCCCCCACCACCCATCAATGCATGGTGTTCTTCGCCTCATCGTTACTCTAGACGGGGAAGATGTTATTGACTGTGAACCAATATTAGGTTATTTACACAGAGGAATGGAAAAAATTGCGGAAAATCGAACAATTATACAATATTTGCCCTATGTAACACGTTGGGATTATTTGGCTACTATGTTCACAGAAGCAATAACAGTAAATGGTCCAGAACTGTTAGGAAATATTCAAGTGCCTAAAAGAGCTGGCTATATCAGAATAATTATGTTGGAATTGAGTCGTATAGCTTCTCATTTGTTATGGCTTGGTCCCTTTATGGCAGATATTGGTGCACAGACTCCTTTCTTCTATATTTTTAGAGAAAGAGAGTTAGTATATGATTTATTCGAAGCTGCCACTGGTATGAGAATGATGCATAATTTTTTTCGTATCGGGGGAGTAGCGTCTGATCTACCTCATGGTTGGATAGATAAATGTTTGGATTTTTGCGATTATTTTTTAACAGGAGTTACTGAATATCAAAAACTTATTACGCGAAATCCTATTTTTTTAGAACGAGTTGAGGGAGTAGGTATTGTTGGTACAGAGGAAGCAAAAAATTGGGGTTTATCGGGACCAATGCTACGAGCTTCCGGAGTACAATGGGATCTTCGTAAAGTTGATCATTATGAGTGTTACGACGAATTTGATTGGGAAATCCAGTGGCAAAAAGAAGGAGATTCCTTAGCTCGTTATTTAGTCCGAATTGGTGAAATGATGGAATCTATAAAAATTATTCAACAGGCTCTGGAAGGAATTCCGGGGGGGCCCTATGAGAATTTAGAAATCCGACGTTTTGATAGAGAAAGGGATTCGGAATGGAACGATTTCGAATATCGATTCATTAGTAAAAAAACTTCTCCTACTTTTGAATTACCGAAACAAGAACTTTATGTGAGAGTCGAAGCCCCAAAAGGAGAATTGGGAATTTTTCTGATAGGAGATCAGAGCGGTTTTCCTTGGAGATGGAAAATTCGTCCGCCGGGTTTTATCAATTTGCAAATTCTTCCTGAATTAGTTAAAAGAATGAAATTGGCCGATATTATGACAATACTAGGTAGTATAGATATCATTATGGGAGAAGTTGATCGTTGA

>lcl|NC_015308.1_cds_YP_004327664.1_24 [gene=rps4] [locus_tag=HebrCp038] [db_xref=GeneID:10351977] [protein=ribosomal protein S4] [protein_id=YP_004327664.1] [location=complement(48698..49303)] [gbkey=CDS]
ATGTCACGTTACCGAGGGCCTCGTTTCAAAAAAATACGTCGTCTGGGGGCTTTGCCGGGACTAACTAGTAAAAGGCCTAAAGTCGGGAGCGATCTTAGAAATCAATCACGCTCCGGTAAAAAATCTCAATATCGTATTCGTTTAGAAGAAAAACAAAAATTGCGTTTTCATTATGGTCTTACAGAACGACAATTACTTAAATATGTTCGTATCGCCGCAAAAGCCAAAGGGTCAACAGGTCAGGTTTTACTACAATTACTTGAAATGCGTTTGGATAACATCCTTTTTCGATTGGGTATGGCGTCAACTATTCCTCGAGCCCGCCAATTAGTTAATCATAGACATATTTTAGTTAATGGTCGTATAGTAGATATACCAAGTTATCGCTGCAAACCCCGAGATATTATTACAGCGAGAGCTGAACAAAAATCTAGAGCTATGATTAAAAATTATCTTGATTCATCCCCCCAGGAGGAATTGCCAAAACATTTGACTCTTCACCCATTCCAATATAAAGGATTGGTCAATCAAATAATAGATAGTAAATGGGTTGGCTTGAAAATAAATGAATTACTAGTGGTAGAATATTATTCTCGTCAGACTTAA

>lcl|NC_015308.1_cds_YP_004327708.1_68 [gene=ndhF] [locus_tag=HebrCp104] [db_xref=GeneID:10352004] [protein=NADH dehydrogenase subunit 5] [protein_id=YP_004327708.1] [location=complement(116048..118279)] [gbkey=CDS]
ATGGAACATATATATCAATATTCATGGATCATATCTTTCGTTACATTGCCAGTCCCTATGTTAATAGGAGTGGGACTCCTGCTTTTTCCGGCAGCAACAAAAAAACTGCGTCGTATGTGGGCTTTTCCAAGCGTTTTCTTGTTAAGTATAGTCATGATTTTTTCAATCGATTTGTCTATTCAGCAAATAAATAGTAGTTTTATTTATCAATATATATGGTCGTGGACTATTAATAATGATTTTTCTTTAGAATTCGGACACTTGATTGACCCACTTACTTCTATTTTGTCAGTATTAATTACTACAGTTGGTATTTTGGTTCTTTTTTATAGTGACAATTATATGTCTCATGATCAAGGTTATTTGAGATTTTTTGCTTATATGAGTTTTTTCACTACTTCAATGTTGGGATTAGTTACTAGTTCTAATTTGATACAAATTTATATTTTTTGGGAATTGGTTGGAGTGTGTTCTTATCTATTAATAGGTTTTTGGTTCACACGACCTATTGCATCGAATGCTTGTCAAAAAGCGTTTGTAACTAATCGTGTAGGGGATTTTGGTTTATTATTAGGGATTTTAGGTCTTTATTGGATAACGGGCAGTTTCGAATTTCGGGATTTGTTCAAAATCTTCAATAACTTGATTTATAATAATCAAGTTAATTTTTTATTTGTTACTTTGTGTGCATTTCTATTATTTTCTGGCGCAATTGCTAAATCGGCGCAATTTCCTCTTCATGTATGGTTACCAGATGCCATGGAAGGGCCTACTCCTATTTCAGCTCTGATACATGCTGCTACTATGGTAGCGGCGGGAATTTTTCTTGTAGCTCGCCTTTTTCCTCTTTTCGTAATTATACCTTTCATAATGAATCTAATAGCTTTGATAGGTATAATAACAGTATTTTTAGGAGTTACTTTAGCTCTTGCTCAAAAAGATATTAAGAGAAGTTTAGCCTATTCTACAATGTCTCAATTGGGTTATACGATGTTAGCTCTAGGTATGGGGTCTTATCGGGCTGCTTTATTTCATTTGATTACTCATGCCTATTCGAAAGCATTGTTGTTTTTAGGATCTGGATCCATTATTCATTCAATGGAAGCTATTCTTGGTTATTCTCCAGATAAGAGTCAAAATATGGTTCTTATGGGTGGTTTAACAAAACATATTCCAATTACAAAAACGGCTTTTTTATTAGGAACACTTTCTCTTTGTGGTATTCCACCCTTCGCCTGTTTTTGGTCCAAAGATGAAATTCTTAATGATAGTTGGTTGTATTCACCTATTTTCGCAATAATAGCCTGTTTCACAGCAGGATTAACTGCATTTTATATGTTTCGGGTTTATTTACTTACTTTTGACGGACATTTTAATGCTCATTTTAAAAATTACAGTGGTAAAAAAAACAGTTCATTTTATTCAATCTCTTTATGGGGTAAAGAAGGATCAAAAATGCTTAACAAAAATTTTCGTTTATTAGCTTTATTAACAATGAATAATAAGGAAAGGGACTCTTTTTTTGGTAAGAACACATATCAAATTGATGGTAATGTAAGAAATATGACGTGGCCCTTTATTACTGTTAAAAATTTTAACACTAAAAGGATTTTTTCCTATCCCCATGAATCGGATAATACTATGTTATTTCCTATGCTTATCTTAGTACTATTTACTTTGTTTATTGGAGCCATAGGAATTCCTTTCAATCAATTCAATCAAGAAGGAATACAGTTGGATATAGATATATTGTCAAAACTTTTAACCCCGTCTTTAAACCTTTTGCATCAAAATCCAGAAAATTCTGTGGATTGGTATGAATTTGTAACAAATGCAATTTTTTCAGTCAGTATAGCTTTTTTCGGAATATTTATAGCGTCCTTTTTATATAAGCCTGTTTATTCATCGTTACTAAATTTTAATTTCTTTAATTCGTTTGCTAAAAAAGGCCCTAAGAGAATTTTTTGGGACAAAATAATAAATGTGATATATAATTGGTCCTCTAATCGAGGTTATATAGATGCTTTTTATGCAATATCTTTTATTGGGGGTATAAGAAAATTGGTTGAATTAATTCATTTTTTTGATAAACGAATAATTGATGGAATCACCAATGGGGTCGGTGTTACCAGTTTCTTTGTAGGAGAGGGTATAAAATATGTAGGAAGTGGTCGCATCTCTTCTTATCTCTTATTTTATTTATTTTATGCATTAATTTTTTTATTAATTTGA

>lcl|NC_015308.1_cds_YP_004327699.1_59 [gene=rpl16] [locus_tag=HebrCp080] [db_xref=GeneID:10351940] [protein=ribosomal protein L16] [protein_id=YP_004327699.1] [location=complement(join(86026..86424,87609..87617))] [gbkey=CDS]
ATGCTTAGTCCCAAAAGAACCCGATTCCGTAAACAACATAGAGGAAGAATGAAAGGAATAGCTTTTCGAGGTAATCGTATTTGTTTCGGCAGATATGCTCTTCAGGCACTTGAACCCGCTTGGATTACATCTAGACAAATAGAAGCGGGGCGACGAGCAATGACACGAAATGCACGCCGCGGTGGAAAAATATGGGTACGCATATTTCCCGACAAACCGGTTACTTTAAGACCTACGGAAACACGTATGGGTTCGGGGAAAGGATCTCCCGAATATTGGGTAGCTGTCGTTAAACCAGGTAGAATACTTTATGAAATGGGCGGAGTAGCAGAAAATATAGCGAGAAAAGCTATTTCAATAGCAGCATCAAAAATGCCTATACGAACTCAATTCATTATTTCGGGATAG

>lcl|NC_015308.1_cds_YP_004327701.1_61 [gene=rpl22] [locus_tag=HebrCp082] [db_xref=GeneID:10351942] [protein=ribosomal protein L22] [protein_id=YP_004327701.1] [location=complement(88549..88950)] [gbkey=CDS]
ATGATAAATAAAAGAAAGAGAAAGAGAGACCCATATACAGAAGTATATGCTTTAGGCCAACATATATGTATGTCCCCTCACAAAGCACGAAGAATAATTGATCAGATTCGTGGACGTTCTTACGAAGAAACACTTATGATACTCGAGCTCATGCCTTATCGAGCATGTTATCCCCTTTTTAAATTGATTTATTCTGCAGCAGCAAATGCTAGTCACAATATGGGTTTCAACGAAGTCAATTTAATCATTAGTAAAGCCGAAGTCAATGAAGGCACTACTGTGAAAAAATTAAAACCTCAGGCTCGAGGACGGGGTTATCTGATAAAAAGATCAACTTGTCATATAACTATTATATTAAAAAATATATCCTTATATGAAGAATATGAAGAATATAACATATGA

>lcl|NC_015308.1_cds_YP_004327697.1_57 [gene=rps8] [locus_tag=HebrCp078] [db_xref=GeneID:10351985] [protein=ribosomal protein S8] [protein_id=YP_004327697.1] [location=complement(84888..85292)] [gbkey=CDS]
ATGGGTAGGGATCCTATTGCTGACATAATAACCTCTATAAGAAATGCTGACATGAATAGAAAAGGAATCGTTCGAATAGCATCTACTAACATCACCGAAAACATTATTAAAATACTTTTAAGAGAAGGTTTTATTGAAAATGTCAGGAAACATAAGGAGGGCAACAAAAAATTTTTGGTTTTAACCCTACGACACAGAAGGAAGAGGAAAGAACCCTATAGAACTAGTCTAAATTTAAAACGGATCAGCCGACCTGGTCTACGAATCTATTCTAACTATCAAAAAATTCCTAGAATTTTGGGCGGGATGGGCATTGTAATTCTTTCTACTTCTCGGGGTATAATGACAGACCGAGAAGCTCGACTCGAAAGAATCGGCGGAGAAATCTTGTGTTATATATGGTAA

>lcl|NC_015308.1_cds_YP_004327703.1_63 [gene=rpl2] [locus_tag=HebrCp084] [db_xref=GeneID:10351944] [protein=ribosomal protein L2] [protein_id=YP_004327703.1] [location=complement(join(89373..89807,90481..90879))] [gbkey=CDS]
ATGGCGATACATTTATACAAAACTTCTACCCCGAGCACACGCAATGGAGCCGTAGACAGTCAAGCGAAATCCAATACACGAAATACACGAAAGAATTTGATCTATGGACAGCATCGTTGTGGTAAAGGCCGTAATGCCAGAGGAATCATTACCGCAAGACATAGAGGGGGAGGTCATAAGCGTCTATACCGTAAAATCGATTTTCGACGGAATGAAAAAGACATATATGGTAGAATCGTAACCATAGAATACGACCCTAATCGAAATGCATACATTTGTCTCATACACTATGGGGATGGTGAGAAGAGATATATTTTACATCCCAGAGGGGCTATAATTGGAGATACCGTTATTTCTGGTACAGAAGTTCCTATAAAAATGGGAAATGCCCTACCTTTGACCGATATGCCCTTAGGCACGGCCATACATAACATAGAAATCACACTTGGAAAGGGTGGACAATTAGCTAGAGCTGCAGGTGCTGTAGCGAAACTGATTGCAAAAGAGGGGAAATCAGCCACATTAAAATTACCTTCGGGGGAGGTTCGTTTAATATCCAAAAACTGCTCAGCAACAGTCGGACAAGTAGGGAATACTGGGGTGAACCAGAAAAATTTGGGTAGAGCCGGATCTAAATGTTGGCTAGGTAAGCGTCCTGTAGTAAGAGGAGTAGTTATGAACCCTGTAGACCATCCCCATGGGGGTGGTGAAGGGAGGGCCCCAATTGGTAGAAAAAAACCCGCAACCCCTTGGGGTTATCCTGCACTTGGAAGAAGAAGTAGAAAAAGGAATAAATATAGTGATAATTTGATTCTTCGTCGCCGTAGTAAATAG

>lcl|NC_015308.1_cds_YP_004327715.1_75 [gene=ndhI] [locus_tag=HebrCp112] [db_xref=GeneID:10351956] [protein=NADH dehydrogenase subunit I] [protein_id=YP_004327715.1] [location=complement(125309..125806)] [gbkey=CDS]
ATGTTTCCCATGGTAACTGGGTTCATGAATTATGGGCAACAAACCATACGAGCTGCAAGGTACATTGGTCAAGGTTTTATGATTACCTTATCTCATGCAAATCGTTTACCTGTAACTATTCAATATCCTTATGAAAAATTAATCACATCGGAGCGTTTCCGCGGTCGAATTCATTTTGAATTTGATAAATGCATTGCTTGTGAAGTATGTGTTCGTGTATGTCCTATAGATCTACCTGTTGTTGATTGGAAATTGGAAACTGACATTCGAAAGAAACGGTTGCTTAATTACAGTATTGATTTCGGAATCTGTATATTTTGTGGCAACTGTGTTGAGTATTGTCCGACAAATTGTTTATCGATGACTGAAGAATATGAGCTTTCTACTTATGATCGTCACGAATTGAATTATAATCAAATTGCTTTAGGTCGTTTACCAATGTCAGTAGTTGACGATTATACAATTCGAACAATTTTGAATTCAACTCAAAAAAAATAG

>lcl|NC_015308.1_cds_YP_004327693.1_53 [gene=petD] [locus_tag=HebrCp073] [db_xref=GeneID:10351934] [protein=cytochrome b6/f complex subunit IV] [protein_id=YP_004327693.1] [location=join(81106..81113,81964..82459)] [gbkey=CDS]
ATGGGAGTAACAAAAAAACCTGACTTGAATGATCCTGTATTAAGAGCTAAATTGGCTAAGGGAATGGGTCATAATTATTACGGAGAACCTGCATGGCCCAATGATCTTTTATATATTTTTCCAGTAGTAATTCTAGGTACTATTGCATGTAATGTAGGATTAGCAGTTCTAGAACCATCAATGATTGGTGAACCTGCGGATCCATTTGCAACGCCTTTGGAAATATTGCCTGAATGGTATTTCTTTCCCGTATTTCAAATACTTCGTACAGTACCCAATAAGTTATTAGGTGTTCTTTTAATGGTTTCAGTACCTGCAGGATTATTAACAGTACCTTTTTTGGAGAATGTTAATAAATTCCAAAATCCATTTCGTCGTCCAGTTGCGACAACCGTCTTTTTGATTGGTACTGCGGTAGCCCTTTGGTTAGGTATTGGAGCAACATTACCTATTGATAAATCCCTAACTTTAGGTCTTTTTCAAATTGATTCAATTGTAAAATAA

>lcl|NC_015308.1_cds_YP_004327667.1_27 [gene=ndhC] [locus_tag=HebrCp044] [db_xref=GeneID:10351909] [protein=NADH dehydrogenase subunit 3] [protein_id=YP_004327667.1] [location=complement(53393..53755)] [gbkey=CDS]
ATGTTTCTGATTTACGAATATGATATATTCTGGGCATTTCTAATAATATCAAGTGTTATTCCTATTTTAGCATTTCTAATTTCCGGAGTTTTATCCCCGATTAGCAAAGGGCCGGAGAAACTTTCTAGTTATGAATCGGGTATAGAACCAATGGGTGATGCTTGGTTACAATTTCGAATCCGTTATTATATGTTTGCTCTAGTTTTTGTTGTTTTTGATGTTGAAACAGTTTTTCTTTATCCATGGGCAATGAGTTTCGATATATTGGGGTTATCCGTATTTATAGAAGCTTTCATTTTCGTGCTTATCCTAATTGTTGGTTCAGTTTATGCATGGAGAAAAGGAGCATTAGAATGGTCTTAG

>lcl|NC_015308.1_cds_YP_004327716.1_76 [gene=ndhA] [locus_tag=HebrCp113] [db_xref=GeneID:10351957] [protein=NADH dehydrogenase subunit 1] [protein_id=YP_004327716.1] [location=complement(join(125888..126427,127535..128086))] [gbkey=CDS]
ATGATAATTGATACAACAGAAATACAAGCTATCAATTCTTTTTCTAGATTAGAATCCTTAAACGAGGTCTATGGAATTATATGGGTGTTTGTCCCGATTTTTATTCTTGTATTGGGAATCACGATAGGCATACTAGTAATTGTATGGTTAGAAAGAGAAATATCTGCAGGGATACAACAACGTATTGGACCTGAATATGCCGGTCCTTTAGGAGTTCTTCAAGCTCTAGCGGATGGGACAAAACTACTTTTCAAAGAGAATCTTTTTCCATCTAGGGGGGATATTCGTTTATTCAGTATCGGACCATCCATAGCAGTCATATCAACTCTATTAAGCTATTCGGTAATTCCTTTTGGCTATCACCTTGTTTTAACTGATCTAAATATTGGTGTTTTTTTATGGATTGCCATTTCAAGTATTGCTCCCATCGGACTTCTTATGTCAGGATATGGATCAAATAATAAATATTCCTTTTTGGGTGGTTTACGAGCTGCTGCTCAATCGATTAGTTATGAAATACCATTAACTCTTTGTGTGTTATCCATATCTCTATTATCTAATAGTTCAAGTACAGTTGATATAGTTGAGGCACAATCAAAATCTGGTTTTTGGGGGTGGAATTTGTGGCGTCAACCTATAGGATTTATCATTTTTTTTATTTCTTCTCTAGCAGAATGTGAGAGATTGCCTTTTGATTTACCAGAAGCAGAAGAAGAATTAGTAGCAGGTTATCAAACCGAATATTCGGGCATCAAATTTGGTTTATTTTATATTGCTTCCTATCTAAACTTATTAGTTTCTTCATTATTTGTAACAGTTCTTTACTTGGGCGGTTGGAATATCTCTATTCCGTATATATTCGTTCCTGAGCTTTTTGAAATAAAAAAAATAGGCGGAGTCTTTGGAACAACAATTGGTATCTTTATTACATTGGTTAAAACTTATTTGTTCTTGTTCATTCCTATCACAACAAGATGGACTTTACCTAGACTAAGAATGGACCAACTTTTAAATCTTGGATGGAAATTTCTTTTACCTATTTCTCTCGGTAATCTATTATTAACAACCTCTTTCCAACTTCTTTCACTATAA

>lcl|NC_015308.1_cds_YP_004327671.1_31 [gene=accD] [locus_tag=HebrCp050] [db_xref=GeneID:10351913] [protein=acetyl-CoA carboxylase beta subunit] [protein_id=YP_004327671.1] [location=60462..61970] [gbkey=CDS]
ATGGAAAAATGGCGGTTCAATTCGATCTTATCCAATGTGGAATTAGGATACAGGTGTAGGCTAAGTAAATCAATGGATAGTTTCAGTTCTCTTGAAAATACCAGTATAAGTGAAGACCCAATTCTAAATGATACAGATAAAAACACCTATAGTTGGAGTAATAGTGACAGCTCTAGTTACAGTAATGTTGATCATTTAGTCGGTGTCAGGGACATTCAGAATTTCAGCGCCGATGAAACTTTTTTAGTTAGGGATAGTAATAGGGACAGTTATTCCATATATTTTGATATTGAAAATAAAGTTTTTGAGATTGACAATGATCATTCTTTTCTGAGTGAACTAAAAAGTTCTTTTTATAGTTATTGGAATTCTAGTTATCTGAATAATGGGTTTAGGAGTGGCGACTCCCACTATGATCATTATATGTATGATACTAAATATAGTTGGAATAATTACATCAATAGTTGCATTGACAGTTATCTTCGCTCTCAAATCTGTATTGATAGTTATATTTTAAGTGGTAGTAACAATTACAGCGAAAGTTACATTTATAGTTACATTTGTGGTGAAAGTGGAAATAGTAGTGAAAGCGATAGTTCCAGTCTAAGAACTAGCACGAGTGGTAGCGATTTAATTATAAGAGAAAATTCTAATGATCTCGATATAACTCAAAAATACAAGCATTTGTGGGTTCAATGCGAAAATTGTTATGGATTAAATTATAAGAAATTTTTTAAGTCAAGAATGAATATTTGTGAACAATGTGGATATCATTTGAAAATGAGTAGTTCAGATAGAATTGAACTTTTGATTGACCTAGGCACTTGGGATCCTATGGATGAAGACATGGTATCTCTGGATCCCATTGAATTTCATTCAGAAGAGGAACCTTATAAAGATCGTATTGATTCTTATCAAAGAAAGACAGGATTAACTGAGGCTGTTCAAACAGGCACAGGTCAACTAAACGGCATTCCCGTAGCAATTGGGGTTATGGATTTTCAGTTTATGGGGGGTAGTATGGGATCCGTAGTAGGTGAGAAAATCACTCGTTTGATCGAGTATGCTACCAATAAATTTTTACCTCTTATTTTAGTGTGTGCTTCCGGAGGAGCACGCATGCAAGAAGGAAGTTTGAGCTTGATGCAAATGGCTAAAATATCTTCTGCATTATATGATTATCAATCAAATAAAAAGTTATTTTATGTATCAATCCTTACATCTCCTACGACTGGTGGGGTGACAGCTAGTTTTGGTATGTTGGGGGATATCATTATTGCTGAACCTAATGCCTATATTGCGTTTGCAGGTAAAAGAGTAATTGAACAAACATTGAATAAGACAGTACCTGAAGGTTCGCAATCGGCCGAATTTTTATTCCATAAGGGCTTATTTGATCTAATCGTACCGCGTAATCTTTTAAAAGGCGTTCTGAATGAGTTACTTCAGCTCCACGATTTCTTTCCTTTGAATCATAAATCAAGTAGAAACCTTAAGTTAAAAAGTTAA

>lcl|NC_015308.1_cds_YP_004327686.1_46 [gene=rps12] [locus_tag=HebrCp127] [db_xref=GeneID:10351928] [protein=ribosomal protein S12] [exception=trans-splicing] [protein_id=YP_004327686.1] [location=join(complement(74171..74284),146419..146650,147187..147212)] [gbkey=CDS]
ATGCCAACTATTAAACAACTTATTAGAAACACAAGACAGCCAATCAGAAATGTCACCAAATCCCCCGCTCTTGGGGGATGTCCTCAGCGCCGAGGAACATGTACTAGGGTGTATACTATCACCCCCAAAAAACCAAACTCTGCCTTACGTAAAGTTGCCAGAGTACGATTAACCTCTGGTTTTGAAATCACTGCTTATATACCTGGTATTGGCCATAATTTACAAGAACATTCTGTAGTCTTAGTAAGAGGGGGAAGGGTTAAGGATTTACCCGGTGTGAGATATCACATTGTTCGAGGAACCCTAGATGCTGTCGGAGTAAAGGATCGTCAACAAGGGCGTTCTAAATATGGGGTCAAAAAGCCAAAATAA

>lcl|NC_015308.1_cds_YP_004327665.1_25 [gene=ndhJ] [locus_tag=HebrCp042] [db_xref=GeneID:10351980] [protein=NADH dehydrogenase subunit J] [protein_id=YP_004327665.1] [location=complement(52069..52545)] [gbkey=CDS]
ATGCAGGGTCGTTTGTCTGCTTGGCTAGTCAAACATGGGCTAGTTCATAGATTTTTGGGTTTTGATTACCAAGGAATAGAGACTTTACAAATAAAGCCCGAAGATTGGCATTCCATTGCTGTCATTTTATATGTATATGGTTACAATTATCTGCGTTCGCAATGTGCCTATGATGTAGCACCGGGCGGGCTGTTAGCTAGTGTATATCATCTTACGAGAATAGAGTATGATATAGATCAACCAGAAGAAGTATGTATAAAAGTATTTGCCCCAAGGAAGAATCCTAGAATTCCGTCTGTTTTCTGGGTTTGGAAAAGTGCGGATTTTCAAGAAAGGGAATCTTATGATATGCTGGGAATCTTTTATGATAATCATCCACGCTTGAAACGTATCTTAATGCCGGAAAGTTGGATAGGGTGGCCCTTACGTAAGGATTATATTGTTCCCAATTTTTATGAAATACAAGATGCTCATTGA

>lcl|NC_015308.1_cds_YP_004327656.1_16 [gene=atpA] [locus_tag=HebrCp023] [db_xref=GeneID:10351898] [protein=ATP synthase CF1 alpha subunit] [protein_id=YP_004327656.1] [location=30166..31689] [gbkey=CDS]
ATGGTAACCATTCGAGCCGACGAGATTAGTAATATTATCCGCGAACGTATTGAGCAATATAATAGGGAAGTAAAGATTGTAAATACCGGTACCGTACTTCAAGTAGGCGACGGCATTGCTCGTATTTATGGTCTTGATGAAGTAATGGCAGGCGAATTAGTAGAATTTGAAGAGGGTACAATAGGCATTGCTCTGAATTTGGAATCAAATAATGTCGGTGTTGTATTAATGGGTGACGGTTTAATGATACAAGAGGGAAGCTCCGTAAAAGCAACAGGAAGAATTGCTCAGATACCTGTGAGTGAGGCTTATTTGGGTCGTGTTATAAATGCCCTAGCTAAACCTATTGACGGTCGAGGTGAAATTTCAGCTTCTGAATCTCGGCTAATTGAATCCCCCGCTCCAGGTATTATTTCGAGACGTTCTGTATATGAGCCTCTTCAAACAGGACTTATTGCTATTGATTCGATGATCCCTATAGGACGTGGTCAACGAGAATTAATTATTGGGGATAGACAGACCGGTAAAACAGCAGTAGCCACAGATACAATTCTCAATCAACAAGGACAAAATGTAATATGTGTTTATGTAGCTATTGGGCAAAAAGCGTCTTCTGTGGCTCAGGTAGTGACTACTTTACAGGAAAGAGGGGCAATGGAGTACACTATTGTGGTAGCCGAAACGGCGGATTCTCCGGCTACATTACAATACCTCGCCCCTTATACAGGAGCGGCTCTGGCTGAATATTTTATGTACCGTGAACGACACACTTTAATCATTTATGATGATCTCTCCAAACAAGCGCAGGCTTATCGCCAAATGTCTCTTCTATTACGAAGACCACCTGGTCGTGAAGCTTATCCAGGAGATGTCTTTTATTTGCATTCACGCCTTTTGGAAAGAGCTGCTAAATTAAGTTCTCGTTTAGGTGAAGGAAGTATGACTGCTTTACCAATAGTCGAGACCCAATCAGGAGACGTTTCGGCTTATATTCCTACTAATGTAATTTCCATTACAGATGGACAAATATTCTTATCCGCCGATCTATTCAATGCTGGAATCAGGCCTGCTATTAATGTGGGTATTTCTGTTTCCAGAGTAGGATCCGCAGCTCAAATAAAAGCTATGAAACAGGTAGCTGGTAAGTTAAAATTGGAATTGGCGCAATTCGCAGAATTAGAAGCCTTTGCGCAATTCGCTTCTGATCTCGATAAAGCTACTCAGAATCAATTGGCAAGAGGTCAACGACTACGCGAGTTGCTCAAACAATCCCAATCAGCTCCTCTCACGGTAGAGGAACAAATAATGACTATTTATACCGGAACGAATGGTTATCTTGATTCATTAGAAATCGGACAAGTAAGGAAATTTCTCGTTGAGTTACGTACCTACTTAAAAACGAATAAACCTCAGTTCCAAGAAATCATATCTTCTACCAAAACATTCACCGAAGAAGCAGAAACCCTTTTGAAAGAAGCTATTCAGGAACAGAAGGAACGTTTTGTAATTCAGGAACAAGTATAA

>lcl|NC_015308.1_cds_YP_004327673.1_33 [gene=ycf4] [locus_tag=HebrCp052] [db_xref=GeneID:10351915] [protein=photosystem I assembly protein Ycf4] [protein_id=YP_004327673.1] [location=63287..63841] [gbkey=CDS]
ATGAGTTGGCGATCAGAACGTATATGGATAGAACTTATAGCGGGGTCTCGAAAAACAAGTAATTTCTGCTGGGCCTTTATACTTTTTTTAGGTTCATTGGGATTTTTATTGGTTGGAATTTCCAGCTATCTTGGCAGAAATTTGATATCTTTATTTCCGTCTCAGCAAATAATTTTTTTCCCACAAGGGATCGTGATGTCTTTCTATGGGATCGCCGGTCTATTTATTAGTTCTTATTTGTGGTGCACAATTTTGTGGAATGTAGGTAGTGGTTATGATCGATTCGATAGAAAAGAAGGAATAGTGTGTATTTTTCGCTGGGGATTTCCGGGAAAAAATCGTCGCATCTTACTACGATTCCTTATGAAAGATATTCAGTCTATTAGAATAGAAGTTAAAGAGGGTATTTATGCTCGGCGTGTCCTTTATATGGAAATCAGAGGCCGGGGGGCTATTCCTTTGACTCGTACTGATGAGAATTTGACTCCACGAGAAATTGAGCAAAAAGTAGCGGAATTGGCCTATTTTTTGCGTGTACCAATTGAAGTATTTTGA

>lcl|NC_015308.1_cds_YP_004327711.1_71 [gene=ndhD] [locus_tag=HebrCp108] [db_xref=GeneID:10351952] [protein=NADH dehydrogenase subunit 4] [protein_id=YP_004327711.1] [location=complement(121688..123190)] [gbkey=CDS]
ATGAATTCTTTTCCTTGGTTAACAATATTTGTAGTTTTACCGATATCCGCGGGTTCCTTAATTTTCCTTTTCCCTCATAGAGGAAATAAAGTAATTAAGTGGTATACTATATTTATATGTGCCTTTGAACTCCTTTTAATGAATTATGTGTTCTCTTATTATTTCCAATTGGACGATCCATTAATCCAATTAACAGAAGATTATAAATGGATCCAATTTTTTGATTTTTACTGGAGATTGGGAATCGATGGATTTTCTTTAGGACCTATTTTACTGACAGGATTTATCACTACTTTAGCTACTTTAGCGGCTCGGCCAATTACTCGGGATTCTCGATTATTCCATTTTCTGATGTTAGCAATGTATAGTGGTCAAATAGGATTATTTTCTTCTCAAGATCTTTTACTTTTTTTTATCATGTGGGAGTTAGAATTAATTCCCGTTTATCTACTTCTATCCATGTGGGGGGGAAAGAAACGTCTGTATTCAGCTACAAAGTTTATTTTGTATACTGCGGGAGGTTCCGTTTTTTTATTAATGGGAGCTTTGGGTATCGCTTTATATGGTTCTAATGAACCGAGATTCCATTTTGAAACATCAGCTAATCAATCATATCCTGTGGCGCTAGAAATATTTTTCTATATTGGATTTCTTATTGCTTTTGCTGTCAAATCACCGATTATACCCTTACATACATGGTTACCAGACACCCATGGGGAAGCACATTATAGTACTTGTATGCTTCTAGCCGGAATCTTATTAAAAATGGGGGCATATGGATTGGTTCGAATCAATATGGAATTATTATCTCACGCTCATTCTATTTTTTCTCCCTGGTTGATAATAGTAGGCGTAATGCAAATAATCTATGCAGCTTCAACATCTCCTGGTCAACGAAATTTAAAAAAAAGAATAGCCTATTCTTCTGTATCTCATATGGGTTTCATAATTATAGGAATTTGCTCTATAAGTGATATGGGACTCAATGGAGCCATTTTACAAATAATATCGCATGGATTTATTGGTGCCGCACTTTTTTTCTTGGCAGGAACGGGTTATGATAGAATACGTCGTGTTTATCTTGATGAAATGGGTGGAATGGCTACCTCAATGCCAAAAATATTCACGACATTCAGTATCTTATCACTAGCTTCCCTTGCATTACCAGGCATGAGCGGTTTTTTTGCGGAATTGATAGTATTTTTTGGAATAATTACCGGCCAAAAATATCTTTTAATGTCAAAAATATTAATTACTTTTGTAATGGCAGTTGGAATGATATTAACTCCTATTTATTTATTATCTATGTTACGCCAGATGTTCTATGGATATAAGCTGTTTAATGCCCCAAACTCTTCTTTTTTTGATTCTGGACCGCGGGAGTTATTTGTTTCGATCTCTATCCTTCTGCCTGTAATAGGTATTGGTATTTATCCGGATTTCGTTTTCTCATTATCAGTTGACAGGGTTGAAGCTATTCTATCTAATTATTTTTATAGATAG

>lcl|NC_015308.1_cds_YP_004327707.1_67 [gene=rps7] [locus_tag=HebrCp091] [db_xref=GeneID:10351991] [protein=ribosomal protein S7] [protein_id=YP_004327707.1] [location=complement(102663..103130)] [gbkey=CDS]
ATGTCACGTCGAGGTACTGCAGAAGAAAAAACTGCAAAATCCGATCCAATTTATCGTAATCGATTAGTTAACATGTTGGTTAACCGTATTCTGAAACACGGAAAAAAATCATTGGCTTATCAAATTATCTATCGAGCCATGAAAAAGATTCAACAAAAGACAGAAACAAATCCACTATCTGTTTTACGTCAAGCAATACGTGGAGTAACTCCCGATATAGCAGTAAAAGCAAGACGTGTAGGCGGATCGACTCAGCAAGTTCCCATTGAAATAGGATCCACACAAGGAAAAGCACTTGCCATTCGTTGGTTATTAGGGGCATCCCGAAAACGTCCGGGTCGAAATATGGCTTTCAAATTAAGTTCCGAATTAGTGGATGCTGCCAAAGGGAGTGGTGATGCCATACGCAAAAAGGAAGAGACTCATAGAATGGCAGAGGCAAATAGAGCTTTTGCACATTTTCGTTAA

>lcl|NC_015308.1_cds_YP_004327705.1_65 [gene=ycf2] [locus_tag=HebrCp087] [db_xref=GeneID:10351986] [protein=hypothetical chloroplast RF2] [protein_id=YP_004327705.1] [location=91507..98418] [gbkey=CDS]
ATGAAAGGACATCAATTCAAATCCTGGATTTTCGAATTGAGAGAGATATTGAGAGAGATCAAGAATTCTCACTATTTCTTAGATTCATGGACCCAATTCAATTCAGTGGGATCTTTCATTCACATTTTTTTCCATCAAGAACGTTTTATAAAACTCTTGGACTCCCGAATTTGGAGTATCTTACTTTCACGCAATTCACAGGGTTCAACAAGCAATCGATATTTCACGATCAAGGGTGTAGTACTATTTGTAGTAGTGGTCCTTATATATCGTATTAACAATCGAAAGATGGTCGAAAGAAAAAATCTCTATTTGACAGGGCTTCTTCCTATACCTATGAATTCCATTGGACCCAGAAATGATACATTGGAAGAATCCTTTGGGTCTTCCAATATCAATAGGTTGATTGTTTCGCTCCTGTATCTTCCAAAAGGAAAAAAGATCTCTGAGAGCTCTTTCCTGGATCCGAAAGAGAGTACTTGGGTTCTCCCAATAACTAAAAAGTGTATCATGTCTGAATCTAACTGGGGTTCGCGGTGGTGGAGGAACTGGATCGGAAAAAAGAGGGATTCTAGTTGTAAGATATCTAATGAAACCGTTGCTGGAATTGAGATCTCATTCAAAGAAAAAGATATCAAATATCTGGAGTTTCTTTTTGTATATTATATGGATGATCCGATCCGCAAGGACCATGATTGGGAATTGTTTGATCGTCTTTCTCCGAGGAAGGGGCGAAACATAATCAACTTGAATTCGGGACAGCTATTCGAAATCTTAGTGAAAGACTGGATTTGTTATCTCATGTTTGCTTTTCGTGAAAAAATACCAATTGAAGTGGAGGGTTTCTTCAAACAACAAGGAGCTGGGTCAACTATTCAATCAAATGATATTGAGCATGTTTCCCATCTCTTCTCGAGAAAGAAGTGGGCTATTTCTTTGCAAAATTGTGCTCAATTTCATATGTGGCAATTCCGCCAAGATCTCTTCGTTAGTTGGGGGAATAATCCGCACGAATCGGATTTTTTGAGTAACATATCGAGGATTTGGTTAGACAATGTGTGGTTGGTAAACAAGGATCGGTTTTTTAGCAAGGCACGGAATATATCGTCAAATATTCAATATGATTCCACAAGATCTAGTTTCGTTCAAGGAAGGAATTCTAGCCAATTGAAGGGATCTTCTGATCAATCCAGAGATCATTTCGATTCCATTAGTAATGAGGATTCGGAATATCACACATTGATCAATCAAAGAAAGATTCAACAACTAAAAGAAAGATCGATTCTTTGGGATCCTTCCTTTCTTCAAACGGAACGAACAGAGATAGAATCAGACCGATTCCCTAAATGCCTTTCTGGATATTCCTCAATGTCCCGGCTATTCACGGAAGGTGAGAAGGAGATGAATAATCATCTGCTTCCGGAAGAAATCGAAGAATTTCTTGGGAATCCTACAAGATCCATTCGTTCTTTTTTCTCTGACAGATCGTCAGAACTTTATCTGGGTTCGAATCCTACTGAGAGGTCCACTAGAGATCAGAAATTGTTGAAGAAAGAACAAGATGTTTCTTTTGTCCCTTCCAGGCGATCGGAAAATAAAGAAATAGTTAATATATTCAAGATAATCACGTATTTACAAAATACCGTCTCAATTCATCCTATTTCATCAGATCCGGGATGTGATATGGTTCTGAAGGATGAACTGGATATGGACAGTTCCAATAAGATTTCTTTCTTGAACAAAAATCCATTTTTTGATTTATTTCATCTATTCCATGATCGGAACGGGGGGGGATACACGTTACACCACGATTTTGAATCAGAAGAGAGATTTCAAGAAATGGCAGATCTATTCACTCTATCAATAACCGAGCCGGATCTGGTGTATCATAAGGGATTTACCTTTTTTATTGATTCCTACGGATTGGATCAAAAACAATTCTTGAATGAGGTATTCAACTCCAGGGATGAATCGAAAAAGAAATCTTTATTGGTTCTACCTCCTATTTTTTATGAAGAGAATGAATCTTTTTATCGAAGGATCAGAAAAAAATGGGTCCGGATCTCCTGCGGGAATGATTTGGAAGATCCAAAACAAAAAATAGTGGTATTTGCTAGCAACAACATAATGGAGGCAGTCAATCAATATGGATTGATCCTAAATCTGATTCAAATCCAATATAGTACCTATGGGTACATAAGAAATGTATTGACTCAATTCTTTTTAATGAATAGATCCGATCGCAACTTCGAATATGGAATTCAAAGGGATCAAATAGGAAATGATACTCTGAATCATAGAACTATAATGAAATATACGATCAACCAACATTTATCGAATTTGAAACAGAGTCAGAAGAAATGGTTCGATCCTCTTATTTTTCTTTCTCGAACCGAGAGATCCATGAATTGGGATCCTAATGCATATAGATACAAATGGTCTAATGGGAGCAAGAATTTCCAGGAACATTTGGAACATTTCATTTCTGAGCAGAAGAGCCGTTTTCTTTTTCAAGTAGTGTTCGATCGATTACGTATTAATCAATATTCGATTGATTGGTCTGAGGTTATCGACAAAAAAGATTTGTCTAAGTCACTTCGTTTCTTTTTGTCCAAGTTACTTCTTTTTTTGTCCAAGTTTCTTCTCTTTTTGTCTAACTCACTTCCTTTTTTCTTTGTGAGTTTCGGGAATATCCCCATTCATAGGTCCGAAATCCATATCTATGAATTGAAAGGTCCGAATGATCAACTCTGCAATCAGCTGGTAGAACCAATAGGTCTTCAAATCGTTCATTTGAAAAAATTGAAACCCTTCTTATTGTTATTGGATGATCATGATACTTCCCAAAAATCTAAATTTTTGATTAATGGAGGAACAATATCACCATTTTTGTTCAATAAGATAACAAAGTGGATGATTGACTCATTCCATACTAGAAATAATCGCAGGAAATCTTTTGATAACACGGATTCCTATTTCTCAATGATATCCCACGATCAAGACAATTGGCTGAATCCCGTGAAACCATTTCATAGAAGTTCATTGATATCTTCTTTTTATAAAGCAAATCGACTTCGATTCTTGAATAATCTACATCACTTCTGCTTCTATTGTAACAAAAGATTCCCTTTTTATGTGGAAAAGGCCCGTATCAAGAATTATGATTTTACGTATGGACAATTCCTCAATATCTTGTTCATTCGCAACAAAATATTTTCTTTGTGCGGCGGTAAAAAAAAACATGCTTTTTTGGAGAGAGATACTATTTCACCAATCGAGTCACAGGTATCTAACATATTCATACCTAATGATTTTCCACAAAGTGGTAACGAAAGGTATAACTTGTACAAATCTTTCCATTTTCCAATTCGATCCGATCCATTCGTTCGTAGAGCTATTTATTCGATCGCAGACATTTCTGGAACACCTCTAACAGAGGGACAAATAGTCAATTTTGAAAGAACTTATTGTCAACCTCTTTCGGATATGAATCTATCTGATTCAGAAGGGAAGAACTTGCATCAGTATCTCAATTTCAATTCAAACATGGGTTTGATTCACACTCCATGTTCTGAGAAATATTTACCATCCGAAAAGAGGAAAAAACGGAGTCTTTGTCTAAAGAAATGTGTTGAAAAAGGGCAGATGTATAGAACCTTTCAACGAGATAATGCTTTTTCAACTCTCTCAAAATGGAATCTATTCCAAACATATATGCCATGGTTCCTTACTTCGACGGGGTACAAATATCTAAATTTGATATTTTTAGATACCTTTTCGGACCTATTACCGATACTAAGTAGCAGTCAAAAATTTGTATCCATTTTTCATGATATTATGCATGGATCAGATATATCATGGCGAATTCTTCAGAAAAAATTGTGTCTTCCACAATGGAATCTGATAAGTGAGATTTCGAGTAAGTGTTTACATAATCTTCTTCTGTCCGAAGAAATGATTCATCGAAATAATGAGCCACCATTGATATCGACACATCTGAGATCGCCAAATGTTCGGGAGTTCCTCTATTCAATCCTTTTCCTTCTTCTTGTTGCTGGATATCTCGTTCGTACACATCTTTTCTTTGTTTCCCGAGCCTATAGTGAGTTACAGACAGAGTTCGAAAAGGTCAAATCTTTGATGATTCCATCATACATGATTGAGTTGCGAAAACTTCTGGATAGGTATCCTACATCTGAACTGAATTCTTTCTGGTTAAAGAATCTCTTTCTAGTTGCTCTGGAACAATTAGGAGATTTTCTAGAAGAAATGCGGGGTTCTGCTTCTGGCGGCAACATGCTATGGGGTGGTGGTCCCGCTTATGGGGTTAAATCAATACGTTCTAAGAAGAAATTTTTTAATATCAATCTCATCGATCTCATAAGTATCATACCAAATCCCATCAATCGAATCACTTTTTCGAGAAATACGAGACATCTAAGTCATACAAGTAAAGAGATTTATTCATTGATAAGAAAAAGAAAAAACGTGAACGGTGATTGGATTGATGATAAAATAGAATCCTTGGTCGCGAACAGTGATTCGATTGATGATAAAGAAAGAGAATTCTTGGTTCAGTTCTCCACCTTAACGACAGAAAAAAGGATTGATCAAATTCTATTGAGTCTGACTCATAGTGATCATTTATCAAAGAATGACTCTGGTTATCAAATGATTGAAGAGCCGGGAGCAATTTATTTACGATACTTAGTTGACATTCATAAAAAGTATCTAATGAATTATGAGTTCAACACACCCTGTTTAGTAGAAAGACGGATATTCCTTGCTTATTATCAGACAACCACTTATTCACAAACCTCGTGTGGGGTGAATAGTTTTCATTTCCCATCTCATGGAAAACCCTTTTCGCTCCGCTTAGCCCTATCCCCCTCTAGGGGTATTTTAGTGATAGGTTCTATAGGAACTGGACGATCCTATTTGGTCAAATACCTAGCGACAAACTCCTATCTTCCTTTCATTACAGTATTTCTGAACAAGTTCCTGGATAACAAGCCTAAGGGTTTTCTTATTGATGATAGTGACGATATTGATGATAGTGACGATATTGATGATAGTGACGATATTGATGATAGTGACGATATTGATGTGAGTGACGATATTGATGTGAGTGACGATATTGACCGTGACTTTGATACGGAGCTGGAGTTTCTAACTAGGATGAATGTGCTAACTATGGATATGATGCCGGAAATAGACCGATTTTATATCACCCTTCAATTCGAATTAGCAAAAGCAATGTCTCCTTGCATAATATGGATTCCAAACATTCATGATCTGGATGTGAATGAGTCGAATTACTTATCCCTCGGTCTATTAGTGAACTATCTCTCCAGGGATTGTGAAAGATGTTCCACTAGAAATATTCTTGTTATTGCTTCGACTCATATTCCCCAAAAAGTGGATCCCGCTCTAATAGCTCCGAATAAATTAAATACATGCATTAAGATACGAAGGCTTCTTATTCCACAACAACGAAAGCACTTTTTTACTCTTTCATATACTAGGGGATTTCACTTGGAAAATAAAATGTTCCATACTAATGGATTCGGGTCCATAACCATGGGTTCCAATGTACGAGATCTTGTAGCACTTACCAATGAGGCCCTATCGATTAGTATTACACAGAAGAAATCAATTATAGACACTAATATAATTAGATCTGCTCTTCATAGACAAACTTGGGATTTGCGATCCCAGGTAAGATCGGTTCAGGATCATGGGATCCTTTTCTATCAGATAGGAAGGGCTGTTGCACAAAATGTATTTCTAAGTAATTGCCCCATAGATCCTATATCTATCTATATGAAGAAGAAATCATGTAACGAAGGGGATTCTTATTTGTACAAATGGTACTTCGAACTTGGAACGAGCATGAAGAAATTAACGATACTTCTTTATCTTTTGAGTTGTTCTGCCGGATCGGTTGCTCAAGACCTTTGGTCTCTACCCGGACCCGATGAAAAAAATGGGATCACTTATTATGGACTTGTTGAGAATGATTCTGATCTAGTTCATGGCCTATTAGAAGTAGAAGGCGCTCTGGTGGGATCCTCACGGACAGAAAAAGATTGCAGTCAGTTTGATAATGATCGAGTGACATTGCTTCTTCGGCCCGAACCAAGGAGTCCCTTAGATATGATGCAAAATGGATCTTGTTCTATCCTTGATCAGAGATTTCTCTATGAAAAATACGAATCGGAGTTTGAAGAAGGGGAAGGAGAAGAAGTCCTCGACCCGCAACAGATAGAGGAGGATTTATTCAATCACATAGTTTGGGCTCCTAGAATATGGCGCCCTTGGGGTTTTCTATTTGATTGTATCGAAAGGCCCAATGAATTGGGATTTCCCTATTGGGCCAGGTCATTTCGGGGCAAGCGGATCATTTATGATGAAGAGGATGAGCTTCAAGAGAATGATTCGGAGTTCTTGCAGAGTGGAACCATGCAGTACCAGATACGAGATAGATCTTCCAAAGAACAAGGCTTTTTTCGAATAAGCCAATTCATTTGGGACCCTGCGGATCCACTCTTTTTCCTATTCAAAGATCAGCCCTTTGTCTCTGTGTTTTCACATCGAGAATTCTTTGCAGATGAAGAGATGTCAAAGGGGCTTCTTACTTCCCAAACAGATCCTCCTACATCTATATATAAACGCTGGTTTATCAAGAATATGCAAGAAAAGCACTTCGAATTGTTGATTCATCGCCAGAGATGGCTTAGAACCAATAGTTCATTATCTAATGGATTTTTCCGTTCTAATACTCTATCCGAGAGTTATCAGTATTTATCAAATCTGTTCCTATCTAACGGAACGCTATTGGATCAAATGACAAAGGCATTGTTGAGAAAAAGATGGCTTTTCCCGGATGAAATGAAAATTGGATTCATGTAA

>lcl|NC_015308.1_cds_YP_004327695.1_55 [gene=rps11] [locus_tag=HebrCp075] [db_xref=GeneID:10351936] [protein=ribosomal protein S11] [protein_id=YP_004327695.1] [location=complement(83725..84141)] [gbkey=CDS]
ATGGCAAAACCTTTACCAAAAATTGGTTCACGAAGAAACGGACGTATTGGTTCGCGTAAGAATTCACGTAAAATACCAAAAGGAGTTATTCATGTTCAAGCAAGTTTTAACAATACTATTGTGACCGTTACAGATGTACGGGGTCGAGTGATTTCTTGGTCCTCCGCTGGCACTTGTGGATTCAGGGGCACAAGAAGAGGAACGCCATTTGCTGCTCAAACCGCAGCAGGAAATGCTATTCGGACAGTAGTGGATCAAGGTATGCAACGAGCAGAAGTCATGATAAAGGGTCCTGGTCTCGGACGAGATGCGGCATTAAGAGCTATTCGCAGAAGTAGTATACTATTAAGTTTCGTCCGGGATGTAACCCCTATGCCACATAATGGCTGCAGACCCCCTAAAAAAAGGCGCGTGTAA

>lcl|NC_015308.1_cds_YP_004327698.1_58 [gene=rpl14] [locus_tag=HebrCp079] [db_xref=GeneID:10351939] [protein=ribosomal protein L14] [protein_id=YP_004327698.1] [location=complement(85530..85898)] [gbkey=CDS]
ATGATCCAATCTCAGACCCATTTGAATGTAGCGGATAACAGCGGAGCTCGAGAATTGATGTGTATTCGAATCATAGGGACTAGTAATCGCCGATATGCTCATATTGGTGACGTTATTGTTGCTGTGATCAAGGAAGCAGCACCAAATTCACCTCTAGAAAGATCAGAAGTAATCAGAGCTGTAATTGTACGTACTTGTAAAGAACTCAAACGTGATAATGGTATGATAATACGATATGATGACAACGCTGCAGTTGTCATTGATCAAGAAGGAAATCCAAAGGGAACTCGAATTTTTGGTGCAATCGCCCGGGAATTGAGACAGTTAAATTTTACTAAAATAGTTTCATTAGCACCTGAAGTGTTATAA

>lcl|NC_015308.1_cds_YP_004327694.1_54 [gene=rpoA] [locus_tag=HebrCp074] [db_xref=GeneID:10351935] [protein=RNA polymerase alpha subunit] [protein_id=YP_004327694.1] [location=complement(82641..83666)] [gbkey=CDS]
ATGGTTCGAGAGAAAGTAACAATATCCACTCGGACACTGCAGTGGAAATGTGTTGAATCAAGAACCGATAATAAACGTCTTTATTATGGACGCTTTATTCTGTCTCCTCTTATGAAAGGCCAAGCCGACACAATAGGCATTGCGATGCGAAGAGCTTTGCTTGGAGAAATAGAAGGAACATGTATCACACGTGCAAAATCTGAGAAAATACCACACGAATTTTCTACTATAGCAGGTATTCAAGAATCAATACATGAAATTTTAATGAATTTGAAAGAAATTGTATTAAGAAGCAATTTGTATGGAACTTGTAACGCATCTATTTGTGTCGAGGGTCCTGGATATGTAACTGCTCAAGACATCATCTTACCGCCTTTTGTGGAAATCATTGATAATACACAGCATATCGCTAGCCTAACGGAAGCAATTGATTTGTGTATTGGATTACAAATCGAGAGGAATCGCGGCTATCGTATAAACCCAACAAATAACTTTCAAGTTCAAGACGGAAGTTATTCTATAGATGCTGTATTCATGCCTGTTCGAAATGCGAATCATAGTGTTCATTCTTATGGAAATGGGAATGAAAAGCAAGAGATACTTTTTCTCGAAATATGGACAAATGGAAGTTTAACTCCTAAAGAAGCACTTCACGAAGCCTCCCGGAATTTGATTGATTTTTTTATTCCTTTTCTACATGCAGAAGAAGAAAACTTACATTTAGAAAAAAATCAACACAAAGTTACTTTACCCCTTTTTACTTTTCATGATAGATTGACTAAATTAAGAAAAAATAAAAAAGAAATAGCATTGAAATACATTTTTATTGACCAATCAGAATTGACTCCTAAGATCTATAATTGCCTCAAAAGGTCCAATATACATACATTATCGGATCTTTTGAAAAAGAGTCAAGAAGATCTTATGAAAATTGAACATTTTCGCATAGACGATGTAAAACATATATTGGGTATTCTAGAAATAGAAAAACATTTCGCAATTGATTTACCAAAGAATAAAATATAA

>lcl|NC_015308.1_cds_YP_004327669.1_29 [gene=atpB] [locus_tag=HebrCp048] [db_xref=GeneID:10351911] [protein=ATP synthase CF1 beta subunit] [protein_id=YP_004327669.1] [location=complement(56040..57536)] [gbkey=CDS]
ATGAGAATAAATCCTACTACTTCTGGTCCGGGAGTTTCCGCGCTTGAAAAAAAGAACCTGGGGCGTATCGCTCAAATTATTGGGCCAGTGCTAGATGTAGCTTTTCCCCCGGGCAAGATGCCTAATATTTACAACGCTCTGGTAGTTAAGGGTCGAGATACTGCCGGTCAAGAAATTAATGTGACTTGTGAAGTACAACAATTATTAGGAAATAATCGAGTTCGCGCTGTAGCTATGAGTGCTACAGATGGTCTAACGAGAGGAATGGAAGTGATTGACACAGGAGCCCCTCTAAGTGTTCCAGTCGGTGGGGCAACTCTAGGACGAATTTTCAACGTGCTTGGAGAACCTGTTGACGATTTAGGTCCTGTAGATACTCGCGCAACATCCCCTATTCATAGATCTGCGCCTGCCTTTATACAGTTAGATACAAAATTATCTATTTTTGAAACAGGAATAAAAGTAGTAGATCTTTTAGCCCCTTATCGCCGTGGAGGAAAAATCGGACTATTCGGGGGAGCTGGAGTGGGTAAAACAGTACTTATTATGGAATTAATCAATAACATTGCGAAAGCTCATGGGGGTGTATCCGTATTTGGCGGAGTAGGCGAACGTACTCGTGAAGGAAATGATCTTTACATGGAAATGAAAGAATCTGGAGTAATTAATGAAGAAAATATTGCAGAATCAAAAGTGGCTCTAGTCTATGGTCAGATGAACGAACCGCCGGGAGCTCGTATGAGAGTTGGTTTGACTGCCCTAACTATGGCGGAATATTTTCGAGATGTTAATGAACAAGACGTACTTCTATTTATCGACAATATCTTCCGTTTCGTCCAAGCCGGATCCGAAGTATCCGCCTTATTGGGTAGAATGCCTTCCGCTGTGGGTTATCAACCTACCCTTAGTACCGAAATGGGCTCTTTACAAGAAAGAATTACTTCTACCAAAGAAGGGTCCATAACTTCTATTCAAGCAGTTTATGTACCTGCGGACGATTTGACTGACCCTGCTCCTGCCACGACATTTGCACATTTAGATGCTACTACTGTACTATCAAGAGGATTAGCTGCTAAAGGTATCTATCCAGCAGTAGATCCTTTAGATTCAACGTCAACTATGCTCCAACCTCAGATCGTTGGTGAGGAACATTATGAAACTGCGCAAAGAGTTAAGCAAACTTTACAACGTTACAAAGAACTTCAGGACATTATAGCTATCCTTGGGTTGGACGAATTATCCGAAGAGGATCGCTTAACTGTAGCAAGAGCACGAAAAATTGAACGTTTCTTATCACAACCCTTTTTCGTAGCAGAAGTATTTACCGGTTCTCGGGGGAAATATGTCGGTCTAGCAGAAACAATTAGAGGGTTTAAATTGATCCTTTCGGGAGAATTAGATAGTCTCCCTGAGCAGGCCTTTTATTTGGTAGGTAATATTGATGAAGCTACTGCGAAGGCTACGAACTTAGAAATGGAGAACAACTTGAAGAAATGA

>lcl|NC_015308.1_cds_YP_004327724.1_84 [gene=rpl2] [locus_tag=HebrCp135] [db_xref=GeneID:10351946] [protein=ribosomal protein L2] [protein_id=YP_004327724.1] [location=join(159522..159920,160594..161028)] [gbkey=CDS]
ATGGCGATACATTTATACAAAACTTCTACCCCGAGCACACGCAATGGAGCCGTAGACAGTCAAGCGAAATCCAATACACGAAATACACGAAAGAATTTGATCTATGGACAGCATCGTTGTGGTAAAGGCCGTAATGCCAGAGGAATCATTACCGCAAGACATAGAGGGGGAGGTCATAAGCGTCTATACCGTAAAATCGATTTTCGACGGAATGAAAAAGACATATATGGTAGAATCGTAACCATAGAATACGACCCTAATCGAAATGCATACATTTGTCTCATACACTATGGGGATGGTGAGAAGAGATATATTTTACATCCCAGAGGGGCTATAATTGGAGATACCGTTATTTCTGGTACAGAAGTTCCTATAAAAATGGGAAATGCCCTACCTTTGACCGATATGCCCTTAGGCACGGCCATACATAACATAGAAATCACACTTGGAAAGGGTGGACAATTAGCTAGAGCTGCAGGTGCTGTAGCGAAACTGATTGCAAAAGAGGGGAAATCAGCCACATTAAAATTACCTTCGGGGGAGGTTCGTTTAATATCCAAAAACTGCTCAGCAACAGTCGGACAAGTAGGGAATACTGGGGTGAACCAGAAAAATTTGGGTAGAGCCGGATCTAAATGTTGGCTAGGTAAGCGTCCTGTAGTAAGAGGAGTAGTTATGAACCCTGTAGACCATCCCCATGGGGGTGGTGAAGGGAGGGCCCCAATTGGTAGAAAAAAACCCGCAACCCCTTGGGGTTATCCTGCACTTGGAAGAAGAAGTAGAAAAAGGAATAAATATAGTGATAATTTGATTCTTCGTCGCCGTAGTAAATAG

>lcl|NC_015308.1_cds_YP_004327721.1_81 [gene=ndhB] [locus_tag=HebrCp129] [db_xref=GeneID:10351948] [protein=NADH dehydrogenase subunit 2] [protein_id=YP_004327721.1] [location=join(148050..148826,149509..150264)] [gbkey=CDS]
ATGATCTGGCATGTACAGAATGAAAACTTCATTCTCGATTCTACGAGAATTTTTATGAAAGCCTTTCATTTGCTTCTCTTCGATGGAAGTTTTATTTTCCCAGAATGTATCCTAATTTTTGGCCTAATTCTTCTTCTGATGATCGATTCAACCTCTGATCAAAAAGATATACCTTGGTTATATTTCATCTCTTCAACAAGTTTAGTAATGAGTATAACGGCCCTATTGTTCCGATGGAGAGAAGAACCTATGATTAGCTTTTCGGGAAATTTCCAAACGAACAATTTCAACGAAATCTTTCAATTTCTTATTTTACTATGTTCAACTCTATGTATTCCTCTATCCGTAGAGTACATTGAATGTACAGAAATGGCTATAACAGAGTTTCTCTTATTCGTATTAACAGCTACTCTAGGAGGAATGTTTTTATGCGGTGCTAACGATTTAATAACTATCTTTGTAGCTCCAGAATGTTTCAGTTTATGCTCCTACCTATTATCTGGATATACCAAGAAAGATGTACGGTCTAATGAGGCTACTACGAAATATTTACTCATGGGTGGGGCAAGCTCTTCTATTCTGGTTCATGCTTTCTCTTGGCTATATGGTTCGTCCGGGGGAGAGATCGAGCTTCAAGAAATAGTGAATGGCCTTATCAATACACAAATGTATAACTCCCCAGGAATTTCAATTGCGCTTATATTCATCACTGTAGGAATTGGGTTCAAGCTTTCCCTAGCCCCTTCTCATCAATGGACTCCTGACGTATACGAAGGATCTCCCACTCCAGTCGTTGCTTTTCTTTCTGTTACTTCGAAAGTAGCTGCTTCAGCTTCAGCCACTCGAATTTTCGATATTCCTTTTTATTTCTCATCAAACGAATGGCATCTTCTTCTGGAAATCCTAGCTATTCTGAGCATGATAGTGGGGAATCTCATTGCTATTACTCAAACAAGCATGAAACGTATGCTTGCATATTCGTCCATAGGTCAAATCGGATATGTAATTATTGGAATAATTGTTGGAGACTCAAATGGTGGATATGCAAGCATGATAACTTATATGCTCTTCTATATCTCCATGAATCTAGGAACTTTTGCTTGTATTGTATTATTTGGTCTACGTACCGGAACTGATAACATTCGAGATTATGCAGGATTATACACGAAAGATCCTTTTTTGGCTCTCTCTTTAGCCCTATGTCTCTTATCCCTAGGAGGTCTTCCTCCACTAGCAGGTTTTTTCGGAAAACTCCATTTATTCTGGTGTGGATGGCAGGCAGGCCTATATTTCTTGGTTTTAATAGGACTCCTTACGAGCGTTGTTTCTATCTACTATTATCTAAAAATAATCAAGTTATTAATGACTGGACGAAACCAAGAAATAACCCCTCACGTGCGAAATTATAGAAGATCCCCTTTAAGATCAAACAATTCCATCGAATTGAGTATGATTGTATGTGTGATAGCATCTACTATACCAGGAATATCAATGAACCCGATTGTTGAAATTGCTCAAGATACCCTTTTTTAG

>lcl|NC_015308.1_cds_YP_004327687.1_47 [gene=clpP] [locus_tag=HebrCp067] [db_xref=GeneID:10351949] [protein=ATP-dependent Clp protease proteolytic subunit] [protein_id=YP_004327687.1] [location=complement(join(74495..74722,75364..75654,76490..76558))] [gbkey=CDS]
ATGCCTATTGGTGTTCCAAAAGTCCCTTTTCGAAATCCTGGGGAAGACGATTCAATTTGGATTGACGTAAACCGACTTTATCGAGAAAGATTACTTTTTTTAGGTCAAGATGTTGATAGCGAGATCTCGAATCAACTTATTGGTCTTATGGTATATCTCAGTATAGAAAGCGAGACAAAAGATTTGTATTTGTTTATAAACTCTCCCGGCGGATGGGTAATACCCGGAATAGCTATTTATGATACTATGCAATTTGTGCGACCCGATGTACAAACAGTATGCATGGGATTAGCTGCTTCAATGGGATCTTTTATTCTGGTCGGAGGAAAAATTACCAAACGTTTAGCATTCCCTCATGCTAGGGTAATGATTCATCAACCTATTGCTGGTTTTTATGAGGCACAAATAGGAGAATTTGTCCTGGAAGCGGAAGAACTACTGAAACTGCGCGAAATCCTCACAAGGATTTATGCACAAAGAACGGGCAAACCCTTATGGGTTGTATCCGAAGACATGGAAAGAGATGTTTTTATGTCAGCAACCGAAGCCCAAGCTCATGGAATTGTTGATCTTGTAGCAGTTGCATAA

>lcl|NC_015308.1_cds_YP_004327655.1_15 [gene=atpF] [locus_tag=HebrCp022] [db_xref=GeneID:10351897] [protein=ATP synthase CF0 B subunit] [protein_id=YP_004327655.1] [location=29548..30102] [gbkey=CDS]
ATGAAAAATGTAACCGATTCTTTCGTTTCCTTGGGTCACTGGCCATCCGCCGGGAGTTTCGGGTTTAATACCGATATTTTAGCAACAAATCTAATAAATCTAAGTGTAGTCCTTGGTGTATTGATTTTTTTTGGAAAGGGAGTGTTAAGTGATTTATTAGATAATCGAAAACAAAGGATTTTGGATACTATTCGAAATTCAGAAAAACTACGCGAGGGGGCTATTGAACAGCTGGAAAAAGCCCGGGCCCGCTTACGGAAAGTGGAAATAGAAGCAGATCAGTTTCGAACGAATGGATATTCTGAGATAGAACGAGAAAAATGGAATTTGATTAATTCAACTTATAAGACTTTGGAACAATTAGAAAATTACAAAAATGAAACCATTCATTTTGAACAACAACGAACGATTAATCAAGTCCGACAACGGGTTTTCCAACAAGCCTTACAAGGAGCTCTAGGAACTCTGAATAGTTGTTTGACCAACGAGTTGCATTTACGTACCATCAATGCTAATCTTGGCATGTTTGGGGCGATAAAAGAAATAACTGATTAG

>lcl|NC_015308.1_cds_YP_004327643.1_3 [gene=matK] [locus_tag=HebrCp004] [db_xref=GeneID:10351964] [protein=maturase K] [protein_id=YP_004327643.1] [location=complement(2397..3905)] [gbkey=CDS]
ATGGAGGAAAGATATTTAGAATTAGATAGATCTCGAAAAAACGACTTCCTATACCCATTTATCTTTCGGGAGTATATTTATACATTCGCTCATGATCATAGTTTAAATAGATCTATTTTGTTGGAAAATGTAGGTTATGACAATAAATCTAGTTTTTTAATTGTAAAACGTTTAATTACTCGAATGTATCAACAGAATCATTTGATTATTTCTGCTAATGATTCTAACCAAAATCCATTTTTTAGATACAACAAGAATTTGTATTATCAAATGATATCAGAGGGCTTTGCAGTTATTGTGGAAATTCCATTTTCCCTACGATTAGTATCTTCTTTAGAAAGGTCAGAGATAGTAAAATCTCATAAATTACGATCAATTCATTCAATATTTCCTTTATTAGAGGACAAATTTCCACATTTAAATTATGTGTCAGATATATTAATACCTTACCCCATCCATCTAGAAAAATTGGTTCAAACCCTTCGCTATTGGGTGAAAGATCCCTCTTCTTTGCATTTATTACGACTCTTTCTTCATGAGTATTGGAATTTGAACAGTCTTATTATTCCAAAGAAATCTATTATTATTTTTATAAAAAGGAATCCAAGATTTTTCTTGTTCCTATATAATTCTCATGTATATGAATACGAATCCATCTTCTTTTTTCTCCGTAACCAATCCTTTCATTTACGATCAATATTTTTGCGAGTCCTTCTTGAACGAATTTTTTTCTATGGAAAAATAGAACATTTTGCGGAAGTCTTTGCTAATGATTTTCAGGCCACCCTGTGGTTGTTCAAGGATCCTTTCATGCATTATGTTAGATATCAAGGAAAATCAATTTTGGCTTCAAAAAATAGGCCTTTTCTGATGAAAAAATGGAAATATTACCTTGTCAACTTATGTCAATATCATTTTTATGTCTGGTTTCAACCAGAAAAGATCTATATAAATTCATTATCCAAGCATTCTCTCAACTTTTTGGGCTATCTTTCAAGTGTACAATTAAATCCTTCAGTGGTACGGAGTCAAATGTTAGAATATTCATTTATAATAGATAATACTATAAAGAAACTCGATACAATAGTTCCAATTATTCCTTTAATTGGATCATTGGCAAAAACGAAATTTTGTAACGCAGTAGGACATCCCATTAGTAAACCGATTCGGGCGGATTCGTCGGATTCTGATATTCTCGACCGATTTGTGCGTATATGCAGAAATCTTTCTCATTATTATAGCGGATCCTCAAAAAAAAAGAGTTTGTATCGAATAAAATATATACTTCGACTTTCTTGTGTTAAAACTTTGGCCCGTAAACACAAAAGTACTGTACGCGCTTTTTTGAAAAGATTAGGTTCGGAATTATTAGAAGAGTTTTTTACGGAGGAAGAACAGATTCTTTCTTTGATCTTTCCAAAAGTTTCTTCTAGTTCGCGCAGGTTATATAGAAGACGTGTTTGGTATTTGGATATTATTTCTATCAATGATTTGGCCAATCATGAATAA

>lcl|NC_015308.1_cds_YP_004327685.1_45 [gene=rpl20] [locus_tag=HebrCp066] [db_xref=GeneID:10351927] [protein=ribosomal protein L20] [protein_id=YP_004327685.1] [location=complement(73009..73362)] [gbkey=CDS]
ATGACCAGAATTAGACGAGGATATATAGCTCGGAGGCGTAGAACAAAAATTCGTTTATTCGCATCAAGCTTTCGCGGGGCCCATTCAAGACTTACTCGAACTATTATTCAACAAAAAATAAGAGCTTTGGTTTCGGCCCATCGGGATAGAGATAGGCAAAAAAGAAATTTTCGTCGTTTGTGGGTCACTCGGATAAATGCAGTAATTCGCGAGAGTATGGTATCCTATAGTTATAGTAGATTAATAAACAATCTGTACAAGAGACAGTTACTTCTTAATCGTAAAATACTTGCACAAATAGCTATATTAAATAGGAATTGTCTTTATATGATTTCCAATGACATTCTAAAATAA

>lcl|NC_015308.1_cds_YP_004327714.1_74 [gene=ndhG] [locus_tag=HebrCp111] [db_xref=GeneID:10351955] [protein=NADH dehydrogenase subunit 6] [protein_id=YP_004327714.1] [location=complement(124365..124895)] [gbkey=CDS]
ATGGATTTGCCTGGACTAATTCATGATTTTCTTTTAGTCTTTCTAGGATTAGGTCTTATATTAGGAGGTCTAGGAGTGGTATTACTTACCAACCCAATTTATTCTGCCTTTTCGTTGGGATTGGTTCTTGTTTGTATATCTTTATTCTATATTTTATCAAACTCTCATTTTGTAGCTGCCGCACAGCTCCTTATTTATGTGGGAGCTATAAATGTTTTAATTATATTTGCCGTGATGTTCATGAATGGTTCAGAATATTACAAAGATTTTAATCTTTGGACTGTTGGAAGCGGGGTTACTTCTTTAGTTTGTACAAGTATTTTTGTTTCACTAATTACTATTCTTCCAGATACGTCATGGTATGGAATTATTTGGACTACAAGAACAAATCAGATTATAGAACAAGATTTGATAAGTAATGGTCAACAAATTGGAATTCATTTATCAACAGATTTTTTTCTTCCATTTGAATTCATTTCAATAATTCTTTTAGTTGCTTTGATAGGTGCGATTGCTGTGGCTCGTCAGTAA

>lcl|NC_015308.1_cds_YP_004327650.1_10 [gene=rpoC1] [locus_tag=HebrCp017] [db_xref=GeneID:10352021] [protein=RNA polymerase beta' subunit] [protein_id=YP_004327650.1] [location=join(18358..18789,19575..21185)] [gbkey=CDS]
ATGATTGATCGGTATAAACATCAACAACTCCGAATTGGATCAGTTTCGCCTCAACAAATAAGTGCTTGGGCCAATAAAATCCTACCTAACGGAGAGATTGTTGGAGAGGTGACAAAACCCTATACTTTTCATTACAAAACCAATAAACCTGAAAAAGATGGATTATTTTGTGAAAGAATTTTTGGGCCTATAAAAAGTGGAATTTGTGCTTGTGGAAATTATCGAGTAATCAGGAATGAAAAAGAAGACCAAAAATTTTGTGAACAATGCGGAGTCGAATTTATGGATTCTCGGATACGAAGATATCAAATGGGCTACATCAAACTAGCATGCCCAGTAACTCATGTGTGGTATTTGAAACGTCTTCCTAGTTATATCGCAAATCTTTTAGATAAACCTCTTAAAGAATTAGAAGGCCTAGTATACTGCGATTTTTCTTTTGCTAGGCCCATAGCTAAAAAACCTACTTTTTTACGATTACGAGGTTCATTCGAATATGAAATCCAATCCTGGAAATACAGTATTCCACTTTTTTTTACTACCCAAGGCTTCGATACATTTCGAAATCGAGAAATTTCTACAGGAGCTGGTGCTATCCGAGAACAATTAGCCGATCTGGATTTGCGAATTATTATAGATTATTCATTGGTAGAATGGAAAGAATTAGGGGAAGAAGGGCCTACCGGGAATGAATGGGAAGATCGAAAAGTTGGAAGAAGAAAGGATTTTTTGGTTAGACGTGTGGAATTAGCTAAGCATTTTATTCGAACAAATATCGAACCAGAATGGATGGTTTTATGTCTATTACCAGTTCTTCCTCCCGAGTTGAGACCGATCATTCAGATAGATGGGGGTAAACTAATGAGTTCAGATATTAATGAACTCTATAGAAGAGTTATCTATCGGAACAATACTCTTATTGATCTATTAACAACAAGTAGATCTACCCCAGGGGAATTAGTAATGTGTCAGGAGAAATTGGTACAAGAAGCCGTGGATACACTTCTTGATAATGGAATCCGCGGACAACCAATGAGGGACGGTCATAATAAGGTTTACAAGTCGTTTTCGGATGTAATTGAAGGCAAAGAAGGAAGATTTCGTGAGACTATGCTTGGCAAACGGGTTGATTATTCGGGGCGTTCTGTCATTGTCGTAGGCCCCTCACTTTCATTACATCGATGTGGATTGCCTCGCGAAATAGCAATAGAGCTTTTCCAGATATTTGTAATTCGTGGTCTAATTAGACAACATCTTGCTTCGAACATAGGAGTTGCTAAGAGTAAAATTCGGGAAAAAGAGCCAATTGTATGGGAAATACTTCACGAAGTTATGCAGGGGCATCCAGTATTACTGAATAGAGCGCCGACTCTGCATAGATTAGGCATACAGGCATTCCAACCCATTTTAGTGGAAGGCCGCGCTATTTGTTTACATCCATTAGTTTGTAAGGGATTCAATGCAGACTTTGATGGGGATCAAATGGCTGTTCATGTACCTTTATCGTTGGAGGCTCAAGCAGAGGCTCGTTTACTTATGTTTTCTCATATGAATCTCTTGTCTCCAGCTATTGGGGATCCCATTTCCGTACCAACTCAAGATATGCTTATTGGGCTCTATGTATTAACAAGCGGGAATTGTCGAGGTATTTGTGCAAATAGGTATAATCCATGTAATCGCAGAAATTATCAAAATAAAAGAATTGACGGTAATAACGATAAATATACGAAAGAACCCTTTTTTTCTAATTCCTATGATGCACTTGGCGCTTATCGGCAGAAAAGAATCCATTTAGATAGTCCTTTGTGGCTCCGGTGGCAGCTAGATCAACGCGCTATTACTTCAAGAGAAGCTCCCATCGAAGTTCACTATGAATCTTTGGGTACCTATCATGAGATTTATGAACACTATCTAATAGTAAAAAATATAAAAAAAGAAATTCTTTGTATATACATTCGAACTACTGTTGGTCATATTTCTCTTTATCGAGAAATCGAAGAAGCTATACAAGGGTTTTGCCAAGCCTGCTCAGATAGTATCTAA

>lcl|NC_015308.1_cds_YP_004327675.1_35 [gene=petA] [locus_tag=HebrCp054] [db_xref=GeneID:10351917] [protein=cytochrome f] [protein_id=YP_004327675.1] [location=65684..66646] [gbkey=CDS]
ATGCAAACCAGAAAGACCTTTTCTTGGATAAAGGAAGAGATTACTCGTTCCATTTCCGTATCGCTCATGATATATATAATAACTTGGGCATCCATTTCAAATGCATATCCCATTTTTGCACAGCAGGGTTATGAAAATCCACGGGAAGCAACTGGTCGTATTGTATGTGCCAATTGTCATTTAGCTAATAAACCCGTGGATATTGAGGTTCCACAAGCGGTACTTCCAGATACTGTATTTGAAGCAGTTGTTCGAATTCCTTATAATATGCAACTGAAACAAGTTCTTGCTAATGGTAAAAAGGGGGCTTTGAATGTGGGGGCTGTTCTTATTTTACCTGAGGGGTTTGAATTAGCCCCTCCCGATCGTATTTCGCCAGAGATGAAAGAAAAGATGGGAAATCTGTCTTTTCAGAGTTATCGCCCCACTAAAAAAAATATTCTTGTGATAGGTCCTGTTCCTGGTCAGAAATATAGTGAAATTACCTTTCCTATTCTTTCTCCGGACCCCGCCGCTAAGAAAGATGTTCACTTTTTAAAATATCCCATATATGTAGGCGGAAACAGGGGAAGGGGTCAGATTTATCCCGACGGGAGCAAGAGCAACAATACGGTTTATAATGCTATAGCAGCAGGTATAGTAAGCAAAATCATACGAAAAGAAAAAGGGGGGTACGAAATAACCATAACGAATGCGTCAGAGGGACGTCAAGTGATTGATATTATACCTCCAGGACCAGAACTTCTTGTTTCAGAAGGCGAATCCATCAAACTTGATCAACCATTAACGAGTAATCCTAATGTGGGTGGATTTGGTCAGGGGGATGCAGAAATAGTACTTCAAGACCCATTACGTGTCCAAGGCCTTTTGTTCTTCTTGGCATCCGTTATTTTGGCACAAATCTTTTTGGTTCTTAAAAAGAAACAGTTTGAGAAGGTTCAATTGTCCGAAATGAATTTCTAG

>lcl|NC_015308.1_cds_YP_004327641.1_1 [gene=rps12] [locus_tag=HebrCp092] [db_xref=GeneID:10352012] [protein=ribosomal protein S12] [exception=trans-splicing] [protein_id=YP_004327641.1] [location=complement(join(103189..103214,103751..103982,74171..74284))] [gbkey=CDS]
ATGCCAACTATTAAACAACTTATTAGAAACACAAGACAGCCAATCAGAAATGTCACCAAATCCCCCGCTCTTGGGGGATGTCCTCAGCGCCGAGGAACATGTACTAGGGTGTATACTATCACCCCCAAAAAACCAAACTCTGCCTTACGTAAAGTTGCCAGAGTACGATTAACCTCTGGTTTTGAAATCACTGCTTATATACCTGGTATTGGCCATAATTTACAAGAACATTCTGTAGTCTTAGTAAGAGGGGGAAGGGTTAAGGATTTACCCGGTGTGAGATATCACATTGTTCGAGGAACCCTAGATGCTGTCGGAGTAAAGGATCGTCAACAAGGGCGTTCTAAATATGGGGTCAAAAAGCCAAAATAA

>lcl|NC_015308.1_cds_YP_004327700.1_60 [gene=rps3] [locus_tag=HebrCp081] [db_xref=GeneID:10351941] [protein=ribosomal protein S3] [protein_id=YP_004327700.1] [location=complement(87811..88467)] [gbkey=CDS]
ATGGGACAAAAAATAAATCCACTTGGTTTCAGACTTGGTACAACCCAAAGTCATCATTCTCTTTGGTTTGCACAACCAAAAAATTACTCTGAGGGTCTACAAGAAGATCAAAAAATAAGAAACTGTATCAAGAATTATGTAAAAAAAAATGCGAAAATATCTTCTGGTATTGAAGGAATTGCACGTATAGAGATTCAAAAACGAATTGATGTGATTCAGGTCATAATATATATGGGATTCCAAAAATTATTAATAGAAAGTAGACCTAAACGAATCGAAGAATTGCAGATGAATGTACAAAAAGAACTTAATTGTGTGAATCGAAAACTCAATATTACTATTACAAGAATTACAAACCCCTATGGGCACCCTACTATTCTTGCAGAATTTATAGCCGGACAATTAAAGAATAGAGTTTCATTTCGCAAAGCAATGAAAAAAGCTATTGAATTAACTGAACAGACAGATACAAAAGGAATTCAAGTGCAAATTGCGGGACGTCTTGACGGAAAAGAAATTGCACGCGTCGAATGGATTAGAGAGGGTAGAGTTCCTCTACAAACCATTGGAGCTAAAATTGATTATTGTTCGTATACAGTTAGAACTATTTATGGGGTATTGGGCATAAAAATTTGGATATTTCCAGACAAGAAATAA

>lcl|NC_015308.1_cds_YP_004327663.1_23 [gene=ycf3] [locus_tag=HebrCp036] [db_xref=GeneID:10351905] [protein=photosystem I assembly protein ycf3] [protein_id=YP_004327663.1] [location=complement(join(46079..46231,46900..47127,47854..47979))] [gbkey=CDS]
ATGCCTAGATCTCGGATAACTGGAAATTTTATTGATAAGACTTTTTCAGTTGTAGCCAATATCTTATTACGAATAATTCCGACAACTTCGGGAGAAAAAGAGGCATTTACTTATTACAGAGATGGTATGTCTGCTCAATCCGAAGGAAATTATGCAGAAGCTTTACAGAATTATTATGAAGCTATGCGGCTAGAAATTGATCCCTATGATCGAAGTTATATACTCTATAATATAGGCCTTATTCACACAAGTAATGGAGAACACACAAAAGCTTTGGAATATTATTTTCGGTCACTAGAACGAAACCCCTTCTTACCACAAGCTTTAAATAATATGGCCGTGATCTGTCATTACCGAGGAGAACAGGCCATTCGGCAGGGAGATTCTGAAATTGCGGAGGCTTGGTTCGATCAAGCCGCGGAGTATTGGAAACAAGCTATAGCGCTTACTCCCGGAAATTATATTGAAGCGCAGAATTGGTTGAAAATCACAAGGCGTTTCGAATAA

>lcl|NC_015308.1_cds_YP_004327661.1_21 [gene=psaB] [locus_tag=HebrCp034] [db_xref=GeneID:10351903] [protein=photosystem I P700 chlorophyll a apoprotein A2] [protein_id=YP_004327661.1] [location=complement(40842..43046)] [gbkey=CDS]
ATGGCATTAAGATTTCCAAGGTTTAGCCAAGGCTTAGCTCAGGACCCCACTACTCGTCGTATTTGGTTTGGTATTGCTACCGCGCATGACTTCGAGAGTCATGATGATATTACGGAGGAACGTCTTTATCAGAATATTTTTGCTTCTCACTTCGGGCAATTAGCAATAATTTTTCTGTGGACTTCCGGAAATCTCTTTCATGTAGCTTGGCAAGGAAATTTTGAAGCATGGGTACAGGACCCTTTACATGTAAGACCTATTGCTCATGCAATTTGGGATCCTCATTTTGGTCAACCGGCCGTGGAAGCTTTTACTCGAGGGGGTGCTCCTGGCCCAGTGAATATCGCTTATTCTGGTGTTTATCAATGGTGGTATACAATCGGTTTACGTACTAATGAAGATCTTTATATTGGAGCTCTTTTTCTATTATTTCTTTCTGCCCTAGCCTTACTAGGGGGTTGGTTACACCTACAACCAAAATGGAAACCGAGCGTTTCGTGGTTCAAAAATGCCGAATCTCGTCTCAATCATCATTTGTCAGGACTATTCGGAGTAAGCTCTTTGGCTTGGACAGGACATTTAGTCCATGTCGCTATTCCCGGCTCCCGGGGGGAATACGTTCGATGGAATAATTTCTTAGATGTATTACCACATCCCCAAGGGTTAGGCCCGCTTTTTACAGGTCAGTGGAATCTTTATGCTCAAAATCCCGATTCAGGTAGTCATTTATTTGGTACCTCCCAAGGAGCGGGAACTGCCATTCTAACCCTTCTCGGGGGGTTCCATCCACAAACACAAAGTTTATGGCTGACCGATATTGCACACCATCATTTAGCTATTGCAGTTATTTTTCTCGTTGCCGGTCATATGTATAGAACTAACTTCGGGATTGGGCACAGTATAAAAGATCTTTTAGAAGCACATATTCCTCCGGGGGGGCGATTGGGGCGTGGACATAAGGGTCTTTATGACACAATCAACAATTCGCTTCATTTTCAATTAGGCCTTGCTCTAGCTTCTTTAGGGGTTATTACTTCCTTAGTGGCTCAACACATGTACTCATTACCTGCTTATGCGTTCATAGCGCAAGACTTTACTACTCAAGCTGCGTTATATACTCATCACCAATACATCGCAGGATTCATCATGACAGGAGCTTTTGCTCATGGAGCTATATTTTTTATTAGAGATTACAATCCGGAACAGAATGAGAATAATGTATTGGCAAGAATGTTAGACCATAAAGAAGCTATCATATCCCATTTAAGTTGGGCCAGTCTCTTTCTGGGATTCCATACTTTGGGACTTTATGTTCATAATGATGTCATGCTTGCTTTTGGTACTCCGGAGAAACAAATCTTAATCGAACCCATATTTGCCCAATGGATACAATCTGCTCACGGTAAAACTTCATATGGGTTCGATGTACTTTTATCTTCAACGAATAGTCCGGCCTTCAATGCGGGTCGAAGCATATGGTTGCCCGGCTGGTTAAATGCTATTAATGAAAATAGTAATTCATTATTCTTAACAATAGGGCCTGGAGACTTCTTGGTTCATCATGCTATTGCTCTAGGTTTACATACAACCACATTGATCTTAGTAAAAGGTGCTTTAGATGCACGCGGTTCGAAGTTAATGCCAGATAAAAAGGATTTTGGTTATAGTTTTCCTTGTGATGGTCCGGGACGCGGCGGTACTTGTGATATTTCGGCTTGGGACGCATTTTATTTGGCGGTTTTCTGGATGTTAAATACCATTGGATGGGTTACTTTTTATTGGCATTGGAAGCACATCACATTATGGCAGGGTAATGTTTCACAGTTTAATGAATCTTCCACTTATTTGATGGGATGGTTAAGAGATTATCTATGGTTAAACTCTTCACAACTTATCAATGGATATAACCCTTTTGGTATGAATAGCTTATCGGTCTGGGCGTGGATGTTCTTATTTGGACATCTTGTTTGGGCTACTGGATTTATGTTTTTAATTTCTTGGCGTGGATATTGGCAAGAATTGATTGAAACTTTAGCATGGGCTCATGAGCGTACACCTTTGGCTAATTTGATTCGATGGAGAGATAAACCAGTAGCTCTTTCCATTGTGCAAGCAAGATTGGTTGGATTAGCTCACTTTTCCGTAGGTTATATCTTCACTTATGCGGCTTTCTTGATTGCCTCTACATCAGGTAAATTTGGTTAA

>lcl|NC_015308.1_cds_YP_004327720.1_80 [gene=rps7] [locus_tag=HebrCp128] [db_xref=GeneID:10352016] [protein=ribosomal protein S7] [protein_id=YP_004327720.1] [location=147271..147738] [gbkey=CDS]
ATGTCACGTCGAGGTACTGCAGAAGAAAAAACTGCAAAATCCGATCCAATTTATCGTAATCGATTAGTTAACATGTTGGTTAACCGTATTCTGAAACACGGAAAAAAATCATTGGCTTATCAAATTATCTATCGAGCCATGAAAAAGATTCAACAAAAGACAGAAACAAATCCACTATCTGTTTTACGTCAAGCAATACGTGGAGTAACTCCCGATATAGCAGTAAAAGCAAGACGTGTAGGCGGATCGACTCAGCAAGTTCCCATTGAAATAGGATCCACACAAGGAAAAGCACTTGCCATTCGTTGGTTATTAGGGGCATCCCGAAAACGTCCGGGTCGAAATATGGCTTTCAAATTAAGTTCCGAATTAGTGGATGCTGCCAAAGGGAGTGGTGATGCCATACGCAAAAAGGAAGAGACTCATAGAATGGCAGAGGCAAATAGAGCTTTTGCACATTTTCGTTAA

>lcl|NC_015308.1_cds_YP_004327688.1_48 [gene=psbB] [locus_tag=HebrCp068] [db_xref=GeneID:10351929] [protein=photosystem II 47 kDa protein] [protein_id=YP_004327688.1] [location=77025..78551] [gbkey=CDS]
ATGGGTTTGCCTTGGTATCGTGTTCATACCGTCGTATTGAATGATCCCGGTCGTTTGCTGTCTGTCCATATAATGCATACAGCTTTGGTTGCTGGTTGGGCCGGTTCGATGGCTCTATATGAATTAGCAGTTTTTGATCCCTCTGACCCCGTTCTCGATCCAATGTGGAGACAAGGTATGTTCGTTATACCCTTTATGACTCGTTTAGGAATAACCAATTCATGGGGTGGTTGGAGTATCACAGGAGGAACTATAACGAATCCGGGTATTTGGAGTTATGAAGGCGTGGCTGGGGCGCATATTGTGTTTTCTGGCTTGTGCTTCTTGGCAGCTATTTGGCATTGGGTGTATTGGGATCTCGAAATATTTTGCGATGAACGTACAGGAAAACCTTCTTTGGATTTGCCCAAGATCTTTGGAATTCATTTATTTCTCTCCGGGGTGGCTTGCTTTGGGTTTGGCGCTTTTCATGTAACCGGATTGTATGGTCCTGGAATATGGGTGTCCGACCCTTATGGACTAACTGGAAAGGTACAACCTGTAAGTCCAGCATGGGGTGTGGAAGGTTTTGATCCTTTTGTTCCGGGAGGAATAGCCTCTCATCATATTGCAGCGGGGACATTGGGCATATTAGCGGGCCTATTCCATCTTAGTGTCCGTCCGCCCCAACGTTTATACAAAGGATTACGTATGGGAAATATTGAAACTGTCCTTTCCAGTAGTATCGCGGCTGTCTTTTTTGCAGCTTTTGTTGTTGCTGGAACTATGTGGTATGGTTCAGCAACTACCCCGATTGAATTATTTGGTCCCACTCGTTATCAATGGGATCAAGGATACTTCCAGCAAGAAATATATCGAAGAGTTAGTGCTGGGCTAGCCGAAAATCAAAGTTTATCCGAAGCTTGGTCTAAAATTCCCGAAAAATTAGCTTTTTATGATTACATCGGCAATAATCCGGCAAAGGGTGGGTTGTTCAGAGCAGGCTCAATGGACAACGGGGATGGAATAGCTGTTGGGTGGTTAGGACATCCTATCTTTAGAGATAAAGAAGGGCGTGAACTTTTTGTACGTCGTATGCCTACTTTTTTTGAAACATTTCCGGTTGTTTTGGTAGACGGAGATGGAATTGTTAGAGCCGATGTTCCTTTTCGAAGGGCAGAGTCGAAGTATAGTGTCGAACAAGTAGGTGTAACTGTTGAGTTCTATGGTGGCGAACTAAACGGAGTCAGTTATAGTGATCCTGCTACTGTGAAAAAATATGCTAGACGCGCTCAATTGGGTGAAATTTTTGAATTAGATCGTGCTACTTTGAAATCCGATGGTGTTTTTCGTAGCAGCCCAAGGGGTTGGTTTACTTTTGGACATGCTTCGTTCGCTCTGCTCTTTTTCTTCGGACACATTTGGCATGGTGCTCGAACTTTGTTCAGAGATGTTTTTGCTGGTATTGATCCAGATTTAGATGCTCAAGTGGAATTTGGAGCATTCCAAAAACTTGGAGATCCAACTACAAGAAGACAAGTAGTCTGA

>lcl|NC_015308.1_cds_YP_004327660.1_20 [gene=rps14] [locus_tag=HebrCp033] [db_xref=GeneID:10351976] [protein=ribosomal protein S14] [protein_id=YP_004327660.1] [location=complement(40421..40723)] [gbkey=CDS]
ATGGCAAGGAAAAGTTTGATTCAGCGGGAGAAGAAGAGGCAAAAATTGGAACAAAAATATCATTTGATGCGTCGATCCTCAAAAAAAGAAATAAGCAAAGTTCCGTCGTTAAGTGATAAATGGGAAATTCATGGAAAGTTACAATCCCCACCGCGGAATAGTGCACCGACACGTCTTCATCGACGTTGTTTTTCGACTGGAAAACCGAGAGCTAACTATCGAGACTTTGGGCTATCCGGACACATACTTCGTGAAATGGTTCATGCATGTTTGTTGCCGGGGGCAACAAGATCAAGTTGGTAA

>lcl|NC_015308.1_cds_YP_004327662.1_22 [gene=psaA] [locus_tag=HebrCp035] [db_xref=GeneID:10351904] [protein=photosystem I P700 chlorophyll a apoprotein A1] [protein_id=YP_004327662.1] [location=complement(43072..45324)] [gbkey=CDS]
ATGATTATTCGTTCGCCGGAACCAGAAGTAAAAATTTTGGTAGATAGGGATCCCATCAAAACTTCTTTCGAGGAATGGGCCAGACCCGGTCATTTCTCAAGAACAATAGCTAAAGGACCTGATACTACCACTTGGATCTGGAACCTACATGCTGATGCTCACGATTTCGATAGCCATACCAGTGATTTGGAGGAGATTTCTCGGAAAGTATTTAGTGCTCATTTCGGCCAACTCTCCATCATCTTTCTTTGGCTGAGTGGCATGTATTTCCACGGTGCTCGTTTTTCCAATTATGAAGCATGGCTAAGCGATCCTACTCACATTGGACCTAGCGCTCAAGTGGTTTGGCCAATAGTGGGCCAAGAAATATTGAATGGCGATGTGGGCGGGGGTTTCCGAGGAATACAAATAACTTCCGGTTTTTTTCAGATTTGGAGAGCATCTGGAATAACTAGTGAATTACAACTGTATTGTACCGCAATTGGTGCATTGGTCTTTGCAGCCTTAATGCTTTTTGCTGGTTGGTTCCATTATCACAAAGCTGCTCCAAAATTGGCTTGGTTCCAAGATGTAGAATCTATGTTGAATCACCATTTAGCGGGGCTACTAGGACTTGGGTCTCTTTCTTGGGCGGGACATCAAGTACATGTATCTTTACCAATTAACCAATTTCTAAACGCTGGAGTGGATCCTAAAGAAATCCCACTTCCTCATGAATTTATCTTGAATCGGGATCTTTTGGCTCAACTTTATCCCAGTTTTGCTGAGGGAGCAACCCCATTTTTTACCTTGAATTGGTCAAAATATTCGGACTTTCTTACTTTTCGTGGAGGATTAGATCCAGTGACTGGGGGTCTATGGCTGACCGATACTGCACACCATCATTTAGCTATTGCAATTCTTTTCCTGATAGCGGGTCACATGTATAGGACTAACTGGGCCATTGGTCATGGTATAAAAGAGATTTTAGAGGCTCATAAAGGTCCATTTACAGGTCAGGGCCATAAAGGCCTATATGAGATCCTAACAACGTCATGGCATGCTCAATTATCTCTTAACCTAGCTATGTTAGGTTCTTTAACCATTGTTGTAGCTCACCATATGTATTCCATGCCCCCTTATCCATATCTAGCTACTGATTATGGTACACAACTGTCATTGTTCACACATCACATGTGGATTGGTGGATTTCTCATAGTTGGTGCTGCTGCGCATGCAGCCATTTTTATGGTAAGAGACTATGATCCAACTACTCGATACAACGATCTATTAGATCGTGTCCTTAGGCATCGTGATGCAATCATATCACATCTCAACTGGGTATGTATATTTTTAGGCTTTCACAGTTTTGGTTTATATATTCATAATGATACCATGAGCGCTTTAGGGCGCCCTCAAGATATGTTTTCAGATATTGCTATACAATTACAACCTGTCTTTGCTCAATGGATACAAAACACCCATGCTTTAGCACCTGGTGCAACGGCTCCTGGTGCAACAGCAAGCACCAGTTTAACTTGGGGGGGTGGTGATTTAGTGGCAGTTGGTGGCAAGGTTGCTTTATTACCGATTCCATTAGGAACCGCGGATTTTTTGGTACATCACATTCATGCATTCACGATTCATGTGACGGTATTGATACTTCTGAAAGGAGTTCTATTTGCCCGCAGCTCTCGTTTGATACCGGATAAAGCAAATCTTGGTTTTCGTTTCCCTTGTGATGGACCTGGAAGAGGGGGAACATGTCAAGTATCCGCTTGGGATCATGTCTTCTTAGGGCTATTTTGGATGTACAATTCCATTTCGGTAGTAATATTCCATTTCAGTTGGAAAATGCAGTCAGATGTTTGGGGTAGTATAAGTGATCAAGGGGTGGTAACTCATATCACGGGAGGAAACTTTGCACAGAGTTCCATTACTATTAATGGATGGCTCCGCGATTTCTTATGGGCACAGGCATCCCAGGTAATTCAGTCTTATGGTTCTTCATTATCTGCATATGGCCTTTTTTTCCTAGGTGCTCATTTTGTATGGGCTTTTAGTTTAATGTTTCTATTCAGCGGTCGTGGTTATTGGCAAGAACTTATTGAATCAATCGTTTGGGCTCATAATAAATTAAAAGTTGCTCCTGCTACTCAGCCTAGAGCCTTGAGCATTATACAAGGACGTGCTGTAGGAGTAACCCATTACCTTCTGGGTGGAATTGCCACAACATGGGCGTTCTTCTTAGCAAGAATTATTGCAGTAGGATAA

3. *Jatropha curcas*

>lcl|NC_012224.1_cds_YP_002720103.1_11 [gene=rpoC1] [locus_tag=JacuC_p011] [db_xref=GeneID:7564839] [protein=RNA polymerase beta' subunit] [protein_id=YP_002720103.1] [location=complement(join(22413..24023,24804..25235))] [gbkey=CDS]
ATGATTGATCGGTATAAACATCAACAACTCCGAATTGGATCAGTTTCGCCTCAACAAATAAGTGCTTGGGCCAATAAAATCCTACCTAACGGAGAGATTGTTGGAGAGGTGACAAAACCCTATACTTTTCATTACAAAACCAATAAACCTGAAAAAGATGGATTATTTTGTGAAAGAATTTTTGGGCCTATAAAAAGTGGAATTTGTGCTTGTGGAAATTATCGAGTAATCAGAAATGAAAAAGAAGACCCAAAATTTTGTGAACAATGCGGAGTCGAGTTTGTTGATTCTCGGATACGAAGATATCAAATGGGCTACATCAAACTGGCATGCCCAGTAACTCATGTGTGGTATTTGAAACGTCTTCCTAGTTATATCGCAAATCTTTTAGATAAACCTCTTAAAGAATTAGAGGGCCTAGTATACTGCGATTTTTCTTTTGCTAGGCCCATAGCTAAAAAACCCACTTTTTTACGATTACGAGGTTCATTCGAATATGAAATCCAATCTTGGAAATACAGTATTCCACTTTTTTTTACTACCCAAGGCTTCGATACATTTCGAAATCGAGAAATTTCTACAGGAGCTGGTGCTATCCGAGAACAATTAGCCGATCTGGATTTGCGAATTATTATAGATTATTCATCGGTAGAATGGAAAGAATTAGGGGAAGAAGGGCCTACAGGGAATGAATGGGAAGATCGAAAAGTTGGAAGAAGAAAGGATTTTTTGGTTAGACGCGTGGAATTAGCTAAGCATTTTATTCGAACAAATATAGAACCAGAATGGATGGTTTTATGTCTATTACCGGTTCTTCCTCCCGAGTTGAGACCAATCATTCAGATAGATGGGGGTAAACTAATGAGTTCAGATATTAATGAACTCTATAGAAGAGTTATCTATCGGAACAATACTCTTATTGATCTATTAACAACAAGTAGATCTACGCCAGGGGAATTAGTAATGTGTCAGGAGAAATTGGTACAAGAAGCCGTGGATACACTTCTTGATAATGGAATCCGCGGACAACCTATGAGGGACGGTCATAATAAGGTTTACAAGTCGTTTTCGGATGTAATTGAAGGCAAAGAAGGAAGATTTCGTGAGACTATGCTTGGCAAACGGGTTGATTATTCGGGGCGTTCTGTCATTGTCGTAGGCCCCTCACTTTCATTACATCGATGTGGATTGCCTCGCGAAATAGCAATAGAGCTTTTCCAGATATTTGTAATTCGTGGTCTAATTAGGCAACATCTTGCTTCGAACATAGGAGTTGCTAAGAGTAAAATTCGGGAAAAAGAACCAATTGTATGGGAAATACTTCAAGAAGTTATGCAGGGACATCCGGTATTACTGAATAGAGCGCCGACTCTGCATAGATTAGGCATACAGGCGTTCCAACCCATTTTAGTGGAAGGCCGTGCTATTTGTTTACATCCATTGGTTTGTAAGGGATTCAATGCAGACTTTGATGGGGATCAAATGGCTGTTCATGTACCTTTATCGTTGGAGGCTCAAACAGAGGCTCGTTTACTTATGTTTTCTCATATGAATCTCTTGTCTCCAGCTATTGGAGATCCCATCTCCGTACCAACTCAAGATATGCTTATTGGGCTCTATGTATTAACAAGCGGGAATCGTCGAGGTATTTGTGCAAATAGGTATAATCCATGTAATCGCAGAAATTCTCAAAATGAAAGAATTGACGATAATAACAATAAATATACGAAAGAACCTTTTTTTTCTAATTCTTATGATGCAATTGGCGCTTTTCGGCGGAAAAGAATCAATTTAGATAGCCCTTTGTGGCTCCGGTGGCAACTAGATCAACGCGCTATTGTTTCAAGAGAAGCTCCCCTCGAAGTTCACTATGAATCTTTAGGTACCTATCATGAGATTTATGAACACTATCTAATAGTAAGAAATATAAAAAAAGAAATTATTTGTATATACATTCGAACTACTGTTGGTCATATTTCTTTTTATCGAGAAATCGAAGAAGCTATACAAGGGTTTTGTCAAGCCTGCTCAGATGGTATCTAA

>lcl|NC_012224.1_cds_YP_002720097.1_5 [gene=atpA] [locus_tag=JacuC_p005] [db_xref=GeneID:7564833] [protein=ATP synthase CF1 alpha subunit] [protein_id=YP_002720097.1] [location=complement(11147..12670)] [gbkey=CDS]
ATGGTAACTATTCGAGCCGACGAGATTAGTAATATTATCCGCGAACGTATTGAACAATATAATAGGGAAGTAAAGATTGTAAATACCGGTACTGTACTTCAAGTAGGCGACGGTATTGCTCGTATTCATGGTCTTGATGAAGTAATGGCAGGGGAATTAGTAGAGTTTGAAGAGGGTACAATAGGCATTGCTCTGAATTTGGAATCAAATAATGTCGGTGTTGTATTAATGGGTGACGGTTTAATGATACAAGAGGGAAGCTCCGTAAAAGCAACAGGAAGAATTGCTCAGATACCGGTGAGTGAGGCTTATTTGGGTCGTGTTATAAATGCTTTGGCTAAACCTATTGACGGTCGAGGTGAAATTTCCGCTTCTGAATCTCGGTTAATTGAATCTCCTGCTCCAGGGATTATTTCTAGACGTTCCGTATATGAGCCTCTTCAAACAGGACTTATTGCTATTGATTCGATGATTCCTATAGGACGCGGTCAACGAGAATTAATTATTGGGGATAGACAGACCGGTAAAACAGCAGTAGCCACAGATACAATTCTCAATCAACAAGGACAAAATGTAATATGTGTTTATGTAGCTATTGGGCAAAAAGCGTCTTCTGTGGCTCAGGTAGTGACTACTTTACAGGAAAGAGGAGCAATGGAGTACACTATTGTGGTAGCCGAAACAGCGGATTCCCCGGCTACATTACAATATCTCGCTCCTTATACAGGAGCAGCTCTGGCTGAATATTTTATGTACCGGGAACGACACACTTTAATCATTTATGATGATCTCTCCAAACAAGCGCAGGCTTATCGCCAAATGTCTCTTCTATTACGAAGACCACCGGGTCGTGAAGCTTATCCAGGAGATGTCTTTTATTTGCATTCACGCCTTTTGGAAAGAGCCGCTAAATCAAGTTCTCGTTTAGGTGAAGGAAGTATGACTGCTTTACCAATAGTTGAGACCCAATCAGGAGACGTTTCGGCTTATATTCCTACTAATGTAATTTCCATTACAGATGGACAAATATTCTTATCCGCCGATCTATTCAATGCTGGAATCAGACCCGCTATTAATGTGGGTATTTCCGTTTCCAGAGTAGGATCTGCGGCTCAAATTAAAGCTATGAAACAGGTAGCTGGTAAGTTAAAATTGGAATTGGCACAATTCGCAGAATTAGAAGCCTTTGCGCAATTCGCTTCTGATCTCGATAAAGCTACTCAGAATCAATTGGCAAGAGGTCAACGATTACGCGAGTTGCTCAAACAATCCCAATCCGCTCCTCTCACAGTGGAGGAACAGATAATGACTATTTATGCCGGAACGACGGGTTATCTTGATTCATTAGAAGTTGGACAAATAAAGAAATTTCTCGTTGAGTTACGTACTTACTTAAAAACGAATAAACCTGAGTTCCGAGAAATAATATCTTCTACTAAAACATTCACCGAAGAAGCAGAAAGCCTTTTGAAAGAAGCTATTCAGGAACTGAAGGAGCGTTTTCTTCTTCAGGAACAAGTATAA

>lcl|NC_012224.1_cds_YP_002720153.1_61 [gene=rpl2] [locus_tag=JacuC_p061] [db_xref=GeneID:7564775] [protein=ribosomal protein L2] [protein_id=YP_002720153.1] [location=complement(join(91732..92202,92832..93224))] [gbkey=CDS]
ATGGCGATACATTTATACAAAACTTCTACCCCGAGCACACGCAATGGAGCCGTAGACAGTCAAGTGAAATCCAATACACGAAATAATTTGATCTATGGACAGCATCGTTGTGGTAAAGGACGTAATGCCAGAGGAATCATTACCGCAAGGCATAGAGGGGGGGGTCATAAGCGTCTATACCGTAAAATCGATTTTCGACGGAATGAAAAAGACATATATGGTAGAATCGTAACCATAGAATACGACCCTAATCGAAATGCATACATTTGTCTCATACACTATGGGGATGGTGAGAAGAGATATATTTTACATCCCAGAGGGGCTATAATTGGAGATACCATTATTTCTGGTACAGAAGTTCCTATAAAAATGGGAAATGCCCTACCTTTGAGTGAGGTTTTGATTGATCAAAAAGAAGAATCTACTTCAACCGATATGCCCTTAGGCACGGCCATACATAACATAGAAATCACACTTGGAAAGGGTGGACAATTAGCTAGAGCTGCAGGTGCTGTAGCGAAACTGATTGCAAAAGAGGGGAAATCGGCCACATTAAAATTACCTTCTGGGGAGGTTCGTTTAATATCCAAAAACTGCTCAGCAACAGTCGGACAAGTAGGCAATACTGGGGTGAACCAGAAAAGTTTGGGTAGAGCCGGATCTAAATGTTGGCTAGGTAAGCGTCCTGTAGTAAGAGGAGTAGTTATGAACCCTGTAGACCATCCCCATGGGGGTGGTGAAGGGAGGGCCCCAATTGGTAGAAAAAAACCCGCAACCCCTTGGGGGTATCCTGCACTTGGAAGAAGAAGTAGAAAAAGGAATAAATATAGTGATAATTTGATTCTTCGTCGCCGTACTAAGTAG

>lcl|NC_012224.1_cds_YP_002720145.1_53 [gene=rps11] [locus_tag=JacuC_p053] [db_xref=GeneID:7564767] [protein=ribosomal protein S11] [protein_id=YP_002720145.1] [location=complement(85598..86014)] [gbkey=CDS]
ATGGCAAAACCTTTACCAAGAATTGGTTCACGCAGAAATGGGCGTGTTGGCTCACGTAAGAATGCGCGTAAAATACCAAAAGGGGTTATTCATGTTCAAGCAAGTTTCAACAACACTATTGTGACCGTTACAGACGTACGGGGTCGAGTAATTTCTTGGTCCTCCGCTGGCACTTGCGGATTCAGGGGCACAAGAAGAGGCACGCCATTTGCTGCTCAAACCGCAGCAGGCAATGCTATTCGGACAGTAATGGATCAAGGTATGCAACAAGCAGAAGTGATGATAAAAGGCCCTGGTCTCGGACGAGATGCGGCATTAAGAGCTATTCGCAGAAGTGGTATACTATTAAGTTTCGTCCGGGATATAACCCCTATGCCACATAATGGCTGCAGGCCCCCTAAAAAAAGGCGTGTGTAA

>lcl|NC_012224.1_cds_YP_002720110.1_18 [gene=rps14] [locus_tag=JacuC_p018] [db_xref=GeneID:7564854] [protein=ribosomal protein S14] [protein_id=YP_002720110.1] [location=complement(41894..42196)] [gbkey=CDS]
ATGGCAAGGAAAAGTTTGATTCAGCGGGAGAAGAAGAGGCAAAAATTGGAACAAAAATATCATTTGATTCGTCGATCCTCAAAAAAAGAAATAAGCGAAGTTCTATCGTTGAGTGATAAATGGGAAATTCATGGAAAGTTACAATCCCTACCGCGAAATAGTGCACCGACACGTCTTCATCGACGTTGTTTTTCGACTGGAAGACCGAGAGCTAACTATCGAGACTTTGGGCTATCTGGACACATACTTCGTGAAATGGTTCATGCATGTTTGTTACCGGGGGTAACAAGATCAAGTTGGTAA

>lcl|NC_012224.1_cds_YP_002720142.1_50 [gene=petB] [locus_tag=JacuC_p050] [db_xref=GeneID:7564764] [protein=cytochrome b6] [protein_id=YP_002720142.1] [location=join(81321..81326,82105..82746)] [gbkey=CDS]
ATGAGTAAAGTTTATGATTGGTTCGAAGAACGTCTCGAGATTCAGGCGATTGCAGATGATATAACTAGTAAATATGTTCCTCCCCATGTCAACATATTTTATTGTTTAGGAGGAATTACGCTTACTTGTTTTTTAGTACAAGTAGCTACGGGATTTGCTATGACTTTTTACTACCGTCCGACCGTTACTGAGGCTTTTGCTTCTGTTCAATACATAATGACTGAAGCTAACTTTGGTTGGTTAATCCGATCAGTTCATCGATGGTCGGCAAGTATGATGGTTTTAATGATGATCCTGCACGTATTTCGTGTGTATCTCACTGGTGGCTTTAAAAAACCTCGTGAATTGACTTGGGTTACGGGCGTGGTTCTTGCTGTATTGACCGCATCTTTTGGTGTAACCGGTTATTCCTTACCTTGGGACCAAATTGGTTATTGGGCAGTCAAAATTGTAACAGGCGTGCCGGAAGCTATTCCTGTAATAGGATCGCCTTTGGTAGAGTTATTACGTGGAAGCGCTAGTGTAGGACAATCCACTTTGACTCGTTTTTATAGTTTACACACTTTTGTATTACCCCTCCTTACTGCCGTATTTATGTTAATGCACTTTTCAATGATACGTAAGCAAGGTATTTCAGGCCCTTTATAG

>lcl|NC_012224.1_cds_YP_002720138.1_46 [gene=psbB] [locus_tag=JacuC_p046] [db_xref=GeneID:7564760] [protein=photosystem II 47 kDa protein] [protein_id=YP_002720138.1] [location=78922..80448] [gbkey=CDS]
ATGGGTTTGCCTTGGTATCGTGTTCATACCGTCGTATTGAATGATCCCGGTCGTTTGCTGTCTGTCCATATAATGCATACAGCCTTGGTTGCTGGTTGGGCCGGTTCGATGGCTCTATATGAATTAGCAGTTTTTGATCCCTCTGACCCCGTTCTCGATCCAATGTGGAGACAGGGTATGTTCGTTATACCCTTCATGACTCGTTTAGGAATAACCAATTCGTGGGGTGGTTGGAGTATCACAGGAGGAACTATAACGAATCCGGGTATTTGGAGTTATGAAGGTGTGGCTGGGGCGCATATTGTGTTTTCTGGCTTGTGCTTTTTGGCAGCTATTTGGCATTGGGTGTATTGGGATCTAGAGGTATTTTGCGATGAACGTACAGGAAAACCTTCTTTAGATTTGCCCAAGATCTTTGGAATTCATTTATTTCTCTCAGGAGTGGCTTGCTTTGGGTTTGGTGCTTTTCATGTAACCGGATTGTATGGTCCTGGAATATGGGTGTCCGATCCTTATGGACTAACAGGAAAGGTACAACCTGTAAGTCCAGCATGGGGTGTGGAAGGTTTTGATCCTTTTGTTCCAGGAGGAATAGCTTCTCATCATATTGCAGCGGGGACATTGGGCATATTAGCGGGCCTCTTCCATCTTAGTGTCCGTCCGCCCCAACGTTTATACAAAGGATTGCGTATGGGAAATATTGAAACTGTCCTTTCCAGTAGTATCGCTGCTGTCTTTTTTGCAGCTTTTGTTGTTGCTGGAACTATGTGGTATGGTTCAGCAACTACCCCGATTGAATTATTTGGTCCTACTCGTTATCAATGGGATCAAGGATACTTCCAGCAAGAAATATATCGAAGAGTTAGTGCTGGGCTAGCCGAAAATCAAAGTTTATCCGAAGCTTGGTCTAAAATTCCCGAAAAATTAGCCTTTTATGATTACATCGGCAATAATCCGGCAAAAGGTGGATTGTTCAGAGCAGGCTCAATGGACAACGGGGATGGAATAGCTGTTGGGTGGTTAGGACATCCTATCTTTAGAGATAAAGAAGGGCGTGAACTTTTTGTACGGCGTATGCCTACTTTTTTTGAAACATTTCCTGTTGTTTTGGTAGATGGAGATGGAATTGTTAGAGCCGATGTTCCTTTTCGAAGGGCAGAGTCAAAGTATAGTGTCGAACAAGTAGGTGTAACTGTTGAGTTCTATGGTGGCGAACTAAACGGAGTCAGTTATAGTGATCCTGCTACTGTGAAAAAATATGCTAGACGCGCTCAATTGGGTGAAATTTTTGAATTAGATCGTGCTACTTTGAAATCTGATGGTGTTTTTCGTAGCAGTCCAAGGGGTTGGTTTACTTTTGGACATGCTTCGTTTGCTCTGCTCTTTTTCTTCGGACACATTTGGCACGGTGCTCGAACTTTGTTCAGAGATGTTTTTGCTGGTATTGATCCAGATTTAGACGCTCAAGTGGAATTTGGAGCATTCCAAAAACTTGGAGATCCAACTACAAGAAGACAAGTAGTCTGA

>lcl|NC_012224.1_cds_YP_002720165.1_73 [gene=ndhG] [locus_tag=JacuC_p074] [db_xref=GeneID:7564800] [protein=NADH dehydrogenase subunit 6] [protein_id=YP_002720165.1] [location=complement(127302..127832)] [gbkey=CDS]
ATGGATTTGCCTGGACTAATACATGATTTTCTTTTAGTCTTTCTGGGATTAGGTCTTATATTAGGAGGTCTAGGAGTGGTATTATTTACCAATCCAATTTATTCTGCTTTTTCGTTGGGATTTGTTCTTGTTTGTATATCTTTATTCTATATTTTATCAAACTCTCATTTTGTAGCTGCTGCACAGCTCCTTATTTATGTGGGAGCTATAAATGTTTTAATTCTATTTGCCGTGATGTTCATGAATGGTTCAGAATATTACAAAGATTTTAATCTTTGGACTGTTGGAAACGGGGTTACTTCCTTAGTTTGTACAAGCATTTTTATTTCACTAATTACTATTATTTCAGATACATCATGGTACGGGATTATTTGGACTACAAGAACAAATCAGATTATAGAACAAGATTTAATAAGTAATGGGCAACAAATTGGAATTCATTTATCAACAGATTTTTTTCTTCCATTTGACTTTATTTCAGTAATTCTTTTAGTTGCTTTGATAGGTGCGATTGCTGTGGCTCGTCAGTAA

>lcl|NC_012224.1_cds_YP_002720125.1_33 [gene=petA] [locus_tag=JacuC_p033] [db_xref=GeneID:7564875] [protein=cytochrome f] [protein_id=YP_002720125.1] [location=67604..68566] [gbkey=CDS]
ATGCAAACTAGAAAGACCTTCTCTTGGATAAAGGAAGAGATTACTCGTTCCATTTCTGTATCGCTCATGGTATATATAATAACTTGGGCATCCATTTCAAACGCATATCCCATTTTTGCACAGCAGGGTTATGAAAATCCACGCGAAGCAACTGGCCGTATTGTATGTGCCAATTGTCATTTAGCTAATAAGCCCGTGGATATTGAGGTTCCACAGGCGGTACTTCCTGATACTGTATTTGAAGCAGTTGTTCGAATCCCTTATGATATGCAACTGAAACAAGTTCTTGCTAATGGTAAAAAAGGAGCTTTGAACGTGGGGGCTGTTCTTATTTTACCTGAGGGGTTTGAATTAGCCCCTCCCGATCGTATTTCGCCAGAGATTAAAGAAAAGATGGGAAATCTGTCTTTTCAGAGTTATCGCCCCACTAAAAAAAATATTCTTGTAATAGGCCCTGTTCCTGGTCAGAAATATAGTGAAATTACCTTTCCTATTCTGTCTCCGGACCCCGCCACTAAGAAAGATGTTCGTTTTTTAAAATATCCCATATATGTAGGCGGAAACAGGGGAAGGGGTCAGATTTATCCCGACGGGAGCAAGAGTAACAATACGGTTTATAATGCTACAGCAGCAGGTATAGTAAGCAAAATCATACGAAAAGAAAAAGGGGGGTACGAAATAACCATAACGGATGCGTCAGAGGGACGTCAAGTGATTGATATTATACCTCCAGGGCCAGAACTTCTTGTTTCAGAAGGCGAATCCATCAAACTGGATCAACCATTAACGAGTAATCCTAATGTGGGTGGATTTGGTCAGGGAGATGCAGAAATAGTGCTTCAAGACCCATTACGTGTCCAAGGGCTTTTTTTCTTCTTGGCATCCGTTATTTTGGCACAAATCTTTTTGGTTCTTAAAAAGAAACAGTTTGAGAAGGTTCAATTGTCCGAAATGAATTTTTAG

>lcl|NC_012224.1_cds_YP_002720108.1_16 [gene=psbC] [locus_tag=JacuC_p016] [db_xref=GeneID:7564849] [protein=photosystem II 44 kDa protein] [protein_id=YP_002720108.1] [location=38212..39633] [gbkey=CDS]
ATGAAAACCTTATATTCCCTGAGGAGGTTCTACCACGTGGAAACGCTCTTTAATGGAACTTTATCTTTAGCCGGTCGTGACCAAGAAACTACCGGTTTCGCTTGGTGGGCCGGGAATGCCCGACTTATCAATTTATCCGGTAAACTACTGGGAGCTCATGTAGCTCATGCTGGATTAATCGTATTCTGGGCCGGAGCAATGAACCTATTTGAAGTGGCTCATTTCGTACCGGAGAAACCAATGTATGAACAAGGATTAATTTTACTTCCCCACCTAGCTACTCTAGGTTGGGGGGTAGGTCCTGGTGGGGAAGTTATAGACACCTTTCCATACTTTGTATCTGGCGTACTTCACTTAATTTCCTCTGCAGTATTGGGCTTTGGCGGTATTTATCATGCACTTCTGGGTCCTGAGACTCTTGAAGAATCTTTTCCATTTTTTGGTTATGTATGGAAAGATAGAAATAAAATGACAACAATTTTAGGTATTCACTTAATCTTGCTAGGTATAGGTGCTTTTCTTCTAGTATTCAAGGCTCTTTATTTTGGGGGCGTATATGATACCTGGGCTCCGGGGGGGGGAGATGTAAGAAAAATTACCAACTTGACCCTTAGCCCAAGTGTTATTTTTGGTTATTTACTAAAATCCCCCTTTGGAGGAGAAGGATGGATTGTTAGTGTGGACGATTTGGAAGATATAATTGGAGGGCATGTATGGTTAGGTTCCATTTGTATACTTGGGGGAATCTGGCATATCTTAACCAAACCCTTTGCATGGGCTCGCCGTGCACTTGTATGGTCCGGAGAGGCTTACTTGTCTTATAGTTTAGGTGCTTTATCCGTTTTTGGTTTCATTGCTTGTTGCTTTGTCTGGTTCAATAATACCGCTTATCCTAGTGAGTTTTACGGGCCTACTGGACCGGAAGCTTCTCAAGCTCAAGCTTTTACTTTTCTAGTTAGAGATCAACGTCTTGGGGCTAACGTGGGATCCGCTCAAGGACCTACCGGGTTAGGTAAATATTTAATGCGTTCGCCTACTGGAGAAGTTATTTTTGGAGGAGAAACTATGCGTTTTTGGGATCTACGTGCTCCTTGGTTAGAACCTCTAAGAGGTCCAAATGGTTTGGACTTGAGTAGGTTGAAAAAAGACATACAACCTTGGCAAGAACGCCGTTCCGCGGAATATATGACCCATGCGCCTTTAGGTTCGTTGAATTCTGTAGGTGGCGTAGCTACCGAGATCAATGCAGTCAATTATGTCTCTCCTAGAAGTTGGTTAGCTACCTCTCATTTTGTTCTAGGGTTCTTCCTATTCGTAGGTCATTTATGGCACGCGGGAAGGGCTCGTGCAGCTGCAGCAGGATTTGAAAAAGGAATTGATCGTGATTTTGAACCTGTTCTTTCCATGACTCCTCTTAACTAA

>lcl|NC_012224.1_cds_YP_002720104.1_12 [gene=rpoB] [locus_tag=JacuC_p012] [db_xref=GeneID:7564840] [protein=RNA polymerase beta subunit] [protein_id=YP_002720104.1] [location=complement(25275..28493)] [gbkey=CDS]
ATGCTCGGGGATGGAAATGAGGGACTGTCTACAATACCTGGATTAAATCAGATACAATTTGAAGGATTTTGCGGGTTCATTGATCAGGGCTTAACAGAAGAACTTTATAAGTTTCCAAAAATGGAAGATACAGATCAAGAAATTGAATTTCAATTATTTGTGGAAACATATCAATTAGTAGAGCCATTGATAAAAGAAAGAGATGCTGTATATGAATCACTTACATATTCTTCTGAATTATATGTATCTGCGGGATTAATTTGGAAAACCAGTAGGGATATGCAAGAACAAACAATTTTTATTGGAAACATTCCTCTAATGAATTCCCTGGGAACTTTTATAATAAATGGAATATACAGAATTGTGATCAATCAAATATTGCAAAGTCCCGGTATCTATTACCGGTCAGAATTGGATCATAACGGAATCCCGGTCTATACCGGCACCATAATATCAGATTGGGGGGGAAGAGTAGAATTAGAGATTGATAGAAAAGCAAGGATATGGGCTCGTGTGAGTAGGAAACAGAAGATATCTATTCTAGTTTTATCATCAGCTATGGGTTTGAATCTAAGAGAAATTTTAGAGAATGTGTGCTGCCCTGAAATTTTCTTATCTTTTCTGAATCTGAATGATAAGGGAAAAAAAAAAATGGGGTCAAAGGAAAATGCCATTTTGGAGTTTTATCAACAATTTACTTGTGTAGGCGGAGATCCAGTATTTTCTGAATCCTTATGTAAGGAATTACAAAAGAAATTCTTTCAACAAAGATGTGAATTAGGAAGGATTGGTCGATTAAATATGAACCGGAGACTGAATCTTGATATACCTCATAACAATACATTTTTGTTACCACGAGATATATTGGCAGCTGCGGATCATTTGATTGGAATGAAATTTGGAATGGGTACACTTGACGATATGAATCATTTAAAAAATAAACGTATTCGTTCTGTAGCGGATCTCTTACAAGATCAATTCGGATTGGCTCTGATTCGTTTAGAAAACGTGGTTAGAGGGACTATATGGGGAGCAATTAGGCATAAATTGATACCGACCCCTCAAAATTTGGTAACTTCAACTCCATTAACAACTACTTATGAATCTTTTTTCGGATTACACCCATTATCTCAAGTTTTGGATCGAACTAATCCATTGACACAAATAGTTCATGGGAGAAAATCGAGTTATTTGGGTCCTGGAGGATTAACGGGACGAACTGCTAGTTTTCGAATACGAGATATCCACCCTAGTCACTATGGGCGCATTTGCCCAATTGACACGTCTGAAGGAATCAATGTTGGACTTATTGGGTCTTTAGCAATTCATGCCAAGATTGGTTATTGGGGGTCTTTAGAAAGCCCATTTTATGGAATCTCTGAGGGATCAAAAAAAGCACGGATGCTTTATTTATCACCAAATAGAGAGGAATACTATAGGGTAGCGGCAGGAAATTCTTTGGCGCTGAATCGAGGTGTTCAGGAAGAACAGGTTGCTCCAGCTCGATATCGTCAAGAATTCCTGACTATTGCATGGGAACAGGTGCATCTTCGAAGTATTTTTCCCTTCCAATATTTTTCTATTGGAGCTTCCCTCATTCCTTTTATCGAGCATAATGATGCGAATCGGGCTTTAATGAGTTCTAATATGCAACGTCAGGCAGTTCCACTTTCTCGATCCGAAAAATGCATTGTTGGAACTGGATTGGAACGCCAAGTTTCTCTAGATTCAGGGGTTCCTGCTATAGCCGAACACGAGGGAAAGATAATTTATACTGATATTGACAAAATCATTTTATCGGGTAATGGAGATACTCTACGCATTCCATTAGTTATGTATCAACGTTCCAACAAAAATACTTGTATGCATCAAAAAACCCAGGTTCAGCGGGGTAAATGCATTAAAAAGGGACAAGTTTTAGCGGATGGTGCCGCTACAGTTGGTGGCGAACTCGCCTTGGGCAAAAACGTATTAGTAGCTTATATGCCATGGGAGGGTTACAATTTTGAGGATGCGGTGCTCATTAGCGAACGTCTGGTATATGAAGATATTTATACTTCTTTTCACATACGGAAATATGAAATTCAGACTCATGTGACAAGCCAAGGACCTGAAAAGATCACTAATGAAATACCGCATCTAGAAGCCCATTTACTCCGAAATTTAGACAAAAATGGAATTGTGATCCTAGGATCTTGGGTGGAGACGGGCGATATTTTAGTAGGTAAATTAAGGCCTCAAATGGCGAAAGAATCATCGTATGCTCCAGAAGATAGATTATTAAGAGCTATACTTGGTATTCAGGTATCGACTTCAAAGGAAACTTGTCTAAAACTACCTATAGGTGGTAGGGGTCGAGTTATTGATGTGAGATGGATCCAGAAAAAGGGGGGTTCCAGTTATAATCCGGAAACGATTCGTGTCTATATTTTACAGAAACGTGAAATCAAAGTGGGTGATAAAGTAGCTGGAAGACATGGAAATAAAGGCATCATTTCCAAAATTTTGCCTAGACAGGATATGCCTTATTTGCAAGATGGAAGACCTGTTGATATGGTCTTCAACCCATTAGGAGTACCTTCACGAATGAATGTAGGACAGATATTTGAATGCTCACTCGGGTTAGCGGGGGGTCTGCTAGATAGACATTATCGAATAGCACCCTTTGATGAGAGATATGAACAAGAGGCTTCGAGAAAACTAGTGTTTTCGGAATTATATGAAGCCAGTAAGCAAACAGCAAATCCGTGGGTATTTGAACCCGAGTATCCGGGAAAAAGTAGAATATTTGATGGAAGAACGGGGGATCCTTTTGAACAGCCTGTTATAATAGGAAAGCCTTATATCTTGAAATTAATTCATCAAGTTGATGATAAAATACATGGACGTTCCAGTGGACATTATGCACTTGTTACACAACAACCCCTTAGAGGAAGGGCCAAGCAAGGGGGACAACGGGTCGGAGAAATGGAGGTTTGGGCTCTAGAGGGATTTGGTGTTTCTCATATTTTACAAGAGATGCTTACTTATAAATCTGATCATATTAGAGCTCGCCAAGAAGTACTTGGTACTACGATCATTGGGGGAACAATACCTAAACCTGAAGATGCTCCAGAATCTTTTCGATTGCTCGTTCGAGAACTACGATCTTTGGCTCTGGAACTGGATCATTTCCTTGTATCTGAGAAGAACTTCCAGATTAATAGGAAGGAAGTTTAA

>lcl|NC_012224.1_cds_YP_002720144.1_52 [gene=rpoA] [locus_tag=JacuC_p052] [db_xref=GeneID:7564766] [protein=RNA polymerase alpha subunit] [protein_id=YP_002720144.1] [location=complement(84516..85535)] [gbkey=CDS]
ATGGTTCGAGAGAAAGTAACAATATCTACTCGGACACTGCAGTGGAAATGTGTTGAATCAAGAACGGACAATAAACGTCTTTATTATGGACGCTTTATTCTATCTCCACTTATGAAAGGCCAAGCTGACACAATAGGCATTGCGATGCGAAGAGCTTTGCTTGGAGAAATAGAAGGAACATGTATCACACGTGCAAAATCTGAGAAAATACCACACGAATTTTCTACTATAGCAGGTATTCAAGAATCAATACATGAAATTTTAATGAATTTGAAAAATGTTGTATTGAAAAGCAATTTGTATGGAACTTGTGACGCGTCTATTTATGTCAAAGGTCCTGGATATGTAACTGCCCAAGACATCATCTTACCGCCTTTTGTGGAAATTATTGATAATACACAGCATATCGCTAGCCTAACGGAATCAATTGATTTGTGTATTGGATTACAAATCGAGAGGAATCGCGGCTATCGTATAAAACCGACAAATAACTTTCAGGACGGAAGTTATCCTATAGATGCTGTATTCATGCCTGTTCGAAATGCGAATCATAGTGTTCATTCTTATGGAAATGGAAATGAAAAGCAAGAGATACTTTTTCTCGAAATATGGACAAACGGAAGTTTAACTCCTAAAGAAGCACTTCATGAAGCCTCCCGAAATTTGATTGATTTATTTATTCCTTTTCTACATGCAGAAGAAGAAAACTTACATTTAGAAATAAATCAACACAAGGTTACTTTACCCCTTTTTACTTTTCATGGTAGATTGACTAAATTAAGAAAAAATCAAAAAGAAATAGCATTGAAATACATTTTTATTGACCAATCAGAATTGACCCCTAAGACCTATAATTGCCTCAAAAGGTCCAATATACATACATTATCGGACCTTTTAAATAAGAGTCAAGAAGATCTTATGAAAATTGAAAATTTTCGCATAGACGATGTAAAACATATATTGGGTATTCTAGAAATAGAAAAACATTTCGCAATTGATTTACCAAAGAATAAAATATAA

>lcl|NC_012224.1_cds_YP_002720170.1_78 [gene=ycf1] [locus_tag=JacuC_p079] [db_xref=GeneID:7564805] [protein=Ycf1] [protein_id=YP_002720170.1] [location=complement(133196..138919)] [gbkey=CDS]
ATGATTTTGAAATCTTTTATACTAGGTAATCTAGTATCCTTATGCATGAAGATAATCAATTCGGTCGTTGTGGTCGGACTCTATTATGGATTTCTGACCACATTCTCCACGGGGCCCTCTTATCTCTTCCTTCTTCGAGCTCGGGTTATAGAAGAAGGAGAAGAAGGAACTGAGAAGAAGGTATCAGCAACAACAGGTTTTATTACGGGACAGCTCATGATGTTCATATCGATCTATTATGCGCCTCTGCATCTAGCATTGGGTAGACCTCATACAATAACTGTCCTAGCTCTACCCTATCTTTTGTTTCATTTCTTCTGGAACAATCACAAACACTTTTTTGATTATGGATCTACTACCAGAAATTCAATGCGTAATCTTAGCATTCAATTTGTATTCCTGAATAATCTCATTTTTCAATTATTCAACCATTTCATTTTACCAAGTTCAATGTTAGTCAGATTAGTCAACATTTATATGTTTCGATGCAACAACAAGATGTTATTTGTAACAAGTAGTTTTGTTGGTTGGTTAATTGGTCACATTTTATTCATGAAATGGGTTGGATTGATATTAGTCTGGATACAGCAAAATAATTCTATTAGATCGAATGTACTTTTTCGATCTAATAAGTACCTTGTGTCAGAATTGAGAAATTCTATGGCTCGAATCTTTAGTATTCTCTTATTTATTACCTGTGTCTACTCTTTAGGCAGAATACCGTCACCCATTTTTACTAAGAAACTGAAAGAAACCTCAGAAGCGGAAGAAAGGGAGGAAGAAACAGATGTAGAAATAGAAACAACTTCCGAAACGAAGGGGACTAAACAGGAACAAGAGGGATCCACCGAAGAAGATCCTTCTTCTTCCCTTTTTTCGGAAGAAAAGGAGGATCCGGACAAAATCGACGAAACGGAAGAGATCCAAGTGAATGGAAAGGAAAAAACAAAGGATGAATTCCATTTTCACTTTAAAGAGACATGCTATAAAAATAGACCACTTTATGAAACTTTTTATCTGGATGGGAATCAAGAAAATTCGAAGTTAGAAATATTGATAGATAAAAAAAATAAAGATCTTTTCTGGTTTGAAAAACCTCTTGTAACTATTCTTTTTGACTCTAAACGTTGGAATCGTCCATTTCGATATATAAAAAATGATCAGTTTGAGAATGCTGTAAGAAAAGAAATGTCACAATATTTTTTTTATACATGTCGAAGTGATGGAAAAGAAAGAATATCTTTTACGTATCCACCCAGTTTGTCAACTTTTTTGGAAATGATACAAAGAAGGATGTCCCTGTTTACAACAGAAAAACTCTCCTCCGATGAATTGTATAATCGTTGGAATTATAAGAATGAACAAAAAAAGAAAAATCTAAGTAATGAATTTATAAAAAGAGTCCAGGCTTTAGATAAGGGATATCTTGCTCTGAATACACTCCAAAAAAGGACTAGATTGTGTAATGATAAAACTAAAAAAGAGTACTTACCTAAAATATATGATCCCTTATTGAGTGGGTCATACCGCGGAAAAATCCAATTTTTTTTTTCACCCTCAATTTTAAATAAAACTTCCATAAAAAATTCTATAGAGATGGTTTGGATAAATAAAATTCATCTTTTTCTTCTTATTACTAATTATCAAAAATTCGAACCAAAAACAGATGTAAAATCATTTTCAACAGAAATTGCTTATTTCTTAAACTTAATTAATGAATTTGCCGGAAAATCAAGATCGAGTTTAAATTTTAAGGAACTCTCTTTATTTCCAGATCACAAAGAAGAAAAAATGGATTTAGAAAATCGAATAAAAATTTTCAAATTTTTATTTGATACAGTTATCGCGAATTCAAAAAATAAAACAATTAGAAATAATTCTATTGGAATAAAAGAAATAAGTAAACAAGTTCCTCGATGGTCATATAAATTAATTGACGATTTGGAACAACAAGAGGGAGAAAATGAAGAAAATGAAGAAAACATGGCGGAAGATCATGAAATTCGTTCACGAAAAGCCAAACGTGTAGTGATTTTTACTGATAATCAACAAAATACTGAGACTTATAATAATACCAAAGATACAACGAATTCTGATCAAATAGACAAAGTGACTTTGATACGTTATTCACAACAATCGGACTTTCGTCGAGACATAATAAAAGGATCTATGCGAGCACAAAGACGCAAAATACCTATATTGGAACTGTTTCAAGCAAATGTGCATTCCCCCCTTTTTTTGGACAGAATAGACAAATCCCTTGTTTTTTCTTTTGATATTTCCGAACTAATGAAAACAATGTTTATAAATTGGACGTGTAAAAACGCAGAATTCACAATTTCGGATTCTACTTATACAGAGAAAAAACAAAAAGAAAGTAAAAAAAAAGAAGACGACAAAAGAGAGGAAAAAGCTCGGATAGAAATAGCCGAAGCCTGGGATAGCATTCTTTTTGCTCAAGCAATAAGAGGGTGTGTTTTAGTAACCCAGTCGATTCTTAGAAAATATATTATATTAACTTTTTTAATAATAACTAAAAATATCATTCGTATACTACTTTTTCAAATTCCCGAATGGTCTGAGGATTTTAAAGATTGGAGTAGAGAAATGCATGTTAAATGCACTTATAATGGAGTTCAATTATCCGAAAAAGAATTTCCAAAAAACTGGTTAACAGACGGGATTCAAATAAAGATCCTATTTCCTTTTCGTCTAAAACCTTGGCACAGATCTAAGTTAAAACTCCCTCCCAAAGATCCAATGAAAAAGAAAACACAAAAACGGATTTTTGTTTTTTTAACAGTTTGGGGAATGGAAGTTGAATTGCCTTTTGGTTCTCCCCGAAAACGGCTTTCCCTCTTTGAACCCATCTTTAAAAAACTCGAAAAAAAAATTCGAAAAATAAAAAAAAAAGGTTTTCGAGTTCTAACAATTTTAGAAGAAAGAAGAAAATTTTTTCTAAAATTATCAAAAGAAAAAAAAATTGGGTTATCAAAAACATTTTTTTTCGAAAAGAAAAAAGAAATAATAAACAAATTTTCAAAATCAAAAAGAAATGCAAATTTATTATCTGGATTTATAAAAGTATATGAATTGAATGAAACTAAAAAAGAAAAAAATTCAATAATCGATAACAATAATGGAACGATTCAAAAATTGTCTACTCCAACTCGATCTATGGCTTGGACAAATTATTCACTAACAGAAAAAAAAATGAAAGATCTTTCTTCTAGAAGAAAGATAATCATAAATCAAATAGAAAAAATGAAAAAAGAAAAGGAAACAAAAATTATAACCTCAAAAATACATATTAATCCTAACAAAATAAGTTATAATGTTAAAAAATTAAAATCATCAAAAAATATTTCACAGATACAGATATTAAAAAGGGGAAATGCTCGATTAGTGCGTAAATTCCATTTTTTTATAAAAATTTTGATTCAAAGGATATACATAGATATCTTTTTAAGTATCATTAATATTCCGAGGATCAATGCACAGCTTTTTCTTGAATCAACAAAAAAATTATTACTAAATATGTTTGCAATAATGAAAAAAAAATCACAAAAAATTGATAAAACAAAGCAAAATACAATTCACTTTCTTTCGATTATGATTATAAAAAAGTTACTTAATAATAGTAATATTGCTGTTATTAATAACAATTCACAGATTTTTTGTGATATATCCTCCTTGTCACAAACATATGTATTTTACAAATTATCACAAATCCAAATTATTAATTTATATAAGTTACGATCTATCCTTCAATATCATGGCCTTTTTCTTAAGAATGAGATAAAGGATTATTTTAGAGCCCAAGGGATATTTAATTCCAAATTAAAAGATAAAAATTTTCAAAATTTCGTAATAAATCAATGGAAAAACTGGTTAAGGGGTCATTATCAATATAAATATGATTTAACTCAGATTAGATGGTCTAGATTAATATCACAAAAATGGCGAAATAGAATCAAGCAACACCATATGGTTCAAAATAAAAAATTTAATAAATTCAATTTATATGAAAAAGACCAATTAAATGAAAAAAGACCAATTAATTCATTACGAAAAAAAAATGATAATGATTTTGCGGCAGATTCATTACCGAATCAAAAAAAGAATTTTCAAAAACACTGTAGATATAATCTTTTATCATATAAATCCATTAATTATGAAAATAAGAAGAACTCATATATTTACGGATTCCCATTACAAGTAAATAAACAAGAGATTTCTTATAATTACAACACAAATAAAAGCAAATTATTTGACATGTTGGAAAGTATTCCTATCAATAATTATCTAGGGGAAGATGATATTAGCGATATGGAGAAAAGCCCGACTAGAAAATATTTGGATTGGAGAATTCTCGATTTTTGTCTTAGAAAAAAGGTCGATATTGAGTCCTGGATTGATATCGGAAGCAAAGAAAAAAAAAATACTAAGACTAGGACTAATAAGTATCAAATAATTGATAAAATCGATAAGAAAAATCTTCTTTTTCTTACAATTCACCAAGATCAAGAAGTCAATTCATCCAATCAAAAAAAAAACCTTTTTGATTGGCTGGGAATGAATGAAGAAATACAAAATCGTCTCATATCCAATTTTGAACTTTGGTTCTTTCGAAAATTTGTGATACTTTATAACACATATAAGATAAAACCATGGGCAATACCCATCCAATTTCTTCTTTTCAATTTTCATAGAAATGAAAATGTTAGTAAAAATACTAAAATCAACGAGAAGAAAAACGGCGATCTTTTTATATCATCGAATGAAAAAAAATTCATTGAATTAGAGAATCGAAATCACGAAGAAAAAGAATCCAAAGACAAAATGGACTTTGGATCAGTTTTCGCAAATCAAGAAAAAAATATTGAAGAAGATTATATGGGATTAGATATGAAAAAACATAGAAATAAAAAACAAAACAAAAGTCATACGGAAGTAGAGCTTGATTTCTTCCTAAAACGGTATTTATGTTTTCAATTAAGATGGAATGATTCTTTAAATGAAAAAATAATCAATAATATCAAAGTATATTGTCTCCTGCTTAGACTGACAAATCCACGAGAAATTCTTATATCTTCTATTCAAAGACAAGAAATAAGTCTGAATATTCTGATGGTTCAGAAGGATTTAACTCTTACTGAATTGATGAAAAAGGGAATATTTATTATCGAACCTGTTCGTTTGTCAGTAAAAAATAATGGACAATTTCTTTTGTATCAAACGGTAGGTATCTTATTAGTTCATAAGAACAAACAACAAATTAATCAAAAATACAGAGAAAAAATCTATATTGATAAAAAGAATTTTACCGAACCTATTGAAAGACATCAAAGTCTAATTGGCAATAGAGACAAAAATGATTATGATTTACTTGTTCCTGAAAATATTTTATTCCCTAAACGTCGTAGAGAATTAAGAATTCTAACTTCTTTCAATTTTAAAAACCAAAATGATATTCATAAAAATGATATTCATATAAATACAGAAATTTTCAACGTTAATAAAATAAAAAACTGTGGTCCTATTTTGGATAAAAGTAAACATTTTGATAGAGATAAAAATAAACTAATTAAATTCAAATTTTTTCTTTGGCCCCATTTTCGATTAGAAGATTTAGCTTGTATGAATCGCTATTGGTTTGATACTAATAATGCCAGTTGCTTCAGTATGGTAAGAATATATATATATCCACGGGCGCAATTTTAG

>lcl|NC_012224.1_cds_YP_002720166.1_74 [gene=ndhI] [locus_tag=JacuC_p075] [db_xref=GeneID:7564801] [protein=NADH dehydrogenase subunit I] [protein_id=YP_002720166.1] [location=complement(128386..128898)] [gbkey=CDS]
ATGTTTCCTATGGTAACTGGGTTCATGAATTATGGGCAACAAACCATGCAAGCTGCAAGGTACATTGGTCAAGGTTTCATGATTACCTTATCCCATGCAAACCGTTTACCTGTAACGATTCAATATCCTTATGAAAAATTAATCACATCGGAGCGTTTCCGCGGTCGAATCCATTTTGAATTTGATAAATGCATTGCTTGTGAAGTATGTGTTCGGGTATGCCCTATAGATCTACCTGTTGTTGATTGGCAATTGGAAACTGACATTCGAAAGAAACGGTTGCTTAATTACAGTATCGATTTTGGAATCTGTATATTTTGTGGCAACTGTGTTGAGTATTGTCCAACAAATTGTTTATCAATGACTGAAGAATATGAACTTTCTACTTATGATCGTCACGAATTGAATTATAATCAAATTGCTTTAGGTCGTTTACCAATGTCAGTAGTTGACGATTATACAATTCGAACAATTTTGAATTCAACTCAACAAAAAATCAACAAAAAAAAATAG

>lcl|NC_012224.1_cds_YP_002720098.1_6 [gene=atpF] [locus_tag=JacuC_p006] [db_xref=GeneID:7564834] [protein=ATP synthase CF0 B subunit] [protein_id=YP_002720098.1] [location=complement(join(12729..13082,13808..13966))] [gbkey=CDS]
ATGAAAAACGTAACCGATTCTTTCGTTTCCTTGGGTCACTGGCCATCCGCCGGGAGTTTCGGGTTTAATACCGATATTTTAGCAACAAATCCAATAAATCTAAGTGTAGTCCTTGGTGTATTAATTTTTTTTGGAAAGGGGGTGTGTGCGAGTTGTTTAGATAATCGAAAACAAAGGATTTTGGATACTATTCGAAATTCAGAAGAACTACGTGAGGGGGCCATTGAACGGCTGGAAAAAGCCCGGGCCCGCTTACGGAAAGTGGAAATAGAAGCAGATCAGTTTCGAATGAATGGATACTCTGAGATAGAACGAGAAAAATCGAATTTGATTAATTCAACTTCTAAGACTTTAGAACAATTAGAAAATTACAAAAACGAAACCATTCATTTTGAACAACAAAGAACGATTAATCAAGTCCGACAACGGGTTTTCCAACAAGCCTTACAAGGAGCTCTAGGAACTCTGAATAGTTCTTTGACCAACGAGTTACATTTACGTACCATCAATTAG

>lcl|NC_012224.1_cds_YP_002720157.1_65 [gene=ndhB] [locus_tag=JacuC_p065] [db_xref=GeneID:7564781] [protein=NADH dehydrogenase subunit 2] [protein_id=YP_002720157.1] [location=complement(join(102419..103168,103851..104627))] [gbkey=CDS]
ATGATCTGGCATGTACAGAATGAAAACTTCATTCTCGATTCTACGAGAATTTTTATGAAAGCCTTTCATTTGCTTCTCTTCGATGGAAGTTTTATTTTCCCAGAATGTATCCTAATTTTTGGCCTAATTCTTCTTCTGATGATCGATTCAACCTCTGATCAAAAAGATATACCTTGGTTATATTTCATCTCTTCAACAAGTTTAGTAATGAGTATAACGGCCCTATTGTTCCGATGGAGAGAAGAACCTATGATTAGCTTTTCGGGAAATTTCCAAACGAACAATTTCAACGAAATCTTTCAATTTCTTATTTTACTATGTTCCACTCTATGTATTCCTCTATCCGTAGAGTACATTGAATGTACAGAAATGGCTATAACAGAGTTTCTCTTATTCCTATTAACAGCTACTCTAGGAGGAATGTTTTTATGCGGTGCTAACGATTTAATAACTATCTTTGTAGCTCCAGAATGTTTCAGTTTATGCTCCTACCTATTATCTGGATATACCAAGAAAGATGTACGGTCTAATGAGGCTACTACGAAATATTTACTCATGGGTGGGGCAAGCTCTTCTATTCTGGTTCATGCTTTCTCTTGGCTATATGGTTCGTCCGGGGGAGAGATCGAGCTTCAAGAAATAGTGAATGGCCTTATCAATACACAAATGTATAACTCCCCAGGAATTTCAATTGCGCTTATATTCATCACTGTAGGAATCGGGTTCAAGCTTTCCCCAGCCCCTTCTCATCAATGGACTCCTGACGTATACGAAGGATCTCCCACTCCAGTCGTTGCTTTTCTTTCTGTTACTTCGAAAGTAGCTGCTTCAGCTTCAGCCACTCGAATTTTCGATATTCCTTTTTATTTCTCATCAAACGAATGGCATCTTCTTCTGGAAATCCTAGCTTTTCTGAGCATGATAGTGGGGAATCTCATTGCTATTACTCAAACAAGCATGAAACGTATGCTTGCATATTCGTCCATAGGTCAAATCGGATATGTAATTATTGGAATAATTGTTGGAGACTCAAATGGTGGATATGCAAGCATGATAACTTATATGCTCTTCTATATCTCCATGAATCTAGGAACTTTTGCTTGTATTGTATTATTTGGTCTACGTACCGGAACTGATAACATTCGAGATTATGCAGGATTATACACGAAAGATCCTTTTTTGGCTCTCTCTTTAGCCCTATGTCTCTTATCCCTAGGAGGTCTTCCTCCACTAGCAGGTTTTTTCGGAAAACTCCATTTATTCTGGTGTGGATGGCAGGCAGGCCTATATTTCTTGGTTTTAATAGGACTCCTTACGAGCGTTGTTTCTATCTACTATTATCTAAAAATAATCAAGTTATTAATGACTGGACGAAACCAAGAAAGAACCTCTCACGTGCGAAATTATAGAACTTTAAGATCAAACAATTCCATCGAATTGAGTATGATTGTATGTGTGATAGCATCTACTATACCGGGAATATCAATGAACCCGATTATTGAAATTGCTCAAGATACCCTTTTTTAG

>lcl|NC_012224.1_cds_YP_002720119.1_27 [gene=atpB] [locus_tag=JacuC_p027] [db_xref=GeneID:7564869] [protein=ATP synthase CF1 beta subunit] [protein_id=YP_002720119.1] [location=complement(57959..59446)] [gbkey=CDS]
ATGAGAATCAATCCTACTACTTCTGGTCCGGGAGTTCCCGCGCTTGAAAAAAAGAACCTGGGGCGTATCGCTCAAATCATTGGGCCAGTACTAGATGTAGCTTTTTCCCCGGGCAAGATGCCTAATATTTACAACGCTCTGGTAGTTAAGGGTCGAGATACTGTCGGGCAAGAAATTAATGTGACTTGTGAAGTACAACAATTATTAGGAAATAATCGAGTTCGGGCTGTAGCTATGAGTGCTACAGATGGTCTAACGCGAGGAATGGAAGTGATTGACACAGGAGCTCCTCTAAGTGTTCCAGTTGGTGGGGCGACTCTAGGACGAATTTTCAACGTGCTTGGAGAACCTGTTGATGATTTAGGTCCTGTAGATACTCGCGCAACATCACCTATTCATAGATCTGCACCTGCTTTTATACAATTAGATACAAAATTATCTATTTTTGAAACAGGAATTAAAGTAGTAGATCTTTTAGCCCCTTATCGCCGTGGAGGAAAAATCGGACTATTCGGGGGAGCTGGAGTGGGTAAAACAGTACTTATTATGGAATTAATCAACAACATCGCGAAAGCTCATGGAGGCGTATCCGTATTTGGCGGAGTAGGCGAACGAACTCGTGAAGGAAATGATCTTTACATGGAAATGAAAGAATCTGGAGTAATTAATCAAGAAAATATTGCAGAATCAAAAGTGGCTCTAGTCTATGGTCAGATGAACGAACCGCCGGGAGCTCGTATGAGAGTTGGTTTGACTGCCCTAACTATGGCGGAATATTTCCGAGATGTTAATGAACAAGACGTACTTCTATTTATCGACAATATCTTCCGTTTCGTCCAAGCAGGATCCGAAGTATCCGCCTTATTGGGTAGAATGCCTTCTGCTGTGGGTTATCAACCTACCCTTAGTACCGAAATGGGTTCTTTACAAGAAAGAATTACTTCCACCAAAGAGGGGTCCATAACTTCTATTCAAGCAGTTTATGTACCTGCGGACGATTTGACTGACCCTGCTCCTGCCACGACATTTGCACATTTAGATGCGACTACTGTACTATCAAGAGGATTAGCTGCTAAAGGTATCTATCCAGCAGTAGATCCTTTAGATTCAACATCAACTATGCTCCAACCTCAGATCGTTGGTGAGGAACATTATGAAACTGCGCAAAGAGTTAAGCAAACTTTACAACGTTACAAAGAACTTCAGGACATTATAGCTATCCTTGGGCTGGACGAATTATCCGAAGAGGATCGTTTAACTGTAGCAAGAGCACGAAAAATTGAGCGTTTCTTATCACAACCCTTTTTCGTAGCAGAAGTATTTACCGGTTCTCCGGGTAAATATGTCGGTCTAGCAGAAACTATTAGAGGGTTTAAATTGATCCTTTCCGGAGAATTAGATAGTCTCCCTGAACAGGCCTTTTATTTGGTAGGTAATATTGATGAAGCTACTGCGAAGGCTACAAACTTAGAAATGGAGAACAAATGA

>lcl|NC_012224.1_cds_YP_002720151.1_59 [gene=rpl22] [locus_tag=JacuC_p059] [db_xref=GeneID:7564773] [protein=ribosomal protein L22] [protein_id=YP_002720151.1] [location=complement(90862..91287)] [gbkey=CDS]
ATGATAAATAAAAGAAAGCGAAAGAGAGACCCATATACGGAAGTATATGCTTTAGGCCAACATATATGTATGTCCGCTCACAAAGCACGAAGAATAATTGATCAGATTCGTGGACGTTCTTATGAAGAAACACTTATGATACTTGAACTCATGCCCTATCGAGCATGTTATCCCATTTTTAAATTGATTTATTCTGCAGGAGCAAATGCTAGTCACAATATGGGTTTCAACGAAGCCAATTTAATCATTAGTAAAGCTGAAGTCAACGAAAGTGCTACTGTGAAAAAATTAAAACCTCAGGCTCGAGGGCGGGGTTATCTGATAAAAAGATCGACTTGTCATATAACTATTGTATTAAAAGATATATCCTTCTATGAAGAATATGAAGAATATGACAAATATCTAGGAAGAGTATTAAAAATATGA

>lcl|NC_012224.1_cds_YP_002720111.1_19 [gene=psaB] [locus_tag=JacuC_p019] [db_xref=GeneID:7564855] [protein=photosystem I P700 chlorophyll a apoprotein A2] [protein_id=YP_002720111.1] [location=complement(42314..44518)] [gbkey=CDS]
ATGGCATTAAGATTTCCAAGGTTTAGCCAAGGCTTAGCTCAGGACCCCACTACTCGTCGTATTTGGTTTGGTATTGCTACCGCACATGACTTCGAGAGTCATGATGATATTACGGAGGAACGGCTTTATCAGAATATTTTTGCTTCCCACTTCGGACAATTAGCAATAATTTTTCTGTGGACTTCCGGAAATCTCTTTCATGTAGCTTGGCAAGGAAATTTTGAAGCATGGGTACAGGACCCTTTACACGTAAGACCTATTGCTCATGCAATTTGGGATCCTCATTTTGGCCAACCGGCCGTGGAAGCTTTTACTCGAGGGGGCGCCCCTGGCCCAGTGAATATCGCTTATTCTGGTGTTTATCAATGGTGGTATACAATCGGTTTACGTACTAACGAAGATCTTTATATTGGAGCTCTTTTTCTATTATTTCTTTCTGCCCTAGCCTTATTAGGGGGTTGGTTACACCTACAACCGAAATGGAAACCGAGCGTTTCGTGGTTCAAAAACGCCGAATCTCGTCTCAATCATCATTTGTCGGGACTATTCGGAGTAAGCTCTTTAGCTTGGACAGGACATTTAGTCCATGTCGCTATTCCTGGTTCCAGGGGCGAATACGTTCGATGGAATAATTTCTTAGATGTATTACCCCATCCCCAAGGATTAGGCCCCCTTTTTACAGGTCAGTGGAATCTTTATGCTCAAAATCCCGATTCAGGTAGTCATTTATTTGGTACCTCCCAAGGATCAGGAACTGCCATTCTAACCCTTCTCGGGGGGTTCCATCCACAAACACAAAGTTTATGGCTGACCGATATTGCACACCATCATTTAGCTATTGCATTTCTTTTTCTCGTTGCCGGTCATATGTATAGAACTAACTTCGGGATTGGGCACAGTATAAAAGATCTTTTAGAAGCACATATTCCTCCCGGGGGGCGATTGGGGAGTGGACATAAGGGTCTTTATGACACAATCAACAATTCGCTTCATTTTCAATTAGGCCTTGCTCTAGCTTCTTTAGGGGTTATTACTTCCTTAGTAGCTCAACACATGTACTCATTACCTGCTTATGCATTCATAGCGCAAGACTTTACTACTCAAGCCGCGTTATATACTCATCACCAATACATCGCAGGATTCATCATGACAGGAGCTTTTGCTCATGGAGCTATATTTTTTATTAGAGATTACAATCCGGAACAGAACGAGAATAATGTATTGGCAAGAATGTTAGACCATAAAGAAGCTATCATATCCCATTTAAGTTGGGCTAGCCTCTTTCTGGGATTCCATACTTTGGGACTTTATGTTCATAATGATGTTATGCTTGCTTTTGGTACTCCGGAGAAACAAATCTTGATCGAACCCATATTTGCCCAATGGATACAATCTGCTCACGGTAAAACTTCATATGGATTCGATGTACTTTTATCTTCAACGAATAGTGCAGCCTTCAATGCAGGTCGAAGCATATGGTTGCCCGGCTGGTTAAATGCTGTTAATGAAAATAGTAATTCATTATTCTTAACAATAGGGCCTGGAGACTTCTTGGTTCATCATGCTATTGCTCTAGGTTTACATACAACCACATTAATCTTAGTAAAAGGTGCTTTAGATGCACGTGGTTCGAAGTTAATGCCAGATAAAAAGGATTTTGGTTATAGTTTTCCTTGCGATGGTCCGGGACGCGGTGGTACTTGTGATATTTCGGCTTGGGACGCATTTTATTTGGCGGTTTTCTGGATGTTAAATACCATTGGATGGGTTACTTTTTATTGGCATTGGAAGCACATCACATTATGGCAGGGTAATGTTTCACAGTTTAATGAATCTTCCACTTATTTGATGGGATGGTTAAGAGATTATCTATGGTTAAACTCTTCACAACTTATCAATGGATATAACCCTTTCGGTATGAATAGCTTATCGGTCTGGGCGTGGATGTTCTTATTTGGACATCTTGTTTGGGCTACTGGATTTATGTTTTTAATTTCTTGGCGTGGATATTGGCAGGAATTGATTGAAACTTTAGCGTGGGCTCATGAACGTACGCCTTTGGCTAATTTGATTCGATGGAGAGATAAACCAGTAGCTCTTTCCATTGTGCAAGCAAGATTGGTTGGATTAGCCCACTTTTCTGTAGGTTATATCTTCACTTATGCGGCTTTCTTGATTGCCTCTACATCAGGTAAATTTGGTTAA

>lcl|NC_012224.1_cds_YP_002720135.1_43 [gene=rpl20] [locus_tag=JacuC_p043] [db_xref=GeneID:7564757] [protein=ribosomal protein L20] [protein_id=YP_002720135.1] [location=complement(74821..75174)] [gbkey=CDS]
ATGACCAGAATTAGACGAGGATATATAGCTCGGAGGCGTAGAACAAAAATTCGTTTATTTGCATCAAGCTTTCGCGGGGCTCATTCAAGACTTACTCGAACTATTATTCAACAAAAAATAAGAGCTTTGATTTCGGCCCATCGGGATAGAGATAGGCAAAAAGGAAATTTTCGTCGTTTGTGGGTCACTCGGATAAATGCAGCAATTCGCGAGAATAAGGTATCCAAAAGTTATAGTAGATTAATAAACAATCTGTACAAGAGACAGTTGCTTCTTAATCGTAAAATACTTGCACAAATCGCTATAGTAAATAAGAATTGTCTTTATATGATTGCCAATGACATTATAAAATAA

>lcl|NC_012224.1_cds_YP_002720112.1_20 [gene=psaA] [locus_tag=JacuC_p020] [db_xref=GeneID:7564856] [protein=photosystem I P700 chlorophyll a apoprotein A1] [protein_id=YP_002720112.1] [location=complement(44544..46796)] [gbkey=CDS]
ATGATTATTCGTTCGCCGGAACCAGAAGTCAAAATTTTGGTAGATAGGGATCCCATCAAAACTTCTTTCGAGGAATGGGCCAGACCCGGTCATTTCTCAAGAACAATAGCTAAGGGACCTGATACTACCACTTGGATCTGGAACCTACATGCTGATGCTCACGATTTCGATAGCCATACCAGTGATTTGGAGGAGATTTCTCGAAAAGTATTTAGTGCTCATTTCGGCCAACTCTCCATCATCTTTCTTTGGCTGAGTGGCATGTATTTCCACGGTGCTCGTTTTTCCAATTATGAAGCATGGCTAAGTGATCCTACTCACATTGGACCTAGCGCCCAAGTGGTTTGGCCGATAGTGGGTCAAGAAATATTGAATGGTGATGTGGGGGGGGGGTTCCGAGGAATACAAATAACCTCCGGTTTTTTTCAGCTTTGGAGAGCATCTGGAATAACTAGTGAATTACAACTGTATTGTACTGCAATTGGTGCATTGGTCTTTGCAACTTTAATGCTTTTTGCTGGTTGGTTCCATTATCACAAAGCTGCTCCAAAATTGGCTTGGTTCCAAGATGTAGAATCTATGTTGAATCACCATTTAGCGGGACTACTAGGACTTGGGTCTCTTTCTTGGGCGGGACATCAAGTACATGTATCTTTACCAATTAACCAATTTCTAAACGCTGGGGTGGATCCTAAGGAAATCCCACTTCCTCATGAATTTATCTTGAATCGGGATCTTTTGGCTCAACTTTATCCCAGTTTTGCTGAGGGAGCAACCCCATTTTTCACCTTGAATTGGTCAAAATATTCGGAATTTCTTACTTTTCGTGGAGGATTAGATCCAGTGACTGGGGGTCTATGGCTGACCGATATTGCACACCATCATTTAGCTATTGCAATTCTTTTCCTGATAGCGGGTCACATGTATAAGACTAACTGGGGCATTGGTCATGGTATAAAAGATATTTTAGAGGCTCATAAAGGTCCATTTACAGGTCAGGGCCATAAGGGCCTATATGAGATCCTAACAACGTCATGGCATGCTCAATTATCTCTTAACCTAGCTATGTTAGGTTCTTTAACCATTGTTGTAGCTCACCATATGTATTCCATGCCCCCTTATCCATATTTAGCTACTGACTATGGTACACAACTGTCATTGTTCACACATCACATGTGGATTGGTGGATTTCTCATAGTTGGTGCTGCTGCACATGCAGCCATTTTTATGGTAAGAGACTATGATCCAACTACTCGATACAACGATCTATTAGATCGTGTTCTTAGGCATCGCGATGCAATCATATCACATCTCAACTGGGTATGTATTTTTTTAGGTTTTCATAGTTTTGGTTTATATATTCATAATGATACCATGAGCGCTTTAGGGCGCCCTCAAGATATGTTTTCCGATACTGCTATACAATTACAACCCGTCTTTGCTCAATGGATACAAAACACCCATGCTTTAGCACCTGGTGCAACGGCTCCTGGTGCAACAGCAAGCACCAGTTTGACTTGGGGGGGTGGTGATTTAGTGGCAGTGGGTGGCAAGGTTGCTTTGTTACCAATTCCATTAGGAACCGCAGATTTTTTGGTACATCACATTCATGCATTTACGATTCATGTGACGGTATTGATACTTCTGAAAGGAGTTCTATTTGCCCGTAGCTCTCGTTTGATACCGGATAAAGCAAATCTTGGTTTTCGTTTCCCTTGTGATGGGCCTGGAAGAGGGGGAACATGTCAAGTATCCGCTTGGGATCATGTCTTCTTAGGGTTATTTTGGATGTACAATTCCATTTCGGTTGTAATATTCCATTTCAGTTGGAAAATGCAGTCAGATGTTTGGGGTAGTATAAGTGATCAAGGGGTGGTAACTCATATCACGGGAGGAAACTTTGCACAGAGTTCCATTACTATTAATGGATGGCTCCGCGATTTCTTATGGGCACAGGCATCCCAGGTAATTCAGTCTTATGGTTCTTCATTATCTGCATATGGCCTTTTTTTCCTAGGTGCTCATTTTGTATGGGCTTTTAGTTTAATGTTTCTATTCAGTGGTCGTGGTTATTGGCAAGAACTTATTGAATCAATCGTTTGGGCTCATAATAAATTAAAAGTTGCTCCTGCTACTCAGCCTAGAGCCTTGAGCATTATACAAGGACGTGCTGTAGGAGTAACCCATTACCTTCTGGGTGGAATTGCCACAACATGGGCGTTCTTCTTAGCAAGAATTATTGCAGTAGGATAA

>lcl|NC_012224.1_cds_YP_002720155.1_63 [gene=ycf2] [locus_tag=JacuC_p063] [db_xref=GeneID:7564778] [protein=Ycf2] [protein_id=YP_002720155.1] [location=93852..100748] [gbkey=CDS]
ATGAAAGGACATCAATTCAAATCCTGGATTTTCGAATTGAGAGAGATATTGAGAGAGATCAAGAATTCTCACTATTTCTTAGATTCATGGACCCAATTCAATTCAGTGGGATCTTTCATTCACATTTTTTTCCATCAAGAACGTTTTATAAAACTCTTGGACTCCCGAATTTGGAGTATCTTACTTTCACGCAATTCACAGGGTTCAACAAGCAATCGATATTTCACGATCAAGGGTGTAGTACTATTTGTAGTAGTGGTTCTTATATATCGTATTAACAATCGAAAGATGGTCGAAAGAAAAAATCTCTATTTGACAGGGCTTCTTCCTATACCTATGAATTCCATTGGACCCAGAAATGATACATTGGAAGAATCCTTTGGGTCTTCCAATATCAATAGGTTGATTGTTTCGCTCCTGTATCTTCCAAAAGGAAAAAAGATCTCTGAGAGCTCTTTCCTGGATCCGAAAGAGAGTACTTGGGTTCTCCCAATAACTAAAAAGTCTGAATCTAACTGGGGTTCGCGGTGGTGGAGGAACTGGATCGGAAAAAAGAGGGATTCTAGTTGTAAGATATCTAATGAAACCGTCGCTGGAATTGAGATCTCATTCAAAGAAAAAGATATCAAATATCTGGAGTTTCTTTTTGTATATTATATGGATGATCCGATCCGCAAGGACCATGATTGGGAATTGTTTGATCGTCTTTCTCCGAGGAAGGGGCGAAACATAATCAACTTGAATTCGGGACAGCTATTCGAAATCTTAGTGAAAGACTGGATTTGTTATCTCATGTTTGCTTTTCGCGAAAAAATACCAATTGAAGTGGAGGGTTTCTTCAAACAACAAGGAGCTGGGTCAACTATTCAATCAAATGATATTGAGCATGTTTCCCATCTCTTCTCGAGAAAGAAGTGGGCTATTTCTTTGCAAAATTGTGCTCAATTTCATATGTGGCAATTCCGCCAAGATCTCTTCGTTAGTTGGGGGAATAATCCGCACGAATCGGATTTTTTGAGGAACATATCGAGAGAGGATTGGATTTGGTTAGACAATGTGTGGTTGGTAAACAAGGATCGGTTTTTTAGCAAGGCACGGAATATATCGTCAAATATTCAATATGATTCCACAAGATCTAGTTTCGTTCAAGGAAGGAATTCTAGCCAATTGAAGGGATCTTCTGATCAATCCAGAGATCATTTCGATTCCATTAGTAATGAGGATTCGGAATATCACACATTGATCAATCAAAGAAAGATTCAACAACTAAAAGAAAGATCGATTCTTTGGGATCCTTCCTTTCTTCAAACGGAACGAACAGAGATAGAATCAGACCGATTCCCTAAATGCCTTTCTGGATATTCCTCAATGTCCCGACTATTCACGGAAGGTGAGAAGGAGATGAATAATCATCTGCTTCCGGAAGAAATCGAAGAATTTCTTGGGAATCCTACAAGATCCATTCGTTCTTTTTTCTCTGACAGATCGTCAGAACTTCATCTGGGTTCGAATCCTACTGAGAGGTCCACTAAATTGTTGAAGAAAGAACAAGATGTTTCTTTTGTCCCTTCCAGGCGATCGGAAAATAAAGAAATAGTTAATATATTCAAGATAATCACGTATTTACAAAATACCGTCTCAATTCATCCTATTTCATCAGATCCGGGATGTGATATGGTTCTGAAGGATGAACTGGATATGGACAGTTCCAATAAGATTTCTTTCTTGAACAAAAATCCATTTTTTGATTTATTTCATCTATTCCATGATCGGAACGGGGGGGGATACACGTTACACCACGATTTTGAATCAGAAGAGAGATTTCAAGAAATGGCAGATCTATTCACTCTATCAATAACCGAGCCGGATCTGGTGTATCATAAGGGATTTACCTTTTTTATTGATTCTTACGGATTGGATCAAAAACAATTCTTGAATGAGGTATTCAACTCCAGGGATGAATCGAAAAAGAAATCTTTATTGGTTCTACCTCCTATTTTTTATGAAGAGAATGAATCTTTTTATCGAAGGATCAGAAAAAAATGGGTCCGGATCTCCTGCGGGAATGATTTGGAAGATCCAAAACAAAAAATAGTGGTATTTGCTAGCAACAACATAATGGAGGCAGTCAATCAATATGGATTGATCCTAAATCTGATTCAAATCCAATATAGTACCTATGGGTACATAAGAAATGTATTGACTCAATTCTTTTTAATGAATAGATCCGATCGCAACTTCGAATATGGAATTCAAAGGGATCAAATAGGAAATGATACTCTGAATCATAGAACTATAATGAAATATACGATCAACCAACATTTATCGAATTTGAAACAGAGTCAGAAGAACTGGTTCGATCCTCTTATTTTTCTTTCTCGAACCGAGAGATCCATGAATTGGGATCCTAATGCATATAGATACAAATGGTCTAATGGGAGCAAGAATTTCCAGGAATATTTGGAACATTTCATTTCTGAGCAGAAGAGCCGTTTTCTTTTTCAAGTAGTGTTCGATCGATTACGTATTAATCAATATTCGATTGATTGGTCTGAGGTTATCGACAAAAAAGATTTGTCTAAGTCACTTCGTTTCTTTTTGTCCAAGTTACTTGTTTTTTTGTCCAAGTTTCTTCTCTTTTTGTCTAACTCACTTCCTTTTTTCTTTGTGAGTTTCGGGAATATCCCCATTCATAGGTCCGAAATCCATATCTATGAATTGAAAGGTCCGAATGATCCACTCTGCAATCAGCTGTTAGAACCAATAGGTCTTCAAATCGTTCATTTGAAAAAATGGAAACCCTTCTTATTGGATGATCATGATACTTCCCAAAAATCGAAATTTTTGATTAATGGAGGAACAATATCACCATTTTTGTTCAATAAGATACCAAAGTGGATGATTGACTCATTCCATACTAGAAATAATCGCAGGAAATCTTTTGATAACACGGATTCCTATTTCTCAATGATATCCCACGATCAAGACAATTGGCTGAATCCCGTGAAACCATTTCATAGAAGTTCATTGATATCTTCTTTTTATAAAACAAATCGACTTCGATTCTTGAATAATCTACATCACTTCTGCTTCTATTGTAACAAAAACAAAAGATTCCCTTTTTATGTGGAAAAGGCCCGTATCAAGAATTATGATTTTACGTATGGACAATTCCTCAATATCTTGTTCATTCGCAACAAAATATTTTCTTTGTGCGGCGGTAAAAAAAAACATGCTTTTGGGGAGAGAGATACTATTTCACCAATCGAGTCACAGGTATCTAACATATTCATACCTAATGATTTTCCACAAAGTGGTAACGAAAGGTATAACTTGTACAAATCTTTCCATTTTCCAATTCGATCCGATCCATTCGTTCGTAGAGCTATTTATTTGATCGCAGACATTTCGGGAACACCTCTAACAGAGGGACAAATAGTCAATTTTGAAAGAACTTATTGTCAACCTCTTTCGGATATGAATCTATCTGATTCAGAAGGGAAGAACTTGCATCAGTATCTCAATTTCAATTCAAACATGGGTTTGATTCACACTCCATGTTCTGAGAAATATTTACCATCCGAAAAGAGGAAAAAACGGAGTCTTTGTCTAAAGAAATGTGTTGAAAAAGGGCAGATGTATAGAACCTTTCAACGAGATAGTGCTTTTTCAACTCTCTCAAAATGGAATCTATTCCAAACATATATGCCATGGTTCCTTACTTCGACAGGGTACAAATATCTAAATTTGATATTTTTAGATACCTTTTCGGACCTATTACCGATACTAAGTAGCAGTCAAAAATTTGTATCCATTTTTCATGATATTATGCACGGATCAGATATATCATGGCGAATTCTTCAGAAAAAATGGTGTCTTCCACAATGGAATCTGATAAGTGAGATTTCGAGTAAGTGTTTACATAATCTTCTTCTGTCCGAAGAAATGATTCATCGAAATAATGAGCCACCATTGATATCGACACATCTGAGATCGCCAAATGTTCGGGAGTTCCTCTATTCAATCCTTTTCCTTCTTCTTGTTGCTGGATATCTCGTTCGTACACATCTTCTCTTTGTTTCCCGAGCCTATAGTGAGTTACAGACAGAGTTCGAAAAGGTCAAATCTTTGATGATTCCATCATACATGATTGAGTTGCGAAAACTTCTGGATAGGTATCCTACATCTGAACTGAATTCTTTCTGGTTAAAGAATCTCTTTCTAGTTGCTCTGGAACAATTAGGAGATTTTCTAGAAGAAATGCGGGGTTTTGCTTCTGGCGGCAACATGCTATGGGGTGGTGGTCCCGCTTATGGGGTTAAATCAATACGTTCTAAGAATAAATTTTTGAATATCAATCTCATCGATCTCATAAGTATCATACCAAATCCCATCAATCGAATCACTTTTTCGAGAAATACGAGACATCTAAGTCATACAAGTAAAGAGATTTATTCATTGATAAGAAAAAGAAAAAACGTGAATGGTGATTGGATTGATGATAAAATAGAATCCTTGGTCGCGAACAGTGATTCGATTGATGATAAAGAAAGAGAATTCTTGGTTCAGTTCTCCACCTTAACGACAGAAAACAGGATTGATCAAATTCTATTGAGTCTGACGCATAGTGATCATTTATCAAAGAATGACTCTGGTTATCAAATGATTGAAGAGCCGGGAGCAATTTATTTACGATACTTAGTTGACATTCATAAAAAGTATCTAATGAATTATGAGTTCAATACACCCTGTTTAGCAGAAAGACGGATATTCCTTGCTTATTATCAGACAACCACTTATTCACAAACCTCGTGTGGGGTGAATAGTTTTCATTTCCCATCTCATGGAAAACCCTTTTCGCTCCGCTTAGCCCTATCCCCCTCTAGGGGTATTTTAGTGATAGGTTCTATAGGAACTGGACGATCCTATTTGGTCAAATACCTAGCGACAAACTCCTATCTTCCTTTCATTACAGTATTTCTGAACAAGTTCCTGGATAACAAGCCTAAGGGCTTTCTTATTGATGATAGTGACGATATTGATGATAGTGACGATATTGATGATAGTGACGATATTGATGATAGTGACGATATTGATGTGAGTGACGATATTGATGATAGTGACGATATCGACCGTGACTTTGATACGGAGCTGGAGTTTCTAACTAGGATGAATGCGCTAACTATGGGTATGATGCCGGAAATAGACCGATTTTATATCACCCTTCAATTCGAATTAGCAAAAGCAATGTCTCCTTGCATAATATGGATTCCAAACATTCATGATTTGGATGTGAATGAGTCGAATTACTTATCCCTCGGTCTATTAGTGAACTATCTCTCCAGGGATTGTGAAAGATGTTCCACTAGAAATATTCTTGTTATTGCTTCGACTCATATTCCCCAAAAAGTGGATCCCGCTCTAATAGCTCCGAATAAATTAAATACATGCATTAAGATACGAAGGCTTCTTATTCCACAACAACGAAAGCACTTTTTTACTCTTTCATATACTAGGGGATTTCACTTGGAAAAGAAAATGCTCCATACTAATGGATTCGGGTCCATAACCATGGGTTCCAATGTACGAGATCTTGTAGCACTTACCAATGAGGCCCTATCGATTAGTATTACACAGAAGAAATCAATTCTAGACACTAATATAATTAGATCCGCTCTTCATAGACAAACTTGGGATTTGCGATCCCAGGTAAGATCGGTTCAGGATCATGGGATCCTTTTCTATCAGATAGGAAGGGCTGTTGCACAAAATGTATTTCTAAGTAATTGCCCCATAGATCCTATATCTATCTATATGAAGAAGAAATCATGTAACGAAGGGGATTCTTATTTGTACAAATGGTACTTCGAACTTGGAATGAGCATGAAGAAATTAACGATACTTCTTTATCTTTTGAGTTGTTCCGCCGGATCGGTTGCTCAAGACCTTTGGTCTCTACCCGGACCCGATGAAAAAAATGGGATCACTTATTATGGACTTGTTGAGAATGATTCTGATCTAGTTCATGGCCTATTAGAAGTCGAAGGCGCTCTGGTGGGATCCTCACGTACAGAAAAAGATTGCAGTCAGTTTGATAATGATCGAGTGACATTGCTTCTTCGGCCCGAACCAAAGAGTCCCTTAGATATGATGCAAAATGGATCTTGTTCTATCCTTGATCAGAGATTTCTCTATGAAAAATACGAATCGGAGTTTGAAGAAGGGGAAGGAGTCCTCGACCCGCAACAGATAGAGGAGGATTTATTCAATCACATAGTTTGGGCTCCTAGAATATGGCGCCCTTGGGGTTTTCTATTTGATTGTATCGAAAGGCCCAATGAATTGGGATTTCCCTATTGGGCCAGGTCATTTCGGGGCAAGCGGATCATTTATGATGAAGAGGATGAGCTTCAAGAGAATGATTCGGAGTTCTTGCAGAGTGGAACCATGCAGTACCAGATACGAGATAGATCTTCCAAAGAACAAGGCTTTTTTCGAATAAGCCAATTCATTTGGGACCCTGCGGATCCACTCTTTTTCCTATTCAAAGATCAGCCCTTTGTCTCTGTGTTTTCACATCGAGAATTCTTTGCAGATGAAGAGATGTCAAAGGGGCTTCTTACTTCCCAAACAGATCCTCCTACATCTATATATAAACGCTGGTTTATCAAGAATACGCAAGAAAAGCACTTCGAATTGTTGATTCATCGCCAGAGATGGCTTAGAACCAATAGTTCATTATCTAATGGATTTTTCCGTTCTAATACTCTATCCGAGAGTTATCAGTATTTATCAAATCTGTTCCTATCTAACGGAACGCTATTGGATCAAATGACAAAGGCATTGTTGAGAAAAAGATGGCTTTTCCCGGATGAAATGAAAATTGGATTCATGTAA

>lcl|NC_012224.1_cds_YP_002720124.1_32 [gene=cemA] [locus_tag=JacuC_p032] [db_xref=GeneID:7564874] [protein=envelope membrane protein] [protein_id=YP_002720124.1] [location=66653..67342] [gbkey=CDS]
ATGAAAAGAAAAGCATTTATTCCCCTTCTATATCTTACATCTATAGTTTTTTTGCCCTGGTGGATCTCTTTTTTTTCTTTTAATAAAAGTTTTGAATCTTGGGTTATTAATTGGTGTAATACTAGTAAATCCGAAACTTTTTTAAATGATATCCAAGAAAAAAGTATTCTAGAAAAATTCATAGAATTAGAGGAACTCGTTCGCTTGGACGAAATGATAAAGGAATACCCGGAAACACATCTACAAGGGTTTCGTACCGGAATCCACAAAGAAACGATCCAATTGATCAAGATGCACAATGAAGATCGTATCCATACGATTTTACACTTCTCGACAAATATAATCTGTTTCGTTATTCTAAGTGGGTATTCTATTCTAAGTAATGAAGAACTTATTATTCTTAATTCGTGGGTTCAAGAATTCCTATATAACTTAAGCGACACAATAAAAGCTTTTTCAATTCTTTTATTAACCGATTTATGTATAGGATTCCACTCACCCCACGGTTGGGAACTAATGATTGGCTCTGTCTACAAAGATTTTGGATTTGCTCATAATGATCAAATTATATCTGGCCTTGTTTCCACTTTTCCAGTCATTCTCGATACAATTTTTAAATATTGGATTTTCCGTTATTTAAATCGTGTATCTCCGTCACTTGTAGTGATTTATCATTCAATGAATGACTGA

>lcl|NC_012224.1_cds_YP_002720136.1_44 [gene=rps12] [locus_tag=JacuC_p044] [db_xref=GeneID:7564758] [protein=ribosomal protein S12] [exception=trans-splicing] [protein_id=YP_002720136.1] [location=join(complement(75975..76088),149328..149570)] [gbkey=CDS]
ATGCCAACAATTAAACAACTTATTAGAAACACAAGACAGCCAATCAGAAATGTCACTAAATCCCCCGCTCTTGGGGGATGTCCTCAGCGCCGAGGAACATGTACTAGGGTGTATACTATCACCCCCAAAAAACCAAACTCTGCCTTACGTAAAGTTGCCAGAGTACGATTAACCTCTGGATTTGAAATCACTGCTTATATACCTGGTATTGGCCATAATTCACAAGAACATTCTGTAGTCTTAGTAAGAGGGGGAAGGGTTAAGGATTTACCCGGTGTGAGATATCACATTGTTCGAGGAACCCTAGATGCTGTCGGAGTAAAGGATCGTCAACAAGGGCGTTCTAGTGCGTTGTAG

>lcl|NC_012224.1_cds_YP_002720134.1_42 [gene=rps18] [locus_tag=JacuC_p042] [db_xref=GeneID:7564756] [protein=ribosomal protein S18] [protein_id=YP_002720134.1] [location=74204..74530] [gbkey=CDS]
ATGGATAAATCCAAACGACTTTTTCTTAAGTCCAAGCGATCTTTTCGTAGGCGTTTGCCCCCGATCCAATCGGGGGATCGAATTGATTATAGAAACATGAGTTTAATTAGTCGATTTATTAGTGAACAAGGAAAAATATTATCTAGACGGGTGAATAGATTGAGTTTAAAACAACAACGATTAATTACTATTGCTATAAAGCAAGCTCGTATTTTATCTTCGTTACCTTTTCTTAATAATGAAAAACAGTTTGAAAAAAGCGAGTTGGTCACTATAACTACTGATCTTAGAACCAGAAAAAAAAAAATAGACTTACTCCTCAATTGA

>lcl|NC_012224.1_cds_YP_002720121.1_29 [gene=accD] [locus_tag=JacuC_p029] [db_xref=GeneID:7564871] [protein=acetyl-CoA carboxylase beta subunit] [protein_id=YP_002720121.1] [location=62443..63924] [gbkey=CDS]
ATGGAAAAACGGTGGTTCAATTCGATCTTATCCAATGTAGAATTAGGATACAGGTGTAGGTTAAGTAAATCAATGGATAGTTTTAGTCCTCTTGAAAATACCAGTATAAGCGAAGACCCAATTCTAAACGATACAGATAAAAACACCCATTGTTGGAGTAATAGTGACAGCTCTAGTTACAGTAATGTTGATCATTTAGTTGGCATTCGGAATTTCAGCGTTGATGAAACTTTTTTAGTTAGGGATAGTAATAGGGACAGTTATTCCATATATTTTGATATTGAAAATCAAGTTTTTGAGATTGAAACTGATCATTCTTTTCTGAATGAACTAGAAAGTTCTTTTTATAGTTATTGGAATTCGAGTTATCTGAATAATGGGTCTAGCAGTGGTGACTTCCACTATGATCATTATATGTCTGATACTAAATATAGTTGGAATAATTACATCAATAGTTGTATTGACAATTATCTTCGCTCTCAAATCTGTATTGATTTAAGTGGTAGTAACAATTACAGTGAGAGTTACATTTATAGTTACATTTGTGGTGAAAGTGGAAATAGTAGTGAAAGTGAGAGTTCCAGTCTAAGAACTAGCACGAATGGTAGCGATTTAACTATAAGAGAAAGTTCTAATGATCTCGATATAACTCAAAAATACAAGCATTTGTGGGTTCAATGCGAAAATTGTTATGGATTAAACTATAAGAAATTTTTGAAGTCAAGAATGAATATTTGTGAACAATGTGGATATCATTTGAAAATGAGTAGTTCAGATAGAATTGAACTTTCGATTGACCTAGGCACTTGGAATCCTATGGATGAAGACATGGTATCTCTGGATCCCATTGAATTTCATTCAGAAGAGGAACCTTATAAAGATCGTATTGATTCTTATCAAAAAAAGACAGGATTAACCGAGGCTGTTCAAACAGGCACAGGTCAACTAAACGGTATTCCCGTAGCAATTGGGGTTATGGATTTTCAGTTTATGGGGGGTAGTATGGGATCCGTAGTAGGGGAAAAAATCACCCGTTTGATTGAGTATGCTACCAATAAATTTTTACCTCTTATTTTAGTGTGTGCTTCCGGAGGAGCACGCATGCAAGAAGGAAGTTTGAGCTTGATGCAAATGGCTAAAATATCTTCTGCTTTATATGATTATCAATCGAATAAAAAGTTATTTTATGTATCAATCCTTACATCTCCTACGACCGGCGGGGTGACAGCTAGTTTTGGTATGTTGGGGGATATCATTATTGCTGAACCTAACGCCTATATTGCATTTGCGGGTAAAAGAGTAATTGAACAAACATTGAATAAGACAGTACCTGAAGGTTCACAATCGGCTGAATTTTTATTCCATAAGGGTTTATTCGATCCAATCGTACCACGTAATCCTTTAAAAGGCGTTTTGAATGAGTTACTTCAGCTTCACGATTTCTTTCCTTTGAATCATAAATCAAGTAGAGCCTTAAGTTAA

>lcl|NC_012224.1_cds_YP_002720115.1_23 [gene=ndhJ] [locus_tag=JacuC_p023] [db_xref=GeneID:7564863] [protein=NADH dehydrogenase subunit J] [protein_id=YP_002720115.1] [location=complement(53707..54183)] [gbkey=CDS]
ATGCAGGGTCGTTTGTCTGCTTGGCTAGTCAAACATGGGCTAGTTCATAGATCTTTGGGTTTTGATTACCAAGGAATAGAGACTTTACAAATAAAGCCCGAAGATTGGCATTCCATTGCTGTCATTTTATATGTATATGGTTACAATTATCTGCGTTCGCAATGTGCCTATGATGTAGCACCGGGCGGGCTGTTAGCTAGTGTATATCATCTTACGAGAATAGAGTATGGTATAGATCAACCAGAAGAAGTATGTATAAAAGTATTTGCCCCAAGGAAGAATCCTAGAATTCCGTCTGTTTTCTGGGTTTGGAAAAGTGCGGATTTTCAAGAAAGGGAATCTTATGATATGCTGGGAATCTTTTATGATAATCATCCGCGTCTGAAACGTATCTTAATGCCGGAAAGTTGGGTAGGGTGGCCCTTACGTAAAGATTATATTGCTCCCAATTTTTATGAAATACAAGACGCTCATTGA

>lcl|NC_012224.1_cds_YP_002720171.1_79 [gene=rps7] [locus_tag=JacuC_p080] [db_xref=GeneID:7564815] [protein=ribosomal protein S7] [protein_id=YP_002720171.1] [location=150189..150656] [gbkey=CDS]
ATGTCACGTCGAGGTACTGCAGAAGAAAAAACTGCAAAATCCGATCCAATTTATCGTAATCGATTAGTTAACATGTTGGTTAACCGTATTCTGAAACACGGAAAAAAATCATTGGCTTATCAAATTATCTATCGAGCCATGAAAAAGATTCAACAAAAGACAGAAACAAATCCACTATCTGTTTTACGTCAAGCAATACGTGGAGTAACTCCCGATATAGCAGTAAAAGCAAGGCGTGTAGGCGGATCGACTCATCAAGTTCCCATTGAAATAGGATCCACACAAGGAAAAGCACTTGCCATTCGTTGGTTATTAGGGGCATCCCGAAAACGTCCGGGTCGAAATATGGCTTTCAAATTAAGTTCCGAATTAGTGGATGCTGCAAAAGGGAGTGGTGATGCCATACGCAAAAAGGAAGAGACTCATAGAATGGCAGAGGCAAATAGAGCTTTTGCACATTTTCGTTAA

>lcl|NC_012224.1_cds_YP_002720093.1_1 [gene=psbA] [locus_tag=JacuC_p001] [db_xref=GeneID:7564824] [protein=photosystem II protein D1] [protein_id=YP_002720093.1] [location=complement(499..1560)] [gbkey=CDS]
ATGACTGCAATTTTAGAGAGACGCGAAAGCGAAAGCCTATGGGGTCGTTTCTGTAACTGGATAACCAGCACTGAAAACCGTCTTTACATTGGATGGTTTGGTGTTTTGATGATCCCTACTTTATTGACCGCAACTTCTGTATTTATTATCGCTTTCATTGCTGCACCTCCGGTAGATATTGATGGTATTCGTGAACCTGTTTCTGGGTCTCTACTTTATGGAAACAATATTATTTCTGGTGCCATTATTCCTACTTCTGCGGCTATAGGTTTGCATTTTTACCCAATATGGGAAGCGGCATCCGTTGATGAATGGTTATACAATGGCGGTCCTTATGAGCTAATTGTTCTACATTTCTTACTTGGTGTAGCTTGTTACATGGGTCGTGAGTGGGAACTTAGTTTCCGTCTGGGTATGCGCCCTTGGATTGCTGTTGCATATTCAGCTCCTGTTGCAGCTGCTACTGCTGTTTTCTTGATCTATCCAATTGGTCAAGGAAGCTTTTCTGATGGTATGCCTCTAGGAATCTCTGGTACTTTCAACTTTATGATTGTATTCCAGGCTGAGCACAACATCCTTATGCACCCATTTCACATGTTAGGCGTAGCTGGTGTATTCGGCGGTTCCCTATTCAGTGCTATGCATGGTTCCTTGGTAACCTCTAGTTTGATCAGGGAAACCACAGAAAATGAATCTGCTAATGAAGGTTACAGATTCGGTCAAGAGGAAGAAACTTATAATATCGTAGCCGCTCATGGTTATTTTGGCCGATTGATCTTCCAATATGCTAGTTTCAACAATTCTCGTTCTTTACATTTCTTCTTAGCTGCTTGGCCTGTAGTAGGTATTTGGTTCACTGCTTTAGGTATTAGCACTATGGCTTTCAACCTAAATGGTTTCAATTTCAACCAATCCGTAGTTGATAGTCAAGGTCGTGTAATTAATACCTGGGCTGATATTATTAACCGTGCTAACCTTGGTATGGAAGTTATGCATGAACGTAATGCTCATAACTTCCCTCTAGACCTAGCTGCTATTGAAGCTCCATCTACAAATGGATAA

>lcl|NC_012224.1_cds_YP_002720123.1_31 [gene=ycf4] [locus_tag=JacuC_p031] [db_xref=GeneID:7564873] [protein=photosystem I assembly protein Ycf4] [protein_id=YP_002720123.1] [location=65493..66047] [gbkey=CDS]
ATGAGTTGGCGATCAGAACGTATATGGATAGAACTTATAGCGGGGTCTCGAAAAACAAGTAATTTCTGCTGGGCCCTTATACTTTTTTTAGGTTCATTGGGTTTTTTTTTGGTTGGAATTTCCAGTTATCTTGGCAAAAATTTGATATCTTTATTTCCATCTCAGCAAATAATTTTTTTTCCACAAGGGATCGTGATGTCTTTCTATGGGATCGCCGGTCTATTTATTAGTTCTTATTTGTGGTGCACAATTTTGTGGAATGTAGGTAGTGGTTATGATCGATTCGATAGAAAAGAAGGAATAGTGTGTATTTTTCGCTGGGGATTTCCTGGAAAAAATCGTCGCATCTTACTCCGATTCCTTATGAAAGATATTCAGTCTATTAGGCTAGAAGTTAAAGAGGGTATTTACGCTCGGCGTGCCCTTTATATGGAAATCCGAGGCCGGGGGGCCATTCCTTTGACTCGTACTGATGAGAATTTGACTCCGCGAGAAATTGAGCAAAAAGTAGCCGAATTGGCCTATTTTTTGCGTGTACCAATTGAAGTATTTTGA

>lcl|NC_012224.1_cds_YP_002720168.1_76 [gene=ndhH] [locus_tag=JacuC_p077] [db_xref=GeneID:7564803] [protein=NADH dehydrogenase subunit 7] [protein_id=YP_002720168.1] [location=complement(131208..132389)] [gbkey=CDS]
ATGAATGTACCAGCTATGCGAAAAGACCTTATGATAGTCAATATGGGTCCCCACCACCCATCAATGCATGGTGTTCTTCGACTCATCGTTACTCTAGATGGCGAAGATGTTATTGACTGTGAACCAATATTAGGTTATTTACACAGAGGCATGGAAAAAATTGCGGAAAATCGAACAATTATACAATATTTGCCCTATGTAACACGTTGGGATTATTTGGCTACTATGTTCACAGAAGCAATAACAGTAAATGGCCCAGAACTGTTAGGAAATATTCAAGTGCCTAAAAGAGCTAGCTATATCAGGGTAATTATGTTGGAATTGAGTCGTATAGCTTCTCATTTATTATGGCTTGGCCCTTTTATGGCGGATATTGGGGCACAGACTCCTTTCTTCTATATTTTTAGAGAGAGAGAGTTAGTATATGATTTATTCGAAGCTGCCACTGGTATGAGAATGATGCATAATTATTTTCGTATTGGGGGAGTAGCGGCTGATCTACCTCATGGCTGGATAGATAAATGTTTGGATTTTTGTGATTATTTTTTAACAAGAGTTGCTGAATATCAAAAACTTATTACGCGAAATCCTATTTTTTTAGAACGAGTTGAAGGAGTTGGTATTGTTGGTACAGAGGAAGCAATAAATTGGGGGTTATCAGGACCAATGCTACGAGCTTCCGGAGTACAATGGGATCTTCGTAAAATGGATCGTTATGAGTGTTACGACGAATTTGATTGGGAAGTCCAGTGGCAAAAAGAAGGGGATTCATTAGCTCGTTATTTAGTCCGAATTGGTGAAACGATGGAATCTATAAAAATTATTCAACAGGCTCTGGAAGGAATTCCGGGGGGGCCCTATGAAAATTTAGAAACCCGACGTTTTGATAAAGAAGGGGATCCAGAATGGAACGATTTCGAATATCGATTCATTAGTAAAAGAACTTCTCCTACTTTTGAATTACCGAAACAAGAACTTTATGTGAGAGTCGAAGCTCCAAAAGGAGAATTGGGAATTTTTCTGATAGGGGATCAGAGCGGTTTTCCTTGGAGATGGAAAATTCGCCCCCCGGGTTTTATCAATTTGCAAATTCTTCCTCAATTAGTTAAAAGAATGAAATTGGCTGATATTATGACAATACTAGGTAGTATAGATATCATTATGGGAGAAGTTGATCGTTGA

>lcl|NC_012224.1_cds_YP_002720167.1_75 [gene=ndhA] [locus_tag=JacuC_p076] [db_xref=GeneID:7564802] [protein=NADH dehydrogenase subunit 1] [protein_id=YP_002720167.1] [location=complement(join(128980..129519,130655..131206))] [gbkey=CDS]
ATGATAATTGATACAACAGAAGTACAAGCTATCAATTCTTTTTCTAGATTAGAATCCTTAAACGAGGTCTATGGACTTCTATGGGCCTTTGTCCCTATTTTGATTCTTGTATTTGGAATCACGATAGGCATACTAGTAATTGTATGGTTAGAAAGAGAAATATCTGCAGGGATACAACAACGTATTGGACCTGAATATGCCGGTCCTTTAGGGGTTCTTCAAGCTCTAGCGGATGGGACAAAACTTCTTTTCAAAGAGAATCTTTTTCCATCTAGGGGAGATACTCGTTTATTCAGTATCGGACCATCCATAGCAGTCATATCAACTTTATTAAGCTATTCAGTAATTCCTTTTGGCTATCACTTTGTTTTAACTGATCTAAATATTGGTGTTTTTTTATGGATTGCCATTTCAAGTATTGCTCCCATTGGACTTCTTATGTCAGGATATGGATCCAATAATAAATATTCTTTTTTAGGTGGTCTACGAGCTGCTGCTCAATCGATTAGTTATGAAATACCATTAAGTCTTTGTGTGTTATCCATATCTCTATTATCTAACAGTTCAAGTACCGTTGATATAGTTGAGGCACAATCAAAATACGGTTTTTGGGGGTGGAATTTGTGGCGTCAACCTATAGGATTTATCATTTTTTTTATTTCTTCTCTAGCAGAGTGTGAAAGATTGCCTTTTGATTTACCAGAAGCAGAAGAAGAATTAGTAGCAGGCTATCAAACCGAATATTCTGGTATAAAATTTGGTTTATTTTATATTGCTTCCTATCTAAACTTATTAGTTTCTTCATTATTTGTAACAGTTCTTTACTTGGGTGGTTGGAATATCCCTATTTCGTATCTATTCGTTCCTGAGCTTTTTCAAATAAATAAAATAGGTGGAGTCTTTGAAACAACAATAGGTATCTTTATTACATTGGTTAAAACTTATTTGTTCTTGTTCATTCCTATTACAACAAGATGGACTTTACCTAGACTAAGAATGGACCAACTTTTAAATCTTGGATGGAAATTTCTTTTACCTATTTCTCTCGGTAATTTATTATTAACAACCTCTTTCCAACTCCTTTCACTATAA

>lcl|NC_012224.1_cds_YP_002720102.1_10 [gene=rpoC2] [locus_tag=JacuC_p010] [db_xref=GeneID:7564838] [protein=RNA polymerase beta'' subunit] [protein_id=YP_002720102.1] [location=complement(18069..22253)] [gbkey=CDS]
ATGGAGGTGCTTATGGTCGAACGGGCCAGTCTGGTCTTTCACAATAAAGTGATAGATGGAACTGCCATTAAACGACTTATTAGCAGATTAATAGATCATTTTGGAATGGCATATACATCACACATCCTGGATCAAGTAAAGACTCTGGGTTTCCAGCAAGCCACTGCTACATCCATTTCATTAGGAATTGATGATCTTTTAACAATACCTTCTAAGGGATGGCTAGTCCAAGATGCTGAACAACAAAGTTTGATTTTGGAAAAACATTATCATTATGGAAATGTACACGTGGTAGAAAAATTACGCCAATCTATTGAGGTATGGTATGCTACAAGTGAATATTTGCGACAAGAAATGAATCTTAATTTTAGGATGACGGAACCCTTTAATCCAGTTCATATAATGTCTTTTTCGGGAGCTAGGGGAAATGCATCTCAAGTACACCAATTAGTAGGTATGAGAGGATTAATGTCGGATCCACAAGGACAAATGATTGATTTACCCATTCAAAGCAATTTACGTGAAGGACTGTCTTTAACAGAATATATCATTTCTTGCTATGGAGCCCGAAAAGGGGTTGTCGATACTGCTGTACGAACATCAGATGCTGGATATCTTACACGTAGACTTGTTGAAGTAGTTCAACATATTGTTGTGCGTAGAACAGATTGTGGCACCACCCGAGGGATCTCCGTGAGTCCTCGAAATGGGACGATGTCGGAAAGGATTTTTATTCAAACATTAATTGGTCGTGTATTAGCAGACAATATATATATGGGTTTACGATGCATTGCCGTTCAAAATCAAGATATTGGGATTGGACTTGCCAATCGATTCATAACCTTTCGAACACAAACAATATCTATTCGAACTCCCTTTACTTGTAGGAGTATGTCTTGGATCTGTCGATTATGTTATGGTCGGAGTCCTACTCATGGCGATCTAGTGGAATTGGGAGAAGCCGTAGGTATTATTGCAGGTCAATCCATTGGAGAGCCGGGTACTCAACTAACATTAAGAACGTTTCATACCGGCGGAGTATTCACAGGGGGTACTGCAGAACATGTGCGAGCCCCCTCTAATGGAAAAATCAAATTTAATGAGGATTTTGTTCATCCCATACGTACACGTCATGGGCATCCTGCTTTTCTATGTTATATAGACTTGTATGTAACTATTGAGAGTCAAGATATTATACATAACGTGACTATTCCACCAAAAAGTTTCCTTTTAGTTCAAAATGATCAATATGTAGAATCAGAACAAGTGATTGCTGAAATTCGGGCGGGAACATACACTTTGAATTTTAAAGAGAAGGTTCGAAAACATATTTATTCCGATTCAGAAGGGGAAATGCACTGGAGTTCTGATGTATACCACGCACCTGAATTTACATATAGTAATGTCCATCTCTTACCAAAAACAAGCCATTTATGGATATTATCGGGAAGTTCGTGCAGATCCAGTATAGTTCCTTTTTCACTACACAAGGATCAAGATCAAATGAACGTTCATTCTATTTCTGTCAAAAGAAGATATATTTCTAGTCTTTCTGTAAATAATGATCAAGTTAAACACAAATTCATTAGTTCAGATTTTTCGGGTAAAAAAGAAAGTAAGATTCCTGATTATTCAGAACTTAATCGAATCATATGTACTGGTCATTGTAATCTCATATATTCTGCTATTCTCTACGAGAATTCTGATTTATTGGCAAAGAGGCGAAGAAATAAATTCATCATCCCATTCCAATCAATTCAAGAACGAGAGAAAGAACTAATGACCCACTCCGCTATCTCGATTGAAATACCTAGAAATGGTATTTTCCGTAGAAATAGTGTTTTTGCTTATTTCGACGATCCCCAATACCGAAGAAAGAGTTCAGGAATTACTAAATCAGGAATTACTAAATATGGGACTATAGGAGTGCATTCAATCGTCAAAAAAGAGGATTTGATTGAGTATCGGGGAGTCAAAGAATTTAAGCCAAAATACCAAATGAAAGTGGATCGCTTTTTTTTCATTCCCGAGGAAGTGTATATTTTCCCCGAATCTTCTTCCCTAATGGTACGGAACAATAGTATCATTGGAGTAGATACACAAATTGCTTTAAATACAAGAAGTCGAGTGGGCGGGTTGGTCCGAGTGGAGAGAAAAAAAAAAAAAATGGAACTTAAAATCTTTTCTGGAGATATCCATTTTCCGGGAGAGACAGATAAGATATCCCGACACAGCGGTATCTTGATACCACCAGGAACGGTAAAAACAAATTCTAAGGAATCAAAAAAAGTGAAAAATTGGATCTATATCCAACGAATCACATCCGCCAAGAAAAAGTATTTTGTTTTGGTTCGACCAGTAATCATATATGAGATAGCGGACGGTATAAATTTAGAAACACTTTTCCCCCAGGATCTATTGCAGGAAAAAGAGAATCTGAAACTTCGAGTTGTCAATTATATTCTTTATGGAAATGGTAAACTAATTCGGGGAATTTCTGACACAAGTATTCAATTAGTTCGTACTTGTTTAGTGTTGAATTGGGACCAAGACAAAAAAAGTTCTTCTATCGAAGAGGCTCGCGCTTCTTTTGTTGAAGTAAGCACAAACGGTCTAATTTGTGATTTCCTAAGAATCAACCTAGCGAAATCCCATATTTCATATATCAGTAGAAAAAGGAATGATCCATCAGGTTCAGGACCGATCTCTAATAATGAGTCAGATCGAACCAATATTAATCCATTTTATCCCATTTATTCCAAGACAAGGATTCAACAATCACTTAAACAAAATCAAGGAACTATTAGTACGTTGTTGAATAGAAATAAGGAATGTCAATCTTTGATAATTTTGTCATCATCTAATTGTTTTCGAATGGATCCATTCAGCGATGCAAAACATCACAATGTAATAAAAGAATCAATTAAAAGAGATCCTATACCTATAATTCCAATTAGAAATTCATTGGGCCCTGTAGGAACAGCCCTTCCAATTGCGAATTTTTATTTATTTTACCTAATATTAATAACTCATAATCAGGTCTCGTTAACTAAATATTGGAAACTTGACAATTTAAAACAGACTTTTCAAGTACTTAAATATTATTTAATGGACGAAAACGGGAGAATTGTTAATCCTGATTCATGCAGTAACAGCGTTTTGAATCCATTCAATTTGAATTGGTATTTTCTCCATCATAATTATTATCATAATTTTTGTGAAGAAAGATTTACAACAATTAGCCTGGGACAGTTTATTTGTGAAAATGTATGTATGGCCAAAAACGGACCCCATCTAAAATCGGGTCAAGTTATAATTGTTCGCATGGACTCGGTAGTAATACGATCAGCAAAGCCCTATTTGGCCGCTCCAGGAGCAACCGTTCATGGCCATTATGGAGAAATCTTTTACGAAGGAGATACATTAGTTACATTTTTATATGAAAAATCGAGATCTGGTGATATAACGCAGGGTCTTCCAAAAGTGGAACAAGTGTTAGAAGTGCGCTCAATTGATTCAATATCGATAAGCCTAGAAAAGAGAGTTGAGGGTTGGAACGAGTGTATAACAAGAATTCTTGGAATTCCTTGGGGATTCTTGATTGGTGCTGAACTAACTATAGTGCAAAGTCGTATCTCTTTGGTTAATAAGATTCAAAAGGTTTATCGATCCCAAGGAGTGCAGATACATAATAGGCATATAGAAATTATTGTACGTCAAATAACATCCAAAGTTTTGGTTTCAGAAGACGGAATGTCAAATGTTTTTTCACCCGGAGAACTAATTGGATTGTTGCGAGCGGAACGAACGGGACGCGCTTTAGAAGAAGCCATCTGTTATCGAGCCATATTATTAGGAATAACGAGAGCATCTCTGAATACTCAAAGTTTCATATCCGAGGCCAGTTTTCAAGAAACTGCTCGCGTTTTAGCAAAAGCCGCTCTCCGCGGTCGTATCGATTGGTTGAAAGGCCTGAAAGAAAACGTTGTTCTAGGTGGTATGATACCCGGTGGTACCGGATTCAAAGGATTAGTGCAAGGCTCAAGGCAACATAATAACATTCCTTTGAAAACCAAAAAGAAGAATTTATTCGAGGGGGAATTTAGAGATAGAGATATTTTATTCCACCACAGAGAGTTATTTGATTCTTGCATTTCAAAAAATTTCTATGATACAGCAAAATAA

>lcl|NC_012224.1_cds_YP_002720164.1_72 [gene=ndhE] [locus_tag=JacuC_p073] [db_xref=GeneID:7564799] [protein=NADH dehydrogenase subunit 4L] [protein_id=YP_002720164.1] [location=complement(126758..127063)] [gbkey=CDS]
ATGATGCTCGAACATGTACTTGTTTTGAGTGCCTATTTATTTTCTATCGGTATCTATGGATTGATCACGAGTCGAAATATGGTTAGAGCCCTTATGTGCCTTGAACTTATACTGAATGCAGTTAATATAAATTTCGTAACATTTTCTGATTTTTTTGATAGTCGCCAACTAAAGGGAAATATTTTTTCAATTTTTGTTATAGCTATCGCAGCCGCTGAAGCAGCTATTGGACCAGCTATTGTTTCATCAATTTATCGTAACAGAAAATCAACCCGTATCAATCAATCGAATTTGTTGAATAAGTAG

>lcl|NC_012224.1_cds_YP_002720094.1_2 [gene=matK] [locus_tag=JacuC_p002] [db_xref=GeneID:7564826] [protein=maturase K] [protein_id=YP_002720094.1] [location=complement(2287..3816)] [gbkey=CDS]
ATGTGGAAATATCAAAGATATTTAGAACTAGATGGATCTCGAAAAAATGACCTCCTATACCCATTTATCTTTCGGGAGTATATTTATATATTTGCTCATGATCATAGTTTAAATAGATCGATTTTGTTGGAAAATGTAGGTTATGACAATAAATCTAGTTTCTTAATTGTAAAACGTTTAATTACTCGAATGTATCAACAGAATCGTTTGGTTTTTTTTTCTTTTTCTGCTAATGATTCTAACCAAAATTCATTTTTTAAGTACAACAAGAATTTGTATTATCAAATGATATCAGAGAGCTTTGCAGTTATTGTGGAAATTCCATTTTCTCTACGGTTAGTATCTTCTTTAGAAAGGTCGGAGATAGTTAAATCTCATAAATTACGATCAATTCATTCAATATTTCCTTTTTTAGAGGACAAATTTCCACATTTAATTTATGTGTCAGATGTATTAATACCTTACCCCATCCATATAGAAAAATTAGTCCAAACCCTTCGCTATTGGATGAAAGATCCCTCTTCTTTGCATTTATTACGACTCTTTCTTCATGAATATTGGAATAGGAACAGTCTTATTATTCAAAAGGGATCTATTTCTATTTTTACAAAAAGTAATCCAAGATTTTTCTTGTTCCTATATAATTCTCATGTATATGAATACGAATCAATCCTCTTTTTTCTTCGTAACCAATCCTTTCATTTACGATCAACATTTTCTCGAGTCCTTCTTGAACGAATTTATTTCTATGGAAAAATAGAACATTTTGCAGAAGTCTTTGCTAATGATTTTCAGACTATCCTATGGTTGGTCAAGTATCCTTTCATGCATTATGTTAGATATCAAGGAAAATCCATTCTGGCTTCAAAAGATGGGCCTCTTCTGATGAAAAAATGGAAATATTACCTTGTCAATTTATGTCAATGTCATTTTTATGTGTGGTTTCAACCAGAAAAGATCTATATAAATTCATTATCCAAGCATTCTCTCAACCTTTTGGGCTATCTTTCAAATGTAAAATTAAATCCTTCGGTCGTACGAAGTCAAATGCTAGAAAATTCATTTCTAATAGATAAAGATAATACTATGAAGAAACTCGATACAATAGTTCCAATTATTCCTTTGATTGGATCATTGTCAAAAACGAAATTTTGTAAGGCAGTAGGACATCCCATTAGTAAACCGGTCCGGACTGATTCATCGGATTCTGATATTATCGACCGATTTGTGTGTATATGCAGAAATCTTTCTCATTATTATAGTGGATCTTCAATAAAAAAGAGTTTGTATCGAGTAAAATATATACTTCGACTTTCTTGTGTTAAAACTTTGATTCGTAAACACAAAAGTACTGTACGCGCTTTTTTGAAAAGATTGGGTTCGGAATTATTAGAAGAATTTTTTACAGAAGAAGAACAAACTCTTTCTTTGATCTTCCCGAGAGTTTCCTCTATTTCGCGCAGGTTATATAGGGGGCGGGTTTGGTATTTGGATATTATTTCTATCAATGATTTGGCCAATCATGAATAA

>lcl|NC_012224.1_cds_YP_002720150.1_58 [gene=rps3] [locus_tag=JacuC_p058] [db_xref=GeneID:7564772] [protein=rps3] [protein_id=YP_002720150.1] [location=complement(90100..90765)] [gbkey=CDS]
ATGGGACAAAAAATAAATCCACTTGGTTTCAGACTTGGTACAACTCAAAGTCATCATTCTCTTTGGTTTGCACAACCAAAAAATTATTCTGAGGATCTACAAGAAGATCAAAAAATAAGAAACTGTATCAAAAATTATGTAAAACAAAATACGAAAATATCTTCTGGTGTTGAGGGAATTGCACGTATAGAGATTCAAAAACGAATCGATGTGATTCAGGTCATGATATATATGGGATTCCAAAAATTATTAATGGAAGGTAGACCTAAACAAATCGAAGAATTACAGATGAATGTACAAAAAGAACTTAATTATGTGAACCGAAAACTCAATATTGCTATTAAAATTAAAAGAATTTCAAATCCTTATGGGCAACCTAATATTCTTGCAGAATTTATAGCCGGGCAGTTAAAGAATAGAGTTTCATTTCGCAAAGCAATGAAAAAAGCTATTGAATTAACTGAACAAACGGATACAAAAGGAATTCAAGTCCAAATTGCGGGGCGTCTTGACGGAAAAGAAATTGCACGCGTCGAATGGATTAGAGAGGGTAGAGTTCCTTTACAAACCATTCAAGCTAAAATTGAGTATTGTTCGTATACAGTTAAAACTATTTATGGGGTATTAGGCATAAAAATTTGGACATTTCTAGATAAAAAAGAATAA

>lcl|NC_012224.1_cds_YP_002720158.1_66 [gene=rps7] [locus_tag=JacuC_p066] [db_xref=GeneID:7564782] [protein=ribosomal protein S7] [protein_id=YP_002720158.1] [location=complement(104932..105399)] [gbkey=CDS]
ATGTCACGTCGAGGTACTGCAGAAGAAAAAACTGCAAAATCCGATCCAATTTATCGTAATCGATTAGTTAACATGTTGGTTAACCGTATTCTGAAACACGGAAAAAAATCATTGGCTTATCAAATTATCTATCGAGCCATGAAAAAGATTCAACAAAAGACAGAAACAAATCCACTATCTGTTTTACGTCAAGCAATACGTGGAGTAACTCCCGATATAGCAGTAAAAGCAAGGCGTGTAGGCGGATCGACTCATCAAGTTCCCATTGAAATAGGATCCACACAAGGAAAAGCACTTGCCATTCGTTGGTTATTAGGGGCATCCCGAAAACGTCCGGGTCGAAATATGGCTTTCAAATTAAGTTCCGAATTAGTGGATGCTGCAAAAGGGAGTGGTGATGCCATACGCAAAAAGGAAGAGACTCATAGAATGGCAGAGGCAAATAGAGCTTTTGCACATTTTCGTTAA

>lcl|NC_012224.1_cds_YP_002720173.1_81 [gene=ORF126] [locus_tag=JacuC_p082] [db_xref=GeneID:7564818] [protein=ORF126] [protein_id=YP_002720173.1] [location=153989..154369] [gbkey=CDS]
ATGAATGGGGAGTCCGCTTTGAAAGCGTCCGCCCTGCAACCACCCCCGAGTATATGCTTCAACAGGAATTACACAAGGGTAGTTGATACAATAGAAACCTCTGGTAAAATGCCCGCCCGTAACCCAACAGATAAAGTACATTACATAGTCCGTTTTAGGGATTGGCGACTTACCCATTCAGTGACTTTGGCACTGGATGTTCCAAAAAGAAAATGGGTACTCTCGGGTCGGGTGAATTCAATAATAGACGTCTGTTGGCATTCCAGCCTTCCTTCTCCTTTCAGGGCCTATCCGAAAGAGAATCCAGTACTTCTTGGTCGTGAATATCTGAATAGGACAAACCGCCCCGTGGATATCTTTGCTTCGGAACAAAACAATTAG

>lcl|NC_012224.1_cds_YP_002720172.1_80 [gene=ndhB] [locus_tag=JacuC_p081] [db_xref=GeneID:7564816] [protein=NADH dehydrogenase subunit 2] [protein_id=YP_002720172.1] [location=join(150961..151737,152420..153169)] [gbkey=CDS]
ATGATCTGGCATGTACAGAATGAAAACTTCATTCTCGATTCTACGAGAATTTTTATGAAAGCCTTTCATTTGCTTCTCTTCGATGGAAGTTTTATTTTCCCAGAATGTATCCTAATTTTTGGCCTAATTCTTCTTCTGATGATCGATTCAACCTCTGATCAAAAAGATATACCTTGGTTATATTTCATCTCTTCAACAAGTTTAGTAATGAGTATAACGGCCCTATTGTTCCGATGGAGAGAAGAACCTATGATTAGCTTTTCGGGAAATTTCCAAACGAACAATTTCAACGAAATCTTTCAATTTCTTATTTTACTATGTTCCACTCTATGTATTCCTCTATCCGTAGAGTACATTGAATGTACAGAAATGGCTATAACAGAGTTTCTCTTATTCCTATTAACAGCTACTCTAGGAGGAATGTTTTTATGCGGTGCTAACGATTTAATAACTATCTTTGTAGCTCCAGAATGTTTCAGTTTATGCTCCTACCTATTATCTGGATATACCAAGAAAGATGTACGGTCTAATGAGGCTACTACGAAATATTTACTCATGGGTGGGGCAAGCTCTTCTATTCTGGTTCATGCTTTCTCTTGGCTATATGGTTCGTCCGGGGGAGAGATCGAGCTTCAAGAAATAGTGAATGGCCTTATCAATACACAAATGTATAACTCCCCAGGAATTTCAATTGCGCTTATATTCATCACTGTAGGAATCGGGTTCAAGCTTTCCCCAGCCCCTTCTCATCAATGGACTCCTGACGTATACGAAGGATCTCCCACTCCAGTCGTTGCTTTTCTTTCTGTTACTTCGAAAGTAGCTGCTTCAGCTTCAGCCACTCGAATTTTCGATATTCCTTTTTATTTCTCATCAAACGAATGGCATCTTCTTCTGGAAATCCTAGCTTTTCTGAGCATGATAGTGGGGAATCTCATTGCTATTACTCAAACAAGCATGAAACGTATGCTTGCATATTCGTCCATAGGTCAAATCGGATATGTAATTATTGGAATAATTGTTGGAGACTCAAATGGTGGATATGCAAGCATGATAACTTATATGCTCTTCTATATCTCCATGAATCTAGGAACTTTTGCTTGTATTGTATTATTTGGTCTACGTACCGGAACTGATAACATTCGAGATTATGCAGGATTATACACGAAAGATCCTTTTTTGGCTCTCTCTTTAGCCCTATGTCTCTTATCCCTAGGAGGTCTTCCTCCACTAGCAGGTTTTTTCGGAAAACTCCATTTATTCTGGTGTGGATGGCAGGCAGGCCTATATTTCTTGGTTTTAATAGGACTCCTTACGAGCGTTGTTTCTATCTACTATTATCTAAAAATAATCAAGTTATTAATGACTGGACGAAACCAAGAAAGAACCTCTCACGTGCGAAATTATAGAACTTTAAGATCAAACAATTCCATCGAATTGAGTATGATTGTATGTGTGATAGCATCTACTATACCGGGAATATCAATGAACCCGATTATTGAAATTGCTCAAGATACCCTTTTTTAG

>lcl|NC_012224.1_cds_YP_002720147.1_55 [gene=rps8] [locus_tag=JacuC_p055] [db_xref=GeneID:7564769] [protein=ribosomal protein S8] [protein_id=YP_002720147.1] [location=complement(86689..87093)] [gbkey=CDS]
ATGGGTAGGGATTCTATTGCTGAAATAATAACCTCTATACGAAATACTGACATGAATAGAAAAGGAACTGTTCGAATAGCATCTACTAATATCACCGAAAACATTATTAAAATACTTTTACGAGAAGGTTTTATTGAAAATGTCAGGAAACATCAGGAAGGCAACAAAAAATTTTTGGTCTTAACCCTACGACATAGAAGGAAGATGAAAGGGCCATATAGAACTAGTCTAAATTTAAAACGAATCAGCCGACCTAGTCTACGAATCTATCCTAACTATCAAAAAATTCCTAGAATTTTGGGCGGGATGGGGATTGTAATTCTTTCTACTTCTCGGGGTATAATGACAGACCGAGAAGCTCGACTAGAAAGAATCGGTGGAGAAATCTTGTGTTATATATGGTAA

>lcl|NC_012224.1_cds_YP_002720148.1_56 [gene=rpl14] [locus_tag=JacuC_p056] [db_xref=GeneID:7564770] [protein=ribosomal protein L14] [protein_id=YP_002720148.1] [location=complement(87606..87974)] [gbkey=CDS]
ATGATCCAATCTCAAACCCATTTGAATGTAGCAGATAACAGTGGAGCCCGAGAATTGATGTGTATTCGAATCATGGGGACTAGTAATCGACGATATGCTCATATTGGTGACGTTATTGTTGCTGTGATCAAGGAAGCAACACCAAATTCACCTCTAGAAAGATCCGAAGTAATAAGAGCTGTAATTGTACGTACTTGTAAAGAACTCAAACGCGATAACGGTATAATAATACGATATGATGATAATGCTGCAGTTGTTATTGATCAAGAAGGAAATCCAAAAGGAACTCGAATTTTTGGTGCAATCGCCCGGGAATTGAGACAGTTAAATTTTACTAAAATAGTTTCATTAGCACCCGAAGTATTATAA

>lcl|NC_012224.1_cds_YP_002720137.1_45 [gene=clpP] [locus_tag=JacuC_p045] [db_xref=GeneID:7564759] [protein=ATP-dependent Clp protease proteolytic subunit] [protein_id=YP_002720137.1] [location=complement(join(76291..76518,77172..77462,78329..78397))] [gbkey=CDS]
ATGCCTATTGGTGTTCCAAAAGTCCCTTTTCGAAATCCTGGGGAAGACGATTCAATTTGGATTGACGTAAACCGACTTTATCGAGAAAGATTACTTTTTTTAGGTCAAGATGTTGATAGCGAAATCTCGAATCAACTTATTGGTCTTATGGTATATCTCAGTATAGAGAGCGAGACAAAAGATTTGTATTTGTTTATAAACTCTCCTGGCGGATGGGTAATACCCGGAATAGCTATTTATGATACTATGCAATTTGTGCGACCAGATGTACAAACAGTATGCATGGGATTAGCTGCTTCAATGGGATCTTTTATCCTGGTCGGAGGAAAAATTACCAAACGTTTAGCATTCCCTCATGCTAGGGTAATGATTCATCAACCTATTGCTGGTTTTTATGAGGCACAAATAGTAGAATTTGTCCTGGAAGCAGAAGAACTACTGAAACTGCGCGAAATCCTCACAAGGATTTATGCACAAAGAACGGGAAAACCCTTATGGATTGTATCCGAAGACATGGAAAGAGATGTTTTTATGTCAGCAACAGAAGCCCAAGCTCATGGAATTGTTGACCTTGTAGCAGTTGCCTAA

>lcl|NC_012224.1_cds_YP_002720143.1_51 [gene=petD] [locus_tag=JacuC_p051] [db_xref=GeneID:7564765] [protein=cytochrome b6/f complex subunit IV] [protein_id=YP_002720143.1] [location=join(82940..82947,83809..84304)] [gbkey=CDS]
ATGGGAGTAACAAAAAAACCTGACTTGAATGATCCTGTATTAAGAGCTAAATTGGCTAAGGGAATGGGTCATAATTATTACGGAGAACCTGCATGGCCCAATGATCTTTTATATATTTTTCCAGTAGTAATTCTAGGTACTATTGCGTGTAATGTAGGATTAGCGGTTCTAGAGCCGTCAATGATTGGTGAACCTGCGGATCCATTTGCAACTCCTTTAGAGATATTGCCTGAATGGTATTTTTTTCCTGTATTTCAAATACTTCGTACAGTACCCAATAAGTTATTGGGTGTTCTTTTAATGGTTTCAGTACCTGCAGGATTATTAACAGTACCCTTTTTGGAGAATGTTAATAAATTCCAAAATCCATTTCGTCGCCCAGTTGCGACAACCGTCTTTTTGATTGGTACTGCAGTAGCCCTTTGGTTAGGTATTGGAGCAACATTACCTATTGATAAATCCCTAACTTTAGGTCTTTTTCAAATTGATTCAATTGTAAAATAA

>lcl|NC_012224.1_cds_YP_002720161.1_69 [gene=ccsA] [locus_tag=JacuC_p070] [db_xref=GeneID:7564796] [protein=cytochrome c biogenesis protein] [protein_id=YP_002720161.1] [location=123330..124304] [gbkey=CDS]
ATGATATTTTCGACTTTAGAACATATATTAACTCATATATCTTTTTCAGTCGTGTCAATTGTAATTACAATTCATTTGATAACCTTATTAGTCGATGAATTCGTAGAATTATATGATTCGTCAGAAAAGGGCATGATAATTACTTTTTTCTGTATAACAGGATTATTAGTTACTCGTTGGATTTTTGGGGGACATTTACCATTAAGTGATTTATATGAATCATTAATCTTTCTTTCCTGGGTTTTTTCCATTATTCATATGGTTCCGTATTTTAAAAAACACAAAAATTTTCTAAGCGCAATAACCGCGCCAAGTACTTTTTTTACCCAAGGATTTACTACTTCGGGTCTTTTAACTGACATGCATCACTCCGAAATCTTAGTGCCCGCTCTCCAATCCCATTGGTTAATGATGCACGTAAGTATGATGATATTGGGCTATGCAGCTCTTTTGTGTGGATCATTATTATCAGTAGCATTTCTAGTAATCACATTTCGAAAAATTATAAGAATTGTTGATAAAAACAATAATTTATTAAATGATTCATTTTCTTTTAGTGAGATACAATATATGACGGAAAGAAAGAATGTTTTAAGAAATATTTCTTTTCTTTCTTCTACTAATAGGAATTATTACAGGTTTCAATTGATTCAACAATTAGATGACTGGGGTTATCGTATTATAAGTATAGGGTTTATCTTTTTAACCATAGGTATTCTTTCGGGAGCAGTCTGGGCTAATGAAGCGTGGGGATCATATTGGAGTTGGGACCCAAAGGAAACTTGGGCATTTATTACGTGGACCATATTTGCGATTTATTTTCATACTCGAACAAATAAAAATTTTGAGGATTTAAATTCGGCAATTGTCGCTTTTATCGGTTTTCTTCTAATTTGGATATGTTATTTTGGAGTTAATTTATTAGGAATAGGTTTGCATAGTTATGGTTCATTTACATTAACAATTAACATCTAA

>lcl|NC_012224.1_cds_YP_002720101.1_9 [gene=rps2] [locus_tag=JacuC_p009] [db_xref=GeneID:7564837] [protein=ribosomal protein S2] [protein_id=YP_002720101.1] [location=complement(17086..17796)] [gbkey=CDS]
ATGATAAGAAGATATTGGAACATTAATTTGGAAGAGATGATAAAAGCAGGAGTTCATTTTGGTCATGGTACTAGAAAATGGAATCCGAGAATGGCACCTTATATCTCTGCAAAGCGTAAAGGTATTCATATTACAAATCTTACTAGAACTGCTCGTTTTTTATCAGAAGCTTGTGATTTAGTTTTTGATGCAGCAAGCAGGAGAAAACAATTCTTAATTGTTGGTACAAAAAATAAAGCAGCGGATTCAGTAGCGCGGGCTGCAATAAGGGCTCGGTGTCATTATGTTAATAAAAAATGGCTTGGCGGTATTTTAACGAATTGGTCCACTACAGAAACTAGACTTCAAAAGTTCAGGGACTTGAGAATGGAACAAAAGGCAGGTAGACTCAACCGTCTTCCGAAAGGAGATGCGGCTCGATTGAAGAGACAGTTAGCTCACTTGCAAACATATCTGGGCGGGATTAAATATATGACGGGGTTACCGGATGTTGTAATAATCGTTGATCAGCAAGAGGAACATACGGCTCTTCGGGAATGTATCACTTTGGGAATTCCAACGATTTGTTTAATTGATACAAACTGTGATCCGGATCTCGCAGATATTTCGATTCCAGCGAATGATGACGCTATAGCTTCAATCCGATTAATTCTTAATAAATTAGTATTTGCAATTTGTGAAGGGCGTTCTAGCTATATACGAAATCCCTGA

>lcl|NC_012224.1_cds_YP_002720176.1_84 [gene=rpl2] [locus_tag=JacuC_p085] [db_xref=GeneID:7564822] [protein=ribosomal protein L2] [protein_id=YP_002720176.1] [location=join(162364..162756,163386..163856)] [gbkey=CDS]
ATGGCGATACATTTATACAAAACTTCTACCCCGAGCACACGCAATGGAGCCGTAGACAGTCAAGTGAAATCCAATACACGAAATAATTTGATCTATGGACAGCATCGTTGTGGTAAAGGACGTAATGCCAGAGGAATCATTACCGCAAGGCATAGAGGGGGGGGTCATAAGCGTCTATACCGTAAAATCGATTTTCGACGGAATGAAAAAGACATATATGGTAGAATCGTAACCATAGAATACGACCCTAATCGAAATGCATACATTTGTCTCATACACTATGGGGATGGTGAGAAGAGATATATTTTACATCCCAGAGGGGCTATAATTGGAGATACCATTATTTCTGGTACAGAAGTTCCTATAAAAATGGGAAATGCCCTACCTTTGAGTGAGGTTTTGATTGATCAAAAAGAAGAATCTACTTCAACCGATATGCCCTTAGGCACGGCCATACATAACATAGAAATCACACTTGGAAAGGGTGGACAATTAGCTAGAGCTGCAGGTGCTGTAGCGAAACTGATTGCAAAAGAGGGGAAATCGGCCACATTAAAATTACCTTCTGGGGAGGTTCGTTTAATATCCAAAAACTGCTCAGCAACAGTCGGACAAGTAGGCAATACTGGGGTGAACCAGAAAAGTTTGGGTAGAGCCGGATCTAAATGTTGGCTAGGTAAGCGTCCTGTAGTAAGAGGAGTAGTTATGAACCCTGTAGACCATCCCCATGGGGGTGGTGAAGGGAGGGCTCCAATTGGTAGAAAAAAACCCGCAACCCCTTGGGGTTATCCTGCACTTGGAAGAAGAAGTAGAAAAAGGAATAAATATAGTGATAATTTGATTCTTCGTCGACGGAGTAAATAG

>lcl|NC_012224.1_cds_YP_002720100.1_8 [gene=atpI] [locus_tag=JacuC_p008] [db_xref=GeneID:7564836] [protein=ATP synthase CF0 A subunit] [protein_id=YP_002720100.1] [location=complement(16131..16874)] [gbkey=CDS]
ATGAATGTTCTATCATGTTCCATAAACACACTAAAAGGGTTATATGATATATCCGGTGTGGAAGTAGGCCAGCATTTCTATTGGAAAATAGGAGGTTTCCAAGTCCATGCCCAAGTACTTATTACTTCTTGGGTTGTAATTACTATCTTATTAGGTTCAGCCATTGTAGCTGTTCGGAATCCACAAACCATTCCGACTGGCGGGCAGAATTTCTTCGAATATGTCCTTGAATTCATTCGGGATGTGAGCAAAACTCAGATTGGAGAGGAATATGGCTCATGGGTCCCCTTTATTGGAACTATGTTTTTATTTATTTTTGTTTCTAATTGGGCTGGGGCGCTTTTGCCTTGGAAGATCATAGAGTTACCTCATGGGGAGTTAGCCGCACCTACGAATGATATAAATACTACCGTTGCTTTAGCTTTACTTACGTCAATAGCATATTTTTATGCGGGCCTTAGGAAAAAAGGATTAGGTTATTTCAGCAAATACATTCAGCCAACTCCAATCCTTTTACCCATTAACATTTTAGAAGATTTCACAAAACCTTTATCACTTAGCTTTCGACTTTTCGGAAATATATTAGCGGACGAATTAGTAGTTGTTGTTCTTGTTTCTTTAGTACCTTCAGTGGTTCCTATACCTGTCATGTTCCTTGGATTATTTACAAGCGGTATTCAAGCTCTTATTTTTGCAACTTTAGCCGCGGCTTATATAGGCGAATCCATGGAGGGGCATCATTGA

>lcl|NC_012224.1_cds_YP_002720114.1_22 [gene=rps4] [locus_tag=JacuC_p022] [db_xref=GeneID:7564859] [protein=ribosomal protein S4] [protein_id=YP_002720114.1] [location=complement(50193..50798)] [gbkey=CDS]
ATGTCACGTTACCGAGGGCCTCGTTTCAAAAAAATACGCCGTCTCGGGGCTTTACCGGGACTAACTAGTAAAAGGCCTAGAGCCGGGAGCGATCTTAGAAATCAATCACGCTCCGGTAAAAAATCTCAATATCGTATTCGTTTAGAAGAAAAGCAAAAATTACGTTTTCATTATGGTCTTACAGAACGACAATTGCTTAAATACGTTCGTATCGCCGCAAAAGCCAAAGGGTCAACGGGTCAGGTTTTACTACAATTACTTGAAATGCGGTTGGATAACATCCTTTTTCGATTGGGTATAGCGTCAACTATTCCTCGAGCCCGCCAATTAGTTAATCATAGACATATTTTAGTTAATGGCCGTATAGTAGATATACCAAGTTATCGCTGCAAACCCCGAGATGTTATTACAGTGAGGGATGAACAAAAATCTAGAGCTATGATTCCAAATTATCTTGATTCATCCCCCCAAGAGGAATTGCCAAAACATTTGACTCTTCACCCAATCCAATATAAAGGATTGGTCAATCAAATAATAGATAGTAAATGGGGTGGCTTAAAAATAAATGAATTGCTAGTGGTAGAATATTATTCTCGTCAGACTTAA

>lcl|NC_012224.1_cds_YP_002720174.1_82 [gene=ycf2] [locus_tag=JacuC_p083] [db_xref=GeneID:7564819] [protein=Ycf2] [protein_id=YP_002720174.1] [location=complement(154840..161736)] [gbkey=CDS]
ATGAAAGGACATCAATTCAAATCCTGGATTTTCGAATTGAGAGAGATATTGAGAGAGATCAAGAATTCTCACTATTTCTTAGATTCATGGACCCAATTCAATTCAGTGGGATCTTTCATTCACATTTTTTTCCATCAAGAACGTTTTATAAAACTCTTGGACTCCCGAATTTGGAGTATCTTACTTTCACGCAATTCACAGGGTTCAACAAGCAATCGATATTTCACGATCAAGGGTGTAGTACTATTTGTAGTAGTGGTTCTTATATATCGTATTAACAATCGAAAGATGGTCGAAAGAAAAAATCTCTATTTGACAGGGCTTCTTCCTATACCTATGAATTCCATTGGACCCAGAAATGATACATTGGAAGAATCCTTTGGGTCTTCCAATATCAATAGGTTGATTGTTTCGCTCCTGTATCTTCCAAAAGGAAAAAAGATCTCTGAGAGCTCTTTCCTGGATCCGAAAGAGAGTACTTGGGTTCTCCCAATAACTAAAAAGTCTGAATCTAACTGGGGTTCGCGGTGGTGGAGGAACTGGATCGGAAAAAAGAGGGATTCTAGTTGTAAGATATCTAATGAAACCGTCGCTGGAATTGAGATCTCATTCAAAGAAAAAGATATCAAATATCTGGAGTTTCTTTTTGTATATTATATGGATGATCCGATCCGCAAGGACCATGATTGGGAATTGTTTGATCGTCTTTCTCCGAGGAAGGGGCGAAACATAATCAACTTGAATTCGGGACAGCTATTCGAAATCTTAGTGAAAGACTGGATTTGTTATCTCATGTTTGCTTTTCGCGAAAAAATACCAATTGAAGTGGAGGGTTTCTTCAAACAACAAGGAGCTGGGTCAACTATTCAATCAAATGATATTGAGCATGTTTCCCATCTCTTCTCGAGAAAGAAGTGGGCTATTTCTTTGCAAAATTGTGCTCAATTTCATATGTGGCAATTCCGCCAAGATCTCTTCGTTAGTTGGGGGAATAATCCGCACGAATCGGATTTTTTGAGGAACATATCGAGAGAGGATTGGATTTGGTTAGACAATGTGTGGTTGGTAAACAAGGATCGGTTTTTTAGCAAGGCACGGAATATATCGTCAAATATTCAATATGATTCCACAAGATCTAGTTTCGTTCAAGGAAGGAATTCTAGCCAATTGAAGGGATCTTCTGATCAATCCAGAGATCATTTCGATTCCATTAGTAATGAGGATTCGGAATATCACACATTGATCAATCAAAGAAAGATTCAACAACTAAAAGAAAGATCGATTCTTTGGGATCCTTCCTTTCTTCAAACGGAACGAACAGAGATAGAATCAGACCGATTCCCTAAATGCCTTTCTGGATATTCCTCAATGTCCCGACTATTCACGGAAGGTGAGAAGGAGATGAATAATCATCTGCTTCCGGAAGAAATCGAAGAATTTCTTGGGAATCCTACAAGATCCATTCGTTCTTTTTTCTCTGACAGATCGTCAGAACTTCATCTGGGTTCGAATCCTACTGAGAGGTCCACTAAATTGTTGAAGAAAGAACAAGATGTTTCTTTTGTCCCTTCCAGGCGATCGGAAAATAAAGAAATAGTTAATATATTCAAGATAATCACGTATTTACAAAATACCGTCTCAATTCATCCTATTTCATCAGATCCGGGATGTGATATGGTTCTGAAGGATGAACTGGATATGGACAGTTCCAATAAGATTTCTTTCTTGAACAAAAATCCATTTTTTGATTTATTTCATCTATTCCATGATCGGAACGGGGGGGGATACACGTTACACCACGATTTTGAATCAGAAGAGAGATTTCAAGAAATGGCAGATCTATTCACTCTATCAATAACCGAGCCGGATCTGGTGTATCATAAGGGATTTACCTTTTTTATTGATTCTTACGGATTGGATCAAAAACAATTCTTGAATGAGGTATTCAACTCCAGGGATGAATCGAAAAAGAAATCTTTATTGGTTCTACCTCCTATTTTTTATGAAGAGAATGAATCTTTTTATCGAAGGATCAGAAAAAAATGGGTCCGGATCTCCTGCGGGAATGATTTGGAAGATCCAAAACAAAAAATAGTGGTATTTGCTAGCAACAACATAATGGAGGCAGTCAATCAATATGGATTGATCCTAAATCTGATTCAAATCCAATATAGTACCTATGGGTACATAAGAAATGTATTGACTCAATTCTTTTTAATGAATAGATCCGATCGCAACTTCGAATATGGAATTCAAAGGGATCAAATAGGAAATGATACTCTGAATCATAGAACTATAATGAAATATACGATCAACCAACATTTATCGAATTTGAAACAGAGTCAGAAGAACTGGTTCGATCCTCTTATTTTTCTTTCTCGAACCGAGAGATCCATGAATTGGGATCCTAATGCATATAGATACAAATGGTCTAATGGGAGCAAGAATTTCCAGGAATATTTGGAACATTTCATTTCTGAGCAGAAGAGCCGTTTTCTTTTTCAAGTAGTGTTCGATCGATTACGTATTAATCAATATTCGATTGATTGGTCTGAGGTTATCGACAAAAAAGATTTGTCTAAGTCACTTCGTTTCTTTTTGTCCAAGTTACTTGTTTTTTTGTCCAAGTTTCTTCTCTTTTTGTCTAACTCACTTCCTTTTTTCTTTGTGAGTTTCGGGAATATCCCCATTCATAGGTCCGAAATCCATATCTATGAATTGAAAGGTCCGAATGATCCACTCTGCAATCAGCTGTTAGAACCAATAGGTCTTCAAATCGTTCATTTGAAAAAATGGAAACCCTTCTTATTGGATGATCATGATACTTCCCAAAAATCGAAATTTTTGATTAATGGAGGAACAATATCACCATTTTTGTTCAATAAGATACCAAAGTGGATGATTGACTCATTCCATACTAGAAATAATCGCAGGAAATCTTTTGATAACACGGATTCCTATTTCTCAATGATATCCCACGATCAAGACAATTGGCTGAATCCCGTGAAACCATTTCATAGAAGTTCATTGATATCTTCTTTTTATAAAACAAATCGACTTCGATTCTTGAATAATCTACATCACTTCTGCTTCTATTGTAACAAAAACAAAAGATTCCCTTTTTATGTGGAAAAGGCCCGTATCAAGAATTATGATTTTACGTATGGACAATTCCTCAATATCTTGTTCATTCGCAACAAAATATTTTCTTTGTGCGGCGGTAAAAAAAAACATGCTTTTGGGGAGAGAGATACTATTTCACCAATCGAGTCACAGGTATCTAACATATTCATACCTAATGATTTTCCACAAAGTGGTAACGAAAGGTATAACTTGTACAAATCTTTCCATTTTCCAATTCGATCCGATCCATTCGTTCGTAGAGCTATTTATTTGATCGCAGACATTTCGGGAACACCTCTAACAGAGGGACAAATAGTCAATTTTGAAAGAACTTATTGTCAACCTCTTTCGGATATGAATCTATCTGATTCAGAAGGGAAGAACTTGCATCAGTATCTCAATTTCAATTCAAACATGGGTTTGATTCACACTCCATGTTCTGAGAAATATTTACCATCCGAAAAGAGGAAAAAACGGAGTCTTTGTCTAAAGAAATGTGTTGAAAAAGGGCAGATGTATAGAACCTTTCAACGAGATAGTGCTTTTTCAACTCTCTCAAAATGGAATCTATTCCAAACATATATGCCATGGTTCCTTACTTCGACAGGGTACAAATATCTAAATTTGATATTTTTAGATACCTTTTCGGACCTATTACCGATACTAAGTAGCAGTCAAAAATTTGTATCCATTTTTCATGATATTATGCACGGATCAGATATATCATGGCGAATTCTTCAGAAAAAATGGTGTCTTCCACAATGGAATCTGATAAGTGAGATTTCGAGTAAGTGTTTACATAATCTTCTTCTGTCCGAAGAAATGATTCATCGAAATAATGAGCCACCATTGATATCGACACATCTGAGATCGCCAAATGTTCGGGAGTTCCTCTATTCAATCCTTTTCCTTCTTCTTGTTGCTGGATATCTCGTTCGTACACATCTTCTCTTTGTTTCCCGAGCCTATAGTGAGTTACAGACAGAGTTCGAAAAGGTCAAATCTTTGATGATTCCATCATACATGATTGAGTTGCGAAAACTTCTGGATAGGTATCCTACATCTGAACTGAATTCTTTCTGGTTAAAGAATCTCTTTCTAGTTGCTCTGGAACAATTAGGAGATTTTCTAGAAGAAATGCGGGGTTTTGCTTCTGGCGGCAACATGCTATGGGGTGGTGGTCCCGCTTATGGGGTTAAATCAATACGTTCTAAGAATAAATTTTTGAATATCAATCTCATCGATCTCATAAGTATCATACCAAATCCCATCAATCGAATCACTTTTTCGAGAAATACGAGACATCTAAGTCATACAAGTAAAGAGATTTATTCATTGATAAGAAAAAGAAAAAACGTGAATGGTGATTGGATTGATGATAAAATAGAATCCTTGGTCGCGAACAGTGATTCGATTGATGATAAAGAAAGAGAATTCTTGGTTCAGTTCTCCACCTTAACGACAGAAAACAGGATTGATCAAATTCTATTGAGTCTGACGCATAGTGATCATTTATCAAAGAATGACTCTGGTTATCAAATGATTGAAGAGCCGGGAGCAATTTATTTACGATACTTAGTTGACATTCATAAAAAGTATCTAATGAATTATGAGTTCAATACACCCTGTTTAGCAGAAAGACGGATATTCCTTGCTTATTATCAGACAACCACTTATTCACAAACCTCGTGTGGGGTGAATAGTTTTCATTTCCCATCTCATGGAAAACCCTTTTCGCTCCGCTTAGCCCTATCCCCCTCTAGGGGTATTTTAGTGATAGGTTCTATAGGAACTGGACGATCCTATTTGGTCAAATACCTAGCGACAAACTCCTATCTTCCTTTCATTACAGTATTTCTGAACAAGTTCCTGGATAACAAGCCTAAGGGCTTTCTTATTGATGATAGTGACGATATTGATGATAGTGACGATATTGATGATAGTGACGATATTGATGATAGTGACGATATTGATGTGAGTGACGATATTGATGATAGTGACGATATCGACCGTGACTTTGATACGGAGCTGGAGTTTCTAACTAGGATGAATGCGCTAACTATGGGTATGATGCCGGAAATAGACCGATTTTATATCACCCTTCAATTCGAATTAGCAAAAGCAATGTCTCCTTGCATAATATGGATTCCAAACATTCATGATTTGGATGTGAATGAGTCGAATTACTTATCCCTCGGTCTATTAGTGAACTATCTCTCCAGGGATTGTGAAAGATGTTCCACTAGAAATATTCTTGTTATTGCTTCGACTCATATTCCCCAAAAAGTGGATCCCGCTCTAATAGCTCCGAATAAATTAAATACATGCATTAAGATACGAAGGCTTCTTATTCCACAACAACGAAAGCACTTTTTTACTCTTTCATATACTAGGGGATTTCACTTGGAAAAGAAAATGCTCCATACTAATGGATTCGGGTCCATAACCATGGGTTCCAATGTACGAGATCTTGTAGCACTTACCAATGAGGCCCTATCGATTAGTATTACACAGAAGAAATCAATTCTAGACACTAATATAATTAGATCCGCTCTTCATAGACAAACTTGGGATTTGCGATCCCAGGTAAGATCGGTTCAGGATCATGGGATCCTTTTCTATCAGATAGGAAGGGCTGTTGCACAAAATGTATTTCTAAGTAATTGCCCCATAGATCCTATATCTATCTATATGAAGAAGAAATCATGTAACGAAGGGGATTCTTATTTGTACAAATGGTACTTCGAACTTGGAATGAGCATGAAGAAATTAACGATACTTCTTTATCTTTTGAGTTGTTCCGCCGGATCGGTTGCTCAAGACCTTTGGTCTCTACCCGGACCCGATGAAAAAAATGGGATCACTTATTATGGACTTGTTGAGAATGATTCTGATCTAGTTCATGGCCTATTAGAAGTCGAAGGCGCTCTGGTGGGATCCTCACGTACAGAAAAAGATTGCAGTCAGTTTGATAATGATCGAGTGACATTGCTTCTTCGGCCCGAACCAAAGAGTCCCTTAGATATGATGCAAAATGGATCTTGTTCTATCCTTGATCAGAGATTTCTCTATGAAAAATACGAATCGGAGTTTGAAGAAGGGGAAGGAGTCCTCGACCCGCAACAGATAGAGGAGGATTTATTCAATCACATAGTTTGGGCTCCTAGAATATGGCGCCCTTGGGGTTTTCTATTTGATTGTATCGAAAGGCCCAATGAATTGGGATTTCCCTATTGGGCCAGGTCATTTCGGGGCAAGCGGATCATTTATGATGAAGAGGATGAGCTTCAAGAGAATGATTCGGAGTTCTTGCAGAGTGGAACCATGCAGTACCAGATACGAGATAGATCTTCCAAAGAACAAGGCTTTTTTCGAATAAGCCAATTCATTTGGGACCCTGCGGATCCACTCTTTTTCCTATTCAAAGATCAGCCCTTTGTCTCTGTGTTTTCACATCGAGAATTCTTTGCAGATGAAGAGATGTCAAAGGGGCTTCTTACTTCCCAAACAGATCCTCCTACATCTATATATAAACGCTGGTTTATCAAGAATACGCAAGAAAAGCACTTCGAATTGTTGATTCATCGCCAGAGATGGCTTAGAACCAATAGTTCATTATCTAATGGATTTTTCCGTTCTAATACTCTATCCGAGAGTTATCAGTATTTATCAAATCTGTTCCTATCTAACGGAACGCTATTGGATCAAATGACAAAGGCATTGTTGAGAAAAAGATGGCTTTTCCCGGATGAAATGAAAATTGGATTCATGTAA

>lcl|NC_012224.1_cds_YP_002720107.1_15 [gene=psbD] [locus_tag=JacuC_p015] [db_xref=GeneID:7564848] [protein=photosystem II protein D2] [protein_id=YP_002720107.1] [location=37203..38264] [gbkey=CDS]
ATGACTATAGCCCTTGGTAAATTTACCAAAGACGAAAATGATTTATTTGATATTATGGATGACTGGTTACGGAGGGACCGTTTCGTTTTTGTAGGTTGGTCCGGTCTATTGCTCTTTCCTTGCGCCTATTTCGCCTTAGGGGGTTGGTTCACAGGTACAACCTTTGTAACCTCATGGTATACCCATGGATTGGCTAGTTCCTATTTGGAAGGCTGCAACTTCTTAACCGCTGCAGTTTCTACTCCTGCTAATAGTTTAGCGCATTCTTTGTTATTACTATGGGGTCCCGAAGCACAAGGGGATTTTACTCGTTGGTGTCAATTAGGCGGTTTGTGGACTTTTGTTGCTCTCCACGGTGCTTTCGGACTAATAGGTTTTATGTTACGTCAATTTGAACTTGCTCGATCCGTGCAATTGCGACCTTATAATGCAATCGCATTCTCTGGTCCAATTGCTGTTTTTGTTTCTGTATTCCTGATTTATCCACTAGGTCAGTCTGGTTGGTTTTTTGCGCCTAGTTTTGGTGTAGCAGCTATATTTCGATTCATCCTCTTTTTCCAAGGGTTTCATAACTGGACGCTGAACCCATTTCATATGATGGGGGTTGCCGGCGTATTAGGTGCTGCTCTGCTATGCGCTATTCATGGTGCTACTGTAGAAAATACTTTATTTGAAGATGGTGATGGTGCAAATACATTCCGTGCTTTTAACCCAACTCAAGCCGAAGAAACTTATTCAATGGTTACCGCTAACCGCTTTTGGTCCCAAATCTTTGGGGTTGCTTTTTCCAATAAACGTTGGTTACATTTCTTTATGTTATTTGTACCAGTAACCGGTTTATGGATGAGTGCTCTTGGAGTAGTCGGTCTGGCTCTGAATCTACGTGCCTATGACTTCGTTTCTCAGGAGATCCGTGCAGCGGAAGATCCTGAATTTGAGACTTTCTACACTAAAAATATTCTCTTAAACGAAGGTATTCGTGCTTGGATGGCGGCTCAAGATCAGCCTCATGAAAACCTTATATTCCCTGAGGAGGTTCTACCACGTGGAAACGCTCTTTAA

>lcl|NC_012224.1_cds_YP_002720117.1_25 [gene=ndhC] [locus_tag=JacuC_p025] [db_xref=GeneID:7564865] [protein=NADH dehydrogenase subunit 3] [protein_id=YP_002720117.1] [location=complement(55043..55405)] [gbkey=CDS]
ATGTTTCTGATTTACGAATATGATATATTCTGGGCATTTCTAATAATATCAAGTGCTATTCCTATTTTAGCATTTCTAATTTCCGGAGTTTTATCCCCGATTAGCAAAGGGCCGGAGAAACTTTCTAGTTATGAATCGGGTATAGAACCAATGGGCGATGCTTGGTTACAATTTCGAATCCGTTACTATATGTTTGCTCTAGTTTTTGTTGTTTTTGATGTTGAAACCGTTTTTCTTTATCCATGGGCAATGAGTTTCGATATATTGGGGTTATCCGCATTTATAGAAGCTTTAATTTTCGTACTTATCCTAATTGTTGGTTTAGTTTATGCGTGGAGAAAAGGAGCGTTAGAATGGTCTTAG

>lcl|NC_012224.1_cds_YP_002720120.1_28 [gene=rbcL] [locus_tag=JacuC_p028] [db_xref=GeneID:7564870] [protein=ribulose-1,5-bisphosphate carboxylase/oxygenase large subunit] [protein_id=YP_002720120.1] [location=60251..61678] [gbkey=CDS]
ATGTCACCACAAACAGAGACTAAAGCAAGTGTTGGATTCAAGGCTGGTGTTAAAGATTATAAATTGACTTATTATACTCCTGAGTATCAAACCAAAGATACTGATATCTTGGCAGCATTCCGAGTAACTCCTCAACCTGGAGTTCCGCCTGAGGAAGCAGGAGCTGCGGTAGCTGCTGAATCTTCTACTGGTACATGGACAACTGTGTGGACCGATGGGCTTACCAGTCTTGATCGTTATAAAGGACGATGCTACGACATCGAGCCCGTTGCTGGAGAAGAAAATCAATATATTGCTTATGTAGCTTACCCCTTAGACCTTTTTGAAGAAGGTTCTGTTACTAACATGTTTACTTCCATTGTGGGTAATGTATTTGGGTTCAAAGCCCTACGCGCCCTACGTCTGGAGGATTTGCGAATCCCTACTGCTTATACTAAAACTTTCCAAGGGCCGCCTCATGGTATCCAAGTTGAGAGAGATAAATTGAACAAGTATGGTCGCCCCCTATTGGGTTGTACTATTAAACCTAAATTGGGGCTATCCGCTAAGAATTATGGTAGAGCGGTTTATGAATGTCTTCGCGGTGGACTTGATTTTACCAAAGATGATGAGAACGTGAATTCCCAACCATTTATGCGTTGGAGAGACCGTTTCTTATTTTGTGCCGAAGCAATTTATAAAGCACAGGCTGAAACAGGTGAAATCAAAGGACATTATTTGAATGCTACTGCAGGTACATGTGAAGAAATGATCAAAAGGGCTGTATTTGCCAGAGAATTAGGAGTTCCTATCGTAATGCATGACTACCTAACAGGGGGATTCACCGCAAATACTTCCTTGGCTCATTATTGCCGTGATAATGGTTTACTTCTTCACATTCACCGCGCAATGCATGCAGTTATTGATAGACAGAAGAATCATGGTATGCATTTTCGTGTACTAGCTAAGGCGTTACGTTTGTCTGGTGGAGATCATATTCACGCTGGTACCGTAGTAGGTAAACTTGAAGGGGAAAGAGACATCACTTTGGGCTTTGTTGATTTACTGCGTGATGATTTTATTGAAAAAGATCGAAGCCGCGGTATTTATTTCACTCAAGATTGGGTCTCTCTACCGGGTGTTTTGCCTGTAGCTTCAGGAGGTATTCACGTTTGGCATATGCCTGCTCTGACCGAGATCTTTGGAGATGATTCCGTACTACAATTCGGTGGAGGAACTTTAGGGCACCCTTGGGGAAATGCACCTGGTGCCGTAGCTAATCGAGTAGCTCTAGAAGCATGTGTACAAGCTCGTAATGAAGGACGTGATCTTGCTCGTGAGGGTAATGAAATTATCCGTGAGGCTAGCAAATGGAGTCCTGAACTAGCTGCTGCTTGTGAAGTATGGAAGGAGATTAAATTTGAATTCCAAGCGATGGATACTTTGTAA

>lcl|NC_012224.1_cds_YP_002720159.1_67 [gene=ndhF] [locus_tag=JacuC_p068] [db_xref=GeneID:7564793] [protein=NADH dehydrogenase subunit 5] [protein_id=YP_002720159.1] [location=complement(118957..121323)] [gbkey=CDS]
ATGGAACATATATATCAATATTCATGGATCATACCTTTCGTTACGTTCACCGTGCCTATATTAATAGGAGCGGGACTCCTACTTTTTCCGGCAGCAACAAAAAAACTTCGTCGTATATGGGTTTTTCCAAGCGTTTTATTGTTAAATATAGTCATGATTTTTTCAATCGATCTGTCTATTCAGCAAATAAATAGCAGTTTTTTATATCAATATATATGGTCGTGGACTATCAATAATGATTTTTCTTTAGAGTTCGGATACTTGATTGACCCACTTACTTCTATTTTGTCAGTATTAATTACTACAGTTGGAATTTTGGTTCTTTTTTATAGTGATAATTATATGTCTCATGATCAAGGCTATTTGAGATTTTTTGCTTATATGAGTTTTTTCAATACTTCAATGTTGGGATTAGTTACTAGTTCTAATTTGATACAAATTTATATTTTTTGGGAATTGGTTGGAATGTGTTCTTATCTATTAATAGGTTTTTGGTTCACACGACCTATTGCATCAAACGCTTGTCAAAAAGCGTTTGTAACTAATCGTGTAGGGGATTTTGGTTTATTATTAGGAATTTTAGGTCTTTATTGGATAACAGGCAGTTTCGAATTTCGGGATTTGTTCAAAATATTCAATAACTTGATTTCTAATAATCAGGTTAATCTTTTATTTGTTACTTTGTGTGCCTTTCTATTATTTTCCGGTGCAATTGCGAAATCGGCGCAATTTCCTCTTCATGTATGGTTACCGGATGCCATGGAAGGGCCTACTCCTATTTCGGCTCTGATACATGCTGCTACTATGGTAGCGGCGGGGATTTTTCTTATAGCTCGACTTTTTCCTCTTTTTGTAGTCACACCTTACATAATGAATCTAATCGCTTTGATAGGTATAATAACAGTATTTTTAGGAGCTACTTTAGCTCTTGCTCAAAAAGATATTAAGAGAAGTTTAGCCTATTCTACAATGTCTCAATTGGGTTATATGATGTTAGCTCTAGGTATGGGGTCTTATCGAGTCGCTTTATTTCATTTGATTACTCATGCCTATTCGAAGGCATTGTTGTTTTTAGGATCTGGATCGATTATTCATTCAATGGAAGCTATTGTTGGTTATTCTCCAGATAAGAGTCAAAATATGGTTCTGATGGGTGGTTTAACAAAACATATTCCAATTACAAAAACTGCTTTTTTATTAGGAACACTTTCCCTTTGTGGTATTCCACCTTTCGCCTGTTTTTGGTCCAAAGATGAGATTCTTAATGATAGTTGGTTGTATTCACCTATTTTTGCAATAATAGCTTGTTTCACAGCAGGATTAACTGCATTTTATATGTTTCGGGTTTATTTACTTACTTTTGAAGGACATTTAAATGTTCATTTTCAAAATTACAAAAACAGTTCATTTTATTCAATCTCTTTATGGGGTAAAGAAGAATCTAAAATGCTTAACAACAATTTTCGTTTATTAGCTTTATCAGCAATGAATAATAATGAAAGGACTTCTTTTTTTTGGAGGAACACATATCAAATTGGCGGTAATTTAAGAAATATGACATGGCCTTTTATTACTATTAAAAATTTTAATACTAAAAGGGTTTTTTCCTATCCCCATGAATCGGATAATACTATGTTACTTCCTATGCTTGTTTTGGTACTATTTACTTTATTTGTTGGAGCCATAGGAATTCCCTTCAATCAATTCAATGAAGAAGGAATGCAGTTGGATATTTTGTCAAAACTTTTAACTCCGTCTTTAAACCTTTTGTACCAAAACCAAAGTGAGTCTGTGGATTGGTATGAATTTGTAACAAATGCCATTTTTTCGGTCAGTATAGCTTTTTTCGGAATATTTATAGCGTCCTTTTTATATAAGCCCGTTTATTCATCGTTACAAAATTGGAATTTCTTTAATTTGTTCACTAAGTTGACTAAAAAGGGTCCTAATAGAATTCTTTGGGACAAAATACTAAATGTGATATATAATTGGTCCTATAATCGAGGTTACATAGATGCTTTTTATGCAATATCTTTTATTGGGGGTATAAGAAAATTGGCCGAATTAATTCATTTTTTTGATAAAAGAATAATTGATGGAATTATCAATGGAGTCGGTCTTACCAGTTTCTTTGTAGGAGAGGGTATAAAATATGTAGGAAGTGGTCGCATCTCTTCTTATCTCTTATTGTATTTATTTTGTGCATTAATCTTTTTAGTAATTTACTACTTTTTTTCAATTTGTAAAATTGAAAAAAAATTTCAATTATTTATTATATATTCTTTTATTTATTTTTTTTATTTTACTTTATTTATATTTCTTTTTTTTTTTATTTTCTATTTTTTTTTATTTTCTATTTATATATAA

>lcl|NC_012224.1_cds_YP_002720113.1_21 [gene=ycf3] [locus_tag=JacuC_p021] [db_xref=GeneID:7564857] [protein=photosystem I assembly protein Ycf3] [protein_id=YP_002720113.1] [location=complement(join(47666..47815,48502..48729,49421..49549))] [gbkey=CDS]
ATGCCTAGATCCCGGACAACTGGAAATTTTATTGATAAGACCTTTTCAATTGTAGCCAATATCTTATTACGAATAATTCCAACAACTTCGGGAGAAAAAGAGGCATTTACTTATTACAGAGATGGTGTGATGTCTGCTCAATCCGAAGGAAATTATGCAGAAGCTTTACAGAATTATTATGAAGCTTTGCGGCTAGAAATTGATCCCTATGATCGAAGTTATATACTCTATAATATAGGCCTTATTCACACAAGTAATGGAGAACACACAAAAGCTTTGGAATATTATTTTCGGGCACTAGAACGAAACCCCTTCTTACCACAAGCTTTAAATAATATGGCCGTGATCTGTCATTACGGAGAACAGGCCATTCGGCAGGGAGATTCTGAAATTGCGGAAGCTTGGTTCGATCAAGCCGCGGAGTATTGGAAACAAGCTATAGCGCTTACTCCCGGAAATTATATTGAAGCGCAGAATTGGTTGAAGATCACAAGGCGTTTCGAATAA

>lcl|NC_012224.1_cds_YP_002720116.1_24 [gene=ndhK] [locus_tag=JacuC_p024] [db_xref=GeneID:7564864] [protein=NADH dehydrogenase subunit K] [protein_id=YP_002720116.1] [location=complement(54312..54989)] [gbkey=CDS]
ATGAATTCCATTGAGTTTCCTTTACTTGATCAAACAACTAAAATTTCAGTTATTTCAACTACATCAAATGATCTTTCAAATTGGTCAAGACTCTCCAGTTTATGGCCACTTCTCTATGGTACCAGTTGTTGCTTCATTGAATTTGCTTCGTTAATAGGCTCACGATTCGACTTTGATCGTTATGGACTAGTACCAAGATCTAGTCCTAGACAAGCGGACCTGATTTTAACAGCCGGCACAGTAACCATGAAAATGGCTCCCTCTTTAGTGAGATTATATGAACAAATGCCAGAACCAAAATATGTTATTGCTATGGGGGCATGTACAATTACAGGGGGGATGTTCAGTACCGATTCTTATAGTACTGTTCGGGGAGTCGATAAGCTAATTCCTGTAGATGTCTATTTGCCAGGCTGTCCACCTAAACCGGAGGCGGTTATAGATGCTATAACAAAACTTCGTAAAAAAATATCTCGAGAAATTTATGAAGATCGAATTAGGTCTCAACCGGGGAATCGGTGTTTTACTACCAATCACAAGTTTAATATTGAACGCACTACTCATACTGGAAATTATGATCGAGGATTACTCTATCAACCGCCGTCTACTTCAAAGATCCCTCCTGAAACATTTTTCAAATATAAAAGGTCAGTATCGTCCCACGAATTAGTAAATTAG

>lcl|NC_012224.1_cds_YP_002720156.1_64 [gene=ORF126] [locus_tag=JacuC_p064] [db_xref=GeneID:7564779] [protein=ORF126] [protein_id=YP_002720156.1] [location=complement(101219..101599)] [gbkey=CDS]
ATGAATGGGGAGTCCGCTTTGAAAGCGTCCGCCCTGCAACCACCCCCGAGTATATGCTTCAACAGGAATTACACAAGGGTAGTTGATACAATAGAAACCTCTGGTAAAATGCCCGCCCGTAACCCAACAGATAAAGTACATTACATAGTCCGTTTTAGGGATTGGCGACTTACCCATTCAGTGACTTTGGCACTGGATGTTCCAAAAAGAAAATGGGTACTCTCGGGTCGGGTGAATTCAATAATAGACGTCTGTTGGCATTCCAGCCTTCCTTCTCCTTTCAGGGCCTATCCGAAAGAGAATCCAGTACTTCTTGGTCGTGAATATCTGAATAGGACAAACCGCCCCGTGGATATCTTTGCTTCGGAACAAAACAATTAG

>lcl|NC_012224.1_cds_YP_002720118.1_26 [gene=atpE] [locus_tag=JacuC_p026] [db_xref=GeneID:7564868] [protein=atpE] [protein_id=YP_002720118.1] [location=complement(57561..57962)] [gbkey=CDS]
ATGACCTTAAATCTTTGTGTACTGACCCCAAATCGAATTGTTTGGGATTCAGAAGTGAAAGAAATCATTTTATCTACTAATAGTGGGCAAATTGGCGTATTACCAAATCATGCGCCTATTGCCACAGCTGTCGATATCGGTATTTTGAGAATACGCCTTAATGCCAAATGGTTAACGATGGCTCTGATGGGTGGTTTTGCTAGAATAGGCAATAATGAGATTACTGTTTTAGTAAATGATGCAGAGAAGGGTAGTGACATTGATCCACAAGAAGCTCAGCAAACTCTTGAAATAGCAGAAGCTAACTTGAGAAAAGCGGAAGGAAAGAGACAAATAATTGAGGCAAATCTAGCTCTCAGACGAGCTAGGGCACGAGTAGAGGCTATCAATGTGATTTCGTAA

>lcl|NC_012224.1_cds_YP_002720149.1_57 [gene=rpl16] [locus_tag=JacuC_p057] [db_xref=GeneID:7564771] [protein=ribosomal protein L16] [protein_id=YP_002720149.1] [location=complement(join(88089..88487,89883..89891))] [gbkey=CDS]
ATGCTTAGTCCCCAAAGGCCCCGATTCCGCAAACAGCATAGAGGACGAATGAAAGGAAAAGCTTTTCGAGCTAATCGTATTTCTTTCGGTAGATATGCTCTTCAGGCACTTGAACCTTCTTGGATTACATCTAGGCAAATAGAAGCGGGGCGACGAACAATGACACGAAATGCACGTCGCGGTGGAAAAATATGGATACGTATATTTCCCGACAAACCAATTACTTTAAGACCTACGGAAACACGTATGGGTTCGGGGAAAGGATCTCCCGAATATTGGGTAGCTGTCGTTAAACCAGGTAGAATCCTTTATGAAATGGGTGGAGTAGCAGAAAATATAGCGAGAAAATCTATTTCAATAACAGCATCAAAAATGCCTGTACGAACTCAACTCATTATTTCGGTATAG

4. *Manihot esculenta*

>lcl|NC_010433.1_cds_YP_001718482.1_67 [gene=rps7] [locus_tag=MaesCp067] [db_xref=GeneID:5999962] [protein=ribosomal protein S7] [protein_id=YP_001718482.1] [location=complement(102864..103331)] [gbkey=CDS]
ATGTCACGTCGAGGTACTGCAGAAGAAAAAACTGCAAAATCCGATCCAATTTATCGTAATCGATTAGTTAACATGTTGGTTAACCGTATTCTGAAACACGGAAAAAAATCATTGGCTTATCAAATTATCTATCGAGCCATGAAAAAGATTCAACAAAAGACAGAAACAAATCCACTATCTGTTTTACGTCAAGCAATACGTGGAGTAACTCCCGATATAGCAGTAAAAGCAAGACGTGTAGGCGGATCGACTCATCAAGTTCCCATTGAAATAGGATCCACACAAGGAAAAGCACTTGCCATTCGTTGGTTATTAGGGGCATCCCGAAAACGTCCGGGTCGAAATATGGCTTTCAAATTAAGTTCCGAATTAGTGGATGCTGCCAAAGGGAGTGGTGATGCCATACGCAAAAAGGAAGAGACTCATAGAATGGCAGAGGCAAATAGAGCTTTTGCACATTTTCGTTAA

>lcl|NC_010433.1_cds_YP_001718448.1_33 [gene=ycf4] [locus_tag=MaesCp032] [db_xref=GeneID:6000057] [protein=photosystem I assembly protein Ycf4] [protein_id=YP_001718448.1] [location=63384..63938] [gbkey=CDS]
ATGAGTTGGCGATCAGAACGTATATGGATAGAACTTATAGCGGGGTCTCGAAAAACAAGTAATTTCTGCTGGGCCTTTATACTTTTTTTAGGTTCATTGGGATTTTTATTGGTTGGAATTTCCAGCTATCTTGGCAGAAATTTGATATCTTTATTTCCGTCTCAGCAAATAATTTTTTTCCCACAAGGGATCGTGATGTCTTTCTATGGGATCGCCGGTCTATTTATTAGTTCTTATTTGTGGTGCACAATTTTATGGAATATAGGTAGTGGTTATGATCGATTCGATAGAAAAGAAGGAATAGTGTGTATTTTTCGCTGGGGATTTCCTGGAAAAAATCGTCGCATCTTACTACGATTCCTTATGAAAGATATTCAGTCTATTAGAATAGAAGTTAAAGAGCGTATTTATGCTCGGCGTGTCCTTTATATGGAAATCAGAGGCCGGGGGGCTATTCCTTTGACTGGTACTGATGAGAATTTGACTCCACGAGAAATTGAGCAAAAAGTAGCGGAATTGGCCTATTTTTTGCGTGTACCAATTGAAGTATTTTGA

>lcl|NC_010433.1_cds_YP_001718425.1_10 [gene=atpI] [locus_tag=MaesCp009] [db_xref=GeneID:5999986] [protein=ATP synthase CF0 A subunit] [protein_id=YP_001718425.1] [location=complement(15427..16176)] [gbkey=CDS]
ATGAATGTTCTATCATGTTCCATCAACACACTAACACTAAGGGGGTTATATGATATATCCGGTGTGGAAGTAGGCCAGCATTTCTATTGGAAAATAGGAGGTTTCCAAGTCCATGCCCAAGTGCTTATTACTTCTTGGGTTGTAATTGCTATCTTATTAGGTTCGGCCATTGTAGCTGTTCGGAATCCACAAACCATTCCAACTGGAGGTCAGAATTTCTTCGAATACGTCCTTGAATTCATTCGAGATGTGAGCAAAACTCAGATTGGAGAGGAATATGGCCCGTGGGTCCCCTTTATTGGAACTATGTTTCTATTTATTTTTGTTTCTAATTGGGCGGGGGCGCTTTTACCTTGGAAGATCATACAGTTACCTCACGGGGAGTTAGCCGCACCTACGAATGATATAAATACTACCGTTGCTTTAGCTTTACTTACGTCAATAGCATATTTTTATGCGGGTCTTAGCAAAAAAGGATTAGGTTATTTCAGTAAATACATTCAACCAACTCCAATCCTTTTACCCATTAACATTTTAGAAGATTTCACAAAACCTTTATCACTTAGCTTTCGACTTTTCGGCAATATATTAGCGGATGAATTAGTAGTTGTTGTTCTTGTTTCTTTAGTACCTTCAGTGGTTCCTATACCTGTCATGTTCCTTGGATTATTTACAAGTGGTATTCAAGCTCTTATTTTTGCAACTTTAGCTGCGGCTTATATAGGCGAATCCATGGAGGGGCATCATTGA

>lcl|NC_010433.1_cds_YP_001718474.1_59 [gene=rpl16] [locus_tag=MaesCp059] [db_xref=GeneID:5999985] [protein=ribosomal protein L16] [protein_id=YP_001718474.1] [location=complement(join(86282..86680,87740..87748))] [gbkey=CDS]
ATGCTTAGTCCCAAAAGAACCCGATTCCGTAAACAACATAGAGGAAGAATGAAAGGAATAGCTTTTCGAGGTAATCGTATTTGTTTCGGCAGATATGCTCTTCAGGCACTTGAACCCGCTTGGATTACATCTAGACAAATAGAAGCGGGGCGACGAGCAATGACACGAAATGCACGCCGCGGTGGAAAAATATGGGTACGTATATTTCCCGACAAACCAGTTACTTTAAGACCTACGGAAACACGTATGGGTTCGGGGAAAGGATCTCCCGAATATTGGGTAGCTGTCGTTAAACCAGGTAGAATACTTTATGAAATGGGCGGAGTAGCCGAAAATATAGCGAGAAAAGCTATTTCAATAGCAGCATCAAAAATGCCTATACGAACTCAATTCATTATTTCAGGATAG

>lcl|NC_010433.1_cds_YP_001718432.1_17 [gene=psbD] [locus_tag=MaesCp016] [db_xref=GeneID:6000044] [protein=photosystem II protein D2] [protein_id=YP_001718432.1] [location=35317..36378] [gbkey=CDS]
ATGACTATAGCCCTTGGTAAATTTACCAAAGACGAAAATGATTTATTTGATATTATGGATGACTGGTTACGGAGGGACCGTTTCGTTTTTGTAGGTTGGTCCGGTCTATTGCTCTTTCCTTGTGCCTATTTCGCCGTAGGGGGTTGGTTCACAGGTACAACCTTTGTAACGTCATGGTATACCCATGGATTGGCCAGTTCCTATTTGGAAGGCTGCAACTTCTTAACCGCCGCAGTTTCTACTCCTGCTAATAGTTTAGCACATTCTTTGTTATTATTATGGGGTCCTGAAGCACAAGGAGATTTTACTCGTTGGTGTCAATTAGGTGGTTTGTGGACTTTTGTTGCTCTCCACGGTGCTTTTGGACTAATAGGTTTTATGTTACGTCAATTTGAACTTGCTCGATCTGTGCAATTGCGACCTTATAATGCAATCGCATTCTCTGGTCCAATTGCTGTTTTTGTTTCTGTATTCCTGATTTATCCATTAGGCCAGTCTGGTTGGTTTTTTGCGCCTAGTTTTGGTGTAGCAGCTATATTTCGATTCATCCTCTTTTTCCAAGGGTTTCATAACTGGACGCTGAACCCATTTCATATGATGGGAGTTGCCGGCGTATTGGGCGCTGCTCTGCTATGCGCTATTCATGGTGCTACTGTAGAAAATACTTTATTTGAAGATGGTGATGGTGCAAATACATTCCGTGCCTTTAACCCAACTCAAGCTGAAGAAACTTATTCAATGGTCACCGCTAACCGCTTTTGGTCTCAAATCTTTGGGGTTGCTTTTTCCAATAAACGTTGGTTACATTTCTTTATGTTATTTGTACCAGTAACCGGTTTATGGATGAGCGCTCTTGGAGTAGTCGGTCTGGCTCTGAATCTACGTGCCTATGACTTCGTTTCTCAGGAAATCCGTGCAGCAGAAGATCCTGAATTTGAGACTTTCTACACTAAAAATATTCTCTTAAACGAAGGTATTCGTGCTTGGATGGCGGCTCAAGATCAGCCTCATGAAAACCTTATATTCCCTGAGGAGGTTCTACCACGTGGAAACGCTCTTTAA

>lcl|NC_010433.1_cds_YP_001718441.1_26 [gene=ndhK] [locus_tag=MaesCp025] [db_xref=GeneID:6000072] [protein=NADH dehydrogenase subunit K] [protein_id=YP_001718441.1] [location=complement(52077..52754)] [gbkey=CDS]
ATGAATTCCATTGAGTTTCCTTTACTTGATCGAACAACTCAAATTTCAGTTATTTCAACTACATCAAATGATCTTTCAAATTGGTCAAGACTCTCCAGTTTATGGCCACTTCTCTATGGTACCAGTTGTTGCTTCATTGAATTTGCTTCATTAATAGGCTCACGATTCGACTTTGATCGTTATGGGCTAGTACCAAGATCTAGTCCTAGACAAGCGGACCTGATTTTAACAGCCGGCACAGTAACCATGAAAATGGCTCCTTCTTTAGTAAGATTATATGAACAAATGCCTGAACCAAAATATGTTATTGCTATGGGGGCATGTACAATTACAGGGGGAATGTTCAGTACCGATTCTTATAGTACTGTTCGGGGGGTCGATAAGCTAATTCCTGTAGATGTCTATTTGCCAGGCTGTCCACCTAAACCGGAGGCGGTTATAGATGCTATAACAAAACTTCGTAAAAAAATATCTCGAGAAATTTATGAAGATCGAATTAGGTCTCAACCGGGGAAACGGTGTTTTACTACTAATCACAAGTTTAATATTGAACGCACTACTCATACCGGAAATTATGATCAAGAATTACTCTATCAATCGCCGTCTACTTCAAAGATCCCTCCTGAAACATTTTTCAAATATAAAAGGTCAGTATCGTCTAACGAATTAGTAAATTAG

>lcl|NC_010433.1_cds_YP_001718480.1_65 [gene=ycf2] [locus_tag=MaesCp065] [db_xref=GeneID:6000000] [protein=Ycf2] [protein_id=YP_001718480.1] [location=91678..98616] [gbkey=CDS]
ATGAAAGGACATCAATTAAAATCCTGGATTTTCGAATTGAGAGAGATATTGAGAGAGATCAAGTCAGTGGGATCTTTCATTCACATTTTTTTCCATCAAGAACGTTTTATAAAACTCTTGGACTCCCGAATTTGGAGTATCTTACTTTCACGCAATTCACAGGGTTCAACAAGCAATCGATATTTCACGATCAAGGGTGTAGTACTATTTGTAGTAGTGGTCCTTATATATCGTATTAACAATCGAAAGATGGTCGAAAGAAAAAATCTCTATTTGACAGGGCTTCTTCCTATACCTATGAATTCCATTGGACCCAGAAATGATACATTGGAAGAATCCTTTTGGTCTTCCAATATCAATAGGTTGATTGTTTCGCTCCTGTATCTTCCAAAAGGAAAAAAGATCTCTGAGAGCTCTTTCCTGGATCCGAAAGAGAGTACTTGGGTTCTCCCAATAACTAAAAAGTGTATCATGTCTGAATCTAACTGGGGCTCGCGGTGGTGGAGGAACTGGATCGGAAAAAAGAGGGATTCTAGTTGTAAGATATCTAATGAAACCGTCGCTGGAATTGAGATCTCATTCAAAGAAAAAGATATCAAATATCTGGAGTTTCTTTTTGTATATTATATGGATGATCCGATCCGCAAGGACCATGATTGGGAATTGTTTGATCGTCTTTCTCCGAGGAAGGGGCGAAACATAATCAACTTGAATTCGGGACAACTATTCGAAATCTTAGTGAAAGACTGGATTTGTTATCTCATGTTTGCTTTTCGTGAAAAAATACCAATTGAAGTGGAGGGTTTCTTCAAACAACAAGGAGCTGGGTCAACTATTCAATCAAATGATATTGAGCATGTTTCCCATCTCTTCTCGAGAAAGAAGTGGGCTATTTCTTTGCAAAATTGTGCTCAATTTCATATGTGGCAATTCCGCCAAGATCTCTTCGTTAGTTGGGGGAATAATCCGCACGAATCGGATTTTTTGAGGAACATATCGAGAGAGAATTGGATTTGGTTAGACAATGTGTGGTTGGTAAACAAGGATCGGTTTTTTAGCAAGGCACGGAATATATCGTCAAATATTCAATATGATTCCACAAGATCTAGTTTCGTTCAAGGAAGGAATTCTAGCCAATTGAAGGGATCTTCTGATCAATCCAGAGATCATTTCGATTCCATTAGTAATGAGGATTCGGAATATCACACATTGATCAATCAAAGAAAGATTCAACAACTAAAAGAAAGATCGATTCTTTGGGATCCTTCCTTTCTTCAAACGGAACGAACAGAGATAGAATCAGACCGATTCCCTAAATGCCTTTCTGGATATTCCTCAATGTCCCGGCTATTCACGGAAGGTGAGAAGGAGATGAATAATCATCTGCTTCCGGAAGAAATCGAAGAATTTCTTGGGAATCCTACAAGATCCATTCGTTCTTTTTTCTCTGACAGATCGTCAGAACTTCATCTGGGTTCGAATCCTACTGAGAGGTCCACTAGAAATCAGAAATTGTTGAAGAAAGAACAAGATGTTTCTTTTGTCCCTTCCAGGCGATCGGAAAATAAAGAAATAGTTAATATATTCAAGATAATCACGTATTTACAAAATACCGTCTCAATTCATCCTATTTCATCAGATCCGGGATGTGATATGGTTCTGAAGGATGAACTGGATATGGACAGTTCCAATAAGATTTCTTTCTTGAACAAAAATCCATTTTTTGATTTATTTCATCTATTCCATGATCGGAACGGGGGGGGATACACGTTACACCACGATTTTGAATCAGAAGAGAGATTTCAAGAAATGGCAGATCTATTCACTCTATCAATAACCGAGCCGGATCTGGTGTATCATAAGGGATTTACCTTTTTTATTGATTCCTACGGATTGGATCAAAAACAATTCTTGAATGAGGTATTCAACTCCAGGGATGAATCGAAAAAGAAATCTTTATTGGTTCTACCTCCTATTTTTTATGAAGAGAATGAATCTTTTTATCGAAGGATCAGAAAAAAATGGGTCCGGATCTCCTGCGGGAATGATTTGGAAGATCCAAAACAAAAAATAGTGGTATTTGCTAGCAACAACATAATGGAGGCAGTCAATCAATATGGATTGATCCTAAATCTGATTCAAATCCAATATAGTACCTATGGGTACATAAGAAATGTATTGACTCAATTCTTTTTAATGAATAGATCCGATCGCAACTTCGAATATGGAATTCAAAGGGATCAAATAGGAAATGATACTCTGAATCATAGAACTATAATGAAATATACGATCAACCAACATTTATCGAATTTGAAACAGAGTCAGAAGAAATGGTTCGATCCTCTTATTTTTATTTTTCTTTCTCGAACCGAGAGATCCATGAATTGGGATCCTAATGCATATAGATACAAATGGTCTAATGGGAGCAAGAATTTCCAGGAACATTTGGAACATTTCATTTCTGAGCAGAAGAGCCGTTTTCTTTTTCAAGTAGTGTTCGATCGATTACGTATTAATCAATATTCGATTGATTGGTCTGAGGTTATCGACAAAAAAGATTTGTCTAAGTCACTTCGTTTCTTTTTGTCCAAGTTACTTCTTTTTTTGTCCAAGTTTCTTCTCTTTTTGTCTAACTCACTTCCTTTTTTCTTTGTGAGTTTCGGGAATATCCCCATTCATAGGTCCGAAATCCATATCTATGAATTGAAAGGTCCGAATGATCAACTCTGCAATCAGCTGGTAGAACCAATAGGTCTTCAAATCGTTCATTTGAAAAAATTGAAACCCTTCTTATTGTTATTGGATGATCATGATACTTCCCAAAAATCGAAATTTTTGATTAATGGAGGAACAATATCACCATTTTTGTTCAATAAGATACCAAAGTGGATGATTGACTCATTCCATACTAGAAATAATCGCAGGAAATCTTTTGATAACACGGATTCCTATTTCTCAATGATATCCCACGATCAAGACAATTGGCTGAATCCCGTGAAACCATTTCATAGAAGTTCATTGATATCTTCTTTTTATAAAGCAAATCGACTTCGATTCTTGAATAATCTACATCACTTCTGCTTCTATTGTAACAAAAGATTCCCTTTTTATGTGGAAAAGGCCCGTATCAAGAATTATGATTTTACGTATGGACAATTCCTCAATATCTTGTTCATTCGCAACAAGATATTTTCTTTGTGCGGCGGTAAAAAAAAACATGCTTTTTTGGAGAGAGATACTATTTCACCAATCGAGTCACAGGTATCTAACATATTTATACCTAATGATTTTCCACAAAGTGGTAACGAAAGGTATAACTTGTACAAATCTTTCCATTTTCCAATTCGATCCGATCCATTCGTTCGTAGAGCTATTTATTCGATCGCAGACATTTCTGGAACACCTCTAACAGAGGGACAAATAGTCAATTTTGAAAGAACTTATTGTCAACCTCTTTCGGATATGAATCTATCTGATTCAGAAGGGAAGAACTTGCATCAGTATCTCAATTTCAATTCAAACATGGGTTTGATTCACACTCCATGTTCTGAGAAATATTTACCATCCGAAAAGAGGAAAAAACGGAGTCTTTGTCTAAAGAAATGTGTTGAAAAAGGGCAGATGTATAGAACCTTTCAACGAGATAATGCTTTTTCAACTCTCTCAAAATGGAATCTATTCCAAACATATATGCCATGGTTCCTTACTTCGACGGGGTACAAATATCTAAATTTGATATTTTTAGATACCTTTTCGGACCTATTACCGATACTAAGTAGCAGTCAAAAATTTTTATCCATTTTTCATGATATTATGCATGGATCAGATATATCATGGCTAATTTTTCAGAAAAGATTGTGGAAGATATGCCGGAATCTGATAAGTGAGATTTCGAGTAAGTGTTTACATAATCTTCTTCTGTCCGAAGAAATGATTCATCGAAATAATGAGCCACCATTGATATCGACACATCTGAGATCGCCAAATGTTCGGGAGTTCCTCTATTCAATCCTTTTCCTTCTTCTTGTTGCTGGATATCTCGTTTGTACACATCTTCTCTTTGTTTCCCACGCCTATAGTGAGTTACAGACAGAGTTCGAAAAGGTCAAATCTTTGATGATTCCATCATACATGATTGAGTTGCGAAAACTTCTGGATAGGTATCCTACATCTGAACTGAATTCTTTCTGGTTAAAGAATCTCTTTCTAGTTGCTCTGGAACAATTAGGAGATTTTCTAGAAGAAATGCGGGGTTCTGCTTCTGGCGGCAACATGCTATGGGGTGGTGGTCCCGCTTATGGGGTTAAATCAATACGTTCTAAGAAGAAATTTTGGAATATCAATCTCATCGATCTCATAAGTATCATACCAAATCCCATCAATCGAATCACTTTTTCGAGAAATACGAGACATCTAAGTCATACAAGTAAAGAGATTTATTCATTGATAAGAAAAAGAAAAAACGTGAACGGTGATTGGATTGATGATAAAATAGAATCCTTGGTCGCGAACAGTGATTGGATTGATGATAAAGAAAGAGAATTCTTGGTTCAGTTCTCCACCTTAACGACAGAAAAAAGGATTGATCAAATTCTATTGAGTCTGACTCATAGTGATCATTTATCAAAGAATGACTCTGGTTATCAAATGATTGAAGAGCCGGGAGCAATTTATTTACGATACTTAGTTGACATTCATAAAAAGTATCTAATGAATTATGAGTTCAACACACCCTGTTTAGCAGAAAGACGGATATTCCTTGCTTATTATCAGACAACCACTTATTCACAAACCTCGTGTGGGGTGAATAGTTTTCATTTCCCATCTCATGGAAAACCCTTTTCGCTCCGCTTAGCCCTATCCCCCTCTAGGGGTATTTTAGTGATAGGTTCTATAGGAACTGGACGATCCTATTTGGTCAAATACCTAGCGACAAACTCCTATCTTCCTTTCGTTACAGTATTTCTGAACAAGTTCCTGAATAACAAGCCTAAGGGTTTTCTTATTGATGATAGTGACGATATTGATGATAGTGACGATATTGATGATAGTGACGATATTGATGCTAGTGACGATATTGATGTGAGTGACGATATTGATGTGAGTGACGACGATATCGACCGTGACTTTGACTTTGATACGGAGCTGGAGTTTCTAACTACGATGGATGCGCTAACTATTGATATGATGCCGGAAATAGAAATAAACCGATTTTATATCACCCTTCAATTCGAATTAGCAAAAGCAATGTCTCCTTGCATAATATGGATTCCAAACATTCATGATCTGGATGTGAATGAGTCGAATTACTTATCCCTCGGTCTATTAGTGAACTATCTCTCCAGGGATTGTGAAAGATGTTCCACTAGAAATATTCTTGTTATTGCTTCGACTCATATTCCCCAAAAAGTGGATCCCGCTCTAATAGCTCCGAATAAATTAAATACATGCATTAAGATACGAAGGTTTCTTATTCCACAACAACGAAAGCACTTTTTTACTCTTTCATATACTAGGGGATTTCACTTGGAAAATAAAATGTTCCATACTAATGGATTCGGGTCCATAACCATGGGTTCCAATGTACGAGATCTTGTAGCACTTACCAATGAGGCCCTATCGATTAGTATTACACAGAAGAAATCAATTATAGACACTAATATAATTAGATCTGCTCTTCATAGACAAACTTGGGATTTGCGATCCCGGGTAAGATCGGTTCAGGATCATGGGATCTTTTTCTATCAGATAGGAAGGGCTGTTGCACAAAATGTATTTCTAAGTAATTGCCCCATAGATCCTATATCTATCTATATGAAGAAGAAATCATGTAACGAAGGGGATTCTTATTTGTACAAATGGTACTTCGAACTTGGAACGAGCATGAAGAAATTAACGATACTTCTTTATCTTTTGAGTTGTTCTGCCGGATCGGTTGCTCAAGACCTTTGGTCTCTACCCGGACCCGATGAAAAAAATGGGATCACTTATTATGGACTTGTTGAGAATGATTCTGATCTAGTTCATGGCCTATTAGAAGTAGAAGGCGCTCTGGTGGGATCCTCACGGACAGAAAAAGATTGCAGTCAGTTTGATAATGATCGAGTGACATTGCTTCTTCGGCCCGAACCAAGGAGTCCCTTAGATATGATGCAAAATGGATCTTGTTCTATCCTTGATCAGAGATTTCTCTATGAAAAATACGAATCGGAGTTTGAAGAAGGGGAAGGAGAAGAAGTCCTCGACCCGCAACAGATAGAGGAGGATTTATTCACTCACATAGTTTGGGCTCCTAGAATATGGCGCCCTTGGGGTTTTCTATTTGATTGTATTGAAAGGCCCAATGAATTGGGATTTCCCTATTGGGCCAGGTCATTTCGGGGCAAGCGGATCATTTATGATGAAGAGATCATTTATGATGAAGAGATCATTTATGATGAAGAGGATGAGCTTCAAGAGAATGATTCGGAGTTCTTGCAGAGTGGAACCATGCAGTACCAGATACGAGATAGATCTTCCAAAGAACAAGGCTTTTTTCGAATAAGCCAATTCATTTGGGACCCTGCGGATCCACTCTTTTTCCTATTCAAAGATCAGCCCTTTGTCTCTGTGTTTTCACATCGAGAATTCTTTGCAGATGAAGAGATGTCAAAGGGGCTTCTTACTTCCCAAACAGATCCTCCTACATCTATATATAAACGCTGGTTTATCAAGAATACGCAAGAAAAGCGCTTCGAATTGTTGATTCATCGCCAGAGATGGCTTAGAACCAATAGTTCATTATCTAATGGATTTTTCCGTTCTAATACTCTATCCGAGAGTTATCAGTATTTATCAAATCTGTTCCTATCTAACGGAACGCTATTGGATCAAATGACAAAGGCATTGTTGAGAAAAAGATGGCTTTTCCCGGATGAAATGAAAATTGGATTCATGTAA

>lcl|NC_010433.1_cds_YP_001718436.1_21 [gene=psaB] [locus_tag=MaesCp020] [db_xref=GeneID:6000030] [protein=photosystem I P700 chlorophyll a apoprotein A2] [protein_id=YP_001718436.1] [location=complement(40072..42276)] [gbkey=CDS]
ATGGCATTAAGATTTCCAAGGTTTAGCCAAGGCTTAGCTCAGGACCCCACTACTCGTCGTATTTGGTTTGGTATTGCTACCGCGCATGACTTCGAGAGTCATGATGATATTACGGAGGAACGTCTTTATCAGAATATTTTTGCTTCTCACTTCGGGCAATTAGCAATAATTTTTCTGTGGACTTCCGGAAATCTCTTTCATGTAGCTTGGCAAGGAAATTTTGAAGCATGGGTACAGGACCCTTTACATGTAAGACCTATTGCTCATGCAATTTGGGATCCTCATTTTGGTCAACCGGCCGTGGAAGCTTTTACTCGAGGGGGTGCTCCTGGCCCAGTGAATATCGCTTATTCTGGTGTTTATCAATGGTGGTATACAATCGGTTTACGTACTAATGAAGATCTTTATATTGGAGCTCTTTTTCTATTATTTCTTTCTGCCCTAGCCTTACTAGGGGGTTGGTTACACCTACAACCAAAATGGAAACCGAGCGTTTCGTGGTTCAAAAATGCCGAATCTCGTCTCAATCATCATTTGTCAGGACTATTCGGAGTAAGCTCTTTGGCTTGGACAGGACATTTAGTCCATGTCGCTATTCCCGGCTCCCGGGGGGAATACGTTCGATGGAATAATTTCTTAGATGTATTACCACATCCCCAAGGGTTAGGCCCGTTTTTTACAGGTCAGTGGAATCTTTATGCTCAAAATCCCGATTCAGGTAGTCATTTATTTGGTACCTCCCAAGGAGCGGGAACTGCCATTCTAACCCTTCTCGGGGGGTTCCATCCACAAACACAAAGTTTATGGCTGACCGATATTGCACACCATCATTTAGCTATTGCATTTATTTTTCTCGTTGCCGGTCATATGTATAGAACTAACTTCGGGATTGGGCATAGTATAAAAGATCTTTTAGAAGCGCATATTCCTCCGGGGGGGCGATTGGGGCGTGGACATAAGGGTCTTTATGACACAATCAACAATTCGCTTCATTTTCAATTAGGCCTTGCTCTAGCTTCTTTAGGGGTTATTACTTCCTTAGTGGCTCAACACATGTACTCATTACCTGCTTATGCGTTCATAGCGCAAGACTTTACTACTCAAGCTGCGTTATATACTCATCACCAATACATCGCAGGATTCATCATGACAGGAGCTTTTGCTCATGGAGCTATATTTTTTATTAGAGATTACAATCCGGAACAGAATGAGAATAATGTATTGGCAAGAATGTTAGACCATAAAGAAGCTATCATATCCCATTTAAGTTGGGCCAGTCTCTTTCTTGGATTCCATACTTTGGGACTTTATGTTCATAATGATGTCATGCTTGCTTTTGGTACTCCGGAGAAACAAATCTTAATCGAACCCATATTCGCCCAATGGATACAATCTGCTCACGGTAAAACTTCATATGGGTTCGATGTACTTTTATCTTCAACGAATAGTCCAGCCTTCAATGCAGGTCGAAGCATATGGTTGCCCGGCTGGTTAAATGCTATTAATGAAAATAGTAATTCATTATTCTTAACAATAGGGCCTGGAGACTTCTTGGTTCATCATGCTATTGCTCTAGGGTTACATACAACCACATTGATCTTAGTAAAAGGTGCTTTAGATGCACGCGGTTCGAAGTTAATGCCAGATAAAAAGGATTTTGGTTATAGTTTTCCTTGTGATGGTCCGGGACGCGGCGGTACTTGTGATATTTCGGCTTGGGACGCATTTTATTTGGCGGTTTTCTGGATGTTAAATACCATTGGATGGGTTACTTTTTATTGGCATTGGAAGCACATCACATTATGGCAGGGTAATGTTTCACAGTTTAATGAATCTTCCACTTATTTGATGGGATGGTTAAGAGATTATCTATGGTTAAACTCTTCACAACTTATCAATGGATATAACCCTTTTGGTATGAATAGCTTATCGGTCTGGGCGTGGATGTTCTTATTTGGACATCTTGTTTGGGCTACTGGATTTATGTTTTTAATTTCTTGGCGCGGATATTGGCAAGAATTGATTGAAACTTTAGCATGGGCTCATGAGCGTACACCTTTGGCTAATTTGATTCGATGGAGAGATAAACCAGTAGCTCTTTCCATTGTGCAAGCAAGATTGGTTGGATTAGCCCACTTTTCCGTAGGTTATATCTTCACTTATGCGGCTTTCTTGATTGCCTCTACATCAGGTAAATTTGGTTAA

>lcl|NC_010433.1_cds_YP_001718435.1_20 [gene=rps14] [locus_tag=MaesCp019] [db_xref=GeneID:6000050] [protein=ribosomal protein S14] [protein_id=YP_001718435.1] [location=complement(39647..39949)] [gbkey=CDS]
ATGGCAAGGAAAAGTTTGATTCAGCGGGAGAAGAAGAGGCAAAAATTGGAACAAAAATATCATTTGATGCGTCGATCCTCAAAAAAAGAAATAAGCAAAGTTCCGTCGTTAAGTGATAAATGGGAAATTCATGGAAAGTTACAATCCCCACCGCGGAATAGTGCACCGACACGTCTTCATCGACGTTGTTTTTCGACTGGAAGACCGAGAGCTAATTATCGAGACTTTGGGCTATCCGGACACATACTTCGTGAAATGGTTCATGCATGTTTGTTGCCGGGGGCAACAAGATCAAGTTGGTAA

>lcl|NC_010433.1_cds_YP_001718418.1_3 [gene=matK] [locus_tag=MaesCp002] [db_xref=GeneID:5999999] [protein=maturase K] [protein_id=YP_001718418.1] [location=complement(2063..3583)] [gbkey=CDS]
ATGGAGGAAAGATATTTAGAATTAGATAGATCTCGAAAAAACGACTTCCTATACCCATTTATCTTTCGGGAGTATATTTATACATTCGCTCATGATCATAGTTTAAATAGATCTATTTTGTTGGAAAATGTAGGTTATGACAATAAATCTAGTTTATTAATTGTAAAACGTTTAATTACTCGAATGTATCAACAGAATCATTTGATTATTTCTGCTAATGATTCTAACCAAAATCTATTTTTTAGGTACAACAAGAATTTGTATTATCAAATGATATCAGAGGGCTTTGCAGTTATTGTGGAAATTCCATTTTCCCTACGATTAGTATCTTCTTTAGATTTAGAAAGGTCAGAGATAGTAAAATCTCATAAATTACGATCAATTCATTCAATATTTCCTTTTTTAGAGGACAAATTTCCACATTTAAATTATGTGTCAGATATATTAATACCTTACCCCATCCATCTAGAAAAATTGGTTCAAACCCTTCGCTATTGGGTGAAAGATCCCTCTTCTTTGCATTTATTACGACTCTTTCTTCATGAGTATTGGAATTTGAACAGTCTTATTATTCCAAAGAAATTTATTACTATTTTTATAAAAAGGAATCCAAGATTTTTCTTGTTCCTATATAATTCTCATGTATATGAATACGAATCCATCTTCTTTTTTCTCCGTAACCAATCCTTTCATTTACGATCAATATTTTTGCGAGTCCTTCTTGAACGAATTTTTTTCTATGGAAAAATAGAACATTTTGCGGAAGTCTTTGCTAATGATTTTCAGGCCATCTTGTGGTTGTTCAAGGATCCTTTCATGCATTATGTTAGATATCAAGGAAAATCAATTCTGGCTTCAAAAGATCGGCCTTTTCTGATGAAAAAATGGAAATATTACCTTGTCAACTTATGTCAATGTCATTTTTATGTCTGGTTTCAACCAGAAAAGATCTATATAAATTCATTATCCAAGCATTCTCTCAACTTTTTGGGCTATCTTTCAAATGTACAATTAAATCCTTTGGTGGTACGGAGTCAAATGTTAGAAAATTCATTTATAATAGATAAAGATAGTACTATGAAGAAACTCGATACAATAGTTCCAATTATTCCTTTAATTGGATCATTGGCAAAAACGAAATTTTGTAACGCAGTAGGACATCCCATTAGTAAACCGATTCGGGCGGATTCGGCGGATTCTGATATTATCGACCGATTTGTGCGTATATGCAGAAATCTTTCTCATTATTATAGCGGATCCTCAAAAAAAAAGAGTTTGTATCGAATAAAATATATACTTCGACTTTCTTGTGTTAAAACTTTGGCCCGTAAACACAAAAGTACTGTACGCGCTTTTTTGAAAAGATTAGGTTCGGAATTATTAGAAGAGTTTTTTACGGAGGAAGAACAGATTCTTTCTTTGATCTTTCCAAAAGTTTCTTCTAGTTCGCGCAGGTTATATAGAGGACGTGTTTGGTATTTGGATATTATTTCTATCAATGATTTGGCCAATCATGAATAA

>lcl|NC_010433.1_cds_YP_001718485.1_70 [gene=ccsA] [locus_tag=MaesCp071] [db_xref=GeneID:6000045] [protein=cytochrome c biogenesis protein] [protein_id=YP_001718485.1] [location=120604..121572] [gbkey=CDS]
ATGATATTCTCGACTTTAGAACATATATTAACACATATATCTTTTTCAGTCGTGTCAATTGTAATTACAATTCATTTGATAACCTTATTAGCCGATGAATTCGTAGAACTATATGATTCGTCAGAAAAGGGCATGATAACTACTTTTTTTTGTATAACAGGATTATTAGTTACTCGTTGGATTTTTTTGGGACATTTACCATTAAGTGATTTATATGAATCATTAATCTTTCTTTCATGGTCTTTTTCCATTATTCATATGGTTCCGTATTTTAAAAAACATAAAAATTTTTTAAGCGCAATAACCGCGCCAAGTACTTTTTTTACCCAAGGGTTTGCTACTTCGGGTCTTTTAACTGACATGCATCAATCCGAAATCTTAGTGCCCGCTCTCCAATCCCAGTGGTTAATGATGCACGTAAGTATGATGATATTGGGCTATGCAGCTCTTTTGTGTGGATCATTATTTTCAGTAGCATTTCTAGTAATCACATTTCGAAAAATCATAAGAATTTTTGATAAAAGCAATAATTTATTAAACAATTCGTTTTTCTTTAGTGAGATACAATATATGGCGGAAAGAAAGAATGTTTTAAGAAATATTTCTTTTCTTTCTTCTAGGAATTATTACAGGTTTCAATTGATTCAACAATTAGATGACTGGGGTTATCGTATTATAAGTATAGGGTTTATCTTTTTAACAATAGGTATTCTTTCGGGAGCAGTCTGGGCTAATGAAGCATGGGGATCGTATTGGAATTGGGACCCAAAAGAAACTTGGGCATTTATTACGTGGACCATATTCGCGATTTATTTCCATATTCGAACAAATAAAAAATTGGAGGGTTTCCATTCCGCAATTGTCGCTTCTATCGGTTTTCTTCTAATTTGGATATGCTATTTTGGAGTTAATTTATTAGGAATAGGACTACATAGTTATGGTTCATTTACATTAACAATTAGTATCTAA

>lcl|NC_010433.1_cds_YP_001718491.1_76 [gene=ndhA] [locus_tag=MaesCp077] [db_xref=GeneID:5999995] [protein=NADH dehydrogenase subunit 1] [protein_id=YP_001718491.1] [location=complement(join(126097..126636,127760..128311))] [gbkey=CDS]
ATGATAATTGATACAACAGAAGTACAAGCTATCAATTCTTTTTCTAGATTAGAATCCTTAAACGAGGTCTATGGAATTATATGGGTGTTTGCCCCGATTTTTATTCTTGTATTGGGAATCACGACAGGCATACTAGTAATTGTATGGTTAGAAAGAGAAATATCTGCAGGGATACAACAACGTATTGGACCTGAATATGCCGGTCCTTTAGGAGTTCTTCAAGCTCTAGCGGATGGGACAAAACTACTTTTCAAAGAGAATCTTTTTCCATCTAGGGGGGATATTCGTTTATTCAGTATCGGACCATCCATAGCAGTCATATCAACTATATTAAGCTATTCGGTAATTCCTTTTGGCTATCACCTTGTTTTAACTGATCTAAATATTGGTGTTTTTTTATGGATTGCCATTTCAAGTATTGCTCCCATCGGACTTCTTATGTCAGGATATGGATCAAATAATAAATATTCCTTTTTGGGTGGTTTACGAGCTGCTGCTCAATCGATTAGTTATGAAATACCATTAACTCTTTGTGTGTTATCCATATCTCTATTATCTAATAGTTCAAGTACAGTTGATATAGTTGAGGCACAATCAAAATCTGGTTTTTGGGGGTGGAATTTGTGGCGTCAACCTATAGGATTTATCATTTTTTTTATTTCTTCTCTAGCAGAATGTGAGAGATTACCTTTTGATTTACCAGAAGCAGAAGAAGAATTAGTAGCAGGTTATCAAACCGAATATTCGGGCATCAAATTTGGTTTATTTTATATTGCTTCCTATCTAAACTTATTAGTTTCTTCATTATTTGTAACAGTTCTTTACTTGGGCGGTTGGAATATCTCTATTCCGTATATATTCGTTCCTGAGCTTTTTGAAATAAAAAAAATGAGCGGAGTCTTTGGAACAACAATTGGTATCTTTATTACATTGGTTAAAACTTATTTGTTCTTGTTCATTCCTATCACAACAAGATGGACTTTACCTAGACTAAGAATGGACCAACTTTTAAATCTTGGATGGAAATTTCTTTTACCTATTTCTCTCGGTAATCTATTATTAACAACCTCTTTCGAACTTCTTTCACTATAA

>lcl|NC_010433.1_cds_YP_001718444.1_29 [gene=atpB] [locus_tag=MaesCp028] [db_xref=GeneID:5999989] [protein=ATP synthase CF1 beta subunit] [protein_id=YP_001718444.1] [location=complement(55809..57311)] [gbkey=CDS]
ATGAGAATCAATCCTACTACTTCTACTTCTGGTCCGGGAGTTTCCGCGCTTGAAAAAAAGAACCTGGGGCGTATCGCTCAAATCATCGGGCCAGTGCTAGATGTAGCTTTTCCCCCGGGCAAGATGCCTAATATTTACAACGCTCTGGTAGTTAAGGGTCGAGATACTGCCGGTCAAGAAATTAATGTGACTTGTGAAGTACAACAATTATTAGGAAATAATCGAGTTCGGGCTGTAGCTATGAGTGCTACAGATGGTCTAACGAGAGGAATGGAAGTGATTGACACAGGAGCCCCTCTAAGTGTTCCAGTCGGTGGGGCAACTCTAGGACGAATTTTCAACGTGCTTGGAGAACCTGTTGACGATTTAGGTCCTGTAGATACTCGCGCAACATCCCCTATTCATAGATCTGCACCTGCCTTTATACAGTTAGATACAAAATTATCTATTTTTGAAACAGGAATTAAAGTAGTAGATCTTTTAGCCCCTTATCGCCGTGGAGGAAAAATCGGGCTATTCGGGGGAGCTGGAGTGGGTAAAACAGTACTTATTATGGAATTAATCAATAACATTGCGAAAGCTCATGGGGGTGTATCCGTATTTGGCGGAGTAGGCGAACGTACTCGTGAAGGAAATGATCTTTACATGGAAATGAAAGAATCTGGAGTAATTAATGAAGAAAATATTGCAGAATCAAAAGTGGCTCTAGTCTATGGTCAGATGAACGAACCGCCGGGAGCTCGTATGAGAGTTGGTTTGACTGCCCTAACTATGGCGGAATATTTTCGAGATGTTAATGAACAAGACGTACTTCTATTTATCGACAATATCTTCCGTTTCGTCCAAGCAGGATCCGAAGTATCCGCCTTATTGGGTAGAATGCCTTCCGCTGTGGGTTATCAACCTACCCTTAGTACCGAAATGGGCTCTTTACAAGAAAGAATTACTTCTACCAAAGAAGGGTCCATAACTTCTATTCAAGCAGTTTATGTACCTGCGGACGATTTGACTGATCCTGCTCCTGCCACGACATTTGCACATTTAGATGCTACTACTGTACTATCAAGAGGATTAGCTGCTAAAGGTATCTATCCAGCAGTAGATCCTTTAGATTCAACGTCAACTATGCTCCAACCTCAGATTGTTGGTGAGGAACATTATGAAACTGCGCAAAGAGTTAAGCAAACTTTACAACGTTACAAAGAACTTCAGGACATTATAGCTATCCTTGGGTTGGACGAATTATCCGAAGAGGATCGCTTAACTGTAGCAAGAGCACGAAAAATTGAGCGTTTCTTATCACAACCCTTTTTCGTAGCAGAAGTATTTACCGGTTCTCCGGGGAAATATGTCGGTCTAGCAGAAACAATTAGAGGGTTTAAATTGATCCTTTCGGGAGAATTAGATAGTCTCCCTGAGCAGGCCTTTTATTTGGTAGGTAATATTGATGAAGCTACTGCGAAGGCTACGAACTTAGAAATGGAGAACAACTTGAAGAAATGA

>lcl|NC_010433.1_cds_YP_001718442.1_27 [gene=ndhC] [locus_tag=MaesCp026] [db_xref=GeneID:6000062] [protein=NADH dehydrogenase subunit 3] [protein_id=YP_001718442.1] [location=complement(52815..53177)] [gbkey=CDS]
ATGTTTCTGATTTACGAATATGATATATTCTGGGCATTTCTAATAATATCAAGTGTTATTCCTATTTTAGCATTTCTAATTTCCGGAGTTTTATCCCCGATTAGCAAAGGGCCGGAGAAACTTTCTAGTTATGAATCGGGTATAGAACCAATAGGCGATGCTTGGTTACAATTTCGAATCCGTTATTATATGTTTGCTCTAGTTTTTGTTGTTTTTGATGTTGAAACAGTTTTTCTTTATCCATGGGCAATGAGTTTCGATGTATTGGGGTTATCCGTATTTATAGAAGCTTTAATTTTCGTGCTTATCCTAATTGTTGGTTCAGTTTATGCATGGAGAAAAGGAGCATTAGAATGGTCTTAG

>lcl|NC_010433.1_cds_YP_001718495.1_80 [gene=rps7] [locus_tag=MaesCp081] [db_xref=GeneID:6000063] [protein=ribosomal protein S7] [protein_id=YP_001718495.1] [location=147378..147845] [gbkey=CDS]
ATGTCACGTCGAGGTACTGCAGAAGAAAAAACTGCAAAATCCGATCCAATTTATCGTAATCGATTAGTTAACATGTTGGTTAACCGTATTCTGAAACACGGAAAAAAATCATTGGCTTATCAAATTATCTATCGAGCCATGAAAAAGATTCAACAAAAGACAGAAACAAATCCACTATCTGTTTTACGTCAAGCAATACGTGGAGTAACTCCCGATATAGCAGTAAAAGCAAGACGTGTAGGCGGATCGACTCATCAAGTTCCCATTGAAATAGGATCCACACAAGGAAAAGCACTTGCCATTCGTTGGTTATTAGGGGCATCCCGAAAACGTCCGGGTCGAAATATGGCTTTCAAATTAAGTTCCGAATTAGTGGATGCTGCCAAAGGGAGTGGTGATGCCATACGCAAAAAGGAAGAGACTCATAGAATGGCAGAGGCAAATAGAGCTTTTGCACATTTTCGTTAA

>lcl|NC_010433.1_cds_YP_001718478.1_63 [gene=rpl2] [locus_tag=MaesCp063] [db_xref=GeneID:6000022] [protein=ribosomal protein L2] [protein_id=YP_001718478.1] [location=complement(join(89524..89958,90632..91030))] [gbkey=CDS]
ATGGCGATACATTTATACAAAACTTCTACCCCGAGCACACGCAATGGAGCCGTAGACAGTCAAGCGAAATCCAATACACGAAATACACGAAAGAATTTGATCTATGGACAGCATCGTTGTGGTAAAGGCCGTAATGCCAGAGGAATCATTACCGCAAGACATAGAGGGGGAGGTCATAAGCGTCTATACCGTAAAATCGATTTTCGACGGAATGAAAAAGACATATATGGTAGAATCGTAACCATAGAATACGACCCTAATCGAAATGCATACATTTGTCTCATACACTATGGGGATGGTGAGAAGAGATATATTTTACATCCCAGAGGGGCTATAATTGGAGATACCATTATTTCTGGTACAGAAGTTCCTATAAAAATGGGAAATGCCCTACCTTTGACCGATATGCCCTTAGGCACGGCCATACATAACATAGAAATCACACTTGGAAAGGGTGGACAATTAGCTAGAGCTGCAGGTGCTGTAGCGAAACTGATTGCAAAAGAGGGGAAATCAGCCACATTAAAATTACCTTCTGGGGAGGTTCGTTTAATATCCAAAAACTGCTCAGCAACAGTCGGACAAGTAGGGAATACTGGGGTGAACCAGAAAAGTTTGGGTAGAGCCGGATCTAAATGTTGGCTAGGTAAGCGTCCTGTAGTAAGAGGAGTAGTTATGAACCCTGTAGACCACCCCCATGGGGGTGGTGAAGGGAGGGCCCCAATTGGTAGAAAAAAACCCGCAACCCCTTGGGGTTATCCTGCACTTGGAAGAAGAAGTAGAAAAAGGAATAAATATAGTGATAATTTGATTCTTCGTCGCCGTAGTAAATAG

>lcl|NC_010433.1_cds_YP_001718481.1_66 [gene=ndhB] [locus_tag=MaesCp066] [db_xref=GeneID:6000013] [protein=NADH dehydrogenase subunit 2] [protein_id=YP_001718481.1] [location=complement(join(100339..101094,101777..102553))] [gbkey=CDS]
ATGATCTGGCATGTACAGAATGAAAACTTCATTCTCGATTCTACGAGAATTTTTATGAAAGCCTTTCATTTGCTTCTCTTCGATGGAAGTTTTATTTTCCCAGAATGTATCCTAATTTTTGGCCTAATTCTTCTTCTGATGATCGATTCAACCTCTGATCAAAAAGATATACCTTGGTTATATTTCATCTCTTCAACAAGTTTAGTAATGAGTATAACGGCCCTATTGTTCCGATGGAGAGAAGAACCTATGATTAGCTTTTCGGGAAATTTCCAAACGAACAATTTCAACGAAATCTTTCAATTTCTTATTTTACTATGTTCAACTCTATGTATTCCTCTATCCGTAGAGTACATTGAATGTACAGAAATGGCTATAACAGAGTTTCTCTTATTCGTATTAACAGCTACTCTAGGAGGAATGTTTTTATGCGGTGCTAACGATTTAATAACTATCTTTGTCGCTCCAGAATGTTTCAGTTTATGCTCCTACCTATTATCTGGATATACCAAGAAAGATGTACGGTCTAATGAGGCTACTACGAAATATTTACTCATGGGTGGGGCAAGCTCTTCTATTCTGGTTCATGCTTTCTCTTGGCTATATGGTTCGTCCGGGGGAGAGATCGAGCTTCAAGAAATAGTGAATGGCCTTATCAATACACAAATGTATAACTCCCCAGGAATTTCAATTGCGCTTATATTCATCACTGTAGGAATTGGGTTCAAGCTTTCCCTAGCCCCTTCTCATCAATGGACTCCTGACGTATACGAAGGATCTCCCACTCCAGTCGTTGCTTTTCTTTCTGTTACTTCGAAAGTAGCTGCTTCAGCTTCAGCCACTCGAATTTTCGATATTCCTTTTTATTTCTCATCAAACGAATGGCATCTTCTTCTGGAAATCCTAGCTATTCTGAGCATGATAGTGGGGAATCTCATTGCTATTACTCAAACAAGCATGAAACGTATGCTTGCATATTCGTCCATAGGTCAAATCGGATATGTAATTATTGGAATAATTGTTGGAGACTCTAATGGTGGATATGCAAGCATGATAACTTATATGCTCTTCTATATCTCCATGAATCTAGGAACTTTTGCTTGTATTGTATTATTTGGTCTACGTACCGGAACTGATAACATTCGAGATTATGCAGGATTATACACGAAAGATCCTTTTTTGGCTCTCTCTTTAGCCCTATGTCTCTTATCCCTAGGAGGTCTTCCTCCACTAGCAGGTTTTTTCGGAAAACTCCATTTATTCTGGTGTGGATGGCAGGCAGGCCTATATTTCTTGGTTTTAATAGGACTCCTTACGAGCGTTGTTTCTATCTACTATTATCTAAAAATAATCAAGTTATTAATGACTGGACGAAACCAAGAAATAACCCCTCACGTGCGAAATTATAGAAGATCCCCTTTAAGATCAAACAATTCCATCGAATTGAGTATGATTGTATGTGTGATAGCATCTACTATACCAGGAATATCAATGAACCCGATTGTTGAAATTGCTCAAGATACCCTTTTTTAG

>lcl|NC_010433.1_cds_YP_001718494.1_79 [gene=ycf1] [locus_tag=MaesCp080] [db_xref=GeneID:5999984] [protein=hypothetical chloroplast RF1] [protein_id=YP_001718494.1] [location=complement(130312..135882)] [gbkey=CDS]
ATGATTTTTCAATCTTTTATACCAGGTAATCTAGTATCCTTATGCATGAAGATAATCAATTCGGTCGTTGTGGTCGGACTCTATTATGGATTTCTGACCACATTCTCCATGGGGCCTTCTTATCTCTTCCTTCTCCGAGCTCGGGTTATAGAAGAAGGAGAAGAAGGAACTGAGAAGAAGGTATCAGCAACAACAGGTTTTATTACGGGACAGCTCATGATGTTCATATCGATCTATTATGCGCCTCTGCATCTAGCATTGGGTAGACCTCATACAATAACTGTCCTAGCTCTACCCTATCTTTTGTTTCATTTCTTCTGGAACAATCACAAACACTTTTTTGATTATGGATCTACTACCAGAAATTCAATGCGTAATCTTAGCATTCAATTTGTATTCCTGAATAATCTCATTTTTCAATTATTCAACCATTTCATTTTACCAAGTTCAATGTTAGTCAGATTAGTCAACATTTATATGTTTCGATGCAACAACAAGATGTTATTTGTAACAAGTAGTTTTGTTGGTTGGTTAATTGGTCACATTTTATTCATGAAATGGGTTGGATTGATATTAGTCTGGATACAGCAAAATAATTCTATTAGATCTAATGTACTTTTTCGATCTAATAAGTACCTTGTGTCAGAATTGAGAAATTCTAGGGCTCGAATCTTTAGTATTCTCTTATTTATTACCTGTGTCTACTCTTTAGGCAGAATACCGTCACCCATTTTTACTAAGAAACTGAAAGAAACCTCAGAAACGGAAGAAAGGGAGGAAGAAACAGATGTAGAAATAGAAAAAACTTCCGAAACGAAGGGGACTAAACAGGAACAAGAGGGATCCACCGAAGAAGATCCTTCTTCTTCCCTTTTTTCGGAAGAAAAGGAGGATCCGGACAAAATCGACGAAACGGAAGAGATCCAAGTGAATGGAAAGGAAAAAACAAAGGATGAATTCCATTTTCACTTTAAAGAGACATGCTATAAAAATAGACCACTTTATGAAACTTTTTATCTGGATGGGAATCAAGAAAATTCGAAGTTAGAAATATTGATAGATAAAAAAAAGAAAGATCTTTTCTGGTTTGAAAAACCTCTTGTAACTATTCTTTTTGACTCTAAACGTTGGAATCGTCCATTTCGATATATAAAAAATGATCAGTTTGAGAATGCTGTAAGAAAAGAAATGTCACAATATTTTTTTTATACATGTCGGAGTGATGGAAAAGAAAGAATATCTTTTACGTATCCACCCAGTTTGTCAACTTTTTTGGAAATGATACAAAGAAAGATATCTCCGTTTACAACAGAAAAACTCTCCTCTGATGAATTGTATAATCGTTGGAATTATAAGAATGAACAAAAAAAGAAAACCCTAAATAATGAATTTATAAACAGAGTCCAGGCTCTAGATAAGGGATATCTTACTCTGAATACACTCGAAAAAAGGGCTGGACTATGTAATGATAAAACTAAAAAAGAGTACTTACCTAAAATTTATGATCCTTTTTTGAGTGGGTCCTGCCGCGGGAAAATCCAATTTTTTTCACCCTCATTCCTAAATATCCTAAATAAAACTTCCATAAAAAATTCCATAGAGATGCTTTGGATAAATAAAATTCATCTTATTCTTCTTATTACTAATTATCAAGAATTTGAATCAAAAACAGATATAGCGAATCCAAAAAATAAAACAATTAGAAAAAATTCTACTGGAATAAAAGAAATAAGTAAACAAGTTCCTCGATGGTCATACAAATTAATTAACGATTTGGAACAACAAGAAAAAAACATACCGCAAGATTCTCAAATGCGTTCACGAAAAACCAAACGATTAGAGATTTTTAATAATAAAAATAAAAAAAAAATATATAAATATATGGATACTTATAATAATACCAAAGATACAAAGAATTCTGATAAAATAAAAATAGAAGATAGAATAAAAATAGAAAAAGCGTCTTTGCTATATTATTCACACCAACCGGACTTTCGCCGATGCATAATAAAAGGATCTGTGCGAGCACAAAGACGCAAAATAACTATTTTTGAACTGTTTCGAGCAAATGTGCATTCTCCCCTTTTTTTGGACAGAATAAACAAATATCTTTTTTTTTTTTTTGATATTGTCGAACTGATAAAAAAAATGTTAAAAACAATGTTTATAAATTGGATGTATAAAAACGCAGAATTCATACTTTCGAATATAAAGAAAAAAACAAAAGAAAGTAAGAAAAAAAAAGAGGACAAAAGAGAAGACAACAAAATAGAAGAAGAAGAAGAAGACAACAAAATAGAAGAAGAAGAAGAAGACAACAAAATAGAAGACAACAAAATAGAAGACAACAAAATAGAAGAACCCACAACAGAGACAATCGATCTGGTAGAAATAGCCGAAGCCTGGGATGATATTCTTTTTGCTCAAATAACAAGAGGTTTTATTTTAGTAACCCAATCCATTTTTAGAAAATATATTCTATTACCTTCATTAATAATAATTAAAAATATCATTCGTATACTTTTTTTAAAAACTCCTGAATGGTCCGAGGATTTCAAAGATTGGAGTAGAGAAATACATGTTAAATGCACCTATAATGGAGTTCAATTATCAGAAAAAGAATTTCCGAAAAACTGGTTAATAGAGGGGATTCAAATAAAGATCCTATTTCCTTTTCGTTTAAAACCTTCGCACAAATCTAAGTTAAAATTCTCTCATAAAGATCAAACGAAAATGAAAAAAAAAGCACAAAAAAATGATTATTTTTTTTTAACAGTTTGGGGAACGGAAGCTGACCTGCCTTTTTCGGCGAGTAGAAAAGAACTTTCACTTTTTAAACCCATCTTTAAAAAACTCAAAAAAAAATTTAGAAAAATGCAAAAAAATGGTTTTCGAGTTATAACAATTTTAGAAGAAAGAAAAAAAATTTTTCTAAATTTCTCAAAAGAAAAAAAAAACTGGATCATACAAAACATTTTTTTTCGAAAAGAAATAATAAACAACCTTTCAAAATCAAAAAGAAATCCAATTCTATTATCTGGATTTAAAGAAGTATATGAATTGAATGAACCTAAAAAAGAAAAAGATTCGATAATCAATAACAATAATGGGACGATTCAAAAATTATCCACCCCAATTCGATCTATGGCTTGGACAAATTATTCACTGACAGAAAAAAAAATGAAAGATCTTTCTGCTAGAAGAAAGATAATCATAAATCAAATAGAAAAAATTAAAAAAGAAAAGGAAAAAAAAATTAGAACCTCGGAAATAAATATAAATATTAGTCCTAACAAAATAAGTTATAATGCTAAAAAATTAAAATCATCAAAAAAGATTTCGCAGATAGTAAAAAAAAGAAATGCTCGATTGGCGCATAAATTCCATTTTTTTATAAAAATTTTGATTGAAAGGATATACATAGATATCTTTTTAGGTATCATTAATATTCCTTTAGGTATCATTAATATTCCAAGGATCAATGCACAACTTTTTCTTGAATCAACAAAAAAAATTATTACTAAATACATTTACAATAATGAAGAAAATCACAAAAAAATTGATAAAACAAATCAAACTACAATTCACTTTATTTCGATTATAAAAAAGTCATGTAATAGTAATGTTGTTGTTATTAATAACAACAACAATTCACAGATTTTTTGTGACATATCCTCCTTGTCACAAGCTTATGTATTTTACAAATTATCACAAATCAAAATTATTAACTTATATAAGTTAAGATCTATCTTTGAATATCATAGCCTTTTTCTGAAGAACGAAATAAAGGATTTTTTTATAGCCCAAGGGCTATTTAATTCCGAATTAAAAGATAAAAATTTTCGAAATTCTGTAATGAATCAATGGAAAAATTGGTTAAGGAGTCATTATCAATATAAATACCATTTATCTCAGATTAGATGGTCTAGATTAACACCACAAAAATGGCGAAATAGAATCAATCAACACCATATGCATATGGTTCAAAATAAAAAATTAAACAAATGGAATTTATATGAAAATGAAAAAGACAGATTAATTCATTACAAAAAAAAAAATGATTTTGAGACAGATTTATTACCGAATCAAAAAGATAATTTTAAAAAACACTATAGATATAATCTTTTAGCATATAAATCTATTAATTATGAAAATAAGAAGGACTTAAATTATCTAGCGGAAGATGATATTATCGATATGGAAAAAAGCGCGGATAGAAAATATTTTGATTGGAGCATTCTCAGTTTTTGTCTTAGAAAGAAGGTTGATATTGAGTCCTGGATCGATACCGGAAGAAAAAATAAAAAAAATACTAAGACTAGGACTAATAAGTATAAAATAATTGATAAAATTGATAAGAAAAAAAAGATTTTTCTTACAATTCACCAAGATCAAGAAGTCAATTCATCCAATCAAAAAAAAAAACCTTTTGACTGGATGGGAATGAATGAAGAAATAAAAAATCGTCTTATATCCGATTTTGAAGTTTGGTTCTTTCAAAAATTTTTGATACTTTACAACACATACAAGATAAAACCATGGGCAATACCCATCAAATTTCTTCTTTTCAATTTTCATTTCTATGAAAATGTTAGTAAAAATAAGAAAATTAACGGGAAAAAAAATAAAAATAGCGATCTTTTTATATCTATATCATCGAATGAAAAAAAAATTATTGAATTAGAGCACGAAGAAAAAGAATACGAAGACCCAGGGGGGGGCTTTGGGTCAGTTTTCCAAAATCAAGAAAAAGATATTGAAGAAGATTATATAGGATTAGATATGAAAAAACATAGAAATGAAAAGCAAAACAAAAGTCATATGGAAGTAGAACTTGATTTCTTCCTAAAACGGTATTTATGTTTTCAATTAGAATGGAATAGTTCTTTAAATAACCAACTAATCGAGAATATCAAATTCTATGGTTTCCTGCTTAAAGTGACAAATCCACGAAAAATTATTATATCTTCTATTCAAAGGCAAGAAATAAATTTGAATATTATGATGGTTCGGAACAGTTTTACTCGTACAGAATTAGTGAAAAGGGGAATATTGATTATCGAACCTGTTCGTCTGTCAGTAAAAAATGATGGACAATTTATTTTGTATCAAATGATAGGTATCTTATTAGTTCATAAAAACAAACAACAAATTAATAAAAAATACAGATATTATGTTGATAAAAAGAATTTTACCGAATTTATTGAAAGACATCAAAGTATAATTGGAAATAGAAAGAAAAATGATTATGATTTACTTGTTCCCGAAAATATTTTATCCCCTAAACGTCGGAGAGAATTAAGAATTCTTTTCAATTTAAAAAATAAAAATGATATTCATATAAATACAGAAATTTTCAACGGCAATAACATAAAAAATTGTAGTCCCATTTTAGGTAAAAGCAAACATTTTGATAGAGATAAAAAGAAACTAATTAAATTACAATTTTTTCTTTGGCCAAATTTTCGATTAGAAGATTTAGCTTGTATGAATCGTTATTGGTTCGATACTAATAATGCCAGTCGGTTCAGTATGGTAAGAATATATATATATCCGCGGTTGAAATTTTGA

>lcl|NC_010433.1_cds_YP_001718437.1_22 [gene=psaA] [locus_tag=MaesCp021] [db_xref=GeneID:5999964] [protein=photosystem I P700 chlorophyll a apoprotein A1] [protein_id=YP_001718437.1] [location=complement(42302..44554)] [gbkey=CDS]
ATGATTATTCGTTCGCCGGAACCAGAAGTCAAAATTTTGGTAGATAGGGATCCCATCAAAACTTCTTTCGAGGAATGGGCCAGACCCGGTCATTTCTCAAGAACAATAGCTAAAGGACCTGATACTACTACTTGGATCTGGAACCTACATGCTGATGCTCACGATTTCGATAGCCATACCAGTGATTTGGAGGAGATTTCTCGGAAAGTATTTAGTGCTCATTTCGGCCAACTCTCCATCATCTTTCTTTGGCTGAGTGGCATGTATTTCCACGGTGCTCGTTTTTCCAACTATGAAGCATGGCTAAGCGATCCTACTCACATTGGACCTAGCGCCCAAGTGGTTTGGCCAATAGTGGGTCAAGAAATATTGAATGGCGATGTGGGCGGGGGTTTCCGAGGAATACAAATAACCTCCGGTTTTTTTCAGATTTGGAGAGCATCTGGAATAACTAGTGAATTACAACTGTATTGTACCGCAATTGGTGCATTGGTCTTTGCAGCCTTAATGCTTTTTGCTGGTTGGTTCCATTATCACAAAGCTGCTCCAAAATTGGCTTGGTTCCAAGATGTAGAATCTATGTTGAATCACCATTTAGCGGGGCTACTAGGACTTGGGTCTCTTTCTTGGGCGGGACATCAAGTACATGTATCTTTACCAATTAACCAATTTCTAAACGCTGGAGTGGATCCTAAAGAAATCCCACTTCCTCATGAATTTATCTTGAATCGGGATCTTTTGGCTCAACTTTATCCCAGTTTTGCTGAGGGAGCAACCCCATTTTTCACCTTGAATTGGTCAAAATATTCGGACTTTCTTACTTTTCGTGGAGGATTAGATCCAGTGACTGGGGGTCTATGGCTGACCGATACTGCACACCATCATTTAGCTATTGCAATTCTTTTCCTGATAGCGGGTCACATGTATAGGACTAACTGGGGCATTGGTCATGGTATAAAAGATATTTTAGAGGCTCATAAAGGTCCATTTACAGGTCAGGGTCATAAAGGCCTATATGAGATCCTAACAACATCATGGCATGCTCAATTATCTCTTAACCTAGCTATGTTAGGTTCTTTAACCATTGTTGTAGCTCACCATATGTATTCCATGCCCCCTTATCCATATCTAGCTACTGATTATGGTACACAACTGTCATTGTTCACACATCACATGTGGATTGGTGGATTTCTCATAGTTGGTGCTGCTGCGCATGCAGCCATTTTTATGGTAAGAGACTATGATCCAACTACTCGATACAACGATCTATTAGATCGTGTCCTTAGGCATCGCGATGCAATCATATCACATCTCAACTGGGTATGTATATTTTTAGGCTTTCACAGTTTTGGTTTATATATTCATAATGATACCATGAGCGCTTTAGGGCGCCCTCAAGATATGTTTTCAGATACTGCTATACAATTACAACCTGTCTTTGCTCAATGGATACAAAACACCCATGCTTTAGCACCTGGTGCAACGGCTCCTGGTGCAACAGCAAGCACCAGTTTAACTTGGGGGGGTGGTGATTTAGTGGCAGTCGGCGGCAAGGTTGCTTTGTTACCGATTCCATTAGGAACCGCGGATTTTTTGGTACATCACATTCATGCATTTACGATTCATGTGACGGTATTGATACTTCTGAAAGGAGTTCTATTTGCCCGCAGTTCTCGTTTGATACCGGATAAAGCAAATCTTGGTTTTCGTTTTCCTTGTGATGGACCTGGAAGAGGGGGAACATGTCAAGTATCCGCTTGGGATCATGTCTTCTTAGGACTATTTTGGATGTACAATTCCATTTCGGTAGTAATATTCCATTTCAGTTGGAAAATGCAGTCAGATGTTTGGGGTAGTATAAGTGATCAAGGGGTGGTAACTCATATCACGGGAGGAAACTTTGCACAGAGTTCCATTACTATTAATGGATGGCTCCGTGATTTCTTATGGGCACAGGCGTCCCAGGTAATTCAGTCTTATGGTTCTTCATTATCTGCATATGGCCTTTTTTTCCTAGGTGCTCATTTTGTATGGGCTTTTAGTTTAATGTTTCTATTCAGCGGTCGTGGTTATTGGCAAGAACTTATTGAATCAATCGTTTGGGCTCATAATAAATTAAAAGTTGCTCCTGCTACTCAGCCTAGAGCCTTGAGCATTATACAAGGACGTGCTGTAGGAGTAACCCATTACCTTCTGGGTGGAATTGCCACAACATGGGCGTTCTTCTTAGCAAGAATTATTGCAGTAGGATAA

>lcl|NC_010433.1_cds_YP_001718490.1_75 [gene=ndhI] [locus_tag=MaesCp076] [db_xref=GeneID:6000073] [protein=NADH dehydrogenase subunit I] [protein_id=YP_001718490.1] [location=complement(125518..126015)] [gbkey=CDS]
ATGTTTCCCATGGTAACTGGGTTCATGAATTATGGGCAACAAACCATACGAGCTGCAAGGTACATTGGTCAAGGTTTCATGATTACCTTATCTCATGCAAATCGTTTACCTGTAACTGTTCAATATCCTTATGAAAAATTAATCACATCGGAGCGTTTCCGCGGTCGAATTCATTTTGAATTTGATAAATGCATTGCTTGTGAAGTATGTGTTCGTGTATGTCCTATAGATCTACCTGTTGTTGATTGGAAATTGGAAACTGACATTCGAAAGAAACGGTTGCTTAATTACAGTATTGATTTCGGAATCTGTATATTTTGTGGCAACTGTGTTGAGTATTGTCCGACAAATTGTTTATCGATGACTGAAGAATATGAGCTTTCTACTTATGATCGTCACGAATTGAATTATAATCAAATTGCTTTAGGTCGTTTACCAATGTCAGTAGTTGACGATTATACAATTCGAACAATTTTGAATTCAACTCAAAAAAAATAG

>lcl|NC_010433.1_cds_YP_001718463.1_48 [gene=psbB] [locus_tag=MaesCp048] [db_xref=GeneID:5999952] [protein=photosystem II 47 kDa protein] [protein_id=YP_001718463.1] [location=77269..78795] [gbkey=CDS]
ATGGGTTTGCCTTGGTATCGTGTTCATACCGTCGTATTGAATGATCCCGGTCGTTTGCTGTCTGTCCATATAATGCATACAGCTTTGGTTGCTGGTTGGGCCGGTTCGATGGCTCTATATGAATTAGCAGTTTTTGATCCCTCTGACCCCGTTCTCGATCCAATGTGGAGACAGGGTATGTTCGTTATACCTTTTATGACTCGTTTAGGAATAACCAATTCATGGGGTGGTTGGAGTATCACAGGAGGAACTATAACGAATCCGGGTATTTGGAGTTATGAAGGCGTGGCTGGGGCGCATATTGTGTTTTCTGGCTTGTGCTTCTTGGCAGCTATTTGGCATTGGGTGTATTGGGATCTAGAAATATTTTGCGATGAACGTACAGGAAAACCTTCTTTGGATTTGCCCAAGATCTTTGGAATTCATTTATTTCTCTCCGGGGTGGCTTGCTTTGGGTTTGGCGCTTTTCATGTAACCGGATTGTATGGTCCTGGAATATGGGTGTCCGACCCTTATGGACTAACCGGAAAGGTACAACCTGTAAGTCCAGCATGGGGTGTGGAAGGTTTTGATCCTTTTGTTCCGGGAGGAATAGCTTCTCATCATATTGCAGCGGGGACATTGGGCATATTAGCGGGCCTATTCCATCTTAGTGTCCGCCCGCCCCAACGTTTATACAAAGGATTACGTATGGGAAATATTGAAACTGTCCTTTCCAGTAGTATCGCGGCTGTCTTTTTTGCAGCTTTTGTTGTTGCTGGAACTATGTGGTATGGTTCAGCAACTACCCCGATTGAATTATTTGGTCCCACTCGTTATCAATGGGATCAAGGATACTTCCAGCAAGAAATATATCGAAGAGTTAGTGCTGGGCTAGCCGAAAATCAAAGTTTATCCGAAGCTTGGTCTAAAATTCCCGAAAAATTAGCTTTTTATGATTACATCGGCAATAATCCGGCAAAAGGTGGGTTGTTCAGAGCAGGCTCAATGGACAACGGGGATGGAATAGCTGTTGGGTGGTTAGGACATCCTATCTTTAGAGATAAAGAAGGGCGTGAACTTTTTGTACGTCGTATGCCTACTTTTTTTGAAACATTTCCGGTTGTTTTGGTAGACGGAGATGGAATTGTTAGAGCCGATGTTCCTTTTCGAAGGGCGGAGTCGAAGTATAGTGTCGAACAAGTAGGTGTAACTGTTGAGTTCTATGGTGGCGAACTAAACGGAGTCAGTTATAGTGATCCTGTTACTGTGAAAAAATATGCTAGACGCGCTCAATTGGGTGAAATTTTTGAATTAGATCGTGCTACTTTGAAATCCGATGGTGTTTTTCGTAGCAGCCCAAGGGGTTGGTTTACTTTTGGACATGCTTCGTTCGCTCTGCTCTTTTTCTTCGGACACATTTGGCATGGTGCTCGAACTTTGTTCAGAGATGTTTTTGCTGGTATTGATCCAGATTTAGATGCTCAAGTGGAATTTGGAGCATTCCAAAAACTTGGAGATCCGACTACAAGAAGACAAGTAGTCTGA

>lcl|NC_010433.1_cds_YP_001718439.1_24 [gene=rps4] [locus_tag=MaesCp023] [db_xref=GeneID:6000010] [protein=ribosomal protein S4] [protein_id=YP_001718439.1] [location=complement(48002..48607)] [gbkey=CDS]
ATGTCACGTTACCGAGGGCCTCGTTTCAAAAAAATACGCCGTCTGGGGGCTTTGCCGGGACTAACTAGTAAAAGGCCTAGAGTCGGGAGCGATCTTAGAAATCAATCACGCTCCGGTAAAAAATCTCAATATCGTATTCGTTTAGAAGAAAAACAAAAATTGCGTTTTCATTATGGTGTTACAGAACGACAATTACTTAAATACGTTCGTATCGCCGCAAAAGCCAAAGGGTCAACAGGTCAGGTTTTACTACAATTACTTGAAATGCGCTTGGATAACATCCTTTTTCGATTGGGTATGGCGTCAACTATTCCTCGAGCCCGCCAATTAGTTAATCATAGACATATTTTAGTTAATGGTCGTATAGTAGATATACCAAGTTATCGCTGCAAACCCCGAGATATTATTACAGCGAGGGATGAACAAAAATCTAGAGCTATGATTCAAAATGATCTTGATTCATCCCCCCAGGAGGAATTGCCAAAACATTTGACTCTTCACCCATTCCAATATAAAGGATTGGTCAATCAAATAATAGATAGTAAATGGGTTGGCTTGAAAATAAATGAATTGCTAGTGGTAGAATATTATTCTCGTCAGACTTAA

>lcl|NC_010433.1_cds_YP_001718473.1_58 [gene=rpl14] [locus_tag=MaesCp058] [db_xref=GeneID:5999954] [protein=ribosomal protein L14] [protein_id=YP_001718473.1] [location=complement(85787..86155)] [gbkey=CDS]
ATGATCCAATCTCAGACCCATTTGAATGTAGCGGATAACAGCGGAGCTCGAGAATTGATGTGTATTCGAATCATAGGGACTAGTAATCGCCGATATGCTCATATCGGTGACGTTATTGTTGCTGTGATCAAGGAAGCAGCACCAAATTCACCTCTAGAAAGATCAGAAGTAATCAGAGCTGTAATTGTACGTACTTGTAAAGAACTCAAACGTGATAATGGTATGATAATACGATATGATGACAACGCTGCAGTTGTCATTGATCAAGAAGGAAATCCAAAGGGAACTCGAATTTTTGGTGCAATCGCCCGGGAATTGAGACAGTTAAATTTTACTAAAATAGTTTCATTAGCACCTGAAGTGTTATAA

>lcl|NC_010433.1_cds_YP_001718422.1_7 [gene=atpA] [locus_tag=MaesCp006] [db_xref=GeneID:6000076] [protein=ATP synthase CF1 alpha subunit] [protein_id=YP_001718422.1] [location=complement(11244..12767)] [gbkey=CDS]
ATGGTAACCATTCGAGCCGACGAGATTAGTAATATTATCCGCGAACGTATTGAGCAATATAATAGGGAAGTAAAGATTGTAAATACTGGTACCGTACTTCAAGTAGGCGACGGCATTGCTCGTATTTATGGTCTTGATGAAGTAATGGCAGGCGAATTAGTAGAATTTGAAGAGGGTACAATAGGCATTGCTCTGAATTTGGAATCAAATAATGTCGGTGTTGTATTAATGGGTGACGGTTTAATGATACAAGAGGGAAGCTCCGTAAAAGCAACAGGAAGAATTGCTCAGATACCTGTGAGTGAGGCTTATTTGGGTCGTGTTATAAATGCCCTAGCTAAACCTATTGACGGTCGAGGTGAAATTTCAGCTTCTGAATCTCGGTTAATTGAATCTCCTGCTCCAGGTATTATTTCGAGACGTTCTGTATATGAGCCTCTTCAAACAGGACTTATTGCTATTGATTCGATGATCCCTATAGGACGTGGTCAACGAGAATTAATTATTGGGGACAGACAGACTGGTAAAACAGCAGTAGCCACAGATACAATTCTCAATCAACAAGGACAAAATGTAATATGTGTTTATGTAGCTATTGGGCAAAAAGCGTCTTCTGTGGCTCAAGTAGTGACTACTTTACAGGAAAGAGGGGCAATGGAGTACACTATTGTGGTAGCCGAAACGGCGGATTCTCCGGCTACATTACAATACCTCGCCCCTTATACGGGAGCGGCTCTGGCTGAATATTTTATGTACCGTGAACGACACACTTTAATCATTTATGATGATCTCTCCAAACAAGCGCAGGCTTATCGCCAAATGTCTCTTCTATTACGAAGACCACCTGGTCGTGAAGCTTATCCAGGAGATGTCTTTTATTTGCATTCACGCCTTTTGGAAAGAGCCGCTAAATTAAGTTCTCGTTTAGGTGAAGGAAGTATGACTGCTTTACCAATAGTTGAGACCCAATCAGGAGACGTTTCGGCTTATATTCCTACTAATGTAATTTCCATTACAGATGGACAAATATTCTTATCCGCCGATCTATTCAATGCTGGAATCAGGCCTGCTATTAATGTGGGTATTTCTGTTTCCAGAGTAGGATCCGCAGCTCAAATAAAAGCTATGAAACAAGTAGCTGGTAAGTTAAAATTGGAATTGGCGCAATTCGTAGAATTAGAAGCCTTTGCGCAATTCGCTTCTGATCTCGATAAAGCTACTCAGAATCAATTGGCAAGAGGTCAACGACTACGCGAGTTGCTCAAACAATCCCAATCCGCCCCTCTCACGGTGGAGGAACAGATAATGACTATTTATACCGGAACGAATGGTTATCTTGATTCATTAGAAATTGGACAAGTAAGGAAATTTCTCGTTGAGTTACGTACCTACTTAAAAACGAATAAACCTCAGTTCCAAGAAATCATATCTTCTACCAAAACATTCACCGAAGAAGCAGAAACCCTTTTGAAAGAAGCTATTCAGGAACAGAAGGAACGTTTTGTAATTCAGGAACAAGTATAA

>lcl|NC_010433.1_cds_YP_001718449.1_34 [gene=cemA] [locus_tag=MaesCp033] [db_xref=GeneID:6000061] [protein=envelope membrane protein] [protein_id=YP_001718449.1] [location=64854..65549] [gbkey=CDS]
ATGAAAAAATGGAAAAAAAAAACATTTATTCCCCTTCTATATCTTACATCTATAGTTTTTTTGCCCTGGTGGGTCTCTTTTTTATTTAATAAAAGTTTGGAATCTTGGATTATTAATTGTTGTAATACTAGTAAATCCGAAACTTTTTTAAATGATATCCAAGAAAAAAGTATTCTAGAAAAATTCATGGAATTAGAAGACCTCGTTCGCTTAAACGAAATAATAAAGGAATACCCGGAAACACATCTACAAAAGTTTCGTATCGGAATCCACAAAGAAACGATCCAATTGATCAAGATGCACAATGAGGATCGTATCCATACGATTTTGCACTTCTCGACAAATATAATCTGTTTCATTATTCTAAGTGGTTATTCTATTCTAAGTAATGAAGAACTTATTATTCTTAATTCTTGGGTTCAAGAATTCCTATATAACTTAAGCGACACAATAAAAGCTTTTTCCATTCTTTTATTAACCGATTTATGTATAGGATTCCATTCACCTCACGGTTGGGAACTAATGATCGGCTCTGTCTACAAAGATTTTGGATTTGCCCATAACGATCAAATTATATCTGGCCTTGTTTCTACTTTTCCAGTCATTCTCGATACCATTTTTAAATATTGGATTTTCCGTTATTTAAATCGCGTATCTCCGTCACTTGTAGTGATTTATCATTCAATGAATGACTGA

>lcl|NC_010433.1_cds_YP_001718486.1_71 [gene=ndhD] [locus_tag=MaesCp072] [db_xref=GeneID:6000015] [protein=NADH dehydrogenase subunit 4] [protein_id=YP_001718486.1] [location=complement(121844..123346)] [gbkey=CDS]
ATGAATTCTTTTCCTTGGTTAACAATATTTGTAGTTTTACCGATATCCGGGGGTTCCTTAATTTTCCTTTTCCCTCATAGAGGAAATAAAGTAATAAAATGGTATACTATATTTATATGCATCTTTGAGCTACTTTTAATGACTTATGCGTTCTCTTATTATTTCCAATTGGACGATCCATTAATCCAATTAACAGAAGATTATAAATGGATCCAATTTTTTGATTTTTACTGGAGATTGGGAATCGATGGATTTTCTTTAGGACCTATTTTACTGACAGGATTTATCACCACTTTAGCTACTTTAGCGGCTCGGCCAATTACTCGGGATTCCCGATTATTTCATTTTCTGATGTTAGCAATGTATAGTGGTCAAATAGGATTATTTTCTTCTCAAGATCTTTTACTTTTTTTTATCATGTGGGAGTTAGAATTAATTCCCGTTTATCTACTTCTATCCATGTGGGGGGGAAAGAAACGTCTGTATTCAGCTACAAAGTTTATTTTGTATACTGCGGGAGGTTCCGTTTTTTTATTAATGGGAGCTTTGGGTATCGCTTTATATGGTTCTAATGAACCAAGATTCCATTTTGAAACATCAGCTAATCAATCATATCCTGTGGCGCTAGAAATATTTTTCTATATTGGATTTCTTATTGCTTTTGCTGTCAAATCACCGATTATACCCTTACATACATGGTTACCAGACACCCACGGGGAAGCACATTACAGTACTTGTATGCTTCTAGCCGGAATCTTATTAAAAATGGGGGCGTACGGATTGGTTCGAATCAATATGGAATTATTACCTCACGCTCATTCTATTTTTTCTCCCTGGTTGATAATAGTAGGCGTAATGCAAATAATCTATGCAGCTTCAACATCTCCTGGTCAACGAAATTTAAAAAAAAGAATAGCCTATTCTTCTGTATCTCATATGGGTTTCATAATTATAGGAATTTGCTCTATAAGTGATATGGGACTCAATGGAGCCATTTTACAAATAATATCACATGGATTTATTGGTGCCGCACTTTTTTTCTTGGCAGGAACGGGTTATGATAGAATACGTCGTGTTTATCTTGATGAAATGGGCGGAATGGCTACCTCAATGCCAAAAATATTCACGACATTCAGTATCTTATCACTAGCTTCCCTTGCATTACCAGGCATGAGCGGTTTTTTTGCGGAATTGATAGTATTTTTTGGAATAATTACCGGCCAAAAATATCTTTTAATGTCAAAAATATTAATTACTTTTGTAATGGCAGTTGGAATGATATTAACTCCTATTTATTTATTATCTATGTTACGCCAGATGTTTTATGGATACAAGCTGTTTAATGCCCCAAACTCTTATTTTTTTGATTCTGGACCGCGGGAGCTATTTGTTTCGATCTCTATCCTTCTGCCTGTAATAGGGATTGGTTTTTATCCGGATTTCGTTTTCTCATTATCAGTTGACCGGGTTGAAGCTATTCTATCTAATTATTTTTATAGATAG

>lcl|NC_010433.1_cds_YP_001718460.1_45 [gene=rpl20] [locus_tag=MaesCp044] [db_xref=GeneID:6000024] [protein=ribosomal protein L20] [protein_id=YP_001718460.1] [location=complement(73205..73558)] [gbkey=CDS]
ATGACCAGAATTAGACGAGGATATATAGCTCGAAGGCGTAGAACAAAAATTCGTCTATTCGCATCAAGCTTTCGCGGGGCCCATTCAAGACTTACTCGAACTATTATTCAACAAAAAATAAGAGCTTTGGTTTCGGCCCATCGGGATAGAGATAGGCAAAAAAGAAATTTTCGTCGTTTGTGGGTTACTCGGATAAATGCAGTAATTCGCGAGAGCACGGTATCCTATAGTTATAGTAGATTAATAAACAATCTGTACAAGAGACAGTTACTTCTTAATCGTAAAATACTTGCACAAATAGCTATATTAAATAGGAATTGTCTTTATATGATTTCCAATGACATTCTAAAATAA

>lcl|NC_010433.1_cds_YP_001718417.1_2 [gene=psbA] [locus_tag=MaesCp001] [db_xref=GeneID:5999949] [protein=photosystem II protein D1] [protein_id=YP_001718417.1] [location=complement(462..1523)] [gbkey=CDS]
ATGACTGCAATTTTAGAGAGACGCGAAAGCGAAAGCCTATGGGGTCGTTTCTGTAACTGGATAACCAGCACTGAAAACCGTCTTTACATTGGATGGTTTGGTGTTTTGATGATCCCTACTTTATTGACCGCAACTTCTGTATTTATTATCGCTTTCATTGCTGCCCCTCCGGTAGATATTGATGGTATTCGTGAACCTGTTTCTGGATCTCTACTTTATGGAAACAATATTATTTCTGGTGCCATTATTCCTACTTCTGCGGCTATAGGTTTGCATTTTTACCCAATATGGGAAGCGGCATCTGTTGATGAATGGTTATACAATGGCGGTCCTTATGAGCTAATTGTTCTACACTTCTTACTTGGTGTAGCTTGTTACATGGGTCGTGAGTGGGAACTTAGTTTCCGTCTGGGTATGCGCCCTTGGATTGCTGTTGCATATTCAGCTCCTGTTGCAGCTGCTACTGCTGTTTTCTTGATCTATCCAATTGGTCAGGGAAGCTTTTCTGATGGTATGCCTCTAGGAATCTCTGGTACTTTCAACTTTATGATTGTATTCCAGGCTGAGCACAACATCCTTATGCACCCATTTCACATGTTAGGCGTAGCTGGTGTATTCGGCGGCTCCCTATTCAGTGCTATGCATGGTTCCTTGGTAACCTCTAGTTTGATCAGGGAAACCACAGAAAATGAATCTGCTAATGAAGGTTACAGATTCGGTCAAGAGGAAGAAACTTATAATATCGTAGCTGCTCATGGTTATTTTGGCCGATTGATCTTCCAATATGCTAGTTTCAACAACTCTCGTTCTTTACATTTCTTCCTAGCTGCTTGGCCTGTAGTAGGTATTTGGTTCACTGCTTTAGGTATTAGCACTATGGCTTTCAACCTAAATGGTTTCAATTTCAACCAATCTGTAGTTGATAGTCAAGGTCGTGTAATTAATACCTGGGCTGATATTATTAACCGTGCTAACCTTGGTATGGAAGTTATGCATGAACGTAATGCTCATAACTTCCCTCTAGACCTAGCTGCTGTCGAAGCTCCATCTACAAATGGATAA

>lcl|NC_010433.1_cds_YP_001718426.1_11 [gene=rps2] [locus_tag=MaesCp010] [db_xref=GeneID:6000041] [protein=ribosomal protein S2] [protein_id=YP_001718426.1] [location=complement(16437..17147)] [gbkey=CDS]
ATGATAAGAAGATATTGGAACATTAATTTTGAAGAGATGATGAAAGCAGGAGTTCATTTTGGTCATGGTACTAGAAAATGGAATCCGAGAATGGCACCTTATATCTCTGCAAAGCGTAAAGGTATTCATATTACAAATCTTACTAGAACTGCTCGTTTTTTATCAGAAGCTTGTGATTTAGTTTTTGATGCAGCAAGTAGGAGAAAGCAATTCTTAATTGTTGGTACCAAAAATAAAGCAGCGGATTCAGTAGCGCGGGCTGCAATAAGGGCTCGGTGTCATTATGTTAATAAAAAATGGCTCGGCGGTATTTTAACGAATTGGTCCACTACAGAAACTAGACTTCAAAAGTTCAGGGACTTGAGAATGGAACAAAAGGCAGGTAGACTCAACCGTCTTCCGAAAGGAGATGCGGCTCGATTGAAGAGACAGTTAGCTCACTTGCAAACATATCTGGGCGGGATTAAATATATGACGGGGTTACCCGATATTGTAATAATCGTTGATCAGCAAGAAGAATATACGGCTCTTCGGGAATGTATGACTTTGGGAATTCCAACAATTTGTTTAATTGATACAAACTGTGACCCGGATCTCGCAGATATTTCGATTCCAACGAATGATGACGCTATAGCTTCAATCCGATTAATTCTTAATAAATTAGTATTTGCAATTTGTGAGGGTCGTTCTAGCTATATACGAAATCCCTGA

>lcl|NC_010433.1_cds_YP_001718489.1_74 [gene=ndhG] [locus_tag=MaesCp075] [db_xref=GeneID:6000007] [protein=NADH dehydrogenase subunit 6] [protein_id=YP_001718489.1] [location=complement(124548..125078)] [gbkey=CDS]
ATGGATTTGCCTGGACTAATTCATGATTTTCTTTTAGTCTTTCTGGGGTTAGGTCTTATATTAGGAGGTCTAGGAGTAGTATTACTTACCAACCCAATTTATTCTGCCTTTTCGTTGGGATTGGTTCTTGTTTGTATATCTTTATTCTATATTTTATCAAACTCTCATTTTGTAGCTGCCGCACAGCTCCTTATTTATGTGGGAGCTATAAATGTTTTAATTATATTTGCCGTGATGTTCATGAATGGTTCAGAATATTACAAAGATTTTAATCTTTGGACTGTTGGAAGCGGGGTTACTTCCTTAGTTTGTACAAGTATTTTTGTTTCACTAATTACTATTATTCCAGATACGTCATGGTACGGAATTATTTGGACTACAAAAACAAATCAGATTATAGAACAAGATTTGATAAGTAATGGCCAACAAATTGGAATTCATTTATCAACAGATTTTTTTCTTCCATTTGAATTCATTTCAATAATTCTTTTAGTTGCTTTGATAGGTGCGATTGCTGTGGCTCGTCAGTAA

>lcl|NC_010433.1_cds_YP_001718483.1_68 [gene=ndhF] [locus_tag=MaesCp069] [db_xref=GeneID:6000055] [protein=NADH dehydrogenase subunit 5] [protein_id=YP_001718483.1] [location=complement(116204..118462)] [gbkey=CDS]
ATGGAACATATATATCAATATTCATGGATCATACCTTTCGTTACATTGCCAGTACCTATGTTAATCGGAGCGGGACTCCTGCTTTTTCCGGCAGCAACAAAAAAACTGCGGCGTATGTGGGCTTTTCCAAGCGTTTTCTTGTTAAGTATAGTCATGATTTTTTCAATCGATTTGTCTATTCAGCAAATAAATAGTAGTTTTATTTATCAATATATATGGTCGTGGACTATCAATAATGATTTTTCTTTAGAGTTCGGACACTTGATTGACCCACTTACTTCTATTTTGTCAGTATTAATTACTACAGTTGGCATTTTGGTTCTTTTTTATAGTGACAATTATATGTCTCATGATCAAGGCTATTTGAGATTTTTTGCTTATATGAGTTTTTTCACTACTTCAATGTTGGGATTAGTTACTAGTTCTAATTTGATACAAATTTATATTTTTTGGGAATTGGTTGGAGTGTGTTCTTATCTATTAATAGGTTTTTGGTTCACACGACCTATTGCATCGAATGCTTGTCAAAAAGCGTTTGTAACTAATCGCGTAGGGGATTTTGGTTTATTATTAGGTATTTTAGGTCTTTATTGGATAACGGGCAGTTTCGAATTTCGGGATTTGTTCAAAATCTTCAATAACTTGATTTATAATAATCAGGTTAATTTTTTATTTGTTACTTTGTGTGCCGTTCTATTATTTTCTGGCGCAATTGCTAAATCGGCGCAATTTCCTCTTCATGTATGGTTACCGGATGCCATGGAAGGGCCTACTCCTATTTCGGCTCTGATACATGCTGCTACTATGGTAGCGGCGGGAATTTTTCTTGTAGCTCGCCTTTTTCCTCTTTTCGTAATTATACCTTTCATAATGAATCTAATAGCTTTGATAGGTATAATAACAGTATTTTTAGGAGCTACTTTAGCTCTTGCTCAAAAAGATATTAAGAGAAGTTTAGCCTATTCTACAATGTCTCAATTGGGTTATATGATGTTAGCTCTAGGTATGGGGTCTTATCGGGCCGCTTTATTTCATTTGATTACTCATGCCTATTCGAAAGCATTGTTGTTTTTAGGATCTGGATCCATTATTCATTCAATGGAAGCTATTCTTGGTTATTCTCCAGATAAGAGTCAAAATATGGTTCTTATGGGTGGTTTAACAAAACATATTCCAATTACCAAAACGGCTTTTTTATTAGGAACACTTTCTCTTTGTGGTATTCCCCCCTTCGCCTGTTTTTGGTCCAAAGATGAAATTCTTAATGATAGTTGGTTGTATTCACCTATTTTCGCAATAATAGCCTGTTTCACAGCGGGATTAACCGCATTTTATATGTTTCGGGTTTATTTACTTACTTTTGACGGACATTTTAATGCTCATTTTCAAAGTTACAGTGGTAAAAAAAACAGTTCATTTTATTCAATATCTTTATGGGGTAAAGAAGGATCAAAAATGCTTAACAAAAATTTGCGTTTATTAGCTTTATTAACAATGAATAATAAGGAAAGGGCTTCTTTTTTTTGGAAGAACACATATCAAATTGATGGTAATGTAAGAAATATGACGTGGCCTTTTATTACTATTCAAAATTTTAACACTAAAAGGATTTTTTCCTATCCCCATGAATCGGATAATACTATGTTATTTCCTATGCTTATCTTAGTACTATTTACTTTGTTTATTGGAGCCATAGGAATTCCTTTCAATCAATTCAATCAAGAAGGAATGCTGTTGGATATAGATATATTGTCAAAACTTTTAACTCCGTCTTTAAACCTTTTGCATCAAAATCCAGAAAATTCGGTGGATTGGTATGAATTTGTAACAAATGCAACTTTTTCAGCCAGTATAGCTTTTTTTGGAATATTTATAGCGTCCTTTTTATATAAGCCTGTTTATTCATCGTTACAAAATTTGAATTTCTTTAATTCGTTCGCTAAAAAAGGTCCTAAGAGAATTCTTTGGGACAAAATAATAAATGTGATATATAATTGGTCCTCTAATCGAGGTTATATAGATGCTTTTTATGCAATATCTTTTATTGGAGGTATAAGAAAATTGGCTGAATTAATTCATTTTTTTGATAAACAAATAATTGATGGAACCCCCAATGGGGTCGGTGTTACTAGTTTCTTTGTAGGAGAGGGTATAAAAAATGTAGGAAGTGGTCGCATCTCTTTTTATCTCTTATTTTATTTATTTTATGCGTTAATCTTCTTATTAATTTACTACTCTGTTTATAAATTCATTATTTAG

>lcl|NC_010433.1_cds_YP_001718498.1_83 [gene=rpl2] [locus_tag=MaesCp085] [db_xref=GeneID:6000012] [protein=ribosomal protein L2] [protein_id=YP_001718498.1] [location=join(159679..160077,160751..161185)] [gbkey=CDS]
ATGGCGATACATTTATACAAAACTTCTACCCCGAGCACACGCAATGGAGCCGTAGACAGTCAAGCGAAATCCAATACACGAAATACACGAAAGAATTTGATCTATGGACAGCATCGTTGTGGTAAAGGCCGTAATGCCAGAGGAATCATTACCGCAAGACATAGAGGGGGAGGTCATAAGCGTCTATACCGTAAAATCGATTTTCGACGGAATGAAAAAGACATATATGGTAGAATCGTAACCATAGAATACGACCCTAATCGAAATGCATACATTTGTCTCATACACTATGGGGATGGTGAGAAGAGATATATTTTACATCCCAGAGGGGCTATAATTGGAGATACCATTATTTCTGGTACAGAAGTTCCTATAAAAATGGGAAATGCCCTACCTTTGACCGATATGCCCTTAGGCACGGCCATACATAACATAGAAATCACACTTGGAAAGGGTGGACAATTAGCTAGAGCTGCAGGTGCTGTAGCGAAACTGATTGCAAAAGAGGGGAAATCAGCCACATTAAAATTACCTTCTGGGGAGGTTCGTTTAATATCCAAAAACTGCTCAGCAACAGTCGGACAAGTAGGGAATACTGGGGTGAACCAGAAAAGTTTGGGTAGAGCCGGATCTAAATGTTGGCTAGGTAAGCGTCCTGTAGTAAGAGGAGTAGTTATGAACCCTGTAGACCACCCCCATGGGGGTGGTGAAGGGAGGGCCCCAATTGGTAGAAAAAAACCCGCAACCCCTTGGGGTTATCCTGCACTTGGAAGAAGAAGTAGAAAAAGGAATAAATATAGTGATAATTTGATTCTTCGTCGCCGTAGTAAATAG

>lcl|NC_010433.1_cds_YP_001718488.1_73 [gene=ndhE] [locus_tag=MaesCp074] [db_xref=GeneID:6000075] [protein=NADH dehydrogenase subunit 4L] [protein_id=YP_001718488.1] [location=complement(123984..124289)] [gbkey=CDS]
ATGATGCTCGAACATGTACTTGTTTTGAGTGCCTATTTATTTTCTATTGGTATCTATGGATTGATCACGAGTCGAAATATGGTTAGAGCCCTTATGTGCCTTGAACTTATACTGAATGCAGTTAATCTAAATTTCGTAACATTTTCTGATTTTTTTGATAGTCGCCAATTAAAAGGAAATATTTTTTCAATTTTTGTTATAGCTATCGCAGCCGCTGAAGCAGCTATTGGACCAGCTATTGTTTCGGCAATTTATCGTAATAGAAAATCAATCCATATCAATCAATCGAATTTGTTGAATAAGTAG

>lcl|NC_010433.1_cds_YP_001718467.1_52 [gene=petB] [locus_tag=MaesCp052] [db_xref=GeneID:5999969] [protein=cytochrome b6] [protein_id=YP_001718467.1] [location=join(79716..79721,80491..81132)] [gbkey=CDS]
ATGAGTAAAGTCTATGATTGGTTCGAAGAACGTCTCGAGATTCAGGCAATTGCAGATGATATAACTAGTAAATATGTTCCTCCCCATGTCAACATATTTTATTGTTTAGGGGGAATTACGCTTACTTGTTTTTTAGTACAAGTAGCTACGGGGTTTGCTATGACTTTTTACTACCGTCCGACGGTTACTGAGGCTTTTGCTTCTGTTCAATACATAATGACTGAAGCTAACTTTGGTTGGTTAATCCGCTCAGTTCATCGATGGTCGGCAAGTATGATGGTTTTAATGATGATCCTGCACGTATTTCGTGTGTATCTCACTGGTGGCTTTAAAAAACCTCGTGAATTGACTTGGGTTACGGGCGTGGTTCTTGCTGTATTGACCGCATCTTTTGGTGTAACTGGTTATTCCTTACCTTGGGACCAAATTGGTTATTGGGCGGTAAAAATTGTAACAGGCGTGCCGGAAGCTATTCCTGTAATAGGATCACCTTTGGTAGAGTTATTACGCGGAAGTGCTAGTGTAGGACAATCCACTTTGACTCGTTTTTATAGTTTACACACTTTTGTATTACCTCTTCTTACTGCCGTATTTATGTTAATGCACTTTCCAATGATACGTAAGCAAGGTATTTCAGGCCCTTTATAG

>lcl|NC_010433.1_cds_YP_001718475.1_60 [gene=rps3] [locus_tag=MaesCp060] [db_xref=GeneID:5999953] [protein=ribosomal protein S3] [protein_id=YP_001718475.1] [location=complement(87947..88603)] [gbkey=CDS]
ATGGGACAAAAAATAAATCCACTTGGTTTCAGACTTGGTACAACCCAAAGTCATCATTCTCTTTGGTTTGCACAACCAAAAAATTACTCTGAGGGTCTACAAGAAGATCAAAAAATAAGAAACTGTATCAAGAATTATGTAAAAAAAAATGCGAAAATATCTTCTGGTATTGAAGGAATTGTACGTATAGAGATTCAAAAACGAATTGATGTGATTCAGGTCATAATATATATGGGATTCCCAAAATTATTAATAGAAAGTAGACCTAAACGAATCGAAGAATTACAGATGAATGTACAAAAAGAACTTAATTGTGTGAATCGAAAATTCAATATTGCTATTACAAGAATTCCAAACCCTTACGGGCACCCTAATATTCTTGGAGAATTTATAGCCGGACAATTAAAAAATAGAGTTTCATTTCGCAAAGCAATGAAAAAAGCTATTGAATTAACTGAACAGGCCAATACAAAAGGAATTCAAGTGCAAATTGCTGGGCGTCTTGACGGAAAAGAAATTGCACGCGTCGAATGGATTAGAGAAGGTAGAGTTCCTCTACAAACCATTGGAGCTAAAATTGATTATTGTTCGTATACAGTTAGAACTATTTATGGGGTATTAGGCATAAAAATTTGGACATTTCCAGACAAGAAATAA

>lcl|NC_010433.1_cds_YP_001718429.1_14 [gene=rpoB] [locus_tag=MaesCp013] [db_xref=GeneID:6000006] [protein=RNA polymerase beta subunit] [protein_id=YP_001718429.1] [location=complement(24616..27828)] [gbkey=CDS]
ATGCTCGGGGATGGAAATGAGGGAATGTCTACAATACCTGGATTTAATCAGATACAATTTGAAGGATTTTGTAGATTCATTGATCAGGGCTTAACAGAAGAACTTTATAAGTTTCCAAAAATTGAAGATACAGATCAAGAAATTGAATTTCAATTATTTGTGGAAACATATCAATTAGTAGAACCCTTGATAAAAGAAAAAGATGCTGTATATGAATCACTTACATATTCTTCTGAATTATATGTATCCGCAGGATTAATTTGGAAAACCAGTAGGGATATGCAAGAACAAACAATTTTTATTGGAAACATTCCTCTAATGAATTCCCTGGGAACTTTTATAATAAATGGAATATACAGAATTGTGATCAATCAAATATTGCAGAGTCCGGGTATCTATTACCGGTCAGAGTTGGAACATAATGGAATTTCGGTCTATACCGGCACCATAATATCAGATTGGGGGGGGAGAGTAGAATTAGAGATTGATAGAAAAGCAAGGATATGGGCTCGTGTGAGTAGGAAACAGAAAATATCTATTCTAGTTCTATCATCAGCTATGGGTTTGAATCTAAAAGAAATTTTAGAGAATGTGTGCTACCCTGAAATTTTCTTATCTTTCCTGAATGATAAGGAAAAAAAAAAAATTGGGTCAAAGGAAAATGCCATTTTGGAGTTTTATCAACAATTTACTTGTGTAGGCGGAGATCCAATATTTTCTGAATCCTTATGTAAGGAATTACAAAAGAAATTCTTTCAACAAAGATGTGAATTAGGAAAGATTGGTCGATTAAATATGAACCGGAAACTGAATCTTGATATACCCCATAACAATACATTTTTGTTACCACGAGATATATTGGCAGCTGCGGATCGTTTGATTGGAATGAAATTTGGAATGGGTACACTTGACGATATGAATCATTTAAAAAATAAACGTATTCGTTCTGTAGCGGATCTCTTACAAGATCAATTCGGATTGGCTCTGATTCGTTTAGAAAATGTAGTTCGAGGGACTATATGTGGAGCAATTAGGCATAAATTGATACCGACCCCTCAAAATTTGGTAACTTCAACTCCATTAACAACCACTTATGAATCTTTTTTCGGATTACACCCATTATCTCAAGTTTTGGATCGAACTAATCCATTGACACAAATAGTTCATGGGAGAAAATTGAGTTATTTGGGTCCTGGAGGATTAACAGGACGAACTGCTAGTTTTCGGATACGAGATATCCACCCTAGTCATTATGGGCGCATTTGCCCAATTGACACGTCTGAAGGAATCAATGTTGGACTTATTGGATCTTTAGCAATTCATGCCAAGATTGGTTATTGGGGGTCTTTAGAAAGCCCATTTTATGAAATCTCTGAGGGATCAAAAAAAGTACGGATGTTTTATTTATCGCCAAATAGAGAGGAATACTATATGGTAGCGGCAGGAAATTCTTTGGCGCTGAATCGAGGTGTTCAGGAAGAACAGGTTGCTCCGGCTCGATATCGTCAAGAATTCCTGACTATTGCATGGGAACGGGTGCATCTTCGAAGTATTTTTCCCTTCCAATATTTTTCTATTGGAGCTTCCCTCATTCCTTTTATCGAGCATAATGATGCGAATCGGGCTTTAATGAGTTCTAATATGCAACGTCAAGCAGTTCCACTTTCTCGGTCCGAAAAATGCATTGTTGGAACTGGATTGGAACGCCAAGTGGCTCTAGATTCAGGGGTTCCTGCTATAGCCGAACACGAGGGAAAGATAATTTATACTGATATTGACAAGATCATTTTATCAGGTAATGGGGATACTCTACGCATTCCATTAGTTAGGTATCAACGTTCCAACAAAAATACTTGTATGCATCAAAAAACCCAGCTTCGGCGGGGTAAATGCATTAAAAAGGGACAAGTTTTAGCGGATGGTGCCGCTACAGTTGGTGGCGAACTTGCCTTGGGCAAAAACGTATTAGTCGCTTATATGCCATGGGAAGGTTACAATTTTGAGGATGCGGTACTCATTAGCGAGCGTCTGGTATATGAAGATATTTATACTTCTTTTCACATACGGAAATATGAAATTCAGACTCATGTGACAAGCCAAGGACCTGAAAGGATCACTAACGAAATACCGCATCTAGAGGCCCATTTACTCCGAAATTTAGACAAAAACGGAATTGTGATGCTAGGATCTTGGGTAGAGACGGGCGATATTTTAGTAGGTAAATTAACGCCTCAAATGGCGAAAGAATCATCGTATGCTCCGGAAGATAGATTATTAAGAGCCGTACTTGGTATTCAGGTATCTACTTCAAAAGAAACTTGTCTAAAACTACCTATAGGTGGTAGGGGTCGAGTTATTGATGTGAGATGGATCCAGAAAAAGGGGGGTTCCTGTTATAATCCGGAAAGGATTTGTGTATATATTTTACAGAAACGTGAAATCAAAGTGGGTGATAAAGTAGCTGGAAGACATGGAAATAAAGGCATCATTTCCAAAATTTTGCCTAGACAAGATATGCCTTATTTGCAAGATGGAAGACCTGTTGATATGGTCTTCAACCCATTAGGAGTACCTTCACGAATGAATGTAGGACAGATATTTGAATGCTCACTCGGGTTAGCGGGAGGTCTGCTAGATAGACATTATCGAATAGCACCCTTTGATGAGAGATATGAACAAGAGGCTTCGAGAAAACTAGTGTTTTCTGAATTATATGAAGCCAGTAAGCAAACAGCAAATCCGTGGGTATTTGAACCCGAGTATCCGGGAAAAAGTAGAATATTTGATGGAAGAACGGGGGATCCTTTTGAACAGCCTGTTATAATAGGAAAGCCTTATATCTTGAAATTAATTCATCAAGTTGATGATAAAATACATGGACGTTCCAGTGGACATTATGCACTTGTTACACAACAACCCCTTAGAGGAAGGGCCAAGCAAGGGGGACAACGGGTCGGAGAAATGGAGGTTTGGGCTCTAGAGGGGTTTGGTGTTTCTCATATTTTACAAGAAATGCTGACTTATAAATCTGATCATATTAGAGCTCGCCAAGAAGTGCTTGGTACTACGATCATTGGAGGAACAATACCTAAACCTGAAGATGCTCCCGAATCTTTTCGATTGCTCGTTCGAGAACTACGATCTTTGGCTCTGGAACTGAATCATTTCCTTGTATCTGAGAAGAACTTCCAGATTACTAGGAAGGAAGCTTAA

>lcl|NC_010433.1_cds_YP_001718468.1_53 [gene=petD] [locus_tag=MaesCp053] [db_xref=GeneID:5999970] [protein=cytochrome b6/f complex subunit IV] [protein_id=YP_001718468.1] [location=join(81340..81347,82177..82672)] [gbkey=CDS]
ATGGGAGTAACAAAAAAACCTGACTTGAATGATCCTGTATTAAGAGCTAAATTGGCTAAGGGAATGGGTCATAATTATTACGGAGAACCTGCATGGCCCAATGATCTTTTATATATTTTTCCAGTAGTAATTCTAGGTACTATTGCATGTAATGTAGGATTAGCGGTTCTAGAACCATCAATGATTGGTGAACCTGCGGATCCATTTGCAACGCCTTTGGAAATATTGCCTGAATGGTATTTCTTTCCCGTATTTCAAATACTTCGTACAGTACCCAATAAGTTATTAGGTGTTCTTTTAATGGTTTCAGTACCTGCAGGATTATTAACAGTACCTTTTTTGGAGAATGTTAATAAATTCCAAAATCCATTTCGTCGTCCAGTTGCGACAACCGTCTTTTTGATTGGTACTGCGGTAGCCCTTTGGTTAGGTATTGGAGCAACATTACCTATTGATAAATCCCTAACTTTAGGTCTTTTTCAAATTGATTCAATTGTAAAATAA

>lcl|NC_010433.1_cds_YP_001718440.1_25 [gene=ndhJ] [locus_tag=MaesCp024] [db_xref=GeneID:5999997] [protein=NADH dehydrogenase subunit J] [protein_id=YP_001718440.1] [location=complement(51488..51964)] [gbkey=CDS]
ATGCAGGGTCGTTTGTCTGCTTGGCTAGTCAAACATGGGCTAGTTCATAGATTTTTGGGTTTTGATTACCAAGGAATAGAGACTTTACAAATAAAGCCCGAAGATTGGCATTCCATTGCTGTCATTTTATATGTATATGGTTACAATTATCTGCGTTCGCAATGTGCCTATGATGTAGCACCGGGCGGGCTGTTAGCTAGTGTATATCATCTTACGAGAATAGAGTATGGTATAGATCAACCAGAAGAAGTATGTATAAAAGTCTTTGCCCCAAGGAAAAATCCTAGAATTCCGTCTGTTTTCTGGGTTTGGAAAAGTGCGGATTTTCAAGAAAGGGAATCTTATGATATGCTGGGAATCTTTTATGATAATCATCCGCGTCTGAAACGTATCTTAATGCCGGAAAGTTGGATAGGGTGGCCCTTACGTAAGGATTATATTGCTCCCAATTTTTATGAAATACAAGATGCTCATTGA

>lcl|NC_010433.1_cds_YP_001718472.1_57 [gene=rps8] [locus_tag=MaesCp057] [db_xref=GeneID:5999992] [protein=ribosomal protein S8] [protein_id=YP_001718472.1] [location=complement(85138..85542)] [gbkey=CDS]
ATGGGTAGGGATCCTATTGCTGACATAATAACCTCTATAAGAAATGCTGACATAAATAGAAAAGGAACCGTTCGAATAGCATCTACTAACATCACTGAAAACATTATTAAAATACTTTTAAGAGAAGGTTTTATTGAAAATGTCAGGAAACATCAGGAGGGCAACAAAAAATTTTTGGTTTTAACCCTACGACATAGAAGGAAGAGGAAAGGACCCTATAGAACTAGTCTAAATTTAAAACGGATCAGCCGACCTGGTCTACGAATCTATTCTAACTATCAAAAAATTCCTAGAATTTTGGGCGGGATGGGCATTGTAATTCTTTCTACTTCTCGGGGTATAATGACAGACCGAGAAGCTCGACTCGAAAGAATAGGCGGAGAAATCTTGTGTTATATATGGTAA

>lcl|NC_010433.1_cds_YP_001718476.1_61 [gene=rpl22] [locus_tag=MaesCp061] [db_xref=GeneID:6000051] [protein=ribosomal protein L22] [protein_id=YP_001718476.1] [location=complement(88701..89102)] [gbkey=CDS]
ATGATAAAGATAAAAAAAAGAAAGAGAAACACATATGAAGTATATGCTTTAGGCCAACATATATGTATGTCCCCTCACAAAGCACGAAGAATAATTGATCAGATTCGTGGACGTTCTTACGAAGAAACACTTATGATACTCGAGCTCATGCCTTATCGAGCATGTTATCCCATTTTTAAATTGATTTATTCCGCAGCAGCAAATGCTAGTCACAATATGGGTTTCAACGAAGCCAATTTAATCATTAGTAAAGCCGAAGTCAACGAAGGCACTACTGTGAAAAAATTAAAACCTCAGGCTCGAGGACGGGGTTATCCGATAAAAAGATCAACTTGTCATATATCTATTGTATTAAAAAATATATCCTTATATGAAGAATATGACGAATATATATATATATGA

>lcl|NC_010433.1_cds_YP_001718445.1_30 [gene=rbcL] [locus_tag=MaesCp029] [db_xref=GeneID:5999955] [protein=ribulose-1,5-bisphosphate carboxylase/oxygenase large subunit] [protein_id=YP_001718445.1] [location=58063..59496] [gbkey=CDS]
ATGTCACCACAAACAGAGACTAAAGCAAGTGTTGGATTCAAGGCTGGTGTTAAAGATTATAAATTGACTTATTATACTCCTGACTATCAAACCAAAGATACTGATATCTTGGCAGCATTCCGAGTAACTCCTCAACCTGGAGTTCCGCCTGAGGAAGCAGGAGCTGCGGTAGCTGCTGAATCTTCTACTGGTACATGGACAACTGTGTGGACCGATGGACTTACCAGTCTTGATCGTTATAAAGGACGATGCTACGGCCTCGAGCCCGTTCCTGGAGAAGAAAATCAATATATTGCTTATGTAGCTTACCCATTAGACCTTTTTGAAGAAGGTTCTGTTACTAACATGTTTACTTCTATTGTGGGTAATGTATTTGGGTTCAAAGCCCTACGCGCCCTACGTCTGGAGGATTTGCGAGTCCCTCCTGCTTATTCTAAAACTTTCCAAGGGCCTCCTCATGGCATCCAAGTTGAGAGAGATAAATTGAACAAGTATGGTCGCCCCCTATTAGGTTGTACTATTAAACCAAAATTGGGGCTATCCGCTAAGAATTACGGTAGAGCAGTTTATGAATGTCTTCGCGGTGGACTTGATTTTACCAAAGACGATGAGAATGTGAACTCCCAACCATTTATGCGTTGGAGAGACCGTTTCTTATTTTGTGCCGAAGCAATTTATAAAGCACAGGCTGAAACAGGTGAAATCAAAGGGCATTATTTGAATGCTACTGCAGGTACATGCGAAGAAATGATCAAAAGGGCTGTATGTGCCAGAGAATTAGGAGTTCCTATCGTAATGCATGACTACTTAACAGGGGGATTCACTGCAAATACTAGCTTGGCTCACTATTGCCGAGATAATGGTTTACTTCTTCACATTCACCGCGCAATGCATGCAGTTATTGATAGACAGAAGAATCATGGTATGCATTTTCGTGTACTAGCTAAGGCTTTACGTCTGTCTGGTGGAGATCATATTCACGCTGGTACCGTAGTAGGTAAACTTGAAGGGGAAAGAGACATTACTTTGGGCTTTGTTGATTTACTGCGTGATGATTTTATTGAAAAAGATCGAAGCCGCGGTATTTATTTCACTCAAGATTGGGTCTCTCTACCAGGTGTTCTGCCTGTAGCTTCAGGGGGTATTCACGTTTGGCATATGCCTGCTCTGACCGAGATCTTTGGAGATGATTCCGTACTACAATTCGGTGGAGGAACTTTAGGGCACCCTTGGGGAAATGCACCCGGTGCCGTAGCTAATCGAGTAGCTCTAGAAGCATGTGTACAAGCTCGTAATGAGGGACGTGATCTTGCTCGTGAGGGTAATGATATTATCCGTGAGGCTAGCAAATGGAGTCCTGAACTAGCTGCTGCTTGTGAAGTATGGAAGGAAATTAAATTTGAATTCGCAGCAGTGGATACTTTGGATAAATAA

>lcl|NC_010433.1_cds_YP_001718446.1_31 [gene=accD] [locus_tag=MaesCp030] [db_xref=GeneID:6000068] [protein=acetyl-CoA carboxylase beta subunit] [protein_id=YP_001718446.1] [location=60304..62031] [gbkey=CDS]
ATGGAAAAACGGTGGTTCAATTCGATCTTATCCAATGTGGAATTAGGATACAGGTGTAGGCTAAGTAAATCAATGGATAGTTTCAGTTCTCTTGAAAATACCAGTATAAGCGAAGACCCAATTCTAAATGATACAGATAAAAACACCCATAGTTGGAGTAATAGTAATAGTAATAGTGACAGCTCTAGTTATAGTAATGTTGATCATTTAGTCGGCGTCAGGGACATTCAGAATTTCGGCGCCGATGAAACTTATTTAGTTAGGGATAGTAATAAGGACAGTTATTCCATATATTTTGATATTGAAAATAAAGTTTTTGAGATTGACAATGATCATTCTTTTCTGAGTGAACTAAAAAGTTCTTTTTATAGTTATTGGAATTCTAGTTATCTGAATAATGGGTCTAGGAGTGGCGACTCCCACTCTGATCATTATATGTATGATACTAAATATAGTTGGAATAATTACATCAATAGTTGCATTGACAGTTATCTTCGCTCTCAAATCTGTATTGATAGTTATATTTTAAGTGGTAGTAACAATTACAGTGAAAGTTACATTTATAGTTACTACATTTATAGTTACTTTTGTGGTGAAAGTGGAAATAGTAGTGAAAGCGAGAGTTCCAGTCTAAGAACTAGCACGAGTGGTAGCGATTTAATTATAAGAGAAAGTTCTAATGATGAAAGTTCTAATGATAATGATGAAAGTTATAATGATAATGATGAAAGTTATAATGATAATGATGAAAGTTATAATGATGATGATGAAAGTTATAATGATGAAAGTTCTAATGATAATGATGAAAGTTATAATGATAATGATGAAAGTTATAATGATGATGATGAAAGTTATAATGATGAAAGTTCTAATGATAATGATGAAAGTTCTAATGATCTCGATATAACTCAAAAATACAAGCATTTGTGGGTTCAATGCGAAAATTGTTATGGATTAAATTATAAGAAATTTTTTAAGTCAAGAATGAATATTTGTGAACAATGTGGATATCATTTGAAAATGAGTAGTTCAGATAGAATTGAACTTTTGATTGACCTAGACACTTGGGATCCTATGGATGAAGACATGGTATCTCTGGATCCCATTGAATTTCATTCAGAAGAGGAACCTTATAAAGATCGTATTGATTCTTATCAAAGAAAGACAGGATTAAGTGAGGCTGTTCAAACAGGCACAGGTCAACTAAACGGCATTCCCGTAGCAATTGGGGTTATGGATTTTCGGTTTATGGGGGGTAGTATGGGATCCGTAGTAGGTGAGAAAATCACTCGTTTGATCGAGTATGCTACCAATAAATTTTTACCTCTTATTTTAGTGTGTGCTTCCGGAGGAGCACGCATGCAAGAAGGAAGTTTGAGCTTGATGCAAATGGCTAAAATATCTTCTGCATTATATGATTATCAATCGAATAAAAAGTTATTTTATGTATCAATCCTTACATCTCCTACGACTGGTGGGGTGACAGCTAGTTTTGGTATGTTGGGGGATATCATTATTGCTGAACCTAATGCCTATATTGCGTTTGCAGGTAAAAGAGTAATTGAACAAACATTGAATAAGACAGTACCTGAAGGTTCGCAATCGGCCGAATTTTTATTCCATAAAGGCTTATTTGATCTAATCGTACCGCGTAATCTTTTAAAGGGCGTTCTGAATGAGTTACTTCAGTTCCACGATTTCTTTCCTTTGAATCATAAATCAAAGTAG

>lcl|NC_010433.1_cds_YP_001718438.1_23 [gene=ycf3] [locus_tag=MaesCp022] [db_xref=GeneID:5999976] [protein=photosystem I assembly protein Ycf3] [protein_id=YP_001718438.1] [location=complement(join(45304..45456,46120..46347,47072..47197))] [gbkey=CDS]
ATGCCTAGATCTCGGATAAATGGAAATTTTATTGATAAGACTTTTTCAGTTGTAGCCAATATCTTATTACGAATAATTCCGACAACTTCGGGAGAAAAAGAGGCATTTACTTATTACAGAGATGGTATGTCTGCTCAATCCGAAGGAAATTATGCAGAAGCTTTACAGAATTATTATGAAGCTATGCGGCTAGAAATTGATCCCTATGATCGAAGTTATATACTCTATAATATAGGCCTTATTCACACAAGTAATGGAGAACACACAAAAGCTTTGGAATATTATTTTCGGGCACTAGAACGAAACCCCTTCTTACCACAAGCTTTAAATAATATGGCCGTGATCTGTCATTACCGAGGAGAACAGGCCATTCGGCAGGGAGATTCTGAAATTGCGGAGGCTTGGTTCGATCAAGCCGCGGAGTATTGGAAACAAGCTATAGCGCTTACTCCCGGAAATTATATTGAAGCGCAGAATTGGTTGAAAATCACAAGGCGTTTCGAATAA

>lcl|NC_010433.1_cds_YP_001718427.1_12 [gene=rpoC2] [locus_tag=MaesCp011] [db_xref=GeneID:5999961] [protein=RNA polymerase beta'' subunit] [protein_id=YP_001718427.1] [location=complement(17421..21602)] [gbkey=CDS]
ATGGAGGTACTTATGGCCGAACGGGCCAATCTGGTCTTTCACAATAAAGCGATAGATGGAACTGCCATTAAACGACTTATTAGCAGATTAATAGATCATTTTGGAATGGCATATACATCACACATCCTGGATCAAGTAAAGACTCTGGGTTTCCAGCAAGCCACCGCTACATCCATTTCATTAGGAATTGATGATCTTTTAACAATACCTTCTAAGGGATGGCTAGTCCAAGATGCTGAACAACAAAGTTTGATTTTGGAAAAACACTATCATTATGGAAATGTACACGCGGTAGAAAAATTACGCCAATCTATTGAGATATGGTATGCTACAAGTGAATATTTGCGACAAGAAATGAACCTGAATTTTAGGATGACGGAACCCTTTAATCCAGTCCATATAATGTCTTTTTCGGGAGCTAGGGGAAATACATCTCAAGTACACCAATTAGTAGGTATGAGAGGATTAATGTCGGATCCACAAGGACAAATGATTGATTTACCCATTCAAAGCAATTTACGCGAGGGACTGTCTTTAACAGAATATATCATTTCTTGCTATGGAGCCCGAAAAGGGGTTGTCGATACTGCTGTACGAACATCAGATGCTGGATATCTTACGCGTAGACTTGTTGAAGTAGTTCAACACATTGTTGTACGTAGAACAGATTGTGGGACCGCCCGAGGGATCTCTGTGAGTCCTCGAAATGGGATGATGCCGGAAAGAATTTTTATTCAAACATTCATTGGTCGTGTATTAGCAGACAATATATATATGGGTCTACGATGCATTGCCATTCGAAATCAAGATATTGGGATTGGGCTTGCCAATCGATTCATAACCTTTCGAACACAAACAATATCTATTCGAACTCCCTTTACTTGTAGGAGTACGTCTTGGATCTGTCGATTATGTTATGGTCGGAGTCCTACTCATGGCGATCTAGTAGAATTGGGGGAAGCCGTAGGTATTATTGCGGGTCAATCCATTGGAGAGCCGGGTACTCAACTAACATTAAGAACGTTTCATACCGGCGGAGTATTCACAGGGGGTACTGCAGAACATGTACGAGCCCCCTCTAATGGAAAAATCAAATTTAATGAGGATTTGGTTCATCCCATACGTACACGTCATGGGCATCCTGCTTTTCTATGTTATATAGACTTGTATGTAACTATTAAGAGTCAAGATATTATACATAACGTGACTATTCCACCAAAAAGTTTCCTTTTAGTTCAAAATGATCAATATGTAGAATCAGAACAAGTGATTGCTGAAATTCGGGCGGGAGCATACACTTTGAATTTTAAAGAGAAGGTCCGAAAACATATTTATTCCGATTCAGAAGGGGAAATGCACTGGAGTACTGATGTGTACCATGCACCCGAATTTACATATAGTAATGTCCATCTCTTACCAAAAACAAGTCATTTATGGATATTATCAGGAAGTTCGTGCAGATCCAGTATAGTTCCTTTTTCACTACACAAGGATCAAGATCAAATGAATGTTCATTCTCTTTCTGTCAAAAGAAGATATATTTCTAGTCCTTCCGTAAATAATGATCAAGTGAAACACAAATTCTTTAGTTCAGATTTTTCGGGTAAAAAAGAAAGTGGGATTCCTGATTATTCAGAACTTAATCGAAGCATATGTACTGGTCATTGTAATCTCATATATTCTACTATTCTCTACAAGAATTCTGATTTATTGGCAAAGAGGCGAAGAAATAAATTCATCATTCCATTCCAATCAATTCAAGAACGAGAGAAAGAACTAATGACCCAATCCGCTATCTCGATTGAGATACCTATAAATGGTATTTTCCGTAGAAATAGTGTTTTTGCTTATTTCGACGATCCCCAATACCGAAAAAAGAGTTCAGGAATTACTAAATATGGGGCTATAGGGGTGCATTCAATCGTCAAAAAAGAGGATTTGATTGAGTATCGGGGAGTCAAAGAATTTAAGCCAAAATACCAAACGAAAGTGGATCGCTTTTTTTTCATTCCCGAGGAAGTGTATATTTTACCCGAATCTTCTTCCCTAATGGTACGGAACAATAGTATTATTGGAGTAGATACACAAATCGCTTTAAATACAAGAAGTCGGGTGGGCGGATTGGTCCGAGTGGAGAGAAAAAAAAAAAAAATGGAACTTAAAATCTTTTCTGGAGATATCCATTTTCCGGGAGAGACAGATAAAATATCCCGACACAGTGATATCTTAATACCACCAGGAACGGTAAAAACAAATTCTAAGGAATCAAAAAAAGTGAAAAATTGGATCTATATCCAACGAATCACACCTACCAAGAAAAAGTATTTTGTTTTGGTTCGACCAGTAATCATATATGAGATAGCGAACGGTATAAATTTAGAAACACTTTTCCCCCAGGATCTATTGCAGGAAAAGGATAATCTGAAACTTCGAGTTGTCAATTATATTCTTTATGGGACTGGTAAACCCATTCGGGGAATTTCTGACACAAGTATTCAATTAGTTCGTACTTGTTTAGTGTTGAATTGGGACCAAGACAAAAAAAGTTCTTCTATCGAAGAGGCCCGCGCTGCTTTTGTTGAAATAAGCACAAATGGTCTGATTCGTGATTTCCTAAGAATCAACCTAGTGAAATTCCATATTTCATATATCGGTAGAAAAAGGAATGATCCATCAGGTTCAGAACCGATCTCTAATAATGGGTCAGATCGTACCAATATTAATCCATTTTATCCCATTTATTCCAAGACAAGGGTTCAACAATCACTTAAACAAAATCAAGGAACTATTAGTACGTTGTTGAATATAAATAAGGAATGTCAATCTTTGATAATTTTGTCATCATCTAATTGTTTTCAAATGGATCCATTCAACGATGTAAAACATCACAATGTAATAAAAGAATCAATTAAAAGAGATCCTATAATTCCAATTAGAAATTCGTTGGGCCCTTTAGGAACAGCCCTTCAAATTGCGAATTTTTATTTATTTTACCATTTAAATTTAATAACTCATAATCAGATCTCGGTAACTAAATATTCGAAACTTTACAATTTAAAACAGACTTTTCAAGTACTTAAATATTATTTAATGGATGAAAACGGGAGAATTGTTAATCCCGATCCATGCAGTAACAGCGTTTTGAATCCATTCAATTTGAATTGGTATTTTCTCCATCATAATTATTGTGAATCTTTCTTCACAATAATTAGCCTGGGACAGTTTATTTGTGAAAATTTATGTATGGCCAAAAACGGACCACATCTAAAATCGGGTCAAGTTATAATTGTTCACATTGACTCTGTAGTAATAAGATCCGCTAAGCCTTATTTGGCCACTCCAGGAGCAACCGTTCATGGCCATTATGGAGAAATCCTTTACGAAGGAAATACATTAGTTACATTTATATATGAAAAATCGAGATCTGGCGATATAACGCAGGGTCTTCCAAAAGTGGAACAAGTGTTAGAAGTGCGTTCAATTGATTCAATATCAATAAACCTAGAAAAGAGAGTGGAGGGTTGGAACGAGTGTATAACAAGAATTCTGGGAATTCCTTGGGGATTCTTGATTGGTACTGAGCTAACTATAGTGCAAAGTCGTATCTCTTTAGTTAATAAGATCCAAAAGGTTTATCGATCCCAAGGGGTGCAGATCCATAATAGGCATATAGAAATTATTGTACGTCAAATAACATCAAAAGTATTGGTTTCAGAAGACGGAATGTCTAATGTTTTTTCACCCGGAGAACTAATTGGATTGTTGCGAGCGGAACGAACGGGACGCGCTTTGGAAGAAGCCATCTGTTACGGGGCCATATTATTGGGAATAACGCGAGCATCTCTGAATACTCAAAGTTTCATATCCGAGGCTAGTTTTCAAGAAACTACTCGCGTTTTAGCAAAAGCTGCTCTCCGCGGTCGTATCGATTGGTTGAAAGGCCTGAAAGAAAACGTTGTTCTAGGCGGTATGATACCCGTTGGTACCGGATTCAAAGGATTAGTGCAAGGCTCAAGGCAACATAAGAACATTCCTTTGAAAACCAAAAAGAAGAATTTATTCGAGGGGGAATTTAGAGATAGAGATATCTTATTCCACCACAGAGAGTTATTTGATTCTTGCATTTCCAAAAATTTATATGATACATCAGAACAATCATTTATAGGATTTAATGATTCCTAA

>lcl|NC_010433.1_cds_YP_001718461.1_46 [gene=rps12] [locus_tag=MaesCp045] [db_xref=GeneID:5999968] [protein=ribosomal protein S12] [exception=trans-splicing] [protein_id=YP_001718461.1] [location=join(complement(74362..74475),146526..146757,147294..147319)] [gbkey=CDS]
ATGCCAACTATTAAACAACTTATTAGAAACACAAGACAGCCAATCAGAAATGTCACCAAATCCCCCGCTCTTGGGGGATGTCCTCAGCGCCGAGGAACATGTACTAGGGTGTATACTATCACCCCCAAAAAACCAAACTCTGCCTTACGTAAAGTTGCCAGAGTACGATTAACCTCTGGATTTGAAATCACTGCTTATATACCTGGTATTGGCCATAATTTACAAGAACATTCTGTAGTCTTAGTAAGAGGGGGAAGGGTTAAGGATTTACCCGGTGTGAGATATCACATTGTTCGAGGAACCCTAGATGCTGTCGGAGTAAAGGATCGTCAACAAGGGCGTTCTAAATATGGGGTCAAAAAGCCAAAATAA

>lcl|NC_010433.1_cds_YP_001718416.1_1 [gene=rps12] [locus_tag=MaesCp046] [db_xref=GeneID:5999972] [protein=ribosomal protein S12] [exception=trans-splicing] [protein_id=YP_001718416.1] [location=complement(join(103390..103415,103952..104183,74362..74475))] [gbkey=CDS]
ATGCCAACTATTAAACAACTTATTAGAAACACAAGACAGCCAATCAGAAATGTCACCAAATCCCCCGCTCTTGGGGGATGTCCTCAGCGCCGAGGAACATGTACTAGGGTGTATACTATCACCCCCAAAAAACCAAACTCTGCCTTACGTAAAGTTGCCAGAGTACGATTAACCTCTGGATTTGAAATCACTGCTTATATACCTGGTATTGGCCATAATTTACAAGAACATTCTGTAGTCTTAGTAAGAGGGGGAAGGGTTAAGGATTTACCCGGTGTGAGATATCACATTGTTCGAGGAACCCTAGATGCTGTCGGAGTAAAGGATCGTCAACAAGGGCGTTCTAAATATGGGGTCAAAAAGCCAAAATAA

>lcl|NC_010433.1_cds_YP_001718462.1_47 [gene=clpP] [locus_tag=MaesCp047] [db_xref=GeneID:6000066] [protein=ATP-dependent Clp protease proteolytic subunit] [protein_id=YP_001718462.1] [location=complement(join(74681..74908,75603..75893,76735..76803))] [gbkey=CDS]
ATGCCTATTGGTGTTCCAAAAGTCCCTTTTCGAAATCCTGGGGAAGACGATTCAACTTGGATTGACATAAACCGACTTTATCGAGAAAGATTACTTTTTTTAGGTCAAGATGTTGATAGCGAGATCGCGAATCAACTTATTGGTCTTATGGTATATCTCAGTATAGAAAACGCGACAAAAGATTTGTATTTGTTTATAAACTCTCCCGGCGGATGGGTAATACCCGGAATAGCTATTTATGATACTATGCAATTTGTGCGACCCGATGTACAAACAGTATGCATGGGATTAGCTGCTTCAATGGGATCTTTTATTCTGGTCGGAGGAAAAATTACCAAACGTTTAGCATTCCCTCATGCTAGGGTAATGATTCATCAACCTATTTCTGGTTATTATGAGGCACAAATAGTAGAATTTGTCCTGGAAGCGGAAGAACTACTGAAACTGCGCGAAATCCTCACAAGGATTTATGCACAAAGAACGGGAAAACCTTTATGGGTTGTATCCGAAGACATGGAAAGAGATGTTTTTATGTCAGCAACCGAAGCCCAAGCTCATGGAATTGTTGATCTTGTAGCAGTTGCATAA

>lcl|NC_010433.1_cds_YP_001718492.1_77 [gene=ndhH] [locus_tag=MaesCp078] [db_xref=GeneID:5999966] [protein=NADH dehydrogenase subunit 7] [protein_id=YP_001718492.1] [location=complement(128313..129494)] [gbkey=CDS]
ATGAATGTACCAGCTACACGAAAAGACCTTATGATAGTTAATATGGGTCCCCACCACCCATCAATGCATGGTGTTCTTCGACTCATCGTTACTCTAGACGGGGAAGATGTTATTGACTGCGAACCAATATTAGGTTATTTACACAGAGGAATGGAAAAAATTGCGGAAAATCGAACAATTATACAATATTTGCCCTATGTAACACGTTGGGATTATTTGGCTACTATGTTCACAGAAGCAATAACAGTAAATGGTCCAGAACTGTTAGGAAATATTCAAGTGCCTAAAAGAGCTGGCTATATCAGAGTAATTATGTTGGAATTGAGTCGTATAGCTTCTCATTTGTTATGGCTTGGCCCTTTTATGGCAGATATTGGTGCACAGACTCCTTTCTTCTATATCTTTAGAGAAAGAGAGTTAGTATATGATTTATTCGAAGCTGCCACTGGTATGAGAATGATGCATAATTTTTTTCGTATCGGAGGAGTAGCGTCTGATCTACCTCATGGTTGGATAGATAAATGTTTGGATTTTTGCGATTATTTTTTAACAGGAGTTACTGAATATCAAAAACTTATTACGCGAAATCCTATTTTTTTAGAACGAGTTGAGGGGGTAGGTATTGTTGGTACAGAAGAAGCAATAAATTGGGGTTTATCAGGACCAATGCTACGAGCTTCTGGAGTACAATGGGATCTTCGTAAAGTTGATCATTATGAGTGTTACGACGAATTTGATTGGGAAATCCAGTGGCAAAAAGAAGGAGATTCCTTAGCTCGTTATTTAGTCCGGATTGGTGAAATGCTGGAATCTATAAAAATTATTCAACAGGCTCTGGAAGGAATTCCGGGGGGGCCCTATGAGAATTTAGAAACCCGACGCTTTGATAGAGAAAGGGATTCGGAATGGAACGATTTCGAATATCGATTCATTAGTAAAAAAACTTCTCCTACTTTTGAATTACCGAAACAAGAACTTTATGTGAGAGTCGAAGCACCAAAAGGAGAATTGGGAATTTTTCTGATAGGGGATCAGAGCGGTTTTCCTTGGAGATGGAAAATTCGTCCGCCAGGTTTTATCAATTTGCAAATTCTTCCTGAATTAGTTAAAAGAATGAAATTGGCTGATATTATGACAATACTAGGTAGTATAGATATCATTATGGGAGAAGTTGATCGTTGA

>lcl|NC_010433.1_cds_YP_001718443.1_28 [gene=atpE] [locus_tag=MaesCp027] [db_xref=GeneID:5999950] [protein=ATP synthase CF1 epsilon subunit] [protein_id=YP_001718443.1] [location=complement(55411..55812)] [gbkey=CDS]
ATGACCTTAAATCTTTGTGTACTGACCCCGAATCGAATTGTTTGGGATTCAGAAGTGAAAGAAATCATTTTATCTACTAATAGTGGACAAATTGGCGTATTACCAAACCATGCACCTATTGCCACAGCTGTCGATATCGGTATTTTGAGAATACGCCTTAATGACCAATGGTTAACGATGGCTCTGATGGGTGGTTTTGCTAGAATAGGCAATAATGAGATTACTGTTTTAGTAAATGATGCAGAGAAGGGTAGTGACATTGATCCACAAGAAGCTCAGCAAACTCTTGAAATAGCAGAAGCTAACTTGAGGAAAGCGGAAGGCAGGAGACAAATAATTGAGGCAAATCTAGCTCTCAGACGAGCTAGGGCACGAGTAGAGGCTCTCAATGAGATTTCGTAA

>lcl|NC_010433.1_cds_YP_001718428.1_13 [gene=rpoC1] [locus_tag=MaesCp012] [db_xref=GeneID:6000060] [protein=RNA polymerase beta' subunit] [protein_id=YP_001718428.1] [location=complement(join(21771..23381,24158..24589))] [gbkey=CDS]
ATGATTGATCGGTATAAACATCAACAACTCCGAATTGGATCAGTTTCGCCTCAACAAATAAGTGCTTGGGCCAATAAAATCCTACCTAACGGAGAGATTGTTGGAGAGGTGACAAAACCCTATACTTTTCATTACAAAACCAATAAACCTGAAAAGGATGGATTATTTTGTGAAAGAATTTTTGGGCCAATAAAAAGTGGAATTTGTGCGTGTGGAAATTATCGAGTAATCAGGAATGAAAAAGAAGACCAAAAATTTTGTGAACAATGCGGAGTCGAATTTGTTGATTCTCGGATACGAAGATATCAAATGGGCTACATCAAACTGGCATGCCCAGTAACTCATGTGTGGTATTTGAAACGTCTTCCTAGTTATATCGCAAATCTTTTAGATAAACCTCTTAAAGAATTAGAAGGCCTAGTATACTGCGATTTTTCTTTTGCTAGGCCCATAGCTAAAAAACCTACTTTTTTACGATTACGAGGTTCATTCGAATATGAAATCCAATCCTGGAAATACAGTATTCCGCTTTTTTTTACTACCCAATGCTTCGATACATTTCGAAATCGAGAAATTTCTACAGGAGCTGGTGCTATCCGAGAACAATTAGCCGATCTGGATTTGCGAATTATTATAGATTATTCATCGGTAGAATGGAAAGAATTAGGGGAAGAAGGGCCTACCGGGAATGAATGGGAAGATCGAAAAGTTGGAAGAAGAAAGGATTTTTTGGTTAGACGTGTGGAATTAGCTAAGCATTTTATTCGAACAAATATCGAACCAGAATGGATGGTTTTATGTCTATTACCAGTTCTTCCTCCCGAGTTGAGACCGATCATTCAGATAGATGGGGGTAAACTAATGAGTTCAGATATTAATGAACTCTATAGAAGAGTTATCTATCGGAACAATACTCTTATTGATCTATTAACAACAAGTAGATCTACCCCAGGGGAATTAGTAATGTGTCAGGAGAAATTGGTACAAGAAGCCGTGGATACACTTCTTGATAATGGAATCCGCGGACAACCAATGAGGGACGGTCATAATAAGGTTTACAAGTCGTTTTCGGATGTAATTGAAGGCAAAGAAGGAAGATTTCGTGAGACTATGCTTGGCAAACGGGTTGATTATTCGGGGCGTTCTGTCATTGTCGTAGGCCCCTCACTTTCATTACATCGATGTGGATTGCCTCGCGAAATAGCAATAGAGCTTTTCCAGATATTTGTAATTCGTGGTCTAATTAGACAACATCTTGCTTCGAACATAGGAGTTGCTAAGAGTAAAATTCGGGAAAAAGAGCCAATTGTATGGGAAATACTTCACGAAGTTATGCAGGGGCATCCAGTATTACTGAATAGAGCGCCGACTCTGCATAGATTAGGCATACAGGCATTCCAACCCATTTTAGTGGAAGGCCGCGCTATTTGTTTACATCCATTAGTTTGTAAGGGATTCAATGCAGACTTTGATGGGGATCAAATGGCTGTTCATGTACCCTTATCGTTGGAGGCTCAAGCGGAGGCTCGTTTACTTATGTTTTCTCATATGAATCTTCTGTCTCCAGCTATTGGAGATCCCATTTCCGTACCAACTCAAGATATGCTTATTGGGCTCTATGTATTAACAAGCGGGAATCGTCGAGGTATTTGTGCAAATAGGTATAATCCATGTAATCGCAGAAATTATCAAAATAAAAGAATTGACGGTAATAACGATAAATATACGAAAGAACCCCTTTTTTCTAATTCCTATGATGCACTTGGCGCTTATCGGCAGAAAAGAATCCATTTAGATAGTCCTTTGTGGCTCCGGTGGCAGCTAGATCAACGCGCTATTACTTCAAGAGAAGCTCCCATCGAAGTTCACTATGAATCTTTGGGTACCTATCATGAGATTTATGAACACTATCTAATAGTAAGAAATATAAAAAAAGAAATTCTTTGTATATACATTCGAACTACTGTTGGTCATATTTCTCTTTATCGAGAAATCGAAGAAGCTATACAAGGGTTTTGCCAAGCCTGCTCAGATGGTATCTAA

>lcl|NC_010433.1_cds_YP_001718469.1_54 [gene=rpoA] [locus_tag=MaesCp054] [db_xref=GeneID:6000056] [protein=RNA polymerase alpha subunit] [protein_id=YP_001718469.1] [location=complement(82862..83893)] [gbkey=CDS]
ATGGTTCGAGAGAAAATAACAATATCCACTCGGACACTGCAGTGGAAATGTATTGAATCAAGAACCGATAATAAACGTCTTTATTATGGACGCTTTATTCTGTCTCCACTTATGAAAGGACAAGCCGACACAATAGGCATTGCGATGCGAAGAGCTTTGCTTGGAGAAATAGAAGGAACATGTATCACACGTGCAAAATCTGAGAAAATACCACACGAATTTTCTACTATAGCAGGTATTCAAGAATCAATACATGAAATTTTAATGAATTTGAAAGAAATTGTATTAAGAAGCAATTTGTATGGAACTTGTGACGCATCCATTTGTGTCAAGGGTCCTGGATATGTAACTGCTCAAGACATCATCTTACCGCCTTTTGTGGAAATCATTGATAATACACAGCATATCGCTAGCCTAACGAAAGCAATTGATTTGTGTATTGGATTACAAATCGAGAGGAATCGTGGCTATCGTATAAAACCAACAAATAACTTTCAAGTTCAAGACGGGAGTTATTCTATAGATGCTGTATTCATGCCTGTTCGAAATGCGAATCATAGTGTTCATTCTTATGGAAATGGGAATGAAAAGCAAGAGATACTTTTTCTCGAAATATGGACAAATGGAAGTTTAACTCCTAAAGAAGCACTTCACGAAGCCTCCCGTAATTTGATTGATTTTTTTATTCCTTTTCTACATGCAGAAGAAGAAAACTTACATTTAGAAATAGAAAAAAATCAACACAAGGTTACTTTACCCCTTTTTTGTTTTCATGATAAATTGACTAAATTAAGAAAAAATAAAAAAGAAATAGTATTGAAATACATTTTTATTGACCAATCAGAATTGACTCCTAAGATCTATAATTGCCTCAAAAGATCCAATATACATACATTATCGGATCTTTTGAATAAGAGTCAAGAAGATCTTATGAAAATTGAACATTTTCGCATAGACGATGTAAAACATATATTGGGTATTCTAGAAATAGAAAAACATTTCACAATTGATTTACCAAAGAATAAAATATAA

>lcl|NC_010433.1_cds_YP_001718423.1_8 [gene=atpF] [locus_tag=MaesCp007] [db_xref=GeneID:6000008] [protein=ATP synthase CF0 B subunit] [protein_id=YP_001718423.1] [location=complement(12838..13392)] [gbkey=CDS]
ATGAAAAATATAACCGATTCTTTCGTTTCCTTGGGTCACTGGCCATCCGCCGGGAGTTTCGGGTTTAATACCGATATTTTAGCAACAAATCTAATAAATCTAAGTGTAGTCCTTGGTGTATTGATTTTTTTTGGAAAGGGGGTGTTAAGTGATTTATTAGATAATCGAAAACAAAGGATTTTGGATACTATTCGAAATTCAGAAAAACTACGCGAGGGGGCTATTGAACAGCTGGAAAAAGCCCGGGCCCGCTTACGGAAAGTGGAAATAGAAGCAGATCAGTTTCGAACGAATGGATATTCTGAGATAGAACGAGAAAAATTGAATTTGATTAATTCAACTTATAAGACTTTGGAACAATTAGAAAATTACAAAAATGAAACCATTCATTTTGAACAACAACGAACGATTAATCAAGTCCGACAACGGGTTTTCCAACAAGCCTTACAAGGAGCTCTAGGAACTCTGAATAGTTGTTTGACCAACGAGTTGCATTTACGTACCATCAATGCTAATCTTGGCATGTTTGGCGCGATAAAAGAAATAACTGATTAG

>lcl|NC_010433.1_cds_YP_001718496.1_81 [gene=ndhB] [locus_tag=MaesCp082] [db_xref=GeneID:6000058] [protein=NADH dehydrogenase subunit 2] [protein_id=YP_001718496.1] [location=join(148156..148932,149615..150370)] [gbkey=CDS]
ATGATCTGGCATGTACAGAATGAAAACTTCATTCTCGATTCTACGAGAATTTTTATGAAAGCCTTTCATTTGCTTCTCTTCGATGGAAGTTTTATTTTCCCAGAATGTATCCTAATTTTTGGCCTAATTCTTCTTCTGATGATCGATTCAACCTCTGATCAAAAAGATATACCTTGGTTATATTTCATCTCTTCAACAAGTTTAGTAATGAGTATAACGGCCCTATTGTTCCGATGGAGAGAAGAACCTATGATTAGCTTTTCGGGAAATTTCCAAACGAACAATTTCAACGAAATCTTTCAATTTCTTATTTTACTATGTTCAACTCTATGTATTCCTCTATCCGTAGAGTACATTGAATGTACAGAAATGGCTATAACAGAGTTTCTCTTATTCGTATTAACAGCTACTCTAGGAGGAATGTTTTTATGCGGTGCTAACGATTTAATAACTATCTTTGTCGCTCCAGAATGTTTCAGTTTATGCTCCTACCTATTATCTGGATATACCAAGAAAGATGTACGGTCTAATGAGGCTACTACGAAATATTTACTCATGGGTGGGGCAAGCTCTTCTATTCTGGTTCATGCTTTCTCTTGGCTATATGGTTCGTCCGGGGGAGAGATCGAGCTTCAAGAAATAGTGAATGGCCTTATCAATACACAAATGTATAACTCCCCAGGAATTTCAATTGCGCTTATATTCATCACTGTAGGAATTGGGTTCAAGCTTTCCCTAGCCCCTTCTCATCAATGGACTCCTGACGTATACGAAGGATCTCCCACTCCAGTCGTTGCTTTTCTTTCTGTTACTTCGAAAGTAGCTGCTTCAGCTTCAGCCACTCGAATTTTCGATATTCCTTTTTATTTCTCATCAAACGAATGGCATCTTCTTCTGGAAATCCTAGCTATTCTGAGCATGATAGTGGGGAATCTCATTGCTATTACTCAAACAAGCATGAAACGTATGCTTGCATATTCGTCCATAGGTCAAATCGGATATGTAATTATTGGAATAATTGTTGGAGACTCTAATGGTGGATATGCAAGCATGATAACTTATATGCTCTTCTATATCTCCATGAATCTAGGAACTTTTGCTTGTATTGTATTATTTGGTCTACGTACCGGAACTGATAACATTCGAGATTATGCAGGATTATACACGAAAGATCCTTTTTTGGCTCTCTCTTTAGCCCTATGTCTCTTATCCCTAGGAGGTCTTCCTCCACTAGCAGGTTTTTTCGGAAAACTCCATTTATTCTGGTGTGGATGGCAGGCAGGCCTATATTTCTTGGTTTTAATAGGACTCCTTACGAGCGTTGTTTCTATCTACTATTATCTAAAAATAATCAAGTTATTAATGACTGGACGAAACCAAGAAATAACCCCTCACGTGCGAAATTATAGAAGATCCCCTTTAAGATCAAACAATTCCATCGAATTGAGTATGATTGTATGTGTGATAGCATCTACTATACCAGGAATATCAATGAACCCGATTGTTGAAATTGCTCAAGATACCCTTTTTTAG

>lcl|NC_010433.1_cds_YP_001718470.1_55 [gene=rps11] [locus_tag=MaesCp055] [db_xref=GeneID:6000046] [protein=ribosomal protein S11] [protein_id=YP_001718470.1] [location=complement(83952..84368)] [gbkey=CDS]
ATGGCAAAACCTTTACCAAAAATTAGTTCACGCAGAAATGGACGTATTGGTTCACGTAAGAATTCACGTAAAATACCAAAAGGCGTTATTCATGTTCAAGCAAGTTTCAACAATACTATTGTGACCATTACAGATGTACGGGGTCGAGTGATTTCTTGGTCCTCCGCTGGCACTTGTGGATTCAGGGGCACAAGAAGAGGAACGCCATTTGCTGCTCAAACCGCAGCAGGAAATGCTATTCGGACAGTAGTGGATCAAGGTATGCAACGAGCAGAAGTCATGATAAAGGGTCCTGGTCTCGGACGAGATGCGGCATTAAGAGCTATTCGCAGAAGTGGTATACTATTAAGTTTCGTCCGGGATGTAACCCCTATGCCACATAATGGCTGCCGACCCCCTAAAAAAAGGCGCGTGTAA

>lcl|NC_010433.1_cds_YP_001718450.1_35 [gene=petA] [locus_tag=MaesCp034] [db_xref=GeneID:5999988] [protein=cytochrome f] [protein_id=YP_001718450.1] [location=65788..66750] [gbkey=CDS]
ATGCAAACTAGAAAGACCTTTTCTTGGATAAAAGAAGAGATTACTCGTTCAATTTCCGTATTGCTCATGATATATATAATAACTTGGGCATCCATTTCAAATGCATATCCCATTTTTGCACAGCAGGGTTATGAAAATCCACGCGAAGCAACTGGTCGTATTGTATGTGCCAATTGTCATTTAGCTAATAAACCCGTGGATATTGAGGTTCCACAAGCGGTACTTCCAGATACTGTATTTGAAGCAGTTGTTCGAATTCCTTATGATATGCAACTGAAACAAGTTCTTGCTAATGGTAAAAAGGGGGCTTTGAACGTGGGGGCTGTTCTTATTTTACCTGAGGGGTTTGAATTAGCCCCTCCCGATCGTATTTCGCCAGAGATGAAAGAAAAGATGGGAAATCTGTCTTTTCAGAGTTATCGCCCCACTAAAAAAAATATTCTTGTGATAGGTCCTGTTCCTGGTCAGAAATATAGTGAAATTACCTTTCCTATTCTTTCTCCGGACCCCGCCGCTAAGAAAGATGTTCACTTTTTAAAATATCCCATATATGTAGGCGGAAACAGGGGAAGGGGTCAGATTTATCCCGACGGGAGCAAGAGCAACAATACGGTTTATAATGCTACAGCAGCAGGTATAGTAAGCAAAATCATACGAAAAGAAAAAGGGGGGTACGAAATAACCATAACGGATGCGTCAGAGGGACGTCAAGTGATTGATATTATACCTCCAGGACCAGAACTTCTTGTTTCAGAAGGCGAATCCATTAAACTTGATCAACCATTAACGAGTAATCCTAATGTGGGTGGATTTGGTCAGGGGGATGCAGAAATAGTACTTCAAGACCCATTACGCGTCCAAGGCCTTTTGTTCTTCTTGGCATCCGTTATTTTGGCACAAATCTTTTTGGTTCTTAAAAAGAAACAGTTTGAGAAGGTTCAATTGTCCGAAATGAATTTCTAG

5. *Ricinus communis*

>lcl|NC_016736.1_cds_YP_005090203.1_47 [gene=clpP] [locus_tag=RCOM_ORF00069] [db_xref=GeneID:11542344] [protein=ATP-dependent Clp protease proteolytic subunit] [protein_id=YP_005090203.1] [location=complement(join(75314..75541,76193..76483,77380..77448))] [gbkey=CDS]
ATGCCTATTGGTGTTCCAAAAGTCCCTTTTCGAAATCCTGGGGAAGACGATTCCATTTGGATTGACGTAAACCGACTTTATCGAGAAAGATTACTTTTTTTAGGTCAAGATGTTGATAGCGAGATCTCGAATCAACTTATTGGTCTTATGGTATATCTCAGTATAGAGAGCGAGACTAAAGATTTGTATTTGTTTATAAACTCTCCTGGGGGATGGGTAATACCCGGAATAGCTATTTATGATACTATGCAATTTGTGCGACCAGATGTACAAACAGTATGCATGGGATTAGCTGCTTCAATGGCATCTTTTATCCTAGTTGGAGGAAAAATTACCAAACGTTTAGCATTCCCTCATGCTAGGGTAATGATTCATCAACCTATTGCTGGTTTTTATGAGGCCCAAATAGGAGAATTTGTCCTGGAAGCAGAAGAACTACTGAAACTGCGCGAAATCCTCACAAGGATTTATGCACAAAGAACGGGAAAACCTTTATGGGTTGTATCCGAAGACATGGAAAGAGATGTTTTTATGTCAGCAGCAGAAGCCCAAGCTCATGGAATTGTTGATCTTGTAGCAGTTGCATAA

>lcl|NC_016736.1_cds_YP_005090213.1_57 [gene=rps8] [locus_tag=RCOM_ORF00079] [db_xref=GeneID:11542355] [protein=ribosomal protein S8] [protein_id=YP_005090213.1] [location=complement(85739..86143)] [gbkey=CDS]
ATGGGCAGGGATCCTATTGCTGAGATAATAACCTCTATACGAAATGCTGACATGAATAGAAAAGGAACCGTTCGAATAGCATTCACTAACATCACTGAAAACATTATTAAAATACTTTTACGAGAAGGTTTTATTGAAAATGTCAGGAAACACCAGGAAGGCAACAAAAAATTTTTGGTTTTAACCCTACGACATAGAAGGAAGAGGAAGGGACCCTATAGAACTAGTCTAAATTTAAAACGGATTAGCCGACCTGGTCTACGAATCTATTCTAACTATCAAAAAATTCCTAGAATTTTGGGCGGGATGGGTATTGTAATTCTTTCTACTTCTCGGGGTATAATGACCGACCGAGAAGCTCGACTAGAAAGAATCGGTGGAGAAATCTTGTGTTATATATGGTAA

>lcl|NC_016736.1_cds_YP_005090186.1_30 [gene=rbcL] [locus_tag=RCOM_ORF00051] [db_xref=GeneID:11542325] [protein=ribulose-1,5-bisphosphate carboxylase/oxygenase large subunit] [protein_id=YP_005090186.1] [location=58961..60388] [gbkey=CDS]
ATGTCACCACAAACAGAGACTAAAGCAAGTGTTGGGTTCAAGGCTGGTGTTAAAGATTATAAATTGACTTATTATACTCCTGAATATGAAACCAAAGATACTGATATCTTGGCAGCATTCCGAGTAACTCCTCAACCTGGAGTTCCGCCTGAGGAAGCAGGAGCTGCAGTAGCTGCTGAATCTTCTACTGGTACATGGACAACTGTGTGGACCGATGGGCTTACCAGTCTTGATCGTTATAAAGGACGATGCTACCACATTGAGCCCGTTGCCGGAGAAGAAAATCAATTTATTGCTTATGTAGCTTACCCCTTAGACCTTTTTGAAGAAGGTTCTGTTACTAACATGTTTACTTCCATTGTGGGTAATGTATTTGGGTTCAAAGCCCTACGCGCCCTACGTCTGGAGGATTTGCGAATCCCTCCTGCTTATACAAAAACTTTCCAAGGGCCGCCTCATGGCATCCAAGTTGAGAGAGATAAATTGAACAAGTATGGTCGCCCTCTATTGGGTTGTACTATTAAACCTAAATTGGGGCTATCCGCTAAGAATTACGGTAGAGCAGTTTATGAATGTCTACGCGGTGGACTTGATTTTACCAAAGATGATGAGAACGTGAACTCCCAACCATTTATGCGTTGGAGAGACCGTTTCTTATTTTGTGCCGAAGCAATTTATAAAGCACAGGCTGAAACAGGTGAAATCAAAGGACATTATTTGAATGCTACTGCAGGTACATGCGAAGAAATGATCAAAAGGGCTGTATTTGCCAGAGAATTAGGAGTTCCTATCGTAATGCATGACTACTTAACAGGGGGATTCACTGCAAATACTACCTTGGCTCATTATTGCCGAGATAATGGTTTACTTCTTCACATTCACCGCGCAATGCATGCAGTTATTGATAGACAGAAGAATCATGGTATGCATTTTCGTGTACTAGCTAAGGCGTTACGTATGTCTGGTGGAGATCATATTCACGCTGGTACCGTAGTAGGTAAACTTGAAGGGGAAAGAGACATCACTCTGGGCTTTGTTGATTTATTGCGTGATGATTTTATTGAAAAAGATCGAAGCCGCGGTATTTATTTCACTCAAGATTGGGTCTCTCTACCTGGTGTTCTGCCTGTAGCTTCAGGGGGTATTCACGTTTGGCATATGCCTGCTCTGACCGAGATCTTTGGAGATGATTCCGTACTACAATTCGGTGGAGGAACTTTAGGGCACCCTTGGGGAAATGCACCCGGTGCCGTAGCTAATCGAGTAGCTCTAGAAGCATGTGTACAAGCTCGTAATGAGGGACGTGATCTTGCTCGCGAGGGTAATGAAATTATCCGCGAAGCTGGCAAATGGAGTCCTGAATTAGCTGCTGCTTGTGAAGTATGGAAGGAGATTAAATTTGAATTCCCAGCAATGGATACTTTGTAA

>lcl|NC_016736.1_cds_YP_005090183.1_27 [gene=ndhC] [locus_tag=RCOM_ORF00046] [db_xref=GeneID:11542320] [protein=NADH dehydrogenase subunit 3] [protein_id=YP_005090183.1] [location=complement(54298..54660)] [gbkey=CDS]
ATGTTTCTGATTTACGAATATGATATATTCTGGGCATTTCTAATAATATCAAGTGTTATTCCTATTTTAGCATTTCTAATTTCCGGAGTTTTATCCCCAATTAGCAAAGGGCCGGAGAAACTTTCTAGTTATGAATCGGGGATAGAACCAATGGGCGATGCTTGGTTACAATTTCGAATCCGTTATTATATGTTTGCTCTAGTTTTTGTTGTTTTTGATGTTGAAACGGTTTTTCTTTATCCATGGGCAATGAGTTTCGATGTATTGGGGTTATCCGTATTTATAGAAGCTTTAATTTTCGTGCTTATCCTAATAGTTGGTTCAGTTTATGCATGGAGAAAAGGAGCATTAGAGTGGTCTTAG

>lcl|NC_016736.1_cds_YP_005090170.1_14 [gene=rpoB] [locus_tag=RCOM_ORF00021] [db_xref=GeneID:11542295] [protein=RNA polymerase beta subunit] [protein_id=YP_005090170.1] [location=complement(25211..28423)] [gbkey=CDS]
ATGCTCGGGGATGGAAATGAGGGAATGTCTACAATACCTGGATTGAATCAGATACAATTTGAAGGATTTTGTAGGTTCATTGATCAGGGCTTAACAGAAGAACTTTATAAGTTTCCAAAAATTGAAGATACAGATCAAGAAATTGAATTTCAATTATTTGTGGAAACATATCAATTAGTAGAACCGTTGATAAAAGAAGGAGATGCTGTATATGAATCACTTACATATTCTTCTGAATTATATATATCCGCGGGATTAATTTGGAAAACTAGTAGGGATATGCAAGAACAAACTATTTTTATTGGAAACATTCCTCTAATGAATTCCCTGGGAACTTTTATAATAAATGGAATATACAGAATTGTGATCAATCAAATATTGCAAAGTCCCGGTATCTATTACCGGTCAGAATTGGATCATAACGGAATTTCGGTCTATACCGGCACCATAATATCAGATTGGGGGGGGCGAGTAGAATTAGAGATTGATAGAAAAGCAAGGATATGGGCTCGTGTAAGTAGGAAACAGAAAATATCTATTCTAGTTCTATTATCCGCTATGGGTTTGAATCTAAGAGAAATTTTAGAGAATGTGCGCTACCCTGAAATTTTCTTATCTTTCCTGAATGATAAGGAAAAAAAAAAAATTGGGTCAAAGGAAAATGCCATTTTGGAGTTTTATCAACAATTTACTTGTGTAGGCGGAGATCCGGTATTTTCTGAATCCTTATGTAAGGAATTACAAAAGAAATTCTTTCAACAAAGATGTGAATTAGGAAGGATTGGTCGATTAAATATGAACCGGAGACTGAATCTTGATATACCTCATAACAATACATTTTTGTTACCGCGAGATATATTGGCAGCTGCGGATCATTTGATTGGAATGAAATTTGGAATGGGTACACTTGACGATATGAATCATTTAAAAAATAAACGTATTCGTTCTGTAGCGGATCTTTTACAAGATCAATTCGGATTGGCTCTGATTCGTTTAGAAAATGTGGTTAGAGGGACTATATGTGGAGCAATTAGGCATAAATTGATACCGACTCCTCAAACTTTGGTAACTTCAACTCCATTAACAACCACTTATGAATCTTTTTTCGGGTTACACCCATTATCTCAAGTTTTGGATCGAACTAATCCATTGACACAAATAGTTCATGGGAGAAAATCGAGTTATTTGGGTCCTGGAGGATTGACAGGGCGAACTGCTAGTTTTCGGATACGAGATATCCACCCTAGTCACTATGGGCGGATTTGCCCAATTGACACATCTGAAGGAATCAATGTTGGACTTATTGGATCTTTAGCAATTCATGCCAAGATTGGTCATTGGGGGTCTTTAGAAAGCCCATTTTATGTAATCTCTGAGGAATCAAAAAAAGTACGGATGTTTTATTTATCACCAAATAGAGAGGAATACCATATGGTAGCGGCAGGAAATTCTTTGGCGCTAAATCGAGGTGTTCAGGAAGAACAGGTTGCCCCAGCTCGATATCGTCAAGAATTCCTGACTATTGCATGGGAACAAGTGCATCTTCGAAGTATTTTTCCCTTCCAATATTTTTCTATCGGAGCTTCCCTCATTCCTTTTATCGAGCATAATGATGCGAATCGGGCTTTAATGAGTTCTAATATGCAACGTCAAGCAGTTCCACTTTCTCGGTCCGAAAAATGCATTGTTGGGACTGGATTGGAACGCCAAGTGGCTCTAGATTCAGGGGTTCCCGCTATAGCCGAACGCGAAGGAAAGATAATTTATACTGATATTGACAAGATCATTTTATCGGGCAATGGGGATACTCTACGCATTCCATTAGTTATGTATCAACGTTCCAACAAAAATACTTGTATGCATCAAAAACCCCAGGTTCCGCGGGGTAAATGCATTAAAAAGGGACAAGTTTTAGCGGATGGTGCCGCTACAATTGGTGGCGAACTCGCCTTGGGCAAAAACGTATTAGTGGCTTATATGCCGTGGGAAGGTTACAATTTTGAGGATGCGGTACTCATTAGCGAACGTCTGGTATATGAAGATATTTATACTTCTTTTCACATACGGAAATATGAAATTCAGACTCATGTGACAAGCCAAGGACCTGAAAGGATCACTAATGAAATACCGCATCTAGAAGCCCATTTACTCCGAAATTTAGACAAAAATGGAATTGTGATGCTAGGATCTTGGGTAGAGACGGGCGATATTTTAGTAGGTAAATTAACGCCTCAAATGGCGAAAGAATCATCGTATGCTCCCGAAGATAGATTATTAAGAGCCATACTTGGTATTCAGGTATCTACTTCAAAGGAAACTTGTCTAAAACTACCTATAGGTGGTAGGGGGCGAGTTATTGATGTGAGATGGGTCCAGAAAAAGGGGGGTTCCAGTTATAATCCGGAAACGATTCGTATATATATTTTACAGAAACGTGAAATCAAAGTGGGTGATAAAGTCGCTGGAAGACATGGAAATAAAGGCATCATTTCCAAAATTTTGCCTAGACAAGATATGCCTTATTTGCAAGATGGAAGGCCTGTTGATATGGTCTTCAACCCATTAGGAGTACCTTCACGAATGAATGTAGGACAGATATTTGAATGCTCACTCGGGTTAGCGGGAGGTCTGCTAGATAGACATTATCGAATAGCACCCTTTGATGAGAGATATGAACAAGAGGCCTCGAGAAAACTTGTATTTTCTGAATTATATGAAGCCAGTAAGCAAACAGCAAATCCGTGGGTATTTGAACCCGAATATCCGGGAAAAAGTAGAATATTTGATGGAAGAACGGGGGATCCCTTTGAGCAGCCTGTTATAATAGGAAAGCCTTATATCTTGAAATTAATTCATCAAGTTGATGATAAAATACATGGACGTTCCAGTGGACATTATGCACTTGTTACCCAACAACCCCTTAGAGGAAGGGCCAAGCAAGGGGGACAACGAGTCGGAGAAATGGAGGTTTGGGCTCTAGAAGGATTTGGTGTTTCTCATATTTTACAAGAAATGCTTACTTATAAATCTGATCATATTAGAGCTCGCCAAGAAGTGCTTGGTACTACAATCATCGGAGGAACAATACCTAAACCTGAAGACGCTCCAGAATCTTTTCGATTGCTCGTTCGAGAACTACGATCTTTGGCTCTGGAACTGAATCATTTCCTTGTATCTGAGAAGAACTTCCAGATGAATAGGAAGGAAGCTTAA

>lcl|NC_016736.1_cds_YP_005090230.1_74 [gene=ndhE] [locus_tag=RCOM_ORF00108] [db_xref=GeneID:11542384] [protein=NADH dehydrogenase subunit 4L] [protein_id=YP_005090230.1] [location=complement(125340..125645)] [gbkey=CDS]
ATGATGCTCGAACATGTACTCGTTTTGAGTGCCTATTTATTTTCTATCGGTATCTATGGATTGATCACGAGTAGAAATATGGTTAGAGCCCTTATGTGCCTTGAACTTATACTGAATGCAGTTAATATAAATTTCGTAACATTTTCTGATTTTTTTGATAGTCGCCAATTAAAGGGAAATATTTTTTCAATTTTTGTTATAGCTATTGCAGCCGCTGAAGCAGCTATTGGACCAGCTATCGTTTCGTCAATTTATCGTAACAGAAAATCAATCCGTATCAATCAATCGAATTTGTTGAATAAGTAG

>lcl|NC_016736.1_cds_YP_005090237.1_81 [gene=rps7] [locus_tag=RCOM_ORF00124] [db_xref=GeneID:11542400] [protein=ribosomal protein S7] [protein_id=YP_005090237.1] [location=148641..149108] [gbkey=CDS]
ATGTCACGTCGAGGTACTGCAGAAGAAAAAACTGCAAAATCCGATCCAATTTATCGTAATCGATTAGTTAACATGTTGGTTAACCGTATTCTGAAACACGGAAAAAAACCATTGGCTTATCAAATTATCTATCGAGCCATGAAAAAGATTCAACAAAAGACAGAAACAAATCCACTATCTGTTTTACGTCAAGCAATACGTGGAGTAACTCCCGATATAGCAGTAAAAGCAAGACGTGTAGGCGGATCGACTCATCAAGTTCCCGTTGAAATAGGATCCACACAAGGAAAAGCACTTGCCATTCGTTGGTTATTAGGGGCATCCCGAAAACGTCCGGGTCGAAATATGGCTTTCAAATTAAGTTCCGAATTAGTGGATGCTGCCAAAGGGAGTGGTGATGCCATACGCAAAAAGGAAGAGACTCATAGAATGGCAGAGGCAAATAGAGCTTTTGCACATTTTCGTTAA

>lcl|NC_016736.1_cds_YP_005090234.1_78 [gene=ndhH] [locus_tag=RCOM_ORF00112] [db_xref=GeneID:11542388] [protein=NADH dehydrogenase subunit 7] [protein_id=YP_005090234.1] [location=complement(129673..130848)] [gbkey=CDS]
ATGAATGTATCAGCTACACGAAAAGACCTTATGATAGTCAATATGGGTCCCCACCACCCATCAATGCATGGTGTTCTTCGACTCATCGTTACTCTAGACGGTGAAGATGTTATTGACTGCGAACCAATTTTAGGTTATTTACACAGAGGGATGGAAAAAATTGCGGAAAATCGAACAATTATACAATATTTGCCCTATGTAACACGCTGGGATTATTTGGCTACTATGTTCACAGAAGCAATAACAGTAAATGGTCCAGAACTGTTAGGAAATATTCAAGTGCCTAAAAGAGCTAGCTATATCAGAGTAATTATGTTGGAATTGAGTCGTATAGCTTCTCATTTGTTATGGCTTGGTCCGTTTATGGCGGATATTGGTGCACAGACTCCTTTCTTTTATATTTTTAGAGAAAGAGAGTTAGTATATGATTTATTCGAAGCTGCCACCGGTATGAGAATGATGCATAATTATTTTCGTATCGGGGGAGTAGCGGCCGATCTACCTCATGGATGGATAGATAAATGTTTGGATTTTTGCGATTATTTTTTAACAGGAATTGCTGAATATCAAAAACTTATTACGCGAAATCCTATTTTTTTAGAACGAGTTGAAGGAGTAGGTATTGTTGGTGCAGAGGAAGCAATAAATTGGGGTTTATCCGGACCAATGCTACGAGCTTCCGGAGTACAATGGGATCTTCGTCAAGTTGATCATTATGAGTGTTACGACGAATTTGATTGGGAAGTCCAGTGGCAAAAAGAAGGAGATTCATTAGCTCGTTATTTAGTCCGAATCGGTGAAATGATGGAATCTATAAAAATTATTCAACAGGCTCTGGAAGGAATCCCGGGGGGGCCCTATGAGAATTTAGAAACCCGACGTTTTGAAAGGGATCCAGAATGGAACGATTTCGAATATCGATTCATTAGTAAAAAAACTTCTCCTACTTTTGAATTACCGAAACAAGAACTTTATGTGAGAGTCGAAGCCCCAAAAGGAGAATTGGGGATTTTTCTGATAGGGGATCAGAGTGGTTTTCCTTGGAGATGGAAAATTCGCCCGCCGGGTTTTATCAATTTGCAAATTCTTCCCGAATTAGTTAAAAGAATGAAATTGGCCGATATTATGACAATACTAGGTAGTATAGATATCATTATGGGAGAAGTTGATCGTTGA

>lcl|NC_016736.1_cds_YP_005090239.1_83 [gene=ycf2] [locus_tag=RCOM_ORF00127] [db_xref=GeneID:11542405] [protein=Ycf2] [protein_id=YP_005090239.1] [location=complement(153326..160186)] [gbkey=CDS]
ATGAAAGGACATCAATTCAAATCCTGGATTTTCGAATTGAGAGAGATATTGAGAGAGATCAAGAATTCTCACTATTTCTTAGATTCATGGACCCAATTCAATTCCGTGGGATCTTTCATTCACATTTTTTTCCATCAAGAACGTTTTATAAAACTCTTGGACTCCCGAATTTGGAGTATCTTACTTTCACGCAATTCACAGGGTTCAACAAGCAATCGATATTTCACGATCAAGGGTGTAGTACTATTTGTAGTAGTGGTCCTTATATATCGTATTAACAATCGAAAGATGGTCGAAAGAAAAAATCTCTATTTGACAGGGCTTCTTCCTATACCTATGAATTCCATTGGACCTAGAAATGATACATTGGAAGAATCCTTTTGGTCTTCCAATATCAATAGGTTGATTGTTTCGCTCCTGTATCTTCCAAAAGGAAAAAAGATCTCTGAGAGCTCTTTCCTGGATCCGAAAGAGAGTACTTGGGTTCTCCCAATAACTAAAAAGTGTATCATGTCTGAATCTAACTGGGGTTCGCGGTGGTGGAGGAACTGGATCGGAAAAAAGAGGGATTCTAGTTGTAAGATATCTAATGAAACCGTCGCTGGAATTGAGATCTCATTCAAAGAAAAAGATATCAAATATCTGGAGTTTCTTTTTGTATATTATATGGATGATCCGATCCGCAAGGACCATGATTGGGAATTGTTTGATCGTCTTTCTCCGAGGAAGGGGCGAAACATAATCAACTTGAATTCGGGACAGCTATTCGAAATCTTAGTGAAAGACTGGATTTGTTATCTCATGTTTGCTTTTCGTGAAAAAATATCAATTGAAGTGGAGGGTTTCTTCAAACAACAAGGAGCTCGGTCAACTATTCAATCAAATGATATTGAGCATGTTTCCCATCTCTTCTCGAGAAAGAAGTGGGCTATTTCTTTGCAAAATTGTGCTCAATTTCATATGTGGCAATTCCGCCAAGATCTCTTCGTTAGTTGGGGGAATAATTCGCACGAATCGGATTTTTTTAGGAACATATCGAGAGAGAATTGGATTTGGTTAGACAATGTGTGGTTGGTAAACAAGGATCGGTTTTTTAGTAAGGCACGGAATATGTCGTCAAATATTCAATATGATTCCACAAGATCTAGTTTCGTTCAAGGAAGGAATTCTAGCCAATTGAAGGGATCTTCTGATCAATCCAGAGATCATTTCGATTCCATTAGTAATGAGGATTCGGAATATCACACATTGATCAATCAAAGAAAGATTCAACAACTAAAAGAAAGATCGATTCTTTGGGATCCTTCCTTTCTTCAAACGGAACGAACAGAGATAGAATCAGACCGATTCCCTAAATGCCTTTCTGGATATTCCTCAATGTCCCGGCTATTCACGGAAGGTGAGAAGGAGATGAATAATCATCTGCTTCCGGAAGAAATCGAAGAATTTCTTGGGAATCCTACAAGATCCATTCGTTCTTTTTTCTCTGACAGATCGTCAGAACTTCATCTGGGTTCGAATCCTACTGAGAGGTTCACTAGAGATCAGAAATTGTTGAAGAAAGAACAAGATGTTTCTTTTGTCCCTTCCAGGCGATCGGAAAATAAAGAAATAGTTAATATATTCAAGATAATCACGTATTTACAAAATACCGTCTCAATTCATCCTATTTCACCAGATCCGGGATGTGATATGGTTCTGAAGGATGAACTGGATATGGACAGTTCCAATAAGATTTCTTTCTTGAACAAAAATCCATTTTTTGATTTATTTCATCTATTCCATGATCGGAACGGGGGGGGATACACGTTACACCGCGATTTTGAATCAGAAGAGAGATTTCAAGAAATGGCGGATCTATTCACTCTATCAATAACCGAGCCGGATCTGGTGTATCATAAGGGATTTACCTTTTTTATTGATTCCTACGGATTGGATCAAAAACAATTCTTGAATGAGGTATTCAACTCCAGGGATGAATCGAAAAAGAAATCTTTATTGGTTCTACCTCCTATTTTTTATGAAGAGAATGAATCTTTTTATCGAAGGATCAGAAAAAAATGGGTCCGGATCTCCTGCGGGAATGATTTTGAAGATCCAAAACAAAAAATAGTGGTATTTGCTAGCAACAACATAATGGAGGCAGTCAATCAATATGGATTGATCCTAAATCTGATTCAAATCCAATATAGTACCTATGGGTACATAAGAAATGTATTGACTCAATTCTTTTTAATGAATAGATCCGATCGCAACTTCGAATATGGAATTCAAAGGGATCCAATAGGAAATGATACTCTGAATCATAGAACTATAATGAAATATACGATCAACCAACATTTATCGAATTTGAAACAGAGTCAGAAGAAATGGTTCGATCCTCTTATTTTTCTTTCTCGAACCGAGAGATCCATGAATTGGGATCCTAATGCATATAGATACAAATGGTCTAATGGGAGCAAGAATTTCCAGGAACATTTGGAACATTTCATTTCTGAGCAGAAGAACCGTTTTCTTTTTCAAGTAGTGTTCGATCGATTACGTATTAATCAATATTCGATTGATTGGTCTGAGGTTATCGACAAAAAAGATTTGTCTAAGTCACTTCGTTTCTTTTTGCCCAAGTTACTTCTTTTTTTGTCCAAGTTTCTTCTCTTTTTGTCTAACTCACTTCCTTTTTTCTTTGTGAGTTTCGGGAATATTCCCATTCATAGGTCCGAAATCCATATCTATGAATTGAAAGGTCCGAATGATCCACTCTGCAATCAGCTGTTAGAATCAATAGGTCTTCAAATCGTTCATTTGAAAAAATGGAAACCCTTCTTATTGGATGATCATGATACTTCCCAAAAATCGAAATTTTTGATTAATGGAGGAACAATATCACCATTTTTGTTCAATAAGATACCAAAGTGGATGATTGACTCATTCCATACTAGAAATAATCGCAGGAAATCTTTTGATAACACGGATTCCTTTTTCTCAATGATATCCCAGGATCAAGACAATTGGCTGAATCCCGTGAAACCATTTTATAGAAGTTCATTGATATCTTCTTTTTATAAAGCAAATCGACTTCGATTCTTGAATAATCTACATCACTTCTGGTTCTATTGTAACAAAAGATTCCCTTTTTATGTGGAAAAGGCCCGTATCAAGAATTATGATTTTACGTATGGACAATTCCTCAATATCTTGTTCATTCGCAACAAAATATTTTCTTTGTGCGGCGGTAAAAAAAAACATGCTTTTTTGGAGAGAGATACTATTTCACCAATCGAGTCACAGGTATCTAACATATTCATACCTAATGATTTTCCACAAAGTGGTAACGAAAGGTATAACTTGTACAAATCTTTCCATTTTCCAATTCGATCCGATCCATTCGTTCATAGAGCGATTTATTCGATCGCAGACATTTCTGGAACACCTCTAACAGAGGGACAAATAGTCAATTTTGAAAGAACTTATTGTCAACCTCTTTCGGATATGAATCTATCTGATTCAGAAGGGAAGAACTTGCATCAGTATCTCAATTTCAATTCAAACATGGGTTTGATTCACACTCCATGTTCTGAGAAATATTTACCATCCGAAAAGAGGAAAAAGCGGAGTCTTTGTCTAAAGAAATGTGTTGAAAAAGGGCAGATGTATAGAACCTTTCAACGAGATAGTGCTTTTTCGACTCTCTCAAAATGGAATCTATTCCAAACATATATGCCATGGTTCCTTACGTCGACAGGGTACAAATTTCTAAATTTGCTATTTTTAGATACCTTTTCGGACCTATTACCGATACTAAGTAGCAGTCAAAAATTTGTATCCATTTTTCATGATATTATGCATGGATCAGATATATCATGGCGAATTCTTCAGAAAAAATTGTGTCTTCCACAATGGAATCTGATAAGTGAGATTTCGAGTAAGTGTTTACATAATCTTCTTCTGTCCGAAGAAATGATTCATCGAAATAATGAGCCACCATTGATATCGACACATCTGAGATCGCCAAATGTTCGGGAGTTCCTCTATTCAATCCTTTTCCTTCTTCTTGTTGCTGGATATCTCGTTCGTACACATCTTCTCTTTGTTTCCCGAGCCTATAGTGAGTTACAGACAGAGTTCGAAAAGGTCAAATCTTTGATGATTCCATCATACATGATTGAGTTGCGAAAACTTCTGGATAGGTATCCTACATCTGAACTGAATTCTTTCTGGTTAAAGAATCTCTTTCTAGTTTCTCTGGAACAATTAGGAGATTTTCTAGAAGAAATGCGGGGTTCTGCTTCTGGCGGCAACATGCTATGGGGTGGTGGTCCCGCTTATGGGGTTAAATCAATACGTTCTAAGAAGAAATTTTTGAATATCAATCTCATCGATCTCATAAGTATCATACCAAATCCCATCAATCGAATCACTTTTTCGAGAAATACGAGACATCTAAGTCATACAAGTAAAGAGATTTATTCATTGATAAGAAAAAGAAAAAACGTGAACGGTGATTGGATTGATGATAAAATAGAATCCTTGGTCGCGAACAGTGATTCGATTGATGATAAAGAAAGAGAATTCTTGGTTCAGTTCTCCACCTTAACGACAGAAAAAAGGATTGATCAAATTCTATTGAGTCTGACTCATAGTGATCATTTATCAAAGAATGACTCTGGTTATCAAGTGATTGAAGAGCCGGGAGCAATTTATTTACGATACTTAGTTGACATTCATAAAAAGTATCTAATGAATTATGAGTTCAATACACCCTGTTTAGCAGAAAGACGGATATTCCTTGCTTATTATCAGACAACCACTTATTCACAAACCTCGTGTGGGGTGAATAGTTTTCATTTCCCATCTCATGGAAAACCCTTTTCGCTCCGCTTAGCCCTATCCCCCTCTAGGGGTATTTTAGTGATAGGTTCTATAGGGACTGGACGATCCTATTTGGTCAAATACCTAGCGACAAACTCCTATCTTCCTTTCATTACAGTATTTCTGAACAAGTTCCTGGATAACAAGCCTAAGGGTTTTCTTATTGATGATAGTGACGATATTGATGATAGTGACGATATTGATGTGAGTGACGATATCGACCGTGACTTTGATACGGAGCTGGAGTTTCTAACTAGGATGAATGCGCTAACTATGGATATGATGCCGGAAATAGACCGATTTTATATCACCCTTCAATTCGAATTAGCAAAAGCAATGTCTCCTTGCATAATATGGATTCCAAACATTCATGATCTGGATGTGAATGAGTCGAATTACTTATCCCTCGGTCTATTAGTGAACTATCTCTCCAGGGATTGTGAAAGATGTTCCACTAGAAATATTCTTGTTATTGCTTCGACTCATATTCCCAAAAAAGTGGATCCCGCTCTAATAGCTCCGAATAAATTAAATACATGCATTAAGATACGAAGGCTTCTTATTCCACAACAACGAAAGCACTTTTTTACTCTTTCATATACTAGGGGATTTCACTTGAAAAAGAAAATGTTCCATACTAATGGATTCGGGTCCATAACCATGGGTTCCAATGTACGAGATCTTGTAGCACTTACCAATGAGGCCCTATCAATTAGTATTACACAGAAGAAATCAATTATAGACACTAATATAATTAGATCTGCTCTTCATAGACAAACTTGGGATTTGCGATCCCAGGTAAGATCGGTTCAGGATCATGGGATCCTTTTCTATCAGATAGGACGGGCTGTTGCACAAAATGTATTTCTAAGTAATTGCCCCATAGATCCTATATCTATCTATATGAAGAAGAAATCTTGTAACGAAGGGGATTCTTATTTGTACAAATGGTACTTCGAACTTGGAACGAGCATGAAGAAATTAACGATACTTCTTTATCTTTTGAGTTGTTCTGCCGGATCGGTTGCTCAAGACCTTTGGTCTCTACCCGGACCCGATGAAAAAAATGGGATCACTTATTATGGACTTGTTGAGAATGATTCTGATCTAGTTCATGGCCTATTAGAAGTAGAAAGCGCTCTGGTGGGATCCTCACGGACAGAAAAAGATTGCAGTCAGTTTGATAATGATCGAGTGACATTGCTTCTTCGGCCCGAACCAAGAAGTCCCTTAGATATGATGCAAAATGGATCTTGTTCTATCCTTGATCAGAGATTTCTCTATGAAAAATACGAATCGGAGTTTGAAGAAGGGGAAGGAGAAGAAGTCCTCGACCCGCAACAGATAGAGGAGGATTTATTCAATCACATAGTTTGGGCTCCTAGAATATGGCGCCCTTGGGGCTTTCTATTTGATTGTATCGAAAGGCCCAATGAATTGGGATTTCCCTATTGGGCCAGGTCATTTCGGGGCAAGCGGATCATTTATAATGAAGAGGATGAGCTTCAAGAGAATGATTCGGAGTTCTTGCAGAGTGGAGCCATGCAGTACCAGATACGAGATAGATCCTCCAAAGAACAAGGCTTTTTTCGAATAAGCCAATTCATTTGGGACCCTGCAGATCCACTCTTTTTCCTATTCAAAGATCAGCCCCTTGTCTCTGTGTTTTCACATCGAGAATTCTTTGCAGATGAAGAGATGTCAAAGGGGCTTCTTACTTCCCAAACGGATCCTCCTACATCTATATATAAATGCTGGTTTATCAAGAATACGCAAGAAAAGCACTTCGAATTGTTGATTCATCGCCAGAGATGGCTTAGAACCAATAGTTCATTATCTAATGGATTTTTCCGTTCGAATACTCTATCCGAGAGTTATCAGTATTTATCAAATCTGTTCCTATCTAACGGAACGCTATTGGATCAAATGACAAAGGCATTGTTGAGAAAAAGATGGCTTTTCCCGGATGAAATGAAAATTGGATTCATGTAA

>lcl|NC_016736.1_cds_YP_005090179.1_23 [gene=ycf3] [locus_tag=RCOM_ORF00038] [db_xref=GeneID:11542312] [protein=photosystem I assembly protein Ycf3] [protein_id=YP_005090179.1] [location=complement(join(46923..47075,47735..47962,48696..48824))] [gbkey=CDS]
ATGCCTAGATCCCGGATAACTGGAAATTTTATTGATAAGACCTTTTCAATTGTAGCCAATATCTTATTACGAATAATTCCGACAACTTCGGGAGAAAAAGAGGCATTTACTTATTACAGAGATGGTGTGATGTCTGCTCAATCCGAAGGAAATTATGCAGAAGCTTTACAGAATTATTATGAAGCTATGCGGCTAGAAATTGATCCCTATGATCGAAGTTATATACTCTATAATATAGGCCTTATTCACACAAGTAATGGAGAACATACAAAAGCTTTGGAATATTATTTTCGGGCACTAGAACGAAACCCCTTCTTACCACAAGCTTTAAATAATATGGCCGTGATCTGTCATTACCGAGGAGAACAGGCCATTCGGCAGGGAGATTCTGAAATTGCGGAGGCTTGGTTCGATCAAGCCGCAGAGTATTGGAAACAAGCTATAGCGCTTACTCCCGGAAATTATATTGAAGCGCAGAATTGGTTGAAGATCACAAGGCGTTTTGAATAA

>lcl|NC_016736.1_cds_YP_005090215.1_59 [gene=rpl16] [locus_tag=RCOM_ORF00081] [db_xref=GeneID:11542357] [protein=ribosomal protein L16] [protein_id=YP_005090215.1] [location=complement(86916..87275)] [gbkey=CDS]
ATGAAAGGAATAGCTTTTCGAGGTAATCGTATTTGTTTCGGCAAATATGCTCTTCAGGCACTTGAACCCGCTTGGATTACATCTAGACAAATAGAAGCGGGACGACGGGCAATGACACGAAATGCACGCCGCGGTGGAAAAATATGGGTACGCATATTTCCCGACAAACCAGTTACTTTAAGACCTACGGAAACACGTATGGGTTCGGGGAAAGGATCTCCTGAATATTGGGTGGCTGTCGTTAAACCAGGTAGAATACTTTATGAAATGAGTGGAGTAGCAGAAAATATAGCAAGAAGGGCTATTTCAATAGCAGCATCAAAAATGCCTATACGAACTCAATTCATTATTTCGGGATAG

>lcl|NC_016736.1_cds_YP_005090169.1_13 [gene=rpoC1] [locus_tag=RCOM_ORF00020] [db_xref=GeneID:11542294] [protein=RNA polymerase beta subunit] [protein_id=YP_005090169.1] [location=complement(join(22374..23903,24750..25184))] [gbkey=CDS]
ATGATTGATCGGTATAAACATCAACAACTCCGAATTGGATCAGTTTCGCCTCAACAAATAAGTGCTTGGGCCAAAAAAATCCTACCTAATGGGGAGATTGTTGGAGAAGTGACAAAACCCTATACTTTTCATTACAAAACCAATAAACCTGAAAAAGGTGGATTGTTTTGTGAAAGAATTTTTGGGCCTATAAAAAGTGGAATTTGTGCTTGTGGAAATTATCGAGTAATCAGAAATGAAAAAGAAGACCAAAAATTTTGTGAACAATGCGGAGTCGAATTTGTTGATTCTCGGATACGAAGATATCAAATGGGCTACATCAAACTGGCATGCCCAGTAACTCATGTGTGGTATTTGAAACGTCTTCCTAGTTATATCGCAAATCTTTTAGATAAACCTCTTAAAGAATTAGAAGGCCTAGTATATTGCGATGTGAAATACAGTATTCCACTTTTTTTTACTGCCCAAGGCTTCGATACATTTCGAAATCGAGAAATTTCTACAGGAGCTGGTGCTATCCGAGAACAATTAGCCGATCTAGATTTGCGAATTATTATAGATTATTCATCGGTAGAATGGAAAGAATTAGGGGAAGAAGGGCCTACGGGGAATGAATGGGAAGATCGAAAAGTTGGAAGAAGAAAGGATTTTTTGGTTCGACGCGTGGAATTAGCTAAGCATTTTATTCGAACAAATATAGAACCAGAATGGATGGTTTTATGTCTATTACCAGTTCTTCCTCCCGAGTTGAGACCGATCATTCAGATAGATGGGGGTAAACTAATGAGTTCAGATATTAATGAACTCTATAGAAGAGTTATCTATCGGAACAATACTCTTATTGATCTATTAACAACAAGTAGATCTACGCCAGGAGAATTAGTAATGTGTCAGGAAAAATTAGTACAAGAAGCCGTGGATACACTTCTTGATAATGGAATCCGCGGACAACCAATGAGAGACGGTCATAATAAGGTTTACAAGTCGTTTTCGGATGTAATTGAAGGCAAAGAAGGAAGATTTCGTGAGACTATGCTTGGAAAACGGGTTGATTATTCGGGGCGTTCTGTCATTGTCGTAGGCCCCTCACTTTCATTACATCGATGTGGATTGCCTCGCGAAATAGCAATAGAACTTTTCCAGATATTTGTAATTCGTGGTCTAATTAGACAACATCTTGCTTCGAACATAGGAGTTGCTAAGAGTAAAATTCGGGAAAAAGAGCCAATTGTATGGGAAATACTTCAGGAAGTTATGCAGGGGCATCCGGTATTACTGAATAGAGCGCCGACTTTGCATAGATTAGGCATACAGGCATTCCAACCCATTTTAGTGGAAGGCCGCGCTATTTGTTTACATCCATTAGTTTGTAAGGGATTCAATGCAGACTTTGATGGGGATCAAATGGCTGTTCATGTACCTTTATCGTTGGAGGCTCAAGCGGAGGCTCGTTTACTTATGTTTTCTCATATGAATCTCTTATCTCCAGCTATTGGAGATCCCATTTCCGTACCAACTCAAGATATGCTTATTGGGCTCTATGTATTAACAAGCAGGAATCGCCGAGGTATTTGTGCAAATAGGTATAATCCATGTAATCACAGAAATTATCAAAATGAAAGAATTTACGATAATAACAATCAATATACGAAAGAATCCTTTTTTTCTAATTCCTATGATGCAATTGGTGCTTATCGGCAGAAAAGAATCAATTTAGATAGTCCTTTGTGGCTCCGTTGGCAACTAGATCAACGCGCTATTGCTTCAAGAGAAGCTCCCGTCGAAGTTCACTATGAATCTTTGGGTACCTATCATGAGATTTATGAACACTATCTAATAGTAAGAAATATAAAAAAAGAAATTCTTTGTATATACATTCGAACTACTGTTGGTCATATTTCTCTTTATCGAGAAATCGAAGAAGCTATACAAGGGTTTTGCCAAGCCGGCTCAGATGGTATCTAA

>lcl|NC_016736.1_cds_YP_005090214.1_58 [gene=rpl14] [locus_tag=RCOM_ORF00080] [db_xref=GeneID:11542356] [protein=ribosomal protein L14] [protein_id=YP_005090214.1] [location=complement(86413..86781)] [gbkey=CDS]
ATGATCCAATCTCAGACCCATTTGAATGTAGCAGATAACAGCGGAGCCCGAGAATTAATGTGTATTCGAATCATAGGGACTAGTAATCGCCGATATGCTCATATTGGTGACGTTATTGTTGCTGTGATCAAGGAAGCGGCACCAAATTCACCTCTAGAAAGATCAGAAGTAATCAGAGCTGTAATTGTACGTACTTGTAAAGAACTCAAACGTGATAACGGTATGATAATACGATATGATGACAATGCTGCAGTTGTCATTGATCAAGAAGGAAATCCAAAGGGAACTCGAATTTTTGGTGCAATCGCCCGAGAATTGAGACAGTTAAATTTTACTAAAATAGTTTCATTAGCGCCTGAAGTATTATAA

>lcl|NC_016736.1_cds_YP_005090216.1_60 [gene=rps3] [locus_tag=RCOM_ORF00082] [db_xref=GeneID:11542358] [protein=ribosomal protein S3] [protein_id=YP_005090216.1] [location=complement(88530..89186)] [gbkey=CDS]
ATGGGACAAAAAATAAATCCACTAGGTTTCCGACTTGGTACAACCCAAAGTCATTATTCTCTTTGGTTTGCTCAACCAAAAAATTACTCTGAGGGTCTACAAGAAGATCAAAAAATAAGAAACTGTATCAAGAATTATGTAAAAAACAATACAAAAATATCTTCTGGTGTTGAGGGAATTGCATGTATAGAGATTCAAAAAAGAATTGATGTGATTCAAGTAATAATATATATGGGATTCACAAAATTATTAATAGAAAGTAAACCTAAACGAATCGAGAAATTACAGATAAATGTACAAAAAGAACTAAATTGTGTGAACCGAAAACTCAATATTGCTATTACAAGAATTTCAAACCCTTATGGGCACCCTAATATTCTTGCAGAATTTATAGCCGGACAATTAAAGAATAGAGTTTCATTTCGCAAAGCAATGAAAAAAGCTATTGAATTAACTGAACAGGCGGATACAAAAGGAATTCAAGTACAAATTGCGGGGCGTCTTGACGGAAAAGAAATTGCACGCGTCGAGTGGATTAGAGAGGGTAGGGTTCCTCTACAAACCATTCGGGCTAAAATTGATTATTGTTCATATACAGTTAGAACCATTTATGGGGTATTAGGCATAAAAATTTGGACATTTCTAGACAAAAAATAA

>lcl|NC_016736.1_cds_YP_005090221.1_65 [gene=ycf2] [locus_tag=RCOM_ORF00088] [db_xref=GeneID:11542364] [protein=Ycf2] [protein_id=YP_005090221.1] [location=92627..99487] [gbkey=CDS]
ATGAAAGGACATCAATTCAAATCCTGGATTTTCGAATTGAGAGAGATATTGAGAGAGATCAAGAATTCTCACTATTTCTTAGATTCATGGACCCAATTCAATTCCGTGGGATCTTTCATTCACATTTTTTTCCATCAAGAACGTTTTATAAAACTCTTGGACTCCCGAATTTGGAGTATCTTACTTTCACGCAATTCACAGGGTTCAACAAGCAATCGATATTTCACGATCAAGGGTGTAGTACTATTTGTAGTAGTGGTCCTTATATATCGTATTAACAATCGAAAGATGGTCGAAAGAAAAAATCTCTATTTGACAGGGCTTCTTCCTATACCTATGAATTCCATTGGACCTAGAAATGATACATTGGAAGAATCCTTTTGGTCTTCCAATATCAATAGGTTGATTGTTTCGCTCCTGTATCTTCCAAAAGGAAAAAAGATCTCTGAGAGCTCTTTCCTGGATCCGAAAGAGAGTACTTGGGTTCTCCCAATAACTAAAAAGTGTATCATGTCTGAATCTAACTGGGGTTCGCGGTGGTGGAGGAACTGGATCGGAAAAAAGAGGGATTCTAGTTGTAAGATATCTAATGAAACCGTCGCTGGAATTGAGATCTCATTCAAAGAAAAAGATATCAAATATCTGGAGTTTCTTTTTGTATATTATATGGATGATCCGATCCGCAAGGACCATGATTGGGAATTGTTTGATCGTCTTTCTCCGAGGAAGGGGCGAAACATAATCAACTTGAATTCGGGACAGCTATTCGAAATCTTAGTGAAAGACTGGATTTGTTATCTCATGTTTGCTTTTCGTGAAAAAATATCAATTGAAGTGGAGGGTTTCTTCAAACAACAAGGAGCTCGGTCAACTATTCAATCAAATGATATTGAGCATGTTTCCCATCTCTTCTCGAGAAAGAAGTGGGCTATTTCTTTGCAAAATTGTGCTCAATTTCATATGTGGCAATTCCGCCAAGATCTCTTCGTTAGTTGGGGGAATAATTCGCACGAATCGGATTTTTTTAGGAACATATCGAGAGAGAATTGGATTTGGTTAGACAATGTGTGGTTGGTAAACAAGGATCGGTTTTTTAGTAAGGCACGGAATATGTCGTCAAATATTCAATATGATTCCACAAGATCTAGTTTCGTTCAAGGAAGGAATTCTAGCCAATTGAAGGGATCTTCTGATCAATCCAGAGATCATTTCGATTCCATTAGTAATGAGGATTCGGAATATCACACATTGATCAATCAAAGAAAGATTCAACAACTAAAAGAAAGATCGATTCTTTGGGATCCTTCCTTTCTTCAAACGGAACGAACAGAGATAGAATCAGACCGATTCCCTAAATGCCTTTCTGGATATTCCTCAATGTCCCGGCTATTCACGGAAGGTGAGAAGGAGATGAATAATCATCTGCTTCCGGAAGAAATCGAAGAATTTCTTGGGAATCCTACAAGATCCATTCGTTCTTTTTTCTCTGACAGATCGTCAGAACTTCATCTGGGTTCGAATCCTACTGAGAGGTTCACTAGAGATCAGAAATTGTTGAAGAAAGAACAAGATGTTTCTTTTGTCCCTTCCAGGCGATCGGAAAATAAAGAAATAGTTAATATATTCAAGATAATCACGTATTTACAAAATACCGTCTCAATTCATCCTATTTCACCAGATCCGGGATGTGATATGGTTCTGAAGGATGAACTGGATATGGACAGTTCCAATAAGATTTCTTTCTTGAACAAAAATCCATTTTTTGATTTATTTCATCTATTCCATGATCGGAACGGGGGGGGATACACGTTACACCGCGATTTTGAATCAGAAGAGAGATTTCAAGAAATGGCGGATCTATTCACTCTATCAATAACCGAGCCGGATCTGGTGTATCATAAGGGATTTACCTTTTTTATTGATTCCTACGGATTGGATCAAAAACAATTCTTGAATGAGGTATTCAACTCCAGGGATGAATCGAAAAAGAAATCTTTATTGGTTCTACCTCCTATTTTTTATGAAGAGAATGAATCTTTTTATCGAAGGATCAGAAAAAAATGGGTCCGGATCTCCTGCGGGAATGATTTTGAAGATCCAAAACAAAAAATAGTGGTATTTGCTAGCAACAACATAATGGAGGCAGTCAATCAATATGGATTGATCCTAAATCTGATTCAAATCCAATATAGTACCTATGGGTACATAAGAAATGTATTGACTCAATTCTTTTTAATGAATAGATCCGATCGCAACTTCGAATATGGAATTCAAAGGGATCCAATAGGAAATGATACTCTGAATCATAGAACTATAATGAAATATACGATCAACCAACATTTATCGAATTTGAAACAGAGTCAGAAGAAATGGTTCGATCCTCTTATTTTTCTTTCTCGAACCGAGAGATCCATGAATTGGGATCCTAATGCATATAGATACAAATGGTCTAATGGGAGCAAGAATTTCCAGGAACATTTGGAACATTTCATTTCTGAGCAGAAGAACCGTTTTCTTTTTCAAGTAGTGTTCGATCGATTACGTATTAATCAATATTCGATTGATTGGTCTGAGGTTATCGACAAAAAAGATTTGTCTAAGTCACTTCGTTTCTTTTTGCCCAAGTTACTTCTTTTTTTGTCCAAGTTTCTTCTCTTTTTGTCTAACTCACTTCCTTTTTTCTTTGTGAGTTTCGGGAATATTCCCATTCATAGGTCCGAAATCCATATCTATGAATTGAAAGGTCCGAATGATCCACTCTGCAATCAGCTGTTAGAATCAATAGGTCTTCAAATCGTTCATTTGAAAAAATGGAAACCCTTCTTATTGGATGATCATGATACTTCCCAAAAATCGAAATTTTTGATTAATGGAGGAACAATATCACCATTTTTGTTCAATAAGATACCAAAGTGGATGATTGACTCATTCCATACTAGAAATAATCGCAGGAAATCTTTTGATAACACGGATTCCTTTTTCTCAATGATATCCCAGGATCAAGACAATTGGCTGAATCCCGTGAAACCATTTTATAGAAGTTCATTGATATCTTCTTTTTATAAAGCAAATCGACTTCGATTCTTGAATAATCTACATCACTTCTGGTTCTATTGTAACAAAAGATTCCCTTTTTATGTGGAAAAGGCCCGTATCAAGAATTATGATTTTACGTATGGACAATTCCTCAATATCTTGTTCATTCGCAACAAAATATTTTCTTTGTGCGGCGGTAAAAAAAAACATGCTTTTTTGGAGAGAGATACTATTTCACCAATCGAGTCACAGGTATCTAACATATTCATACCTAATGATTTTCCACAAAGTGGTAACGAAAGGTATAACTTGTACAAATCTTTCCATTTTCCAATTCGATCCGATCCATTCGTTCATAGAGCGATTTATTCGATCGCAGACATTTCTGGAACACCTCTAACAGAGGGACAAATAGTCAATTTTGAAAGAACTTATTGTCAACCTCTTTCGGATATGAATCTATCTGATTCAGAAGGGAAGAACTTGCATCAGTATCTCAATTTCAATTCAAACATGGGTTTGATTCACACTCCATGTTCTGAGAAATATTTACCATCCGAAAAGAGGAAAAAGCGGAGTCTTTGTCTAAAGAAATGTGTTGAAAAAGGGCAGATGTATAGAACCTTTCAACGAGATAGTGCTTTTTCGACTCTCTCAAAATGGAATCTATTCCAAACATATATGCCATGGTTCCTTACGTCGACAGGGTACAAATTTCTAAATTTGCTATTTTTAGATACCTTTTCGGACCTATTACCGATACTAAGTAGCAGTCAAAAATTTGTATCCATTTTTCATGATATTATGCATGGATCAGATATATCATGGCGAATTCTTCAGAAAAAATTGTGTCTTCCACAATGGAATCTGATAAGTGAGATTTCGAGTAAGTGTTTACATAATCTTCTTCTGTCCGAAGAAATGATTCATCGAAATAATGAGCCACCATTGATATCGACACATCTGAGATCGCCAAATGTTCGGGAGTTCCTCTATTCAATCCTTTTCCTTCTTCTTGTTGCTGGATATCTCGTTCGTACACATCTTCTCTTTGTTTCCCGAGCCTATAGTGAGTTACAGACAGAGTTCGAAAAGGTCAAATCTTTGATGATTCCATCATACATGATTGAGTTGCGAAAACTTCTGGATAGGTATCCTACATCTGAACTGAATTCTTTCTGGTTAAAGAATCTCTTTCTAGTTTCTCTGGAACAATTAGGAGATTTTCTAGAAGAAATGCGGGGTTCTGCTTCTGGCGGCAACATGCTATGGGGTGGTGGTCCCGCTTATGGGGTTAAATCAATACGTTCTAAGAAGAAATTTTTGAATATCAATCTCATCGATCTCATAAGTATCATACCAAATCCCATCAATCGAATCACTTTTTCGAGAAATACGAGACATCTAAGTCATACAAGTAAAGAGATTTATTCATTGATAAGAAAAAGAAAAAACGTGAACGGTGATTGGATTGATGATAAAATAGAATCCTTGGTCGCGAACAGTGATTCGATTGATGATAAAGAAAGAGAATTCTTGGTTCAGTTCTCCACCTTAACGACAGAAAAAAGGATTGATCAAATTCTATTGAGTCTGACTCATAGTGATCATTTATCAAAGAATGACTCTGGTTATCAAGTGATTGAAGAGCCGGGAGCAATTTATTTACGATACTTAGTTGACATTCATAAAAAGTATCTAATGAATTATGAGTTCAATACACCCTGTTTAGCAGAAAGACGGATATTCCTTGCTTATTATCAGACAACCACTTATTCACAAACCTCGTGTGGGGTGAATAGTTTTCATTTCCCATCTCATGGAAAACCCTTTTCGCTCCGCTTAGCCCTATCCCCCTCTAGGGGTATTTTAGTGATAGGTTCTATAGGGACTGGACGATCCTATTTGGTCAAATACCTAGCGACAAACTCCTATCTTCCTTTCATTACAGTATTTCTGAACAAGTTCCTGGATAACAAGCCTAAGGGTTTTCTTATTGATGATAGTGACGATATTGATGATAGTGACGATATTGATGTGAGTGACGATATCGACCGTGACTTTGATACGGAGCTGGAGTTTCTAACTAGGATGAATGCGCTAACTATGGATATGATGCCGGAAATAGACCGATTTTATATCACCCTTCAATTCGAATTAGCAAAAGCAATGTCTCCTTGCATAATATGGATTCCAAACATTCATGATCTGGATGTGAATGAGTCGAATTACTTATCCCTCGGTCTATTAGTGAACTATCTCTCCAGGGATTGTGAAAGATGTTCCACTAGAAATATTCTTGTTATTGCTTCGACTCATATTCCCAAAAAAGTGGATCCCGCTCTAATAGCTCCGAATAAATTAAATACATGCATTAAGATACGAAGGCTTCTTATTCCACAACAACGAAAGCACTTTTTTACTCTTTCATATACTAGGGGATTTCACTTGAAAAAGAAAATGTTCCATACTAATGGATTCGGGTCCATAACCATGGGTTCCAATGTACGAGATCTTGTAGCACTTACCAATGAGGCCCTATCAATTAGTATTACACAGAAGAAATCAATTATAGACACTAATATAATTAGATCTGCTCTTCATAGACAAACTTGGGATTTGCGATCCCAGGTAAGATCGGTTCAGGATCATGGGATCCTTTTCTATCAGATAGGACGGGCTGTTGCACAAAATGTATTTCTAAGTAATTGCCCCATAGATCCTATATCTATCTATATGAAGAAGAAATCTTGTAACGAAGGGGATTCTTATTTGTACAAATGGTACTTCGAACTTGGAACGAGCATGAAGAAATTAACGATACTTCTTTATCTTTTGAGTTGTTCTGCCGGATCGGTTGCTCAAGACCTTTGGTCTCTACCCGGACCCGATGAAAAAAATGGGATCACTTATTATGGACTTGTTGAGAATGATTCTGATCTAGTTCATGGCCTATTAGAAGTAGAAAGCGCTCTGGTGGGATCCTCACGGACAGAAAAAGATTGCAGTCAGTTTGATAATGATCGAGTGACATTGCTTCTTCGGCCCGAACCAAGAAGTCCCTTAGATATGATGCAAAATGGATCTTGTTCTATCCTTGATCAGAGATTTCTCTATGAAAAATACGAATCGGAGTTTGAAGAAGGGGAAGGAGAAGAAGTCCTCGACCCGCAACAGATAGAGGAGGATTTATTCAATCACATAGTTTGGGCTCCTAGAATATGGCGCCCTTGGGGCTTTCTATTTGATTGTATCGAAAGGCCCAATGAATTGGGATTTCCCTATTGGGCCAGGTCATTTCGGGGCAAGCGGATCATTTATAATGAAGAGGATGAGCTTCAAGAGAATGATTCGGAGTTCTTGCAGAGTGGAGCCATGCAGTACCAGATACGAGATAGATCCTCCAAAGAACAAGGCTTTTTTCGAATAAGCCAATTCATTTGGGACCCTGCAGATCCACTCTTTTTCCTATTCAAAGATCAGCCCCTTGTCTCTGTGTTTTCACATCGAGAATTCTTTGCAGATGAAGAGATGTCAAAGGGGCTTCTTACTTCCCAAACGGATCCTCCTACATCTATATATAAATGCTGGTTTATCAAGAATACGCAAGAAAAGCACTTCGAATTGTTGATTCATCGCCAGAGATGGCTTAGAACCAATAGTTCATTATCTAATGGATTTTTCCGTTCGAATACTCTATCCGAGAGTTATCAGTATTTATCAAATCTGTTCCTATCTAACGGAACGCTATTGGATCAAATGACAAAGGCATTGTTGAGAAAAAGATGGCTTTTCCCGGATGAAATGAAAATTGGATTCATGTAA

>lcl|NC_016736.1_cds_YP_005090189.1_33 [gene=ycf4] [locus_tag=RCOM_ORF00054] [db_xref=GeneID:11542328] [protein=photosystem I assembly protein Ycf4] [protein_id=YP_005090189.1] [location=64401..64955] [gbkey=CDS]
ATGAGTTGGCGATCAGAACGTATATGGATAGAACTTATAGCGGGGTCTCGCAAAACAAGTAATTTCTGCTGGGCCTTTATACTTTTTTTAGGTTCATTGGGATTTTTATTGGTTGGAATTTCCAGTTATCTTGGCAGAAATTGGATATCTTTATTTCCGTCTCAGCAAATAATTTTTTTTCCACAAGGGATCGTGATGTCTTTCTATGGGATCGCCGGTCTATTTATTAGTTCTTATTTGTGGTGCACAATTTTGTGGAATGTAGGTAGTGGTTATGATCGATTCGATAGAAAAGAAGGAATAGTGTGTATTTTTCGCTGGGGATTCCCTGGAAAAAATCGTCGCATCTTCCTCCGATTCCTTATGAAGGATATTCAGTCTATTAGAATAGAAGTGAAAGAGGGTATTTTTGCTCGGCGTGTCCTTTATATGGAAACCAGAGGCCGAGGGTCCATTCCTTTGACTCGTACTGATGAGAATTTGACTCCACGAGAGATTGAGCAAAAGGTAGCGGAATTGGCCTATTTTTTGCGTGTACCAATTGAAGTATTTTGA

>lcl|NC_016736.1_cds_YP_005090184.1_28 [gene=atpE] [locus_tag=RCOM_ORF00049] [db_xref=GeneID:11542323] [protein=ATP synthase CF1 epsilon subunit] [protein_id=YP_005090184.1] [location=complement(56256..56657)] [gbkey=CDS]
ATGACCTTAAATCTTTGTGTACTGACCCCAAATCGAATTGTTTGGGATTCAGAAGTGAAAGAAATCATTTTATCTACTAATAGTGGACAAATTGGCGTATTATCAAATCATGCGCCTATTGCCACAGCAGTCGATATCGGTATTTTGAGAATACGCCTTAATGACCAATGGTTAACGATGGCTCTGATGGGCGGTTTTGCTAGAATAGGCAATAATGAGATTACTATTTTAGTAAACGATGCAGAGAAGGGTAGTAACATTGATCCACAAGAAGCTCAGAAAAATCTTGAAATAGCAGAAGCTAACTTGAGAAAGGCGGAAGGCAGGAGACAAATAATTGAGGCAAATCTAGCTCTCAGACGAGCTAGGGCACGCGTAGAGGCTATCAATGTGATTTCGTAA

>lcl|NC_016736.1_cds_YP_005090209.1_53 [gene=petD] [locus_tag=RCOM_ORF00075] [db_xref=GeneID:11542350] [protein=cytochrome b6/f complex subunit IV] [protein_id=YP_005090209.1] [location=82868..83392] [gbkey=CDS]
ATGTCCGGTTCCTTCGGAGGATGGATTTATAAGAATTCACCTATCCCAATAACAAAAAAACCTGACTTGAATGATCCTGTATTAAGGGCTAAATTGGCTAAGGGAATGGGTCATAATTATTACGGAGAACCCGCATGGCCCAATGATCTTTTATATATTTTTCCGGTAGTAATTCTAGGTACTATTGCATGTAATGTAGGATTAGCGGTTCTAGAACCATCAATGATTGGTGAACCTGCGGATCCATTTGCAACTCCTTTGGAAATATTGCCTGAATGGTATTTCTTTCCTGTATTTCAAATACTTCGTACAGTACCCAATAAGTTATTGGGTGTTCTTTTAATGGTTTCAGTACCTGCAGGATTATTAACAGTACCCTTTTTGGAGAATGTTAATAAATTCCAAAATCCATTTCGTCGTCCAGTTGCGACAACCGTCTTTTTGGTTGGTACTGCAGTAGCCCTTTGGTTAGGTATTGGAGCAACATTGCCTATTGATAAATCCCTAACTTTAGGTCTTTTTTAA

>lcl|NC_016736.1_cds_YP_005090176.1_20 [gene=rps14] [locus_tag=RCOM_ORF00035] [db_xref=GeneID:11542309] [protein=ribosomal protein S14] [protein_id=YP_005090176.1] [location=complement(41268..41570)] [gbkey=CDS]
ATGGCAAGAAAAAGTTTGATTCAGCGGGAGAAGAAGAGGCAAAAATTGGAACAAAAATATCATTTGATTCGTCGATCCTCAAAAAAAGAAATAAGCAAAGTTCCGTCGTTGAGTGATAAATGGGAAATTCATGGAAAGTTACAATCCCCACCGCGAAATAGTGCACCGACACGTCTTCATCGACGCTGTTTTTCGACTGGAAGACCGAGAGCTAACTATCGAGACTTTGGACTATCCGGACACATACTTCGTGAAATGGTTCATGCATGTTTGTTACCGGGGGCAACAAGATCGAGTTGGTAA

>lcl|NC_016736.1_cds_YP_005090236.1_80 [gene=ycf1] [locus_tag=RCOM_ORF00114] [db_xref=GeneID:11542390] [protein=hypothetical chloroplast RF1] [protein_id=YP_005090236.1] [location=complement(131574..136991)] [gbkey=CDS]
ATGATTTTTAAATCTTTTATACTAGGTAATCTAGTATCCTTATGCATGAAGATAATCAATTTGGTCGTTGTGGTCGGACTCTATTATGGATTTCTGACCACATTCTCCATGGGGCCCTCTTATCTCTTCCTTCTCCGAGCTCGGGTTATAGAAGAAGGAGAAGAAGGAACTGAGAAGAAGGTATCAGCAACAACAGGTTTTATTACGGGACAGCTCATGATGTTCATATCGATCTATTATGCGCCTCTGCATCTAGCATTGGGTAGACCTCATACAATAACTGTCCTAGCTCTACCCTATCTTTTGTTTCATTTCTTCTGGAACAATCACAAACACTTTTTTGATTATGGAGCTACTACCAGAAATTCAATGCGTAATCTTAGCATTCAATTTGTATTCCTGAATAATCTCATTTTTCAATTATTCAACCATTTCATTTTACCAAGTTCAATGTTAGTCAGATTAGTCAACATTTATATGTTTCGATGCAACAACAAGATGTTATTTGTAACAAGTAGTTTTGTTGGTTGGTTAATTGGTCACATTTTATTCATGAAATGGGTTGGATTGATATTAGTCTGGATACAACAAAATAATTCTATTAGATCTAATGTACTTTTTCGATCTAATAAGTACCTTGTGTCAGAATTGAGAAATTCTATGGCTCGAATCTTTAGTATTCTCTTATTTATTACCTGCGTCTACTCTTTAGGCAGAATACCGTCACCCATTTTTACTAAGAAACTGAAAGAAACCTCAGAAACGGAAGAAAGGGAGGAAAGTGAGGAAGAAACAGATGTAGAAATAGAAACAACTTCCGAAACGAAGGGGACTAAACAGGGATCCACCGAAGAAGATCCTTCTTCTTCCCTTTTTTCGGAAGAAAAGGAGGATCGGGACAAAATCGACGAAACGGAAGAGATCCAAGTGAATGGAAAGGAAAAAACAAAGGATGAATTCCATTTTCACTTTAACGAGACATGCTATAAAAATAGACCACTTTATGAAACTTTTTATCTGGATGGGAATCAAGAAAATTCGAAGTTAGAAATATTGATAGAAAAAAAAAAGAAAAATCTTTTCTGGTTTGAAAAACCTCTTGTAACTATTCTTTTTGACTCGAAACGTTGGAATCGTCCATTACGATATATAAAAAATGATCAGTTTGAGAATGCTGTAAGAAATGAAATGTCACAATATTTTTTTTATACATGTCGAAGTGATGGAAAAGAAAGAATATCTTTCACGTATCCGCCCAGCTTGTCAACTTTTTTTGAAATGGTACAGAAAAAGATTTTTCTGTTCACAACAACAACAGAGAAACTCTCCTCTGATGAATTGTATAATCATTGGAATTATAAGAATGAACAAAAAAAGAAAAATCTAAGTAATGAATTTATAAACAGAGTCCAGGCTCTAGATAAGGGATATCTTGTTCTGAATACACTCGAAAAAAGGACTAGATTATGTAATGATAAAACGAAAAAAGAGTACTTACCAAAAATATATGATCCCTTATTGAGTGGGTCCTACCGTGGAAGAACAAAATTTTTTTTTTCACCCTCAACACTAAATATAAATAAAACTTCTATAAAAAATTCCACGGAGATGGTTTGGATAAATAAAATTCATCTTATTCTTCTTATTAGTAATTATATAGAATTTGAACCAAAAACAGATAGAAAATCATTTTCAACAGAAATTGCTTATTTCTTAAACTTAATTAATGAATTTGCCGGAAAATCGACATCAAGTTTCAATTTTAAGGGACTCCCTTTATTTCCAGATTACAAAGAAGAAAAAATGGATTTAGAACTCTTACAAATTCTAAAATTTGTATTTGATCCAGTTCTAGCGAATTCAAAAAATAAAACAATTAAAAATAATTCTCTTGGAATAAAAGAAATAAATAAACAAGTTCCTCATTGGTCATACAAATTAATTGACGATTTGGAACAACAAGAGGGAGAAAATGAAGAAAACATGGCGGAAGATTATGAAATTCGTTCACGAAAAGCCAAACGTGTAGTAATTTTTACCGATAATCAAGAAAATACAGCTACTTATAATAATACCAAAGATACGACAGATTTTGACCAAATAGACGAAGTTACTTTGATCCGTTATTCGCAACAATCAGACTTTCGTCGAGACATAATAAAAGGATCCACGCGAGCACAAAGACGCAAAATAGCTATTTTTGAACTGTTTCAAGCAAATGCGCATTCGCCCCTTTTTTTGGACAGAATAGACAAATCTCTTTTTTTTTCTTTTGATATTTTCGAACTGATAAAAACAATGTTTATAAATTGGATGTGTAAAAACGCAGAATTCACAATTTCAGATTATACTTATACAGAGAAAAAAACAAAAGAAAGTAAGAAAAAAGACGAGGACACAAGAGAAGACGACAAAAGAGAGGAAAGAACTCGGATAGAAATAGCTGAAGCCTGGGATAGCATTCTTTTTGCTCAAATAATAAGAGGTTGTATTTTAGTAACCCAATCGATTCTTAGAAAATATATTATATTACCTTTATTAATAATAGCTAAAAATTGCATTCGTATACTATTTTTTCAAATTCCCGAATGGTCGGAGGATTTAAAGGATTGGAGTAGAGAAATGCATATTAAATGCACTTATAATGGAGTTCAATTATCAGAAAAAGAATTTCCGAAAAACTGGTTAACAGACGGGATTCAAATAAAGATCCTATTTCCTTTTCGTCTAAAACCTTGGCACAGATCTAAGTTAAAATTCCCTCATAAAGATCCAATGAAAAAGAAAGTACAAAGAAATGATTTTTGTTTTTTAACAGTTTTGGGGATGGAGGCTGAACTGCCTTTTGGTTTTCCCCGAAAACGGCTTTCACTTTTTGAACCCATCTTTAAAAAACTTGAAAAAAAAATTATAAAAATGAAAAAAAAGGGTTTTCGAATTAGAAGAACTTTAGAAGAAAGAAGAAAATTATTTCTAAATTTATCAAAAGAAAAAGCAAACTGGGTCATCCAAAACTCTTTTTTTCGAAAAGACATAATAAACAAAATAAACAAATTTGAAAAATCAAAAAGAAACCGAATTCTATTATCTGGAGTTAAAGAAGTATATGAATTGAATGCAACTAAAAAAGAAAAAGATTCGCTAATCAATAAGAATAATCGGGCGAGTCAGAAAATGTCCACCTCAACGCGATCTATGGTTTGGACAAATTTTTCACTGACAGAAAAAAAAATGAAAGATCTTTCTGCTAGACGAAAGATAATCTTAAATCAAATAGAAAAAATTTCAAAAGAAAAGGAAAAAAAAATTATAACCTCGGAGATAAATATTAGTCCTAACAAAATAAGTTATAATGCTAAAACATTAAAATTAAAATCATCAAAAAATATTTCACAGATATTAAAAAGAAGAAATGCGCAATTAGCGCATAAATTCCATTTTTTTATAAAAATTTTGATTGAAAGGATATACATAGATATCTTTTTAGGTATCATTAATATTCCGAGGATCAATGCACAGCTTTTTCTTGAATCAACAAAAAAAATTGTTACTAAATACATTTACAATAATGAAGAAAATAAAAAAAAAATTGATAAAACAAACCAAAATACAATTCACTTTATTTCGATTATAAATATCAAAAAGTCACTTAATAATAGCAATATTGATGTTATTAATAAGAATTCACAGATTCTTTGCGACATATCCTCCTTGTCACAAGCATATGTATTTTACAAATTATCACAAATCAAAATTATTAACTTATATAAGTTAAGATCTATCCTTCAATATCATGGACTTTTTTTAAAGAATGAAATAAAGGATTATTTTAGAGTCCAAGGGCTATTTAATTTCGAATTAAAAGAAAAAAATGTTAGAAATTCTGTAATGAATCAATGGAAAAACTGGTTAAGGAGTCATTATCAATATAAATACGATTTATCTCAGATTAGATGGTCTAGATTAATACCACAAAAATGGCGAAATAGAATCAATCAATACCAACACCCTATGGTTCAAAACAAAAAATTAAACAAATGGAATTTATATGAAAAAGACAAATTAATTCATTACGAAAAAAAAAAGGATTTTGAGGCAGACTCATTACCGAATCAAAAAGATAATTTTAAAAAACACTATAAATATAATCTTTTATCATATAAATCTATTAATTATGAAAATAAGAAGGACTCATATATTTATGGATCACCATTACAAATAAATAATAAACAAAAGATTTCTTATAATTACAACACAAATAAAAGTAACTTTTTCGACATGTTAGAAGGTATTCCTATCAATAATTATCTAGCGGAAGATGATATTATCGATATGGCGAAAAGCCCAGATAGAAAATATTTTGATTGGAAAATTCTCAATTTTTGTCTTAGAGAGAAGGTCGAGATTGAATCCTGGATCGATACCGGAAGCGAACATAAAAAAAATACTAAGACTACTAAGTATCAAATAATTGAGAAAATGGATAAAAAAAAGATTTTTTTTATTACAATTCACCAAGATCAAGAAGTCAATTCATCCAATCAAAAAAAAGATATTGAAAAAGATTATATGGAATTAGATATGAAAAAACATAGACATAAAAAGAAAAACAAAAGTCATACGGAAGTAGAACTTGATTTCTTTCTAAAACGATATTTATATTTTCAATTAAGATGGAATGGTTCTTTAAATCAAAAAATAATCAATAATATCAAAGTATATTGTCTCCTGCTTAAACTGACAAATCCACGCGAAATTATTATATCTTCTATTCAAAGGCAAGAAATAAGTCTGAATATTTTGATGATTCAGAAGGATTTAACTCTTACAGAATTGATGAAAAAGAGAATATTTACTATCGAACCTATTCGTCTATCGGTAAAAAATAATGGACAATTTCTTTTGTATCAAACCATAAGTATCTTATTATTTCATAAGAACAAACAACAAATAAATCAACAATACAGAGATAAAATCGATTCTATTTCTATTGAAAGGCAGCAAGGTATAATTGGAAATAGAGACAAAAATGGTTATGATTTACTTGTTCCCGAAAATGTTTTATCCCCGAAACGTCGTAGAGAATTAAGAATTCTAATTTCTTTCAATTTAAAAAATAAAAACAATATTAATATTTATATAAATACCAAAATTTGCACTGGTAATAACAAAAAAAACTGTGGTCCCGTTTTTGATAAAAGCAAAGATTTTGATAAAGAGAAAAATAAACTAATTAAATTCAAATTTTTTCTTTGGCCCCATTTTCGATTAGAAGATTTAGCTTGTATGAATCGTTATTGGTTCGACACTAATAATTCCAGCCGGTTCGGTATGGTAAGGATATGTATCTATCCGCGGTTGAAATTTTGTAATGGTAAGGGTGCATTTTTCCTATATCCCATAGGATACTTGATCTAG

>lcl|NC_016736.1_cds_YP_005090217.1_61 [gene=rpl22] [locus_tag=RCOM_ORF00083] [db_xref=GeneID:11542359] [protein=ribosomal protein L22] [protein_id=YP_005090217.1] [location=complement(89196..89678)] [gbkey=CDS]
ATGATAAATAAAAGAAAGAAAAAGAGAGATCCATATACGGAAGTATATGCTTTAGGCCAACATATATGTATGTCCACTCACAAAGCACGAAGAATAATTGATCAGATTCGTGGACGTTCTTATGAAGAAACACTTATGATACTCGAGCTTATGCCTTATCGAGCATGTTATCCCATTTTAAAATTAATTTATTCTGCAGCAGCAAACGCTCGCCACAATATGGGTTTCAACGAAGCCAATTTAATCATTAGTAAAGCCGAAGTCAACGAAGGTGCTACTGTGAAAAAATTAAAACCTCAGGCTCGAGGGCGGGGTTATCTGATAAAAAGATCGACCTGTCATATAACTATTGTATTAAAAAATATATCTTTATATGAAGAATATGAAGAATATGAAGAATATAACATATGCTTAAAAAACCGGGGGTGGATAAAAAAAACGAAATCCACAGATGCGACATTTCATGATATGTATAGTAAGTAG

>lcl|NC_016736.1_cds_YP_005090211.1_55 [gene=rps11] [locus_tag=RCOM_ORF00077] [db_xref=GeneID:11542352] [protein=ribosomal protein S11] [protein_id=YP_005090211.1] [location=complement(84579..84995)] [gbkey=CDS]
ATGGCAAAACCTTTACCAAGAATTGGTTCACGCAGGAATGGACGTATTGGTTCACGTAAGAGTGCGCATAAAATACCAAAAGGAGTTATTCATGTTCAAGCAAGTTTCAACAATACTATTGTGACCGTTACAGATGTACGGGGTCGAGTGATTTCTTGGTCCTCCGCCGGCACTTGTGGATTCAGGGGCACAAGAAGAGGAACGCCATTTGCAGCTCAAACCGCAGCAGGAAATGCTATTCGAACAGTAGTGGATCAAGGTATGCAACGGGCAGAAGTCATGATAAAAGGTCCTGGTCTCGGGCGAGATGCAGCATTAAGAGCTATTCGCAGAAGTGGTATATTATTAAGCTTCGTCCGGGATGTAACCCCTATGCCACATAATGGCTGCAGGCCCCCTAAAAAAAGGCGTGTGTAA

>lcl|NC_016736.1_cds_YP_005090210.1_54 [gene=rpoA] [locus_tag=RCOM_ORF00076] [db_xref=GeneID:11542351] [protein=RNA polymerase alpha subunit] [protein_id=YP_005090210.1] [location=complement(83515..84513)] [gbkey=CDS]
ATGATTCGAGAGAAAGTAACAATATCTACTCGGACACTGCAGTGGAAATGTGTTGAATCAAGAACTGACAATAAGCGTCTTTTTTATGGACGCTTTATTCTGTCACCACTTATGAAAGGCCAAGCCGACACAATAGGCATTGCGACGCGAAGAGCTTTGCTTGGAGAAATCGAAGGAACATGTATCACACGTGCAAAATCTGAGAAAATACCACACGAATTTTCGACTATAGCAGGTATTCAAGAATCAATACATGAAATTTTAATGAATTTGAAAGAAATTGTATTGAGAAGCAATTTGTATGGAACTTGTGACGCGTCTATTTGTGTCAAGGGTCCTGGATATATAACTGCTCAAGACATCATTTTACCACCTTTTGTAGAAATCATTGATAATACACAGCATATCGCTAGCCTAACGGAACCAATTGATTTGTGTATTGGATTACAAATCGAGAGAAATCGCGGCTATCGTATAAAACCGACAAATAACTTTCACGAGGGAAGTTATCCTATAGATGCTGTATTTATGCCTGTTCGAAATGCGAATCATAGTGTTCATTCTTATGGAAATGGGAATGAAAAGCAAGAGATACTTTTTCTCGAAATATGGACAAATGGGAGTTTAACTCCTAAAGAAGCACTTCATGAAGCCTCCCGGAATTTGATTGATTTATTTATTCCTTTTCTACATGCAGAAGAAGAAAACTTCCATTTAGAAAAAAATCAACACAAGGTTACTTTACCTCTTTTTACCTTTTATGATAGATTGACTAAATTAAGAAAAAATCAAAACGAAATAACATTAAAATACGTTTTTATTGACCAATCAGAATTGACTCCTAAGATCTATAATTGCCTCAAAAGATCCAATATACATACATTATCGGACCTTTTGAATAAGAGTCAAGAAGATCTTATGAAAATTGAACATTTTCGTATAGACGATGTAAAACATATATTGGGTATTCTAGAAATAGAAAAAAATTTCGCAATTTAA

>lcl|NC_016736.1_cds_YP_005090231.1_75 [gene=ndhG] [locus_tag=RCOM_ORF00109] [db_xref=GeneID:11542385] [protein=NADH dehydrogenase subunit 6] [protein_id=YP_005090231.1] [location=complement(125895..126428)] [gbkey=CDS]
ATGGATTTGCCTGGACTAATACATGATTTTCTTTTATTTTTTATGGGATTAGGTCTTATATTAGGAGGTCTAGGAGTGGTATTACTTACCAACCCAATTTATTCTGCCTTTTCATTGGGATTGGTTCTTGTTTGTATATCTTTATTCTATATTTTATCAAACTCTCATTTTGTAGCTGCTGCGCAGCTCCTTATTTATGTGGGAGCCATAAACGTTTTAATTATATTTGCCGTGATGTTCATGAATGGTTCAGAATATTACAAAGATTTTAATCTTTGGACTGTTGGAACCGGAGTTACTTCCTTAATTTGTACAAGTATTTTTGTTTCACTAATTACTATTATTCCAGATACGTCATGGTACGGGATTATTTGGACTACAAAAACAAATCAGATTATAGAACAAGATTTAATAAGTAATGGTCAACAAATTGGAATTCATTTATCAACAGATTTTTTTCTTCCGTTCGAATTCATTTCAATAATTCTTTTAGTTGCTTTGATAGGTGCGATTGCTGCGGCTCGTCAGCAATAA

>lcl|NC_016736.1_cds_YP_005090201.1_45 [gene=rpl20] [locus_tag=RCOM_ORF00068] [db_xref=GeneID:11542342] [protein=ribosomal protein L20] [protein_id=YP_005090201.1] [location=complement(73797..74150)] [gbkey=CDS]
ATGACCAGAATTAGACGAGGATATATAGCTCGGAGGCGTAGAACAAAAATTCGTTTATTCGCATCAAGCTTTCGCGGGGCTCATTCAAGACTTACTCGAACTATTATTCAACAAAAAATAAGAGCTTTGGCTTTGGCCCATCGGGATAGAGATAGGCAAAAAAGAAATTTTCGTCGTTTGTGGGTCACTCGGATAAATGCAGTAATTCGTGAGAATAGGGTATCCTATAGTTATAGTATATTAATAAATAATCTGTACAAGAGACAGTTGCTTCTTAATCGTAAAATACTTGCACAAATAGCTATATTAAATAGGAATTGCCTTTATATGATTTCCAATGACATTATCAAATAA

>lcl|NC_016736.1_cds_YP_005090178.1_22 [gene=psaA] [locus_tag=RCOM_ORF00037] [db_xref=GeneID:11542311] [protein=photosystem I P700 chlorophyll a] [protein_id=YP_005090178.1] [location=complement(43917..46169)] [gbkey=CDS]
ATGATTATTCGTTCGCCGGAACCAGAAGTAAAAATTTTGGTAGATAGGGATCCCGTCAAAACTTCTTTCGAGGAATGGGCTAGACCGGGTCATTTCTCAAGAACAATAGCTAAAGGACCAGATACTACCACTTGGATCTGGAACCTACATGCTGATGCTCACGATTTCGATAGCCATACCAGTGATTTGGAGGAGATTTCTCGAAAAGTATTTAGTGCTCATTTCGGCCAACTCTCAATCATCTTTCTTTGGCTGAGTGGCATGTATTTCCACGGTGCTCGTTTTTCCAATTATGAAGCATGGCTAAGTGATCCTACTCACATTGGACCTAGTGCCCAAGTGGTTTGGCCAATAGTGGGCCAAGAAATATTGAATGGTGATGTGGGGGGGGGGTTCCGAGGAATACAAATAACCTCCGGTTTTTTTCAGATTTGGAGAGCATCTGGAATAACTAGTGAATTACAACTGTATTGTACCGCAATTGGTGCATTGGTCTTTGCAGCCCTTATGCTTTTTGCCGGTTGGTTCCATTATCACAAAGCTGCGCCAAAATTGGCTTGGTTCCAAGATGTAGAATCTATGTTGAATCACCATTTAGCGGGGCTACTAGGACTTGGGTCTCTTTCTTGGGCAGGGCATCAAGTACATGTCTCTTTACCAATTAACCAGTTTCTAAACGCTGGAGTGGATCCTAAAGAAATCCCACTTCCTCATGAATTTATCTTGAATCGGGATCTTTTGGCTCAACTTTATCCCAGTTTTGCTGAGGGAGCAACCCCATTTTTCACCTTGAATTGGTCAAAATATTCGGAATTTCTTACTTTTCGTGGAGGATTAGATCCAGTGACTGGGGGTCTATGGCTGACCGATATTGCACACCATCATTTAGCTATTGCAATTCTTTTCCTGGTAGCTGGTCACATGTATAGGACTAACTGGGGTATTGGTCATGGTATAAAAGATATTTTAGAGGCTCATAAAGGCCCATTTACAGGTCAGGGCCATAAAGGCCTATATGAGATCCTAACAACGTCGTGGCATGCTCAATTATCTCTTAACCTAGCTATGTTGGGTTCTTTAACCATTGTTGTAGCTCACCATATGTATTCCATGCCTCCTTATCCATATCTAGCTACTGACTATGGTACACAACTGTCATTGTTCACACATCACATGTGGATTGGTGGATTTCTCATAGTTGGTGCTGCTGCGCATGCAGCCATTTTTATGGTAAGAGACTATGATCCAACTACTCGATACAACGATCTATTAGATCGTGTCCTGAGGCATCGCGATGCAATCATATCACATCTCAATTGGGTATGTATATTTTTAGGCTTTCACAGTTTTGGTTTATATATTCATAATGATACCATGAGTGCTTTAGGGCGCCCTCAAGATATGTTTTCAGATACTGCTATACAATTACAACCCGTCTTTGCTCAATGGATACAAAACACCCATGCTATTGCACCGGGTGCAACGGCTCCTGGTGCAATAGCAAGCACCAGTTTGACTTGGGGGGGTGGCGATTTAGTGGCAGTGGGTGGCAAGGTTGCTTTGTTACCGATTCCATTAGGAACCGCGGATTTTATGGTACATCACATTCATGCATTTACGATTCATGTGACAGTATTGATACTTCTGAAAGGAGTTCTATTTGCCCGTAGCTCTCGTTTGATACCGGATAAAGCAAATCTTGGTTTTCGTTTCCCTTGTGATGGACCTGGAAGAGGGGGAACATGTCAAGTATCCGCTTGGGATCATGTCTTCTTAGGGCTGTTTTGGATGTACAATTCAATTTCGGTAGTAATATTCCATTTCAGTTGGAAAATGCAGTCAGATGTTTGGGGTAGTATAAGTGATCAAGGGGTGGTAACTCATATCACGGGAGGAAACTTTGCACAGAGTTCCATTACTGTTAATGGGTGGCTCCGCGATTTCTTATGGGCACAGGCATCCCAGGTAATTCAGTCTTATGGTTCTTCATTATCTGCATATGGCCTTTTTTTCCTAGGCGCTCATTTTGTATGGGCTTTTAGTTTAATGTTTCTATTCAGTGGTCGTGGTTATTGGCAAGAACTTATTGAATCAATCGTGTGGGCTCATAATAAATTAAAAGTTGCTCCTGCTACTCAGCCTCGAGCCTTGAGCATTATACAAGGACGTGCTGTAGGAGTAACCCATTACCTTCTGGGTGGAATTGCCACAACATGGGCGTTCTTCTTAGCAAGAATTATTGCAGTAGGATAA

>lcl|NC_016736.1_cds_YP_005090232.1_76 [gene=ndhI] [locus_tag=RCOM_ORF00110] [db_xref=GeneID:11542386] [protein=NADH dehydrogenase subunit I] [protein_id=YP_005090232.1] [location=complement(126805..127317)] [gbkey=CDS]
ATGTTTCCCATGGTAACTGGGTTCATGAATTATGGGCAACAAACCATACGAGTTGCACGGTATATTGGTCAAAGTTTCATGATTACCTTATCCCATGCAAATCGTTTACCTGTAACTATTCAATATCCTTATGAAAAATTAATCACATCGGAGCGTTTCCGCGGTCGAATCCATTTTGAATTTGATAAATGCATTGCTTGTGAAGTATGTGTTCGGGTATGTCCTATAGATCTACCTGTTGTTGATTGGAAATTGGAAACTGACATTCGAAAGAAACGGTTGCTTAATTACAGTATTGATTTCGGAATCTGTATATTTTGTGGCAACTGTGTTGAGTATTGTCCAACAAATTGTTTATCAATGACTGAAGAATATGAACTTTCCACTTATGATCGTCACGAATTGAATTATAATCAAATTGCTTTAGGTCGTTTACCAATGTCAGTAGTCGACGATTATACAATTCGAACAATTTTGAATTCAACTAGAAACAAAAAAGATAACCCGCTTTGA

>lcl|NC_016736.1_cds_YP_005090224.1_68 [gene=ycf1] [locus_tag=RCOM_ORF00101] [db_xref=GeneID:11542377] [protein=hypothetical chloroplast RF1] [protein_id=YP_005090224.1] [location=115822..117018] [gbkey=CDS]
ATGATTTTTAAATCTTTTATACTAGGTAATCTAGTATCCTTATGCATGAAGATAATCAATTTGGTCGTTGTGGTCGGACTCTATTATGGATTTCTGACCACATTCTCCATGGGGCCCTCTTATCTCTTCCTTCTCCGAGCTCGGGTTATAGAAGAAGGAGAAGAAGGAACTGAGAAGAAGGTATCAGCAACAACAGGTTTTATTACGGGACAGCTCATGATGTTCATATCGATCTATTATGCGCCTCTGCATCTAGCATTGGGTAGACCTCATACAATAACTGTCCTAGCTCTACCCTATCTTTTGTTTCATTTCTTCTGGAACAATCACAAACACTTTTTTGATTATGGAGCTACTACCAGAAATTCAATGCGTAATCTTAGCATTCAATTTGTATTCCTGAATAATCTCATTTTTCAATTATTCAACCATTTCATTTTACCAAGTTCAATGTTAGTCAGATTAGTCAACATTTATATGTTTCGATGCAACAACAAGATGTTATTTGTAACAAGTAGTTTTGTTGGTTGGTTAATTGGTCACATTTTATTCATGAAATGGGTTGGATTGATATTAGTCTGGATACAACAAAATAATTCTATTAGATCTAATGTACTTTTTCGATCTAATAAGTACCTTGTGTCAGAATTGAGAAATTCTATGGCTCGAATCTTTAGTATTCTCTTATTTATTACCTGCGTCTACTCTTTAGGCAGAATACCGTCACCCATTTTTACTAAGAAACTGAAAGAAACCTCAGAAACGGAAGAAAGGGAGGAAAGTGAGGAAGAAACAGATGTAGAAATAGAAACAACTTCCGAAACGAAGGGGACTAAACAGGGATCCACCGAAGAAGATCCTTCTTCTTCCCTTTTTTCGGAAGAAAAGGAGGATCGGGACAAAATCGACGAAACGGAAGAGATCCAAGTGAATGGAAAGGAAAAAACAAAGGATGAATTCCATTTTCACTTTAACGAGACATGCTATAAAAATAGACCACTTTATGAAACTTTTTATCTGGATGGGAATCAAGAAAATTCGAAGTTAGAAATATTGATAGAAAAAAAAAAGAAAAATCTTTTCTGGTTTGAAAAACCTCTTGTAACTATTCTTTTTGACTCGAAACGTTGGAATCGTCCATTACGATATATAAAAAATGATCAGTTTGAGAATGCTGGAATGCTATATAAATGTTAA

>lcl|NC_016736.1_cds_YP_005090168.1_12 [gene=rpoC2] [locus_tag=RCOM_ORF00019] [db_xref=GeneID:11542293] [protein=RNA polymerase beta subunit] [protein_id=YP_005090168.1] [location=complement(17988..22205)] [gbkey=CDS]
ATGGAGGTACTTATGGCCAAACGGGCCAATCTGGTCTTTCACAATAAAGTGATAGATGGAACTGCCATTAAACGACTTATTAGCAGATTAATAGATCATTTTGGAATGGCATATACATCACACATCCTGGATCAAGTAAAGACTCTGGGTTTCCAGCAAGCCACTGCTACATCCATTTCATTAGGAATTGATGATCTTTTAACAATACCTTCTAAGGGATGGCTAGTTCAAGATGCTGAACAACAAAGTTTGATTTTGGAAAAACACTATCATTATGGAAATGTACACGCGGTAGAAAAATTACGCCAATCTATTGAGATATGGTATGCTACAAGTGAATATTTGCGACAAGAAATGAATCTTAATTTTAGGATGACGGAACCCTTTAATCCAGTCCATATAATGTCTTTTTCGGGAGCTAGGGGAAATGTATCTCAAGTACACCAATTAGTAGGTATGAGAGGATTAATGTCGGATCCACAAGGACAAATGATCGATTTACCTATTCAAAGCAATTTACGCGAAGGACTATCTTTAACAGAATATATCATTTCTTGCTACGGAGCCCGCAAAGGGGTTGTCGATACCGCTGTACGAACATCAGATGCTGGATATCTTACGCGTAGACTTGTTGAAGTAGTTCAACACATTGTTGTACGTAGAACAGATTGTGGCACCACCCGAGGGATTTCTGTGAGTCCTCAAAATGGGATGATGTCGGAAAGAATTTTTATTCAAACATTAATTGGTCGTGTATTAGCAGACAATATATATATGGGTCTACGATGCATTGCCATTCGAAATCAAGATATTGGGATTAGACTTGCCAATCGATTCATAACCTTTCGAACACAAACAATATCTATTCGAACTCCCTTTACTTGTAGGAGTACGTCTTGGATCTGTCGATTATGTTATGGCCGGAGTCCTACTCATGGCGATCTAGTGGAATTGGGGGAAGCCGTAGGTATTATTGCAGGTCAATCCATTGGAGAGCCGGGTACTCAACTAACATTAAGAACATTTCATACCGGCGGAGTATTCACAGGGGGTACTGCAGAACATGTACGAGCCCCCTCTAATGGAAAAATAAAATTTAACGAGGATTTGGTTCATCCCATACGTACACGTCATGGGCATCCTGCTTTTCTATGTTATATAGACTTGTATGTAACTATTGAAAGTCACGATATTATACATAACGCTACTATTCCACCAAAGAGTTTCCTTTTAGTTCAAAACAATCAATATGTAGAATCAGAACAAGTGATTGCTGAAATTCGGGCGGGAACATACACTTTGAATTTTAAAGAGAAGGTTCGAAAACATATTTATTCCGATTCAGAAGGGGAAATGCACTGGAGTACTGATGTATACCATGCACCTGAATTTACATATAGTAATGTCCATCTCTTACCAAAAACAAGTCATTTATGGATATTATCAGGAAATTCGTGCAGATCCAGTATAGTTCCTTTTTCGCTCCATAAGGATCAAGATCAAATGAACGTTCATTCTCTTTCTATCAAAAGAAGATATATTTCTAGTCCTTCCGTAAATTCCGTAAATAATGATCAAGTTAAACCCAAATTCTTTAGTTCAGATTTTTCGGGTAAAAAACCAAGTAGGATTCCTTATTATTCAGAACTTAATCGAATCGTATGTACTGGTCATTGTAATCTCATATATCCCGCGATTCTCTACGAGAATTCTGATTTATTGGCAAAGAGGCGAAGAAATAAATTCATCATCCCATTCCAATCAATTCAAGAACAAGAAAAAAAACTAATGACCCGCTCCTCGGCTATCTCGATTGAAATACCGCTAAATGGTATTTTCCGTAGAAATAGTGTTTTTGCTTATTTTGACGATCCCCAATACCGAAGAAAGAGTTCGGGAATTACTAAATATGGGGCTATAGGGGTGCATTCAATCGTCAAAAAAGAAGATTTGATTGAGTATCGGGGAGTCAAAGAATTTAAGCCAAAATACCAAATGAAGGTAGATCGCTTTTTTTTCATTCCCGAAGAAGTGTATATTTTACCCGAATCTTCTTCCCTAATGGTACGGAACAATAGTATCATTGGAGTAGATACACCAATCACTTTAAATACAAGAAGTCGGGTGGGCGGATTGGTCCGAGTGGAGAGAAAAAAAAAAAAAATTGAGCTTAAAATCTTTTCTGGAGATATCCATTTTCCGGGAGAGACAGATAAAATATCCCGACACAGTGGTATCTTGATACCACCAGGAATGGTAAAAACAAATTCTAAGGAATCAAAAAAACAGAAAAACTGGATCTATATCCAACGAATCGCACCTACCAGGAAAAAGTATTTTGTTTTGGTTCGACTAGTAATCATATATGAGATAGCGAACGGTATAAATTTAGAAACACTTTTCCCCCGGGATTTATTGCAGGAAAAGGATAATCTGAAACTTCGAGTTGTCAATTATATTCTTTCTGGAAATGGTAAACCGATTCGAGGAATTTCTGACACAAGTATTCAATTAGTTCGTACTTGTTTAGTGTTGAATTGGGACCAAGAAAAAAAAAGTTCTTCTATCGAAGAGGCCCGCGCTTCCTTTGTTGAAGTAAACACAAATGGTCTGATTCGTGATTTCTTAAGAATCAACCTAGTGAAATCCCATATTTCATATATCAGTAGAAAAAGGAATGATCCATCGGGTTCAGGGCCGATCTCTAATAATGGGGCAAATCACACCAATATTAATCCATTTTATCCCATCTATTTCAAGACAAGGATTCAACAATCACTTAAACAAAATCAAGGAACTATTAGTACGTTGTTGAATAGAAATAAGGAATGTCAATCTTTGATAATTTTGTCATCATCTAATTGTTTTCGAATGGATCCCTTCAACGATGTAAAACATCACAATGTAATAAAAGAATCAATTAAAAGAGATCCTATAATTCCAATTAGAAATTCGTTGGGCCCTTTAGGAACAGCCCTTCAAATTGCGAATTTGTATTTATTTTACCATTTAAATTTAATAACTCATAATCGGATCTCAGTAACTAAATATTTGAAACTTGACAATTTAAAACAGACTTTTCGAGTACTTAAATATTATTTAATGGACGAAAACGGGAGAGTTGTTAATCCCGATCCATGCAGTAACAGTGTTTTGAATCCATTCAATTTGAATTGGTATTTTCTCCATCATAATTATCATCATAATTATTGTCATAATTATTGTGAAGAAAGCTTCACAATAATTAGCCTGGGACAGTTTATTTGCGAAAATGTCTGTATGGCCAAAAACGGACCACATCTAAAATCGGGTCAAGTTATAATTATTCACATTGGCTCTGTAGTAATAAGATCCGCTAAGCCTTATTTGGCCACTCCGGGAGCAACCGTTCATGGCCATTATGGAGAAATCCTTTACGAAGGAGATACATTAGTTACATTTATATATGAAAAGTCGAGATCTGGTGATATAACGCAGGGTCTTCCAAAAGTAGAACAAGTGTTAGAAGTGCGTTCAATTGATTCAATATCGATAAACCTAGAAAAGAGAGTTGGGGGTTGGAACGAATGTATACCAAGAATTCTTGGAATTCCTTGGGGATTCTTGATTGGTACTGAGCTAACTATAGTGCAAAGTCGCATCTCTTTGGTTAATAAGATCCAAAGGGTTTATCGATCCCAAGGGGTGCAGATCCATAATAGGCATATAGAAATTATTGTACGTCAAATAACATCAAAAGTATTGGTTTCAGAAGACGGAATGTCTAATGTTTTTTCACCCGGAGAACTAATTGGATTGTTGCGAGCGGAACGAACGGGACGTGCTTTGGAAGAAGCCATCTGTTACCGAGCCATATTATTGGGAATAACGAGAGCATCCCTGAATACTCAAAGTTTCATATCCGAGGCCAGTTTTCAAGAAACTGCTCGTGTTTTAGCAAAAGCCGCTCTCCGCGGTCGTATCGATTGGTTGAAAGGCCTGAAAGAAAACGTTGTTCTAGGCGGTATGATACCCGTTGGTACCGGATTCAAAGGATTAGTACAAGGTTCAAGGCAATATAAGAACATTCCTTTGAAAACCAAAAAGAATAATTTATTCGGGGGGGAATTTAGAGATAGAGATATTTTATTCCACCACAGAGAGTTATTTTATTCTTGCATTTCAAAAAATTTCTACGATACATCAGAACAATCATTTATAGGATTTAATGATTCCTAA

>lcl|NC_016736.1_cds_YP_005090225.1_69 [gene=ndhF] [locus_tag=RCOM_ORF00102] [db_xref=GeneID:11542378] [protein=NADH dehydrogenase subunit 5] [protein_id=YP_005090225.1] [location=complement(117322..119571)] [gbkey=CDS]
ATGGAACATATATATCAATATTCATGGATCATACCTTTTGTTACATTCCCAGCTCCTATGTTAATAGGAGCGGGACTCCTACTTTTTCCGGCGGCAACAAAAAAACTTCGTCGTATGTGGGCTTTTCCAAGCCTTTTCTTGTTAAGTATAGTCATGATTTTTTCAATCGACTTGTCTATTCACCAAATAAATAGCAGTTTTATTTATCAATATATATGGTCGTGGACTATCAATAATGATTTTTCTTTAGAATTCGGACACTTGATTGACCCACTTACTTCTATTTTGTCAGTATTAATTACTACAGTTGGAATTATGGTTCTTTTTTATAGTGACAATTATATGTCTCATGATCAAGGCTATTTGAGATTTTTTGCTTATATGAGTTTTTTCAATACTTCAATGTTGGGATTAGTTACTAGTTCTAATTTGATACAAATTTATATTTTTTGGGAGTTGGTTGGAATGTGTTCTTATCTATTAATAGGGTTTTGGTTCACACGACCTATTGCATCGAATGCTTGTCAAAAAGCCTTTGTAACTAATCGTGTAGGGGATTTTGGTTTATTATTGGGAATTTTGGGCATTTATTGGATAACGGGCAGTTTCGAATTTCGGGATTTGTTCAAAATATTGAATAACTTGATTTCTAATAATCAGGTTCATTTTTTATTTGTTACTTTGTGTGCCTTTCTAGTATTTTCTGGCGCAATTGCTAAATCGGCGCAATTTCCTCTCCATGTATGGTTACCGGATGCCATGGAAGGGCCTACTCCTATTTCGGCTCTAATACATGCTGCTACTATGGTAGCGGCAGGAATTTTTCTTGTAGCTCGACTTTTTCCTCTTTTTGTAGTCATACCTTACATAATGAATCTAATAGCTTTGATAGGTATAATAACAGTATTTTTAGGAGCTACTTTAGCTCTTGCTCAAAAAGATATTAAGAGAAGTTTAGCCTATTCTACAATGTCTCAATTAGGTTATATGATGTTAGCTCTAGGTATGGGGTCTTATCGAGTCGCTTTATTTCATTTGATTACTCATGCCTATTCGAAAGCATTATTGTTTTTAGGATCTGGATCCATTATTCATTCAATGGAAGCTATTGTTGGTTATTCTCCAGATAAGAGTCAAAATATGATTCTTATGGGTGGTTTAACAAAACATATTCCAATTACAAAAACTGCTTTTTTTTTAGGAACACTTTCTCTTTGTGGTATTCCACCCTTCGCCTGTTTTTGGTCCAAAGATGAAATTCTTAACGATAGTTGGTTGTATTCACCGGTTTTCGCAATAATAGCTTGTTTCACGGCCGGATTAACTGCATTTTATATGTTTCGGGTTTATTTACTTACTTTTGAAGGACATTTAAATGTTCATTTAAAAAATTACAGTGGTAAAAAAAACAGTTCATTTTATTCAATATCTTTATGGGGTAAAGAAGGATCAAAAATGCTTAACGAAAGTTTGGGTTTATTACCTTTCTTAACAACGAATAATAACGAAAGGGCTTCTTTTTTTTGGAAGAACACATATCAAATTGATGGTAATGTAAGAAAGATGATGTGGCCTTTTATTACTATTAAAAATTTTAACACTAAAAGGATTTTTGCCTATCCTCATGAATCGGATAATACTATGTTATTTCCTATGCTTGTCTTGGTACTATTTGCTTTGTTTATTGGAGCCATAGGAATTCCTTTCAATCAAATTCCTTTCAATCAAAAAGGAATGGAGGTGGATATATTGTCAAAACTGTTAACTCCGTCTTTAAACCTTTTGCATCAAAATTCAAAGAATTCTGTGGATTGGTATGAATTTGTAACAAATGCAATTTTTTCAGTCAGTATAGCTTTTTTCGGAATATTTATAGCGTCCTCTTTATATAAGCCTGTTTATTCATCGTTACAAAATTTGAATTTCTTGAATTCGCTCGCTAAAAAAGGTTCTAAGAGAATTCTTTGGGACAAAATAATAAACGTCATATATAACTGGTCCTATAATCGAGGTTACATAGATGCTTTTTATGAAATATCTTTTATTGGAGGTATAAGAAAATTAGCTGAATTAATTCATTTTTTTGATAAACGAATAATTGATGGAATTACCAATGGAGTTGGTGTTACCAGTTTCTTTGTAGGAGAGGGTATAAAATATGTAGGAAGCGGTCGCATCTCTTCTTATCTCTTATTATATTTATTTTATGCATTAATCTTTTTATTAATTTATTCTTTTTACAATTTGTAA

>lcl|NC_016736.1_cds_YP_005090163.1_7 [gene=atpA] [locus_tag=RCOM_ORF00014] [db_xref=GeneID:11542288] [protein=ATP synthase CF1 alpha subunit] [protein_id=YP_005090163.1] [location=complement(11342..12865)] [gbkey=CDS]
ATGGTAACCATTCGAGCCGACGAGATTAGTAATATTATCCGCGAACGTATTGAGCAATATAATAGGGAAGTAAAGATTGTAAATACGGGTACCGTACTTCAAGTAGGCGACGGCATTGCTCGTATTTATGGTCTTGATGAAGTAATGGCAGGTGAATTAGTAGAATTTGAAGAGGGTACAATAGGCATTGCTCTGAATTTGGAATCAAATAATGTCGGTGTCGTTTTAATGGGTGACGGTTTAATGATACAAGAGGGAAGTTCCGTAAAAGCAACAGGAAGGATTGCTCAGATACCGGTGAGTGAGGCTTATTTGGGTCGTGTTATAAATGCCCTGGCTAAACCTATTGACGGTCGAGGTGAAATTTCAGCTTCTGAATCGCGGTTAATTGAATCTCCCGCTCCTGGTATTATTTCGAGACGTTCCGTATACGAGCCTCTTCAAACAGGACTTATTGCTATTGATTCGATGATCCCCATAGGACGCGGTCAGCGGGAATTGATTATTGGGGACAGACAGACCGGTAAAACAGCAGTAGCCACAGATACAATTCTCAATCAACAAGGACAAAATGTAATATGTGTTTATGTAGCTATTGGGCAAAAAGCGTCTTCTGTGGCTCAGGTAGTGACTACTTTACAGGAAAGAGGGGCAATGGAGTACACTATTGTGGTAGCCGAAACGGCGGATTCTCCGGCTACATTACAATACCTCGCTCCTTATACAGGAGCAGCTCTGGCTGAATATTTTATGTACCGTGAACGACACACCTTAATCATTTATGATGATCTCTCCAAACAAGCGCAGGCTTATCGCCAAATGTCGCTTCTATTACGAAGACCACCTGGTCGTGAAGCTTATCCCGGAGATGTCTTTTATTTGCATTCACGCCTTTTGGAAAGAGCTGCTAAATCAAGTTCCCGTTTAGGTGAAGGAAGTATGACTGCTTTACCAATAGTCGAGACCCAATCAGGAGACGTTTCGGCTTATATTCCTACTAATGTAATTTCCATTACAGATGGACAAATATTCTTATCCGCCGATTTATTCAATGCTGGAATCAGGCCTGCTATTAATGTGGGTATTTCCGTTTCCAGAGTAGGATCCGCTGCTCAAATTAAAGCTATGAAACAAGTAGCTGGTAAGTTAAAATTGGAATTGGCGCAATTCGCAGAATTAGAAGCCTTTGCGCAATTCGCTTCGGATCTTGATAAAGCTACTCAGAATCAATTGGCAAGAGGTCAACGATTACGCGAGTTGCTCAAACAATCCCAATCAGCTCCTCTCACGGTGGAGGAACAGATAATGACTATTTATACCGGAACGAATGGTTATCTTGATTCATTAGAAATTGGACAAGTAAGGAAATTTCTCGTTGAGTTACGTACCTACTTAAAAACGAATAAACCTCAGTTCCAAGAAATCATATCTTCTACCAAAACATTCACTGAAGAGGCGGAAACCCTTTTGAAAGAAGCTATTCAGGAACAGAAGGAACGTTTTCTACTTCAGGAACAAGTCTAA

>lcl|NC_016736.1_cds_YP_005090159.1_3 [gene=matK] [locus_tag=RCOM_ORF00005] [db_xref=GeneID:11542412] [protein=maturase K] [protein_id=YP_005090159.1] [location=complement(2387..3907)] [gbkey=CDS]
ATGGAGGAATATCAAAGATATTTAGAACTAGATAGATCTCGAAAAAATAACTTCCTATACCCATTTATCTTTCGGGAGTATATTTATACATTTGCTCATGATCACAGTTTAAATAGATCTACTTTGTTGGAAAATTTAGGTTATGACAATAAATCTAGTCTATTAATTGTAAAACGTTTAATTACTCGAATGTATCAACAGAACCATTTGATTATTTCTGCTAATGATTCTAATCAAAATCCATTTTTTAAGTACAACAAGAATTTATATTATCAAATGATATCAGAGGGCTTTGCAGTTATTGTGGAAATTCCATTTTCCCTACGATTAGTATCTTCTTTAGAAAGGTCAGAGATAGTAAAATCTCATAAATTACGATCAATTCATTCAATATTTCCTTTTTTAGAGGACAAATTTCCACATTTAAATTATGTGTCAGATGTATTAATACCTTACCCCATCCATCTAGAAAAATTGGTTCAAATCCTTCGCTATTGGGTGAAAGATCCCTCTTCTTTGCATTTATTACGACTCTTTCTTCATGAGTATTGGAATTGGAACAGTTTTATTATTCCAAAGAAATCAATTTCTATTTTTACAAAAAGTAATCCAAGATTTTTCGTGTTCCTATATAATTCTCATGTATATGAATATGAATCCCTCTTCTTTTTTCTCCGTAACCAATCCTTTCATTTACGATCAACATTTTCTCGAGTACTTCTTGAACGAATTTTTTTCTATGGAAAAATAGAACATTTTGCGGAAGTCTTTGCTAATGATTTTCAGGCCATCCTATGGTTGTTCAAGGACCCTTTCATGCATTATGTTAGATATCAAGGAAAATCTGTTTTGGCTTCAAAAGATGGGCCTCTTCTGATGAAAAAATGGAAATATTACCTTGTCCATTTATGTCAATGTCATTTTTATGTGTGGTTTCAACCGGAAAAGATCTATATAAATTCATTATCTAAGCATTCTCTCAACTTTTTGGGCTATCTTTCAAATGTACAATTTAATCCTTCGTTGGTACGGAGTCAAATGATAGAAAATTCATTTATAATAGATAAAGATAATACTATGAAGAAACTCGATACAATAGTTCCAATTATTCCTTTAATTAGATCATTGGCAAAAATGAAATTTTGTAACGCAGCAGGACATCCCATTAGTAAACCGACCTGGGCGGATTCGGCAGATTCTGAGATTATCGACCGATTTGTGCGTATATACAGAAATCTTTCTCATTATTATAGCGGATCCTCAAAAAAAACGAATTTGTATCGAATAAAATATATACTTCGACTTTCTTGTGTTAAAACTTTGGCTCGTAAACACAAAAGTAGTGTACGCGCTTTTTTGAAAAGATTAGGTTCGGAATTTTTAGAAGAATTTTTTACGGAGGAAGAACAGATTCTTTTTTTGATCTTCCCAAGAGTTTCTTCTATTTCGCGCAGGTTATATAGAGAACGGATTTGGTATTTGGATATTATTTCTATCAATGATTTGGCCAATCATGAATAG

>lcl|NC_016736.1_cds_YP_005090228.1_72 [gene=ndhD] [locus_tag=RCOM_ORF00106] [db_xref=GeneID:11542382] [protein=NADH dehydrogenase subunit 4] [protein_id=YP_005090228.1] [location=complement(123195..124715)] [gbkey=CDS]
ATGTATCTTGCCTTTACCACGAATTCTTTTCCTTGGTTAACAATATTTGTAGTTTTACCGATATCTGCGGGTTCCTTAATTTTCTTTTTCCCCCATAGAGGAAATAAGGTAATTAGGTGGTATACTTTGTTTATATGTATTTTAGAACTCCTTTTAATGACTTATGCGTTCTCTTATTATTTCCAATTGGACGACCCATTAATCCAATTAACAGAAGATTATAAATGGATCCAATTTTTTGATTTTTACTGGAGATTGGGAATAGATGGATTTTCTTTAAGCCCTATTTTACTGACAGGATTTATCACCACTTTAGCTACTTTAGCGGCTTGGCCAATTACTCGTGATTCCCGCTTATTTAATTTTCTGATGTTAGCAATGTATAGTGGTCAAATAGGATTATTTTCTTCTCAAGATCTTTTGCTTTTTTTTATCATGTGGGAGTTAGAATTAATTCCCATTTATCTACTTCTATCCATGTGGGGGGGAAAGAAACGTCTGTATTCAGCTACAAAGTTTATTTTGTATACTGCCGGGGGTTCCGTTTTTTTATTAATGGGAGCTTTGGGTATCGCTTTATATGGTTCCAATGAACCAAGATTCAATTTTGAAACATCAGCCAATCAATCATATCCTGTGGCGCTAGAAATATTTTTTTATATTGGATTTCTTATTGCTTTTGCTGTCAAATTACCGATTATACCCTTACATATATGGTTACCAGACACCCATGGGGAAGCACATTACAGTACTTGTATGCTTCTAGCCGGAATCTTATTAAAAATGGGGGCGTATGGGTTGGTTCGAATCAATATGGAATTATTACCTCATGCTCATTCTATCTTTTCTCCCTGGTTGATAATAGTAGGCGTCATGCAAATAATCTATGCAGCTTCAACATCTCCTGGTCAACGAAATTTAAAAAAAAGAATAGCCTATTCTTCTGTATCTCATATGGGTTTCATAATTATAGGAATTTGCTCTATAAGTGATATGGGACTCAATGGAGCCATTTTACAAATAATATCACATGGGTTTATTGGCGCTGCACTTTTTTTCTTAGCAGGAACGGGTTATGATAGAATACGTCGTGTGTATCTTGATGAAATGGGCGGAATGGCTACACTAATGCCAAAAATATTCACGACATTCAGTATCTTATCACTAGCTTCCCTTGCATTACCAGGCATGAGCGGTTTTTTTGCGGAATTGGCAGTATTTTTTGGAATAATTACCGGCCAAAAATATCTTTTAATGTCAAAAATAGTAATTACTTTTGGAATGGCAGTTGGAATGATATTAACTCCTATTTATTTATTATCTATGTTACGCCAGATGTTTTATGGATACAAGCTGTTTAATGCTCCAAACTCTTATTTTTTTGATTCTGGACCGAGGGAGTTATTTGTTTCGATCTCTATCCTTCTGCCTGTAATAGGTATTGGTATTTATCCGGATTTCCTTTTTTCATTATCAGTTGACAGGGTCGAAGCTCTTCTATCTAATTATTTTTATAGATAG

>lcl|NC_016736.1_cds_YP_005090219.1_63 [gene=rpl2] [locus_tag=RCOM_ORF00085] [db_xref=GeneID:11542361] [protein=ribosomal protein L2] [protein_id=YP_005090219.1] [location=complement(join(90498..90968,91598..91999))] [gbkey=CDS]
ATGGCGATACATTTATACAAAACTTCTACCCCGAGCACACGCAATGGAGCCGTAGACAGTCAAGTGAAATCCAATACACGAAATACACGAAATAATTTGATCTATGGACAGCATCGTTGTGGTAAAGGCCGTAACGCCAGAGGAATCATTACCGCAAGGCATAGAGGGGGAGGTCATAAGCGTCTATACCGTAAAATCGATTTTCGACGGAATGAAAAAGACATATATGGGAGAATCGTAACCATAGAATACGACCCTAATCGAAATGCATACATTTGTCTCATACACTATGGGGATGGTGAGAAGAGATATATTTTACATCCCAGAGGGGCTATAATTGGAGATACCATTATTTCTGGTACAGAAGTTCCTATAAAAATGGGAAATGCCCTACCTTTGAGTGAGGTTTTGATTGATCAAAAAGAAGAATCTACTTCAACCGATATGCCCTTAGGCACGGCCATACATAACATAGAAATCACATTTGGAAAGGGTGGACAATTAGCTAGAGCTGCAGGTGCTGTAGCGAAACTGATTGCAAAAGAGGGGAAATCGGCCACATTAAAATTACCTTCTGGGGAGGTTCGTTTAATATCCAAAAACTGCTCAGCAACAGTCGGACAAGTAGGGAATACTGGGGTGAACCAGAAAAGTTTGGGTAGAGCCGGATCTAAATGTTGGCTAGGTAAGCGTCCTGTAGTAAGAGGAGTAGTTATGAACCCTGTAGACCATCCCCATGGGGGTGGTGAAGGGAGGGCCCCAATTGGTAGAAAAAAACCCGCAACCCCTTGGGGTTATCCTGCACTTGGAAGAAGAAGTAGAAAAAGGAATAAATATAGTGATAATTTGATTCTTCGTCGCCGTAGTAAATAG

>lcl|NC_016736.1_cds_YP_005090157.1_1 [gene=rps12] [locus_tag=RCOM_ORF00001a] [db_xref=GeneID:11542404] [protein=ribosomal protein S12] [exception=trans-splicing] [protein_id=YP_005090157.1] [location=complement(join(104781..105023,74989..75099))] [gbkey=CDS]
ATGCCAACTATTAAACAACTTATTAGAAACACAAGACAGCCAATCAGAAATATCACCAAATCCCCCGCTCTCGGGGGATGTCCTCAGCGCCGAGGAACATGTACTAGGGTGACTATCACCCCCAAAAAACCAAACTCTGCCTTACGTAAAGTTGCCAGAGTACGATTAACCTCTGGATTTGAAATCACTGCTTATATACCTGGTATTGGCCATAATTCACAAGAACATTCTGTAGTCTTAGTAAGAGGGGGAAGGGTTAAGGATTTACCCGGTGTGAGATATCACATTGTTCGAGGGACCCTAGATGCTGTCGGAGTAAAGGATCGTCAACAAGGGCGTTCTAGTGCGTTGTAG

>lcl|NC_016736.1_cds_YP_005090164.1_8 [gene=atpF] [locus_tag=RCOM_ORF00015] [db_xref=GeneID:11542289] [protein=ATP synthase CF0 B subunit] [protein_id=YP_005090164.1] [location=complement(join(12926..13396,14113..14256))] [gbkey=CDS]
ATGAAAAATGTAACCGATTCTTTCGTTTCCTTGGGTCACTGGCCATCCGCCGGGAGTTTCGGATTTAATACCGATATTTTAGCAACAAATCCAATAAATCTAAGTGTAGTCCTTGGTGTATTGATTTTTTTTGGAAAGGGGGTGGTTTGGTTCGGGAAGGGATCATGGAAGTTTTGCAATGAATGGAAAGATAATCTACTTTCATTAACGGATTTATTAGATAATCGAAAACAAAGGATTTTGGATACTATTCGAAATTCAGAAGAACTACGTGAGGGGGCCATTGAACAGCTGGAAAAAGCCCGGGCCCGCTTACGGAAAGTGGAAATAGAAGCAGATCAGTTTCGAATGAATGGATACTCTGAGATAGAACGAGAAAAGTTGAATTTGATTAATTCAACTTATAAAACTTTGAAACAATTAGAAAATTACAAAAATGAAACCATTCATTTTGAACAACAAAGAACGATTAATCAAGTCCGACAACGGGTTTTCCAACAAGCCTTACAGGGAGCTCTAGGAACTCTGAATAGTTGTTTGACCAACGAGTTACATTTACGTACCATCAATGCTAATCTTGGCATGTTTGGGGCGATAAAAGAAATAACTGATTAG

>lcl|NC_016736.1_cds_YP_005090191.1_35 [gene=petA] [locus_tag=RCOM_ORF00056] [db_xref=GeneID:11542330] [protein=cytochrome f] [protein_id=YP_005090191.1] [location=66745..67707] [gbkey=CDS]
ATGCAAACTAGAAAGACCTTTTCTTGGATAAAGCAAGAGATTACTCGTTCCATTTCCGTATCGCTCATGATATATATAATAACTTGTGCATCCATTTCAAATGCATATCCCATTTTTGCACAGCAGGGTTATGAAAATCCGCGCGAAGCAACTGGCCGTATTGTATGTGCCAATTGTCATTTAGCTAATAAACCCGTGGATATTGAAGTTCCACAAGCGGTACTTCCTGATACTGTATTTGAAGCAGTCGTTCGAATTCCTTATGATATGCAACTGAAACAAGTTCTTGCTAATGGTAAAAAGGGGGCTTTGAATGTGGGGGCTGTTCTTATTTTACCTGAGGGGTTTGAATTAGCCCCTCCTAATCGTATTTCGCCAGAGATGAAAGAAAAGATGGGAAATCTGTCTTTTCAGAGTTATCGCCCCACAAAAAAAAATATTCTTGTGATAGGTCCTGTTCCTGGTCAGAAATATAGTGAAATTACCTTTCCTATCCTTTCTCCGGACCCCGCCACTAAGAAAGATGTTCACTTTTTCAAATATCCCATATATGTAGGCGGAAACAGGGGAAGGGGTCAGATTTATCCCGACGGGAGCAAGAGTAACAATACGGTTTATAATGCTACAGCAGCAGGTATAGTAAGCAAAATCATACGAAAAGAAAAGGGGGGGTACGAAATAACCATAACGGATGCGTCAGAGGGACGTCAAGTGATTGATATTATACCTCCAGGACCAGAACTTCTTGTTTCAGAAGGCGAATCCATCAAACTTGATCAACCATTAACGAGTAATCCTAATGTGGGTGGATTTGGTCAGGGGGATTCAGAAATAGTACTTCAAGACCCATTACGTGCCCAAGGCCTTTTGTTCTTCTTGGCGTCGGTTATTTTGGCACAAATCTTTTTGGTTCTTAAAAAGAAACAGTTTGAGAAGGTTCAATTGTCCGAAATGAATTTCTAG

>lcl|NC_016736.1_cds_YP_005090204.1_48 [gene=psbB] [locus_tag=RCOM_ORF00070] [db_xref=GeneID:11542345] [protein=photosystem II 47 kDa protein] [protein_id=YP_005090204.1] [location=77996..79522] [gbkey=CDS]
ATGGGTTTGCCTTGGTATCGTGTTCATACCGTCGTATTAAATGATCCCGGTCGTTTGCTGTCTGTCCATATAATGCATACAGCTTTAGTTGCTGGTTGGGCCGGTTCGATGGCTCTTTATGAATTAGCAGTTTTTGATCCCTCTGACCCCGTTCTCGATCCAATGTGGAGACAAGGTATGTTCGTTATACCCTTCATGACTCGTTTAGGAATAACCAATTCATGGGGTGGTTGGAGTATCACAGGAGGAACTATAACGAATCCGGGTATTTGGAGTTATGAAGGCGTGGCTGGGGCGCATATTGTGTTTTCTGGCTTGTGCTTCTTGGCAGCTATTTGGCATTGGGTGTATTGGGATCTAGAAATATTTTGCGATGAACGTACAGGAAAACCTTCTTTGGATTTGCCAAAGATCTTTGGAATTCATTTATTTCTCTCAGGGGTGGCTTGCTTTGGGTTTGGCGCTTTTCATGTAACCGGGTTGTATGGTCCTGGAATATGGGTGTCCGATCCTTATGGACTAACTGGAAAGGTACAACCCGTAAGTCCAGCATGGGGTGTGGAAGGGTTTGATCCTTTTGTTCCGGGAGGAATAGCCTCTCATCATATTGCAGCGGGTACATTGGGTATATTAGCGGGCCTATTCCATCTTAGTGTGCGTCCACCCCAACGTTTATACAAAGGATTACGTATGGGAAATATTGAAACTGTCCTTTCCAGTAGTATCGCTGCTGTCTTTTTTGCAGCTTTTGTTGTTGCTGGAACTATGTGGTATGGTTCAGCAACTACTCCGATTGAATTATTTGGTCCCACTCGTTATCAATGGGATCAAGGATACTTCCAGCAAGAAATATATCGAAGAGTTAGTGCTGGGCTGGCCGAAAATCAAAGTTTATCTGAAGCTTGGTCTAAAATTCCCGAAAAATTAGCTTTTTATGATTATATCGGCAATAATCCGGCAAAAGGTGGATTGTTCAGAGCAGGCTCAATGGACAATGGAGATGGAATAGCTGTTGGGTGGTTAGGACATCCTATCTTTAGAGATAAAGAAGGGCGTGAGCTTTTTGTACGTCGTATGCCTACTTTTTTTGAAACATTTCCAGTTGTTTTGGTAGACGGAGATGGAATTGTTAGAGCCGATGTTCCTTTTCGAAGGGCAGAGTCAAAGTATAGTGTCGAACAAGTAGGTGTAACCATTGAGTTCTATGGTGGCGAACTAAATGGAGTCAGTTATAGTGATCCTGCTACTGTGAAAAAATATGCTAGACGCGCTCAATTGGGTGAAATTTTTGAATTAGATCGCGCTACTTTGAAATCCGATGGTGTTTTTCGTAGCAGTCCAAGGGGTTGGTTTACTTTTGGACATGCTTCGTTCGCTCTGCTCTTTTTTTTCGGACACATTTGGCATGGTGCTCGAACTTTGTTCAGAGATGTTTTTGCTGGCATTGATCCAGATTTAGACGTTCAAGTGGAATTTGGAGCATTCCAAAAAATTGGAGATCCGACTACAAGAAGACAGGTAGTCTGA

>lcl|NC_016736.1_cds_YP_005090190.1_34 [gene=cemA] [locus_tag=RCOM_ORF00055] [db_xref=GeneID:11542329] [protein=envelope membrane protein] [protein_id=YP_005090190.1] [location=65796..66482] [gbkey=CDS]
ATGAAAAAAAAAACATTTATTCCCTTTCTATATCTTACAGCTATAGTTTTTTTGCCCTGGTGGATCTCTTTTTTATTTAATAAAAGTTTGGAATCTTGGGTTATTAATTGGTGTAATACTAGTAAATCCGAAACTTTTGTAAATGATATTCAAGAAAAAAGTATTCTAGAAAAGTTCATAGAATTAGAGGAACTCGTTCGCTTGGACGAAATGATAAAGGAATATCCGGAAACACATCTACAAAAGTTTCGTATCGGAATTCACAAAGAAACAATCCAATTGATCAAGATGCACAATGAGGATTATATCCATACGATTTTGCACTTCTCGACAAATATAATCTGTTTCGTTATTCTAAGTGGTTATTCTATTTTAAGTAATGAAGAACTTATTATTCTTAATTCTTGGGTTCAAGAATTCCTATATAATTTAAGCGACACAATAAAAGCTTTTTTCATTCTTTTATTAACCGATTTATGTATAGGATTCCATTCACCCCATGGTTGGGAACTAATGATTGGCTCGGTCTACAAAGATTTTGGGTTTGCTCATAATGATCAAATTATATCTGGCCTTGTTTCCACTTTTCCAGTCATCCTCGATACAATTTTTAAATATTTGATTTTCCGTTATTTAAATCGCGTATCTCCGTCACTTGTAGTGATTTATCATTCAATGAATGACTGA

>lcl|NC_016736.1_cds_YP_005090173.1_17 [gene=psbD] [locus_tag=RCOM_ORF00029] [db_xref=GeneID:11542303] [protein=photosystem II protein D2] [protein_id=YP_005090173.1] [location=36911..37972] [gbkey=CDS]
ATGACTATAGCCCTTGGTAAATTTACCAAAGACGAAAATGATTTATTTGATATTATGGATGACTGGTTACGGAGGGACCGTTTCGTTTTTGTAGGTTGGTCCGGTCTATTGCTCTTTCCTTGTGCCTATTTCGCCGTAGGGGGTTGGTTCACAGGTACAACCTTTGTAACCTCATGGTATACCCACGGATTGGCCAGTTCCTATTTGGAAGGTTGCAACTTCTTAACCGCCGCAGTTTCTACTCCTGCTAATAGTTTAGCACATTCTTTGTTATTACTATGGGGTCCTGAAGCACAAGGAGATTTTACTCGTTGGTGTCAATTAGGTGGTTTGTGGACTTTTGTTGCTCTCCACGGTGCTTTCGGGCTAATAGGTTTTATGTTACGTCAATTTGAACTTGCTCGATCTGTGCAATTGCGACCTTATAATGCAATCGCATTCTCTGGTCCAATTGCTGTTTTTGTTTCTGTATTCCTGATTTATCCACTAGGTCAGTCTGGTTGGTTTTTTGCGCCTAGTTTTGGTGTAGCAGCTATATTTCGATTCATCCTCTTTTTCCAAGGGTTTCATAACTGGACGCTGAACCCATTTCATATGATGGGAGTTGCCGGCGTATTGGGCGCTGCTCTGCTGTGCGCTATTCATGGTGCTACTGTAGAAAATACTTTATTTGAAGATGGTGATGGTGCAAATACATTCCGTGCTTTTAACCCAACTCAAGCTGAAGAAACTTATTCAATGGTCACCGCTAACCGCTTTTGGTCTCAAATCTTTGGGGTTGCTTTTTCCAATAAACGTTGGTTACATTTCTTTATGTTATTTGTACCCGTAACCGGTTTATGGATGAGCGCTCTTGGAGTAGTCGGTCTGGCTCTGAATCTACGTGCCTATGACTTCGTTTCTCAGGAAATTCGTGCAGCGGAAGATCCTGAATTTGAGACTTTCTACACTAAAAATATTCTCTTAAACGAAGGTATTCGGGCTTGGATGGCGGCTCAAGATCAGCCTCATGAAAACCTTATATTCCCTGAGGAGGTTCTACCACGTGGAAACGCTCTTTAA

>lcl|NC_016736.1_cds_YP_005090223.1_67 [gene=rps7] [locus_tag=RCOM_ORF00091] [db_xref=GeneID:11542367] [protein=ribosomal protein S7] [protein_id=YP_005090223.1] [location=complement(103705..104172)] [gbkey=CDS]
ATGTCACGTCGAGGTACTGCAGAAGAAAAAACTGCAAAATCCGATCCAATTTATCGTAATCGATTAGTTAACATGTTGGTTAACCGTATTCTGAAACACGGAAAAAAACCATTGGCTTATCAAATTATCTATCGAGCCATGAAAAAGATTCAACAAAAGACAGAAACAAATCCACTATCTGTTTTACGTCAAGCAATACGTGGAGTAACTCCCGATATAGCAGTAAAAGCAAGACGTGTAGGCGGATCGACTCATCAAGTTCCCGTTGAAATAGGATCCACACAAGGAAAAGCACTTGCCATTCGTTGGTTATTAGGGGCATCCCGAAAACGTCCGGGTCGAAATATGGCTTTCAAATTAAGTTCCGAATTAGTGGATGCTGCCAAAGGGAGTGGTGATGCCATACGCAAAAAGGAAGAGACTCATAGAATGGCAGAGGCAAATAGAGCTTTTGCACATTTTCGTTAA

>lcl|NC_016736.1_cds_YP_005090174.1_18 [gene=psbC] [locus_tag=RCOM_ORF00030] [db_xref=GeneID:11542304] [protein=photosystem II 44 kDa protein] [protein_id=YP_005090174.1] [location=37920..39341] [gbkey=CDS]
ATGAAAACCTTATATTCCCTGAGGAGGTTCTACCACGTGGAAACGCTCTTTAATGGAACTTTATCTTTAGCCGGTCGTGACCAAGAAACCACCGGTTTCGCTTGGTGGGCTGGGAATGCCCGACTTATCAATTTATCCGGTAAACTACTGGGAGCCCATGTAGCCCATGCTGGATTAATCGTATTCTGGGCCGGAGCAATGAACCTATTCGAAGTGGCTCATTTCGTACCAGAGAAGCCAATGTATGAACAAGGATTAATTTTACTTCCCCACCTAGCTACTCTAGGTTGGGGGGTAGGCCCTGGTGGGGAAGTTATAGACACCTTTCCATACTTTGTATCTGGCGTACTTCACTTAATTTCCTCCGCAGTATTGGGCTTTGGCGGTATTTATCATGCACTTCTGGGTCCTGAGACTCTTGAAGAATCTTTTCCATTTTTTGGTTATGTATGGAAAGATAGAAATAAAATGACAACAATTTTAGGTATTCACTTAATCTTGCTAGGTATAGGTGCTTTTCTTCTAGTATTCAAGGCTCTTTATTTTGGGGGCGTATATGATACCTGGGCTCCGGGGGGGGGAGATGTAAGAAAAATTACTAACTTAACCCTTAGCCCAAGTGTTATTTTCGGTTATTTACTAAAATCCCCTTTTGGAGGAGAAGGATGGATTGTTAGTGTGGACGATTTGGAAGATATAATTGGAGGGCATGTATGGTTAGGTTCCATTTGTATACTGGGTGGAATCTGGCATATCTTAACCAAACCCTTTGCATGGGCTCGCCGTGCACTTGTATGGTCTGGAGAGGCTTACTTGTCTTATAGTTTAGGTGCTTTATCCGTTTTTGGTTTCATTGCTTGTTGCTTTGTCTGGTTCAATAATACCGCTTACCCTAGTGAGTTTTACGGGCCTACTGGACCAGAAGCTTCTCAAGCTCAAGCTTTTACTTTTCTAGTTCGAGATCAACGTCTTGGGGCTAACGTGGGATCTGCTCAAGGACCTACCGGGTTAGGTAAATATTTAATGCGTTCGCCTACCGGAGAAGTTATTTTTGGAGGCGAAACTATGCGTTTTTGGGATCTGCGTGCTCCTTGGTTAGAACCTCTAAGAGGTCCAAATGGTTTGGACTTGAGTAGGTTGAAAAAAGACATACAACCTTGGCAAGAACGCCGTTCCGCAGAATATATGACCCACGCGCCTTTAGGTTCGTTAAATTCTGTAGGTGGCGTAGCTACCGAAATCAATGCAGTCAATTATGTCTCTCCTAGAAGTTGGTTAGCTACCTCTCATTTTGTTCTAGGGTTCTTCCTATTCGTAGGTCATTTATGGCACGCGGGAAGGGCTCGTGCAGCTGCAGCAGGATTTGAAAAGGGAATTGATCGTGATTTTGAACCCGTTCTTTCCATGACTCCTCTTAACTAA

>lcl|NC_016736.1_cds_YP_005090241.1_85 [gene=rpl2] [locus_tag=RCOM_ORF00130] [db_xref=GeneID:11542411] [protein=ribosomal protein L2] [protein_id=YP_005090241.1] [location=join(160814..161215,161845..162315)] [gbkey=CDS]
ATGGCGATACATTTATACAAAACTTCTACCCCGAGCACACGCAATGGAGCCGTAGACAGTCAAGTGAAATCCAATACACGAAATACACGAAATAATTTGATCTATGGACAGCATCGTTGTGGTAAAGGCCGTAACGCCAGAGGAATCATTACCGCAAGGCATAGAGGGGGAGGTCATAAGCGTCTATACCGTAAAATCGATTTTCGACGGAATGAAAAAGACATATATGGGAGAATCGTAACCATAGAATACGACCCTAATCGAAATGCATACATTTGTCTCATACACTATGGGGATGGTGAGAAGAGATATATTTTACATCCCAGAGGGGCTATAATTGGAGATACCATTATTTCTGGTACAGAAGTTCCTATAAAAATGGGAAATGCCCTACCTTTGAGTGAGGTTTTGATTGATCAAAAAGAAGAATCTACTTCAACCGATATGCCCTTAGGCACGGCCATACATAACATAGAAATCACATTTGGAAAGGGTGGACAATTAGCTAGAGCTGCAGGTGCTGTAGCGAAACTGATTGCAAAAGAGGGGAAATCGGCCACATTAAAATTACCTTCTGGGGAGGTTCGTTTAATATCCAAAAACTGCTCAGCAACAGTCGGACAAGTAGGGAATACTGGGGTGAACCAGAAAAGTTTGGGTAGAGCCGGATCTAAATGTTGGCTAGGTAAGCGTCCTGTAGTAAGAGGAGTAGTTATGAACCCTGTAGACCATCCCCATGGGGGTGGTGAAGGGAGGGCCCCAATTGGTAGAAAAAAACCCGCAACCCCTTGGGGTTATCCTGCACTTGGAAGAAGAAGTAGAAAAAGGAATAAATATAGTGATAATTTGATTCTTCGTCGCCGTAGTAAATAG

>lcl|NC_016736.1_cds_YP_005090227.1_71 [gene=ccsA] [locus_tag=RCOM_ORF00105] [db_xref=GeneID:11542381] [protein=cytochrome c biogenesis protein] [protein_id=YP_005090227.1] [location=121903..122883] [gbkey=CDS]
ATGATATTTTCAACTTTAGAACATATATTAACTCATATATCTTTTTCAGTCGTGTCACTTGTAATTACAATTCATTTGATAACTTTAGTAGTCGATGAATTCGTTGAACTATATGATTCGTCAGAAAAGGGCATGATAACCACTTTTTTCTGTATAACAGGATTATTAGTTACTCGTTGGATTTCTGGGGGACATTTACCATTAAGCGATTTATATGAATCATTAATCTTTCTTTCATGGGCTTTTTCCATTATTCATATGGTTCCGTATTTTAAAAAACATAAAAATTATTTAAGCGCAATAACCGCCCCAAGTACTTTTTTTACCCAAGGGTTTGCTACTTCAGGCCTTTTAACTAACACGCATCAATCCAAAATCTTAGTGCCTGCTCTCCAATCCCAGTGGTTAATGATGCACGTAAGTATGATGATATTGGGCTATGCAGCTCTTTTGTGTGGATCATTATTATCAGTAGCATTTCTAGTAATCACATTTCGAAAAATCATAAGAATTGTTGATGTTGATAAAAGCAATAATTTATTAAATGATTCATTTTCCTTTAGCTTTAGTGGGATACAATATATGATGGAAAGAAAGAATGTTTTAAAAAATATTTCTTTTCTTTCTTCTAGGAATTATTACAGGTTTCAATTGATTCAACAATTAGATGACTGGGGTTATCGTATTATAAGTATAGGGTTTATCTTTTTAACCATAGGTATTCTTTCGGGAGCAGTCTGGGCTAATGAAGCATGGGGATCATATTGGAATTGGGACCCCAAGGAAACTTGGGCATTTATTACGTGGACCATATTCGCAATTTATTTTCATACTCGAACAAATAAAAATTTTGAGGGTTTAAATTCCGCAATTGTCGCTTCTATTGGTTTTCTTATAATTTGGATATGCTATTTTGGAGTTAATTTATTAGGAATAGGACTACATAGTTATGGTTCATTTACATTAACAATTAACATCTAA

>lcl|NC_016736.1_cds_YP_005090177.1_21 [gene=psaB] [locus_tag=RCOM_ORF00036] [db_xref=GeneID:11542310] [protein=photosystem I P700 chlorophyll a] [protein_id=YP_005090177.1] [location=complement(41687..43891)] [gbkey=CDS]
[truncated: 100,110 more chars]
