# Supplementary material for: Comparative analysis of codon usage patterns in chloroplast genomes of six Euphorbiaceae species
Source: PeerJ. 2020 Jan 6;8:e8251. doi: 10.7717/peerj.8251 (PMC6951282; doi:10.7717/peerj.8251)
Supplement: Table S1 — Notes:Thebold indicates preference codons (RSCU > 1; (Sharp & Li, 1986)), the shaded portions indicate the lowest RFSC values of synonymous codons for each amino acid (low-frequency codons) and the border indicates high-frequency codons (Zhou, Tong & Shi, 2007). [file peerj-08-8251-s003.docx]

| **Amino acid** | **Codon** | ***Euphorbia esula*** | | | ***Hevea brasiliensis*** | | | ***Jatropha curcas*** | | | ***Manihot esculenta*** | | | ***Ricinus communis*** | | | ***Vernicia fordii*** | | |
| --- | --- | --- | --- | --- | --- | --- | --- | --- | --- | --- | --- | --- | --- | --- | --- | --- | --- | --- | --- |
|  |  | **Count** | **RSCU** | **RFSC** | **Count** | **RSCU** | **RFSC** | **Count** | **RSCU** | **RFSC** | **Count** | **RSCU** | **RFSC** | **Count** | **RSCU** | **RFSC** | **Count** | **RSCU** | **RFSC** |
| A(Ala) | GCT | 528 | **1.80** | 45.05 | 553 | **1.80** | 45.11 | 573 | **1.82** | 45.55 | 535 | **1.78** | 44.47 | 566 | **1.78** | 44.57 | 560 | **1.76** | 44.06 |
|  | GCC | 182 | 0.62 | 15.53 | 197 | 0.64 | 16.07 | 197 | 0.63 | 15.66 | 197 | 0.66 | 16.38 | 208 | 0.66 | 16.38 | 213 | 0.67 | 16.76 |
|  | GCA | 336 | **1.15** | 28.67 | 349 | **1.14** | 28.47 | 356 | **1.13** | 28.30 | 332 | **1.10** | 27.60 | 359 | **1.13** | 28.27 | 364 | **1.15** | 28.64 |
|  | GCG | 126 | 0.43 | 10.75 | 127 | 0.41 | 10.36 | 132 | 0.42 | 10.49 | 139 | 0.46 | 11.55 | 137 | 0.43 | 10.79 | 134 | 0.42 | 10.54 |
| C(Cys) | TGT | 195 | **1.52** | 75.88 | 208 | **1.51** | 75.36 | 211 | **1.50** | 75.09 | 191 | **1.50** | 75.20 | 206 | **1.46** | 72.79 | 217 | **1.51** | 75.35 |
|  | TGC | 62 | 0.48 | 24.12 | 68 | 0.49 | 24.64 | 70 | 0.50 | 24.91 | 63 | 0.50 | 24.80 | 77 | 0.54 | 27.21 | 71 | 0.49 | 24.65 |
| D(Asp) | GAT | 805 | **1.61** | 80.58 | 833 | **1.62** | 80.80 | 847 | **1.61** | 80.51 | 740 | **1.61** | 80.70 | 802 | **1.59** | 79.64 | 839 | **1.60** | 79.83 |
|  | GAC | 194 | 0.39 | 19.42 | 198 | 0.38 | 19.20 | 205 | 0.39 | 19.49 | 177 | 0.39 | 19.30 | 205 | 0.41 | 20.36 | 212 | 0.40 | 20.17 |
| E(Glu) | GAA | 976 | **1.51** | 75.72 | 981 | **1.52** | 75.93 | 995 | **1.50** | 74.92 | 915 | **1.55** | 77.35 | 1008 | **1.52** | 75.96 | 1031 | **1.51** | 75.42 |
|  | GAG | 313 | 0.49 | 24.28 | 311 | 0.48 | 24.07 | 333 | 0.50 | 25.08 | 268 | 0.45 | 22.65 | 319 | 0.48 | 24.04 | 336 | 0.49 | 24.58 |
| F(Phe) | TTT | 913 | **1.34** | 66.89 | 914 | **1.31** | 65.57 | 935 | **1.32** | 66.12 | 827 | **1.33** | 66.75 | 931 | **1.31** | 65.38 | 934 | **1.31** | 65.41 |
|  | TTC | 452 | 0.66 | 33.11 | 480 | 0.69 | 34.43 | 479 | 0.68 | 33.88 | 412 | 0.67 | 33.25 | 493 | 0.69 | 34.62 | 494 | 0.69 | 34.59 |
| G(Gly) | GGT | 519 | **1.35** | 33.86 | 518 | **1.30** | 32.56 | 528 | **1.30** | 32.49 | 489 | **1.30** | 32.47 | 521 | **1.27** | 31.85 | 523 | **1.27** | 31.83 |
|  | GGC | 154 | 0.40 | 10.05 | 165 | 0.41 | 10.37 | 161 | 0.40 | 9.91 | 166 | 0.44 | 11.02 | 171 | 0.42 | 10.45 | 178 | 0.43 | 10.83 |
|  | GGA | 607 | **1.58** | 39.60 | 642 | **1.61** | 40.35 | 649 | **1.60** | 39.94 | 597 | **1.59** | 39.64 | 662 | **1.62** | 40.46 | 657 | **1.60** | 39.99 |
|  | GGG | 253 | 0.66 | 16.50 | 266 | 0.67 | 16.72 | 287 | 0.71 | 17.66 | 254 | 0.67 | 16.87 | 282 | 0.69 | 17.24 | 285 | 0.69 | 17.35 |
| H(His) | CAT | 438 | **1.51** | 75.65 | 451 | **1.56** | 77.89 | 456 | **1.55** | 77.29 | 409 | **1.54** | 77.17 | 461 | **1.55** | 77.61 | 457 | **1.50** | 74.92 |
|  | CAC | 141 | 0.49 | 24.35 | 128 | 0.44 | 22.11 | 134 | 0.45 | 22.71 | 121 | 0.46 | 22.83 | 133 | 0.45 | 22.39 | 153 | 0.50 | 25.08 |
| I(Ile) | ATT | 987 | **1.50** | 49.92 | 1040 | **1.47** | 48.85 | 1041 | **1.48** | 49.34 | 976 | **1.47** | 49.09 | 1042 | **1.47** | 48.85 | 1057 | **1.49** | 49.60 |
|  | ATC | 355 | 0.54 | 17.96 | 394 | 0.56 | 18.51 | 399 | 0.57 | 18.91 | 363 | 0.55 | 18.26 | 417 | 0.59 | 19.55 | 401 | 0.56 | 18.82 |
|  | ATA | 635 | 0.96 | 32.12 | 695 | 0.98 | 32.64 | 670 | 0.95 | 31.75 | 649 | 0.98 | 32.65 | 674 | 0.95 | 31.60 | 673 | 0.95 | 31.58 |
| K(Lys) | AAA | 1021 | **1.52** | 76.02 | 994 | **1.51** | 75.36 | 1008 | **1.51** | 75.56 | 933 | **1.55** | 77.43 | 969 | **1.47** | 73.58 | 1007 | **1.50** | 75.21 |
|  | AAG | 322 | 0.48 | 23.98 | 325 | 0.49 | 24.64 | 326 | 0.49 | 24.44 | 272 | 0.45 | 22.57 | 348 | 0.53 | 26.42 | 332 | 0.50 | 24.79 |
| L(Leu) | TTA | 797 | **2.01** | 33.49 | 802 | **1.93** | 32.17 | 814 | **1.91** | 31.81 | 772 | **2.05** | 34.20 | 815 | **1.92** | 32.04 | 814 | **1.90** | 31.73 |
|  | TTG | 472 | **1.19** | 19.83 | 499 | **1.20** | 20.02 | 503 | **1.18** | 19.66 | 435 | **1.16** | 19.27 | 509 | **1.20** | 20.01 | 513 | **1.20** | 20.00 |
|  | CTT | 498 | **1.26** | 20.92 | 527 | **1.27** | 21.14 | 549 | **1.29** | 21.45 | 462 | **1.23** | 20.47 | 524 | **1.24** | 20.60 | 546 | **1.28** | 21.29 |
|  | CTC | 144 | 0.36 | 6.05 | 160 | 0.39 | 6.42 | 174 | 0.41 | 6.80 | 134 | 0.36 | 5.94 | 172 | 0.41 | 6.76 | 164 | 0.38 | 6.39 |
|  | CTA | 306 | 0.77 | 12.86 | 338 | 0.81 | 13.56 | 350 | 0.82 | 13.68 | 307 | 0.82 | 13.60 | 352 | 0.83 | 13.84 | 354 | 0.83 | 13.80 |
|  | CTG | 163 | 0.41 | 6.85 | 167 | 0.40 | 6.70 | 169 | 0.40 | 6.60 | 147 | 0.39 | 6.51 | 172 | 0.41 | 6.76 | 174 | 0.41 | 6.78 |
| M(Met) | ATG | 488 | 1.00 | 100.00 | 551 | 1.00 | 100.00 | 548 | 1.00 | 100.00 | 501 | 1.00 | 100.00 | 560 | 1.00 | 100.00 | 558 | 1.00 | 100.00 |
| N(Asn) | AAT | 949 | **1.57** | 78.56 | 944 | **1.56** | 78.15 | 958 | **1.54** | 77.20 | 840 | **1.55** | 77.56 | 960 | **1.54** | 76.98 | 965 | **1.55** | 77.57 |
|  | AAC | 259 | 0.43 | 21.44 | 264 | 0.44 | 21.85 | 283 | 0.46 | 22.80 | 243 | 0.45 | 22.44 | 287 | 0.46 | 23.02 | 279 | 0.45 | 22.43 |
| P(Pro) | CCT | 364 | **1.55** | 38.76 | 376 | **1.57** | 39.37 | 391 | **1.59** | 39.86 | 361 | **1.63** | 40.79 | 382 | **1.57** | 39.30 | 387 | **1.56** | 39.05 |
|  | CCC | 180 | 0.77 | 19.17 | 178 | 0.75 | 18.64 | 184 | 0.75 | 18.76 | 156 | 0.71 | 17.63 | 182 | 0.75 | 18.72 | 183 | 0.74 | 18.47 |
|  | CCA | 286 | **1.22** | 30.46 | 281 | **1.18** | 29.42 | 282 | **1.15** | 28.75 | 255 | **1.15** | 28.81 | 283 | **1.16** | 29.12 | 290 | **1.17** | 29.26 |
|  | CCG | 109 | 0.46 | 11.61 | 120 | 0.50 | 12.57 | 124 | 0.51 | 12.64 | 113 | 0.51 | 12.77 | 125 | 0.51 | 12.86 | 131 | 0.53 | 13.22 |
| Q(Gln) | CAA | 667 | **1.55** | 77.29 | 660 | **1.52** | 76.21 | 687 | **1.55** | 77.54 | 602 | **1.56** | 77.98 | 653 | **1.52** | 76.11 | 676 | **1.53** | 76.64 |
|  | CAG | 196 | 0.45 | 22.71 | 206 | 0.48 | 23.79 | 199 | 0.45 | 22.46 | 170 | 0.44 | 22.02 | 205 | 0.48 | 23.89 | 206 | 0.47 | 23.36 |
| R(Arg) | CGT | 295 | **1.26** | 20.95 | 296 | **1.25** | 20.82 | 309 | **1.28** | 21.31 | 279 | **1.31** | 21.83 | 294 | **1.23** | 20.49 | 308 | **1.26** | 21.08 |
|  | CGC | 104 | 0.44 | 7.39 | 107 | 0.45 | 7.52 | 107 | 0.44 | 7.38 | 104 | 0.49 | 8.14 | 115 | 0.48 | 8.01 | 105 | 0.43 | 7.19 |
|  | CGA | 329 | **1.40** | 23.37 | 310 | **1.31** | 21.80 | 329 | **1.36** | 22.69 | 278 | **1.31** | 21.75 | 309 | **1.29** | 21.53 | 335 | **1.38** | 22.93 |
|  | CGG | 95 | 0.40 | 6.75 | 112 | 0.47 | 7.88 | 105 | 0.43 | 7.24 | 101 | 0.47 | 7.90 | 114 | 0.48 | 7.94 | 101 | 0.41 | 6.91 |
|  | AGA | 438 | **1.87** | 31.11 | 450 | **1.90** | 31.65 | 436 | **1.80** | 30.07 | 393 | **1.85** | 30.75 | 446 | **1.86** | 31.08 | 450 | **1.85** | 30.80 |
|  | AGG | 147 | 0.63 | 10.44 | 147 | 0.62 | 10.34 | 164 | 0.68 | 11.31 | 123 | 0.58 | 9.62 | 157 | 0.66 | 10.94 | 162 | 0.67 | 11.09 |
| S(Ser) | TCT | 510 | **1.72** | 28.70 | 516 | **1.68** | 27.92 | 516 | **1.63** | 27.13 | 468 | **1.72** | 28.73 | 516 | **1.67** | 27.80 | 535 | **1.69** | 28.25 |
|  | TCC | 271 | 0.92 | 15.25 | 290 | 0.94 | 15.69 | 311 | 0.98 | 16.35 | 245 | 0.90 | 15.04 | 304 | 0.98 | 16.38 | 305 | 0.97 | 16.10 |
|  | TCA | 345 | **1.16** | 19.41 | 383 | **1.24** | 20.73 | 387 | **1.22** | 20.35 | 326 | **1.20** | 20.01 | 379 | **1.23** | 20.42 | 382 | **1.21** | 20.17 |
|  | TCG | 182 | 0.61 | 10.24 | 177 | 0.57 | 9.58 | 190 | 0.60 | 9.99 | 140 | 0.52 | 8.59 | 188 | 0.61 | 10.13 | 181 | 0.57 | 9.56 |
|  | AGT | 367 | **1.24** | 20.65 | 370 | **1.20** | 20.02 | 384 | **1.21** | 20.19 | 352 | **1.30** | 21.61 | 369 | **1.19** | 19.88 | 390 | **1.24** | 20.59 |
|  | AGC | 102 | 0.34 | 5.74 | 112 | 0.36 | 6.06 | 114 | 0.36 | 5.99 | 98 | 0.36 | 6.02 | 100 | 0.32 | 5.39 | 101 | 0.32 | 5.33 |
| T(Thr) | ACT | 475 | **1.66** | 41.56 | 482 | **1.62** | 40.57 | 507 | **1.65** | 41.32 | 454 | **1.64** | 41.12 | 474 | **1.58** | 39.40 | 501 | **1.63** | 40.76 |
|  | ACC | 201 | 0.70 | 17.59 | 207 | 0.70 | 17.42 | 209 | 0.68 | 17.03 | 189 | 0.68 | 17.12 | 215 | 0.71 | 17.87 | 220 | 0.72 | 17.90 |
|  | ACA | 366 | **1.28** | 32.02 | 375 | **1.26** | 31.57 | 388 | **1.26** | 31.62 | 349 | **1.26** | 31.61 | 384 | **1.28** | 31.92 | 375 | **1.22** | 30.51 |
|  | ACG | 101 | 0.35 | 8.84 | 124 | 0.42 | 10.44 | 123 | 0.40 | 10.02 | 112 | 0.41 | 10.14 | 130 | 0.43 | 10.81 | 133 | 0.43 | 10.82 |
| V(Val) | GTT | 442 | **1.48** | 36.89 | 451 | **1.44** | 36.02 | 471 | **1.46** | 36.54 | 418 | **1.44** | 35.88 | 432 | **1.39** | 34.75 | 462 | **1.43** | 35.73 |
|  | GTC | 153 | 0.51 | 12.77 | 143 | 0.46 | 11.42 | 148 | 0.46 | 11.48 | 136 | 0.47 | 11.67 | 152 | 0.49 | 12.23 | 158 | 0.49 | 12.22 |
|  | GTA | 432 | **1.44** | 36.06 | 454 | **1.45** | 36.26 | 467 | **1.45** | 36.23 | 437 | **1.50** | 37.51 | 469 | **1.51** | 37.73 | 476 | **1.47** | 36.81 |
|  | GTG | 171 | 0.57 | 14.27 | 204 | 0.65 | 16.29 | 203 | 0.63 | 15.75 | 174 | 0.60 | 14.94 | 190 | 0.61 | 15.29 | 197 | 0.61 | 15.24 |
| W(Trp) | TGG | 404 | 1.00 | 100.00 | 408 | 1.00 | 100.00 | 438 | 1.00 | 100.00 | 375 | 1.00 | 100.00 | 433 | 1.00 | 100.00 | 435 | 1.00 | 100.00 |
| Y(Tyr) | TAT | 679 | **1.63** | 81.32 | 745 | **1.67** | 83.71 | 741 | **1.66** | 82.89 | 686 | **1.66** | 82.85 | 750 | **1.65** | 82.69 | 743 | **1.65** | 82.37 |
|  | TAC | 156 | 0.37 | 18.68 | 145 | 0.33 | 16.29 | 153 | 0.34 | 17.11 | 142 | 0.34 | 17.15 | 157 | 0.35 | 17.31 | 159 | 0.35 | 17.63 |
| TER | TAA | 38 | **2.15** | 71.70 | 32 | **1.75** | 58.18 | 32 | **1.66** | 55.17 | 30 | **1.64** | 54.55 | 31 | **1.69** | 56.36 | 34 | **1.79** | 59.65 |
|  | TAG | 9 | 0.51 | 16.98 | 13 | 0.71 | 23.64 | 16 | 0.83 | 27.59 | 15 | 0.82 | 27.27 | 15 | 0.82 | 27.27 | 15 | 0.79 | 26.32 |
|  | TGA | 6 | 0.34 | 11.32 | 10 | 0.55 | 18.18 | 10 | 0.52 | 17.24 | 10 | 0.55 | 18.18 | 9 | 0.49 | 16.36 | 8 | 0.42 | 14.04 |
|  | | | | | | | | | | | | | | | | | | | |
