# Supplementary material for: Comparative analysis of codon usage patterns in chloroplast genomes of six Euphorbiaceae species
Source: PeerJ. 2020 Jan 6;8:e8251. doi: 10.7717/peerj.8251 (PMC6951282; doi:10.7717/peerj.8251)
Supplement: Table S1 — Notes: A: Arabidopsis thaliana; E: Escherichia coli; Ee: Euphorbia esula; Hb: Hevea brasiliensis; Jc: Jatropha curcas; Me: Manihot esculenta; P: Populus trichocarpa; Rc: Ricinus communis; S: Saccharomyces cerevisiae; Vf: Vernicia fordii. The shadow indicates ratios ≤ 0.5 or ≥ 2. [file peerj-08-8251-s004.docx]

| **Amino acid** | **Codon** | **Ee/A** | **Hb/A** | **Jc/A** | **Me/A** | **Rc/A** | **Vf/A** | **Ee/P** | **Hb/P** | **Jc/P** | **Me/P** | **Rc/P** | **Vf/P** | **Ee/E** | **Hb/E** | **Jc/E** | **Me/E** | **Rc/E** | **Vf/E** | **Ee/S** | **Hb/S** | **Jc/S** | **Me/S** | **Rc/S** | **Vf/S** |
| --- | --- | --- | --- | --- | --- | --- | --- | --- | --- | --- | --- | --- | --- | --- | --- | --- | --- | --- | --- | --- | --- | --- | --- | --- | --- |
| A(Ala) | GCT | 0.80 | 0.82 | 0.83 | 0.86 | 0.82 | 0.80 | 1.03 | 1.05 | 1.06 | 1.11 | 1.06 | 1.03 | 1.46 | 1.48 | 1.50 | 1.56 | 1.49 | 1.46 | 1.07 | 1.09 | 1.10 | 1.15 | 1.10 | 1.07 |
|  | GCC | 0.76 | 0.80 | 0.78 | 0.87 | 0.83 | 0.84 | 0.80 | 0.84 | 0.82 | 0.92 | 0.87 | 0.88 | 0.31 | 0.33 | 0.32 | 0.36 | 0.34 | 0.34 | 0.62 | 0.65 | 0.64 | 0.71 | 0.68 | 0.69 |
|  | GCA | 0.83 | 0.83 | 0.83 | 0.86 | 0.84 | 0.84 | 0.72 | 0.72 | 0.72 | 0.75 | 0.73 | 0.73 | 0.70 | 0.71 | 0.71 | 0.73 | 0.72 | 0.72 | 0.89 | 0.90 | 0.90 | 0.93 | 0.91 | 0.91 |
|  | GCG | 0.60 | 0.59 | 0.60 | 0.70 | 0.63 | 0.60 | 1.47 | 1.43 | 1.46 | 1.71 | 1.52 | 1.47 | 0.17 | 0.17 | 0.17 | 0.20 | 0.18 | 0.17 | 0.88 | 0.85 | 0.87 | 1.02 | 0.91 | 0.88 |
| C(Cys) | TGT | 0.80 | 0.83 | 0.82 | 0.83 | 0.81 | 0.84 | 0.75 | 0.77 | 0.77 | 0.78 | 0.76 | 0.79 | 1.53 | 1.58 | 1.57 | 1.58 | 1.54 | 1.60 | 1.04 | 1.07 | 1.06 | 1.07 | 1.05 | 1.09 |
|  | TGC | 0.37 | 0.39 | 0.40 | 0.40 | 0.44 | 0.40 | 0.30 | 0.32 | 0.32 | 0.32 | 0.36 | 0.32 | 0.39 | 0.41 | 0.41 | 0.42 | 0.46 | 0.42 | 0.56 | 0.59 | 0.60 | 0.60 | 0.66 | 0.60 |
| D(Asp) | GAT | 0.95 | 0.95 | 0.95 | 0.92 | 0.90 | 0.93 | 0.83 | 0.83 | 0.83 | 0.81 | 0.79 | 0.81 | 1.08 | 1.08 | 1.08 | 1.05 | 1.03 | 1.06 | 0.92 | 0.92 | 0.92 | 0.90 | 0.88 | 0.91 |
|  | GAC | 0.49 | 0.48 | 0.49 | 0.47 | 0.49 | 0.50 | 0.58 | 0.58 | 0.59 | 0.56 | 0.59 | 0.60 | 0.45 | 0.44 | 0.45 | 0.43 | 0.45 | 0.46 | 0.41 | 0.41 | 0.41 | 0.40 | 0.42 | 0.43 |
| E(Glu) | GAA | 1.23 | 1.19 | 1.19 | 1.21 | 1.21 | 1.22 | 1.04 | 1.01 | 1.00 | 1.03 | 1.02 | 1.03 | 1.10 | 1.07 | 1.06 | 1.09 | 1.09 | 1.10 | 0.92 | 0.90 | 0.89 | 0.91 | 0.91 | 0.92 |
|  | GAG | 0.42 | 0.40 | 0.42 | 0.38 | 0.41 | 0.42 | 0.41 | 0.40 | 0.42 | 0.38 | 0.40 | 0.42 | 0.76 | 0.73 | 0.77 | 0.69 | 0.74 | 0.77 | 0.70 | 0.68 | 0.71 | 0.64 | 0.68 | 0.71 |
| F(Phe) | TTT | 1.80 | 1.75 | 1.75 | 1.73 | 1.76 | 1.74 | 1.52 | 1.47 | 1.48 | 1.45 | 1.48 | 1.46 | 1.70 | 1.64 | 1.65 | 1.62 | 1.65 | 1.63 | 1.51 | 1.46 | 1.46 | 1.44 | 1.47 | 1.45 |
|  | TTC | 0.94 | 0.97 | 0.95 | 0.91 | 0.98 | 0.97 | 1.11 | 1.14 | 1.12 | 1.07 | 1.16 | 1.15 | 1.15 | 1.18 | 1.16 | 1.11 | 1.20 | 1.19 | 1.06 | 1.09 | 1.06 | 1.02 | 1.10 | 1.09 |
| G(Gly) | GGT | 1.01 | 0.97 | 0.97 | 1.00 | 0.97 | 0.96 | 1.24 | 1.20 | 1.20 | 1.24 | 1.19 | 1.18 | 0.92 | 0.89 | 0.88 | 0.91 | 0.88 | 0.87 | 0.94 | 0.90 | 0.90 | 0.93 | 0.90 | 0.89 |
|  | GGC | 0.72 | 0.75 | 0.72 | 0.82 | 0.76 | 0.79 | 0.65 | 0.67 | 0.65 | 0.74 | 0.69 | 0.71 | 0.24 | 0.25 | 0.24 | 0.27 | 0.25 | 0.26 | 0.68 | 0.70 | 0.67 | 0.77 | 0.72 | 0.74 |
|  | GGA | 1.08 | 1.11 | 1.10 | 1.12 | 1.13 | 1.10 | 1.16 | 1.18 | 1.17 | 1.20 | 1.21 | 1.18 | 2.91 | 2.98 | 2.95 | 3.02 | 3.03 | 2.96 | 2.40 | 2.46 | 2.43 | 2.49 | 2.50 | 2.45 |
|  | GGG | 1.07 | 1.09 | 1.15 | 1.13 | 1.14 | 1.13 | 0.95 | 0.96 | 1.02 | 1.01 | 1.01 | 1.01 | 0.96 | 0.98 | 1.04 | 1.02 | 1.03 | 1.02 | 1.82 | 1.85 | 1.96 | 1.93 | 1.93 | 1.93 |
| H(His) | CAT | 1.37 | 1.36 | 1.35 | 1.35 | 1.37 | 1.34 | 1.19 | 1.18 | 1.17 | 1.17 | 1.19 | 1.17 | 1.39 | 1.38 | 1.37 | 1.37 | 1.39 | 1.36 | 1.39 | 1.38 | 1.37 | 1.37 | 1.39 | 1.36 |
|  | CAC | 0.70 | 0.61 | 0.63 | 0.63 | 0.63 | 0.71 | 0.73 | 0.64 | 0.66 | 0.66 | 0.66 | 0.75 | 0.62 | 0.54 | 0.56 | 0.56 | 0.56 | 0.63 | 0.78 | 0.68 | 0.70 | 0.71 | 0.70 | 0.80 |
| I(Ile) | ATT | 1.98 | 2.02 | 1.98 | 2.07 | 1.99 | 2.00 | 1.45 | 1.48 | 1.45 | 1.52 | 1.46 | 1.46 | 1.43 | 1.46 | 1.43 | 1.49 | 1.44 | 1.44 | 1.41 | 1.44 | 1.41 | 1.48 | 1.42 | 1.43 |
|  | ATC | 0.83 | 0.89 | 0.88 | 0.89 | 0.93 | 0.88 | 1.01 | 1.08 | 1.07 | 1.09 | 1.13 | 1.07 | 0.63 | 0.68 | 0.67 | 0.68 | 0.71 | 0.67 | 0.89 | 0.96 | 0.95 | 0.96 | 1.00 | 0.95 |
|  | ATA | 2.17 | 2.30 | 2.17 | 2.35 | 2.20 | 2.17 | 1.82 | 1.93 | 1.83 | 1.97 | 1.85 | 1.82 | 5.07 | 5.37 | 5.07 | 5.47 | 5.14 | 5.06 | 1.54 | 1.63 | 1.54 | 1.66 | 1.56 | 1.53 |
| K(Lys) | AAA | 1.43 | 1.35 | 1.34 | 1.38 | 1.29 | 1.33 | 1.29 | 1.22 | 1.21 | 1.25 | 1.17 | 1.20 | 1.32 | 1.25 | 1.24 | 1.28 | 1.20 | 1.23 | 1.05 | 0.99 | 0.98 | 1.01 | 0.95 | 0.98 |
|  | AAG | 0.42 | 0.41 | 0.41 | 0.38 | 0.44 | 0.41 | 0.43 | 0.42 | 0.41 | 0.38 | 0.44 | 0.41 | 1.30 | 1.27 | 1.25 | 1.16 | 1.34 | 1.26 | 0.45 | 0.44 | 0.43 | 0.40 | 0.46 | 0.44 |
| L(Leu) | TTA | 2.70 | 2.63 | 2.62 | 2.77 | 2.64 | 2.60 | 2.30 | 2.25 | 2.23 | 2.36 | 2.25 | 2.22 | 2.47 | 2.41 | 2.39 | 2.53 | 2.41 | 2.38 | 1.31 | 1.28 | 1.27 | 1.34 | 1.28 | 1.26 |
|  | TTG | 0.97 | 1.00 | 0.98 | 0.95 | 1.00 | 1.00 | 0.79 | 0.81 | 0.80 | 0.77 | 0.82 | 0.81 | 1.45 | 1.49 | 1.47 | 1.42 | 1.50 | 1.49 | 0.75 | 0.77 | 0.76 | 0.73 | 0.77 | 0.77 |
|  | CTT | 0.89 | 0.91 | 0.93 | 0.87 | 0.89 | 0.92 | 0.74 | 0.76 | 0.77 | 0.73 | 0.74 | 0.76 | 1.83 | 1.88 | 1.92 | 1.80 | 1.84 | 1.89 | 1.74 | 1.79 | 1.82 | 1.71 | 1.75 | 1.80 |
|  | CTC | 0.39 | 0.41 | 0.44 | 0.38 | 0.44 | 0.41 | 0.44 | 0.47 | 0.50 | 0.43 | 0.50 | 0.47 | 0.56 | 0.61 | 0.65 | 0.55 | 0.64 | 0.61 | 1.15 | 1.24 | 1.32 | 1.13 | 1.31 | 1.23 |
|  | CTA | 1.33 | 1.42 | 1.45 | 1.41 | 1.46 | 1.45 | 1.09 | 1.17 | 1.18 | 1.16 | 1.20 | 1.19 | 3.30 | 3.52 | 3.58 | 3.50 | 3.62 | 3.59 | 0.98 | 1.05 | 1.07 | 1.04 | 1.08 | 1.07 |
|  | CTG | 0.72 | 0.71 | 0.70 | 0.68 | 0.72 | 0.72 | 0.48 | 0.47 | 0.47 | 0.46 | 0.48 | 0.48 | 0.14 | 0.14 | 0.14 | 0.13 | 0.14 | 0.14 | 0.67 | 0.66 | 0.66 | 0.64 | 0.67 | 0.67 |
| M(Met) | ATG | 0.86 | 0.94 | 0.91 | 0.93 | 0.94 | 0.92 | 0.89 | 0.98 | 0.95 | 0.97 | 0.98 | 0.96 | 0.78 | 0.85 | 0.83 | 0.85 | 0.85 | 0.84 | 1.01 | 1.10 | 1.07 | 1.09 | 1.10 | 1.08 |
| N(Asn) | AAT | 1.83 | 1.77 | 1.76 | 1.72 | 1.77 | 1.76 | 1.48 | 1.42 | 1.41 | 1.38 | 1.43 | 1.41 | 2.20 | 2.12 | 2.11 | 2.06 | 2.12 | 2.11 | 1.15 | 1.10 | 1.10 | 1.07 | 1.11 | 1.10 |
|  | AAC | 0.53 | 0.53 | 0.55 | 0.53 | 0.57 | 0.54 | 0.72 | 0.71 | 0.74 | 0.71 | 0.76 | 0.73 | 0.52 | 0.51 | 0.54 | 0.52 | 0.55 | 0.53 | 0.45 | 0.44 | 0.47 | 0.45 | 0.48 | 0.46 |
| P(Pro) | CCT | 0.84 | 0.84 | 0.85 | 0.88 | 0.84 | 0.84 | 0.98 | 0.98 | 1.00 | 1.03 | 0.98 | 0.98 | 2.15 | 2.15 | 2.19 | 2.25 | 2.15 | 2.15 | 1.16 | 1.16 | 1.18 | 1.22 | 1.16 | 1.16 |
|  | CCC | 1.46 | 1.40 | 1.42 | 1.34 | 1.41 | 1.40 | 1.49 | 1.43 | 1.45 | 1.37 | 1.44 | 1.43 | 1.34 | 1.28 | 1.30 | 1.22 | 1.29 | 1.28 | 1.14 | 1.09 | 1.11 | 1.04 | 1.10 | 1.09 |
|  | CCA | 0.77 | 0.73 | 0.72 | 0.72 | 0.72 | 0.73 | 0.74 | 0.70 | 0.69 | 0.70 | 0.70 | 0.70 | 1.45 | 1.38 | 1.36 | 1.37 | 1.37 | 1.38 | 0.67 | 0.64 | 0.63 | 0.63 | 0.64 | 0.64 |
|  | CCG | 0.55 | 0.58 | 0.59 | 0.60 | 0.60 | 0.62 | 1.17 | 1.25 | 1.27 | 1.29 | 1.29 | 1.33 | 0.22 | 0.23 | 0.23 | 0.24 | 0.24 | 0.24 | 0.89 | 0.94 | 0.96 | 0.97 | 0.97 | 1.00 |
| Q(Gln) | CAA | 1.48 | 1.42 | 1.45 | 1.41 | 1.38 | 1.41 | 1.38 | 1.32 | 1.34 | 1.31 | 1.29 | 1.31 | 1.92 | 1.84 | 1.87 | 1.83 | 1.79 | 1.83 | 1.05 | 1.01 | 1.03 | 1.00 | 0.98 | 1.00 |
|  | CAG | 0.56 | 0.57 | 0.54 | 0.51 | 0.55 | 0.55 | 0.48 | 0.49 | 0.46 | 0.44 | 0.48 | 0.48 | 0.29 | 0.29 | 0.28 | 0.26 | 0.29 | 0.28 | 0.70 | 0.71 | 0.67 | 0.64 | 0.70 | 0.69 |
| R(Arg) | CGT | 1.41 | 1.37 | 1.40 | 1.41 | 1.34 | 1.39 | 1.72 | 1.67 | 1.71 | 1.72 | 1.63 | 1.69 | 0.63 | 0.61 | 0.62 | 0.63 | 0.60 | 0.62 | 1.99 | 1.93 | 1.97 | 1.99 | 1.89 | 1.95 |
|  | CGC | 1.18 | 1.17 | 1.15 | 1.25 | 1.25 | 1.12 | 1.00 | 0.99 | 0.97 | 1.05 | 1.05 | 0.95 | 0.21 | 0.21 | 0.21 | 0.23 | 0.23 | 0.20 | 1.72 | 1.72 | 1.68 | 1.82 | 1.82 | 1.64 |
|  | CGA | 2.25 | 2.05 | 2.13 | 2.01 | 2.02 | 2.16 | 2.58 | 2.35 | 2.45 | 2.30 | 2.31 | 2.47 | 3.63 | 3.32 | 3.45 | 3.25 | 3.26 | 3.49 | 4.72 | 4.31 | 4.48 | 4.22 | 4.24 | 4.53 |
|  | CGG | 0.84 | 0.95 | 0.88 | 0.94 | 0.96 | 0.84 | 0.72 | 0.82 | 0.75 | 0.81 | 0.82 | 0.72 | 0.65 | 0.74 | 0.68 | 0.73 | 0.74 | 0.65 | 2.41 | 2.75 | 2.52 | 2.71 | 2.76 | 2.41 |
|  | AGA | 0.99 | 0.99 | 0.94 | 0.94 | 0.97 | 0.96 | 0.96 | 0.96 | 0.91 | 0.91 | 0.94 | 0.93 | 6.51 | 6.47 | 6.15 | 6.17 | 6.33 | 6.30 | 0.89 | 0.88 | 0.84 | 0.84 | 0.86 | 0.86 |
|  | AGG | 0.58 | 0.56 | 0.61 | 0.51 | 0.59 | 0.60 | 0.50 | 0.48 | 0.53 | 0.44 | 0.51 | 0.52 | 3.33 | 3.23 | 3.53 | 2.95 | 3.40 | 3.46 | 0.69 | 0.67 | 0.73 | 0.61 | 0.70 | 0.71 |
| S(Ser) | TCT | 0.87 | 0.85 | 0.84 | 0.85 | 0.84 | 0.86 | 1.07 | 1.05 | 1.03 | 1.04 | 1.04 | 1.06 | 2.53 | 2.47 | 2.42 | 2.45 | 2.44 | 2.50 | 0.94 | 0.92 | 0.90 | 0.91 | 0.90 | 0.92 |
|  | TCC | 1.04 | 1.08 | 1.14 | 1.00 | 1.12 | 1.11 | 1.36 | 1.41 | 1.48 | 1.30 | 1.45 | 1.44 | 1.31 | 1.36 | 1.43 | 1.25 | 1.41 | 1.39 | 0.82 | 0.85 | 0.90 | 0.79 | 0.88 | 0.87 |
|  | TCA | 0.81 | 0.87 | 0.86 | 0.81 | 0.85 | 0.85 | 0.75 | 0.81 | 0.80 | 0.75 | 0.79 | 0.79 | 1.91 | 2.05 | 2.03 | 1.90 | 2.00 | 1.99 | 0.79 | 0.85 | 0.85 | 0.79 | 0.83 | 0.83 |
|  | TCG | 0.84 | 0.79 | 0.84 | 0.69 | 0.83 | 0.79 | 1.57 | 1.48 | 1.55 | 1.28 | 1.55 | 1.47 | 0.90 | 0.85 | 0.89 | 0.73 | 0.89 | 0.84 | 0.91 | 0.86 | 0.90 | 0.74 | 0.90 | 0.85 |
|  | AGT | 1.13 | 1.10 | 1.12 | 1.15 | 1.08 | 1.13 | 1.05 | 1.02 | 1.04 | 1.06 | 1.01 | 1.05 | 1.66 | 1.62 | 1.65 | 1.69 | 1.60 | 1.67 | 1.11 | 1.09 | 1.11 | 1.13 | 1.07 | 1.11 |
|  | AGC | 0.39 | 0.41 | 0.41 | 0.39 | 0.36 | 0.36 | 0.39 | 0.41 | 0.41 | 0.39 | 0.36 | 0.36 | 0.27 | 0.29 | 0.29 | 0.28 | 0.26 | 0.26 | 0.45 | 0.48 | 0.48 | 0.46 | 0.42 | 0.42 |
| T(Thr) | ACT | 1.17 | 1.15 | 1.18 | 1.18 | 1.11 | 1.16 | 1.42 | 1.40 | 1.44 | 1.44 | 1.35 | 1.41 | 2.25 | 2.21 | 2.28 | 2.27 | 2.14 | 2.23 | 1.01 | 0.99 | 1.02 | 1.02 | 0.96 | 1.00 |
|  | ACC | 0.84 | 0.84 | 0.83 | 0.84 | 0.86 | 0.87 | 1.04 | 1.04 | 1.03 | 1.04 | 1.07 | 1.08 | 0.38 | 0.38 | 0.37 | 0.38 | 0.39 | 0.39 | 0.68 | 0.68 | 0.67 | 0.68 | 0.70 | 0.70 |
|  | ACA | 1.00 | 1.00 | 1.01 | 1.01 | 1.01 | 0.97 | 1.03 | 1.02 | 1.04 | 1.04 | 1.03 | 0.99 | 1.92 | 1.91 | 1.93 | 1.94 | 1.93 | 1.86 | 0.89 | 0.88 | 0.89 | 0.89 | 0.89 | 0.86 |
|  | ACG | 0.57 | 0.67 | 0.65 | 0.66 | 0.69 | 0.70 | 0.99 | 1.18 | 1.14 | 1.16 | 1.22 | 1.23 | 0.29 | 0.35 | 0.34 | 0.34 | 0.36 | 0.36 | 0.54 | 0.65 | 0.63 | 0.64 | 0.67 | 0.67 |
| V(Val) | GTT | 0.70 | 0.69 | 0.71 | 0.70 | 0.65 | 0.69 | 0.79 | 0.78 | 0.80 | 0.79 | 0.73 | 0.77 | 1.03 | 1.02 | 1.04 | 1.03 | 0.96 | 1.01 | 0.86 | 0.85 | 0.87 | 0.86 | 0.80 | 0.85 |
|  | GTC | 0.51 | 0.47 | 0.47 | 0.48 | 0.49 | 0.50 | 0.58 | 0.53 | 0.54 | 0.55 | 0.55 | 0.57 | 0.44 | 0.40 | 0.40 | 0.41 | 0.41 | 0.42 | 0.56 | 0.51 | 0.51 | 0.52 | 0.53 | 0.54 |
|  | GTA | 1.88 | 1.91 | 1.93 | 2.01 | 1.95 | 1.95 | 1.82 | 1.86 | 1.87 | 1.95 | 1.89 | 1.89 | 1.68 | 1.71 | 1.72 | 1.79 | 1.74 | 1.74 | 1.58 | 1.60 | 1.62 | 1.69 | 1.64 | 1.64 |
|  | GTG | 0.42 | 0.49 | 0.48 | 0.46 | 0.45 | 0.46 | 0.44 | 0.50 | 0.49 | 0.47 | 0.46 | 0.47 | 0.29 | 0.33 | 0.33 | 0.31 | 0.31 | 0.31 | 0.68 | 0.79 | 0.77 | 0.73 | 0.72 | 0.74 |
| W(Trp) | TGG | 1.39 | 1.36 | 1.43 | 1.37 | 1.43 | 1.41 | 1.25 | 1.22 | 1.29 | 1.23 | 1.28 | 1.27 | 1.15 | 1.12 | 1.18 | 1.12 | 1.17 | 1.16 | 1.67 | 1.64 | 1.72 | 1.64 | 1.71 | 1.70 |
| Y(Tyr) | TAT | 2.00 | 2.13 | 2.07 | 2.14 | 2.11 | 2.07 | 1.79 | 1.91 | 1.86 | 1.92 | 1.89 | 1.85 | 1.77 | 1.88 | 1.84 | 1.89 | 1.87 | 1.83 | 1.56 | 1.65 | 1.61 | 1.66 | 1.64 | 1.60 |
|  | TAC | 0.49 | 0.44 | 0.46 | 0.47 | 0.47 | 0.47 | 0.71 | 0.64 | 0.66 | 0.68 | 0.68 | 0.68 | 0.56 | 0.50 | 0.52 | 0.53 | 0.53 | 0.53 | 0.45 | 0.41 | 0.42 | 0.44 | 0.44 | 0.44 |
| TER | TAA | 1.82 | 1.48 | 1.45 | 1.52 | 1.42 | 1.53 | 4.09 | 3.34 | 3.27 | 3.42 | 3.19 | 3.45 | 0.82 | 0.67 | 0.65 | 0.68 | 0.64 | 0.69 | 1.49 | 1.21 | 1.19 | 1.24 | 1.16 | 1.25 |
|  | TAG | 0.78 | 1.08 | 1.31 | 1.37 | 1.23 | 1.22 | 0.97 | 1.36 | 1.63 | 1.71 | 1.54 | 1.52 | 1.29 | 1.81 | 2.18 | 2.28 | 2.06 | 2.03 | 0.78 | 1.08 | 1.31 | 1.37 | 1.23 | 1.22 |
|  | TGA | 0.22 | 0.35 | 0.34 | 0.38 | 0.31 | 0.27 | 0.37 | 0.60 | 0.58 | 0.65 | 0.53 | 0.46 | 0.24 | 0.38 | 0.37 | 0.41 | 0.34 | 0.30 | 0.37 | 0.60 | 0.58 | 0.65 | 0.53 | 0.46 |
